# Supplementary material for: Monozygotic twins discordant for schizophrenia differ in maturation and synaptic transmission
Source: Mol Psychiatry. 2024 May 4;29(10):3208–22. doi: 10.1038/s41380-024-02561-1 (PMC11449799; doi:10.1038/s41380-024-02561-1)
Supplement: Supplementary file 1 — Supplementary material [file 41380_2024_2561_MOESM1_ESM.pdf]

Supplementary Figure 1. Pathway enrichment between different comparisons of the 3 groups – KEGG and GO Biological Processes. A. The top enriched KEGG pathways for the pooled schizophrenia neurons compared to the control twins' neurons. B. The top enriched GO biological Process terms for the pooled schizophrenia neurons compared to the control twins' neurons. C. The top enriched KEGG pathways for the pooled co-twin siblings' neurons compared to the control twins' neurons. D. The top enriched GO biological Process terms for the pooled co-twin siblings' neurons compared to the control twins neurons. E. The top enriched KEGG pathways for the pooled schizophrenia neurons compared to the co-twin siblings' neurons. F. The top enriched GO biological Process terms for the pooled schizophrenia neurons compared to the co-twin siblings' neurons.

Supplementary Figure 2. Pathway enrichment between different comparisons of the 3 groups – GO cellular component. A. The top enriched pathways for the pooled schizophrenia neurons compared to the control twins' neurons. B. The top enriched pathways for the pooled co-twin siblings' neurons compared to the control twins' neurons. C. The top enriched pathways for the pooled schizophrenia neurons compared to the co-twin siblings' neurons.

Supplementary Figure 3. NMDA excitatory post synaptic currents are reduced in neurons derived from the schizophrenia patients and their co-twins. The percentage of neurons that exhibited NMDA synaptic currents after blocking their AMPA and GABA synaptic currents with CNQX and bicuculine are presented.

Supplementary Figure 4. A comparison of DG transcriptome compared to human postmortem DG. A. The Y axis shows gene expression in our DG neurons and the x axis shows the expression of DEG between postmortem DG vs. hippocampus. B. Grouping the DEG that are overexpressed in the DG (in orange) compared to the DEG that have reduced expression in the DG vs. hippocampus (in blue), we observe a significant enrichment in the genes that are expressed in our DG neurons and our overexpressed in the DG of the human postmortem ($p=6.875*e^{-49}$ using a t-test).

Supplementary Figure 5. ICC for NESTIN (green), PAX6 (red), and DAPI (blue). Representative images and a quantification performed on 4-7 images for each of the lines. Approximately 90% of the cells in the NPC culture were positive for both markers confirming their cell identity.

Supplementary Figure 6. ICC for EMX2 (green), PAX6 (red), and DAPI (blue) study. Representative images and a quantification that was performed on 4-7 images for each of the lines. Approximately 90% of the cells in the NPC culture were positive for both markers confirming hippocampal NPC cell identity.

Supplementary Figure 7. H2O2 application reduced the excitability of neurons derived from both the schizophrenia patients and their co-twin, but the effect was larger in the co-twin.

Supplementary Figure 8. Morphological differences between the 3 groups. A. The soma area of the neurons derived from the schizophrenia patients was smaller compared to the other two groups of the co-twins and the controls. B. The total dendritic length of the neurons derived from the schizophrenia patients and their co-twins was smaller compared to neurons derived from controls.

Supplementary Figure 9. ICC for Prox1, NeuN, Map2, DAPI in DG neurons. A. Representative images are shown for all the lines. B. The quantification shows that there were no significant differences between the three groups.

Supplementary Figure 10. ICC for GABA, vGlut1, Map2, DAPI in DG neurons. A. Representative images are shown for all the lines. B. The quantification of vGlut1/DAPI shows no significant differences between the three groups. C. The quantification of GABA/DAPI shows no significant differences between the groups.

Supplementary Figure 11. Calbindin1 and SHISA3 have a reduced expression in the schizophrenia

patients compared to their unaffected co-twins (depressive disorder). A. Representative traces of ICC for Calbindin1 in DG neurons derived from the schizophrenia patients and their co-twin. B. A quantification over 6-7 images of each of the lines confirms the reduced gene expression that we have reported also at the protein level. C. Representative traces of ICC for SHISA3 in DG neurons derived from the schizophrenia patients and their co-twin. D. A quantification over 6-7 images of each of the lines confirms the reduced gene expression that we have reported also at the protein level.

Supplementary File 1. Karyotyping results of iPSCs.

Supplementary File 2. Analysis of RNA seq results pooling the affected twins vs. the pooled co-twins (KEGG and Biological pathways).

Supplementary File 3. RNA sequencing results.

Supplementary File 4. Analysis of RNA seq results pooling the affected twins vs. the pooled controls (KEGG and Biological pathways).

Supplementary File 5. Analysis of RNA seq results pooling the co-twins vs. the pooled controls (KEGG And pathways).

Supplementary File 6. Statistical analysis using a 2-way ANOVA for the number of spikes in the MEA recordings.

Supplementary File 7. Statistical analysis using a 2-way ANOVA for the number of bursts in the MEA recordings.

Supplementary File 8. Statistical analysis using a 2-way ANOVA for the number of network bursts in the MEA recordings

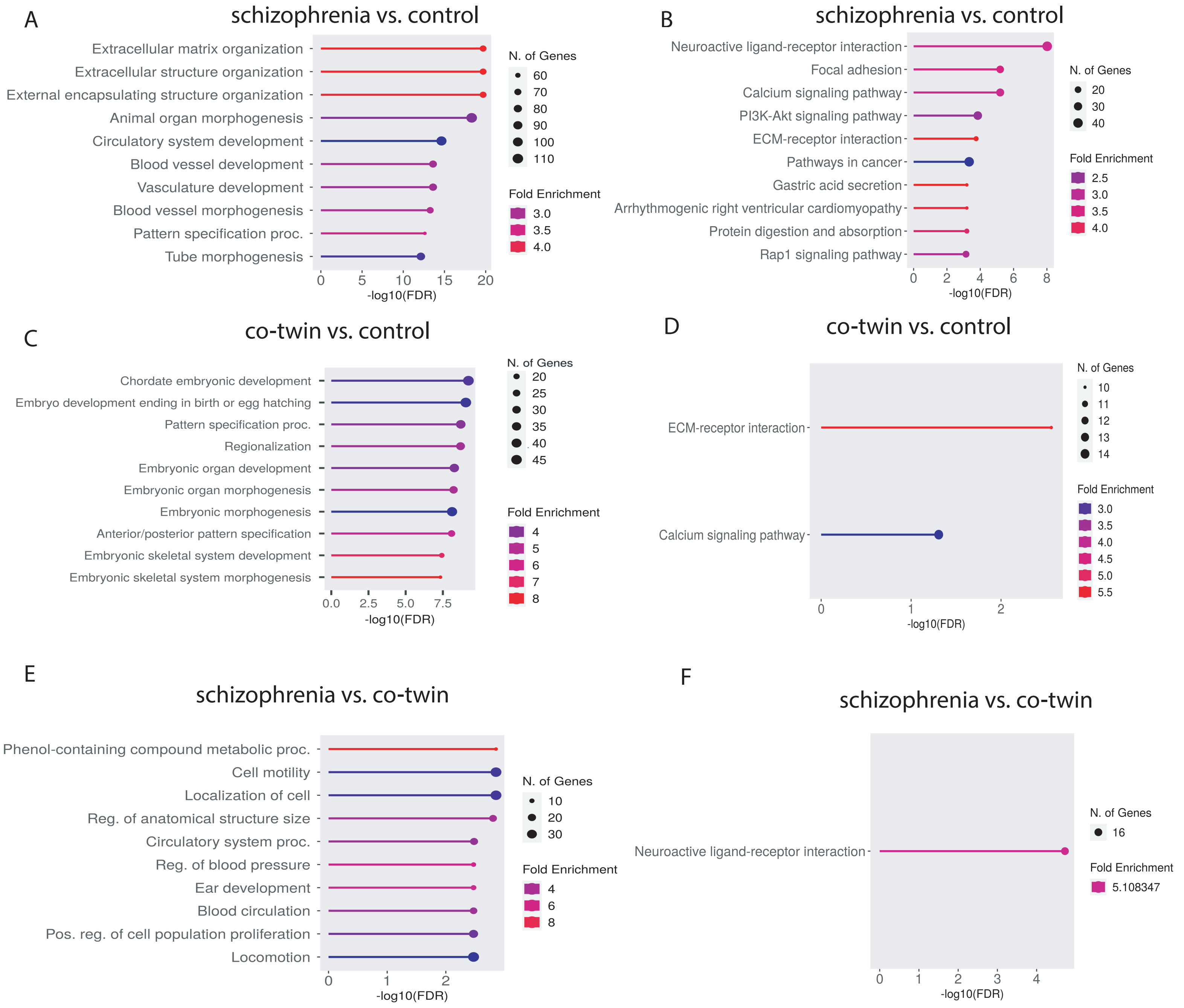

A
schizophrenia vs. control
Extracellular matrix organization
Extracellular structure organization
External encapsulating structure organization
Animal organ morphogenesis
Circulatory system development
Blood vessel development
Vasculature development
Blood vessel morphogenesis
Pattern specification proc.
Tube morphogenesis
0 5 10 15 20
-log10(FDR)
N. of Genes
60 70 80 90 100 110
Fold Enrichment
3.0 3.5 4.0
B
schizophrenia vs. control
Neuroactive ligand-receptor interaction
Focal adhesion
Calcium signaling pathway
PI3K-Akt signaling pathway
ECM-receptor interaction
Pathways in cancer
Gastric acid secretion
Arrhythmogenic right ventricular cardiomyopathy
Protein digestion and absorption
Rap1 signaling pathway
0 2 4 6 8
-log10(FDR)
N. of Genes
20 30 40
Fold Enrichment
2.5 3.0 3.5 4.0
C
co-twin vs. control
Chordate embryonic development
Embryo development ending in birth or egg hatching
Pattern specification proc.
Regionalization
Embryonic organ development
Embryonic organ morphogenesis
Embryonic morphogenesis
Anterior/posterior pattern specification
Embryonic skeletal system development
Embryonic skeletal system morphogenesis
0.0 2.5 5.0 7.5
-log10(FDR)
N. of Genes
20 25 30 35 40 45
Fold Enrichment
4 5 6 7 8
D
co-twin vs. control
ECM-receptor interaction
Calcium signaling pathway
0 1 2
-log10(FDR)
N. of Genes
10 11 12 13 14
Fold Enrichment
3.0 3.5 4.0 4.5 5.0 5.5
E
schizophrenia vs. co-twin
Phenol-containing compound metabolic proc.
Cell motility
Localization of cell
Reg. of anatomical structure size
Circulatory system proc.
Reg. of blood pressure
Ear development
Blood circulation
Pos. reg. of cell population proliferation
Locomotion
0 1 2
-log10(FDR)
N. of Genes
10 20 30
Fold Enrichment
4 6 8
F
schizophrenia vs. co-twin
Neuroactive ligand-receptor interaction
0 1 2 3 4
-log10(FDR)
N. of Genes
16
Fold Enrichment
5.108347

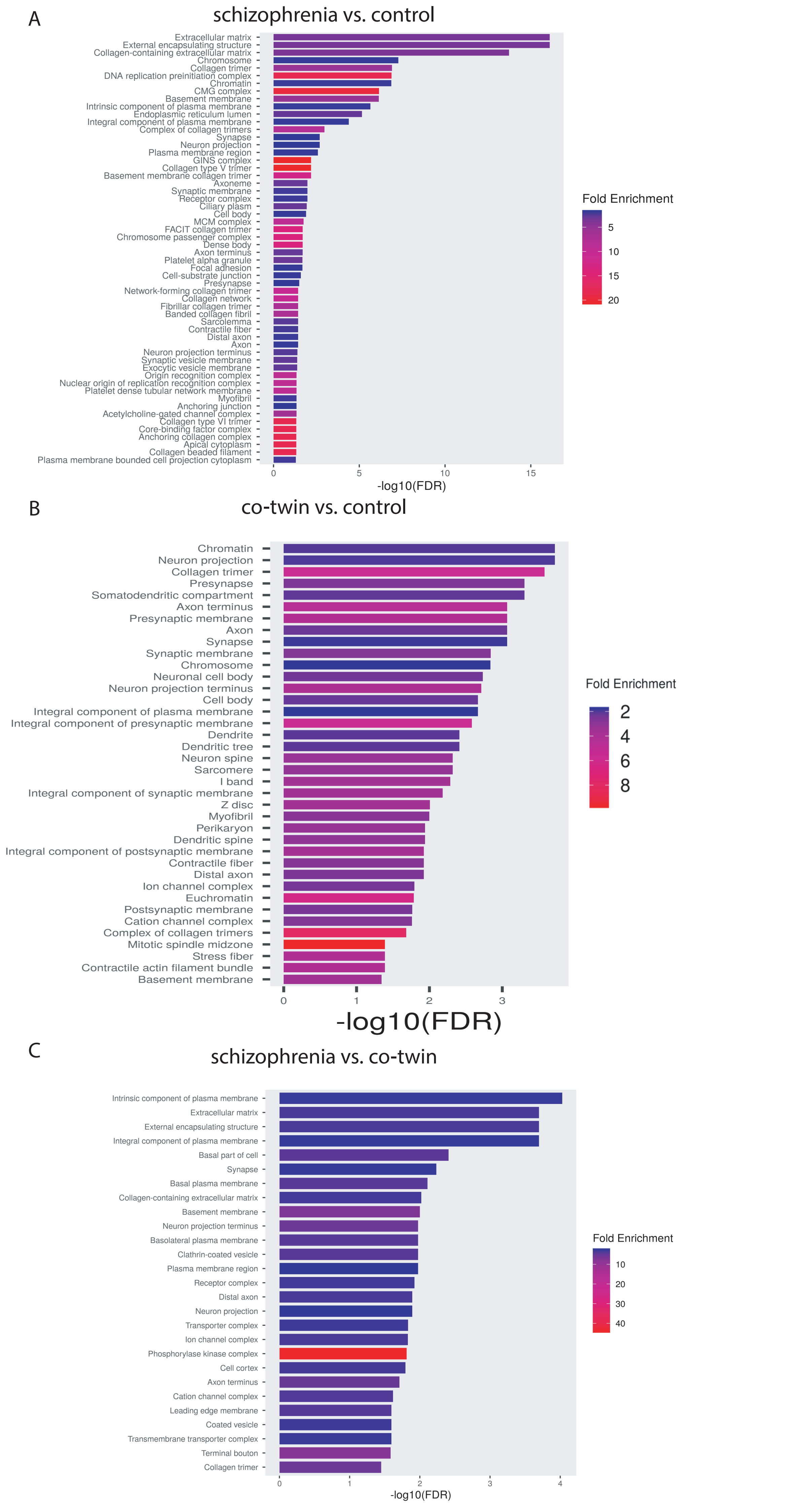
A
schizophrenia vs. control
Extracellular matrix
External encapsulating structure
Collagen-containing extracellular matrix
Chromosome
Collagen trimer
DNA replication preinitiation complex
Chromatin
CMG complex
Basement membrane
Intrinsic component of plasma membrane
Endoplasmic reticulum lumen
Integral component of plasma membrane
Complex of collagen trimers
Synapse
Neuron projection
Plasma membrane region
GINS complex
Collagen type V trimer
Basement membrane collagen trimer
Axoneme
Synaptic membrane
Receptor complex
Ciliary plasm
Cell body
MCM complex
FACIT collagen trimer
Chromosome passenger complex
Dense body
Axon terminus
Platelet alpha granule
Focal adhesion
Cell-substrate junction
Presynapse
Network-forming collagen trimer
Collagen network
Fibrillar collagen trimer
Banded collagen fibril
Sarcolemma
Contractile fiber
Distal axon
Axon
Neuron projection terminus
Synaptic vesicle membrane
Exocytic vesicle membrane
Origin recognition complex
Nuclear origin of replication recognition complex
Platelet dense tubular network membrane
Myofibril
Anchoring junction
Acetylcholine-gated channel complex
Collagen type VI trimer
Core-binding factor complex
Anchoring collagen complex
Apical cytoplasm
Collagen beaded filament
Plasma membrane bounded cell projection cytoplasm
0
5
10
15
-log10(FDR)
Fold Enrichment
5
10
15
20
B
co-twin vs. control
Chromatin
Neuron projection
Collagen trimer
Presynapse
Somatodendritic compartment
Axon terminus
Presynaptic membrane
Axon
Synapse
Synaptic membrane
Chromosome
Neuronal cell body
Neuron projection terminus
Cell body
Integral component of plasma membrane
Integral component of presynaptic membrane
Dendrite
Dendritic tree
Neuron spine
Sarcomere
I band
Integral component of synaptic membrane
Z disc
Myofibril
Perikaryon
Dendritic spine
Integral component of postsynaptic membrane
Contractile fiber
Distal axon
Ion channel complex
Euchromatin
Postsynaptic membrane
Cation channel complex
Complex of collagen trimers
Mitotic spindle midzone
Stress fiber
Contractile actin filament bundle
Basement membrane
0
1
2
3
-log10(FDR)
Fold Enrichment
2
4
6
8
C
schizophrenia vs. co-twin
Intrinsic component of plasma membrane
Extracellular matrix
External encapsulating structure
Integral component of plasma membrane
Basal part of cell
Synapse
Basal plasma membrane
Collagen-containing extracellular matrix
Basement membrane
Neuron projection terminus
Basolateral plasma membrane
Clathrin-coated vesicle
Plasma membrane region
Receptor complex
Distal axon
Neuron projection
Transporter complex
Ion channel complex
Phosphorylase kinase complex
Cell cortex
Axon terminus
Cation channel complex
Leading edge membrane
Coated vesicle
Transmembrane transporter complex
Terminal bouton
Collagen trimer
0
1
2
3
4
-log10(FDR)
Fold Enrichment
10
20
30
40

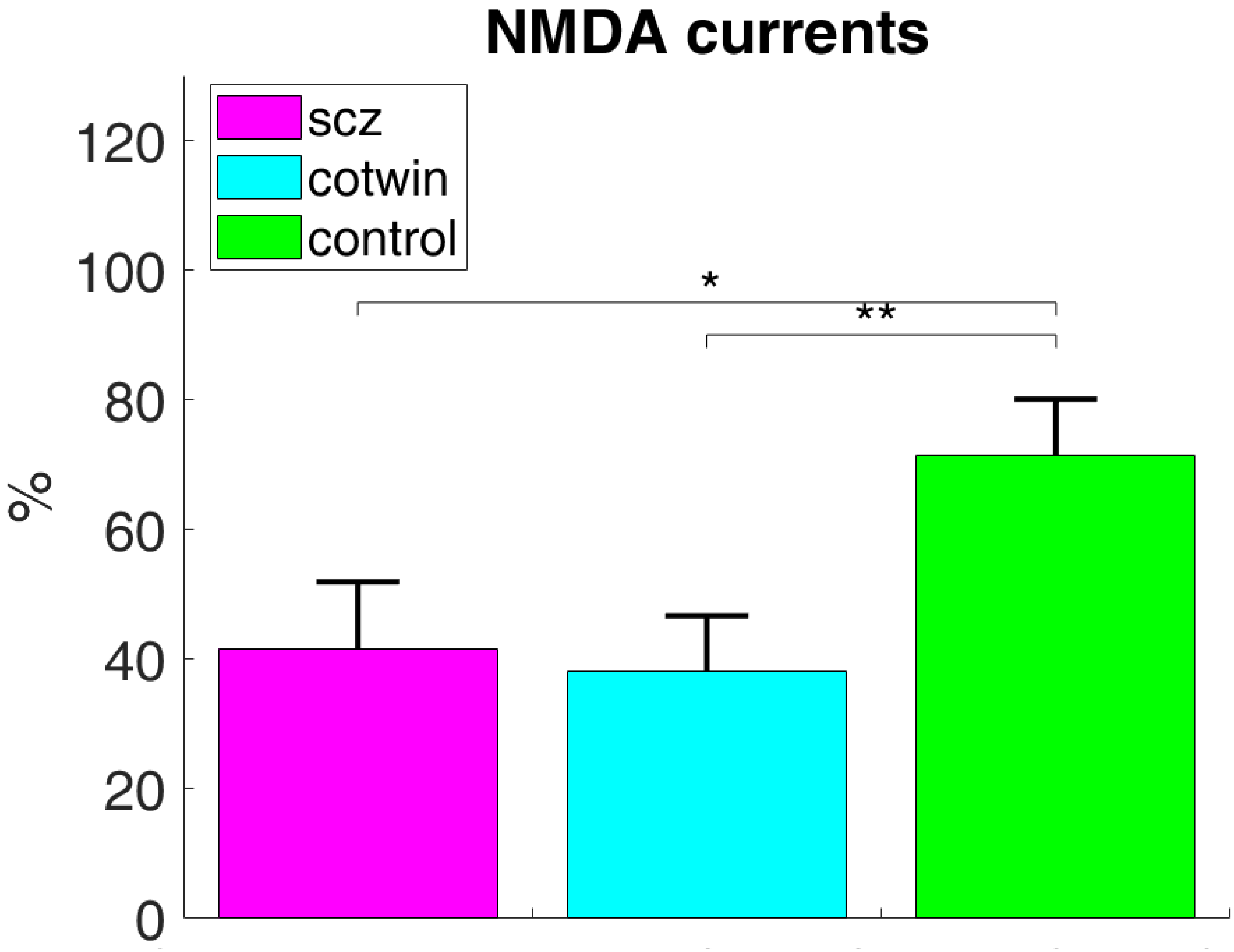

NMDA currents
scz
cotwin
control
%
0
20
40
60
80
100
120
*
**

A

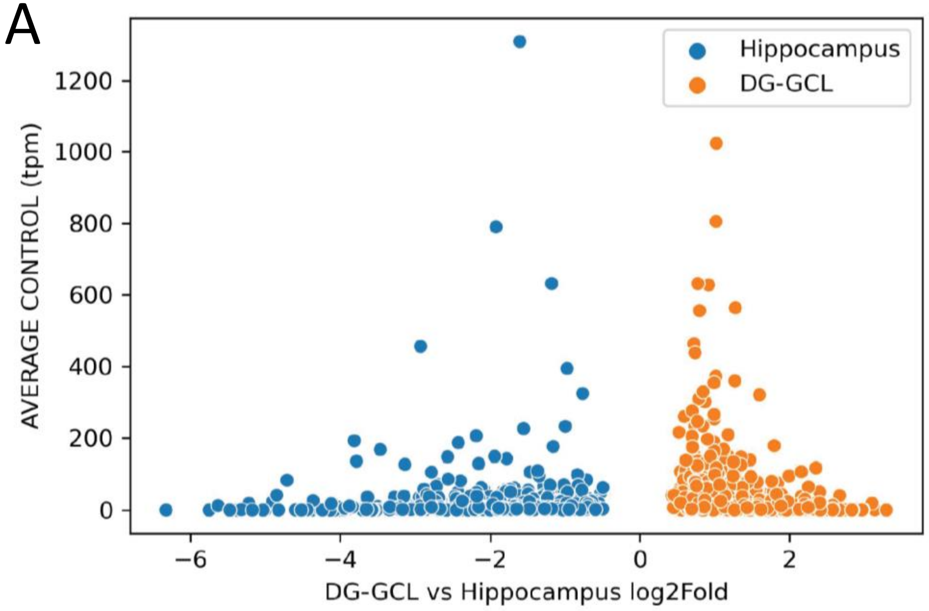

Hippocampus
DG-GCL
AVERAGE CONTROL (tpm)
1200
1000
800
600
400
200
0
−6
−4
−2
0
2
DG-GCL vs Hippocampus log2Fold

B

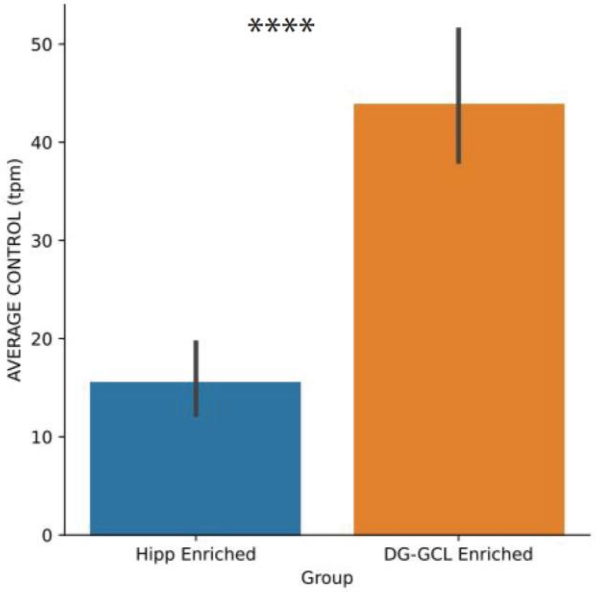

****
AVERAGE CONTROL (tpm)
50
40
30
20
10
0
Hipp Enriched
DG-GCL Enriched
Group

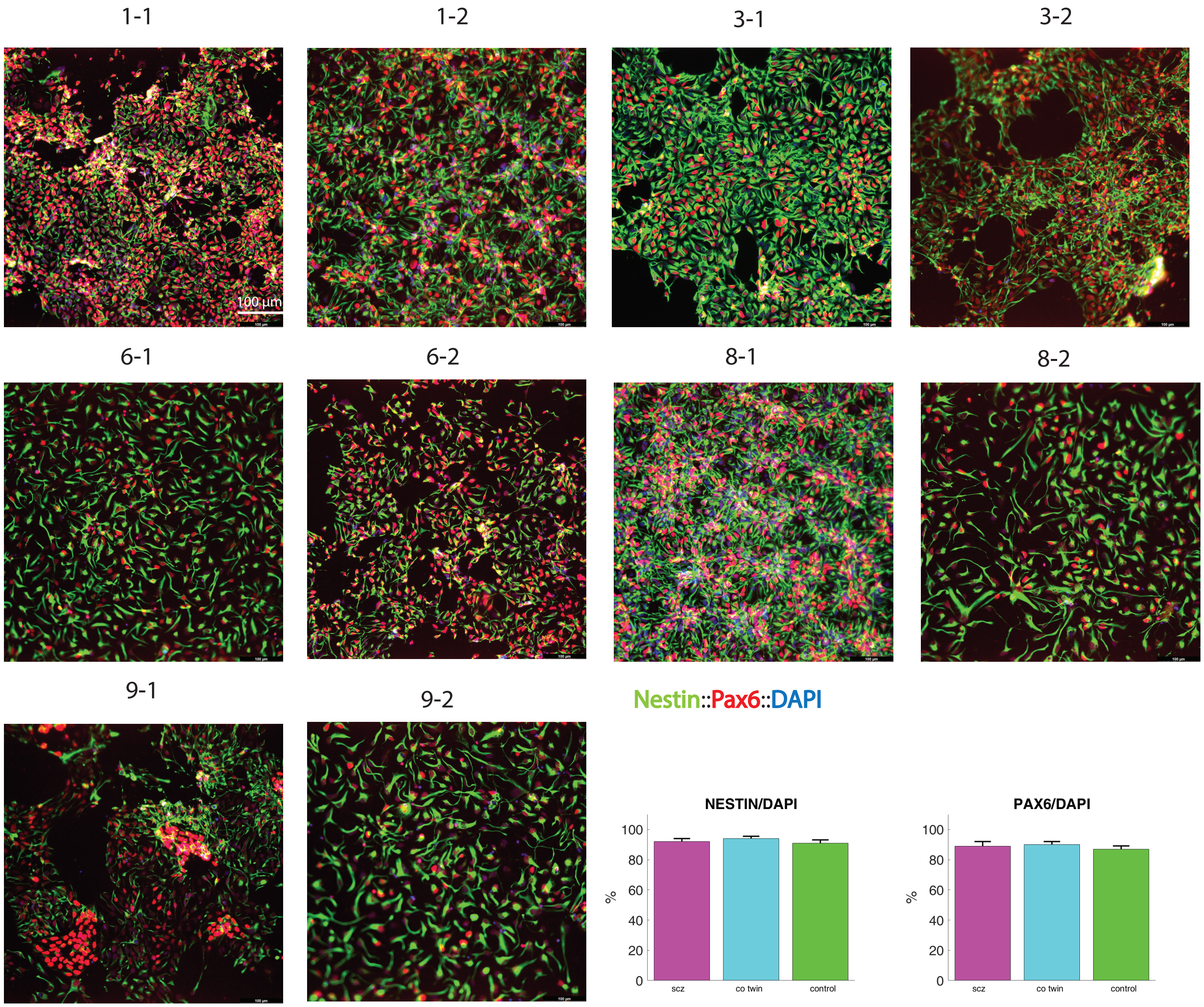
1-1
1-2
3-1
3-2
100 µm
6-1
6-2
8-1
8-2
9-1
9-2
Nestin::Pax6::DAPI
NESTIN/DAPI
PAX6/DAPI
%
100
80
60
40
20
0
scz
co twin
control

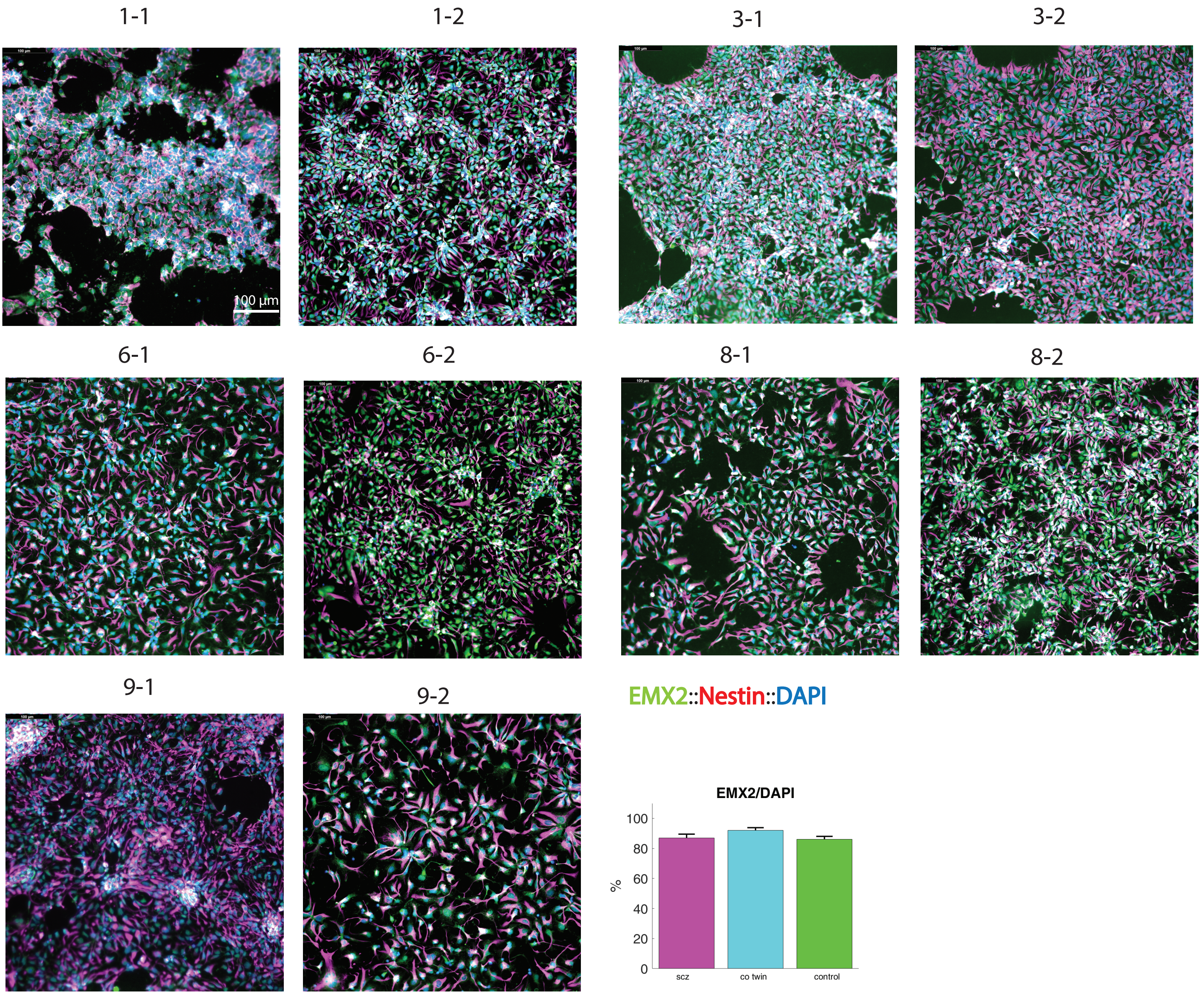
1-1
1-2
3-1
3-2
100 µm
6-1
6-2
8-1
8-2
9-1
9-2
EMX2::Nestin::DAPI
EMX2/DAPI
%
100
80
60
40
20
0
scz
co twin
control

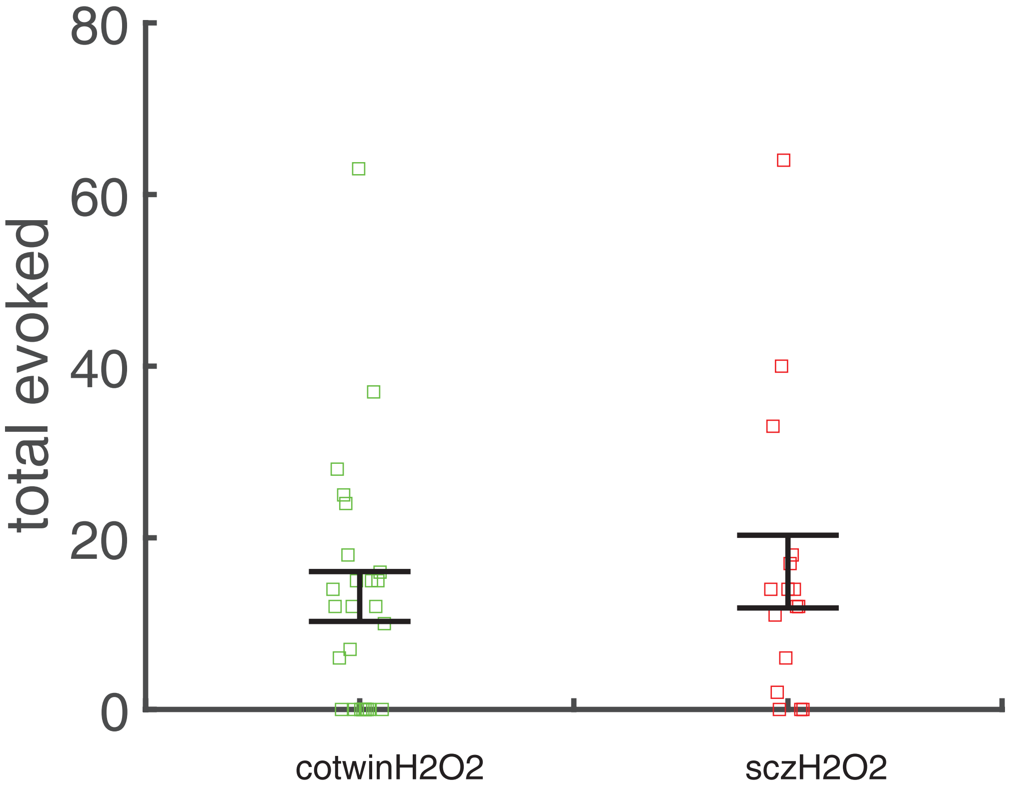

80
60
40
20
0
total evoked
cotwinH2O2
sczH2O2

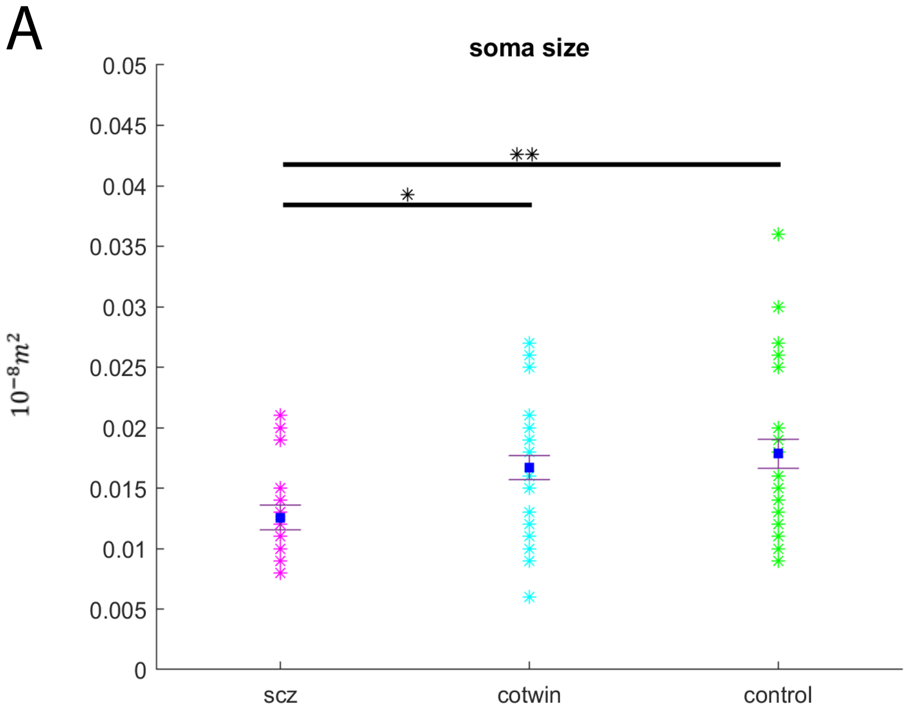
A
soma size
$10^{-8}m^2$
0.05
0.045
0.04
0.035
0.03
0.025
0.02
0.015
0.01
0.005
0
**
*
scz
cotwin
control

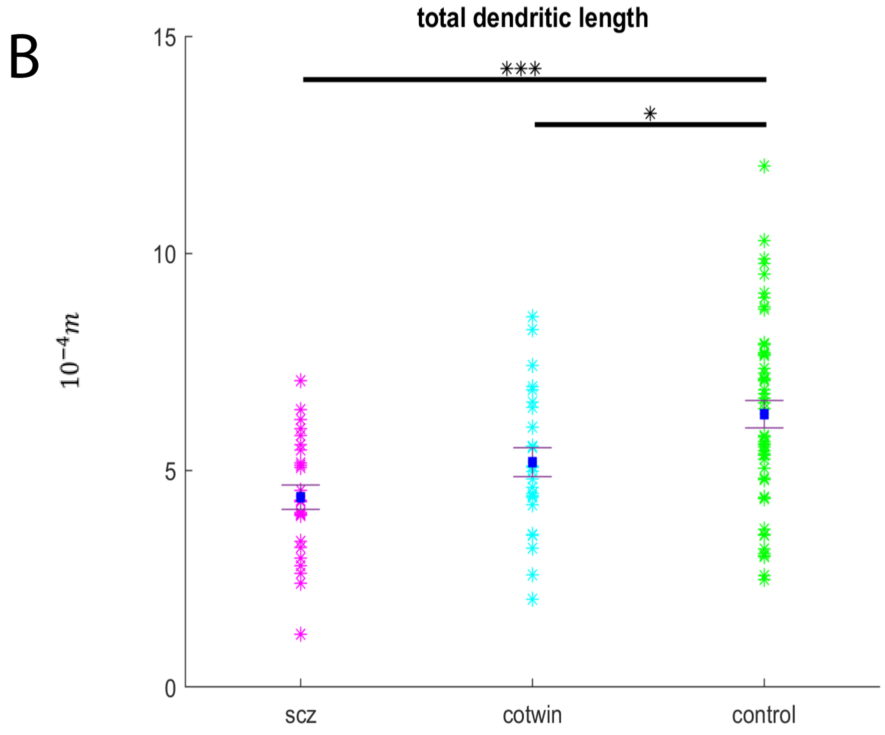
B
total dendritic length
$10^{-4}m$
15
10
5
0
***
*
scz
cotwin
control

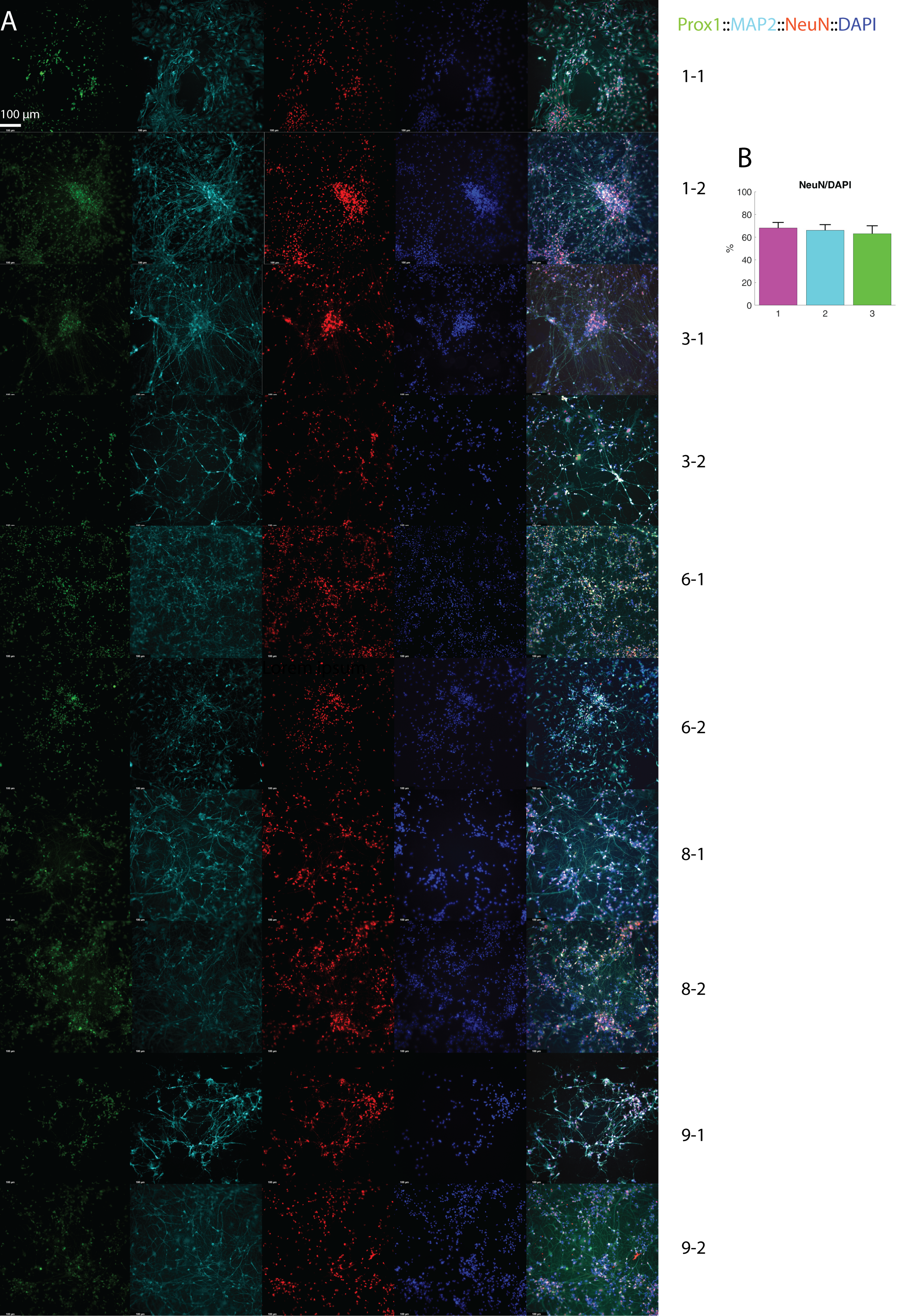
A
100 µm
Prox1::MAP2::NeuN::DAPI
1-1
1-2
3-1
3-2
6-1
6-2
8-1
8-2
9-1
9-2
Lorem ipsum
B
NeuN/DAPI
100
80
60
40
20
0
%
1
2
3

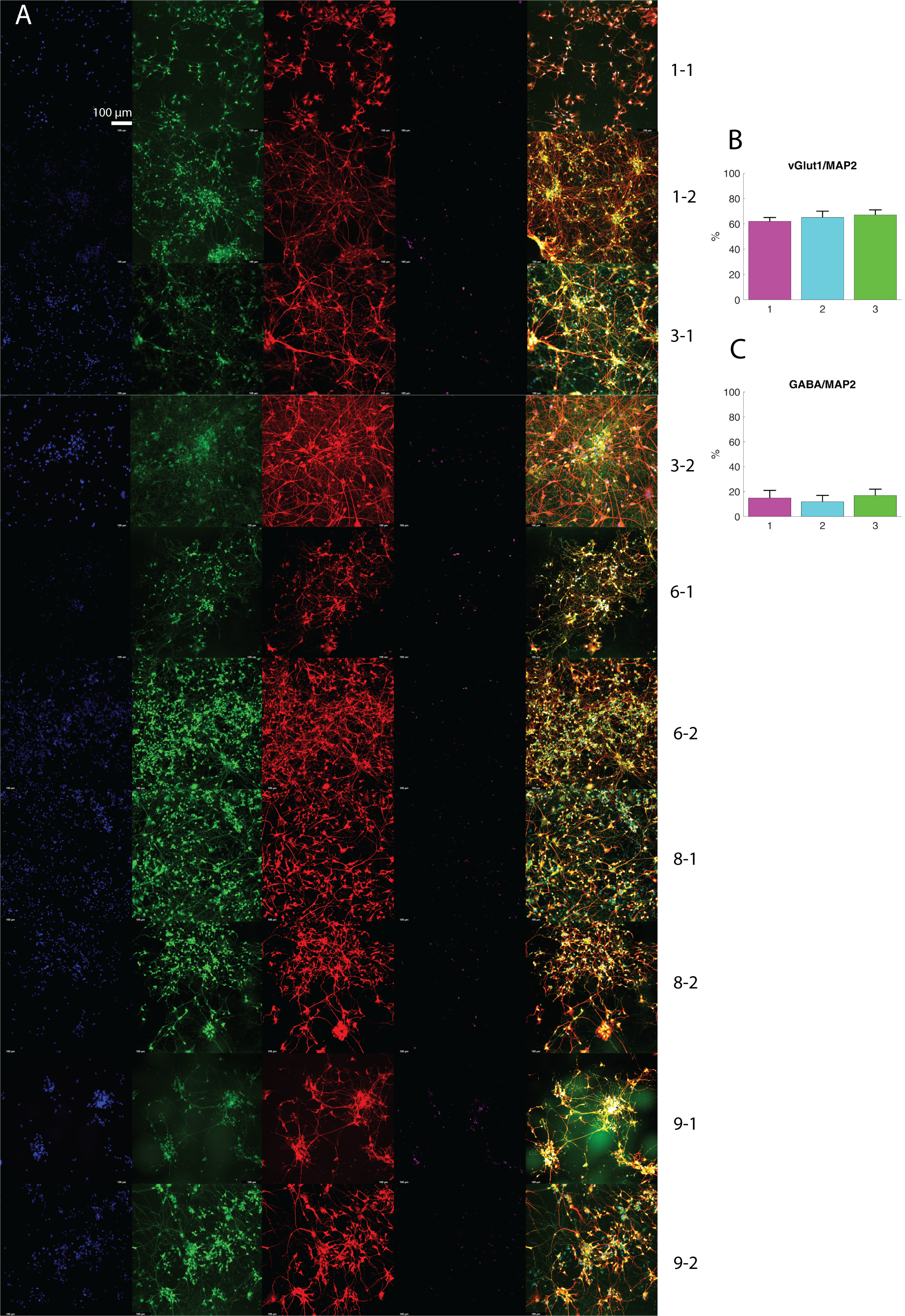
A
100 µm
1-1
1-2
3-1
3-2
6-1
6-2
8-1
8-2
9-1
9-2
B
vGlut1/MAP2
100
80
60
40
20
0
%
1
2
3
C
GABA/MAP2
100
80
60
40
20
0
%
1
2
3

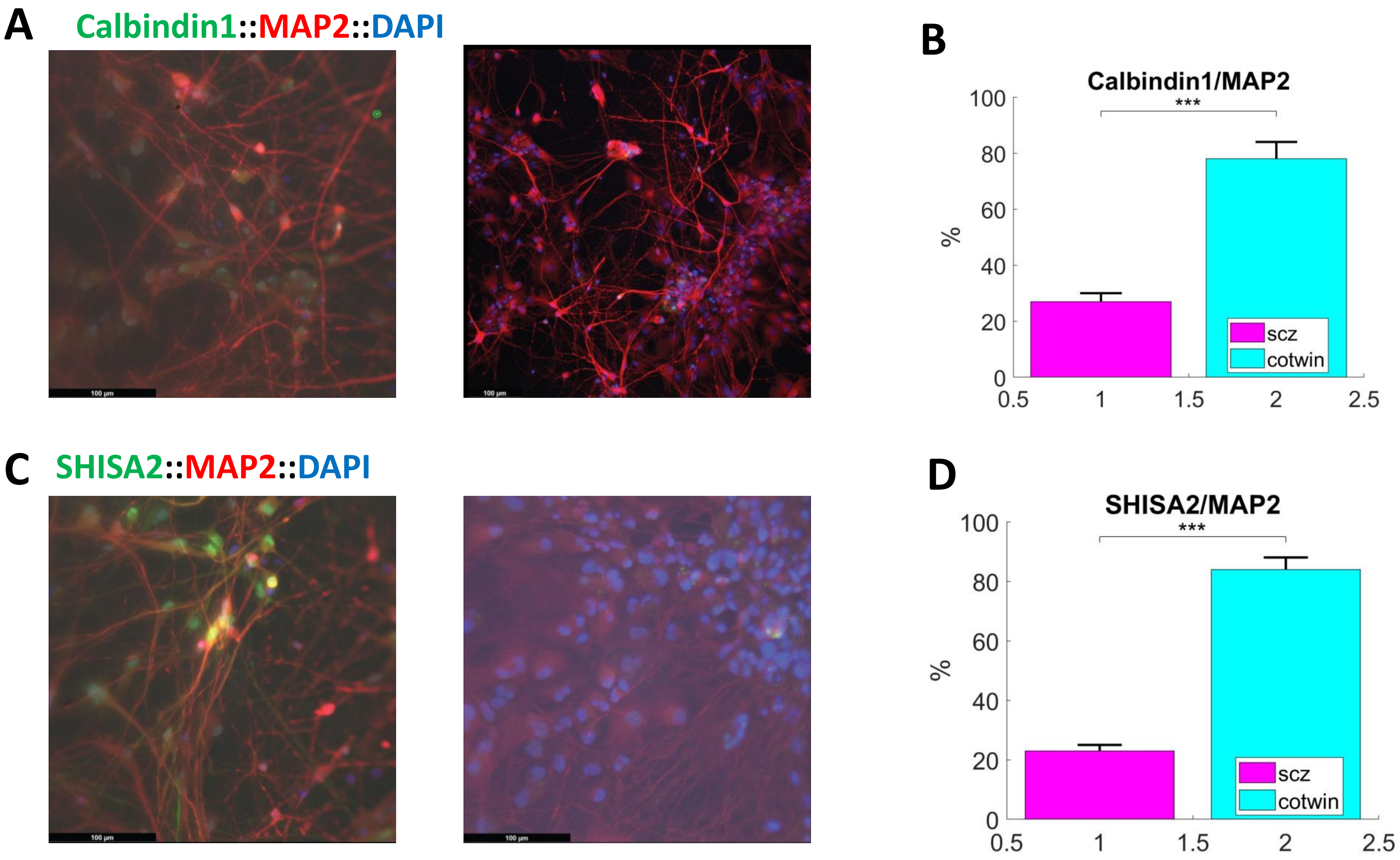
A
Calbindin1::MAP2::DAPI
B
Calbindin1/MAP2
***
%
100
80
60
40
20
0
0.5
1
1.5
2
2.5
scz
cotwin
C
SHISA2::MAP2::DAPI
D
SHISA2/MAP2
***
%
100
80
60
40
20
0
0.5
1
1.5
2
2.5
scz
cotwin

WiCell

Chromosome Analysis Report: 012533

Date Reported: Wednesday, November 13, 2013

Cell Line: MZ Twin 8.1 c6

Passage#: 5

Date of Sample: 11/5/2013

Specimen: iPSC

Results: 46,XX[19]
Nonclonal finding: 47,XX,+2

Cell Line Gender: Female

Reason for Testing: Normal KT

Investigator: Carl Dargitz, Salk

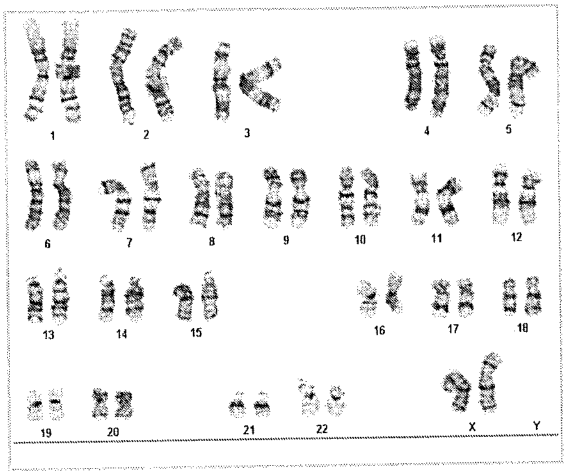

Cell: 31

Slide: 1

Slide Type: Karyotype

Total Counted: 20

Total Analyzed: 8

Total Karyotyped: 4

Band Resolution: 425 - 500

Interpretation:

This is a normal karyotype. No clonal abnormalities were detected at the stated band level of resolution.

There is one nonclonal finding, listed above. Nonclonal findings likely result from technical artifact, but may be due to a developing clonal abnormality or to low-level mosaicism.

Completed by: Erik McIntire, CG(ASCP)

Reviewed and Interpreted by: Karen Dyer Montgomery, PhD, FACMG

A signed copy of this report is available upon request.

Date:__________ Sent By:____ Sent To:__________ QC Review By: ____

Limitations: This assay allows for microscopic visualization of numerical and structural chromosome abnormalities. The size of structural abnormality that can be detected is >3-10Mb, dependent upon the G-band resolution obtained from this specimen. For the purposes of this report, band level is defined as the number of G-bands per haploid genome. It is documented here as "band level", i.e., the range of bands determined from the four karyograms in this assay. Detection of heterogeneity of clonal cell populations in this specimen (i.e.,mosaicism) is limited by the number of metaphase cells examined, documented here as "# of cells counted".

This assay was conducted solely for listed investigator/institution. The results may not be relied upon by any other party without the prior written consent of the Director of the WiCell Cytogenetics Laboratory. The results of this assay are for research use only. If the results of this assay are to be used for any other purpose, contact the Director of the WiCell Cytogenetics Laboratory.

Chromosome Analysis Report: 011420

Date Reported: Wednesday, August 14, 2013
Cell Line: MZ-Twin-8.2 c3
Passage#: 5
Date of Sample: 8/6/2013
Specimen: iPSC
Results: 46,XX

Cell Line Gender: Female
Reason for Testing: Normal KT

Investigator: Ken Diffenderfer, Salk Institute

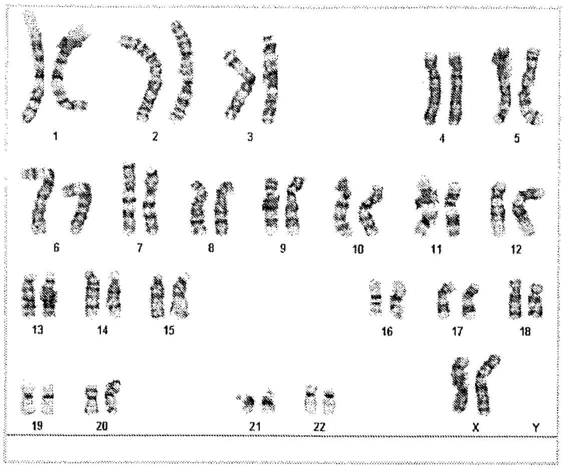

Cell: 18
Slide: 1
Slide Type: Karyotype

Total Counted: 20
Total Analyzed: 8
Total Karyotyped: 4
Band Resolution: 425 - 475

Interpretation:

This is a normal karyotype. No clonal abnormalities were detected at the stated band level of resolution.

Completed by: Kim Leonhard, CG(ASCP)
Reviewed and Interpreted by: Karen Dyer Montgomery, PhD, FACMG

A signed copy of this report is available upon request.

Date:________________________ Sent By:____ Sent To:______________________ QC Review By: ____

Limitations: This assay allows for microscopic visualization of numerical and structural chromosome abnormalities. The size of structural abnormality that can be detected is >3-10Mb, dependent upon the G-band resolution obtained from this specimen. For the purposes of this report, band level is defined as the number of G-bands per haploid genome. It is documented here as "band level", i.e., the range of bands determined from the four karyograms in this assay. Detection of heterogeneity of clonal cell populations in this specimen (i.e.,mosaicism) is limited by the number of metaphase cells examined, documented here as "# of cells counted".

This assay was conducted solely for listed investigator/institution. The results may not be relied upon by any other party without the prior written consent of the Director of the WiCell Cytogenetics Laboratory. The results of this assay are for research use only. If the results of this assay are to be used for any other purpose, contact the Director of the WiCell Cytogenetics Laboratory.

Chromosome Analysis Report: 011395

Date Reported: Friday, August 09, 2013
Cell Line: MZ-Twin-9.2 c6
Passage#: 5
Date of Sample: 8/1/2013
Specimen: iPSC
Results: 46,XX

Cell Line Gender: Female
Reason for Testing: normal KT

Investigator: Ken Diffenderfer, Salk Institute

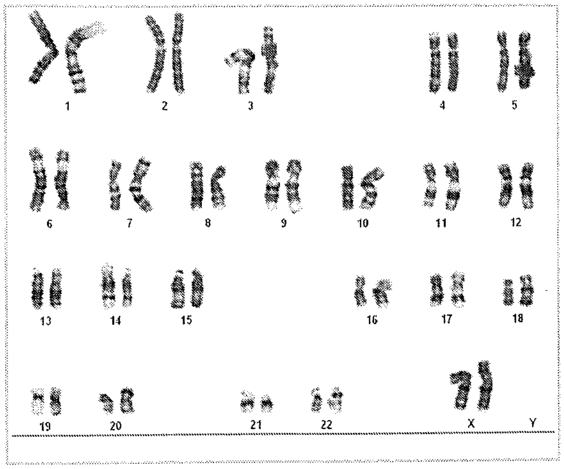

Cell: 18
Slide: 1
Slide Type: Karyotype

Total Counted: 20
Total Analyzed: 8
Total Karyotyped: 4
Band Resolution: 400 - 450

Interpretation:

This is a normal karyotype. No clonal abnormalities were detected at the stated band level of resolution.

Completed by: Erica Schutter, CG(ASCP)
Reviewed and Interpreted by: Karen Dyer Montgomery, PhD, FACMG
A signed copy of this report is available upon request.

Date:________________________ Sent By:____ Sent To:_____________________ QC Review By: ____

Limitations: This assay allows for microscopic visualization of numerical and structural chromosome abnormalities. The size of structural abnormality that can be detected is >3-10Mb, dependent upon the G-band resolution obtained from this specimen. For the purposes of this report, band level is defined as the number of G-bands per haploid genome. It is documented here as "band level", i.e., the range of bands determined from the four karyograms in this assay. Detection of heterogeneity of clonal cell populations in this specimen (i.e.,mosaicism) is limited by the number of metaphase cells examined, documented here as "# of cells counted".

This assay was conducted solely for listed investigator/institution. The results may not be relied upon by any other party without the prior written consent of the Director of the WiCell Cytogenetics Laboratory. The results of this assay are for research use only. If the results of this assay are to be used for any other purpose, contact the Director of the WiCell Cytogenetics Laboratory.

Chromosome Analysis Report: 014357

Date Reported: Monday, June 16, 2014
Cell Line: 3/2 cl. 24
Passage#: 7
Date of Sample: 6/6/2014
Specimen: iPSC
Results: 46,XX

Cell Line Gender: Female
Reason for Testing: new cell line

Investigator: Ana Paula Diniz Mendes, Salk Institute

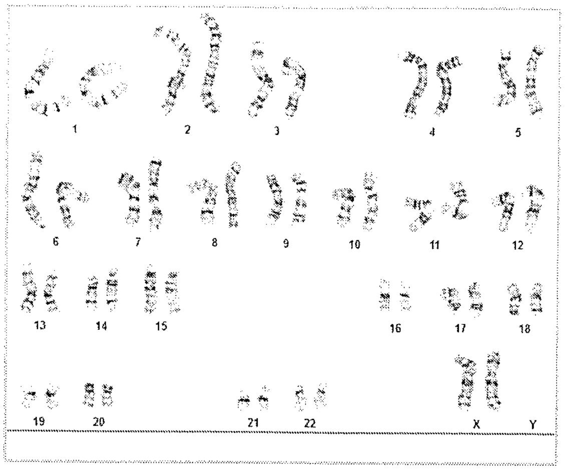

Cell: 43
Slide: 2
Slide Type: Karyotype

Total Counted: 20
Total Analyzed: 8
Total Karyotyped: 4
Band Resolution: 425 - 475

Interpretation:

This is a normal karyotype. No clonal abnormalities were detected at the stated band level of resolution.

Completed by: Kim Leonhard, CG(ASCP)
Reviewed and Interpreted by: Karen Dyer Montgomery, PhD, FACMG
A signed copy of this report is available upon request.

Date:________________________ Sent By:____ Sent To:______________________ QC Review By: ____

Limitations: This assay allows for microscopic visualization of numerical and structural chromosome abnormalities. The size of structural abnormality that can be detected is >3-10Mb, dependent upon the G-band resolution obtained from this specimen. For the purposes of this report, band level is defined as the number of G-bands per haploid genome. It is documented here as "band level", i.e., the range of bands determined from the four karyograms in this assay. Detection of heterogeneity of clonal cell populations in this specimen (i.e.,mosaicism) is limited by the number of metaphase cells examined, documented here as "# of cells counted".

This assay was conducted solely for listed investigator/institution. The results may not be relied upon by any other party without the prior written consent of the Director of the WiCell Cytogenetics Laboratory. The resu lts of this assay are for research use only. If the results of this assay are to be used for any other purpose, contact the Director of the WiCell Cytogenetics Laboratory.

Unless otherwise mutually agreed in writing, the services provided to you hereunder by WiCell Research Institute, Inc. ("WiCell") are governed solely by WiCell's Terms and Conditions of Service, found at www.wicell.org/privacyandterms. Any terms you may attach to a purchase order or other document that are inconsistent, add to, or conflict with WiCell's Terms and Conditions of Service are null and void and of no legal force or effect.

Chromosome Analysis Report: 014356

Date Reported: **Monday, June 16, 2014**
Cell Line: **1/2 cl. 1**
Passage#: **7**
Date of Sample: **6/6/2014**
Specimen: **iPSC**
Results: **46,XY**

Cell Line Gender: **Male**
Reason for Testing: **new cell line**

Investigator: **Ana Paula Diniz Mendes, Salk Institute**

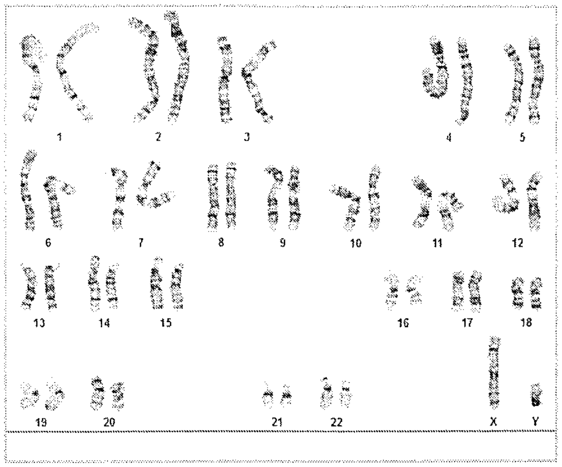

Cell: **25**
Slide: **1**
Slide Type: **Karyotype**

Total Counted: **20**
Total Analyzed: **10**
Total Karyotyped: **4**
Band Resolution: **425 - 500**

Interpretation:

This is a normal karyotype. No clonal abnormalities were detected at the stated band level of resolution.

Completed by: **Kim Leonhard, CG(ASCP)**
Reviewed and Interpreted by: **Karen Dyer Montgomery, PhD, FACMG**
A signed copy of this report is available upon request.

*Date:*______________________ *Sent By:*____ *Sent To:*____________________ *QC Review By:* ____

Limitations: This assay allows for microscopic visualization of numerical and structural chromosome abnormalities. The size of structural abnormality that can be detected is >3-10Mb, dependent upon the G-band resolution obtained from this specimen. For the purposes of this report, band level is defined as the number of G-bands per haploid genome. It is documented here as "band level", i.e., the range of bands determined from the four karyograms in this assay. Detection of heterogeneity of clonal cell populations in this specimen (i.e.,mosaicism) is limited by the number of metaphase cells examined, documented here as "# of cells counted".

This assay was conducted solely for listed investigator/institution. The results may not be relied upon by any other party without the prior written consent of the Director of the WiCell Cytogenetics Laboratory. The resu lts of this assay are for research use only. If the results of this assay are to be used for any other purpose, contact the Director of the WiCell Cytogenetics Laboratory.

Unless otherwise mutually agreed in writing, the services provided to you hereunder by WiCell Research Institute, Inc. ("WiCell") are governed solely by WiCell's Terms and Conditions of Service, found at www.wicell.org/privacyandterms. Any terms you may attach to a purchase order or other document that are inconsistent, add to, or conflict with WiCell's Terms and Conditions of Service are null and void and of no legal force or effect.

Chromosome Analysis Report: 014392

Date Reported: Thursday, June 19, 2014
Cell Line: 1/2 Clone 4
Passage#: 8
Date of Sample: 6/12/2014
Specimen: iPSC
Results: 46,XY

Cell Line Gender: Male
Reason for Testing: New cell line

Investigator: Ana Diniz Mendes, Salk Institute

Nonclonal findings: 47,XY,+8

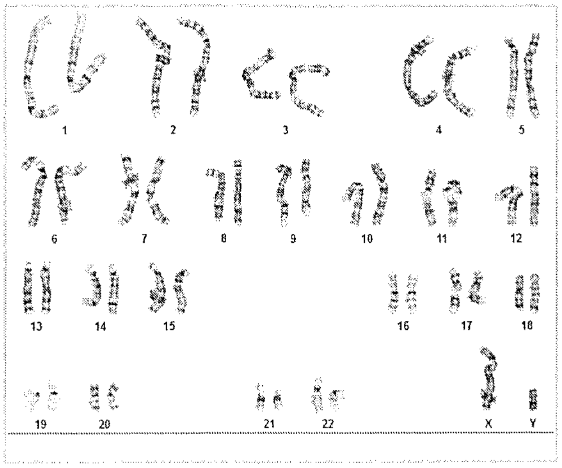

Cell: 28
Slide: 5
Slide Type: Karyotype

Total Counted: 40
Total Analyzed: 8
Total Karyotyped: 4
Band Resolution: 450 - 525

Interpretation:

This is a normal karyotype. No clonal abnormalities were detected at the stated band level of resolution.

There is one nonclonal finding, listed above. Standard analysis requires that chromosomes are counted in twenty cells. Twenty additional cells were examined with no further evidence of this nonclonal aberration. Nonclonal findings likely result from technical artifact, but may be due to a developing clonal abnormality or to low-level mosaicism.

Completed by: Kim Leonhard, CG(ASCP)
Reviewed and Interpreted by: Karen Dyer Montgomery, PhD, FACMG
A signed copy of this report is available upon request.

Date:__________ Sent By:____ Sent To:__________ QC Review By: ____

Limitations: This assay allows for microscopic visualization of numerical and structural chromosome abnormalities. The size of structural abnormality that can be detected is >3-10Mb, dependent upon the G-band resolution obtained from this specimen. For the purposes of this report, band level is defined as the number of G-bands per haploid genome. It is documented here as "band level", i.e., the range of bands determined from the four karyograms in this assay. Detection of heterogeneity of clonal cell populations in this specimen (i.e.,mosaicism) is limited by the number of metaphase cells examined, documented here as "# of cells counted".

This assay was conducted solely for listed investigator/institution. The results may not be relied upon by any other party without the prior written consent of the Director of the WiCell Cytogenetics Laboratory. The resu lts of this assay are for research use only. If the results of this assay are to be used for any other purpose, contact the Director of the WiCell Cytogenetics Laboratory.

Unless otherwise mutually agreed in writing, the services provided to you hereunder by WiCell Research Institute, Inc. ("WiCell") are governed solely by WiCell's Terms and Conditions of Service, found at www.wicell.org/privacyandterms. Any terms you may attach to a purchase order or other document that are inconsistent, add to, or conflict with WiCell's Terms and Conditions of Service are null and void and of no legal force or effect.

Chromosome Analysis Report: 014358

Date Reported: Monday, June 16, 2014
Cell Line: 9/1 cl. 4
Passage#: 20
Date of Sample: 6/6/2014
Specimen: iPSC
Results: 46,XX

Cell Line Gender: Female
Reason for Testing: new cell line

Investigator: Ana Paula Diniz Mendes, Salk Institute

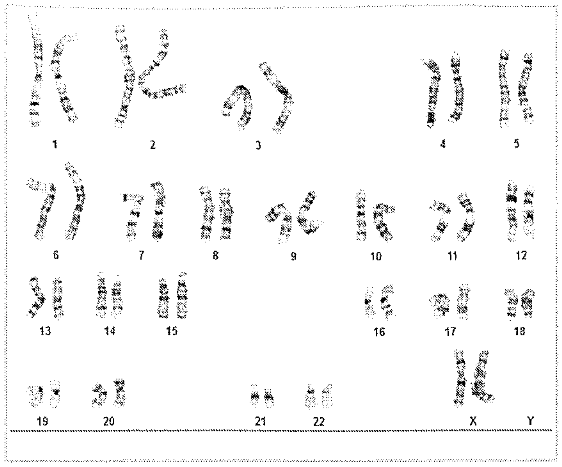

Cell: 19
Slide: 1
Slide Type: Karyotype

Total Counted: 20
Total Analyzed: 11
Total Karyotyped: 5
Band Resolution: 450 - 525

Interpretation:

This is a normal karyotype. No clonal abnormalities were detected at the stated band level of resolution.

Completed by: Kim Leonhard, CG(ASCP)
Reviewed and Interpreted by: Karen Dyer Montgomery, PhD, FACMG
A signed copy of this report is available upon request.

Date:________________________ Sent By:____ Sent To:______________________ QC Review By: ____

Limitations: This assay allows for microscopic visualization of numerical and structural chromosome abnormalities. The size of structural abnormality that can be detected is >3-10Mb, dependent upon the G-band resolution obtained from this specimen. For the purposes of this report, band level is defined as the number of G-bands per haploid genome. It is documented here as "band level", i.e., the range of bands determined from the four karyograms in this assay. Detection of heterogeneity of clonal cell populations in this specimen (i.e.,mosaicism) is limited by the number of metaphase cells examined, documented here as "# of cells counted".

This assay was conducted solely for listed investigator/institution. The results may not be relied upon by any other party without the prior written consent of the Director of the WiCell Cytogenetics Laboratory. The resu lts of this assay are for research use only. If the results of this assay are to be used for any other purpose, contact the Director of the WiCell Cytogenetics Laboratory.

Unless otherwise mutually agreed in writing, the services provided to you hereunder by WiCell Research Institute, Inc. ("WiCell") are governed solely by WiCell's Terms and Conditions of Service, found at www.wicell.org/privacyandterms. Any terms you may attach to a purchase order or other document that are inconsistent, add to, or conflict with WiCell's Terms and Conditions of Service are null and void and of no legal force or effect.

Chromosome Analysis Report: 010722

Date Reported: Wednesday, May 08, 2013
Cell Line: hIPSC 3d5c9ou3d2f3_d #6 (1/1/6)
Passage#: 8
Date of Sample: 4/26/2013
Specimen: iPSC
Results: 46,XY

Cell Line Gender: Male
Reason for Testing: new cell line
Investigator: Janos Reithelyi, Salk Institute

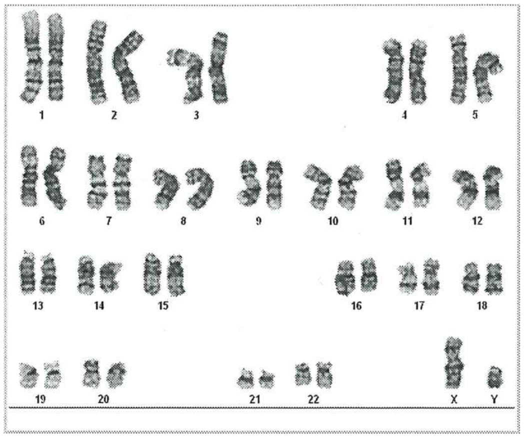

Cell: 22
Slide: 2
Slide Type: Karyotype

Total Counted: 20
Total Analyzed: 8
Total Karyotyped: 4
Band Resolution: 400 - 425

Interpretation:

This is a normal karyotype. No clonal abnormalities were detected at the stated band level of resolution.

Completed by: Kim Leonhard, CG(ASCP)
Reviewed and Interpreted by: Karen Dyer Montgomery, PhD, FACMG

A signed copy of this report is available upon request.

Date:________________ **Sent By:**____ **Sent To:**________________ **QC Review By:** ____

Limitations: This assay allows for microscopic visualization of numerical and structural chromosome abnormalities. The size of structural abnormality that can be detected is >3-10Mb, dependent upon the G-band resolution obtained from this specimen. For the purposes of this report, band level is defined as the number of G-bands per haploid genome. It is documented here as "band level", i.e., the range of bands determined from the four karyograms in this assay. Detection of heterogeneity of clonal cell populations in this specimen (i.e.,mosaicism) is limited by the number of metaphase cells examined, documented here as "# of cells counted".

This assay was conducted solely for listed investigator/institution. The results may not be relied upon by any other party without the prior written consent of the Director of the WiCell Cytogenetics Laboratory. The results of this assay are for research use only. If the results of this assay are to be used for any other purpose, contact the Director of the WiCell Cytogenetics Laboratory.

Chromosome Analysis Report: 010346

Date Reported: Wednesday, March 20, 2013
Cell Line: hIPSC 3d5c9ou3d2f3_d #2 (1/1/2)
Passage#: 9
Date of Sample: 3/15/2013
Specimen: iPSC
Results: 46,XY

Cell Line Gender: Male
Reason for Testing: new cell line
Investigator: Janos Rethelyi, Salk Institute

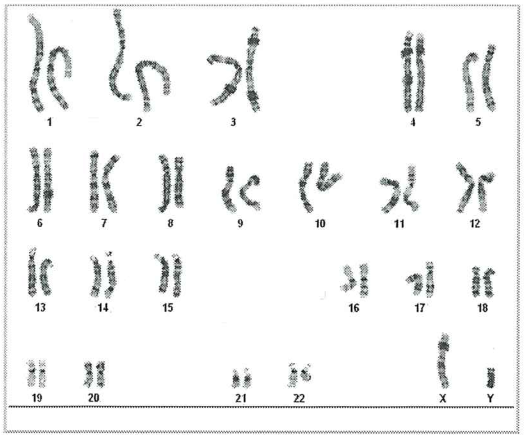

Cell: 33
Slide: 2
Slide Type: Karyotype

Total Counted: 20
Total Analyzed: 8
Total Karyotyped: 4
Band Resolution: 425 - 500

Interpretation:

This is a normal karyotype. No clonal abnormalities were detected at the stated band level of resolution.

Completed by: Kim Leonhard, CG(ASCP)
Reviewed and Interpreted by: Julie Leana Cox, PhD, FACMG

A signed copy of this report is available upon request.

Date:__________ **Sent By:**____ **Sent To:**__________ **QC Review By:** ____

Limitations: This assay allows for microscopic visualization of numerical and structural chromosome abnormalities. The size of structural abnormality that can be detected is >3-10Mb, dependent upon the G-band resolution obtained from this specimen. For the purposes of this report, band level is defined as the number of G-bands per haploid genome. It is documented here as "band level", i.e., the range of bands determined from the four karyograms in this assay. Detection of heterogeneity of clonal cell populations in this specimen (i.e.,mosaicism) is limited by the number of metaphase cells examined, documented here as "# of cells counted".

This assay was conducted solely for listed investigator/institution. The results may not be relied upon by any other party without the prior written consent of the Director of the WiCell Cytogenetics Laboratory. The results of this assay are for research use only. If the results of this assay are to be used for any other purpose, contact the Director of the WiCell Cytogenetics Laboratory.

Chromosome Analysis Report: 009793

Date Reported: Thursday, January 31, 2013
Cell Line: hIPSC 3e6eb0u050e5_d #D (6/2/D)
Passage#: 8
Date of Sample: 1/25/2013
Specimen: iPSC
Results: 46,XY

Cell Line Gender: Male
Reason for Testing: new cell line
Investigator: Janos Rethelyi, Salk Institute

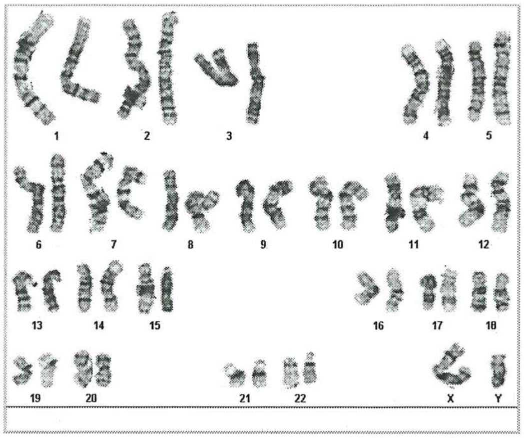

Cell: 22
Slide: 4
Slide Type: Karyotype

Total Counted: 20
Total Analyzed: 8
Total Karyotyped: 4
Band Resolution: 350 - 450

Interpretation:

This is a normal karyotype. No clonal abnormalities were detected at the stated band level of resolution.

Completed by: Kim Leonhard, CG(ASCP)
Reviewed and Interpreted by: Karen Dyer Montgomery, PhD, FACMG

A signed copy of this report is available upon request.

Date:____________________ **Sent By:**____ **Sent To:**____________________ **QC Review By:** ____

Limitations: This assay allows for microscopic visualization of numerical and structural chromosome abnormalities. The size of structural abnormality that can be detected is >3-10Mb, dependent upon the G-band resolution obtained from this specimen. For the purposes of this report, band level is defined as the number of G-bands per haploid genome. It is documented here as "band level", i.e., the range of bands determined from the four karyograms in this assay. Detection of heterogeneity of clonal cell populations in this specimen (i.e.,mosaicism) is limited by the number of metaphase cells examined, documented here as "# of cells counted".

This assay was conducted solely for listed investigator/institution. The results may not be relied upon by any other party without the prior written consent of the Director of the WiCell Cytogenetics Laboratory. The results of this assay are for research use only. If the results of this assay are to be used for any other purpose, contact the Director of the WiCell Cytogenetics Laboratory.

Chromosome Analysis Report: 009658

Date Reported: Thursday, January 10, 2013
Cell Line: hIPSC 8677d8u3fbf7_d #B (3/2/B)
Passage#: 11
Date of Sample: 1/3/2013
Specimen: iPSC
Results: 46,XX

Cell Line Gender: Female
Reason for Testing: new cell line. Resubmission

Investigator: Janos Rethelyi, Salk Institute

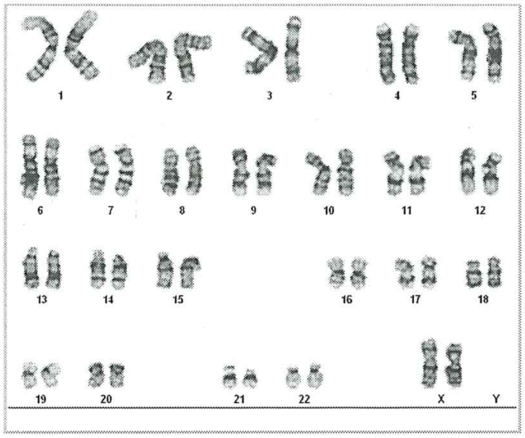

Cell: 16
Slide: 1
Slide Type: Karyotype

Total Counted: 20
Total Analyzed: 8
Total Karyotyped: 4
Band Resolution: 400 - 450

Interpretation:

This is a normal karyotype. No clonal abnormalities were detected at the stated band level of resolution.

Completed by: Kim Leonhard, CG(ASCP)
Reviewed and Interpreted by: Karen Dyer Montgomery, PhD, FACMG

A signed copy of this report is available upon request.

Date:____________________ Sent By:____ Sent To:____________________ QC Review By: ____

Limitations: This assay allows for microscopic visualization of numerical and structural chromosome abnormalities. The size of structural abnormality that can be detected is >3-10Mb, dependent upon the G-band resolution obtained from this specimen. For the purposes of this report, band level is defined as the number of G-bands per haploid genome. It is documented here as "band level", i.e., the range of bands determined from the four karyograms in this assay. Detection of heterogeneity of clonal cell populations in this specimen (i.e.,mosaicism) is limited by the number of metaphase cells examined, documented here as "# of cells counted".

This assay was conducted solely for listed investigator/institution. The results may not be relied upon by any other party without the prior written consent of the Director of the WiCell Cytogenetics Laboratory. The results of this assay are for research use only. If the results of this assay are to be used for any other purpose, contact the Director of the WiCell Cytogenetics Laboratory.

WiCell

Chromosome Analysis Report: 009660

Date Reported: Wednesday, January 09, 2013

Cell Line Gender: Female

Cell Line: hIPSC 2aea5cu3fbf7_d #1 (3/1/1)

Reason for Testing: new cell line. Resubmission

Passage#: 12

Date of Sample: 1/3/2013

Investigator: Janos Rethelyi, Salk Institute

Specimen: iPSC

Results: 46,XX

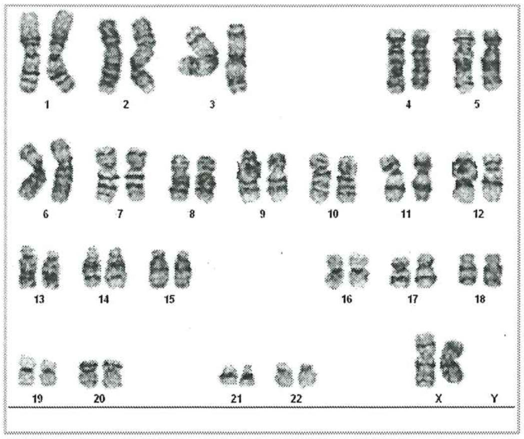

Cell: 4

Slide: 2

Slide Type: Karyotype

Total Counted: 20

Total Analyzed: 8

Total Karyotyped: 4

Band Resolution: 400 - 450

Interpretation:

This is a normal karyotype. No clonal abnormalities were detected at the stated band level of resolution.

Completed by: Kim Leonhard, CG(ASCP)

Reviewed and Interpreted by: Julie Leana Cox, PhD, FACMG

A signed copy of this report is available upon request.

Date:______________________ **Sent By:**____ **Sent To:**____________________ **QC Review By:** ____

Limitations: This assay allows for microscopic visualization of numerical and structural chromosome abnormalities. The size of structural abnormality that can be detected is >3-10Mb, dependent upon the G-band resolution obtained from this specimen. For the purposes of this report, band level is defined as the number of G-bands per haploid genome. It is documented here as "band level", i.e., the range of bands determined from the four karyograms in this assay. Detection of heterogeneity of clonal cell populations in this specimen (i.e.,mosaicism) is limited by the number of metaphase cells examined, documented here as "# of cells counted".

This assay was conducted solely for listed investigator/institution. The results may not be relied upon by any other party without the prior written consent of the Director of the WiCell Cytogenetics Laboratory. The results of this assay are for research use only. If the results of this assay are to be used for any other purpose, contact the Director of the WiCell Cytogenetics Laboratory.

Chromosome Analysis Report: 009467

Date Reported: Friday, December 07, 2012
Cell Line: 2aeaJcu3fbf7_d #A (3/1/A)
Passage#: 9
Date of Sample: 11/30/2012
Specimen: iPSC
Results: 46,XX

Cell Line Gender: Female
Reason for Testing: new cell line

Investigator: Janos Rethelyi, Salk Institute

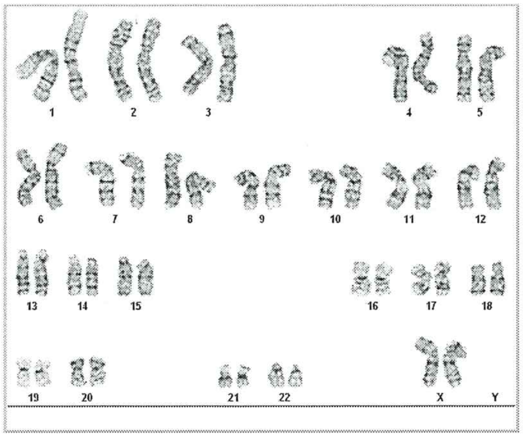

Cell: 17
Slide: 1
Slide Type: Karyotype

Total Counted: 20
Total Analyzed: 8
Total Karyotyped: 4
Band Resolution: 425 - 450

Interpretation:

This is a normal karyotype. No clonal abnormalities were detected at the stated band level of resolution.

Completed by: Erik McIntire, CG(ASCP)
Reviewed and Interpreted by: Karen Dyer Montgomery, PhD, FACMG

A signed copy of this report is available upon request.

Date:______________________ **Sent By:**____ **Sent To:**____________________ **QC Review By:** ____

Limitations: This assay allows for microscopic visualization of numerical and structural chromosome abnormalities. The size of structural abnormality that can be detected is >3-10Mb, dependent upon the G-band resolution obtained from this specimen. For the purposes of this report, band level is defined as the number of G-bands per haploid genome. It is documented here as "band level", i.e., the range of bands determined from the four karyograms in this assay. Detection of heterogeneity of clonal cell populations in this specimen (i.e.,mosaicism) is limited by the number of metaphase cells examined, documented here as "# of cells counted".

This assay was conducted solely for listed investigator/institution. The results may not be relied upon by any other party without the prior written consent of the Director of the WiCell Cytogenetics Laboratory. The results of this assay are for research use only. If the results of this assay are to be used for any other purpose, contact the Director of the WiCell Cytogenetics Laboratory.

Chromosome Analysis Report: 009426

Date Reported: Wednesday, November 28, 2012

Cell Line Gender: Male

Cell Line: 7b77adu050e5_d #1 (6/1/1)

Reason for Testing: New cell line

Passage#: 11

Date of Sample: 11/16/2012

Investigator: Janos Rethelyi, Salk Institute

Specimen: iPSC

Results: 46,XY

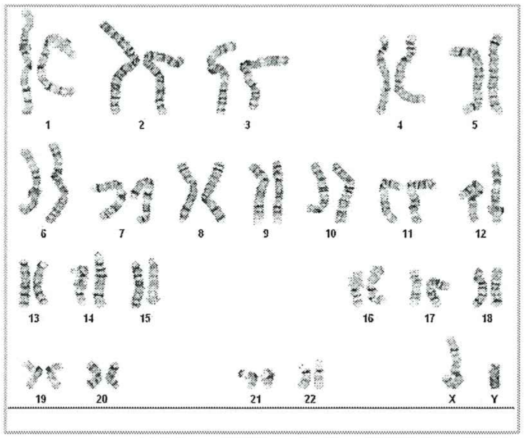

Cell: 12

Slide: 4

Slide Type: Karyotype

Total Counted: 20

Total Analyzed: 8

Total Karyotyped: 4

Band Resolution: 450 - 600

Interpretation:

This is a normal karyotype. No clonal abnormalities were detected at the stated band level of resolution.

Completed by: Kim Leonhard, CG(ASCP)

Reviewed and Interpreted by: Karen Dyer Montgomery, PhD, FACMG

A signed copy of this report is available upon request.

Date:_____________________ **Sent By:**____ **Sent To:**____________________ **QC Review By:** ____

Limitations: This assay allows for microscopic visualization of numerical and structural chromosome abnormalities. The size of structural abnormality that can be detected is >3-10Mb, dependent upon the G-band resolution obtained from this specimen. For the purposes of this report, band level is defined as the number of G-bands per haploid genome. It is documented here as "band level", i.e., the range of bands determined from the four karyograms in this assay. Detection of heterogeneity of clonal cell populations in this specimen (i.e.,mosaicism) is limited by the number of metaphase cells examined, documented here as "# of cells counted".

This assay was conducted solely for listed investigator/institution. The results may not be relied upon by any other party without the prior written consent of the Director of the WiCell Cytogenetics Laboratory. The results of this assay are for research use only. If the results of this assay are to be used for any other purpose, contact the Director of the WiCell Cytogenetics Laboratory.

schizophrenia vs. coTwin - Biological Pathways

Pathway	Enrichmen	nGenes	Pathway G	Fold Enrichment
regulation of anatomical structure size	0.000311	19	522	4.08251634
cell motility	0.000311	39	1806	2.422097909
localization of cell	0.000311	39	1806	2.422097909
phenol-containing compound metabolic process	0.000701	9	115	8.777877238
locomotion	0.000701	40	1992	2.252244271
cell migration	0.001273	34	1602	2.380461923
signal release	0.001378	17	498	3.828815261
regulation of ion transport	0.001378	21	745	3.16160679
positive regulation of developmental process	0.001378	31	1424	2.44172381
regulation of transport	0.001378	37	1874	2.21450656
response to endogenous stimulus	0.00151	34	1660	2.297289157
regulation of secretion by cell	0.001939	18	593	3.404572959
regulation of secretion	0.001939	19	650	3.278574661
positive regulation of cell population proliferation	0.002181	25	1052	2.665441176
regulation of blood pressure	0.002506	10	195	5.75188537
ear development	0.002506	11	239	5.162256953
circulatory system development	0.002801	26	1150	2.535831202
regulation of catecholamine secretion	0.002902	6	59	11.40628116
catecholamine secretion	0.002902	6	60	11.21617647
neural crest cell migration	0.002902	6	60	11.21617647
monoamine transport	0.002902	7	90	8.72369281
regulated exocytosis	0.003075	11	254	4.857399259
cell-cell signaling	0.003075	34	1777	2.146032639
inner ear development	0.003158	10	210	5.341036415
regulation of multicellular organismal development	0.003317	30	1483	2.268950061
modulation of chemical synaptic transmission	0.003435	15	474	3.549422934
regulation of trans-synaptic signaling	0.003435	15	475	3.541950464
export from cell	0.003435	22	915	2.696785599
positive regulation of heart contraction	0.003446	5	40	14.02022059
cation transport	0.003446	27	1274	2.377054668
secretion by cell	0.003516	21	857	2.748421306
positive regulation of blood circulation	0.003593	5	41	13.67826399
regulation of amine transport	0.003593	7	100	7.851323529
secretion	0.003593	23	1001	2.577143445

blood vessel development	0.003655	19	734	2.903369931
ion transport	0.003745	32	1677	2.140236417
cell junction organization	0.003789	19	739	2.883726021
regulation of developmental growth	0.003881	12	327	4.116028063
circulatory system process	0.003881	17	615	3.100406504
blood vessel diameter maintenance	0.003975	8	143	6.274784039
regulation of tube diameter	0.003975	8	143	6.274784039
regulation of cellular component size	0.003975	13	383	3.807057288
regulation of tube size	0.003995	8	144	6.23120915
enzyme linked receptor protein signaling pathway	0.003995	23	1025	2.516800574
ameboidal-type cell migration	0.004261	14	446	3.520772883
amine transport	0.00432	7	108	7.269744009
regulation of cell size	0.004563	9	191	5.285109332
vasculature development	0.004636	19	765	2.785717032
phenol-containing compound biosynthetic process	0.004675	5	47	11.93210263
regulation of chemotaxis	0.004774	10	241	4.654015133
regulation of anatomical structure morphogenesis	0.004774	22	975	2.530829563
gland development	0.00483	14	458	3.428525559
tube development	0.00483	24	1123	2.397045728
catecholamine transport	0.004913	6	78	8.627828054
positive regulation of heart rate	0.005398	4	26	17.25565611
forebrain development	0.00552	13	409	3.565043866
blood circulation	0.00552	15	527	3.192460096
cellular divalent inorganic cation homeostasis	0.005843	15	531	3.168411432
positive regulation of cell differentiation	0.006103	21	933	2.524541328
positive regulation of multicellular organismal process	0.006103	31	1687	2.061063845
vasoconstriction	0.00616	6	83	8.108079376
neural crest cell development	0.006479	6	84	8.011554622
response to gonadotropin	0.007217	4	29	15.47058824
thyroid gland development	0.007217	4	29	15.47058824
cellular calcium ion homeostasis	0.007684	14	489	3.211175268
sensory organ development	0.007684	16	614	2.922782142
mesenchymal cell development	0.008022	6	89	7.561467284
angiogenesis	0.008022	15	555	3.031399046
cellular response to endogenous stimulus	0.008022	27	1409	2.149302801
positive regulation of epithelial cell proliferation	0.008135	9	218	4.63053157
regulation of cytosolic calcium ion concentration	0.008135	12	377	3.570135747
divalent inorganic cation homeostasis	0.008135	15	558	3.015101202
transmembrane transport	0.008135	30	1653	2.035603715

cellular metal ion homeostasis	0.008313	16	625	2.871341176
stem cell development	0.008318	6	91	7.395281189
response to corticosteroid	0.008318	8	173	5.1866712
regulation of neurotransmitter secretion	0.008529	6	92	7.314897698
anterograde trans-synaptic signaling	0.008529	18	764	2.642554666
chemical synaptic transmission	0.008529	18	764	2.642554666
positive regulation of cytosolic calcium ion concentration involved in phospholipase C-activating G protein-coupled signaling pathway	0.00856	4	32	14.02022059
vascular process in circulatory system	0.009137	10	276	4.063832055
regulation of renal system process	0.009145	4	33	13.59536542
exocytosis	0.009145	12	388	3.468920558
calcium ion homeostasis	0.009145	14	508	3.091072256
trans-synaptic signaling	0.009145	18	773	2.611787535
regulation of exocytosis	0.009245	9	227	4.446942213
generation of neurons	0.009252	28	1522	2.063422741
olfactory bulb development	0.009897	4	34	13.19550173
pyramidal neuron migration to cerebral cortex	0.010006	2	3	74.7745098
blood vessel morphogenesis	0.010083	16	647	2.773706701
regulation of vasoconstriction	0.010089	5	63	8.901727358
neural crest cell differentiation	0.010089	6	98	6.867046819
cell junction assembly	0.010089	13	457	3.190597245
long-term synaptic potentiation	0.010548	6	99	6.797682709
positive regulation of cell motility	0.010776	15	588	2.861269508
olfactory lobe development	0.011077	4	36	12.4624183
regulation of vesicle-mediated transport	0.011077	15	591	2.846745297
response to growth factor	0.011077	17	726	2.62637741
regulation of locomotion	0.011077	22	1085	2.274247764
cellular chemical homeostasis	0.011119	19	867	2.457985616
plasma membrane bounded cell projection morphogenesis	0.011585	16	662	2.710858361
regulation of nervous system development	0.012013	13	470	3.102346683
striated muscle tissue development	0.012137	9	242	4.171305299
positive regulation of cellular component movement	0.012137	15	600	2.804044118
cell projection morphogenesis	0.012137	16	667	2.690537084
dopamine secretion	0.012511	4	38	11.80650155
regulation of dopamine secretion	0.012511	4	38	11.80650155
developmental growth involved in morphogenesis	0.012511	9	244	4.137114272
synaptic signaling	0.012511	18	810	2.49248366
positive regulation of locomotion	0.012568	15	605	2.780870199
phospholipase C-activating G protein-coupled receptor signaling pathway	0.013085	6	107	6.28944475
mammary gland development	0.013085	7	150	5.234215686
response to glucocorticoid	0.013085	7	150	5.234215686

potassium ion transport	0.013085	9	248	4.070386622
brain development	0.013085	18	816	2.474156574
regulation of cellular component movement	0.013085	22	1109	2.225030499
positive regulation of cell communication	0.013085	31	1830	1.900008036
regulation of cell motility	0.013138	21	1038	2.26916865
mesenchymal cell differentiation	0.013181	9	249	4.054039688
regulation of systemic arterial blood pressure by hormone	0.013316	4	40	11.21617647
neurotransmitter secretion	0.013316	7	152	5.165344427
signal release from synapse	0.013316	7	152	5.165344427
cell fate commitment	0.013316	10	306	3.665417147
epithelium development	0.013316	24	1271	2.117924747
neurogenesis	0.013316	30	1757	1.915112659
positive regulation of signaling	0.013316	31	1836	1.893798859
negative regulation of chemotaxis	0.013425	5	72	7.789011438
cell part morphogenesis	0.013425	16	686	2.616017836
cation transmembrane transport	0.013425	20	972	2.307855241
central nervous system development	0.013425	22	1123	2.197291918
regulation of regulated secretory pathway	0.013689	7	154	5.098262032
tube morphogenesis	0.013702	19	902	2.362609234
regulation of neurotransmitter transport	0.013744	6	111	6.062798092
regulation of cell migration	0.013744	20	977	2.296044313
mammary gland epithelial cell differentiation	0.013815	3	18	18.69362745
stabilization of membrane potential	0.013815	3	18	18.69362745
dopamine metabolic process	0.013815	4	42	10.68207283
positive regulation of animal organ morphogenesis	0.013815	4	42	10.68207283
mammary gland epithelium development	0.013815	5	74	7.578497615
regulation of extent of cell growth	0.013815	6	113	5.955491931
adenylate cyclase-modulating G protein-coupled receptor signaling pathway	0.013815	9	257	3.9278439
embryonic organ morphogenesis	0.013815	10	312	3.594928356
hormone secretion	0.013815	10	313	3.583442962
mesenchyme development	0.013815	10	313	3.583442962
neuron differentiation	0.013815	26	1449	2.012564446
cell projection organization	0.013815	28	1606	1.955497766
regulation of hormone levels	0.014071	14	562	2.794065313
synaptic vesicle exocytosis	0.01409	6	114	5.903250774
regulation of axonogenesis	0.01409	7	158	4.969192107
positive regulation of cell migration	0.01409	14	563	2.789102497
cellular cation homeostasis	0.014221	16	700	2.563697479

axon development	0.0147	13	501	2.910385112
regulation of renal sodium excretion	0.015027	3	19	17.70975232
specification of animal organ identity	0.015218	4	44	10.19652406
outflow tract morphogenesis	0.015218	5	77	7.283231474
regulation of behavior	0.015218	5	77	7.283231474
regulation of blood circulation	0.015218	9	264	3.823696524
cartilage development	0.015561	8	212	4.232519423
hormone transport	0.015561	10	322	3.483284618
kidney development	0.015561	10	322	3.483284618
cerebral cortex radially oriented cell migration	0.016101	4	45	9.969934641
cellular response to gonadotropin stimulus	0.016187	3	20	16.82426471
serotonin transport	0.016187	3	20	16.82426471
regulation of ion transmembrane transport	0.016187	13	511	2.853430413
regulation of plasma membrane bounded cell projection organization	0.016187	15	644	2.612463464
metal ion homeostasis	0.016187	16	714	2.513428901
muscle cell proliferation	0.016208	8	215	4.173461012
organic hydroxy compound transport	0.016252	9	269	3.752624098
neuron projection morphogenesis	0.016266	15	647	2.600350032
cellular ion homeostasis	0.016266	16	717	2.502912462
plasma membrane bounded cell projection organization	0.016266	27	1563	1.937535283
cellular component morphogenesis	0.016303	17	789	2.416666667
diencephalon development	0.016365	5	80	7.010110294
distal convoluted tubule development	0.016575	2	5	44.86470588
metanephric distal convoluted tubule development	0.016575	2	5	44.86470588
response to human chorionic gonadotropin	0.016575	2	5	44.86470588
head development	0.016575	18	865	2.33400204
axonogenesis	0.016669	12	452	2.977745966
regulation of system process	0.016782	14	584	2.688809428
mammary gland alveolus development	0.017019	3	21	16.02310924
mammary gland lobule development	0.017019	3	21	16.02310924
renal sodium excretion	0.017019	3	21	16.02310924
renal system development	0.017019	10	331	3.388572952
positive regulation of angiogenesis	0.017399	7	170	4.618425606
positive regulation of vasculature development	0.017399	7	170	4.618425606
neurotransmitter transport	0.017399	8	221	4.060154378
positive regulation of MAP kinase activity	0.017401	6	124	5.427182163
telencephalon development	0.017401	9	276	3.657448849
organic hydroxy compound metabolic process	0.017401	14	590	2.661465603
behavior	0.017401	15	659	2.552999197
metal ion transport	0.017401	19	949	2.245599083

regulation of cell projection organization	0.017575	15	660	2.549131016
regulation of cell population proliferation	0.017603	30	1835	1.833707325
animal organ morphogenesis	0.017833	21	1105	2.131581049
regulation of excretion	0.018372	3	22	15.2947861
cardiac muscle tissue development	0.018442	8	225	3.987973856
positive regulation of MAPK cascade	0.018442	13	528	2.761558601
adenylate cyclase-inhibiting G protein-coupled receptor signaling pathway	0.018823	5	85	6.597750865
axon extension	0.020199	6	129	5.216826265
chemotaxis	0.020199	15	672	2.503610819
developmental cell growth	0.02024	8	229	3.918314924
positive regulation of sprouting angiogenesis	0.020263	3	23	14.6297954
cellular homeostasis	0.020387	19	967	2.203798893
regulation of systemic arterial blood pressure mediated by a chemical signal	0.020443	4	51	8.797001153
taxis	0.020902	15	676	2.488796554
bundle of His development	0.020932	2	6	37.3872549
negative regulation of transmission of nerve impulse	0.020932	2	6	37.3872549
radial glia-guided pyramidal neuron migration	0.020932	2	6	37.3872549
sprouting angiogenesis	0.020932	6	131	5.137180063
neuron projection extension	0.021399	7	180	4.361846405
wound healing	0.02152	12	475	2.833560372
regulation of cell differentiation	0.021541	28	1700	1.847370242
regulation of endothelial cell migration	0.021784	7	181	4.337747806
sensory organ morphogenesis	0.022134	9	291	3.468920558
endothelial cell migration	0.022212	8	235	3.818272841
regulation of neurotransmitter levels	0.022212	8	235	3.818272841
response to lipid	0.022262	19	980	2.174564826
positive regulation of protein serine/threonine kinase activity	0.02261	8	236	3.802093719
ion transmembrane transport	0.022975	22	1218	2.025910364
ear morphogenesis	0.023094	6	135	4.98496732
peptide transport	0.023094	9	294	3.433523409
regulation of transmembrane receptor protein serine/threonine kinase signaling pathway	0.023445	9	295	3.421884347
forebrain neuron development	0.023527	3	25	13.45941176
dopamine transport	0.024282	4	55	8.157219251
forebrain generation of neurons	0.024282	4	55	8.157219251
positive regulation of fibroblast proliferation	0.024282	4	55	8.157219251
regulation of cardiac muscle tissue growth	0.024282	4	55	8.157219251

inorganic cation transmembrane transport	0.024282	17	840	2.269940476
cell morphogenesis	0.024664	20	1071	2.094524084
positive regulation of ion transport	0.024824	9	299	3.37610663
cellular response to growth factor stimulus	0.025053	15	697	2.413811292
adenylate cyclase-inhibiting serotonin receptor signaling pathway	0.025289	2	7	32.04621849
epinephrine secretion	0.025289	2	7	32.04621849
His-Purkinje system development	0.025289	2	7	32.04621849
metanephric distal tubule development	0.025289	2	7	32.04621849
positive regulation of protein kinase C activity	0.025289	2	7	32.04621849
regulation of epinephrine secretion	0.025289	2	7	32.04621849
regulation of protein kinase C activity	0.025289	2	7	32.04621849
sodium ion homeostasis	0.025289	4	56	8.011554622
axoneme assembly	0.025289	5	95	5.903250774
negative regulation of neuron projection development	0.025289	6	140	4.806932773
peptide hormone secretion	0.025289	8	244	3.677434908
positive regulation of intracellular signal transduction	0.025289	20	1078	2.080923278
regulation of growth	0.025425	15	702	2.396618904
cerebral cortex cell migration	0.025812	4	57	7.871001032
regulation of feeding behavior	0.026769	3	27	12.4624183
catecholamine metabolic process	0.026982	4	58	7.735294118
catechol-containing compound metabolic process	0.026982	4	58	7.735294118
regulation of neural precursor cell proliferation	0.026982	5	97	5.781534263
synapse assembly	0.026982	7	193	4.068043279
endocrine system development	0.027264	6	143	4.70608803
peptide secretion	0.027415	8	249	3.603590834
regulation of axon extension	0.027788	5	98	5.722539016
regulation of angiogenesis	0.02813	9	309	3.266847516
cardiac muscle tissue morphogenesis	0.028181	4	59	7.604187438
mammary gland epithelial cell proliferation	0.028182	3	28	12.01733193
negative regulation of axon extension involved in axon guidance	0.028182	3	28	12.01733193
positive regulation of cardiac muscle tissue growth	0.028182	3	28	12.01733193
positive regulation of neuroblast proliferation	0.028182	3	28	12.01733193
sperm axoneme assembly	0.028182	3	28	12.01733193
urogenital system development	0.02867	10	373	3.007017821
inorganic ion transmembrane transport	0.028921	18	942	2.143218434
regulation of systemic arterial blood pressure	0.029215	5	100	5.608088235

morphogenesis of a branching epithelium	0.029336	7	198	3.965314914
regulation of vasculature development	0.02937	9	313	3.225098666
regulation of MAPK cascade	0.02937	15	719	2.339953367
aggressive behavior	0.029526	2	8	28.04044118
cell proliferation in midbrain	0.029526	2	8	28.04044118
regulation of serotonin secretion	0.029526	2	8	28.04044118
activation of protein kinase B activity	0.029526	3	29	11.60294118
negative regulation of amine transport	0.029526	3	29	11.60294118
renal tubular secretion	0.029526	3	29	11.60294118
synaptic vesicle cycle	0.029526	7	199	3.945388708
regulation of membrane potential	0.029526	11	442	2.791356135
cation homeostasis	0.029526	16	794	2.260186694
regulation of neuron differentiation	0.029695	7	200	3.925661765
stem cell differentiation	0.030248	8	257	3.4914168
regulation of neuron projection development	0.030248	11	444	2.778782459
motile cilium assembly	0.030702	4	62	7.236242884
regulation of heart growth	0.030702	4	62	7.236242884
regulation of synaptic vesicle exocytosis	0.030702	4	62	7.236242884
cardiac atrium morphogenesis	0.03156	3	30	11.21617647
cilium movement	0.03156	7	203	3.867647059
cell morphogenesis involved in neuron differentiation	0.03156	13	585	2.49248366
regulation of G protein-coupled receptor signaling pathway	0.032635	6	152	4.42743808
inorganic ion homeostasis	0.033352	16	809	2.218279648
regulation of neurogenesis	0.0334	10	386	2.9057452
regulation of cell growth	0.034006	11	453	2.72357486
organ induction	0.034113	3	31	10.85436433
synapse organization	0.034346	11	454	2.717575797
epithelial cell proliferation	0.034808	11	455	2.711603103
hyaluronan catabolic process	0.035103	2	9	24.9248366
positive regulation of oxidoreductase activity	0.035103	4	65	6.902262443
regulation of heart rate	0.035103	5	107	5.241203958
regulation of transmembrane transport	0.035516	13	596	2.446481445
negative regulation of multicellular organismal process	0.035516	20	1130	1.985163977
positive regulation of cartilage development	0.035667	3	32	10.51516544
positive regulation of heart growth	0.035667	3	32	10.51516544
negative regulation of axonogenesis	0.035667	4	66	6.797682709
feeding behavior	0.035667	5	108	5.192674292
regulation of synapse assembly	0.035667	5	108	5.192674292
heart development	0.035667	13	598	2.438299233
ion homeostasis	0.035667	16	819	2.191194426
positive regulation of transport	0.035667	18	973	2.07493501
neural precursor cell proliferation	0.035703	6	157	4.286436868
positive regulation of kinase activity	0.035703	12	528	2.549131016

response to hormone	0.036027	17	898	2.123329621
morphogenesis of a branching structure	0.036829	7	212	3.703454495
MAPK cascade	0.038294	16	827	2.169997866
regulation of synaptic plasticity	0.03854	7	214	3.668842771
positive regulation of cation transmembrane transport	0.038573	6	160	4.206066176
cell morphogenesis involved in differentiation	0.038726	15	753	2.234298102
amide transport	0.039159	9	335	3.013301141
cardiac neural crest cell migration involved in outflow tract morphogenesis	0.039844	2	10	22.43235294
epinephrine transport	0.039844	2	10	22.43235294
regulation of postsynaptic cytosolic calcium ion concentration	0.039844	2	10	22.43235294
regulation of resting membrane potential	0.039844	2	10	22.43235294
regulation of sulfur metabolic process	0.039844	2	10	22.43235294
regulation of uterine smooth muscle contraction	0.039844	2	10	22.43235294
serotonin secretion	0.039844	2	10	22.43235294
positive regulation of Rho protein signal transduction	0.039844	3	34	9.896626298
regulation of axon extension involved in axon guidance	0.039844	3	34	9.896626298
transmembrane receptor protein serine/threonine kinase signaling pathway	0.039844	10	402	2.790093649
homeostatic process	0.040307	29	1916	1.697646752
glial cell migration	0.041161	4	70	6.409243697
muscle tissue morphogenesis	0.041161	4	70	6.409243697
inner ear morphogenesis	0.041461	5	114	4.919375645
vesicle-mediated transport in synapse	0.042399	7	220	3.568783422
regulation of cellular amine metabolic process	0.042506	3	35	9.613865546
response to oxygen-containing compound	0.043024	27	1752	1.728520346
regulation of cell junction assembly	0.043069	7	221	3.552635081
branching morphogenesis of an epithelial tube	0.043287	6	166	4.054039688
neuron projection development	0.043305	18	1001	2.01689487
response to wounding	0.043327	13	619	2.355578257
cerebral cortex radial glia-guided migration	0.043827	3	36	9.346813725
positive regulation of blood pressure	0.043827	3	36	9.346813725
positive regulation of vascular associated smooth muscle cell proliferation	0.043827	3	36	9.346813725
regulation of animal organ formation	0.043827	3	36	9.346813725
sperm flagellum assembly	0.043827	3	36	9.346813725

telencephalon glial cell migration	0.043827	3	36	9.346813725
cardiac muscle tissue growth	0.043827	4	72	6.23120915
cellular amine metabolic process	0.043827	5	117	4.793237808
potassium ion transmembrane transport	0.043827	7	223	3.520772883
connective tissue development	0.043827	8	283	3.170650592
embryonic organ development	0.043827	11	480	2.570373775
positive regulation of transferase activity	0.043827	13	622	2.344216947
positive regulation of developmental growth	0.043906	6	168	4.005777311
metanephric collecting duct development	0.044077	2	11	20.39304813
olfactory bulb interneuron differentiation	0.044077	2	11	20.39304813
penile erection	0.044077	2	11	20.39304813
serotonin metabolic process	0.044077	2	11	20.39304813
uterine smooth muscle contraction	0.044077	2	11	20.39304813
regulation of morphogenesis of an epithelium	0.044077	4	73	6.145850121
telencephalon cell migration	0.044077	4	73	6.145850121
regulation of synapse organization	0.045358	7	226	3.47403696
excretion	0.045902	3	37	9.094197138
response to mineralocorticoid	0.045902	3	37	9.094197138
epithelial cell development	0.046067	7	227	3.458732832
muscle tissue development	0.046071	10	417	2.689730568
cardiac chamber development	0.04612	6	171	3.935500516
animal organ formation	0.047475	4	75	5.981960784
regulation of muscle cell apoptotic process	0.047475	4	75	5.981960784
axon extension involved in axon guidance	0.048427	3	38	8.854876161
cardiac atrium development	0.048427	3	38	8.854876161
neuron projection extension involved in neuron projection guidance	0.048427	3	38	8.854876161
cardiac neural crest cell development involved in outflow tract morphogenesis	0.048645	2	12	18.69362745
cellular response to follicle-stimulating hormone stimulus	0.048645	2	12	18.69362745
distal tubule development	0.048645	2	12	18.69362745
dopamine biosynthetic process	0.048645	2	12	18.69362745
positive regulation of renal sodium excretion	0.048645	2	12	18.69362745
pyramidal neuron development	0.048645	2	12	18.69362745
short-term memory	0.048645	2	12	18.69362745
thrombin-activated receptor signaling pathway	0.048645	2	12	18.69362745
forebrain cell migration	0.048645	4	76	5.903250774
regulation of cartilage development	0.048645	4	76	5.903250774
regulation of organic acid transport	0.048645	4	76	5.903250774
amine metabolic process	0.048645	5	122	4.596793635

positive regulation of synaptic transmission	0.048645	6	175	3.845546218
regulation of synapse structure or activity	0.048645	7	232	3.384191176
positive regulation of nervous system development	0.048645	8	292	3.07292506
regulation of body fluid levels	0.048645	10	422	2.657861723
cellular response to oxygen-containing compound	0.048645	21	1272	1.851727248
muscle organ morphogenesis	0.049277	4	77	5.82658518
regulation of cardiac muscle cell proliferation	0.049543	3	39	8.627828054
positive regulation of ion transmembrane transport	0.049561	6	176	3.823696524

schizophrenia vs. co-Twin - Biological Pathways

Genes
SCIN SEMA3C EDN1 AVPR1A SEMA3E AGTR1 RIN3 F2RL1 XK FSTL4 WNT5A FN1 GCH1 HTR1B
SEMA3C FGF1 ASTN2 DISC1 TACR3 SEMA3E ITGBL1 CD44 EDN1 ANGPT2 CEMIP SMOC2 FN1 AGTR1 PLA2G7 F11R F2RL1 BMPER ERBB4 EPHA8 NR2E1 LAMA4 WNT5A SATB2 PTPRB FER GDF6 FSTL1
SEMA3C FGF1 ASTN2 DISC1 TACR3 SEMA3E ITGBL1 CD44 EDN1 ANGPT2 CEMIP SMOC2 FN1 AGTR1 PLA2G7 F11R F2RL1 BMPER ERBB4 EPHA8 NR2E1 LAMA4 WNT5A SATB2 PTPRB FER GDF6 FSTL1
TG GCH1 TYRP1 WNT5A PAX8 TPH2 SNCA TACR3 HTR1A
EPHA8 SEMA3C FGF1 ASTN2 FER DISC1 TACR3 SEMA3E ITGBL1 CD44 EDN1 ANGPT2 CEMIP SMOC2 FN1 AGTR1 PLA2G7 F11R F2RL1 BMPER ERBB4 NR2E1 LAMA4 WNT5A SATB2 PTPRB SNCA GDF6
SEMA3C FGF1 ASTN2 DISC1 SEMA3E ITGBL1 CD44 EDN1 ANGPT2 CEMIP SMOC2 FN1 AGTR1 PLA2G7 F11R F2RL1 BMPER ERBB4 EPHA8 NR2E1 LAMA4 WNT5A SATB2 PTPRB FER GDF6 FSTL1 CCDC141
SYN3 SYT3 CPLX3 EDN1 SNCA HTR1B ABCA12 CHRNB3 CHRNA6 ADRA2A F2RL1 MC4R HTR1A PAX8
VIP SYT3 SHISA9 EDN1 CEMIP SNCA KCNK6 KCNQ4 KCNK15 HTR1B CHRNA6 CACNA2D3 KCNC2
SOX6 EDN1 SMOC2 FGF1 NELL1 ZNF703 SYT3 ANGPT2 WNT2 NR2E1 WNT5A FN1 PAX8 SLC39A12 GDF6 DISC1 LRRTM1 LRRN3 FOXG1 ERBB4 NKX2-5 VWC2 MAP3K5 BMPER SCIN PIEZO1 SULT1E1
VIP FER SYT3 CPLX3 SHISA9 ADRA2A EDN1 CEMIP FCN3 ABCA12 SNCA F2RL1 KCNK6 WNT5A KCNQ4 SGIP1 KCNK15 HTR1B CHRNB3 CHRNA6 CACNA2D3 LRRTM1 KCNC2 AVPR1A HTR1A RGS7 NKX2-5
FGF1 HTR1B FSTL1 HTR1A VWC2 CALCR CD44 SOX6 SMOC2 PAX8 AGTR1 GDF6 ZNF703 IFT88 EPHA8 EDN1 NR2E1 WNT5A TICAM1 TPH2 FER BMPER GJB2 KCNC2 AVPR1A MC4R AQP4 SLC29A2 ERBB4
VIP FER SYT3 CPLX3 ADRA2A EDN1 F2RL1 HTR1B ABCA12 SNCA CHRNB3 CHRNA6 AVPR1A HTR1A
VIP FER SYT3 CPLX3 ADRA2A EDN1 F2RL1 HTR1B ABCA12 SNCA CHRNB3 CHRNA6 AVPR1A HTR1A
FGF1 OSMR VIP TGFA ERBB4 EDN1 WNT2 WNT5A FN1 AVPR1A ZNF703 FOSL2 NR2E1 TICAM1 DISC1 FOXG1 NKX2-5 HMX2 MAP3K5 AGTR1 HTR1B FER ID2 ADRA2A HTR1A
TRHDE EDN1 AVPR1A AGTR1 KCNK6 GCH1 NPY1R F2RL1 TACR3 ID2
IFT88 EDN1 CALB1 WNT5A KCNQ4 PAX8 BMPER GJB2 FOXG1 OTOL1 HMX2
ANGPT2 ISM1 FN1 PTPRB BMPER COL18A1 EDN1 SMOC2 FGF1 SEMA3C DSP WNT2 NR2E1 LAMA4 WNT5A PAX8 SLC39A12 TGFA SEMA3E ERBB4 NKX2-5 RBM20 AGTR1 SOX6 NPY1R ID2
VIP SYT3 HTR1B SNCA CHRNA6 ADRA2A
VIP SYT3 HTR1B SNCA CHRNA6 ADRA2A
SEMA3C SEMA3E FN1 EDN1 ERBB4 ALX1
VIP SYT3 SNCA HTR1B CHRNA6 HTR1A ADRA2A
FER SYT3 CPLX3 ADRA2A SNCA F2RL1 HTR1B CHRNB3 CHRNA6 ABCA12 SCIN
CALB1 WNT2 WNT5A HTR1B SNCA CHRNB3 CHRNA6 DKK2 PTCHD1 GJB2 HTR1A SYN3 SYT3 CPLX3 SHISA9 EDN1 NR2E1 ABCA12 ADRA2A DISC1 LRRTM1 F2RL1 MC4R NKX2-5 ZNF703 PAX8 TSHZ3 VIP
IFT88 CALB1 WNT5A KCNQ4 PAX8 BMPER GJB2 FOXG1 OTOL1 HMX2
SEMA3C ISM1 SOX6 BMPER SEMA3E EDN1 SMOC2 FGF1 NELL1 TG FSTL4 ANGPT2 WNT2 NR2E1 LAMA4 WNT5A FN1 PAX8 ABCA12 SLC39A12 DISC1 LRRTM1 LRRN3 FOXG1 ERBB4 NKX2-5 SCIN GDF6
CALB1 CPLX3 SHISA9 EDN1 NR2E1 HTR1B SNCA CHRNB3 CHRNA6 ADRA2A DISC1 LRRTM1 TSHZ3 FAM107A SYN3
CALB1 CPLX3 SHISA9 EDN1 NR2E1 HTR1B SNCA CHRNB3 CHRNA6 ADRA2A DISC1 LRRTM1 TSHZ3
VIP FER STXBP6 SYN3 ATP4B SYT3 CPLX3 ADRA2A EDN1 SNCA F2RL1 HTR1B ABCA12 CHRNB3
EDN1 GCH1 AVPR1A TACR3 NKX2-5
TRPC7 KCNK6 KCNQ4 KCNK15 KCNK1 VIP SLC39A12 KCNC2 ATP4B SYT3 SHISA9 EDN1 CEMIP SNCA ANO10 PIEZO1 SLC15A3 HTR1B CHRNA6 CACNA2D3 HTR1A RGS7 NKX2-5 CALCR GPR35 PLPP4
VIP FER STXBP6 SYN3 SYT3 CPLX3 ADRA2A EDN1 SNCA F2RL1 HTR1B ABCA12 CHRNB3 CHRNA6
EDN1 GCH1 AVPR1A TACR3 NKX2-5
VIP SYT3 SNCA HTR1B CHRNA6 AVPR1A ADRA2A
VIP FER STXBP6 SYN3 SYT3 CPLX3 ADRA2A EDN1 SNCA F2RL1 HTR1B ABCA12 CHRNB3 CHRNA6

ANGPT2 ISM1 PTPRB BMPER COL18A1 SMOC2 FGF1 SEMA3C EDN1 WNT2 NR2E1 LAMA4 FN1
TRPC7 KCNK6 KCNQ4 KCNK15 KCNK1 VIP CHRNB3 CHRNA6 SLC39A12 KCNC2 ATP4B SYT3 SHISA9 EDN1 CEMIP SNCA ANO10 TG PIEZO1 ABCC3 SLC15A3 HTR1B CACNA2D3 SLC22A15 AVPR1A HTR1A
FN1 SNCA SYNPO WNT5A GJB2 IL1RAPL2 DSP FER DISC1 LRRTM1 SEMA3E LRRN3 ERBB4 ZNF703
SEMA3C SEMA3E EDN1 SYT3 FSTL4 WNT2 WNT5A FN1 DISC1 NPY1R ERBB4 NKX2-5
TRHDE EDN1 AVPR1A AGTR1 KCNK6 GCH1 HTR1B ADRA2A NPY1R F2RL1 TACR3 HTR1A NKX2-5 DSP
EDN1 AVPR1A AGTR1 GCH1 HTR1B ADRA2A F2RL1 HTR1A
EDN1 AVPR1A AGTR1 GCH1 HTR1B ADRA2A F2RL1 HTR1A
SCIN SEMA3C SEMA3E EDN1 RIN3 F2RL1 XK FSTL4 WNT5A FN1 DISC1 AQP4 FER
EDN1 AVPR1A AGTR1 GCH1 HTR1B ADRA2A F2RL1 HTR1A
EPHA8 ANGPT2 FGF1 FER BMP3 GDF6 TGFA FSTL1 ERBB4 OTOL1 VWC2 SMOC2 SNCA IGSF1 ADRA2A BMPER ZNF703 FSTL4 WNT5A OSMR PPM1L ADAMTSL2 NGEF
SEMA3C FGF1 SEMA3E ANGPT2 SMOC2 FN1 BMPER EDN1 NR2E1 WNT5A FER FSTL1 ERBB4 ALX1
VIP SYT3 SNCA HTR1B CHRNA6 AVPR1A ADRA2A
SEMA3C SEMA3E EDN1 XK FSTL4 WNT5A FN1 DISC1 AQP4
ANGPT2 ISM1 PTPRB BMPER COL18A1 SMOC2 FGF1 SEMA3C EDN1 WNT2 NR2E1 LAMA4 FN1
GCH1 TYRP1 WNT5A TPH2 SNCA
SEMA3C SEMA3E ANGPT2 FGF1 PLA2G7 F2RL1 EDN1 WNT5A SMOC2 RIN3
SEMA3C ISM1 BMPER SEMA3E SMOC2 FGF1 F11R SYT3 XK FSTL4 EDN1 ANGPT2 WNT2 NR2E1 WNT5A FN1 PAX8 SLC39A12 DISC1 CD44 NGEF FGD5
ZNF703 SEMA3C EDN1 FGF1 WNT5A PAX8 TGFA SLC29A2 ERBB4 NKX2-5 TG WNT2 ID2 SOX3
ANGPT2 ISM1 FGF1 PTPRB BMPER COL18A1 SMOC2 SEMA3C EDN1 CALB1 WNT2 NR2E1 WNT5A FN1 PAX8 ABCA12 SLC39A12 TGFA GJB2 SEMA3E NKX2-5 ADAMTSL2 AGTR1 ID2
VIP SYT3 SNCA HTR1B CHRNA6 ADRA2A
EDN1 GCH1 AVPR1A TACR3
SEMA3E NR2E1 WNT5A DISC1 CCDC141 PTCHD1 KCNC2 AVPR1A FOXG1 ERBB4 RTN4RL1 ID2 SOX3
TRHDE EDN1 AVPR1A AGTR1 KCNK6 GCH1 HTR1B ADRA2A NPY1R F2RL1 TACR3 HTR1A NKX2-5 DSP
CALCR TRPC7 EDN1 CALB1 AGTR1 SLC39A12 F2RL1 GPR35 CEMIP WNT5A SNCA XK HTR1B DISC1 AVPR1A
SOX6 EDN1 NELL1 WNT2 NR2E1 FN1 PAX8 GDF6 DISC1 FOXG1 NKX2-5 ZNF703 VWC2 MAP3K5 SCIN
SOX6 TICAM1 VIP AVPR1A EDN1 SMOC2 FGF1 ADRA2A F2RL1 NELL1 ZNF703 ANGPT2 WNT2 NR2E1 WNT5A FN1 PAX8 GCH1 SLC39A12 GBP5 DISC1 LRRTM1 TACR3 LRRN3 FOXG1 ERBB4 NKX2-5 BMPER
EDN1 AVPR1A AGTR1 HTR1B ADRA2A HTR1A
SEMA3C SEMA3E FN1 EDN1 ERBB4 ALX1
PAX8 EPHA8 EDN1 GJB2
EDN1 PAX8 NKX2-5 TG
CALCR TRPC7 EDN1 CALB1 AGTR1 F2RL1 GPR35 CEMIP WNT5A SNCA XK HTR1B DISC1 AVPR1A
IFT88 CRYBA2 EDN1 CALB1 NR2E1 WNT5A KCNQ4 PAX8 BMPER GJB2 FOXG1 OTOL1 HMX2 LCTL
SEMA3C SEMA3E FN1 EDN1 ERBB4 ALX1
ANGPT2 ISM1 PTPRB BMPER COL18A1 SMOC2 FGF1 EDN1 NR2E1 FN1 SLC39A12 TGFA SEMA3E
FGF1 HTR1B FSTL1 HTR1A VWC2 CD44 SOX6 SMOC2 PAX8 AGTR1 GDF6 ZNF703 IFT88 EPHA8 EDN1 NR2E1 WNT5A FER BMPER GJB2 SLC29A2 ERBB4 ADAMTSL2 WNT2 ADRA2A FAM107A SNCA
FGF1 VIP TGFA ZNF703 WNT2 WNT5A NKX2-5 HMX2 AGTR1
CALCR TRPC7 CALB1 AGTR1 F2RL1 GPR35 EDN1 CEMIP WNT5A SNCA HTR1B AVPR1A
CALCR TRPC7 EDN1 CALB1 AGTR1 SLC39A12 F2RL1 GPR35 CEMIP WNT5A SNCA XK HTR1B DISC1
TRPC7 KCNK6 ABCC3 KCNQ4 KCNK15 KCNK1 CHRNB3 CHRNA6 SLC39A12 ANO10 KCNC2 ATP4B SHISA9 EDN1 CEMIP SLC15A3 SNCA GJB2 SLC29A2 PIEZO1 ABCA12 SLC35F4 CACNA2D3 SLC22A15

CALCR TRPC7 EDN1 CALB1 AGTR1 SLC39A12 F2RL1 GPR35 ATP4B CEMIP WNT5A SNCA XK HTR1B
SEMA3C SEMA3E FN1 EDN1 ERBB4 ALX1
CALCR EDN1 HTR1B TPH2 GJB2 AVPR1A AQP4 FAM107A
CPLX3 HTR1B SNCA CHRNB3 CHRNA6 ADRA2A
CALB1 HTR1B SNCA CHRNB3 CHRNA6 PTCHD1 HTR1A SYN3 CPLX3 SHISA9 EDN1 NR2E1 ADRA2A
CALB1 HTR1B SNCA CHRNB3 CHRNA6 PTCHD1 HTR1A SYN3 CPLX3 SHISA9 EDN1 NR2E1 ADRA2A
F2RL1 GPR35 AGTR1 EDN1
EDN1 AVPR1A AGTR1 GCH1 HTR1B ADRA2A F2RL1 HTR1A ABCC3 SLC29A2
EDN1 F2RL1 AVPR1A AGTR1
FER STXBP6 SYT3 CPLX3 ADRA2A SNCA F2RL1 HTR1B CHRNB3 CHRNA6 ABCA12 SCIN
CALCR TRPC7 EDN1 CALB1 AGTR1 F2RL1 GPR35 CEMIP WNT5A SNCA XK HTR1B DISC1 AVPR1A
CALB1 HTR1B SNCA CHRNB3 CHRNA6 PTCHD1 HTR1A SYN3 CPLX3 SHISA9 EDN1 NR2E1 ADRA2A
FER SYT3 ADRA2A F2RL1 HTR1B CHRNB3 CHRNA6 SNCA STXBP6
IFT88 EPHA8 SEMA3C WNT2 WNT5A NRK ASTN2 DISC1 SEMA3E SYT3 XK FSTL4 EDN1 NR2E1 FN1 SATB2 SLC39A12 GDF6 CCDC141 FEV FOXG1 ERBB4 RTN4RL1 VWC2 NKX2-5 NGEF ID2 SOX3
NR2E1 WNT5A ERBB4 ID2
DISC1 FOXG1
ANGPT2 ISM1 PTPRB BMPER COL18A1 SMOC2 FGF1 EDN1 NR2E1 FN1 SLC39A12 TGFA SEMA3E NKX2-
AVPR1A AGTR1 EDN1 ADRA2A HTR1A
SEMA3C SEMA3E FN1 EDN1 ERBB4 ALX1
FN1 GJB2 IL1RAPL2 WNT5A FER LRRTM1 LRRN3 ERBB4 ZNF703 SNCA F11R FAM107A PCDHB6
CALB1 NR2E1 SNCA LRRTM1 TSHZ3 FAM107A
SEMA3C FGF1 TACR3 SEMA3E EDN1 CEMIP SMOC2 FN1 PLA2G7 F2RL1 FER ZNF703 WNT5A ADRA2A
NR2E1 WNT5A ERBB4 ID2
FER SYT3 ADRA2A FCN3 SNCA F2RL1 WNT5A SGIP1 HTR1B CHRNB3 CHRNA6 LRRTM1 CPLX3 RIN3
FGF1 FSTL1 VWC2 CD44 SOX6 SMOC2 GDF6 ZNF703 EDN1 ANGPT2 WNT5A FER BMPER KCNC2
SEMA3C FGF1 TACR3 SEMA3E EDN1 ANGPT2 CEMIP SMOC2 FN1 PLA2G7 F2RL1 BMPER NR2E1
CALCR TRPC7 EDN1 CALB1 AGTR1 SLC39A12 F2RL1 GPR35 ATP4B CEMIP WNT5A SNCA XK HTR1B
EPHA8 SEMA3C NRK SEMA3E SYT3 XK FSTL4 EDN1 NR2E1 WNT5A FN1 DISC1 CCDC141 FOXG1 CD44 NGEF
SEMA3C SEMA3E TG FSTL4 WNT2 NR2E1 WNT5A FN1 DISC1 LRRTM1 LRRN3 FOXG1 ID2
EDN1 SEMA3C DSP WNT2 WNT5A ERBB4 NKX2-5 SOX6 ID2
SEMA3C FGF1 TACR3 SEMA3E EDN1 CEMIP SMOC2 FN1 PLA2G7 F2RL1 FER ZNF703 WNT5A ADRA2A FAM107A
EPHA8 SEMA3C NRK SEMA3E SYT3 XK FSTL4 EDN1 NR2E1 WNT5A FN1 DISC1 CCDC141 FOXG1 CD44
SYT3 HTR1B SNCA CHRNA6
SYT3 HTR1B SNCA CHRNA6
SEMA3C SEMA3E FGF1 SYT3 FSTL4 EDN1 WNT5A FN1 DISC1
CALB1 HTR1B SNCA CHRNB3 CHRNA6 PTCHD1 HTR1A SYN3 CPLX3 SHISA9 EDN1 NR2E1 ADRA2A
SEMA3C FGF1 TACR3 SEMA3E EDN1 CEMIP SMOC2 FN1 PLA2G7 F2RL1 FER ZNF703 WNT5A ADRA2A
VIP F2RL1 GPR35 AGTR1 ADRA2A EDN1
ZNF703 WNT5A TGFA SLC29A2 ERBB4 WNT2 ID2
CALCR EDN1 TPH2 GJB2 AVPR1A AQP4 FAM107A

KCNK6 KCNQ4 KCNK15 KCNK1 VIP KCNC2 ATP4B RGS7 ADRA2A
WNT2 SOX6 SEMA3E NR2E1 WNT5A DISC1 CCDC141 PTCHD1 KCNC2 AVPR1A FOXG1 ERBB4 RGS7
SEMA3C FGF1 TACR3 SEMA3E EDN1 ANGPT2 CEMIP SMOC2 FN1 PLA2G7 F2RL1 BMPER NR2E1 LAMA4 FER ERBB4 ZNF703 DSP RIN3 WNT5A ADRA2A FAM107A
TICAM1 CD44 CALB1 FGF1 BMP3 GDF6 TGFA F2RL1 GPR35 EDN1 SMOC2 WNT5A FN1 NTS ADRA2A DKK2 F11R BMPER SEMA3E EPHA8 SLC15A3 NR2E1 SNCA DISC1 LRRTM1 ERBB4 MAP3K5 CALCR
SEMA3C FGF1 TACR3 SEMA3E EDN1 ANGPT2 CEMIP SMOC2 FN1 PLA2G7 F2RL1 BMPER NR2E1
SEMA3C SEMA3E FN1 EDN1 WNT2 WNT5A ERBB4 ALX1 ZNF703
EDN1 AVPR1A AGTR1 F2RL1
SYN3 CPLX3 SNCA HTR1B CHRNB3 CHRNA6 ADRA2A
SYN3 CPLX3 SNCA HTR1B CHRNB3 CHRNA6 ADRA2A
WNT2 SOX6 WNT5A EDN1 NR2E1 FEV FOXG1 ERBB4 NKX2-5 ID2
F11R COL18A1 DSP FGF1 F2RL1 ZNF703 SEMA3C FOSL2 EDN1 CALB1 WNT2 WNT5A PAX8 ABCA12
IFT88 EPHA8 SEMA3C WNT2 WNT5A NRK ASTN2 DISC1 SEMA3E SYT3 XK FSTL4 EDN1 NR2E1 FN1 SATB2 PTPRB SLC39A12 GDF6 CCDC141 FEV FOXG1 ERBB4 RTN4RL1 VWC2 NKX2-5 NGEF SOX6 ID2
TICAM1 CD44 CALB1 FGF1 BMP3 GDF6 TGFA F2RL1 GPR35 EDN1 SMOC2 WNT5A FN1 NTS SNCA ADRA2A DKK2 F11R BMPER SEMA3E EPHA8 SLC15A3 NR2E1 DISC1 LRRTM1 ERBB4 MAP3K5 CALCR
SEMA3C SEMA3E ANGPT2 WNT5A RIN3
EPHA8 SEMA3C NRK SEMA3E SYT3 XK FSTL4 EDN1 NR2E1 WNT5A FN1 DISC1 CCDC141 FOXG1 CD44
TRPC7 KCNK6 KCNQ4 KCNK15 KCNK1 SLC39A12 KCNC2 ATP4B SHISA9 EDN1 CEMIP SNCA PIEZO1
SCIN SOX6 HAPLN1 WNT2 SEMA3E NR2E1 WNT5A PAX8 DISC1 CCDC141 PTCHD1 KCNC2 AVPR1A
FER SYT3 ADRA2A F2RL1 HTR1B CHRNB3 CHRNA6
ANGPT2 ISM1 PTPRB BMPER COL18A1 SMOC2 FGF1 EDN1 WNT2 NR2E1 WNT5A FN1 PAX8
CPLX3 SNCA HTR1B CHRNB3 CHRNA6 ADRA2A
SEMA3C FGF1 SEMA3E EDN1 ANGPT2 CEMIP SMOC2 FN1 PLA2G7 F2RL1 BMPER NR2E1 LAMA4 FER
ZNF703 ERBB4 ID2
KCNK6 KCNK15 KCNK1
GCH1 SNCA TACR3 HTR1A
EDN1 WNT2 FGF1 WNT5A
ZNF703 WNT5A ERBB4 WNT2 ID2
SEMA3C SEMA3E FSTL4 WNT5A FN1 DISC1
CALCR HTR1B VIP ADRA2A GPR26 MC4R HTR1A EDN1 NPY1R
ALX1 EDN1 WNT5A KCNQ4 SATB2 PAX8 FOXG1 NKX2-5 HMX2 ID2
EDN1 ABCA12 ADRA2A MC4R HTR1A CPLX3 PAX8 VIP MYRIP AGTR1
SEMA3C SEMA3E FN1 EDN1 WNT2 WNT5A ERBB4 ALX1 NKX2-5 ZNF703
IFT88 EPHA8 SEMA3C WNT2 WNT5A NRK SEMA3E SYT3 XK FSTL4 EDN1 NR2E1 FN1 SLC39A12 GDF6 DISC1 CCDC141 FEV FOXG1 ERBB4 RTN4RL1 VWC2 NKX2-5 NGEF ID2 SOX3
SCIN IFT88 EPHA8 SEMA3C NRK SPAG17 DISC1 SEMA3E FER SYT3 XK FSTL4 EDN1 NR2E1 WNT5A FN1 SLC39A12 TTC12 CCDC141 F2RL1 CFAP46 FOXG1 CFAP65 RTN4RL1 CD44 FSIP2 NGEF FGD5
TG EDN1 SULT1E1 PAX8 ABCA12 ADRA2A ALDH1A1 MC4R HTR1A CPLX3 VIP MYRIP AGTR1 PLA2G7
CPLX3 SNCA HTR1B CHRNB3 CHRNA6 ADRA2A
SEMA3C SEMA3E XK FSTL4 WNT5A FN1 DISC1
SEMA3C FGF1 SEMA3E EDN1 CEMIP SMOC2 FN1 PLA2G7 F2RL1 FER ZNF703 WNT5A ADRA2A
CALCR TRPC7 EDN1 CALB1 AGTR1 SLC39A12 F2RL1 GPR35 ATP4B CEMIP WNT5A SNCA XK HTR1B

EPHA8 SEMA3C SEMA3E XK FSTL4 EDN1 NR2E1 WNT5A FN1 DISC1 CCDC141 FOXG1 RTN4RL1
EDN1 AVPR1A AGTR1
WNT2 FGF1 WNT5A PAX8
SEMA3C EDN1 WNT5A NKX2-5 NPY1R
HTR1B MC4R TACR3 HTR1A SGIP1
AVPR1A AGTR1 EDN1 GCH1 ADRA2A TACR3 HTR1A NKX2-5 DSP
SOX6 EDN1 WNT5A SATB2 BMP3 CD44 GDF6 SCIN
EDN1 ABCA12 ADRA2A MC4R HTR1A CPLX3 PAX8 VIP MYRIP AGTR1
IFT88 FGF1 GDF6 ANGPT2 CALB1 WNT5A PAX8 BMPER ERBB4 AGTR1
NR2E1 DISC1 CCDC141 FOXG1
PAX8 EPHA8 EDN1
SNCA HTR1B HTR1A
SHISA9 EDN1 CEMIP SNCA KCNK6 KCNQ4 KCNK15 CACNA2D3 KCNC2 RGS7 CALCR GPR35 ADRA2A
SEMA3C SEMA3E FER XK FSTL4 NR2E1 WNT5A FN1 SLC39A12 DISC1 F2RL1 RTN4RL1 CD44 IFT88 NGEF
CALCR TRPC7 EDN1 CALB1 AGTR1 SLC39A12 F2RL1 GPR35 ATP4B CEMIP WNT5A SNCA XK HTR1B
VIP EDN1 WNT2 ERBB4 NKX2-5 MAP3K5 HTR1B ID2
VIP SYT3 SNCA HTR1B CHRNA6 HTR1A ABCC3 AGTR1 ADRA2A
EPHA8 SEMA3C NRK SEMA3E SYT3 XK FSTL4 EDN1 NR2E1 WNT5A FN1 DISC1 CCDC141 FOXG1 NGEF
CALCR TRPC7 EDN1 CALB1 AGTR1 SLC39A12 F2RL1 GPR35 ATP4B CEMIP WNT5A SNCA XK HTR1B
IFT88 EPHA8 SEMA3C NRK SPAG17 DISC1 SEMA3E FER SYT3 XK FSTL4 EDN1 NR2E1 WNT5A FN1 SLC39A12 CCDC141 F2RL1 CFAP46 FOXG1 CFAP65 RTN4RL1 CD44 TTC12 FSIP2 NGEF FGD5
EPHA8 SEMA3C NRK SEMA3E SYT3 XK FSTL4 EDN1 NR2E1 WNT5A FN1 DISC1 CCDC141 FOXG1 NKX2-
SEMA3E WNT5A PTCHD1 KCNC2 SOX3
CALB1 PAX8
CALB1 PAX8
EDN1 GJB2
WNT2 SOX6 SEMA3E NR2E1 WNT5A DISC1 CCDC141 PTCHD1 KCNC2 AVPR1A FOXG1 ERBB4 RGS7
EPHA8 SEMA3C SEMA3E XK FSTL4 EDN1 NR2E1 WNT5A FN1 DISC1 CCDC141 FOXG1
VIP AVPR1A AGTR1 EDN1 GCH1 ADRA2A F2RL1 TACR3 HTR1A GPR35 NKX2-5 SHISA9 DSP TSHZ3
TGFA ERBB4 ID2
TGFA ERBB4 ID2
EDN1 AVPR1A AGTR1
IFT88 FGF1 GDF6 ANGPT2 CALB1 WNT5A PAX8 BMPER ERBB4 AGTR1
SMOC2 FGF1 ANGPT2 NR2E1 SLC39A12 BMPER WNT5A
SMOC2 FGF1 ANGPT2 NR2E1 SLC39A12 BMPER WNT5A
SYN3 CPLX3 SNCA SLC29A2 HTR1B CHRNB3 CHRNA6 ADRA2A
FGF1 EDN1 WNT5A ADRA2A TGFA MAP3K5
NR2E1 WNT5A DISC1 CCDC141 AVPR1A FOXG1 ERBB4 RTN4RL1 ID2
TG GCH1 SULT1E1 FGF1 SNCA TYRP1 WNT5A PAX8 TPH2 ALDH1A1 TACR3 HTR1A MALRD1 ACSS2
VIP CALB1 NR2E1 HTR1B SNCA AFF2 LRRTM1 NPY1R PTCHD1 AVPR1A MC4R TACR3 HTR1A ID2
TRPC7 KCNK6 KCNQ4 KCNK15 KCNK1 VIP SLC39A12 KCNC2 ATP4B CEMIP SNCA EDN1 CACNA2D3

SEMA3C SEMA3E FER XK FSTL4 NR2E1 WNT5A FN1 SLC39A12 DISC1 F2RL1 RTN4RL1 CD44 IFT88
FGF1 OSMR VIP TGFA ERBB4 EDN1 WNT2 WNT5A FN1 NELL1 AVPR1A ZNF703 DLEC1 FOSL2 NR2E1 TICAM1 DISC1 LMO1 FOXG1 NKX2-5 HMX2 MAP3K5 AGTR1 SCIN HTR1B FER ID2 ADRA2A HTR1A
FGF1 ALX1 SEMA3C EDN1 DSP CALB1 WNT2 WNT5A KCNQ4 SATB2 PAX8 TGFA FOXG1 ERBB4 NKX2-
EDN1 AVPR1A AGTR1
EDN1 DSP WNT2 WNT5A ERBB4 NKX2-5 SOX6 ID2
CD44 FGF1 EDN1 WNT5A ADRA2A TGFA F2RL1 BMPER EPHA8 GDF6 MAP3K5 CALCR ERBB4
HTR1B HTR1A ADRA2A EDN1 NPY1R
SEMA3C SEMA3E EDN1 WNT5A FN1 DISC1
EPHA8 SEMA3C FER SEMA3E ANGPT2 FGF1 AGTR1 PLA2G7 F2RL1 EDN1 WNT5A CCDC141 FOXG1
SEMA3C SEMA3E EDN1 SYT3 FSTL4 WNT5A FN1 DISC1
SLC39A12 BMPER FGF1
CALCR TRPC7 EDN1 CALB1 AGTR1 SLC39A12 F2RL1 GPR35 ATP4B CEMIP WNT5A SNCA XK HTR1B
EDN1 AVPR1A AGTR1 F2RL1
EPHA8 SEMA3C FER SEMA3E ANGPT2 FGF1 AGTR1 PLA2G7 F2RL1 EDN1 WNT5A CCDC141 FOXG1
NKX2-5 ID2
AVPR1A GPR35
DISC1 FOXG1
BMPER NR2E1 SLC39A12 SEMA3E AGTR1 FGF1
SEMA3C SEMA3E SYT3 EDN1 WNT5A FN1 DISC1
DSP FGF1 FN1 SMOC2 WNT5A F11R F2RL1 ADRA2A TGFA MAP3K5 CD44 EDN1
SEMA3C SOX6 SEMA3E EDN1 NELL1 FSTL4 MBNL3 WNT2 NR2E1 WNT5A FN1 PAX8 ABCA12 GDF6 DISC1 FOXG1 NKX2-5 ZNF703 VWC2 MAP3K5 SCIN PIEZO1 SULT1E1 AGTR1 F11R ALX1 ID2 SOX3
FGF1 ANGPT2 SMOC2 BMPER NR2E1 WNT5A EDN1
EDN1 CALB1 WNT5A KCNQ4 PAX8 FOXG1 HMX2 LCTL WNT2
FGF1 ANGPT2 SMOC2 BMPER NR2E1 FSTL1 WNT5A EDN1
SYN3 CPLX3 SNCA HTR1B CHRNB3 CHRNA6 ADRA2A SLC29A2
TICAM1 CALCR GCH1 SNCA ZNF703 IFT88 EDN1 NR2E1 HTR1B TPH2 FER GJB2 AVPR1A TACR3 AQP4
FGF1 EDN1 CEMIP WNT5A SNCA ADRA2A TGFA MAP3K5
TRPC7 KCNK6 KCNQ4 KCNK15 KCNK1 CHRNB3 CHRNA6 SLC39A12 KCNC2 ATP4B SHISA9 EDN1 CEMIP SNCA PIEZO1 SLC15A3 CACNA2D3 ANO10 RGS7 CALCR GPR35 ADRA2A
EDN1 WNT5A KCNQ4 PAX8 FOXG1 HMX2
EDN1 SLC15A3 ABCA12 ADRA2A MC4R CPLX3 PAX8 VIP MYRIP
BMP3 GDF6 FSTL1 VWC2 IGSF1 ZNF703 WNT5A BMPER ADAMTSL2
SEMA3E DISC1 FOXG1
SYT3 SNCA HTR1B CHRNA6
SEMA3E NR2E1 DISC1 FOXG1
WNT5A FN1 FOSL2 WNT2
EDN1 WNT2 ERBB4 NKX2-5

TRPC7 KCNK6 KCNQ4 KCNK15 KCNK1 SLC39A12 KCNC2 ATP4B CEMIP SNCA EDN1 SLC15A3 CACNA2D3 RGS7 CALCR GPR35 ADRA2A
EPHA8 SEMA3C NRK SEMA3E COL18A1 FN1 F11R SYT3 XK FSTL4 EDN1 NR2E1 WNT5A FER DISC1
EDN1 CEMIP SNCA KCNC2 AVPR1A RGS7 NKX2-5 CALCR ADRA2A
FGF1 FSTL1 VWC2 CD44 SOX6 SMOC2 GDF6 ZNF703 EDN1 ANGPT2 WNT5A BMPER ERBB4 ADAMTSL2 WNT2
HTR1B HTR1A
VIP ADRA2A
NKX2-5 ID2
CALB1 PAX8
CEMIP WNT5A
VIP ADRA2A
CEMIP WNT5A
ATP4B EDN1 AVPR1A AGTR1
SPAG17 CFAP46 CFAP65 TTC12 FSIP2
SEMA3C SEMA3E FSTL4 WNT5A RTN4RL1 NGEF
EDN1 ABCA12 ADRA2A MC4R CPLX3 PAX8 VIP MYRIP
TICAM1 CD44 FGF1 F2RL1 GPR35 EDN1 WNT5A FN1 NTS ADRA2A F11R TGFA BMPER SEMA3E EPHA8 SLC15A3 GDF6 ERBB4 MAP3K5 CALCR
SEMA3C SEMA3E EDN1 FAM107A SYT3 FSTL4 WNT2 WNT5A FN1 DISC1 NPY1R AVPR1A ERBB4 NKX2-
NR2E1 DISC1 CCDC141 FOXG1
MC4R TACR3 SGIP1
GCH1 SNCA TACR3 HTR1A
GCH1 SNCA TACR3 HTR1A
NR2E1 WNT5A DISC1 FOXG1 ID2
IL1RAPL2 WNT5A LRRTM1 LRRN3 ERBB4 SNCA PCDHB6
EDN1 WNT5A PAX8 NKX2-5 TG SOX3
EDN1 ABCA12 ADRA2A MC4R CPLX3 PAX8 VIP MYRIP
SEMA3C SEMA3E WNT5A FN1 DISC1
ISM1 BMPER SMOC2 FGF1 ANGPT2 NR2E1 SLC39A12 SEMA3E WNT5A
DSP WNT2 WNT5A NKX2-5
ZNF703 WNT5A ID2
SEMA3C SEMA3E WNT5A
EDN1 WNT2 ERBB4
NR2E1 DISC1 FOXG1
CFAP65 TTC12 FSIP2
IFT88 FGF1 GDF6 ANGPT2 CALB1 WNT5A PAX8 BMPER ERBB4 AGTR1
TRPC7 KCNK6 KCNQ4 KCNK15 KCNK1 SLC39A12 KCNC2 ATP4B CEMIP SNCA EDN1 SLC15A3
EDN1 AVPR1A AGTR1 KCNK6 F2RL1

FGF1 SEMA3C EDN1 WNT2 WNT5A PAX8 SEMA3E
ISM1 BMPER SMOC2 FGF1 ANGPT2 NR2E1 SLC39A12 SEMA3E WNT5A
CD44 FGF1 NRK EDN1 WNT5A FN1 ADRA2A TGFA F2RL1 BMPER EPHA8 GDF6 MAP3K5 CALCR ERBB4
NR2E1 AVPR1A
WNT2 WNT5A
HTR1B HTR1A
WNT5A ADRA2A FGF1
SNCA HTR1B ADRA2A
EDN1 AVPR1A AGTR1
SNCA CPLX3 HTR1B CHRNB3 CHRNA6 ADRA2A SYN3
KCNK6 PIEZO1 KCNK15 KCNK1 CHRNB3 CHRNA6 EDN1 SNCA KCNC2 GPR35 DSP
CALCR TRPC7 EDN1 CALB1 AGTR1 SLC39A12 F2RL1 GPR35 ATP4B CEMIP WNT5A SNCA XK HTR1B
NR2E1 GDF6 FOXG1 VWC2 NKX2-5 ID2 SOX3
SEMA3C SEMA3E SOX6 FN1 EDN1 ERBB4 ALX1 NKX2-5
SEMA3C SEMA3E XK FSTL4 NR2E1 WNT5A FN1 SLC39A12 DISC1 RTN4RL1 NGEF
SPAG17 CFAP65 TTC12 FSIP2
EDN1 WNT2 ERBB4 NKX2-5
HTR1B CHRNB3 CHRNA6 ADRA2A
WNT2 WNT5A NKX2-5
SPAG17 TACR3 CFAP46 CFAP65 TTC12 FSIP2 AQP4
EPHA8 SEMA3C SEMA3E XK FSTL4 EDN1 NR2E1 WNT5A FN1 DISC1 CCDC141 FOXG1 NGEF
EDN1 SNCA HTR1B RGS7 FRMPD1 CALCR
CALCR TRPC7 EDN1 CALB1 AGTR1 SLC39A12 F2RL1 GPR35 ATP4B CEMIP WNT5A SNCA XK HTR1B
SEMA3C SEMA3E FSTL4 WNT2 NR2E1 WNT5A FN1 DISC1 FOXG1 ID2
SEMA3C SEMA3E EDN1 FAM107A SYT3 FSTL4 WNT5A FN1 DISC1 AVPR1A AGTR1
WNT2 FGF1 WNT5A
SNCA SYNPO WNT5A IL1RAPL2 DISC1 LRRTM1 SEMA3E LRRN3 ERBB4 NGEF PCDHB6
FGF1 VIP TGFA BMPER ZNF703 WNT2 WNT5A NKX2-5 HMX2 AGTR1 ID2
CD44 CEMIP
EDN1 GCH1 SNCA AGTR1
EDN1 GCH1 AVPR1A TACR3 DSP
SHISA9 EDN1 CEMIP SNCA KCNK6 KCNQ4 KCNK15 CACNA2D3 KCNC2 RGS7 CALCR GPR35 ADRA2A
SEMA3C ISM1 SEMA3E ANGPT2 FN1 F2RL1 CALCR FSTL4 NR2E1 LAMA4 WNT5A PAX8 ADRA2A AVPR1A AQP4 GPR35 SOX6 ID2 ALDH1A1 EDN1
SOX6 WNT5A GDF6
EDN1 WNT2 ERBB4
SEMA3C SEMA3E FSTL4 WNT5A
HTR1B NPY1R MC4R TACR3 SGIP1
IL1RAPL2 WNT5A LRRTM1 LRRN3 SNCA
FN1 EDN1 SEMA3C DSP WNT2 WNT5A PAX8 ERBB4 NKX2-5 RBM20 SOX6 NPY1R ID2
CALCR TRPC7 EDN1 CALB1 AGTR1 SLC39A12 F2RL1 GPR35 ATP4B CEMIP WNT5A SNCA XK HTR1B
VIP EDN1 CEMIP FCN3 ABCA12 SNCA F2RL1 WNT5A SGIP1 KCNC2 AVPR1A RGS7 NKX2-5 CALCR
WNT2 NR2E1 WNT5A DISC1 FOXG1 ID2
EPHA8 FGF1 TGFA ERBB4 CD44 EDN1 CEMIP WNT5A SNCA ADRA2A TNFSF15 MAP3K5

CALCR PAX8 AGTR1 IFT88 EPHA8 EDN1 NR2E1 HTR1B TPH2 FER GJB2 AVPR1A MC4R AQP4 SLC29A2
FGF1 SEMA3C EDN1 WNT2 WNT5A PAX8 SEMA3E
CD44 FGF1 NRK EDN1 WNT5A FN1 ADRA2A TGFA F2RL1 BMPER MAP3K5 EPHA8 GDF6 PPM1L
CALB1 SHISA9 NR2E1 SNCA LRRTM1 TSHZ3 FAM107A
EDN1 CEMIP SNCA KCNC2 RGS7 CALCR
EPHA8 SEMA3C SEMA3E COL18A1 FN1 XK FSTL4 EDN1 NR2E1 WNT5A FER DISC1 CCDC141 FOXG1 NGEF
EDN1 SLC15A3 ABCA12 ADRA2A MC4R CPLX3 PAX8 VIP MYRIP
SEMA3C EDN1
VIP ADRA2A
WNT5A CALB1
KCNK6 KCNK1
SNCA BHMT
ADRA2A TACR3
HTR1B HTR1A
F2RL1 GPR35 F11R
SEMA3C SEMA3E WNT5A
BMP3 GDF6 FSTL1 VWC2 IGSF1 BMPER ZNF703 WNT5A PPM1L ADAMTSL2
CALCR TRPC7 EDN1 CALB1 AGTR1 SLC39A12 F2RL1 GPR35 ATP4B CEMIP WNT5A SNCA XK LAMA4 ARMCX1 HTR1B ABCA12 ADRA2A DISC1 FSTL1 AVPR1A LMO1 MC4R AQP4 MALRD1 ID2 SGIP1
NR2E1 PTPRB DISC1 FOXG1
DSP WNT2 WNT5A NKX2-5
WNT5A KCNQ4 PAX8 FOXG1 HMX2
SNCA CPLX3 HTR1B CHRNB3 CHRNA6 ADRA2A SYN3
TACR3 HTR1A BHMT
TICAM1 HTR1B HTR1A EDN1 GCH1 AGTR1 SNCA ZNF703 MAP3K5 IFT88 ANGPT2 ABCA12 ADRA2A FER GJB2 KCNC2 AVPR1A MC4R TACR3 AQP4 SLC29A2 RGS7 ATP4B WNT5A CALCR ALDH1A1 WNT2
IL1RAPL2 WNT5A LRRTM1 LRRN3 SNCA F11R FAM107A
FGF1 EDN1 WNT2 WNT5A PAX8 SEMA3E
IFT88 EPHA8 SEMA3C NRK SEMA3E SYT3 XK FSTL4 EDN1 NR2E1 WNT5A FN1 SLC39A12 DISC1
DSP FGF1 FN1 SMOC2 WNT5A F11R F2RL1 ADRA2A TGFA RTN4RL1 MAP3K5 CD44 EDN1
NR2E1 DISC1 FOXG1
AVPR1A TACR3 ID2
EDN1 MAP3K5 HTR1B
WNT2 FGF1 WNT5A
CFAP65 TTC12 FSIP2

NR2E1 DISC1 FOXG1
EDN1 WNT2 ERBB4 NKX2-5
GCH1 SNCA TACR3 HTR1A BHMT
KCNK6 KCNQ4 KCNK15 KCNK1 KCNC2 ATP4B RGS7
SOX6 EDN1 WNT5A SATB2 BMP3 CD44 GDF6 SCIN
ALX1 EDN1 WNT2 WNT5A KCNQ4 SATB2 PAX8 FOXG1 NKX2-5 HMX2 ID2
EPHA8 FGF1 TGFA ERBB4 CD44 EDN1 CEMIP WNT5A SNCA ADRA2A TNFSF15 MAP3K5 AGTR1
EDN1 SYT3 WNT2 FN1 DISC1 ERBB4
CALB1 PAX8
WNT5A ERBB4
VIP AVPR1A
TPH2 HTR1A
ADRA2A TACR3
WNT2 WNT5A PAX8 FGF1
NR2E1 DISC1 CCDC141 FOXG1
WNT5A IL1RAPL2 DISC1 LRRTM1 LRRN3 SNCA NGEF
EDN1 AVPR1A AGTR1
EDN1 HTR1B AVPR1A
F11R COL18A1 F2RL1 FOSL2 WNT5A ABCA12 FER
EDN1 XK SEMA3C DSP WNT2 WNT5A ERBB4 NKX2-5 SOX6 ID2
SEMA3C DSP WNT2 WNT5A PAX8 NKX2-5
WNT2 FGF1 WNT5A PAX8
EDN1 PAX8 NKX2-5 MAP3K5
SEMA3C SEMA3E WNT5A
WNT2 WNT5A NKX2-5
SEMA3C SEMA3E WNT5A
SEMA3C EDN1
EPHA8 EDN1
CALB1 PAX8
GCH1 SNCA
EDN1 AVPR1A
DISC1 FOXG1
PTCHD1 CALB1
SNCA F2RL1
NR2E1 DISC1 CCDC141 FOXG1
SOX6 WNT5A GDF6 SCIN
EDN1 HTR1B SNCA AVPR1A
GCH1 SNCA TACR3 HTR1A BHMT

CALB1 NR2E1 SNCA LRRTM1 TSHZ3 FAM107A
WNT5A IL1RAPL2 DISC1 LRRTM1 LRRN3 SNCA NGEF
WNT2 NR2E1 FN1 DISC1 LRRTM1 LRRN3 FOXG1 ID2
FN1 F11R F2RL1 EDN1 ABCA12 ADRA2A SLC29A2 ERBB4 AQP4 VIP
TICAM1 HTR1B HTR1A EDN1 AGTR1 ZNF703 MAP3K5 IFT88 ABCA12 ADRA2A FER GJB2 KCNC2 AVPR1A AQP4 SLC29A2 WNT5A GCH1 ALDH1A1 WNT2 SNCA
DSP WNT2 WNT5A NKX2-5
WNT2 ERBB4 NKX2-5
EDN1 CEMIP SNCA KCNC2 RGS7 CALCR

schizophrenia vs. coTwin - KEGG

Pathway	Enrichment FDR	nGenes	Pathway Genes	Fold Enrichment	Genes
Neuroactive ligand-receptor interaction	2.87E-05	16	362	4.957426064	CHRNB3 ADRA2A AGTR1 EDN1 F2RL1 GPR35 HTR1A HTR1B MC4R NPY1R NTS AVPR1A TACR3 VIP CALCR CHRNA6

Transcript,	chr	start	end	strand	Length	Copies	Annotatioı	Control1_:	Control1_2
NM_1738(	chr16	15395754	15409686	+	2234	2	MPV17L|N	0	0
NM_0144:	chr5	1.33E+08	1.33E+08	-	9557	1	AFF4|AF5(	17.663	26.385
NM_0011(	chr19	10304803	10309557	-	1974	1	ZGLP1|GA	3.873	4.08
NR_13462	chr22	36703876	36767089	+	552	1	LOC10537.	0.234	0.298
NR_02449	chr15	50354174	50358306	+	4132	1	GABPB1-A	7.074	6.941
NM_0183!	chr3	53816297	53846393	-	3695	1	CHDH|-|-|	0.255	0.196
NM_0010:	chr12	92702843	92708549	+	1853	1	C12orf74|	0	0
NR_10685	chr19	52222020	52222098	+	78	1	MIR6801|l	52.566	58.125
NM_1334!	chr16	68539213	68567136	+	4634	1	ZFP90|FIK	19.346	16.484
NM_0010{	chr17	7839917	7854800	+	6680	1	KDM6B|JN	34.853	30.053
NM_0012:	chr10	1.14E+08	1.14E+08	-	4255	1	DCLRE1A|I	3.594	3.731
NR_02728	chr17	68198660	68200302	+	1642	1	LOC44046	0.047	0.142
NR_03332	chr7	72948293	72954763	-	1756	1	NSUN5P2|	3.275	2.699
NM_0011!	chr17	39941474	39944734	+	875	1	LRRC3C|-|	0.029	0
NM_0148:	chr3	1.51E+08	1.51E+08	-	2483	1	P2RY14|BI	0.016	0.006
NM_0069:	chr11	1.21E+08	1.21E+08	+	6939	1	SC5D|ERG	8.475	6.567
NM_0011‹	chr20	36751791	36773827	-	2188	1	DSN1|C20	3.524	2.83
NR_10994	chr20	47983226	47984313	+	626	1	LINC01523	0	0
NR_13566	chr15	39782906	39785616	+	2710	1	LOC10537(	0.466	0.349
NM_0011:	chr7	1.29E+08	1.29E+08	-	4695	1	KCP|CRIM	0.049	0.053
NM_1706:	chr5	1.34E+08	1.34E+08	-	2006	1	SKP1|EMC	140.986	145.799
NR_02700	chr8	7268241	7271114	-	2873	3	LINC00965	0	0
NM_0013(	chr19	4343527	4360086	+	1642	1	MPND|-|-	20.392	18.802
NM_0013:	chr3	64515655	64687689	-	7187	1	ADAMTS9	2.384	4.561
NM_0046!	chr1	1.5E+08	1.5E+08	+	2445	1	PRPF3|HPI	16.738	16.296
NM_0013‹	chr4	54229089	54281978	+	3041	1	PDGFRA|C	1.899	2.743
NM_0175!	chr3	1.23E+08	1.23E+08	+	7689	1	PARP14|A	0.086	0.718
NM_0156(	chr19	47256474	47271953	+	2064	1	CCDC9|-|-	7.203	8.74
NM_0021‹	chr17	48529445	48530910	-	1010	1	HOXB1|HC	0	0
NR_10236	chr11	65111854	65116235	+	1537	1	TM7SF2|A	68.242	47.356
NM_0012{	chr3	1.34E+08	1.34E+08	+	2876	1	CDV3|H41	29.342	35.224
NM_0056(	chr4	1982714	2009235	-	2455	1	NELFA|NE	32.72	30.524
NM_1532:	chr1	16197854	16212671	-	3323	1	ARHGEF19	1.424	1.377
NM_0025!	chr11	57338367	57370076	+	1443	1	P2RX3|P2)	33.453	33.089
NR_03819	chr9	13927971	13945607	+	381	1	LINC00583	0	0
NM_0159(	chr8	1.02E+08	1.02E+08	-	10822	1	UBR5|DD5	11.578	17.357
NM_0010:	chr3	1.84E+08	1.84E+08	+	1921	1	AP2M1|AF	309.739	290.126
NM_0010‹	chr1	2.43E+08	2.43E+08	-	6791	2	CEP170|F/	0	0
NM_0010(	chr7	1E+08	1E+08	+	3172	1	SPDYE3|-|	1.203	1.144
NM_0011‹	chr1	1426128	1427787	+	489	1	TMEM88B	0.29	0.392
NM_0028{	chr3	1.53E+08	1.53E+08	+	8410	1	RAP2B|-|-	3.658	5.733
NM_0314:	chr8	1.3E+08	1.3E+08	-	2698	1	GSDMC|M	0	0.025
NM_0181‹	chr3	1.39E+08	1.39E+08	+	917	1	FAIM|FAIN	0.028	0.134
NM_0010:	chr13	23888889	23892103	+	798	1	C1QTNF9B	0.226	0.343
NM_0165(	chr10	1.26E+08	1.26E+08	+	1434	1	BCCIP|TOI	21.669	21.252
NM_0011!	chr1	1.57E+08	1.57E+08	-	1036	1	CRABP2|C	93.142	66.872
NM_0013:	chr20	34088298	34112356	-	2660	1	EIF2S2|EIF	15.041	13.141
NR_10804	chr1	1.1E+08	1.1E+08	-	2349	1	CYMP-AS1	0	0
NM_0013:	chr2	1.08E+08	1.08E+08	+	1210	1	SULT1C3|S	0	0

NM_0328(	chr6	1.44E+08	1.44E+08	+	1905	1	LTV1|C6or	6.558	8.463
NM_0013:	chr12	8602166	8612867	-	2778	1	AICDA|AIC	0	0
NM_0013(	chr1	66533569	66751139	+	10133	1	SGIP1|-|-|	0.811	0.812
NM_1471!	chr9	1.1E+08	1.1E+08	+	7467	1	PALM2-AK	3.994	2.062
NM_0011:	chr12	51238724	51243933	+	2083	1	DAZAP2|P	84.844	87.988
NM_0038:	chr1	2556365	2565622	+	3491	2	TNFRSF14	0	0
NM_0012!	chr20	47209214	47356889	-	5010	1	ZMYND8|I	26.634	26.336
NM_0012!	chr8	1.45E+08	1.45E+08	+	2119	1	ZNF7|HF.1	11.543	13.51
NM_0030(	chr5	1.32E+08	1.32E+08	+	3268	1	SLC22A5|(	0.896	1.806
NM_0012!	chr17	76000906	76005999	+	1560	1	CDK3|-|-|	5.232	5.891
NR_13424	chr5	1.34E+08	1.34E+08	+	575	1	LOC10537!	0.224	0.071
NR_00294	chr19	50882096	50896398	-	1120	1	KLKP1|KAI	0.069	0.024
NM_0027:	chr9	69012533	69014123	-	1588	1	PRKACG|B	0.162	0.147
NM_0247:	chr11	63938970	63957319	+	3671	1	NAA40|NA	31.48	33.629
NM_0035!	chr17	38765691	38799905	-	5720	2	PIP4K2B|P	0	0
NR_11064	chr9	68953088	68975841	-	2219	1	LOC10192	0	0
NM_0003!	chr17	30248195	30292166	-	2523	1	BLMH|BH	25.828	25.945
NR_11040	chrX	1.26E+08	1.26E+08	+	636	1	LOC10192	0	0
NM_0307!	chr14	63684216	63728095	-	3361	1	SGPP1|SPI	1.577	1.789
NR_12585	chr6	75383186	75458899	+	837	1	LOC10192	0	0
NR_02962	chr1	2.2E+08	2.2E+08	-	109	1	MIR215|M	0	0
NM_0307(	chr19	10512742	10517450	-	2337	1	S1PR5|ED(	0.199	0.182
NM_1827(	chr19	39515113	39520686	+	1696	1	SELENOV|	1.437	0.8
NM_0011(	chr1	1.79E+08	1.79E+08	-	11554	1	ABL2|ABLI	3.964	5.152
NM_0012!	chr17	36103827	36105621	+	647	6	CCL4L2|AT	0	0
NM_0011(	chr1	2.05E+08	2.05E+08	+	9914	1	NFASC|NF	63.185	51.902
NR_03038	chr8	1.44E+08	1.44E+08	-	88	1	MIR661|M	0	0
NM_0013:	chr1	53196429	53214197	+	3018	1	CPT2|CPT:	10.424	9.935
NM_0332!	chr2	18562871	18589580	-	2679	1	NT5C1B|A	0.019	0.015
NM_0174!	chr11	5507300	5509985	-	2403	1	UBQLN3|T	0	0.006
NM_0013:	chr19	48740888	48745932	-	1172	1	IZUMO1|I:	0.253	0.21
NM_0212:	chr19	56595296	56624176	+	3122	1	ZNF71|EZI	23.111	20.532
NR_03069	chr11	70085410	70189546	+	3570	1	ANO1|DO(	0.488	0.038
NM_0039(	chr21	46235125	46285394	-	6164	1	MCM3AP|	22.913	22.581
NM_0010:	chr1	1.5E+08	1.5E+08	-	492	1	HIST2H2BI	0.393	0.724
NM_0013:	chr21	28928145	28992959	-	7685	1	LTN1|C21(	1.926	3.372
NM_0013:	chr5	1.16E+08	1.16E+08	+	1395	1	AP3S1|CL/	19.53	16.741
NM_0013:	chr2	37196461	37204743	+	739	1	CEBPZOS|(	9.474	6.728
NR_03336	chr20	63038011	63053863	+	1228	1	LINC01056	0	0.022
NR_12538	chr11	1.31E+08	1.31E+08	-	1447	1	LOC10050	0.018	0
NR_10500	chr8	48620403	48623896	+	350	1	LOC10192!	0	0
NM_0221(	chrX	1.2E+08	1.2E+08	-	2317	1	CXorf56|-|	9.599	10.919
NR_13093	chr2	66383306	66392446	+	1672	1	LINC01873	0	0
NR_13428	chr5	17807274	17811855	+	819	1	LOC64624	2.33	0.803
NR_10385	chr17	47898386	47941404	-	2179	1	SP2-AS1|-	0.379	1.263
NM_0012:	chr1	2.44E+08	2.44E+08	+	3742	1	ZBTB18|C:	20.901	74.649
NR_03171	chr19	15449548	15449608	+	60	1	MIR1470|	0.43	0
NM_0190!	chr10	96043402	96060868	+	4097	1	CCNJ|bA6!	5.432	4.864
NM_0010!	chr16	30778455	30787219	-	6093	1	ZNF629|ZI	31.924	28.902
NR_02723	chr2	91636683	91659949	-	601	1	LOC65434:	1.116	0.593

NR_04555	chr8	1.2E+08	1.2E+08	-	2013	1	ENPP2|AT	10.857	7.75
NR_02710	chr3	13650721	13746635	+	988	1	LINC0062C	0	0.166
NR_03751	chr8	1.23E+08	1.23E+08	+	96	1	MIR548AA	0	0
NM_0010:	chr12	1.13E+08	1.13E+08	+	1714	1	OAS1|E18,	0.015	0.184
NM_0050:	chr12	11351823	11355590	-	1168.7	3	PRB1|PM|	0	0
NR_11937	chr1	83801516	83860546	-	1988	1	LOC10192	0.091	0.014
NM_0008	chr4	47935977	48012944	-	2770	1	CNGA1|CN	0.009	0.015
NM_1523	chr1	1.16E+08	1.16E+08	+	3220	1	MAB21L3|	0.02	0
NM_0184	chr1	1.16E+08	1.16E+08	+	4706	1	SLC22A15|	1.97	1.385
NM_0013	chr7	99493231	99500308	-	2068	1	ZNF394|ZI	6.266	5.067
NM_0011:	chr9	1.22E+08	1.22E+08	-	3242	1	TTLL11|C9	4.228	3.333
NM_0142	chr9	1.22E+08	1.22E+08	-	837	1	NDUFA8|C	80.596	83.066
NM_0227	chr2	2.32E+08	2.32E+08	+	2566	1	COPS7B|C	30.395	33.154
NM_0012	chr6	46220730	46491323	-	3289	1	RCAN2|CS	28.927	19.273
NM_0060	chrX	1.54E+08	1.54E+08	-	944	1	LAGE3|CV	16.813	14.437
NR_11007	chr14	70608798	70641298	-	2938	1	LOC10192	0.075	0.159
NM_0011:	chr22	31281593	31292534	-	2387	1	PIK3IP1|H	4.192	7.207
NM_0011	chr19	33386949	33521893	-	1803	1	PEPD|PRO	37.079	36.024
NM_0054	chr16	11267857	11269303	-	593	1	TNP2|TP2	0	0
NM_0309	chr6	88047789	88066832	+	1490	1	SPACA1|S/	0	0
NM_0012	chr20	35278906	35284717	-	1046	1	EIF6|CAB|	102.063	86.346
NM_0065	chr1	28668728	28715603	+	2682	1	GMEB1|PS	8.581	9.821
NM_0011	chr4	1.21E+08	1.21E+08	-	2394	1	TNIP3|ABI	0.151	0.149
NR_11083	chr7	40964678	40979939	-	3249	1	LINC0145C	0	0
NM_2076	chr21	42216077	42297244	+	2906	1	ABCG1|AB	10.955	9.53
NR_13655	chr5	56910062	56917338	+	1198	1	SETD9|C5c	1.593	0.812
NR_12637	chr4	1.46E+08	1.46E+08	-	592	1	SMAD1-AS	7.34	11.406
NM_1524	chr15	57591904	57685364	+	2300	1	MYZAP|GC	0.297	0.06
NM_0012	chr7	1.11E+08	1.12E+08	-	1200	1	IMMP2L|II	4.921	5.056
NM_0025	chr3	1.97E+08	1.97E+08	+	6122	1	PAK2|PAK	22.68	18.874
NR_02679	chr6_GL0C	2541918	2548174	+	1717.8	6	HCG27|bC	0.083	0.056
NM_0028	chr7	1.5E+08	1.5E+08	-	733	1	RARRES2|I	4.943	2.56
NR_02697	chr6	29726601	29749049	-	1899	6	HLA-F-AS1	0	0
NM_0013	chr6	32756098	32763553	-	1225	8	HLA-DQB2	0	0
NM_0529	chr16	24846231	24911628	+	2337	1	SLC5A11|k	0.061	0.129
NM_0013	chr17	782329	792509	+	1503	1	MRM3|RN	7.969	9.56
NM_0011	chr2	42169338	42332548	+	5363	1	EML4|C2o	7.455	9.314
NM_0011	chr1	31906421	31919658	-	3429	1	PTP4A2|H	44.952	47.906
NR_00327	chr17	19061912	19062128	+	216	2	SNORD3B-	0	0
NR_00277	chr14	1.02E+08	1.02E+08	-	3453	1	DIO3OS|C	0.153	0.111
NR_10375	chr6	1.68E+08	1.68E+08	-	1401	1	KIF25-AS1	0.028	0.049
NM_0208	chr14	23273635	23286100	-	3737	1	HOMEZ|KI	5.438	4.373
NR_03169	chr20	2652777	2652842	+	65	1	MIR1292|	0	0
NR_10503	chr8	80034870	80171200	-	4023	1	TPD52|D5	5.067	3.071
NM_0010	chr8	13566843	13568288	+	1443	1	C8orf48|-|	0.813	0.712
NR_10352	chr7	65714611	65750924	-	1504	1	LOC44124	0.351	0.446
NM_0013	chr7	1.51E+08	1.51E+08	-	2584	1	IQCA1L|IQ	0	0
NM_0010	chr18	68715254	69055189	+	2805	1	CCDC102B	0.565	0.566
NM_0207	chr1	19074506	19210252	-	15774	1	UBR4|RBA	23.86	32.957
NM_0212	chr19_GL9	288603	294640	-	1354.8	5	LILRA5|CD	0	0

NM_0010:	chr15	72159806	72197795	-	3278	1	GRAMD2|-	0.118	0.038
NM_0069	chr13	95552693	95579756	+	2483	1	CLDN10|C	0.452	0.359
NM_0011	chrX	1.53E+08	1.53E+08	+	3220	1	ZNF185|S	2.258	2.412
NM_0061	chr16	30350766	30355361	-	3412	1	CD2BP2|F	48.728	55.479
NM_0027	chr21	18269116	18403653	-	3917	1	TMPRSS15	6.699	0.189
NR_02415	chr22	19017834	19020248	+	2414	1	DGCR9|D(	2.222	2.559
NM_0011	chr11	10507887	10509176	-	1287	1	MTRNR2L8	0.09	0.074
NM_0011	chr15	48737938	48811146	-	5608	1	CEP152|M	0.283	0.659
NM_0003	chr18	31591767	31599023	+	922	1	TTR|CTS|(	5.342	1.278
NR_03976	chr22	41923222	41923297	-	75	1	MIR378I|-	0	2.009
NR_02712	chr20	39213781	39224748	-	1510	1	LINC01734	0	0
NM_0326	chr20	31637888	31644997	+	677	1	COX4I2|C(	0	0
NM_0010:	chr14	61277371	61281812	-	4439	1	TMEM30B	0.009	0.003
NM_0013	chr7	1.08E+08	1.08E+08	-	3718	1	LAMB4|-|-	0.087	0.055
NM_0013	chr7	55019021	55208080	+	5528	1	EGFR|ERB	1.322	4.197
NM_0582	chr1	84408351	84415008	+	1003	1	DNASE2B|	0.836	0.191
NM_0030	chr19	47778585	47784686	+	872	1	SELENOW	253.286	230.509
NR_12585	chr6	1.34E+08	1.34E+08	-	1183	1	LOC10192	0	0
NM_0013	chr19	18636965	18670499	+	1747	1	KLHL26|-|	7.978	9.871
NR_02707	chr3	1.39E+08	1.39E+08	-	530	1	PISRT1|NC	0	0
NM_0013	chr2	9423537	9473110	+	2237	1	CPSF3|CPS	20.427	20.23
NM_1942	chr5	1.39E+08	1.39E+08	-	670	1	SPATA24|(	4.522	4.334
NM_0054	chr12	6747992	6752916	-	1380	1	MLF2|NTN	195.121	203.837
NM_1390	chr15	99971438	1E+08	-	6334	1	ADAMTS17	0.085	0.616
NM_0013	chr19	50152548	50163281	-	934	1	IZUMO2|C	0	0
NM_0062	chr12	11265914	11269707	-	1094	2	PRB3|G1|	0	0
NM_0012	chr3	51826883	51830858	+	1259	1	IQCF3|-|-|	0.02	0.033
NM_1522	chr4	7043429	7057950	+	4306	1	TADA2B|A	8.677	9.985
NM_0176	chr13	1.03E+08	1.03E+08	+	3851	1	BIVM|-|-|	7.352	9.119
NR_04652	chr13	66825169	66915031	+	532	1	PCDH9-AS	0	0
NM_0013	chr2	1.28E+08	1.28E+08	-	4023	1	SAP130|-|	18.239	20.666
NM_0011	chr8	85328229	85341714	-	1187	1	CA1|CA-I|	0	0.115
NM_0324	chr5	1.42E+08	1.42E+08	-	4786	1	PCDH1|PC	11.999	15.503
NM_1391	chr6	1.5E+08	1.5E+08	-	952	1	RAET1E|LE	0.054	0.029
NR_14454	chr7	1.08E+08	1.08E+08	-	729	1	LOC10192	3.414	4.058
NM_0011	chr3	1.57E+08	1.57E+08	+	3675	1	TIPARP|AF	3.529	6.739
NM_0031	chr11	1.21E+08	1.22E+08	+	10923	1	SORL1|C1	2.268	2.295
NM_0010	chr7	1.44E+08	1.44E+08	-	931	2	OR2A42|-	0	0
NM_0002	chr17	40861178	40867210	-	1982	2	KRT12|K1	0	0
NM_0013	chr6	82169987	82247754	-	5974	1	IBTK|BTBD	5.601	5.356
NM_1452	chr1	2.28E+08	2.28E+08	-	2683	1	TRIM11|B	12.302	12.099
NM_1991	chr5	1.71E+08	1.71E+08	+	1350	1	NPM1|B23	49.072	60.521
NM_0011	chrX	10469265	10567603	-	1651	1	MID1|BBB	10.816	13.705
NM_0013	chr5	43121461	43176324	+	3028	1	ZNF131|ZE	6.017	8.816
NM_0148	chr5	95731146	95796367	+	5552	1	RHOBTB3|	12.61	5.696
NM_1307	chr20	23450404	23452845	-	548	1	CST11|CST	0	0
NM_0021	chr6_GL0	4453378	4458806	-	3476.9	7	HLA-DOA|	0	0
NR_13489	chr6	42929130	42963880	+	1269	1	CNPY3-GN	8.535	9.779
NR_03896	chr7	1.02E+08	1.02E+08	+	2902	1	LOC10063	18.958	20.645
NM_0010	chr11	1584342	1585283	-	939	3	KRTAP5-1|	0	0

NM_0806	chr12	1.25E+08	1.25E+08	+	1267	1	BRI3BP|BN	2.921	2.789
NR_03893	chr10	33759713	33772680	-	2955	1	LINC00838	0	0
NM_0013	chr1	45343883	45491168	-	2756	1	TESK2|-|-|	1.263	1.252
NR_04632	chr2	96208416	96242623	+	2478	1	STARD7-AS	5.843	5.771
NM_0011	chr15	69014695	69057161	+	2702	1	NOX5|-|-|	0.014	0.005
NM_0014	chr10	5765223	5813549	-	2399	1	GDI2|HEL-	118.723	120.344
NR_00334	chr15	25189414	25189495	+	81	4	SNORD115	0	0
NR_00326	chr22_KI2	69142	86923	-	2023.5	2	GGT3P|GG	0	0
NM_0033	chr6	80004605	80042527	+	2986	1	TTK|CT96|	0.613	1.693
NM_0013	chr9	85941142	86022290	+	2616	1	NAA35|EG	8.29	11.095
NM_0013	chr2	1.32E+08	1.32E+08	+	1861	1	ZNF806|-|	0.159	0.213
NM_0184	chr12	8032763	8055522	+	5469	1	FOXJ2|FH	10.069	13.479
NM_0012	chr3	1.34E+08	1.34E+08	-	4987	1	AMOTL2|L	17.777	22.228
NM_0034	chr19	57614219	57624717	+	4935	1	ZNF134|pI	8.721	11.643
NR_03626	chr2	2.32E+08	2.32E+08	+	81	4	MIR1244-2	10.506	14.712
NM_1523	chr1	28592200	28594576	+	1079	1	RAB42|-|-	0.275	0.241
NM_0010	chr17	38057693	38068634	+	2089	7	TBC1D3C|	0	0
NM_0190	chr5	55256055	55307722	-	4663	1	DHX29|DD	4.087	5.775
NM_0010	chr4	69931081	69946570	+	937	1	CSN1S1|C	0.028	0.015
NM_0047	chr2	1.7E+08	1.7E+08	+	2690	1	PPIG|CARS	4.299	7.673
NR_00367	chr4	1.44E+08	1.44E+08	+	1988	1	GUSBP5|G	0.006	0.014
NM_1523	chr14	1E+08	1E+08	-	2350	1	SLC25A29|	59.919	58.268
NM_0011	chr22	44172956	44208469	+	3447	1	PARVG|-|-	0.041	0.099
NR_11079	chr18	40066286	40099233	+	1222	1	LINC01477	0	0
NR_10461	chr5	3177835	3181232	+	647	1	LINC01377	0.12	0.021
NM_0809	chr7	1.24E+08	1.24E+08	+	2731	1	ASB15|-|-	0.024	0
NM_0033	chr10	1E+08	1E+08	+	1988	1	WNT8B|-|	0.324	0.083
NM_0011	chr9	65282101	65285209	-	3106	1	FOXD4L5|	0.033	0.053
NM_0323	chr9	1.01E+08	1.01E+08	-	4281	1	TMEM246	21.772	18.525
NM_0050	chr1	1.61E+08	1.61E+08	-	4338	1	ADAMTS4	1.584	11.241
NM_0010	chr6	1.27E+08	1.28E+08	-	11804	1	SOGA3|C6	18.448	23.848
NR_03660	chr19	52797408	52821669	-	4464	1	ZNF28|KO	0.965	2.666
NM_0164	chr14	95534986	95544718	+	1167	1	GLRX5|C1	33.93	26.913
NM_0005	chr2	2.33E+08	2.33E+08	+	1731	1	SAG|RP47	0	0
NM_0314	chr9	1.31E+08	1.31E+08	+	3429	1	AIF1L|C9o	21.813	21.123
NM_0064	chr16	367384	370569	-	1132	1	MRPL28|N	84.468	74.922
NM_0582	chr7	1.57E+08	1.57E+08	+	2464	1	DNAJB6|D	25.588	19.089
NR_02664	chr15	24164779	24169906	-	5127	1	PWRN2|N	0	0
NM_0013	chr17	48048305	48061545	+	4796	1	NFE2L1|LC	77.931	81.4
NM_0309	chr14	1.03E+08	1.03E+08	+	1543	1	AMN|PRO	0.142	0.142
NM_2073	chr8	664200	731239	-	1805	1	ERICH1|HS	4.136	4.879
NM_0010	chr3	98168700	98169641	+	940	1	OR5H15|-	0	0
NM_1785	chr4	1.88E+08	1.88E+08	+	1885	1	TRIML1|RI	0	0
NM_0146	chr16	31074422	31083512	+	6203	1	ZNF646|-|	12.59	11.562
NM_0010	chr11	4946159	4947100	-	940	1	OR51A4|-	0	0
NR_03771	chr17	7012417	7017524	+	1901	1	RNASEK-C	116.048	114.216
NM_0182	chr2	3497919	3519579	-	1506.5	2	ADI1|APL1	1.472	1.246
NM_0013	chr16	4696510	4734377	-	2084	1	ANKS3|-|-	11.675	10.766
NR_13460	chr6	44546161	44551881	-	746	1	LOC10537	0	0
NM_0013	chr3	49029707	49094373	-	3122	1	QRICH1|-|	44.863	53.463

NR_11000	chr3	69999744	70015300	+	2022	1	SAMMSON	0	0
NM_0004	chr17	41619440	41624630	-	1564	1	KRT17|39.	0.033	0.21
NM_1816	chr1	2.06E+08	2.06E+08	+	4076	1	MFSD4A|N	0.604	0.591
NM_0013	chr19	53235381	53254850	-	3717	1	ZNF677|-|	0.867	1.898
NR_10674	chr17	37614931	37615039	-	108	2	MIR378J|h	0	0
NM_0012	chr1	1.83E+08	1.83E+08	+	1843	1	NPL|C112	0.833	1.427
NM_1471	chr7	91940875	92110673	+	12387	1	AKAP9|AK	1.603	3.033
NM_0010	chr19	1103926	1106789	+	942	1	GPX4|GPx	329.632	218.643
NM_0013	chr10	13643706	13707567	-	5899	1	FRMD4A|(	31.107	23.219
NM_0041	chr13	28003274	28100592	-	3816	1	FLT3|CD13	0	0.025
NR_02699	chr15	77993233	77994225	-	992	1	LOC91450	0.143	0.304
NM_1523	chr1	18480930	18485986	+	5054	1	KLHDC7A|	0.01	0.011
NR_11191	chr3	50365334	50368197	+	958	1	CYB561D2	8.775	8.879
NM_0011	chr20	46021690	46060150	+	6013	1	SLC12A5|E	4.533	6.046
NM_0012	chr6	28349914	28369177	+	4254	1	ZKSCAN3|Z	4.355	4.273
NM_1537	chr6	1.17E+08	1.17E+08	+	9781	1	FAM26E|C	2.829	1.102
NR_12049	chrX	72938163	72938958	+	450	2	FAM236A|	0	0
NR_10226	chr10	68414064	68472121	-	4110	1	DNA2|DNA	0.998	1.62
NR_02724	chr18	64080009	64149026	-	865	1	LINC00305	0.03	0
NM_0010	chr20	44302838	44311252	-	4676	1	FITM2|C20	4.39	5.056
NM_0013	chrX	1.53E+08	1.53E+08	-	1940	2	MAGEA2B	0	0
NM_0011	chr7	73302516	73308867	-	2360	1	NSUN5|NO	6.944	5.27
NM_0013	chr11_KI2	124171	130157	+	1205	2	LOC10192	0	0
NM_0012	chr11	1.18E+08	1.18E+08	-	2174	1	JAML|AMI	0	0.006
NR_00303	chr12	54608187	54610462	-	777	1	GLYCAM1|	0	0
NM_0010	chr15	82262811	82284926	+	3125	1	SAXO2|FA	0.256	0.21
NM_0223	chr1	1.8E+08	1.8E+08	-	7069	1	TOR1AIP2	3.657	4.162
NM_0163	chr1	1.48E+08	1.48E+08	-	1796	1	ACP6|ACP	4.157	3.676
NM_0011	chr11	1.19E+08	1.19E+08	-	758	1	CCDC153|-	0.953	0.831
NM_0160	chr22	37849372	37888782	+	2076	1	EIF3L|EIF3	254.423	316.8
NR_03340	chr19	1952527	1954549	-	1310	1	CSNK1G2-A	0.089	0.021
NM_0153	chr8	30095406	30108071	+	2706	1	LEPROTL1	26.688	28.751
NR_04632	chr1	2.03E+08	2.03E+08	+	762	1	PCAT6|KD	16.244	14.2
NR_03617	chr10	1.3E+08	1.3E+08	-	75	1	MIR4297|-	0	0
NM_0062	chr14	94363638	94366702	-	1197.5	2	SERPINA2|	0	0
NR_13074	chr12	1.26E+08	1.26E+08	+	2441	1	LOC10012	0	0
NR_00258	chr11	811681	811814	+	133	1	SNORA52|	3.975	1.751
NM_0013	chr11	67056818	67072017	+	930	1	RHOD|ARH	0.042	0.088
NR_02966	chr8	64379149	64379257	+	108	1	MIR124-2|	0.358	0
NR_04984	chr13	92490163	92490220	+	57	1	MIR548AS	0	0
NM_1334	chr1	40861056	40862346	-	1288	1	CITED4|-|-	4.325	3.775
NM_0306	chr22	35648377	35668409	+	10134	1	APOL6|AP	0.008	0.031
NM_0010	chr1	13254198	13263435	+	1849	2	PRAMEF5|	0	0
NM_1445	chr1	2.13E+08	2.13E+08	-	4687	1	ANGEL2|C	4.82	5.859
NR_02677	chr10	42513511	42552870	-	8704	1	ZNF37BP|I	1.188	3.223
NR_12536	chrX	45848074	45851490	-	372	1	LOC40158	0.069	0.037
NM_0012	chr13	48075728	48095141	-	2172	1	MED4|ARC	13.885	12.877
NM_0013	chr21	43741384	43762307	+	7218	1	PDXK|C21	14.183	18.735
NM_0012	chr22	29205912	29259597	+	2089	1	EMID1|EM	11.857	13.074
NM_0013	chr6	13615327	13632470	+	902	1	NOL7|C6o	32.048	31.919

NR_10429	chr1	1.63E+08	1.63E+08	-	2121	1	LOC10042	0	0
NM_0013	chr12	1.25E+08	1.25E+08	+	3119	1	AACS|ACS	17.16	22.01
NM_0142	chr5	1.71E+08	1.71E+08	+	3297	1	GABRP|-|-	0.016	0.025
NR_07724	chr12	97730048	97756517	-	2942	1	LOC64371	0.18	0.042
NM_0011	chr19	3474407	3480542	-	902	1	SMIM24|C	0.229	0.197
NM_0530	chr11	66016752	66026517	-	2605	1	CATSPER1	0.035	0.042
NM_0021	chr9	21206181	21207143	-	960	1	IFNA10|IF	0	0
NM_0011	chr6	31661229	31665862	-	2657.4	7	GPANK1|A	0	0
NR_12588	chr4	1.33E+08	1.33E+08	-	2813	1	LOC10192	0.72	0.813
NM_0200	chr1	2.4E+08	2.4E+08	+	6409	1	FMN2|-|-|	13.149	8.284
NM_1985	chr10	73744373	73772175	+	4433	1	SEC24C|-|	50.966	52.313
NM_0204	chr11	66314487	66317044	-	2555	1	CD248|CD	0.923	0.606
NR_03714	chr6	42727576	42728194	+	618	1	ATP6V0CP	0	0
NR_01544	chr7	4973985	4998169	+	2251	1	RNF216P1	15.047	14.275
NM_0012	chr6	1.23E+08	1.23E+08	+	1748	1	SMPDL3A|	2.884	3.706
NR_13076	chrX	1.54E+08	1.54E+08	+	1368	1	LCA10|LC	0.594	0.38
NR_11023	chr2	2.19E+08	2.19E+08	-	1003	1	LINC01494	0.026	0.014
NM_0010	chr3	1.65E+08	1.65E+08	-	5962	1	SI|-|-|3q2	0.002	0.007
NM_0001	chr19	5830626	5839731	-	3132	1	FUT6|FCT	0.004	0.048
NM_0224	chr7	71132537	71713601	+	3505	1	WBSCR17|	8.093	19.168
NM_0011	chr3	71771655	71785206	-	1607	1	PROK2|BV	0.072	0.128
NR_13296	chr1	39514866	39516669	+	1803	1	OXCT2P1|	0.029	0.023
NR_10461	chr5	987177	997340	-	3515	2	LOC10050	0	0
NR_02424	chr19	47923779	47923899	+	120	10	SNAR-A14	0	0
NR_00329	chr15	25172635	25172716	+	81	1	SNORD115	0	0
NM_0181	chr3	1.09E+08	1.09E+08	-	2808	1	DPPA4|24	7.37	7.766
NM_0012	chr12	57625895	57632681	-	2416	1	B4GALNT1	35.916	36.283
NM_0190	chr4	1.38E+08	1.38E+08	-	5921	1	PCDH18|P	6.724	4.444
NM_0055	chr10	1.23E+08	1.23E+08	+	1624	1	HMX2|H6	0.016	0
NM_0012	chr1	2.45E+08	2.45E+08	+	1167	1	EFCAB2|C	1.713	1.338
NR_11069	chr3	50273119	50277171	+	1720	1	SEMA3B|L	3.538	2.612
NM_0190	chr1	2.33E+08	2.33E+08	+	5452	1	MAP10|KI	0	0.085
NR_11050	chr14	18976750	18980793	-	2565	1	LOC10050	0	0
NR_10977	chr6	57260524	57263346	-	1457	1	LOC10050	0.035	0.019
NM_0012	chr3	50296402	50298889	-	1191	1	NAT6|FUS	9.786	9.994
NM_0165	chr4	1.19E+08	1.19E+08	+	2589	1	MYOZ2|C	0.01	0.106
NM_0202	chr12	1.02E+08	1.02E+08	+	1556	1	CHPT1|CP	5.113	6.884
NM_0161	chr19	40778219	40796944	+	1179	1	RAB4B|-|-	37.718	36.328
NM_0147	chr6	1.27E+08	1.27E+08	-	8116	1	KIAA0408|	15.728	22.945
NM_0180	chr11	66070353	66244747	+	4467	1	PACS1|MF	35.061	46.899
NR_13211	chr13	95672838	95676925	-	2903	1	DNAJC3-A	1.177	1.17
NR_02685	chr15	30103732	30131745	+	1141	2	ULK4P3|D	0	0.024
NR_03983	chr11	2173063	2173138	+	75	1	MIR4686|	0	0
NR_10463	chr5	39520431	39524708	+	596	1	LINC02104	0.022	0
NM_0164	chr3	48673839	48685933	-	2985	1	NCKIPSD|	11.066	10.054
NM_0223	chr11	68261335	68272001	-	2096	1	C11orf24|	19.894	20.402
NM_0328	chr9	35659344	35665281	-	3752	1	ARHGEF39	7.003	6.396
NM_0010	chr19	53467735	53494292	+	4182	1	ZNF813|-|	0.607	0.894
NM_1524	chr1	3752401	3771645	+	2160	1	CCDC27|-|	0.066	0.044
NM_0022	chr2	1.36E+08	1.36E+08	-	6260	1	LCT|LAC|L	0.021	0.018

NM_0010(	chr22	30635815	30647893	+	2547	1	SLC35E4|-	5.26	5.41
NM_1385:	chr6	22569449	22571662	+	2211	1	HDGFL1|P	0.087	0.074
NM_0010	chr19	7069460	7087967	+	5979	1	ZNF557|-|	2.195	2.566
NR_13652	chr19	51271266	51281234	+	823	1	LINC01872	0	0
NM_0011	chr4	1.02E+08	1.02E+08	+	3028	1	BANK1|BA	0	0
NM_0012	chr4	47642835	47838106	-	2409	1	CORIN|AT	0.037	0.057
NM_0011	chr12	48340010	48350891	-	4361	1	ZNF641|-|	12.571	16.36
NM_0071	chr18	54269479	54298234	+	6046	1	POLI|RAD3	1.941	1.534
NM_0013	chr2	37925319	38067137	+	1445	1	RMDN2|B	0.428	0.493
NM_0063	chr9	35732320	35737008	+	1840	1	CREB3|LU	56.752	59.604
NM_0012	chr6	1.34E+08	1.34E+08	-	2262	1	SGK1|SGK	8.282	13.237
NM_1825	chr4	44622337	44651641	-	929	1	YIPF7|Fin	0.18	0.103
NM_0179	chr13	1.13E+08	1.14E+08	+	3071	1	TMCO3|C	20.715	18.942
NR_11069	chr9	10613206	10620420	+	2831	1	PTPRD-AS	0.077	0.029
NM_0160	chr20	59033145	59042846	-	2555	1	PRELID3B|	15.886	11.456
NM_0013	chr9	6757641	7175648	+	4639	1	KDM4C|G	6.101	5.89
NM_0012	chr15	32615144	32639937	+	5131	2	ARHGAP11	0.005	0.003
NM_0011	chr7	1.51E+08	1.51E+08	-	1926	1	ASB10|GL	0	0
NM_1459	chr12_GL3	48058	102080	+	3269	2	SLC5A8|AI	0	0
NR_07341	chr17	18439908	18442894	+	1808	1	KRT16P1|I	0	0
NM_0010	chr1	6244192	6245578	+	691	1	HES3|bHL	1.232	0.377
NR_00221	chr1	1.46E+08	1.46E+08	+	3797	2	NUDT4P1|	0	0
NR_04630	chrY	9349580	9355182	-	463	5	FAM197Y5	0	0
NR_04011	chr18	14179097	14227050	+	3691	1	ANKRD20A	0.007	0.007
NR_03062	chr1	1.66E+08	1.66E+08	-	55	1	MIR921|M	0	0
NR_13073	chr17	69232975	69235012	-	2037	1	PRO1804|	0	0
NR_12037	chr15	82443332	82444869	-	535.2	4	LOC10272	0	0
NR_11060	chr2	66921511	66922490	-	426	1	LINC01628	0	0
NM_0230	chr2	2.41E+08	2.41E+08	+	2167	1	CAPN10|C	15.256	14.133
NM_0012	chr6_GL0C	3127534	3144980	-	3855	7	EHMT2|B	0	0
NM_0023	chr8	97869021	98036718	+	4149	1	MATN2|-|	3.67	3.229
NM_0012	chr4	9325165	9326757	+	1591	8	USP17L30	0	0
NM_0054	chr19	4402663	4443397	+	3326	1	CHAF1A|C	6.567	4.892
NM_1779	chr3	57556247	57563547	+	5501	1	PDE12|2'-	3.152	2.985
NR_13551	chr21	42496539	42497443	+	446	1	LOC10193	0.347	0.092
NM_0314	chr1	6424788	6460944	+	3516	1	ESPN|DFN	11.658	9.443
NR_00218	chr8	1.01E+08	1.01E+08	+	702	1	NACAP1|F	0.496	0.488
NM_0011	chr6	42101119	42142977	-	6735	1	C6orf132|	0.004	0.004
NR_13429	chr8	22254576	22275162	-	513	1	LOC10050	3.82	4.192
NM_0043	chr16	49277918	49281831	-	2429	1	CBLN1|-|-	134.407	20.56
NM_1982	chr2	62500221	62506469	-	1919	1	TMEM17|	2.211	3.29
NR_12165	chr3	1.24E+08	1.24E+08	+	704	1	MYLK-AS1	5.751	5.448
NR_13092	chr17	76679569	76709597	-	1565	1	MXRA7|PS	33.012	40.767
NM_0011	chr22	35299275	35347994	+	2302	1	TOM1|-|-|	15.027	15.298
NR_12652	chr20	63939829	63956447	-	1831	1	UCKL1|UC	29.942	25.307
NM_0011	chr16	87302798	87317420	-	966	1	C16orf95|	3.844	2.524
NR_00349	chr15	25222354	25222427	+	73	1	SNORD115	0	0
NR_13490	chr16	10760919	10818794	-	1646	1	TVP23A|F	1.856	1.864
NM_0159	chr6	43054029	43059545	-	1318	1	MRPL2|C	20.729	19.87
NR_03971	chr12	47364186	47364269	-	83	1	MIR4494|	0	0

NR_03414	chr11	1.16E+08	1.16E+08	-	2240	1	LINC0090C	0.046	0.012
NM_0015!	chrX	70133468	70166323	+	1651	1	IGBP1|ALF	48.435	60.562
NM_0012!	chr5	33440696	33468091	+	2670	1	TARS|ThrF	39.429	45.185
NM_0010:	chrY	20756068	20781032	+	880	1	RPS4Y2|RI	0.22	0.296
NR_02748	chr17	62264706	62275655	-	2048	1	TBC1D3P2	0	0
NM_0014{	chr7	55964577	56000182	+	1992	1	GBAS|NIPS	21.69	21.212
NM_0013:	chr6	1.7E+08	1.7E+08	+	4002	1	C6orf120|	5.171	4.367
NM_0010(	chr11	1.24E+08	1.24E+08	-	928	1	OR8B4|OF	0	0
NR_11025	chr2	6366010	6375422	-	4206	1	LINC01247	0	0
NM_0071;	chr13	99981772	99986765	+	2970	1	ZIC2|HPE5	47.124	22.238
NR_00332	chr15	25056859	25056955	+	96	2	SNORD116	0	0.143
NR_13640	chr17	43938261	43938962	+	537	1	LINC01976	0	0
NR_03657	chr19	18249950	18255419	-	683	1	LOC72996	0	0.02
NM_0010(	chr9	1.23E+08	1.23E+08	+	931	1	OR1L1|HG	0	0
NM_0023:	chr5	1.22E+08	1.22E+08	-	5309	1	LOX|AAT1	0.432	0.529
NM_0010(	chr13	25301528	25342423	+	4282	1	NUP58|NU	12.102	12.206
NR_10689	chr7	44051766	44051829	+	63	1	MIR6837|	22.512	16.306
NR_12633	chr20	58863756	58888810	-	678	1	LOC10192	0.057	0.04
NM_0012{	chr11	93741594	93763102	+	2578	1	C11orf54|	5.602	5.515
NM_0011‹	chr17	49223370	49230766	-	2168	1	PHOSPHO:	0.036	0.069
NR_02444	chr17	8358413	8360541	-	2128	1	LOC10012:	4.508	4.383
NM_1768{	chr12_GL8	327525	328424	-	898	2	TAS2R45|(	0	0
NM_0004‹	chr17	64319416	64390878	-	6812	1	PECAM1|(	0.006	0.026
NM_0010(	chr11	4449295	4450361	+	1064	1	OR52K2|C	0	0
NM_0012(	chr6	1.68E+08	1.68E+08	+	7425	1	AFDN|AF6	37.722	49.132
NM_0221‹	chr7	1.28E+08	1.28E+08	-	3703	1	LRRC4|NA	12.448	11.759
NR_03886	chr1	1137017	1144054	+	1615	1	LINC01342	0	0
NR_03892	chr10	22252072	22258548	+	6476	1	LOC10013(	0	0.023
NM_1735!	chr6	37005647	37029069	+	3027	1	FGD2|ZFY'	0.017	0.009
NM_0012{	chr11_KI2	106102	142116	+	5531.5	2	PHRF1|PPI	0.114	0.121
NR_12591	chr4	53695901	53734358	+	1613	1	LOC10050(	0	0
NM_0013‹	chr14	77335021	77377109	-	7778	1	TMED8|FA	11.405	8.689
NM_0160:	chr17	59697306	59707598	-	898	1	PTRH2|BIT	8.687	12.95
NR_10695	chrX	53195411	53195488	-	77	1	MIR6895|	0.335	0
NM_1525:	chr22	41699514	41799455	+	3973	1	MEI1|SPA	0.039	0.024
NM_0010(	chr19	16719976	16817963	+	7624	1	NWD1|-|-	0.1	0.174
NM_0201{	chr3	1.29E+08	1.29E+08	+	1611	1	HMCES|C3	26.771	29.154
NM_0219(	chr3	1.25E+08	1.25E+08	-	9532	1	ZNF148|BI	2.049	5.913
NM_0332;	chr6	30163206	30172696	+	2215	8	TRIM15|R	0	0
NR_03887	chr21	17777404	17792509	-	429	1	C21orf91-(	0.03	0.16
NM_0011:	chr8	55879827	56012447	+	4080	1	LYN|JTK8|	0.313	0.577
NM_0013:	chr11	13492045	13496175	-	842	1	PTH|PTH1	0	0
NM_0010;	chr18	26133852	26193355	+	1802	1	PSMA8|PS	0	0
NM_0048:	chr15	82848977	82952724	-	1948	1	HOMER2|/	6.341	8.578
NR_03978	chr6	41598723	41598788	+	65	1	MIR4641|-	10.91	16.436
NR_02702	chr1	2.04E+08	2.04E+08	-	1289	1	LINC00628	0	0
NM_0012‹	chr22	44069117	44169232	+	1568	1	PARVB|CG	1.053	0.751
NR_03886	chr6	1.14E+08	1.14E+08	-	2747	1	LINC01268	0.061	0.08
NM_2068!	chr19	36818322	36838384	-	2136	1	ZNF790|-|	1.461	2.167
NR_03823	chr8	56518319	56559823	-	2517	1	LINC00968	0.01	0

NM_0011(	chr4	1.69E+08	1.69E+08	+	4413	1	PALLD|CG	10.936	20.137
NM_0175	chr3	1.87E+08	1.87E+08	-	914	1	CRYGS|CR	1.129	1.544
NR_13507	chr1	53851719	53889831	-	1560	1	YIPF1|DJ1	32.606	39.195
NR_03741	chrX	20017088	20017187	-	99	1	MIR23C|h	0	0
NR_03031	chr6	1.07E+08	1.07E+08	+	95	1	MIR587|M	0	0
NR_10682	chr16	50292616	50292675	+	59	1	MIR6771|	0	0
NR_01542	chr2	88765902	88806595	+	1713	1	ANKRD36E	0.008	0
NR_10433	chr12	53750447	53751835	-	579	1	CISTR|CIST	0.134	0.166
NM_0005	chr16	172847	173710	+	600	1	HBA2|HBA	0.129	0
NM_0158	chr7	64974452	64991036	-	6398	1	ZNF117|H	2.866	4.584
NR_02996	chr14	1.01E+08	1.01E+08	+	113	1	MIR431|M	2.853	2.545
NM_0204	chr2	71182738	71227103	-	6294	1	PAIP2B|-|	4.357	3.765
NM_0010	chr2	1.82E+08	1.82E+08	-	2859	1	CERKL|RP2	0.356	0.067
NM_0142	chr19_KI2	101019	144455	-	1575.3	63	KIR2DL1|C	0	0
NR_13564	chr17	2017716	2020706	+	1912	1	LOC10537	0.047	0.014
NM_0010	chrX	1.52E+08	1.52E+08	+	1716	1	MAGEA4|	0	0
NM_0190	chr2	2.34E+08	2.34E+08	+	2389	1	UGT1A8|U	0.032	0
NM_0021	chr11	68903851	68940601	+	3937	1	IGHMBP2|	7.532	9.571
NM_0051	chr18	214520	268059	-	2097	1	THOC1|HP	7.55	7.642
NM_1817	chr11	2461454	2849110	+	2306	2	KCNQ1|AT	0.017	0.095
NM_0190	chr18	68673688	68715116	-	4727	1	TMX3|PDI	3.434	4.694
NM_2070	chr1	66837599	66924887	-	1736	1	WDR78|D	1.404	1.278
NR_12063	chr10	10419538	10462349	-	501	1	LOC10192	0.051	0.027
NR_13582	chr1	2.19E+08	2.2E+08	-	683	1	LOC10272	0	0.12
NM_0011	chr8	42271302	42332653	+	3985	1	IKBKB|IKK	5.07	5.733
NR_02689	chr9	76398599	76400038	+	1439	1	RPSAP9|L	0.134	0.124
NM_0206	chr16	4461677	4474895	-	1371	2	NMRAL1|	0	0
NM_0012	chr4	1.03E+08	1.03E+08	-	8200	1	CENPE|CE	0.131	0.281
NR_13563	chr19	28606688	28615229	+	4449	1	LOC10272	0.012	0.003
NR_03412	chr10	17386936	17408286	+	554	1	ST8SIA6-A	0.07	0.148
NM_1527	chr3	1.96E+08	1.96E+08	-	672	1	TCTEX1D2	44.341	27.476
NM_0012	chr22	22887780	22895833	+	935	1	IGLL5|IGL|	0	0
NM_1524	chr19	36182060	36214673	-	2016	1	ZNF565|-|	3.62	5.102
NR_12036	chr15	82088594	82097693	+	2561	1	LINC01583	0	0.021
NM_0220	chr14	90061765	90185857	+	2518	1	KCNK13|K	0.748	0.196
NM_1731	chr2	1.13E+08	1.13E+08	-	1045	1	IL36B|FIL1	0	0
NM_1985	chr5	43486701	43515171	-	2518	1	C5orf34|-|	0.42	0.647
NM_0018	chr22	20774095	20787720	+	2214	1	SERPIND1|	0.006	0.019
NR_03661	chr8	95946985	95948348	-	1363	1	LOC10050	0	0
NM_0156	chr17	27294080	27313619	+	2821	1	WSB1|SW	48.205	40.232
NM_0145	chr19_KI2	116409	142846	-	1464.4	37	KIR2DS5|C	0	0
NM_0022	chr17	41476689	41481140	-	1661	1	KRT35|HA	0	0
NM_0011	chr4	653177	670892	+	2760	1	PDE6B|CS	1	1.424
NM_0012	chr10	74826413	75032622	+	7603	1	KAT6B|GT	9.59	13.185
NR_10704	chr19	42351131	42351205	+	74	1	MIR8077|	0.697	0.37
NM_0059	chr7	1.01E+08	1.01E+08	+	11347	1	MUC3A|M	0.126	0.297
NR_13552	chr19	41114432	41128381	+	1779	1	CYP2F1|C	0	0
NM_0010	chr7	1.22E+08	1.23E+08	-	5901	1	CADPS2|C	2.945	1.005
NR_03977	chr5	1.29E+08	1.29E+08	+	78	1	MIR4633|	0	0
NR_02707	chr15	74126373	74129278	-	2564	1	LOC28373	1.398	0.86

NR_04654	chr13	1.13E+08	1.13E+08	+	1560	1	GRTP1-AS1	0.537	0.579
NR_13804	chr9	1.35E+08	1.35E+08	-	334	1	COL5A1-AS	0	0.041
NM_0013(	chr1	1020123	1056119	+	7353	1	AGRN|CM	46.58	51.953
NM_0207:	chr19	12576106	12610809	-	6091	1	ZNF490|-|	2.403	2.633
NM_0806(	chr11	65077455	65084143	-	2568	1	CDCA5|SO	8.48	4.699
NM_0246(	chr15	75346990	75355251	+	1884	1	NEIL1|FPG	4.004	3.097
NR_11053	chr22	50583026	50595281	+	681	1	CHKB-AS1	3.541	3.057
NR_00219	chr11	1995176	1997875	-	2343	2	H19|ASM|	0	0
NM_0013(	chr7	1.06E+08	1.06E+08	+	6277	1	CDHR3|CD	0.571	2.621
NR_00295	chrX	54814370	54814500	+	130	1	SNORA11|	2.975	2.002
NM_0068(	chr1	37956980	37990110	-	2838	1	SF3A3|PRI	40.561	36.776
NR_11070	chr19	36573070	36594716	+	893	1	ZNF529-AS	3.278	2.638
NM_0212	chr10	73631612	73641757	-	1575	1	MYOZ1|CS	0.72	0.278
NM_0210(	chr6	26198784	26199293	-	507	1	HIST1H2AI	0.458	0.324
NR_12593	chr4	1.55E+08	1.55E+08	+	446	1	LOC10272	0.347	0.983
NM_0148!	chr1	1.54E+08	1.54E+08	-	5658	1	DENND4B	16.366	15.692
NM_0058:	chrX	48893447	48898143	-	941	1	TIMM17B|	66.016	48.704
NM_0160(	chr2	30146884	30160533	+	2254	1	YPEL5|CGI	38.309	43.789
NM_0013:	chr4	39698044	39782790	+	5146	1	UBE2K|E2	10.849	13.447
NM_0012!	chr19	40321060	40348409	-	2088	1	C19orf47|	11.195	12.608
NM_0246:	chr22	19846138	19854848	-	6630	1	C22orf29|	6.169	7.098
NR_00322	chr14	1.01E+08	1.01E+08	+	68	1	SNORD11	0	0
NM_0176!	chr19	45692483	45703990	+	2302	1	QPCTL|gQ	3.82	2.975
NM_0149:	chr15	1.01E+08	1.01E+08	-	4545	1	CHSY1|CH	9.858	10.093
NM_0012!	chrX	1.05E+08	1.05E+08	-	1365	1	TEX13A|-|	0	0
NM_0013:	chr9	36190856	36212062	+	866	1	CLTA|LCA|	193.194	178.03
NM_0012!	chr1	24514314	24537020	+	2178	1	RCAN3|DS	1.575	8.226
NM_0146!	chr16	4958317	5019155	+	6438	1	SEC14L5|F	0.373	0.602
NM_0013(	chr5	1.35E+08	1.35E+08	+	6459	1	JADE2|JAD	1.048	1.351
NM_0012{	chr1	39955112	39969968	+	1900	1	MFSD2A|N	17.8	14.094
NR_02698	chr1	87129765	87169203	+	1400	1	LINC0114C	0.037	0.02
NR_02671	chr17	18665923	18673181	-	7258	1	FOXO3B|F	2.62	2.342
NM_1527(	chr17	7402974	7404131	-	475	1	TMEM256	59.011	43.254
NM_0013(	chr11	1.23E+08	1.23E+08	+	1916	1	CRTAM|CI	0.047	0.007
NM_0012!	chr11	66480013	66509659	+	2971	1	DPP3|DPP	25.748	23.402
NM_0010	chr2	2.37E+08	2.38E+08	+	3671	1	MLPH|SLA	0.341	0.134
NM_0011(	chr11	1.14E+08	1.14E+08	+	688	1	CLDN25|-|	0	0
NR_03064	chr3	1.9E+08	1.9E+08	+	87	1	MIR944|M	0	0
NM_0165(	chr5	1.34E+08	1.34E+08	-	1609	1	CDKL3|NK	2.773	2.086
NM_0321:	chr10	1.01E+08	1.01E+08	-	1011	1	MRPL43|L	49.521	45.535
NM_1446(	chr16	49373808	49399431	+	1076	1	C16orf78|	0.048	0
NM_0041:	chr7	93921704	93926514	+	950	1	GNG11|GI	75.502	34.935
NM_0160:	chr8	91070196	91087095	+	3289	1	OTUD6B|(	2.219	2.503
NM_0012!	chrX	49205063	49233371	-	5825	1	CACNA1F|	0.007	0.021
NM_0062	chr19	46346994	46390975	+	2217	1	PPP5C|PP!	60.385	64.889
NR_02790	chr6	33586106	33593338	-	2105	1	LINC0033E	0.025	0.046
NR_03025	chr14	1.01E+08	1.01E+08	+	77	1	MIR539|M	0	0
NM_2014!	chr9	68241945	68300035	+	1706	1	CBWD3|b/	0	0
NM_0121:	chr12	1.17E+08	1.17E+08	+	4644	1	FBXW8|FB	7.53	7.055
NR_00392	chr9	14993327	15019724	+	918	1	LOC38970	1.18	0.925

NR_13664	chr18	79638928	79679745	-	1106	2	LOC28424	0	0
NM_0013:	chr9	1.29E+08	1.29E+08	-	1122	1	ZDHHC12|	7.113	4.688
NR_04620	chr8	30382119	30385401	-	906	1	RBPMS-AS	0.028	0.015
NR_04653	chr13	93818424	93858473	-	644	1	GPC6-AS2|	0	0
NR_03621	chr1	1.12E+08	1.12E+08	-	63	1	MIR4256|-	0.409	0
NM_0013(	chr17	28703197	28711091	-	1579	1	PROCA1|-	8.574	4.424
NM_0011(	chr11	71434411	71448431	-	2631	1	DHCR7|SL	47.879	46.193
NM_0011(	chr11	17385249	17389331	-	2752	1	KCNJ11|BI	2.394	4.604
NM_0012{	chr7	43112599	43563339	+	6472	1	HECW1|NI	7.283	21.923
NM_0005(	chr1	1.5E+08	1.5E+08	+	2168	1	FCGR1A|C	0.065	0.063
NM_0012(	chr10	16436943	16513745	+	3206	1	PTER|HPH	0.374	0.056
NM_0013:	chr17_KI2	1611993	1628727	+	1670	2	C17orf78|	0	0
NM_0050:	chr17	4945650	4949086	-	1360	1	PFN1|ALS:	184.784	157.193
NM_0010	chr15	90529886	90645345	+	5231	1	CRTC3|TO	8.353	12.197
NR_13069	chr2	2.42E+08	2.42E+08	+	768.7	3	LINC01881	0.151	0.41
NM_0036!	chr3	9757345	9769984	-	1478	1	CAMK1|C/	13.766	13.428
NM_0011	chr15	49621029	49645136	+	2615	1	DTWD1|M	0.976	1.299
NM_0046(	chr6	1.33E+08	1.33E+08	-	3835	1	VNN1|HDI	0	0
NM_0030	chr14	69775835	69797289	-	1624	1	SLC10A1|I	0.064	0.084
NM_0050{	chr12	26195573	26234775	+	4526	1	SSPN|DAG	0.367	0.605
NM_0010:	chr3	1.19E+08	1.19E+08	-	3423	1	IGSF11|BT	0.753	4.546
NM_0210:	chrX	1.01E+08	1.01E+08	+	874	1	RPL36A|L3	0.974	0.925
NM_1334{	chr12	57619910	57626151	+	2393	1	SLC26A10|	17.7	20.291
NM_0042!	chr17	75626845	75667189	-	3691	1	RECQL5|R	7.685	7.44
NM_0330	chr12	52377812	52385633	-	2062	1	KRT84|HB	0	0
NM_0012!	chr3	55465715	55481398	-	5575	1	WNT5A|h\	0.925	3.58
NM_0010:	chr14	22900645	22919187	-	2488	1	RBM23|C/	54.072	54.975
NM_0007!	chr11_KI2	167178	170576	+	1356	2	DRD4|D4D	0	0.01
NR_03416	chr1	1.09E+08	1.09E+08	+	532	1	SPATA42|/	0	0.103
NR_03990	chr19	49854591	49854651	+	60	1	MIR4749|	95.627	71.453
NM_0808:	chr9	1.37E+08	1.37E+08	+	2109	1	SLC34A3|I	0.587	0.403
NR_13424	chr5	1.39E+08	1.39E+08	-	589	1	LOC10537!	0.175	0.023
NR_00000	chr1	92837289	92837383	+	94	1	SNORD21|	1.783	1.311
NR_11023	chr17	50468329	50474845	+	872	1	ACSF2|AC!	5.914	6.079
NM_0202	chr22	38681949	38684761	+	1373	1	TOMM22|	62.373	55.816
NR_12047	chr12	80763154	80770715	+	1255	1	LINC0149C	0	0
NR_03788	chr2	2.01E+08	2.01E+08	-	2047	1	LINC01792	0.082	0
NR_12642	chr6	31764136	31765588	-	633	6	SAPCD1-A!	0	0
NM_0013:	chr1	74026018	74198187	-	2375	1	LRRIQ3|LR	0.125	0
NR_03611	chr10	1.02E+08	1.02E+08	-	80	1	MIR3158-:	0	0
NM_1737:	chr17	12991612	13018064	-	3775	1	ELAC2|CO	21.289	19.245
NM_1526	chr12	1946048	2004535	-	2105	1	DCP1B|DC	10.878	9.793
NR_10370	chr21	44506044	44516575	+	2546	1	TSPEAR-A!	2.785	1.275
NM_0044!	chr17	67825664	67984378	+	10831	1	BPTF|FAC:	5.176	7.145
NM_1523:	chr1	24056041	24112175	-	5761	1	MYOM3|-	0.049	0.017
NM_0011:	chr1	2.06E+08	2.06E+08	-	3047	1	RAB29|RA	2.67	8.851
NM_0055:	chr21	33402896	33437521	+	1856.5	2	IFNGR2|AI	0.396	0.524
NM_0018!	chr6	69866556	70212454	+	8673	1	COL19A1|	0.042	0.172
NM_0027(	chr7	1.43E+08	1.43E+08	+	810	2	PRSS1|TRI	0	0
NM_0011!	chr4	1093308	1113564	-	1047	1	RNF212|ZI	0	0.078

NM_0013	chr15	83107486	83137359	+	1666	1	TM6SF1|-|	1.726	1.817
NM_0037	chr6	1.08E+08	1.08E+08	-	1754	1	SNX3|Grd	36.255	39.67
NM_1994	chr1	2.05E+08	2.05E+08	-	7760	1	DSTYK|CA	5.52	7.606
NM_1489	chr10	89579497	89643891	-	6189	1	PANK1|PA	2.542	2.589
NM_1383	chr10	72692131	72887694	+	2939	1	MCU|C10	7.625	10.304
NR_03969	chr9	1.37E+08	1.37E+08	+	70	1	MIR4479|	0	0
NM_0013	chr16	3024019	3027755	+	1306	1	THOC6|W	14.809	15.448
NR_03742	chr16	70529499	70529599	+	100	1	SNORD111	0	0
NM_0230	chr19	51889235	51905052	-	3220	1	ZNF649|-|	8.012	11.034
NM_0016	chr3	57571363	57597488	-	1732	1	ARF4|ARF	127.684	125.922
NM_1386	chr6	51615347	52087625	-	16166	1	PKHD1|AR	0.032	0.039
NR_04980	chr18	67081584	67081686	+	102	1	MIR5011|	0	0
NM_0176	chr6	81745730	81752711	-	5607	1	FAM46A|	3.705	1.182
NM_0140	chr11	63998558	64166113	-	1244	1	MACROD1	9.68	14.677
NR_03016	chr10	17845107	17845193	+	86	1	MIR511|M	0	0
NM_0058	chr19	676389	683392	+	2504	1	FSTL3|FLR	6.23	5.738
NM_1333	chr1	1.99E+08	1.99E+08	-	671	2	ATP6V1G3	0	0
NM_0034	chr1	2.28E+08	2.28E+08	-	479	1	HIST3H3|H	0.215	0
NM_0003	chr9	97674909	97697409	-	1461	1	XPA|XP1|	6.178	6.206
NM_1536	chr11	73950319	73970287	+	1865	1	DNAJB13|	0.353	0.257
NM_0146	chr2	11534116	11642786	+	8447	1	GREB1|-|-	5.652	2.633
NM_0011	chrX	1.35E+08	1.35E+08	-	2982	1	ZNF75D|D	2.062	1.727
NM_0198	chrX	16587999	16712979	-	3855	1	CTPS2|-|-|	13.034	9.97
NM_0046	chr11	36483767	36510313	-	7919	1	TRAF6|MG	3.178	3.601
NR_00439	chr17	76561633	76561706	+	73	1	SNORD1A|	1.06	0.938
NR_13276	chr10	32901334	32901472	-	138	1	SNORA86|	1.962	5.657
NM_0010	chr17	73165021	73171923	+	2960	1	SSTR2|-|-|	32.905	38.796
NR_13653	chr20	56577563	56596126	+	692	1	LINC0171	0	0
NM_2069	chr14	1E+08	1E+08	-	1400	1	DEGS2|C1	0.157	0.294
NM_0012	chr11	66466327	66477337	+	2502	1	PELI3|-|-|	10.817	11.808
NM_0010	chr14	94376747	94390692	-	3192	2	SERPINA1|	0	0
NM_0010	chr11	33015864	33033582	+	1744	1	DEPDC7|T	1.257	0.84
NM_0067	chr9	88388382	88478707	+	4526	1	SPIN1|SPI	35.533	50.17
NR_02986	chr4	1.13E+08	1.13E+08	-	67	1	MIR367|M	0	0
NM_0011	chr5	1.63E+08	1.63E+08	+	2898	1	HMMR|CD	0.436	0.889
NM_0013	chr7	1.51E+08	1.51E+08	+	1896	1	GIMAP1-G	0	0
NM_0013	chr3	19879474	19947025	-	2390	1	EFHB|CFA	0.324	0.499
NM_1982	chr20	37346128	37405432	+	4022	1	SRC|ASV|	44.813	62.44
NM_0023	chrX	30230436	30237493	+	2299	1	MAGEB3|	0	0
NM_0248	chr1	35433490	35557436	-	4789	1	KIAA0319L	29.486	31.564
NR_03159	chr19	10403458	10403538	-	80	1	MIR1181|	19.663	10.102
NM_0211	chr6_GL00	2934713	2951124	-	1997	7	ABHD16A|	0	0
NM_0146	chr1	1.56E+08	1.56E+08	+	3633	1	SLC25A44|	22.305	31.488
NM_1536	chr1	2.13E+08	2.13E+08	+	2329	1	FAM71A|	0.011	0
NM_0012	chr1	26234153	26278810	+	3818	1	CEP85|CC	11.158	14.475
NM_0011	chr2	38875962	38882709	+	734	1	MORN2|B	26.7	24.035
NM_0020	chr8	30578514	30658221	-	1526	1	GTF2E2|F	19.112	22.125
NR_03157	chr2	25328640	25328721	-	81	1	MIR1301|	0	0.338
NM_0808	chr4	1.76E+08	1.76E+08	-	3020	1	ASB5|ASB	0	0
NR_03032	chr8	9741672	9741768	+	96	1	MIR597|M	0	0

NM_0013:	chr19	21142010	21186002	+	3869	1	ZNF431|-|	1.42	2.17
NR_02984	chr22	21653304	21653385	+	81	1	MIR130B|	0	2.536
NR_03333	chr4	1249440	1253007	+	2928	1	CTBP1-AS2	10.489	11.194
NR_04639	chr3	1.75E+08	1.75E+08	-	404	1	NAALADL2	0	0
NM_0012{	chr16	30923055	30948783	+	3651	1	FBXL19|C)	51.51	41.024
NR_04673	chr3	42732575	42745158	+	550	1	CCDC13-A!	0	0.025
NM_0529‹	chr1	53946326	53968168	+	1744	1	LRRC42|dJ	33.357	29.499
NM_0123!	chr17	8249278	8270491	+	5355	1	PFAS|FGAI	11.796	8.978
NM_0334:	chr7	1.18E+08	1.18E+08	-	5945	1	CTTNBP2|	2.349	16.291
NR_04746	chr20	37619298	37623119	-	540	1	LINC0048!	0	0
NM_0042:	chr9	1.07E+08	1.07E+08	-	2880	1	KLF4|EZF|	1.406	0.176
NR_11072	chr1	1.78E+08	1.78E+08	+	1662	1	LINC01741	0	0
NM_0012!	chr7	1.42E+08	1.42E+08	+	7822	2	MGAM2|-	0	0
NM_0011	chr10	92048640	92243279	-	5737	1	CPEB3|-|-	1.863	2.192
NM_0013(	chr10	93757809	93798174	+	2229	1	LGI1|ADLT	1.192	3.681
NM_0011!	chr4	1.69E+08	1.7E+08	-	5510	1	NEK1|NY-I	1.949	2.516
NM_0029	chr19	50723348	50725724	+	1401	1	CLEC11A|(	7.602	6.668
NM_0037:	chr1	56494747	56579584	-	3300	1	PLPP3|Dri	4.915	10.638
NM_0306‹	chr4	1.4E+08	1.4E+08	-	7348	1	SETD7|KM	4.027	7.753
NR_00257	chr13	27255401	27255526	+	125	1	SNORA27|	1.238	4.821
NM_1758!	chr13	28138506	28295338	+	5611	1	PAN3|-|-|	2.976	3.947
NM_0012{	chr15	56365424	56445997	+	1767	1	TEX9|-|-|1	1.496	1.155
NR_02799	chr17	35918081	35930776	-	892	2	RDM1|RAI	0	0
NM_0012{	chr7	1.01E+08	1.01E+08	-	696	1	ZASP|-|-|	4.631	2.952
NR_13427	chr5	17404019	17441694	+	294	1	LOC10192!	0	0.093
NR_02148	chr11	1572741	1599184	+	2263	3	KRTAP5-A!	0	0
NR_02968	chr7	1.58E+08	1.58E+08	-	86	1	MIR153-2|	0	0
NR_00300	chr14	95533355	95533629	-	274	1	SCARNA13	2.635	2.399
NM_0059(	chr11	2944430	2992377	-	2500	2	NAP1L4|N	0.985	1.003
NR_10979	chr8	64373328	64387504	+	6554	1	MIR124-2I	8.001	3.572
NR_03742	chr21	8208473	8208652	+	179	2	MIR3648-1	0	0
NM_1532:	chr19	39538806	39540198	-	1390	1	EID2|CRI2	18.236	18.486
NM_0011‹	chr12	80444346	80680189	+	8019	1	PTPRQ|DF	0.035	0.012
NR_03793	chr4	25160672	25198505	+	1118	1	SEPSECS-A	0.15	0.123
NM_0064:	chr10	15097180	15104257	+	1179	1	RPP38|-|-	9.7	9.759
NM_0024{	chr7	10931953	10940186	-	2052	1	NDUFA4|(	68.558	70.374
NR_02951	chr7	1E+08	1E+08	-	79	1	MIR93|MI	0.979	0.347
NM_0007:	chr7	81946444	82443806	-	7530	1	CACNA2D1	10.409	6.468
NM_0056	chr6	1.46E+08	1.46E+08	-	3459	1	EPM2A|EF	3.332	4.118
NR_02421	chr19	47939541	47939660	+	119	1	SNAR-C4|-	0	0
NM_0037‹	chr2	1.69E+08	1.69E+08	-	3388.5	2	ABCB11|A	0.015	0.024
NM_0060!	chr1	1.46E+08	1.46E+08	-	2886	1	PIAS3|ZMI	16.012	13.825
NM_0179‹	chr18	36187517	36268722	+	2710	1	MOCOS|H	0.2	0.147
NM_0060:	chr18	49562031	49592908	+	4155	1	LIPG|EDL|	5.058	3.985
NM_0023(	chr22	37675606	37679802	+	552	1	LGALS1|G,	18.289	36.327
NM_0012(	chr11	78015715	78080219	-	2336	1	NDUFC2-K	35.54	39.613
NR_00001	chrX	1.54E+08	1.54E+08	+	134	1	SNORA70|	20.302	15.537
NM_0202‹	chr6	1.58E+08	1.59E+08	+	11103	1	TULP4|TU!	6.516	5.275
NR_10383	chr16	54039393	54040373	+	868	1	FTO-IT1|-|	0.357	0.252
NM_0011‹	chrX	7898763	7927739	-	2743	1	PNPLA4|D	0.39	0.629

NR_12580	chr17	50281581	50287855	-	742	1	TMEM92-/	0.07	0
NR_04986	chr7	97964405	97964481	-	76	1	MIR5692C	0	0
NR_03985	chr14	74480133	74480204	-	71	1	MIR4709|(	54.661	51.894
NR_03035	chr12	1.16E+08	1.16E+08	-	94	1	MIR620|M	0	0
NM_0324:	chrX	1.08E+08	1.08E+08	+	7286	1	FRMPD3|-	4.198	4.166
NR_12158	chr9	3668086	3691756	+	1104	1	RFX3-AS1|	0.292	0.335
NM_0010:	chr3	10026387	10101937	+	5105	1	FANCD2|F	2.349	1.747
NM_0058‹	chr2	1.52E+08	1.52E+08	-	5685	1	STAM2|Hk	4.116	4.05
NM_0052:	chrX	47635520	47650604	-	2804	1	ELK1|-|-|X	13.583	16.335
NM_0224!	chr16	58059	72631	-	2972	1	RHBDF1|C	1.631	1.212
NM_0010:	chr15	60419609	60479160	-	7046	1	ICE2|BRCC	2.187	2.745
NR_13277	chr11	32143742	32143912	-	170	1	SNORA88|	0	0
NR_13650	chr1	24415750	24472983	+	4835	1	NIPAL3|DJ	6.573	8.847
NM_0000:	chr12	1.21E+08	1.21E+08	+	1935	1	ACADS|AC	1.799	1.727
NM_0028:	chr22	41244777	41286251	-	3876	1	RANGAP1|	35.148	39.561
NR_00158	chr22	30481290	30481756	-	466	1	SDC4P|-|-	0	0
NM_1489!	chr6	655939	656964	-	1023	1	HUS1B|-|-	0.038	0.174
NR_13656	chr22	18487127	18500594	+	2915	1	FAM230A|	0	0
NM_1334:	chr5	36876759	37065819	+	9792	1	NIPBL|CDL	3.464	5.839
NM_0011‹	chrY	2935477	2982506	+	4990	1	ZFY|ZNF91	0.881	1.186
NM_1992(	chr13	52377168	52406494	-	3182	1	THSD1|TM	0.567	3.521
NM_0013(	chr2	1.57E+08	1.57E+08	-	3628	1	ERMN|JN|	0.135	0.068
NM_0010:	chr19	4502180	4517704	-	6346	1	PLIN4|KIA/	0.305	2.305
NM_0184(	chr17	31851865	31859307	-	931	1	COPRS|C1	45.051	53.626
NM_0155:	chr3	52316319	52400497	+	13030	1	DNAH1|DI	1.645	1.493
NM_0244!	chr1	1.13E+08	1.13E+08	+	2463	1	WNT2B|W	9.868	1.335
NM_0013:	chr1	1.13E+08	1.13E+08	+	3343	1	MOV10|fS	2.777	2.708
NR_07507	chr14	59919713	60063559	+	4882	1	LRRC9|-|-	0.127	0.135
NR_04024	chr13	37534940	37551536	+	3309	1	LINC00547	0.016	0
NM_0012!	chr9	1.37E+08	1.37E+08	+	1596	1	NOXA1|NY	0.881	1.768
NM_0249:	chr4	25312774	25370383	+	2782	1	ZCCHC4|H	2.776	2.447
NM_0210:	chr8	7055300	7056739	-	454	1	DEFA5|DE	0	0
NM_0182:	chr22	46050459	46054144	-	1832	1	PRR34|C2:	0.007	0.105
NM_2074:	chr8	52534037	52565461	-	1164	1	FAM150A|	0.022	0.047
NR_04000	chr2	1.77E+08	1.77E+08	-	1055	1	LINC01116	0	0
NM_0011:	chr5	57214074	57264679	+	3414	1	GPBP1|GP	18.74	20.08
NR_03029	chr4	11368827	11368921	+	94	1	MIR572|M	0	0
NM_0063:	chr16	29858356	29863288	-	1898	1	CDIPT|PIS	48.103	48.964
NM_0010(	chr1	2.48E+08	2.48E+08	+	1131	1	OR2L2|HS	0	0.048
NM_0012‹	chr6	83552886	83709408	-	4127	1	SNAP91|A	20.557	19.518
NM_0147:	chr6	43037617	43053945	-	5472	1	CUL7|3M1	22.333	17.309
NR_03829	chr6	3182818	3195767	-	1439	1	LOC10050	23.171	19.598
NR_10230	chr1	2.28E+08	2.28E+08	+	947	1	ZNF678|-|	1.225	1.88
NM_0145:	chr14	52639915	52695931	-	5494	1	ERO1A|ER	5.367	3.854
NM_0011‹	chr2	2.16E+08	2.16E+08	+	3364	1	TMEM169	32.36	26.034
NM_0013‹	chr17	36534762	36539303	+	3037	2	PIGW|Gwt	0	0
NM_0013(	chr14	75053225	75064049	-	600	1	ACYP1|AC	27.871	18.696
NM_0010!	chr9	35561830	35563899	-	1000	1	FAM166B|	1.057	0.438
NM_0010(	chr9	1.05E+08	1.05E+08	+	1039	1	OR13D1|C	0	0
NR_00278	chr1	46843095	46900475	-	1462	1	CYP4Z2P|-	0	0

NM_0010:	chr16	71729002	71809073	-	6822	1	AP1G1|AD	18.061	20.505
NR_13555	chr3	57693205	57696651	+	2972	1	LOC10192!	0	0.037
NR_04644	chr14_GL(	53589	115073	-	832	1	MAFIP|MI	0	0
NM_0012{	chr13	69700593	70108493	-	3955	1	KLHL1|MR	3.596	5.884
NR_02450	chr13	40450934	40481006	-	2081	1	LINC0059{	0.006	0
NM_0807‹	chr20	45888472	45891287	-	1436	1	NEURL2|C	1.742	1.955
NM_0251:	chr1	2.08E+08	2.08E+08	-	11409	1	PLXNA2|O	27.851	30.491
NR_12648	chr15	70635249	70637314	+	2065	1	SALRNA2|:	0	0
NR_03396	chr5	28926870	28927313	+	443	1	LSP1P3|-|-	0.349	0.618
NM_0010:	chr12	1.22E+08	1.22E+08	-	899	1	IL31|IL-31	0	0.015
NM_0334(	chr14	23520855	23551649	-	9170	1	ZFHX2|ZFH	13.45	6.929
NM_0010:	chr9	34621458	34628014	-	1231	1	ARID3C|-|	0.45	0.59
NM_0010(	chr14	20060045	20060983	+	937	1	OR4L1|OR	0	0
NR_12207	chr16	24661422	24671062	-	2904	1	LINC0156:	0.049	0.047
NR_13847	chr12	56416373	56449465	-	5117	1	TIMELESS|	11.561	10.557
NM_0019:	chr16	2239872	2251601	-	1042	1	ECI1|DCI|-	26.863	15.971
NM_0010(	chr22	49994513	50012626	-	3618	1	IL17REL|-|	0.043	0.004
NR_00293	chr19	53599631	53603497	-	1221	1	LOC28437!	0	0
NR_00230	chrX	72044409	72044961	+	552	1	RPS26P11	0.047	0.099
NM_0012{	chr6	93240021	93419582	-	6609	1	EPHA7|EH	7.712	4.899
NM_0010{	chr14	1.04E+08	1.04E+08	+	1954	1	ASPG|C14	0	0.042
NM_0530!	chr1	1.52E+08	1.52E+08	-	5163	1	THEM4|CT	1.523	1.613
NM_0529:	chr6	28571630	28587335	-	4871	2	ZBED9|Bu!	0.064	0.059
NM_0011‹	chr4	79977508	80073323	-	2286	1	ANTXR2|C	4.089	3.271
NM_1392:	chr4	56647988	56681706	-	1413	1	HOPX|CAN	0.283	2.239
NM_0148:	chr9	1.35E+08	1.35E+08	+	4926	1	PPP1R26|I	14.134	20.128
NM_1981{	chr3	1.91E+08	1.91E+08	+	3223	1	OSTN|MU	0.08	0.008
NM_0003!	chr22	30607083	30627060	+	2061	1	TCN2|D22	17.066	8.885
NM_0203:	chr22	37658730	37666932	+	2001	1	PDXP|CIN	64.964	82.942
NM_0011(	chr2	27099353	27119127	-	1115	1	CGREF1|C	6.094	11.13
NM_0011:	chr10	71396934	71707507	+	3927	1	CDH23|CD	2.2	1.817
NM_0220(	chr3	1.2E+08	1.2E+08	+	2732	1	NR1I2|BXF	0.019	0.01
NM_0010:	chr6	49499958	49528064	+	2122	1	GLYATL3|(	0	0.006
NM_0011:	chrX	70423031	70433391	+	2052	1	GDPD2|GI	5.033	3.838
NR_10706	chr16	17052	17119	-	67	4	MIR6859-:	0.385	0.204
NR_07357	chr17	42667915	42677030	-	3023	1	PLEKHH3|-	18.745	17.471
NM_2015!	chr6	1.09E+08	1.09E+08	+	7308	1	FOXO3|AF	3.513	5.505
NM_0529!	chrX	71578024	71613583	+	3163	1	GCNA|ACF	1.044	1.516
NM_0013:	chr2	1.27E+08	1.27E+08	-	1868	1	BIN1|AMP	59.732	43.731
NR_04538	chr12	1.22E+08	1.22E+08	+	893	1	CLIP1-AS1	0.144	0
NR_13495	chr6_GL0(	3527442	3537006	-	5757.9	7	NOTCH4|I	0	0
NM_1387!	chr14	1.05E+08	1.05E+08	+	1955	1	PLD4|C14(	0.053	0.014
NR_12063	chr10	10058722	10063502	+	571	1	LOC10192:	0.113	0
NM_0011‹	chr10	80540819	80646560	+	3478	1	SH2D4B|-|	0.159	0.488
NR_12581	chr8	29748309	29798492	+	2329	1	LINC0209!	0.028	0.006
NM_0010:	chrX	1.08E+08	1.08E+08	-	1742	1	TSC22D3|I	39.916	50.652
NM_1524(	chr5	75674199	75712455	-	1947	1	POC5|C5o	6	6.725
NM_1816!	chr18	59295404	59318649	-	1959	1	CPLX4|CP)	0.007	0.461
NR_04652	chr13	99690098	99690971	-	676	1	CLYBL-AS2	0.038	0
NM_0067(	chr3	50287732	50292595	-	2095	1	IFRD2|IFN	7.305	5.753

NR_04749	chr1	47179250	47180339	+	659	1	LINC00853	0.078	0.062
NM_0176:	chr10	72332830	72355149	-	3187	1	DNAJB12|	13.1	13.869
NM_0011!	chr12	9669708	9699555	+	5167	1	CLEC2D|CI	0.434	0.591
NM_1449:	chr2	1.09E+08	1.09E+08	+	2392	1	CCDC138|	0.943	1.42
NM_0181:	chr1	1.51E+08	1.51E+08	-	3164	1	GOLPH3L|	5.02	6.753
NR_12541	chr8	1.11E+08	1.11E+08	-	555	1	LINC01608	0	0
NM_0042!	chr11	66332071	66336529	-	2672	1	RIN1|-|-|1	1.366	1.153
NR_03899	chr15	79191707	79283945	-	691	1	ANKRD34C	0	0.099
NM_0324!	chrX	1.2E+08	1.2E+08	-	1303	2	RHOXF2|C	0	0
NM_0010(	chr1	1.04E+08	1.04E+08	+	1825	3	AMY1B|AI	0	0
NR_12587	chr4	47914227	47990737	+	1663	1	LOC10192	0.047	0.107
NR_03659	chr1	1.1E+08	1.1E+08	-	2606	1	LOC44060	0.124	0.074
NM_0179:	chr6	78934419	79078298	-	11928	1	PHIP|BRW	2.448	6.35
NM_0025!	chr17	3277890	3278974	-	1082	1	OR3A2|OL	0	0
NR_03031	chr6	1.26E+08	1.26E+08	+	82	1	MIR588|M	0	0
NM_0012:	chr3	39096599	39107900	-	2846	1	GORASP1|	21.103	21.291
NM_0012!	chr19	54738511	54848566	+	1213.8	39	KIR2DS2|1	0	0
NM_0012!	chr5	60751789	60844274	-	3824	1	ELOVL7|-|	0.047	0.115
NR_00334	chr15	25227109	25227190	+	81	1	SNORD115	0	0
NM_1736:	chr16	22092538	22156966	+	4568	1	VWA3A|-|	0.206	0.315
NR_02680	chr15	68803821	68807101	-	3280	1	ANP32A-IT	0.106	0.209
NR_10805	chr10	8259331	8268305	-	911	1	LINC00708	0	0
NM_0010:	chr9	1.37E+08	1.37E+08	+	1700	1	CCDC183|	4.452	4.149
NM_0189(	chr5	1.41E+08	1.41E+08	+	5359	1	PCDHA4|C	24.353	20.031
NR_00283	chr8	1.22E+08	1.22E+08	+	2283	1	HAS2-AS1|	0.186	0.174
NM_0011!	chr2	1.6E+08	1.6E+08	-	3798	1	CD302|BII	0.268	0.4
NM_0011:	chr19	10984152	11062282	+	5282	1	SMARCA4	181.283	158.517
NM_0160!	chr8	1.01E+08	1.01E+08	-	2739	1	ZNF706|H:	10.187	10.347
NM_0211:	chr11	839721	842529	-	872	1	POLR2L|RI	56.793	29.436
NM_0181:	chr6	49463383	49493107	+	1717	1	CENPQ|C6	0.773	1.276
NM_0013(	chr14	92923100	92935296	+	1583	2	CHGA|CG/	0	0
NM_0011:	chr7	69598919	70793068	+	6495	1	AUTS2|FBI	46.228	63.264
NM_0012!	chr12	1.11E+08	1.11E+08	+	4474	1	SH2B3|IDI	3.078	4.629
NM_0004	chr7	95298357	95324572	-	1741	1	PON1|ESA	0.059	0.079
NM_0145:	chr19	54137720	54155708	+	2867	10	CNOT3|LE	0.971	0.784
NM_0010(	chr1	2.48E+08	2.48E+08	+	937	1	OR2T8|OR	0	0
NR_02687	chr1	47432135	47434641	-	2506	1	FOXD2-AS:	0.031	0.022
NM_0013:	chr4	1.1E+08	1.1E+08	-	2172	1	CFI|AHUS:	0.125	1.337
NR_13464	chr18	75696083	75712403	-	1915	1	LINC01898	0	0
NM_0806:	chr17	58083419	58090257	+	1517	1	DYNLL2|D	138.063	167.542
NM_0010:	chr14	73537224	73543794	+	1598	1	ACOT1|AC	0.178	0.18
NR_03620	chr19	42133445	42133513	-	68	1	MIR4323|	0	0
NM_0055!	chr17	41609779	41612827	-	1707	1	KRT16|CK:	0	0
NM_0011:	chr22	19479826	19520612	+	1820	1	CDC45|CD	4.718	1.874
NM_0218(	chr7	1.33E+08	1.34E+08	+	4172	1	EXOC4|SE	39.002	51.091
NM_0012!	chr12	68610839	68660606	+	2009	1	RAP1B|K-F	4.493	5.57
NM_0050:	chr19	14950033	14972918	-	1854	1	SLC1A6|E/	1.447	5.098
NM_0012!	chr17	39757715	39864188	-	8998	1	IKZF3|AIO	0.006	0.003
NR_02769	chr4	16226663	16258187	+	2493	1	TAPT1-AS1	4.862	2.956
NM_0013:	chr19	41860446	41871416	+	518	1	RPS19|DB.	1026.002	805.585

NR_13639	chr17	67244776	67262080	+	846	1	LOC10192	0.152	0.081
NM_1818(	chr4	2071958	2242164	-	3228	1	POLN|POL	0.479	0.569
NM_0011(	chr10	1.23E+08	1.23E+08	+	1071	1	HMX3|NK	0.024	0.064
NM_0010	chr19	39125780	39179406	+	2296	1	PAK4|-|-|	18.728	16.268
NR_10345	chr6	28121795	28137316	-	2183	1	ZSCAN16-/	5.056	4.26
NM_1446	chr1	1.6E+08	1.6E+08	+	3823	1	ATP1A4|A	0.007	0.011
NM_0184	chr11	64555601	64572875	+	3873	1	SLC22A11|	0.023	0
NM_0039	chr1	85265776	85276904	-	3100	1	BCL10|CAI	3.814	3.866
NM_0012	chrX	1.12E+08	1.12E+08	+	3971	1	ALG13|CD	5.059	6.129
NM_0164(	chr5	1.46E+08	1.46E+08	-	4791	1	LARS|HSP(	24.6	26.985
NM_0201	chr3	1.39E+08	1.39E+08	+	1112	1	MRPS22|C	26.715	26.014
NM_0013	chr14	75081379	75127080	-	6058	1	NEK9|APU	12.183	7.999
NM_0013(	chr13	1.13E+08	1.13E+08	-	9401	1	LOC10192	0.306	0.134
NM_1730	chr15	42404779	42412317	+	1262	1	CAPN3|CA	1.206	1.595
NM_0155	chr15	69160597	69272205	+	5073	1	GLCE|HSEI	3.457	10.735
NR_02667	chr12	1.31E+08	1.31E+08	+	2468	1	LINC01257	0.005	0.006
NR_03402	chr13	74552843	74557120	+	2058	1	LINC00347	0.013	0.007
NM_0012	chr10	27504174	27542237	+	4928	1	RAB18|RA	13.456	12.719
NM_0191	chr19	1040103	1065571	+	6769	1	ABCA7|AB	7.85	8.056
NM_0154	chr8	1.03E+08	1.03E+08	+	2628	1	DCAF13|G	10.19	11.023
NM_0047	chr19	19623656	19628230	-	1791	1	LPAR2|ED	31.733	24.595
NM_0013	chr12	1.11E+08	1.12E+08	-	3721	1	ATXN2|AT	14.494	13.458
NR_03648	chr7	1.42E+08	1.42E+08	-	2051	2	TRY2P|-|-	0	0
NM_0059	chr1	37809567	37859620	-	7959	1	MTF1|MTI	2.592	2.757
NM_0012	chr19	47340487	47342015	+	1353	1	C5AR2|C5	0	0.01
NM_0528	chr17	50693190	50707909	-	4162	1	ANKRD40|	9.755	10.12
NM_0241(	chr19	6440064	6459770	-	3448	1	SLC25A23|	29.059	32.471
NM_0013(	chr4	56978640	57031162	+	3868	1	POLR2B|P	51.824	72.791
NM_0010(	chr4	1.86E+08	1.86E+08	+	2390	1	FAM149A|	6.452	13.943
NM_0219	chr13	48233138	48262096	+	1888	1	ITM2B|AB	54.586	74.797
NM_0009(	chr20	49503874	49568170	-	5591	1	PTGIS|CYP	2.562	2.906
NM_0011	chr16	77199452	77213079	+	864	1	SYCE1L|M	4.283	2.98
NM_0010	chr17	76467548	76470117	+	1020	1	AANAT|DS	0.948	0
NR_00294	chr3	1.31E+08	1.31E+08	+	2258	1	NUDT16P1	0	0.018
NR_13661	chr4	1.53E+08	1.53E+08	-	1122	1	LOC72987	0.563	0.317
NM_0204	chr16	67842310	67844195	+	1883	1	THAP11|C	28.067	30.311
NM_0012	chr6	41683978	41734401	-	2152	1	TFEB|ALPH	1.342	2.877
NM_0012	chr14	35122552	35274078	+	1581	1	KIAA0391|	10.887	12.475
NM_0178(	chr8	73976142	73982783	+	2080	1	TMEM70|	8.784	9.357
NR_03719	chr2	1.08E+08	1.08E+08	+	1870	1	SULT1C2P	0	0
NM_0011(	chr6_GL0C	4715069	4725463	-	2184	5	WDR46|BI	0.012	0
NR_02706	chr6	1.14E+08	1.14E+08	+	2002	1	FLJ34503|	0.013	0.034
NM_0045(	chr11	65787034	65797219	+	3016	1	OVOL1|HC	0.077	0.041
NM_0010	chr1	2.35E+08	2.35E+08	+	2738	1	GGPS1|GC	12.244	12.842
NM_0029(	chr6_GL0C	3208062	3215057	-	1548	6	NELFE|D6	0	0
NM_1536	chr12	6693791	6700843	-	2359	1	PIANP|C1	40.162	41.19
NM_0220(	chr18	10670247	11148762	-	9659	1	PIEZO2|C1	2.687	1.032
NM_0010	chr19	708953	748330	+	2732	1	PALM|-|-|	193.058	162.269
NR_12578	chr13	62212282	62249951	+	665	1	LINC01075	0	0
NM_0028	chrX	1.08E+08	1.08E+08	-	1567	1	PSMD10|c	30.436	37.14

NM_0011	chrX	1.56E+08	1.56E+08	+	2527	2	VAMP7|SY	0	0
NR_13298	chr8	95066808	95072920	-	2630	1	LOC10537	0.005	0.01
NM_1383	chr18	21650897	21704805	-	2053	1	ABHD3|LA	2.223	1.881
NM_0010	chr22	23834503	23839012	-	3090	2	DERL3|C2	0	0
NM_0013	chr12	69359710	69390870	+	1332	1	YEATS4|4	8.296	8.545
NM_0175	chr7	6615896	6624290	+	3573	1	ZNF853|-|	14.416	19.34
NM_0011	chr11	34105564	34146911	+	3797	1	NAT10|AL	13.454	15.851
NM_0120	chr1	39084167	39487138	+	17594	1	MACF1|AE	22.793	28.552
NR_13297	chr10	7186680	7186784	-	104	1	SNORD12	0	0
NM_1994	chrX	1.04E+08	1.04E+08	+	2943	1	PLP1|GPM	4.197	14.595
NM_0012	chr12	7812512	7891196	-	3469	1	SLC2A14|	0.476	0.347
NR_13563	chr17	82290046	82292814	-	1808	1	LINC0197	0.157	0.129
NM_0010	chr2	87755960	87985790	+	6668.5	2	RGPD1|RG	0.029	0.107
NR_07302	chr20	46499630	46513559	-	3860	1	ZNF334|-|	2.472	2.569
NR_04980	chr6	33438331	33438437	+	106	2	MIR5004|	0	0
NM_0013	chr1	2.07E+08	2.07E+08	+	1739	1	CD55|CR|	1.275	0.323
NR_11084	chr17	78360448	78364931	+	892	1	LOC10192	0.347	0.399
NM_0010	chr11	56122373	56123311	+	937	1	OR8H3|OF	0	0
NM_1768	chr19	55947832	55988629	+	3922	1	NLRP8|CLI	0	0
NM_0011	chr16	2205731	2209417	+	1710	1	MLST8|GB	41.953	37.695
NR_10993	chr5	8839732	8881524	+	777	1	LOC10192	0.116	0.035
NR_12539	chrX	97528364	97564535	-	974	1	DIAPH2-AS	0	0
NM_1988	chr2	85657317	85668741	-	2826	1	SFTPB|PSF	0.018	0
NM_0042	chr5	1.57E+08	1.57E+08	-	1096	1	MED7|AR	6.282	7.248
NM_0025	chr6	1.42E+08	1.42E+08	-	2473	1	NMBR|BB	0.031	0.138
NM_1749	chr19	7677057	7679833	+	1299	1	MCEMP1|	0.03	0.032
NR_02940	chr11	1309708	1310707	+	999	1	TOLLIP-AS	1.381	1.92
NM_0073	chr16	29790713	29805385	+	2104	1	KIF22|A-3	26.406	20.858
NM_0013	chr17	45023338	45061129	-	1953	1	DCAKD|-|	42.186	41.905
NM_0011	chr2	2.19E+08	2.19E+08	-	3529	1	PTPRN|IA-	33.956	34.749
NR_03621	chr1	2.1E+08	2.1E+08	-	66	1	MIR4260|	0	0
NM_0012	chr4	1.06E+08	1.06E+08	+	5440	1	ARHGEF38	0.055	0.121
NM_0013	chr1	16538724	16540035	+	1309	1	FAM231B|	0	0
NM_0147	chr9	98084353	98119353	-	4603	1	TRIM14|-|	0.109	0.155
NM_0041	chr19	49513179	49526428	+	1621	1	FCGRT|FC	3.444	3.819
NM_1785	chr1	35573370	35595326	+	2178	1	TFAP2E|AI	2.332	3.434
NR_03791	chr10	1.23E+08	1.23E+08	-	2612	1	FAM24B-C	0.39	0.241
NM_0011	chr3	1.97E+08	1.97E+08	+	5728	1	FBXO45|F	5.249	5.538
NR_00349	chr15	25213659	25213729	+	70	1	SNORD115	0	0
NR_13508	chr12	6875765	6889358	-	399	1	LOC10536	1.196	2.094
NR_04985	chr14	59646962	59647020	-	58	1	MIR5586|	0	0
NM_1985	chr6	87282979	87329278	-	2275	1	GJB7|CX2	0.057	0.096
NR_03400	chr3	1.57E+08	1.57E+08	+	788	1	LINC00881	0	0.017
NM_0019	chr8	6935820	6938338	-	614	1	DEFA4|DE	0	0
NM_2136	chr5	1.77E+08	1.77E+08	-	999	1	PDLIM7|LI	45.585	32.44
NM_0013	chr9	1.13E+08	1.13E+08	-	4026	1	INIP|C9orf	7.936	8.216
NM_0143	chr10	84139429	84153555	+	2438	1	GHITM|DE	65.25	62.71
NR_02942	chr5_GL33	1069427	1288202	+	1933.2	6	SMA4|SM	0.32	0.184
NR_12160	chr9	25780056	25812968	+	1231	1	LINC01241	0	0
NR_03039	chrX	50013241	50013337	+	96	1	MIR660|M	0	0

NM_0010	chr11	66337333	66345111	-	1343	1	BRMS1|-|	33.045	31.535
NM_0011	chr19	55642097	55643470	+	1022	1	ZNF580|-|	49.492	46.867
NM_0050	chr16	23678772	23690367	+	2265	1	PLK1|PLK|	8.789	7.275
NM_1392	chr9	97600080	97633680	-	4416	1	TSTD2|C9	15.007	20.077
NM_0011	chr19	56810077	56825006	-	8707	1	PEG3|PW	0.025	0.024
NR_10689	chr7	1E+08	1E+08	+	70	1	MIR6840|	1.105	1.761
NM_0030	chr6	1.09E+08	1.09E+08	+	1668	1	SMPD2|IS	3.911	3.588
NR_05197	chr20	50040707	50041629	-	594	1	TRERNA1|	0	0
NM_0013	chr2	2.19E+08	2.19E+08	-	1560	1	DNPEP|AS	10.885	12.494
NR_00394	chr11	6713146	6721879	-	8733	1	GVINP1|G	0.022	0.017
NR_04681	chr5	1.43E+08	1.43E+08	+	386	1	ARHGAP2	0	0
NM_0319	chr22	30291990	30326966	-	1986	1	TBC1D10A	8.641	11.407
NM_0224	chr2	2.19E+08	2.19E+08	-	1549	1	RNF25|AO	29.741	29.728
NM_0010	chr17	58982639	59106921	-	3607	1	TRIM37|M	15.349	19.237
NM_0010	chr6	1.28E+08	1.28E+08	-	3858	2	THEMIS|C	0	0
NM_0198	chr8	16992825	17002165	-	1011	1	FGF20|FG	0	0
NM_0335	chr2	1.1E+08	1.1E+08	+	1211	2	LIMS3|PIN	0	0
NR_04548	chr2	1.36E+08	1.36E+08	+	1860	1	LOC10050	0.042	0.007
NR_03410	chr20	44656451	44671739	-	1152	1	LINC0126	0.604	2.414
NM_0011	chr11	72576140	72642460	-	4101	1	PDE2A|CG	2.738	20.186
NM_1755	chr20	62302896	62308862	+	1464	1	ADRM1|A	77.792	73.331
NM_1984	chr5	50396197	50441400	-	4298	1	EMB|GP7	0.324	0.099
NM_0012	chr14	1.02E+08	1.02E+08	+	3702	1	ZNF839|C	6.673	7.022
NM_1828	chr14	46839625	47343246	-	5229	1	MDGA2|M	0.976	0.574
NR_10350	chr19_KI2	576155	584960	+	3081	4	LILRA1|CD	0	0
NM_0064	chr13	24420931	24512810	-	5433	1	PARP4|AD	2.143	2.168
NM_1784	chr1	1.53E+08	1.53E+08	+	815	1	LCE5A|LEF	0	0
NM_0142	chr22	32218476	32255331	-	2003	1	SLC5A4|DJ	0	0.068
NM_0013	chr2	28981298	29052230	+	2957	1	FAM179A|	0.122	0.199
NM_0329	chr11	1.19E+08	1.19E+08	+	2877	1	CXCR5|BL	0.134	0.348
NM_0010	chr9	1.14E+08	1.15E+08	-	2875	1	WHRN|CIF	10.064	17.909
NR_04043	chr8	66419587	66428977	-	4365	1	RRS1-AS1|	0	0
NR_02985	chrX	50008964	50009027	+	63	1	MIR362|M	0	0
NM_0144	chr11	7088934	7091148	+	2212	1	RBMXL2|H	0.029	0.012
NM_1526	chr19	12461184	12484818	-	4893	1	ZNF709|-|	1.447	1.724
NM_0026	chr20	45174899	45176544	+	550	1	PI3|ESI|SK	0.094	0
NM_0011	chr17	38428476	38512392	+	5880	2	ARHGAP2	0.621	0.857
NM_2070	chr15	57219457	57288516	+	3960	1	TCF12|CR	46.182	38.594
NM_0013	chr16	55479998	55506691	+	3122	1	MMP2|CL	6.166	9.02
NR_00301	chr4	52713249	52713370	+	121	1	SNORA26|	1.385	3.056
NR_10413	chr6	1.09E+08	1.09E+08	-	714	1	ARMC2-AS	0	0
NR_04651	chr13	1.11E+08	1.11E+08	-	416	1	ARHGEF7-	0.31	0.527
NM_0011	chr19	13206443	13506460	-	8577	1	CACNA1A|	0.818	3.667
NR_02948	chr9	94176347	94176433	+	86	1	MIRLET7F	0	0
NM_0013	chr4	17486393	17512234	-	1552	1	QDPR|DH	23.777	34.446
NM_0010	chrX	23703660	23743290	-	1683	1	ACOT9|AC	6.864	7.137
NR_00437	chrX	1.1E+08	1.1E+08	-	71	1	SNORD96	0	0
NR_03835	chr10	73796514	73801399	-	1456	1	ZSWIM8-A	28.417	32.559
NM_0010	chrX	1.53E+08	1.53E+08	+	6313	1	ZNF275|-|	6.656	8.08
NR_12062	chr10	1.04E+08	1.04E+08	+	697	1	LOC10192	0.111	0.039

NR_00326	chr3	1.96E+08	1.96E+08	+	2289.4	8	SDHAP2|S	0	0
NM_0124!	chr19	2425624	2427916	-	1990	1	TIMM13|T	23.973	20.958
NM_1450!	chr12	1.23E+08	1.23E+08	-	1795	1	RILPL2|RLI	3.082	2.602
NM_1722	chr17	69244435	69327182	-	8136	1	ABCA5|AB	0.295	0.296
NR_12597	chr1	1.16E+08	1.16E+08	-	573	1	LOC10192	0.27	0.526
NM_1722!	chr19	3762667	3767565	+	618	1	MRPL54|L	40.349	28.879
NM_0010	chr9	39355669	39361962	+	4253	1	SPATA31A	0	0
NR_13563	chrX	17528435	17587160	+	1552	1	LOC10192	0	0
NM_0038!	chr17	76074801	76077541	+	1353	1	GALR2|GA	0.172	0.243
NM_0011	chr21	30289145	30289514	-	367	1	KRTAP25-1	0	0
NM_0013	chr13	42886388	42992312	-	3106	1	EPSTI1|BR	0.008	0
NM_0041	chr14	54474491	54489026	-	4116	1	GMFB|GM	8.721	11.87
NM_0011!	chr17	59859480	59892945	-	2317	1	TUBD1|TU	1.614	1.945
NM_0012(	chr20	6768098	6780278	+	3160	1	BMP2|BD/	0.739	0.598
NR_10987	chr5	1.53E+08	1.53E+08	-	2172	1	LINC0147C	0	0.202
NM_0011(	chr17	6651739	6653298	+	1557	1	C17orf100	11.544	16.319
NM_0190!	chr2	1.78E+08	1.79E+08	+	2459	1	PLEKHA3|I	3.576	4.033
NM_0013	chr1	33013044	33036911	-	838	1	AK2|ADK2	12.832	18.699
NR_12052	chr7	29199040	29208970	-	1295	1	LOC10272	0	0
NR_00355	chr7	90119400	90125600	+	2015	1	DPY19L2P	0.019	0.197
NR_12637	chr13	75876886	75881125	+	315	1	LMO7DN-I	0	0
NM_1982	chr20	16729964	16741772	+	1572	1	SNRPB2|M	36.13	35.079
NM_0182!	chr4	37826660	37862938	+	3222	1	PGM2|MS	1.945	2.053
NR_11087	chr6	40878886	41023280	-	2237	1	LOC10192	0.207	0.435
NM_0011	chr12	1.04E+08	1.05E+08	+	5748	1	CHST11|C	5.852	16.649
NM_0012	chr17	6684713	6713421	-	3224	1	SLC13A5|E	0.136	0.251
NM_0037(	chr1	20806292	21111383	-	6007	1	EIF4G3|eI	22.293	20.118
NR_10691	chrX	1.54E+08	1.54E+08	+	66	1	MIR6858|I	1.563	0.623
NR_11008	chr7	84532476	84584318	+	532	1	LOC10192	0	0
NM_0011	chr19	55385932	55392083	+	889	1	RPL28|L28	270.72	195.04
NM_0012!	chr4	9224896	9226488	+	1591	1	USP17L13	0	0
NM_0021(	chr11	1.19E+08	1.19E+08	-	1590	1	H2AFX|H2	46.092	37.654
NM_1734!	chrX	1.07E+08	1.07E+08	+	1643	1	PIH1D3|C	0.047	0.008
NR_03785	chr1	1.68E+08	1.68E+08	-	2438	1	LOC10050	0	0.006
NM_0012	chr19	14081632	14085795	-	1439	1	C19orf67|	0.108	0.057
NM_0159	chr2	63121400	63588733	-	3289	1	WDPCP|BI	1.509	1.433
NM_0013(	chrX	35919734	36385319	+	9877	1	CFAP47|CI	0.023	0.011
NM_1818	chr15	84601018	84623716	+	3742	1	ZSCAN2|ZI	20.736	22.76
NR_13576	chr1	16533891	16535652	-	1544	1	LINC01783	0.301	0.328
NR_10672	chr1	43838622	43838683	+	61	1	MIR6079|I	0	0
NM_1537	chr4	5711197	5814304	+	6407	1	EVC|DWF-	3.115	3.121
NM_1391	chr11	43880807	43920275	+	1557	1	ALKBH3|A	15.775	15.967
NM_0011(	chr10	1.22E+08	1.22E+08	-	1389	1	NSMCE4A	19.447	18.835
NR_03836	chr10	1.32E+08	1.32E+08	-	3379	1	LINC01164	0.015	0.008
NR_10676	chr21	39447010	39447069	+	59	1	MIR6508|I	0	0
NM_0071(	chr6	35468401	35470781	+	711	1	RPL10A|C	265.504	230.441
NM_1522	chr1	18839599	18859661	-	2513	1	TAS1R2|G	0.005	0
NM_0072	chr17	49575936	49583809	+	5548	1	NXPH3|NF	1.222	2.93
NM_0010(	chr9	25676389	25678858	-	2467	1	TUSC1|TS(	1.719	2.621
NR_13459	chr6	1.61E+08	1.61E+08	+	691	1	LOC10537	0	0

NR_02727	chr4	9367874	9369070	+	1196	1	USP17L6P	0	0
NM_0011!	chr11	1.2E+08	1.2E+08	+	9730	1	ARHGEF12	18.782	21.61
NR_03172	chr12	1E+08	1E+08	+	65	1	MIR1827|I	0	0
NR_13487	chr18	23296015	23437969	-	2937	1	TMEM241	7.612	9.462
NM_1784:	chr1	1.53E+08	1.53E+08	+	589	1	LCE2C|LEP	0	0
NM_2136:	chr8	53715543	53843311	-	2117	1	ATP6V1H|	38.345	47.309
NM_0146{	chr8	52110832	52409879	-	6302	1	ST18|NZF3	11.439	5.749
NM_1781!	chrX	1.11E+08	1.11E+08	-	9099	1	DCX|DBCN	107.389	132.307
NM_0011:	chr14	20556093	20560931	-	1131	1	RNASE9|H	0	0
NR_03967	chr16	50878773	51409868	+	83.5	2	MIR548AI|	0	0
NM_0011‹	chr22	25027974	25197448	+	10481	1	KIAA1671|	1.823	2.047
NM_0011‹	chr15	67521184	67527303	+	1293	1	C15orf61|	2.802	5.138
NM_0011:	chrX	72578814	72714181	-	5913	1	PHKA1|PH	0.329	0.769
NM_0219(	chr11	12674422	12944737	+	9401	1	TEAD1|AA	5.109	7.684
NM_0010(	chr12	6328757	6342117	-	2214	1	TNFRSF1A	21.064	19.89
NR_04977	chr13	92701390	92719974	-	374	1	GPC5-AS1|	2.275	0.146
NM_0065(	chr19	12884423	12887203	-	1609	1	KLF1|EKLF	0.272	0.443
NM_0045‹	chr11	78068347	78080219	-	2470	1	NDUFC2|E	43.352	49.315
NM_1827(	chr3	4361145	4467282	-	2150	1	SUMF1|AA	2.765	3.275
NM_0203!	chr11	89797655	89808575	-	2153	1	TRIM49|R	0	0
NM_0184:	chr5_KI27(	985599	1097797	+	11034	3	BDP1|HSA	0.002	0.002
NM_0033!	chr8	48008435	48061894	+	1518	1	UBE2V2|D	23.57	22.819
NR_10701	chr19	55123225	55123292	-	67	1	MIR7975|I	0	0
NM_0029:	chr13	1.14E+08	1.14E+08	+	1505.5	2	GRK1|GPR	0.043	0.009
NR_13120	chr19	50830530	50851143	+	460	1	LOC10537:	0	0
NM_0000‹	chr17	66212029	66229438	-	1191	1	APOH|B2G	0.238	0.012
NM_0041!	chrY	1403139	1452977	-	2118	2	ASMTL|AS	0	0
NR_03840	chr5	1.76E+08	1.76E+08	+	960	1	LOC10050:	0	0
NR_12632	chr3	1.86E+08	1.86E+08	+	1523	1	IGF2BP2-A	0	0.009
NM_0212!	chr14	56118375	56301313	+	5688	1	PELI2|-|-|	3.786	6.887
NM_0182:	chr1	2.29E+08	2.3E+08	-	4142	1	NUP133|h	18.011	16.901
NR_10405	chr13	20938564	20949428	-	745	1	LINC00367	0	0
NM_0025{	chr2	79157006	79159754	-	801	1	REG3A|HII	0	0
NM_0010:	chr13	29024611	29505947	+	6923	1	MTUS2|CA	3.047	0.88
NM_1452!	chr14	52730165	52774987	+	4850	1	STYX|-|-|1	1.265	1.149
NM_0058(	chr3	1.36E+08	1.37E+08	-	5130	1	STAG1|SA	6.61	6.969
NM_1824{	chr7	1.35E+08	1.35E+08	+	983	1	STRA8|-|-	0	0
NR_03076	chr10	1.04E+08	1.04E+08	-	97	1	MIR936|M	0	0
NR_12644	chr1	2.32E+08	2.32E+08	+	730	1	DISC1-IT1|	0.389	0.319
NR_02687	chr19	39638685	39642401	+	1827	1	LOC10012!	0	0
NM_0190‹	chr10	26438337	26567803	+	2620	1	APBB1IP|I	0	0
NM_0003(	chr20	23045633	23049664	-	4029	1	THBD|AHU	0.08	0
NM_1457:	chr4	89112817	89114901	+	2082	1	TIGD2|HEI	2.322	2.27
NR_02337	chr1	2.29E+08	2.29E+08	-	120	10	RNA5S11|	0	0
NR_02666	chr7_KI27(	102335	103468	-	1133	2	RPL19P12	0	0
NM_0041:	chr17	48958556	48968593	-	703	1	GIP|-|-|17	0.055	0.039
NR_12066	chr10	85431943	85432772	-	658	1	LOC10192!	0	0
NM_1334!	chr19	10316213	10333638	-	3577	1	RAVER1|-|	12.778	11.997
NM_0010(	chr17	40388521	40417950	-	5716	1	TOP2A|TO	5.247	12.525
NR_03839	chr22	17256860	17266733	-	1911	1	CECR3|-|-	0	0.014

NM_0011:	chr1	21217247	21290273	-	5067	1	ECE1|ECE|	16.219	19.852
NM_0319(	chrX	1.33E+08	1.33E+08	-	2791	1	USP26|-|-	0	0
NM_0010;	chr5	1.19E+08	1.19E+08	+	2001	1	TNFAIP8|(	0.348	0.287
NM_0013:	chr4	1.03E+08	1.03E+08	+	3866	1	NFKB1|CV	2.174	2.625
NM_0010:	chr1	53894187	53911086	+	1712	1	DIO1|5DI|	0.008	0.024
NM_0040!	chr9	1.21E+08	1.21E+08	-	3189	1	STOM|BNI	1.338	0.919
NM_1815:	chr20	45551152	45579326	-	1260	1	WFDC8|C2	0	0.022
NM_1522:	chr10	58191517	58267934	-	6125	1	IPMK|-|-|:	1.175	0.982
NM_0179!	chr9	74946583	74952886	-	2321	1	C9orf40|-|	5.505	5.105
NR_11038	chrX	43176994	43226598	+	2222	1	LOC10192	0	0
NR_10987	chr20	21610787	21703585	-	1248	1	LINC01726	0	0
NR_04656	chr21	29372501	29373980	+	800	1	BACH1-IT2	0.064	0.051
NR_02966	chr9	95085208	95085304	+	96	1	MIR23B|M	0.537	0
NM_0055{	chr19	44809059	44821421	+	2436	1	BCAM|AU	5.351	4.301
NR_03376	chr4	1115197	1123164	-	529	1	TMED11P|	0	0.207
NR_10409	chr1	1.69E+08	1.69E+08	-	1124	1	LINC0097C	0	0.024
NR_13276	chr1	1.56E+08	1.56E+08	-	147	1	SCARNA26	0	0
NM_0012<	chr9	1.12E+08	1.12E+08	-	4331	1	PTBP3|RO	5.254	4.791
NM_0069(	chr16	71474073	71489351	-	2735	1	ZNF19|KO	3.875	4.227
NR_13190	chr11	1.05E+08	1.05E+08	-	2190	1	CASP1P2|-	0	0
NM_0204:	chr20	44111697	44187578	-	4777	1	JPH2|CMH	0.019	0.052
NM_0218;	chr4	1.55E+08	1.55E+08	-	1750	1	FGG|-|-|4	0	0
NM_0012(	chrX	53422856	53431120	+	1273	1	RIBC1|261	2.411	0.936
NM_0070!	chr10	77975149	78029540	-	6602	1	POLR3A|A	21.223	24.572
NM_0052:	chr12	96194382	96269835	+	4198	1	ELK3|ERP|	0.829	1.093
NM_0012(	chr8	7414863	7416832	-	294	2	DEFB4B|D	0	0
NR_10463	chr5	42155833	42175345	-	1905	1	LOC10192	0	0
NM_0010(	chr7	1.49E+08	1.49E+08	-	3212	1	ZNF425|-|	5.905	7.104
NM_0011:	chr2	1.58E+08	1.58E+08	+	2398	1	UPP2|UDF	0.113	0.086
NM_0221:	chr9	84275850	84368498	-	4515	1	SLC28A3|(	0.003	0.012
NM_0247:	chr15	34140674	34210096	-	2728	1	KATNBL1|	3.091	3.821
NR_12044	chr12	49292631	49324576	-	4158	1	LOC10192	45.521	3.614
NM_0201:	chr4	1.61E+08	1.62E+08	-	4817	1	FSTL5|-|-|	15.918	6.682
NM_0189<	chr21	32268219	32279065	-	1564	1	MIS18A|B:	5.911	4.852
NR_03618	chr11	81890741	81890836	-	95	1	MIR4300|-	0	0
NM_0012<	chr17	49845910	49848837	+	2095	1	FLJ45513|	0.055	0.039
NM_0012{	chr10	1.02E+08	1.02E+08	+	4568	1	PPRC1|PR	5.998	8.117
NM_0010{	chr10	1.03E+08	1.03E+08	+	4103	1	WBP1L|C1	27.21	35.807
NM_0012<	chr11	95790461	95832693	+	3088	1	CEP57|MV	9.269	10.552
NM_0168<	chr9	89005771	89178767	-	9754	1	SHC3|N-SH	9.405	15.228
NM_0144;	chr9	33240164	33248567	+	396	1	SPINK4|HE	0	0.035
NR_02680	chr9	66970162	66977317	-	1881	1	FAM74A3|	0	0
NM_0071(	chr6_GL0C	2892241	2893950	+	642	8	LST1|B144	0	0
NM_0328:	chr9	94726669	95087218	+	2905	1	C9orf3|AC	1.855	1.759
NM_0013:	chr2	55236620	55269248	-	2466	1	MTIF2|-|-	6.755	4.954
NR_13620	chr4	1.43E+08	1.43E+08	-	789	1	LOC10537	1.046	0.312
NM_1524;	chr19	9466355	9498603	-	2790	1	ZNF560|-|	2.311	2.376
NM_0052!	chr2	28392912	28414649	+	3985	1	FOSL2|FRA	1.223	4.235
NR_02436	chr21	41679302	41697336	+	451	1	LINC00111	0	0
NM_0056{	chr2	2.19E+08	2.19E+08	-	2997	1	ABCB6|AB	6.423	7.943

NM_18098	chr13	94601850	94634645	+	8864	1	GPR180|IT	0.684	2.02
NM_00108	chr2	1.7E+08	1.71E+08	+	6095	1	MYO3B|-|	0.154	0.281
NM_00119	chr9	1.15E+08	1.15E+08	-	724	1	LOC10050	0	0
NR_13423	chr8	1.44E+08	1.44E+08	-	3366	1	PARP10|A	1.36	0.932
NM_00672	chr6	1.61E+08	1.61E+08	+	5369	1	MAP3K4|N	4.39	6.546
NM_00215	chr12	1.22E+08	1.22E+08	-	1407	1	HPD|4-HP	2.767	13.57
NM_00049	chr22	25219645	25231869	+	755	1	CRYBB2|C	0.017	0.073
NR_03616	chr20	63238779	63238842	+	63	1	MIR3196|	0	0
NM_17223	chr6	50713544	50773033	+	2030	1	TFAP2D|T	1.061	0.074
NR_03407	chr11	1.05E+08	1.05E+08	-	614	1	LOC64373	0.063	0
NM_00114	chr8	65644653	65708338	+	1463	1	MTFR1|CH	7.28	6.966
NR_02658	chr12	45062618	45065411	-	2793	1	RACGAP1P	0.642	0.598
NM_01607	chr15	83138052	83208018	-	2426	1	HDGFRP3|	36.06	35.53
NR_00438	chr15	66347206	66347342	-	136	1	SCARNA14	0.19	0
NM_00643	chr11	308107	309410	+	726	1	IFITM2|1-8	8.898	2.547
NR_03389	chr17	27418005	27431623	+	3070	1	TBC1D3P5	0	0.018
NM_00218	chr2	2.19E+08	2.19E+08	-	2067	1	IHH|BDA1	0.05	0.033
NM_15232	chr14	96392111	96489427	+	3280	1	AK7|AK 7|	0.668	0.372
NM_03063	chr7	36153227	36301543	+	4677	1	EEPD1|HS	1.001	3.503
NM_01614	chr19	50659257	50716938	-	9188	1	SHANK1|S	11.513	11.015
NM_02196	chr1	26911484	26914076	-	1267	1	NR0B2|SH	0	0
NR_02410	chr7	72829373	72834006	+	848	1	SBDSP1|SE	13.41	16.572
NM_00255	chr11	59574398	59616144	-	5067	1	OSBP|OSB	17.377	20.658
NM_00100	chrX	1.54E+08	1.54E+08	-	2658	1	DNASE1L1	6.083	3.937
NR_04663	chr13	66990886	67002000	+	426	1	PCDH9-AS4	0	0
NM_00130	chr19	11420605	11435782	-	1852	1	CCDC151|	4.651	2.293
NR_04987	chr7	97963663	97963721	+	58	2	MIR5692A	0	0
NM_02404	chr16	29454501	29458224	+	1162	2	SLX1B|GIY	0	0
NR_02435	chr21	27722379	27751233	+	520	1	LINC00113	0	0
NM_00132	chr2	1.18E+08	1.18E+08	+	2260	1	INSIG2|INS	5.853	4.818
NM_01661	chrX	12906620	12923169	+	4348	1	TLR8|CD28	0	0
NM_05295	chr11	1.25E+08	1.25E+08	+	1593	1	PANX3|PX	0.016	0
NM_03195	chr17	41008521	41009114	-	591	2	KRTAP3-1|	0	0
NM_00126	chr17	30116807	30186475	+	2413	1	NSRP1|CC	3.468	4.706
NR_03605	chr19	20399272	20399354	-	82	1	MIR1270|	0	0
NM_00133	chr17	7857727	7862282	+	3691	1	CYB5D1|-|	9.033	7.403
NM_01786	chr3	1.97E+08	1.97E+08	+	3031	1	PIGX|PIG-X	8.274	8.13
NM_01537	chr7	98281667	98291527	+	786	1	BRI3|I3|-|	52.099	38.251
NM_01894	chr9	98056677	98083083	+	1232	1	NANS|HEL	18.493	20.301
NM_01828	chr6	1.7E+08	1.7E+08	-	1659	1	PHF10|BA	6.995	6.473
NR_12064	chr10	69215333	69232490	-	1928	1	LOC10192	0	0.007
NR_13464	chr4	1.37E+08	1.37E+08	+	3155	1	LOC72930	0	0
NM_19815	chr3	69739435	69968337	+	4803	1	MITF|CMN	0.166	0.174
NM_00128	chr1	2.23E+08	2.23E+08	+	3720	1	BROX|C1o	2.956	3.384
NR_10427	chr22	20021069	20067164	+	3162	1	TANGO2|C	6.288	7.892
NM_00119	chr2	1.61E+08	1.61E+08	+	2030	1	TANK|I-TR	8.867	15.687
NM_20731	chrX	1.04E+08	1.04E+08	+	7593	1	FAM199X|	3.318	3.682
NM_00118	chr3	1.7E+08	1.7E+08	+	4807	1	MYNN|OS	2.264	3.009
NM_03279	chr12	1.22E+08	1.22E+08	+	1491	1	ORAI1|CRA	3.398	4.85
NM_00111	chr17	8753106	8758559	-	915	1	SPDYE4|-|	0	0

NM_1446!	chr9	1.36E+08	1.36E+08	-	6891	1	NACC2|BE	2.232	4.031
NM_0182:	chr1	2508531	2526628	-	2658	2	PANK4|-|-	0	0
NM_0219:	chr6_GL0C	4629541	4636649	-	2907.3	6	RXRB|DAU	0	0
NR_11032	chr19	34677639	34686397	+	2438	1	ZNF302|H:	6.272	9.663
NM_0012:	chr7	1E+08	1E+08	-	2582	1	ZNF3|A8-5	33.482	38.163
NM_0010:	chr3	9792495	9807105	+	1452	1	ARPC4|AR	111.894	105.51
NM_0023(	chr14	55129217	55145430	+	992	1	LGALS3|CE	9.254	4.391
NR_02685	chr13	19008259	19012634	+	2134	1	LINC00442	0	0
NM_0189:	chr5	1.41E+08	1.41E+08	+	3869	1	PCDHB4|P	0.467	0.513
NM_0010(	chr11	58023881	58181566	+	2433	1	OR9Q1|-|-	0	0
NM_0185!	chr13	44989530	45028269	+	1404	1	GPALPP1|/	8.026	7.405
NR_10678	chr1	12578957	12579023	-	66	1	MIR6730|I	0	0
NR_03388	chr13	29936531	29950488	+	691	1	LINC00544	0	0
NM_0012!	chr19	56141166	56160893	+	2054	1	ZNF444|EZ	26.823	26.721
NM_0011:	chr12	47709735	47725572	-	2447	1	ENDOU|P1	0.026	0.106
NM_0162:	chr17	28042662	28196378	+	3525	1	NLK|-|-|1:	13.073	9.38
NM_1722‹	chrX	1268800	1309935	+	1814	2	CSF2RA|CI	0	0
NM_0012(	chr2	24077433	24084331	-	947	1	TP53I3|PI(	25.692	11.759
NR_02850	chr15	20916692	20929296	-	958	5	NF1P2|NF	0	0
NM_0132‹	chr12	85979259	86838904	-	1925	1	MGAT4C|(	3.275	1.259
NM_0007:	chr11	62908679	62921540	-	2819	1	CHRM1|HI	0.018	2.211
NR_12639	chr16	9441294	9444985	-	532	1	LINC01177	0	0
NM_0013:	chr20	46118254	46129745	+	1464	1	CD40|Bp5	0.167	0.159
NM_0012:	chr17	49700934	49707951	-	1575	1	SLC35B1|U	44.01	48.944
NR_10462	chr1	9425094	9428916	-	2361	1	LOC10050	0.743	0.963
NM_1768:	chr19	55708432	55738402	-	3473	1	NLRP9|CLI	0.134	0.083
NM_2071:	chr10	1.33E+08	1.33E+08	+	1691	1	PAOX|PAC	5.231	4.155
NM_0809:	chr1	1.99E+08	1.99E+08	+	3177.5	2	PTPRC|B2:	0.012	0.039
NR_02684	chr21	38121451	38156511	+	456	1	DSCR8|C2	0	0.12
NR_13465	chr6	19802164	19804759	-	1830	1	LOC10050	0.528	0.419
NM_0007{	chr20	54153449	54173977	-	3252	1	CYP24A1|(	0	0.042
NM_0012{	chr2	74557883	74648338	-	2528	1	M1AP|C2c	0.031	0.049
NM_0011!	chr10	1.11E+08	1.11E+08	-	2051	1	BBIP1|BBI	11.932	9.864
NM_0169‹	chr7	1.23E+08	1.23E+08	-	993	1	TAS2R16|T	0	0
NM_0217:	chr8	24294040	24336099	+	2119	1	ADAM28|/	0.006	0.084
NR_03824	chr10	92418661	92419606	+	945	1	MARK2P9|	0.15	0.043
NR_13488	chr19	50785728	50786210	-	274	1	LOC10537:	0.659	0.85
NM_0009:	chr8	73290639	73293634	-	828	1	RPL7|L7|h	0.638	0.232
NR_14429	chr7	1.35E+08	1.35E+08	+	2020	1	AGBL3|CC	0.268	0.386
NM_0012(	chr5	1.76E+08	1.76E+08	+	1772	1	FAM153B|	0	0
NM_0012{	chr3	63833870	63863923	-	883	1	THOC7|NI	37.439	32.606
NM_0012{	chr1	1.67E+08	1.67E+08	+	1684	1	MAEL|CT1	0.337	0.22
NM_0010(	chr11	4768979	4769917	-	937	1	OR51F1|O	0	0
NM_0057:	chr15	77044018	77071228	-	3844	1	TSPAN3|TI	105.486	110.937
NM_1788(	chr3	31532501	31637622	+	4519	1	STT3B|CD(	14.551	16.622
NM_0034:	chr7	1E+08	1E+08	+	9439	1	ZKSCAN1|	20.535	19.153
NM_0010!	chr12	1.04E+08	1.04E+08	+	3841	1	TXNRD1|G	25.767	29.541
NM_0007:	chr11	1.18E+08	1.18E+08	-	734	1	CD3D|CD3	0	0
NR_02455	chr10	1.03E+08	1.03E+08	-	1829	1	CALHM2|F	1.516	1.663
NM_0012!	chr11	75768733	75801536	+	2316	1	DGAT2|AR	0.913	3.992

NM_0012{	chr3	99833144	99876203	-	3021	1	FILIP1L|DC	0.653	1.038
NR_04001	chr17	331205	333488	+	1010	3	LOC10050	0	0
NM_0011!	chr5	1.19E+08	1.2E+08	+	2606	1	HSD17B4|	34.658	42.389
NM_0146!	chr1	30869466	30908633	-	5114	1	SDC3|SDC	153.237	149.912
NM_0024;	chr11	1.03E+08	1.03E+08	-	2707	1	MMP13|C	0	0.005
NM_0012:	chr1	1.5E+08	1.5E+08	-	2375	1	SV2A|SV2	88.213	99.478
NR_10355	chr5	38556786	38671216	+	3377	1	LIFR-AS1|-	1.726	1.602
NM_0010(	chr19	110679	111596	+	916	1	OR4F17|O	0	0
NR_00368	chr2	73644919	73685567	+	1585	1	ALMS1P1|	0.212	0.199
NR_11039	chrX	1.08E+08	1.08E+08	-	2086	1	LOC10192	0	0
NR_07510	chr10	87357720	87370695	+	5536	1	NUTM2D|	0.38	0.45
NM_0013;	chr12	7123681	7130014	-	1286	1	RBP5|CRB	1.323	1.619
NR_03961	chr3	1.04E+08	1.04E+08	-	83	1	MIR548AB	0	0
NM_0013:	chr1	1.46E+08	1.46E+08	-	2588	1	TXNIP|ARF	114.118	138.976
NR_13504	chr12	1.22E+08	1.22E+08	+	807	1	LOC10050	0.176	0.441
NM_0010{	chr8	8317736	8381834	-	4719	1	PRAG1|NA	14.639	19.043
NR_10685	chr19	40369846	40369907	+	61	1	MIR6796|I	136.121	107.78
NM_0039{	chr11	27040962	27127807	+	1875	1	BBOX1|BB	0.784	2.111
NM_0209:	chr2	2.02E+08	2.02E+08	-	6882	1	ALS2|ALS2	9.287	12.206
NM_1980:	chr11_KI2	67163	84556	+	2758	2	LRRC56|-|	0.379	0.169
NM_0247!	chr19	15226919	15232420	-	1801	1	EPHX3|AB	0.286	0.175
NR_02960	chr9	1.2E+08	1.2E+08	-	71	1	MIR147A|	0	0
NR_12636	chr11	72570656	72573222	+	2445	1	LINC01537	0.011	0.174
NR_12582	chr8	41534305	41578368	-	406	1	LOC10272	1.239	0.574
NM_1529;	chr20	35626031	35664956	-	1962	1	CPNE1|CO	84.826	108.683
NM_0044!	chr19	46918676	47005076	+	8880	1	ARHGAP35	34.985	43.675
NM_0018;	chr1	1.55E+08	1.55E+08	+	811	1	CKS1B|CKS	5.469	4.661
NR_10682	chr16	2463967	2464038	+	71	1	MIR6768|I	0.182	0
NM_0228;	chr2	27491883	27495259	-	1662	1	FNDC4|FR	38.339	41.561
NR_13363	chr11	64890686	64893449	-	2519	1	MIR194-2H	0	0.038
NR_00361	chr16	69976299	70065948	-	4156	1	PDXDC2P|	1.294	1.43
NM_0010!	chr6_GL0C	1207200	1210386	+	987.9	7	HLA-F|CDA	0	0
NM_0033;	chr6	35497874	35512938	-	2145	1	TULP1|LCA	0.048	0.038
NM_1817;	chr4	1.85E+08	1.85E+08	+	936	1	ANKRD37|	7.783	2.766
NR_03748	chr11	1.02E+08	1.02E+08	-	85	1	MIR3920|-	0	0
NR_10407	chr1	1.21E+08	1.21E+08	+	163	1	RNVU1-4|	0	0
NM_1757:	chr1	2.08E+08	2.08E+08	+	1823	1	CR1L|-|-|1	0.05	0
NR_02404	chr11	76680166	76721789	-	2778	1	GUCY2EP|	0	0
NM_0011(	chr17	54961206	54968785	-	902	1	COX11|CC	15.667	15.307
NM_1526!	chr3	23203293	23590805	+	1752	1	UBE2E2|U	21.283	35.321
NM_1525!	chr15	34102073	34104390	+	2315	1	PGBD4|-|-	1.214	1.213
NR_07308	chr1	2.01E+08	2.01E+08	-	1622	1	PHLDA3|T	7.488	10.083
NM_2068{	chr1	93180716	93278730	+	4292	1	CCDC18|N	0.15	0.447
NM_0042(	chrX	1.3E+08	1.3E+08	-	2249	1	AIFM1|AIF	17.772	22.443
NM_1815:	chr17	40766238	40772159	-	1720	1	KRT26|CK2	0	0
NR_12373	chr11	3638506	3642316	-	610	1	ART5|ART	0.444	0.606
NM_0011⁄	chr10	53808692	54801291	-	5730	1	PCDH15|C	0.614	0.54
NM_1526:	chr19	57668935	57679152	+	2239	1	ZSCAN4|ZI	0.006	0.006
NM_0177!	chr9	1.01E+08	1.01E+08	+	2312	2	PLPPR1|LF	0	0
NM_1733⁄	chr8	1.33E+08	1.34E+08	-	6756	1	ST3GAL1|(	3.094	9.634

NM_0050!	chrY	21511338	21525786	+	1907	3	RBMY1A1|	0	0
NM_1342(	chr17	76527348	76537905	-	2160	1	CYGB|HGE	3.958	2.695
NM_0063!	chr17	60443149	60526240	-	6484	1	APPBP2|A	6.471	7.436
NM_0001	chr19	48965309	48966879	+	865	1	FTL|LFTD|	849.449	440.236
NM_0122	chr18	79863668	79899816	+	1398	1	KCNG2|KC	1.697	0.382
NM_0000!	chr15	90717327	90815462	+	4531	1	BLM|BS|R	0.913	0.801
NR_13456	chr21	33976382	33977691	+	1132	1	LOC10192	0.057	0.157
NM_0010(	chr9	1.23E+08	1.23E+08	+	934	1	OR1L6|HG	0	0
NR_13558	chr2	1.04E+08	1.04E+08	-	589	1	LINC01831	0.044	0.07
NM_0011	chr9	1.37E+08	1.37E+08	+	4711	1	NDOR1|NI	8.465	9.586
NR_02782	chr6_GL0C	1739329	1746646	+	4614.8	6	HLA-L|HLA	0.073	0.122
NM_0003(	chr22	32800816	32863041	+	5479	1	TIMP3|HSI	5.631	4.007
NM_0008!	chr4	1.86E+08	1.86E+08	+	2280	1	KLKB1|KLK	0.04	0.042
NM_0159	chr8	96239398	96261613	-	1460	1	MTERF3|C	7.215	8.34
NM_0169	chr7	1.42E+08	1.42E+08	+	898	1	TAS2R4|T2	0.416	0.595
NM_0012	chr11	78435961	78574864	-	2022	1	NARS2|DF	13.295	15.641
NM_0331!	chr17	28553388	28571869	-	2859	1	PIGS|-|-|1	34.581	29.948
NR_10702	chr11	23419105	23419190	-	85	1	MIR8054|I	0	0
NM_0033	chr19	10350528	10380572	-	4221	1	TYK2|IMD	9.46	9.764
NR_02836	chr6	33583699	33589026	+	993	1	GGNBP1|-	0.039	0.276
NR_03617	chr22	30731557	30731641	+	84	1	MIR3200|	0	0
NR_02802	chr16	19113932	19121630	+	7181	1	ITPRIPL2|-	0.205	0.031
NM_1307(	chr7	1.17E+08	1.17E+08	-	1850	1	ASZ1|ALP1	0	0
NM_0010	chr19	8321500	8322396	+	376	1	RPS28|DB	66.731	51
NR_02730	chr1	2.48E+08	2.48E+08	-	1909	1	GCSAML-A	0	0
NM_0156	chr7	1.16E+08	1.16E+08	+	2773	1	TES|TESS|	10.08	7.053
NM_0144!	chr6	47231527	47309947	-	3634	1	TNFRSF21	13.223	13.727
NR_02987	chr14	1.01E+08	1.01E+08	+	66	1	MIR379|M	0	0
NR_10385	chr8	60402171	60413818	-	3482	1	LINC01301	0.507	0.574
NM_0013	chr10	99329099	99394330	+	5797	1	CNNM1|A	0.26	0.371
NM_0010(	chr11	46936689	47162247	+	1729	1	C11orf49|	74.833	71.392
NR_00296	chr12	48654382	48654518	-	136	1	SNORA2C|	0.569	1.007
NR_00297	chr11	8685439	8685569	+	130	1	SNORA3B|	2.975	4.952
NM_0013(	chr6	85505497	85594156	-	3463	1	SNX14|RG	8.683	10.031
NR_10690	chr8	1.45E+08	1.45E+08	-	60	1	MIR6850|I	2.579	0.913
NM_0010	chr10	49014245	49115532	-	6405	1	VSTM4|C1	0.815	1.101
NM_0176!	chr19	9648662	9675100	-	5562	1	ZNF562|-|	5.014	5.561
NM_0012	chr16	67806878	67828068	+	2218	1	TSNAXIP1|	5.453	3.545
NR_00335	chr15	25247345	25247426	+	81	1	SNORD115	0	0
NM_0011(	chr6_GL0C	1908201	1928166	-	2966.8	8	DHX16|DE	0	0
NR_13529	chr14	1.05E+08	1.05E+08	-	3814	1	LOC10192	0	0
NM_0528!	chr17	34961530	34963186	+	1654	1	ZNF830|C	10.422	9.54
NR_03839	chr20	3106911	3150867	+	1787	1	UBOX5-AS	0.75	1.088
NR_12045	chr17	6893777	6900349	+	2202	1	ALOX12P2	0.012	0.05
NR_03961	chr1	30739156	30739232	-	76	1	MIR4420|	0	0
NM_0012(	chr17	18744013	18779349	+	3426	1	FBXW10|F	0.015	0.028
NR_13650	chr18	508568	515294	-	497	1	LINC01925	0	0
NR_02696	chr22	27919376	27924963	+	945	1	TTC28-AS1	3.479	4.189
NM_1450!	chr16	70454595	70480274	+	3881	1	FUK|1110(	4.279	3.949
NM_0043	chr15	78340324	78348230	+	766	1	CRABP1|C	620.537	806.872

NR_03411	chr22	42090933	42125349	+	1369.7	7	NDUFA6-A	0	0
NM_0010(	chr10	1.1E+08	1.1E+08	+	3040	1	MXI1|MAI	22.525	28.358
NM_0055:	chr15	78149377	78170542	+	2652	1	IDH3A|-|-	7.827	8.661
NR_03624	chr7	73711317	73711397	+	80	1	MIR4284|i	0	0
NR_04521	chr11	1.17E+08	1.17E+08	-	1236	1	LOC10065	5.779	7.669
NM_0010(	chr7	1.44E+08	1.44E+08	+	952	1	OR2F2|OR	0	0
NR_00332	chr15	25074113	25074216	+	103	1	SNORD116	0	0
NR_13657	chr22	18178026	18205863	-	2926	1	LOC10099	0	0
NR_03750	chr6	1.67E+08	1.67E+08	-	105	1	MIR3939|i	0.246	0
NM_0020	chr12	64713442	64759446	-	5112	1	GNS|G6S|	26.667	30.199
NM_0252(	chr6	30556709	30564696	+	3398	7	PRR3|CAT	0	0
NM_0140	chr15	45092654	45114161	-	6310	1	DUOX2|LN	0.137	0.022
NM_0121!	chrX	1.55E+08	1.55E+08	+	1699	3	F8A1|DXS!	1.419	1.403
NR_03406	chr11	1.05E+08	1.05E+08	-	1571	1	CASP12|C	0	0
NM_0178:	chr21	33488055	33491483	-	1784	2	DNAJC28|	0	0
NM_0246	chr1	37802942	37808193	-	1829	1	YRDC|DRI	13.105	15.015
NM_0011	chr3	1.96E+08	1.96E+08	-	5062	1	TFRC|CD7	19.442	18.952
NM_0012	chr15	89681531	89690784	-	2622	1	PEX11A|PI	3.457	2.335
NM_0013	chr21	37006202	37019658	+	1879	1	RIPPLY3|D	0.117	0.022
NM_0011(	chr17	41059241	41060092	-	849	2	KRTAP2-3|	0	0
NM_0013	chr19	38647616	38730532	+	3928	1	ACTN4|AC	39.206	31.516
NM_0013	chr10	1.02E+08	1.02E+08	+	3104	1	NFKB2|CV	3.34	2.996
NR_03412	chr5	81237565	81301569	-	2072	1	CKMT2-AS	1.456	1.494
NM_0054(	chr2	45651904	46187990	+	5503	1	PRKCE|PK	2.575	5.662
NM_0058	chr10	1.1E+08	1.1E+08	-	2022	1	SMNDC1|	12.549	14.056
NR_13301	chr22	23772819	23784316	+	2093	2	MMP11|S	0	0
NM_0140	chr6_GL0C	2421726	2423058	-	1133	6	C6orf15|S	0	0
NM_0011(	chr13	31739542	31802872	+	2712	1	RXFP2|GP	0.029	0.01
NR_12033	chr15	38040393	38062340	+	401	1	LOC10192	0	0
NR_11052	chr1	1.99E+08	1.99E+08	-	765	1	LINC01222	0	0
NM_0528!	chr19	20167214	20200490	+	3891	1	ZNF486|KI	0.954	1.119
NR_12589	chr4	4920808	4988671	+	666	1	LOC10192	0.019	0.041
NM_0011(	chr3	46242381	46266706	+	1772	1	CCR3|CC-(	0	0
NM_0178	chr13	36168208	36214615	-	2180	1	SOHLH2|S	0.018	0.377
NM_0012!	chr4	83407343	83455883	-	3386	1	HELQ|HEL	2.003	1.946
NR_03890	chr11	43909290	43920944	-	1920	1	ALKBH3-A!	0.094	0.071
NM_0324:	chr19	55284166	55312535	+	3060	1	BRSK1|hS	98.306	103.157
NM_0010	chr3	16257061	16264989	-	3986	1	DPH3|DEL	10.037	8.275
NR_12587	chr6	84689458	84709534	+	601	1	TBX18-AS1	0	0
NM_0010:	chr7	23105734	23175419	+	3162	1	KLHL7|CIS	28.03	36.144
NM_0013(	chr17_GLC	952322	1166895	+	2507.3	3	CRHR1-IT1	0.01	0
NM_0176	chr7	1.02E+08	1.02E+08	-	2116	1	ALKBH4|A	12.26	12.292
NM_0216	chr4	1.7E+08	1.7E+08	-	6336	1	MFAP3L|N	0.533	0.515
NM_0045	chr11	63536801	63546458	+	759	1	RARRES3|	0.238	0.83
NR_07340	chr1	61654242	61656128	+	1886	1	MGC3479(	0.014	0
NM_0012	chr8	93134095	93166850	-	583	1	C8orf87|-|	0.022	0.141
NM_0154	chr2	86024446	86106155	-	8483	1	POLR1A|A	9.987	11.874
NM_0183(	chr11	76860873	77026797	+	7365	1	ACER3|AP	2.183	2.181
NM_0011	chr11	20022556	20121601	+	8052	1	NAV2|HEL	22.182	9.596
NR_10705	chr1	1.54E+08	1.54E+08	-	88	1	MIR8083|	0	0

NM_0012:	chr6	1.3E+08	1.3E+08	-	1878	1	SAMD3|-|	0.666	0.357
NM_0012(	chr5	1.73E+08	1.73E+08	+	1249	1	BNIP1|NIP	12.945	16.066
NM_0010<	chr11	62123973	62153163	+	4125	1	INCENP|-|	5.539	5.21
NM_0039(	chr6	1.33E+08	1.33E+08	-	1144	1	TAAR5|PN	0	0
NM_0185!	chrX	1.53E+08	1.53E+08	+	1989	1	GABRQ|TH	1.348	2.823
NM_1992<	chr9	65737146	65738396	+	1249	1	FOXD4L4|	0	0.164
NR_02984	chr14	1.01E+08	1.01E+08	+	62	1	MIR299|M	0	0
NM_0049(	chrX	1.52E+08	1.52E+08	-	3141	1	GABRE|-|-	0.374	0.497
NR_03897	chr10	49121839	49151547	+	1994	1	FAM170B-	0	0.007
NM_0013:	chr2	2.07E+08	2.07E+08	-	2410	1	MDH1B|RI	1.867	1.148
NM_0180:	chr7	1.28E+08	1.28E+08	-	2788	1	RBM28|AI	7.464	6.686
NM_0011:	chrX	1.32E+08	1.32E+08	-	11255	1	MBNL3|CH	0.148	0.166
NM_0011(	chr4	39822863	39977956	-	7141	1	PDS5A|PI(	8.567	11.163
NR_03163	chr7	18127220	18127309	-	89	1	MIR1302-6	0	0
NM_0221(	chr20	45934678	45948023	+	2678	1	PCIF1|C20	30.794	33.925
NM_0011:	chr16	67279524	67289500	+	4516	1	PLEKHG4|.	0.106	0.452
NM_0017(	chr6	68635740	69389511	+	5603	1	ADGRB3|E	6.657	11.186
NM_0010:	chrX	1.04E+08	1.04E+08	-	6221	1	SLC25A53|	1.293	1.651
NM_0164:	chr17	48026167	48037806	-	912	1	COPZ2|zet	1.555	0.165
NM_0013(	chr11	1.08E+08	1.08E+08	-	4024	1	ALKBH8|A	1.782	1.937
NM_0061:	chr8	22165140	22212326	+	3803	1	BMP1|OI1	3.099	3.072
NM_0010(	chr3	48620842	48621756	-	477	1	TMEM89|-	0.054	0.115
NM_0010:	chr11	1.05E+08	1.05E+08	-	690	1	CARD16|C	0	0
NM_0059:	chr1	1.54E+08	1.54E+08	-	957	1	S100A2|C/	0.337	0.315
NR_04002	chr18	76794732	76822295	-	1292	1	LOC10013	0.489	0.636
NR_11011	chr12	1.06E+08	1.06E+08	+	2389	1	CASC18|-|	0.016	0.052
NM_0011<	chr21	36460621	36480090	-	1748	1	CLDN14|D	0	0
NM_2034:	chr4	7939001	7940296	+	1293	1	LOC38919	1.625	1.6
NR_00222	chr17	32150368	32151571	-	1203	1	ARGFXP2|	0.021	0.102
NR_03827	chr10	50021182	50067803	-	2170	1	FAM21EP|	0.22	0.139
NR_13074	chr12	14477	32015	-	1864	2	LOC10028	0	0
NM_0211!	chr20	7977353	8019829	-	6154	1	TMX4|DJ9	14.104	12.684
NM_0013:	chr11	62728480	62738636	+	1047	1	TTC9C|-|-	26.76	26.884
NM_0011:	chr3	1.29E+08	1.29E+08	-	3356	1	CNBP|CNE	63.069	61.632
NM_0308!	chr8	1.43E+08	1.43E+08	+	4679	1	ZNF696|-|	4.74	4.107
NR_00302	chr1	1.09E+08	1.09E+08	+	419	1	SCARNA2|	0.431	0.36
NM_0013<	chr17	2030112	2043431	+	2084	1	DPH1|DEC	26.486	26.08
NR_02725	chr2	38131105	38181851	+	1750	1	CYP1B1-AS	0.022	0.008
NM_0012:	chr2	2.3E+08	2.3E+08	+	3043	1	SP140|LYS	0.042	0.09
NR_03972	chr14	1.01E+08	1.01E+08	+	83	1	MIR2392|-	0	0
NR_12057	chr11	1.25E+08	1.25E+08	+	1470	1	LOC10192	0.184	0.158
NM_0070!	chr1	50441263	50960264	-	2562	1	FAF1|CGI-(	24.715	31.762
NM_0203:	chr1	25242237	25247519	-	1495	1	RSRP1|C1(	9.142	8.127
NM_0012<	chr20	33235996	33243309	+	1051	1	BPIFA1|LU	0	0
NM_0806(	chr14	31446036	31457474	-	2676	1	DTD2|C14	5.165	5.856
NM_0331:	chr7	12589523	12653603	+	2555	1	SCIN|-|-|7	0.035	0.048
NM_1825(	chr4	68226653	68245694	-	2633	1	TMPRSS11	0	0.005
NM_0240!	chr19	17309528	17323297	+	4070	1	DDA1|C19	23.24	20.771
NM_0011:	chr19	43007657	43026479	-	1168	1	PSG11|PSI	0	0.023
NR_12044	chr12	52093569	52108319	+	1000	1	OR7E47P|	0.09	0.192

NM_03308	chrX	1.52E+08	1.52E+08	+	1062	1	FATE1|CT4	0	0
NM_0013:	chr1	33145402	33176550	-	2931	1	TRIM62|D	14.829	15.978
NM_00068	chr10	1.11E+08	1.11E+08	+	3870	1	ADRA2A|A	0.543	1.334
NM_0185(	chr17	50381233	50397553	-	2906	1	LRRC59|PI	48.867	50.937
NM_0070:	chr21	26917912	26967120	-	9653	1	ADAMTS5	0.15	0.176
NM_1828:	chr11	77216558	77287418	-	2515	1	GDPD4|GI	0	0.005
NM_0012	chr15	84172490	84182233	+	493	1	UBE2Q2L|	0	0
NM_0007:	chr7	43758673	43807342	+	1071	1	BLVRA|BL\	38.115	36.564
NM_0041!	chr14	50329591	50395924	-	1602	1	CDKL1|KKI	1.36	1
NM_0012	chr1	1.75E+08	1.75E+08	+	6845	1	RABGAP1L	4.071	4.062
NR_10442	chr17_GL3	122756	126183	-	789	2	LINC02091	0	0
NM_0012:	chr16_KI2	1021570	1036245	+	1027	4	NPIPA2|NI	0.075	0.04
NM_0012!	chr12	55574415	55575344	+	928	1	OR2AP1|C	0	0
NM_1524:	chr8	87870743	87874068	-	3323	1	DCAF4L2|\	0	0
NM_0011:	chr19	57389850	57402551	+	4544	1	ZNF548|-|	7.701	8.898
NM_0048!	chr15	55318935	55355648	+	2183	1	PIGB|GPI-I	2.735	2.29
NM_0021	chr1	32886497	32894646	+	1400	1	HPCA|BDF	4.172	25.251
NM_0013:	chr1	43996483	44031123	-	3069	1	SLC6A9|GI	6.545	10.354
NM_0012{	chr3	1092658	1403608	+	3661	1	CNTN6|NE	0.039	0.026
NM_0063!	chr11	65570459	65571888	+	781	1	SSSCA1|p2	25.209	28.131
NM_0013	chr10	44911316	44937010	+	2828	1	TMEM72|	0.032	0.029
NM_0147(	chr12	1.09E+08	1.09E+08	-	4349	1	SART3|DS/	22.615	25.01
NM_0018	chr13	1.1E+08	1.11E+08	+	6413	1	COL4A2|IC	10.262	20.216
NM_0320	chr19	46410329	46413662	-	3331	1	CCDC8|3M	5.5	4.244
NM_1758:	chr19	34956354	34964049	-	3882	1	ZNF792|-|	2.295	3.306
NR_02402	chr21	25385820	25431701	-	1471	1	LINC0015٤	0.009	0.149
NM_0132{	chr20	14323993	14337667	-	3826	1	FLRT3|HH:	8.883	3.426
NM_0013:	chr12	12611833	12645108	+	3807	1	CREBL2|-|	14.16	11.017
NM_0057:	chr1	1.15E+08	1.15E+08	-	3203	1	TSPAN2|N	0.511	0.616
NM_1735:	chr15	81134303	81149175	+	1566	1	CFAP161|(	0.313	0.044
NM_0070:	chr10	44976261	44978882	-	2056	1	C10orf10|	1.323	2.465
NM_0541:	chr3	16174677	16229746	+	4629	1	GALNT15|	1.175	1.074
NR_04603	chr4	73869392	73871302	+	1188	1	CXCL1|FSF	0	0
NR_02789	chr3	1.3E+08	1.3E+08	+	9138	1	COL6A4P2	0.13	0.076
NR_11099	chr9	84063443	84094579	+	2000	1	LOC10192	0.013	0.041
NR_03413	chr4	65669961	65693386	+	1173	1	EPHA5-AS:	8.464	8.804
NM_0011	chr11	89710299	89717872	+	1346	1	TRIM77|TI	0	0
NM_0211:	chr11	67350765	67353596	-	1745	1	POLD4|PC	7.123	4.812
NM_0026:	chr1	63593276	63660246	+	2460	1	PGM1|CD	9.854	12.606
NM_0031!	chr6	1.71E+08	1.71E+08	+	1894	1	TBP|GTF2I	14.738	18.846
NR_13092	chr7	1.16E+08	1.16E+08	-	1993	1	LOC10272	0	0
NR_03979	chr7	93716928	93717005	+	77	1	MIR4652|	0	0
NM_0124(	chr1	6221193	6235984	-	4808	1	ICMT|HSTI	14.149	12.372
NM_0033!	chr11	74000281	74009237	-	2416	1	UCP3|SLC:	1.297	0.93
NM_0002(	chr19	10270841	10286615	+	3237	1	ICAM1|BB	0.617	0.165
NR_03162	chr1	18897071	18897148	-	77	1	MIR1290|	0	0
NM_0251!	chr12	1.07E+08	1.07E+08	-	1775	1	MTERF2|N	5.855	6.073
NM_0012!	chr16	30740191	30745196	+	2095	1	TMEM265	5.133	4.838
NM_0013:	chr2	2.38E+08	2.38E+08	+	6581	1	ASB1|ASB-	9.582	12.369
NR_10698	chr11	1.25E+08	1.25E+08	-	49	30	MIR7641-:	2.892	10.056

NR_12595	chr1	1.06E+08	1.06E+08	-	1822	1	LINC0167	0	0
NM_0011	chr16	30023334	30024702	+	809	1	C16orf92|	28.943	23.974
NR_10372	chr7	1E+08	1E+08	+	1259	1	PVRIG2P|-	6.011	6.887
NM_0133	chr2	2.42E+08	2.42E+08	+	2862	1	ATG4B|AP	41.672	46.274
NR_03169	chr17	16282014	16282088	+	74	1	MIR1288|	0	0
NR_02713	chr11	45771432	45772358	+	647	1	DKFZp779	0.438	0.085
NM_0065	chr1	1.55E+08	1.55E+08	-	3176	2	FAM189B|	0	0
NM_0151	chr8	1.43E+08	1.43E+08	-	2943	1	ARC|Arg3.	2.642	4.454
NM_1826	chrX	11293413	11300761	+	738	1	AMELX|AI	0	0.019
NR_03962	chr1	1.93E+08	1.93E+08	+	59.7	3	MIR4426|	444.069	393.234
NR_10700	chr14	64785626	64785686	-	60	1	MIR7855|	0.43	0
NM_0013	chr16	4750814	4767218	-	2433	1	ZNF500|Z	6.757	7.127
NR_04702	chr13	1.07E+08	1.07E+08	+	1220	1	LINC0044	0	0
NR_12636	chr10	77350872	77376613	+	1228	1	KCNMA1-	0	0
NR_04665	chr3	44617128	44624797	-	621	1	ZNF197-A	0.81	1.169
NR_03369	chr1	23369218	23371837	+	689	1	ZNF436-A	5.988	3.936
NM_0011	chr17	76384609	76387860	+	1813	1	SPHK1|SP	2.112	1.201
NR_10373	chr15	66547468	66565979	-	2307	1	LCTL|KLG|	2.616	1.817
NR_04626	chr5	36871361	36876694	-	5333	1	NIPBL-AS1	5.399	6.246
NM_0035	chr5	1.38E+08	1.38E+08	-	1218	1	NME5|NM	4.213	2.688
NM_0012	chr19	6494319	6502848	-	2393	1	TUBB4A|D	108.04	111.94
NM_0000	chr3	98579446	98593611	-	2700	1	CPOX|CPC	3.978	3.333
NM_0029	chr19	13961530	14006322	-	4354	1	RFX1|EFC|	4.066	4.322
NM_1775	chr1	6555278	6579757	+	2026	1	TAS1R1|G	0.076	0.074
NR_13519	chr14	90697333	90699374	+	1952	1	LOC10537	0	0
NR_02715	chr16	21446683	21502281	-	5023	1	SMG1P3|-	0.252	0.289
NR_11072	chr19	37497161	37507087	-	873	1	ZNF793-A	7.724	7.845
NR_10467	chr5	1.13E+08	1.13E+08	+	829	1	LOC10246	1.151	1.404
NM_1815	chr20	36541484	36549823	+	1030	1	MYL9|LC2	0	0.332
NR_03614	chr17	30117086	30117160	-	74	1	MIR3184|	0	0
NR_10675	chrX	28495555	28495663	-	108	1	MIR6134|	0	0
NR_13626	chr7	81515747	81561819	-	643	1	LOC10536	0	0
NM_0011	chr12	1.08E+08	1.08E+08	-	5105	1	CMKLR1|C	0.01	0.016
NM_0013	chr5	1.79E+08	1.79E+08	-	2465	1	ZNF354A|	3.614	4.695
NM_0061	chr14	24080107	24084623	-	1969	1	NRL|D14S	0.72	1.774
NM_1524	chr19	35224801	35228725	-	1175	1	FAM187B|	0.011	0
NM_0011	chr11	72080331	72110782	+	3699	1	LRTOMT|C	11.123	9.439
NM_1523	chr10	96593312	96720522	-	4792	1	PIK3AP1|E	0.1	0.097
NR_03390	chr16	3263768	3267566	+	2440	1	LINC00921	0.275	0.298
NR_13631	chr2	1.85E+08	1.85E+08	-	612	1	LOC10537	0	0
NM_0044	chr14	99793408	99942058	+	4439	1	EML1|BH|	8.908	17.597
NM_0325	chr16	1351899	1363351	+	1232	1	GNPTG|C1	19.916	18.244
NR_13554	chr3	1.19E+08	1.19E+08	-	591	1	LOC10537	0	0.046
NM_0331	chrY	18546688	18548592	+	1423	2	HSFY1|HS	0	0
NM_1990	chr9	1.37E+08	1.37E+08	+	1293	1	CYSRT1|C	4.517	4.746
NR_03334	chr19	44105339	44113183	-	3359	1	LOC10037	5.359	13.057
NM_0000	chr16	56961923	56983844	+	1699	1	CETP|BPIF	0.015	0
NM_0329	chr4	88520978	88523801	-	1352	1	PYURF|PR	47.778	54.555
NR_04605	chr14	23512810	23559581	+	1129	1	THTPA|TH	30.938	30.451
NR_04651	chr13	48077137	48079993	+	541	1	MED4-AS1	19.996	18.507

NM_0000[illegible]	chr11	1.18E+08	1.18E+08	+	1302	1	CD3G|CD3	0	0
NM_0169[illegible]	chrX	24693947	24996985	+	5400	1	POLA1|NS	3.386	3.016
NM_0012[illegible]	chr1	23010834	23015850	-	505	1	C1orf234|	0.051	0.19
NM_1536[illegible]	chr21	37065364	37073158	-	797	1	PIGP|DCR[illegible]	10.37	7.321
NM_0530[illegible]	chr3	1.5E+08	1.5E+08	-	2100	1	PFN2|D3S	234.182	224.912
NM_0011[illegible]	chr7	30028361	30084662	+	7821	1	PLEKHA8|[illegible]	5.338	5.201
NM_0054[illegible]	chr11	45885496	45906465	+	3191	1	MAPK8IP1	74.904	68.949
NR_03028	chr2	32532153	32532246	+	93	1	MIR558|M	0	0
NM_0013[illegible]	chrX	71144831	71171201	+	3403	1	NLGN3|HN	27.511	30.493
NM_0035[illegible]	chr1	2.07E+08	2.07E+08	+	2162	1	DYRK3|DY	4.938	7.324
NM_0143[illegible]	chr19	15380048	15419121	-	2197	1	AKAP8L|H	56.768	84.489
NM_0013[illegible]	chr19	10922768	10928667	-	1552	1	YIPF2|Fin[illegible]	28.105	24.543
NM_0012[illegible]	chr2	2.41E+08	2.41E+08	+	896	1	PPP1R7|SI	43.012	45.662
NR_10415	chr8	32025736	32139477	+	396	1	NRG1-IT1|	0	0
NM_0020[illegible]	chr1	1.71E+08	1.71E+08	+	2291	1	FMO4|FM	0.163	0.12
NM_0011[illegible]	chr1	2.31E+08	2.31E+08	+	2937	1	COG2|LDL	13.403	16.001
NR_11037	chr15	66582190	66685798	+	832	1	LINC0116[illegible]	0	0
NM_0010[illegible]	chr10	73909969	73922777	-	2506	1	C10orf55|	0.103	0.443
NM_0012[illegible]	chr12	1.05E+08	1.05E+08	-	3225	1	APPL2|DIP	16.851	24.638
NM_0532[illegible]	chr6	1.33E+08	1.33E+08	+	1045	1	TAAR8|GP	0	0
NM_0012[illegible]	chr9	1.33E+08	1.33E+08	+	834	1	SURF2|SU	33.038	20.874
NM_0530[illegible]	chr3	1.51E+08	1.51E+08	+	10395	1	MED12L|N	0.478	0.76
NR_02970	chr14	1.01E+08	1.01E+08	+	83	1	MIR154|M	0	0
NM_0011[illegible]	chr19	36743478	36772814	-	7714	1	ZNF850|ZN	0.209	0.316
NM_0013[illegible]	chr9	1.33E+08	1.33E+08	+	713	1	SPACA9|C[illegible]	15.172	10.604
NM_0012[illegible]	chr22	24719034	24774720	-	3458	1	PIWIL3|HI[illegible]	0	0.012
NM_0044[illegible]	chr14	69380123	69398304	-	795	1	ERH|DROE	185.374	187.434
NM_1522[illegible]	chr17	35350310	35373701	-	4992	1	SLFN11|SL	0	0.005
NR_03883	chr4	1.71E+08	1.71E+08	+	1780	1	LOC10050[illegible]	0	0.015
NM_0010[illegible]	chr4	1.51E+08	1.51E+08	+	926	1	RPS3A|FTE	23.114	23.726
NM_0328[illegible]	chr15	50707540	50765713	-	1986	1	SPPL2A|IM	3.305	3.407
NR_13550	chr15	1.01E+08	1.01E+08	-	727	1	SNRPA1|L[illegible]	12.574	12.001
NM_0011[illegible]	chr11	1.19E+08	1.19E+08	+	5282	1	PHLDB1|L[illegible]	36.466	39.05
NM_1447[illegible]	chr5	1.41E+08	1.41E+08	+	1618	1	ZMAT2|Pt	67.041	87.168
NM_0011[illegible]	chr4	94207608	94291292	+	4996	1	SMARCAD	8.243	10.111
NR_00232	chr7	1.17E+08	1.17E+08	+	1567	1	ST7-OT4|N	0.592	0.909
NR_04622	chr4_GL0[illegible]	161987	178171	-	1739	1	ANKRD20[illegible]	0	0
NM_0052[illegible]	chr9	1.23E+08	1.23E+08	+	1591	1	GPR21|-|-	0.665	1.507
NR_13393	chr7	1.48E+08	1.48E+08	+	790	1	LOC10537[illegible]	0	0.052
NM_0326[illegible]	chr4	53460270	53558269	-	2828	1	LNX1|LNX	7.34	6.941
NM_0241[illegible]	chr17	42101406	42112733	-	2599	1	DHX58|D1	0.327	0.337
NR_03980	chr7	44881748	44881800	-	52	1	MIR4657|[illegible]	2.975	11.326
NM_0013[illegible]	chr19	39445546	39476668	+	3591	1	SUPT5H|S[illegible]	66.467	74.149
NM_0318[illegible]	chr4	70628744	70646819	+	5663	1	ENAM|AD	0	0.002
NM_1758[illegible]	chr19	9185594	9188817	+	3221	1	OR7D2|HT	0.1	0.179
NM_0010[illegible]	chr9	1.23E+08	1.23E+08	+	991	1	OR1N2|OF	0	0
NR_10693	chr7	1.01E+08	1.01E+08	+	71	1	MIR6875|[illegible]	0	0.579
NR_11018	chr2	1.71E+08	1.71E+08	-	582	1	LOC10192[illegible]	0.31	0.235
NR_13503	chr12	6963245	6964447	+	349	1	LOC10536[illegible]	0	0.078
NM_0146[illegible]	chr1	43946806	43968022	+	4005	1	IPO13|IMF	24.776	19.962

NM_0010:	chr11	47465937	47489024	-	4450	1	CELF1|BRU	28.05	29.804
NR_03963	chr2	1.84E+08	1.84E+08	+	69	1	MIR548AE	0	0
NR_12040	chr13	62789034	62796692	-	343	1	LINC00448	0	0
NR_03624	chr7	1.02E+08	1.02E+08	+	84	1	MIR4285|-	0	0
NR_03972	chr13	1.14E+08	1.14E+08	+	80	1	MIR4502|-	0	0.171
NM_0044:	chr1	1.51E+08	1.51E+08	-	1233	1	ENSA|ARP	66.873	46.679
NM_0011(	chr17	39637080	39664201	+	2817	1	STARD3|C.	19.965	27.112
NM_0021(	chr4	1.44E+08	1.44E+08	-	522	1	GYPB|CD2	0	0
NR_12041	chr13	94704744	94708486	-	2378	1	LOC10192	0.13	0.57
NM_0013‹	chr1	22636762	22639682	+	1114	1	C1QA|-|-|	0	0
NM_0013:	chr15	41559972	41579338	+	3915	1	TYRO3|BY	14.31	11.269
NR_04698	chr11	1.27E+08	1.27E+08	+	491	1	KIRREL3-A:	0	0
NR_03995	chr3	87226189	87226277	-	88	1	MIR4795|-	0	0
NR_13566	chr17_KI2	1168474	1173025	-	1499	2	LOC10272	0.92	1.27
NM_0047(	chr5	1.16E+08	1.16E+08	-	4323	1	ATG12|AP	8.19	6.669
NM_0121!	chr8	1.41E+08	1.41E+08	-	3479	1	AGO2|EIF2	6.608	5.646
NM_0149:	chr2	69457995	69643845	-	21241	1	AAK1|-|-|	4.251	4.672
NM_0010!	chr19	41363966	41384082	+	733	1	TMEM91|	8.443	6.353
NM_0010:	chr6	1.33E+08	1.33E+08	+	495	1	RPS12|S12	418.166	358.17
NR_02958	chr3	1.2E+08	1.2E+08	-	61	1	MIR198|M	21.56	17.065
NR_02458	chrX	73944324	74070382	+	1665	1	JPX|DCBAI	1.843	2.024
NM_0010:	chr15	40844445	40857655	+	2320	1	SPINT1|HA	0.456	0.531
NM_0010(	chr11	4586791	4587905	+	1112	1	OR52I2|OI	0	0
NR_10983	chr22	23538102	23547306	+	1197	1	PCAT14|-|	0.022	0
NR_11058	chr2	5634141	5691118	-	697	1	LINC01248	0.647	0.806
NR_13557	chr3	1.21E+08	1.21E+08	-	595	1	LINC02049	0	0
NR_00298	chr16	58559796	58559931	-	135	1	SNORA50A	1.051	1.116
NM_0011!	chr19	55379244	55384259	-	659	1	TMEM238	0	0.125
NM_0323:	chr19	11553043	11559236	-	1013	1	ELOF1|ELF	64.366	61.684
NR_02990	chrX	77005404	77005501	-	97	1	MIR325|M	0	0
NR_02811	chrX	47658833	47660111	+	563	1	UXT-AS1|-	9.687	7.591
NM_0151:	chr9	70258962	70354873	+	5938	1	SMC5|SM(	1.372	2.939
NR_10699	chr17	77089417	77089497	+	80	1	MIR6516|I	3.707	1.027
NM_0240:	chr20	44247258	44280917	+	2790	1	GDAP1L1|	98.878	111.687
NR_14431	chr5	75511750	75605819	+	10406	1	POLK|DINI	0.965	1.183
NR_03063	chr19	53982307	53982397	+	90	1	MIR935|M	1.862	3.196
NR_01535	chr22	17036570	17058792	+	2722	1	CECR7|SAI	0.014	0.735
NM_0018!	chr20	62817062	62841159	+	2451	1	COL9A3|D	3.177	3.638
NR_02675	chr2	1.3E+08	1.3E+08	-	4511	1	FAR2P1|H	0.066	2.9
NR_03032	chr8	99536636	99536730	-	94	1	MIR599|M	0	0
NM_0052:	chr17	49990005	49995224	-	2597	1	DLX3|AI4|	0.01	0.084
NR_02941	chr9	85806002	85842879	+	1755	1	LOC38976	1.33	1.99
NR_00256	chr11	62853663	62853732	-	69	1	SNORD30|	2.99	2.978
NM_0012!	chr1	44739818	44767766	+	2735	1	KIF2C|CT1	6.558	5.444
NM_0013:	chr14	73957916	74019332	-	3266	1	ENTPD5|C	4.666	4.911
NR_02692	chr2	2.38E+08	2.38E+08	-	843	1	LOC15117‹	1.621	0.764
NM_0011:	chr16	11547722	11586950	-	2465	1	LITAF|PIG:	8.102	15.331
NM_1989:	chr1	1.59E+08	1.59E+08	+	1958	1	PYHIN1|IF	0	0
NM_0012!	chr4	9215405	9216997	+	1591	3	USP17L11	0	0
NM_1532‹	chr6	44000600	44007615	+	5232	1	C6orf223|	0.047	0.081

NM_0010(	chr17	58169656	58170579	+	922	1	OR4D2|BC	0	0
NM_0011!	chr3	9792548	9836356	+	3973	1	ARPC4-TTL	17.19	15.031
NM_0037!	chr11	7987320	7996171	+	1246	1	EIF3F|EIF3	62.46	63.428
NM_0011:	chr12	4538784	4560047	+	2257	1	RAD51AP1	1.08	1.45
NM_1982:	chr4	3369997	3439913	+	2882	1	RGS12|-|-	27.527	26.187
NM_0011(	chrX	52751204	52761536	-	1319	2	SSX2B|CT5	0	0
NR_04690	chr1	2.21E+08	2.21E+08	-	560	1	HLX-AS1|-	0	0.049
NR_03964	chr2	2.39E+08	2.39E+08	-	99	1	MIR4441|-	0	0.138
NR_13512	chr9	1.24E+08	1.24E+08	-	2144	1	LOC10050.	0.758	1.457
NM_0032(	chr6	1.34E+08	1.34E+08	+	1265	1	TCF21|POI	0	0
NR_03033	chr10	96828669	96828764	-	95	1	MIR607|M	0	0
NM_0013:	chr19	52527656	52556050	+	3203	1	ZNF808|-|	0.753	1.445
NM_1994	chr2	1.97E+08	1.98E+08	-	2287	1	HSPD1|CP	51.219	42.936
NM_0013:	chr3	1.84E+08	1.84E+08	+	989.5	2	LINC02054	0.013	0
NM_0011(	chr4	6782732	6884172	+	7762	1	KIAA0232|	13.827	17.553
NM_0057(	chr12	6976185	7018538	-	2282	1	LPCAT3|C3	13.306	16.296
NM_0011!	chr14	1.04E+08	1.04E+08	+	1497	1	ZFYVE21|H	18.165	15.408
NM_0806(	chr20	63005979	63007035	-	1054	1	BHLHE23|	1.541	5.094
NR_03166	chr10	14436576	14436661	+	85	1	MIR1265|	0	0
NM_0153:	chr5	1.55E+08	1.55E+08	+	6574	1	LARP1|LAI	59.401	60.912
NR_02402	chr18	59035679	59053214	+	586	1	OACYLP|A	0.088	0
NR_02440	chr16	86331850	86345679	-	2611	1	LINC00917	0	0
NR_02678	chr3	1.98E+08	1.98E+08	+	6906	1	LMLN|GP6	1.419	0.827
NM_0807	chr14	46651016	46651825	-	807	1	RPL10L|RI	0	0.136
NR_13583	chr15	40906809	40909351	+	598	1	LOC10537(	1.509	1.351
NM_0013(	chr22	38861427	38872334	-	6104	1	CBX6|-|-|:	17.437	20.988
NM_0012٤	chr19	44954993	44993346	+	2464	1	CLPTM1|-	67.942	70.636
NM_0012!	chr15	82430783	82439386	+	4232	2	GOLGA6L9	0.165	0.155
NM_0183!	chrY	281394	303355	+	5295	2	PLCXD1|LI	0	0
NR_03792	chr15	55355223	55498584	-	4822	1	DYX1C1-C(	3.169	3.508
NM_0012:	chr11	1.12E+08	1.12E+08	+	1937	1	DIXDC1|C(	10.271	8.896
NR_13627	chr2	1.72E+08	1.72E+08	+	2946	1	METAP1D	2.018	2.892
NM_0054!	chr3	1.09E+08	1.09E+08	-	919	1	GUCA1C|G	0.098	0
NM_0251(	chr11	1.17E+08	1.17E+08	-	6182	1	SIK3|L19|(	13.886	12.895
NR_03058	chrX	1.46E+08	1.46E+08	-	78	1	MIR891A|	0	0
NM_0012:	chr4	1.22E+08	1.22E+08	-	2856	1	CCNA2|CC	4.271	3.467
NM_0032:	chr4	48135783	48269797	-	3600	1	TEC|PSCTI	0.054	0.137
NM_0017!	chr21	36069924	36073177	+	1316	1	CBR1|CBR	16.234	16.268
NM_0010:	chr17	77140923	77217099	+	2995	1	SEC14L1|F	47.833	40.478
NR_10677	chr16	2106669	2106753	-	84	3	MIR6511B	0	0
NR_13590	chr10	50739688	50757186	+	3172	1	ASAH2B|A	0.809	0.825
NR_00259	chr8	66922474	66922549	-	75	1	SNORD87|	0	0.913
NR_13232	chr16	3144291	3149946	+	1358	1	CASP16P|(	0.057	0.01
NM_0061:	chrX	1.49E+08	1.5E+08	-	1396	1	IDS|MPS2	50.604	46.9
NM_0012٤	chr10	1.22E+08	1.22E+08	-	4813	1	ATE1|-|-|:	6.936	5.931
NM_0032!	chr17	78174079	78187204	-	1586	1	TK1|TK2|-	4.471	2.565
NM_0010(	chr11	58422265	58423313	-	1046	1	OR5B2|OF	0	0
NM_0011!	chr17	35934519	35943710	-	949	2	LYZL6|HEL	0	0
NM_2074:	chr16	1088226	1096244	-	3399	1	C1QTNF8|	0	0.024
NM_0012!	chr12	7354961	7444185	-	4622	1	CD163L1|(	0.064	0.041

NR_10457	chr8	53880872	53959304	+	1563	1	RGS20|RG	4.611	3.943
NM_2015	chr8	1.43E+08	1.43E+08	-	2370	1	MAFA|RIP	0.245	0.705
NM_0809	chr12	1.09E+08	1.09E+08	+	2043	1	UNG|DGU	26.418	26.958
NM_0068	chr1	1.51E+08	1.51E+08	+	2154	1	MLLT11|A	530.159	522.146
NM_0010	chr1	1.61E+08	1.61E+08	-	2291	1	DEDD|CAS	28.505	31.436
NR_12596	chr1	2.34E+08	2.34E+08	-	573	1	LOC10192	1.98	2.343
NM_0002	chr7	1.28E+08	1.28E+08	+	3422	1	LEP|LEPD|	0	0.004
NM_0067	chr5	1.38E+08	1.38E+08	+	2260	1	MYOT|LGI	0	0.109
NM_0012	chr2	2.07E+08	2.07E+08	-	7947	1	KLF7|UKLF	21.049	19.747
NM_0221	chr5	65517766	65563168	-	1718	1	CENPK|AF	1.028	1.451
NR_03624	chr5	31936102	31936159	-	57	1	MIR4279|	0	0
NM_0010	chrX	35798342	35803735	+	1814	1	MAGEB16	0	0.015
NM_0209	chr4	1.19E+08	1.19E+08	+	3491	1	METTL14|	4.462	4.594
NM_0042	chr16	1989945	1994275	+	2045	1	SYNGR3|-|	33.164	29.249
NR_10692	chr17	40193597	40193663	+	66	1	MIR6867|	1.367	0.83
NR_02337	chr1	2.29E+08	2.29E+08	-	120	10	RNA5S14|	0	0
NM_1471	chr1	53506233	53734204	-	2804	1	GLIS1|-|-|	0.074	0.2
NM_0013	chr1	1.6E+08	1.6E+08	+	2967	1	SLAMF8|B	0.217	0.046
NM_0011	chr14	1.01E+08	1.01E+08	-	4190	1	RTL1|MAR	6.834	7.427
NM_0013	chr6_GL0C	3007752	3011942	+	1123.1	7	CSNK2B|C	6.222	4.012
NM_1735	chr6	41026901	41039199	-	3117	1	UNC5CL|N	0.749	0.62
NM_0053	chr8	56112942	56113982	-	1039	1	MOS|MSV	0	0
NR_02832	chr19	197016	202209	-	4871	1	LINC01002	0.003	0
NM_1984	chr11	7485369	7511375	+	2898	1	OLFML1|U	1.904	0.213
NM_0160	chr9	37779714	37785092	-	1835	1	EXOSC3|C	7.856	6.412
NR_10686	chr19	55230885	55230952	-	67	1	MIR6804|	1.155	2.044
NR_10344	chr3	9216895	9219508	+	1218	1	SRGAP3-A	0	0.011
NM_0022	chr12	9595274	9607901	-	732	1	KLRB1|CD	0	0
NM_0013	chr19	6396359	6412404	+	511	1	LOC39087	0	0
NM_1723	chr12	1791957	1918704	-	5303	1	CACNA2D	0.044	0.031
NM_0011	chr1	1.57E+08	1.57E+08	-	1513	1	SH2D2A|F	0	0
NM_0013	chr11	1.13E+08	1.13E+08	+	2309	1	TTC12|TP	2.049	1.358
NR_04856	chr12	1E+08	1E+08	+	1777	1	ACTR6|AR	8.46	10.706
NR_10462	chr9	1.28E+08	1.28E+08	-	2367	1	ST6GALNA	28.26	35.397
NM_0012	chr7	1.44E+08	1.44E+08	+	3108.5	2	CTAGE8|-|	0	0
NR_03170	chr4	36426366	36426428	-	62	1	MIR1255B	0	0
NR_04558	chr5	1.41E+08	1.41E+08	-	1463	1	SRA1|SRA	37.297	31.392
NM_0025	chr8	1.19E+08	1.19E+08	+	2594	1	NOV|CCN	0.91	1.79
NM_0012	chr1	2.01E+08	2.01E+08	+	7368	1	CAMSAP2|	9.168	14.508
NM_1991	chr9	68302867	68305084	+	2215	1	FOXD4L3|	0	0
NM_0021	chr19	35040506	35066573	+	1769	1	HPN|TMP	0.051	0.046
NR_02401	chr2	2.4E+08	2.4E+08	-	2015	1	PP14571|-	2.879	3.032
NM_0010	chr11	5199735	5200700	-	964	1	OR51V1|C	0	0
NM_0039	chr10	1.01E+08	1.02E+08	+	6040	1	BTRC|BET	12.296	17.137
NM_0183	chrX	16786432	16844519	+	4384	1	TXLNG|CX	3.462	4.636
NM_1985	chr15	89627970	89655451	-	4537	1	KIF7|ACLS	2.813	3.882
NR_00255	chr16	30204316	30206927	-	1473.5	2	LOC38824	0.061	0
NR_03385	chr12	1.03E+08	1.03E+08	-	3714	1	LINC0048	0	0
NR_03978	chr6	91521660	91521737	+	77	1	MIR4643|	0	0
NR_03411	chr6	1.47E+08	1.47E+08	-	3061	1	STXBP5-A	1.676	1.741

NM_0322(	chr16	57017074	57083524	+	6674	1	NLRC5|CLI	0.044	0.17
NM_0013(	chr1	1.98E+08	1.98E+08	-	2478	1	DENND1B	1.592	0.415
NM_0012;	chr8	1.1E+08	1.1E+08	+	2185	1	EBAG9|EB	7.842	8.839
NM_0144:	chr14	24305187	24311370	-	2233	1	CIDEB|-|-|	10.526	10.152
NR_04010	chr20	24199767	24224588	+	2372	1	LINC01721	0	0
NM_0330(	chr17	41084207	41085144	+	934	2	KRTAP4-7|	0	0
NM_0011:	chr1	1.52E+08	1.52E+08	-	3564	1	RPTN|-|-|	0	0
NM_1784:	chr1	1.53E+08	1.53E+08	+	286	1	LCE3B|LEP	0	0
NM_0011(	chr7	549197	727676	-	2410	1	PRKAR1B|	6.88	22.79
NM_0011(	chr5	36606355	36688334	+	4024	1	SLC1A3|EA	11.452	46.132
NR_02696	chr9	1.36E+08	1.36E+08	-	5021	1	DKFZP434A	0.018	0.095
NR_03370	chr1	3735984	3747373	-	4685	1	TP73-AS1|	10.059	9.642
NM_0057!	chr14	23636364	23645639	+	1665	1	DHRS2|HE	3.376	1.777
NM_0174:	chrX	1.3E+08	1.3E+08	-	3219	1	APLN|APE	0.697	0.234
NR_12637	chr2	1.72E+08	1.72E+08	+	945	1	DLX2-AS1|	0	0.029
NR_13527	chr14	90515806	90517209	-	305	1	LOC10537(	0	0
NR_11068	chr1	2.31E+08	2.31E+08	+	791	1	LOC10192	0.636	0.346
NR_03617	chr10	1.13E+08	1.13E+08	+	84	1	MIR4295|-	0	0
NR_13194	chr5	44495145	44510282	-	1096	1	BRCAT107	0	0.012
NR_12063	chr10	4051726	4089013	+	2283	1	LOC10192	0.006	0
NR_02676	chr8	29721260	29748109	-	1394	1	LINC0058S	0.102	0.039
NM_0038	chr17	32487087	32491253	+	3850	1	CDK5R1|C	85.502	125.265
NM_1342(	chr15	60488284	60592508	-	10657	1	RORA|NR1	0.364	0.999
NM_0010(	chr10	17315414	17454387	-	6783	1	ST8SIA6|S	0.422	0.02
NM_0012!	chr12	55973913	55996683	+	5291	1	RAB5B|-|-	64.48	64.571
NM_0015(	chr1	7915871	7943165	-	5982	1	TNFRSF9|4	0.183	0.032
NM_0010:	chr9	1.12E+08	1.12E+08	+	1250	1	GNG10|-|-	1.413	1.468
NM_1384:	chrX	1.03E+08	1.03E+08	+	3714	1	GPRASP2|	20.583	17.964
NM_0226	chr17	63872012	63873729	-	593	1	CSH2|CS-2	0	0
NR_10804	chr8	1.27E+08	1.27E+08	-	2793	1	CCAT1|CA	0	0.005
NR_10694	chr17	8144994	8145071	-	77	1	MIR6883|	0.67	0
NM_0012!	chr14	24429935	24441341	+	6157	1	KHNYN|KI	21.565	18.527
NR_02679	chr14	55781135	55796674	-	2004	1	LINC0052C	0.039	0
NM_0012;	chr17	43875359	43900633	-	4225	1	MPP2|DLG	50.457	42.864
NR_13277	chr17	37244801	37244975	+	174	2	SNORA90|	0	0
NM_0011!	chr5	1.48E+08	1.48E+08	+	599	1	SPINK6|BU	0.452	0
NR_03991	chr21	40212352	40212431	-	79	1	MIR4760|-	0	0
NM_0013:	chr3	1.13E+08	1.14E+08	-	5185	1	SPICE1|CC	1.907	1.783
NR_10680	chr11	1256605	1256670	+	65	1	MIR6744|	0	0
NM_0011(	chr1	2.32E+08	2.32E+08	+	6681	1	DISC1|C1c	2.547	2.782
NM_0011	chr9	42183659	42189887	+	4208	1	SPATA31A	0	0
NM_0224(	chr12	56020905	56038435	+	5441	1	IKZF4|EOS	7.505	4.551
NM_0011:	chr7	1.44E+08	1.44E+08	+	4875	1	TCAF2|FAI	0.098	0.098
NM_0012	chr6	1.44E+08	1.44E+08	-	3142	1	PLAGL1|LC	2.827	3.296
NM_0021;	chr9	21216373	21217311	-	936	1	IFNA16|IFI	0	0
NM_1538:	chr16	88453606	88535166	+	3229	1	ZFPM1|FC	6.365	7.423
NM_0013:	chr1	1.53E+08	1.53E+08	-	490	1	S100A8|6(	0	0
NM_1390(	chr12	56162359	56189574	-	4805	1	SMARCC2|	109.44	105.557
NM_0018	chr22	26599398	26618027	-	913	1	CRYBB1|C	0.367	0.045
NR_02403	chr4	52712394	52714138	+	912	1	DANCR|A(	24.147	37.712

NM_0011:	chr15	64963021	64989506	-	1640	1	SPG21|AC	56.055	61.536
NR_00272	chr11	3626460	3637559	+	1206	1	TRPC2|-|-	0	0
NM_1384!	chr8	1.44E+08	1.44E+08	-	1663	1	CYHR1|CH	27.407	26.356
NR_13656	chr22	18733914	18757894	+	1850	1	LINC01662	0	0
NM_0012!	chr17	37406886	37465653	+	1083	2	TADA2A|A	0.321	0.24
NM_0153!	chr22	43955381	43996532	+	1739	1	SAMM50|	37.123	38.074
NM_1524:	chr12	75391070	75424040	+	1762	1	GLIPR1L2|	0.212	0.132
NR_03030	chr5	96079138	96079212	+	74	1	MIR583|M	0	0
NM_2073(	chr13	38687036	38887130	+	16136	1	FREM2|-|-	0.446	1.169
NM_0006!	chr10	1.14E+08	1.14E+08	+	2859	1	ADRB1|AD	0.059	0.196
NM_1785!	chr14	1.02E+08	1.02E+08	+	1604	1	PPP2R5C|I	19.485	24.542
NR_13462	chr22	38570500	38571358	+	699	1	LOC10537.	0.037	0.078
NM_0005‹	chr12	1.21E+08	1.21E+08	+	3404	1	HNF1A|HN	1.087	0.889
NR_10674	chr1	22633258	22633366	-	108	1	MIR6127|I	0	0
NM_0010(	chrY	17768980	17770560	-	1578	2	XKRY2|XKI	0	0
NM_0018:	chr19	42580243	42594930	-	2295	1	CEACAM8	0	0
NM_0012(	chr1	2.34E+08	2.34E+08	+	724	1	COA6|C1o	11.255	8.438
NM_0011:	chr19	53138704	53159069	-	4227	1	ZNF347|ZI	1.455	2.479
NR_04665	chr3	42530896	42532606	-	178	1	VIPR1-AS1	0	0
NM_0140‹	chr16	14071313	14266779	+	8647	1	MKL2|MR	4.268	3.689
NM_0011(	chr11	1.19E+08	1.19E+08	+	2274	1	PDZD3|IKE	0.028	0.006
NR_11196	chr14	21521081	21526408	-	1827	1	SALL2|COI	61.151	56.797
NM_0177(	chr14	30559123	30620061	+	5785	1	G2E3|KIA/	1.656	2.05
NM_2135!	chr12	1.09E+08	1.09E+08	-	3340	1	FOXN4|-|-	0.22	0.332
NM_0324:	chr19	52397868	52418404	+	3969	1	ZNF528|-|	4.009	7.333
NM_0012‹	chr12	1.23E+08	1.23E+08	-	3328	1	ABCB9|ES	6.152	7.668
NM_0012!	chr9	85546539	85742029	-	4327	1	AGTPBP1|	11.702	11.795
NM_0159!	chr5	1.74E+08	1.74E+08	+	2445	1	HMP19|-|	346.99	579.812
NM_0124(	chr4	1.64E+08	1.64E+08	-	703	1	ANP32C|P	2.219	4.403
NR_00350	chr6	26871487	26956554	-	1191	1	GUSBP2|G	0.097	0.081
NM_0029:	chrX	1.54E+08	1.54E+08	-	1458	1	RENBP|RB	6.977	4.209
NM_0247!	chr9	1.38E+08	1.38E+08	+	5078	1	EHMT1|EL	27.6	27.289
NR_05199	chr13	60013303	60044356	+	336	1	DIAPH3-AS	0.384	0.285
NM_0010(	chr21	25724480	25735654	-	656	1	ATP5J|ATF	162.171	155.72
NR_02966	chr9	95085445	95085541	+	96	1	MIR27B|M	0	0.143
NR_11005	chr1	44222595	44244283	-	494	1	ERI3-IT1|-	1.436	0.915
NM_0012!	chr6	44387514	44450424	+	6414	1	CDC5L|CD	7.693	9.249
NM_1825(	chr13	42781550	42791549	+	3296	1	FAM216B|	0.012	0.104
NM_0013:	chr16	8892094	8956380	-	5174	1	USP7|HAU	25.124	27.778
NR_02442	chr5	6583276	6588500	+	3050	1	LINC01018	0.072	0.108
NR_03322	chr16	69118009	69132590	-	2863	1	CHTF8|CTI	46.372	46.732
NR_13389	chr12	1.09E+08	1.09E+08	-	3988	1	KCTD10|B	32.505	28.957
NM_0212:	chr7	44138864	44141317	-	588	1	MYL7|MYI	0.724	0
NM_0043:	chr8	18056299	18084998	-	2652	1	ASAH1|AC	28.179	30.219
NR_13617	chr2	1.74E+08	1.74E+08	+	1877	1	LINC0196C	0.062	0
NM_0010:	chr6	41546426	41602384	+	5930	1	FOXP4|hFI	17.085	23.001
NM_1944(	chrX	1.07E+08	1.07E+08	+	2831	1	RNF128|G	0	0.01
NR_04855	chr1	2.18E+08	2.18E+08	+	1580	1	LINC0021C	0	0
NM_0148:	chr1	1.8E+08	1.8E+08	+	13416	1	CEP350|C/	1.527	2.748
NM_0012(	chr2	1.98E+08	1.98E+08	+	3719	1	MOB4|2C‹	9.787	10.835

NR_04637	chr4	73508803	73528533	+	759	1	LOC72804	0	0
NM_0157	chr8	1.02E+08	1.02E+08	-	4921	1	RRM2B|M	3.776	4.771
NM_0010	chr1	1.68E+08	1.68E+08	+	930	1	TIPRL|TIP|	18.744	22.49
NR_13234	chr12	91984976	92142831	-	2287	1	LINC0161	0.778	1.03
NM_0041	chr8	27322402	27459391	+	4131	1	PTK2B|CA	4.46	5.61
NR_02792	chr3	15165362	15205959	-	2244	1	COL6A4P1	0.011	0.024
NM_0011	chr14	54396955	54420216	+	762	1	CDKN3|CD	4.89	4.566
NR_13513	chr9	88066436	88077900	-	522	1	LOC10272	0.272	0.052
NM_0011	chr4	6269850	6303265	+	3626	1	WFS1|CTR	8.57	9.893
NM_0010	chr3	41199451	41240448	+	3397	1	CTNNB1|C	174.443	172.255
NR_10433	chr10	8052243	8053422	-	728	1	GATA3-AS	1.24	3.217
NR_00392	chr5	22142352	22152270	+	569	1	PMCHL1|-	0	0
NR_00324	chr20	63095493	63102319	-	894	1	HAR1B|HA	0.101	0.199
NM_0012	chr12	1.21E+08	1.21E+08	-	4488	1	CAMKK2|C	29.912	34.096
NM_0121	chr1	2.02E+08	2.02E+08	-	3962	1	LMOD1|1	0.267	0.674
NR_02713	chr1	15111815	15152464	-	6974	1	TMEM51-A	7.968	11.323
NR_03627	chr7	56955785	56955864	-	79	2	MIR4283-2	0	0
NR_03388	chr2	779840	868426	-	1362.5	2	LINC0111	0	0
NR_03994	chr2	1.89E+08	1.89E+08	-	67	1	MIR1245B	0	0
NM_0217	chr19	49739754	49763258	-	1799	1	TSKS|PPP1	0.036	0.015
NR_12590	chr4	33896341	34039893	-	2397	1	LOC10192	0	0.006
NR_03032	chr7	32732981	32733077	+	96	1	MIR550A2	0	0
NR_12062	chr10	1.15E+08	1.15E+08	+	2375	1	LOC10192	0.016	0.017
NM_0013	chr8	49911673	50709183	+	2131	1	SNTG1|G1	0.781	1.935
NM_0011	chr19	7846500	7864976	+	3714	1	EVI5L|-|-|	35.195	44.37
NM_0010	chrX	1.51E+08	1.51E+08	+	4731	1	VMA21|M	8.604	9.071
NM_0321	chr5	1.35E+08	1.35E+08	+	2372	1	PCBD2|DC	3.12	1.727
NM_0010	chr1	1.62E+08	1.62E+08	+	2092	1	FCGR2B|C	0	0
NR_02687	chr3	1.93E+08	1.93E+08	+	2193	1	MGC2889	0.341	0.319
NR_04004	chr19	35405607	35416840	+	3307	1	LINC01531	0	0
NM_0246	chr1	52726459	52827341	+	8126	1	ZYG11B|Z	6.275	8.433
NM_0219	chr6_GL0	3876736	3891707	-	1182.5	2	HLA-DRB4	0	0
NR_13366	chr3	1.91E+08	1.91E+08	-	505	1	OSTN-AS1	0.128	0.108
NR_03983	chr11	46376402	46376484	+	82	1	MIR4688|	0	0
NM_1825	chrX	1.36E+08	1.36E+08	+	3829	1	INTS6L|DD	2.482	2.651
NM_1825	chr2	11133053	11146790	+	1050	1	C2orf50|-|	0.81	0.339
NM_0012	chr22	46335401	46357340	+	1704	1	TRMU|LC	13.59	12.821
NM_0070	chr8	79610814	79666175	+	2216	1	STMN2|SC	1021.314	895.498
NM_0047	chr14	49783521	49853203	-	4761	1	NEMF|NY-	2.486	4.275
NM_0184	chr5	1.8E+08	1.8E+08	-	2252	1	RNF130|G	32.176	29.955
NM_0020	chr8	1.43E+08	1.43E+08	+	717	1	GML|LY6D	0	0
NM_0124	chr4	1.66E+08	1.66E+08	+	7247	1	TLL1|ASD	0.192	0.112
NM_0203	chr9	19507452	19787019	-	10828	1	SLC24A2|	0.542	1.211
NR_02700	chr19	46210242	46214837	+	1628	1	LOC93429	0.04	0
NM_1529	chr15	89088150	89202360	+	8722	1	ABHD2|HS	12.555	16.711
NM_0012	chr5	10353639	10440388	+	9526	1	MARCH6|	32.667	34.809
NR_00392	chr13	50994511	51066157	-	3005	1	GUCY1B2|	0	0.228
NR_00309	chr11	62851988	62855888	-	1107	1	SNHG1|LI	72.504	88.134
NM_0011	chr1	86914652	87098442	+	1571	1	HS2ST1|dJ	4.063	5.144
NR_10419	chr6_GL0	3276510	3283759	-	759	7	C2-AS1|-|-	0	0

NM_0011	chr8	22161671	22164478	+	967	1	SFTPC|BRI	0	0.028
NM_0011	chr22	17834336	17906886	-	3067	1	MICAL3|M	17.926	23.468
NM_1452	chr5	204760	218182	-	1415	1	CCDC127|	25.167	28.178
NR_03884	chr17	38925168	38929384	+	3690	1	LINC00672	0.604	1.225
NM_0010	chr12	52069974	52077495	+	1260	1	ATG101|C	30.955	39.221
NR_13808	chr15	93896060	93900599	-	788	1	LINC0157	0	0
NM_0010	chr17	28905253	28951490	-	4458	1	PHF12|PF	23.097	23.01
NM_0044	chr5	1.78E+08	1.78E+08	+	1666	1	HNRNPAB	99.116	106.14
NM_0031	chr20	61974798	62065810	-	3673	2	TAF4|TAF	1.022	1.078
NR_03896	chr6	11043758	11079144	+	1612	1	ELOVL2-AS	0.504	0.782
NM_0012	chr7	1.29E+08	1.29E+08	+	5347	1	CALU|-|-|	30.004	28.498
NR_14452	chr22	26512532	26514567	+	1657	1	LOC10050	6.49	5.646
NR_00152	chrY	22144965	22146831	+	489	2	TTTY6|LIN	0	0
NM_0011	chr1	1.65E+08	1.65E+08	-	3345	1	LMX1A|LM	0.328	0.102
NR_10695	chr6_GL0	2610911	2611014	-	84.3	7	MIR6891|	0	0
NM_0184	chrX	1.03E+08	1.03E+08	-	857	1	BEX1|BEX	388.805	407.394
NR_12640	chr2	34677557	34722563	+	1128	1	LINC0132	0	0.049
NR_03019	chr19	53702737	53702819	+	82	1	MIR518B|	0	0
NM_0037	chr13	1.1E+08	1.1E+08	-	6994	1	IRS2|IRS-2	3.215	6.183
NM_0054	chr4	69841212	69860152	-	1770	1	SULT1E1|	0.044	0.046
NM_0122	chrX	37990817	37991317	+	498	1	HYPM|CX	0	0
NM_0050	chr10	1.02E+08	1.02E+08	-	1382	1	PITX3|ASN	0.009	0.02
NM_0159	chr17	7288252	7294557	-	1572	1	YBX2|CON	8.858	17.147
NM_0011	chr5	6599239	6633360	-	3247	1	NSUN2|M	14.919	15.207
NR_02680	chr17	18524566	18526846	-	2280	1	FAM106A|	0	0
NM_0011	chr12	25496316	25648562	-	2480	1	LMNTD1|	0.052	0.044
NR_04001	chr19	37823723	37855196	-	1709	1	LOC10063	0.566	0.088
NM_0330	chr22	39223359	39240909	-	2692	1	PDGFB|IB	2.62	3.221
NM_0012	chr18	24162046	24311506	-	2929	1	OSBPL1A|	5.102	4.69
NR_02699	chr3	1.29E+08	1.29E+08	+	2188	1	H1FX-AS1|	1.903	1.002
NR_02654	chrX	23833348	23907940	-	964	1	APOO|FAM	23.821	26.442
NM_0201	chr2	1.28E+08	1.28E+08	+	10736	1	UGGT1|HU	9.191	10.168
NM_0048	chr5	78360503	78480739	+	6246	1	SCAMP1|S	6.511	8.533
NM_0210	chr6	27133038	27133535	+	495	1	HIST1H2A	0.834	0.664
NM_0011	chr18	50795120	50825384	-	4880	1	MRO|B29	0.132	0.211
NM_0013	chr17	46923117	46967000	+	1117	1	GOSR2|Bo	22.301	24.132
NR_03052	chr14	1.01E+08	1.01E+08	+	97	1	MIR770|M	0	0
NM_0159	chr12	12974865	13000309	-	1202	1	HEBP1|HB	12.014	12.865
NM_0036	chr16	29992321	29996096	-	3039	1	HIRIP3|-|-	15.061	12.931
NR_03888	chr9	90020654	90041499	-	2716	1	MIR4290H	0	0
NR_04666	chr2	1.67E+08	1.67E+08	-	1869	1	XIRP2-AS1	0	0
NR_03653	chr10	22435425	22437929	-	2504	1	LOC10049	4.181	3.052
NM_0065	chr3	1.9E+08	1.9E+08	+	3266	1	CLDN16|H	0.008	0.046
NM_0000	chr1	56929210	56966140	-	2148	1	C8B|C82|-	0	0
NM_0012	chr19	36605305	36628597	+	2795	1	ZNF382|K	2.883	3.205
NR_02960	chr19	4770670	4770779	+	109	1	MIR7-3|M	0	0
NR_03967	chr7	1.02E+08	1.02E+08	+	62	1	MIR4467|	0	1.767
NR_03410	chr11	1.13E+08	1.13E+08	-	710	1	NCAM1-AS	85.042	57.219
NR_02695	chr3	1.29E+08	1.29E+08	+	2751	1	LOC90246	0.211	0.055
NM_0020	chr16	58707131	58734357	-	2450	1	GOT2|KAT	82.281	103.918

NM_0003(	chr1	1.61E+08	1.61E+08	+	1692	1	PPOX|PPC	14.867	12.11
NM_0006;	chr4	99070979	99088788	-	2640	1	ADH5|ADH	48.531	45.755
NM_0145;	chr22	42796526	42857402	-	2815	1	ARFGAP3|	14.602	14.855
NM_0012;	chr19	45162928	45178243	-	736	1	TRAPPC6A	17.781	12.171
NM_0203;	chr1	25617468	25786207	+	4627	1	MAN1C1|I	10.508	6.838
NR_00331	chr15	25209918	25209999	+	81	1	SNORD115	0	0
NM_0011(	chr5	1.38E+08	1.38E+08	-	5357	1	FAM13B|A	8.061	13.04
NM_0013:	chr9	1.26E+08	1.26E+08	+	2751	1	PBX3|-|-|S	124.084	120.629
NM_0146‹	chr18	2916994	3011947	-	6207	1	LPIN2|-|-|	7.449	8.136
NM_0056!	chr3	1.2E+08	1.2E+08	-	418	1	COX17|-|-	36.675	32.506
NM_0034‹	chr4	337807	373902	+	1636	1	ZNF141|D	0.67	1.03
NM_0031‹	chr11	4384897	4393696	-	1920	1	TRIM21|R	1.679	2.768
NR_03663	chr2	63117851	63119542	-	1691	1	DBIL5P2|E	0.023	0.032
NM_0182:	chr1	2.25E+08	2.26E+08	-	13095	1	ENAH|ENA	8.326	9.761
NM_0807;	chr20	646624	653370	-	2685	1	SRXN1|C2	9.815	11.391
NR_13064	chr22_KI2	120081	144844	-	2198	2	ZNF280B|!	0	0
NM_0012;	chr22	24555650	24574964	+	3788	1	SNRPD3|S	33.248	34.167
NM_1332!	chr2	43886224	43996005	-	6579	1	LRPPRC|CI	13.185	14.018
NM_0004!	chr15	74719542	74725610	-	2673	1	CYP1A1|A	0.666	1.245
NM_0011;	chrX	1.36E+08	1.36E+08	-	4429	1	MAP7D3|I	0.652	0.634
NR_12065	chr10	76888044	76978593	+	800	1	KCNMA1-A	0	0.017
NM_0013:	chr11	66011991	66013505	+	604	1	CST6|-|-|1	0	0
NM_0243:	chr16	54283300	54286466	-	2302	1	IRX3|IRX-1	17.974	18.106
NM_0010{	chr17	81239311	81241298	+	555	1	NDUFAF8|	19.375	14.684
NR_00282	chr2	1.31E+08	1.31E+08	-	2733	1	NOC2LP2|	1.816	1.824
NM_0031;	chr10	29457348	29735801	-	6678	1	SVIL|-|-|1	3.858	6.986
NR_10699	chr2	2.07E+08	2.07E+08	+	98	1	MIR7845|I	0.921	0.14
NM_0073‹	chr15	50908672	51005900	+	6736	1	AP4E1|CP!	1.059	1.322
NR_04007	chr18	51392042	51468378	+	3186	1	LINC0163C	0.138	0.15
NM_0063(	chrX	53374149	53422728	-	9757	1	SMC1A|CD	18.319	12.869
NM_0013:	chr1	47004240	47050754	+	2049	1	CYP4X1|C	0.019	0.16
NM_0227;	chr11	706120	727727	+	3118	1	EPS8L2|EP	1.451	1.814
NR_11046	chr2	1.76E+08	1.76E+08	-	3779	1	HAGLR|HC	0	0.011
NM_0181;	chr17	47941523	47949308	+	3456	1	PNPO|HEL	2.343	5.933
NR_02952	chrX	1.34E+08	1.34E+08	-	80	1	MIR106A|	0	0
NM_1736;	chr6	1.17E+08	1.18E+08	+	2014	1	DCBLD1|d	2.446	2.7
NR_11093	chr16	76634998	76658478	+	2194	1	LINC02125	0	0
NM_1825{	chr2	2.1E+08	2.1E+08	+	13425	1	UNC80|C2	0.859	1.142
NM_0030‹	chr2	1.03E+08	1.03E+08	+	5601	1	SLC9A2|NI	0.009	0.032
NM_0149!	chr14	59263441	59371405	+	5793	1	DAAM1|-|	15.843	13.458
NR_00294	chr1	1632780	1634651	+	900	2	MMP23A|	0	0
NM_0209(	chr18	45847609	45967339	-	12652	1	EPG5|HEE	3.616	4.14
NM_0062:	chr17	1517989	1562816	-	3627	1	PITPNA|HI	57.635	63.874
NM_0011‹	chr19	40072992	40090938	-	3593	1	ZNF780A|;	1.36	1.281
NM_0207:	chrX	1.19E+08	1.19E+08	-	7809	1	KIAA1210|	0.003	0.002
NM_0010;	chr14	21209136	21269479	-	3216	1	HNRNPC|(	106.78	130.692
NM_0010:	chr11	33141431	33161491	-	622	1	CSTF3|CST	26.388	32.327
NM_0010:	chr1	1324763	1328896	+	2188	1	CPTP|GLTI	16.694	19.162
NR_03037	chr19	45674928	45675024	+	96	1	MIR642A|	0	0
NR_12637	chr13	1.03E+08	1.03E+08	+	490	1	LINC0130S	0	0

NM_0229:	chr12	1.22E+08	1.22E+08	-	2588	1	VPS33A|-|	17.058	14.692
NM_0011	chr2	1.74E+08	1.74E+08	+	1452	1	SP9|ZNF9	0.426	5.405
NR_00157	chr8	11339637	11368452	+	1348	1	TDH|SDR1	0.124	0.264
NM_0013(	chr12	52360006	52364622	-	1928	1	KRT85|EC	0	0
NM_0010:	chr10	88586762	88606976	+	1471	1	LIPJ|LIPL1	0	0
NM_0246	chr11	66510648	66533613	+	3351	1	BBS1|BBS	19.792	13.023
NR_12580	chr5	1.74E+08	1.75E+08	+	1073	1	LINC01411	0	0.013
NM_0011!	chr19	56404349	56425031	+	2536	1	ZNF583|-|	4.194	4.105
NM_0190!	chr9	1.01E+08	1.01E+08	-	1017	1	MRPL50|N	18.801	19.071
NM_1307	chr5	1.77E+08	1.77E+08	-	1524	1	RAB24|-|-	27.259	31.465
NM_0013	chr10	38094336	38115692	+	895	1	ZNF37A|K	1.772	2.081
NM_0011	chr16	15594369	15643166	-	7722	2	KIAA0430|	0	0
NM_1536:	chr15	34358888	34367194	-	1892	1	LPCAT4|A	29.215	29.023
NM_0122!	chr11	78215290	78341880	-	6047	1	GAB2|-|-|	15.665	29.333
NR_12212	chr7	27615974	27627492	+	1090	1	TSL|-|-|7p	8.233	0.691
NR_04755	chr11	4187152	4202653	+	446	1	LOC10050	0.173	0.092
NR_03395	chr12	1.21E+08	1.21E+08	+	3769	1	P2RX7|P2	0.164	0.309
NR_10237	chr2	2.27E+08	2.27E+08	-	2488	1	LOC65484	0.223	0.479
NM_0011:	chr19	3522956	3536757	+	3431	1	FZR1|CDC	31.003	34.588
NM_0012(	chr2	27370496	27377533	+	2293	1	SNX17|-|-	56.145	49.752
NM_0013	chr11	10750987	10779755	+	4500	1	CTR9|SH2	11.868	10.595
NM_0073!	chr4	89894901	89954629	+	4959	1	MMRN1|E	6.053	0.378
NM_0012	chr17	48723168	48724758	+	573	1	PRAC2|C1	0.09	0
NM_0012(	chr7	74093156	74122525	+	3143	1	LIMK1|LIM	56.095	54.592
NM_0121(	chr16	57245126	57253633	+	2092	1	ARL2BP|B	49.09	48.516
NR_03981	chr9	1.34E+08	1.34E+08	+	61	1	MIR4669|	0	0
NM_0050:	chr12	53452102	53481162	+	3154	1	PCBP2|HN	185.79	179.286
NR_02947	chr9	94175957	94176036	+	79	1	MIRLET7A	0	0
NM_0013:	chr13	77661374	77662803	-	1427	1	LOC10012	0	0
NM_0181:	chr1	1.74E+08	1.74E+08	+	3329	1	DARS2|AS	6.104	3.991
NM_0011	chr1	1.52E+08	1.52E+08	+	448	1	RIIAD1|C1	16.836	11.587
NM_0144!	chr9	35649301	35650950	-	1228	1	SIT1|SIT|S	0.189	0.435
NM_0013:	chr2	1.91E+08	1.91E+08	+	3904	1	NAB1|-|-|	4.957	11.157
NR_00393	chr12	12875477	12876136	+	659	1	RPL13AP2	0.607	0.457
NM_0013:	chr19	11164267	11195820	-	5177	1	KANK2|AN	2.169	2.257
NM_0011!	chr8	22599601	22604149	+	1946	1	C8orf58|-|	14.762	13.31
NM_0012	chr17	42900797	42914433	+	4077	1	G6PC|G6P	0	0
NM_0011!	chr3	1.8E+08	1.8E+08	+	945	1	NDUFB5|C	53.225	44.802
NM_0208	chr4	48341596	48426198	+	6001	1	SLAIN2|KI	6.721	8.123
NM_0010	chr9	1.35E+08	1.35E+08	-	717	1	C9orf116|	8.901	6.247
NR_02684	chr9	97390837	97396691	-	2011	1	LOC28635	0	0
NM_0011:	chr2	2.1E+08	2.11E+08	+	5684	1	CPS1|CPS	7.168	8.702
NM_0333	chr22	37906148	37942458	+	4477	1	MICALL1|	17.942	16.037
NM_0012	chr11	74840910	74949187	-	4617	1	XRRA1|-|-	4.583	4.616
NM_0323:	chr17	39728500	39730563	-	1432	1	MIEN1|C1	37.816	39.637
NM_0056:	chr2	38981549	39120463	-	8293	1	SOS1|GF1	3.759	6.215
NM_0011(	chr9	88535101	88544547	+	802	1	NXNL2|C9	0.177	0.102
NM_0012!	chr17	5486374	5490810	+	2031	1	MIS12|25	9.922	10.44
NM_0228(	chr5	1.38E+08	1.38E+08	-	1938	1	CDC25C|C	1.783	1.548
NM_0011(	chr15	98968230	99005656	-	1132	1	PGPEP1L|-	0.046	0.036

NM_0051:	chr11	66843412	66846532	+	1461	1	RCE1|FACI	11.49	9.281
NM_0007:	chr11	1.18E+08	1.18E+08	+	1502	1	CD3E|IMD	0	0
NM_1825:	chr6	44280160	44297721	-	1676	1	TCTE1|D6S	1.439	1.569
NR_13449	chr10	14972564	14988047	+	533	1	LOC10537	0.677	0.257
NR_14532	chr19	57746796	57758161	+	3215	1	ZNF776|-|	10.82	12.342
NR_00350	chr7	32727950	32729983	+	2033	1	ZNRF2P1|-	0.412	0.33
NM_0011!	chr5	1.78E+08	1.78E+08	-	2979	1	FAM193B|	27.124	32.613
NM_0022:	chr12	4809176	4851112	+	5974	1	KCNA6|HE	8.931	15.664
NM_0058(	chr21	33025908	33029195	+	2510	1	OLIG2|BHI	5.471	4.3
NM_0010/	chr3	1.39E+08	1.39E+08	+	4070	1	FOXL2NB|	0.013	0.007
NM_0013:	chr8	21908873	21913708	-	1409	1	DOK2|p56	0.009	0.01
NM_0011/	chr18	79902420	79951653	-	2483	1	PQLC1|-|-	27.298	31.278
NM_0013(	chr17	2303943	2325264	+	2312	1	SRR|ILV1|	32.786	36.168
NM_0010{	chr11	1.25E+08	1.25E+08	-	4029	1	TMEM218	8.442	7.931
NM_0010:	chr6	28432655	28443502	-	3155	1	ZSCAN23|:	2.052	1.476
NM_0010{	chr16	84009667	84042157	-	1414	1	SLC38A8|F	0.41	0.184
NR_03031	chr5	1.49E+08	1.49E+08	-	96	1	MIR584|M	0	0
NR_12053	chr11	70056230	70063924	-	546	1	LOC10192:	0	0
NM_1813:	chr6	1.37E+08	1.37E+08	-	2624	1	IL22RA2|C	0	0
NM_0013(	chr15	65611405	65660918	+	3892	1	SLC24A1|(	0.775	0.69
NM_0528;	chr6	52236686	52244500	-	803	1	IL17F|CAN	0	0
NR_13547	chr10	49942033	49988221	+	1856	1	TIMM23B|	1.869	2.192
NM_0020(	chr15	49423178	49487326	+	3930	1	FGF7|HBG	0	0.014
NR_04641	chr8	12379267	12379789	-	522	1	LOC64935:	0	0.026
NM_0043{	chr5	1.73E+08	1.73E+08	-	1665	1	NKX2-5|CH	0.077	0.337
NM_0071/	chr17	19411178	19417276	+	3212	1	RNF112|B	3.296	9.104
NR_11037	chr10	17641284	17643918	-	2634	1	STAM-AS1	0.264	0.406
NM_0013:	chr3	38039205	38122741	+	5588	1	DLEC1|CF/	0.102	0.076
NR_10987	chr20	12950340	12951627	+	597	1	LINC01723	0	0
NM_0059(	chr12	57088894	57095476	+	2702	1	NAB2|MA	49.932	50.89
NM_0312(	chr16	2537964	2603190	+	6849	1	PDPK1|PD	10.879	11.743
NR_13579	chr1	1.69E+08	1.69E+08	+	565	1	LOC10192:	0	0
NM_1759(	chr18	75195110	75209348	-	7726	1	ZADH2|PR	2.717	4.554
NM_0150{	chr12	48995150	48999305	-	3740	1	DDN|-|-|1	3.537	2.538
NM_0010/	chr2	1.78E+08	1.78E+08	+	1515	1	DFNB59|P	0.783	0.949
NM_0004(	chr3	24117154	24494822	-	7389	1	THRB|C-EF	0.4	0.415
NM_0010{	chr19	34481975	34501180	+	2194	1	WTIP|-|-|:	2.098	2.222
NR_10380	chr12	1.17E+08	1.17E+08	-	4194	1	LOC10050(	0.289	0.284
NR_02676	chr1	45645781	45646685	-	904	1	RPS15AP1(	0.043	0.106
NM_0124:	chr1	1.14E+08	1.14E+08	-	3468	1	PTPN22|L\	0.03	0.028
NR_02449	chr10	38428146	38452153	+	5245	1	LINC00999	0.005	0.624
NR_03974	chr16	30875266	30875323	-	57	1	MIR4519|-	1.357	0.24
NM_0012!	chr6	14117256	14136917	+	2299	1	CD83|BL1:	5.889	13.351
NM_0038{	chr3	20040032	20154404	+	4804	1	KAT2B|CA	6.994	2.726
NR_02962	chr1	1.99E+08	1.99E+08	-	109	1	MIR181A1	0	0
NM_0011!	chr8	1.44E+08	1.44E+08	-	1184	2	EEF1D|EF-	0	0
NM_0012/	chr9	33524413	33573003	+	3735	1	ANKRD18E	0.007	0.004
NM_0011(	chr20	20056646	20172333	+	1931	1	CFAP61|C:	1.249	1.39
NM_0134/	chr7	1.4E+08	1.4E+08	-	3141	1	MKRN1|RI	71.224	86.521
NM_0019:	chr2	1.62E+08	1.62E+08	-	3872	1	DPP4|ADA	0.087	0.516

NM_0224{	chr15	85759326	85794958	-	3678	1	KLHL25|EN	27.263	38.399
NM_0013(	chr8	1823976	1958641	+	5655	2	ARHGEF10	0	0
NR_00324	chr15	84506168	84516847	-	5461	1	GOLGA6L5	0.104	0.033
NM_0011(	chr19	48968125	48993353	-	3409	1	GYS1|GSY	28.688	18.028
NM_0043:	chr11	3645131	3664416	+	1305	1	ART1|ART:	0	0.01
NR_03748	chr1	25906362	25906454	-	92	1	MIR3917|r	1.822	0.596
NM_0121:	chr9	87966444	87974780	-	2321	1	CDK20|CC	15.771	14.647
NM_0182!	chr7	1.35E+08	1.35E+08	+	2051	1	TMEM140	7.255	6.999
NR_12595	chr1	99472420	99487804	-	411	1	LINC01708	0.031	0
NM_0017(	chr19	827826	832018	+	911	2	AZU1|AZA	0	0
NR_02723	chrX_KI27(	1	1982	+	2109.7	3	LINC00685	0.257	0.104
NM_0011(	chr2	95274448	95291307	+	4711	1	PROM2|PI	0.744	0.651
NR_11079	chr18	44323435	44531697	-	2064	1	LINC01478	0	0.013
NR_04690	chr12	31589923	31615351	+	561	1	DENND5B-	0.414	0.293
NM_0011!	chr10	96000091	96032684	+	2100	1	CC2D2B|C	0	0.007
NM_0151:	chr1	66999332	67054397	-	6446	1	SLC35D1|U	2.024	3.039
NM_0010:	chr6	1.55E+08	1.55E+08	+	2653	1	TIAM2|STI	4.413	13.408
NM_0011:	chr10	95907965	95938658	+	1166	1	C10orf131	0.133	0.047
NM_0022;	chr17	41459513	41467386	-	1996	1	KRT32|HA	0	0
NR_02655	chrX	15674916	15703351	+	989	1	CA5BP1|C/	3.155	2.534
NR_04989	chr18	12200779	12224710	+	2708	1	C18orf61|	0	0
NM_1392(	chr2	1.91E+08	1.91E+08	-	2773	1	STAT1|CAI	5.905	9.133
NR_10685	chr19	49791866	49791934	+	68	1	MIR6799|I	9.481	8.258
NM_0012!	chr6	31652410	31658210	+	708	7	APOM|G3	0	0
NM_0010(	chr21	33642478	33838498	+	5409	1	ITSN1|ITSI	13.449	11.864
NM_0012{	chr16	21597221	21657473	+	2906	1	METTL9|C	68.385	72.35
NM_0013(	chr5	1.39E+08	1.39E+08	-	1772	1	TMEM173	0.065	0
NR_03060	chr1	1.54E+08	1.54E+08	-	78	1	MIR190B|	0	0
NM_0028:	chr12	6766317	6770952	+	1201	1	PTMS|Para	659.854	676.503
NM_0142:	chr6	33204637	33206831	+	984	6	HSD17B8|	0	0
NM_0013(	chr19	10086119	10093252	+	2017	1	C19orf66|	7	13.941
NM_0160<	chr14	24138965	24141588	-	827	1	EMC9|C14	21.203	18.285
NR_03613	chr15	92904399	92904475	+	76	1	MIR3175|r	0	0
NM_0307!	chr11	18245228	18248674	-	588	1	SAA2|-|-|:	0	0.14
NR_11083	chr17	76799044	76807102	+	3082	1	LINC0208C	0.008	0.022
NR_02149	chr15	92567818	92572263	-	1758	1	LINC0093C	0.073	0.07
NR_03841	chr17	58337202	58353727	+	2447	1	TSPOAP1-/	9.037	7.769
NR_03620	chr19	2250639	2250718	+	79	1	MIR4321|-	23.665	13.87
NR_11080	chr17	50214694	50215420	+	452	1	LINC01969	0	0
NM_0049<	chr5	1.7E+08	1.7E+08	+	6043	1	DOCK2|IM	0.132	0.233
NM_0011:	chr4	1.1E+08	1.1E+08	-	3182	1	ELOVL6|F/	6.341	6.005
NM_0058:	chr12	55743280	55753370	+	4744	1	GDF11|BN	25.695	22.148
NM_0001(	chr16	88643289	88651084	-	715	1	CYBA|p22-	0.811	0.479
NM_0012{	chr19	44212528	44237268	+	2901	1	ZNF227|-|	5.293	7.347
NM_0146:	chr5	1.76E+08	1.77E+08	+	4502	1	FAF2|ETE/	22.43	23.993
NM_0012;	chr14	96363452	96387290	+	2139	1	GSKIP|C14	7.113	5.398
NR_13658	chr10	48306639	48439359	+	5835	1	MAPK8|JN	28.136	33.962
NR_03894	chr2	85537467	85538886	-	1419	1	PARTICL|P	5.306	8.118
NM_0184(	chr10	58269102	58289259	+	2103	1	CISD1|C1C	9.994	12.206
NR_02762	chr19	34576733	34677031	-	2041	1	SCGB1B2P	5.155	6.838

NR_04566	chr10	68106117	68212016	+	4931	1	MYPN|CM	0.016	0.011
NR_13743	chr12	9240003	9243049	+	2056	1	LINC00987	0.546	0.5
NM_0012!	chr11	1.26E+08	1.26E+08	+	1714	1	ST3GAL4|(	10.306	18.212
NM_0177:	chr11	60492778	60507428	+	1156	1	MS4A12|N	0	0
NR_03982	chr10	1.11E+08	1.11E+08	+	65	1	MIR4680|-	4.959	5.057
NM_0000:	chr2	1.75E+08	1.75E+08	-	1970	1	CHRNA1|A	0.877	0.16
NR_03329	chr1	45493926	45500079	-	2079	1	CCDC163|	3.095	3.307
NM_0011(	chr9	1.36E+08	1.36E+08	-	1403	1	SOHLH1|C	0	0
NM_0028(	chr11	47418769	47426473	-	1566	1	PSMC3|TB	99.303	87.308
NM_1447(	chr2	60940413	61018259	-	3819	1	PUS10|CC	0.594	0.473
NM_0011(	chr7	1.21E+08	1.21E+08	+	2808	1	CPED1|C7	0	0
NM_0012:	chr20	36236417	36256941	+	2480	1	AAR2|C20	30.508	36.524
NM_0010(	chr11	6785017	6786014	+	995	1	OR2AG1|C	0	0
NM_0333:	chr19	49031873	49033238	+	727	1	CGB2|-|-|	0	0
NM_0010(	chr11	5131692	5132642	-	949	1	OR52A5|C	0	0
NM_0011(	chr11	18396266	18408218	+	2295	1	LDHA|GSD	29.81	25.037
NR_02679	chr6	26521706	26527384	+	5678	1	HCG11|CT	0.014	0.017
NM_1827(	chr19	42754233	42765679	-	1434	1	PSG8|PSG	0	0
NR_02454	chr6	31711976	31714065	-	568	7	LY6G6E|C(	0	0
NM_0149(	chr12	77831289	78213012	+	9719	1	NAV3|PON	6.515	6.939
NR_03382	chr4	7030554	7046231	-	4296	1	LOC10012!	29.524	42.003
NR_03988	chr17	31094350	31094425	-	75	1	MIR4733|-	0	0
NR_13118	chr10	3318695	3750763	+	2236	1	LOC10537(	0	0
NM_0011:	chrX	11111286	11123084	+	2252	1	HCCS|CCH	10.569	8.983
NR_13507	chr2	1.08E+08	1.08E+08	-	385	1	LINC01593	0	0
NR_00330	chr15	25194921	25195001	+	80	1	SNORD115	0	0
NM_0223(	chr14	20768427	20770948	+	892	1	EDDM3B|I	0	0
NM_0065!	chr4	1.1E+08	1.1E+08	+	1547	1	RRH|-|-|4	0.083	0.186
NR_13424	chr7	85421122	85489338	+	829	1	LINC00972	0	0
NM_0203	chr10	97572441	97583886	+	1500	1	ANKRD2|A	0.06	0.018
NM_0022!	chr1	2.04E+08	2.04E+08	-	709	1	KISS1|HH1	0.218	0
NM_0164:	chr9	33264879	33282069	+	2060	1	CHMP5|C!	40.909	41.423
NR_10462	chr5	29380089	29395976	-	667	1	LINC02064	0	0
NR_13576	chr2	2.42E+08	2.42E+08	-	529	2	LOC28509	0	0
NM_0010	chr1	27612290	27626595	-	2411	1	FGR|SRC2	0.032	0.102
NM_0011:	chr2	58046712	58159920	+	1766	1	VRK2|-|-|:	1.884	3.312
NM_0010	chr17	7579488	7582111	+	1766	1	CD68|GP1	1.833	2.8
NM_0012!	chr3	38697346	38794010	-	5553	1	SCN10A|F	0	0
NR_03612	chr11	59595077	59595158	-	81	1	MIR3162|	0	0
NR_10354	chr22	24270817	24442360	+	6269	1	SPECC1L-A	16.534	14.685
NM_2125!	chrX	1.01E+08	1.01E+08	-	2846	1	XKRX|XKR:	0.032	0.096
NR_03389	chr7	1.43E+08	1.44E+08	+	5011	1	EPHA1-AS:	0.015	0.03
NM_0042:	chr2	1.96E+08	1.96E+08	-	5297	1	STK17B|DI	0.324	0.615
NM_0013:	chr15	91099588	91302565	+	12544	1	SV2B|HsT:	0.502	0.531
NR_04987	chrX	29574278	29574358	+	80	1	MIR4666B	0	0
NR_02714	chr2	1.1E+08	1.1E+08	+	771	2	LOC44089	0	0
NM_0162:	chr5	26880600	27038582	-	3061	1	CDH9|-|-|	1.441	0.984
NM_0239:	chr3	1.51E+08	1.51E+08	-	1516	1	GPR87|FK!	0.034	0.063
NR_03390	chr14	20919341	20920176	+	603	1	ECRP|-|-|:	0	0
NM_1307:	chr2	1.24E+08	1.25E+08	+	5348	1	CNTNAP5|	1.733	1.962

NR_12204	chr14	20588224	20607221	+	2026	1	LOC25402	0	0
NM_0033	chr17	4269217	4366674	-	4200	1	UBE2G1|E	15.497	20.699
NM_1826	chr7	55887277	55942226	+	4529	1	ZNF713|-|	4.128	4.518
NM_0016	chr11	47239302	47248847	-	2131	1	ACP2|LAP	28.086	23.158
NM_0011	chr3	52779926	52792068	+	2359	1	ITIH1|H1P	0.016	0.012
NR_00300	chr1	1.56E+08	1.56E+08	-	128	1	SCARNA4|	0	0.428
NR_13551	chr21	42777819	42779994	-	510	1	LINC0166	1.416	3.599
NM_0001	chr4	1.19E+08	1.19E+08	-	2243	1	FABP2|FA	0	0
NM_0013	chr3	1.39E+08	1.39E+08	-	1046	1	CEP70|BIT	2.071	3.719
NM_0012	chr2	2.34E+08	2.34E+08	+	5280	1	MROH2A|	0.002	0.01
NM_0054	chr7	1.44E+08	1.44E+08	+	5471	1	ARHGEF5|	0	0
NM_1332	chr19	54874093	54891420	+	2510	10	FCAR|CD8	0	0
NM_0013	chr1	1.55E+08	1.55E+08	+	1152	1	LOC10192	13.319	8.263
NR_03412	chr2	1.14E+08	1.14E+08	+	850	1	LINC01191	0	0.064
NM_0189	chr5	1.41E+08	1.42E+08	+	4721	1	PCDHGB3|	19.694	78.817
NR_04609	chr14	88024550	88087344	+	827	1	LINC0114	0	0
NM_0211	chr1	1.69E+08	1.69E+08	-	1906	1	CCDC181|	3.687	2.393
NM_1489	chr17	19237377	19336715	+	4652	1	EPN2|EHB	18.104	20.981
NM_0050	chrX	3604343	3713634	-	6073	1	PRKX|PKX	6.686	10.406
NM_0003	chr17	63938554	63972918	-	7779	1	SCN4A|CN	0.005	0.004
NM_0040	chr17	75941511	75979434	-	7565	1	ACOX1|AC	6.284	8.187
NR_01538	chr11	19710934	19714672	-	3738	1	LOC10012	0.183	0.652
NM_0013	chr2	2.3E+08	2.3E+08	+	2572	1	SP140L|-|	0.02	0.011
NR_04700	chr13	73564244	73587879	+	363	1	LINC00392	0	0
NM_0208	chr17	7717354	7833744	+	14867	1	DNAH2|DI	0.396	0.328
NM_0011	chr16	67289490	67326758	-	2893	1	KCTD19|-|	0.134	0.066
NR_14445	chr13	74231444	74259976	+	3351	1	LINC00402	0	0.016
NM_1735	chr5	55625845	55712335	-	2518	1	SLC38A9|U	5.617	6.06
NM_0013	chr22	50190550	50199599	+	1731	1	TRABD|LP	13.795	14.093
NR_03157	chr6	98024531	98024619	+	88	1	MIR2113|	0	0
NR_12595	chr1	627380	629009	-	795	1	LOC10192	0	0
NR_02407	chr12	71848294	71850983	-	2689	1	MRS2P2|-	0.086	0.143
NM_1993	chr1	1.67E+08	1.67E+08	-	8115	1	ILDR2|C1o	6.448	5.43
NM_0068	chr11	65890404	65891635	+	1229	1	CCDC85B|	22.64	21.933
NM_0241	chr19	9523224	9538645	-	7093	1	ZNF426|K-	2.721	3.393
NM_0013	chr2	96705930	96740092	-	2215	1	LMAN2L|N	15.461	17.42
NR_04073	chr1	42767245	42775742	+	895	1	C1orf50|-|	24.029	21.471
NM_0013	chr4	40810027	41214618	-	8793	1	APBB2|FE	12.14	11.364
NM_0020	chr14	64986789	65062652	+	2805	1	FNTB|FPTI	13.79	13.277
NM_0012	chr2	1.71E+08	1.71E+08	+	2410	1	GORASP2|	42.886	55.345
NR_02985	chr4	1.13E+08	1.13E+08	-	67	1	MIR302D|	0	0
NR_11075	chr18	55881688	55920199	-	2306	1	LINC0141	0	0
NM_1814	chr2	1.08E+08	1.09E+08	+	6952	1	GCC2|GCC	0.714	0.841
NM_1734	chr22	43132206	43143397	-	2053	1	MCAT|FAS	11.462	10.988
NR_03888	chr21	34745825	34748367	+	777	1	LINC0142	0.05	0.053
NM_0011	chr16	4777614	4788521	-	1375	1	SEPT12|SF	0.356	0.359
NM_0013	chr16	58279997	58295047	-	1624	1	PRSS54|CT	1.818	1.982
NM_0203	chr14	21287977	21351301	+	3920	1	RPGRIP1|C	0.046	0.038
NR_03967	chr6	75428407	75428473	+	66	1	MIR4463|-	0	0
NM_0179	chr2	28849821	28870309	-	1835	1	TRMT61B|	9.155	9.263

NR_03393	chr2	1.31E+08	1.31E+08	-	2226	2	LOC64674	0	0
NM_0013	chr8	35544392	35796550	+	8871	1	UNC5D|PF	1.718	0.957
NM_0013	chr16	56361452	56424995	-	3203	1	AMFR|GP	31.906	43.695
NM_0030	chr17	40627728	40647851	-	2404	1	SMARCE1|	73.548	51.711
NR_03029	chr4	350157	350252	+	95	1	MIR571|M	0.271	0
NM_0013	chr6	79201245	79234738	-	863	1	HMGN3|P	62.749	83.849
NM_1815	chr10	47747649	47762793	+	1524	2	SYT15|CHI	0.254	0.279
NM_1774	chrX	30247154	30252038	+	1704	1	MAGEB1|	0	0
NM_1336	chr2	1.73E+08	1.73E+08	+	7169	1	MAP3K20|	1.05	2.421
NM_0185	chr2	2.01E+08	2.01E+08	+	2284	1	STRADB|A	8.744	10.009
NM_0013	chr2	2.17E+08	2.17E+08	+	990	1	IGFBP2|IB	456.742	249.701
NM_0012	chr10	37776526	37857636	-	948	1	ZNF248|b	0.027	6.039
NM_0013	chr17	75109952	75130265	+	2092	1	ARMC7|-|	10.687	10.659
NM_0010	chr10	46375590	46391783	+	2085	1	ANXA8L1|	0	0
NM_1533	chr11	18698735	18704785	+	3646	1	TMEM86A	2.829	4.549
NR_13513	chr9	35646270	35647475	+	948	1	LOC10192	0.027	0.101
NM_0011	chr8	11769663	11787345	+	2056	1	NEIL2|NEI	13.671	13.517
NM_0120	chrX	7048920	7148190	-	2134	1	PUDP|DXF	13.643	11.72
NM_0030	chr1	1.13E+08	1.13E+08	-	3902	1	SLC16A1|I	10.726	11.45
NM_0012	chr13	1.11E+08	1.11E+08	+	2304	1	NAXD|CAF	37.623	46.352
NM_0177	chr7	983199	989640	+	2293	1	CYP2W1|-	0.056	0.137
NR_03714	chr20	1773365	1779746	-	743	1	LOC10028	0.26	0.092
NR_03416	chr17	49248243	49258665	+	719	1	FLJ40194|	0	0
NM_0151	chr14	1.03E+08	1.03E+08	+	5742	1	RCOR1|CC	5.47	7.869
NM_0033	chr14	1.03E+08	1.03E+08	+	7624	1	TRAF3|CAI	6.724	8.07
NM_0331	chr17	41148924	41149802	-	876	1	KRTAP4-5|	0	0
NM_0530	chr4	69280499	69295050	+	1825	1	UGT2B28|	0	0
NM_0165	chr11	73872668	73876845	-	812	1	COA4|CHC	35.425	30.97
NR_01535	chr4	84966818	85007015	+	1565	1	WDFY3-AS	2.208	1.689
NM_0062	chr8	38787204	38853028	+	7787	1	TACC1|Ga	4.401	5.919
NR_11079	chr18	12075	15930	+	823	2	LOC10272	0	0
NR_13237	chr15	1.01E+08	1.01E+08	+	639	1	PCSK6-AS1	0	0
NM_0011	chr3	1.72E+08	1.72E+08	+	1874	1	TMEM212	0	0
NM_0013	chr14	64449106	64505213	-	1435	1	ZBTB25|C	1.384	2.358
NM_1784	chr3	1.93E+08	1.93E+08	-	3363	1	MB21D2|	4.148	4.729
NR_03833	chr4	1.46E+08	1.46E+08	-	1176	1	LINC01095	0	0
NM_0011	chr3	1.01E+08	1.01E+08	+	1719	1	TFG|HMSN	62.667	54.692
NM_0333	chr11	72814406	72829635	+	2125	1	ATG16L2|	1.341	1.592
NR_12060	chr10	30364327	30371907	-	2872	1	GOLGA2P6	0.009	0.048
NM_0250	chr17	8310822	8322516	+	4122	1	ARHGEF15	0.025	0.007
NM_0033	chr9	1.34E+08	1.34E+08	-	4705	1	VAV2|VAV	58.669	34.585
NM_0010	chr6	36315758	36336885	-	3721	1	C6orf222|	0.049	0.029
NR_03408	chr1	93310109	93345811	-	4024	1	CCDC18-AS	1.551	1.474
NR_13565	chrX	1.31E+08	1.31E+08	+	925	1	LOC10537	0	0
NR_02414	chr14	1.01E+08	1.01E+08	+	494	1	MEG8|Bsr	0	0
NM_0001	chr15	80152891	80186582	+	1781	1	FAH|-|-|1	3.938	4.768
NM_0011	chr6	35342558	35428191	+	3445	1	PPARD|FA	9.417	12.413
NM_0013	chr4	1.48E+08	1.48E+08	-	3366	1	PRMT9|PF	2.593	3.28
NM_1389	chr3	1.13E+08	1.13E+08	-	951	1	CD200R1|	0.081	0
NM_0179	chr17	81556885	81637153	-	4405	1	NPLOC4|N	29.253	34.872

NM_0031(	chr1	1.79E+08	1.79E+08	+	6904	1	SOAT1|AC	1.739	1.875
NM_0011!	chr2	1.74E+08	1.74E+08	+	2952	1	SCRN3|SE!	4.529	5.749
NM_0011(	chr1	43147923	43156396	+	511	1	FAM183A|	1.842	0.885
NM_0056:	chr17	34319047	34321402	+	1245	1	CCL8|HC1	0	0
NR_00349	chr15	25268517	25268549	+	32	1	SNORD115	0	0
NM_0035:	chr6	27865329	27865798	+	467	1	HIST1H2AI	0.083	0.029
NM_0003{	chr3	48856923	48898993	-	1881	1	SLC25A20|	9.727	7.384
NR_12210	chr17	51336715	51342571	+	2642	1	LINC02072	0	0.005
NM_0156(	chr1	1.8E+08	1.8E+08	+	4039	1	TOR1AIP1	6.346	5.853
NM_1527:	chr11	85683844	85686277	-	2431	1	CCDC89|-|	0.164	0.101
NM_0013:	chr19	10333776	10339834	-	2254	1	ICAM3|CD	3.627	3.184
NR_04749	chr2	11393981	11403077	+	1327	1	LINC0057C	0.019	0
NM_0010{	chrX	1.24E+08	1.24E+08	-	5569	1	THOC2|CX	3.283	6.941
NR_04659	chrX	20139968	20140444	+	376	1	EIF1AX-AS:	0	0
NM_0207!	chr6	1.22E+08	1.22E+08	-	3190	1	SERINC1|T	105.254	139.688
NM_0325(	chr6	99919910	99994238	-	2136	1	MCHR2|GI	0.024	0
NM_0013:	chr16	56451524	56477495	+	2794	1	OGFOD1|T	16.733	17.967
NM_0011!	chr14	23306860	23311759	+	3554	1	BCL2L2|BC	11.286	10.491
NM_0010(	chr10	75037836	75058514	-	659	1	DUPD1|DU	0	0
NM_0010:	chr1	1.54E+08	1.54E+08	-	546	1	S100A13|-	27.015	7.576
NM_1944:	chr18	9913958	9960021	+	6851	1	VAPA|VAP	16.4	20.676
NM_0150	chr10	86435256	86521784	-	6281	1	WAPL|FOE	4.945	5.761
NM_0048(	chr2	1.28E+08	1.28E+08	-	2319	1	POLR2D|H	19.71	20.501
NR_02705	chr22	21001922	21003272	+	1261	1	THAP7-AS:	5.368	4.453
NR_04675	chr3	1.93E+08	1.93E+08	+	633	1	ATP13A5-/	0.224	0.195
NM_0013:	chr1	1.51E+08	1.51E+08	+	1735	1	GABPB2|G	1.241	1.374
NR_02455	chr6	27357823	27375374	-	2884	1	ZNF204P|:	1.229	0.712
NM_0072:	chr12	57216795	57226449	+	1800	1	NXPH4|NF	24.755	32.812
NR_13494	chr13	61424689	61427946	-	750	1	LOC10192	0	0.018
NR_02940	chr10	87342411	87343574	+	1163	1	LINC00863	1.608	1.46
NM_0051{	chr8	85438827	85449038	+	1742	1	CA3|CAIII|	0.155	0.448
NR_02442	chr9	1.28E+08	1.28E+08	+	2105	1	PTGES2-AS	0.123	0.104
NR_10444	chr6	37212178	37258155	-	1405	1	TMEM217	1.377	1.267
NM_0017	chr5	1.8E+08	1.8E+08	+	4933	1	CANX|CN	129.52	103.409
NM_0322:	chr3	1.93E+08	1.94E+08	-	7567	1	ATP13A4|	0.148	0.206
NR_10703	chr11	11783135	11783222	-	87	1	MIR8070|	0	0
NR_13211	chr16	2154782	2155358	-	355	1	SNHG19|S	21.174	21.838
NR_03611	chr11	46451807	46451887	+	80	1	MIR3160-:	0	0
NM_0012(	chr4	71741694	71804041	-	1763	1	GC|DBP|D	0	0.109
NM_2071:	chr6	1.58E+08	1.58E+08	+	7481	1	GTF2H5|C	5.081	6.084
NM_0012!	chr15	43794174	43800097	-	2310	1	SERINC4|-	21.947	16.401
NM_0008:	chr13	1.14E+08	1.14E+08	-	2518	1	GAS6|AXL	25.526	28.118
NR_02438	chr3	1.97E+08	1.97E+08	+	1390	1	NCBP2-AS:	25.203	28.783
NM_0059:	chr18	13825544	13826862	+	1316	1	MC5R|MC	0.088	0.042
NM_0327!	chr22	41459717	41468704	-	1059	1	PHF5A|INI	36.306	38.983
NM_0149	chr1	1.56E+08	1.56E+08	-	2976	1	KIAA0907|	16.862	19.878
NR_03607	chr1	2.49E+08	2.49E+08	+	66	1	MIR3124|	0.586	0.208
NM_0013(	chr2	1.31E+08	1.31E+08	+	4251	1	PLEKHB2|I	52.253	61.058
NM_0013:	chr2	1.14E+08	1.14E+08	-	2348	1	SLC35F5|-	2.136	2.415
NM_0177:	chr1	68474152	68497221	-	4573	1	DEPDC1|D	0.056	0.24

NM_0146(	chr1	43650126	43705518	+	4485	1	KDM4A|JH	38.062	53.114
NM_1817:	chr17	62423885	62450093	+	2508	1	METTL2A|	8.22	8.694
NR_10676	chr12	48132797	48132867	+	70	1	MIR6505|I	18.603	19.371
NM_0012{	chr7	1.07E+08	1.07E+08	+	6972	1	PIK3CG|PI	0.007	0.012
NM_0190(	chr15	23643549	23647846	-	4295	1	MAGEL2|M	11.63	11.806
NM_0013(	chr10	49818275	49942027	-	3848	1	PARG|PAR	4.326	4.827
NM_0252:	chr6	1.5E+08	1.5E+08	+	3171	1	ULBP1|N2	0.667	0.743
NM_1308(	chr11	64803514	64810563	-	2717	1	MEN1|ME	29.972	32.491
NM_0309(	chr6	1.32E+08	1.32E+08	-	1756	1	OR2A4|OF	0	0
NM_0011:	chr1	92474761	92486071	-	2685	1	GFI1|GFI-1	0.048	0.051
NM_0010:	chrX	71911088	72143574	+	3669	1	NHSL2|-|-	0.766	0.665
NM_0012(	chr1	2.43E+08	2.44E+08	-	1559	2	AKT3|MPF	0	0
NM_1818{	chr4	1.03E+08	1.03E+08	-	3929	1	UBE2D3|E	32.048	37.758
NM_0010:	chr2	74135401	74147912	-	460	1	BOLA3|MI	24.133	15.037
NM_0010(	chr17	38936432	38967402	-	2289	1	FBXO47|-|	0	0
NM_0144(	chr6	54308405	54390152	+	1791	1	TINAG|TIN	0.13	0.122
NM_0011¿	chr6	49550400	49552095	+	1693	1	C6orf141|	0.046	0.146
NM_0010:	chr13	49308650	49444126	-	3551	1	CAB39L|M	6.674	7.826
NM_0012!	chr12	564296	610413	-	862	1	NINJ2|-|-|	0.09	0.079
NM_0013(	chr1	2.01E+08	2.01E+08	-	6835	1	KIF14|MK!	0.115	0.327
NM_0006:	chr17	63928736	63932354	-	1265	1	CD79B|AG	0.02	0.011
NR_13501	chr12	93894965	93943606	-	540	1	LOC10536!	0.86	0.355
NM_0012(	chr3	46719583	46736431	-	978	1	PRSS46|TE	0	0
NR_00360	chr22	16827474	16829335	+	1379	1	HSFY1P1|(	0	0
NM_0013:	chr1	1.75E+08	1.76E+08	-	5061	1	TNR|TN-R	0.313	0.628
NM_0010(	chr1	1.57E+08	1.57E+08	+	2552	1	NTRK1|MT	1.617	4.986
NM_0122(	chr9	34989641	34998433	+	2440	1	DNAJB5|H	93.039	107.583
NR_03037	chr20	50585786	50585879	+	93	1	MIR645|M	0	0
NM_0058!	chr6	1.6E+08	1.6E+08	+	1540	1	ACAT2|-|-	66.36	110.03
NM_0013:	chr11	64938230	64972091	-	2039	1	MAJIN|C1:	0	0
NM_0141¿	chr7	1.46E+08	1.48E+08	+	8113	2	CNTNAP2|	1.639	3.115
NM_1991:	chr22	22646312	22647898	+	841	1	GGTLC2|G	0	0
NM_0012{	chr20	3162617	3173592	-	4093	1	LZTS3|PRC	19.978	26.316
NR_04675	chr3	43346919	43351962	-	3472	1	SNRK-AS1|	5.492	7.16
NR_00281	chr9	63315243	63334520	-	3176	1	AQP7P1|b	0.008	0.022
NM_0166(	chr5	1.38E+08	1.38E+08	+	4387	1	FAM53C|C	33.816	47.189
NM_0246!	chr19	17555594	17583162	+	3693	1	COLGALT1	21.521	19.042
NM_1942!	chr8	1.24E+08	1.24E+08	-	4298	1	TMEM65|-	4.422	2.824
NM_0012(	chr10	1.23E+08	1.23E+08	-	736	1	FAM24B|-	1.594	1.898
NM_0044:	chr11_KI2	36288	54157	-	4464	3	DUSP8|C1	0.179	0.153
NM_1824!	chr12	95867048	95942650	-	1936	1	CCDC38|-|	0.08	0.05
NR_03838	chr3	1.57E+08	1.57E+08	-	3334	1	LINC0088€	0.019	0.025
NM_0012{	chr11	1.19E+08	1.19E+08	+	3655	1	NLRX1|CLI	0.236	0.386
NR_12605	chr20	41340853	41360585	+	4334	1	LPIN3|LIPI	0.967	0.834
NR_04754	chr9	21994791	22077890	+	987	1	CDKN2B-A	0	0
NM_0179(	chr11	72097292	72103387	-	1172	1	LAMTOR1	73.422	67.866
NM_0013:	chr21	7048859	7087230	+	2348	4	LOC10272¿	0	0
NR_11196	chr10	46759023	46786770	-	480	1	GLUD1P7|	0.188	0.571
NM_0182(	chr16	46657979	46689232	-	4964	1	VPS35|ME	30.795	31.654
NM_0051:	chr17	61942605	62065282	-	10433	1	MED13|AI	5.516	7.968

NM_0010:	chr3	1.26E+08	1.26E+08	+	1033	1	ROPN1B|-	0.1	0.199
NM_0251:	chr19	40717103	40740861	+	3377	1	ITPKC|IP3-	12	11.515
NM_0010:	chr1	9588874	9604962	+	3899	1	TMEM201	8.274	8.277
NM_0150:	chr4	48497363	48780299	-	11640	1	FRYL|AF4p	5.762	7.784
NR_13557	chr17	49289206	49362114	-	4658	1	ZNF652|-|	4.62	4.97
NR_13192	chr1	2.12E+08	2.12E+08	+	799	1	LINC01693	0	0
NM_0124	chr7	90154375	90164827	+	1306	1	STEAP1|PI	0.168	0.094
NM_0011	chr4	36284022	36344785	+	3354	1	DTHD1|-|-	0	0.004
NR_00297	chr1	28580381	28580512	-	131	1	SNORA44|	3.642	6.273
NM_0012!	chr1	2.3E+08	2.3E+08	-	3346	1	PGBD5|-|-	25.456	41.234
NM_0321(	chr11	95066877	95071224	+	4345	1	SRSF8|DSI	12.576	12.326
NM_0040!	chr14	67360317	67386516	+	4155	1	EIF2S1|EIF	14.318	16.822
NR_02707	chr8	1.03E+08	1.03E+08	-	494	1	BAALC-AS2	1.801	1.913
NM_0025	chr17	31294650	31297362	-	1891	1	OMG|OM(	0.191	0.239
NR_07338	chr11	71794363	71813859	-	1584	1	ALG1L9P|-	0.179	0.553
NR_03387	chr12	1.27E+08	1.27E+08	-	616	1	LINC00944	0	0
NR_13568	chr15	86938797	86988426	-	496	1	LOC10272	0	0.028
NM_2015!	chr14	81175453	81221231	-	5944	1	GTF2A1|TI	4.419	4.793
NM_0146!	chr9	1.34E+08	1.34E+08	+	3705	1	ADAMTSL2	0.268	0.248
NR_13517	chr16	74406289	74407219	-	461	1	LOC10537	0	0
NM_0037(	chr2	85577491	85582033	+	771	1	VAMP8|EI	1.254	1.279
NM_0013(	chr20	36876121	36894231	+	1985	1	TLDC2|C2(	0.721	1.145
NM_0012	chr15	39799022	39829276	-	3677	1	GPR176|H	6.406	9.071
NR_03658	chr17	10794913	10804099	-	1528	1	LINC00675	0.051	0.009
NM_0011:	chr5	95646777	95657145	+	1637	1	RFESD|-|-	0.032	0.1
NM_0179!	chr2	86106182	86142157	+	6708	1	PTCD3|MI	6.672	8.206
NM_0010(	chr5	1201595	1225115	+	5158	1	SLC6A19|I	0	0
NM_0011!	chr16	1363205	1379683	-	3966	1	UNKL|C16	6.879	6.051
NM_0150(	chr16	49487531	49822738	-	7642	1	ZNF423|EI	25.512	27.441
NM_0223	chr7	35632660	35695162	-	2899	1	HERPUD2|	11.444	11.698
NM_0011	chr9	1.09E+08	1.09E+08	-	6554	1	PTPN3|PTI	0.51	0.598
NM_0013	chr10	45374166	45446119	+	2468	2	ALOX5|5-L	0	0
NM_0030	chr9	17578954	17797128	+	2722	1	SH3GL2|CI	7.972	51.316
NM_0160(	chr6	1.25E+08	1.25E+08	-	1607	1	HDDC2|C6	28.25	26.346
NM_0056:	chr7	1.29E+08	1.29E+08	+	3723	1	SMO|CRJS	14.095	9.485
NM_0013	chr9	5041693	5128183	+	4632	1	JAK2|JTK1	1.225	1.224
NM_0331	chr20	31819375	31834697	+	2778	1	MYLK2|KN	0.274	0.133
NR_03897	chr3	1.31E+08	1.31E+08	-	1974	1	LOC33987	0.078	0.09
NR_00295	chr7	75943783	75943916	+	133	1	SNORA14A	0.388	0.206
NM_0010!	chr20	18383367	18467185	-	3477	1	DZANK1|A	4.172	3.9
NM_0010!	chr5	1.33E+08	1.33E+08	-	2877	1	SEPT8|SEI	16.223	16.201
NM_0012!	chr13	76952489	76958641	+	2084	1	ACOD1|CA	0.025	0
NR_13633	chr16	22385769	22407597	+	826	1	MFSD13B|	0.031	0.083
NM_0010:	chr15	74069857	74082550	-	3156	1	GOLGA6A|	0	0
NM_0010!	chr16	90056566	90075930	-	3210	1	PRDM7|PI	0.012	0.004
NM_0012	chr3	40387156	40428626	+	2813	1	ENTPD3|C	0.289	0.521
NR_11014	chr6	1.44E+08	1.44E+08	-	1468	1	PHACTR2-/	0.07	0.009
NM_0008!	chr1	1.1E+08	1.1E+08	+	1416	1	GSTM4|GS	4.462	4.498
NR_02968	chr17	28861533	28861618	-	85	1	MIR144|M	0	0
NR_00356	chr16	18571248	18598285	+	2714	1	ABCC6P1|	0.01	0.03

NM_1384(	chr8	1.43E+08	1.43E+08	+	1337	1	GLI4|HKR4	12.141	11.402
NR_12537	chr15	44728988	44732851	+	2052	2	LOC10041	0	0
NM_0013:	chr2	1.59E+08	1.59E+08	-	1535	1	WDSUB1|l	3.007	3.605
NM_0023!	chr6	1.14E+08	1.14E+08	+	4302	1	MARCKS|8	109.58	102.562
NR_13528	chr2	18986451	19026971	-	1945	1	LINC01376	0.239	0.211
NM_0223!	chr2	1.9E+08	1.9E+08	-	2305	1	OSGEPL1|(	4.436	5.027
NM_0011(	chr20	50123737	50153798	-	2278	1	TMEM189	14.308	12.609
NM_0010:	chr9	1.36E+08	1.36E+08	+	739	1	PAEP|GD|	0	0
NM_0013:	chr13	1.11E+08	1.11E+08	+	4884	1	ARHGEF7|	23.469	29.166
NM_0026(	chr6	1.19E+08	1.19E+08	+	3017	1	PLN|CMD1	0	0.018
NR_10423	chr22	32359906	32371264	-	1135	1	RFPL3S|N(	0.807	0.748
NM_0012:	chr15	20835372	20866314	-	1674	2	POTEB2|-|	0	0
NM_0012:	chrX	55000323	55007873	+	1907	1	APEX2|AP	6.044	6.033
NR_10695	chr7	1.43E+08	1.43E+08	+	114	1	MIR6892|l	1.357	1.802
NM_0071!	chr12	65050624	65121566	-	2228	1	WIF1|WIF-	2.216	3.486
NM_0314	chr3	46497995	46500949	+	813	1	RTP3|LTM	0	0
NR_00369	chr20	49280313	49280415	+	102	1	SNORD12E	0.253	1.746
NM_1457:	chr4	1.53E+08	1.53E+08	-	2468	1	TIGD4|-|-|	0.413	0.211
NR_03389	chr8	20973986	20995119	+	1639	1	LINC02153	0	0
NM_0325!	chr10	1.14E+08	1.14E+08	-	3965	1	AFAP1L2|(	2.146	3.209
NM_0032(	chr2	2.17E+08	2.17E+08	-	397	1	TNP1|TP1	0	0
NM_0012:	chr15	58988146	59097419	+	5767	1	RNF111|A	8.319	8.507
NM_0012(	chr10	1.03E+08	1.03E+08	-	404	1	USMG5|D.	112.499	126.053
NR_11063	chr20	63359988	63371177	+	3259	1	LOC10013(	1.092	0.563
NM_0060:	chr11	5689587	5710863	+	3081	1	TRIM22|G	0.176	0.636
NM_0012(	chr14	23237918	23274285	+	2419	1	RNF212B|(	0.554	0.606
NR_10348	chr1	1.72E+08	1.72E+08	-	4047	1	DNM3OS|	0.016	0.007
NM_0010:	chr13	1.03E+08	1.03E+08	-	1096	1	METTL21C	0	0
NM_0247	chr1	2.23E+08	2.23E+08	-	2563	1	HHIPL2|KI.	0.07	0.027
NM_0010!	chr11	1.15E+08	1.16E+08	-	4212	1	CADM1|BI	40.649	39.995
NR_03883	chr4	1.65E+08	1.65E+08	+	3120	1	LINC01207	0	0
NR_02406	chr3	8571782	8574668	+	2886	1	LINC00312	0.036	0.019
NM_0121:	chr15	33968888	34065086	+	3034	1	CHRM5|HI	0.225	0.262
NR_11043	chr8	69424871	69448244	-	1459	1	LINC01603	0	0
NM_0011(	chr7	76480510	76505995	+	1570.5	2	DTX2|RNF	1.231	1.125
NM_0011:	chr3	58492096	58502360	+	1554	1	KCTD6|KC.	10.28	13.732
NM_1331:	chr5	1.41E+08	1.41E+08	-	2123	1	APBB3|FE(	33.579	27.871
NM_0012:	chr12	62466817	62597583	+	10325	1	MON2|-|-	2.532	3.046
NR_11053	chr22	20957092	20964680	+	683	1	LINC01637	0.038	0
NR_03977	chr5	9053816	9053895	-	79	1	MIR4636|r	0	0
NM_0132:	chr2	1.68E+08	1.68E+08	-	3254	1	STK39|DCI	15.865	9.955
NM_0162	chr3	1.01E+08	1.01E+08	-	8331	1	IMPG2|IPI	0.228	0.27
NM_0013(	chr17	5116438	5175031	+	8464	1	USP6|HRP	0.078	0.325
NR_00333	chr15	25089922	25090015	+	93	1	SNORD116	0.277	0.442
NR_03175	chr13	24162416	24162504	+	88	1	MIR2276|-	0	0
NR_11192	chr2	24076874	24169638	+	746	1	FAM228B|	10.024	5.967
NM_0149:	chr18	31829173	31943128	-	6223	1	TRAPPC8|(	2.35	4.098
NM_0150!	chr12	1E+08	1E+08	-	5152	1	UHRF1BP1	2.105	4.131
NR_10460	chr5	1855970	1856568	-	491	1	LINC02116	0.053	0
NM_0036!	chr11	55993681	55994625	-	943	1	OR5F1|OR	0	0

NM_0011:	chr2	2.01E+08	2.01E+08	+	1290	1	CFLAR|CAS	3.348	1.465
NM_0010:	chr17	8802723	8867677	-	3033	1	PIK3R6|C1	0	0
NM_0011(	chr10	88761406	88778242	+	1187	1	LIPN|ARCI	0	0
NM_0013:	chr17	42980562	42993692	+	3831	1	RUNDC1|-	12.466	11.47
NM_1942	chr5	1.41E+08	1.41E+08	-	1597	1	DND1|RBN	5.199	4.957
NM_0012	chr19	33299934	33302564	-	2628	1	CEBPA|C/E	0.908	1.772
NR_12600	chr16	87057784	87063991	+	4080	1	LOC44039	0	0.003
NM_0010(	chr6	42890265	42890816	-	549	1	C6orf226|	23.063	22.679
NM_0011!	chr13	1.01E+08	1.01E+08	-	2658	1	GGACT|A2	1.819	1.376
NM_0010	chrX	72845042	72848802	+	1092	2	DMRTC1B	0	0
NM_0010(	chr16	20309574	20327620	-	2991	1	GP2|ZAP7	0	0
NM_1749:	chr22	32413847	32457386	-	1964	1	BPIFC|BPII	0.112	0.091
NM_1532	chr20	270863	290778	-	1591	1	C20orf96|	18.137	11.949
NM_0010:	chrX	1.4E+08	1.4E+08	-	5982	1	ATP11C|A	2.233	2.1
NM_0221(	chr2	1.62E+08	1.62E+08	-	3582	1	IFIH1|AGS	0.022	0.34
NM_0012	chr6	5998000	6007605	-	1552	1	NRN1|NRN	81.897	25.682
NR_04674	chr3	1.96E+08	1.96E+08	+	888	1	UBXN7-AS	0.305	0.139
NM_0011(	chr2	86143932	86195770	-	2981	1	IMMT|HM	48.91	46.086
NR_00297	chr6	31623079	31623210	+	131	7	SNORA38|	0	0
NM_0160:	chr9	1.28E+08	1.28E+08	+	1567	1	COQ4|CGI	30.057	18.146
NR_07350	chr17	44847902	44850480	+	498	1	HIGD1B|C	0.104	0.275
NR_04006	chr1	2E+08	2E+08	-	1412	1	LINC00862	0	0.01
NM_0011!	chr7	1.41E+08	1.41E+08	+	10551	1	TMEM178	14.443	24.885
NR_10703	chr15	62304658	62304734	-	76	1	MIR8067|	0	0
NM_0012!	chr14	95086228	95158010	-	10131	1	DICER1|D(	7.421	6.521
NM_0013:	chr19	38335787	38370949	+	3590	1	CATSPERG	2.615	2.358
NR_11041	chr14	61556272	61570194	-	507	1	LOC10192	0	0
NM_0059!	chr1	2.32E+08	2.32E+08	+	2641	1	TSNAX|TR	11.468	9.278
NR_13015	chr1	77946482	77979205	-	2298	1	FUBP1|FBI	68.793	60.301
NM_0012	chr12	29223623	29335616	+	3440	1	FAR2|HEL-	0.251	3.372
NM_0182(	chr5	77430933	77492540	-	3887	1	WDR41|M	11.656	14.529
NM_0012!	chr17	64449115	64468643	+	1152	1	MILR1|Alle	0	0
NR_03771	chr17	7012417	7014533	+	804	1	RNASEK|-|	145.228	142.558
NR_03664	chr17	18537801	18625617	-	8679	1	CCDC144B	0	0.014
NR_10467	chr5	1.13E+08	1.13E+08	-	473	1	LOC10246	0.136	0
NR_12643	chr15	69592200	69695751	+	458	1	PCAT29|-|	0	0.06
NM_0144(	chr22	41561010	41576666	+	2524	1	CSDC2|PIF	24.944	31.475
NM_1722	chr5	1.56E+08	1.57E+08	+	1563	1	SGCD|35D	0.165	0.018
NM_0013(	chr3	1.84E+08	1.84E+08	+	3499	1	CHRD|-|-|	3.777	2.783
NM_0011!	chr1	1.53E+08	1.53E+08	+	1225	1	PRR9|-|-|	0	0
NM_0161!	chr14	93934153	93957421	-	2746	2	ASB2|ASB-	0	0
NR_03745	chr8	1.29E+08	1.29E+08	-	85	1	MIR3686|	0	0
NR_03960	chr14	1E+08	1E+08	-	95	1	MIR151B|	0	0
NR_02424	chr11	3380961	3409148	+	1466	1	TSSC2|-|-|	0.405	1.261
NM_0007	chr9	1.34E+08	1.34E+08	+	2746	1	DBH|DBM	0.277	0.274
NM_1825(	chr6	1.43E+08	1.43E+08	-	6245	1	ADAT2|DE	0.421	0.406
NM_0325!	chr17	53822878	53825212	+	2332	1	KIF2B|-|-|	0	0
NM_0011	chr11	43311955	43344530	+	3586	1	API5|AAC-	13.336	13.609
NR_00296	chr11	9428766	9428954	+	188	1	SNORA23|	0.411	0.219
NR_02681	chr6	31173735	31177899	-	590	7	PSORS1C3	0	0

NM_0245	chr16	68843606	69085182	+	4798	1	TANGO6|T	4.853	5.906
NR_12162	chr1	1.18E+08	1.18E+08	-	433	1	LOC10099	0	0
NM_0030	chr1	1.61E+08	1.61E+08	+	2844	1	SDHC|CYB	12.726	11.862
NM_0306	chr12	12473282	12562514	-	6303	1	DUSP16|M	14.622	27.727
NM_0221	chrX	53082360	53088546	+	2819	1	TSPYL2|CD	12.226	14.382
NM_0012	chrUn_GL	108007	139339	-	1813	1	LOC10028	0	0
NM_0010	chr7	1233018	1236977	+	1592	1	UNCX|UN	27.544	23.987
NM_0013	chr10	1.04E+08	1.04E+08	+	7669	1	SLK|LOSK|	1.838	2.027
NM_0042	chrY	13703567	13706022	+	1663	1	TMSB4Y|T	2.659	2.207
NR_10671	chr21	43609887	43609989	-	102	1	MIR6070|	0	0
NR_00323	chr14	1.01E+08	1.01E+08	+	72	1	SNORD11	0	0
NM_1720	chr7	72890627	72948304	+	5465	1	POM121|	8.984	9.832
NM_0203	chr20	20389628	20712623	-	9384	1	RALGAPA2	11.624	16.428
NM_0012	chr16	56932090	56943881	+	1755	1	HERPUD1|	41.56	62.538
NM_0010	chr6	50043575	50048651	-	339	1	DEFB112|	0	0
NM_0132	chr3	46712116	46717883	-	1339	1	PRSS50|C	0.019	0
NM_0062	chr19	9835207	9849689	+	1114	1	PIN1|DOD	82.095	66.96
NM_0013	chr13	48653711	48711225	+	4812	1	CYSLTR2|	0.024	0.302
NM_0011	chr19	47130823	47210636	+	2310	1	SAE1|AOS	69.993	77.332
NM_0011	chr7	77538035	77640071	+	2992	1	PTPN12|P	12.277	20.816
NM_1344	chr4	979075	993440	-	1168	1	SLC26A1|	0.673	0.809
NM_1450	chr19	16479057	16496192	-	1281	1	CALR3|C	0.101	0.021
NR_01544	chr8	1.44E+08	1.44E+08	+	1000	2	BREA2|-|-	0	0
NM_0012	chr1	67092176	67134971	-	2167	1	C1orf141|	0	0
NM_0227	chr3	1.26E+08	1.26E+08	-	4623	1	OSBPL11|	5.344	5.463
NM_0012	chr19	41549518	41586844	+	1682.5	2	CEACAM2	0	0
NM_0156	chr9	60914407	60920650	+	4217	2	SPATA31A	0	0
NR_10467	chr5	1.17E+08	1.17E+08	-	392	1	LOC10246	0	0.07
NM_0011	chr3	1.6E+08	1.6E+08	-	4139	1	IFT80|ATD	2.835	3.16
NM_0011	chr19	35292086	35313806	+	2405	1	MAG|GM	0	0
NR_10408	chr1	1.48E+08	1.48E+08	+	160	1	RNVU1-3|	0.161	0.171
NM_0034	chr12	6852127	6866631	+	3093	1	USP5|ISO	75.714	76.934
NM_1817	chr17	16342534	16353498	-	1157	1	CENPV|31	74.753	75.446
NM_0013	chr19	36111203	36113711	+	617	1	OVOL3|H	0.167	0.133
NR_12597	chr1	1.19E+08	1.19E+08	+	1215	1	LOC10192	1.931	1.668
NM_0012	chr3	1.08E+08	1.08E+08	+	2277	1	HHLA2|B7	0.011	0.054
NR_00439	chr7	1.49E+08	1.49E+08	-	112	1	RNY1|HY1	0	0.122
NR_11071	chr1	1.9E+08	1.9E+08	+	1339	1	LINC01351	1.348	1.33
NM_1446	chr1	1.56E+08	1.56E+08	+	744	1	TSACC|C1	0.503	0.35
NM_0042	chr17	74748612	74769360	+	2008	1	SLC9A3R1	12.521	16.917
NM_0012	chr10	1E+08	1E+08	-	2629	1	BLOC1S2|	21.81	19.86
NM_0013	chr18	26852038	26865844	-	5293	1	AQP4|MI	0.085	1.938
NM_1738	chr19	53266760	53267665	-	904	1	VN1R4|V1	0	0
NR_00153	chrY	6406244	6428444	-	1625	2	TTTY2|NC	0	0
NR_13227	chr11	17695267	17696939	-	553	1	LOC10272	0	0
NM_0010	chrY	24045230	24048014	-	2361	2	CDY1B|CD	0	0
NR_03609	chr6	27147626	27147688	+	62	1	MIR3143|	0	0.221
NM_1979	chr1	1.69E+08	1.69E+08	-	1594	1	NME7|CF	18.766	14.909
NM_0010	chr13	52617470	52643784	+	2216	1	HNRNPA1	6.493	6.632
NM_0010	chr1	2.49E+08	2.49E+08	-	952	1	OR2T27|-|	0	0

NM_0010(	chr6	31706907	31710595	+	890	7	LY6G6F|C6	0	0
NR_13451	chr1	2.1E+08	2.1E+08	-	385	1	LOC10193(	0.234	0
NM_0031(	chr6	21593741	21598618	+	4875	1	SOX4|EVI1	185.756	210.49
NM_0808	chr20	18587893	18763916	+	1378	1	DTD1|C20	142.624	147.626
NM_0011(	chr12	1.09E+08	1.09E+08	+	4225	1	MYO1H|-|	0.055	0.078
NM_0145	chr15	51829628	51912134	+	4654	1	TMOD3|U	7.436	5.333
NM_0010	chrX	73563148	73687102	+	6901	1	CHIC1|BR)	0.338	0.419
NR_12871	chr21	6859171	6859256	+	85	3	MIR8069-2	0	0
NM_0069	chr20	325571	330228	+	4655	1	SOX12|SO	44.514	48.685
NM_0012	chr4	9210657	9212249	+	1591	1	USP17L10	0	0
NM_0017	chr1	1.17E+08	1.17E+08	-	1145	1	CD58|LFA-	0.687	1.292
NR_04655	chr12	15151923	15155281	+	332	1	RERG-AS1	0	0
NR_03651	chr4	780149	781848	-	615	1	LOC10012	2.201	1.893
NM_0012	chr3	64010549	64023472	-	1215	1	PSMD6|Rp	61.539	67.301
NR_10405	chr13	91087255	91089617	-	374	1	LINC0038C	0	0
NR_11015	chr7	1.57E+08	1.57E+08	+	4045	1	LOC10192	0.51	0.45
NR_02665	chr12	54126071	54132842	+	1393	1	LOC40004	0.352	1.101
NR_00372	chr4	70067386	70085271	+	764	1	CSN1S2AP	0	0
NM_1816	chr11	1.12E+08	1.12E+08	-	2127	1	PPP2R1B|	8.741	6.633
NM_1446	chr4	86876205	86892422	-	901	1	C4orf36|-|	0.386	0.441
NM_1942	chr5	1.47E+08	1.47E+08	-	1258	1	GPR151|G	0.102	0.054
NR_12036	chr15	81324333	81443076	+	1070	1	TMC3-AS1	1.205	1.28
NM_0012	chr21	44328944	44338338	-	2128	1	C21orf2|LI	11.736	10.447
NM_0324	chr21	34073523	34143034	+	985	1	MRPS6|C2	126.173	106.684
NR_07307	chr1	1.55E+08	1.55E+08	+	2592	2	HCN3|-|-|	0	0
NM_0013	chr2	11179759	11345407	-	7803	1	ROCK2|RC	2.133	5.568
NM_0010	chr7	1.25E+08	1.25E+08	-	3931	1	POT1|CMI	3.021	4.146
NM_1532	chr6	1.39E+08	1.39E+08	-	4673	1	TXLNB|C6	0.102	0.076
NM_0010	chr14	20688773	20694186	+	738	1	ANG|ALS9	3.704	1.299
NM_0065	chr3	50347488	50351055	-	1651	1	NPRL2|FFE	29.364	23.146
NM_0012	chr1	92074436	92075472	-	1034	1	SETSIP|SE	3.641	2.676
NM_0003(	chr6	1.61E+08	1.61E+08	+	3516	1	PLG|-|-|6	0.007	0.019
NM_0012	chr14	58439681	58537213	+	4297	1	KIAA0586|	1.974	2.34
NR_12576	chr14	70698698	70712153	+	598	1	LINC0126	0.129	0.16
NR_10977	chr16	75226383	75228203	+	856	1	LOC10050	0.166	0.688
NM_1534	chr3	9902828	9916400	+	2550	1	IL17RE|-|-	0.197	0.199
NM_0064	chr11	65919257	65921577	+	996	1	DRAP1|NC	112.818	92.524
NM_0013	chr12	21354960	21379980	+	1888	1	IAPP|DAP|	0	0.022
NM_0011(	chrX	80014756	80031769	+	2344	2	TBX22|AB	0	0
NM_0013	chrX	1.4E+08	1.4E+08	+	2679	1	F9|F9 p22	0	0
NR_13528	chr16	52258564	52280299	+	383	1	CASC22|LI	0	0
NR_03898	chr9	95463609	95470019	+	1953	1	LOC10050	1.228	2.63
NM_0010	chr9	5919008	6008003	-	7194	1	KIAA2026|	2.317	5.323
NR_03396	chr10	94279290	94287070	-	1404	1	PLCE1-AS1	0.23	0.41
NR_00216	chr20	34833575	34834462	-	887	1	HMGB3P1	0	0.062
NM_0011	chr17	80220401	80253509	+	2704	1	SLC26A11|	12.25	11.271
NM_0010(	chr1	1.59E+08	1.59E+08	-	937	1	OR6N1|OF	0	0
NM_0121	chrX	49331626	49338952	+	513	1	GAGE8|CT	0	0
NR_02813	chr10	1.12E+08	1.12E+08	-	2955	1	GUCY2GP|	0	0
NR_07339	chr8	737605	1137777	+	570.3	3	ERICH1-AS	0	0.096

NM_0071!	chr11	3357927	3379222	-	3041	1	ZNF195|H	11.651	14.08
NR_00271	chr5	1.38E+08	1.38E+08	+	2985	1	NPY6R|NP	0	0.009
NM_1527	chr12	75334639	75370389	+	998	1	GLIPR1L1|	0.375	0.137
NR_10683	chr16	68233426	68233499	-	73	1	MIR6773|I	0.353	0
NR_03062	chrX	74218547	74218618	-	71	1	MIR374B|	0	0
NM_0008!	chr5	75337168	75362101	+	4560	1	HMGCR|LI	90.71	68.383
NM_0010	chr19	22752183	22784171	-	7875	1	ZNF99|C1!	0	0.003
NM_0047	chr16	29973867	29992261	+	4639	1	TAOK2|M/	27.074	28.365
NM_0012(	chr10	1.33E+08	1.33E+08	-	8218	1	CFAP46|C	0.364	0.267
NR_02939	chr10	48010134	48031640	-	2131	1	AGAP12P|	0.03	0.006
NR_03899	chr12	1.09E+08	1.09E+08	-	976	1	USP30-AS1	0.621	0.744
NM_0013	chr17	63894908	63896661	-	911	1	CSH1|CS-1	0	0
NR_12158	chr9	1.07E+08	1.07E+08	-	952	1	LINC0150!	0.027	0
NM_1336	chr21	45263928	45287896	-	2858	1	POFUT2|C	17.098	15.135
NR_10466	chr5	57650660	57670989	+	1659	1	LOC10192	0	0
NM_0007	chr14	96256210	96264763	+	1296	1	BDKRB1|B	0	0.021
NR_03832	chr8	23336208	23366125	+	4371	1	LOC10050	0.189	0.363
NM_0010(	chr11	1.24E+08	1.24E+08	-	925	1	OR8D1|JC	0	0
NM_1824	chr22	37180166	37188290	-	1646	1	C1QTNF6|	9.196	6.782
NR_02983	chr1	2.08E+08	2.08E+08	-	87	1	MIR29C|M	1.186	1.259
NR_07721	chr4	1.41E+08	1.41E+08	-	1889	1	TNRC18P1	0.143	0.152
NM_0156!	chr7	1.49E+08	1.49E+08	-	3064	1	ZNF777|-|	15.557	16.902
NM_1987	chr20	64057086	64072347	+	1622	1	TCEA2|TFI	50.509	49.417
NR_00369	chr2	2.02E+08	2.02E+08	+	111	1	SNORD11E	0	0
NM_0023!	chr17	81918269	81927711	-	5033	1	MAFG|hM	8.933	12.271
NM_0011	chr16	87707812	87765992	-	1744	1	KLHDC4|-|	11.807	11.097
NM_0005	chrX	38352483	38421450	+	1635	1	OTC|OCTD	0.11	0.025
NR_12061	chr10	99526350	99531177	-	761	1	LINC01475	0	0
NM_0027	chr17	66302808	66810744	+	8751	1	PRKCA|AA	4.892	13.043
NM_0162	chr15	64387804	64455303	+	2030	1	TRIP4|ASC	20.414	16.153
NR_13656	chr6	39048781	39091303	+	5454	1	GLP1R|GLI	0.033	0.028
NM_1446	chr19	53793601	53824403	-	3851	1	NLRP12|CI	0.007	0.021
NM_0011	chr5	72955981	73090522	+	4855	1	FCHO2|-|-	0.823	1.704
NM_0003!	chr1	1.51E+08	1.51E+08	-	1800	1	CTSK|CTS(	2.966	2.945
NM_0013	chrX	1.01E+08	1.01E+08	+	2000	1	CSTF2|Cst	9.76	10.328
NM_0010(	chr14	21154937	21156025	-	1087	1	OR5AU1|(	0	0.013
NR_02686	chr3	63819365	63848636	+	1282	1	C3orf49|-|	0.111	0.096
NM_1530	chr4	1.65E+08	1.65E+08	+	2171	1	FAM218A|	0.511	0.991
NM_0174	chr3	52221080	52226163	-	3866	1	TLR9|CD2	0.474	0.942
NM_0020	chr12	2794942	2805421	+	3729	1	FKBP4|FKE	51.999	55.512
NM_0124	chr1	2.2E+08	2.2E+08	-	7292	1	RAB3GAP2	5.087	5.355
NM_0014(	chr1	1.09E+08	1.09E+08	+	10504	1	CELSR2|AI	56.043	46.787
NM_0321	chr1	50417551	50423447	-	3078	1	DMRTA2|-	0.507	7
NM_0010(	chr1	1.75E+08	1.75E+08	+	2867	1	CACYBP|G	20.836	17.744
NR_11067	chr19	3141576	3155175	-	2315	1	LOC10099	0	0
NR_03366	chr7	34346512	34758272	-	4854	1	NPSR1-AS1	0	0.062
NM_0035	chr6	26251651	26252075	+	422	1	HIST1H2BI	0.886	1.493
NR_02775	chr16	73092349	73093773	+	1075	1	HCCAT5|F.	0.156	0.051
NM_0532	chr16	631012	634116	+	1944	1	WFIKKN1|	4.072	2.311
NM_0240	chr11	32829935	32858123	+	5530	1	PRRG4|PR	0.005	0

NR_03162	chr9	40929010	40929092	-	82	1	MIR1299|I	0	0
NR_13428	chr5	10504990	10522084	-	968	1	LOC38927	0	0
NR_03036	chr19	3961414	3961512	-	98	1	MIR637|M	5.526	7.827
NM_0021(	chr9	1.15E+08	1.15E+08	-	8569	1	TNC|150-2	11.905	38.756
NM_0011	chr16	590077	629273	+	2562	1	RAB40C|R	23.095	23.978
NM_0013	chr8	64586593	64798791	-	2678	1	CYP7B1|CI	0.159	1.253
NR_10682	chr12	1.13E+08	1.13E+08	+	85	1	MIR6762|I	0.455	0.161
NR_11033	chr17	48595958	48602341	+	431	1	HOXB-AS3	0	0
NM_0069	chr12	7919228	7936296	-	3926	1	SLC2A3|GI	24.772	13.826
NR_03647	chr15	28788243	28789392	+	893	2	LOC10028	0	0
NM_0191	chr1	32216197	32222371	-	1121	1	TMEM234	7.568	5.266
NM_0011	chr5	1.58E+08	1.58E+08	-	4003	1	CLINT1|CL	11.128	11.996
NR_13236	chr13	65866047	65878219	+	620	1	LINC01052	0	0
NM_0012	chr6_GL0C	4444672	4466382	-	2278	7	VPS52|ARI	0	0
NR_03993	chr2	2.08E+08	2.08E+08	+	74	1	MIR4775|-	2.091	0.925
NM_0013(	chr11	59171430	59208587	+	5375	1	DTX4|RNF	37.997	45.759
NM_1484	chr16	28823048	28837237	+	3782	1	ATXN2L|A	49.736	56.255
NM_0010(	chr11	71581855	71582875	-	1018	1	KRTAP5-11	0	0.013
NR_03383	chr8	74599866	74758352	+	2119	1	MIR2052H	0.03	0
NM_0006	chr22_KI2	81782	101331	+	1230	2	NCF4|CGD	0	0
NM_1766	chr16	566995	569495	+	2077	1	NHLRC4|-|	1.744	0.508
NR_12582	chr8	89717399	89757711	-	1596	1	LOC10192	1.511	1.794
NR_13117	chr11	27471731	27482433	+	533	1	LOC10537	0.024	0
NR_03748	chr3	99964314	99964398	-	84	1	MIR3921|-	0	0
NR_12584	chr6	99424871	99431314	+	1007	1	LOC10192	2.266	1.714
NR_10999	chr14	81441987	81450157	-	634	1	LOC10050	0.203	0.151
NM_0058	chr17	7888851	7912757	+	7182	1	CHD3|Mi-	90.197	101.715
NR_02940	chr1	2.43E+08	2.43E+08	-	3816	2	LINC01347	0	0
NM_0011	chr20	2692878	2760108	+	2891	1	EBF4|COE	9.036	15.024
NM_0013	chr17	42700275	42745096	-	4576	1	EZH1|KMT	14.333	15.041
NM_0021	chr17	48542656	48545031	-	1599	1	HOXB2|HC	0.024	12.438
NM_1522	chr5	1.72E+08	1.72E+08	-	3010	1	UBTD2|DC	28.049	31.408
NM_0044	chr1	2.36E+08	2.36E+08	-	4863	1	GNG4|-|-|	67.689	60.573
NR_13501	chr9	62840645	62847206	+	3121	1	PTGER4P2	0.062	0.004
NM_0068	chr12	1.24E+08	1.24E+08	+	2120	1	TMED2|P2	90.68	82.628
NM_0066	chr11	72754729	72793705	-	1979	1	STARD10|	14.653	19.608
NM_0149	chr12	1.22E+08	1.22E+08	+	8402	1	MLXIP|MI	11.212	14.082
NR_02971	chr17	7017615	7017701	-	86	1	MIR195|M	0	0
NM_0013	chr1	16673003	16673657	+	652.3	3	FAM231C|	0	0
NM_0011	chr11	1.2E+08	1.2E+08	+	4113	1	TMEM136	4.16	5.561
NM_0021	chr9	21165637	21166660	-	1021	1	IFNA21|IFI	0	0
NR_03746	chr6	5148233	5148322	-	89	1	MIR3691|	0	0
NR_10403	chr2	73957968	73981439	-	543	1	DGUOK-AS	22.13	21.214
NM_0245	chr11	14878010	14892205	-	1612	1	CYP2R1|-|	3.831	8.429
NR_13448	chr10	43777566	43778825	-	402	1	LOC10272	0	0
NR_04007	chr10	6326544	6335981	+	2960	1	LOC39971	0.057	0.134
NM_0322	chr5	6371926	6378526	-	1022	1	MED10|L6	36.195	35.596
NR_12050	chr7	1.17E+08	1.17E+08	-	1353	1	LINC0151C	0	0
NR_03899	chr13	79406309	79424333	+	2464	1	RBM26-AS	1.13	1.051
NM_0011	chr12	26938372	26960746	+	974	1	FGFR1OP2	5.189	5.892

NR_12642	chr4	1.05E+08	1.05E+08	-	748	1	TET2-AS1|	0	0.037
NR_03995	chr3	24521362	24521435	-	73	1	MIR4792|	0	0
NM_0010	chr19	4324043	4338850	-	1384	1	STAP2|BKS	5.729	4.483
NM_0002	chr3	46857867	46863483	-	920	1	MYL3|CMI	0.21	0.015
NR_04687	chr3	1.89E+08	1.89E+08	-	695	1	TPRG1-AS1	0	0.02
NR_13502	chr12	42646583	42686701	+	560	1	LOC10536	0	0
NM_0011	chrX	49879867	49882564	+	2695	1	USP27X|N	7.133	6.328
NR_10697	chr11	64341849	64341904	-	55	1	MIR7155|	0	0
NR_00299	chr3	1.29E+08	1.29E+08	-	138	1	SNORA7B|	0	0.199
NR_03964	chr3	25664873	25664939	-	66	1	MIR4442|	0.586	0.208
NM_0013	chr1	1.83E+08	1.83E+08	+	4506	1	DHX9|DDX	71.12	72.452
NM_0012	chr17	35587263	35726417	+	5698	1	AP2B1|AD	141.213	152.028
NR_02749	chr21	45419716	45425070	-	2338	1	COL18A1-A	0.011	0
NM_0033	chr10	17228259	17237593	+	2124	1	VIM|CTRC	367.968	380.918
NM_0148	chr12	6494132	6531966	+	4772	1	NCAPD2|C	15.606	16.625
NM_2135	chr10	20779974	21174187	-	6931	1	NEBL|LASI	3.773	1.715
NM_0012	chr14	93237550	93333092	-	7222	2	BTBD7|FU	0	0
NM_0012	chr1	1.15E+08	1.15E+08	+	3416	1	SYCP1|CT8	0	0
NM_0012	chr6_GL00	4513552	4529761	-	1686.9	8	HLA-DPA1	0	0
NR_02967	chr2	1.36E+08	1.36E+08	+	81	1	MIR128-1|	0	0
NR_02658	chr15	28737583	28758362	+	5237	2	WHAMMP	0	0
NR_13237	chr1	1.87E+08	1.87E+08	+	421	1	LINC01037	0.306	0.033
NM_0011	chr6	1.36E+08	1.36E+08	-	4225	1	MAP7|E-N	2.679	0.632
NM_0011	chr3	1.22E+08	1.22E+08	-	2613	1	ILDR1|DFN	0	0
NR_03396	chr7	520391	525232	+	3503	1	HRAT92|-|	0.029	0.059
NM_1759	chr4	1391552	1395994	+	4440	1	CRIPAK|-|	1.676	2.81
NM_0209	chr10	49613108	49665104	+	2097	1	CHAT|CHC	0.67	0.928
NM_0010	chr4	1639881	1683991	-	2800	1	FAM53A|D	0.437	0.318
NM_0334	chr8	85107088	85146079	+	3854	1	LRRCC1|CI	0.606	0.974
NM_1825	chr20	33081512	33111751	+	2156	1	BPIFB4|C2	0	0
NM_0011	chr6	8413067	8435567	-	1847	1	SLC35B3|C	3.316	5.206
NM_1815	chr7	99923269	99929620	-	1113	1	GJC3|CX29	0	0.062
NM_0808	chr17	2041983	2043431	+	1072	1	OVCA2|-|-	37.406	33.54
NM_0064	chr12	57530050	57547331	-	2098	1	DCTN2|DC	225.027	245.038
NR_10687	chr22	25455646	25455711	+	65	1	MIR6817|	4.166	2.739
NM_0010	chr2	74425861	74440583	-	2180	1	RTKN|-|-|	10.155	10.562
NR_03975	chr19	44653686	44653732	-	46	1	MIR4531|	0	0
NM_1817	chr1	1.48E+08	1.48E+08	-	3177	1	GJA5|ATFE	0	0.034
NM_0010	chr7	1.21E+08	1.21E+08	-	2490	1	FAM3C|GS	2.242	3.438
NR_12045	chr12	57618405	57621903	-	2440	1	LOC10192	0.914	1.039
NM_0012	chr9	1.36E+08	1.36E+08	+	767	1	LCN1|PMF	0.521	2.857
NM_0012	chr15	67819704	67833836	+	2591	1	SKOR1|CO	16.884	26.839
NM_0240	chr11	78100942	78139653	-	1653	1	ALG8|CDG	10.6	9.993
NM_2074	chr3	42905910	42919333	+	5605	1	ZNF662|-|	0.005	0.005
NM_0017	chr1	1.17E+08	1.17E+08	+	1558	1	CD2|LFA-2	0.017	0
NM_0012	chr9	1.35E+08	1.35E+08	+	2489	1	OLFM1|AN	81.521	83.921
NM_0012	chrX	1.4E+08	1.4E+08	+	1242	1	LOC38989	1.796	3.022
NR_10700	chr11	1880045	1880147	+	102	1	MIR7847|	0.253	0
NM_0060	chr19	29207260	29213229	-	1220	1	UQCRFS1|	55.833	59.043
NM_0039	chr12	12891022	12913666	+	2845	1	GPRC5A|G	0.086	0.193

NR_13626	chr7	12497246	12541135	+	499	1	LOC10272.	0	0.137
NM_0013(	chr8	27284886	27311319	-	4105	1	TRIM35|H	9.137	10.387
NM_0006(	chr9	1.37E+08	1.37E+08	+	868	1	C8G|C8C|-	0.342	0.079
NR_03414	chr12	9055589	9065070	-	2207	1	LINC00612	0.047	0.031
NM_0001;	chr3	50191610	50197696	+	2455	1	GNAT1|CS	0.105	0.145
NM_0012!	chr19	21804949	21852068	-	4922	2	ZNF43|HTI	0.359	0.504
NM_0012{	chr1	1.51E+08	1.51E+08	+	4115	1	ADAMTSL4	0.423	0.32
NM_0178‹	chr11	61430125	61446767	+	1213	1	SDHAF2|C	33.451	30.612
NR_12870	chr17	64425069	64425170	-	101	1	MIR1273E	0	0
NM_1527;	chr7	19719315	19772781	-	3358	1	TMEM196	1.67	1.742
NR_03756	chr11	65122183	65127369	+	2074	1	MRPL49|C	42.435	43.211
NM_0013:	chrX	14873405	14922166	+	2313	1	MOSPD2|-	1.455	1.196
NM_1719!	chrX	1.55E+08	1.55E+08	-	3508	1	RAB39B|M	9.935	9.906
NM_0808:	chr20	2101882	2148552	+	6405	1	STK35|CLI	7.545	9.501
NR_02454	chr1	14362	29370	-	1758	1	WASH7P|I	0	0
NM_0151!	chr11	1.12E+08	1.12E+08	+	5725	1	SIK2|LOH1	19.317	13.309
NM_0004(	chr5	1.51E+08	1.51E+08	+	3682	1	GM2A|GM	7.277	6.532
NM_0013(	chr1	1.73E+08	1.73E+08	+	1845	1	FASLG|ALI	0	0
NM_0040‹	chr15	44711487	44718159	+	981	2	B2M|IMD4	8.609	4.58
NM_0209(	chr2	1.2E+08	1.2E+08	+	6580	1	EPB41L5|E	7.332	9.095
NR_10385	chr11	7437636	7465835	-	4494	1	LOC10050(	0.063	0.088
NM_0807(	chr17	3565446	3609411	-	4145	1	TRPV1|VR	5.251	4.316
NR_13121	chr16	11819835	11828832	-	595	1	BCAR4|-|-	0	0
NM_0011!	chr1	1.58E+08	1.58E+08	-	5361	1	FCRL5|BXI	0	0
NR_03831	chr11	1.18E+08	1.18E+08	-	378	1	TMPRSS4-,	0	0
NR_13078	chr2	2.17E+08	2.17E+08	+	391	1	LINC01921	0	0
NM_0186!	chr18	50968017	50988120	+	2227	1	ELAC1|D2!	6.924	6.704
NR_02969	chr14	1.01E+08	1.01E+08	+	96	1	MIR127|M	8.058	5.564
NM_0010!	chr20	43916142	44069614	+	2406	1	TOX2|C20	22.02	12.627
NM_0010(	chr1	54225431	54406395	-	3154	1	SSBP3|CSI	94.183	63.196
NM_0013;	chr12	52274645	52309163	+	2234	1	KRT86|HB	0.012	0
NM_0010(	chr11	1.05E+08	1.05E+08	-	460	1	CARD17|II	0	0
NM_0212‹	chr6	89364616	89412276	-	4917	1	RRAGD|RA	3.202	4.791
NM_0011‹	chr19	52369917	52385793	+	2256	1	ZNF880|-|	2.726	4.044
NR_03415	chr20	32587479	32608892	+	842	1	LOC14995(	0.092	0.033
NM_0047{	chr22	20859004	20891213	+	4254	1	SNAP29|C	13.239	12.921
NM_0011‹	chr12	1.05E+08	1.05E+08	+	1358	1	C12orf75|.	10.14	10.439
NR_10421	chr1	1.46E+08	1.46E+08	-	3807	1	NBPF25P|'	0.105	0.137
NR_11059	chr2	1.61E+08	1.61E+08	-	570	1	LOC10192!	0	0
NM_0011:	chr19	54573831	54587561	+	1704.5	4	LILRA2|CD	0	0
NM_0010{	chr11	1.34E+08	1.34E+08	+	2922	1	GLB1L3|-|	0.093	0.047
NM_0187:	chr17	73193034	73208506	+	3018	1	COG1|CD(	18.285	21.145
NM_0010;	chr8	1E+08	1E+08	-	3298	1	FBXO43|EI	0.023	0.058
NM_0011!	chr2	2.33E+08	2.33E+08	+	2142	1	PRSS56|M	1.836	6.727
NM_0002;	chrX	9725413	9765965	-	1685	1	GPR143|N	1.301	0.658
NM_0010;	chr1	1.61E+08	1.61E+08	-	1115	1	NR1I3|CAI	9.471	11.695
NM_1827(	chr17	439980	445940	-	3617	1	RFLNB|CFI	4.98	5.692
NM_0246!	chr19	8848844	8981342	-	43730	1	MUC16|C/	0	0
NM_0010(	chr8	43292442	43363185	+	1823	1	POTEA|A2	0	0
NM_0175:	chrX	1.53E+08	1.53E+08	-	1352	1	HAUS7|U(	6.723	6.393

NM_0582:	chr5	1.79E+08	1.79E+08	+	2190	1	ZNF354B|I	1.166	1.833
NM_0808:	chrX	41724155	41730135	+	3344	1	GPR82|-|-	0.015	0
NM_1736:	chr15	38454127	38487710	+	4378	1	FAM98B|-	5.708	6.67
NM_0250(	chr12	43354209	43551921	-	5693	1	ADAMTS2(	0.086	0.029
NM_0010:	chr4	1.22E+08	1.22E+08	+	1629	1	EXOSC9|PI	14.753	14.429
NM_0010(	chr11	4367263	4368386	-	1121	1	OR52B4|C	0	0
NM_0010:	chr17	28755978	28842839	-	4174	1	FAM222B|	17.407	16.362
NR_00371	chr22	24251621	24265524	+	5217	1	POM121L9	0.168	0.084
NR_03891	chr8	19183676	19245522	-	1006	1	LOC10012:	0	0.014
NM_0011‹	chr7	56064224	56080672	+	1887	1	SUMF2|pF	46.333	50.926
NR_10988	chr20	33992488	33994367	-	579	1	RALY-AS1|	1.247	1.017
NM_0044(	chr2	1.72E+08	1.72E+08	-	2299	1	DLX2|TES-	0.118	5.952
NM_0140‹	chr14	22871717	22881711	+	6904	1	LRP10|LRF	8.594	7.023
NM_0010:	chr1	2.23E+08	2.23E+08	-	1088	1	SUSD4|PR	5.451	7.541
NR_02460	chr1	86634276	86655376	+	3397	1	CLCA3P|CI	0	0
NR_02675	chr18	14337423	14342524	+	2872	1	CYP4F35P|	0	0.005
NR_04675	chr7	20141916	20153531	+	635	1	MACC1-AS	0	0
NM_0028!	chr12	1.2E+08	1.2E+08	-	3678	1	PXN|-|-|1:	22.054	20.519
NM_0012(	chr2	79523183	80648863	+	3821	1	CTNNA2|C	67.035	50.547
NM_0013:	chr15	75335996	75340277	+	820	1	COMMD4|	32.454	20.362
NM_2135(	chr2	2.03E+08	2.04E+08	-	9718	1	RAPH1|AL	3.391	3.367
NR_03747	chr12	69584723	69584822	+	99	1	MIR3913-2	0	0
NM_0157:	chr17	744421	752261	-	3737	1	GEMIN4|H	9.409	11.26
NR_14447	chr6	38481596	38482527	+	650	1	BTBD9-AS:	0	0.021
NM_0181!	chr12	1.07E+08	1.07E+08	+	5043	1	RIC8B|RIC:	6.716	7.393
NR_03612	chr13	61199798	61199880	-	82	1	MIR3169|-	0	0
NM_1524!	chr1	43991608	43996526	+	1450	1	CCDC24|-|	7.772	7.075
NM_1824!	chr11	1.15E+08	1.15E+08	+	1771	1	NXPE2|FA	0	0
NR_12065	chr10	42673013	42691759	-	1731	1	LINC01518	0.015	0.079
NM_0010(	chrX	1.04E+08	1.04E+08	+	1296	1	TCEAL4|NI	58.23	59.195
NR_12616	chr12	63808845	63822156	-	559	1	TMEM5-AS	0.992	1.911
NM_0013:	chr10	87751512	87818231	-	4327	1	ATAD1|AF	20.099	21.332
NR_04986	chr6	6169334	6169409	+	75	1	MIR5683|-	0	0
NR_03611	chr9	1.28E+08	1.28E+08	-	83	1	MIR3154|r	0	0
NM_0011‹	chr15	67166155	67195195	+	5435	1	SMAD3|HS	7.613	6.351
NM_0030:	chr3	47585888	47781915	-	5727	1	SMARCC1|	37.557	33.978
NM_0024‹	chrX	65667644	65741931	+	3955	1	MSN|HEL7	50.818	40.263
NR_00002	chr1	44776492	44776589	+	97	1	SNORD46|	1.595	0.565
NM_1525‹	chr4	88257609	88284831	-	6581	1	PPM1K|BD	3.115	3.78
NR_04610	chr14	18630538	18633634	-	1270	1	LOC64242(	0	0
NM_0012‹	chr1	10430747	10452003	+	1377	1	APITD1-CC	7.088	4.993
NM_0175:	chr3	1.14E+08	1.14E+08	+	3741	1	GRAMD1C	0.362	0.524
NM_0012!	chr3	25340802	25597931	+	2585	1	RARB|HAP	1.023	1.288
NM_0011(	chr14	1E+08	1.01E+08	+	1981	1	WDR25|C:	14.885	17.064
NR_10408	chr1	1.21E+08	1.21E+08	-	166	1	RNVU1-19	0	0
NM_0050(	chr9	1.31E+08	1.31E+08	+	7562	1	NUP214|C	12.45	12.65
NM_1994‹	chr19	18899514	18919390	-	967	1	COPE|epsi	167.522	144.647
NM_0156(	chr5	1.41E+08	1.41E+08	+	2919	1	PCDHB5|P	14.576	20.276
NR_00359	chr1	1.1E+08	1.1E+08	+	1232	1	CYMP|-|-|	0	0.022
NR_03015	chr14	1.01E+08	1.01E+08	+	90	1	MIR412|M	0	0

NR_11028	chr2	75524068	75542706	+	1182	1	LOC10192	0	0
NM_0034	chr3	49554489	49671549	+	15945	1	BSN|ZNF2	21.938	20.982
NM_0011	chr7	1.29E+08	1.29E+08	+	2857	1	STRIP2|FA	0.609	0.345
NM_0331	chr20	33283135	33309878	+	1709	1	BPIFB1|C2	0	0.04
NM_0247	chr14	95181939	95319908	-	12735	1	CLMN|-|-|	0.797	0.229
NM_0153	chr9	1.29E+08	1.29E+08	+	5649	1	NUP188|K	16.952	17.45
NR_03658	chr2	1.15E+08	1.15E+08	-	727	1	DPP10-AS	3.21	3.881
NM_0037	chr6	1.67E+08	1.67E+08	-	1239	1	RNASET2|	13.133	13.454
NM_0151	chr17	17042760	17192648	+	10935	1	MPRIP|M-	29.6	30.092
NR_11017	chr14	1.01E+08	1.01E+08	+	992	1	SNHG24|-	0	0.014
NM_1723	chr17	1279663	1300987	+	3166.5	2	TUSC5|DS	0.077	0.022
NM_0207	chr15	84816680	84873482	+	10901	1	ALPK3|MA	0.61	0.259
NM_0010	chr1	1.52E+08	1.52E+08	-	9627	1	HRNR|FLG	0	0
NM_0011	chr4	1.44E+08	1.44E+08	-	6614	1	FREM3|-|-	0.019	0.027
NM_0013	chr12	1.22E+08	1.22E+08	+	2752	1	B3GNT4|B	25.595	24.02
NM_1447	chr5	34839164	34899459	+	1343	1	TTC23L|M	0.941	0.459
NM_0246	chr1	6521347	6554598	-	6698	1	NOL9|Grc	3.584	3.622
NR_03398	chr18	64213083	64260055	-	2404	1	LINC0153	0	0
NM_1532	chr10	15095385	15097319	-	1710	1	C10orf111	0.558	0.585
NM_0332	chr12	57941504	57957269	+	1310	2	ATP23|KU	0	0
NM_0034	chr2	1.36E+08	1.36E+08	-	1670	1	CXCR4|CD	129.298	48.776
NM_0010	chr2	1.74E+08	1.74E+08	-	4276	1	OLA1|DO	19.199	22.067
NM_0153	chr2	33513875	33564731	+	4317	1	RASGRP3|	0.317	0.041
NR_00299	chr21	32377187	32377322	-	135	1	SNORA80	0	0
NM_1446	chr4	1.76E+08	1.76E+08	-	1225	1	SPATA4|S	0.305	0.246
NM_0051	chr19	35868445	35879797	+	2482	1	APLP1|AP	460.29	342.944
NM_0176	chr10	1.03E+08	1.03E+08	+	4188	1	CNNM2|A	5.797	6.832
NM_0156	chrX	10015755	10144478	+	6429	1	WWC3|BN	5.872	4.864
NM_0013	chr2	1.31E+08	1.31E+08	-	5198	1	FAM168B|	60.407	69.06
NM_0012	chr4	1.75E+08	1.75E+08	+	2945	1	ADAM29|	0.079	0.102
NM_0160	chr1	38012712	38024825	+	2040	1	UTP11|CG	15.32	15.517
NR_00145	chr11	1.23E+08	1.23E+08	-	87	1	SNORD14	2.223	0.787
NR_03976	chr22	48780295	48780353	+	58	1	MIR4535|	0	0
NR_03415	chr19	51591783	51594380	-	1699	1	LINC0153	2.057	2.007
NM_0011	chr12	42458338	42483614	-	4244	1	PRICKLE1|	4.627	5.8
NR_00318	chr20	45363169	45410610	+	1338	1	SYS1-DBN	28.842	29.267
NR_12655	chr2	16050427	16085689	+	980	1	GACAT3|L	0.026	0.028
NM_0134	chr2	1.83E+08	1.83E+08	-	4964	1	NCKAP1|H	20.029	36.925
NM_0248	chr20	38748454	38772446	+	2468	1	ACTR5|Ar	6.347	6.854
NM_0309	chr1	2.05E+08	2.05E+08	-	3428	1	NUAK2|SN	5.969	5.53
NM_0178	chr20	49219295	49244077	+	2672	1	DDX27|DR	15.234	13.979
NM_0002	chr2	27086743	27100751	+	2405	1	KHK|-|-|2	7.731	11.237
NM_0017	chr20	61252426	61940617	+	5931.5	2	CDH4|CAD	0.059	4.577
NM_0011	chrX	1.5E+08	1.5E+08	-	1958	2	HSFX2|-|-	0.04	0.077
NR_12543	chr8	10840026	10846509	+	442	1	LOC10192	0.292	0.217
NM_0030	chr10	86958531	86963260	+	819	1	SNCG|BCS	24.213	12.326
NM_0012	chr2	47783955	47806953	+	4051	1	MSH6|GT	21.726	26.173
NM_0022	chr3	1.6E+08	1.61E+08	-	8962	1	KPNA4|IP	7.841	8.924
NR_03018	chr19	53682159	53682245	+	86	1	MIR520F|	0	0
NM_0150	chr16	3500945	3539051	+	4131	1	CLUAP1|C	8.958	6.916

NM_0326	chr14	1.02E+08	1.02E+08	-	972	1	CINP|-|-|1	19.234	17.614
NR_04077	chr1	1.55E+08	1.55E+08	-	1182	1	DCST1-AS1	2.705	2.016
NM_1307	chr19	58346806	58353499	-	1756	1	A1BG|A1B	7.21	5.14
NR_03041	chr17	59137758	59137872	-	114	1	MIR454|M	0	0
NM_0011	chr1	1.15E+08	1.15E+08	-	2377	1	AMPD1|M	0.011	0.006
NR_12634	chr16	9446013	9455505	-	443	1	LINC01195	0	0
NM_0012	chr1	92842160	92961522	-	2420	1	FAM69A|-	2.914	4.273
NM_0251	chr6	1.53E+08	1.53E+08	+	3039	1	MYCT1|M	0.59	0.005
NM_0055	chr9	1.28E+08	1.28E+08	+	836	1	LCN2|24p	0.031	0
NR_10980	chr9	99585786	99819889	-	1537	1	LOC10192	0	0.125
NM_0010	chr11	6869755	6870666	+	910	1	OR10A2|C	0	0
NM_0174	chr14	60709538	60724134	-	6270	1	SIX4|AREC	0.218	0.306
NM_0028	chr18	7567316	8406861	+	5528	1	PTPRM|PT	14.519	18.261
NM_0011	chr6	22287244	22302853	-	1025	1	PRL|GHA1	37.146	1.724
NM_0051	chr11	1.19E+08	1.19E+08	+	11213	1	CBL|C-CBL	11.299	12.975
NR_12066	chr10	85193421	85198905	-	1196	1	LINC0151	0	0
NM_0164	chr2	36356227	36551135	+	5592	1	CRIM1|CR	18.824	7.542
NM_0012	chr6	24797321	24798896	-	817	1	C6orf229|	0	0.05
NM_0013	chr12	1.21E+08	1.21E+08	+	6081	1	MLEC|KIA	35.146	39.357
NM_1524	chrX	64185117	64205744	-	8439	1	AMER1|FA	6.081	6.354
NM_0163	chr1	47333797	47378839	+	2922	1	CMPK1|CK	18.453	21.699
NM_0250	chr11	62337302	62393415	+	2333	1	ASRGL1|A	20.714	23.778
NR_03071	chr19	7920982	7923251	+	1442	1	SNAPC2|P	17.302	22.027
NR_03036	chr17	17813836	17813931	-	95	1	MIR33B|M	0	0
NM_0010	chrY	22168774	22183008	-	1700.5	2	RBMY1J|-|	0	0
NR_13626	chr19	54532692	54545741	+	3495.5	4	KIR3DX1|K	0	0
NR_02676	chr1	2.11E+08	2.11E+08	+	752	1	LINC00467	2.726	3.206
NM_0065	chr9	19049374	19051023	+	1647	1	RRAGA|FI	82.41	102.208
NM_0333	chr19	51491227	51500393	-	1859	1	SIGLEC12|	0	0
NM_0011	chr10	98457077	99235875	-	4162	1	HPSE2|HP	0.003	0
NM_0002	chr4	88007647	88077779	+	5062	1	PKD2|APK	2.41	3.123
NM_0179	chr9	4679553	4708398	+	3506	1	CDC37L1|	3.685	5.383
NM_0011	chr1	1.63E+08	1.63E+08	+	3048	1	RGS4|RGP	5.876	10.933
NM_0013	chr3	1.84E+08	1.84E+08	+	3513	1	VWA5B2|-	1.215	1.813
NR_13616	chr2	558201	562092	+	2197	1	LOC10537	0	0
NM_0012	chr14	73478484	73558947	-	3531	1	HEATR4|-|	0.252	0.303
NR_10802	chr5	1.74E+08	1.74E+08	-	737	1	LINC01485	0	0
NM_0178	chr1	1.86E+08	1.86E+08	+	3880	1	C1orf27|C	1.615	2.429
NR_00370	chr6	8086408	8086533	-	125	1	SCARNA27	0.206	0.657
NR_03399	chr7	1.39E+08	1.39E+08	-	457	1	LOC10012	30.724	31.59
NM_1526	chr4	1.65E+08	1.65E+08	+	1837	1	TRIM60|R	0	0
NM_0012	chr2	1.11E+08	1.11E+08	-	3439	1	BUB1|BUE	1.796	2.557
NM_0024	chr10	1.29E+08	1.3E+08	+	1365	1	MGMT|-|-	1.681	3.502
NR_00001	chr19	17862588	17862720	+	132	1	SNORA68|	1.563	2.387
NM_0069	chr17	75167730	75183003	-	1060	1	SUMO2|H	58.738	62.774
NM_0013	chr5	33523535	33892192	-	8495	1	ADAMTS1	0.507	0.416
NM_0011	chr22	30524930	30546682	-	1248	1	SEC14L6|-	0.196	0
NM_0002	chr13	27920031	27926314	+	2569	1	PDX1|GSF	0	0.027
NR_12049	chr12	81279189	81312422	+	963	1	LOC10272	0.134	0.199
NR_02942	chr7	56423704	56448375	-	1661	1	LOC65022	0	0

NR_02960	chr15	88611825	88611934	+	109	1	MIR7-2|M	0	0
NM_0154	chr3	45224464	45226322	-	1856	1	TMEM158	28.204	19.608
NR_00303	chr11	93731503	93731573	-	70	1	SNORD6|r	1.658	1.174
NR_13619	chr4	1.74E+08	1.74E+08	+	4341	1	HAND2-AS	0	0
NM_0323	chr7	73680918	73683451	-	2531	1	DNAJC30|	6.786	6.251
NM_0012	chr17	47844914	47851150	-	3801	1	SP6|EPFN	0.526	0.238
NR_04640	chr21	46462471	46469305	+	423	1	DIP2A-IT1	0.061	0.065
NR_00276	chr14	1.01E+08	1.01E+08	+	1585	1	MEG3|FP5	0.155	0.104
NM_1303	chr9	8314246	8733946	-	8191	1	PTPRD|HP	19.27	12.083
NM_0050	chr21	5130871	5154730	-	3237	2	PWP2|EH	1.067	1.159
NR_13197	chr1	1.51E+08	1.51E+08	+	437	1	GBAT2|-|-	11.684	13.885
NM_0148	chr4	1.45E+08	1.45E+08	-	1450	1	ANAPC10|	2.472	4.204
NM_0322	chr10	98247686	98268250	-	3640	1	LOXL4|LO	0.039	0.053
NR_10688	chr3	1.28E+08	1.28E+08	-	65	1	MIR6825|	4.166	4.425
NR_11069	chr1	1.63E+08	1.63E+08	+	2629	1	LOC10192	0.01	0.021
NM_0045	chr17	48607233	48611021	-	1362	1	HOXB7|HH	0.019	0.161
NM_1779	chr9	1.3E+08	1.3E+08	-	4506	1	ASB6|-|-|	18.402	22.628
NR_00000	chr11	75400391	75400538	+	147	1	SNORD15A	0.263	0.28
NR_13614	chr10	43912402	43914506	+	823	1	LINC00841	0	0
NM_0038	chrX	77447675	77456522	+	620	1	FGF16|FG	0	0
NM_0010	chr19	4522531	4535196	-	2432	1	PLIN5|LSD	0.726	2.011
NM_0011	chr6	83166130	83193293	-	2059	1	PGM3|AG	7.12	7.218
NM_0013	chr16	18983934	19063940	+	4368	1	TMC7|-|-|	1.488	1.489
NR_03644	chr15	82725898	82739780	-	1649	1	ACTG1P17	0.149	0.274
NM_0011	chrX	48897930	48903143	+	927	1	PQBP1|MI	109.045	97.859
NR_12534	chr19	46057678	46077629	-	3224	1	LOC40070	0.304	0.191
NM_0155	chr16	30357101	30370201	-	3277	1	TBC1D10B	41.281	34.947
NM_0144	chr3	29281312	29991318	+	1723	1	RBMS3|-|	1.908	0.636
NM_1989	chr19	14799174	14800136	-	961	1	OR7C1|CI	0	0
NM_0012	chr6	2948159	2971309	-	1464	1	SERPINB6|	11.15	7.307
NM_1984	chr19	45149750	45160150	-	4682	1	NKPD1|-|-	0.284	0.377
NM_1384	chr13	76880169	76886405	-	6234	1	KCTD12|C	6.805	4.047
NM_1783	chr1	1.53E+08	1.53E+08	-	691	1	LCE1C|LEP	0	0
NM_0010	chr1	32209094	32216196	+	1338	1	DCDC2B|-	0.858	0.655
NM_0182	chr9	33041852	33076716	-	7077	1	SMU1|BW	8.086	8.311
NM_0206	chr16	401858	412487	+	1573	2	DECR2|PD	0.016	0
NR_10994	chr5	15191646	15266560	-	1067	1	LINC0214	0	0
NM_0068	chr5	1.77E+08	1.77E+08	-	1822	1	LMAN2|C5	53.945	48.067
NM_0175	chr10	85599555	86366493	-	5831	1	GRID1|Glu	2.784	2.307
NM_0012	chr19	50996008	51001702	-	846	1	KLK8|HNP	0	0
NR_12039	chr13	1.11E+08	1.11E+08	-	4069	1	LOC10106	0.222	0.289
NM_0011	chr13	36301638	36370180	-	4811	1	SPG20|SP	5.387	6.671
NR_02462	chr21	32772100	32797706	+	1186	2	C21orf62-	0	0.035
NM_0181	chr19	17719452	17734515	+	3309	1	MAP1S|BF	32.088	29.89
NM_0157	chr19	10013249	10022278	+	1706	1	RDH8|PRR	0.008	0.008
NM_0028	chr3	1.57E+08	1.57E+08	+	1933	1	PTX3|TNF	9.999	11.132
NM_0180	chr7	4799109	4883704	-	3653	1	RADIL|RA	12.283	8.489
NM_0012	chr12	55725443	55727900	-	797	1	CD63|LAN	386.578	266.327
NM_0220	chr22	21642253	21644299	+	869	1	SDF2L1|-|	6.187	6.257
NM_0221	chr1	78889764	79006810	-	3602	1	ADGRL4|E	0.007	0.053

NR_13275	chr19	12703597	12703671	-	74	1	SNORD135	0.348	0.185
NR_03623	chr2	79649294	79649359	-	65	1	MIR4264|-	0	0
NR_03740	chr8	1.17E+08	1.17E+08	-	72	1	MIR3610|	1.791	0.761
NM_0049	chr20	49363877	49482644	-	11843	1	KCNB1|DR	5.04	4.875
NR_12598	chr1	49257411	49269294	+	571	1	LOC10192	0	0
NR_03409	chr11	10541236	10599932	+	658	1	MRVI1-AS1	1.058	0.479
NM_0323	chr1	15617458	15661057	+	2668	1	DDI2|-|-|1	3.127	3.63
NM_0011	chrX	18170936	18220904	-	2118	1	BEND2|CX	0	0
NR_03165	chr17	81133196	81133308	-	112	1	MIR1250|	0.23	0
NM_0011	chr2	2.24E+08	2.24E+08	-	2465	1	FAM124B|	0.01	0.028
NM_0010	chr5	81233320	81266398	+	1470	1	CKMT2|SM	0.096	0.168
NM_0012	chr14	23953735	23969281	+	935	1	DHRS4|CR	3.227	2.886
NR_02734	chr7	29684772	29685821	-	1049	1	ZNRF2P2|-	0.91	1.018
NM_0123	chr12	49866896	49903977	-	4730	1	FAIM2|LF	21.316	31.836
NR_00258	chrX	1.2E+08	1.2E+08	-	131	1	SNORA69|	0.591	0.209
NM_0011	chr18	58149333	58401540	+	8286	1	NEDD4L|N	36.911	45.963
NM_0189	chr7	1.42E+08	1.42E+08	+	1147	1	TAS2R5|T2	0.652	0.74
NR_12577	chr2	2.18E+08	2.18E+08	-	1179	1	CATIP-AS2	0.011	0
NM_0305	chr8	1.45E+08	1.45E+08	-	2824	1	ZNF34|KO	7.812	7.508
NM_0011	chr11	36272292	36289449	-	2841	1	COMMD9|	24.739	26.116
NM_0010	chr11	10573091	10652301	-	6013	1	MRVI1|IRA	1.194	1.38
NM_1985	chr15	90001491	90082200	+	4511	1	ZNF710|-|	14.697	12.719
NM_0012	chr19	53431974	53458261	+	3986	1	ZNF761|ZN	1.265	2.471
NM_0035	chr7	66205272	66360451	+	2043	1	TPST1|TAN	17.778	21.715
NM_0010	chr11	56105043	56105981	+	937	1	OR8H2|OF	0	0
NR_10698	chr8	41660444	41660507	+	63	1	MIR486-2|	0	0
NM_0009	chr8	53225716	53251697	-	5016	1	OPRK1|K-C	2.478	1.562
NR_11058	chr2	1.6E+08	1.6E+08	+	2451	1	LOC64307	1.604	2.096
NR_02741	chrX	1.54E+08	1.54E+08	+	1597	1	AVPR2|AD	0.178	0.094
NM_0015	chr5_GL33	965419	997916	+	1933	5	GTF2H2|B	0.22	0.305
NM_0051	chr4	93828927	93829991	+	1063	1	ATOH1|AT	66.517	2.757
NM_1990	chr21	41885110	41926690	-	5831	1	C2CD2|C2	1.411	1.116
NR_00244	chr15	66501252	66501318	-	66	1	SNORD18C	1.172	2.075
NM_0012	chr8	1.42E+08	1.42E+08	-	3191	1	TSNARE1|-	5.738	5.413
NM_0008	chr3	49024329	49029442	-	1675	1	IMPDH2|II	145.931	121.899
NM_0145	chr2	1.9E+08	1.9E+08	-	3345	1	SLC40A1|F	0.648	1.47
NR_00303	chr11	93733228	93733300	-	72	1	SNORD5|r	1.433	1.712
NM_0064	chr12	69585428	69601577	+	1936	1	CCT2|99D	183.486	192.713
NR_10702	chr16	82688931	82689019	+	88	1	MIR8058|	0	0
NM_0011	chr7	12211222	12237264	+	6310	1	TMEM106	3.797	5.928
NM_0011	chr11	22846923	22860426	-	793	1	CCDC179|	0	0
NM_0013	chr6	30983732	30988548	+	2274.3	7	MUC21|C6	0	0
NM_0012	chr15	99136323	99251226	-	3287	1	TTC23|HC	3.903	4.759
NM_0190	chr1	1.66E+08	1.66E+08	-	4461	1	TMCO1|HI	17.512	14.689
NM_0011	chr9	1.38E+08	1.38E+08	+	1269	1	FAM157B|	0	0
NR_03617	chr10	1.25E+08	1.25E+08	-	87	1	MIR4296|-	0	0
NR_11059	chr4	62071755	62161765	-	459	1	ADGRL3-A	0.309	0.298
NM_0012	chr14	20455131	20457772	+	1541	1	APEX1|AP	159.232	160.044
NR_03625	chrX	50010672	50010750	+	78	1	MIR500B|	0	0
NR_12118	chr9	74952965	74997380	+	1804	1	C9orf41-A	0.043	0.175

NM_0012	chr10	62049211	62096948	+	7016	1	ARID5B|D	2.665	5.115
NM_1526	chr1	75206390	75611114	-	2495	1	SLC44A5|	1.586	5.863
NM_0012	chr10	97222173	97292673	-	5380	1	ARHGAP1	5.117	5.074
NM_0010	chr1	2.49E+08	2.49E+08	-	946	1	OR2T29|-|	0	0
NM_0010	chr6	1.37E+08	1.37E+08	+	2399	1	SLC35D3|	0.011	0.011
NM_0010	chr11	56093308	56094240	+	931	1	OR8I2|OR	0	0
NM_0320	chr10	44959771	44994724	+	2482	1	RASSF4|A	24.68	28.785
NM_1985	chr12	1.03E+08	1.03E+08	-	1372	1	C12orf42|	0.056	0.02
NR_00272	chrX	15785716	15787589	-	1873	1	INE2|NCR	0.496	0.673
NM_0007	chr19	49022869	49024375	-	914	1	CGB3|CGB	0.028	0
NM_0212	chr14	77265491	77271312	-	1870	1	NGB|-|-|1	0.476	1.187
NM_0032	chr4	1.86E+08	1.86E+08	+	3034	1	TLR3|CD2	0.004	0.027
NM_0016	chr12	48936209	48957469	-	3530	1	ARF3|-|-|	80.046	89.488
NM_0012	chr9	37753803	37778972	+	2176	1	TRMT10B|	5.961	5.785
NM_0054	chrX	50910735	50916641	+	1258	1	BMP15|G	0	0
NR_12056	chr11	46171955	46177105	+	930	1	LOC10192	0	0
NM_0143	chr19	48872392	48876062	+	2378	1	PPP1R15A	122.515	104.657
NR_02401	chr6	40378424	40379892	+	1083	1	TDRG1|LI	0.083	0.139
NM_1527	chr11	60756867	60776731	+	1664	1	MS4A15|-	0	0
NM_0230	chr5	37106228	37249428	-	11145	1	C5orf42|H	1.772	2.762
NM_0025	chr11	73218298	73242427	+	8597	1	P2RY2|HP	0.075	0.035
NM_0001	chr9	95099054	95317709	-	4575	1	FANCC|FA	6.423	6.344
NM_0046	chr20	603993	610266	-	1223	1	TCF15|EC	0.221	0.437
NR_11069	chr1	96254069	96374125	-	1988	1	LINC01787	0	0
NR_03393	chr4	1.4E+08	1.4E+08	-	2012	1	SCOC-AS1|	0.692	0.347
NM_0013	chr3	86937969	86991123	-	10251	1	VGLL3|VG	0.37	0.211
NM_0227	chr1	37489961	37514819	-	4807	1	MEAF6|C1	31.168	30.201
NM_0247	chr1	37534449	37554344	-	4599	1	SNIP1|PM	5.725	6.677
NM_0145	chr1	93869458	93879206	-	2713	1	DNTTIP2|	6.863	10.218
NM_0156	chr20	62183025	62202754	+	2927	1	MTG2|GT	8.577	9.855
NR_10408	chr1	1.48E+08	1.48E+08	-	163	1	RNVU1-1|	0	0.168
NM_1536	chr16	89575768	89597246	+	2415	1	CPNE7|-|-	0.988	1.083
NM_0044	chrX	1.1E+08	1.1E+08	-	5005	1	ACSL4|AC	8.447	8.106
NR_13430	chr8_KI27	174621	175992	-	1276	2	LOC10192	0	0
NM_1451	chr14	63312835	63318879	-	538	1	GPHB5|B5	0	0
NM_0011	chrX	24362761	24365424	+	2662	1	SUPT20HL	0	0.01
NM_0012	chr9	1.33E+08	1.33E+08	-	2318	1	SURF6|RR	14.356	13.715
NR_13077	chr5	67793213	67801058	+	1554	1	LOC10192	0	0
NM_0013	chrY	21511338	21525554	+	1565	4	RBMY1B|-	0	0
NM_0013	chr16	20733666	20741964	-	3993	1	THUMPD1	5.289	5.571
NM_0010	chr21	6484623	6499256	-	943	2	U2AF1|FP	0	0
NM_0012	chr17	43527843	43545937	-	2134	1	ETV4|E1A-	14.253	4.044
NM_0328	chr16	69132596	69169034	+	1929	2	UTP4|CIR	5.688	7.086
NR_03334	chr2	2.42E+08	2.42E+08	-	824	1	BOK-AS1|	0.36	0.399
NM_0011	chr17	48070052	48101521	-	2225	1	CBX1|CBX	101.473	104.805
NR_13562	chr14	35762794	35762925	-	131	1	SNORA101	0	0
NM_0012	chr20	38472806	38578861	+	8499	1	RALGAPB|	10.918	13.523
NM_0073	chr1	21976894	21989354	+	1024	1	CELA3B|C	0	0
NM_1488	chr12	56923154	56934405	-	1759	1	SDR9C7|R	0.088	0.093
NR_03379	chr7	1E+08	1E+08	-	2298	1	TAF6|ALY	51.821	47.463

NM_0151{	chr9	77177445	77385190	+	9984	1	VPS13A|CI	0.765	1.539
NM_2015	chr1	54138995	54153006	-	1953	1	CDCP2|-|-	0.158	0.042
NR_12596	chr1	1.46E+08	1.46E+08	+	741	1	LINC0171S	1.688	1.46
NR_04561	chr6	27324761	27325962	+	1201	1	VN1R10P|	0	0
NM_0033{	chr2	1.75E+08	1.75E+08	-	4575	1	WIPF1|PRI	1.93	1.775
NM_2033	chr1	2.05E+08	2.05E+08	-	1329	1	TMEM81|	6.19	5.916
NM_0205	chr14	35401510	35404754	-	1559	1	NFKBIA|IK	15.209	18.476
NM_0013	chr19	56507843	56530214	+	6147	1	ZNF471|EI	1.825	2.199
NM_0010(	chr17	82942149	83051810	-	3010	2	B3GNTL1|	0.334	0.132
NM_0012{	chr17	18327860	18363563	-	2424	1	SHMT1|CS	5.798	4.916
NM_0019	chr19	859659	863569	+	1117	2	CFD|ADIPS	0	0
NR_11060	chr2	2.1E+08	2.1E+08	+	2136	1	LANCL1-AS	0.157	0.128
NR_13431	chr8	85172077	85177041	-	484	1	LOC10272	0.08	0.057
NM_1814	chr17	74609883	74623758	-	3519	1	CD300E|C	0	0
NM_0068	chr11	64186115	64204548	+	2185	1	STIP1|HEL	149.694	164.164
NM_0332(	chr16	23690305	23713500	-	3596	1	ERN2|IRE1	0.054	0.034
NM_1825(	chr14	98711613	98717766	+	1283	1	C14orf177	0	0
NM_0013(	chr6	75601509	75679867	+	3993	1	SENP6|SSI	3.491	5.248
NM_0013	chr10	1.12E+08	1.13E+08	+	4411	1	VTI1A|MN	7.17	7.723
NM_1775	chrX	54920563	54931431	+	2544	1	TRO|MAG	68.856	75.199
NM_0010	chr8	36784324	36936126	+	3714	1	KCNU1|KC	0.01	0.015
NR_10411	chr14	96943579	96945394	+	358	1	LINC00618	0.072	0.459
NM_0050	chr4	1.59E+08	1.59E+08	-	1822	1	PPID|CYP-	18.696	15.892
NM_0175	chr11	1.08E+08	1.08E+08	-	3160	1	SLC35F2|H	0.326	0.772
NR_00258	chr3	1.87E+08	1.87E+08	+	69	1	SNORD2|F	2.429	3.573
NR_12051	chr18	6511416	6590653	+	597	1	LINC01387	0	0
NR_03023	chrX	1.47E+08	1.47E+08	-	73	1	MIR510|M	0	0
NM_0011	chr7	31751179	32070862	-	4784	1	PDE1C|Hc	0.267	4.174
NM_2073	chr11	67289291	67302484	+	2123	1	ANKRD13D	34.18	29.884
NM_0160(	chr4	83455932	83461723	+	1093	1	MRPS18C|	10.192	11.078
NR_04989	chr15_KI2	271844	271925	+	81	5	MIR5701-2	0	0
NR_05198	chr13	32496211	32498492	-	2281	1	MINOS1P1	0.04	0.03
NM_0013	chr16	1776738	1781822	-	1487	1	SPSB3|C16	53.646	51.96
NR_02950	chrX	1.34E+08	1.34E+08	-	74	1	MIR92A2|	0	0
NR_03030	chr4	38868032	38868127	+	95	1	MIR574|M	0	0
NM_0012{	chr17	42288056	42311942	+	4101	1	STAT5A|M	0.358	0.13
NM_1826	chr2	1.05E+08	1.05E+08	+	1445	1	MRPS9|M	15.285	14.161
NM_0050	chr11	17276739	17331523	+	1596	1	NUCB2|HE	12.522	12.332
NM_0015(	chr4	76021116	76023536	-	1210	1	CXCL10|C	0.021	0
NR_10690	chr8	1.44E+08	1.44E+08	-	68	1	MIR6849|	7.205	7.453
NM_0174	chr20	49113339	49188370	-	3309	1	STAU1|PP	55.693	59.557
NR_10682	chr16_KI2	738858	738917	-	59	5	MIR6770-1	0	0
NM_1307	chrX	52369010	52375683	+	634	1	XAGE2|CT	0	0
NR_13641	chr17	32141226	32143135	-	1597	1	LOC10537	1.405	1.003
NM_1526	chr2	2.32E+08	2.32E+08	+	1417	1	TEX44|C2c	0	0
NR_13518	chr1	2.06E+08	2.06E+08	-	2109	1	PM20D1|(	0.024	0.019
NM_0010(	chr11	58214745	58215722	+	976	1	OR1S1|OR	0	0
NR_12601	chr21	28048414	28137609	+	1747	1	LINC01697	0	0
NM_0040	chr10	1.32E+08	1.32E+08	-	1625	1	BNIP3|NIP	35.967	11.784
NM_0251	chr13	39009865	39038076	-	5154	1	PROSER1|	11.415	12.012

NR_03978	chr6	44435641	44435722	+	81	1	MIR4642|-	0	0
NR_10412	chr4	1.23E+08	1.23E+08	+	3737	1	IL21-AS1|-	0	0.004
NM_0001	chr2	26190635	26244726	-	3015	1	HADHA|EC	57.903	69.453
NR_02938	chr8	6968141	6969113	-	289	1	DEFA10P|-	0	0
NM_0012	chr6	1.33E+08	1.33E+08	-	1754	1	VNN3|HSA	0	0
NR_03885	chr15	26115813	26133037	+	718	1	LINC0092	0	0
NM_0166	chr15	50285880	50355408	-	1602	1	GABPB1|B	8.797	9.029
NM_0164	chr3	14651746	14672659	+	2947	1	CCDC174|	6.777	7.571
NM_0058	chr1	1.81E+08	1.81E+08	-	5063	1	STX6|-|-|1	11.671	18.537
NM_0003	chr8	63059489	63086053	-	2625	1	TTPA|ATT	0.074	0.063
NM_0161	chr13	37636636	37869802	-	3423	1	TRPC4|HT	1.16	0.492
NM_2071	chr1	1.51E+08	1.51E+08	-	6134	1	POGZ|MR	30.737	36.951
NM_0011	chr9	34723053	34729538	-	4292	1	FAM205A|	0.006	0.006
NM_0011	chr17	49132769	49169989	+	1793	1	B4GALNT2	0	0
NM_0226	chr8	25841730	26045124	-	4854	1	EBF2|COE	12.301	11.369
NM_0322	chr8	1.44E+08	1.44E+08	+	1765	1	MAF1|-|-|	71.502	72.924
NR_02725	chr8	93740111	93740819	+	708	1	RBM12B-A	1.293	1.548
NR_10696	chr6	35470508	35470579	+	71	1	MIR7111|	2.906	1.543
NM_0042	chr3	1.43E+08	1.43E+08	+	4133	1	CHST2|C6	10.448	11.374
NR_03785	chr6_GL0	3007552	3024174	-	2154.5	8	ATP6V1G2	0.03	0.076
NR_02410	chr21	41141500	41148064	-	2017	1	LINC00323	0	0.007
NM_0159	chr15	31326855	31377899	+	6823	2	KLF13|BTE	0.826	0.783
NM_0046	chrX	1.54E+08	1.54E+08	+	1337	1	FAM50A|9	71.883	67.205
NM_2013	chr1	2.03E+08	2.03E+08	+	5804	1	PRELP|MS	0.047	0.172
NM_0053	chr11	5268350	5270143	-	811	1	HBE1|HBE	0	0
NM_0054	chrX	1.42E+08	1.42E+08	+	4315	1	MAGEC1|	0	0
NM_0156	chr4	17632086	17781512	-	3822	1	FAM184B|	1.299	1.358
NR_12592	chr4	99950494	1E+08	+	494	1	LOC25688	0.313	0
NR_03332	chr22	26657482	26676477	+	9979	1	MIAT|C22	120.429	56.743
NM_0035	chr1	1.5E+08	1.5E+08	-	531	2	HIST2H2A	0	0
NR_04769	chr2	2.15E+08	2.15E+08	+	476	1	VWC2L-IT1	0	0
NM_1735	chr4	46035770	46124065	-	6757	1	GABRG1|-	0.004	0.071
NR_13196	chr7	1.2E+08	1.2E+08	-	1578	1	LVCAT5|-|	0.025	0
NR_03617	chr22	48274364	48274415	+	51	1	MIR3201|-	0	0
NM_0011	chr7	1.29E+08	1.29E+08	+	5043	1	AHCYL2|A	13.164	12.502
NM_0054	chr17	34356452	34358610	+	846	1	CCL13|CK	0	0
NM_0012	chr10	27110111	27154420	-	4108	1	YME1L1|F	26.255	23.84
NM_0011	chr6	1.22E+08	1.22E+08	+	2629	1	HSF2|HSF	16.297	21.486
NM_0066	chr1	11026523	11047239	-	2442	1	MASP2|M	1.341	1.542
NR_00296	chr13	45337480	45337609	-	129	1	SNORA31|	2.499	3.61
NM_0312	chr9	1.37E+08	1.37E+08	-	1074	1	RNF208|-|	19.304	14.781
NM_0209	chrX	76427653	76431353	+	3698	1	MAGEE1|I	11.101	14.942
NR_13093	chr11	77571089	77575662	+	1842	1	LOC64602	0	0
NM_0132	chr16	69311384	69325043	+	2163	1	VPS4A|SK	92.49	108.348
NM_0011	chr19	1554669	1568058	-	2659	1	MEX3D|M	22.024	22.253
NM_0122	chr10	52314281	52317657	+	1799	1	DKK1|DKK	0.702	0.35
NM_1784	chr9	1.37E+08	1.37E+08	-	3825	1	SAPCD2|C	5.67	6.209
NM_1390	chr5	1.8E+08	1.8E+08	-	4326	1	MAPK9|JN	14.977	17.762
NR_11077	chr18	11490048	11506983	+	523	1	LINC0125	0	0
NM_0151	chr11	64894532	64917250	-	6330	1	ATG2A|-|-	6.274	6.067

NM_0227:	chr19	11355386	11365698	+	2715	1	PLPPR2|LF	84.907	85.512
NM_0013(	chr11	1.17E+08	1.17E+08	+	4084	1	PAFAH1B2	34.747	45.843
NR_00369	chr14	45110875	45110973	+	98	1	SNORD127	0	0
NM_0010(	chr12	55320600	55321538	+	937	1	OR6C1|OS	0	0
NM_1785!	chr7	64519877	64563127	-	3044	1	ZNF680|-|	0.618	1.404
NM_0012(	chr14	74941830	74955764	-	1840	1	PGF|D12S	2.018	0.409
NM_0013(	chr7	26639085	26647270	+	1291	1	C7orf71|-|	0.02	0
NM_1985(	chr3	1.43E+08	1.43E+08	-	2104	1	PAQR9|-|-	0.404	0.605
NR_12587	chr6	4186320	4190029	-	801	1	LOC10272	0	0
NR_10675	chr12	66251082	66251157	+	75	1	MIR6502|	0	0
NM_0027!	chr7	42916861	42932206	-	1456	1	PSMA2|H(	82.488	84.769
NR_00219	chrX	42777366	42778235	-	869	1	PPP1R2P9	0	0
NM_0011!	chr10	1.11E+08	1.11E+08	+	3558	1	PDCD4|H7	7.61	9.651
NM_0066(	chr19	45379634	45405054	-	3105	1	PPP1R13L	1.57	1.032
NM_0206!	chr20	19212646	19722897	+	3870	1	SLC24A3|I	3.412	8.586
NR_03650	chr7	32758286	32762924	+	4638	1	LINC00997	0.595	0.437
NR_10466	chr5	56457795	56481769	-	651	1	LINC01948	0	0.021
NM_1522!	chr1	45583988	45618906	+	2258	1	NASP|FLB	44.973	40.133
NM_0011(	chr2	1.91E+08	1.91E+08	+	5152	1	MYO1B|M	12.065	9.632
NR_12585	chr6	1.37E+08	1.37E+08	+	759	1	LOC10192	0	0
NR_03825	chr12	1.27E+08	1.27E+08	+	2255	1	LINC00943	0.006	0.14
NM_1537	chr1	1.55E+08	1.55E+08	-	381	1	DPM3|CD	34.416	22.433
NM_0068(	chr8	42152946	42171183	+	3600	1	AP3M2|AF	22.27	26.081
NM_0011!	chrX	1.54E+08	1.54E+08	+	2088	1	ATP6AP1|	131.38	116.7
NM_0149!	chr10	62374157	62402458	+	4201	1	ZNF365|Su	0.666	1.255
NM_1818	chr9	1050620	1057554	+	2161	1	DMRT2|-|	0.054	0.07
NM_0011	chr4	2930561	2934859	-	1742	1	MFSD10|T	38.207	29.659
NR_04610	chr10	11071541	11105488	-	2270	1	CELF2-AS2	0	0
NM_0013	chrX	1.36E+08	1.36E+08	-	1206	3	CT45A9|-|	0	0
NM_0013!	chr18	34493290	34829328	+	1718	1	DTNA|D18	9.569	11.481
NM_0056	chr14	20781051	20782467	+	1057	1	RNASE6|R	0	0
NR_11051	chr22	37352191	37354839	-	1962	1	LOC10050	0.013	0.405
NM_1524	chr8	1.25E+08	1.25E+08	+	3270	1	ZNF572|-|	0.497	0.532
NM_2033	chr15	63597353	63602421	+	1515	1	FBXL22|Fb	0.17	0.217
NM_1994	chr11	1.3E+08	1.3E+08	-	6287	1	PRDM10|I	3.212	3.505
NM_0307!	chr19	45795710	45815347	-	2476	1	RSPH6A|R	0.047	0.066
NM_0336	chr18	65750252	65881966	+	3741	1	CDH7|CDI	3.509	0.714
NM_0006	chr4	99123657	99144298	-	1993	1	ADH4|ADI	0	0.021
NM_0071	chr17	57971549	57988254	-	4625	1	VEZF1|DB	18.104	15.871
NM_0018	chr19	39731253	39738029	-	627	1	CLC|GAL1(	0	0.066
NM_0011!	chr1	1.61E+08	1.61E+08	+	1510	1	NIT1|-|-|1	34.343	27.521
NR_10372	chr4	75479037	75509296	-	4159	1	RCHY1|AR	4.25	5.523
NM_0013	chr22	27187516	27189215	+	480	1	LOC10537	0	0
NM_0013	chr14	85530025	85628696	+	7809	1	FLRT2|-|-|	1.828	1.494
NM_0145(	chr22	39014260	39018820	+	1103	1	APOBEC3C	0.339	0.658
NM_0012(	chr4	81030965	81057625	+	5823	1	BMP3|BM	0.1	0.016
NM_1758!	chr21	30880645	30881555	-	908	1	KRTAP11-1	0	0.015
NR_13814	chr8_KI27(	93264	99654	-	3473	2	PYCRL|PY(	0	0
NR_10270	chrX	1.5E+08	1.5E+08	-	591	1	MAGEA8-/	0.044	0.116
NR_03982	chr10	87503881	87503954	+	73	1	MIR4678|-	0	0

NM_17851	chr11	1.13E+08	1.13E+08	+	2533	1	ANKK1|PK	0.107	0.049
NR_03405	chr4	13526319	13531417	-	2118	1	LINC01097	0	0
NM_0013	chr17	9802386	9822071	-	876	1	GSG1L2|-|	0	0
NM_0012	chr14	22986901	23001037	-	1821	1	C14orf93|	7.151	7.868
NM_1450	chr18	50227194	50266522	-	1840	1	CFAP53|C	1.444	1.094
NM_0049	chr16_KI2	1607547	1800965	+	6530	2	ABCC1|AB	0.316	0.298
NR_13239	chr9	1.2E+08	1.2E+08	+	2772	1	LINC01613	0	0
NM_0808	chr1	1.61E+08	1.61E+08	-	1140	1	ITLN2|HL-	0	0.096
NR_12030	chr16	25100564	25111555	-	570	1	LCMT1-AS	1.244	1.802
NM_0209	chr9	35736866	35749228	-	3592	1	GBA2|ADC	40.102	49.106
NM_0012	chr5	271621	314974	+	1041	1	PDCD6|AL	63.254	74.235
NR_02415	chr1	1.62E+08	1.62E+08	+	2492	1	HSPA7|HS	0.021	0.011
NM_0062	chr4	75859873	75902528	-	3394	1	PPEF2|PPI	0.038	0.008
NM_0011	chr4	2741660	2756025	-	1680	1	TNIP2|ABI	8.972	8.43
NM_0012	chr8	30638600	30645952	+	788	1	SMIM18|-	9.245	7.335
NR_13449	chr10	3833950	3834728	+	534	1	LOC10537	0	0
NM_0013	chr21	39445865	39515506	+	958	1	SH3BGR|2	6.46	9.622
NM_0211	chrX	49341211	49529981	+	490	3	GAGE7|AL	0	0
NM_0012	chr20	44751804	44810338	-	5202	1	RIMS4|C2	28.731	41.27
NM_0059	chr4	1.87E+08	1.87E+08	-	1285	1	MTNR1A|	0.09	0.053
NM_0161	chr19	2321521	2328615	-	514	1	LSM7|YNL	197.14	141.127
NR_10354	chr8	1.24E+08	1.24E+08	-	1743	1	FER1L6-AS	0.081	0.031
NM_0307	chr2	2.38E+08	2.38E+08	-	1429	1	ILKAP|ILK	31.571	31.094
NM_0324	chr7	74289475	74405943	+	5440	1	CLIP2|CLI	69.366	80.467
NM_1382	chr1	1.51E+08	1.51E+08	+	2148	1	BNIPL|BNI	0.324	0.414
NR_00334	chr15	25230838	25230919	+	81	1	SNORD115	0	0
NM_0010	chr5	1.42E+08	1.42E+08	-	5734	1	DIAPH1|D	22.356	19.244
NM_0011	chr14	69191477	69244020	+	4937	1	EXD2|C14	14.748	17.95
NM_0048	chrX	79360384	79367552	-	1826	1	ITM2A|BR	0.035	0.03
NM_0016	chr7	38383696	38631567	-	3403	1	AMPH|AN	34.448	47.1
NM_0000	chr17	4897769	4903074	-	2446	1	CHRNE|AC	1.866	1.887
NM_0013	chr11	31432399	31509628	-	733	1	IMMP1L|I	17.238	15.229
NR_03999	chr5	88268882	88436674	+	605	1	TMEM161	18.349	75.934
NM_0013	chr3	57757256	57930016	+	5375	1	SLMAP|SL	2.663	2.709
NM_0186	chr12	68746156	68766073	+	2340	1	SLC35E3|E	5.918	4.127
NM_0004	chr1	25959767	25998157	-	3568	1	PAFAH2|H	3.91	4.131
NR_13467	chr4	26859624	26860599	-	493	1	LOC10537	0.863	0.445
NR_03171	chr16	69565808	69565868	-	60	1	MIR1538|	0	0
NM_0011	chr4	15002674	15070153	+	6864	1	CPEB2|CP	1.599	2.662
NR_12067	chr10	1.03E+08	1.04E+08	-	685	1	NEURL1-A	0	0.2
NM_2016	chr5	1.34E+08	1.34E+08	+	2866	1	TCF7|TCF-	3.365	1.08
NM_0010	chr1	2.42E+08	2.42E+08	-	1186	1	MAP1LC3C	4.751	2.102
NM_0309	chr9	1.28E+08	1.28E+08	+	2643	1	URM1|C9	26.553	21.818
NM_0250	chr15	64815630	64825668	-	2674	1	PIF1|C15o	0.796	1.296
NM_0047	chr19	18593225	18606850	-	1793	1	CRLF1|CIS	10.966	6.799
NM_0013	chr15	82342756	82349475	-	1429	2	GOLGA6L1	0.072	0.029
NR_07343	chr13	23907284	23949315	-	5091	1	ANKRD20A	0.01	0.167
NR_04024	chr19	40890826	40900508	+	2506	1	CYP2G1P|	0.051	0.071
NM_1817	chr11	1.19E+08	1.19E+08	+	1145	1	FOXR1|DL	0.034	0.251
NM_0011	chr5	80650000	80651035	-	1033	1	MTRNR2L	0.487	0.411

NR_13122	chr5	79936579	79967626	+	558	1	LINC0145	0	0
NR_10386	chr8	1565510	1621664	-	437.5	4	DLGAP2-A	0	0
NM_0176	chr18	49043802	49460709	-	2609	1	DYM|DM	15.844	22.239
NM_0178	chr5	1.78E+08	1.78E+08	-	847	1	NHP2|DK	61.895	56.114
NM_1385	chr15	69414246	69448427	+	3698	1	KIF23|CH	1.21	1.77
NR_04756	chr15	28844961	28848675	-	1150	1	GOLGA6L7	0.067	0
NM_0010	chr9	1.23E+08	1.23E+08	-	955	1	OR1B1|OF	0.027	0.029
NR_00299	chrX	1.15E+08	1.15E+08	+	127	1	SNORA35|	0	0
NM_0010	chr1	24556076	24609327	+	3984	1	NCMAP|C	0.01	0
NR_12047	chr12	23175636	23191587	+	1636	1	LOC10192	0.008	0.033
NM_1384	chr2	68130149	68157560	-	2270	1	WDR92|-|	4.942	4.109
NM_0251	chr3	1.14E+08	1.14E+08	-	6153	1	NAA50|M	5.419	5.703
NM_0010	chr10	49679638	49694902	+	637	1	C10orf53|	0.04	0
NM_1525	chr3	1.19E+08	1.19E+08	+	1809	1	C3orf30|T	0	0
NR_13394	chr4	58524515	58536328	-	424	1	LOC10537	0	0
NR_02331	chr12	6943816	6943878	+	62	1	RNU7-1|R	2.911	3.314
NM_0021	chr20	31605283	31606514	+	989	1	ID1|ID|bH	45.16	2.922
NR_13297	chr2	1.11E+08	1.11E+08	-	99	1	SNORD132	0	0
NM_0041	chr9	1.12E+08	1.12E+08	+	1494	1	DNAJC25-	1.295	1.293
NM_0068	chr9	99222288	99230619	+	556	1	SEC61B|-|	115.346	127.387
NM_0062	chr13	95677139	95794989	+	5577	1	DNAJC3|A	1.776	2.083
NM_1991	chr13	25161679	25172167	-	9934	1	AMER2|FA	5.276	7.739
NM_0210	chr17	41377669	41382384	-	1704	3	KRT34|HA	0	0
NM_0055	chr9	5890909	5909822	+	1517	1	MLANA|M	0	0.036
NM_0331	chr11	5323298	5324352	-	1052	1	OR51B2|H	0	0
NM_1738	chr5	1.16E+08	1.16E+08	+	4599	1	LVRN|APC	0.126	0.143
NM_0204	chr3	1.8E+08	1.8E+08	-	1172	1	MRPL47|C	41.794	39.49
NR_13465	chr4	1.72E+08	1.72E+08	-	1469	1	LOC44105	1.404	2.331
NM_0013	chr2	2.41E+08	2.41E+08	-	5065	1	HDLBP|HB	92.484	101.718
NM_0218	chr10	67796669	67838179	-	1205	1	DNAJC12|	29.639	32.054
NR_10240	chr17	78484910	78503056	+	5157	1	DNAH17-A	0.005	0
NM_0013	chr3	1.13E+08	1.13E+08	+	4297	1	BOC|CDO	16.416	23.881
NR_13432	chr7_KI27	134324	135417	+	565	3	LOC10537	0	0
NM_0203	chr3	1.93E+08	1.93E+08	+	1057	1	HRASLS|A-	2.025	0.752
NM_0012	chr19_KI2	36995	218790	+	1454.6	39	KIR2DS4|C	0	0
NM_0021	chr5	1.77E+08	1.77E+08	-	3047	1	HK3|HKIII|	0.262	0.463
NM_0012	chrX	47145218	47186815	+	3146	1	RBM10|D	52.316	43.42
NM_0012	chr17	39628498	39636625	+	1410	1	PPP1R1B|	2.469	12.9
NM_0011	chr3	52246170	52250599	+	1820	1	PPM1M|P	3.953	4.425
NR_03166	chr3	1.64E+08	1.64E+08	-	85	1	MIR1263|	0	0
NM_0011	chr16	75229030	75266809	-	3169	1	BCAR1|CA	39.327	31.915
NR_03822	chr10	75296383	75361678	+	1522	1	ZNF503-AS	0.008	0
NM_0027	chr12	49002272	49018846	-	1715	1	PRKAG1|A	59.769	62.863
NM_2058	chr9	1.11E+08	1.11E+08	-	949	1	OR2K2|HS	0	0.072
NM_0011	chr9	1.36E+08	1.36E+08	+	2051	1	GPSM1|A	64.078	67.61
NR_11080	chr17	57522248	57523597	-	560	1	LOC10192	0	0
NM_0011	chr6	33289635	33290934	+	695	5	PFDN6|H2	0	0
NM_0022	chr12	26335337	26833198	-	12524	1	ITPR2|ANI	0.609	0.372
NM_0035	chr6	26225155	26225616	+	459	1	HIST1H3E|	0.843	1.82
NR_10696	chr3	1.23E+08	1.23E+08	+	85	1	MIR7110|	1.517	0.645

NM_0154:	chr20	36891824	36951843	-	3171	1	SAMHD1|(	4.103	5.214
NM_0808:	chr20	227258	229886	+	694	1	DEFB129|(	0	0
NR_04669	chr3	13476988	13480053	-	507	1	HDAC11-A	0.203	0.027
NR_02997	chr17	28861369	28861440	-	71	1	MIR451A|	0	0
NM_0227!	chr3	1.24E+08	1.24E+08	-	4168	1	CCDC14|-|	4.857	7.397
NR_02995	chrX	1.34E+08	1.34E+08	-	68	1	MIR20B|M	0	0
NM_0035	chr6	26204645	26205021	+	374	1	HIST1H4E|	2.206	1.648
NM_0010:	chr6	1.48E+08	1.48E+08	+	6320	1	SAMD5|dJ	5.284	1.257
NR_12161	chr4	94743668	94757533	-	640	1	BMPR1B-A	0	0.064
NR_04984	chr11	46753125	46753192	-	67	1	MIR5582|	0	0
NM_0012	chr14	94110733	94116699	+	500	2	IFI27|FAM	0	0
NR_03164	chr10	54607874	54607957	-	83	1	MIR548F1	0	0
NM_0011	chr1	47760528	47996890	-	7020	1	TRABD2B|	0.617	0.14
NM_0010(	chr17	81833492	81835050	-	794	1	PPP1R27|I	0.097	0.121
NM_0012(	chr19	45995461	46018616	+	1807	1	CCDC61|-|	2.433	2.031
NM_0529	chr10	37125857	37232567	+	4367	1	ANKRD30A	0	0
NM_0149	chr16	77190919	77199646	+	3324	1	MON1B|H	12.389	9.906
NM_0012	chr6_GL00	3459484	3473740	+	5384	9	C4B_2|-|-	0	0
NM_0010(	chr2	1.06E+08	1.06E+08	+	2401	1	NCK2|GRB	41.408	48.541
NM_0330	chr12	55757270	55817756	-	917	1	SARNP|CIF	60.39	60.404
NM_0010	chr4	7430294	7434973	-	4677	1	PSAPL1|-|	0.033	0
NM_0010:	chr11	11962996	12008639	-	2569	1	DKK3|REIC	72.327	110.051
NR_04641	chr15	1.02E+08	1.02E+08	+	2049	1	OR4F13P|	0	0
NM_0008:	chr12	13561476	13980088	-	5926	1	GRIN2B|EI	1.236	3.449
NM_0013(	chr11	1.11E+08	1.11E+08	-	5403	1	COLCA1|C	0.086	0.046
NM_0011	chr10	21534645	21743630	+	4830	1	MLLT10|A	8.641	8.349
NM_1825	chr19	463346	474983	-	1598	1	ODF3L2|C	0.097	0.086
NR_03790	chr3	1.55E+08	1.55E+08	-	792	1	LOC10050	0.065	0.138
NM_0206:	chr12	4368227	4379728	-	3013	1	FGF23|AD	0.009	0.014
NM_1534	chr19_KI2	707066	719117	+	1471.3	34	KIR3DL3|C	0	0
NR_02963	chr5	1.69E+08	1.69E+08	-	109	1	MIR218-2|	0	0
NM_0003	chr11	62612741	62615121	+	1874	1	ROM1|RO	1.018	2.514
NM_1736(	chr5	1.76E+08	1.76E+08	+	2430	1	ARL10|ARI	11.806	16.932
NR_02682	chr3	10284419	10285746	+	1327	1	LINC00852	0.311	0.485
NM_0308(	chr12	51061205	51083671	-	4585	1	CSRNP2|C	40.593	55.89
NM_0010	chr16	3457985	3486963	+	2626	1	NAA60|HA	23.018	30.101
NR_02148	chr7	1570073	1589625	+	3853	1	PSMG3-AS	2.67	3.704
NM_0143	chr11	1.34E+08	1.34E+08	+	2188	1	ACAD8|AC	15.952	22.743
NM_0013	chr12	45729709	45893916	+	5664	1	ARID2|BAI	6.927	7.627
NM_2032	chr5	1.81E+08	1.81E+08	-	2634	1	TRIM7|GN	0.984	2.179
NR_11024	chr2	1.5E+08	1.5E+08	+	858	1	LOC10192	1.713	3.48
NR_03984	chr1	18883202	18883275	-	73	1	MIR4695|	0	0
NM_0011(	chr9	97501180	97601743	+	3249	1	TMOD1|D	8.469	13.861
NM_0067	chr11	60971641	61020376	+	3275	1	CD6|TP12	0.047	0.071
NR_03823	chr2	2.31E+08	2.31E+08	+	1889	1	LOC15148	0.526	0.399
NM_1733!	chrX	52644061	52654900	-	1330	1	SSX7|-|-|X	0	0
NM_0011	chr5	58969041	59356901	-	7725	1	PDE4D|AC	8.01	6.777
NM_0013:	chr11	1.2E+08	1.2E+08	-	2357	1	TRIM29|A	0.016	0
NR_02691	chr3	1.52E+08	1.52E+08	+	1286	1	AADACP1|	0	0
NR_00282	chr15	22494837	22590831	+	6039	3	HERC2P2|	0.589	1.109

NM_1988	chr12	1.31E+08	1.31E+08	+	5362	1	ADGRD1|	0.277	0.255
NM_0011	chr17	7259903	7263193	-	1435	1	CLDN7|CE	0.054	0.067
NM_1387	chr7	23597379	23644708	+	2467	1	CCDC126|	2.143	2.504
NR_13520	chr2	1.32E+08	1.32E+08	+	1616	1	FAM201B|	0.016	0.28
NR_12641	chr13	77599756	77606551	-	565	1	SCEL-AS1|	0	0.048
NM_1522	chr8	7487669	7489593	+	326	3	DEFB105A	0	0
NR_02705	chr15	21289604	21297829	-	8226	2	LOC64621	0	0
NR_13624	chr6	32255173	32350039	+	717.9	8	LOC10192	0	0
NM_0153	chr14	67746520	67816589	-	9631	1	ZFYVE26|	4.588	5.537
NM_0042	chr7	1.03E+08	1.03E+08	+	3904	1	PMPCB|B	12.679	13.525
NR_10504	chr5	1.26E+08	1.26E+08	+	562	1	LINC0203	0	0
NM_0060	chr7	30424527	30478777	-	4480	1	NOD1|CA	0.786	0.853
NM_0012	chr19	55785397	55832094	-	3180	1	NLRP11|C	0.032	0.508
NM_0011	chr11	64223799	64225869	-	842	1	TRPT1|-|-	40.64	27.166
NM_0010	chr21	31038159	31038476	-	315	1	KRTAP19-	0	0
NM_0011	chr1	2.28E+08	2.28E+08	+	893	1	GUK1|GM	142.666	123.074
NR_03037	chr19	19435063	19435158	+	95	1	MIR640|	0	0
NR_03071	chr15	70079372	70079468	-	96	1	MIR629|	0	0
NM_0011	chr11	28108251	28333507	+	4336	1	METTL15|	0.975	0.806
NM_0013	chrX	47445178	47483269	-	4660	1	ZNF41|M	2.233	2.672
NM_0228	chr18	2537525	2571503	-	3676	1	METTL4|H	1.841	1.833
NR_02428	chr9	35909483	35911620	+	1544	1	LINC00961	0.025	0.062
NR_03813	chr2	1.75E+08	1.75E+08	-	2036	1	CHN1|AR	37.756	29.432
NR_02453	chr6	1.05E+08	1.05E+08	-	1385	1	POPDC3|P	1.099	0.356
NR_03167	chr14	64095024	64095125	-	101	1	MIR548H1	0	0
NM_1390	chr12	1.21E+08	1.21E+08	-	4116	1	SPPL3|IM	24.794	32.109
NM_0018	chr14	1.04E+08	1.04E+08	-	1448	1	CKB|B-CK|	585.416	600.03
NM_0022	chr13	46125923	46182324	-	3772	1	LCP1|CP6	0.273	0.153
NM_0068	chr3	50205246	50220979	+	2453	1	SLC38A3|	2.665	4.121
NM_0010	chr1	9848276	9910258	-	2954	1	CTNNBIP1	40.173	36.969
NM_0160	chr2	24067584	24076444	-	765	1	SF3B6|CG	134.328	136.702
NR_00314	chr5	90558800	91164216	+	19160	1	ADGRV1|	2.956	8.592
NM_0043	chr15	78921750	78945098	-	1491	1	CTSH|ACC	2.689	3.877
NM_0239	chr11	78015715	78023274	-	1667	1	KCTD14|-|	0.495	1.504
NM_0012	chr1	1.15E+08	1.15E+08	-	3667	1	CSDE1|D1	148.393	147.007
NR_04759	chr13	24882274	24922889	-	5014	1	CENPJ|BM	2.139	2.579
NR_12578	chr2	15920399	15936018	+	1037	1	MYCNUT|	0	0.026
NR_12604	chr9	62897376	62899777	+	2257	1	LOC72867	0.171	0.231
NR_04660	chr3	1.26E+08	1.26E+08	+	433	1	ALDH1L1-	0	0
NR_12581	chr8	56445807	56552067	+	2639	1	LOC10192	0.366	0.114
NR_03022	chr19	53762344	53762430	+	86	1	MIR519A2	0	0
NM_1525	chr3	1.27E+08	1.27E+08	-	991	1	C3orf22|-|	0	0
NM_1531	chr6	1.6E+08	1.6E+08	+	1781	1	SLC22A1|	0.203	0.4
NR_02683	chr3	24099974	24103247	-	2239	1	LINC00691	0	0
NM_1536	chr3	97822040	97944986	+	10728	1	CRYBG3|D	3.225	0.776
NR_12586	chr6	1.51E+08	1.51E+08	-	882	1	LOC10272	0	0
NM_0012	chr20	33731760	33792269	+	3562	1	ZNF341|-|	1.929	2.646
NM_0190	chr2	1.9E+08	1.9E+08	+	2463	1	ASNSD1|N	13.255	15.465
NM_0029	chr2	20200797	20225166	-	3194	1	SDC1|CD1	9.297	12.299
NM_0183	chr12	28190427	28550166	+	2533	1	CCDC91|H	0.229	2.877

NM_1993:	chr17	7420360	7421632	+	977	1	SPEM1|C1	0.29	0.308
NR_02423	chr11	1.3E+08	1.3E+08	+	2862	1	LINC00167	0.14	0.172
NR_13506	chr11	36063284	36077901	-	4133	1	LOC10192:	0	0.003
NM_0201!	chr19	41386371	41397351	-	985	1	EXOSC5|R	9.268	8.343
NR_03157	chr1	2.24E+08	2.24E+08	-	137	1	MIR320B2	0	0
NM_0004	chr7	1.08E+08	1.08E+08	+	4905	1	SLC26A4|[	0.016	0.011
NR_02997	chrX	1.52E+08	1.52E+08	-	84	1	MIR452|N	0	0
NM_1458(	chr22	28687743	28741834	-	1755	1	CHEK2|CD	2.042	2.006
NM_0012{	chr4	73073966	73223114	-	9789	1	ANKRD17|	12.726	14.285
NM_0013:	chr3	38282294	38318575	+	2144	1	SLC22A14|	0.09	0.332
NR_10226	chrX	45731088	45731757	+	669	1	LOC39245:	0	0
NM_1749:	chr11	1.34E+08	1.34E+08	-	898	1	SPATA19|(	0	0
NM_0061!	chr15	55826919	55993746	-	5841	1	NEDD4|NE	0.318	0.387
NM_0166(	chr5	1.38E+08	1.38E+08	+	2175	1	REEP2|C5(	79.898	85.715
NR_11057	chr2	22536479	22538287	+	436	1	LINC01884	0	0
NR_03374	chr19	36421174	36422897	+	1723	1	LOC64418!	0.045	0.103
NM_0011(	chr19	49877425	49888750	+	2255	1	TBC1D17|	24.363	26.459
NR_14494	chr12	82358497	82479237	+	2009	1	METTL25|	0.642	0.477
NR_12204	chr1	916818	919692	-	1442	1	LOC10013(	2.217	4.274
NR_10676	chr7	1.35E+08	1.35E+08	-	83	1	MIR6509|I	0	0
NM_2035(	chr12	7729415	7749473	-	1213	1	CLEC4C|BI	0	0
NM_0053:	chr6	26017032	26017812	-	778	1	HIST1H1A|	0	0
NR_03784	chr20	14884253	14929518	-	692	1	MACROD2	0	0.059
NR_03973	chr14	1.06E+08	1.06E+08	-	51	2	MIR4507|	0	0
NR_02963	chrX	66018870	66018979	+	109	1	MIR223|N	0	0
NR_02942	chr1	1.11E+08	1.11E+08	-	603	1	PGCP1|-|-	0	0
NM_0013:	chr19	12730640	12734775	-	942	1	C19orf43|	239.816	234.623
NM_0013(	chr1	48727523	48776969	-	1841	1	BEND5|C1	24.743	21.881
NM_0053{	chr2	2.33E+08	2.33E+08	+	1140	1	NEU2|SIAI	0	0
NM_0324!	chr12	1.07E+08	1.07E+08	+	3360	1	RFX4|NYD	15.879	16.595
NM_0013:	chr10	43606407	43617904	+	1827	1	ZNF485|-|	0.868	0.915
NR_02746	chr16	22419546	22437715	-	2485	1	RRN3P3|-|	0.4	0.546
NR_02703	chr22	46039907	46044868	-	1606	1	LINC0089!	0.088	0.026
NM_1384(	chr9	1.38E+08	1.38E+08	-	1362	1	ZMYND19	30.445	34.625
NM_0010:	chr1	1.46E+08	1.46E+08	-	10828	1	NBPF10|A	0.019	0.154
NM_0123:	chr19	8520790	8577447	-	4143	1	MYO1F|-|	0.131	0.116
NM_0001(	chr2	38067603	38076181	-	5149	1	CYP1B1|CI	0.771	0.08
NR_00312	chr8	38600175	38601257	-	1082	1	RNF5P1|-|	12.024	7.697
NM_0011	chr19	14053367	14059159	-	2253	1	PALM3|-|	7.154	4.493
NR_07359	chr15	49858238	50119222	-	5502	1	ATP8B4|A	0.009	0.032
NM_0212:	chr19	2933218	2944971	-	2034	1	ZNF77|pT:	5.851	7.589
NM_0010{	chr2	2.14E+08	2.15E+08	+	4094	1	VWC2L|-|	0.472	0.304
NM_0529!	chr8	68330722	68819023	+	2669	1	C8orf34|V	0.077	0.426
NM_0163!	chr12	1.2E+08	1.2E+08	-	1178	1	TRIAP1|HS	16.834	18.72
NR_12065	chr10	25924378	25933710	-	547	1	LOC10192!	0	0
NM_1391!	chr2	2.02E+08	2.02E+08	+	3571	1	CDK15|AL:	0.137	0.031
NR_10506	chr5	1.64E+08	1.65E+08	+	578	1	LOC10254(	0.223	0.166
NM_0066!	chr20	35226736	35277001	+	4322	1	MMP24|N	23.937	38.22
NM_0019	chr5	1.4E+08	1.4E+08	-	2350	1	HBEGF|DT	5.744	10.41
NM_0011:	chr4	1.19E+08	1.19E+08	+	4850	1	SYNPO2|-|	0.128	0.22

NM_1989!	chr2	2.41E+08	2.41E+08	+	1078	1	AQP12A|A	0	0
NR_00231	chr2	1.75E+08	1.75E+08	+	1039.5	2	H3F3AP4||	31.195	31.927
NR_00300	chr2	2.33E+08	2.33E+08	+	277	1	SCARNA5|	0	0.396
NM_0012(	chr8	94371960	94436952	-	3082	1	RAD54B|R	1.753	2.382
NR_04711	chr5	1.47E+08	1.47E+08	-	443	1	PPP2R2B-I	0	0
NM_0011(	chr3	49940044	50077252	+	2207	1	RBM6|3G2	47.566	51.53
NM_0010	chrX	19533965	19799799	-	4435	1	SH3KBP1|(	8.158	18.533
NR_03609	chr4	1.52E+08	1.52E+08	-	89	1	MIR3140|ı	0	0
NR_03820	chr8	95268836	95810143	+	216	1	C8orf37-A!	0	0
NM_0036	chr22	29508167	29553747	-	2408	1	THOC5|C2	18.275	16.877
NM_0230	chrX	1.2E+08	1.2E+08	-	2353	1	UPF3B|HU	5.671	6.496
NM_0150	chr12	1.22E+08	1.22E+08	+	8176	1	SETD1B|KI	8.875	7.877
NM_0000(	chr1	2.01E+08	2.01E+08	-	6122	1	CACNA1S|	0.011	0.043
NM_1816!	chr17	67991099	67993649	-	1418	1	C17orf58|	9.52	9.321
NM_1334	chr19	42220271	42227053	+	2870	1	ZNF526|-|	4.861	4.582
NM_1825	chr14	67470266	67515304	-	4063	1	TMEM229	2.415	14.358
NR_10678	chr21	8205315	8205406	+	91	7	MIR6724-1	0	0
NM_0068!	chr12	75480733	75501936	+	3899	1	GLIPR1|CR	1.171	1.648
NR_03645	chr19	20398658	20424965	-	633	1	ZNF826P|Z	0.509	1.428
NM_0012	chr3	1.71E+08	1.71E+08	-	3302	1	SLC2A2|GI	0	0
NM_1813	chr22	44752558	44862784	+	1618	1	ARHGAP8|	1.944	6.307
NM_0012(	chr3	35641018	35794496	+	3146	1	ARPP21|A	2.611	0.483
NM_1526	chr16	74671855	74701148	-	2750	1	MLKL|hMI	0.038	0.03
NM_1525(	chr9	213108	215893	-	2783	1	C9orf66|-|	0	0
NM_1989	chr20	58981208	58995133	+	2288	1	NELFCD|H	52.126	51.316
NR_12203	chr9	1.26E+08	1.26E+08	-	696	1	LOC51145	0.185	0.02
NM_0027	chr3	1.7E+08	1.7E+08	+	4864	1	PRKCI|DXS	5.286	4.28
NM_0012	chr12	47079603	47236663	+	1858	1	PCED1B|F/	0.291	0.229
NR_03980	chr8	1.25E+08	1.25E+08	-	80	1	MIR4662B	0	0
NM_0012	chr22	37051736	37063390	+	1495	1	KCTD17|-|	30.962	24.252
NM_0246	chr11	77821114	77872352	+	546	1	AAMDC|C	11.618	13.672
NM_1523!	chr3	1.31E+08	1.31E+08	+	6112	1	NUDT16|-	4.263	6.593
NM_0010	chr3	1.84E+08	1.84E+08	-	1986	1	ABCC5|AB	19.347	17.532
NR_03173	chr16	85741621	85741700	-	79	1	MIR1910|	0	0
NM_0139	chr14	80197525	80211627	-	6137	1	DIO2|5DII	0.174	3.388
NM_0046	chr2	1.51E+08	1.51E+08	-	1469	1	NMI|-|-|2	0.035	0.028
NM_0010	chr8	7495846	7509311	+	385	3	DEFB107A	0	0
NM_0040!	chr1	23506428	23531220	-	5184	1	E2F2|E2F-	2.671	1.604
NM_0012!	chr2	2.38E+08	2.38E+08	+	2892	1	ERFE|C1Q	2.586	0.265
NR_03717	chr6	32844086	32846500	+	1270	8	PSMB8-AS	0	0
NM_0807	chr20	46541031	46550574	-	1953	1	OCSTAMP	0.026	0.014
NM_0051(	chr1	1.13E+08	1.13E+08	-	1702	1	PPM1J|PP	3.454	4.265
NM_0001	chr1	2.41E+08	2.42E+08	-	1855	1	FH|FMRD|	33.002	40.471
NR_04011	chr17_GL(	1576739	1743339	+	3925	3	NSF|SKD2	0	0
NR_13582	chr1_KI27(	4205	7201	+	1787	1	LOC10272	0	0.031
NM_0011	chr3	37243191	37366879	+	7843	1	GOLGA4|(	1.402	2.354
NM_0208	chr4	1.85E+08	1.85E+08	-	4875	1	CFAP97|KI	5.142	6.046
NM_0011	chr1	89821014	89936430	+	3782	1	LRRC8D|LI	10.998	13.085
NM_0010(	chr1	2.48E+08	2.48E+08	-	922	1	OR13G1|C	0	0
NM_0011	chr4	1.06E+08	1.06E+08	-	1581	1	INTS12|IN	20.796	24.734

NM_00108	chr9	1.21E+08	1.21E+08	-	6202	1	MEGF9|EG	13.097	17.264
NM_00222	chr3	52794768	52809009	+	3013	1	ITIH3|H3P	0.171	0.114
NM_0011(	chr5	16067365	16179788	-	1522	1	MARCH11	0.339	0.027
NM_1749(	chr19	38403135	38409088	+	1265	1	FAM98C|-	12.445	10.557
NM_17742	chr1	27773183	27824452	+	3068	1	STX12|STX	38.71	43.381
NM_18275	chr1	3624992	3630130	+	2420	1	TPRG1L|FA	24.764	20.325
NM_00128	chr5	1.73E+08	1.73E+08	+	761	1	LOC10192	0.186	0.504
NM_00127	chr1	2.01E+08	2.01E+08	-	1045	1	TNNT2|CN	0	0
NM_00103	chr15	89776358	89778751	+	1607	1	MESP2|SC	0.072	0.034
NM_00103	chr6	13786549	13814560	-	5460	1	MCUR1|C6	4.565	6.663
NR_02731	chr19	28965131	28969148	+	3442	1	LINC00906	0	0
NM_00034	chr2	31524586	31580971	-	2439	1	SRD5A2|-|	0	0.051
NR_03815	chr14	93207056	93229215	+	3550	2	UBR7|C14	0	0
NM_00127	chr1	1.5E+08	1.5E+08	+	2051	1	VPS45|H1	32.086	34.673
NM_00128	chr16	4476340	4510347	+	1597	2	HMOX2|H	0.016	0.017
NM_00131	chr20	63554143	63556707	+	1114	1	FNDC11|C	2.975	2.693
NR_03021	chr19	53734877	53734963	+	86	1	MIR518D|	0	0
NM_0013(	chr12	51888009	51891721	+	1646	1	ANKRD33|	0.055	0.033
NM_01224	chr16	30443625	30445975	-	2348	1	SEPHS2|SP	32.393	49.719
NM_00112	chr11	2883218	2885765	-	1752	2	CDKN1C|B	0.029	0
NR_11073	chr19	51888025	51900464	+	577	1	ZNF649-AS	4.223	6.291
NM_0011(	chr3	58008400	58172255	+	9348	1	FLNB|ABP-	10.769	17.612
NM_00719	chr8	37762583	37779768	+	2559	1	PROSC|-|-	14.012	15.019
NM_0309(	chr1	2.48E+08	2.48E+08	+	1108	3	OR2T1|OR	0	0
NM_00112	chr4	1166933	1173211	-	1797	1	SPON2|DII	7.627	3.041
NR_10417	chr19	35788876	35797909	-	2183	1	LINC01529	0.006	0.025
NM_0064(	chr1	1.85E+08	1.85E+08	-	4182	1	IVNS1ABP	33.791	61.637
NR_11039	chrX	4627200	4633572	-	1181	1	LOC10192	0	0.012
NR_03997	chr13	41961169	41981565	+	2075	1	VWA8-AS1	0.286	0.396
NR_02682	chr9	34394	35864	-	1127	3	FAM138C|	0	0
NM_01594	chr5	52787940	52802617	+	2915	1	PELO|CGI-	6.878	8.068
NR_10981	chr9	1.36E+08	1.36E+08	+	2435	1	LINC01502	0	0
NM_0312(	chr16	1827224	1828907	+	1681	1	FAHD1|C1	11.129	8.173
NM_19932	chr17	39868202	39877896	+	2247	1	ZPBP2|ZPE	0	0
NR_03758	chr17	33529787	33533760	-	629	1	AA06|AA0	0	0
NM_00369	chr8	1.43E+08	1.43E+08	-	797	1	LY6D|E48|	0	0
NM_01461	chr3	1.83E+08	1.83E+08	+	7288	1	ATP11B|AT	1.426	2.203
NM_00108	chr8	25419258	25425040	-	1275	1	GNRH1|GN	0.405	0.451
NR_02654	chr21	39605606	39612822	-	1348	1	B3GALT5-A	0.23	1.047
NM_0013(	chr18	35338883	35344467	-	934	1	ZNF24|KO	16.027	14.225
NM_03272	chr2	2.19E+08	2.19E+08	+	3219	1	PLCD4|-|-	3.937	3.949
NM_08038	chr8	7470308	7475082	-	277	3	DEFB104A	0	0
NM_00132	chr2	74421723	74425755	+	1120	1	WDR54|-|	63.12	31.674
NM_19854	chr1	11691724	11720279	+	1764	1	DRAXIN|A	60.608	53.499
NM_0011(	chr2	2.01E+08	2.02E+08	-	1756	1	ALS2CR11	0	0
NM_01911	chr6_KI27(	9687	14891	-	1275.5	8	HLA-DRA|I	0	0
NM_00103	chr2	2.41E+08	2.41E+08	+	1550	1	DUSP28|D	3.51	3.093
NM_17882	chr9	87882857	87888899	+	4441	1	SPATA31E1	0.006	0.019
NM_00707	chr12	1.09E+08	1.09E+08	+	1646	1	FICD|HIP1	1.238	1.606
NM_0013(	chr1	2.03E+08	2.03E+08	+	6420	1	PPFIA4|-|-	5.82	3.685

NM_0022!	chr3	39406689	39412541	+	1161	1	RPSA|37LF	65.067	60.793
NM_0011!	chr14	1.05E+08	1.05E+08	+	2565	1	TEX22|-|-|	0.357	0.272
NM_0011!	chrX	55181391	55182511	-	1118	1	MTRNR2L1	0.035	0.11
NM_0209!	chr5	1.41E+08	1.41E+08	+	4529	1	PCDHB16|	2.084	2.108
NM_1738:	chr6	1.09E+08	1.09E+08	+	2504	1	CEP57L1|C	1.189	1.428
NM_0013(	chr14	35085468	35122543	-	1805	1	PPP2R3C|(	11.301	10.631
NM_0004:	chr6_GL0C	3336470	3340350	-	2063	8	NEU1|NAN	0	0
NR_13564	chr2	2.18E+08	2.18E+08	-	2118	1	TMBIM1|L	0.359	0.944
NM_0124	chr2	70900590	70933445	+	1141	1	VAX2|DRE	2.882	4.574
NR_03161	chr22	20249134	20249211	-	77	1	MIR1286|	0	0
NM_0055!	chr12	52487174	52493397	-	2439	1	KRT6A|CK-	0	0.034
NM_0012	chr10	49366116	49396016	-	946	1	DRGX|DRC	0.273	0.159
NM_0163!	chr15	65530489	65578355	+	3195	1	HACD3|B-	89.076	75.079
NM_0002(	chr17	51153559	51162089	+	804	1	NME1|AW	187.501	162.695
NM_0011	chr7	44104361	44114565	+	4063	1	AEBP1|AC	11.218	7.474
NM_0012{	chr15	51938025	51971801	-	1996	1	LEO1|RDL	9.773	8.31
NM_1525{	chr11	1.08E+08	1.08E+08	-	1022	1	C11orf65|	0.164	0.067
NM_0013(	chr18	27950963	28036585	-	3838	1	CDH2|CD3	101.703	91.978
NM_0220!	chr2	47520776	47570331	-	1421	1	KCNK12|K	3.384	1.995
NM_0030(	chr17	82321024	82334045	-	2275	1	SECTM1|K	0.13	0.181
NM_0036!	chr2	1.78E+08	1.78E+08	-	1816	1	PRKRA|DY	7.966	8.123
NM_0124(	chr20	63981078	64033100	+	3078	1	PRPF6|AN	83.292	79.953
NM_0012!	chr1	2.01E+08	2.01E+08	-	9249	1	KIF21B|-|-	125.751	122.195
NM_0010:	chr6_GL0C	304250	313530	-	2320	7	ZNF311|zf	0	0.006
NM_0248	chr19	40222208	40226690	-	1810	1	CNTD2|CC	0.185	0.31
NM_0176:	chr9	1.36E+08	1.37E+08	-	9271	1	NOTCH1|A	19.477	18.936
NM_1825!	chr6	1.69E+08	1.7E+08	-	3564	1	WDR27|-|	4.254	4.081
NR_11016	chr7	1.13E+08	1.13E+08	-	701	1	HRAT17|-|	0	0
NM_0011!	chr3	9866710	9880254	-	1189	1	CIDEC|CID	0.022	0
NR_02690	chr17_KI2	302243	304003	-	1756.7	3	MAPK8IP1	0	0
NM_0058	chr1	1.15E+08	1.15E+08	-	1292	1	BCAS2|DA	21.775	16.581
NM_0011!	chr17	8729928	8744836	-	1150	1	CCDC42|C	0.022	0.012
NR_12598	chr1	46133371	46139080	+	355	1	LOC10192!	0.327	1.119
NR_10677	chr19	39829716	39829802	-	86	1	MIR6719|	0.45	0
NM_0073	chr20	62804800	62814000	+	2436	1	OGFR|-|-|	27.92	26.438
NM_0011:	chr1	1.55E+08	1.55E+08	-	3467	1	SHC1|SHC	20.997	11.374
NM_2073(	chr2	1.33E+08	1.34E+08	-	7572	1	NCKAP5|E	4.73	6.293
NM_0247:	chr12	1.17E+08	1.17E+08	-	5271	1	C12orf49|	31.67	33.306
NM_0060	chr13	44432143	44436924	-	3209	1	TSC22D1|	79.422	95.444
NM_0012!	chr4	9215405	9216997	+	1591	3	USP17L20	0	0
NR_12036	chr15	85754941	85758725	-	987	1	LOC10192!	0.536	0.416
NM_0132	chrX	48831092	48835638	-	1052	1	PCSK1N|B	172.113	161.76
NM_0223!	chr20	44745847	44752313	+	2528	1	KCNK15|K	5.753	7.52
NM_1450	chr5	1.59E+08	1.59E+08	+	2393	1	UBLCP1|C	3.96	5.712
NM_0012{	chr20	63272785	63289790	+	3163	1	ARFGAP1|	29.272	31.062
NM_0020:	chr14	45115599	45134529	-	1338	1	FKBP3|FKE	84.492	73.665
NM_0010(	chr4	69995931	70002456	+	563	1	STATH|STF	0	0
NR_03965	chr4	52712682	52712747	+	65	1	MIR4449|-	0	0
NM_0011	chr12	1.33E+08	1.33E+08	+	2841	1	PGAM5|B)	16.651	18.301
NM_0011(	chr3	33798571	33869707	+	5942	1	PDCD6IP|/	7.122	7.549

NM_0048	chr19	54906150	54913071	+	982.8	16	NCR1|CD3	0	0
NM_0247	chr11	1.23E+08	1.23E+08	-	2946	1	CLMP|ACA	1.663	1.111
NM_1768	chr12_KI2	229392	230412	-	1018	3	TAS2R31|	0.165	0.04
NR_02712	chr5	1.36E+08	1.36E+08	-	2039	1	LINC0195	0	0
NM_0012	chr14	69050920	69152599	-	5727	1	DCAF5|BC	25.449	31.436
NM_0010	chr9	38406528	38424447	-	3552	1	IGFBPL1|I	1027.191	303.941
NR_00213	chr6_GL0C	1053607	1055649	-	2033.4	8	HCG4|HC	0	0
NM_0012	chr22	40824535	40857008	-	3537	1	ST13|AAG	16.659	13.747
NM_0183	chr20	13008979	13166764	+	3831	1	SPTLC3|C	0.101	0.029
NM_0223	chr1	1.79E+08	1.79E+08	+	2114	1	TOR3A|AD	10.088	10.483
NM_0529	chr1	15247272	15398126	+	5057	1	FHAD1|-|-	0.173	0.092
NM_0035	chr6	27815044	27815489	+	443	1	HIST1H2BI	0	0.031
NM_0528	chr22	50170731	50180295	+	3047	1	PANX2|PX	3.229	4.185
NM_0036	chr10	13586939	13630868	+	1693	1	PRPF18|P	10.304	9.288
NR_13265	chr3	1.52E+08	1.52E+08	-	1292	1	TMEM14E	0	0
NM_0067	chr1	30732468	30757836	-	2230	1	LAPTM5|C	0.237	0.074
NM_0064	chr1	1.46E+08	1.46E+08	+	3890	1	POLR3C|R	9.993	10.31
NR_02420	chr2	87455455	87521511	+	494	1	CYTOR|C2	9.318	6.239
NR_10242	chr7	90396292	90398236	+	1944	1	LOC10140	0.027	0.014
NM_0003	chr12	47841537	47905031	-	4641	1	VDR|NR1I	0.047	0.035
NR_04982	chr2	62205826	62205917	+	91	1	MIR5192|	0	0.151
NM_0011	chrX	38149335	38220924	-	1832	1	SRPX|DRS	2.083	1.832
NM_0011	chr11	72216775	72221950	+	1106	1	FOLR2|BE	0	0.012
NR_03173	chr6	1.67E+08	1.67E+08	-	79	1	MIR1913|	0	0
NM_0176	chr5	1.77E+08	1.77E+08	+	4083	1	CDHR2|PC	0.663	0.564
NM_0178	chr19	57571566	57578875	-	2503	1	ZNF416|-|	7.433	10.332
NM_0065	chr13	20566816	20691437	+	3156	1	IFT88|D13	1.977	2.192
NR_12045	chr12	68431842	68451484	-	570	1	LOC10050	0.023	0.096
NR_04620	chr9	30388935	30408454	-	1252	1	LINC01242	0	0
NM_0143	chr2	1.9E+08	1.9E+08	-	1923	1	HIBCH|HIE	4.754	5.42
NM_0011	chr13	37562582	37598844	-	3186	1	POSTN|OS	0.405	0.099
NR_13656	chr22	18361830	18386526	-	1850	1	LINC0166C	0	0
NM_0012	chr2	3544892	3558371	-	1855	1	RNASEH1|	8.932	10.714
NM_0013	chr3	1.31E+08	1.31E+08	+	2549	1	NEK11|-|-	0.486	0.704
NM_0808	chr14	55027126	55049488	+	6879	1	SOCS4|SO	2.319	3.562
NM_1532	chrX	80669488	80809734	-	12750	1	BRWD3|BI	2.079	1.886
NM_0011	chr8	58583505	58659407	-	3476	1	NSMAF|FA	4.136	5.926
NR_13430	chr8	1.44E+08	1.44E+08	+	1282	2	LOC10192	0	0
NM_0010	chr2	24790267	24793382	-	568	1	PTRHD1|C	28.375	25.296
NM_0070	chr8	1.41E+08	1.41E+08	+	2704	2	PTP4A3|P	0	0
NM_0012	chrX	54933134	54998534	-	1576	1	PFKFB1|F6	0.712	0.947
NM_0012	chrX	37042882	37043794	-	910	1	FTH1P18|	0	0
NM_0011	chr5	1.51E+08	1.51E+08	-	3219	1	ZNF300|-|	9.837	12.638
NM_0144	chr8	42374068	42377156	-	812	1	DKK4|DKK	0.143	0.067
NR_02983	chr9	1.28E+08	1.28E+08	-	96	1	MIR219A2	1.88	0.571
NM_0013	chr11	1.25E+08	1.25E+08	+	3417	1	LOC40331	0.294	0.232
NM_0013	chr12	38316755	38329726	+	6317	1	ALG10B|A	0.604	0.278
NM_0010	chr21	42403862	42447680	+	2426	1	UBASH3A|	0.021	0
NM_0011	chr17	80415167	80430371	+	1261	1	ENDOV|-|	8.558	9.081
NM_1735	chr6	1.54E+08	1.55E+08	-	3213	1	CNKSR3|M	0.353	1.339

NM_0011	chr7	1.57E+08	1.57E+08	-	1615	1	MNX1|HB	0.136	0.348
NR_13510	chr11	12030875	12061785	+	3306	1	LOC10537	0.012	0
NR_10344	chr18	61748176	61754695	+	3213	1	LINC01544	0	0
NM_0133	chr21	32741339	32771858	-	2790	1	PAXBP1|B	4.778	7.747
NM_0011	chr22	45671798	45845307	+	3125	1	ATXN10|E	81.685	101.709
NM_0012	chr4	56338285	56387508	-	3170	1	AASDH|AC	3.266	4.705
NM_0060	chr2	2.39E+08	2.39E+08	-	8947	1	HDAC4|AH	4.162	5.158
NM_0053	chr16	31393312	31426505	+	3880	1	ITGAD|AD	0	0
NM_1735	chr11	6497296	6520867	+	3207	1	DNHD1|C1	1.6	1.683
NM_0010	chr1	1.52E+08	1.52E+08	-	9117	1	FLG2|IFPS	0	0
NM_0328	chr15	63121800	63142065	+	1968	1	LACTB|G2	4.01	2.854
NM_0010	chr12	11133285	11134244	-	958	3	TAS2R30|	0	0
NM_0051	chr5	1.8E+08	1.8E+08	-	3072	1	GFPT2|GF	8.726	9.894
NM_0013	chr4	1.85E+08	1.85E+08	+	3302	1	SNX25|MS	3.452	3.024
NM_0010	chr5	1.15E+08	1.15E+08	-	2484	1	CCDC112|	5.845	8.133
NM_0010	chr2	64524305	64593004	+	4025	1	AFTPH|Nb	8.028	10.461
NM_0142	chr5	1.5E+08	1.5E+08	+	3819	1	SLC6A7|PI	0.115	0.251
NR_03610	chr8	17681578	17681657	-	79	1	MIR548V|	0	0
NM_0011	chr17	59565525	59608352	+	3417	1	DHX40|AR	17.157	15.192
NM_0015	chr1	88852638	88891618	-	1636	1	GTF2B|TF	27.797	30.375
NM_0048	chr17	7591230	7614897	-	2968	1	FXR2|FMR	63.386	75.698
NM_0032	chr17	78852977	78925390	-	3648	1	TIMP2|CS	66.733	132.521
NM_1738	chr16	21983865	22001110	-	1075	1	PDZD9|C1	0.48	0.204
NM_0044	chr1	1.51E+08	1.51E+08	+	2132	1	ECM1|URI	1.457	2.056
NM_0013	chr1	1.11E+08	1.11E+08	+	928	1	C1orf162|	1.639	1.771
NM_0014	chr5	1.6E+08	1.6E+08	+	572	1	FABP6|I-1	0.586	1.173
NM_0011	chr6	1.16E+08	1.17E+08	-	1101	1	TRAPPC3L	0.141	0
NM_0012	chr14	24136158	24138967	+	954	1	PSME1|HE	52.506	58.808
NR_03017	chr12	94834398	94834513	+	115	1	MIR492|M	0	0
NM_0175	chr19	14826327	14828464	-	2135	1	OR7A5|HT	0	0
NM_0240	chr22	41938721	41947164	-	958	1	CENPM|C	4.966	3.546
NM_0039	chr4	76306026	76311130	+	2947	1	STBD1|GE	1.623	0.734
NM_0032	chr13	1.03E+08	1.03E+08	+	5464	1	TPP2|TPP-	4.347	5.502
NR_00394	chr1	1.74E+08	1.74E+08	-	59	1	SNORD75|	0	0
NM_0012	chr11	1.19E+08	1.19E+08	+	3365	1	C2CD2L|D	12.277	17.487
NM_1447	chr3	42708382	42773253	-	2812	1	CCDC13|-|	1.1	0.833
NR_02750	chr1	16645574	16650420	+	2622	1	MST1P2|M	0.231	0.34
NR_00308	chr14	1.01E+08	1.01E+08	+	76	1	SNORD112	0	0
NM_0010	chr3	72888234	72975371	+	1535	1	GXYLT2|G	0.21	1.829
NR_00442	chr1	1.46E+08	1.46E+08	-	163	9	RNU1-4|H	0	0
NM_0052	chr1	1.48E+08	1.48E+08	+	1362	1	GJA8|CAE	0	0
NM_1735	chr12	460377	561892	+	3416	1	B4GALNT3	1.212	1.395
NR_03612	chr11	96341438	96341526	+	88	1	MIR1260B	2.344	0.778
NM_0245	chr19	35937120	35945195	+	2510	1	LRFN3|FIG	18.662	17.457
NR_04002	chr16	2658389	2673439	-	8333	1	ERVK13-1|	2.824	2.306
NM_0540	chr8	11330986	11332186	+	1198	1	SLC35G5|	0.161	0.183
NM_0010	chrX	1.07E+08	1.07E+08	-	3777	1	MORC4|Z	2.233	1.287
NM_0012	chr1	1.11E+08	1.11E+08	+	1197	1	CHIA|AMC	0	0
NR_04500	chr11	59748812	59749581	+	769	1	OR10V2P|	0.017	0
NM_1537	chrY	18546671	18548591	+	1439	2	HSFY2|HSI	0	0

NM_00128	chr20	46349528	46364458	-	2226	1	SLC35C2|E	27.571	24.705
NM_03078	chr8	1.03E+08	1.03E+08	-	3110	1	SLC25A32|	4.357	3.4
NM_01415	chr16	58249936	58283830	+	5255	1	CCDC113|	3.437	3.328
NR_04639	chr17	6755837	6775645	+	3366	1	XAF1|BIRC	0	0
NM_17349	chrX	23334868	23396801	+	5286	1	PTCHD1|A	0.227	0.386
NR_10706	chr16	14930820	14930879	+	59	5	MIR6770-3	0	0
NM_00477	chr17	39404285	39451274	-	8129	1	MED1|CRS	13.756	14.388
NM_00108	chr3	14489111	14540343	-	7695	1	GRIP2|-|-|	1.24	1.522
NM_21255	chr9	32783499	32789201	+	5379	1	TMEM215	0	0.003
NR_02705	chr17	77088643	77094986	+	2166	1	SNHG20|C	4.101	5.217
NR_03966	chr4	1.85E+08	1.85E+08	-	57	1	MIR4455|-	0	0
NM_00127	chr17	7217125	7225267	+	2205	1	ACADVL|A	41.914	39.029
NR_13511	chr1	1.57E+08	1.57E+08	-	383	1	LOC10192	0.135	0.215
NM_17754	chr19	45687454	45691992	-	669	1	SNRPD2|S	268.547	205.496
NR_03846	chr22	20198730	20204918	-	2223	1	LOC28486	0.035	0.037
NM_01829	chr6	1.07E+08	1.07E+08	+	4105	1	QRSL1|Ga	4.168	5.229
NM_00128	chr15	74318541	74336141	+	2010	1	CCDC33|C	0.038	0.034
NR_12119	chr6	1.12E+08	1.12E+08	+	533	1	LOC10192	0	0.051
NR_10681	chr12	57512688	57512750	+	62	1	MIR6758|I	51.158	58.544
NM_00245	chr5	7869104	7901122	+	3281	1	MTRR|MS	3.977	4.646
NR_00298	chr11	2963771	2963893	-	122	2	SNORA54|	0	0
NM_17822	chr1	1.57E+08	1.57E+08	-	6012	1	IQGAP3|-|	1.654	1.219
NM_00289	chr11	66638617	66646473	+	1692	1	RBM4|LAF	105.906	108.273
NM_00106	chr11	59852808	59866568	-	1556	1	TCN1|HC|	0.025	0.009
NR_02799	chr6	1.37E+08	1.37E+08	-	1357	1	NHEG1|-|-	0	0
NR_10677	chr14	21023314	21023386	-	72	1	MIR6717|I	6.268	8.561
NR_03825	chr15	35546195	35859001	+	1474	1	DPH6-AS1	0.14	0.046
NR_00244	chr9	1.33E+08	1.33E+08	+	71	1	SNORD36A	35.048	31.445
NM_05283	chr6	1.33E+08	1.33E+08	-	2409	1	SLC18B1|C	8.296	15.027
NM_13842	chr14	1.05E+08	1.05E+08	-	18308	1	AHNAK2|C	2.869	3.272
NR_10461	chr5	3496372	3504004	-	622	1	LINC01017	0	0
NM_00120	chr12	70638073	70788930	-	2828	1	PTPRR|EC-	1.19	1.346
NM_00133	chr2	88067780	88113383	+	4379	1	SMYD1|BC	0.024	0.034
NM_00087	chr1	19665287	19680966	+	3383	1	HTR6|5-HT	0.011	0.093
NM_18249	chr19	43607224	43619862	-	1390	1	ZNF428|C1	116.153	136.832
NM_00102	chrX	49074433	49079905	-	1612	1	WDR45|JN	14.501	14.088
NM_00091	chr3	8750409	8769614	-	4354	1	OXTR|OT-I	0.139	0.573
NR_03392	chrX	47707191	47736628	+	1893	1	CXXC1P1|(	0	0
NM_00070	chr19	35550193	35563658	-	3535	1	ATP4A|AT	0.069	0.062
NM_02097	chr16	77788564	77980107	+	3780	1	VAT1L|-|-	93.406	62.64
NM_01780	chr14	20447048	20455108	-	1612	1	OSGEP|GC	19.148	15.175
NM_15250	chr20	5750387	5864407	+	1654	1	C20orf196	5.574	6.675
NR_03034	chr10	1.04E+08	1.04E+08	-	94	1	MIR609|M	0	0
NM_00127	chr12	27022522	27030673	+	2697	1	MED21|SF	5.684	6.099
NM_13915	chr19	4675231	4723843	-	4246	1	DPP9|DP9	8.187	7.658
NM_02083	chr1	20664014	20718017	-	3954	1	KIF17|KIF1	5.217	2.013
NR_03883	chr15	20282744	20291558	+	858	1	CHEK2P2|-	0	0
NR_03898	chr10	85577731	85607215	+	2099	1	GRID1-AS1	0.068	0.026
NR_04596	chr12	52897187	52949866	-	2141	1	KRT8|CAR	0.44	0.205
NM_02409	chr8	1.2E+08	1.2E+08	-	2258	1	DSCC1|DC	0.759	1.535

NM_0122:	chr16	83899125	83916182	+	2188	1	MLYCD|M	6.405	5.44
NM_0012:	chr11	64226258	64230016	+	890	1	NUDT22|-	19.731	12.681
NR_03607	chr2	2.07E+08	2.07E+08	+	74	1	MIR3130-2	0	0
NR_03621	chr1	3127975	3128035	+	60	1	MIR4251|-	0	0
NR_12588	chr4	1.44E+08	1.44E+08	+	1294	1	LOC10192	0	0.011
NM_0023	chr2	1.6E+08	1.6E+08	-	6894	1	LY75|CD2(	0	0.006
NM_0181:	chr3	1.36E+08	1.36E+08	-	4692	1	MSL2|MSL	6.128	8.416
NM_0152:	chr1	92168858	92184725	+	4330	1	KIAA1107|	1.471	1.673
NR_03163	chr12	1.13E+08	1.13E+08	-	142	1	MIR1302-1	0	0
NR_02446	chr17	42753926	42761257	-	1678	1	RAMP2-AS	1.583	2.898
NM_0018(	chr19	36150922	36152869	-	756	1	COX7A1|C	0.051	0
NR_04010	chr15	72782835	72798199	+	2727	1	ADPGK-AS	0.671	0.804
NM_0012	chrX	77910656	78050395	+	8271	1	ATP7A|DS	1.478	1.328
NR_00329	chr12	1.32E+08	1.32E+08	+	1529	1	EP400NL|-	1.172	1.747
NM_0046:	chr3	1.29E+08	1.29E+08	+	2224	1	RAB7A|PR	165.453	187.17
NM_0064	chr22	19875518	19941992	-	2099	1	TXNRD2|S	3.428	1.194
NR_02669	chr17	42579508	42609427	-	3770	1	FAM134C|	25.517	24.793
NR_13527	chr21	45234340	45258730	+	539	1	LINC00334	0.742	0.584
NM_0010(	chr19	9565728	9584533	-	1611	1	ZNF121|D	2.393	3.732
NM_0010	chr16	2530035	2531408	-	1371	1	CEMP1|CF	5.266	5.135
NR_04024	chr2	1.45E+08	1.45E+08	+	425	1	ZEB2-AS1|	3.762	16.823
NR_13530	chrX	1.51E+08	1.51E+08	-	1611	1	GPR50-AS1	0.024	0.221
NM_0011	chr3	46574555	46582462	+	1765	1	TDGF1|CR	0	0
NM_0314!	chr1	28259452	28282491	+	3516	1	SESN2|HIS	16.755	23.95
NR_03890	chr11	83072066	83073712	+	670	1	RAB30-AS1	8.41	5.479
NM_0012!	chr7	1E+08	1E+08	-	2237	1	ZCWPW1|	3.061	2.535
NM_0186(	chr8	7881204	7882658	+	508	3	DEFB103B	0	0
NM_0011	chr1	2.47E+08	2.47E+08	-	1716	1	ZNF669|-|	8.288	11.191
NM_0244	chr1	1.87E+08	1.87E+08	+	2908	1	PLA2G4A|	0.133	0.127
NM_0061	chr12	1.13E+08	1.13E+08	+	6620	1	OAS3|p10	0	0.031
NM_0142	chr10	17589959	17617374	-	1298	1	HACD1|CA	2.344	1.868
NM_1526(	chr1	54806063	54842264	+	1776	1	LEXM|C1o	0.044	0.046
NM_0011!	chr6	1.06E+08	1.06E+08	+	5156	1	PRDM1|BI	0.01	0.003
NM_0010	chr9	41126251	41128975	-	2722	1	FOXD4L6|-	0	0.01
NM_0054	chr5	42799880	42811922	-	2079	1	SELENOP|S	4.527	7.287
NR_02436	chr9	1.13E+08	1.13E+08	+	5799	1	FAM225A|	0	0
NM_0033!	chr12	48965340	48971858	-	2362	1	WNT10B|S	3.728	3.259
NR_13120	chrX	1.14E+08	1.14E+08	-	494	1	XACT|-|-|X	0	0
NM_0190(	chr3	1.22E+08	1.22E+08	-	4180	1	WDR5B|-|	2.927	2.641
NM_0071!	chr12	66189198	66254614	+	8322	1	IRAK3|ASF	0.079	0.051
NR_02847	chr5	65944805	65945573	-	768	1	LOC10030	0	0
NM_0012	chr14	94462721	94476333	-	1644	1	SERPINA9|	0	0
NR_03326	chr17	82389210	82418637	-	4495	1	OGFOD3|(	4.486	4.79
NM_0011(	chrX	1.2E+08	1.2E+08	+	1587	1	SOWAHD|	0.447	0.242
NM_0168	chr3	9749944	9766669	+	1944	1	OGG1|HM	18.524	10.576
NM_0162	chr5	1.51E+08	1.51E+08	-	3872	1	DCTN4|DY	33.246	34.593
NM_0011	chr2	38563186	38603036	-	2763	1	HNRNPLL|	17.644	18.986
NR_00277	chr1	63548980	63550636	+	1656	1	DLEU2L|B(	0.327	0.289
NM_1809!	chr5	1.02E+08	1.02E+08	-	5319	1	SLCO4C1|(	0.002	0.021
NM_0013	chr2	1.72E+08	1.72E+08	+	4377	1	DYNC1I2|I	26.258	24.293

NM_0011	chr5	1.81E+08	1.81E+08	-	2643	1	MGAT1|G	23.528	21.927
NR_04981	chr19	49682117	49682195	+	78	1	MIR5088|	51.078	42.32
NM_1392	chrY	3579085	3580041	+	857	1	TGIF2LY|T	0	0
NM_1826	chr7	1.05E+08	1.05E+08	-	3670	1	SRPK2|SFF	22.737	32.242
NM_0008	chr1	1.1E+08	1.1E+08	-	4116	1	GSTM3|GS	12.095	15.947
NR_13518	chr16	86722091	86737826	+	539	1	LOC10272	0	0
NR_02425	chr3	1.3E+08	1.3E+08	-	2111	1	FAM86HP	1.356	1.11
NR_00308	chr16	32877476	32885142	-	3830	1	SLC6A10P	0.003	0.007
NM_0134	chr7	1E+08	1E+08	+	1307	1	PILRA|FDF	1.608	1.834
NR_03985	chr14	22956950	22957029	-	79	1	MIR4707|	6.365	1.734
NR_03837	chr21	9781849	9821061	-	1200	1	LINC01667	0	0
NR_10977	chr9	81689713	81776899	+	1444	1	LOC10192	0.027	0.085
NM_0012	chr19	50873433	50880567	+	2669	1	KLK2|KLK2	0	0.01
NM_0040	chr12	56271699	56300391	-	2963	1	CS|-|-|12c	72.779	73.279
NR_00217	chrY	23936727	23941622	-	341	2	TTTY3B|LN	0	0
NM_0010	chr19	4472196	4502220	+	2363	1	HDGFRP2|	71.823	63.489
NM_0059	chr1	1.53E+08	1.53E+08	-	673	1	SPRR2A|-|	0	0
NM_0011	chr3	47850690	48089279	-	6016	1	MAP4|-|-|	56.471	51.403
NM_0184	chr3	10115649	10127190	+	1171	1	BRK1|C3o	149.316	134.327
NM_0011	chr8	19313976	19396218	+	3237	1	SH2D4A|P	0.012	0.021
NM_0011	chrX	1.27E+08	1.27E+08	+	1223	1	PRR32|CX	0	0.011
NM_0177	chr3	31660825	31981850	-	4199	1	OSBPL10|	0.491	0.6
NM_0012	chr2	20248692	20327386	-	5856	1	PUM2|PU	17.44	22.468
NM_0010	chr22	32475237	32498831	+	1747	1	FBXO7|FB	55.028	58.418
NM_0010	chr3	1.41E+08	1.41E+08	+	3273	1	PXYLP1|A	30.522	37.94
NM_0183	chr4	26583924	26756213	+	3094	1	TBC1D19|	4.405	4.139
NM_0019	chr19	852209	856246	+	999	2	ELANE|EL	0	0
NM_0010	chr15	22838641	22868384	+	2986	1	NIPA2|-|-|	15.385	14.995
NM_0012	chr7	65873099	65959563	+	5953	1	VKORC1L1	9.586	9.76
NR_10678	chr9	1.37E+08	1.37E+08	-	77	1	MIR6722|	0	0
NM_0013	chr11	1.17E+08	1.17E+08	-	926	1	APOA1|ap	0.181	0.059
NM_0012	chr7	37921319	37951940	+	2078	1	EPDR1|EP	8.631	6.15
NR_04007	chr1	1.99E+08	1.99E+08	-	601	1	MIR181A1	0.322	0.479
NM_0040	chrX	1595455	1643081	+	1322	2	ASMT|ASN	0	0
NM_0011	chr18	12407896	12432237	+	1711	1	PRELID3A|	1.861	3.634
NR_10679	chr1	1.56E+08	1.56E+08	-	63	1	MIR6738|	11.052	5.218
NM_0007	chr7	99705037	99735200	-	2067	1	CYP3A7|C	0.112	0.139
NR_03839	chr11	1.03E+08	1.03E+08	+	2634	1	WTAPP1|-	0.049	0
NM_0149	chr13	72755402	72782128	-	7543	1	DIS3|2810	4.28	5.462
NM_0013	chr17	54900691	54961967	+	2161	1	TOM1L1|S	4.415	3.835
NM_0010	chr16	23182719	23216879	+	3484	1	SCNN1G|E	0.007	0.071
NM_0180	chr6	1.07E+08	1.08E+08	+	6223	1	SOBP|JXC	14.124	13.351
NR_11080	chr17	28926275	28944748	+	680	1	LOC10192	0.739	0.342
NR_03015	chr14	1.01E+08	1.01E+08	+	79	1	MIR410|M	0	0
NM_0058	chr13	95019829	95301446	-	5823	1	ABCC4|M	0.635	1.098
NM_0010	chr11	56700388	56701305	+	916	2	OR9G1|OF	0	0
NR_04608	chr11	1.11E+08	1.11E+08	-	2583	1	LOC10013	0	0
NR_00322	chr14	1.01E+08	1.01E+08	+	69	1	SNORD113	0	0
NM_1817	chr2	23940506	24000823	+	1884	1	UBXN2A|U	4.086	4.9
NM_0160	chr5	1.29E+08	1.29E+08	+	1933	1	ISOC1|CGI	8.398	7.192

NM_0012(	chr19	49474561	49486231	+	1066	1	FLT3LG|FL	0.665	0.206
NM_0010(	chr11	55838752	55839738	+	985	1	OR5D16|C	0	0
NM_0032	chr20	2295967	2341079	+	2662	1	TGM3|TGI	0.116	0.082
NM_0013	chr1	77979158	78017964	+	2651	1	DNAJB4|D	2.271	2.273
NM_0012	chr8	1.4E+08	1.4E+08	-	2322	1	KCNK9|K2	0.366	0.602
NM_0163(	chr15	55181314	55197033	-	1465	1	RSL24D1|(	37.026	33.985
NR_03323	chr9	1.23E+08	1.23E+08	-	6323	1	STRBP|HEI	9.914	9.83
NM_0174	chr22	18149991	18177395	+	2022	1	USP18|ISG	0.217	0.664
NM_0049(	chr19	13150468	13154904	+	2042	1	IER2|ETR1	26.627	41.654
NM_0031	chr16	89508388	89557768	+	3074	1	SPG7|CAR	31.332	32.278
NR_11074	chr18	55105904	55124306	-	3064	1	LINC0192	0	0
NM_0147	chr11	73308618	73369380	+	7790	1	ARHGEF17	5.172	8.007
NM_0245	chrX	1.2E+08	1.2E+08	-	1574	1	NKAP|-|-|	7.43	6.144
NM_1942	chr8	8702156	8704107	+	1949	1	CLDN23|C	0	0.007
NM_0055	chr9	14081843	14314046	-	8253	1	NFIB|CTF|	2.5	33.292
NR_01540	chr7	44039049	44042483	+	1710	1	LINC00957	2.006	1.746
NM_0028	chr1	1.51E+08	1.51E+08	+	1319	1	PSMD4|AI	104.712	91.881
NM_0011	chr5	73120561	73131817	+	1257	1	TMEM171	0	0.011
NR_10353	chr8	375931	383174	-	4009	1	FAM87A|-	0.045	0.048
NR_12059	chr22	42364400	42369208	-	818	1	LINC01315	14.17	10.432
NM_1390(	chr6	26087281	26095241	+	2145	1	HFE|HFE1	0.018	0.07
NM_0011	chr5	1.79E+08	1.79E+08	+	2322	1	ZNF454|-|	1.893	3.144
NM_0160	chr1	2.18E+08	2.18E+08	+	7764	1	RRP15|CG	1.184	1.341
NR_04622	chr6	2245753	2413591	+	1581	1	GMDS-AS1	0.824	0.892
NR_03038	chr16	770183	770277	+	94	1	MIR662|M	0	0
NM_0069	chr21	34073523	34106262	+	11619	1	SLC5A3|B(	6.274	3.84
NM_0000	chr9	1.13E+08	1.13E+08	-	3169	1	ALAD|ALA	12.373	10.071
NM_0132	chr17	75667116	75708062	+	2712	1	SAP30BP|I	32.367	36.268
NR_13581	chr1	1.21E+08	1.21E+08	-	550	1	LOC10192	0	0
NM_1758	chr21	30812697	30813252	-	553	1	KRTAP8-1|	0	0
NM_0038(	chr19	6661253	6670588	-	4771	1	TNFSF14|(	0	0.003
NM_0020(	chr4	1.23E+08	1.23E+08	+	6769	1	FGF2|BFGI	1.432	1.338
NM_1445	chr10	75233971	75236012	-	966	1	COMTD1|-	9.17	10.195
NR_12157	chr9	63017995	63028857	+	1258	3	LOC10192	0	0
NM_0007	chr19	40447784	40465818	-	849	1	BLVRB|BV	25.164	20.425
NM_1537(	chr11	1.08E+08	1.08E+08	-	4301	1	KDELC2|-|	0.857	0.494
NM_0011(	chr6	29672392	29677154	-	1723.5	8	ZFP57|C6c	0	0
NR_12047	chr12	1.08E+08	1.08E+08	+	1709	1	LOC10192	1.992	1.683
NM_0183	chr3	1.84E+08	1.84E+08	+	2549	1	ABCF3|EST	19.419	21.806
NM_0203	chr11	22338121	22379500	+	3908	1	SLC17A6|I	22.003	3.757
NM_0011	chr3	1.36E+08	1.36E+08	+	4364	1	PPP2R3A|	6.887	7.338
NM_0186	chr4	5014586	5019470	-	995	1	CYTL1|C17	0	0.014
NM_1532	chr19	57527325	57540876	+	4077	1	ZNF549|-|	4.133	5.117
NM_0010	chr14	24426534	24429525	-	2339	1	CBLN3|PR	0.524	0.24
NR_03649	chr2	61141595	61144975	-	2552	1	LOC33980	6.341	3.58
NR_03648	chr18	4264602	4296000	+	3217	1	DLGAP1-A	0	0.004
NM_0012	chr1	81699770	81992738	+	5963	1	ADGRL2|C	20.611	9.05
NR_11082	chr17	68628154	68679606	+	2611	1	LINC01482	0	0
NM_1943	chr22	39529093	39532855	-	921	1	RPS19BP1	74.323	59.502
NR_03748	chr3	79507887	79507969	+	82	1	MIR3923|-	0	0

NM_0243:	chr1	2.28E+08	2.28E+08	-	1421	1	C1orf35|M	14	12.83
NM_0010(	chr11	1.24E+08	1.24E+08	-	940	1	OR8B2|OF	0	0
NM_0067:	chr7	27100354	27102775	-	1774	1	HOXA2|HC	0.073	1.374
NM_0139	chr19	15807007	15808126	-	1117	1	OR10H1|-	0	0
NR_10673	chr3	1.24E+08	1.24E+08	+	105	1	MIR6083|I	0	0
NM_0016!	chr21	33903453	33915854	-	797	1	ATP5O|AT	397.401	350.932
NR_11066	chr19	22532626	22533494	+	566	1	LINC01233	0	0
NR_00154	chrY	22296798	22298876	-	1560	1	TTTY5|LIN	0	0
NM_0013(	chr2	1.08E+08	1.08E+08	+	5122	1	SLC5A7|CI	0.106	0.019
NM_0013!	chr22_KI2	267288	267961	+	511.5	4	DDT|DDCT	0.63	0.187
NR_04650	chrY	14793642	14804033	-	528	1	NLGN4Y-A	0.122	0.026
NR_12034	chr15	67834308	67838879	-	1985	1	LOC10192!	1.487	3.823
NR_12577	chr13	30419439	30422244	-	533	1	LINC01058	0.024	0.026
NM_0147!	chr2	1.44E+08	1.45E+08	-	9231	1	ZEB2|HSP(	6.741	27.817
NM_0174:	chr2	2.19E+08	2.19E+08	-	2283	1	PRKAG3|A	0	0
NM_0007(	chr15	74748843	74756600	+	3118	1	CYP1A2|CI	0.004	0
NM_0010(	chr5	1.36E+08	1.36E+08	+	6930	1	SMAD5|D\	3.684	5.196
NM_1523:	chr10	1.28E+08	1.28E+08	-	1138	1	CLRN3|TM	0.045	0.072
NM_1749:	chr8	1.27E+08	1.27E+08	-	5477	1	FAM84B|E	5.443	3.724
NM_0011(	chr9	1.29E+08	1.29E+08	+	1170	1	PHYHD1|-	0.121	0.164
NM_0013(	chr16	48244167	48353979	+	4810	1	LONP2|LO	11.315	13.552
NM_0011(	chr7	45000188	45076470	+	1709	1	CCM2|C7c	34.81	32.644
NR_02794	chr12	991208	1495933	+	8486	1	ERC1|Cast	14.895	21.49
NM_0164:	chr18	47032572	47035617	-	3043	1	ELOA2|Hs	0.025	0.023
NM_0326	chr11	55883297	55891808	+	1618	1	TRIM51|SI	0	0
NR_00159	chr6	29750807	29751148	-	341.7	7	IFITM4P|d	0	0
NR_03339	chr11	65213840	65242757	+	2367	1	SLC22A20|	0.409	0.689
NR_11194	chr21	39313907	39314915	+	1008	1	BRWD1-IT:	0.985	1.318
NM_0011:	chr7	76201893	76287292	+	3608	1	SRRM3|-|-	19.272	15.174
NR_11013	chr3	7952805	8016307	-	2679	1	LOC10192	0.014	0.102
NM_0012:	chr17	41041318	41167743	-	443.8	6	KRTAP9-7|	0	0
NM_0011(	chr17	65137338	65210662	+	1640	1	RGS9|PERI	3.499	4.485
NR_04748	chr13	90060250	90119717	-	3856	1	LINC00559	0	0
NM_0029	chr9	34709005	34710167	-	889	1	CCL21|6Ck	0	0
NM_0013:	chr10	1.32E+08	1.32E+08	+	6076	1	JAKMIP3|(	0.842	1.037
NM_0325	chr1	26051305	26067634	-	1759	1	TRIM63|IF	0.007	0.047
NM_0011(	chr17	5419641	5433020	+	1356	1	RPAIN|HRI	77.542	81.425
NR_02768	chr20	64034344	64039962	+	5260	1	LINC00176	1.262	1.503
NR_00154	chrY	9317061	9322874	-	423	1	RBMY1A3I	0	0
NM_0022(	chr5	52989326	53094779	+	7837	1	ITGA2|BR|	0.214	2.029
NM_1537!	chr2	25232961	25252315	-	3587	1	DNMT3A|I	40.427	28.146
NM_0013:	chr3	41246599	41962440	-	4517	1	ULK4|FAM	0.828	0.631
NR_03988	chr17	80419418	80419493	+	75	1	MIR4730|-	0	1.278
NM_0013(	chr5	43376646	43412391	-	1351	1	CCL28|CCI	0.01	0
NR_02710	chr5	1.74E+08	1.74E+08	+	1782	1	LOC28559	0.398	0.469
NM_0013(	chr8	11676919	11760002	+	2647	1	GATA4|AS	0.005	0.062
NM_1526!	chr19	12969618	12974753	+	1728	1	DAND5|CE	0.007	0.159
NM_0062	chr11	64924671	64934478	+	2734	1	PPP2R5B|I	48.4	59.527
NM_0306(	chr1	1.53E+08	1.53E+08	+	766	1	SMCP|HSN	0	0
NM_0207	chr20	3846799	3876123	+	11762	1	MAVS|CAI	7.132	8.641

NM_0012	chr16	8852942	8868361	-	2702	1	CARHSP1|	92.454	98.008
NM_0010	chr5	1.77E+08	1.77E+08	+	1963	1	EIF4E1B|-|	0.158	0.488
NR_10227	chr19	50825289	50831523	-	1183	1	KLK15|AC	0.022	0.093
NR_13424	chr5	1.33E+08	1.33E+08	-	1206	1	LOC10537	0.053	0.057
NM_0039	chr15	63608618	63833948	-	15117	1	HERC1|M	17.276	17.858
NR_12543	chr8	9188999	9202856	+	709	1	LOC10192	0	0.039
NM_0035	chr6	26021679	26022050	+	369	1	HIST1H4A|	0	0
NM_0011	chr22	26169474	26383597	+	6328	1	SEZ6L|-|-|	16.954	82.641
NM_0037	chr4	95162505	95549210	-	9860	1	UNC5C|U	4.625	5.575
NM_0124	chr6	1.53E+08	1.53E+08	-	1459	1	RGS17|RG	2.846	3.145
NR_03786	chr1	34975699	34978706	-	1884	1	LOC65316	0.383	0.451
NM_0210	chr22	29438583	29442455	+	1492	1	RFPL1|RN	1.979	6.142
NM_1522	chr22	37560464	37569403	+	2157	1	CDC42EP1	1.871	3.467
NM_0003	chr11	6612766	6619461	-	3518	1	TPP1|CLN	12.432	16.761
NM_0321	chr17	50508384	50531501	+	3169	1	MYCBPAP|	0.024	0.052
NM_1471	chr18	12658739	12725741	+	974	1	PSMG2|CL	20.849	29.925
NR_10806	chr7_KI27	105523	111266	+	2466	3	LOC10050	0	0
NR_11021	chr2	1.01E+08	1.01E+08	-	768	1	LOC10192	0	0
NM_0013	chr2	21123986	21144366	+	7154	1	TDRD15|-	0	0
NR_03162	chr12	48654444	48654530	-	86	1	MIR1291|	0.6	0.637
NM_0012	chr2	1.92E+08	1.92E+08	+	3440	1	NABP1|OE	0.375	0.382
NM_0061	chr12	12661061	12696187	-	1737	1	GPR19|-|-	10.956	9.242
NM_1524	chr4	1.17E+08	1.17E+08	-	2024	1	TRAM1L1|	0.115	3.309
NM_0012	chr18	57147062	57150408	+	3344	1	BOD1L2|B	0.513	0.299
NR_10378	chr3	1.26E+08	1.26E+08	-	760	1	CCDC37-A	0	0.036
NM_0012	chr2	2.34E+08	2.34E+08	-	2926	1	HJURP|FA	3.19	2.706
NM_0013	chr11	20599400	20655064	+	2707	1	SLC6A5|G	0.048	0.066
NM_0012	chr20	25006230	25032706	-	3494	1	ACSS1|AC	0.705	0.8
NM_0012	chr17	68515394	68551316	+	1200	1	PRKAR1A|	140.979	119.392
NM_0010	chr5	66144218	66183616	+	6768	1	SREK1|SF	2.9	4.133
NR_03408	chr15	63587710	63600827	-	1719	1	USP3-AS1|	2.363	4.151
NM_1449	chr14	23047061	23057538	-	3424	1	CDH24|CD	10.303	8.381
NR_03411	chr6	1.11E+08	1.12E+08	+	2192	1	TRAF3IP2-	5.553	5.88
NM_0013	chr20	24469806	24666617	+	2082	1	SYNDIG1|	2.613	6.888
NM_0010	chr11	1.24E+08	1.24E+08	-	934	1	OR8D2|JC	0	0
NR_00291	chr10	1.19E+08	1.19E+08	-	127	1	SNORA19|	0	0
NM_0012	chr5	76609095	76708132	+	4078	1	IQGAP2|-|	0.715	0.719
NR_12642	chr12	1.13E+08	1.13E+08	+	377	1	LHX5-AS1|	20.315	26.013
NR_04009	chr12	20014685	20098868	+	523	1	LOC10050	0	0
NR_03891	chr12	1.07E+08	1.07E+08	-	859	1	LOC10050	1.561	1.626
NM_0009	chr10	1.17E+08	1.17E+08	+	1478	1	PNLIP|PL|	0	0
NM_0180	chr1	26879382	26890378	-	1503	1	GPN2|ATP	13.185	12.084
NM_0045	chr12	18261540	18648418	+	4810	1	PIK3C2G|	0.011	0.085
NM_2016	chr19	3594506	3606833	-	1935	1	TBXA2R|B	0.007	0.163
NM_0011	chr19	12646508	12666777	-	3195	1	MAN2B1|	14.596	13.174
NM_0046	chr7	1.42E+08	1.42E+08	+	5300.5	2	MGAM|M	0.046	0.018
NR_04750	chr13	18738100	18740099	-	539	2	LINC00417	0	0
NM_0227	chr11	22622533	22625841	-	3306	1	FANCF|FA	9.773	6.923
NM_0013	chr16	784974	788383	-	1854	1	RPUSD1|C	14.403	14.369
NM_0013	chr16	19501693	19522145	-	2854	1	GDE1|363	37.172	37.036

NM_0321(	chr18	67506582	67516730	-	9277	2	DSEL|C18c	0	0
NR_13198	chr12	1.05E+08	1.05E+08	-	546	1	KCCAT198	0	0
NM_1452:	chr2	2.31E+08	2.31E+08	+	3621	1	B3GNT7|b	0.463	0.367
NM_0122:	chr16	1256272	1258493	+	848	1	TPSD1|MC	0	0
NR_03031	chr6	45197674	45197770	-	96	1	MIR586|M	0	0
NM_0010:	chr11	56375624	56376499	+	910.5	2	OR8U8|-|-	0	0
NM_0011(	chr4	1.12E+08	1.12E+08	+	5342	1	ALPK1|84:	0.374	2.623
NR_12637	chr5	75047719	75052643	-	532	1	LINC01336	0.048	0.309
NR_02989	chr12	95308420	95308513	+	93	1	MIR331|M	0	0
NM_0011(	chr20	38601934	38650652	+	2821	1	ARHGAP4(	0.11	0.165
NM_0012!	chr19	11538717	11550323	+	1624	1	CNN1|HEL	1.699	1.257
NR_13296	chr11	26285585	26288007	-	839	1	LOC10537(	0	0
NM_0012{	chr15	22604338	22617854	-	5169.2	4	GOLGA8J|	0	0
NM_0143!	chr16	2148650	2154140	+	1613	1	RAB26|V4	1.367	6.547
NR_12572	chr12	66130751	66134449	+	621	1	LLPH-AS1|	0.125	0.044
NM_0011:	chr5	95813849	95822873	-	1074	1	GLRX|GRX	38.752	48.526
NM_0252!	chr7	2631969	2664802	+	4806	1	TTYH3|-|-	151.755	145.144
NR_10994	chr20	50172570	50176671	+	702	1	LINC01273	0.331	0.156
NR_12059	chr2	1.79E+08	1.79E+08	+	1954	1	LOC10192	0.185	0.042
NR_02696	chr21	43805758	43812567	-	4596	1	AATBC|-|-	0.233	0.215
NR_04698	chr13	85363603	85544662	+	997	1	LINC00351	0	0
NM_0010:	chr6	1.36E+08	1.36E+08	-	6729	1	BCLAF1|B	5.969	9.27
NM_0011(	chr8	13083363	13116244	-	5770	1	DLC1|ARH	0.686	1.564
NM_0012{	chr16	27460613	27549930	-	6993	1	GTF3C1|TI	37.912	39.17
NM_0049(	chr10	47300224	47313583	+	2651	1	GDF10|BIF	7.923	1.705
NM_0010‹	chr8	10672637	10700593	+	1030	1	C8orf74|-|	0	0
NM_0012{	chr16	87326987	87392107	-	5706	1	FBXO31|FI	11.599	18.296
NM_0011‹	chr6	13304951	13328583	-	1078	1	TBC1D7|M	24.268	32.705
NM_0013:	chr6	5261018	5771592	+	1872	1	FARS2|CO	9.629	9.351
NR_03370	chr1	1.72E+08	1.72E+08	-	2844	1	VAMP4|V/	2.752	3.415
NR_02688	chr16	56192617	56194525	-	1908	1	DKFZP434I	0.351	0.732
NM_0052!	chr16	86578509	86581698	+	3187	1	FOXL1|FKH	0.028	0.004
NR_13427	chr5	76606611	76608969	-	1582	1	LOC10192!	0.016	0
NM_0010:	chr2	1.31E+08	1.31E+08	-	3664	1	WTH3DI|-	1.928	2.8
NM_1453:	chr10	43194533	43266919	-	3223	1	RASGEF1A	0.204	0.654
NR_03989	chr17	75784521	75784607	-	86	1	MIR4738|	0.3	0
NM_0012!	chr9	1.28E+08	1.28E+08	-	1335	1	PTGES2|C!	32.403	27.794
NM_0023:	chr4	3503597	3532497	-	10526	1	LRPAP1|A:	8.99	9.797
NM_0024:	chr22	27748277	27801498	-	7552	1	MN1|MGC	34.989	29.603
NM_0022{	chr12	48567684	48570046	-	722	1	LALBA|LYZ	0	0
NM_0139:	chr6_GL0C	661784	662816	+	1030	6	OR12D2|C	0	0
NM_0011:	chrX	1.54E+08	1.54E+08	-	1332	1	BCAP31|6(	92.916	85.956
NR_11018	chr11	59133973	59142808	-	2242	1	LOC10192	0.242	0.593
NR_00302	chr1	12507246	12507397	+	151	2	SNORA59E	0	0
NM_0051:	chr4	15935570	15938740	-	1353	1	FGFBP1|F(	0.486	1.276
NM_0010(	chr7	15200318	15562015	-	2460	1	AGMO|TM	0	0
NR_04002	chr20	37676910	37683234	+	2108	1	LOC10028	0.012	0.11
NR_01541	chr18	76528655	76559827	+	2240	1	LINC00908	0.052	0.055
NM_0011:	chrX	1.54E+08	1.54E+08	-	1492	1	PNCK|BST	2.428	4.296
NM_0006(	chr7	22727142	22732002	+	1182	1	IL6|BSF-2|	0	0

NR_13553	chr3	1.88E+08	1.88E+08	-	749	1	LINC01991	0	0
NM_0309	chr8	1.44E+08	1.44E+08	-	1805	1	SHARPIN|S	24.901	21.293
NM_0016	chr1	2.28E+08	2.28E+08	+	1881	1	ARF1|-|-|	295.255	291.54
NM_0011	chr13	49247911	49293486	+	2295	1	CDADC1|N	3.382	3.784
NM_1452	chr13	1.12E+08	1.12E+08	+	774	2	SPACA7|C	0	0
NR_10431	chr16	1782923	1789191	+	1186	1	NUBP2|CF	46.932	45.272
NM_1452	chr16	4608883	4614926	-	1408	1	UBALD1|F	12.948	14.573
NM_0011	chrX	23908006	23939507	+	1530	1	CXorf58|-|	0.076	0.045
NM_0252	chr6_GL0C	3242852	3254573	-	2895	6	VWA7|C6	0	0
NM_0306	chr17	74950744	74972805	-	3321	1	HID1|C17	31.319	35.494
NM_0013	chr17	32266176	32324661	+	4627	1	RHBDL3|R	18.514	20.902
NM_0183	chr6	69675749	69797157	-	2290	1	LMBRD1|	12.955	13.685
NR_04703	chr15	25266591	25266661	+	70	1	SNORD115	0	0
NM_1446	chr19	49388218	49417999	+	2361	1	CCDC155|	0.044	0.075
NM_1303	chr18	319355	500729	-	3122	1	COLEC12|	2.821	0.448
NM_0042	chr5	79373824	79513836	-	4301	1	HOMER1|	3.337	5.067
NM_0045	chrX	20149911	20266632	-	7687	1	RPS6KA3|	2.296	2.598
NM_0212	chr3	1.34E+08	1.34E+08	+	2817	1	SRPRB|AP	14.994	17.086
NM_0013	chr1	2.31E+08	2.31E+08	+	2153	1	CAPN9|GC	0.042	0.076
NR_03762	chr2	1.1E+08	1.1E+08	+	852	2	LOC10028	0	0
NR_10687	chr20	45425510	45425573	+	63	1	MIR6812|	1.023	0.87
NM_0012	chr2	2.02E+08	2.03E+08	+	12055	1	BMPR2|BM	6.793	8.836
NM_1982	chr20	2461635	2470853	-	998	1	SNRPB|CC	106.042	89.003
NR_10682	chr15	89326739	89326810	-	71	1	MIR6766|	2.179	2.894
NM_0312	chr10	99610518	99620464	-	1549	1	SLC25A28|	13.867	16.677
NM_0011	chrX	71714374	71718285	-	1843.5	2	CXorf49B|	0	0
NR_03620	chr18	50126499	50126563	-	64	1	MIR4320|-	0	0
NM_1533	chr13	27545913	27620583	-	4792	1	LNX2|PDZ	1.878	2.921
NR_11001	chr2	37826247	37875863	-	2020	1	LINC00211	0.006	0
NM_0010	chr11	58358125	58359069	-	943	1	OR5B17|C	0	0
NR_14455	chr2	10690010	10692095	+	2085	1	RN7SL832	0.668	0.788
NM_0330	chrX	50284540	50351912	+	4497	1	CCNB3|-|-	1.967	1.87
NM_0065	chr6_GL0C	393697	414690	-	2670	7	TRIM27|R	0	0
NM_0025	chr12	5494132	5495299	+	1165	1	NTF3|HDN	1.893	5.526
NM_1720	chr2	1.7E+08	1.7E+08	+	7912	1	UBR3|ZNF	4.509	5.067
NM_0065	chrX	74421493	74533929	+	4133	1	SLC16A2|	41.831	43.368
NM_0321	chr3	14675099	14773036	+	3309	1	C3orf20|-|	0.051	0.017
NM_0190	chr2	67397310	67410401	+	3275	1	ETAA1|ET	0.945	1.472
NM_0330	chr9	92974436	93036236	+	3333	1	FGD3|ZFY	1.555	3.822
NR_03842	chrX	3271820	3284653	+	671	1	LINC01546	0.192	0.122
NR_00156	chrX	73820651	73852753	-	19274	1	XIST|DXS1	0.009	0.019
NM_0010	chr1	53014938	53051617	+	1412	1	SCP2|NLT	25.705	22.592
NM_1446	chr19	2252251	2256423	-	1136	1	JSRP1|JP-4	0.113	0
NR_10674	chr17	49288346	49288454	-	108	1	MIR6129|	0	0.127
NM_0011	chr16	2513726	2520223	+	1015	1	ATP6V0C|	229.847	250.514
NM_0149	chr7	1.3E+08	1.3E+08	+	6426	1	KLHDC10|	17.211	15.801
NR_12164	chr9	62801465	62813486	+	2872	1	LINC0141C	0	0.005
NR_01536	chr9	41100985	41107812	-	3178	1	LOC44089	0.008	0
NM_0010	chr6_GL0C	3459484	3473740	+	5384	9	C4B|C4B1	0	0
NM_0010	chr11	57181747	57191714	+	5403	1	LRRC55|-|	23.229	38.949

NM_0013(	chr9	1.13E+08	1.13E+08	-	1391	1	HDHD3|28	11.438	10.792
NR_10347	chr10	68341107	68407294	-	2155	1	RUFY2|RA	11.404	14.403
NM_0188:	chr1	4655045	4783791	+	2915	1	AJAP1|MC	0.119	2.974
NR_03413	chr12	1.26E+08	1.26E+08	-	2074	1	LINC0093S	0	0
NM_0012{	chr12	48904110	48921576	+	1765	1	CCDC65|C	3.952	2.972
NR_13364	chr20	50934874	50958564	-	1095	1	DPM1|CD(	21.301	25.042
NM_0528:	chr6_GL0C	1462669	1471655	-	2996.4	8	TRIM10|H	0	0
NR_13195	chr4	44016861	44022061	+	2511	1	LVCAT1|-|	0	0.005
NM_0010(	chr11	56252200	56253222	+	1021	2	OR5T3|OR	0	0
NM_0227:	chr22	19130279	19144677	-	5373	1	DGCR14|D	9.709	11.698
NM_1445(	chr15	40594249	40662683	+	7579	1	KNL1|AF1!	0.26	0.719
NR_03396	chr1	95474737	95479356	+	2751	1	LINC01761	0.019	0
NR_03830	chr11	42187743	42253690	-	473	1	LOC10050	0	0
NM_0043:	chr12	1.03E+08	1.03E+08	+	2480	1	ASCL1|ASH	23.193	17.734
NM_0011(	chr1	23304690	23344364	-	7651	1	HNRNPR|H	24.13	25.679
NM_0335:	chrX	48458537	48470256	-	1974	1	SLC38A5|J	0.425	0.271
NR_00291	chr20	38427306	38427443	-	137	1	SNORA71A	1.412	0.8
NR_02443	chr15	57300365	57307769	+	2513	1	LINC00926	0.313	0.463
NM_0012{	chr6	43450352	43455624	-	1598	1	DLK2|DLK-	2.905	3.514
NM_1383:	chr19	23652906	23687215	-	2203	1	ZNF675|TI	2.107	3.587
NM_0071(	chr1	34986166	35031968	-	5126	1	ZMYM6|B	2.332	2.653
NM_0049	chr3	50675241	51384198	+	8700	1	DOCK3|M(	9.51	12.87
NM_0012{	chr7	1.01E+08	1.01E+08	-	2268	1	LRCH4|LRI	14.593	10.665
NM_1528!	chr9	35088688	35096601	-	2451	1	PIGO|HPM	8.459	6.74
NM_0247!	chr10	86935541	86957668	-	4170	1	MMRN2|E	0.439	0.424
NM_0012{	chr16	4425805	4456774	+	2285	2	DNAJA3|H	0.011	0
NR_04520	chr14	74881891	74903747	+	2779	1	DLST|DLTS	40.954	52.117
NM_0013:	chr22	27048144	27060518	-	2379	1	LOC28489:	0.309	0.708
NM_0011(	chr12	2890867	2941140	+	1987	1	TULP3|TU	12.316	12.339
NM_0324:	chr7	2906076	3043945	-	4404	1	CARD11|B	0.029	0.016
NR_03740	chr13	49996415	49996501	-	86	1	MIR3613|-	1.199	0.637
NM_0012{	chr7	1.24E+08	1.24E+08	-	5530	1	NDUFA5|E	7.424	6.167
NM_0012	chr3	1.29E+08	1.29E+08	-	2329	1	MBD4|ME	7.418	6.122
NM_0010:	chr5	16473038	16617058	-	3273	1	FAM134B|	1.871	8.575
NM_0071(	chr6_GL0C	853599	854661	+	1060	7	OR2H2|FA	0	0
NM_0012{	chr21	34516443	34613843	-	2199	1	RCAN1|AD	21.114	67.109
NR_10682	chr16	4671318	4671390	+	72	1	MIR6769A	8.058	12.936
NR_03742	chr12	1660315	1660380	-	65	1	MIR3649|-	0	0
NM_0013:	chr5	24487100	24593499	-	2920	1	CDH10|-|-	5.034	5.84
NR_11066	chr1	2.24E+08	2.24E+08	+	1320	1	LOC10192	3.614	3.009
NM_0225(	chr17	63917193	63918852	-	720	1	GH1|GH|(	0	0
NM_0013(	chr12	1.23E+08	1.23E+08	-	6752	1	PITPNM2|	2.227	2.741
NR_13521	chr1	1.58E+08	1.58E+08	-	2899	1	FCRL3|CD:	0.022	0.094
NR_03652	chr9	97238426	97297314	+	3202	1	LOC10049	0.072	0.128
NM_0008:	chr7	30964021	30979531	+	1592	1	GHRHR|GI	0	0
NM_0012!	chr1	2.31E+08	2.31E+08	-	1342	1	LOC14937	0.01	0.051
NR_10376	chr22	19171706	19172832	+	1126	1	LINC01311	0.962	0.693
NM_0012{	chr11	83259093	83286407	-	4327	1	CCDC90B|	11.556	12.203
NM_0051{	chr10	22321210	22331485	+	3418	1	BMI1|FLVI	8.808	11.233
NM_0062:	chr2	1.05E+08	1.05E+08	+	4503	1	POU3F3|B	33.008	52.978

NR_02440	chr16	88663373	88675506	+	2426	1	SNAI3-AS1	4.411	3.568
NM_0204	chrY	23129355	23199107	-	3353	4	DAZ4|pDP	0	0
NR_02674	chrX	49876724	49879356	-	2288	1	USP27X-AS	0.952	0.629
NM_0001	chr6	6144078	6320691	-	3846	1	F13A1|F13	0.013	0.011
NM_0208	chr2	2.24E+08	2.24E+08	-	4650	1	WDFY1|FE	10.789	8.059
NM_0582	chr17	58692573	58734342	+	1311	1	RAD51C|B	6.314	6.551
NM_0013	chr3	48751585	48847850	-	1373	1	PRKAR2A|	24.144	20.97
NM_0011	chr12	1.11E+08	1.11E+08	-	3144	1	FAM109A|	10.987	11.636
NM_0013	chr3	1.06E+08	1.06E+08	-	6583	1	CBLB|Cbl-I	25.799	16.672
NM_0013	chr19	1905214	1926017	+	2634	1	SCAMP4|S	25.018	27.222
NR_00214	chr1	2.48E+08	2.48E+08	+	924	1	OR2L1P|H	0	0.044
NR_11081	chr17	59965096	59973101	+	422	1	LOC10192	0.611	0.649
NR_00361	chr5	1.78E+08	1.78E+08	+	1821	3	LOC72855	0	0
NR_05196	chr13	96941281	96984172	-	731	1	LINC0035	0.088	0.019
NM_0013	chr1	24899511	24965010	-	4164	1	RUNX3|AN	0.028	0.03
NM_0068	chr14	73569059	73575658	+	1774	1	ACOT2|CT	0.567	0.409
NR_02769	chr3	19979961	20012273	-	1604	1	PP2D1|C3	1.857	1.708
NR_02727	chr16	2737076	2752600	-	839	1	SRRM2-AS	0.907	0.359
NM_0031	chr8	1.2E+08	1.2E+08	-	4992	1	TAF2|CIF1	5.933	5.205
NM_0141	chr16	67227113	67229279	+	786	1	TMEM208	34.744	27.063
NR_03991	chr20	54068408	54068485	-	77	1	MIR4756|	0	0
NR_00395	chr6	1.47E+08	1.47E+08	-	2155	1	KATNBL1P	0.933	1.036
NM_0164	chr11	61392393	61398863	+	1280	1	TMEM216	3.304	6.816
NM_0209	chr16	2911979	2940157	+	2525	1	FLYWCH1|	28.085	28.837
NR_10672	chr10	2076019	2076089	-	70	1	MIR6072|	0	0
NM_0179	chr22	46267964	46294008	+	2594	1	TTC38|LL2	8.012	7.588
NM_0050	chr8	71841542	71844496	-	2054	1	MSC|ABF-	1.124	0.147
NM_0013	chr14	1.05E+08	1.05E+08	+	470	1	CRIP1|CRH	1.18	0.699
NM_1445	chr14	20457853	20461612	-	1818	1	TMEM55B	31.794	36.766
NR_03324	chr2	2.06E+08	2.06E+08	-	999	1	GCSHP3|-|	2.543	4.25
NR_10705	chr19	44758657	44758721	+	64	1	MIR8085|	0	0
NM_0012	chr2	20684014	20823130	-	3799	1	LDAH|C2o	10.487	10.229
NM_0011	chr2	1.7E+08	1.7E+08	+	4221	1	PHOSPHO	12.258	9.466
NM_1825	chr2	1.19E+08	1.19E+08	-	2045	1	C1QL2|C1	0.025	0.208
NR_04611	chr2	1.1E+08	1.1E+08	+	2330.5	2	LINC01123	0	0.006
NM_0062	chr20	43457864	43463604	+	3738	1	SRSF6|B52	31.289	35.869
NM_0010	chr22	30378814	30387313	-	1910	1	RNF215|-|	19.914	18.753
NM_0013	chr1	1.55E+08	1.55E+08	-	942	1	PMVK|HU	46.824	44.159
NM_0013	chr19	19865830	19893484	+	2177	1	ZNF253|BI	4.211	9.186
NM_0582	chr8	7450564	7452365	-	440	2	SPAG11B|	0	0
NR_04979	chr2	2.33E+08	2.33E+08	-	99	1	MIR5001|	0.521	0.277
NM_0027	chr1	2.14E+08	2.14E+08	+	8165	1	PROX1|-|-	0.434	1.124
NR_10692	chr12	1.12E+08	1.12E+08	-	63	1	MIR6861|	11.052	14.567
NM_0012	chr9	1.05E+08	1.06E+08	+	7616	1	FSD1L|CCI	2.903	2.867
NM_0012	chr17	50547089	50555852	+	2653	1	SPATA20|	10.026	12.148
NM_0004	chr17	40818117	40822611	-	2130	1	KRT10|BCI	7.312	7.312
NM_0013	chr22	39130774	39152650	-	3792	1	CBX7|-|-|	3.234	3.843
NR_13450	chr10	61406642	61453450	-	2816	1	TMEM26|	0.114	0.024
NR_10997	chr12	27389789	27446634	-	809	1	ARNTL2-AS	0.032	0.119
NM_0243	chr16	55324559	55330760	+	2321	1	IRX6|IRX-3	2.772	4.875

NR_02677	chr6	27019366	27020306	-	940	1	LOC10027(	0.439	0.525
NR_11197	chr7	66553468	66592407	-	1684	1	GS1-124K5	15.282	15.779
NM_0159(	chr19	19516210	19528204	+	535	1	NDUFA13|	343.593	292.809
NM_0013:	chr7	99506005	99533822	+	3990	1	ZKSCAN5|;	11.443	11.878
NM_0152(	chr12	1.11E+08	1.11E+08	+	6822	1	CUX2|CDP	12.78	8.778
NM_0219!	chrX	1.03E+08	1.03E+08	-	661	1	TMSB15A|	196.562	184.174
NM_0012!	chr1	1.61E+08	1.61E+08	+	2706	1	TOMM40L	22.228	25.455
NR_04657	chr3	56940040	56960852	+	484	1	ARHGEF3-/	0	0
NR_03366	chrY	12406116	12421590	-	819	1	GYG2P1|G	0	0
NR_02696	chr6	1.33E+08	1.33E+08	+	1210	1	LINC0032(	0.224	0.034
NM_0052!	chr13	26755201	26760785	-	4845	1	GPR12|GP	0.303	4.362
NR_10400	chr7	75391949	75395413	-	871	2	LOC10010	0.311	0.173
NM_0011(	chr8	1.44E+08	1.44E+08	-	1317	2	CCDC166|	0	0
NM_1749:	chr5	36035017	36066921	-	2376	1	UGT3A2|-	0.087	0.035
NM_0062!	chr14	61321443	61550980	+	3844	1	PRKCH|PK	0.06	0.278
NM_0054;	chr15	73319859	73369264	-	7218	1	HCN4|SSS:	9.08	9.414
NM_0012(	chr14	87933014	87993271	-	3796	1	GALC|-|-|:	0.985	0.981
NM_1987:	chr1	70852353	71047808	-	1810	1	PTGER3|EI	0.114	0.386
NR_13464	chr6	46670444	46687833	-	374	1	LOC10192(	0.103	0
NR_12652	chr5	441836	443160	-	1324	1	EXOC3-AS:	6.963	6.673
NM_0068;	chr11	66345372	66347690	-	2066	1	B4GAT1|B	62.183	68.26
NR_10993	chr20	36507702	36573275	-	1202	1	DLGAP4-A:	0.064	0.057
NM_0010:	chr20	31257664	31259632	+	264	1	DEFB115|I	0	0
NM_0011!	chrX	88747225	88754784	+	1586	1	CPXCR1|C	0	0
NM_0010:	chr4	41981696	41986467	+	4769	1	DCAF4L1|\	0.073	0.086
NR_04702	chr13	32809190	32911652	-	1351	1	LINC00423	0.038	0
NM_0186!	chr5	1.54E+08	1.54E+08	-	4443	1	FAM114A2	5.882	5.232
NM_0010!	chr8	1.32E+08	1.32E+08	-	1766	1	OC90|PLA	0	0.016
NR_11027	chr2	2E+08	2E+08	+	2339	1	LINC01877	0	0
NM_0013:	chr17	68421281	68457524	-	1839	1	WIPI1|ATC	14.226	7.277
NR_12210	chr3_KI27(	81285	103902	+	935	8	LINC0096!	0.262	0.059
NR_13234	chr22_KI2:	270138	270389	+	251	1	GSTT1-AS1	0.668	0.928
NM_0013:	chr2	2.42E+08	2.42E+08	+	963	1	ING5|p28I	8.971	7.339
NM_0013:	chr10	63167221	63522075	-	8484	1	JMJD1C|KI	2.308	7.217
NM_0153:	chr4	1.22E+08	1.22E+08	+	15489	1	KIAA1109|	3.748	6.128
NM_0012(	chr4	46918900	46993505	-	10992	1	GABRA4|-	0.011	0.079
NR_03401	chr16	35505088	35507170	-	1298	1	TP53TG3H	0	0
NM_0145!	chr1	19920307	19923617	-	481	1	PLA2G2E|(	0	0
NR_03838	chr10	89829493	89840861	+	4285	1	LINC00865	0	0.003
NR_02336	chr1	2.29E+08	2.29E+08	-	120	10	RNA5S2|R	0	0
NR_11054	chr14	58828288	59017328	+	746	1	LINC0150C	0	0.055
NM_0010:	chr19	5904841	5910252	+	1666	1	VMAC|-|-|	2.345	2.483
NM_0057:	chr4	98470367	98658661	-	3387	1	TSPAN5|N	24.23	46.732
NR_04521	chr18	31542146	31556911	-	1980	1	DSG2-AS1	0.085	0.076
NM_0058(	chr21	38054011	38121360	-	1093	1	DSCR4|DC	0	0
NM_0153(	chr14	76787243	76812940	-	4099	1	ANGEL1|C	4.954	7.719
NM_0012!	chr19	45769709	45779619	-	2495	1	DMPK|DM	30.464	22.98
NM_0307!	chr11	75434640	75525554	-	3155	1	GDPD5|GI	22.199	22.549
NM_0132	chr16	67229389	67247522	-	3830	1	FHOD1|FH	2.367	2.232
NM_0011(	chr6	82364244	82367416	+	2876	1	TPBG|5T4	31.812	5.791

NM_0121:	chr19	15049480	15058292	+	3135	1	CASP14|-|	0.012	0.004
NM_0067:	chrX	1.2E+08	1.2E+08	+	5205	1	ZBTB33|ZI	4.471	6.06
NR_13763	chr14	24214310	24232385	-	603	1	NEDD8-MI	146.896	121.615
NM_2069(	chr16	85707518	85751083	-	891	1	C16orf74|	0.275	0.984
NM_1826!	chr20	33055331	33073637	+	1539	1	BPIFB3|C2	0	0
NM_0010(	chr17	4731408	4733610	+	854	1	MED11|HS	19.159	15.734
NR_02667	chr16	21300849	21318591	+	2301	1	CRYM-AS1	0	0.042
NM_0203(	chrY	24833820	24907040	+	3495	3	DAZ3|pDP	0	0
NR_13046	chr14	27633205	27633278	-	73	1	MIR3171|-	0	0
NM_0153:	chr14	1.04E+08	1.04E+08	-	4940	1	PPP1R13B	4.706	8.778
NM_0010(	chr10	5878087	5889897	-	1585	1	ANKRD16|	6.5	6.403
NR_03742	chr12	1.04E+08	1.04E+08	+	130	1	MIR3652|-	21.522	10.958
NM_0065:	chr2	2.19E+08	2.19E+08	+	1694	1	WNT6|-|-|	0.015	0.089
NM_0221!	chr10	71747556	71773580	-	4765	1	VSIR|B7-H	0.038	0.144
NR_04755	chr7	55179750	55188949	-	2819	1	EGFR-AS1|	0.114	0.369
NM_0335!	chr20	46684365	46689637	-	3369	1	TP53RK|BI	10.368	17.035
NM_0010²	chr5	6633322	6669562	+	2322	1	SRD5A1|S!	23.632	20.976
NR_02671	chr10	1.11E+08	1.11E+08	-	652	1	RPL13AP6	1.048	1.05
NR_13509	chr11	1.06E+08	1.06E+08	-	405	1	LOC10536!	0	0
NM_0011:	chr1	84302651	84350798	+	1399	1	SAMD13|I	2.829	3.642
NM_0067(	chr12	1.12E+08	1.12E+08	+	2634	1	TRAFD1|FI	43.512	53.805
NM_0033²	chr12	93408312	93442250	-	2546	1	UBE2N|HE	37.708	49.86
NM_0011(	chr22	37469063	37486387	-	2025	1	MFNG|-|-	19.617	7.528
NM_0011!	chr14	31482944	31487945	-	1073	1	GPR33|-|-	0	0
NR_02808	chrY	17500958	17516742	+	2033	2	FAM41AY1	0	0
NM_0025:	chr1	1.15E+08	1.15E+08	-	4445	1	NRAS|ALP.	19.35	17.663
NR_11052	chr4	124476	202306	+	1222	1	ZNF718|-|	1.804	2.006
NM_2034(	chr5	90458203	90474768	-	4276	1	MBLAC2|-	1.191	1.14
NM_0189:	chr5	1.41E+08	1.42E+08	+	4593	1	PCDHGB6|	20.714	88.99
NM_0181²	chr17	41848519	41865431	-	7405	1	KLHL11|-|	1.87	2.181
NM_0036(	chr5	78002326	78294755	-	4008	1	AP3B1|AD	4.443	5.273
NM_2073:	chrX	70062491	70064179	+	1686	1	OTUD6A|I	0	0
NM_1825:	chr2	68945232	68952970	-	792	1	GKN2|BRI(	0	0.035
NM_0146(	chr8	1.45E+08	1.45E+08	+	5361	1	LRRC14|LF	13.124	13.825
NM_0323!	chr3	49908869	49930012	-	2222	1	MON1A|S.	12.789	14.788
NM_0011!	chr4	1E+08	1.01E+08	-	3985	1	EMCN|EM	0.036	0.007
NM_0146!	chr12	1.08E+08	1.08E+08	+	4887	1	WSCD2|-|	5.2	2.052
NR_03985	chr14	65044688	65044769	+	81	1	MIR4706|I	0	0
NM_0010:	chr17	41722639	41734646	-	3847	1	HAP1|HAF	3.747	3.842
NM_0013:	chr10	1.26E+08	1.26E+08	-	766	1	TEX36|C1(	0	0
NM_0210:	chr17	21376387	21419867	+	5225	1	KCNJ12|IR	0.358	0.446
NM_0069:	chr18	21612269	21630245	+	1622	1	SNRPD1|H	40.811	39.166
NM_2034:	chr17	7252053	7259940	+	1302	1	ELP5|C17c	44.573	44.994
NM_0332:	chr5	1.03E+08	1.03E+08	+	3044	1	C5orf30|-|	8.128	6.974
NM_0144(	chr1	36136569	36149514	-	1306	1	TRAPPC3|I	77.627	90.845
NM_0047(	chr11	76349957	76380965	-	3315	1	THAP12|D	6.165	9.028
NM_1524{	chr1	925741	944581	+	2538	1	SAMD11|I	8.682	11.927
NM_0210:	chr15	84655129	84658571	-	1025	1	NMB|-|-|:	10.742	1.991
NM_0054:	chr11	74454841	74467555	-	3065	1	KCNE3|HC	0.008	0
NR_00329	chr15	25174927	25175008	+	81	1	SNORD115	0	0

NM_0012:	chr17	47651196	47683638	+	3924	1	KPNB1|IM	63.672	55.874
NM_0012:	chr16	3658037	3717597	-	2118	1	TRAP1|HS	56.706	66.804
NR_10269	chr18	3962353	4013943	+	752	1	DLGAP1-A:	0	0.018
NM_0012(	chr6	46652915	46678190	+	2711	1	SLC25A27|	5.16	4.87
NR_00438	chr8	54330686	54330895	+	209	1	RNU105C|	0	0
NM_0239:	chr19	17305668	17306843	+	964	1	MRPL34|L	43.281	33.276
NR_00318	chr7	75156578	75171998	-	1431	1	NCF1C|SH	0	0
NM_0168:	chr12	6462238	6470899	-	1306	1	VAMP1|SF	3.446	2.643
NR_02673	chr14	28772704	28778560	+	811	1	LINC01551	0.111	5.573
NM_0013:	chr9	93096123	93113295	+	896	1	CARD19|B	31.874	26.997
NR_03020	chr19	53726922	53726986	+	64	1	MIR526A2	0	0
NR_13517	chr16	11341810	11345212	-	712	1	LOC10537	0.145	0.212
NM_0010!	chr1	25819906	25832942	+	1887	1	MTFR1L|F.	74.204	80.585
NR_03166	chr7	1.02E+08	1.02E+08	-	113	1	MIR548O|	0	0
NM_0011-	chr19	18929201	18940352	-	1406	1	HOMER3|I	34.856	35.11
NM_1826-	chr14	34752731	34875647	-	5892	1	BAZ1A|AC	2.578	3.912
NR_02949	chr19	13836587	13836659	-	72	1	MIR23A|M	0	0
NR_12066	chr10	82229014	82232920	-	486	1	NRG3-AS1	0.212	0.085
NM_1736:	chr17	48762231	48794324	-	2531	1	TTLL6|TTL	0.148	0.076
NM_0011-	chr10	12129641	12165404	+	2449	1	SEC61A2|-	31.131	38.58
NM_0046:	chr11	76186326	76206530	-	1920	1	WNT11|H'	0.181	0.649
NM_0219:	chr6	35452361	35467104	+	2543	1	FANCE|FA	9.177	9.35
NR_11053	chr22_KI2	79522	81029	+	914	2	LINC0165!	0	0
NM_0012!	chr6	46159182	46171010	-	3519	1	ENPP5|NP	0.553	0.954
NM_0018(	chr7	1.3E+08	1.3E+08	+	1316	1	CPA2|-|-|	0.549	1.072
NR_13468	chr4	1.71E+08	1.71E+08	-	629	1	LOC10050	0	0
NM_0013(	chr12	96489571	96875555	+	9712	1	CFAP54|C:	0.1	0.055
NM_0011:	chr3	1.7E+08	1.7E+08	-	1827	1	LRRC34|-|	0.099	0.202
NM_0002!	chrX	1.51E+08	1.51E+08	+	3419	1	MTM1|CN	2.432	2.556
NR_02410	chr18	3594114	3597379	+	990	1	DLGAP1-A:	2.605	1.176
NR_02704	chr1	9652610	9654586	-	1821	1	PIK3CD-AS	0	0
NM_0010-	chr13	77698881	77764242	+	2337	1	SLAIN1|C1	36.336	75.94
NM_0156!	chr16	11834196	11851585	-	5126	1	RSL1D1|C!	16.777	18.266
NR_05201	chr6_GL0C	1544684	1636359	-	710.6	7	HCG17|HC	0.49	0.559
NR_00272	chr10	79920178	79923119	+	1421	1	MBL1P|CC	0.345	0.183
NR_00278	chr15	72675782	72686149	-	1114	1	HIGD2B|H	0.046	0.025
NM_0015!	chrX	1.54E+08	1.54E+08	-	1536	1	TEX28|CX	0	0
NR_02676	chr4	74054038	74055399	-	1361	1	PPBPP2|PI	0	0
NM_0314:	chr12	2825348	2835055	-	2769	1	NRIP2|-|-|	0.959	0.777
NR_13640	chr17	404535	414023	-	385	1	LOC10537	12.257	13.057
NM_0205:	chr12	68248245	68253501	-	1140	1	IL22|IL-21	0.136	0.024
NR_12059	chr11	8768778	8810276	+	969	1	LOC10272-	0.04	0
NR_10418	chr14	76781733	76786724	-	1682	1	LOC10050	32.004	42.427
NM_0011!	chr5	97166746	97183301	-	2218	1	RIOK2|RIC	3.098	3.051
NR_03417	chr2	2.18E+08	2.18E+08	+	3608	1	RUFY4|ZF\	0	0
NR_13091	chr17	50478800	50485980	+	2167	1	RSAD1|-|-	13.399	22.622
NM_0010(	chr7	1.44E+08	1.44E+08	-	931	2	OR2A1|-|-	0	0
NM_0210:	chr8	20246165	20255292	-	5454	1	LZTS1|F37	31.799	35.9
NM_0015:	chr9	33025203	33039907	+	2378	1	DNAJA1|D	211.197	190.336
NM_0012!	chr16	8569500	8590193	-	893	1	TMEM114	0.058	0.061

NM_0209(	chr19	57120141	57131925	+	3600	1	USP29|HC	0	0
NM_0000⁄	chr5	78777211	78986534	-	6064	1	ARSB|ASB	6.838	6.668
NM_0010{	chr4	1.57E+08	1.57E+08	+	5247	1	GRIA2|GLU	5.49	19.385
NM_0184;	chr2	1.86E+08	1.87E+08	+	2121	1	ZC3H15|H	16.431	22.176
NR_03059	chrX	1.46E+08	1.46E+08	-	78	1	MIR891B|	0	0
NM_1785:	chr12	61708248	62192839	-	4129	1	FAM19A2|	1.733	1.818
NM_0334:	chr14	24606480	24609720	-	964	1	GZMH|CCI	0	0
NM_0010{	chr8	47260878	47736001	+	3358	1	SPIDR|KIA	8.62	8.806
NM_0183:	chr7	1.51E+08	1.51E+08	+	1960	1	GIMAP4|I/	0	0.007
NR_03038	chr17	67471491	67471585	-	94	1	MIR548D2	0	0
NR_13178	chr10	59173963	59176464	-	2501	1	CCEPR|CCI	5.918	7.443
NM_0037:	chr11	6621324	6655849	-	10736	1	DCHS1|CD	37.305	39.796
NR_03989	chr18	22933349	22933438	+	89	1	MIR4741|	0	0.616
NM_0012!	chr3	42502612	42537573	+	2415	1	VIPR1|HVI	0.043	0.051
NM_0010⁄	chr4	1.75E+08	1.75E+08	-	3618	1	GLRA3|-|-	0.15	0.288
NM_0013(	chr10	75431646	76557376	+	839	1	C10orf11|	0.046	0.294
NM_0239⁄	chr11	60429589	60447792	+	715	1	MS4A5|CD	0	0
NR_00439	chr17	76561108	76561193	+	85	1	SNORD1B|	0	0
NM_0010(	chr5	1.76E+08	1.76E+08	+	4670	1	CPLX2|921	61.094	57.12
NM_0155:	chr17	21126928	21191972	+	1832	1	DHRS7B|C	9.825	9.323
NR_12577	chr13	79566727	79571446	+	633	1	LINC0106{	0.081	0.13
NM_0027!	chr1	1.51E+08	1.51E+08	+	916	1	PSMB4|HN	247.116	236.229
NM_0011;	chr15	51447724	51622770	-	10554	1	DMXL2|PE	4.958	7.8
NM_0057:	chr10	79347464	79355333	+	2192	1	PPIF|CYP3	19.228	26.4
NM_0011!	chr5	1.22E+08	1.22E+08	+	436	1	LOC10050.	0	0
NM_0152{	chr15	42158701	42208326	-	4827	1	VPS39|TLI	33.317	38.075
NM_0013:	chr2	37084451	37099244	+	3854	1	GPATCH11	4.547	4.531
NM_1784!	chr20	58150927	58161127	+	751	1	C20orf85|	0	0
NR_02832	chr1	490756	495445	-	4367	2	LOC10013.	0.03	0.072
NM_0246;	chr4	82900650	82919969	+	3903	1	THAP9|hT	0.641	0.976
NR_03998	chr8	68911803	69104190	-	2362	1	LINC01592	0	0
NM_0010:	chr7	1.03E+08	1.03E+08	+	1972	2	SPDYE2|SI	0	0
NR_00326	chr15	99790156	99806927	-	3826	1	DNM1P46	0.044	0.032
NM_0176{	chrX	1.07E+08	1.07E+08	-	1751	1	NUP62CL|	0.376	0.438
NR_14436	chr14	61715558	61751097	-	530	1	LOC10537	0.097	0
NM_0066:	chr1	2.03E+08	2.03E+08	-	9622	1	KDM5B|CT	52.03	68.747
NM_0010;	chr21	30337013	30337694	-	679	1	KRTAP27-1	0	0
NR_13064	chr17	76274049	76307680	-	2431	1	QRICH2|-|	3.723	5.24
NM_0011!	chr19	7669086	7670454	+	463	1	RETN|ADS	0.139	0
NR_10371	chr10	1.21E+08	1.21E+08	+	2174	1	LINC01561	0.012	0.359
NR_12616	chr7	5513854	5525545	+	1822	1	LOC22194	0.021	0.105
NM_1735:	chr9	1.35E+08	1.35E+08	+	1915	1	C9orf62|-|	0	0
NM_0011⁄	chr16	20806445	20849668	+	2662	1	LOC81691	3.332	4.255
NM_0013:	chr10	70431936	70447948	-	1871	1	NODAL|HT	0.6	1.193
NM_0208{	chr20	63293186	63330933	+	4134	1	COL20A1|	0.396	0.093
NM_1538(	chr9	32629454	32635669	-	6213	1	TAF1L|TAI	0.554	0.679
NM_0011!	chr4	82818509	82891280	-	4269	1	SEC31A|AI	43.993	43.623
NM_0011⁄	chr12	44508275	44875661	-	3188	1	NELL2|NRI	213.81	119.192
NM_0201!	chr2	96184861	96208835	-	3373	1	STARD7|G	64.399	81.252
NM_0013:	chr3	12312380	12434356	+	1262	1	PPARG|CII	0.041	0.076

NM_0011!	chr2	32628020	32821051	+	2649	1	TTC27|-|-|	3.641	4.333
NM_0010(	chr1	2.49E+08	2.49E+08	-	949	1	OR2T11|O	0	0
NM_0012(	chr20	58689131	58715844	+	2063	1	NPEPL1|b/	6.919	6.865
NR_10376	chr11	10308313	10430673	+	1015	1	CAND1.11	0.013	0
NM_0010{	chr6	84732496	84764236	-	6058	1	TBX18|CAI	0.004	0
NM_0178:	chr16	55509001	55586670	+	5372	1	LPCAT2|A(	0.283	0.209
NR_02671	chr10	96750266	96750923	+	657	1	RPL13AP5	73.77	63.169
NM_0045‹	chr7	1.41E+08	1.41E+08	+	489	1	NDUFB2|A	159.099	135.514
NR_03165	chr22	45200954	45201019	-	65	1	MIR1249|I	1.19	0.421
NR_10810	chr1	58785151	58899712	+	819	1	LINC01135	0.094	0.067
NM_0012	chr10	1.04E+08	1.04E+08	-	6493	1	ITPRIP|DA	6.072	4.687
NM_0307	chr20	59019254	59026654	+	3490	1	TUBB1|-|-	0.964	0.483
NM_0146(	chr17	74987632	75005797	+	3526	1	CDR2L|HU	14.129	11.246
NM_0142{	chrX	1.11E+08	1.11E+08	-	3580	1	CAPN6|CA	0.223	0.677
NR_03897	chr20	11266659	11273383	-	2272	1	LOC33959	0	0.006
NR_11011	chr7	1.01E+08	1.01E+08	+	1095	1	LOC10192	2.108	1.226
NM_0031	chr2	72887383	72892160	+	1454	1	SPR|SDR3	0.142	1.366
NM_0794	chr19	10566462	10568979	-	1140	1	CDKN2D|I	106.202	91.578
NM_0024	chr11	62270155	62273157	+	501	1	SCGB2A2|	0	0
NM_0011!	chr20	1978756	1993748	-	2467	1	PDYN|AD(	0.214	0.922
NM_0582‹	chr2	1.35E+08	1.35E+08	+	6912	1	CCNT2|CY	5.531	6.983
NR_13804	chrX	1.56E+08	1.56E+08	-	1229	2	WASIR1|N	0	0
NM_0028	chr12	1.22E+08	1.22E+08	+	2344	1	PSMD9|Rp	20.017	18.5
NM_0045!	chr3	1.86E+08	1.86E+08	-	4277	1	TRA2B|Htı	39.38	40.079
NM_0011:	chr19	55452979	55461682	-	904	1	ISOC2|-|-|	26.7	21.864
NM_0010(	chr22	31122923	31134697	+	2272	1	INPP5J|INI	4.807	8.591
NM_0012‹	chr2	41935368	41953803	-	3715	1	C2orf91|-|	0.031	0.037
NM_0227‹	chr1	2.21E+08	2.21E+08	+	2234	1	MARC1|M	3.192	4.157
NR_13547	chr17	35568114	35574313	+	823	1	LINC02001	16.356	19.089
NR_04058	chr7	72997295	73005943	-	2158	1	STAG3L3|!	0.03	0.076
NM_0013(	chr3	3126916	3149022	+	2205	1	TRNT1|CC	2.503	2.497
NM_0010(	chr11	7927718	7928662	-	943	2	OR10A6|C	0	0
NM_0045(	chr14	67589306	67600300	-	1432	1	PIGH|GPI-	11.723	11.048
NM_1785:	chr5	64165843	64217786	+	1721	1	RNF180|R	1.086	1.624
NR_03650	chr15	49156298	49158625	+	2327	1	NDUFAF4F	0.028	0.006
NM_0026	chr22	41576886	41589867	-	1273	1	PMM1|Se	54.866	42.651
NM_0018(	chr19	35648223	35658784	+	584	1	COX6B1|C	322.403	257.945
NM_0005	chr1	2.07E+08	2.07E+08	-	1622	1	IL10|CSIF|	0	0
NM_0062:	chr19	36113709	36115304	-	864	1	POLR2I|RF	60.99	46.988
NM_1994	chr3	1.21E+08	1.22E+08	-	8748	1	POLQ|PRC	0.187	0.343
NR_02984	chr11	1.12E+08	1.12E+08	+	76	1	MIR34C|M	0	0
NM_0206{	chr10	1.03E+08	1.03E+08	+	2453	1	AS3MT|CY	3.958	4.651
NR_10678	chr1	12029158	12029222	+	64	1	MIR6729|I	2.82	3.21
NM_0010{	chr19	54630411	54637530	+	2460	5	LILRB1|CD	0	0
NM_0057{	chr17	81887835	81891586	-	1089	1	ALYREF|AL	51.515	37.632
NR_13091	chr5	87215735	87239201	-	2895	1	LINC0194	0	0.009
NR_03027	chr1	1E+08	1E+08	+	67	1	MIR553|M	0	0
NR_13650	chr18	76491652	76493915	+	1195	1	LOC10192	0.896	1.089
NM_1450{	chr16	27224994	27268792	-	1052	1	NSMCE1|N	26.498	48.148
NR_13275	chr10	3134104	3134298	+	194	1	SNORD142	0	0.282

NM_0151[illegible]	chr3	46979683	47009704	+	8772	1	NBEAL2|B	1.943	2.861
NM_0052[illegible]	chr7	97005978	97011040	+	1870	1	DLX6|-|-|[illegible]	0.014	2.095
NM_2074[illegible]	chr6	35776594	35779552	+	425	1	CLPSL2|AA	0.061	0.032
NM_0011[illegible]	chr9	1.37E+08	1.37E+08	+	3116	1	RABL6|C9[illegible]	44.473	39.869
NM_0010[illegible]	chr14	21032820	21034785	-	1603	1	RNASE13|	0.016	0.043
NM_0012[illegible]	chr4	94451857	94668227	+	3817	1	PDLIM5|EI	5.158	2.612
NM_0010[illegible]	chr10	78033760	78040715	+	626	1	RPS24|DB[illegible]	924.994	798.54
NR_13123	chrX	1.2E+08	1.2E+08	+	2098	1	RHOXF1-A[illegible]	0.049	0.039
NM_0012[illegible]	chr1	2.03E+08	2.03E+08	-	2036	1	ADIPOR1|[illegible]	47.382	52.487
NR_12642	chr13	1.13E+08	1.13E+08	-	452	1	F10-AS1|-	0.057	0.03
NM_0013[illegible]	chr13	1.14E+08	1.14E+08	+	2211	1	CDC16|AN	33.881	32.877
NM_0061[illegible]	chr4	84493283	84498234	-	1111	1	NKX6-1|NI	0.801	1.541
NM_1523[illegible]	chr19	11767000	11784078	+	4442	1	ZNF441|-|	1.17	1.582
NM_0066[illegible]	chr19	29606263	29617255	+	2585	1	POP4|RPP	15.208	14.624
NM_0161[illegible]	chr8	30063012	30083208	-	2147	1	SARAF|FO[illegible]	283.813	278.144
NR_03780	chr11	1.17E+08	1.17E+08	+	824	1	BACE1-AS|	6.165	6.566
NM_0006[illegible]	chr21	33266360	33297234	+	1952	1	IL10RB|CD	7.12	7.655
NM_0330[illegible]	chr7	1.51E+08	1.51E+08	-	1437	1	FASTK|FAS	31.35	26.946
NR_12592	chr4	1.08E+08	1.08E+08	-	2803	1	LOC10192[illegible]	0.207	0.269
NM_0012[illegible]	chr19	54353624	54370556	-	2736.2	5	LAIR1|CD3	0	0
NM_1981[illegible]	chr3	69054730	69080373	-	2058	1	UBA3|NAE	17.636	24.08
NM_0036[illegible]	chr1	2.42E+08	2.42E+08	+	3176	1	EXO1|HEX	1.746	1.471
NR_03788	chr20	63657810	63698698	+	5734	1	RTEL1-TNF	3.904	3.64
NM_0150[illegible]	chr21	43659551	43696079	+	5069	1	RRP1B|KIA	15.017	16.413
NM_0185[illegible]	chr4	1.12E+08	1.12E+08	+	5952	1	AP1AR|2C	3.145	3.882
NM_0160[illegible]	chr7	66987703	66995601	-	1598	1	SBDS|CGI-	25.553	21.643
NM_0013[illegible]	chr17	31937010	32001045	+	4413	1	SUZ12|CH	3.24	5.397
NM_0201[illegible]	chr3	1.59E+08	1.59E+08	-	1108	1	LXN|ECI|T	0.454	0.297
NR_13824	chr11	2295640	2303049	-	2727	1	C11orf21|	0.028	0.04
NR_10675	chr3	32506283	32506389	-	106	1	MIR548AY	0	0
NM_0012[illegible]	chr12	190077	214205	-	3160	1	SLC6A12|[illegible]	0.208	0.186
NR_10684	chr19	6736712	6736778	-	66	1	MIR6791|[illegible]	4.298	5.811
NM_0053[illegible]	chr19	35358586	35360487	+	1668	1	FFAR3|FFA	0.077	0
NM_0033[illegible]	chr8	1.09E+08	1.09E+08	+	1410	1	TRHR|TRH	0.101	0.039
NR_03989	chr17	81400716	81400778	-	62	1	MIR4740|[illegible]	0.832	0.663
NM_0067[illegible]	chr6	1.43E+08	1.43E+08	-	9712	1	HIVEP2|HI	3.287	6.067
NM_1986[illegible]	chr21	44579455	44580604	-	1147	1	KRTAP10-[illegible]	0	0
NM_0036[illegible]	chr4	8592660	8619761	+	2232	1	CPZ|-|-|4[illegible]	0.352	0.178
NM_0010[illegible]	chr1	1.11E+08	1.11E+08	-	1802	1	LRIF1|C1o	4.45	5.8
NM_0012[illegible]	chr16	88863340	88866660	-	1211	1	PABPN1L|	0.213	0.17
NM_0160[illegible]	chr12	1.02E+08	1.02E+08	-	1006	1	WASHC3|[illegible]	32.9	31.873
NM_0029[illegible]	chr17	36088256	36090160	-	787.5	4	CCL3|G0S[illegible]	0	0
NM_0011[illegible]	chr11	1.26E+08	1.26E+08	-	2993	1	SRPRA|DP	31.809	24.639
NM_0011[illegible]	chr19	405443	409170	-	3120	1	C2CD4C|F[illegible]	12.98	10.729
NM_0011[illegible]	chr19	2841435	2860474	+	8538	1	ZNF555|-|	2.51	3.618
NM_0011[illegible]	chr14	24310151	24312053	+	1556	1	LTB4R2|BI	1.608	1.769
NM_0013[illegible]	chr3	53161521	53192717	+	2647	1	PRKCD|AL	1.919	4.078
NM_0010[illegible]	chr20	43726161	43727002	-	763	1	GTSF1L|C[illegible]	0	0
NR_00258	chr2	2.06E+08	2.06E+08	+	69	1	SNORD51|	0	0.397
NM_0012[illegible]	chr4	1.76E+08	1.76E+08	-	3100	1	GPM6A|G	154.543	286.762

NM_0011(	chr12	57460135	57472264	+	3386	1	GLI1|GLI|-	0.103	0.231
NM_0010:	chr10	15211645	15371059	-	3940	1	FAM171A1	37.46	40.298
NR_03383	chr9	96065839	96101912	+	2413	1	LOC15843.	0.011	0.079
NM_1308‹	chr16	80540952	80550809	+	661	1	DYNLRB2|	1.131	0.746
NR_13456	chr7	1.32E+08	1.32E+08	+	463	1	LOC10192.	0	0
NM_1523:	chr16	29778240	29781648	+	1801	1	ZG16|JCLN	0	0
NR_00155	chrY	7699357	7701247	-	190	1	TTTY16|N(	0	0
NM_0184:	chr18	24426645	24453530	+	3745	1	IMPACT|R	6.225	5.57
NM_0328(	chr1	18107746	18378483	+	1932	1	IGSF21|-|-	3.243	7.813
NR_03616	chr22	17764180	17764259	-	79	1	MIR3198-1	0	0
NM_0010(	chr19	10718053	10831910	+	3632	1	DNM2|CM	32.635	40.359
NM_0064:	chr12	4615508	4649047	-	3311	1	AKAP3|AK	1.916	1.986
NM_0064‹	chr6	43225629	43229473	-	640	1	DNPH1|C6	12.732	9.802
NR_10975	chr9	34985411	34989379	-	746	1	DNAJB5-AS	0.156	0.257
NM_0059(	chr6	1.19E+08	1.19E+08	-	5012	1	MAN1A1|I	1.772	0.85
NM_0012(	chr5	56511394	56606232	-	930	1	C5orf67|-|	0.042	0.015
NR_02994	chrX	1.35E+08	1.35E+08	-	97	1	MIR424|M	0	0
NM_0806:	chr20	32443059	32483582	-	6092	1	NOL4L|C2(	33.394	30.465
NM_1530(	chr18	10454628	10488701	+	2555	1	APCDD1|B	53.431	15.15
NM_0013(	chr3	1.87E+08	1.87E+08	+	1425	1	FETUB|16(	0	0.048
NM_0012(	chr22	40857081	40862781	+	1440	1	XPNPEP3|.	2.955	2.045
NM_0011(	chr11	67364168	67374177	-	1756	1	CLCF1|BSF	0.933	1.568
NM_0008:	chr5	1.61E+08	1.62E+08	-	7283	1	GABRB2|-	0.432	0.809
NM_2073:	chr3	1.28E+08	1.28E+08	+	5261	1	KBTBD12|	0.012	0.008
NM_0011‹	chr3	39183215	39192596	-	6030	1	XIRP1|CM	0.006	0.014
NM_0011(	chr8	30131671	30144684	-	1658	1	MBOAT4|I	0.218	0.256
NM_0069(	chr21	26836287	26845409	-	4640	1	ADAMTS1	0.22	0.484
NR_03665	chr9	1.12E+08	1.12E+08	+	3199	1	HSDL2|C9(	6.743	5.733
NR_02403	chr16	31700613	31707424	+	1767	1	CLUHP3|C	3.991	1.868
NM_0011‹	chr8	1.07E+08	1.07E+08	-	4353	1	ANGPT1|A	0.424	0.497
NR_10697	chr10	67403351	67403410	-	59	1	MIR7151|I	0	0
NM_0011(	chr7	90245174	90311063	+	3823	1	CFAP69|C:	0.145	0.143
NM_0004(	chr11	1.17E+08	1.17E+08	-	1455	1	APOA4|-|-	0	0
NM_0010:	chr3	1.26E+08	1.26E+08	-	802	1	ALG1L|AL(	1.013	0.512
NR_02734	chr13	91347820	91354575	+	923	1	MIR17HG|	0.224	0.445
NM_0163:	chr3	52903567	53046073	-	4535	1	SFMBT1|R	3.77	5.672
NM_0026(	chr2	2.32E+08	2.32E+08	-	1186	1	PDE6D|JB	62.25	76.823
NM_2070(	chr17	36103673	36105621	+	816.7	6	CCL4L1|AT	0	0
NM_0010‹	chr1	1.58E+08	1.58E+08	+	1908	1	CD1E|CD1	0	0
NM_0010‹	chr5	65624731	65666127	+	3068	1	TRAPPC13	4.308	6.139
NR_04611	chr5	1.75E+08	1.75E+08	-	2140	1	LINC01951	0	0
NM_0021:	chr6	12012491	12164999	+	8863	1	HIVEP1|CI	3.039	2.944
NM_0010:	chrX	55075056	55078903	+	501	1	PAGE2B|C	0	0.219
NM_0247:	chr15	77108156	77420104	-	11807	1	PEAK1|SG	2.121	2.879
NM_0011‹	chr11	33858576	33869963	-	1590	1	LMO2|RB1	30.661	9.157
NM_0013:	chr2	61065870	61138034	+	2979	1	KIAA1841|	4.982	3.223
NM_0224(	chr5	43444252	43483890	-	2756	1	TMEM267	1.156	1.585
NM_0050(	chr6	1E+08	1E+08	-	3982	1	SIM1|bHLI	0	0
NR_03883	chr2	21687434	21710652	-	2124	1	LINC01822	0	0
NM_1758‹	chr20	4148779	4187747	+	2064	1	SMOX|C2(	11.557	14.765

NR_10684	chr17	44207771	44207839	-	68	1	MIR6782|I	0	1.611
NM_0012	chr6	29942470	29945884	+	1600.2	8	HLA-A|HLA	3.279	4.134
NM_0012	chr11	64305528	64316740	+	2255	1	ESRRA|ERI	9.137	8.528
NM_0206	chr16	87602887	87698155	+	3995	1	JPH3|CAGI	12.316	26.067
NR_10418	chr3	1.95E+08	1.95E+08	+	427	1	XXYLT1-AS	0	0
NR_03041	chr3	1.84E+08	1.84E+08	+	84	1	MIR1224|I	1.381	1.304
NM_0013	chr2	2.18E+08	2.18E+08	-	9972	1	TNS1|MST	0.817	3.204
NR_00159	chr22	16601911	16648830	+	1421	1	TPTEP1|ps	0	0
NR_12040	chr11	89488853	89491565	-	610	1	NOX4|KOX	0.296	0.045
NM_1527	chr13	21492689	21604216	-	1919	1	MICU2|11	10.011	9.065
NM_0038	chr3	67374717	67654614	-	2341	1	SUCLG2|G	0.788	1.188
NM_0007	chr4	26481396	26490420	-	1675	1	CCKAR|CC	0.062	0.008
NM_0227	chr22	46684410	46738255	-	4430	1	CERK|LK4|	51.46	56.173
NM_0012	chr6	43076814	43161720	+	4124	1	PTK7|CCK-	22.163	32.037
NR_04025	chr3	1.5E+08	1.5E+08	+	1695	1	WWTR1-A	0.183	0.137
NM_0025	chr9	1.16E+08	1.16E+08	+	10946	1	PAPPA|AS	0.364	0.444
NM_1815	chr13	37000541	37009616	+	1400	1	EXOSC8|C	16.338	16.358
NM_0012	chr20	63698642	63707853	-	2447	1	ARFRP1|A	15.586	14.682
NR_02412	chr17	752333	755336	+	3003	1	DBIL5P|EL	0.412	0.588
NM_1532	chr10	11823398	11872277	+	3327	1	PROSER2|	0.267	0.823
NR_02775	chr16	65284499	65576300	-	1751	1	LINC00922	0.015	0.102
NM_0160	chr1	1.5E+08	1.5E+08	+	873	1	BOLA1|CG	4.475	10.434
NM_0052	chr11	22674773	22813001	+	2015	1	GAS2|-|-|	0.48	0.455
NM_0055	chr5	67182276	67196789	-	2710	1	CD180|LY6	0	0.01
NR_02693	chrX	1.39E+08	1.39E+08	-	2907	1	LINC0088	0.931	0.74
NR_04620	chr19	32390050	32405539	-	948	1	LOC40068	0.53	0.419
NR_02400	chr2	1.14E+08	1.14E+08	-	1658	1	DDX11L2|	0.98	0.529
NM_0046	chr17	65528565	65561622	-	4219	1	AXIN2|AXI	22.737	11.337
NM_0013	chr8	1.25E+08	1.25E+08	-	3825	1	WASHC5|I	9.307	9.55
NM_0059	chr12	1.15E+08	1.15E+08	-	4714	1	TBX3|TBX3	0.011	0.02
NR_03397	chr3	64078361	64087363	-	2125	1	LINC00994	0.012	0.039
NR_11057	chr2	10878267	10885118	+	461	1	LINC01954	0	0.03
NM_0582	chr8	1.23E+08	1.24E+08	-	6782	1	FBXO32|FI	1.234	10.696
NM_0035	chr12	92772509	92929331	-	7972	1	EEA1|MST	0.532	0.696
NM_0004	chr8	1.43E+08	1.43E+08	-	3524	1	CYP11B1|	0	0
NM_0026	chr6_GL00	4859723	4865178	+	2173	2	PHF1|MTF	2.35	1.922
NR_03030	chr5	59703606	59703703	-	97	1	MIR582|M	0	0
NR_07341	chr15	58690473	58693607	-	3134	1	HSP90AB4	0.082	0.114
NR_03172	chr13	40727828	40727875	-	47	1	MIR320D1	0	0
NM_2034	chrX	51406915	51408837	+	1920	1	CXorf67|-|	0	0.05
NM_0144	chr9	1E+08	1E+08	+	4936	1	INVS|INV|	3.417	3.044
NM_0012	chr4	25377226	25418498	+	2644	1	ANAPC4|A	4.686	5.512
NR_04642	chr19	35667948	35673291	-	793	1	UPK1A-AS	0.146	0.017
NM_0011	chr8	22995848	23020197	+	4945	1	RHOBTB2|	32.368	31.131
NR_00311	chr3	1.29E+08	1.29E+08	-	2075	1	RPL32P3|-	4.872	6.06
NR_14443	chr6	1.59E+08	1.59E+08	-	6218	1	RSPH3|CIL	1.769	1.28
NM_0012	chr19	22286450	22317176	+	3830	1	ZNF729|-|	0	0.011
NM_0010	chr14	74853033	74863834	-	3193	1	PROX2|PR	0.267	0.541
NM_0013	chr15	75226753	75229690	-	713	1	LOC10537	0	0.038
NM_0059	chr16	56682470	56684196	+	399	1	MT1X|MT	2.003	2.884

NM_0178[illegible]	chr12	47773184	47782753	+	3029	1	SLC48A1|[illegible]	8.854	13.141
NM_2069[illegible]	chr9	70783862	70869058	-	1278	1	TRPM3|G[illegible]	2.401	1.05
NR_04701	chr13	30803206	30810645	+	904	1	LINC0039[illegible]	0	0
NR_00331	chr15	25206262	25232468	+	80.3	3	SNORD11[illegible]	0	0
NM_1721[illegible]	chr10	73812501	73874591	-	3640	1	CAMK2G|[illegible]	20.088	37.569
NM_0046[illegible]	chrY	24045230	24048014	-	2361	2	CDY1|CDY	0	0
NM_0252[illegible]	chr19	40530488	40560752	+	2407	1	SPTBN4|Q	4.376	7.17
NM_0012[illegible]	chr12	1.23E+08	1.23E+08	-	1111	1	CDK2AP1|	62.959	74.76
NM_0010[illegible]	chr1	1.55E+08	1.55E+08	-	1302	1	RUSC1-AS[illegible]	28.906	20.135
NM_0013[illegible]	chr4	1.28E+08	1.28E+08	+	5546	1	ABHD18|C	1.083	1.561
NM_1775[illegible]	chr7	1.3E+08	1.31E+08	+	2367	1	MEST|PEG	40.538	46.802
NM_2012[illegible]	chr6	8013567	8064414	-	2677	1	BLOC1S5|[illegible]	4.075	3.802
NR_12157	chr9	1.37E+08	1.37E+08	+	543	1	NALT1|LIN	0.237	0.151
NM_0012[illegible]	chr3	1.86E+08	1.86E+08	-	4064	1	IGF2BP2|I[illegible]	13.706	8.045
NR_03822	chr10	84279980	84294659	+	2679	1	LINC0085[illegible]	0	0.031
NR_10226	chr3	1.61E+08	1.61E+08	+	2841	1	LINC0206[illegible]	0.018	0.024
NM_1525[illegible]	chr8	25458931	25507917	+	3777	1	CDCA2|PP	0.645	0.678
NR_11099	chr9	61205260	61212373	+	1941	1	FAM74A4|	0	0
NM_0013[illegible]	chr21	7048859	7087230	+	2348	4	LOC10272[illegible]	0	0
NM_0160[illegible]	chrX	1.3E+08	1.3E+08	-	4606	1	ZDHHC9|C	34.896	35.429
NM_0320[illegible]	chr1	2.31E+08	2.31E+08	+	3577	1	SPRTN|C1[illegible]	2.967	2.941
NM_0010[illegible]	chr17	32927910	32941649	+	1710	1	TMEM98|[illegible]	39.985	46.281
NM_0011[illegible]	chr9	1.32E+08	1.32E+08	+	2801	1	POMT1|L[illegible]	31.348	31.458
NM_0020[illegible]	chr13	21671076	21704501	+	4525	1	FGF9|FGF-	1.123	1.205
NM_0030[illegible]	chr15	32641613	32697098	+	1290	2	SCG5|7B2	0	0
NM_0246[illegible]	chr19	58126328	58150781	-	3407	1	ZNF329|-|	12.897	17.585
NM_0005[illegible]	chr4	1.42E+08	1.42E+08	+	1992	1	IL15|IL-15	0.11	0.028
NM_1533[illegible]	chr17	7041630	7043923	-	1797	1	SLC16A11|	0.165	0.16
NM_0158[illegible]	chr3	50341106	50345746	-	1780	1	ZMYND10	6.541	2.924
NM_0011[illegible]	chr3	1.12E+08	1.12E+08	-	3334	1	GCSAM|G[illegible]	0.062	0.029
NM_0013[illegible]	chrX	51332809	51337525	+	1934	1	NUDT10|[illegible]	11.187	11.268
NM_0014[illegible]	chr17	7705202	7711375	+	3212	1	EFNB3|EF[illegible]	114.175	86.583
NM_0011[illegible]	chr10	87504466	87553461	+	2660	1	MINPP1|H	7.024	6.689
NM_0013[illegible]	chr11	1157953	1201141	+	17400	1	MUC5AC|[illegible]	0	0
NM_0013[illegible]	chr12	93378550	93403248	+	4450	1	NUDT4|DI	1.078	2.179
NR_02685	chr9	81913437	81919927	+	4860	1	SPATA31D	0.005	0
NM_0191[illegible]	chr6_GL0[illegible]	3479477	3547707	-	10852.9	7	TNXB|EDS	0	0
NM_0010[illegible]	chr2	10122736	10131419	+	3254	1	RRM2|R2|	9.212	4.622
NM_0204[illegible]	chr20	51782717	51802523	-	4819	1	SALL4|DR[illegible]	2.004	2.228
NR_13464	chr4	25531040	25569346	-	574	1	LOC10192[illegible]	0	0
NM_0048[illegible]	chr19	38319844	38329009	+	2650	1	KCNK6|K2	0.38	0.3
NM_0013[illegible]	chr1	17580553	17697875	+	4218	1	ARHGEF10	17.965	15.145
NM_0012[illegible]	chr20	17941596	17968991	-	2202	1	SNX5|-|-|[illegible]	8.08	10.375
NM_0152[illegible]	chr7	11370435	11832198	-	10556	1	THSD7A|-|	19.966	20.23
NM_1390[illegible]	chr11	1.3E+08	1.3E+08	+	5667	1	ADAMTS1[illegible]	1.194	1.871
NM_0012[illegible]	chr19	41796590	41811553	+	1051	1	CEACAM3	0	0
NR_03668	chr8	6987178	6989721	-	493	2	DEFT1P2|-	0	0
NM_0177[illegible]	chr17	74776483	74839783	+	4737	1	TMEM104	6.021	6.035
NM_1722[illegible]	chr17	3860323	3890743	-	3482	1	CAMKK1|[illegible]	10.109	11.238
NM_0054[illegible]	chr9	98288082	98709197	-	5767	1	GABBR2|G	2.609	7.515

NR_11084	chr6	49815493	49824766	-	1733	1	LOC10192	0	0
NM_0183	chr14	45203190	45253402	-	4758	1	MIS18BP1	0.266	0.797
NR_00279	chr13	52488993	52587090	+	2589	1	TPTE2P3|T	0.055	0.048
NR_02677	chr14	1.02E+08	1.02E+08	+	638	1	LINC0023	0.02	0.15
NR_02745	chr14	1.06E+08	1.06E+08	+	1749	2	LINC00221	0	0
NM_0010	chrX	73077338	73079512	-	2172	1	PABPC1L2	0.445	0.435
NM_0247	chr8	76681280	76867285	+	13945	1	ZFHX4|ZF	12.277	5.178
NM_1735	chr9	15553007	15974039	+	6563	1	CCDC171|	0.45	1.177
NR_02938	chr10	1.01E+08	1.01E+08	+	1320	1	LBX1-AS1|	0.02	4.784
NR_03764	chr1	54641740	54742655	+	6318	1	MROH7-T	5.722	6.679
NR_04548	chr1	85276358	85278088	+	886	1	LOC64662	1.63	1.685
NR_03840	chr2	94755326	94761184	-	2395	1	FAM95A|-	0	0
NM_0013	chr19	45710629	45723496	-	2965	1	FBXO46|2	7.41	7.821
NM_0012	chr17	62481113	62615480	+	5063	1	TLK2|HsH	4.711	4.583
NM_0132	chr15	51681353	51721026	+	3296	1	SCG3|SGI	32.445	30.586
NM_0010	chr15	41660412	41769943	+	11390	1	MGA|MAD	3.998	5.522
NM_0013	chr15	88635637	88656344	+	1441	1	ISG20|CD	0.17	0.143
NR_12871	chr21	8205315	8205406	+	91	7	MIR6724-	0	0
NM_0012	chr18	5392381	5628991	-	3888	1	EPB41L3|	26.931	38.586
NM_0010	chr2	1.08E+08	1.08E+08	+	2785	1	SULT1C2|	0.106	0.01
NM_0145	chrX	1.36E+08	1.37E+08	+	2761	1	HTATSF1|	52.209	36.036
NM_0224	chr10	1.12E+08	1.12E+08	-	2212	1	ZDHHC6|D	12.316	11.703
NM_0054	chr12	48978453	48982613	+	2278	1	WNT1|BM	1.783	0.084
NM_0010	chr19	53632056	53637009	+	643	1	DPRX|-|-|	0	0
NR_03989	chr17	64500774	64500839	-	65	1	MIR3064|	51.376	73.542
NR_12542	chr8	1.34E+08	1.34E+08	+	578	1	LOC10192	0	0
NM_0013	chr17	18780995	18801429	+	800	1	TVP23B|C	3.852	3.202
NM_0013	chr10	1.02E+08	1.02E+08	-	3181	1	LDB1|CLIN	108.129	120.194
NM_0155	chr17	40922696	40937643	-	2199	2	KRT23|CK	0	0
NM_1388	chr2	95592018	95599771	+	1666	1	TRIM43|T	0	0.049
NM_0330	chr1	53459399	53467487	+	1903	1	DMRTB1|-	0	0.022
NM_0314	chr16	84122139	84145195	-	3660	1	HSDL1|SD	46.399	60.293
NM_0331	chr2	55634063	55693910	-	4583	1	PNPT1|CO	1.919	2.717
NM_0320	chr3	1.83E+08	1.83E+08	+	4119	1	B3GNT5|B	3.691	2.404
NR_03961	chr1	51059837	51059905	+	68	1	MIR4421|	0	0
NM_0013	chr1	2.23E+08	2.23E+08	+	4899	1	MIA3|ARN	5.68	6.305
NM_0124	chr2	74458400	74460891	+	1217	1	WBP1|WB	83.696	67.157
NM_0047	chr19	32581188	32587452	+	596	1	PDCD5|TF	31.368	30.979
NM_0245	chr1	1.57E+08	1.57E+08	-	878	1	MRPL24|L	41.015	38.283
NM_0165	chr5	75027464	75030899	-	3433	1	GCNT4|C2	0.053	0.299
NR_13486	chr15	72345204	72376473	-	2001	1	HEXA|TSD	24.788	20.371
NM_0013	chr14	21669912	21671017	-	1103	1	OR4E1|OR	0	0
NR_03962	chr1	1.79E+08	1.79E+08	+	85	1	MIR4424|	0	0
NM_0011	chr4	67910301	67963514	-	2407	1	TMPRSS11	0	0
NM_0012	chr7	1.01E+08	1.02E+08	+	3018	1	COL26A1|	14.863	20.491
NR_03342	chr1	1.02E+08	1.02E+08	+	1779	1	DNAJA1P5	0.029	0.015
NR_04980	chr13	55174454	55174548	+	94	1	MIR5007|	0	0
NM_0045	chr1	1.54E+08	1.54E+08	-	1871	1	ILF2|NF45	195.538	237.615
NR_11028	chr2	77915934	77918008	+	631	1	LINC01851	0	0
NR_13583	chr2	54972191	55049190	-	1636	1	RTN4|ASY	112.731	162.405

NM_0142	chr11	61241176	61251443	+	1369	1	PGA5|Pg5	0.057	0.08
NM_0123	chr7	1.21E+08	1.21E+08	-	2554	1	TSPAN12|	1.802	2.145
NM_0153	chr15_KI2	1394881	1854904	-	4695	2	FAM189A1	0.434	0.971
NM_0010	chr9	1.05E+08	1.05E+08	+	958	1	OR13F1|O	0	0
NM_0010	chr11	5947347	5948294	+	946	1	OR56A3|C	0	0
NR_03842	chr1	2.05E+08	2.05E+08	+	2777	1	LEMD1-AS	0.098	0.252
NM_1471	chr10	1.26E+08	1.26E+08	-	1908	1	MMP21|H	0.068	0.029
NR_10464	chr12	1.27E+08	1.27E+08	-	1329	1	LOC10192	0	0
NM_0010	chrX	85092286	85108968	-	2146	1	SATL1|-|-|	1.154	0.945
NM_0010	chr11	75422394	75430629	-	1931	1	KLHL35|-|	25.166	27.954
NR_00333	chr15	25094036	25094129	+	93	1	SNORD116	0	1.473
NR_13297	chr9	1.38E+08	1.38E+08	-	1915	1	LOC10537	0	0
NM_1825	chrX	1.04E+08	1.04E+08	+	727	1	TMEM31|	1.1	0.848
NM_0039	chr13	77895481	77918831	-	2521	1	EDNRB|AE	5.989	3.477
NR_12651	chr4	5524569	5527800	+	777	1	LINC01587	1.095	0.317
NM_0011	chr19	11089362	11133829	+	4761	1	LDLR|FH|F	22.234	18.058
NR_02678	chr12	8180209	8201000	+	3258	1	FAM66C|-	0.443	1.127
NM_0010	chr19	9093245	9094186	+	940	1	OR1M1|O	0	0
NR_00359	chrY	6406244	6428444	-	1625	2	TTTY2B|-|	0	0
NR_13670	chrX	14865193	14873069	-	590	1	FANCB|FA	0.131	0.534
NM_1720	chr1	1.82E+08	1.82E+08	-	2497	1	TEDDM1|	0.01	0.099
NR_11059	chr2	1.66E+08	1.66E+08	+	736	1	LOC10272	0.245	1.005
NR_03996	chr17	64501214	64501313	-	99	1	MIR5047|	58.086	87.855
NM_1818	chr19	43827321	43848898	+	2556	1	ZNF283|H	1.286	1.597
NR_02426	chr12	1E+08	1E+08	-	2430	1	GOLGA2P5	0.44	0.637
NM_0013	chr10	1E+08	1E+08	-	3074	1	ERLIN1|C1	5.641	6.519
NM_0012	chr6_GL0	2611478	2614855	-	1568.4	7	HLA-C|D6	5.138	5.633
NM_1706	chr5	74721204	74767371	-	3086	1	GFM2|EF-	12.004	12.188
NM_0325	chr19	917342	921015	+	1618	1	KISS1R|AX	3.108	1.507
NM_0541	chr3	98087173	98088102	+	928	1	OR5AC2|H	0	0
NR_03412	chr20	50292720	50314919	+	2273	1	LINC0127	0	0.096
NM_2073	chr11	60867542	60875691	+	1958	1	ZP1|HEL16	0.066	0.112
NM_0149	chr19	32345608	32387667	+	7616	1	ZNF507|-|	2.528	3.19
NM_0122	chr5	1.77E+08	1.77E+08	+	4318	1	ZNF346|JA	9.896	11.248
NR_10699	chr16	24203116	24203231	+	115	1	MIR1273H	0	0
NR_10999	chr3	67654697	67747110	+	959	1	SUCLG2-A	0.067	0.129
NR_13568	chr15	66931537	66956515	-	713	1	LOC10272	0	0
NM_0196	chr17	82614562	82648535	-	2575	1	WDR45B|	56.561	61.756
NM_0073	chr14	52004802	52069228	-	5022	1	NID2|NID-	0.634	0.502
NM_0146	chr19	47713344	47743134	+	3576	1	EHD2|PAS	0.916	2.11
NM_1992	chr17	81666731	81673906	+	2100	1	CCDC137|	14.797	12.686
NR_04563	chr1	1.63E+08	1.63E+08	-	1481	1	RGS5|MST	0.235	0.518
NM_0067	chr17	41436369	41441344	-	2828	1	KRT38|HA	0	0
NM_0061	chr1	1.63E+08	1.63E+08	+	3144	1	DDR2|MIG	0.205	0.719
NM_1533	chr6	1.11E+08	1.11E+08	+	3912	1	MFSD4B|K	1.269	1.099
NR_04499	chr22	44606328	44625419	-	821	1	LINC0022	0.283	0
NM_1759	chr16	29742465	29746019	-	2593	1	C16orf54|	0.025	0.005
NM_0010	chr11	54603069	54603998	-	928	1	OR4C46|-|	0	0
NM_1749	chr7	44756188	44769880	+	4985	1	ZMIZ2|NE	39.423	37.777
NM_0011	chr19	55044324	55063217	-	1922	10	RDH13|SD	0	0

NR_02969	chr21	16590237	16590325	+	88	1	MIR125B2	0	0
NM_1447	chr5	1.59E+08	1.59E+08	-	3351	1	RNF145|-|	32.182	34.518
NM_0013	chr12	1.04E+08	1.04E+08	-	2113	1	GLT8D2|-|	1.202	1.595
NM_0013	chr1	35720218	35769950	-	4725	1	CLSPN|-|-	1.092	0.951
NR_07315	chr20	31377163	31390649	-	461	1	DEFB119|	0	0
NM_0046	chr12	80792521	80937915	-	6093	1	LIN7A|LIN	9.73	5.861
NM_0010	chr14	23042167	23044060	+	1891	1	PSMB11|E	0	0.007
NM_1450	chr11	58834066	58844524	-	1371	1	GLYATL2|E	0.226	0.609
NM_0010	chr19	54273821	54281178	-	2625.6	5	LILRB2|CD	0	0
NM_0189	chr2	1.83E+08	1.83E+08	+	5893	1	DNAJC10|	6.577	6.759
NM_1943	chrX	1.04E+08	1.04E+08	+	661	2	TMSB15B|	0	0
NR_02666	chr9	3898646	3901248	+	728	1	GLIS3-AS1	0.337	0.809
NM_1524	chr12	1565993	1594165	-	2107	1	FBXL14|Fb	11.101	12.046
NM_1785	chr11	57460537	57477539	+	2215	1	RTN4RL2|	20.769	25.669
NM_0012	chr1	85062298	85133138	+	2878	1	WDR63|D	0.202	0.076
NM_0010	chr2	1.87E+08	1.88E+08	-	1152	1	TFPI|EPI|L	0.403	1.569
NM_0012	chr2	47403067	47483228	+	3006	1	MSH2|CO	12.139	11.164
NR_11094	chr16	921033	934524	+	1320	1	LMF1-AS1	0.127	0.083
NM_0058	chr19	12796820	12801910	-	1030	1	PRDX2|HE	459.534	359.819
NR_02334	chr9	1.34E+08	1.34E+08	-	124	1	RNU6ATA	0.208	0
NM_0155	chr6	1.54E+08	1.54E+08	-	6801	1	IPCEF1|PI	0.091	0.278
NM_0322	chr2	2.27E+08	2.27E+08	+	4847	1	RHBDD1|F	2.123	3.546
NM_0012	chr2	1.62E+08	1.62E+08	-	2706	1	FAP|DPPI	0.014	0.035
NM_0012	chr15	82755365	82806070	-	6383	1	FSD2|SPR	0.232	0.288
NM_0038	chr5	1.42E+08	1.42E+08	-	1928	1	HDAC3|HD	58.93	58.98
NM_0011	chr12	42326145	42441963	+	1431	1	PPHLN1|C	29.697	30.141
NM_0011	chr4	15374551	15446167	+	4460	1	C1QTNF7|	0	0.003
NM_0183	chr17	59155499	59206709	+	6461	1	PRR11|-|-	1.782	2.109
NR_12540	chr3	1.94E+08	1.94E+08	+	1191	1	LINC02037	0	0
NM_0221	chrX	1190496	1212723	-	1596	2	CRLF2|CRL	0	0
NR_12596	chr1	1.12E+08	1.12E+08	-	2764	1	LOC10192	2.967	3.409
NM_0043	chr2	2.41E+08	2.41E+08	-	8871	1	KIF1A|ATS	220.499	238.749
NR_03078	chr21	25573980	25574044	+	64	1	MIR155|M	0	0
NM_0184	chr12	68201349	68225791	-	1040	1	IL26|AK15	0.012	0
NM_0012	chr8	1.15E+08	1.16E+08	-	9506	1	TRPS1|GC	0.768	1.206
NR_02435	chrX	1.35E+08	1.35E+08	+	5238	1	SMIM10L2	8.549	13.085
NM_0010	chr12	32790746	32896846	-	4292	1	PKP2|ARV	0.436	0.89
NM_0010	chr1	1.56E+08	1.56E+08	-	6121	1	MEX3A|M	58.729	48.265
NM_0013	chr8	98189826	98294393	-	4453	1	NIPAL2|N	0.104	0.218
NM_1307	chr19	496490	505343	+	1530	1	MADCAM1	1.551	0.895
NM_1735	chr11	18704305	18726230	-	4309	1	IGSF22|IG	0.335	0.715
NM_0013	chr21	45075051	45226563	+	3286	1	ADARB1|A	7.283	7.119
NM_0045	chr17	42124976	42155044	-	1690	1	RAB5C|L1	115.98	133.445
NR_12161	chr6	1.38E+08	1.38E+08	-	754	1	LOC10050	0.086	0
NR_00319	chr17	16786892	16804505	+	3893	1	USP32P1|	0.003	0.056
NR_12593	chr1	70706453	70786468	+	2079	1	LINC01788	0	0
NM_1471	chr4	5562419	5708567	-	4386	1	EVC2|LBN	0.032	0.041
NR_11937	chr1	83575789	83860996	-	3161	1	LINC01725	0	0.017
NR_00214	chr7	99273301	99297971	+	2251	1	MYH16|M	0.063	0.043
NM_0309	chr1	1.83E+08	1.83E+08	-	2462	1	SHCBP1L|	0	0

NR_02664	chr20	46463660	46465292	-	1632	1	MKRN7P|I	0.577	0.504
NM_0070{	chr22	50767492	50783659	-	2172	1	RABL2B|-|	11.926	9.579
NM_0010:	chr5_GL33	134919	153209	-	1176	2	AK6|AD-0(	0.318	0.128
NM_0011(	chr9	88647179	88652160	-	678	1	LOC28623:	0	0
NM_0010(	chr11	56742828	56743811	-	982	2	OR9G4|OI	0	0
NR_13211	chr1	2.24E+08	2.24E+08	-	2936	1	GTF2IP20|	0.733	0.947
NM_0309⁄	chr5	34017858	34043266	-	3599	1	C1QTNF3|	2.364	1.298
NM_0029⁄	chr7	7636563	7718607	-	1965	1	RPA3|REP/	7.546	5.667
NM_0011ś	chrX	1.07E+08	1.07E+08	+	3598	1	CXorf57|-|	3.186	2.691
NM_0145:	chr19_GL9	450550	478414	+	1070.3	36	KIR2DS1|C	0	0
NM_0012(	chr9	97029677	97039643	-	4314	1	CTSV|CATI	1.004	0.422
NM_1814ś	chr12	1.14E+08	1.14E+08	-	3722	1	TBX5|HOS	0	0.007
NM_2074(	chr20	257736	261095	+	2116	1	DEFB132|I	0	0
NR_00158	chr11	5241955	5243592	-	656	1	HBBP1|HB	0.275	0
NM_0312:	chr11	28020616	28108199	-	3444	1	KIF18A|M!	0.153	0.541
NM_0041:	chr11	18020537	18040788	-	1803	1	TPH1|TPRI	0.064	0.175
NM_1730:	chr2	2.07E+08	2.07E+08	+	1198	1	CPO|-|-|2	0.086	0.217
NM_0219:	chr11	44260728	44310166	-	5460	1	ALX4|CRS!	0.052	0.048
NM_0196(	chr15	52581321	52678634	-	4213	1	FAM214A|	2.681	4.789
NM_0049:	chr17	81117296	81131948	-	5074	1	AATK|AAT	3.026	4.435
NM_0132ś	chr7	23504782	23532041	-	1833	1	TRA2A|AV	42.908	42.025
NM_0011⁄	chrX	37349275	37457295	+	4487	1	PRRG1|PR	1.741	1.902
NM_0011ś	chr20	1309911	1325869	-	1457	1	SDCBP2|SI	1.858	2.566
NM_0327⁄	chr6	11713655	11779047	-	1796	1	ADTRP|AI(	0	0.015
NM_1942ś	chr11	18606401	18634473	-	5726	1	SPTY2D1|!	3.004	2.682
NM_0228:	chr5_KI27(	274862	302936	+	1564	5	SMN2|BCI	0	0
NM_0041(	chr1	1.61E+08	1.61E+08	+	584	1	FCER1G|F(	0.662	0.422
NM_0010:	chr1	11762193	11789585	+	4649	1	C1orf167|	2.382	2.189
NM_0012:	chr12	59596040	59789854	+	11930	1	SLC16A7|I	0.175	0.135
NR_12601	chr21	28116094	28228667	-	4480	1	LINC01695	0	0.003
NR_03163	chr2	2.07E+08	2.07E+08	-	149	1	MIR1302-⁄	0	0
NR_10411	chr6_GL0C	162286	163074	+	408	6	HCG14|dJ:	0	0
NM_0010(	chr11	4914719	4915663	-	943	1	OR51G2|C	0	0
NR_03986	chr16	2274620	2274691	+	71	1	MIR4717|-	0	0
NM_1991ś	chr3	46742091	46744755	-	941	1	PRSS45|TE	0	0.015
NR_10505	chr5	1.48E+08	1.48E+08	-	2095	1	LOC10254(	1.139	1.608
NM_1450!	chr19	54465025	54473290	-	887	4	CDC42EP5	0	0
NM_0050:	chr5	1.15E+08	1.15E+08	-	2716	1	PGGT1B|B	1.348	1.281
NM_0328:	chr19	36009120	36014239	-	968	1	ALKBH6|A	17.382	12.424
NM_0041ś	chr3	1.38E+08	1.38E+08	+	2036	1	SOX14|SO	3.534	2.651
NM_0010(	chr22	45192242	45240769	-	6203	1	KIAA0930|	40.898	45.587
NR_04578	chr2	1.69E+08	1.69E+08	-	2889	1	CERS6-AS1	21.708	26.626
NM_0011ś	chr16	1333605	1349441	+	4537	1	BAIAP3|B/	38.274	26.908
NR_14453	chr11	75813514	75814797	-	681	1	LOC10050(	0.492	0.322
NM_0179!	chr17	50532687	50543750	+	3915	1	EPN3|-|-|:	0.204	0.154
NM_0148ś	chr4	88725954	89057195	-	5856	1	FAM13A|/	3.292	3.37
NM_0010:	chr2	1.19E+08	1.19E+08	+	619	1	DBI|ACBD:	106.147	169.254
NM_0062(	chr10	93612588	93665672	+	2945	1	PDE6C|AC	0.009	0.033
NM_0159⁄	chr7	876554	896434	+	2077	1	GET4|C7oı	33.64	30.592
NM_0010ś	chr1	8318085	8344167	+	2516	1	SLC45A1|I	9.014	10.055

NR_03719	chr19	21483715	21503238	+	3749	1	LINC00664	0.01	0.164
NR_03972	chr14	93948226	93948302	-	76	2	MIR4506|-	0	0
NR_13491	chr16	20623237	20691256	-	1708	1	ACSM1|BU	0.06	0.032
NM_0011	chr16	20796341	20806473	-	3491	1	ERI2|EXOD	1.777	1.817
NM_0042	chr10	71964362	72013564	+	6968	1	CHST3|C6	6.761	8.223
NM_0003	chr16	23302270	23381299	+	2582	1	SCNN1B|B	0.045	0.133
NR_12648	chr15	99014704	99031046	+	476	1	LUNAR1|-	0	0
NM_0011	chr2	55172551	55232563	-	4882	1	CLHC1|C2	0.911	1.147
NM_0012	chr16	88940037	88951524	+	1560	1	LOC10012	0.355	0.527
NR_02148	chr3	1.68E+08	1.69E+08	+	3826	1	EGFEM1P|	1.338	0.44
NR_03606	chr1	1.7E+08	1.7E+08	+	84	1	MIR3119-2	0	0
NM_0529	chr2	1.89E+08	1.89E+08	-	1450	1	DIRC1|-|-|	0	0
NR_13394	chr7	1.24E+08	1.24E+08	+	1736	1	LOC10537	0	0
NM_0228	chr5	1.71E+08	1.71E+08	+	4546	1	RANBP17|	1.069	1.455
NR_13274	chr5	1.6E+08	1.6E+08	+	2316	1	MIR3142H	0.028	0
NR_03317	chr5	1.1E+08	1.1E+08	+	1263	1	LINC01848	0	0.065
NR_12538	chr6	29036021	29076740	+	723	7	LOC10012	0	0
NR_12541	chr8	79768142	79802842	+	617	1	LINC01607	0.522	0.799
NM_0011	chr11	57484531	57514885	-	2346	1	SLC43A1|L	0.984	1.057
NR_11084	chr17	78617384	78632057	-	2256	1	LINC02081	0	0
NM_0039	chr9	1.31E+08	1.31E+08	+	3136	1	FUBP3|FB	16.598	15.16
NR_12582	chr8	96371926	96386648	-	647	1	LOC10272	0	0
NR_02730	chr19	27790493	27793940	-	2048	1	LINC00662	2.178	2.936
NR_03063	chr16	2271747	2271840	+	93	1	MIR940|M	0.97	0.442
NM_0164	chr11	94962622	94973612	-	1567	1	CWC15|AD	54.84	48.276
NM_0011	chr8	6499651	6563263	-	5256	1	ANGPT2|A	0.706	0.49
NM_0037	chr18	33851100	34223551	-	3944	1	NOL4|CT1	5.705	7.626
NM_0010	chr12	6666477	6689572	-	2882	1	ZNF384|C	27.979	30.008
NR_03747	chr5	1.71E+08	1.71E+08	-	104	1	MIR3912|	0	0
NM_0065	chr11	62242252	62244808	+	433	1	SCGB1D2|	0	0
NM_0013	chr16	85799698	85807003	+	684	1	COX4I1|C	498.963	452.823
NM_0021	chr6	32637406	32643652	+	1535	1	HLA-DQA1	0	0
NM_0012	chr19	44259880	44275317	+	2756	1	ZNF233|-|	0.299	2.117
NR_12037	chr15	90620009	90717140	-	650	1	CRTC3-AS1	3.273	4.678
NM_0012	chr8	84705927	84921843	+	1676	1	RALYL|HN	30.703	34.945
NM_0012	chr3	1.59E+08	1.59E+08	+	2232	1	MFSD1|SN	8.469	9.659
NM_0010	chr19	50516893	50568045	-	2952	1	LRRC4B|H	82.165	84.521
NM_0012	chr3	32685145	32773875	+	2714	1	CNOT10|-	7.938	8.398
NR_03170	chr12	79419257	79419321	+	64	1	MIR1252|	0	0
NR_13732	chr2	1.05E+08	1.05E+08	-	5560	1	TGFBRAP1	8.705	10.881
NM_0012	chr8	1.32E+08	1.32E+08	-	10682	1	KCNQ3|BF	1.998	7.565
NM_0012	chr10	68721144	68792377	+	4688	1	CCAR1|-|-	6.177	10.46
NM_0024	chr5	53560633	53683341	+	671	1	NDUFS4|A	90.658	77.079
NR_13551	chr21	26393635	26518592	+	417	1	CYYR1-AS1	0	0.066
NM_0012	chr12	53210566	53232256	-	2482	1	RARG|NR1	3.122	5.513
NM_0176	chr4	1.83E+08	1.83E+08	+	3584	1	CDKN2AIP	9.634	9.325
NR_07315	chr1	2.28E+08	2.28E+08	-	882	1	C1orf145|	0.789	1.444
NR_13011	chr7	1E+08	1E+08	-	1530	1	TSC22D4|	10.492	10.062
NM_0008	chr6	52750087	52763563	-	1300	1	GSTA2|GS	0	0
NR_00370	chr9	92292461	92292593	-	132	1	SNORA84|	0.098	0

NM_0012(	chr9	41891890	42129504	-	6350	1	CNTNAP3E	0.136	0.032
NR_02666	chr12	1.1E+08	1.1E+08	-	804	1	FAM222A-	0.16	0.426
NM_0010(	chr1	1.58E+08	1.58E+08	+	940	1	OR10K1|C	0	0
NM_0012{	chr9	1.37E+08	1.37E+08	-	1059	1	EXD3|mut	3.421	3.427
NM_1532	chrX	21655349	21658387	-	3036	1	KLHL34|-|	0.212	0.329
NR_04000	chr3	75672281	75679303	+	1277	1	LINC0096C	2.736	3.4
NM_1532!	chr19	36636621	36666853	-	3960	1	ZNF461|G	1.934	2.407
NM_0057(	chr11	2400488	2403878	+	1731	1	TSSC4|-|-|	24.811	23.509
NM_0056	chr6	34877778	34888071	-	1555	1	TAF11|MC	38.15	37.612
NM_0307!	chr2	16549462	16665866	-	4685	1	FAM49A|-	29.414	32.227
NR_03158	chrX	1.15E+08	1.15E+08	+	84	1	MIR764|h:	0	0
NM_0055!	chr1	1.16E+08	1.16E+08	-	2506	1	NHLH2|HE	13.398	15.501
NR_00336	chr15	25269783	25269858	+	75	1	SNORD115	0	0
NR_13426	chr5	29880560	29882103	+	388	1	LOC10537	0	0
NR_03156	chrX	1.15E+08	1.15E+08	+	68	1	MIR1264|	0	0
NM_0207	chr17	29255836	29294148	-	10874	1	NUFIP2|18	4.799	5.629
NM_0331(	chr19	32978592	33064918	-	3514	1	RHPN2|P7	6.15	3.489
NM_1532(	chr17	74326212	74355820	+	3621	1	KIF19|KIF1	3.418	0.768
NM_0011(	chr8	1.44E+08	1.44E+08	-	3234	2	MROH6|C	0	0
NR_02732	chr8	1.25E+08	1.25E+08	+	3575	1	LINC00964	0	0.019
NR_13276	chr4	82898140	82898225	-	85	1	SNORD144	0	0.161
NM_0252	chr6	28124556	28130086	+	1330	1	ZSCAN16|	16.635	13.234
NM_0013	chr19	11145494	11155812	-	1401	1	SPC24|SPE	3.276	2.298
NM_0028	chr2	1.2E+08	1.2E+08	+	2254	1	RALB|-|-|	15.199	14.128
NR_00323	chr20	38446654	38450921	+	1082	1	SNHG11|C	26.919	22.191
NR_03388	chr2	1.73E+08	1.73E+08	-	2529	1	MAP3K20-	0.326	0.921
NM_0010	chr1	1.82E+08	1.82E+08	-	7668	1	GLUL|GLN	46.945	52.759
NR_12052	chr7	1.15E+08	1.15E+08	+	1183	1	LINC01393	0	0.046
NR_10382	chr10	46219025	46269608	-	982	1	ANTXRLP1	0	0
NR_02418	chr14	93997296	94011695	+	1861	2	LINC00521	0	0
NR_03894	chr10	1.04E+08	1.04E+08	+	2020	1	SH3PXD2A	0	0.007
NR_12062	chr10	1.33E+08	1.33E+08	-	2617	1	LINC01166	0.256	0.696
NM_0011	chr3	58427630	58433852	-	1460	1	PDHB|PDH	48.368	51.223
NR_11050	chr2	6626109	6633787	-	291	1	LINC01246	0	0.047
NR_10672	chr9	95065350	95065446	+	96	1	MIR6081|	0	0
NM_0012	chr22	50343317	50445089	+	4000	1	PPP6R2|KI	29.826	34.801
NM_1533	chr1	1.09E+08	1.09E+08	+	2369	1	ATXN7L2|-	9.154	10.436
NM_0013(	chr17	11977439	11997510	-	2318	1	ZNF18|HD	7.771	8.958
NM_0011	chrX	1.3E+08	1.3E+08	+	7334	1	BCORL1|B	14.455	11.491
NM_0020	chr22	23070483	23125034	+	3203	1	GNAZ|-|-|	61.553	76.324
NR_04982	chr14	1.07E+08	1.07E+08	-	114	2	MIR5195|-	0	0
NM_0307!	chr17	46762506	46818760	-	3347.3	3	WNT3|INT	0	0.029
NM_0013(	chr7	1E+08	1E+08	-	2043	1	C7orf43|-|	16.131	18.088
NM_0141(	chr3	12557014	12583711	+	2836	1	MKRN2|H:	20.368	21.391
NR_04620	chr9	62452490	62522018	+	1164	1	LINC01189	0	0
NR_03017	chr16	14303967	14304049	+	82	1	MIR193B|	0	0
NM_0332	chr5	65589680	65624360	-	2400	1	TRIM23|A	3.804	4.703
NM_0013	chr22	26491240	26512506	-	3253	1	TFIP11|NT	25.926	26.897
NM_0582	chr16	70250231	70251930	-	1697	1	EXOSC6|E	8.836	8.394
NM_1768!	chr9	21480840	21482313	-	1471	1	IFNE|IFN-E	0	0

NR_00245	chr16	29449345	29449915	+	570	2	LOC60672	0	0.024
NR_07357	chr3	1.12E+08	1.12E+08	+	2566	1	ABHD10|-	20.496	19.142
NM_0010	chr10	84220491	84225589	-	3146	1	LRIT2|LRR	0	0.039
NM_0011	chr17	44656394	44675797	+	4670	1	MEIOC|C1	0.064	0.164
NM_0010	chrX	1.36E+08	1.36E+08	-	1001	3	CT45A6|C	0	0
NM_0022	chr1	2.27E+08	2.27E+08	-	6152	1	ITPKB|IP3-	2.416	4.655
NM_0010	chr4	64278459	64409460	-	2800	1	TECRL|CPV	0.018	0.064
NR_02967	chr20	63178500	63178586	+	86	1	MIR124-3|	0	0
NM_0010	chr4	71187286	71572087	+	7665	1	SLC4A4|HI	0.168	0.842
NM_1826	chrX	1.08E+08	1.08E+08	+	3119	1	VSIG1|170	0	0.004
NM_0198	chrX	46573687	46598496	+	2470	1	CHST7|C6	1.101	1.758
NM_0010	chr3	1.61E+08	1.62E+08	+	1429	1	OTOL1|C1	0.623	0.383
NM_0174	chr9	1.15E+08	1.15E+08	+	1241	1	DEC1|CTS	0	0.022
NR_13853	chr5	1.48E+08	1.48E+08	+	654	1	SPINK13|H	0.118	0.084
NM_1750	chr9	1.28E+08	1.28E+08	-	1597	1	ST6GALNA	5.934	8.714
NM_0156	chrX	69505235	69532508	+	4014	1	FAM155B|	10.15	13.499
NM_0013	chr19	4043304	4065529	-	6313	1	ZBTB7A|FE	1.544	1.64
NM_1985	chr11	11270874	11622014	-	2502	1	GALNT18|	11.224	10.002
NR_03030	chr5	36147892	36147988	-	96	1	MIR580|M	0	0
NR_03788	chr4	99088857	99301356	+	4815	1	LOC10050	0.367	0.552
NR_03846	chr10	80207710	80219657	+	2159	1	LINC00857	0	0
NM_0012	chr12	57604622	57609804	+	2063	1	DTX3|RNF	79.261	91.524
NR_12602	chr8	1.35E+08	1.35E+08	+	516	1	NCRNA002	0.15	0
NM_0066	chr9	1.09E+08	1.09E+08	-	1404	1	ACTL7B|Ta	0.064	0.02
NR_03339	chr12	9417691	9448172	-	3882	1	DDX12P|C	0.143	0.205
NM_0010	chr11	56232106	56233185	-	1078	2	OR5T2|OR	0	0
NR_04854	chr3	45482695	45509545	-	1850	1	LARS2-AS1	0.042	0
NM_0012	chr17	32142454	32225771	+	3109	1	RHOT1|AR	12.918	16.147
NM_0013	chr2	2.19E+08	2.19E+08	+	1076	1	BCS1L|BCS	32.006	25.879
NR_00155	chrX	26558337	26561052	+	2715	1	VENTXP1|	0	0
NR_02830	chr3	1.07E+08	1.07E+08	+	3259	1	DUBR|DUI	6.144	4.922
NM_0012	chr6	2999816	3019876	+	1150	1	NQO2|DH	27.693	27.43
NM_0031	chr2	1.7E+08	1.7E+08	+	1692	1	SSB|LARP3	18.197	20.537
NR_04664	chr3	1.49E+08	1.49E+08	+	412	1	HLTF-AS1|	3.255	1.164
NM_0015	chr4	57031071	57110385	-	1122	1	IGFBP7|AC	0.299	0.439
NR_03793	chr14	70468879	70483756	-	3034	1	ADAM20P	0.127	0.275
NM_0010	chr6	38168451	38640148	-	8736	1	BTBD9|dJ3	6.562	7.759
NM_0177	chr1	1.18E+08	1.18E+08	+	5716	1	FAM46C|-	0.065	0.079
NM_0041	chr4	41256881	41268429	+	1113	1	UCHL1|HE	846.799	1089.756
NM_0249	chr3	13316230	13420319	-	7166	1	NUP210|G	6.15	6.478
NR_13391	chr19	42428538	42443048	-	935	1	CXCL17|DI	0	0
NR_03746	chr9	1.35E+08	1.35E+08	-	147	1	MIR3689B	0	0
NR_13300	chr15	89362521	89398487	+	4073	1	MIR9-3HG	8.579	11.656
NM_0020	chr19	6379569	6393280	-	2536	1	GTF2F1|B	68.311	60.735
NR_11062	chr1	95510116	95515464	+	1559	1	LOC10099	0	0.237
NM_0027	chr6	1.71E+08	1.71E+08	-	909	1	PSMB1|H	137.899	130.807
NM_0013	chr17	4710494	4721500	+	1828	1	ARRB2|AR	58.832	52.698
NM_0010	chr3	1.87E+08	1.87E+08	-	2501	1	MASP1|3M	13.058	13.549
NM_0141	chr8	1.23E+08	1.23E+08	-	5534	1	ATAD2|AN	2.088	3.638
NM_1333	chr5	1.77E+08	1.77E+08	+	3716	1	UNC5A|UN	14.136	24.442

NM_0122(	chr11	62707594	62709206	+	946	1	GNG3|-|-|	181.885	149.35
NR_11087	chr17	72034107	72057734	-	579	1	LOC10272.	0	0
NM_0026(	chr16	81779258	81962693	+	8672	1	PLCG2|AP	0.123	0.199
NR_03170	chrX	1.47E+08	1.47E+08	-	83	1	MIR513B|	0	0
NR_13458	chr18	27336991	27342656	-	444	1	LOC10537.	0	0.062
NM_0147!	chr15	43327776	43330622	-	2844	1	LCMT2|PP	9.511	11.91
NM_0149(	chr1	1.09E+08	1.09E+08	-	4336	1	WDR47|-|	31.365	47.772
NM_0150!	chr13	41566825	41961085	-	7113	1	VWA8|KIA	2.251	2.313
NM_0046!	chr8	17296797	17413531	-	5271	1	MTMR7|-|	3.65	2.97
NM_0148!	chr1	6590724	6602898	-	4510	1	KLHL21|-|	4.897	5.977
NM_0011	chr6	96794122	96837477	-	6083	1	GPR63|PS	0.524	1.004
NM_0040!	chr17	43766121	43779000	-	4117	1	DUSP3|VH	26.235	20.228
NR_03167	chr15	52277117	52277200	-	83	1	MIR1266|	0	0
NM_0050	chr19	18153178	18170533	+	3948	1	PIK3R2|M	33.429	52.887
NR_13123	chr17	48294347	48307829	+	725	1	THRA1/BT	0.071	0
NM_0203!	chr3	1.46E+08	1.46E+08	-	3295	1	PLSCR4|TF	0.137	0.279
NM_0013	chrX	1.42E+08	1.42E+08	+	675	1	LOC64518.	0	0.02
NM_0012	chr19	54662819	54670359	+	3888	5	LILRB4|CD	0	0
NR_13298	chr9	1.33E+08	1.33E+08	-	103.7	3	SNORD141	2161.752	2457.403
NM_0012!	chr12	7789396	7796061	+	2049	1	NANOG|-|	0	0
NR_13582	chr15	1.01E+08	1.01E+08	-	3255	1	LOC10192	0.079	0.021
NM_0012	chr14	21070260	21090247	+	5600	1	ARHGEF40	21.254	16.908
NR_04747	chr17	43221417	43229011	-	961	1	LINC00854	0.067	0.043
NM_0035	chr6	26240426	26240793	+	365	1	HIST1H4F|	0	0.038
NM_1533(	chr10	35605410	35608935	+	1576	1	GJD4|CX4(	0.016	0.026
NM_0046	chr9	99105089	99154191	+	6504	1	TGFBR1|A	15.5	10.127
NM_0050(	chr10	1E+08	1E+08	+	5460	1	SCD|FADS.	179.285	119.069
NM_0181	chr16	74621399	74666881	-	4937	1	RFWD3|RI	7.237	7.829
NM_0012	chr12	1.22E+08	1.22E+08	-	1853	1	DIABLO|D	55.394	50.464
NR_03995	chr3	19314848	19314931	-	83	1	MIR4791|	0	0
NM_0239	chr12	10938254	10939207	-	952	3	TAS2R14|	0	0
NM_0010(	chr9	37588415	37592639	-	742	1	TOMM5|C	74.581	63.206
NM_0010	chr1	2.03E+08	2.03E+08	+	2759	1	ADORA1|F	6.15	14.377
NM_0013	chr16	20933111	21150807	-	12464	1	DNAH3|DI	0.013	0.002
NM_0247(	chr9	27325209	27529852	-	6519	1	MOB3B|C!	1.007	3.656
NM_0019	chr1	1E+08	1E+08	-	10802	1	DBT|BCAT	1.16	1.401
NM_0010(	chr19	9102269	9103306	-	1036	1	OR7G2|OF	0	0
NR_02449	chr10	47535581	47553523	-	4688	1	BMS1P6|E	0.204	0.257
NM_0040	chr15	79960890	79971301	-	883	1	BCL2A1|A	0	0
NR_13556	chr3	86481943	86496996	+	1319	1	LINC0207C	0	0.239
NM_0010	chr9	1.29E+08	1.29E+08	+	839	1	C9orf106|	0	0.049
NR_03387	chr2	69962263	70087015	-	2375	1	PCBP1-AS1	5.195	6.05
NR_03992	chr2	32635255	32635331	+	76	1	MIR4765|	0	0
NM_1525	chr22	30661458	30667890	-	3048	1	DUSP18|D	7.796	5.073
NR_13427	chr8	57746149	57750197	+	2457	1	LOC28617	0.068	0.05
NM_0203	chr16	57447425	57461275	+	1678	1	COQ9|C16	35.376	27.859
NR_03341	chr17	32040336	32042832	+	2496	1	SH3GL1P1	0.429	0.269
NM_0010(	chr17	81189593	81222989	-	3522	1	CEP131|A	11.942	8.365
NM_0230(	chr20	3686970	3707128	-	6698	1	SIGLEC1|C	0.218	0.17
NM_0580(	chr22	20707691	20858812	-	6695	1	PI4KA|PI4	22.823	28.157

NR_03965	chr1	2.29E+08	2.29E+08	-	51.2	8	MIR4454|-	0	0
NM_0011(	chr15	73560003	73633412	-	2084	1	NPTN|GP5	49.687	63.083
NM_0326:	chr19	35545600	35547527	+	886	1	TMEM147	88.523	71.793
NM_0184:	chr14	58200115	58235635	+	2426	1	ACTR10|A	57.415	52.631
NR_00320	chr14	1.01E+08	1.01E+08	+	68	1	SNORD114	0	0
NM_0058(	chr3	37862178	37984469	+	4413	1	CTDSPL|C3	1.811	3.597
NR_02434	chr12	1.21E+08	1.21E+08	-	2454	1	HNF1A-AS:	0	0
NM_0012(	chr19	1524074	1535456	+	1917	1	PLK5|PLK-	0.834	0.9
NM_0189(	chr7	25118651	25125361	-	5533	1	CYCS|CYC|	15.219	12.407
NR_11194	chr8	1296034	1302607	-	3046	2	LOC28608:	0	0
NR_12579	chr2	32557521	32574818	-	586	1	BIRC6-AS2	0	0.023
NR_13812	chr19	37334624	37364455	+	2602	1	HKR1|ZNF	19.474	21.714
NR_11090	chr16	47144068	47162746	+	711	1	ITFG1-AS1	10.645	10.152
NM_0011(	chr17	12665890	12768948	+	8548	1	MYOCD|M	0	0.01
NM_0010:	chr10	68285081	68332927	-	1213	1	PBLD|HEL-	2.105	1.615
NM_0048(	chr14	1.04E+08	1.04E+08	-	647	1	C14orf2|M	133.518	114.657
NM_0013:	chr1	2.28E+08	2.28E+08	+	7530	1	IBA57|C1c	2.306	1.452
NM_0206:	chr2_GL38	57520	77061	-	1337.5	2	SPC25|AD(	0.125	0.092
NM_0060(	chr9	1.37E+08	1.37E+08	+	1585	1	TUBB4B|B	147.254	114.899
NR_04989	chr8	80241389	80241473	+	84	1	MIR5708|-	0	0
NR_03836	chr7	77683851	77697345	-	1975	1	APTR|RSBI	5.634	5.021
NM_0012(	chr3	1.14E+08	1.14E+08	-	1547	1	DRD3|D3D	0.042	0
NM_0325:	chr3	1.09E+08	1.09E+08	-	568	1	RETNLB|FI	0	0
NM_0011(	chr11	1.26E+08	1.26E+08	-	2410	1	RPUSD4|-|	12.572	12.776
NR_04002	chr6	1.66E+08	1.66E+08	+	3906	1	LOC10028(	0.961	1.034
NM_2033:	chr11	26994081	26997085	+	3002	1	FIBIN|-|-|:	0.614	0.858
NM_0011:	chr12	57610180	57617245	+	2230	1	ARHGEF25	37.593	58.301
NM_0013:	chr20	408050	410764	+	1223	1	RBCK1|C2(	16.995	14.302
NM_0007(	chr7	1.01E+08	1.01E+08	+	1324	1	EPO|EP|M	0.156	0.093
NM_0011(	chr9	273048	465259	+	7093	1	DOCK8|HE	0.009	0.004
NM_0201(	chr14	24439766	24442801	-	1223	1	SDR39U1|	72.596	72.696
NM_0176(	chr19	55090913	55117600	-	2941	1	PPP1R12C	22.81	25.014
NR_11032	chr19	16034120	16042135	+	1433	1	LINC00905	0	0
NR_04684	chr13	44146470	44158222	-	475	1	SMIM2-IT1	0	0.029
NM_0013(	chr15	55317184	55319194	-	944	1	PIGBOS1|I	6.679	6.021
NM_0013(	chr3	1.5E+08	1.5E+08	+	4764	1	TSC22D2|1	4.42	4.431
NM_0142(	chr20	59863557	59933663	-	5534	1	SYCP2|SCF	0	0.017
NR_03898	chr6	33889511	33896907	-	3148	1	LINC01016	0	0
NR_02695	chr17	8220630	8224043	-	2091	1	LINC00324	2.855	2.404
NM_0010(	chr15	90352245	90361483	+	1832	1	ZNF774|-|	2.034	2.437
NR_03386	chr9	13406380	13431329	-	2325	1	LINC01235	0.011	0.059
NR_13512	chr12	1.02E+08	1.02E+08	+	2163	1	PARPBP|A	0.489	0.45
NM_0013(	chr14	24205651	24208248	+	1012	1	TSSK4|C14	2.089	2.098
NR_12584	chr6	1.28E+08	1.28E+08	+	1810	2	LOC10192:	0	0
NM_1522(	chr12	1.07E+08	1.07E+08	+	3409	1	TMEM263	5.004	5.4
NM_0210:	chr2	2.34E+08	2.34E+08	+	2369	1	UGT1A9|H	0.033	0
NR_04616	chr9	94176570	94204566	-	1287	1	MIRLET7D	0.351	0.447
NM_1525(	chr12	87980039	88029399	-	1690	1	C12orf50|	0	0.008
NR_02667	chr9	1.23E+08	1.23E+08	-	5983	1	MIR600HG	6.084	6.33
NM_0159:	chr13	28659004	28678956	+	1453	1	POMP|C13	44.732	44.928

NM_0203(	chr8	67422170	67746385	-	1891	1	CPA6|CPA	0	0
NM_0184(	chr9	5357966	5437937	-	990	1	PLGRKT|A	24.875	22.123
NR_00271	chr17	43300044	43300231	-	187	10	RNU2-1|R	0	0
NM_0013:	chr1	1.93E+08	1.93E+08	-	549	1	GLRX2|CG	3.617	3.543
NM_0010!	chr3	4493348	4847840	+	10037	1	ITPR1|ACV	0.41	0.629
NM_0330	chr3	15427557	15442613	+	4480	1	EAF1|-|-|3	19.824	20.102
NM_1303	chr3	57268351	57278952	-	2194	1	ASB14|-|-	1.023	1.024
NM_0027!	chr17	4796144	4798503	+	844	1	PSMB6|DE	152.308	128.401
NM_0052!	chr7	1.07E+08	1.07E+08	+	2961	1	GPR22|-|-	0.505	0.093
NM_0011(	chr2	47086991	47155378	-	882	1	C2orf61|-|	0.117	0.326
NR_03388	chr1	20359801	20428794	-	3771	1	LINC01141	0.003	0.011
NR_03062	chr12	24212421	24212495	+	74	1	MIR920|M	0	0
NM_1384	chr1	34850362	34859816	-	5757	1	SMIM12|C	7.382	7.088
NM_0013	chr19	11342776	11346305	-	710	1	TMEM205	57.131	46.898
NM_0019(	chr20	23875935	23879743	-	744	1	CST5|-|-|2	0	0
NR_03982	chr10	89063336	89063410	+	74	1	MIR4679-1	0	0
NM_0010(	chr4	1.45E+08	1.46E+08	+	2868	1	SMAD1|BS	8.555	13.43
NR_03747	chr7	71307674	71307768	+	94	1	MIR3914-2	0	0
NR_02438	chr15	21371511	21373968	-	2457	2	CXADRP2|	0	0
NM_0014(	chr19	1286154	1301430	+	2136	1	EFNA2|ELI	26.861	15.339
NM_0023	chr2	95025655	95053992	+	1131	1	MAL|-|-|2	2.519	1.744
NM_0010:	chr1	62436304	62451804	+	3403	1	USP1|UBP	3.759	7.543
NM_1816(	chr21	30613686	30613904	-	217	1	KRTAP6-1|	0	0
NM_0012(	chr13	1.03E+08	1.03E+08	+	5118	1	BIVM-ERC(	12.072	12.479
NM_0244	chr18	31129251	31162856	-	4192	1	DSC1|CDH	0.043	0.062
NM_0013	chr1	46040140	46130673	-	4852	1	PIK3R3|p5	52.618	58.15
NM_0012(	chr9	1.32E+08	1.32E+08	-	2143	1	UCK1|URK	31.587	35.85
NR_13628	chr9	97155893	97238742	-	3902	1	ANKRD18C	0.007	0.011
NR_04703	chr15	25108268	25108352	+	84	1	SNORD116	0	0.163
NM_0012!	chr6	24544104	24583227	-	5296	1	KIAA0319|	1.227	1.536
NM_0122(	chr22	38219291	38273034	-	3600	1	TMEM184	22.184	28.619
NR_04983	chr22	22886267	22886379	+	112	1	MIR5571|-	0	0
NM_0013	chr21	42974510	43033582	+	4987	1	PKNOX1|P	6.332	7.108
NR_03652	chr19	56478124	56495436	+	1425	1	ZNF667-AS	19.462	24.299
NM_0216	chr5	1.16E+08	1.16E+08	-	3199	1	TICAM2|M	0.544	0.381
NM_1471:	chr6_GL00	2836620	2840722	-	1048	8	NCR3|1C7	0	0
NM_0325!	chr2	1.13E+08	1.13E+08	+	1360	1	IL1F10|FIL	0	0
NR_02968	chr2	2.19E+08	2.19E+08	-	89	1	MIR153-1|	0	0.154
NM_0012	chr19	9412426	9435578	-	3726	1	ZNF266|H:	11.077	11.741
NM_1827	chr7	1.06E+08	1.06E+08	-	2293	1	SYPL1|H-S	3.453	5.645
NM_0012!	chrX	1.36E+08	1.36E+08	-	1221	1	CT45A10|-	0	0
NM_1788	chr16	3539036	3577404	-	6389	1	NLRC3|CLI	0.438	0.439
NM_0010!	chr18	36111000	36129394	-	1656	1	SLC39A6|L	47.284	40.264
NM_0308:	chr16	67674533	67719370	-	2070	1	GFOD2|-|-	39.976	42.355
NR_02960	chr11	72615063	72615130	-	67	1	MIR139|M	0	0
NM_0062(	chr5_KI27(	130718	131533	-	1200.3	3	PROP1|CP	0	0
NR_10506	chr5	1.5E+08	1.5E+08	-	404	1	NDST1-AS:	1.883	1.763
NR_10980	chr9	1.04E+08	1.04E+08	+	447	1	LOC10192	0	0
NM_0012(	chr5	73498425	73505623	+	1000	1	BTF3|BET/	204.284	196.58
NM_1385!	chr2	60451167	60553498	-	2351	1	BCL11A|B(	21.57	38.166

NR_10416	chr10	62339588	62375127	-	2617	1	LOC28304	0.015	0.042
NM_0144	chr10	70478808	70568450	+	4547	1	PALD1|KIA	4.248	6.353
NM_0011	chr11	47565430	47573461	+	2544	1	PTPMT1|D	16.132	17.132
NM_0211	chr11	1947272	1956609	+	735	2	MRPL23|L	4.473	2.944
NM_0011	chr2	2.33E+08	2.33E+08	+	7700	1	GIGYF2|G	12.905	13.21
NM_0011	chrX	1.5E+08	1.5E+08	+	2079	1	MAGEA8|	0	0.026
NR_13569	chr15	21717666	21718307	-	555	2	LOC10272	0	0
NM_0071	chr11	1.19E+08	1.19E+08	-	1837	1	TREH|TRE	0.119	0.067
NM_0015	chr4	1.84E+08	1.84E+08	+	2461	1	ING2|ING	4.228	3.161
NR_04755	chr10	43645942	43648019	+	902	1	ZNF32-AS	5.832	6.469
NR_12591	chr4	15004942	15427914	-	624	1	LOC10192	0.062	0.088
NM_1768	chr12	10985913	10986912	-	997	3	TAS2R50|	0	0
NR_03760	chr16	30194433	30204329	+	2205	2	SLX1B-SUL	0.076	0.043
NR_03618	chr10	1.31E+08	1.31E+08	-	80	1	MIR378C|	0	0
NM_0013	chr19	18990887	19033571	-	3593	1	SUGP2|SF	59.11	66.061
NR_12590	chr4	1.76E+08	1.76E+08	+	1205	1	LOC10192	0.342	0.523
NM_1449	chr17	82023302	82031151	+	2682	1	LRRC45|-|	9.264	9.412
NM_0011	chr2	2.28E+08	2.28E+08	+	833	1	CCL20|CK	0	0
NM_0013	chr2	1.13E+08	1.13E+08	+	1754	1	CBWD2|-|	2.286	2.226
NM_0012	chr1	1.14E+08	1.14E+08	-	4360	1	SYT6|sytV	1.993	5.13
NM_0007	chr17	39173456	39197703	-	3726	1	CACNB1|C	26.86	26.571
NM_0165	chrX	1.32E+08	1.32E+08	+	3322	1	STK26|MA	1.18	0.924
NM_0045	chr19	50432903	50466326	+	3568	1	MYBPC2|N	0.17	0.407
NR_13124	chr6	52146814	52151023	-	363	1	LINCMD1|	0	0
NR_12049	chr10	5514244	5526246	-	3594	1	CALML3-A	0	0
NM_0063	chr9	1.29E+08	1.29E+08	-	4280	1	ZER1|C9or	31.704	37.008
NM_0013	chr9	83968083	83980271	-	2855	1	HNRNPK|A	146.9	138.529
NR_04758	chr3	53225636	53256114	-	2040	1	TKT|HEL-S	124.251	124.233
NM_0123	chr7	78017057	79453574	-	6856	1	MAGI2|AC	5.98	6.265
NR_02728	chr20	63953384	63956985	+	3601	1	UCKL1-AS1	1.801	2.145
NR_10686	chr2	2.38E+08	2.38E+08	+	57	1	MIR6811|	0	0
NM_0540	chrX	1.37E+08	1.37E+08	-	1525	1	GPR101|G	0.042	0.072
NR_03625	chrX	1.55E+08	1.55E+08	-	98	3	MIR1184-	0	0
NR_12600	chr12	52609851	52615305	+	3805	1	KRT73-AS1	0	0
NR_03990	chr19	53787677	53787742	-	65	1	MIR371B|	0	0
NR_11007	chr14	81605347	81623061	-	1184	1	LINC01467	0	0
NM_0010	chr1	1.59E+08	1.59E+08	-	988	1	OR10J3|O	0	0
NM_1471	chr11	18933813	18935002	-	1187	1	MRGPRX1	0	0
NM_1984	chr1	1.74E+08	1.74E+08	-	2663	1	ANKRD45|	3.094	4.675
NM_0041	chrX	1.54E+08	1.54E+08	-	1470	1	IDH3G|H-I	41.233	47.604
NM_0012	chr15	40253178	40277487	+	2534	1	PAK6|PAK	3.74	9.594
NM_0012	chr20	5950652	5995206	+	3748	1	MCM8|C2	1.51	1.444
NR_13237	chr7	1.59E+08	1.59E+08	+	1205	1	LINC01022	0	0
NM_1386	chr22	37746234	37759956	+	1719	1	TRIOBP|DI	9.188	4.741
NM_0204	chr12	68686951	68745814	+	6215	1	NUP107|N	4.902	5.408
NR_03835	chr5	1.78E+08	1.78E+08	+	1065	1	FAM153C|	0	0
NM_0121	chr7	1.31E+08	1.31E+08	-	3117	1	COPG2|2-	35.102	36.653
NM_0010	chr12	25108289	25195162	-	2477	1	CASC1|LA	0.588	0.398
NM_0010	chr10	97614553	97634156	-	2326	1	MORN4|C	45.936	48.511
NM_0010	chr10	97850238	97871578	+	6441	1	GOLGA7B|	1.025	7.592

NM_0532:	chr21	34669163	34718227	+	4024	1	CLIC6|CLIC	1.243	0.051
NM_0059:	chr2	85538978	85545280	+	2995	1	MAT2A|M	54.794	69.194
NR_11066	chr17	44646364	44650349	-	2022	1	LINC0118C	0.006	0
NR_11074	chr17	34169376	34183632	-	1071	1	LINC01989	0	0
NR_03403	chr2	1.97E+08	1.97E+08	-	502	1	LOC10013(	0	0
NR_11031	chr15	43630562	43648063	-	1505	1	CATSPER2	0.625	0.473
NM_0013:	chr16	18791667	18801370	-	2325	1	ARL6IP1|A	129.761	125.812
NM_0012{	chr9	34458752	34520989	+	2577	1	DNAI1|CIL	1.111	0.728
NM_1450‹	chr11	6318946	6320510	-	1033	1	PRKCDBP|	0.025	0.013
NM_0011:	chr10	75111635	75182123	+	6774	1	SAMD8|HI	5.647	7.886
NM_0334(	chr20	63558086	63567754	-	7491	1	HELZ2|PDI	5.513	3.88
NM_0012(	chr10	1.14E+08	1.14E+08	+	2265	1	CASP7|CA:	2.448	1.754
NR_02711	chr5	1.59E+08	1.59E+08	-	2005	1	LINC01845	0	0
NR_00333	chr15	25082767	25082860	+	93	1	SNORD116	0	0
NM_0012:	chr15	32441914	32455634	-	5167	2	GOLGA8O	0	0.003
NM_0011:	chr4	70704937	70789897	+	1808	1	RUFY3|RIF	56.252	52.455
NR_13297	chr10	28073237	28073378	-	141	1	SNORD13C	0	0
NM_0013:	chr8	1.08E+08	1.08E+08	-	2463	1	RSPO2|CR	18.751	1.479
NM_0010‹	chr1	45329242	45339957	-	1677	1	MUTYH|M	12.655	12.235
NM_0013:	chr8	42174718	42207724	-	2940	1	PLAT|T-PA	4.149	5.991
NR_02937	chrX	18890296	18894975	+	1293	1	PHKA2-AS:	2.433	2.839
NR_03391	chr4	1.81E+08	1.81E+08	-	589	1	LINC0029C	0	0
NM_1985(	chr3	1.93E+08	1.93E+08	-	3626	1	ATP13A5|-	0.036	0.034
NM_0024:	chr16	15703135	15857030	-	6839	2	MYH11|A/	0	0
NR_10378	chr1	2.27E+08	2.27E+08	-	1345	1	ITPKB-IT1|	0	0
NR_12578	chr13	53131746	53151900	-	560	1	LINC01065	0	0
NM_0014:	chrX	49341190	49529985	+	515	3	GAGE5|CT	0	0
NM_0012{	chr4	499200	539921	+	3434	1	PIGG|GPI7	9.852	10.315
NR_03742	chr5	1.41E+08	1.41E+08	+	82	1	MIR3655|-	0.314	0
NR_02733	chr19	58559186	58574797	+	2600	1	MZF1-AS1	2.886	2.271
NR_02401	chr13	99351420	99352027	+	607	1	FKSG29|-|	0	0
NR_03771	chr17	35099793	35119869	-	2275	1	RAD51D|E	4.738	7.369
NM_1731(	chr2	1.62E+08	1.63E+08	-	2441	1	KCNH7|ER	0.729	2.222
NM_1994:	chr20	25078543	25082379	-	1191	1	VSX1|CAA	0	0
NM_0011:	chr6	1.59E+08	1.59E+08	-	3099	1	EZR|CVIL|	81.796	110.244
NM_0011!	chr16	3371053	3372283	-	1228	1	MTRNR2L4	0.031	0.134
NR_07306	chr6	30688047	30691129	-	1358	7	NRM|NRM	0	0
NR_02747	chr9	87008451	87042126	-	2014	1	LOC44017:	0.058	4.774
NM_0010:	chr11	1.06E+08	1.06E+08	+	3332	1	GRIA4|GLU	8.532	4.016
NM_1458(	chr1	2.36E+08	2.36E+08	+	2764	1	EDARADD|	37.664	16.497
NR_02969	chr14	1.01E+08	1.01E+08	+	72	1	MIR134|M	0	0
NR_03033	chr10	51299573	51299655	+	82	1	MIR605|M	0	0
NM_0012:	chr15	78104606	78131535	-	1436	1	CIB2|DFNE	24.018	28.434
NR_03413	chr18	73154058	73264498	-	2648	1	LOC40065:	0.005	2.747
NM_0042:	chr6	6588701	6654983	+	872	1	LY86|MD-	0	0
NM_0010(	chr3	1.88E+08	1.88E+08	-	1342	1	RTP2|Z3C)	0	0
NM_0247!	chr8	67064368	67196614	+	4687	1	CSPP1|CSF	1.926	2.651
NR_13296	chr1	44252344	44252559	-	215	1	SNORA11C	0	0
NM_0035:	chr6	27810064	27810536	+	470	1	HIST1H3H	1.015	0.816
NM_0011!	chr2	48440769	48515392	+	3028	1	PPP1R21|(	12.655	18.447

NR_03039	chrX	74218377	74218461	-	84	1	MIR421|M	1.228	0.489
NM_0124(	chr11	6474683	6481479	-	3697	1	ARFIP2|PC	26.526	30.588
NR_03759	chr4	1.45E+08	1.45E+08	-	1130	1	HHIP-AS1|	0.194	0.388
NM_0068z	chr19	15114974	15125799	-	2313	1	ILVBL|209	37.309	29.437
NM_0529:	chr17	41825082	41836271	-	1411	1	NT5C3B|N	84.881	92.627
NM_0013:	chr1	1.2E+08	1.2E+08	+	1624	1	HSD3B1|3	0	0
NM_1458(	chr7	1.36E+08	1.36E+08	-	3878	1	MTPN|GCI	39.369	44.531
NM_0012{	chr7	1.29E+08	1.29E+08	+	1696	1	FAM71F1|	0.585	0.63
NM_0012(	chr11	49053714	49059112	-	1346	1	TRIM64C|-	0	0.01
NR_03988	chr17	28861655	28861730	-	75	1	MIR4732|	0	0
NM_1446:	chr17	81228277	81239091	-	2273	1	TEPSIN|C1	6.813	6.743
NR_00351	chr11	2129112	2161209	-	5125	1	INS-IGF2|I	1.379	0.139
NR_00366	chr5	80350605	80351966	-	1361	1	CRSP8P|-|	6.016	8.383
NM_0011:	chr3	3149633	3179717	-	2575	1	CRBN|MR	10.896	13.234
NM_0013z	chr11	1.31E+08	1.31E+08	-	3867	1	SNX19|CH	19.812	22.417
NM_0068!	chr7	6461081	6484218	-	2853	1	KDELR2|EI	44.415	43.026
NM_0012{	chr4	6987508	7033118	+	4008	1	TBC1D14|-	50.76	72.343
NR_10705	chr16	28390982	28391051	-	69	2	MIR6862-2	0	0
NM_0011(	chr19	8302127	8308356	-	1119	1	CD320|8D	29.739	26.672
NM_1816:	chr21	30348399	30348606	-	206	1	KRTAP23-1	0	0
NM_0013:	chr17	42798887	42811587	+	2999	1	CNTD1|CN	5.568	5.709
NR_13450	chr20	47352561	47354632	+	1806	1	LOC10192	0.243	0.281
NM_0010{	chr18	5890185	5892104	-	1917	1	TMEM200	6.867	5.194
NM_0012(	chr4	56467596	56503681	+	3674	1	SRP72|BM	20.8	22.592
NM_0179:	chr14	90278054	90332137	-	3994	1	NRDE2|C1	3.745	3.954
NR_03609	chr5	1.6E+08	1.6E+08	+	81	1	MIR3142|-	0	0
NM_0062z	chr14	63373892	63543361	-	4095	1	PPP2R5E|-	8.652	12.931
NM_0012!	chr19	4910367	4962154	+	3852	1	UHRF1|ICE	4.398	3.005
NR_10993	chr21	45972970	45974953	+	1065	1	LOC10192	0.048	0.026
NM_0012:	chr6	15522808	15663058	-	1297	1	DTNBP1|B	19.087	31.882
NM_0013(	chr1	2476315	2505530	+	5787	2	PLCH2|PL(	0	0
NM_0010:	chr16	28292519	28323849	+	4986	1	SBK1|SBK|	152.444	168.35
NM_0011(	chr8	21692018	21788834	-	3005	1	GFRA2|GD	3.617	3.601
NM_0017!	chr14	94304248	94323351	-	1485	2	SERPINA6|	0	0
NM_1452(	chr11	67455347	67461774	+	4012	1	CABP4|CR	0.035	0.027
NM_0012(	chr17	78231310	78240987	+	2403	1	TMEM235	0.123	0.011
NM_0005!	chr6_GL0(	4088779	4097541	-	2939	8	TAP1|ABC	0	0
NM_0012{	chr15	42210452	42273584	-	3028	1	TMEM87A	13.128	12.43
NM_0010(	chr17	36165681	36176624	-	2093.7	3	TBC1D3B|	0	0
NR_03832	chr1	87353526	87371655	-	883	1	LINC01364	0	0
NM_0012:	chr10	31805398	31908926	-	4698	1	ARHGAP12	5.717	6.33
NM_0010z	chr3	1.61E+08	1.61E+08	-	2301	1	SPTSSB|AI	0.134	0.113
NM_0011!	chr7	1.08E+08	1.08E+08	-	6282	1	NRCAM|-|	28.092	29.271
NM_0011!	chr16	70480569	70523554	-	2739	1	COG4|CD(	45.506	42.771
NM_0011!	chr4	1.28E+08	1.28E+08	+	3755	1	PLK4|MC(	1.02	1.627
NM_0011:	chr12	16188485	16277685	-	2913	1	SLC15A5|-	0.009	0.019
NM_0012(	chr13	35476749	35672737	+	4071	1	NBEA|BCL	27.358	18.417
NM_0012{	chr6	1.7E+08	1.7E+08	+	3248	2	FAM120B|	0	0
NR_04011	chr9	87858043	87859056	-	1013	1	LOC39236	0	0
NM_0013:	chr3	48440238	48444208	+	568	1	TMA7|CCI	14.687	11.382

NM_0027!	chr22	50263713	50270393	-	2417	1	MAPK11|I	24.016	23.217
NM_0011{	chr19	48238261	48255946	-	620	1	CARD8|CA	4.367	3.778
NM_1527{	chrX	30827442	30889394	-	6774	1	TAB3|MAI	4.954	5.298
NM_0059(	chr17	8474205	8630761	-	7626	1	MYH10|NI	38.317	35.044
NR_10805	chr10	4406469	4410612	+	2261	1	LINC0070:	0.034	0.145
NR_03029	chr3	1.96E+08	1.96E+08	+	96	8	MIR570|M	0	0
NM_0327:	chr3	1.98E+08	1.98E+08	+	2285	1	LRCH3|-|-	10.766	8.446
NM_0053:	chr22	37805107	37807436	+	2327	1	H1F0|H10	129.4	244.427
NM_0160:	chr17	57838926	57850072	-	1485	1	MRPS23|C	43.812	41.073
NM_0164{	chr1	45688175	45694436	+	1466	1	TMEM69|	13.984	14.407
NM_0010:	chr11	61222349	61231695	+	1372	1	PGA4|-|-|	0	0
NR_02813	chr3	1.79E+08	1.79E+08	-	2122	1	KCNMB3|I	0.194	0.265
NM_0025!	chr7	1.04E+08	1.04E+08	-	1942	1	ORC5|ORC	6.095	7.829
NM_0012(	chr7	23710167	23832511	+	3230	1	STK31|SGI	0.036	0.208
NM_1730!	chr7	1.01E+08	1.01E+08	+	8348	1	ZAN|-|-|7	0	0.002
NM_0010!	chr19	13877249	13880757	+	829	1	NANOS3|I	7.046	5.221
NR_11061	chr1	23281309	23285518	-	3509	1	LINC0135!	0.305	0.496
NM_0005!	chr12	5948874	6124675	-	8785	1	VWF|F8VV	0.015	0.012
NR_04637	chr13	29476515	29487750	-	1111	1	MTUS2-AS	0.116	0.136
NM_0004:	chr17	44372181	44389601	-	3399	1	ITGA2B|BI	0.77	1.161
NM_2074:	chr10	1.28E+08	1.28E+08	+	3174	1	FOXI2|-|-|	0.004	0
NR_00326	chr3	1.96E+08	1.96E+08	-	2474	1	SDHAP1|S	2.001	1.655
NM_0011‹	chr2	1.3E+08	1.3E+08	+	3335	1	PTPN18|B	7.69	7.019
NM_0013(	chr18	45724123	45752520	+	3792	1	SLC14A1|I	0	0.004
NM_0805‹	chr12	6946577	6961316	+	2141	1	PTPN6|HC	0.391	0.518
NM_0010:	chr16	74408631	74421470	-	1928	1	CLEC18B|I	0.535	0.291
NM_1524(	chr4	55556525	55592212	-	853	1	PDCL2|GC	0.06	0
NR_10381	chr13	22210285	22276520	+	2140	1	LINC0054C	0.048	0
NM_0011(	chr4	1.08E+08	1.08E+08	-	2629	1	LEF1|LEF-:	7.116	18.855
NR_02400	chr4	1.83E+08	1.83E+08	-	2178	1	WWC2-AS:	0.036	0.038
NM_1722!	chr4	1.46E+08	1.46E+08	+	5934	1	MMAA|cb	1.16	1.639
NR_04673	chr2	27049683	27050264	-	405	1	AGBL5-AS:	0.318	0.541
NR_04666	chr1	1.55E+08	1.55E+08	+	441	1	UBE2Q1-A	13.771	15.343
NR_07304	chr19	42198593	42220152	-	1573	1	DEDD2|FL	32.836	28.726
NM_0012:	chrX	52184823	52192268	-	2557	2	MAGED4|I	0.03	0.043
NM_0228:	chr4	52590961	52659335	-	7954	1	USP46|-|-	3.608	4.305
NM_0010{	chr17	74356416	74361819	-	1674	1	BTBD17|B	23.53	18.819
NR_11003	chr14	24442953	24502197	+	2113	1	LOC10192	0.989	0.895
NM_0011{	chrX	18989307	19122637	-	4711	1	ADGRG2|(	0.03	0.052
NR_03166	chr11	90869121	90869202	-	81	1	MIR1261|I	0	0
NR_04701	chr13	1.01E+08	1.01E+08	-	407	1	LINC00411	0	0
NR_11085	chr17	79915252	79926725	-	5265	1	LINC0197!	0.34	0.229
NR_03750	chr14	47760995	47761104	-	109	1	MIR548Y|-	0	0
NM_0039:	chr3	40457292	40462372	+	849	1	RPL14|CA(	163.424	157.604
NM_0011‹	chr17	41105389	41106488	+	1097	2	KRTAP4-9|	0	0
NM_0010‹	chr15	73443158	73560012	+	917	1	REC114|C:	0	0.03
NM_0149:	chr7	98106885	98209632	+	8929	1	LMTK2|AA	7.98	10.204
NM_0010(	chr11	56612555	56613502	-	946	1	OR5M1|O	0	0
NM_0012!	chrX	21940573	21994837	+	1669	1	SMS|MRSI	28.19	28.765
NM_0012(	chr19	42924132	42937178	-	1665	1	PSG7|PSB(	0	0

NM_0207	chr2	19910266	19990123	-	6884	1	WDR35|CI	2.216	2.469
NM_0007	chr11	15073600	15078631	+	1024	1	CALCB|CA	1.423	1.097
NM_0144	chr7	6022247	6059229	-	4448	1	EIF2AK1|H	48.582	48.389
NM_0307	chrX	81113701	81201942	-	2117	1	HMGN5|N	0.238	0.582
NM_0809	chr17	7101322	7114310	-	1192	1	ASGR2|AS	0.097	0.149
NM_0013	chr3	1.41E+08	1.42E+08	+	5622	1	RASA2|GA	1.342	1.956
NR_03408	chr9	1.12E+08	1.12E+08	-	1092	1	LRRC37A5	0	0
NM_2034	chr17	63774189	63819317	+	3920	1	DDX42|DD	39.733	41.996
NM_0069	chr2	2.34E+08	2.34E+08	+	1009	1	SPP2|SPP-	0	0
NM_0021	chr2	1.97E+08	1.98E+08	+	947	1	HSPE1|CPI	31.955	26.179
NM_0010	chr10	79555852	79560461	-	2247	1	SFTPA2|C	0.052	0.189
NM_0013	chr12	8056844	8066471	-	3539	1	C3AR1|AZ	0	0.012
NM_0015	chr3	10164879	10243743	+	3454	1	IRAK2|IRA	0.605	1.162
NR_00128	chr7	1614470	1616692	+	2222	1	TFAMP1|N	0.122	0.154
NR_03022	chr20	34990376	34990497	+	121	1	MIR499A|	0.852	1.019
NM_0010	chr11	4848237	4849208	-	970	1	OR51S1|O	0	0
NM_0014	chr17	76136334	76141299	-	2635	1	FOXJ1|FKH	6.053	3.197
NM_1451	chr1	1.6E+08	1.6E+08	-	4319	1	PIGM|GPI-	3.105	2.727
NM_0528	chr18	56651385	57029805	+	7154	1	WDR7|TRA	7.748	7.323
NM_0013	chr1	10449719	10452003	+	1227	1	CORT|CST-	1.986	1.753
NM_0011	chr6	1.31E+08	1.31E+08	+	2510	1	SMLR1|-|-	0	0
NM_0010	chrX	1.2E+08	1.2E+08	+	464	1	AKAP14|A	3.862	6.819
NM_0163	chr22	24495283	24526585	+	2055	1	UPB1|BUP	0.119	0.26
NM_0023	chr2	66435400	66572759	+	3166	1	MEIS1|-|-	28.3	15.843
NR_12037	chr15	63390230	63437536	+	1197	1	LOC10272	0.022	0.114
NR_03034	chr11	65444458	65444557	+	99	1	MIR612|M	0	0
NM_0012	chr5	1.3E+08	1.3E+08	+	569	1	KIAA1024L	1.11	1.083
NM_0221	chr15	88459478	88467402	-	994	1	MRPL46|C	26.436	26.567
NM_0309	chr10	1.01E+08	1.01E+08	+	3076	1	SFXN3|BA	48.996	46.457
NR_10688	chr5	1.32E+08	1.32E+08	-	69	1	MIR6830|	0.747	1.985
NR_12210	chr6	4610565	4611919	+	1188	1	KU-MEL-3	0	0
NM_2073	chr3	30726200	30894661	-	3638	1	GADL1|AD	0.028	0
NR_13511	chr9	1.33E+08	1.33E+08	-	551	1	LOC10537	0.983	0.82
NM_0011	chr5	1.26E+08	1.26E+08	+	2580	1	GRAMD3|	0.07	0.345
NM_0191	chr16	5071809	5087379	+	3919	1	ALG1|CDG	4.708	4.802
NM_0012	chr11	62662817	62665216	-	646	1	C11orf98|	89.416	75.673
NM_0184	chr16	81006498	81022882	+	1440	1	CENPN|BN	5.05	4.566
NR_03991	chr21	26953961	26954043	+	82	1	MIR4759|	0	0
NM_0012	chr5	10971841	11904043	-	4892	1	CTNND2|C	69.196	52.985
NM_0011	chr1	16985958	17011972	-	3709	1	ATP13A2|	51.911	52.679
NM_0011	chr18	48919853	48949333	-	2286	1	SMAD7|CF	4.461	2.373
NM_0012	chr7	50458436	50561071	-	1795	1	DDC|AADC	3.584	1.221
NM_0012	chr9	1.36E+08	1.36E+08	-	8952	1	SEC16A|KI	23.326	24.629
NM_0017	chr7	1.35E+08	1.35E+08	+	1779	1	BPGM|DP	27.425	25.038
NR_12586	chr6	43995724	44003650	-	2011	1	LOC10192	0.5	0.627
NM_1384	chr22	39994949	40030039	+	2064	1	FAM83F|-	0.05	0
NM_1814	chr2	2.22E+08	2.22E+08	-	2022	1	PAX3|CDH	14.749	12.017
NR_02799	chr15	20669468	20756151	-	545	1	NBEAP1|B	0	0.126
NM_0182	chr11	66590169	66593083	-	2912	1	CCDC87|-|	0.398	0.296
NM_0000	chr8	85463902	85481492	+	1657	1	CA2|CA-II|	3.649	1.405

NR_00361	chr7	1E+08	1E+08	-	1511	1	PMS2P1|P	7.995	7.769
NR_03612	chr13	41101019	41101100	-	81	1	MIR3168|-	0	0
NR_11065	chrX	1.09E+08	1.09E+08	+	1139	1	LOC10192	0.034	0.096
NR_02777	chr7	74890768	74906225	+	1323	1	PMS2P5|P	0.965	1.18
NM_0012	chr19	1576671	1592761	-	2394	1	MBD3|-|-|	60.638	56.899
NR_13511	chr1	19210386	19240704	+	620	1	LOC10192	5.365	6.252
NM_0003	chrX	48683797	48691426	+	1816	1	WAS|IMD	1.612	1.508
NR_00273	chrX	1.37E+08	1.37E+08	-	72	1	SNORD61|	0	0
NM_0011	chr1	2.31E+08	2.31E+08	-	3537	1	C1orf198|	17.315	19.002
NM_0013	chr3	50569152	50584990	+	5873	1	HEMK1|HI	6.553	6.003
NM_0243	chr16	30104810	30113557	-	1086	1	GDPD3|GI	4.06	3.431
NM_1333	chr6	52362128	52407777	+	4734	1	PAQR8|C6	4.145	3.371
NM_0010	chr7	1.44E+08	1.44E+08	-	931	1	OR2A7|HS	0	0
NR_02807	chr6	28378821	28399767	-	4337	1	ZSCAN12|	4.602	3.543
NM_0011	chr12	1.14E+08	1.14E+08	-	3608	1	RBM19|-|	8.062	9.643
NM_0055	chr13	1.13E+08	1.13E+08	+	2529	1	LAMP1|CD	59.41	62.262
NR_12572	chr13	97172382	97179622	-	777	1	LINC0045	0	0
NM_0011	chr9	1E+08	1.01E+08	+	2422	1	MSANTD3	56.014	60.771
NM_1449	chr19	12525370	12551542	-	2942	1	ZNF564|-|	2.178	2.542
NM_0061	chr11	1.25E+08	1.25E+08	+	1209	1	NRGN|RC	45.335	182.559
NR_03616	chr20	31607186	31607240	+	54	1	MIR3193|	0	0
NM_0011	chr10	19048771	19734478	+	6838	1	MALRD1|	0.1	0.07
NR_03992	chr22	33436582	33436669	-	87	1	MIR4764|	0	0
NM_0156	chr4	83263824	83284914	-	1633	1	COQ2|CL6	3.174	6.702
NR_02428	chr8	9900064	9903329	-	2919	1	LINC0059	20.495	19.877
NM_0807	chr6	24126186	24147529	+	2402	1	NRSN1|VN	18.707	28.751
NR_10700	chr16	81533902	81533966	+	64	1	MIR7854|	0	0
NR_11072	chr19	41535498	41536904	+	475	2	LINC0148	0	0
NM_0011	chr7	23680114	23702650	+	1199	1	FAM221A|	4.624	3.61
NM_0325	chr6	99271161	99349655	-	11508	1	FAXC|C6o	5.222	4.88
NM_0247	chr1	15571699	15585110	-	3145	1	AGMAT|-|	0.115	0.148
NM_0221	chr10	1.24E+08	1.25E+08	+	1676	1	LHPP|HDH	7.993	16.034
NR_10698	chr11	1.04E+08	1.04E+08	+	60	1	MIR7641-1	0	0
NR_10695	chrX	53198889	53198945	-	56	1	MIR6894|	2.533	4.403
NM_0010	chr11	60396014	60417755	+	3295	1	MS4A14|N	0	0.004
NM_0046	chr3	52401004	52410105	-	3682	1	BAP1|HUC	44.861	48.04
NR_03410	chr16	54918863	54928778	-	919	1	CRNDE|CR	21.438	18.243
NR_11091	chr16	51762519	51772646	+	434	1	LINC01571	0.059	0.189
NM_0241	chr17	44186986	44191731	+	1917	1	TMUB2|FF	31.188	31.595
NM_0011	chr17	42844583	42850712	+	2592	1	AOC2|DAC	2.766	3.99
NM_0011	chr4	1.56E+08	1.56E+08	+	2679	1	GUCY1A3|	2.801	0.649
NM_0321	chr5	1.27E+08	1.27E+08	+	3634	1	PHAX|RNU	8.92	9.027
NR_03998	chrX	1.04E+08	1.04E+08	-	2317	1	LOC28643	0.506	0.502
NM_0013	chr19	57719747	57727624	-	2303	1	ZNF671|-|	1.775	5.388
NM_1985	chrX	54748899	54798240	-	4953	1	ITIH6|ITIH	0	0.003
NM_0012	chr1	36095236	36125084	-	4383	1	COL8A2|FI	0.141	0.309
NM_0039	chr3	1.8E+08	1.8E+08	+	7910	1	USP13|ISC	5.969	3.139
NR_10682	chr14	1E+08	1E+08	+	60	1	MIR6764|	7.306	4.566
NM_0150	chr13	45962179	46052778	-	6409	1	ZC3H13|KI	8.091	8.743
NM_0327	chr2	74472832	74483230	-	4328	1	CCDC142|	3.649	3.421

NR_10275	chr15_KI2	4751339	4795189	+	1231	2	LOC10099	0	0
NM_0142	chr17	27472010	27623692	+	4468	1	KSR1|KSR|	11.961	14.681
NR_01536	chr5	1.28E+08	1.28E+08	-	2951	1	LINC01184	0.454	0.594
NM_0123	chr2	11658178	11670203	-	1602	1	NTSR2|NT	0	0
NR_00144	chr10	64825528	64826877	+	1349	1	ANXA2P3|	0.067	0.234
NM_0247	chr5	795605	850986	-	2591	1	ZDHHC11|	2.08	1.269
NM_0011	chr12	6870936	6873285	-	1199	1	SPSB2|GR	6.183	6.694
NM_0012	chrX	1336616	1382689	+	1464	2	IL3RA|CD1	0	0
NM_0015	chr13	49220338	49222377	+	1236	1	MLNR|GPI	0.094	0.166
NM_1449	chrX	75368427	75523502	-	6029	1	ZDHHC15|	1.982	2.715
NM_0163	chr15	44427381	44527231	+	4859	1	CTDSPL2|	4.559	6.591
NM_0014	chrX	49560904	49568208	+	495	3	GAGE12I|	0	0
NM_0012	chr7	92560758	92590070	-	2442	1	FAM133B|	0.269	0.365
NM_0330	chr20	297563	300319	+	2754	1	ZCCHC3|C	23.928	21.973
NR_00156	chr3	1.7E+08	1.7E+08	-	450	1	TERC|DKC	0	0.091
NR_12163	chr1	63320884	63323675	-	586	1	FOXD3-AS	0.418	0.795
NM_0010	chr2	2.19E+08	2.19E+08	-	787	1	RESP18|-|	0.18	0.453
NM_0157	chr22	31134807	31140607	-	2690	1	PLA2G3|G	0.417	0.601
NR_12636	chr13	93045341	93057926	+	307	1	LINC00363	0	0
NR_10373	chr2	95807052	95826981	-	3125	1	LINC00342	0.64	1.381
NR_02764	chr15	50944129	50946565	+	2436	1	DCAF13P3	0.423	0.287
NM_1991	chr20	57648392	57710614	-	4558	1	PMEPA1|S	7.553	12.21
NM_1524	chr15	37934607	37951422	+	1231	1	TMCO5A|	0	0
NR_00001	chr19	49490907	49490972	+	65	1	SNORD34|	5.157	8.218
NM_0178	chr1	53214100	53220639	-	1096	1	C1orf123|	27.587	27.069
NM_0029	chr7	75811796	75813715	-	356	1	CCL24|Ckb	0.145	0
NR_03973	chr15	65719246	65719332	-	86	1	MIR4511|	0	0
NM_0323	chr19	34404398	34426167	+	1148	1	PDCD2L|-|	16.948	15.737
NM_0149	chr10	1.05E+08	1.05E+08	+	5735	1	SORCS3|S	3.746	2.498
NR_04665	chrX	24146225	24149654	-	414	1	ZFX-AS1|-	0	0.033
NR_01536	chr20	38420620	38435319	-	1037	1	SNHG17|-	15.194	14.767
NR_12573	chrY	18872501	19076003	-	720	1	TTTY14|CY	3.958	2.188
NM_0121	chr4	1.74E+08	1.74E+08	-	2555	1	FBXO8|DC	4.754	4.61
NM_0230	chr8	1.45E+08	1.45E+08	+	2610	1	C8orf33|-|	39.026	39.422
NM_0013	chr11	7665095	7672891	-	1162	1	CYB5R2|B	0.178	0.471
NR_12593	chr1	76011324	76019356	+	366	1	LOC10192	0	0.037
NM_0011	chr5	1.41E+08	1.41E+08	-	1522	1	CD14|-|-|	0.957	0.657
NM_0012	chr2	75701783	75710985	-	1160	1	GCFC2|C2	0.178	1.901
NR_00310	chr17	43125557	43145228	+	1507	1	NBR2|NCF	2.028	1.854
NM_0010	chr9	1.37E+08	1.37E+08	-	2618	1	NRARP|-|-	8.23	7.403
NM_0011	chr15	40278372	40282586	-	4213	1	ANKRD63|	0.233	0.156
NR_00330	chr15	25185631	25185712	+	80	3	SNORD115	0	0.171
NR_03171	chr1	2.36E+08	2.36E+08	-	60	1	MIR1537|	0	0
NM_0012	chr6	1.43E+08	1.43E+08	+	1402	1	AIG1|AIG-	23.304	26.915
NM_0011	chr11	1.19E+08	1.19E+08	-	2037	1	SLC37A4|	20.919	18.881
NR_00333	chr15	25088803	25088896	+	93	1	SNORD116	1.941	1.767
NM_0246	chr6	1.47E+08	1.47E+08	+	5251	1	ADGB|C6c	0.005	0.044
NR_03978	chr6_GL0C	2146051	2146140	+	89	6	MIR4640|	0	0
NR_11076	chr18	6256744	6260934	+	2045	1	L3MBTL4-A	0	0
NR_12060	chr5	90158339	90290071	-	567	1	LINC01339	0	0

NR_10374	chr9	33677268	33688010	+	868	1	PTENP1-A	0.906	0.41
NM_0306	chr11	62646848	62653302	-	3281	1	INTS5|INT	12.776	14.737
NR_03970	chr11	47400970	47401042	+	72	1	MIR4487|	0	0
NM_1527	chr12	1.06E+08	1.06E+08	+	2218	1	TCP11L2|-	3.058	4.138
NM_0010	chrX	1.02E+08	1.02E+08	-	1527	1	TCEAL6|T	0.034	0.027
NM_0011	chr19	17933015	17943985	+	980	1	CCDC124|	79.966	76.284
NM_0011	chr10	13317438	13348298	-	2979	1	SEPHS1|SE	20.675	20.649
NM_0012	chrY	9337510	9340278	+	1106.5	2	TSPY8|-|-|	0	0
NM_0045	chr6	44258166	44265788	-	2573	1	NFKBIE|IK	7.311	9.039
NM_0003	chr3	93873037	93974090	-	3563	1	PROS1|PR	2.497	1.468
NM_0328	chr12	1.1E+08	1.1E+08	+	3135	1	FAM222A|	9.073	11.521
NR_03162	chr5	1.54E+08	1.54E+08	+	141	1	MIR1294|	0	0
NM_0010	chr20	33217310	33227806	+	1065	1	BPIFA3|C2	0	0
NM_0013	chr5	13690329	13944480	-	15491	1	DNAH5|C	0.102	0.132
NR_13556	chr2	1.03E+08	1.03E+08	-	705	1	LINC0179	0	0
NR_04003	chr18	24628182	24662198	+	2318	1	LINC0191	0.033	0.065
NM_0309	chr11	9778667	10294207	-	7397	1	SBF2|CMT	8.999	12.362
NM_0012	chr8	1.23E+08	1.23E+08	-	1519	1	ZHX1-C8o	9.261	14.869
NM_0181	chr12	31959419	31993107	+	6222	1	KIAA1551|	1.245	2.457
NR_11049	chr2	6649107	6650519	-	687	1	MIR7515H	0	0.04
NM_0206	chr20	58309715	58367507	+	8642	1	RAB22A|-|	4.061	3.931
NM_0014	chr19	18868546	18896144	-	2554	1	GDF1|DO	38.408	32.473
NM_0012	chr22	20507542	20587632	+	3081	1	MED15|A	23.142	24.055
NM_0013	chr9	96816271	96854488	-	4190	1	ZNF782|-|	0.637	0.889
NM_0003	chr6	42696595	42722620	-	3022	1	PRPH2|AC	0.004	0.018
NM_0011	chr17	78146353	78166283	+	1083	1	C17orf99|	0	0
NM_0011	chr9	1.29E+08	1.29E+08	+	7848	1	SPTAN1|E	181.559	271.68
NM_0035	chr2	2.02E+08	2.02E+08	+	3848	1	FZD7|FzE3	3.089	7.941
NM_2058	chr5	1.46E+08	1.46E+08	-	4835	1	PRELID2|-	0.005	0.173
NM_0006	chr9	1.14E+08	1.14E+08	+	836	1	ORM2|AG	0	0.18
NM_0010	chr6	73368557	73369792	-	673	1	OOEP|C6o	0	0
NM_0051	chr22	50523568	50525605	-	987	1	SCO2|CE	22.652	20.081
NM_1526	chr5	1.13E+08	1.13E+08	+	10185	1	DCP2|NU	2.433	3.398
NM_1471	chr8	57994554	58149718	+	4361	1	FAM110B|	12.988	18.612
NM_0032	chr14	75958061	75982022	-	3485	1	TGFB3|AR	3.348	5.239
NM_0033	chr5	1.4E+08	1.4E+08	+	2693	1	UBE2D2|E	50.142	58.104
NM_0017	chr1	20588951	20618907	+	953	1	CDA|CDD|	0	0
NM_0010	chr1	32539145	32605950	+	7361	1	ZBTB8A|B	2.986	2.903
NM_0248	chr9	5784572	5833081	-	5324	1	ERMP1|FX	1.882	2.318
NR_13365	chr3	1.84E+08	1.84E+08	-	512	1	HTR3E-AS	0	0
NM_0012	chr11	89911472	89917710	-	1466.5	2	TRIM49D1	0	0
NR_12638	chr13	1.1E+08	1.1E+08	+	435	1	LINC0039	0	0
NM_0060	chr9	1.31E+08	1.31E+08	+	6068	1	LAMC3|O	0.155	0.023
NM_0028	chr1	2.03E+08	2.03E+08	-	3223	1	RABIF|MS	9.453	10.208
NR_04641	chr4	9358382	9359974	+	1592	1	USP17L9P	0	0
NM_0182	chr17	55719627	55722864	-	1751	1	TMEM100	2.356	4.154
NR_10685	chr19	15179283	15179350	-	67	1	MIR6795|	3.464	2.044
NR_13657	chrX	71662991	71666868	-	1810	1	LOC10012	0	0
NM_0056	chr1	1.53E+08	1.53E+08	-	461	1	S100A12|	0	0
NR_02733	chr16	90042902	90047783	-	1387	1	URAHP|U	3.969	4.009

NM_0013	chr10	11018498	11331455	+	2533	1	CELF2|BRU	17.638	28.027
NR_00218	chr20	53874501	53875709	-	1208	1	SUMO1P1	0	0
NR_03977	chr5	82840155	82840224	+	69	1	MIR3977|	0	0
NM_0010	chr15	34525283	34536021	-	4252	1	GOLGA8B|	1.295	2.567
NR_03614	chr16_KI2	875612	875705	-	93	7	MIR3180-1	0	0
NR_11017	chr3	1.37E+08	1.37E+08	-	967	1	NCK1-AS1	2.867	1.544
NR_10992	chr21	18561265	18759812	-	658	1	MIR548XH	0.02	0.167
NM_0152	chr18	8717371	8832777	+	6073	1	MTCL1|CC	9.027	6.414
NM_0012	chr19	39997036	40021041	+	7940	1	ZNF546|ZN	0.461	0.621
NR_10684	chr17	75498548	75498628	+	80	1	MIR6785|	2.74	4.28
NM_0808	chrX	91434598	91438584	+	3443	1	PABPC5|P	1.247	1.07
NR_04752	chr9	62376256	62435519	+	2221	1	FGF7P6|K	0	0
NR_13275	chr1	8494800	8494913	-	113	1	SNORD128	0	0
NM_0068	chr12	95474046	95515837	+	3491	1	METAP2|M	13.13	11.837
NR_03964	chr2	1.77E+08	1.77E+08	+	72	2	MIR4444-1	2.149	0.38
NR_02433	chr1	2.1E+08	2.1E+08	-	798	1	SERTAD4-A	2.44	1.699
NM_0016	chrX	2903970	2929375	-	5171	1	ARSD|ASD	3.421	2.289
NM_0012	chr6	31115088	31120475	-	2576.5	6	CDSN|HTS	0	0
NR_13505	chr1	19591802	19596832	-	205	1	LOC10537	0	0
NM_0012	chr1	7784429	7845180	+	6240	1	PER3|FASP	1.692	2.443
NM_0013	chr2	61177418	61188925	+	3107	1	AHSA2|AH	9.105	11.378
NM_0011	chr7	1.03E+08	1.03E+08	+	2058.5	2	ARMC10|P	1.434	1.105
NM_0146	chr1	30931506	31065717	-	5355	1	PUM1|HSP	35.577	36.264
NR_12207	chr11	45371397	45388508	+	2592	1	LOC39988	0.06	0.069
NR_02776	chr3	1.94E+08	1.94E+08	-	1144	1	DPPA2P3|	0	0
NM_0229	chr2	2.24E+08	2.24E+08	+	1742	1	MRPL44|C	31.197	37.34
NR_10386	chr11	1.25E+08	1.25E+08	+	2903	1	LOC10050	0.635	0.67
NM_1450	chr10	45454585	45594906	-	4822.5	2	MARCH8|	1.299	1.071
NM_0011	chr1	16440672	16460089	+	1993	1	NECAP2|-|	13.469	13.401
NR_00323	chrX	1.04E+08	1.04E+08	+	975	2	H2BFXP|-|	0.463	0.478
NM_0011	chrX	1.15E+08	1.15E+08	+	3466	1	RBMXL3|C	0	0
NM_0019	chr1	70411218	70439851	+	2126	1	CTH|-|-|1	3.584	4.271
NM_0031	chr6	35833034	35921180	-	4344	1	SRPK1|SFR	16.951	15.797
NM_0010	chr10	1.03E+08	1.03E+08	-	3052	1	CALHM1|F	0.017	0
NM_0149	chr1	2.04E+08	2.04E+08	-	7389	1	PLEKHA6|	41.106	18.012
NR_03993	chr2	86193026	86193108	-	82	1	MIR4779|	0	0
NR_04003	chr8	11576535	11581341	+	1924	1	LINC00208	0	0
NM_0039	chr5	1.8E+08	1.8E+08	+	2892	1	SQSTM1|A	67.16	64.133
NR_10694	chr17	40026332	40026409	-	77	1	MIR6884|	14.066	13.697
NM_0010	chr19	46039748	46041016	-	567	1	IGFL4|-|-|	0.182	0.266
NM_0190	chr1	1E+08	1E+08	+	3130	1	TRMT13|C	0.964	1.523
NM_0169	chr7	1.42E+08	1.42E+08	+	1098	1	TAS2R3|T2	0.411	0.112
NM_0012	chr1	1.12E+08	1.12E+08	+	3031	1	LINC0175	0.289	0.425
NM_0013	chr2	1.1E+08	1.1E+08	-	3325	1	SEPT10|-|	3.261	3.18
NM_0013	chr15	99251809	99390732	+	5958	1	LRRC28|-|	4.687	5.129
NR_03665	chr20	49278642	49289258	+	498	1	ZFAS1|C20	249.895	224.268
NM_0011	chr16	1072756	1081454	+	2653	1	SSTR5|SS-	0.039	0.093
NR_07724	chr6	1.5E+08	1.5E+08	+	968	1	RPS18P9|R	7.299	6.735
NR_02673	chr6	10671418	10694606	-	535	1	C6orf52|-|	13.689	11.495
NM_0207	chr1	69760175	70123488	+	6218	1	LRRC7|DE	0.877	3.355

NM_00114	chr19	42297033	42302800	-	851	1	PAFAH1B3	283.066	228.166
NM_20695	chr22	22547696	22559361	-	2829	2	PRAME|CT	0	0
NM_15228	chr8	81732453	81759513	+	1838	1	CHMP4C|S	0	0.007
NM_00100	chr1	2.49E+08	2.49E+08	-	937	1	OR2T10|O	0	0
NM_00130	chr6	1.11E+08	1.11E+08	-	6001	1	CDK19|CD	6.351	10.725
NM_02473	chr8	1.44E+08	1.44E+08	+	1748	2	GSDMD|D	0	0
NM_00737	chr12	1.18E+08	1.18E+08	+	2136	1	RFC5|RFC3	13.521	10.927
NM_00103	chrX	1.01E+08	1.01E+08	+	2376	1	HNRNPH2	51.509	53.451
NR_13458	chr18	37274460	37276400	+	466	1	LOC10537	0.221	0.059
NM_00117	chr17	4967992	4987665	-	4371	1	CAMTA2|-	12.719	15.126
NM_00114	chr4	25914192	25929879	+	1009	1	SMIM20|C	20.407	21.503
NM_01897	chr16	75647737	75657443	+	2173	1	TERF2IP|D	200.113	265.09
NR_10676	chr11	62792702	62792771	-	69	1	MIR6514|I	1.495	1.191
NM_00100	chr11	5820336	5821301	+	964	1	OR52N2|C	0	0
NR_13446	chr8	1.34E+08	1.34E+08	-	542	1	LOC10537	0	0
NM_00101	chr3	1.98E+08	1.98E+08	+	3642	1	FYTTD1|U	20.739	21.332
NR_02444	chr1	1.47E+08	1.47E+08	-	1070	1	LOC72898	0	0
NR_02968	chr16	56858518	56858601	+	83	1	MIR138-2|	0	0
NM_02085	chr4	76435100	76783252	+	11006	1	SHROOM3	2.451	4.462
NM_21359	chr12	1.09E+08	1.09E+08	+	2125	1	ISCU|2310	21.813	24.229
NR_00332	chr15	25067787	25067883	+	96	2	SNORD116	0	0
NM_00129	chr17	38127970	38138868	+	2047	5	TBC1D3E|T	0	0
NM_00116	chr9	15170844	15307360	-	3233	1	TTC39B|C9	0.69	0.907
NR_03166	chr1	68183518	68183610	-	92	1	MIR1262|I	0	0
NM_00130	chr11	1.3E+08	1.3E+08	-	6848	1	ZBTB44|BT	2.005	3.49
NM_00100	chr1	2.47E+08	2.47E+08	-	952	1	OR2B11|-|	0	0
NR_13517	chr16	70188322	70189252	+	461	1	LOC10537	0	0
NR_07303	chr19	38390292	38399883	+	4999	1	SPRED3|Ev	18.405	13.333
NR_13460	chr6	1.16E+08	1.16E+08	-	509	1	LOC10537	0	0
NM_00078	chr4	70993548	71030912	+	2596	1	DCK|-|-|4	12.933	14.13
NR_11065	chr6	46903471	46910027	+	1726	1	LOC10192	0	0
NM_02410	chr19	16646148	16660157	-	1366	1	SMIM7|C1	33.631	29.901
NM_00363	chr10	73801911	73811831	-	3979	1	NDST2|HS	10.502	11.418
NM_00113	chr2	2.38E+08	2.38E+08	+	3961	1	TRAF3IP1|	3.144	2.472
NM_00289	chr14	58298504	58373733	+	5746	1	ARID4A|RI	2.035	3.423
NM_01842	chr6	44127554	44155519	+	3258	1	TMEM63B	43.101	49.113
NM_00110	chr19	18588685	18592337	+	873	1	C19orf60|	33.733	28.885
NM_00225	chr19_KI27	54292	65238	-	1569.6	30	KIR2DL4|C	0	0
NM_13928	chrX	1.2E+08	1.2E+08	-	764	1	RHOXF1|C	0.608	0.645
NM_00131	chr20	17968891	17991122	+	2000	1	MGME1|C	6.95	8.41
NM_00128	chrX	48903612	48911958	-	1710	1	SLC35A2|C	9.463	9.3
NM_01439	chr4	99816824	99870189	+	2926	1	DAPP1|BA	0.004	0
NM_01758	chr9	1.33E+08	1.33E+08	+	2748	1	CACFD1|C	42.35	44.71
NM_03238	chr5	1.55E+08	1.55E+08	-	3019	1	FAXDC2|C	2.648	2.513
NM_01496	chr19	1107634	1174283	-	4836.5	2	SBNO2|KIA	0.016	0
NM_00688	chr2	43222402	43226606	-	3689	1	ZFP36L2|E	2.807	4.344
NM_15274	chr7	1E+08	1E+08	-	2579	1	GPC2|-|-|	201.501	164.226
NM_00103	chr11	67662162	67681214	-	2482	1	ALDH3B2|	0	0
NM_01463	chr22	21919419	21952878	-	5175	1	PPM1F|CA	10.24	10.653
NM_00131	chr20	35459983	35511975	+	7872	1	CEP250|C-	14.024	11.2

NM_1489(	chr1	6461154	6466195	-	1476	1	TNFRSF25	7.329	9.141
NM_0147	chr2	6917392	7044178	+	5728	1	RNF144A|	13.242	21.944
NM_0221(	chr9	22446841	22452473	+	2298	1	DMRTA1|I	0.039	0.924
NR_03984	chr11	74720268	74720337	-	69	1	MIR4696|	0	0
NM_0013	chr2	2.19E+08	2.19E+08	+	2696	1	STK16|KR(	14.347	17.116
NR_13430	chr10	1039420	1056716	-	2713	1	IDI1|IPP1|	30.435	27.328
NM_0139	chr6	31727037	31730265	-	1328	7	DDAH2|DI	0	0.01
NM_0011	chr4	1.85E+08	1.85E+08	-	2777	1	CCDC110|	0.084	0.089
NM_0223	chr8	90791693	90959402	+	5125	1	NECAB1|E	0.73	0.748
NM_0189(	chr5	1.41E+08	1.41E+08	+	6842	1	PCDHA11|	12.69	13.092
NM_0010(	chr19	58387269	58394804	+	733	1	RPS5|S5|-	501.738	388.861
NM_0050	chr2	2.08E+08	2.08E+08	+	2699	1	PTH2R|PTI	0.067	0.421
NR_11055	chr14	75574904	75579588	-	2140	1	LOC10272	0.205	0.166
NR_03064	chr16	2090195	2090284	-	89	1	MIR1225|I	2.753	4.771
NM_0012	chr13	51767993	51804162	-	1189	1	DHRS12|S	3.036	3.156
NM_1818	chrY	13985772	13986508	-	541	2	VCY1B|BP	0	0
NM_1878	chr2	27282429	27307439	+	1924	1	TRIM54|M	0.013	0.107
NM_0012	chrX	64915802	65034744	-	2712	1	ZC4H2|HC	42.793	57.197
NM_0012	chr13	46053187	46105076	-	1643	1	CPB2|CPU	0.031	0.025
NM_1446	chr17	28897781	28903071	-	1914	1	DHRS13|S	42.682	42.014
NM_0067	chr19_GL9	832361	849155	+	1764.9	32	KIR3DL2|3	0	0
NM_0174	chr6	99369472	99394206	-	1256	1	COQ3|DHI	5.985	9.837
NR_12061	chr10	94081950	94108794	-	951	1	PLCE1-AS2	0	0
NM_0065	chrX	37838836	37847636	-	2209	1	DYNLT3|R	0.047	0.732
NM_0013	chr1	1.58E+08	1.58E+08	+	1966	1	CD1D|CD1	0.039	0.007
NR_03788	chr5	1.59E+08	1.59E+08	+	1575	1	LOC28562	0.016	0.009
NM_0030	chr12	50085200	50100711	+	3414	1	SMARCD1	78.94	80.81
NM_1781(	chr17	74924275	74933911	+	2138	1	OTOP2|-|-	0	0.006
NR_04024	chr12	30795681	30802711	+	1890	1	LINC00941	0.02	0.043
NM_0305	chr16	74873573	74985119	-	3635	1	WDR59|CI	16.355	21.109
NM_0147	chr1	65264694	65415869	+	5782	1	DNAJC6|D	19.686	18.091
NM_0052(	chr17	1421353	1456267	-	2856	2	CRK|CRKII	0.971	0.652
NM_0035	chr6	27838662	27839110	+	446	1	HIST1H2BI	0.231	0.706
NM_0529	chr1	27005020	27012842	-	2370	1	FAM46B|-	1.055	1.295
NM_0013	chr19	52031379	52048561	-	4506	1	ZNF432|-|	2.552	3.477
NM_0013(	chr7	34658239	34849978	+	1406	1	NPSR1|AS	0.028	0.049
NM_0037	chr1	44822266	44842944	-	3749	1	PTCH2|PT	0.829	1.787
NR_10803	chr10	49296283	49298893	-	693	1	C10orf71-/	0	0
NM_0011(	chr4	98261376	98443861	+	3470	1	RAP1GDS1	12.254	16.472
NM_0012	chr2	30910465	31129640	-	2496	1	GALNT14|	6.044	2.036
NR_13559	chrX	48506529	48508839	-	646	1	LOC10192	0.24	0.212
NM_0010(	chr1	22563502	22589619	+	1855	1	EPHA8|EEI	4.247	3.714
NR_10700	chr4	1.46E+08	1.46E+08	+	105	1	MIR7849|I	0.123	0
NM_0059(	chr18	47833095	47930599	-	10415	1	SMAD2|JV	5.557	6.477
NR_11027	chr2	2.01E+08	2.01E+08	-	873	1	LOC10192	1.521	1.083
NR_02949	chr17	1713903	1713987	-	84	2	MIR22|MI	0	0
NM_0144(	chr19	43460794	43465679	-	1699	1	LYPD3|C4.	1.723	3.588
NM_0046(	chr15	28111037	28322152	-	15245	1	HERC2|D1	1.455	1.632
NM_0010(	chr22	49960495	49964072	+	2371	1	PIM3|pim-	13.617	15.684
NM_1767	chr11	532242	535567	-	1243	2	HRAS|C-B/	0	0

NM_0161ٰ	chr17	75135243	75154683	-	1603	1	HN1|ARM	465.404	557.16
NR_02969	chr3	49020618	49020709	-	91	1	MIR191|M	0	0
NM_1523	chr1	40531561	40548169	+	2090	1	ZNF684|-|	0.907	0.931
NM_1828	chr14	74239472	74262738	+	2988	1	VSX2|CHX	0.125	0.248
NM_0207	chr3	1.29E+08	1.29E+08	-	1702	1	KIAA1257|	1.47	1.215
NR_13458	chr22	27310651	27317840	+	5434	1	LOC10050	0.133	0.149
NR_00257	chr1	1.74E+08	1.74E+08	-	620	1	GAS5|NCR	87.634	96.697
NM_0323	chr16	55566672	55567680	+	1006	1	CAPNS2|-|	0	0
NR_02685	chr4	39480255	39481903	+	1648	1	LOC40112	1.776	1.82
NM_0013	chr9	1.28E+08	1.28E+08	+	2844	1	TTC16|-|-|	0.159	0.212
NM_0210	chr22	29480192	29491288	+	3713	1	NEFH|CM	1.372	1.206
NR_10696	chr16	16324588	16324654	+	66	7	MIR6511A	0	0
NR_13688	chr15	62242529	62248795	-	2907	1	GOLGA2P1	0.009	0.005
NM_0063	chr4	1721490	1745178	+	2819	1	TACC3|ERI	12.029	8.42
NM_0014	chr3	48636463	48662915	-	11925	1	CELSR3|AI	35.883	38.328
NM_0010	chr1	92218016	92245810	+	743	1	C1orf146|	0	0
NM_0178	chr8	56957929	56993871	-	7223	1	IMPAD1|G	9.638	10.718
NM_2137	chr15	69452820	69455545	+	432	1	RPLP1|LP1	447.481	326.667
NM_0010	chr11	4803433	4804380	-	946	1	OR52R1|C	0	0
NR_02748	chr4	212600	255984	+	2605	1	ZNF876P|-	0.213	0.415
NM_2073	chr2	98793846	98936221	-	3857	1	KIAA1211L	0.348	0.195
NM_0011	chr6	44270743	44279445	+	4614	1	TMEM151	50.129	64.086
NM_0067	chr6	41053201	41064891	+	1629	1	APOBEC2|	0.293	0.16
NR_04982	chr8	1.3E+08	1.3E+08	-	119	1	MIR5194|	0	0
NR_02339	chr8	1.45E+08	1.45E+08	-	5425	1	ZNF252P|	3.211	3.174
NM_0012	chr8	27235323	27258439	-	2252	1	STMN4|RE	168.056	293.853
NR_04514	chr1	63440770	63523273	-	1049	1	ITGB3BP|	4.818	4.27
NM_0013	chr16	2236467	2238711	+	1294	1	DNASE1L2	3.647	3.43
NR_03892	chr11	65569220	65570413	-	1193	1	SSSCA1-AS	2.302	3.571
NM_0013	chr2	2.08E+08	2.08E+08	+	1225	1	CREB1|CR	9.325	10.533
NR_00307	chr9	1.23E+08	1.23E+08	-	106	1	SNORD90|	0	0
NR_10807	chr3	4490317	4493163	-	1430	1	ITPR1-AS1	0.442	0.45
NM_0571	chr6	43014103	43021298	+	2006	1	KLHDC3|P	103.527	131.276
NM_1471	chrX	1.33E+08	1.33E+08	-	4441	1	HS6ST2|-|	3.522	3.828
NR_03789	chr3	1.95E+08	1.95E+08	+	3643	1	LINC01968	0	0
NR_10377	chr1	2.4E+08	2.4E+08	-	890	1	CHRM3-AS	0	0
NR_01539	chr3	1.08E+08	1.08E+08	+	1303	1	LINC00636	0.03	0
NM_1816	chr7	88759368	89337057	+	4678	1	ZNF804B|-	0.019	0.085
NM_1721	chr19	39296325	39298672	+	849	1	IFNL1|IL-2	0	0
NM_0010	chr11	59443169	59444116	+	946	1	OR5A1|OF	0	0.014
NM_0330	chr3	1.25E+08	1.25E+08	-	2865	1	MUC13|DI	0	0.033
NR_04700	chr13	74419173	74435159	+	722	1	LINC00381	0	0.019
NM_0176	chr3	1.84E+08	1.84E+08	+	7318	1	KLHL24|DI	5.753	5.458
NM_0011	chr1	1512530	1534687	+	2314	1	ATAD3A|H	10.464	9.932
NM_0189	chr17	82374277	82375586	+	1307	1	UTS2R|GP	0.039	0.01
NM_0241	chr17	80178508	80209331	+	4146	1	CARD14|B	0.641	0.644
NM_0072	chr16	75566351	75577881	+	996	1	GABARAPL	203.344	228.339
NM_0013	chr7	75493627	75504304	+	3137	1	SPDYE5|-|	0.025	0.061
NM_0016	chr12	89588049	89656067	-	6757	1	ATP2B1|PI	6.097	8.21
NM_0045	chr4	1.52E+08	1.52E+08	-	2218	1	GATB|HSP	15.445	16.674

NM_1784!	chr5	1.27E+08	1.27E+08	-	4189	1	MARCH3|I	0.613	1.207
NR_12166	chr3	9192493	9194453	+	1229	1	SRGAP3-A!	0	0
NM_0013:	chr8	1.41E+08	1.41E+08	+	578	1	LOC10537.	0.112	0.308
NM_0013(	chr6	84034119	84090886	+	2138	1	MRAP2|C6	2.515	1.352
NR_02986	chr14	1.01E+08	1.01E+08	+	67	1	MIR376A1	0	0
NM_0201(	chr1	1.09E+08	1.09E+08	+	2745	1	TMEM167	17.276	19.64
NM_0010{	chrX	1.21E+08	1.21E+08	-	1288.7	10	CT47A6|C	0	0
NM_0012(	chr17	28328325	28335489	-	871	1	IFT20|-|-|	32.878	26.042
NM_0218:	chr20	3146519	3159886	-	2859	1	FASTKD5|(	10.206	11.92
NM_0211(	chr3	1.47E+08	1.47E+08	-	2199	1	PLSCR1|M	0.668	0.847
NM_0174:	chr7	77310751	77416400	-	3218	1	GSAP|PIOI	0.032	0.157
NR_03173	chrX	1.15E+08	1.15E+08	+	79	1	MIR1911|I	0	0
NR_10463	chr5	35938801	35939991	+	391	1	LOC10050(	0	0
NM_0013:	chr8	1.24E+08	1.24E+08	+	2880	1	FAM91A1|	2.44	3.134
NM_1536:	chr3	9703826	9729908	+	2027	1	CPNE9|-|-	2.379	0.588
NR_04621	chr5	38282269	38290986	-	1570	1	EGFLAM-A	0	0
NM_1526:	chr3	1.96E+08	1.97E+08	-	5336	1	RNF168|hI	3.151	2.79
NM_0581{	chr21	46301130	46323899	-	2989	1	C21orf58|	3.537	2.181
NM_0156:	chr7	5898710	5925972	+	1807	1	CCZ1|C7oı	4.246	4.048
NM_0159:	chr1	42846573	42852475	+	1607	1	ZNF691|Zf	10.093	9.776
NR_03668	chr7	32580941	32719168	-	1884	1	DPY19L1P:	2.956	2.595
NM_0055(	chr5	1.19E+08	1.19E+08	+	11432	1	DMXL1|-|-	1.256	2.371
NR_02703	chr6	1.34E+08	1.34E+08	+	3514	1	LINC01312	0	0
NR_02420	chr10	73134524	73168095	-	1814	1	ECD|GCR2	16.668	16.4
NR_10995	chr21	22009158	22098459	-	891	1	LINC01687	0	0.015
NM_0020:	chr11	1.29E+08	1.29E+08	+	3970	1	FLI1|EWSF	0.01	0.059
NM_0010:	chr10	89332479	89340968	+	2453	1	IFIT3|CIG-(	0.247	0.43
NR_03648	chr3	13933056	13936944	+	3372	1	FGD5P1|-|	0.027	0.016
NM_0023(	chr17	27631148	27649560	+	1656	1	LGALS9|HI	0.444	0.405
NM_0019(	chr18	31447769	31478702	+	5533	1	DSG3|CDH	0	0
NM_2068:	chr19	54923509	54947505	-	3238	10	NLRP7|CLI	0	0
NR_02439	chr14	1.05E+08	1.05E+08	+	2517	1	LINC00638	0.717	0.566
NM_0179(	chr7	30010583	30026801	-	5075	1	FKBP14|EI	2.271	2.961
NR_11013	chr14	20995837	20999163	-	1394	1	LOC10192!	7.908	8.922
NM_0133:	chr12	41437739	41574590	+	3233	1	PDZRN4|L	9.998	2.877
NM_1722(	chr6_GL0C	4494116	4508814	-	3492.2	5	TAPBP|NG	0.033	0.043
NM_0024!	chr11	1223065	1262176	+	17865	1	MUC5B|M	0	0
NM_0328!	chr15	40807076	40814569	+	1928	1	ZFYVE19|A	15.91	15.267
NR_02702	chr1	1.56E+08	1.56E+08	+	1963	1	ASH1L-AS1	1.57	1.207
NM_0181:	chr10	1.21E+08	1.21E+08	+	4703	1	WDR11|BI	11.375	9.914
NM_0060:	chr7	75769524	75789746	-	556	1	CCL26|IM/	0	0.049
NM_0176(	chr15	56630176	56734086	-	4952	1	ZNF280D|:	1.14	1.64
NR_10992	chr21	44802241	44804716	+	1364	1	LINC01424	0.255	0.412
NR_03622	chr2	2.07E+08	2.07E+08	-	86	1	MIR2355|ı	0	0
NR_13501	chr12	90293318	90300698	+	876	1	LOC10536!	0	0
NM_0032:	chr16	4257186	4273000	-	2131	1	TFAP4|AP-	5.052	5.11
NM_0151!	chr10	73241956	73247331	-	2362	1	DNAJC9|H	11.562	8.803
NR_02733	chr1	1.21E+08	1.21E+08	-	513	1	HIST2H2B/	0	0.027
NM_0210:	chr6	26250142	26250607	-	463	1	HIST1H3F|	0	0.089
NM_0325!	chr12	1.21E+08	1.22E+08	-	5282	1	KDM2B|C)	18.903	23.709

NM_0011:	chr6_GL0C	2156133	2167060	-	2717.5	8	PPP1R18|I	0.019	0.03
NM_0005{	chr5	40909252	40984640	+	5938	1	C7|-|-|5p:	0.276	0.009
NM_0207(	chrX	1.1E+08	1.1E+08	+	5420	1	RGAG1|M.	0.754	1.418
NR_12118	chr9	72871729	72874109	-	578	1	LINC01474	0	0
NR_04983	chr15	80581103	80581239	+	136	1	MIR5572|-	0.284	0.302
NR_02689	chr15	62637172	62645181	-	2312	1	MGC1588!	0	0
NM_1452(	chr13	37103260	37105664	-	2402	1	CSNK1A1L	0.929	1.101
NM_0011{	chr3	1.42E+08	1.43E+08	-	8202	1	ATR|FCTC!	1.038	1.825
NM_0062!	chr4	81087370	81215117	-	4801	1	PRKG2|PR	0.459	0.345
NM_0063{	chr17	15588663	15619704	-	2749	1	CDRT1|C1	0.061	0.04
NM_1523	chr17	44034635	44067619	-	2476	1	LSM12|PN	0.479	0.459
NM_0026{	chr11	65261852	65298685	+	3634	1	POLA2|-|-	6.145	4.926
NM_1529!	chr1	76074704	76630984	+	3252	1	ST6GALNA	5.84	5.716
NM_0012	chr3	49887003	49903637	-	4367	1	MST1R|CD	0.484	0.677
NR_10270	chr10	1.26E+08	1.26E+08	-	442	1	FANK1-AS:	0	0
NM_0002:	chr17	47253842	47312711	+	4877	1	ITGB3|BDI	0.095	0.393
NM_0012{	chr12	6534659	6538375	+	1377	1	GAPDH|G:	1856.998	959.835
NM_0006:	chr14	96204798	96244329	+	4192	1	BDKRB2|B	0.092	0.294
NR_03408	chr3	1.96E+08	1.96E+08	+	1825	1	LINC00885	0.014	0
NM_0012:	chr1	2.44E+08	2.44E+08	+	395	1	C1orf100|	0.065	0.035
NM_0010:	chr8	1.24E+08	1.24E+08	+	6008	1	FER1L6|C8	0.176	0.075
NM_0010(	chr21	39296437	39313786	-	2632	1	BRWD1|C:	26.296	25.24
NM_0010:	chr5	1.15E+08	1.15E+08	-	902	1	TRIM36|H	20.913	11.526
NM_0012	chr16_KI2	8557	203127	-	2920	2	PARN|DAN	0	0
NM_0035:	chr6	26124145	26124690	+	543	1	HIST1H2A(	8.881	10.191
NR_02422	chr19	47923779	47923899	+	120	10	SNAR-A11	0	0
NR_10242	chr6	1.59E+08	1.59E+08	+	346	1	EZR-AS1|-	3.689	3.8
NM_0240:	chr12	1.32E+08	1.32E+08	+	1668	1	NOC4L|NE	17.856	17.581
NR_03969	chr9	1.35E+08	1.35E+08	-	71	1	MIR3689E	0	0
NM_0010:	chr8	58411264	58451501	+	5058	1	UBXN2B|p	9.455	11.926
NR_03611	chr11	46451805	46451889	-	84	1	MIR3160-:	0	0
NM_0011!	chr6_GL0C	3049386	3051610	+	1455	7	LTA|LT|TN	0	0
NM_1307!	chr7	1.51E+08	1.51E+08	+	4411	1	GIMAP1|H	0	0
NM_0159(	chr7	55951918	55955340	+	580	1	MRPS17|H	30.5	33.51
NM_0012	chr6	37170146	37175428	+	2701	1	PIM1|PIM	8.464	8.134
NM_0155	chr7	99416739	99438839	-	5495	1	PTCD1|-|-	14.482	14.652
NM_0012!	chr3	1.96E+08	1.96E+08	-	3463	1	PPP1R2|IP	7.16	6.206
NR_10435	chr2	2.06E+08	2.06E+08	+	3702	1	GPR1-AS|(	0.007	0.004
NR_00258	chr3	1.87E+08	1.87E+08	+	134	1	SNORA63|	4.907	5.315
NM_0011(	chr17	10521148	10549623	-	6044	1	MYH2|IBM	0	0
NM_0528!	chr19	12954156	12957254	-	759	1	GADD45GI	76.698	60.689
NR_10384	chr13	21303512	21344859	-	585	1	LINC0053!	0.264	0.304
NR_12033	chr15	41921417	41929286	+	1170	1	EHD4-AS1	0.022	0.023
NM_0248(	chr1	26159020	26162628	+	1520	1	FAM110D|	0.119	0.225
NM_0321!	chr6	1.11E+08	1.11E+08	+	3680	1	RPF2|BXD	2.771	2.159
NM_1446!	chr8	51319577	51809445	-	4782	1	PXDNL|PN	0.221	0.475
NM_1706:	chr6	25726909	25727345	+	434	1	HIST1H2B/	0	0
NM_1532(	chr1	2.1E+08	2.1E+08	+	4995	1	SYT14|SCA	1.285	1.78
NM_0070(	chr19	35666516	35678485	+	1260	1	UPK1A|TS	0.072	0.022
NM_0012{	chr1	3772761	3775982	+	514	1	SMIM1|Ve	2.157	0.373

NM_0240:	chr17	48621157	48626473	-	2696	1	HOXB9|H(	0.01	0.066
NM_0004(	chr2	2.34E+08	2.34E+08	+	2350	1	UGT1A1|B	0.033	0
NM_0060{	chr1	2.2E+08	2.2E+08	-	2454	1	BPNT1|HE	12.715	13.033
NR_12541	chr8	1.11E+08	1.11E+08	-	452	1	LINC0160S	0	0
NM_0011(	chrX	1.49E+08	1.49E+08	+	13619	1	AFF2|FMR	6.454	7.624
NR_10683	chr17	38914979	38915042	+	63	1	MIR6779|l	4.093	4.783
NR_00328	chr21	8212572	8212727	+	155	8	RNA5-8S5|	0	0
NM_0010{	chr19	54816464	54867095	+	1604.3	33	KIR3DS1|(	0	0
NM_0013:	chr19	8065402	8148869	-	9021	1	FBN3|-|-|:	3.718	4.344
NM_1530	chr14	1.04E+08	1.04E+08	+	4726	1	TDRD9|C1	0.008	0.087
NM_0056!	chr16	69355561	69385988	-	2971	1	TERF2|TRE	27.184	33.171
NM_0010	chr11	62665307	62667451	+	1377	1	METTL12|	2.968	2.556
NM_0012	chr2	2.27E+08	2.27E+08	+	1620	1	MFF|C2or	40.829	50.89
NM_0051!	chrX	73447254	73454585	+	851	1	CDX4|-|-|:	0	0
NM_0013(	chr1	33082178	33120531	+	1853	1	AZIN2|AD(	5.316	5.78
NM_0012!	chr5	1.53E+08	1.54E+08	+	5301	1	GRIA1|GLU	21.012	60.253
NR_10702	chr5	1.73E+08	1.73E+08	+	81	1	MIR8056|l	0	0
NM_0013:	chrX	1.07E+08	1.07E+08	-	5199	1	RBM41|-|-	0.811	1.207
NM_0010(	chr15	1.02E+08	1.02E+08	+	937	1	OR4F6|OR	0	0
NM_0011:	chr16	1444933	1475084	-	4128	1	CLCN7|CL(	21.155	25.393
NR_13841	chrX	63670196	63671502	-	398	1	ARHGEF9-l	0	0
NR_02704	chr13	51813347	51845150	-	3980	1	LINC00282	0.279	0.616
NR_03990	chr19	49933064	49933137	+	73	1	MIR4751|-	18.545	36.213
NR_11055	chr14	77069180	77076191	-	1814	1	LOC10272	0.05	0.038
NM_0010(	chrX	69616067	70039471	+	5264	1	EDA|ECTD	1.002	2.087
NM_1818(	chr11	31262624	31369810	-	1731	1	DCDC1|-|-	0.253	0.135
NR_11020	chr2	96919106	96925837	+	2257	1	LOC10192	0.017	0.006
NM_0571(	chrX	87517712	87669596	+	2534	1	KLHL4|DKI	2.32	0.805
NR_04651	chr13	29872613	29888405	-	911	1	LINC00297	0	0
NM_0069:	chr6_GL0(	3206579	3217530	+	4140	6	SKIV2L|17(	0	0.003
NM_0050	chr9	81583683	81689535	-	4101	1	TLE1|ESG|	37.938	40.54
NM_0010:	chr12	31791185	31792241	-	1054	1	H3F3C|H3	20.392	18.934
NM_0048	chr6	1.31E+08	1.31E+08	+	2460	1	AKAP7|AK	4.083	3.987
NR_12207	chr2	1.11E+08	1.11E+08	-	3208	1	ACOXL-AS:	0.068	0.043
NM_0245!	chr17	80991841	81000133	+	1654	1	CHMP6|VI	24.462	25.829
NM_0151(	chr3	1.3E+08	1.3E+08	-	6939	1	PLXND1|P	1.674	3.375
NR_03408	chr16	70755060	70773251	+	2221	1	VAC14-AS:	0.615	0.66
NR_13457	chr20	43894718	43895471	+	527	1	LINC01728	0	0
NM_0168	chr17_GL(	760288	893837	+	5531	3	MAPT|DDI	0.105	0.134
NR_02707	chr16	56092987	56191094	-	2673	1	LOC28385	0.834	0.984
NM_2033	chr3	49803254	49805030	+	1036	1	FAM212A|	3.472	4.469
NM_0012!	chr12	1.33E+08	1.33E+08	+	3081	1	ZNF26|HEI	2.879	3.668
NM_0013:	chr19	45733251	45764539	+	2737	1	BHMG1|H	0.009	0.035
NM_0011!	chr1	2.01E+08	2.01E+08	-	1910	1	CSRP1|CRI	1.526	3.098
NR_00255	chr11	62853904	62853968	-	64	1	SNORD29|	12.692	15.195
NM_0012(	chr9	1.15E+08	1.15E+08	-	6393	1	TNFSF15|T	0	0
NR_03718	chr6_GL0(	838422	842584	+	1876	8	LINC01015	0	0
NM_0124	chr20	57329775	57343993	+	1802	1	SPO11|CT:	0	0
NR_03888	chr2	85815130	85826788	+	3105	1	LOC28495	0.062	0.013
NM_0120	chr10	22316383	22320317	+	932	1	COMMD3|	45.058	69.382

NM_0012!	chr4	68815993	68832017	+	1633	4	UGT2B10|	0	0
NM_0246(	chr4	1.48E+08	1.48E+08	+	3031	1	ARHGAP1(	0.306	0.682
NR_03402	chr7	7075770	7096786	-	21016	1	LOC10013	0.001	0
NM_0012{	chr10	63133247	63155031	+	1811	1	NRBF2|CO	6.059	4.848
NM_0241.	chr2	1.2E+08	1.2E+08	-	5935	1	TMEM185	3.485	4.44
NM_1774(	chr1	2.03E+08	2.03E+08	-	7592	1	SYT2|CMS	4.669	4.269
NM_0182(	chr2	25739384	25878443	-	7160	1	ASXL2|AS)	1.974	2.426
NM_0038.	chr8	89757747	89791064	+	2572	1	RIPK2|CAF	1.96	2.114
NM_0189!	chr22	37639677	37656043	+	2556	1	SH3BP1|A	1.372	2.899
NR_07354	chr14	1.02E+08	1.02E+08	-	1151	1	MOK|RAG	10.989	6.831
NM_0204(	chr22	50217689	50244971	-	5992	1	TUBGCP6|	12.689	13.5
NR_12597	chr1	39565070	39573203	+	637	1	LOC10192!	16.456	13.633
NR_00295	chr12	48667457	48667593	-	136	1	SNORA2B|	0.095	0.403
NR_10240	chr16	31201885	31203452	+	1093	1	PYCARD-A!	0.177	0.263
NM_1779!	chr4	4188803	4226894	-	1861	1	OTOP1|-|-	0.028	0.007
NR_02727	chr13	18195297	18232016	+	1954	1	FAM230C|	0	0
NM_0030!	chr10	1.17E+08	1.17E+08	+	3854	1	SLC18A2|S	0.616	0.448
NM_0013:	chr1	65149003	65232145	+	6626	1	AK4|AK 4|	2.267	1.612
NM_0018/	chr21	45981749	46005049	+	4188	1	COL6A1|B	76.24	53.98
NM_0150!	chr22	21417404	21451461	+	6791	1	HIC2|HRG:	13.123	6.892
NR_11047	chr22	46085997	46113928	+	4561	1	MIRLET7BI	0.011	0.129
NR_10462	chr1	1.01E+08	1.01E+08	-	1211	1	LOC10192:	0.256	0.339
NR_03158	chr13	52810050	52810140	+	90	1	MIR759|h:	0	0
NR_10679	chr1	43448539	43448611	+	72	1	MIR6735|I	0.358	0.571
NR_03625	chr8	28505116	28505182	-	66	1	MIR4288|-	0	0
NM_0336/	chr5	1.72E+08	1.72E+08	-	4459	1	FBXW11|E	32.727	41.595
NM_0011!	chr20	9514358	9530524	+	1903	1	LAMP5|BA	4.573	16.713
NM_0166:	chr6	24649977	24666887	-	1916	1	TDP2|AD0	11.682	13.468
NM_0150:	chr19	4969113	5153597	+	5578	1	KDM4B|JN	40.222	33.565
NR_02983	chr9	4850297	4850375	+	78	1	MIR101-2|	0	0
NM_0048!	chr17	9250471	9575958	-	958	1	STX8|CARI	42.61	45.838
NM_0044(	chr8	17864391	17895538	-	1239	1	FGL1|HFRI	0	0.033
NM_1781.	chr2	1.79E+08	1.79E+08	-	10428	1	SESTD1|SC	6.537	6.926
NR_03391	chr9	91063294	91075132	-	5245	1	LOC10012!	0.017	0.031
NR_11937	chr8	1.27E+08	1.27E+08	-	571	1	PCAT2|CA	0	0
NM_0010.	chr11	78171244	78188629	-	3393	1	KCTD21|K	8.786	11.287
NM_0013:	chr17	75909574	75938232	-	4648	1	FBF1|Alb|	3.298	2.732
NM_0064/	chr17	1650629	1684882	-	7250	2	PRPF8|HPI	0.007	0.011
NR_10344	chr6	52577298	52583993	+	2060	1	TRAM2-AS	3.486	3.91
NM_0013:	chr13	30457916	30464327	-	4263	1	HMGB1|H	11.206	9.71
NR_03819	chr19	19561707	19618916	-	1621	1	PBX4|-|-|:	6.833	4.504
NM_0011(	chr14	75985753	76083749	+	822	1	IFT43|C14	41.739	40.441
NM_0063(	chr7	96688767	96709891	-	493	1	SEM1|DSS	98.519	77.153
NR_11088	chr6	73693903	73696131	-	1971	1	LOC10192:	0.026	0
NR_12587	chr6	1.59E+08	1.59E+08	+	767	1	LOC10272/	0	0
NM_1488!	chr7	1.14E+08	1.15E+08	+	1399	1	FOXP2|CA	7.972	6.178
NR_03032	chr7	1.28E+08	1.28E+08	+	99	1	MIR593|M	0	0
NM_0011/	chr14	92936914	93115919	-	2791	2	ITPK1|ITRI	0	0
NM_0012{	chr12	69653609	69699416	-	3260	1	BEST3|VM	0.008	0.273
NM_2125!	chr4	70518572	70532743	+	995	1	AMTN|UN	0	0

NR_03981	chr9	92527984	92528058	-	74	1	MIR4670|-	0	0
NR_04687	chr13	84140602	84606768	+	805	1	LINC00333	0	0.051
NR_13556	chr2	2.08E+08	2.08E+08	+	435	1	LINC01857	0	0
NM_0059!	chr1	1.51E+08	1.51E+08	-	1523	1	VPS72|CFL	125.836	112.742
NR_11043	chr1	1E+08	1E+08	-	763	1	RTCA-AS1|	0.49	0.79
NM_0219(	chr14	95709967	95714196	-	1390	1	TCL1A|TCL	0	0
NR_10988	chr20	21570024	21615722	+	563	1	LINC01727	0	0
NM_0011:	chr2	2.24E+08	2.24E+08	-	2175	1	SERPINE2|	13.148	28.786
NM_0182;	chr14	74348463	74360008	+	3412	1	VRTN|C14	0.008	0.004
NM_0017<	chr5	1.35E+08	1.35E+08	+	2236	1	CAMLG|C/	30.642	33.134
NR_13514	chr9	1.25E+08	1.25E+08	-	292	1	LOC10537(	0.088	0
NR_11063	chr1	82973883	82986208	-	1519	1	LINC01361	0	0.243
NM_0132{	chr5	1.64E+08	1.64E+08	+	2145	1	MAT2B|M	21.471	18.678
NM_0143:	chr22	17084959	17115694	+	8593	1	IL17RA|CA	9.651	4.286
NM_0012!	chr16	2033264	2039026	+	1797	1	SLC9A3R2	9.256	14.696
NM_0011(	chr1	16048762	16057326	+	2159	1	CLCNKB|C	0.137	0.019
NM_1453:	chr2	27771717	27779741	+	406	1	MRPL33|C	92.7	98.949
NM_0010:	chr9	35817017	35828747	-	2938	1	FAM221B|	0.123	0.112
NR_13528	chr9	1.37E+08	1.37E+08	-	3399	1	LINC01451	0.008	0.044
NR_04749	chr13	88626853	88630397	+	1597	1	LINC0056C	0	0
NM_0133(	chr19	44052011	44067994	+	2412	1	ZNF223|-|	5.271	6.298
NR_03966	chr5_KI27(	92689	92731	+	42	2	MIR4456|-	0	0
NM_0012(	chr1	1.51E+08	1.51E+08	+	1939	1	TNFAIP8L2	22.755	19.708
NM_0010{	chr8	7847880	7863797	+	750	1	SPAG11A|	0	0
NM_0011<	chr15	88512481	88546703	-	2290	1	DET1|-|-|:	7.928	9.48
NR_10705	chr8	1.44E+08	1.44E+08	-	64	1	MIR7112|I	3.022	4.708
NM_1522:	chrX	1.03E+08	1.03E+08	+	1179	1	TCEAL7|M	66.698	31.03
NM_1390<	chr3	1.49E+08	1.49E+08	-	4486	1	HLTF|HIP1	8.792	7.05
NR_03416	chr2	1.57E+08	1.57E+08	-	2018	1	FAM133DI	0.141	0.061
NM_0059:	chr15	43804535	43824753	-	2052	1	MFAP1|-|-	30.525	28.903
NM_0060:	chr19	6361452	6368904	+	1143	1	CLPP|DFN	56.154	48.149
NM_0015:	chr6_GL0C	2164504	2170407	+	1700	6	GTF2H4|P.	0	0
NM_0066(	chr1	26921722	26946871	+	1802	1	NUDC|HN	65.498	55.191
NM_0802{	chr17	68974632	69060995	-	6260	1	ABCA9|ES	0.194	0.125
NM_0011:	chr3	1.68E+08	1.68E+08	+	1666	1	SERPINI1|I	17.552	25.191
NR_10382	chr10	42408174	42472240	-	3296	1	CCNYL2|C:	0	0.025
NM_0012!	chr20	2814987	2815830	+	612	1	C20orf141	0	0
NR_03962	chr2	11540605	11540677	-	72	1	MIR4429|I	0	0
NR_00303	chr20	49280683	49280772	+	89	1	SNORD12|	2.463	1.385
NM_0022!	chr19	43766533	43781257	-	2229	1	KCNN4|DH	0.075	1.217
NM_1992{	chr19	42302132	42310821	+	1525	1	PRR19|-|-	44.083	37.409
NM_0157:	chr6	1.55E+08	1.55E+08	-	2028	1	NOX3|GP!	0.076	0.128
NM_0321:	chr13	41191406	41194575	-	3167	1	KBTBD7|-|	5.407	5.077
NM_0011!	chr3	1.57E+08	1.57E+08	+	1933	1	LEKR1|-|-|	0	0.028
NM_1385(	chr19	45212621	45234211	-	1707	1	EXOC3L2|)	0.015	0.032
NM_0065!	chr11	74592530	74643060	+	3810	1	POLD3|P6	4.684	4.526
NR_03382	chr1	2.31E+08	2.31E+08	-	762	1	SNRPD2P2	0	0
NM_1993(	chr20	35002404	35092815	-	3125	1	TRPC4AP|(	56.909	73.412
NM_0012!	chr1	67307364	67396900	+	3766	1	IL12RB2|-|	0.017	0.04
NR_12157	chr9	66022661	66027819	+	2287.5	2	GXYLT1P3	0	0

NM_0061{	chr7	97984701	97990104	-	582	1	OCM2|OC	0	0
NR_13563	chr17	62808502	62836371	+	1807	1	LOC10192	0	0
NR_02988	chr14	1E+08	1E+08	+	98	1	MIR342|M	0	0
NM_0011	chr3	38496272	38526305	+	2989	1	EXOG|END	7.005	8.505
NM_0010(	chr1	2.48E+08	2.48E+08	+	1045	3	OR2T4|OR	0	0
NM_0011	chr19	11505916	11529172	-	1048	1	ECSIT|SITP	39.738	44.659
NM_0031	chr1	23380909	23424779	-	1737	1	TCEA3|TFI	0.408	0.615
NM_0014	chr15	45361124	45378782	-	2575	1	GATM|AG	2.243	1.346
NR_03157	chr11	43559656	43559753	+	97	1	MIR670|h	0	0
NM_0012	chr19	16829387	16880353	+	5032	1	SIN3B|-|-|	27.327	32.786
NR_00127	chr19	40924265	40950660	+	2901	1	CYP2B7P|	0.169	0.453
NM_2074	chr1	1.45E+08	1.45E+08	+	2387	1	FAM72D|	0.022	0.023
NM_0011	chr3	9363033	9386791	+	3763	1	THUMPD3	8.199	7.931
NR_00001	chr17	28720550	28720616	+	66	1	SNORD42E	0	1.038
NR_12599	chr1	8424645	8435016	+	690	1	LOC10272	0.168	0.06
NM_0011	chr12	55684570	55712305	-	3632	1	ITGA7|-|-|	12.23	11.544
NM_0012	chr8	7976393	7977985	-	1591	1	USP17L3|	0	0
NM_0240	chr12	1.02E+08	1.02E+08	-	1281	1	NUP37|p3	8.807	8.19
NM_0010(	chr11	5058650	5059627	-	976	1	OR52E2|-|	0	0
NM_1781	chr2	1.72E+08	1.72E+08	+	2377	1	DLX1|-|-|	0.071	14.146
NR_12639	chr13	1.14E+08	1.14E+08	-	313	1	LINC01054	0	0
NM_0184	chr10	1.32E+08	1.32E+08	+	5727	1	PPP2R2D|	13.814	16.043
NM_0015	chr11	30410071	30580493	-	2394	1	MPPED2|	3.129	25.443
NM_1942	chr2	1.85E+08	1.85E+08	+	4679	1	ZNF804A|	2.119	2.289
NM_0010(	chr11	56375624	56376553	+	865.5	2	OR8U1|-|-	0	0
NR_12597	chr1	33350352	33363245	+	487	1	LOC10192	0.265	0.169
NM_0055(	chr3	32951574	32954911	+	1653	1	CCR4|CC-C	0.016	0
NM_0039	chr14	23559563	23567836	-	2623	1	AP1G2|G2	8.234	5.927
NR_10802	chr5	1.74E+08	1.74E+08	-	567	1	LINC01484	0.045	0.048
NM_0011	chr7	996987	1138257	-	1257	1	C7orf50|Y	73.002	72.637
NM_0023	chrX	30241940	30244191	+	2249	1	MAGEB4|	0	0
NM_0012	chr6	1.27E+08	1.27E+08	+	2078	1	RNF146|-|	29.429	36.398
NR_10704	chr19	51206929	51207009	-	80	1	MIR8074|	0	0
NM_0013(	chr16	75443244	75464737	-	4608	1	TMEM170	4.457	4.512
NM_2015	chr15	74174564	74176871	+	2107	1	ISLR|HsT1	0.875	0.871
NM_1767	chr1	54609177	54623527	-	1971	1	FAM151A|	0.059	0.021
NM_0309	chr11	66268585	66277492	+	1947	1	RAB1B|-|-	56.415	52.896
NM_0012	chr15	92900321	93028007	+	9323	1	CHD2|EEC	15.668	22.001
NR_11066	chr4	1.2E+08	1.2E+08	-	528	1	LINC01365	0	0
NM_0012	chr9	1.33E+08	1.33E+08	-	2898	1	SURF4|ER	69.077	68.329
NM_0154	chr14	31291788	31420582	-	7805	1	HEATR5A|	1.687	2.813
NM_0013(	chr12	76048592	76085033	-	2494	1	NAP1L1|N	64.111	73.444
NM_0013(	chr19	16161368	16173525	-	558	1	CIB3|KIP3	0.277	0
NM_1527	chr6	1.21E+08	1.21E+08	-	5087	1	TBC1D32|	0.621	0.88
NM_0010	chr19	35758143	35769175	+	2133	1	PROSER3|	6.553	3.859
NM_1829	chr19	4815924	4831742	-	2718	1	TICAM1|II	0.076	0.459
NM_0219	chr4	1.74E+08	1.74E+08	-	2364	1	HAND2|DI	0.022	0.081
NR_00153	chrY	6443434	6447077	+	305	2	TTTY21|N	0	0
NM_0058	chr13	21140514	21149085	+	2295	1	SAP18|2H	66.642	57.951
NM_0012	chr20	3107573	3159910	-	4333	1	UBOX5|RN	4.52	5.905

NM_0328:	chr16	28950997	28966446	+	3848	1	NFATC2IP|	10.233	7.535
NM_0013:	chr2	1.3E+08	1.3E+08	+	3060	1	IMP4|BXD	16.972	19.377
NR_13425	chr5	1.19E+08	1.19E+08	-	522	1	LOC10537!	0.568	0.499
NM_0011(	chr17	37514797	37609438	-	7531	2	SYNRG|AP	0	0
NM_1533:	chr17	15229777	15260844	-	1734	1	PMP22|CM	4.692	5.995
NM_0067:	chr5	32585499	32604079	+	3500	1	SUB1|P15	20.375	29.883
NR_12595	chr1	26462757	26467537	-	779	1	LOC10192:	0.265	0.141
NR_02806	chrY	7273972	7381547	+	7209	1	PRKY|PRK:	1.381	1.83
NR_02442	chr12	8356964	8390752	-	659	1	LINC00937	0.235	0
NR_11082	chr17	66398069	66416854	-	1352	1	PRKCA-AS:	0	0
NM_0034!	chrX	1.54E+08	1.54E+08	+	1663	1	TMEM187	4.311	4.176
NR_02949	chrX	1.34E+08	1.34E+08	-	95	1	MIR19B2|	0	0
NM_0142(	chr11	16738601	16758354	+	3973	1	C11orf58|	28.422	26.977
NR_02452	chr2	1.53E+08	1.53E+08	+	1793	1	ARL6IP6|A	7.975	7.143
NM_0540:	chr11	18120955	18138480	+	1634	1	MRGPRX3	0	0
NM_0013:	chr22	42380408	42432395	-	5484	1	NFAM1|CI	0.292	0.252
NM_0013(	chr18	13726660	13764556	+	4138	1	RNMT|CM	13.897	13.753
NM_0201/	chr12	57234903	57240692	-	1181	1	NDUFA4L2	3.548	3.479
NR_12571	chr1	2.18E+08	2.18E+08	+	1369	1	TGFB2-OT:	0.122	1.551
NM_0011/	chr11	71999927	72002528	+	1033	1	IL18BP|IL1	4.344	4.641
NR_00334	chr15	25228967	25229048	+	81	1	SNORD115	0	0
NM_0013(	chr11	61949884	61964463	+	2399	1	BEST1|ARI	3.864	7.017
NR_04853	chr18	57054573	57072119	-	2585	1	LINC-ROR|	0	0.016
NM_1983{	chr17	50561068	50627471	+	7829	1	CACNA1G|	6.691	12.061
NM_1391(	chr10	1.15E+08	1.15E+08	+	3402	1	TRUB1|PU	6.473	5.572
NM_0012{	chr2	2E+08	2E+08	+	6107	1	SPATS2L|[	3.794	4.389
NM_0011{	chr13	24680411	24711785	+	3709	1	ATP12A|#!	0.007	0.041
NR_03624	chr8	10666978	10667070	+	92	1	MIR4286|I	0	0
NM_1991:	chr5	10225508	10249909	-	2664	1	FAM173B|	4.438	3.676
NR_04511	chr10	1.02E+08	1.02E+08	+	967	1	KCNIP2-AS	3.427	5.652
NM_0145(	chr12	70366282	70434292	+	4720	1	KCNMB4|-	7.345	13.865
NM_0010{	chr2	1.31E+08	1.31E+08	-	688	1	MZT2A|FA	37.106	22.795
NR_03620	chr19	10230413	10230485	+	72	1	MIR4322|-	1.254	1.522
NR_13046	chr14	1.06E+08	1.06E+08	-	59	2	MIR4539|-	0	0
NR_02962	chr14	1.04E+08	1.04E+08	+	109	1	MIR203A|	0	0
NM_0011!	chr15	89784895	89814840	-	3627	1	ANPEP|AP	0.014	0.011
NR_03742	chr5	38557502	38557561	-	59	1	MIR3650|-	4.152	3.25
NM_1782:	chr7	1E+08	1E+08	+	1432	1	PILRB|FDF	11.975	11.641
NM_0010:	chr1	1.54E+08	1.54E+08	+	345	1	RPS27|MP	1.607	0.675
NM_0010:	chr10	96304328	96338564	+	2024	1	DNTT|TDT	0	0
NM_0010(	chr3	1.14E+08	1.14E+08	-	13698	1	USF3|KIAA	1.643	2.258
NM_0201/	chr15	70881342	70892433	-	2042	1	THAP10|-|	1.44	1.838
NM_0011(	chrX	1.2E+08	1.2E+08	-	3414	1	TMEM255	17.95	6.086
NM_0010(	chr1	2.49E+08	2.49E+08	-	934	1	OR14I1|OI	0	0
NM_0530/	chr9	1.37E+08	1.37E+08	-	1553	1	TMEM203	33.965	38.507
NM_0037/	chr6	39188971	39229475	-	3776	1	KCNK5|K2	0.027	0.007
NM_0010(	chr12	88035491	88050160	+	2865	1	C12orf29|	2.286	1.989
NM_0245{	chr16	4796962	4802950	-	1728	1	ROGDI|KT:	33.234	39.474
NM_0029:	chr6	1.11E+08	1.11E+08	-	10801	1	REV3L|PO	1.518	4.555
NR_05199	chr10	37793938	37795175	-	1237	1	ZNF33BP1	0	0.022

NR_03390	chr9	63832050	63859634	-	1461	1	FRG1JP|-|	0	0
NM_0011	chr7	1.51E+08	1.51E+08	-	1084	1	CDK5|LIS7	55.475	53.335
NR_02838	chr3	38451027	38454820	-	2515	1	ACVR2B-A	1.036	1.372
NM_0072	chr12	51352049	51391416	-	4506	1	GALNT6|G	0.006	0.088
NR_02994	chrX	1.34E+08	1.34E+08	-	70	1	MIR18B|M	0	0
NM_0165	chr2	2.38E+08	2.38E+08	+	2513	1	SCLY|SCL|	7.127	5.941
NR_10237	chr9	33934297	33934375	-	78	1	SNORD121	0	0
NR_12638	chr2	70887871	70889984	+	646	1	LINC01143	0.02	0
NM_0013	chr6_GL0C	2871031	2872847	+	621	6	AIF1|AIF-1	0	0
NM_0201	chr5	1.16E+08	1.16E+08	-	5808	1	FEM1C|EU	2.047	4.78
NM_0186	chr12	1.33E+08	1.33E+08	+	950	1	PXMP2|PM	26.642	16.667
NM_0011	chr7	87628413	87832298	+	4037	1	RUNDC3B|	4.443	2.969
NM_0013	chr1	2.31E+08	2.31E+08	+	8322	1	TRIM67|TI	8.117	9.974
NR_00335	chr15	25250859	25250940	+	81	1	SNORD115	0	0
NM_0036	chr17	60600183	60666279	+	4769	1	PPM1D|PF	8.073	10.558
NM_0010	chr16	46802047	46831162	-	1377	1	C16orf87|	8.015	11.359
NM_0324	chr3	9890587	9894347	+	1654	1	JAGN1|GL	26.278	24.578
NM_0058	chr16	46955362	46973714	-	3037	1	DNAJA2|C	36.829	38.196
NR_12636	chr1	9680845	9687574	-	529	1	PIK3CD-AS	6.093	4.92
NR_03412	chr5	43065187	43066971	-	1784	1	LOC10013	1.178	1.175
NM_0010	chr19	12014717	12035741	-	2346	1	ZNF433|-|	3.05	3.217
NM_0012	chr14	1.05E+08	1.05E+08	-	2846	1	BRF1|BRF	27.667	30.922
NM_1775	chr12	1.23E+08	1.23E+08	-	2062	1	HCAR2|GF	0.025	0.007
NR_03618	chr10	14383200	14383277	-	77	1	MIR4293|	0	0
NM_0012	chr2	11155372	11178872	+	1778	1	PQLC3|C2	1.632	1.132
NM_0145	chr17	38352124	38405593	+	7966	2	SOCS7|NA	0.405	0.841
NM_0011	chrX	1.53E+08	1.53E+08	-	3109	1	PNMA5|-|	0.046	0.251
NM_0037	chr4	75724522	75814289	+	4155	1	USO1|P11	6.628	6.293
NM_0162	chr1	20742678	20786688	-	3906	1	HP1BP3|H	64.652	64.053
NR_13524	chr3	14799331	14811414	-	3827	1	LINC02011	0	0
NM_1984	chr19	40740856	40749923	-	2745	1	C19orf54|	10.362	7.974
NR_10809	chr4	1.78E+08	1.78E+08	-	756	1	LINC0109	0	0
NM_0330	chr2	2.02E+08	2.02E+08	-	2434	1	MPP4|ALS	0.037	0.045
NM_0162	chr16	5024844	5033941	-	2197	1	NAGPA|AF	2.471	2.849
NM_0010	chr6	1.46E+08	1.46E+08	-	7301	1	SHPRH|bA	2.495	2.85
NR_03995	chr3	48644194	48644280	-	86	1	MIR4793|	16.642	21.82
NM_0011	chr1	1.56E+08	1.56E+08	+	2974	1	SEMA4A|C	4.981	5.366
NR_03884	chr3	28575278	28758337	+	1649	1	LINC00693	1.274	2.508
NR_03784	chr2	74455023	74460891	+	2104	1	INO80B-W	60.086	50.498
NM_0143	chr16	46689646	46698394	+	1619	1	ORC6|ORC	4.954	4.898
NM_0165	chr11	77666229	77820834	-	5116	1	RSF1|HBX	7.604	12.931
NR_02765	chr21	29305204	29361896	+	2770	1	BACH1|BA	6.037	3.689
NM_0012	chr10	58334979	58370753	+	2615	1	UBE2D1|E	6.696	6.835
NM_0010	chr16	55802852	55833163	-	1996	2	CES1|ACA	0	0
NM_0012	chr3	36380487	36548006	+	2889	1	STAC|STA	0.768	1.209
NR_03986	chr15	88611847	88611924	-	77	1	MIR3529|	0	0
NR_04568	chr11	1.24E+08	1.24E+08	+	864	1	OR8G1|HS	0	0
NM_0325	chrX	46599251	46759172	-	10003	1	SLC9A7|NI	2.766	3.738
NM_1781	chr16	1997652	2009821	-	3353	1	ZNF598|-|	16.412	14.514
NM_0249	chr2	1.66E+08	1.66E+08	+	11680	1	CSRNP3|F	7.334	6.364

NR_12160	chr9	20683302	20684750	-	421	1	FOCAD-AS	0.184	0.293
NR_03977	chr12	17673299	17673394	+	95	1	MIR3974|-	0	0
NM_1522	chr11	73376360	73397474	+	3428	1	RELT|TNFF	4.07	6.125
NR_11003	chr20	52620838	52650426	+	4382	1	LINC01524	0.006	0.025
NM_0013	chr1	1.21E+08	1.21E+08	-	1841	1	FAM72B|p	0.063	0.097
NR_13122	chr5	1.42E+08	1.42E+08	-	688	1	SPRY4-IT1	0.562	0.259
NR_07303	chr14	56800707	56805663	-	1951	1	OTX2|CPH	20.659	12.44
NR_13392	chr4	75514433	75547332	+	1159	1	THAP6|-|-	3.115	4.219
NR_12636	chr6	81813286	81814192	-	553	1	LINC0152	0	0
NM_0030	chr16	48360543	48385318	-	1999	1	SIAH1|SIA	18.853	25.763
NM_1779	chr19	48552172	48599427	+	1191	1	SULT2B1|	0.844	0.506
NM_0010	chr1	44213453	44220679	+	1572	1	DMAP1|D	37.147	33.964
NM_0011	chr1	1.5E+08	1.5E+08	-	455	1	HIST2H3D	0.255	0
NM_0011	chr15	41503642	41513887	-	2717	1	LTK|TYK1|	0.085	0.116
NR_10681	chr11	68044794	68044957	+	163	1	MIR6753|	0.158	0
NM_0009	chr2	2.16E+08	2.17E+08	+	412	1	RPL37A|L3	1677.905	1409.33
NR_12609	chr1	41478775	41484683	-	1157	1	EDN2|ET-2	0.134	0.13
NM_0012	chr4	1.41E+08	1.41E+08	+	1816	1	ZNF330|H	14.839	15.673
NM_1523	chr3	1.51E+08	1.51E+08	-	2056	1	ERICH6|C3	0.038	0.02
NR_11065	chr8	70471134	70485687	+	2363	1	LOC10192	0	0
NR_02459	chr20	47318502	47320754	+	2252	1	LOC10013	0.011	0.018
NM_0541	chr12	55331701	55332636	+	934	1	OR6C3|OS	0	0
NM_0010	chr17	17843508	17972470	-	5642	1	TOM1L2|-	19.279	23.104
NM_0314	chr11	1.19E+08	1.19E+08	-	3908	1	MFRP|MC	0.785	0.368
NR_03845	chr7	32456899	32458494	-	1595	1	LOC10013	0.097	0.026
NM_0010	chr15	49155759	49329805	+	3216	1	GALK2|GK	5.06	5.132
NM_0025	chr8	1.19E+08	1.19E+08	-	2339	1	TNFRSF11	0.606	0.673
NM_0011	chr22	50568861	50578667	-	2557	1	CPT1B|CP	21.365	18.491
NM_0010	chr6	49960292	49964105	-	207	1	DEFB114|	0	0
NM_0011	chr12	71839707	71926849	+	5726	1	TBC1D15|	2.189	2.61
NM_0807	chr10	70672506	70762439	+	5531	1	ADAMTS1	0.105	0.225
NM_1481	chr19	38941401	38952687	-	2168	1	FBXO17|F	1.035	0.43
NM_1532	chr8	73064543	73093272	-	3791	1	SBSPON|C	0.044	0.069
NM_0011	chr22	20080232	20111877	+	4406	1	DGCR8|C2	16.818	19.513
NR_03885	chr21	30209151	30211783	-	421	1	LINC00307	0	0
NR_12871	chrUn_GL	148704	148795	+	91	7	MIR6724-2	0	0
NM_0037	chr16	11254417	11256182	-	1212	1	SOCS1|CIS	0.489	0.915
NM_0022	chr3	1.25E+08	1.25E+08	-	4362	1	ITGB5|-|-|	2.069	6.085
NM_1720	chr7	1.51E+08	1.51E+08	-	3182	1	KCNH2|ER	29.341	23.085
NR_02951	chr7	1.3E+08	1.3E+08	-	76	1	MIR96|DF	0	0
NM_0012	chr16	4795378	4796491	+	598	1	SMIM22|-	0.539	0.412
NM_1944	chr16	75111860	75116772	-	1990	1	LDHD|DLD	1.212	0.695
NM_0035	chr17	3126610	3127551	-	940	1	OR1G1|OF	0	0
NM_0012	chr1	1.61E+08	1.61E+08	-	3884	1	ARHGAP3	0.574	0.677
NM_1768	chrX	45148373	45200901	-	4649	1	CXorf36|4	0.006	0.029
NM_0010	chr7	1.37E+08	1.37E+08	+	2383	1	CHRM2|H	0.276	0.448
NM_0201	chr7	39566376	39572881	+	894	1	YAE1D1|C	16.485	16.148
NR_02686	chr16	2839573	2842751	-	2961	1	PRSS30P|I	0	0
NR_03341	chr12	87782886	87784711	-	1825	1	MKRN9P|	2.847	3.618
NM_1768	chr7	1.27E+08	1.27E+08	-	4481	1	ZNF800|-|	0.915	1.253

NR_02447	chr8	1.23E+08	1.23E+08	-	1155	1	FAM83A-A	0	0.012
NM_0010!	chr22	24219654	24245142	-	2385	1	GGT5|GGL	0.119	0.069
NM_0064!	chr22	37570246	37580017	-	522	1	LGALS2|HI	0.148	0.682
NR_03387	chr2	1.45E+08	1.45E+08	+	4781	1	TEX41|DKI	0	0
NM_0042!	chr15	74920276	74938154	-	762	1	COX5A|CC	161.219	143.333
NR_04981	chr7	1.02E+08	1.02E+08	+	84	1	MIR5090|	1.842	1.468
NM_0010!	chr8	7428894	7430348	-	508	3	DEFB103A	0	0
NM_1533	chr4	7040850	7043001	-	2149	1	CCDC96|-|	1.104	1.02
NR_12577	chr5	1.24E+08	1.24E+08	-	731	1	LINC0117C	0.159	0.112
NM_0011	chr17	78004237	78108835	+	9747	1	TNRC6C|-|	8.186	4.344
NR_10980	chr3	29264194	29280939	-	877	1	RBMS3-AS	0.426	0.234
NM_1782	chr15	88877285	88895626	-	1976	1	HAPLN3|E	0.392	0.575
NM_0011!	chr11	32387776	32430817	-	2416	1	WT1|AWT	0	0.017
NM_0335	chr11	77878720	77994671	-	3124	1	INTS4|INT	7.289	8.554
NM_0010!	chr11	65576038	65592645	+	5159	1	EHBP1L1|-	2.642	2.7
NM_0010	chr14	54842006	54902824	-	1970	1	GCH1|DYT	0.903	0.751
NM_0006	chr11	1.13E+08	1.13E+08	+	5942	1	NCAM1|CI	219.842	168.836
NM_0206!	chr17	16546963	16569212	-	7630	1	ZNF287|ZI	1.653	1.962
NR_13517	chr16	71513811	71517846	+	365	1	LOC10537	0	0
NM_0022	chr1	2.11E+08	2.11E+08	-	8012	1	KCNH1|EA	1.862	0.913
NR_03976	chr11	36010098	36010204	+	106	1	MIR3973|	0	0
NM_0012(	chr4	1.67E+08	1.67E+08	-	1485	1	SPOCK3|H	3.43	3.726
NM_1446	chr16	10627504	10694945	-	1600	1	TEKT5|CT1	0.073	0.077
NM_0164!	chr6	1.42E+08	1.42E+08	+	3345	1	VTA1|C6o	18.934	18.717
NR_12601	chr6	5003816	5043589	+	1318	1	LYRM4-AS	1.164	0.842
NM_1388	chr5	1.76E+08	1.76E+08	+	885	1	HIGD2A|R	64.977	47.173
NM_0011	chr3	52287820	52293005	+	1365	1	GLYCTK|H	7.802	4.044
NM_0013(	chr17	14069402	14208679	+	2993	1	COX10|-|-	5.79	6.292
NM_0004	chr19	15159633	15200981	-	8036	1	NOTCH3|C	16.919	13.678
NM_0012!	chr19	6467204	6481808	-	2687	1	DENND1C	1.166	1.371
NM_0012	chrX	1.02E+08	1.02E+08	+	1735	2	TCP11X2|-	0	0
NM_0011	chr20	35115357	35147358	-	1790	1	EDEM2|C2	14.622	15.633
NM_0250	chr10	1.17E+08	1.17E+08	-	5708	1	HSPA12A|	7.741	14.333
NR_11194	chr15	82536750	82540457	-	777	2	RPS17|DB	0	0
NM_0052	chr8	7015869	7018294	-	486	1	DEFA3|DE	0	0
NM_0010	chr3	42091254	42225890	+	5213	1	TRAK1|MI	50.827	34.593
NM_0015	chr6	55174273	55282620	+	1807	1	HCRTR2|O	1.955	0.697
NM_0023	chr12	57128499	57213342	+	14797	1	LRP1|A2M	53.392	60.48
NM_0001	chr17	4932275	4935030	+	2519	1	GP1BA|BD	0.804	0.75
NR_00323	chr14	1.01E+08	1.01E+08	+	70	1	SNORD113	0	0
NM_0163	chr5	62412746	62628589	+	4357	1	IPO11|Rar	3.267	3.103
NM_0010(	chr12	55400529	55401467	+	937	1	OR6C65|-|	0	0
NR_10500	chr8	48551567	48698510	+	2368	1	LOC10192	0.016	0
NR_03039	chrX	1.35E+08	1.35E+08	-	96	1	MIR542|M	0	0
NM_0013	chr19	50958631	50969673	-	1346	1	KLK6|Bssp	0	0.02
NM_0071	chr6	1.44E+08	1.45E+08	+	12355	1	UTRN|DM	0.614	0.368
NR_04610	chr6	1.19E+08	1.19E+08	-	3463	1	LOC28576	0	0
NM_0002(	chrX	43948776	43973675	-	1999	1	NDP|EVR2	1.367	9.586
NM_0121(	chr19	18612872	18621039	+	1609	1	TMEM59L	91.553	129.394
NM_0042!	chr6	57172306	57185214	+	2047	1	BAG2|BAG	0.768	0.709

NM_0012!	chr9	36572862	36677683	+	2143	1	MELK|HPK	3.267	2.652
NR_10686	chr19	55239912	55239976	-	64	1	MIR6802|I	5.641	4.922
NR_04703	chr13	45418162	45420371	+	699	1	SLC25A30-	0.277	0.372
NM_0012!	chr4	4235542	4248242	-	1253	1	TMEM128	10.691	13.938
NM_0010(	chr11	56290127	56291062	-	934	2	OR8H1|OF	0	0
NM_0011!	chr1	1.8E+08	1.8E+08	+	2463	1	TDRD5|TU	0	0.006
NR_13394	chr4	58984282	59046959	+	693	1	LOC10537	0	0
NR_03756	chr22	21103016	21122286	+	2328	1	BCRP2|BC	0.083	0.082
NM_0012(	chr20	3471018	3616044	+	3698	1	ATRN|DPP	14.18	15.145
NM_0013:	chr22	40346500	40366748	+	1670	1	ADSL|AMF	52.84	46.209
NM_0067:	chr11	65314818	65322429	+	2019	1	CDC42EP2	6.501	7.741
NR_13428	chr5	74865893	74867276	+	1383	1	LOC44108	0.457	0.446
NM_0011:	chr12	51093756	51173143	-	3695	1	TFCP2|LBF	18.634	17.018
NM_0011(	chr4	1.64E+08	1.64E+08	-	5492	1	MARCH1|I	2.115	2.392
NM_0049:	chr14	95686417	95692643	+	1160	1	TCL1B|SYN	0	0
NR_01538	chr19	58351970	58355183	+	2126	1	A1BG-AS1	5.986	5.554
NM_0010!	chr3	1.43E+08	1.43E+08	+	7434	1	U2SURP|S	5.883	8.662
NM_0020:	chr18	63327738	63367273	-	5186	1	KDSR|DHS	4.622	4.78
NM_0012!	chr3	58192626	58211003	-	977	1	DNASE1L3	0.013	0
NM_1309(	chr4	46734830	46909235	-	538	1	COX7B2|-|	0	0
NM_0010:	chrX	1.35E+08	1.35E+08	-	1866	1	CT55|BJH(	0.014	0.015
NR_00154	chrY	8783320	8817382	-	822	1	TTTY11|N(	0	0
NM_0010!	chr11	57761762	57819180	+	5674	1	CTNND1|(	49.083	73.241
NR_12060	chr3	1.91E+08	1.92E+08	+	1175	1	LINCR-000	0.845	0.326
NM_1829:	chr3	39467573	39516003	+	3077	1	MOBP|-|-	0.021	0.018
NM_0154!	chr15	75201880	75212169	+	4422	1	C15orf39|	1.644	3.382
NM_0013(	chr16_KI2:	575341	609661	+	3672.5	2	RRN3|A-2:	0	0
NR_00291	chr17	7574713	7574847	+	134	1	SNORA48|	2.79	3.578
NM_0012!	chr9	1.36E+08	1.36E+08	+	2154	1	PMPCA|AI	27.11	30.014
NR_03174	chr3	48316360	48316459	-	99	1	MIR2115|-	0	0
NM_0122:	chr7	76609986	76627303	-	1543	2	POMZP3|F	0	0
NM_1444!	chr20	62238485	62296213	+	3994	1	OSBPL2|D	20.163	22.102
NM_0010!	chr1	1.67E+08	1.67E+08	+	4111	1	DUSP27|-|	0.009	0.02
NM_0030:	chr10	97766751	97771999	-	1878	1	SFRP5|SAF	0.034	0.255
NM_0071!	chr7	1.51E+08	1.51E+08	+	4666	1	ABCB8|ES	9.177	10.08
NR_03161	chr8	1.28E+08	1.28E+08	+	86	1	MIR1207|I	0	0
NM_0050:	chr6	1.32E+08	1.32E+08	+	3205	1	ENPP3|B1	0.052	0.295
NM_0013(	chr8	27733316	27772653	-	3533	1	CCDC25|-|	9.777	7.099
NR_10810	chr7	69596138	69597495	-	556	1	LOC10050	0.348	0.493
NM_0023:	chr1	53226892	53238610	-	749	1	MAGOH|N	27.199	23.645
NM_0243	chr9	37800786	37867668	+	7912	1	DCAF10|W	3.601	4.974
NM_0331(	chr10	84194635	84218696	+	6762	1	CDHR1|CC	0.98	2.117
NM_0012:	chr1	2.37E+08	2.37E+08	+	4890	1	ACTN2|CN	0.216	0.515
NR_03745	chr4	1.59E+08	1.59E+08	-	92	1	MIR3688-1	0	0
NM_1825:	chr19	58034066	58054631	+	2046	1	ZSCAN1|N	2.703	2.51
NM_0336:	chr12	1.17E+08	1.17E+08	-	4219	1	FBXO21|FI	27.99	38.799
NR_03611	chr11	48096782	48096858	+	76	1	MIR3161|-	0	0
NM_0012!	chr2	1.54E+08	1.54E+08	-	2355	1	LOC10014	0.033	0.093
NM_0013:	chr1	2.28E+08	2.28E+08	+	1892	1	SNAP47|C	29.31	35.538
NM_0012!	chr7	1.5E+08	1.5E+08	+	3748	1	KRBA1|-|-	5.786	6.56

NR_02940	chr2	73234236	73253022	+	1776	1	CCT7|CCTI	238.094	254.775
NM_0322(	chr2	69013144	69249327	+	5873	1	ANTXR1|A	10.351	8.629
NM_1816:	chr21	30561098	30561289	-	190	1	KRTAP19-7	0	0
NM_0250(	chr4	55948808	56033363	+	5707	1	CEP135|CI	0.233	0.521
NM_0328!	chr8	1.23E+08	1.23E+08	+	4618	1	FAM83A|E	0.006	0.006
NM_0248(	chr16	58515479	58520532	+	2901	1	SETD6|-|-	3.871	4.915
NM_0009:	chr17	7484379	7514618	+	6706	1	POLR2A|P	69.662	63.064
NM_0012(	chr1	1.55E+08	1.55E+08	-	1074	1	MUC1|AD	5.931	2.41
NM_0013:	chr2	9405681	9422660	-	1964	1	ITGB1BP1|	31.15	28.231
NM_0019!	chr22	45502839	45563362	+	2278	1	FBLN1|FBL	30.473	38.283
NR_02727	chr4	40042917	40057199	-	1610	1	LOC34496	9.506	8.261
NM_0028{	chr1	1.12E+08	1.12E+08	+	5018	1	RAP1A|C2	6.955	5.953
NR_11063	chr1	2.3E+08	2.3E+08	+	474	1	LOC10192	0	0.029
NR_10979	chr9	89969416	89987486	+	528	1	LOC10192	0	0.078
NM_1755:	chr7	1.5E+08	1.5E+08	+	4180	1	GIMAP8|I/	0.006	0.003
NR_00220	chr7	73154929	73207296	+	3605	2	GTF2IP1|V	7.707	7.941
NM_0012{	chr7	80646653	80674418	+	1649	1	CD36|BDP	1.587	0.706
NM_0012{	chr6	1.17E+08	1.17E+08	-	2349	1	GPRC6A|G	0	0
NR_13425	chr5	73132008	73150592	-	447	1	LOC10537!	0	0
NM_0012:	chr5	75611476	75671846	+	2536	1	ANKDD1B|	0.046	0.113
NR_10809	chr7	47621939	47629893	+	2757	1	LINC01447	0	0
NR_07340	chr5_GL33	920682	926772	+	5280.7	3	LOC44108	0	0.005
NM_1526{	chr4	1.53E+08	1.53E+08	-	3207	1	TMEM154	0.125	0.128
NM_0529:	chr8	1.43E+08	1.43E+08	+	3698	1	RHPN1|OI	5.317	6.923
NR_02697	chr6	6346465	6622826	-	2015	1	LY86-AS1|	0	0.02
NM_0011:	chr9	69205162	69255208	+	4475	1	TJP2|C9DL	1.219	1.27
NR_02948	chr13	50048973	50049061	-	88	1	MIR16-1|N	0	0
NM_0207!	chr15	42575659	42720998	+	15549	1	STARD9|K	4.042	2.777
NM_0219/	chr6	37819531	38154623	+	3325	1	ZFAND3|T	58.081	66.347
NR_03968	chr9	20411148	20411238	-	90	1	MIR4473|-	0	0
NM_2074:	chr12	1.24E+08	1.24E+08	+	11687.5	2	DNAH10|-	0.006	0.019
NR_03377	chrX	51681213	51682514	-	1301	2	CENPVL1|(	0	0
NM_0021!	chr16	82035253	82098534	+	1427	1	HSD17B2|	7.779	0.681
NM_0013:	chr3	26623700	26710774	+	1423	1	LRRC3B|LF	9.269	38.367
NR_03975	chr17	82668233	82668307	-	74	1	MIR4525|-	0	0
NR_03650	chr14	89417354	89419793	+	1075	1	FOXN3-AS:	1.091	0.726
NR_01537	chrX	46545490	46548475	+	2146	1	ZNF674-AS	3.683	5.674
NR_11082	chr7	1.47E+08	1.47E+08	-	2137	1	LOC10192:	0	0
NR_13564	chr17	45168805	45171485	-	2046	1	LOC10537	7.228	7.223
NM_0013:	chr1	25362249	25420872	-	1527	1	RHCE|CD2	1.52	0.987
NR_03392	chr3	1.94E+08	1.94E+08	+	1121	1	LINC00884	0.265	0.623
NM_0065:	chr19	18442663	18522127	-	4013	1	ELL|C19or	3.862	3.458
NM_0026:	chr12	1.02E+08	1.02E+08	-	761	1	PMCH|MC	0.695	0.396
NM_0325:	chr19	14619117	14674918	-	2405	1	ADGRE3|E	0	0
NR_04519	chr5	9546200	9550297	+	1484	1	SNHG18|-	1.581	0.618
NM_1446(	chr11	95165513	95231082	-	9431	1	SESN3|SES	3.608	12.089
NM_0044:	chr17	28879339	28897697	-	2619	1	FLOT2|ECS	64.285	57.241
NM_1982/	chr12	1.22E+08	1.22E+08	-	5749	1	CLIP1|CLIF	7.414	9.08
NM_0010/	chr18	74435728	74457268	-	2036	1	FAM69C|(	2.394	3.909
NR_02665	chr18	2943215	2946623	-	3408	1	LOC72789	0.216	0.289

NM_0064	chr5	1.77E+08	1.77E+08	+	2389	1	RGS14|-|-	5.413	5.768
NR_03052	chr1	1.57E+08	1.57E+08	-	113	1	MIR765|	0.228	0.121
NM_1452	chr2	99181079	99199557	+	4528	1	MRPL30|L	11.057	11.915
NR_13190	chr2	2.36E+08	2.36E+08	+	662	1	AGAP1-IT1	0.156	0.041
NM_0121	chr5	1.36E+08	1.36E+08	+	1784	1	FBXL21|FE	0.022	0
NM_0073	chr17	40140254	40172178	+	4164	1	CASC3|BT	51.289	64.613
NM_0010	chr11	5736448	5737538	+	1088	1	OR56B1|C	0	0
NM_1735	chr22	31681348	31750134	-	10817	1	PRR14L|C	5.569	6.783
NM_0068	chr12	1.13E+08	1.13E+08	-	1594	1	SDS|SDH|	0.016	0.129
NM_0011	chr11	89968504	89974072	+	1525	1	TRIM64|C	0	0
NM_0012	chr2	2.33E+08	2.33E+08	+	1591	1	EFHD1|M	6.629	10.193
NR_00438	chr19	50604963	50605085	+	122	1	SNAR-F|-|	0	0
NM_0012	chr10	47467994	47484158	-	1864	1	ANXA8|A	0	0
NM_0011	chr1	95234155	95247225	+	1131	1	RWDD3|R	4.663	3.161
NR_02728	chr1	2.13E+08	2.13E+08	-	940	1	FLVCR1-AS	1.92	2.04
NR_12157	chr9	1.36E+08	1.36E+08	-	1114	1	LOC10192	11.481	12.517
NR_04663	chr13	66977389	66985776	+	519	1	PCDH9-AS	0	0
NM_0141	chr12	55818022	55821175	+	1328	1	ORMDL2|	23.078	16.812
NM_0010	chr5	77210881	77428255	+	4321	1	PDE8B|AD	0.838	1.182
NM_0013	chr2	2.01E+08	2.01E+08	-	9488	1	FAM126B|	1.298	1.911
NR_11016	chr14	97458816	97464159	+	1166	1	LOC10192	0.022	0.059
NM_0013	chr5	1.81E+08	1.81E+08	-	2483	1	TRIM52|R	4.071	4.049
NM_0011	chr8	1.04E+08	1.05E+08	-	4084	1	LRP12|ST	11.719	12.882
NM_0011	chr14	52859488	52951097	-	2144	1	FERMT2|K	10.362	14.336
NM_0150	chr13	77044657	77327042	-	14940	1	MYCBP2|	7.022	11.058
NM_0121	chr13	51361565	51453139	-	7445	1	INTS6|DBI	1.529	2.088
NR_13641	chr17	76950474	76969199	-	650	1	LOC10537	0.377	0.253
NM_0004	chr21	31659622	31668930	+	958	1	SOD1|ALS	203.765	190.243
NR_00302	chr16	1965184	1965310	+	126	1	SNORA78|	1.842	1.087
NM_0013	chr7	1.17E+08	1.17E+08	+	5432	1	MET|AUT	0.052	0.111
NM_0020	chr1	1.52E+08	1.52E+08	-	12742	1	FLG|ATOD	0	0
NR_10680	chr1	2.28E+08	2.28E+08	-	61	1	MIR6742|	1.902	0.898
NM_0010	chr1	99708703	99765793	-	2660	1	FRRS1|SD	0.276	0.036
NM_2015	chr2	85418931	85437031	+	1956	1	SH2D6|-|-	0.165	0.231
NM_0024	chr14	23381990	23408277	-	5900	1	MYH6|AS	0.013	0.402
NM_0044	chr2	1.64E+08	1.65E+08	-	2371	1	GRB14|-|-	0.473	2.161
NM_0011	chr19	42507306	42528509	-	3169	1	CEACAM1	0.028	0.03
NR_03613	chr14	1.01E+08	1.01E+08	+	77	1	MIR1193|	0	0
NM_0058	chr17	63550435	63594281	+	6372	1	DCAF7|AN	97.893	104.462
NM_1991	chr18	616700	650293	+	1853	1	CLUL1|RA	0.195	0.85
NM_0045	chr12	54028410	54030823	+	1677	1	HOXC6|CP	0	0.057
NR_13619	chr4	69182100	69216767	+	2968	1	LOC10537	0	0
NM_0219	chr6	1.6E+08	1.6E+08	+	3228	1	SLC22A3|	0.04	0.479
NM_0195	chr20	5544434	5611026	-	5437	1	GPCPD1|E	5.94	6.908
NR_02969	chr1	1.56E+08	1.56E+08	-	88	1	MIR9-1|M	0	0.156
NR_02425	chr19	46830585	46830704	-	119	1	SNAR-E|-|	0.217	0
NM_0327	chr5	1.57E+08	1.57E+08	-	2421	1	HAVCR2|C	0.128	0.458
NR_02664	chr11	1665599	1667856	+	1427	3	FAM99A|-	0	0
NM_0012	chr6	43506965	43510343	-	1082	1	LRRC73|C	6.161	2.912
NM_0011	chrX	1.55E+08	1.55E+08	-	1974	1	MPP1|AA	10.411	9.152

NM_0013(	chr19	36115828	36125948	+	880	1	TBCB|CG2	289.268	241.674
NR_10500	chr5	74306410	74308325	-	386	1	LINC01335	0	0
NM_0012!	chr16	29830515	29848039	+	2554	1	MVP|LRP|	4.054	3.577
NR_13423	chr4	88284930	88331421	+	1586	1	LOC10536!	0.268	0.484
NM_0155(	chr5	1.39E+08	1.39E+08	-	5624	1	LRRTM2|-	5.213	2.908
NM_1526	chr2	88524651	88529585	+	790	1	TEX37|C2c	0.033	0.017
NM_0020!	chr5	1.51E+08	1.51E+08	+	1780	1	GPX3|GPx	9.134	11.358
NM_0019:	chr17	49972766	49974959	+	1759	1	DLX4|BP1	0.022	0.039
NM_0196:	chr19	43716062	43720017	+	1602	1	IRGC|CINE	0.072	0.026
NR_13503	chr1	1.19E+08	1.19E+08	-	664	1	LOC10537:	0	0
NR_01538	chr18	5238100	5246506	+	3974	1	LINC00667	14.337	13.99
NR_13525	chr14	73460935	73463642	-	656	1	LOC10192:	0.039	0.063
NM_0041	chr19	14566078	14572077	-	541	1	NDUFB7|E	199.217	134.54
NM_0161:	chr8	96645227	97143503	+	1939	1	CPQ|LDP|	0.206	0.042
NM_0012!	chr8	232550	247340	+	2713	1	ZNF596|-|	1.535	2.59
NM_0312!	chr4	1.39E+08	1.39E+08	+	3864	1	RAB33B|SI	0.908	1.662
NM_0011(	chr19	50621977	50639835	-	2467	1	SYT3|SytII	24.041	15.274
NM_0325!	chr7	22941259	23014151	-	6138	1	FAM126A|	6.127	13.878
NM_0010	chr1	1.5E+08	1.5E+08	-	531	2	HIST2H2A/	0	0
NM_1784	chr3	1.33E+08	1.33E+08	+	2502	1	ACKR4|CC	0.01	0.011
NM_1537:	chr1	1.46E+08	1.46E+08	-	3984	1	LIX1L|-|-|:	14.793	12.858
NM_0010(	chrY	17878260	17880219	-	1957	2	CDY2B|CD	0	0
NR_03897	chr14	21200079	21205503	-	5424	1	LINC00641	14.47	16.682
NM_0062(	chr9	75890644	76195889	+	5794	1	PCSK5|PC!	1.268	1.116
NM_0010	chr6	55434373	55579214	-	2460	1	HMGCLL1|	1.426	0.963
NM_0072(	chrX	66021738	66040125	-	1842	1	VSIG4|CRI,	0	0
NR_03789	chr5	1.35E+08	1.35E+08	-	2156	1	C5orf66-A!	0	0
NR_03400	chrX	71697196	71703406	+	1061	2	LINC00891	0	0
NM_0025!	chr4	30720415	30731216	+	4642	1	PCDH7|BH	1.4	1.366
NM_1810	chr3	52545389	52685780	-	7577	1	PBRM1|B/	17.316	15.232
NM_0010:	chr19	2096869	2099584	+	950	1	IZUMO4|C	2.28	1.6
NM_0011(	chr21	32790673	32813743	-	3961	2	C21orf62|	0	0
NM_0050!	chr17	56887909	56914048	-	5733	1	TRIM25|EI	1.649	4.004
NM_0152	chr17	20999593	21043039	-	5205	1	USP22|US	167.345	223.755
NR_02461	chr3	55657211	55659469	-	2258	1	ERC2-IT1|(	0.029	0.018
NM_0010:	chr20	31303212	31308585	-	306	1	DEFB116|I	0	0
NM_0060!	chr7	83958343	84194901	-	5640	1	SEMA3A|C	1.148	1.953
NM_0012!	chr5	1.38E+08	1.38E+08	+	2302	1	PKD2L2|TI	2.408	3.695
NR_13562	chr3	93980139	94055281	+	3327	1	ARL13B|AI	1.659	1.511
NM_0228:	chr15	88467453	88480767	+	3630	1	MRPS11|H	14.872	16.165
NM_0061(	chr11	13962637	14268133	+	5120	1	SPON1|VS	1.69	5.947
NM_0012(	chr19	57830294	57846238	+	3334	1	ZNF587B|-	1.508	1.882
NM_0245:	chr1	1.93E+08	1.93E+08	+	5920	1	CDC73|C1	2.664	3.135
NR_13275	chr17	82089960	82090140	-	180	1	SNORD134	0.143	0.076
NM_0012!	chrX	44873175	45112612	+	5499	1	KDM6A|K/	4.159	5.604
NM_1824!	chr21	10413532	10491615	+	1879	1	BAGE2|CT	0	0.007
NR_04748	chr13	27829759	27921404	-	1998	1	PDX1-AS1|	0	0
NM_0012(	chr21	45514715	45534628	-	2614	1	SLC19A1|(	11.039	6.875
NM_0174(	chr12	53985162	53990278	+	1955	1	HOXC10|H	0.152	0.869
NM_0541:	chr20	31368618	31373902	+	1133	1	DEFB118|(	0	0

NM_0009	chr10	73007222	73096974	-	2829	1	P4HA1|P4	18.745	6.415
NM_0164	chr5	1.39E+08	1.39E+08	+	1475	1	PAIP2|PAI	101.233	98.609
NR_12650	chr22	31933521	31945362	-	1183	1	C22orf24|	0.981	0.521
NR_00293	chr10	1.33E+08	1.33E+08	+	4890	1	SCART1|-|	0.414	0.417
NM_0012	chr8	66562175	66613249	-	4992	1	MYBL1|A-	0.111	0.132
NM_0000	chr6	80106610	80346270	+	1554	1	BCKDHB|B	16.362	8.171
NM_0188	chr7	98291650	98401115	-	3654	1	BAIAP2L1|	0.709	0.675
NR_04664	chrX	49198839	49202454	+	667	1	SYP-AS1|-	2.03	1.848
NM_1387	chr13	1.03E+08	1.03E+08	-	1389	1	TEX30|C1	2.386	3.175
NR_03613	chr16	543277	543366	+	89	1	MIR3176|	0.869	1.539
NR_13567	chr17	27072389	27089241	-	761	1	LOC10537	0	0.234
NM_0012	chr9	35099776	35103195	-	1308	1	STOML2|H	100.89	75.051
NM_0064	chr1	1.1E+08	1.1E+08	-	1472	1	ALX3|FND	3.46	5.369
NR_10502	chr5	88408982	88439090	-	1620	1	LINC0206	0.056	0.719
NM_1785	chr8	1.44E+08	1.44E+08	-	3703	1	NRBP2|TR	10.265	10.801
NR_03892	chr6	27761744	27763187	+	825	1	LOC10013	0	0
NM_0053	chrX	1.4E+08	1.4E+08	-	3774	1	MCF2|ARH	0.089	0.243
NM_0123	chr6	27957241	27958182	+	940	1	OR2B6|OR	0.069	0.073
NM_0012	chr19	11721265	11739009	-	2393	1	ZNF823|H	2.387	1.98
NM_0001	chr19	11377205	11384342	-	2431	1	EPOR|EPO	5.277	4.817
NR_12066	chr10	32434369	32446056	-	4847	1	LOC10192	0	0.023
NM_1750	chr17	3673228	3696404	-	2220	1	P2RX5|LR	1.853	1.962
NM_0012	chr2	1.97E+08	1.97E+08	-	1834	1	GTF3C3|T	7.515	8.17
NM_0063	chr6	26538344	26546936	+	1964	1	HMGN4|H	38.221	41.182
NR_00368	chr9	33952764	33952854	-	90	1	SNORD121	0	0
NR_12038	chr13	99726243	99729001	-	701	1	CLYBL-AS1	0	0
NR_02971	chr17	31559996	31560083	+	87	1	MIR193A|	0	0
NM_0326	chr18	74534457	74585026	+	2185	1	CNDP1|CN	0.159	0.194
NM_0330	chr17	41184100	41184895	-	735	1	KRTAP4-1|	0	0
NR_10977	chr16	32170043	32188113	-	2694	1	HERC2P4|	0	0
NM_0152	chr15	79432516	79472300	+	6736	1	KIAA1024|	2.984	1.586
NR_04540	chr14	61747039	61749089	-	2050	1	HIF1A-AS2	3.428	3.18
NM_0013	chr5	1.09E+08	1.09E+08	+	10669	1	FER|PPP1	1.984	3.696
NM_0218	chr12	27710773	27756304	+	1887	1	MRPS35|H	17.711	17.399
NR_03843	chr2	7421261	7450250	+	917	1	LOC10050	0.028	0.06
NM_0012	chr1	1.56E+08	1.56E+08	-	1688	1	PAQR6|PR	5.683	5.859
NM_0012	chr15	33149784	33194733	-	3960	1	FMN1|FM	0.033	0.028
NM_0010	chr9	1.28E+08	1.28E+08	-	4148	1	FAM102A|	9.244	10.137
NM_0058	chr17	48830988	48865245	+	3667	1	CALCOCO	7.827	6.682
NM_0166	chr18	51174550	51197681	-	4138	1	MEX3C|BN	15.408	17.47
NM_0011	chr2	2.42E+08	2.42E+08	+	2030	2	NEU4|-|-|	0	0
NR_07310	chr19	57681969	57689801	+	3595	1	ZNF551|-|	2.672	5.403
NM_0012	chr6_GL0	3312162	3317010	+	490	5	C6orf48|D	0	0
NM_0246	chr17	82716706	82728017	+	1818	1	FN3KRP|F	20.95	19.589
NM_1738	chr19	53258292	53259602	+	1308	1	VN1R2|V1	0.02	0.01
NM_0013	chr17	74770529	74776425	-	1960	1	NAT9|EBS	19.656	16.786
NM_0012	chr18	46334224	46460820	+	4844	1	RNF165|A	30.565	23.455
NM_0250	chr1	45339670	45343978	+	2382	1	TOE1|hCa	7.968	9.258
NR_13238	chr22	15746674	15778289	+	569	1	LINC01297	0	0
NR_04586	chrX	1.01E+08	1.02E+08	+	2859	1	ARMCX4|	2.196	1.792

NM_0010:	chr9	1.37E+08	1.37E+08	-	1445	1	AGPAT2|1	12.34	7.574
NR_10418	chr22	38991559	38998220	-	567.7	3	APOBEC3B	0.045	0.024
NR_00369	chr2	96010551	96023136	+	848	1	FAHD2CP|	6.477	8.69
NM_0011(	chr10	95755652	95877266	+	12376	1	ENTPD1|A	2.489	1.762
NM_0013:	chr22	25742144	26031041	+	8531	1	MYO18B|I	0.053	0.18
NR_07350	chr1	16367030	16398148	+	3639	1	SZRD1|C1(	44.534	44.08
NR_03391	chr8	1.29E+08	1.29E+08	-	1150	1	LINC00977	0.022	0
NR_11708	chr19	21082147	21125080	+	632	1	ZNF714|-|	4.162	3.381
NM_0010!	chrX	52495668	52500811	+	615	2	XAGE1E|C	0	0
NM_0208(	chr4	26860691	27025381	+	5140	1	STIM2|-|-	2.19	7.898
NM_0011(	chr3	1.53E+08	1.54E+08	+	575	1	C3orf79|-|	0	0.095
NR_02703	chr22	46053846	46058522	+	1632	1	PRR34-AS1	0.032	0.613
NM_0021!	chr5	1.32E+08	1.32E+08	-	3542	1	IRF1|IRF-1	0.477	0.654
NM_0012:	chr4	6640091	6642743	+	2058	1	MRFAP1|F	206.271	230.733
NM_0198‹	chr2	1.53E+08	1.53E+08	-	1468	1	RPRM|REF	36.406	83.134
NM_0157:	chr3	1.28E+08	1.28E+08	+	2199	1	PODXL2|E	126.853	168.033
NM_0013:	chr2	62215521	62224731	+	2611	1	B3GNT2|3	1.96	4.454
NM_0010:	chr17	5501399	5584512	-	5080	1	NLRP1|CA	0.096	0.059
NM_0163:	chr2	1.88E+08	1.89E+08	+	3484	1	GULP1|CE	1.325	1.565
NM_0314:	chr5	1.41E+08	1.41E+08	+	4617	1	PCDHA1|P	18.878	18.07
NM_0333‹	chr11	65592855	65595996	-	1293	1	KCNK7|K2	0.439	0.381
NM_0013:	chr17	64506736	64537951	+	2873	1	CEP95|CCI	10.43	11.671
NR_10692	chr17	4970086	4970150	-	64	1	MIR6865|I	2.619	6.206
NR_02744	chr4	7097424	7103376	-	2138	1	FLJ36777|	0.096	0.006
NR_13014	chr18	3255436	3262359	-	846	1	LOC10496:	19.188	11.932
NM_0013(	chr7	1.49E+08	1.49E+08	+	3477	1	ZNF282|H	17.481	21.867
NM_0012(	chr11	1.1E+08	1.1E+08	-	1803	1	RDX|DFNE	14.088	10.886
NR_03976	chr9	36864254	36864308	-	54	1	MIR4540|	0	0
NM_0021!	chr7	41685114	41703108	-	6059	1	INHBA|ED	0.055	0.323
NM_0012ε	chr20	50190583	50192691	+	2106	1	CEBPB|C/E	2.982	5.372
NM_1458:	chr17	66877273	66885278	+	919	1	CACNG5|-	7.955	10.731
NR_11042	chr19	22520995	22527949	-	3350	1	LOC10192!	0	0
NM_0072(	chr9	1.25E+08	1.25E+08	-	454	1	RPL35|L35	586.171	463.888
NR_11061	chr20	7347433	7368501	+	1648	1	LINC01706	0	0
NR_03740	chr12	1.28E+08	1.28E+08	+	86	1	MIR3612|	0	0
NR_10353	chr6	28943784	28944538	+	754	6	LINC01556	0	0
NM_0012‹	chr17	42025576	42038026	-	2587	1	ZNF385C|-	2.507	2.287
NR_12206	chr7	27121919	27128760	+	1044	1	HOXA-AS2	0.025	0.013
NR_10701	chr19	11495544	11495622	-	78	1	MIR7974|I	0	0
NM_0010(	chrY	22071756	22096007	-	1231	2	PRY2|PTPI	0	0
NM_0010ε	chr17	9576627	9643461	+	1976	1	CFAP52|W	0.803	0.728
NM_0011‹	chr12	3615328	3753200	-	2773	1	CRACR2A|	0.256	0.054
NR_11091	chr16_KI2	17232	19833	+	1715.3	6	TP53TG3|I	0	0
NM_0011(	chr15	75258558	75273455	+	5359	1	GOLGA6C|	0	0
NR_11090	chr16	5601169	5616250	-	783	1	LINC0157C	0	0
NR_04567	chr5	1.81E+08	1.81E+08	+	1868	1	LINC00847	0.587	0.777
NM_0176:	chr1	35808227	35857889	+	7040	1	AGO4|EIF:	9.155	8.775
NR_12594	chr1	18065657	18074411	+	2467	1	LINC01654	0	0
NM_0051!	chr2	2.33E+08	2.33E+08	+	2173	1	CHRNG|A(	0.012	0.032
NR_00324	chr20	63101292	63104385	+	2792	1	HAR1A|HA	0.416	1.962

NM_0144:	chr4	75913656	75941013	-	1850	1	NAAA|ASA	0.195	0.814
NM_0324:	chr1	63523301	63572693	+	2386	1	EFCAB7|-|	3.939	4.179
NM_0001(	chr10	1.03E+08	1.03E+08	-	1860	1	CYP17A1|(	0.118	0.015
NR_11016	chr3	1.5E+08	1.5E+08	+	471	1	LINC01213	0	0
NR_03016	chr7	1.37E+08	1.37E+08	+	127	1	MIR490|M	0	0
NM_0013(	chr10	3775996	3785281	-	4656	1	KLF6|BCD:	7.283	10.39
NM_0010(	chr11	1.25E+08	1.25E+08	+	979	1	OR8A1|OF	0.079	0.196
NM_0133!	chr6	41780762	41787372	+	1636	1	PRICKLE4|	3.097	3.123
NM_0011-	chr1	42430330	42456267	-	1593	1	ZMYND12	1.271	0.671
NR_03036	chr17	76736450	76736548	-	98	1	MIR636|M	17.63	16.632
NM_1816:	chr21	30541535	30541864	-	327	1	KRTAP19-6	0	0
NM_0012{	chr17	1569254	1605034	-	7482.5	2	SLC43A2|L	0.246	0.355
NR_13297	chr22	41073608	41073721	-	113	1	SNORD14C	0.228	0.242
NR_11041	chr14	35081081	35113130	+	907	1	LOC10192	9.226	10.073
NR_07339	chr19	53212948	53216102	+	3154	1	ZNF818P|Z	0.294	0.495
NM_0012(	chr18	72742314	72867575	-	2344	1	NETO1|BC	1.359	2.168
NM_0012(	chr12	55954105	55966062	-	1877	1	PMEL|D12	11.465	4.984
NM_1735!	chr12	56237807	56258359	-	8626	1	ANKRD52|	12.207	13.713
NM_0331:	chr11	5301014	5301946	-	931	1	OR51B4|H	0	0.015
NM_0132!	chr11	6926423	6958047	+	3649	1	ZNF215|B/	0.424	0.176
NM_0065(	chr2	79085023	79088024	-	798	1	REG1B|PSI	0	0.017
NM_0025(	chr1	1.15E+08	1.15E+08	-	1044	1	NGF|Beta-	0.296	0.276
NM_0013:	chr16	24729728	24826226	+	8249	1	TNRC6A|C	9.882	10.798
NR_02963	chrX	45746157	45746266	-	109	1	MIR221|M	0	0.251
NM_0140(	chrX	55452089	55453568	+	1477	1	MAGEH1|/	75.624	79.901
NM_0332-	chr15	73994673	74043376	+	2085	1	PML|MYL|	8.961	9.808
NM_0012{	chr5	72219409	72320257	-	2736	1	MRPS27|M	27.205	34.763
NM_0010(	chr17_KI2	1032391	1075353	+	5159	3	LRRC37A2	0.047	0.088
NM_0011:	chr22	19036282	19122454	-	4350	1	DGCR2|DG	42.877	46.586
NM_0026-	chr1	2.04E+08	2.04E+08	-	7570	1	PIK3C2B|C	16.562	16.364
NM_0043!	chr5	1.77E+08	1.77E+08	-	2926	1	DBN1|D0S	392.484	369.136
NM_0012{	chr6	1.09E+08	1.09E+08	-	4023	1	PPIL6|PPIa	2.372	2.85
NR_11036	chr18	21825479	21831406	-	4231	1	MIR133A1	0	0.01
NR_13115	chr20	57710183	57712780	+	2597	1	NKILA|-|-|	3.977	4.457
NM_0027!	chr9	1.24E+08	1.24E+08	-	1005	1	PSMB7|Z|	208.9	184.467
NR_02718	chr2	1.2E+08	1.2E+08	-	2014	1	LINC01101	0	0
NM_0012(	chr12	1.24E+08	1.25E+08	-	8777	1	NCOR2|CT	11.978	21.525
NM_0014:	chrX	24054948	24078811	+	3452	1	EIF2S3|EIF	23.09	17.716
NR_02686	chr7	1.57E+08	1.57E+08	-	2288	1	C7orf13|M	8.216	13.398
NR_02424	chr19	49031669	49031787	-	118	1	SNAR-G2|-	0	0
NM_0062!	chr10	6427143	6580301	-	3265	1	PRKCQ|PR	0.924	0.65
NM_0324(	chr7	73440398	73522285	-	6093	1	BAZ1B|WE	27.355	23.314
NM_0010:	chr1	91260766	91404869	-	4890	1	HFM1|ME	0.343	0.345
NM_0010-	chr4	76313039	76407305	-	3600	1	CCDC158|	0.018	0
NM_0205:	chr20	24962944	24992789	-	2210	1	APMAP|BS	66.2	70.648
NM_1985-	chr19	41425359	41428730	-	1869	1	B3GNT8|B	1.311	0.33
NR_03994	chr2	88082519	88082599	-	80	1	MIR4780|-	0	0
NM_1524:	chr22	39021113	39033251	+	2494	1	APOBEC3D	0.01	0.143
NM_0013:	chr17	5213012	5234860	-	2185	1	SCIMP|C1	0.649	0.621
NM_0013:	chr19	9810267	9819103	-	1792	1	FBXL12|Fb	14.462	12.673

NM_0008‹	chr5	1.79E+08	1.79E+08	-	6129	1	GRM6|CSI	0.032	0.17
NM_0044‹	chr1	2.2E+08	2.2E+08	-	4981	1	EPRS|EARS	19.373	23.929
NM_0026:	chr4	73980825	73982124	-	851	1	PF4|CXCL4	0	0
NM_0012{	chr15	40287897	40307973	-	4599	1	PLCB2|PLC	0.193	0.161
NM_0012(	chr6	89177504	89231288	-	2917	1	GABRR1|-	0.553	0.089
NM_0013(	chr3	1.68E+08	1.68E+08	-	4226	1	GOLIM4|G	6.59	5.442
NR_12045	chr12	67929235	67970017	+	469	1	LINC0147S	0.11	0
NM_0010(	chrX	1.2E+08	1.2E+08	-	414	1	RPL39|L39	36.874	58.328
NM_0528(	chr5	1.81E+08	1.81E+08	-	461	1	SCGB3A1|	0	0
NM_0022(	chr12	10412315	10420595	-	1016	1	KLRC3|NK	0	0.081
NR_04521	chr9	1.21E+08	1.22E+08	-	1480	1	GGTA1P|G	0	0.037
NM_0013(	chr2	27014754	27027219	+	1946	1	MAPRE3|E	80.064	93.261
NM_0010(	chr1	2.48E+08	2.48E+08	+	937	1	OR2M5|O	0	0
NM_0013:	chr20	2481821	2508915	-	3484	1	ZNF343|dJ	6.091	5.791
NM_0010:	chr6	1.44E+08	1.44E+08	+	982	1	ZC2HC1B|	0	0.07
NM_0010{	chr12	1.25E+08	1.25E+08	-	2599	1	SCARB1|C	8.969	15.515
NM_2012(	chr2	2.06E+08	2.06E+08	+	2567	1	NRP2|NP2	6.263	15.97
NR_02727	chr19	1267471	1270260	-	1354	1	CIRBP-AS1	2.104	2.286
NR_04855	chr2	2.2E+08	2.2E+08	+	4222	1	SLC4A3|AI	19.084	21.6
NM_0010:	chr17	32350138	32370449	+	2217	1	ZNF207|B	61.914	62.647
NM_0010:	chr7	28685981	28825894	+	7886	1	CREB5|CR	0.437	4.629
NM_0012S	chr6_GL0C	1975313	1980539	+	2057.1	8	TUBB|CDC	0.056	0.166
NR_02967	chr3	52294219	52294308	-	89	1	MIR135A1	0.145	0
NM_0059S	chr5	1.72E+08	1.72E+08	-	5998	1	STK10|LOI	4.953	4.574
NM_0041:	chr12	53500921	53506431	+	1396	1	TARBP2|L	22.056	20.261
NR_03396	chr4	75354076	75362566	+	1139	1	LOC44102	0	0
NM_0013:	chr22	30356635	30367096	+	1754	1	CCDC157|	8.277	4.748
NR_03017	chr14	1.01E+08	1.01E+08	+	80	1	MIR494|M	0	0
NR_03606	chr15	21843750	21843824	+	74	4	MIR3118-2	0	0
NR_02448	chr18	74591774	74597835	-	3472	1	LINC0090S	5.422	5.539
NM_0012{	chr11	8947201	8965011	-	1726	1	TMEM9B|	23.613	27.918
NR_00292	chr5	1.12E+08	1.12E+08	+	132	1	SNORA13|	1.172	2.387
NM_1385:	chr6	1.26E+08	1.26E+08	+	3372	1	HINT3|HIN	9.012	8.9
NM_0029{	chr17	34285668	34288180	+	920	1	CCL11|SCY	0.014	0
NM_0186(	chrX	1.36E+08	1.36E+08	+	3024	1	SAGE1|CT	0	0.005
NM_0201!	chr10	70150205	70170529	-	2992	1	SAR1A|SA	36.78	39.1
NR_10803	chr10	97828478	97849798	-	415	1	LINC0086E	0	0
NM_0044{	chr2	1.66E+08	1.66E+08	-	3254	1	GALNT3|G	0.075	0.029
NM_1817(	chr8	94249257	94262319	-	2061	1	GEM|KIR|	1.595	1.901
NM_0063S	chr6_GL0C	821111	825425	-	986	7	UBD|FAT1	0	0
NM_0013(	chr9	34889064	34895778	-	976	1	FAM205C|	0	0.098
NR_00320	chr14	1.01E+08	1.01E+08	+	70	1	SNORD114	0	0
NR_03175	chr17	43444806	43444885	+	79	1	MIR2117|	0	0
NM_0012(	chr19	7888505	7902023	+	3455	1	LRRC8E|-|	0.123	0.139
NR_11077	chr18	9121265	9136720	-	666	1	NDUFV2-A	1.181	0.638
NR_02798	chr1	1.78E+08	1.78E+08	-	2336	1	RASAL2-AS	0.447	0.322
NR_00294	chr19	11665736	11667089	-	1353	1	HNRNPA1I	0.2	0.213
NR_13573	chr7	66847450	66884273	+	1022	1	GTF2IP23|	0.492	0.67
NR_07339	chr2	43230836	43596046	-	5888	1	THADA|AF	3.407	4.192
NM_0013(	chr3	1.56E+08	1.56E+08	-	1818	1	C3orf33|A	1.326	1.311

NM_0210(	chr20	25407683	25448555	+	3284	1	GINS1|PSF	4.201	3.57
NR_02735	chr12	52210930	52223813	+	1048	1	LINC00592	0	0
NM_0013:	chr1	1.09E+08	1.09E+08	+	2784	1	GPSM2|CN	5.706	5.195
NM_0203:	chr7	16599776	16645817	-	2543	1	ANKMY2|Z	19.251	23.225
NR_03028	chr1	1.52E+08	1.52E+08	+	95	1	MIR554|M	0	0
NM_0012!	chr9	27109141	27230178	+	4304	1	TEK|CD20:	0.303	0.038
NR_03341	chr5_GL33	962951	997920	+	4359	5	GTF2H2B|	0.121	0.167
NM_0807!	chr20	45629746	45631192	+	478	1	WFDC10A	0	0
NR_10997	chr17	69593987	69903000	+	617	1	LINC01483	0	0.089
NM_0012!	chr8	1.04E+08	1.04E+08	+	1467	1	DCSTAMP	0.018	0.009
NM_0248:	chr9	26840685	26892828	-	2807	1	CAAP1|C9	4.028	3.884
NR_00292	chr1	2.03E+08	2.03E+08	+	2318	1	LOC14870!	0.506	0.892
NR_11078	chr18	31343368	31426988	-	726	1	DSG1-AS1	0	0
NM_1825:	chr3	28241633	28319772	+	619	1	CMC1|C3c	9.936	16.972
NR_04687	chr3	21957139	21979828	+	417	1	ZNF385D-/	0	0
NR_00334	chr15	25101574	25101667	+	93	1	SNORD116	0.277	0.442
NM_0012(	chr1	1.54E+08	1.54E+08	+	2158	1	CHTOP|C1	55.23	61.421
NM_0210:	chr8	1.21E+08	1.21E+08	-	4966	1	SNTB1|59-	0.332	0.361
NM_0012:	chr2	1.81E+08	1.81E+08	+	1342	1	UBE2E3|U	73.47	75.834
NR_11022	chr2	1.05E+08	1.05E+08	-	569	1	UTAT33|-|	8.679	9.629
NR_00314	chr16	1964996	1965504	+	231	1	SNHG9|NC	15.238	10.139
NR_10386	chr6_GL0C	1316422	1320070	+	657.1	8	ZNRD1|HT	0.02	0
NM_0011‹	chr1	46671833	46673584	+	847	1	TEX38|ATI	0.304	0.016
NM_0006(	chr9	1.14E+08	1.14E+08	+	831	1	ORM1|AG	0	0.016
NM_0010:	chr3	1.27E+08	1.27E+08	-	1128	1	TXNRD3NE	0.8	0.801
NM_0010(	chr15	21637909	21638992	+	1081	4	OR4M2|O	0	0
NM_0224:	chr2	43838964	43878466	+	2650	1	ABCG8|GE	0	0.005
NM_0012!	chr4	1.23E+08	1.23E+08	+	2348	1	SPRY1|hSF	10.4	6.318
NM_1446:	chr1	43269994	43274002	+	1780	1	TMEM125	0.014	0
NM_0012‹	chr12	6444885	6451718	+	1312	1	CD27|S15:	0.197	0.115
NM_0013:	chr2	69643805	69826477	+	3441	1	ANXA4|AN	1.296	1.493
NM_0210‹	chr12	49086656	49094819	-	4696	1	DHH|GDX\	0.187	0.193
NM_0010!	chr4	1.04E+08	1.04E+08	-	1746	1	TACR3|HH	0	0.322
NM_0220:	chr1	26890488	26900471	-	2120	1	GPATCH3|	9.609	9.885
NM_0011!	chr7	26360884	26374329	+	2423	1	SNX10|OP	7.088	3.612
NM_0012(	chr1	19596977	19658456	+	2019	1	MINOS1-N	11.303	22.957
NM_0012:	chr12_KI2	366121	370959	+	4836.7	3	SMIM10L1	0.746	0.651
NM_1525!	chr15	38252851	38357249	+	7246	1	SPRED1|N	2.089	2.459
NM_0175‹	chr1	1.67E+08	1.67E+08	+	5799	1	POGK|BAS	27.119	31.258
NR_11045	chrX	53094157	53144067	+	2420	1	KANTR|LIN	0.464	0.657
NM_0211!	chr16	3014712	3018187	-	1366	1	CLDN6|-|-	8.599	3.329
NR_00330	chr15	25199448	25199529	+	81	1	SNORD115	0	0
NM_0012!	chr2	2.03E+08	2.03E+08	-	872	1	ICA1L|ALS	1.612	2.136
NM_0017(	chr7	73536353	73557735	-	1703	1	BCL7B|-|-	25.658	25.15
NM_0003‹	chr2	44275458	44320823	+	2308	1	SLC3A1|AT	7.301	7.78
NM_1984(	chr7	4905989	4959213	-	2335	1	MMD2|PA	0.26	0.868
NR_03784	chr1	19293247	19295736	-	2489	1	LOC10050	3.284	2.465
NM_0177:	chr1	99645875	99694541	+	2568	1	PALMD|C1	2.234	3.115
NM_0013(	chr10	6010694	6062370	-	2991	1	IL2RA|CD2	0	0
NR_02978	chr9	1.25E+08	1.25E+08	+	88	1	MIR181B2	0	0

NM_0221	chr6_GL0C	3370071	3371654	-	1340	4	FKBPL|DIR	0	0
NM_1452	chr15	72118290	72140970	+	1565	1	SENP8|DE	2.554	2.844
NM_2075	chr15	45114325	45118103	+	1430	1	DUOXA2|	0.117	0.067
NM_0012	chr17	4556927	4560596	-	1954	1	GGT6|-|-|	0	0.007
NM_0043	chr8	99877995	99894014	-	915	1	COX6C|-|-	143.632	158.436
NR_00154	chrY	9329880	9334832	-	257	1	TTTY20|N	0	0
NR_11029	chr2	2.15E+08	2.15E+08	+	2236	1	LOC10192	0.006	0.067
NR_00367	chr20	26103416	26114041	-	417	1	NCOR1P1|	0	0
NM_2073	chr12	56468517	56470983	+	2101	1	SPRYD4|-|	4.253	3.651
NM_1447	chr1	1.57E+08	1.57E+08	+	1113	1	NAXE|AIB	68.001	64.609
NM_0306	chr19	41193207	41207539	+	2613	1	CYP2S1|C	0.242	0.11
NM_0011	chr9	33252471	33264761	-	3864	1	BAG1|BAG	21.803	19.826
NR_02441	chr10	10934940	10952163	-	1037	1	LINC0071C	0	0
NR_02675	chr21	13843133	13848364	-	1273	1	CYP4F29P	0	0
NM_0012	chr6	1.37E+08	1.37E+08	-	3373	1	IL20RA|CR	0.008	0
NR_04748	chr13	94960041	94961319	+	1278	1	LINC00557	0.02	0
NR_10672	chr5	1510762	1510856	-	94	1	MIR6075|	0	0
NR_11054	chr22	26903292	26920610	+	561	1	LINC01422	0.253	0.024
NM_0144	chr4	94298556	94342876	-	1607	1	HPGDS|GS	0	0.009
NM_0011	chr14	91114013	91225396	+	2765	1	C14orf159	11.173	14.574
NM_0027	chr11	14504876	14520445	-	1242	1	PSMA1|H	86.299	93.365
NM_0048	chr13	99501374	99564048	+	3496	1	TM9SF2|P	36.018	39.555
NR_02427	chr7	45723787	45769018	-	2224	1	SEPT7P2|	3.391	3.615
NM_1768	chr1	1.53E+08	1.53E+08	+	4274	1	S100A7A|	0	0
NM_0010	chr11	92352096	92896469	+	19020	1	FAT3|CDH	4.576	3.841
NM_0013	chr9	1.14E+08	1.14E+08	+	9277	1	ZNF618|F	21.581	15.098
NM_1749	chr14	60435956	60486046	-	2743	1	C14orf39|	0	0
NR_10426	chr9	1.06E+08	1.06E+08	+	7492	1	RALGAPA1	1.292	1.538
NM_0011	chr3	1.23E+08	1.23E+08	-	5100	1	ADCY5|AC	13.475	22.184
NM_0013	chr16	10743869	10769349	+	1188	1	NUBP1|NE	22.032	17.502
NR_13501	chr12	95858237	95858839	-	435	1	LOC10536	0.059	0.252
NM_1523	chr1	35713876	35719189	-	2930	1	C1orf216|	0.436	5.483
NM_0012	chr1	1.1E+08	1.1E+08	+	3665	1	AHCYL1|D	36.538	43.606
NM_0012	chrX	40735396	40738701	+	900	1	MED14OS	0.974	0.746
NR_10417	chr13	44400250	44405984	-	1075	1	TUSC8|LIN	0.024	0.051
NR_03031	chr5	1.69E+08	1.69E+08	-	93	1	MIR585|M	0	0
NM_0033	chr5	1.34E+08	1.34E+08	-	1982	1	VDAC1|PC	89.441	77.248
NM_0323	chr18	35241030	35258433	+	1475	1	ZNF397|Z	12.928	14.152
NM_0010	chr14	20640696	20641691	-	994	1	OR6S1|OR	0	0
NR_10413	chr6	95560096	95577451	-	492	1	MANEA-AS	0.812	0.445
NM_0011	chr12	43758944	43789543	+	3965	1	IRAK4|IPD	1.86	2.152
NM_1526	chr10	1.33E+08	1.33E+08	+	6754	1	KNDC1|C1	4.816	8.483
NR_10677	chr6_GL0C	3475011	3475097	-	86	7	MIR6721|	0	0
NR_03988	chr17	38825838	38825892	+	54	2	MIR4727|	0	0
NM_0013	chr7	93130054	93148388	-	6771	1	SAMD9L|	0.015	0.073
NM_0072	chr17	43044295	43124115	-	3658	1	BRCA1|BR	0.99	0.813
NM_0056	chr3	1.7E+08	1.7E+08	+	2756	1	CLDN11|O	0.089	0.065
NR_10999	chr20	319687	325252	-	1391	1	NRSN2-AS	2.53	2.068
NR_13277	chr3	48604920	48605150	-	230	1	SNORA94|	0	0
NM_0010	chr7	38259643	38273647	-	1021	1	TARP|CD3	0	0

NM_0069(	chr7	6374495	6403967	+	2333	1	RAC1|MIG	93.659	103.717
NR_02434	chr9	1.38E+08	1.38E+08	+	1750	1	EHMT1-IT1	0.147	0.078
NR_03971	chr11	90555774	90555857	-	83	1	MIR4490|-	0	0
NR_03389	chr19	22596257	22603550	+	2023	1	GOLGA2P9	0	0
NM_1523:	chr12	1.05E+08	1.05E+08	+	616	1	C12orf45|	8.121	9.672
NM_0028:	chr15	40695129	40732158	+	2287	1	RAD51|BR	1.934	1.156
NR_03618	chr12	97995383	97995448	-	65	1	MIR4303|-	0	0
NM_0021:	chr6_GL0C	4128937	4135377	-	1389	8	HLA-DMB|	0	0
NM_0010(	chr11	35138870	35232402	+	4578	1	CD44|CDV	1.197	1.93
NR_00145	chr11	1.23E+08	1.23E+08	-	86	1	SNORD14D	1.949	2.389
NM_0309!	chr5	1.5E+08	1.5E+08	-	3997	1	TIGD6|-|-|	2.761	2.865
NR_02659	chr5	34905261	34915626	-	4519	1	RAD1|HRA	8.802	9.005
NM_0045!	chr20	17613678	17682283	-	3688	1	RRBP1|ES/	6.947	6.039
NM_2033	chr15	48823737	48963444	-	4542	1	SHC4|RaLF	1.38	0.956
NM_0010:	chr1	46203334	46221256	+	1845	1	LURAP1|C	1.775	3.363
NR_13550	chr15	90904190	90922585	+	6272	1	MAN2A2|I	12.926	18.257
NM_1785:	chr19	52113091	52139938	-	4350	1	ZNF616|-|	2.35	4.071
NR_13463	chr6	85387219	85390186	-	2511	1	LOC10192:	0	0
NM_0159!	chr14	67337864	67360003	-	1795	1	ATP6V1D|	55.661	61.312
NM_0013:	chr10	87050202	87094087	-	2921	1	GLUD1|GD	22.41	22.128
NR_03966	chr5	1.35E+08	1.35E+08	+	73	1	MIR4461|i	1.236	0.188
NM_0150:	chr20	41178449	41300099	-	9985	1	ZHX3|TIX1	4.615	9.31
NM_0049:	chr1	19338773	19485641	-	1768	1	CAPZB|CA	165.515	167.773
NR_02449	chr1	25819954	25823606	-	3652	1	LOC64647	4.558	5.622
NM_0153!	chr12	1.33E+08	1.33E+08	+	4396	1	ZNF10|KO	2.989	3.434
NM_0251(	chr16	2460114	2464963	+	1616	1	C16orf59|	6.79	4.179
NM_1768:	chr3	94057922	94062987	-	3923	1	DHFR2|DH	2.107	3.038
NM_0012	chr16	89321133	89325348	+	1438	1	LOC10028	0	0.019
NM_0067(	chr6	34287196	34392680	-	2387	1	NUDT3|DI	48.257	69.225
NM_0007(	chr13	1.14E+08	1.14E+08	-	1489	1	ATP4B|AT	0.035	0.083
NR_03036	chr15	75353611	75353685	-	74	1	MIR631|M	3.31	3.517
NR_03787	chr8	1.23E+08	1.23E+08	-	5033	1	ZHX1|-|-|!	5.08	7.743
NM_0174:	chr16	81238691	81291142	+	2414	1	BCO1|BCD	1.479	0.17
NM_0013(	chr4	1692731	1712741	-	2106	1	SLBP|HBP	20.883	24.227
NM_1706(	chr7	1.52E+08	1.52E+08	-	16801	1	KMT2C|HA	4.875	9.938
NM_0071(	chr19	2236817	2248679	+	1639	1	SF3A2|PRF	84.929	72.647
NR_03021	chr19	53741313	53741407	+	94	1	MIR517C|	0	0
NR_10465	chr16	74192392	74215521	-	962	1	LOC10192:	0	0.028
NM_0052!	chr16	86567251	86568931	+	1678	1	FOXC2|FKI	0.077	0.057
NR_13121	chr10	90947300	90992132	+	498	1	XLOC_008.	0	0
NM_0010	chr2	2.02E+08	2.02E+08	-	5413	1	TMEM237	9.054	8.55
NM_0043	chrX	1.53E+08	1.53E+08	-	1073	1	CETN2|CA	73.468	59.115
NM_0011!	chr9	6215786	6257983	+	2571	1	IL33|C9orf	0.236	3.719
NM_0045!	chr5	1.37E+08	1.37E+08	-	4815	1	SPOCK1|SI	6.973	17.736
NM_0144:	chr20	45373880	45378317	-	974	1	TP53TG5|(	1.919	1.688
NR_13190	chr3	6892637	6894017	-	508	1	GRM7-AS2	0	0
NM_0009!	chr3	1.98E+08	1.98E+08	+	476	1	RPL35A|DI	812.235	680.448
NM_0012:	chr4	40193649	40244764	+	1867	1	RHOH|ARH	0.566	0.103
NR_10385	chr16	86196181	86199718	+	437	1	LINC01082	0	0
NM_0013:	chr11	47572197	47579015	-	2392	1	KBTBD4|B	29.587	30.228

NM_0013(	chr19	51263883	51269328	+	1227	1	SIGLECL1|(	0	0
NR_13493	chr19	2458937	2462185	-	367	1	LINC01775	0	0.075
NM_0323:	chr3	1.84E+08	1.84E+08	+	985	1	ECE2|-|-|3	4.922	3.018
NM_0175{	chr11	35662752	35811053	+	6056	1	TRIM44|A	23.839	23.61
NM_0149{	chr5	1.5E+08	1.5E+08	+	5384	1	HMGXB3|I	13.439	17.307
NR_13456	chr20	58773425	58774811	+	542	1	LOC10537.	0	0
NM_0010(	chr7	1.08E+08	1.08E+08	+	5763	1	BCAP29|B.	1.62	1.704
NM_0012.	chr2	2.19E+08	2.19E+08	+	1059	1	ZFAND2B|	23.973	29.502
NM_0312{	chr6	1.44E+08	1.44E+08	-	734	1	SF3B5|SF3	139.843	124.71
NR_04538	chr8	1.3E+08	1.3E+08	-	2030	1	ASAP1-IT2	0.064	0.034
NM_0012!	chr7	31529462	31658720	+	3807	1	CCDC129|-	0	0.115
NR_04499	chr6_GL0C	4443936	4449287	+	697.7	7	HCG25|dJ:	0	0
NM_0011:	chr4	1.08E+08	1.08E+08	+	6138	1	SGMS2|SN	0.074	0.069
NM_0043:	chr4	15702950	15732173	+	1469	1	BST1|CD1!	0.518	0.569
NM_0186{	chr19	11239619	11241943	+	866	1	ANGPTL8|	0.06	0.079
NM_0013.	chr2	95085391	95121557	-	3302	1	MRPS5|M	18.313	17.791
NM_0017.	chr20	38304150	38337503	+	1828	1	BPI|BPIFD	0.268	0.082
NM_0247{	chr11	47659591	47715376	-	3572	1	AGBL2|CC	0.018	0.004
NM_0003{	chr3	1.22E+08	1.22E+08	+	4906	1	CASR|CAR	0.005	0.003
NM_1526:	chrX	80420447	80445311	+	3025	1	FAM46D|(	0	0
NM_1785.	chr19	8697397	8698830	-	1431	1	ACTL9|HSI	0	0
NM_1817.	chr8	17027238	17122639	+	3971	1	MICU3|EF	1.13	1.404
NM_0037(	chr2	1.72E+08	1.72E+08	-	3978	1	SLC25A12|	8.492	12.774
NM_0163.	chr1	1.63E+08	1.63E+08	+	1512	1	HSD17B7|	6.686	9.159
NM_0018‹	chr1	23873970	23913327	-	1771	1	CNR2|CB-.	0	0
NM_1815(	chr5	68290639	68301821	+	5663	1	PIK3R1|AG	4.012	8.852
NM_2015(	chr1	1.62E+08	1.62E+08	+	2479	1	FCGR2C|C	0	0.017
NM_0529!	chr15	43276281	43302255	-	2297	1	TGM7|TGI	0	0
NR_03398	chr19	34816154	34832869	-	2074	1	LINC01801	0.808	1.242
NM_0013:	chr12	53006258	53042215	+	3921	1	EIF4B|EIF-.	37.316	45.821
NR_02952	chr5	1.69E+08	1.69E+08	-	77	1	MIR103A1	0	0
NR_00217	chrY	22144987	22146834	+	470	2	TTTY6B|LII	0	0
NM_0184.	chr12	10618939	10674292	-	2982	1	STYK1|NO	0.009	0.014
NM_1825‹	chr6	35805294	35824075	+	2141	1	LHFPL5|DI	0.108	0.16
NM_0012!	chr1	87212669	87251331	+	1146	1	LOC10192.	0	0.012
NM_0010‹	chr8	6977649	6980092	-	493	2	DEFA1B|H	0	0
NR_10679	chr1	2.26E+08	2.26E+08	-	62	1	MIR6741|I	9.774	9.279
NM_0220.	chr17	60043191	60078931	-	3913	1	HEATR6|A	8.913	9.591
NM_1334(	chr7	2679522	2716529	+	5507	1	AMZ1|-|-|	0.173	0.266
NM_1750!	chr12	52565819	52573825	-	2777	1	KRT74|AD	0	0
NM_0000!	chr2	2.27E+08	2.27E+08	+	8043	1	COL4A3|-|	0.043	0.017
NM_0021.	chr6	32517377	32530229	-	1160	1	HLA-DRB5	0	0
NM_0011(	chr11	65538559	65558228	-	4528	1	LTBP3|DA!	7.572	17.064
NM_0011.	chr1	54781079	54801268	-	1970	1	TTC22|-|-|	0	0
NM_1830(	chr17	8172979	8176396	-	1750	1	TMEM107	14.824	8.273
NM_0012(	chr1	1.6E+08	1.6E+08	-	2192	1	IGSF8|CD3	45.427	61.505
NR_12044	chr11	1.34E+08	1.34E+08	-	972	1	LOC64652.	0	0.085
NM_0208:	chr14	60971449	60981079	-	5289	1	TRMT5|CC	2.255	2.647
NR_02676	chr20	33193605	33210462	+	1194	1	BPIFA4P|E	0	0
NM_0325(	chr5	80319971	80321841	+	1868	1	SPZ1|NYD-	0	0

NR_03894	chr22	25102417	25112692	-	968	1	LOC10012	0.4	0.594
NM_0013	chr22	29259852	29268163	-	1629	1	RHBDD3|C	24.054	20.768
NM_1384	chr7	7968743	8089079	+	4783	1	GLCCI1|FA	11.489	8.686
NR_04768	chr13	1E+08	1E+08	-	458	1	PCCA-AS1|	0	0.06
NR_02793	chr12	92421531	92431002	+	2272	1	CLLU1|-|-|	0	0.006
NM_0806	chr5	1.34E+08	1.34E+08	-	1249	1	CDKN2AIP	24.053	23.687
NM_0011	chr22_KI2	138079	161750	-	1977	2	PRODH|HS	0.372	0.152
NR_00297	chr1	1.56E+08	1.56E+08	-	133	1	SNORA80E	0.194	0.618
NR_02751	chr10	79682975	79688894	+	5919	1	BEND3P3|	1.758	1.555
NR_03827	chr15	84570649	84580181	+	2676	1	LINC0093	0.039	0.077
NR_13432	chr8	55517188	55520978	-	641	1	LOC10537	0	0
NM_1784	chr20	35285731	35292422	-	3160	1	FAM83C|C	0.004	0
NM_0011	chr11	1732410	1750594	-	3361	2	IFITM10|D	0.541	0.754
NM_0010	chrX	70234655	70240661	+	1390	1	AWAT1|D	0	0
NR_02978	chr20	62554306	62554376	+	70	1	MIR1-1|M	0	0
NR_10346	chr3	51671627	51704323	+	1033	1	TEX264|ZS	24.576	23.774
NM_0011	chr16	28974775	28984548	+	1961	1	SPNS1|HS	30.626	26.605
NM_0060	chr1	10032983	10181238	+	5463	1	UBE4B|E4	23.767	20.813
NM_1383	chr14	24051997	24069728	+	4558	1	CARMIL3|	13.241	15.503
NM_0012	chr4	3441933	3449495	+	2117	1	HGFAC|HG	2.911	2.523
NR_12051	chr7	1738631	1742310	-	672	1	ELFN1-AS1	0.211	0
NR_11056	chr2	67213408	67244574	+	527	1	LOC10192	0.049	0
NM_0018	chr1	2.07E+08	2.07E+08	+	3972	1	CR2|C3DR	0	0
NM_0053	chr1	43337804	43354464	+	3631	1	MPL|C-MF	0.007	0.015
NM_0010	chr5	35904296	35938635	-	965	1	CAPSL|-|-|	0.668	0.114
NM_0013	chr7	1.43E+08	1.43E+08	+	874	2	PRSS2|TRY	0	0
NR_03898	chr6	1.43E+08	1.43E+08	-	1900	1	LINC01277	0.014	0.058
NR_13582	chr14	70187108	70232583	+	2973	1	LOC64654	0	0.005
NM_0012	chr9	1.25E+08	1.25E+08	+	6590	1	GAPVD1|G	8.028	9.904
NM_0010	chr7	64666083	64711582	+	5838	1	ZNF107|Y8	0.161	0.711
NM_0012	chr2	2.06E+08	2.06E+08	-	2155	1	GPR1|-|-|	0.67	2.504
NM_0190	chr4	40423255	40515973	-	4478	1	RBM47|NI	0.446	0.128
NM_0228	chr1	20139330	20150386	+	2712	1	PLA2G2F|-	0	0
NR_10684	chr18	10759584	10759649	-	65	1	MIR6788|	0	0
NM_0011	chr3	12539781	12545464	-	1079	1	MKRN2OS	0.191	0.317
NM_0042	chr9	1.32E+08	1.32E+08	-	1397	1	MED27|CF	17.554	20.756
NM_0012	chr17	50464492	50468866	-	1689	1	CHAD|SLR	0.176	0.219
NM_0144	chr1	62597487	62606305	+	2942	1	ANGPTL3|	0	0.028
NM_0032	chr8	73008862	73047752	+	2889	1	TERF1|PIN	2.343	2.385
NM_0011	chr5	34656328	34832612	+	4880	1	RAI14|NO	10.035	5.277
NM_0010	chr12	55469200	55470138	-	937	1	OR6C70|-|	0	0
NM_1985	chr10	1.16E+08	1.16E+08	+	1688	1	CCDC172|	0.015	0
NM_1533	chr19	12610918	12629862	+	2489	1	ZNF791|-|	5.232	8.199
NM_0010	chrX	52495821	52500811	+	478	2	XAGE1B|C	0	0
NM_0010	chr1	450740	451678	-	937	3	OR4F29|O	0	0
NM_0208	chr7	77122617	77295204	+	3312	1	CCDC146|	0.588	0.893
NM_1527	chr2	69960091	69962265	-	2172	1	ASPRV1|M	2.737	2.68
NM_0012	chr22	20117424	20127357	+	1126	1	RANBP1|H	75.002	66.21
NM_1736	chr4	1.54E+08	1.54E+08	-	1558	1	RNF175|-|	3.939	4.888
NM_0155	chr19	36014660	36032895	-	3356	1	CLIP3|CLIF	161.43	163.254

NM_0013	chr17	29073510	29140379	-	6447	1	MYO18A|I	39.49	43.883
NM_0225	chrX	41447460	41475652	+	2625	1	NYX|CLRP	0.029	0.005
NM_0013	chr7	50437320	50450406	-	10366	1	FIGNL1|-|-	0.968	1.214
NM_0072	chr3	48465520	48467645	+	1624	1	TREX1|AG	4.946	3.323
NR_04636	chr19	14408798	14419383	-	1420	1	DDX39A|B	62.969	44.457
NM_0013	chr1	12746186	12761169	+	929	1	C1orf158|	0.25	0.206
NM_0011	chr11	72685069	72722358	-	4699	1	ARAP1|CE	4.13	5.34
NM_1527	chr7	27525440	27663001	-	1988	1	HIBADH|N	9.904	6.394
NM_0057	chr1	1.84E+08	1.84E+08	-	2071	1	ARPC5|AR	57.949	55.648
NM_0177	chr5	1.41E+08	1.41E+08	+	2571	1	WDR55|-|	9.905	8.615
NR_04610	chrX	46887419	46899704	-	516	1	LINC01545	0.125	0
NM_0010	chr21	30281309	30282958	-	1647	1	KRTAP24-1	0	0
NR_03989	chr18	58451080	58451152	-	72	1	MIR3591|-	0	0
NM_0011	chr13	1.05E+08	1.05E+08	+	917	1	DAOA|LG7	0	0
NM_0011	chr19	53044738	53051127	+	2478	1	ERVV-2|EN	0.021	0.022
NM_0244	chr1	1.2E+08	1.2E+08	-	11430	1	NOTCH2|A	3.712	12.239
NR_02439	chr18	39206924	39751995	-	2143	1	MIR924HG	0	0.281
NM_0012	chr19	21291160	21329412	-	3874	1	ZNF708|K	1.751	1.892
NR_03614	chr16	14901508	14901591	+	83	7	MIR3179-1	0	0
NM_0329	chr11	64269828	64284704	-	964	1	BAD|BBC2	107.16	81.287
NR_03163	chr20	50614636	50614785	-	149	1	MIR1302-5	0	0
NM_0011	chr22	27978014	28679865	-	11770	1	TTC28|TPF	17.786	16.063
NR_02959	chr14	23388596	23388666	-	70	1	MIR208A|	0	0
NM_0041	chr1	89106133	89126159	-	4119	1	GBP2|-|-|	0.019	0.033
NM_0010	chr22	39487224	39492196	+	4970	1	MGAT3|GI	22.695	23.982
NM_0013	chr5	1.74E+08	1.74E+08	+	8365	1	CPEB4|CPI	6.008	7.164
NM_0064	chr17	41801953	41812199	-	2592	1	P3H4|LEPI	15.674	13.866
NR_03973	chr15	35926856	35926923	+	67	1	MIR4510|	0	0
NR_02669	chrX	1.04E+08	1.04E+08	+	1260	1	ZCCHC18|	10.028	10.61
NR_12576	chr11	27047186	27218768	-	500	1	BBOX1-AS1	0.077	0.11
NM_0013	chr4	1.09E+08	1.09E+08	+	557	1	RPL34|L34	290.925	287.957
NR_04600	chr15	33236476	33247555	-	848	1	TMCO5B|-	0	0
NM_1526	chr5	72439899	72507422	-	6271	1	ZNF366|D	0.004	0.004
NM_1529	chr4	70226071	70235251	+	558	1	FDCSP|C4	0	0
NM_0056	chr21	41464551	41508158	-	3188	1	TMPRSS2|	0	0.026
NM_0012	chr1	27404226	27490187	-	5180	1	WASF2|IM	13.08	10.68
NR_10992	chr21	38857539	38858862	+	1050	1	LOC10192	0	0
NM_1446	chr1	1.79E+08	1.8E+08	+	3614	1	AXDND1|C	0	0.015
NM_0010	chr9	35041095	35045991	+	3263	1	C9orf131|	0.095	0.113
NM_0064	chr8	1.19E+08	1.19E+08	+	3187	1	COLEC10|	0.073	0.09
NM_1817	chr12	1.24E+08	1.24E+08	+	2145	1	RFLNA|CFI	1.533	3.212
NM_0012	chr12	57693955	57721557	+	2681	1	OS9|ERLE	101.921	88.471
NR_03606	chr15_KI2	164332	164406	+	74	4	MIR3118-3	0	0
NM_0018	chr4	67472271	67545538	-	3328	1	CENPC|CE	0.786	1.416
NR_03022	chrX	50009722	50009805	+	83	1	MIR501|M	0	0
NM_0013	chr17	2776921	3037741	+	6529	1	RAP1GAP2	7.725	6.633
NR_13525	chr14	28783491	28785787	-	1653	1	LOC10537	0.008	1.152
NM_0163	chr16	1938229	1943326	-	1398	1	MSRB1|HS	15.799	15.519
NM_0011	chr15	78266218	78282196	+	2865	1	DNAJA4|M	0.225	0.32
NR_12034	chr15	63928274	63935953	+	1068	1	LOC10192	0	0.064

NM_1527:	chr6	1.16E+08	1.16E+08	+	3115	1	NT5DC1|C	3.738	0.976
NM_0010(	chr17	14235856	14236833	-	563	1	CDRT15|-|	0.046	0.024
NR_02687	chr6	1.25E+08	1.25E+08	-	5335	1	RNF217-AS	0.075	0.072
NM_0011!	chr7	1.51E+08	1.51E+08	+	3928	1	SLC4A2|AE	28.958	39.32
NM_0121{	chr1	47416072	47418052	+	1978	1	FOXE3|CTI	0.15	0.09
NM_0320!	chr22	31325804	31346263	-	3668	1	PATZ1|MA	37.689	36.509
NM_0807!	chr13	71437966	71867192	-	5220	1	DACH1|DA	21.536	10.034
NM_0013:	chr22	16783416	16821699	-	1683	1	XKR3|XRG	0	0
NM_0208	chr12	76351798	76559809	-	7215	1	OSBPL8|M	6.085	6.479
NM_0010	chr17	43640390	43661894	-	2162	1	MEOX1|KF	0.453	0.412
NM_0211{	chr5	1.44E+08	1.44E+08	+	380	1	HMHB1|H	0	0
NR_11062	chr20	33787373	33811099	-	642	1	ZNF341-AS	0.241	0.171
NR_13505	chr16	31563222	31567715	-	929	1	FRG2KP|-|	0	0
NM_1828:	chr14	24265538	24271627	-	1957	1	RABGGTA|	20.404	17.05
NM_0162	chr4	87336525	87391303	-	1878	1	HSD17B11	10.793	11.597
NR_12164	chr4	40316485	40330419	+	954	1	LOC10106	0.189	0.287
NM_0010:	chr6	99542387	99568814	-	2071	1	CCNC|Cyc	9.6	12.288
NM_0123:	chrX	70290029	70420924	+	4468	1	KIF4A|KIF4	3.215	2.241
NR_03061	chr6	30584332	30584417	+	85	7	MIR877|M	0	0
NR_10686	chr19	55245186	55245250	-	64	1	MIR6803|	0	0
NM_0806(	chr2	55519595	55545080	+	1296	1	CFAP36|BA	44.371	25.756
NM_0330!	chrX	72845042	72848802	+	1092	2	DMRTC1|-	0	0
NM_1334!	chr19	6306499	6333629	-	1080	1	ACER1|ALI	0.012	0.013
NR_02987	chr14	1.01E+08	1.01E+08	+	75	1	MIR382|M	0	0
NM_2069:	chr1	25800176	25818222	+	4216	1	SELENON|	124.299	60.363
NR_03972	chr14	50299855	50299946	-	91	1	MIR4504|	0	0
NR_11079	chr18	23994215	24015460	-	930	1	TTC39C-AS	0.499	0.707
NM_0011:	chr11	59755059	59805882	+	6349	1	STX3|STX3	1.578	3.469
NR_02692	chr22	30922308	30926653	+	2131	1	MORC2-AS	6.268	7.597
NR_04700	chr13	60672955	60695800	+	508	1	LINC00378	0	0
NM_0011	chr8	98944403	98952104	+	1871	1	OSR2|-|-|	0.007	0
NM_2073(	chr9	1.37E+08	1.37E+08	+	3354	1	UAP1L1|-|	1.58	1.683
NR_04609	chr20	60138492	60322256	+	2038	1	MIR646HG	0	0
NR_13559	chr2	56074654	56090382	-	3473	1	LINC01813	0	0
NM_0012{	chr11	65088372	65089109	-	507	1	TMEM262	0.203	0.135
NM_0053{	chr8	24913761	24919093	+	3266	1	NEFM|NEF	133.763	48.728
NR_03174	chr8	1.13E+08	1.13E+08	+	90	1	MIR2053|	0	0
NM_0012	chr9	72149351	72252224	+	2057	1	GDA|CYPII	0.025	0.04
NR_02655	chr16	46469337	46569097	-	6106	1	ANKRD26F	0.025	0
NM_0011:	chr3	1.27E+08	1.27E+08	-	2794	1	TXNRD3|T	1.874	1.691
NR_02655	chr1	1.68E+08	1.68E+08	-	2387	1	MPC2|BRF	20.699	22
NM_0186(	chr16	68358327	68448506	-	5258	1	SMPD3|NS	31.461	24.422
NR_02832	chr1	490756	495445	-	4367	2	LOC10013	0.03	0.072
NM_0011(	chr3	38454299	38493142	+	11360	1	ACVR2B|A	14.266	5.228
NM_0010(	chr22	42074251	42079438	+	1916.8	4	FAM109B|	0.087	0.079
NM_0189:	chr5	1.41E+08	1.41E+08	+	2772	1	PCDHB2|P	17.066	17.497
NM_0069:	chr9	74497336	74687201	+	3592	1	RORB|NR1	1.87	1.373
NR_03171	chr2	2.32E+08	2.32E+08	-	56	1	MIR1471|	0	0
NM_1526(	chr1	2.47E+08	2.47E+08	+	5220	1	CNST|C1or	6.301	6.109
NR_04008	chr2	11099851	11132176	-	1626	1	FLJ33534|	0	0.059

NM_0062:	chr13	78599095	78603560	-	3810	1	POU4F1|B	11.553	6.104
NM_0156:	chr17	7561992	7571969	+	2481	1	SENP3|SM	41.528	46.971
NR_00484	chr15	82355136	82415611	+	1522	2	UBE2Q2P2	0.059	0.027
NM_0042(	chr19	11322046	11339668	-	4266	1	RAB3D|D2	18.981	17.505
NM_0528:	chr9	1.34E+08	1.34E+08	+	3055	1	WDR5|BIG	26.614	28.905
NR_00359	chrY	6471031	6473630	-	528	2	TTTY8B|N(	0	0
NM_0010(	chr3	1.12E+08	1.12E+08	+	1194	1	TAGLN3|N	676.573	471.32
NM_0319‹	chr5	1.41E+08	1.41E+08	-	1432	1	SLC25A2|(	0.054	0.077
NM_0032:	chr6	35473597	35497084	-	2976	1	TEAD3|DT	10.857	13.435
NM_0010:	chr12	95030409	95073628	-	1856	1	NR2C1|TR	6.343	6.561
NR_11083	chr17	75047184	75065889	+	3575	1	KCTD2|-|-	34.129	43.371
NM_0011(	chr5	1.31E+08	1.32E+08	-	8036	1	RAPGEF6|	3.167	3.62
NR_03160	chr11	64368602	64368703	+	101	1	MIR1237|I	0	0
NR_12063	chr10	14074284	14087879	+	1061	1	LOC10192:	0.061	0.052
NM_0016!	chr2	70935868	70965431	+	1923	1	ATP6V1B1	0.282	0.584
NR_13191	chr13	88142867	88236082	+	556	1	LOC10537(	0	0
NR_13448	chr5	1.27E+08	1.27E+08	-	910	1	LOC10272:	0.368	0.452
NR_11012	chr17	72030654	72041297	+	3575	1	LINC01152	0.307	0.506
NR_13693	chr5	5140330	5320302	+	4824	1	ADAMTS1(	0.326	0.287
NM_0786:	chr1	50969970	50974637	+	1269	1	CDKN2C|II	1.524	2.094
NM_0011:	chr3	13480344	13506424	+	2805	1	HDAC11|H	15.091	13.653
NM_0050(	chr16	1944579	1954678	-	1517	1	RPL3L|-|-|	0.068	0.018
NM_0010:	chr11	1870924	1892263	+	1508	1	LSP1|WP3	0.154	0
NM_1450:	chr11	69004394	69013382	-	2267	1	MRGPRF|(	0.023	0.012
NM_0011‹	chr2	1.11E+08	1.11E+08	+	2353	1	ACOXL|-|-	0.104	0.256
NM_0029!	chr9	1.34E+08	1.34E+08	+	5518	1	RXRA|NR2	7.335	9.107
NM_0023:	chr8	23296897	23404209	-	3794	1	LOXL2|LOI	2.895	1.949
NM_0313(	chr4	82353314	82373996	-	2190	1	HNRNPD|/	113.722	108.463
NM_0013:	chr8	1.44E+08	1.44E+08	-	1308	2	TSTA3|FX|	0	0
NM_0012(	chr9	33921693	34048949	-	3943	1	UBAP2|UE	16.248	14.576
NM_0013:	chr19_KI2	38286	52629	-	1812.2	30	KIR3DL1|C	0	0
NM_0005‹	chr19	38433700	38587564	+	15283	1	RYR1|CCO	0.562	0.273
NM_1387‹	chr1	1.54E+08	1.54E+08	-	1741	1	C1orf43|H	191.484	179.485
NR_03811	chr17	76557764	76565348	+	2432	1	SNHG16|N	10.412	13.979
NM_0010(	chrX	1.21E+08	1.21E+08	-	1288.7	10	CT47A9|C	0	0
NM_0013(	chr1	1.5E+08	1.5E+08	+	1196	1	CIART|C1c	5.369	4.054
NM_1530:	chr10	47365496	47384338	-	3557	1	ZNF488|-|	3.193	7.582
NM_0529:	chr17	1779535	1829881	-	4321	1	SMYD4|ZN	6.287	6.999
NM_0158‹	chr1	15476101	15491400	+	913	1	CELA2B|EL	0.028	0.18
NR_04631	chr22	30368811	30377905	-	7603	1	KIAA1656|	1.808	1.551
NR_13364	chrX	17970173	18104644	-	727	1	LINC0145€	3.316	4.126
NR_10807	chr10	6774303	6779180	-	701	1	LINC0070€	0	0
NM_0163:	chr3	1.79E+08	1.79E+08	+	3007	1	ZNF639|AI	5.896	7.821
NM_0010:	chr8	1.43E+08	1.43E+08	-	3586	1	JRK|JH8|je	3.79	3.655
NM_0061(	chr12	85874295	85882992	+	1233	1	NTS|NMN·	0.544	49.023
NR_12542	chr8	1.34E+08	1.34E+08	-	1038	1	LOC10192	0	0
NR_12605	chr13	19182033	19187465	+	571	1	LOC10192:	0	0
NM_2015!	chr12	69608565	69611162	-	2595	1	LRRC10|HI	0	0
NM_0175(	chr11	1.11E+08	1.12E+08	-	1005	1	BTG4|APR	0.013	0.014
NM_0013:	chr4	1.09E+08	1.09E+08	-	1986	1	ETNPPL|A(	0.162	0.124

NM_0010(	chr17	59331692	59401734	+	5225	1	YPEL2|FKS	2.986	4.252
NR_13528	chr6	28984749	28988536	+	673	7	HCG16|HC	0	0
NM_0161:	chr16	1493351	1510459	+	3305	1	TELO2|CLK	12.722	11.169
NM_0012!	chr9	27948086	29213000	-	3147	1	LINGO2|LE	2.479	3.578
NR_00281	chr12	9648047	9658414	+	2610	1	LOC37444:	1.275	1.191
NR_13121	chr12	1.21E+08	1.21E+08	+	496	1	XLOC_009!	0	0
NM_0011!	chr22	38957522	38992778	+	1096	2	APOBEC3A	0	0
NM_0011:	chr17	41046541	41047316	-	772	2	KRTAP2-1|	0	0
NR_03983	chr1	5862672	5862741	-	69	1	MIR4689|	0	0
NR_10419	chr14	1.01E+08	1.01E+08	+	422	1	MIR381HG	0	0
NR_04750	chr12	53935328	53939643	-	1405	1	HOXC13-A	0	0.039
NM_0017!	chr11	3000922	3057451	-	2499	2	CARS|CAR	1.053	1.694
NM_0048{	chr15	65327127	65378040	-	4463	1	IGDCC3|H!	17.415	14.872
NR_00321	chr14	1.01E+08	1.01E+08	+	70	1	SNORD114	0	0
NM_0322{	chr19	11451328	11480988	-	4652	1	ELAVL3|HU	75.72	87.579
NM_0309‹	chr6	29306690	29307655	+	964	7	OR14J1|O	0	0
NM_0324!	chr6_GL0C	3314930	3315373	+	1165.8	10	STK19|D6!	0	0
NM_0013(	chr12	1.13E+08	1.13E+08	+	4919	1	TPCN1|TP(	3.402	3.085
NR_11007	chr1	20184243	20186486	-	507	1	UBXN10-A	0.305	0.162
NM_0011‹	chr9	1.33E+08	1.33E+08	-	2349	1	SLC2A6|GI	16.955	12.484
NM_0012!	chr8	27491168	27544922	+	2086	1	EPHX2|CEI	9.457	5.693
NM_1525:	chr2	2.08E+08	2.08E+08	+	3646	1	CCNYL1|-|	4.332	4.054
NM_0206‹	chr11	8920076	8933006	-	1916	1	C11orf16|	0.074	0.114
NM_0011:	chr10	1.03E+08	1.03E+08	-	3416	1	NT5C2|GN	34.487	31.231
NM_0007:	chr9	1.38E+08	1.38E+08	+	9742	1	CACNA1B|	9.856	8.71
NM_0013:	chr12	1.21E+08	1.21E+08	+	3842	1	RNF10|RIE	71.548	85.822
NM_0011(	chr7	5527148	5530601	-	1804	1	ACTB|BRW	2391.054	2193.747
NM_0013:	chr13	25468774	26025851	+	9311	1	ATP8A2|A	4.161	5.391
NM_0012‹	chr5	39371674	39425233	-	4522	1	DAB2|DO(	0.73	0.242
NM_1818(	chr1	6264272	6358944	-	1403	1	ACOT7|AC	169.499	154.631
NM_0010:	chr12	1820267	1836752	+	3943	1	LRTM2|-|-	0.128	0.333
NM_0307{	chr5	1317744	1345070	-	2174.5	2	CLPTM1L|	8.876	9.304
NM_0010!	chr3	11154528	11263253	+	4274	1	HRH1|H1-	0.118	0.24
NM_0804:	chr1	3021482	3022903	+	1419	1	ACTRT2|A	0	0
NR_13625	chr6	83709442	83726495	+	520	1	LOC10537	0.025	0.026
NR_12616	chr17	20417292	20419050	+	1758	1	FAM106B|	0.015	0.078
NM_0805:	chr3	15450133	15521751	-	2885	1	COLQ|CM!	1.39	2.246
NR_10467	chr5	1.07E+08	1.07E+08	-	568	1	LINC0195C	0	0
NR_12596	chr1	1.76E+08	1.76E+08	+	1802	1	LINC01657	0	0.008
NM_0243(	chr19	46746046	46758575	+	3416	1	FKRP|LGM	10.467	10.409
NR_13580	chr12	51848223	51852729	+	424	1	LOC10536!	0.912	0.485
NM_0056!	chr22	19449941	19479215	-	1769	1	UFD1L|UF	32.296	30.065
NR_03338	chr2	63043966	63048521	-	1881	1	LOC10013:	5.689	3.612
NM_0013(	chr7	94585224	94656209	-	1565	1	SGCE|DYT	23.159	18.905
NR_07337	chr3	1.28E+08	1.28E+08	+	3490	1	MCM2|BN	23.452	18.595
NM_0031‹	chrY	2786855	2787741	-	884	1	SRY|SRXX1	0.671	0.062
NR_10344	chr5	37840438	37874892	+	598	1	GDNF-AS1	0.28	0.092
NR_03971	chr11	74399237	74399333	+	96	1	MIR548AL	0	0
NM_0120{	chr10	97332497	97334701	-	2202	1	FRAT2|-|-	8.098	10.593
NM_1990(	chr7	1.04E+08	1.05E+08	+	3171	1	LHFPL3|LH	1.118	0.842

NR_12033	chr15	45251581	45279222	-	667	1	LOC10192	0.116	0.082
NM_0190	chr16	83968632	84002774	+	1591	1	NECAB2|E	170.038	24.097
NM_0010	chr17	20449863	20467535	-	1230	1	LGALS9B|-	0	0
NR_00335	chr15	25241746	25241827	+	81	1	SNORD115	0	0
NR_03980	chr8	9048445	9048518	+	73	1	MIR4660|	0	0
NM_0133	chr14	1.05E+08	1.05E+08	-	3774	1	GPR132|G	0.014	0.102
NM_1830	chr1	2.28E+08	2.28E+08	+	1236	1	PRSS38|M	0	0
NM_0141	chr16	9091680	9119698	+	3973	1	C16orf72|	9.22	9.484
NR_03156	chr5	1.76E+08	1.76E+08	+	85	1	MIR1271|	0	0
NR_04753	chr19	51167328	51173524	+	1485	1	SIGLEC17P	0	0
NM_0156	chr2	27444373	27489811	-	5417	1	IFT172|BB	9.099	7.022
NR_13298	chr18	55452533	55482941	+	395	1	TCF4-AS1|	0.065	0
NM_0225	chr6	33272075	33276504	+	469.2	6	RPS18|D6	0.055	0
NR_03160	chr2	1.32E+08	1.32E+08	-	114	1	MIR663B|	0	0
NR_12538	chr8	6615603	6708209	-	4127	1	MCPH1-AS	1.387	1.832
NM_0012	chr17	19089461	19097563	+	1909.7	3	LOC79999	0	0
NM_1785	chr7	1.29E+08	1.29E+08	+	2927	1	TSPAN33|	7.066	10.267
NM_0006	chr12	68154770	68159741	-	1204	1	IFNG|IFG|	0	0
NM_0067	chr16	67893272	67929678	+	3476	1	PSKH1|-|-	13.064	15.912
NR_03341	chr10	71319253	71363390	+	1953	1	SLC29A3|E	2.74	3.268
NM_0010	chr19	34926903	34945172	+	2580	1	ZNF30|KO	4.193	5.251
NM_0006	chr20	4220631	4249012	-	2662	1	ADRA1D|	0.107	0.149
NM_0010	chr9	41131309	41189364	-	1702	1	CBWD6|-|	0.5	0.507
NM_0068	chr20	59028678	59032367	-	434	1	ATP5E|AT	336.478	274.824
NM_2033	chr9	1.37E+08	1.37E+08	-	753	1	LCN15|PR	0	0
NM_0012	chr5	1.6E+08	1.6E+08	+	1469	1	TTC1|TPR	33.125	35.291
NM_0183	chr12	1.02E+08	1.02E+08	+	3534	1	DRAM1|D	0.427	0.725
NR_00298	chr1	39567374	39567510	-	136	1	SNORA55|	0.474	1.007
NM_0244	chr19	1383884	1395589	+	776	1	NDUFS7|C	91.633	70.709
NM_0011	chr1	1.6E+08	1.61E+08	-	2404	1	SLAMF6|C	0	0
NM_0245	chr1	44653247	44674608	-	2358	1	TMEM53|	4.424	4.571
NM_0023	chrX	30215558	30220089	+	1608	1	MAGEB2|	0	0
NR_02962	chr1	2.09E+08	2.09E+08	+	109	1	MIR205|M	0	0
NM_0042	chr2	74654228	74683854	+	6091	1	SEMA4F|M	10.736	10.398
NM_0010	chr17	49838309	49848017	-	560	1	TAC4|EK|	0	0.024
NM_0159	chr8	1.38E+08	1.38E+08	-	6940	1	FAM135B|	0	0.002
NM_0022	chr17	41500981	41505613	-	1680	1	KRT13|CK	0	0
NR_02681	chr21	35037931	35039426	-	1495	1	RUNX1-IT1	0	0
NM_1527	chr12	1.22E+08	1.22E+08	+	1900	1	LRRC43|-|	0.482	0.223
NR_02693	chr20	35520742	35529652	-	2109	1	C20orf173	0.049	0.026
NR_11012	chr7	20328301	20331762	-	813	1	LOC10192	0.777	0.708
NM_0010	chrY	22984263	23005463	+	1193.7	3	BPY2B|VC	0	0
NM_1526	chr1	66752457	66779047	+	2257	1	TCTEX1D1	0.017	0.018
NM_0012	chr1	1.55E+08	1.55E+08	+	2339	1	RUSC1|NE	28.422	15.155
NM_0055	chr6	41638457	41654244	+	1610	1	MDFI|I-M	13.638	19.627
NM_0147	chr5	1.8E+08	1.8E+08	+	5716	1	MAML1|M	12.363	12.947
NM_0011	chr10	37601438	37602931	-	1491	1	MTRNR2L	0.052	0.018
NM_0012	chr1	1.61E+08	1.61E+08	-	1515	1	CD48|BCM	0	0
NM_1526	chrX	78656069	78659328	-	2644	1	ZCCHC5|M	0	0
NM_0010	chr3	1.34E+08	1.34E+08	+	2772	1	TF|HEL-S-	0.795	1.492

NM_0149(	chr17	58755869	58985179	+	6536	1	PPM1E|Ca	12.345	11.951
NR_11001	chr7	17463445	17558909	-	438	1	LOC10192	0	0
NM_0334!	chr17	75874164	75878575	-	2255	1	TRIM47|G	1.618	1.707
NR_11069	chr1	26225320	26229840	+	1074	1	LOC10192	0	0
NM_0027	chr17	43940802	43942467	-	456	1	PPY|PNP|	0.17	0
NM_0010(	chr22	39040668	39044423	+	800	1	APOBEC3F	0.032	0.086
NM_0012	chr6	1.39E+08	1.39E+08	-	2706	1	GVQW2|-|	0.052	0.086
NM_0010(	chrX	8464830	8466510	+	1170	1	VCX3B|VC	0	0
NM_0012	chr16	90019629	90044971	+	3187	1	GAS8|CILD	6.882	6.124
NM_0014	chr5	1.52E+08	1.52E+08	-	14511	1	FAT2|CDH	0.083	0.063
NM_0160	chr7	1.21E+08	1.21E+08	+	2870	1	WNT16|-|	0.009	0.005
NM_0328	chr3	1.23E+08	1.23E+08	+	3326	1	DIRC2|RC(	3.714	5.901
NR_02412	chr9	96019732	96021755	-	1719	1	LINC00092	0.045	0.056
NM_0224	chr2	43812472	43838819	-	2725	1	ABCG5|ST	0.024	0.025
NM_0067	chr6_GL0C	3655068	3704158	-	1794.6	8	C6orf10|T	0	0
NR_04655	chr3	3039661	3061145	-	1008	1	CNTN4-AS	0	0
NM_0188	chr2	1.96E+08	1.96E+08	-	12327	1	DNAH7|-|	0.167	0.119
NM_0012	chr9	1.37E+08	1.37E+08	-	504	1	TMEM210	0.153	0
NM_0011	chr19	37738302	37779590	-	2250	1	ZNF573|-|	1.129	1.431
NM_0012	chr6	36676460	36687339	+	2099	1	CDKN1A|C	69.363	56.028
NM_0040	chr6	1.1E+08	1.1E+08	-	1526	1	DDO|DAS(	0	0.072
NM_0312	chr8	30831544	30849017	-	10107	1	TEX15|CT4	0.005	0.123
NM_0042	chr22	38705802	38733587	+	5033	1	GTPBP1|G	34.902	40.272
NM_0039	chr19	58555712	58558976	-	1516	1	UBE2M|U	53.471	53.839
NM_0142	chr3	10816200	10940733	+	4280	1	SLC6A11|(	0.045	1.274
NM_0012(	chr9	34551432	34590140	-	1917	1	CNTFR|-|-	90.47	121.937
NM_1784	chr1	1.53E+08	1.53E+08	-	268	1	LCE3A|LEF	0	0
NR_14446	chr3	1.09E+08	1.09E+08	+	559	1	MORC1-AS	0	0
NM_0012	chr10	73110375	73131828	+	2069	1	NUDT13|-	1.122	0.728
NM_0011	chr1	40374633	40423326	+	2476	1	SMAP2|SN	64.139	82.16
NM_0011	chr4	13540830	13544490	-	2237	1	NKX3-2|B	0.035	0.588
NR_13666	chr16	67248872	67272191	+	3552	1	SLC9A5|NI	2.334	4.677
NM_0174	chr1	22710838	22921500	+	11018	1	EPHB2|CA	49.587	47.762
NM_0036	chr19	6413108	6424811	-	3228	1	KHSRP|FBI	76.035	70.501
NM_0245	chr3	1.94E+08	1.94E+08	-	7294	1	ATP13A3|	3.251	4.539
NM_0060(	chr2	2.19E+08	2.19E+08	-	2070	1	TUBA4A|A	3.961	5.757
NM_0011	chr1	1.55E+08	1.55E+08	+	1326	1	FDPS|FPPS	176.279	130.483
NM_1788	chr10	18659384	18678011	+	3581	1	ARL5B|AR	3.838	3.764
NR_03033	chr9	1.23E+08	1.23E+08	-	78	1	MIR601|M	0	0
NR_12543	chr8	22877972	22888022	+	3887	1	LOC10192	0	0.004
NR_12204	chr2	30346646	30350158	+	690	1	LOC28504	0.149	0.079
NM_0153	chr19	7395113	7472485	+	5197	1	ARHGEF18	16.454	17.049
NM_0011	chrX	13318649	13320399	-	1748	1	ATXN3L|M	0	0
NM_1534	chr11	7795974	7796942	-	967	2	OR5P2|JC(	0	0
NM_0150	chr14	44962190	45074431	+	6244	1	FAM179B|	2.352	2.374
NR_00131	chr6_GL0C	1181786	1184367	-	2587.4	7	HCG4B|H(	0.01	0
NM_0013	chr19	57583976	57593890	+	4290	1	ZIK1|ZNF7	10.567	10.715
NR_03762	chr2	1.1E+08	1.1E+08	+	877	1	LOC10050	0	0
NM_0010	chr17	27878314	27893383	-	1475	1	LYRM9|C1	12.019	12.908
NR_10977	chr3	1.13E+08	1.13E+08	-	5175	1	LINC0127	0.232	0.18

NR_12067	chr10	45444568	45453121	-	461	2	LOC1027	0	0
NM_0211	chr2	1.76E+08	1.76E+08	+	810	1	HOXD12|	0	0
NM_0012	chr1	1.47E+08	1.47E+08	+	7070	1	NBPF12|C	0.936	1.176
NM_0044	chrX	15345591	15384413	-	2065	1	VEGFD|FI	0.094	0.046
NM_0072	chr19	15615218	15629637	+	1891	1	CYP4F8|C	0	0
NM_1818	chr6	87590067	87667454	+	2550	1	ORC3|LAT	7.155	7.95
NM_0046	chr16	31033491	31040167	+	1467	1	STX4|STX4	16.418	18.589
NM_1447	chr22	20965130	20981360	+	2342	1	AIFM3|AI	0.275	0.415
NM_0011	chr12	31659675	31669082	+	2103	1	ETFBKMT|	1.226	1.231
NM_0012	chr12	54181455	54188994	-	655	1	SMUG1|F	26.437	24.906
NR_03971	chr11	1.19E+08	1.19E+08	+	79	1	MIR4492|	0.163	0.347
NM_0140	chr2	46698960	46762788	+	4418	1	SOCS5|CIS	3.321	3.999
NM_0010	chr14	20033844	20034758	-	913	1	OR4K13|C	0	0
NR_00443	chr19	47918429	47918550	+	121	2	SNAR-A1|	0	0
NR_12591	chr4	17171757	17186057	-	1696	1	LOC10192	0	0
NM_0184	chr1	11664093	11674352	+	1541	1	FBXO6|FB	0.987	0.942
NM_0013	chr9	1.28E+08	1.28E+08	-	1690	1	TRUB2|CL	21.003	19.93
NR_12062	chr10	1.04E+08	1.04E+08	-	1010	1	LOC10192	0	0
NM_0011	chr12	1.3E+08	1.3E+08	+	2775	2	PIWIL1|CT	0	0
NM_0024	chr17	47209348	47223679	+	846	1	MYL4|ALC	0.107	0.065
NM_0010	chrX	49341114	49529985	+	591	6	GAGE12F|	0	0
NR_12052	chr7	1.01E+08	1.01E+08	-	381	1	LOC1027	1.895	0.072
NM_0048	chr19	16574907	16628204	-	3179	1	MED26|C	5.151	5.179
NR_13453	chr22	44881163	45009610	-	3419	1	PHF21B|B	106.553	85.087
NR_11079	chr1	23521930	23528419	+	1209	1	LOC10192	0.299	0.091
NM_0013	chr22	22244780	22245515	+	632	1	VPREB1|C	0	0
NR_02987	chr14	1.01E+08	1.01E+08	+	60	1	MIR380|M	0	0
NR_13558	chr1	95310928	95318263	-	380	1	LINC0176	0	0
NM_1985	chr1	2.31E+08	2.31E+08	-	1494	1	FAM89A|	5.636	5.171
NR_02453	chr2	1.14E+08	1.14E+08	-	510	1	RPL23AP7	7.129	4.915
NR_00244	chr9	1.33E+08	1.33E+08	+	74	1	SNORD24|	0.697	5.368
NM_0157	chr1	1.6E+08	1.6E+08	-	3867	1	DCAF8|GA	28.454	30.603
NM_0154	chr11	35431829	35526031	-	3173	1	PAMR1|D	0.272	0.181
NM_0013	chr7	6010725	6023834	+	1012	1	AIMP2|JT	33.508	36.097
NM_0054	chr1	66797741	66801259	-	714	1	INSL5|PR	0	0.019
NM_0034	chr14	91232532	91253880	-	2810	1	GPR68|AI	0.67	0.356
NR_12595	chr1	1.68E+08	1.68E+08	-	614	1	LOC10192	0	0
NM_0026	chr20	32192504	32207743	-	5651	1	PLAGL2|Z	9.48	7.875
NM_0057	chr11	1.18E+08	1.18E+08	-	2853	1	MPZL2|EV	0	0
NM_0012	chr10	1.17E+08	1.17E+08	+	2849	1	ENO4|C10	0.448	0.399
NM_0010	chr5	1.8E+08	1.8E+08	-	1029	1	MRNIP|C5	24.546	19.487
NM_0011	chr10	1.15E+08	1.15E+08	+	2846	1	FAM160B1	3.633	4.312
NM_0010	chr17	7281735	7288048	+	3136	1	SLC2A4|G	0.456	0.533
NR_02995	chr1	1169005	1169087	+	82	1	MIR429|M	0	0
NR_00296	chr4	1.18E+08	1.18E+08	+	130	1	SNORA24|	0.793	0.105
NM_0159	chr4	17577304	17607967	+	2085	1	LAP3|HEL-	8.868	9.144
NM_0012	chr15	31069391	31101726	-	981	2	TRPM1|CS	0	0
NR_00393	chr7	66809993	66844826	-	2415	1	GTF2IRD1	4.026	4.044
NM_1736	chr1	42380797	42424229	+	10562	1	RIMKLA|F	7.307	5.017
NR_00232	chr3	39411054	39411207	+	153	1	SNORA62|	3.118	5.55

NM_0013:	chr3	1.12E+08	1.12E+08	+	2248	1	CD200|M(	83.051	66.657
NM_1781:	chr17	74877311	74893811	-	2335	1	FADS6|FP:	0.033	0.012
NM_0011!	chr14	19712904	19714332	-	1081	1	OR11H2|C	0	0
NR_02939	chr17	20501514	20504498	-	1948	1	KRT16P3|I	0	0
NM_1784!	chr1	1.11E+08	1.11E+08	-	1875	1	DRAM2|C(	6.663	8.182
NR_02437	chr1	3064072	3067725	-	618	1	LINC00982	0.25	2.482
NM_0204(	chr19	43353659	43363172	+	2211	1	CD177|HN	0	0
NM_0011:	chr5	1.62E+08	1.62E+08	+	3988	1	GABRA1|E	0.336	0.216
NM_0007!	chr9	1.29E+08	1.29E+08	-	2757	1	CRAT|CAT	30.417	38.826
NM_0307(	chr1	1.58E+08	1.58E+08	-	2575	1	FCRL2|CD:	0	0
NR_13274	chr4	1.04E+08	1.04E+08	-	4335	1	CXXC4|ID/	7.007	3.273
NM_0529(	chr5	140258	189972	+	11495	1	PLEKHG4B	3.521	1.97
NM_0012{	chr20	18288283	18316996	+	2243	1	ZNF133|ZI	14.072	18.973
NM_0247:	chr7	1.51E+08	1.51E+08	-	3698	1	GIMAP6|I/	0	0
NR_04988	chr10	641689	641778	-	89	1	MIR5699|ı	0	0
NR_10460	chr17	12760134	12790284	-	3725	1	LOC10012:	0	0
NM_0010:	chr5	43039080	43040345	-	1263	1	ANXA2R|A	1.399	1.876
NM_0220!	chr20	45948653	45972194	-	4422	1	ZNF335|M	9.432	11.423
NR_00350	chr7	32916815	32943170	-	1291	1	RP9P|-|-|:	7.97	8.021
NR_00320	chr14	1.01E+08	1.01E+08	+	70	1	SNORD114	0	0
NM_1450!	chrX	75274059	75304897	+	2503	1	UPRT|FUR	8.623	8.433
NR_07318	chr19	42397148	42652355	+	1471	1	LIPE-AS1|-	2.358	2.942
NM_0012:	chr22	38957522	38963183	+	1383	1	APOBEC3A	0	0
NM_0181(	chr12	26905179	26938321	-	2957	1	INTS13|AS	4.823	5.378
NM_0012:	chr19	44426251	44448578	-	4827	1	ZNF229|-|	2.847	2.199
NR_02982	chr11	64891355	64891439	-	84	1	MIR194-2|	0	0
NM_1523:	chr13	30932697	30975016	+	989	1	TEX26|C1:	0.235	0.111
NM_0133(	chr19	44113362	44134816	+	4471	1	ZNF225|-|	1.465	1.942
NM_1342!	chrY	6910686	7091683	+	2292	1	TBL1Y|TBL	0.135	0.173
NM_0143:	chr16	67873023	67884514	+	4780	1	EDC4|GE1	26.599	23.912
NR_10347	chr19	22608016	22608501	+	485	1	LOC10099(	0	0
NR_12590	chr4	1.75E+08	1.75E+08	+	1720	1	LOC10192:	0.202	0.024
NM_0208(	chr11	30863603	30992686	-	4737	1	DCDC5|-|-	0.509	0.24
NM_0012!	chr17	28954929	29006440	-	4029	1	SEZ6|BSRF	19.444	54.193
NM_0013(	chr10	59736990	59753445	-	422	1	MRLN|LIN	0.183	0.195
NM_0012!	chr18	26226883	26391686	+	4312	1	TAF4B|SP(	0.158	0.375
NM_0001:	chr20	34928433	34955817	-	1904	1	GSS|GSHS	25.069	29.415
NR_13630	chr9	40499981	40578494	+	2503	1	LOC10272:	0.082	0.038
NM_0061!	chr3	1.37E+08	1.37E+08	+	4415	1	NCK1|NCK	3.049	3.462
NM_0221:	chr5	32124718	32174319	-	2670	1	GOLPH3|G	26.482	29.072
NM_0174:	chr8	28494205	28574268	+	13777	1	FZD3|Fz-3	7.562	7.734
NM_0010(	chr11	6169355	6170326	-	970	1	OR52B2|C	0	0
NM_0010(	chr12	53441649	53446643	+	1039	1	PRR13|TXI	14.581	14.396
NM_1533(	chr10	79382327	79445627	-	4925	1	ZCCHC24|(	1.262	1.374
NM_0013:	chr16	28638361	28658630	+	1291	1	NPIPB8|-|	0	0
NR_03624	chr5	6827853	6827921	-	68	1	MIR4278|	0	0
NM_0011(	chr15	78593053	78621295	-	2021	1	CHRNA3|L	23.86	8.499
NM_0010:	chr14	69879397	70032366	+	3684	1	SMOC1|O/	8.501	70.668
NM_0010!	chrX	49341150	49529985	+	556.7	6	GAGE12G|	0	0
NR_03619	chr14	54878113	54878193	-	80	1	MIR4308|	0	0

NM_1451;	chr19	2714567	2721392	-	3394	1	DIRAS1|Di	51.547	69.506
NM_0011:	chr3	46407558	46409523	+	1589	1	CCRL2|ACI	0	0
NR_03888	chr7	30548357	30577779	-	3864	1	LOC40132(	1.405	1.865
NM_0013:	chr4	849275	932390	-	4273	1	GAK|DNAJ	20.974	22.647
NM_0196(	chr20	2794069	2800646	-	2384	1	CPXM1|CF	44.603	24.901
NM_0039:	chr6	4021335	4064983	+	7465	1	PRPF4B|PI	4.152	5.899
NM_0012;	chrX	75053172	75156340	-	2507	1	ABCB7|AB	6.208	6.103
NM_0011<	chr12	6965352	6970753	-	1432	1	PHB2|BAP	210.699	197.822
NM_0062;	chr21	46598618	46605122	-	1130	1	S100B|NEI	14.537	25.782
NM_0328!	chr3	1.97E+08	1.97E+08	-	2197	1	CEP19|C3(	8.111	7.662
NM_0030(	chr1	1.7E+08	1.7E+08	-	3152	1	SELP|CD62	0	0
NR_02456	chr18	35290272	35310766	+	5131	1	ZNF271P|(	4.616	5.958
NR_03888	chr21	32728114	32732913	+	2941	1	PAXBP1-A!	3.599	4.247
NR_00306	chr1	30968163	30968237	-	74	1	SNORD103	0	0
NR_13078	chr15	24441127	24447967	+	845	1	PWRN3|-|	0	0
NM_0012{	chr19	18008168	18014102	+	2490	1	ARRDC2|C	0.932	1.067
NM_0011(	chr14	32939253	33804176	+	5830	1	NPAS3|M(	6.9	6.247
NM_0312:	chr20	33657087	33674458	-	1922	1	NECAB3|A	17.717	14.16
NM_0010:	chr4	1.72E+08	1.73E+08	+	2834	1	GALNTL6|(	1.21	2.363
NR_02404	chr8	7312846	7319876	+	187	3	DEFB109P	0	0
NM_1738:	chr2	2.42E+08	2.42E+08	+	1794	2	RTP5|C2oi	0	0
NM_0012{	chr21	42472486	42496354	-	1306	1	RSPH1|CT	2.596	1.426
NR_03019	chr19	53700015	53700101	+	86	1	MIR518F|I	0	0
NR_03995	chr1	64579847	64579921	+	74	1	MIR4794|-	0	0
NM_0030:	chr7	37905933	37916923	-	2965	1	SFRP4|FRF	1.074	0.453
NR_11190	chr20	435477	462553	-	2039	1	TBC1D20|(	21.759	26.104
NR_10692	chr16	28390982	28391051	-	69	2	MIR6862-1	0	0
NR_11054	chr21	13546033	13558460	+	499	1	LINC01674	0	0
NR_13392	chr7	1.3E+08	1.3E+08	-	516	1	LOC10537.	0	0
NM_0012!	chr6	1.17E+08	1.17E+08	+	3646	1	FAM26D|(	0.421	0.034
NM_0011!	chr9	1.29E+08	1.29E+08	+	2515	1	PTPA|PP2/	74.321	84.753
NR_12055	chr11	38618329	38646356	-	472	1	LOC10331:	0	0
NM_0011(	chr14	50905217	50944530	-	2723	1	PYGL|GSD	3.887	3.335
NM_0058<	chr15	65659123	65792293	-	8664	1	DENND4A	1.604	2.463
NM_0067(	chr6	38675926	38703176	-	2038	1	GLO1|GLC	63.297	69.278
NR_03623	chr4	7460028	7460118	+	90	1	MIR4274|-	0	0
NR_10462	chr5	16373361	16441102	-	2126	1	LINC0215C	0.024	0.045
NM_0011:	chr1	1.6E+08	1.6E+08	-	2493	1	C1orf204|	0.403	0.121
NR_12372	chr16	3052125	3059370	-	2334	1	MMP25-A!	1.585	1.444
NR_13429	chr8	82912104	82961924	+	610	1	LOC10192	0	0
NM_0013:	chr1	1.63E+08	1.63E+08	+	2154	1	UAP1|AGX	11.391	12.832
NM_0013(	chr1	1.5E+08	1.5E+08	+	420	1	C1orf54|-|	39.724	37.178
NR_04001	chr17	79019209	79027655	-	963	1	C1QTNF1-/	0	0
NM_0014(	chr11	62559601	62573988	-	1526	1	EEF1G|EF1	290.543	323.361
NM_2034:	chr12	1.08E+08	1.08E+08	+	2257	1	ASCL4|HA!	0.006	0
NM_0013:	chr6	42929130	42939287	+	1643	1	CNPY3|CA	32.85	30.754
NM_0012{	chr9	1.1E+08	1.1E+08	-	464	1	TXNDC8|S	0	0
NR_10694	chr22	41252992	41253050	-	58	1	MIR6889|I	15.339	11.335
NM_0175{	chr10	1.25E+08	1.25E+08	+	4931	1	ZRANB1|T	18.949	23.605
NM_0165:	chr15	63189529	63267774	+	4865	1	RAB8B|-|-	8.648	8.134

NM_0010	chr2	1.78E+08	1.78E+08	-	1848	1	PDE11A|P	0.244	0.296
NM_0055	chr10	1.01E+08	1.01E+08	+	2102	1	TLX1|HOX	0.012	0.691
NR_03890	chr11	1.29E+08	1.29E+08	-	1295	1	SENCR|FLI	0	0
NM_0013	chr1	88684144	88836255	+	5875	1	PKN2|PAK	1.751	3.122
NM_0209	chr19	48837097	48868627	-	3061	1	PLEKHA4|	3.669	3.401
NM_0011	chr1	91500847	91525764	+	3171	1	CDC7|CDC	4.835	4.186
NM_0011	chr1	2.44E+08	2.45E+08	+	3170	1	C1orf101|	0.114	0.017
NM_0207	chr2	8728857	8837625	-	7329	1	KIDINS220	54.447	58.722
NM_0011	chr16	4624824	4690974	+	3850	1	MGRN1|R	45.251	59.089
NM_0011	chr18	63949354	63960411	+	619	1	HMSD|AC	0	0
NM_1785	chr17	1934677	2025345	-	3621	1	RTN4RL1|	14.279	10.932
NM_2075	chr19	14091695	14114188	-	2470	1	PRKACA|P	72.522	54.505
NM_0013	chr17	19343445	19362738	-	683	1	B9D1|B9|	17.16	8.864
NM_0038	chr18	23452823	23483135	+	4018	1	RIOK3|SU	13.776	9.974
NM_0012	chr1	27977273	28088637	-	1783	1	EYA3|-|-|	4.491	5.7
NM_0206	chr16	8674587	8784575	+	4796	1	ABAT|GAB	43.189	33.44
NM_1809	chr17	76079182	76082804	+	1462	1	ZACN|L2|	34.2	33.643
NR_02850	chr17	1711504	1716272	-	1844	2	MIR22HG|	0	0
NR_10691	chr16	17052	17119	-	67	4	MIR6859-1	0.385	0.204
NM_0143	chr6	43336070	43369443	-	7994	1	ZNF318|H	7.132	7.673
NR_00244	chr15	66502022	66502091	-	69	1	SNORD18	1.121	0
NM_0021	chr19	49151199	49155424	-	2376	1	HRC|-|-|1	0.613	0.386
NR_14434	chr14	24630956	24634269	-	828	1	GZMB|C1	0.109	1.241
NM_1532	chr5	93740309	93741603	-	1292	1	POU5F2|S	0	0
NM_0012	chr8	1.25E+08	1.25E+08	+	2804	1	TRIB1|C8F	7.247	9.784
NM_0181	chr17	59210010	59215250	+	3260	1	SMG8|C17	14.824	16.306
NR_10399	chr9	1.33E+08	1.33E+08	+	3338	1	STKLD1|C	0.834	1.243
NR_10688	chr3	1.7E+08	1.7E+08	+	59	1	MIR6828|	0	0
NR_12061	chr10	1.28E+08	1.28E+08	+	3005	1	LINC01163	0.013	0.036
NR_03399	chr3	1.73E+08	1.73E+08	-	1906	1	TNFSF10|	0	0
NM_1816	chr21	44637356	44638455	+	1097	1	KRTAP10-1	0	0
NM_1447	chr2	2.19E+08	2.19E+08	-	2030	1	SLC23A3|	0.057	0.243
NM_0181	chr8	19817407	19852075	+	2515	1	INTS10|C8	12.878	16.044
NM_0012	chr22	20148586	20149908	-	625	1	CCDC188|	2.682	0.833
NM_0010	chr4	3249040	3256615	+	1665	1	MSANTD1	0.511	0.658
NM_0133	chr22	29767369	29770413	+	912	1	UQCR10|H	125.344	98.312
NR_03164	chr4	1.13E+08	1.13E+08	+	92	1	MIR1243|	0	0
NR_02959	chr7	25949919	25949986	-	67	1	MIR148A|	0	0
NM_0146	chr1	1.62E+08	1.62E+08	+	4452	1	NOS1AP|6	4.023	8.731
NM_0005	chr17	58192724	58205174	+	2706	1	EPX|EPO|	0.8	0.719
NR_13652	chr19	35947115	35964070	+	2421	1	LOC10537	0.533	0.651
NM_0177	chr2	96859736	96869998	-	3535	1	SEMA4C|	35.635	35.244
NM_0013	chr10	69318181	69401881	+	3472	1	HK1|HK|H	112.057	110.022
NR_03394	chr3	45678165	45688882	-	2297	1	LIMD1-AS1	6.944	7.633
NR_11089	chr16	32673528	32676129	-	1881.3	6	TP53TG3B	0	0
NM_1992	chr5	95620276	95621580	+	1303	1	GPR150|P	0.04	0.221
NM_1336	chr15	40872214	40874289	-	1700	1	RHOV|AR	0.736	1.853
NR_04757	chr3	1.95E+08	1.95E+08	+	1430	1	TMEM44-	3.607	2.941
NM_0010	chr3	48688003	48717278	-	1792	1	IP6K2|IHP	100.291	138.659
NR_00365	chr16	56643804	56644941	+	500	1	MT1DP|M	0	0

NM_0012	chr9	34379020	34381600	-	628	1	C9orf24|C	1.827	0.785
NM_0180	chr1	1.09E+08	1.09E+08	+	3695	1	PRPF38B|	7.52	8.463
NR_00329	chr15	25176832	25176913	+	81	1	SNORD11	0	0
NM_1447	chr13	97222296	97394120	+	4717	1	MBNL2|M	0.67	1.356
NM_0010	chr3	1.97E+08	1.97E+08	-	4940	1	DLG1|DLG	3.878	3.993
NM_0011	chr12	48921959	48924881	-	537	1	FKBP11|F	5.21	4.515
NM_0148	chr5	74766991	74776912	+	1386	1	NSA2|CDK	18.745	18.273
NM_0011	chr10	1.17E+08	1.17E+08	-	1963	1	VAX1|MC	0	0.084
NM_0010	chrX	1.12E+08	1.12E+08	+	2953	1	ZCCHC16|	0.022	0.023
NM_0029	chr16	1962061	1964826	-	933	1	RPS2|LLRE	127.069	113.687
NM_0012	chr11	75562056	75572804	+	2269	1	SERPINH1|	25.071	14.506
NM_1827	chr7	20782274	20786889	-	3630	1	SP8|BTD|-	0.17	11.444
NM_0151	chr3	16933090	17090606	+	3945	1	PLCL2|PLC	4.357	7.611
NM_1785	chr3	1.35E+08	1.35E+08	-	5691	1	KY|MFM7	0.181	0.221
NM_0124	chr17	75845699	75855420	-	1934	1	WBP2|GR	98.981	99.711
NM_0012	chr13	73054976	73077542	+	2946	1	KLF5|BTEB	0.193	0.195
NM_0320	chr9	1.09E+08	1.09E+08	-	7977	1	TMEM245	7.427	7.492
NR_02694	chr11	60615751	60687148	+	844	1	LINC00301	0	0
NM_0012	chr19	18279694	18281656	-	1960	1	JUND|AP-	128.764	141.422
NR_10698	chr9	1.11E+08	1.11E+08	-	58	1	MIR7702|	0	0
NR_10276	chr18	22166898	22168968	-	1786	1	GATA6-AS	0	0.008
NR_02440	chr6	52664401	52669153	+	3551	1	LOC73010	8.725	7.036
NM_0010	chrX	17737449	17754988	+	2693	1	SCML1|-|-	2.016	1.979
NR_02670	chr5	1.41E+08	1.41E+08	+	97	1	VTRNA1-1	0	0.141
NM_0011	chr4	1.55E+08	1.55E+08	+	3441	1	FGB|HEL-S	0.015	0.004
NM_0008	chr21	33503931	33542158	-	2225.5	2	GART|AIRS	30.207	31.038
NM_1834	chr12	1.09E+08	1.1E+08	+	5369	1	UBE3B|BP	14.678	14.603
NM_0014	chr2	88122989	88128131	-	557	1	FABP1|FA	0	0
NR_11038	chrX	39367285	39391774	-	874	1	LINC01282	0	0
NR_11069	chr19	32102091	32105916	-	2901	1	LINC01782	0	0
NM_0011	chrX	49058966	49069858	+	3432	1	CCDC120|	8.877	8.401
NM_0013	chr19	43253282	43269563	-	1176	1	PSG9|PS3	0	0
NR_03741	chr20	44408120	44408203	+	83	1	MIR3646|	0	0
NM_0011	chr7	74964818	75031532	+	7980	1	GATSL2|C	23.223	25.195
NM_1452	chr10	1.26E+08	1.26E+08	+	1283	1	FANK1|HS	2.543	1.601
NM_0012	chr16	89657804	89670458	+	1405	1	SPATA33|	10.847	6.259
NM_0025	chr1	2.36E+08	2.36E+08	-	5859	1	NID1|NID|	0.414	0.764
NR_04651	chr13	94712716	94715945	+	2984	1	SOX21-AS	1.983	4.659
NM_0012	chr19	1269268	1273172	+	1388	1	CIRBP|CIR	348.718	343.994
NM_0040	chr6	41921227	41933046	+	1981	1	BYSL|BYST	15.256	18.198
NR_10690	chr8	1.44E+08	1.44E+08	-	69	1	MIR6848|	9.156	12.307
NM_0055	chr1	1.6E+08	1.6E+08	+	2555	1	NHLH1|HE	82.952	123.332
NR_02961	chr18	35904818	35904926	-	108	1	MIR187|M	0	0
NM_0010	chr17	79089345	79516148	-	2973	1	RBFOX3|F	7.481	9.343
NM_0012	chr2	1.28E+08	1.28E+08	-	1735	1	LIMS2|LG	0.26	0.434
NR_10344	chr5_KI27	14687	24728	+	1039	2	LOC10099	0	0
NR_03985	chr12	54231397	54231476	-	79	1	MIR3198-	78.177	41.438
NM_1813	chr22	44702198	44862784	+	2144	1	PRR5-ARH	2.436	5.788
NM_0043	chr12	31073845	31104791	+	3749	1	DDX11|CH	5.145	5.199
NM_1785	chr17	15732277	15744784	+	1852	1	TBC1D26|	0.077	0.251

NR_02994	chr3	49020148	49020234	-	86	1	MIR425|M	0	0
NM_00132	chr21	6111073	6123778	+	4789	2	LOC102724	0.797	0.586
NM_0134	chrX	1.42E+08	1.42E+08	-	414	2	SPANXA1|	0	0
NM_0219	chr7	93906508	93911265	+	715	1	GNGT1|GN	0.072	0.038
NR_11073	chr19	51152923	51181966	-	1758	1	LOC10192	0	0
NR_10994	chr20	44377743	44395706	-	644	1	HNF4A-AS	0	0
NM_0010	chr6	7541575	7586717	+	7973	1	DSP|DCWI	0	0.01
NM_0049	chr22	38426328	38444028	-	1898	1	KCNJ4|HIR	4.945	9.908
NM_0012	chr2	2.24E+08	2.25E+08	-	6455	1	CUL3|CUL	9.678	12.954
NR_04666	chr13	1.11E+08	1.11E+08	-	1237	1	ARHGEF7-	3.94	3.399
NM_0033	chr11	62419035	62423206	+	447	1	SCGB1A1|	0.26	0.214
NR_11030	chr10	75403877	75408982	+	1796	1	ZNF503-AS	2.778	2.814
NM_0010	chr12	51912329	51923361	+	4115	1	ACVRL1|A	0.06	0.003
NR_03614	chr16	26025237	26025310	+	73	1	MIR548W	0	0
NR_03024	chrX	1.47E+08	1.47E+08	-	87	2	MIR514A3	0	0
NM_0012	chr18	12093849	12129749	+	4717	1	ANKRD62|	0	0
NR_13133	chr5	55160118	55167301	+	3698	1	GPX8|EPL	2.343	0.563
NM_0012	chr19	29698886	29715056	-	4238	1	C19orf12|	15.51	15.394
NM_0190	chrX	57135650	57137625	-	1315	1	SPIN2A|D	0.578	0.073
NR_11070	chr19	36685439	36687449	-	551	1	LINC01534	2.106	2.088
NM_0013	chr12	12357079	12471221	+	6012	1	BORCS5|L	3.903	5.548
NR_00365	chr7	43940895	44019194	-	6495	1	POLR2J4|F	2.763	3.22
NM_0014	chr11	10797046	10809035	-	3887	1	EIF4G2|AA	429.419	422.599
NR_04635	chr8	7769584	7771313	+	604	1	FAM90A1	0	0
NR_13212	chr5	1.33E+08	1.33E+08	-	404	1	TH2LCRR|	0.702	1.627
NR_07313	chr22	26450883	26483863	-	5149	1	HPS4|BLO	14.396	18.11
NM_0012	chr7	2475137	2476383	-	442	1	GRIFIN|-|-	0	0
NR_03623	chr3	49274120	49274186	+	66	1	MIR4271|	10.745	8.094
NR_10997	chr16	634427	636366	-	634	1	METTL26|	47.141	36.489
NM_0252	chr20	33007578	33023709	+	1902	1	BPIFB2|BP	0	0
NM_0252	chr17	38530024	38605930	-	7029	2	SRCIN1|P1	0	0
NM_0010	chr14	88385644	88438461	+	1905	1	SPATA7|H	7.154	5.723
NM_0323	chr12	55820960	55829636	-	3321	1	DNAJC14|	25.771	24.482
NR_03062	chr1	93846832	93846911	+	79	1	MIR760|M	0.979	0.867
NM_0011	chr1	61081862	61462788	+	9201	1	NFIA|CTF|	0.895	15.102
NM_0012	chr20	50267486	50279795	+	2004	1	LINC01272	0	0
NR_02970	chr5	1.6E+08	1.6E+08	+	98	1	MIR146A|	0.395	0
NM_0010	chr2	1.65E+08	1.65E+08	+	8527	1	SCN2A|BF	2.893	4.48
NM_0203	chr3	45823318	45842158	-	4051	1	LZTFL1|BB	2.766	1.9
NR_03748	chr3	1.59E+08	1.59E+08	+	88	1	MIR3919|	0	0
NR_12650	chr15	92470234	92471546	-	477	1	LOC10461	0.324	0.086
NM_0013	chrX	1.31E+08	1.31E+08	+	2552	1	ARHGAP36	0.642	0.274
NM_0013	chr17	81681165	81683713	-	1060	1	ARL16|-|-	39.264	37.124
NM_0036	chr6	53126962	53148826	-	2756	1	GCM1|GC	0.056	0.01
NR_04659	chr3	1.85E+08	1.85E+08	-	569	1	LINC0184	0	0
NR_02445	chr2	46827864	46859006	+	1207	1	LINC0111	0.47	0.704
NM_0013	chr14	33924215	33950826	-	2181	1	EGLN3|HI	7.532	3.624
NR_10696	chr9	1.37E+08	1.37E+08	-	60	1	MIR7114|	0	0.685
NM_0042	chr19	6739682	6751526	+	2017	1	TRIP10|CI	9.806	10.75
NM_0221	chr19	47994651	48025153	+	1050	1	ELSPBP1|E	0	0.026

NR_14440	chr17	42552436	42554802	-	2366	1	LOC10878	10.785	9.917
NM_0011	chr3	42257824	42263947	-	744	1	CCK|-|-|3	0.191	0
NM_0053	chr14	1.02E+08	1.02E+08	-	3351	1	HSP90AA1	286.223	220.943
NM_0183	chr5	60596912	60700166	-	2491	1	DEPDC1B|	3.163	2.98
NM_0183	chr6	485138	693141	-	4444	1	EXOC2|SE	12.435	15.771
NR_00152	chrY	22851584	22852715	+	164	3	TTTY17A|	0	0
NM_1388	chr10	80336106	80356758	-	753	1	DYDC1|DP	0	0.073
NM_1525	chr17	8797110	8799349	-	2237	1	MFSD6L|-	0	0
NR_13123	chrX	1.04E+08	1.04E+08	-	648	1	LOC10537	0.657	0.655
NM_0012	chr11	1.19E+08	1.19E+08	-	6138	1	DDX6|HLR	32.074	45.297
NR_10428	chr22	24540423	24555935	-	3253	1	GUCD1|C2	15.767	16.362
NR_13120	chr8	1.22E+08	1.23E+08	-	565	1	LOC10537	0.091	0.121
NM_0012	chr1	1.11E+08	1.11E+08	-	3214	1	DENND2D	0.024	0.034
NR_03965	chr3	1.84E+08	1.84E+08	+	85	1	MIR4448|	0	0
NM_1392	chr5	1.11E+08	1.11E+08	+	6567	1	WDR36|G	2.077	2.645
NM_1523	chr19	39531533	39532867	-	1332	1	EID2B|EID	7.337	10.746
NM_0010	chr3	32818018	32897826	+	8679	1	TRIM71|LI	0.578	0.712
NM_0064	chr5	90474864	90514552	+	3275	1	POLR3G|R	0.114	0.184
NR_10674	chr7	1.33E+08	1.33E+08	+	107	1	MIR6133|	0	0
NR_13494	chr3	1.82E+08	1.83E+08	-	591	1	LINC01995	0.065	0.185
NM_0012	chr7	897900	945816	-	2106	1	ADAP1|CE	21.716	21.105
NR_12033	chr15	57319138	57325039	+	981	1	LINC01413	0	0
NR_11012	chr3	6731172	6805449	-	461	1	GRM7-AS3	0.084	0
NM_0064	chr10	90871952	90902190	+	2340	1	RPP30|TS	7.477	8.657
NM_0158	chr8	74824537	74855044	+	6739	1	PI15|CRIS	0.073	0.382
NM_0012	chr6	39335100	39725405	-	3897	1	KIF6|C6or	0.112	0.116
NR_03380	chr10	28519917	28532354	-	5373	1	WAC-AS1|	3.607	4.762
NM_0024	chr11	1.03E+08	1.03E+08	-	3044	1	MMP8|CL	0	0.022
NM_0006	chr4	3766569	3768526	+	1955	1	ADRA2C|A	0.706	1.955
NR_12050	chr6	72630495	72678558	+	559	1	KCNQ5-IT1	0	0
NM_0011	chr19	55488503	55519099	+	4894	1	SSC5D|S5I	1.842	0.971
NM_1523	chr11	30323099	30338223	+	2372	1	ARL14EP|	6.604	8.737
NR_10673	chr11	12163683	12163767	+	84	1	MIR6124|	0	0
NM_0022	chr17	41523617	41528389	-	1463	1	KRT19|CK	0.009	0.243
NR_00357	chr19	52968251	52993531	-	2993	1	ZNF702P|	0.297	2.965
NR_02730	chr19	10041356	10074137	+	3733	1	C3P1|CPLI	0	0
NM_0012	chr15	84235773	84245368	+	4551	2	GOLGA6L4	0	0.018
NM_2068	chr11	60785348	60801305	+	2286	1	MS4A10|	0	0
NR_11039	chrX	72998388	73002856	-	1163	2	PABPC1L2	0	0
NM_0219	chr11	60455809	60470752	+	3322	1	MS4A1|B1	0	0
NM_0038	chr8	1.44E+08	1.44E+08	+	2053	1	GPAA1|GA	56.052	46.428
NR_03616	chr21	18686090	18686164	-	74	1	MIR548X|	0	0
NM_0017	chr1	1.58E+08	1.58E+08	+	2584	1	CD1C|BDC	0	0
NR_03845	chr17	50866679	50868371	+	1413	1	TOB1-AS1	2.09	2.569
NR_02691	chr17	74209996	74213321	-	2483	1	MGC1627	1.065	1.219
NM_0011	chr1	1.51E+08	1.51E+08	+	4422	1	SETDB1|E	29.4	27.196
NR_12586	chr6	1.69E+08	1.69E+08	-	541	1	LOC10192	0	0
NM_0012	chr3	46921730	46976767	-	854	1	CCDC12|-|	41.141	38.332
NM_0011	chr6_GL0	2886791	2900471	-	3602.7	7	BAG6|BAG	0.004	0.004
NM_0133	chr13	36949771	36999367	-	1201	1	ALG5|bA4	19.431	23.151

NM_0013(	chr7	54542325	54571080	+	2983	1	VSTM2A|V	12.504	14.367
NR_02847	chr16	59754141	59755191	-	1050	1	APOOP5|-	1.817	1.318
NM_0011!	chr17	41189887	41190639	+	751	1	KRTAP9-1|	0	0
NM_0011;	chr15	89316305	89334795	-	4411	1	POLG|MD	13.119	16.911
NR_00357	chr15	43875826	43878171	-	2345	1	PIN4P1|-|-	1.276	1.046
NM_0146:	chr5	95463895	95555005	-	5659	1	TTC37|KIA	6.532	9.715
NM_0005!	chr6	31575567	31578335	+	1624.2	8	TNF|DIF|T	0	0
NM_1527(	chr16	1833988	1872101	-	1700	1	MEIOB|C1	0.44	0.524
NM_0010:	chr20	62387065	62388520	+	388	1	RPS21|HLI	523.349	357.605
NR_02682	chr12	65758021	65826974	-	1141	1	RPSAP52|I	0.023	0.096
NM_0011<	chr7	1.13E+08	1.13E+08	-	4758	1	GPR85|SR	2.523	2.559
NM_1733!	chr12	71938846	72032441	+	2347	1	TPH2|ADH	0.06	0.263
NM_0528!	chr12	39755021	40105859	-	6991	1	SLC2A13|H	1.333	0.746
NM_0011<	chr22	41690543	41698136	+	4971	1	C22orf46|	5.698	4.07
NM_0032!	chrX	47582291	47586791	+	908	1	TIMP1|CL(	21.172	13.441
NM_0334<	chr5	1.42E+08	1.42E+08	-	4298	1	FCHSD1|N	10.356	11.839
NR_02985	chr4	1.13E+08	1.13E+08	-	72	1	MIR302B|	0	0.19
NR_11014	chr7	1.03E+08	1.04E+08	+	2721	1	LOC10192	0.009	0
NM_0013(	chr16	30530367	30534970	-	3414	1	ZNF747|-|	2.746	2.636
NM_0165:	chr4	38664169	38701508	+	5607	1	KLF3|BKLF	4.689	3.611
NM_0010(	chr7	1.44E+08	1.44E+08	+	2584	1	CTAGE15|	0	0
NM_0147:	chrX	12138466	12724523	+	8446	1	FRMPD4|N	0.24	0.522
NM_0057(	chr7	1.22E+08	1.22E+08	-	5845	1	AASS|LKR/	1.992	3.956
NM_0248(	chr6	41189749	41201187	-	3816	1	TREML2|C	0	0.004
NM_0012!	chr19	51713112	51723992	-	2101	1	HAS1|HAS	0.264	0.9
NM_0046:	chr6	1.67E+08	1.67E+08	-	1263	1	TCP10|TCI	0	0
NR_03744	chr8	1801125	1801192	+	67	2	MIR3674|-	0	0
NR_02678	chr6	1.7E+08	1.7E+08	-	2416	2	LINC00242	0	0
NR_03367	chr19	18919675	18928633	+	1752	1	DDX49|R2	42.824	42.186
NM_0012!	chr1	2.29E+08	2.29E+08	-	775	1	BTNL10|B	0.083	0.018
NM_0010(	chr9	1.22E+08	1.22E+08	-	967	1	OR1J1|OR	0	0
NR_02415	chr5	470510	472965	-	1741	1	PP7080|-|	8.902	8.969
NM_0035!	chr7	1.49E+08	1.49E+08	+	3185	1	CUL1|-|-|	19.541	29.463
NR_02951	chr11	1.22E+08	1.22E+08	-	79	1	MIR100|M	0	0
NR_03987	chr16	81385018	81385093	+	75	1	MIR4720|-	0	1.096
NM_0124:	chr16	70523788	70577668	+	9692	1	SF3B3|RSE	33.734	37.568
NM_1308<	chr7	1.39E+08	1.39E+08	-	3010	1	ATP6V0A4	0.09	0.077
NR_03887	chr21	21723293	21737319	-	609	1	LINC00317	0	0
NR_04983	chr2	1.1E+08	1.1E+08	-	90	2	MIR4436B	0	0
NR_04766	chr8	12537079	12665611	-	3973	1	LOC72973	0.065	0.114
NM_1525!	chr6	7590199	7611967	+	4162	1	SNRNP48|	1.67	2.031
NM_0065(	chr19	39971005	39981764	+	1901	1	PSMC4|M	81.681	72.311
NR_02434	chr17	7913322	7915947	-	2523	1	LOC28402	2.627	2.812
NR_11080	chr17	65100812	65111094	+	697	1	LOC10050	0.647	0.373
NM_0032!	chr1	1.86E+08	1.86E+08	-	9653	1	TPR|-|-|1(	4.353	6.696
NR_10466	chr16	87271906	87292452	-	699	1	LOC10192	0	0
NR_04005	chr17	77546941	77565021	+	4227	1	LOC10050	0.47	0.052
NM_1447(	chr5	1.7E+08	1.7E+08	+	2007	1	FOXI1|FKH	0	0.027
NM_0011:	chr22	21545669	21551084	+	5413	2	RIMBP3B|	0.014	0
NM_0010;	chr2	87757201	87825767	+	5475	2	RGPD2|NU	0	0.068

NM_1736	chr2	1.01E+08	1.01E+08	-	2959	1	RNF149|D	5.734	6.277
NM_0011	chr7	1.36E+08	1.36E+08	+	2493	1	C7orf73|P	31.036	29.004
NM_0010	chr7	43875894	43926411	-	3827	1	URGCP|UF	12.176	17.978
NM_0011	chr19	38303560	38315805	-	2728	1	YIF1B|FinC	29.823	29.694
NR_13494	chr13	1.12E+08	1.12E+08	-	1727	1	LOC10050	0.015	0
NM_1526	chr3	1.41E+08	1.41E+08	+	2547	1	TRIM42|PI	0	0
NM_0251	chr19	45546281	45584864	-	7869	1	OPA3|MG	4.023	2.827
NM_0061	chr6	4115693	4135597	-	1381	1	ECI2|ACBD	23.042	29.11
NR_00356	chr7	1.03E+08	1.03E+08	-	2442	2	DPY19L2P	1.505	1.01
NM_0013	chrX	9912372	9949443	+	4674	1	SHROOM2	22.984	14.339
NM_1827	chr5	38403532	38465480	+	4153	1	EGFLAM|A	0.301	0.069
NR_13460	chr6	1.18E+08	1.18E+08	+	403	1	LOC10537	0.128	0.034
NM_0012	chr4	47847233	47914667	-	3698	1	NFXL1|CD	2.242	2.978
NM_0010	chr11_JH1	129056	129994	+	937	2	OR8K3|OF	0	0
NR_00244	chr15	66503245	66503314	-	69	1	SNORD18A	0	0
NM_0022	chr17	3714625	3801243	-	3828	1	ITGAE|CD1	4.227	3.982
NR_03970	chr11	10508270	10508326	-	56	1	MIR4485|	0	0
NM_1529	chr16	31215962	31217093	-	543	1	PYDC1|AS	0.332	0.177
NM_0010	chr5	1.48E+08	1.48E+08	+	602	1	SPINK14|S	0	0.023
NM_0180	chr1	32364633	32394461	-	3316	1	BSDC1|-|-	54.475	66.866
NM_0133	chr7	2251770	2314475	-	4725	1	SNX8|Mvp	5.542	5.888
NM_0012	chr2	1.71E+08	1.71E+08	+	594	1	ERICH2|-|	0.933	0.369
NR_13427	chr5	38148480	38153715	+	2571	1	LINC0211	0	0
NR_04668	chr13	79477364	79481231	-	428	1	NDFIP2-AS	0	0
NR_00260	chr19	3982507	3982572	-	65	1	SNORD37|	0	0.211
NR_13530	chr9	91119062	91182762	+	451	1	LINC00484	0.057	0.03
NM_0019	chr3	1.85E+08	1.85E+08	-	3841	1	EHHADH|E	1.306	0.831
NM_0011	chr12	1.32E+08	1.33E+08	+	4528	1	FBRSL1|-|	16.066	17.711
NR_07299	chr10	24981979	25016156	-	1936	1	ENKUR|C1	1.365	1.358
NM_0011	chr17	4498843	4539037	+	3429	1	SPNS2|-|-	12.194	13.266
NM_0208	chr3	3799431	3849834	+	5956	1	LRRN1|FIG	25.291	25.844
NM_0156	chr14	93184951	93187086	+	905	2	TMEM251	0	0
NM_0012	chr11	60515413	60542718	+	741	1	MS4A13|-	0	0
NM_0011	chr19	52154872	52171643	-	3326	1	ZNF836|-|	1.376	2.627
NM_0314	chr11	60699574	60715811	+	1344	1	MS4A8|4S	0.7	0.927
NR_02834	chr7	63349070	63351774	+	651.5	2	LOC10028	0	0
NR_11093	chr16	82953230	82990298	-	1490	1	LOC10192	0	0
NM_0013	chr7	21428034	21514822	+	6113	1	SP4|HF1B	3.027	3.141
NR_00304	chr6	85677589	85677659	-	70	1	SNORD50B	0.368	1.957
NM_0183	chr5	31532266	31555058	+	3601	1	C5orf22|-|	8.45	7.333
NM_0019	chr16	67929570	67931875	-	1173	1	CTRL|CTRL	3.111	3.456
NM_0063	chr17	75038860	75046979	-	614	1	ATP5H|AT	178.22	169.004
NM_0177	chr6	20534457	21232403	+	3253	1	CDKAL1|-|	10.004	12.922
NM_0010	chr1	2.27E+08	2.27E+08	+	7699	1	STUM|C1c	5.843	7.1
NR_03159	chr17	19344506	19344574	-	68	1	MIR1180|	0	0
NM_0011	chr1_KI27	104160	104944	-	782	4	PPIAL4E|C	0	0.018
NR_11031	chr6	1.68E+08	1.68E+08	-	3854	1	FRMD1|bA	0	0.004
NR_00218	chr7	1.29E+08	1.29E+08	+	2016	1	TPI1P2|-|-	0.659	0.727
NM_0165	chr19	37564253	37594792	-	2290	1	ZNF571|H	1.436	2.01
NM_0132	chr16	88638593	88640474	+	1040	1	IL17C|CX2	0.099	0.079

NM_0014	chrX	49570435	49577753	+	518	3	GAGE6|CT	0	0
NR_10418	chr2	46568257	46580238	-	1516	1	LOC10050	0.051	0.208
NM_0063	chr16	75293710	75433489	-	1270	1	CFDP1|BC	113.808	101.433
NM_0011	chr19	32691961	32713796	+	2962	1	NUDT19|R	0.331	0.125
NR_13070	chr2	97283935	97291779	+	701	2	LOC10050	0	0
NM_0010	chr19	9126012	9126950	-	937	1	OR7G3|OS	0	0
NM_0011	chr15	71110244	71115500	-	1825	1	CT62|-|-|	0.042	0.03
NR_02743	chrUn_GL(	56349	99642	-	3655	1	LOC28378	1.626	4.032
NM_0012	chr12	1.21E+08	1.21E+08	-	2543	1	C12orf43|	13.989	15.69
NR_10685	chr19	7617439	7617505	+	66	1	MIR6792|	0	0
NR_02671	chrX	1400531	1413586	+	1785	2	ASMTL-AS	0	0
NM_0012	chr17	18244817	18258556	-	4146	1	FLII|FLI|FL	31.571	26.367
NM_0011	chr8	38411138	38467845	-	4935	1	FGFR1|BF	24.107	37.006
NM_0174	chr20	57391398	57409330	+	2382	1	RBM38|HS	12.092	14.45
NM_0011	chr5	1.57E+08	1.57E+08	+	3215	1	NIPAL4|AF	0.024	0.009
NR_02986	chr14	1.01E+08	1.01E+08	+	69	1	MIR369|M	0	0
NM_1538	chr6	47656590	47697797	+	5113	1	ADGRF2|G	0	0.062
NR_03995	chr3	1.34E+08	1.34E+08	+	79	1	MIR4788|	0	0
NM_0017	chr12	4273736	4305356	+	6515	1	CCND2|KI	19.414	22.193
NM_0011	chr1	2.05E+08	2.05E+08	-	690	1	LEMD1|CT	27.842	25.766
NM_1525	chr9	82979585	83063128	-	5557	1	RASEF|RA	0.306	0.027
NM_0012	chr19	19192766	19201869	+	1197	1	RFXANK|A	40.738	35.77
NM_0013	chr7	47795291	47819846	+	801	1	C7orf69|-|	0	0
NM_0010	chr14	91271323	91417844	-	7511	1	CCDC88C|	8.113	9.428
NM_1816	chr21	30501872	30502090	-	217	1	KRTAP19-5	0	0
NR_13432	chr19	12064699	12077816	+	2807	1	ZNF844|-|	4.111	5.221
NR_02702	chr21	29748175	29764006	+	1088	1	GRIK1-AS1	0.178	0.063
NM_0012	chr1	2.13E+08	2.13E+08	-	1553	1	NSL1|C1or	5.214	4.957
NM_0010	chr2	2.41E+08	2.41E+08	+	6801	1	SNED1|SS	0.284	0.427
NR_00277	chr10	91152006	91153080	-	1074	1	NUDT9P1|	0	0.217
NR_00217	chrY	22936455	22973284	+	1342	3	TTTY4B|LI	0	0
NM_0179	chr19	49446293	49451858	-	1235	1	PIH1D1|N	56.669	53.668
NM_0143	chr9	32455302	32526324	-	4736	1	DDX58|RI	1.116	1.698
NR_13119	chr21	42599404	42615095	-	1686	1	LINC01671	0.008	0.008
NM_0157	chr17	4833340	4898061	+	4873	1	MINK1|B5	16.69	18.942
NM_0156	chr19	54115410	54131719	+	2167	10	PRPF31|N	0	0
NM_0529	chr12	1.25E+08	1.26E+08	+	10901	1	TMEM132	2.775	10.772
NR_12592	chr4	94117820	94207556	-	1005	1	LOC10192	0	0
NR_12638	chr13	42842412	42872744	-	591	1	LINC0042	0	0
NR_03175	chrX	1.54E+08	1.54E+08	-	69	1	MIR718|h	0	0
NM_0336	chr9	39072767	39288303	-	5203	1	CNTNAP3|	0.367	0.182
NM_1532	chr19	55600340	55603138	+	1129	1	ZNF524|-|	6.921	6.115
NR_02659	chr10	73699151	73730514	-	1948	1	BMS1P4|B	1.152	1.322
NM_0221	chr1	39769525	39771348	-	1821	1	OXCT2|FK	0.035	0.06
NR_11098	chr17	4271431	4271894	-	463	2	LOC10302	0.223	0.089
NM_0053	chr6_GL0	3071342	3073770	+	2426	5	HSPA1A|H	0	0
NM_0010	chr12	1.09E+08	1.09E+08	+	9195	1	ACACB|AC	1.509	1.518
NM_0010	chr19	7112255	7294302	-	9302	1	INSR|CD2	6.095	5.132
NM_0528	chr19	2511220	2702748	-	4239	1	GNG7|-|-|	8.1	15.749
NR_03983	chr1	22719517	22719598	+	81	1	MIR4684|	0	0

NM_2147:	chr4	70154187	70166609	+	4790	1	PRR27|C4(	0	0
NM_2034(	chr4	6707701	6709879	-	1588	1	MRFAP1L1	111.836	132.399
NM_0010:	chrX	48383516	48393343	+	1218	2	SSX4B|CT5	0	0
NR_00325	chrX	2752296	2774996	+	360	2	XGY2|XG|	0	0
NM_0011(	chr1	1.71E+08	1.71E+08	+	3141	1	MROH9|A	0	0
NR_02704	chr7	1.5E+08	1.5E+08	-	3065	1	ATP6V0E2-	5.881	7.467
NM_0011(	chr5	1.08E+08	1.08E+08	-	5189	1	FBXL17|FE	4.145	4.706
NR_02790	chr6	1.68E+08	1.68E+08	-	2309	1	AFDN-AS1	6.595	8.157
NR_11020	chr2	15690782	15718961	+	1865	1	LINC01804	0	0
NR_03890	chr11	46846411	46874416	+	1537	1	LRP4-AS1|	3.515	3.903
NR_00216	chr13	41442564	41443766	+	1202	1	OR7E37P|	0	0
NM_0013:	chr19	37632488	37655499	-	4273	1	ZFP30|ZNI	4.306	5.145
NM_0011:	chr1	1.48E+08	1.48E+08	-	735	1	PPIAL4G|C	0	0
NR_03173	chr20	63941465	63941544	-	79	1	MIR1914|I	1.959	1.04
NR_02841	chr12	1500491	1504424	+	2242	1	LINC00942	0.058	0.024
NM_0198!	chr2	74958648	74969732	+	690	1	POLE4|YH	9.194	20.129
NR_02428	chr10	31316528	31319095	-	2211	1	ZEB1-AS1|	3.656	3.147
NM_0008	chr5	60873832	60945078	-	2030	1	ERCC8|CKI	6.758	7.84
NM_0239:	chr12	10809094	10810168	-	1072	3	TAS2R9|T2	0	0
NM_0013:	chr10	68332011	68343196	+	2382	1	HNRNPH3	74.416	73.022
NM_0121!	chr2	1.62E+08	1.62E+08	+	3685	1	GCA|GCL|	1.48	3.546
NM_0145	chr3	8501807	8568125	+	1749	1	LMCD1|-|-	1.791	1.37
NM_0013:	chr17	55266222	55325176	+	5158	1	HLF|-|-|17	0.392	0.64
NM_2034!	chr9	4662294	4665272	+	2976	1	PLPP6|PDI	6.382	5.822
NR_00314	chr19	53431974	53444672	+	2930	1	TPM3P9|-	1.932	1.58
NM_0013:	chr4	39182404	39285810	+	4366	1	WDR19|A	8.168	8.072
NR_11072	chr19	37251885	37265535	+	908	1	LINC01535	0.781	0.121
NM_0011!	chr18	49481178	49487274	-	1484	1	C18orf32|	44.884	44.737
NM_0123	chr1	17308195	17364000	+	2247	1	PADI4|PAI	0	0
NM_0166:	chr7	37848597	37900400	+	2291	1	NME8|CIL	0	0
NM_0011(	chr15	78540405	78549262	+	1175	1	PSMA4|H(	91.472	112.409
NM_0013:	chr16	53695631	53703938	-	888	1	RPGRIP1L|	1.655	1.388
NM_0011:	chr19	51022231	51026616	-	1173	1	KLK11|PRS	0	0
NR_10978	chr17	79707266	79712317	+	4235	1	LINC02078	0.085	0.01
NR_03613	chr15	90006755	90006841	+	86	1	MIR3174|-	0	1.274
NR_11100	chr1	1.44E+08	1.44E+08	-	2209	2	DRD5P2|-	0	0
NR_02407	chr19	6952500	6990846	-	2716	1	ADGRE4P|	0	0.005
NR_00331	chr15	25201323	25201404	+	81	3	SNORD115	0	0
NR_02336	chr1	2.29E+08	2.29E+08	-	120	10	RNA5S1|R	0	0
NM_0012	chr17	59619689	59696956	+	8553	1	CLTC|CHC	45.549	48.864
NR_11006	chr12	67709047	67729475	-	3130	1	LOC10192	0	0.048
NM_0189:	chr5	1.41E+08	1.41E+08	+	5215	1	PCDHA7|C	16.612	15.885
NM_0011:	chr17	74579972	74592231	-	638	1	CD300LD|	0	0
NR_00245	chr4	425435	474209	-	1892	1	ABCA11P|	3.094	3.895
NM_0010:	chr22	32149532	32159256	-	794	1	C22orf42|	5.505	7.608
NM_0012!	chr1	975199	982117	-	3058	1	PERM1|C1	0.017	0.009
NR_10703	chr11	28477481	28477548	-	67	1	MIR8068|I	0	0
NM_0059!	chr16	56669814	56671129	+	392	1	MT1H|MT	0	0
NM_0048!	chr19	50358472	50365830	-	1542	1	NAPSA|KA	0.485	0.266
NM_0012	chrX	1.53E+08	1.53E+08	+	1659	2	MAGEA2|(	0	0

NM_0013	chr1	2.01E+08	2.01E+08	-	1739	1	DDX59|O	6.406	7.333
NM_0003	chr1	68428824	68449959	-	2592	1	RPE65|BC	0.378	0.888
NM_1532	chr9	68536582	68540867	-	1239	1	TMEM252	0.458	0
NR_03668	chr8	6987178	6989721	-	493	2	DEFT1P|D	0	0
NR_02715	chr4	53985499	53987282	-	1783	1	RPL21P44	0.08	0.161
NM_0011	chr19	2997638	3029614	-	2262	1	TLE2|ESG|	4.001	16.682
NR_03963	chr2	52702522	52702615	-	93	1	MIR4431|	0	0
NM_0218	chr15	74812853	74825758	+	1720	1	LMAN1L|E	0.12	0.016
NR_02740	chr9	1.24E+08	1.24E+08	+	5711	1	LOC10012	22.886	27.332
NM_0010	chr1	1.53E+08	1.53E+08	-	667	1	SPRR2F|-|	0	0
NR_00328	chr22_KI2	130204	135280	+	5069.5	4	RNA28S5|	0.305	0.281
NM_0190	chr12	19129692	19376399	+	4311	1	PLEKHA5|	8.802	7.244
NM_0067	chr11	747432	765024	+	1233	1	TALDO1|T	130.692	124.595
NM_0012	chr19	43984170	43998325	+	2608	1	ZNF155|p	5.888	6.103
NM_0054	chr18	20949742	21111851	-	6613	1	ROCK1|P1	1.841	2.5
NR_13118	chr21	37063041	37064066	-	1025	1	LOC10537	0.893	0.414
NM_0012	chr6	4995046	5004063	-	1050	1	RPP40|RN	5.415	5.101
NM_0048	chr6	1.34E+08	1.34E+08	+	1319	1	TBPL1|MG	22.542	28.754
NM_0012	chr1	1.17E+08	1.17E+08	-	2283	1	VTCN1|B7	0.401	0.864
NM_0210	chr11	644220	695754	-	1746.5	2	DEAF1|M	43.025	46.82
NM_0010	chr7	64990355	65006746	-	3262	1	ERV3-1|E	15.289	15.377
NM_0011	chr11	61508800	61511018	+	2216	1	LRRC10B|	14.232	14.259
NR_12161	chr6	71407865	71420745	-	9496	1	LINC00472	0.539	0.739
NM_0007	chr19	40991299	41018396	+	3041	1	CYP2B6|C	0	0
NM_0041	chr22	31399553	31434186	+	1439	1	DRG1|NE	47.506	48.468
NM_0012	chr20	23491101	23496018	+	753	1	CST8|CRE	0	0
NM_1523	chr12	1E+08	1E+08	-	1147	1	DEPDC4|D	0.416	0.669
NR_00305	chr11	46762389	46762499	-	110	1	SNORD67|	0	0
NM_0047	chr1	1.81E+08	1.81E+08	+	8479	1	XPR1|IBG	29.827	39.249
NR_00392	chr1	1.11E+08	1.11E+08	+	2795	1	CHIAP2|-|	0	0
NR_02406	chr6	28090807	28095715	+	4053	1	ZSCAN12P	0.872	1.348
NR_03389	chr6	1.39E+08	1.39E+08	+	997	1	FLJ46906|	3.712	3.435
NM_0121	chr17	81712236	81721016	+	1981	1	SLC25A10|	5.981	3.277
NR_00367	chr9	39813784	39874566	-	4704	1	FGF7P3|K	0.014	0.087
NM_0245	chr6	1.51E+08	1.51E+08	+	2596	1	ARMT1|C	4.823	3.873
NM_0024	chr11	1.03E+08	1.03E+08	-	1869	1	MMP12|H	0.014	0.125
NM_0120	chr8	1.44E+08	1.44E+08	-	3702	1	DGAT1|A	16.408	17.179
NM_0012	chr12	56663341	56688408	-	1922	1	PTGES3|P	69.988	78.419
NM_0011	chr12	25959031	26072874	+	5535	1	RASSF8|C	1.377	1.339
NM_0013	chr15	64165517	64356259	-	2260	1	CSNK1G1|	6.458	8.079
NM_0019	chr1	1.97E+08	1.97E+08	-	2203	1	F13B|FXIII	0	0
NM_0012	chr10	1.02E+08	1.02E+08	-	3362	1	PSD|EFA6	44.061	41.034
NM_0165	chr9	1.3E+08	1.3E+08	-	1807	1	C9orf78|H	33.636	34.64
NM_0012	chr2	2.15E+08	2.15E+08	-	5437	1	BARD1|-|-	1.055	1.081
NM_0040	chr22	25199850	25207359	+	855	1	CRYBB3|C	0.015	0.032
NR_04979	chr5	1.73E+08	1.73E+08	+	98	1	MIR5003|	0	0
NM_0028	chr1	1.54E+08	1.54E+08	-	1225	1	RAB13|GI	19.177	12.993
NM_1833	chr3	10300929	10321188	-	1426	1	SEC13|D3	87.904	73.576
NM_0224	chr7	1.23E+08	1.23E+08	-	3780	1	SLC13A1|	0	0
NR_13447	chr10	42871521	42874060	+	1712	1	LOC10192	0	0

NM_0204(	chr9	1.33E+08	1.33E+08	-	1570	1	ABO|A3G/	0	0
NM_0060:	chr1	26696031	26782110	+	8563	1	ARID1A|B:	42.841	36.241
NM_0012:	chr15	42412437	42491197	-	8431	1	ZNF106|SI	7.163	7.21
NM_0174:	chr10	25934073	26212536	+	5747	1	MYO3A|D	0.056	0.243
NM_0147:	chr16	1510427	1612108	-	5235	1	IFT140|M:	21.854	19.887
NR_13077	chr16	33769377	33784275	+	5346.5	2	ENPP7P13	0	0
NM_0012:	chr2	2.19E+08	2.19E+08	+	809	1	LOC10099	0.367	0.491
NM_0024:	chr2	1.34E+08	1.34E+08	+	8127	1	MGAT5|GI	27.048	40.958
NM_0153	chr3	45388583	45548836	+	4179	1	LARS2|HL/	12.483	12.098
NM_0541:	chr6	33721666	33746985	-	2686	1	IP6K3|IHP	0.019	0.01
NM_0010(	chr7	1.42E+08	1.42E+08	+	943	1	OR9A4|-|-	0	0
NM_0052:	chr19	17231885	17245342	-	1798	1	NR2F6|EA	19.182	11.732
NR_02950	chr22	41900944	41901012	+	68	1	MIR33A|M	0	0.201
NM_0011(	chr11	1.18E+08	1.18E+08	-	1794	1	FXYD6|-|-	327.655	394.931
NM_0227(	chr15	88621296	88632281	+	3110	1	AEN|ISG2(	13.432	16.679
NR_11060	chr2	2.15E+08	2.15E+08	+	818	1	LOC10272	0	0
NR_02443	chr1	1.04E+08	1.04E+08	+	1982	1	ACTG1P4|	0.631	0.926
NM_0147:	chr1	46675159	46719064	-	5754	1	EFCAB14|I	5.302	5.368
NM_1817!	chr17	74367506	74372600	+	1384	1	GPR142|G	0	0.02
NM_0010:	chr7	6329409	6348959	-	2210	1	FAM220A|	16.82	23.304
NM_0010:	chr1	13281304	13285137	-	1552	2	PRAMEF8|	0	0
NR_03396	chr5	71220226	71259295	-	590.9	7	GUSBP9|-	1.004	0.835
NM_0010(	chr2	1.27E+08	1.27E+08	-	4675	1	CYP27C1|-	0.425	0.202
NM_0012:	chr16	2253116	2267337	-	2036	1	RNPS1|E5.	65.512	62.565
NM_0010	chr12	32746544	32755953	-	2166	1	YARS2|CG	5.203	5.742
NM_0133:	chr1	63367590	63438562	+	3354	1	ALG6|CDG	1.795	1.695
NM_0123:	chr5	40711576	40755970	-	5499	1	TTC33|OSI	5.062	4.127
NM_0013(	chr20	58216126	58228653	-	3402	1	ANKRD60|	0	0
NR_03405	chr2	2.31E+08	2.31E+08	+	3131	1	PSMD1|P1	41.089	46.599
NR_00392	chr5	71375785	71385993	+	1837.3	3	PMCHL2|-	0	0
NM_0203:	chr19	55485190	55487568	+	1345	1	NAT14|KLI	75.961	51.56
NR_10377	chr1	1.52E+08	1.52E+08	+	2854	1	FLG-AS1|-	0	0
NM_0043(	chr16	68737290	68835542	+	4800	1	CDH1|Arc-	0.067	0.419
NM_0037:	chr3	50317790	50322850	-	1999	1	HYAL2|LU(	43.95	41.036
NM_0011(	chr5	93617725	94111699	-	4393	1	FAM172A|	12.946	9.416
NM_2012:	chr12	12813346	12829981	+	1657	1	DDX47|E4	25.515	32.436
NM_1392:	chr9	33461353	33473943	-	3494	1	NOL6|NR/	7.908	8.562
NR_03409	chr2	46816668	46822660	+	1039	1	LINC0111٤	0.025	0
NM_0011!	chr1	42176539	42335215	-	5098	1	FOXJ3|-|-|	12.329	19.382
NM_0334:	chr9	37887597	37904353	-	1399	1	SLC25A51|	3.751	3.828
NM_0012(	chr7	1.22E+08	1.22E+08	+	5527	1	PTPRZ1|HI	14.431	132.866
NR_03898	chr6	1.32E+08	1.32E+08	+	999	1	LINC01013	0.129	0.041
NM_1445:	chr19	48296452	48320075	-	3204	1	CCDC114|	0.185	0.133
NR_10464	chrX	45505388	45527239	+	2920	1	LINC01204	0.066	0.014
NM_1817:	chr12	48328980	48330279	+	1297	1	H1FNT|H1	0.229	0.127
NR_12042	chr13	1.12E+08	1.12E+08	-	1928.5	2	LOC10192:	0	0
NR_03887	chr13	68861285	68885325	-	3430	1	LINC0055C	0	0
NM_0042(	chr16	569968	584136	+	2972	1	PIGQ|GPI1	28.39	21.02
NM_0140!	chr4	68447449	68497604	+	2124	2	TMPRSS11	0	0
NM_0012	chr18	55222331	55510769	-	7734	1	TCF4|E2-2	5.28	41.75

NM_0176!	chr6	1.19E+08	1.19E+08	-	4808	1	MCM9|C6	1.142	1.105
NR_04974	chr15	84200078	84200689	-	611	3	LOC64242	0.717	0.897
NM_0210	chr1	56997906	58250539	-	2561	1	DAB1|-|-|	5.13	13.804
NR_00271	chr1	1.71E+08	1.71E+08	+	2428	1	TOP1P1|-|	1.752	1.766
NM_0207	chr5	1.44E+08	1.44E+08	+	5177	1	KCTD16|-|	0.677	2.498
NM_0325	chr3	66998303	67011208	+	4676	1	KBTBD8|T	0.56	0.425
NM_0012!	chr13	75871038	75883812	+	1162	1	LMO7DN|	0	0.024
NM_0011	chr8	17643794	17722575	-	3637	1	MTUS1|A	7.285	6.858
NR_02737	chr7	1.05E+08	1.05E+08	-	1828	1	LHFPL3-AS	0.042	0.022
NM_0012	chr12	81257975	81758507	-	5507	1	PPFIA2|-|-	8.007	7.477
NM_0191	chr7	27141052	27143668	-	1653	1	HOXA5|H	0	0.157
NM_0150	chr1	19215664	19251559	-	6646	1	EMC1|CA	12.496	13.765
NM_0071	chr3	1.3E+08	1.3E+08	+	1885	1	TRH|Pro-T	33.564	3.989
NM_0044	chr9	23690105	23826065	-	3798	1	ELAVL2|H	45.915	36.897
NM_0012	chr2	2.1E+08	2.1E+08	+	3166	1	RPE|RPE2-	4.089	3.898
NM_0013	chr5	1.32E+08	1.32E+08	-	1545	1	MEIKIN|-|	0	0
NM_0011	chr11	66002079	66004146	+	1090	1	BANF1|BA	70.63	63.32
NM_0163!	chr5	1877427	1882766	-	2214	1	IRX4|IRXA	3.395	4.349
NR_00333	chr15	25091785	25091878	+	93	1	SNORD116	0.555	0.147
NM_0011!	chr20	45405993	45410610	+	987	1	DBNDD2|	33.573	34.472
NM_0187	chr12	1.09E+08	1.09E+08	-	6407	1	SVOP|-|-|	13.439	13.532
NM_0012!	chr12	51424810	51474577	+	2301	1	SLC4A8|N	36.635	32.561
NM_0064!	chr17	31303770	31314112	-	1991	1	EVI2B|CD	0	0
NM_1827	chr8	47960203	47978159	+	4780	1	MCM4|CD	13.738	12.697
NM_1382	chrX	1.28E+08	1.28E+08	-	1439	1	ACTRT1|A	0	0
NR_04558	chr17	28648356	28662189	-	1259	1	SDF2|-|-|	49.515	55.745
NM_0013	chr20	45091216	45101112	-	5424	1	KCNS1|Kv	0.048	0.023
NM_0012!	chr12	8645943	8662888	-	2893	1	MFAP5|A	0.009	0
NM_0011!	chr15	26543546	26716765	-	5572	1	GABRB3|E	17.498	26.73
NR_07358	chr5	62578735	62628589	+	1814	1	IPO11-LRR	4.123	3.511
NM_1387	chr6	1.5E+08	1.5E+08	+	1099	1	GINM1|C6	9.245	16.289
NM_0012	chr11	36594493	36659291	+	666	1	C11orf74|	20.173	31.26
NR_10386	chr12	67520082	67567131	+	1768	1	LOC10050	0.095	0.302
NM_0012	chr17	15436015	15467611	-	2492	1	CDRT4|-|-	3.28	9.036
NM_1736	chr13	50015254	50020922	+	1523	1	KCNRG|DL	5.74	6.412
NM_0012	chr9	1.21E+08	1.21E+08	-	4143	1	PHF19|MT	1.621	2.85
NR_02446	chr11	67317839	67391687	+	846	1	LOC10013	2.545	3.044
NR_03786	chr4	1.38E+08	1.38E+08	-	2005	1	LINC00616	0.045	0.041
NM_0156	chr19	2476125	2478259	+	1369	1	GADD45B|	4.182	2.811
NM_0004!	chr19	17871973	17895174	+	3559	1	SLC5A5|N	0.062	0.046
NM_0320	chr6	1.43E+08	1.44E+08	-	2539	1	FUCA2|dJ	7.262	3.76
NM_0010	chr3	1.31E+08	1.31E+08	+	3434	1	ATP2C1|A	23.955	27.238
NM_0031	chr17	28662198	28702684	+	6377	1	SUPT6H|S	32.322	33.309
NM_0035	chr6	26188710	26189076	-	365	1	HIST1H4D	0.565	0.3
NM_0009	chr3	1.02E+08	1.02E+08	-	554	1	RPL24|HE	340.164	308.554
NR_02985	chrX	1.34E+08	1.34E+08	-	74	1	MIR363|M	0	0
NM_0177	chrX	40051246	40097466	-	6304	1	BCOR|AN	26.738	22.603
NM_0070	chr17	49788619	49829096	+	3629	1	KAT7|HBO	18.02	26.669
NM_0013	chr19	54461796	54463711	-	1912.5	4	LENG9|-|-	0	0
NM_1388	chr6	1.59E+08	1.59E+08	-	1307	1	TAGAP|AR	0.197	0.398

NR_02747	chr15	30103547	30131734	+	871	5	ULK4P2|D	0	0.031
NM_0056(	chr14	92703807	92748702	-	2041	1	LGMN|AEI	23.93	24.099
NR_12536	chr17	10383132	10625540	+	3929	1	MYHAS|-|	0	0
NM_0160(	chr2	43774039	43810010	+	1384	1	DYNC2LI1|	10.602	8.462
NM_1384:	chr6	1.36E+08	1.36E+08	-	1519	1	MTFR2|DU	0.569	0.325
NM_0176:	chr17	8188333	8190246	-	1911	1	BORCS6|C	11.922	9.604
NM_0013(	chr22	35066136	35087387	+	3088	1	ISX|Pix-1|I	0.013	0
NM_0141!	chr3	1.38E+08	1.38E+08	+	3206	1	ARMC8|GI	9.053	11.279
NM_1383!	chr19	1491005	1497925	+	1527	1	REEP6|C1!	12.843	11.867
NR_13488	chr19	45085329	45090391	-	633	1	LOC10537.	13.545	13.156
NM_0041:	chr20	31844300	31845617	-	1315	1	FOXS1|FKI	0.049	0
NM_0319!	chr17	40999193	40999886	-	691	2	KRTAP3-2|	0	0
NR_03034	chr11	28056815	28056910	+	95	1	MIR610|M	0	0
NR_03990	chr1	2.35E+08	2.35E+08	-	82	1	MIR4753|	0	0
NM_0068:	chr20	1118593	1172246	+	8154	1	PSMF1|PI3	20.689	18.669
NM_0011:	chr3	56620132	56683107	-	8270	1	FAM208A|	4.287	5.807
NM_0013(	chr20	63735701	63739107	+	1555	1	LIME1|LIM	9.171	8.985
NR_00231	chr1	53438370	53440020	-	923	1	SLC25A3P1	0	0
NM_1756:	chr19	5894670	5904013	-	797	1	NDUFA11|	152.457	132.021
NM_0027!	chr19	43167743	43186536	-	1632	1	PSG5|FL-N	0	0
NR_10417	chr6	1.7E+08	1.7E+08	+	3068.5	2	LINC01624	0	0
NR_02686	chr9	92809611	92838457	+	2297	1	ANKRD19P	1.145	1.181
NM_0011‹	chr12	6618835	6631649	-	2691	1	LPAR5|GP	0.153	0.066
NM_0010:	chr10	7158624	7409341	-	7899	1	SFMBT2|-	2.95	3.742
NM_0311!	chr12	54280690	54285246	+	1926	1	HNRNPA1|	29.951	26.491
NM_0012!	chr15	36594869	36808489	+	818	1	C15orf41|	10.214	9.662
NR_11009	chr15	82750564	82757206	+	796	1	SNHG21|F	2.948	2.736
NM_0013:	chr5	1.39E+08	1.39E+08	+	2464	1	CTNNA1|C	111.243	99.798
NM_0010(	chr11	5453408	5454477	+	1067	1	OR51I2|OI	0	0.013
NM_0334‹	chr1	15456728	15472091	+	910	1	CELA2A|EI	0.028	0.151
NR_03716	chr19	56393676	56399170	+	1314	1	ZNF582-AS	9.018	15.605
NM_0010:	chr10	26746596	26861087	-	3617	1	ABI1|ABI-1	7.725	9.035
NM_0011!	chr19	3630181	3700479	-	4979	1	PIP5K1C|L	27.431	33.518
NM_0013!	chrX	1.54E+08	1.54E+08	+	2374	1	DUSP9|MI	1.428	2.937
NR_03384	chr10	1.26E+08	1.26E+08	-	2522	1	FLJ37035|	0.818	0.673
NR_07509	chr3	1.81E+08	1.82E+08	+	3432	1	SOX2-OT|I	0.853	2.263
NM_1757:	chr3	3090173	3110374	-	1641	1	IL5RA|CD1	0	0.008
NR_04511	chr16	11555	14090	+	1649	1	DDX11L10	0	0
NR_12604	chr9	39803496	39810159	-	1036	1	GLIDR|LIN	1.207	1.243
NR_03157	chr5	55172262	55172353	-	91	1	MIR449C|	0	0
NM_0010(	chr12	21015696	21090106	+	1909	1	SLCO1B7|I	0.007	0
NM_2033!	chr11	494512	507283	-	1743	2	RNH1|RAI	0	0
NR_10352	chr6	83853543	83857516	+	568	1	RIPPLY2|C	1.43	0.916
NM_0010:	chrX	69160738	69165522	-	2550	1	PJA1|PRAJ	83.909	110.887
NM_0061:	chr12	52674736	52680407	-	2440	1	KRT1|CK1	0	0
NM_0124(	chr22	50274979	50307572	-	6296	1	PLXNB2|M	60.722	50.326
NM_0010:	chr1	1.1E+08	1.1E+08	+	6384	1	SLC6A17|I	8.194	3.868
NM_2034(	chr17	46978156	46979248	-	1090	1	RPRML|-|-	2.212	2.186
NM_0011:	chr7	1.17E+08	1.17E+08	+	2431	1	CAV1|BSC	0.44	1.668
NM_0011(	chr16	25216964	25228932	+	1300	1	AQP8|AQI	0.278	0.316

NM_1390	chr20	157470	159163	+	510	1	DEFB127|	0	0
NM_0012	chr10	46461099	46465881	-	1796	2	NPY4R|NP	0.014	0.023
NM_0022	chr3	49121114	49133166	-	5774	1	LAMB2|LA	16.654	11.702
NM_0011	chr19	17747718	17788568	+	3091	1	FCHO1|-|-	26.755	24.026
NM_0010	chr19	44699151	44710714	+	1683	1	CEACAM1	0	0
NR_13583	chr1	86703498	86704493	-	995	1	LOC10537	0.531	0.977
NR_02680	chr4	38612701	38664628	-	2360	1	KLF3-AS1|	1.634	2.182
NM_0044	chr1	1.55E+08	1.55E+08	+	1559	1	EFNA1|B6	4.226	8.909
NM_0000	chr1	2.36E+08	2.36E+08	-	13442	1	LYST|CHS|	1.376	3.138
NR_11073	chr19	49856465	49859289	-	1096	1	PTOV1-AS	54.986	50.939
NM_0219	chr1	2.21E+08	2.21E+08	+	2272	1	HLX|HB24	0.034	0.048
NM_0068	chr6	1.47E+08	1.47E+08	+	1204	1	RAB32|-|-	1.446	1.217
NM_0013	chr1	9729021	9824526	-	5149	1	CLSTN1|AI	110.778	134.129
NM_1737	chr22	19443150	19448232	-	1972	1	C22orf39|	12.083	10.717
NM_0150	chr1	23019448	23083691	+	3014	1	KDM1A|A	162.383	176.248
NR_03749	chr8	12727232	12727304	-	72	1	MIR3926-1	0	0
NM_0207	chr5	65722196	65829283	+	8646	1	NLN|AGTE	8.045	6.784
NM_0012	chr15	66336670	66356716	-	1195	1	TIPIN|-|-|	4.305	5.02
NM_0124	chr1	40161369	40240921	+	6240	1	RLF|ZN-15	3.659	4.774
NM_1735	chrX	26192440	26195646	+	1945	1	MAGEB6|	0	0
NR_13394	chr7	1.01E+08	1.01E+08	+	527	1	LOC10537	2.055	3.457
NM_0012	chr10	24466505	24545316	+	3071	1	KIAA1217|	0.546	1.285
NM_0012	chr8	1.44E+08	1.44E+08	-	1819.5	2	PUF60|FIR	0.184	0.181
NM_0010	chrX	41688973	41697277	+	1923	1	GPR34|LYI	0.04	0.021
NR_03970	chr10	1.04E+08	1.04E+08	-	69	1	MIR4482|	0.934	0.199
NM_0013	chr3	1.12E+08	1.12E+08	+	1626	1	CD96|TAC	0.095	0.051
NM_1525	chrX	1.48E+08	1.48E+08	+	1013	1	FMR1NB|	0	0.054
NM_0065	chr3	47562238	47578869	-	2151	1	CSPG5|NG	17.581	21.943
NM_0037	chr17	50634857	50691702	+	5133	1	ABCC3|AB	0.048	0.016
NM_0041	chr5	1.39E+08	1.39E+08	-	3487	1	HSPA9|CR	61.75	66.258
NM_0013	chr5	1.11E+08	1.11E+08	+	11687	1	CAMK4|Ca	1.492	2.064
NM_1979	chr4	86823468	86849263	-	1494	1	SLC10A6|	0.035	0.009
NM_0331	chr3	48561718	48563768	-	1481	1	UCN2|SRP	0.122	0.074
NM_0010	chr11	48324941	48325930	+	988	1	OR4C3|OF	0	0
NM_0020	chr19	3136032	3163769	+	2285	1	GNA15|GN	0.028	0.024
NR_12580	chr8	24295814	24548618	-	881	1	LOC10192	0	0
NR_02725	chr17	81165507	81183164	+	1571	1	AATK-AS1|	0.14	0.017
NM_1451	chr6	1.34E+08	1.34E+08	-	5592	1	SLC2A12|	1.153	1.186
NR_02442	chr10	78943326	79067448	-	2229	1	ZMIZ1-AS1	0.48	0.602
NM_0013	chr2	9474529	9488624	+	988	1	IAH1|-|-|2	0.287	2.689
NM_0012	chr14	72619296	72894116	-	1153	1	DPF3|BAF	4.238	7.021
NR_00350	chr2	1.06E+08	1.06E+08	+	837	1	PLGLA|PL	0	0
NR_03368	chr1	31629862	31644617	-	1815	1	PEF1|ABP	56.902	52.63
NM_0011	chr11	1.19E+08	1.19E+08	-	1708	1	IFT46|C11	26.98	30.762
NM_0528	chr1	1.68E+08	1.68E+08	+	2982	1	RCSD1|CA	0	0
NM_0012	chr9	35673918	35681157	+	1541	1	CA9|CAIX|	0.067	0.169
NM_0035	chr6	26123467	26123904	-	436	1	HIST1H2B	0.798	0.157
NM_0010	chr11	1.04E+08	1.04E+08	+	2612	1	DDI1|-|-|1	0	0
NM_0153	chr9	34086383	34126773	-	3660	1	DCAF12|C	33.35	36.544
NM_0013	chr10	1.11E+08	1.11E+08	+	3893	1	SHOC2|SI	8.869	11.09

NM_0040:	chr11_KI2	142448	145898	-	1873	2	IRF7|IMD3	0.089	0.08
NM_0055{	chr14	74057665	74084493	-	4670	1	ALDH6A1|	4.981	4.699
NR_04636	chr19	53282290	53284349	+	493	1	FAM90A27	0	0
NM_0010(	chr7	1.44E+08	1.44E+08	+	931	1	OR2A12|C	0	0
NM_0041(	chr16	66921679	66925536	-	1460	1	RRAD|RAD	0.839	0.685
NM_1990:	chr3	49020475	49023493	+	888	1	NDUFAF3|	49.294	39.024
NM_2014(	chr8	12137168	12138760	-	1591	1	USP17L2|I	0	0.009
NR_03162	chr1	1.71E+08	1.71E+08	-	78	1	MIR1295A	0	0
NR_02681	chr15	1.02E+08	1.02E+08	+	1127	1	FAM138E|	0.023	0
NR_11000	chr20	42685483	42688562	+	1991	1	LOC10192	0	0
NM_0012·	chr11	67393305	67398412	+	1976	1	RAD9A|RA	10.962	12.941
NR_00307	chr2	1.01E+08	1.01E+08	-	113	1	SNORD89|	3.309	2.667
NM_0134:	chr18	59267035	59273393	-	3192	1	RAX|MCO	0.016	0.026
NM_0011:	chr16	71845991	71857333	+	7881	1	ATXN1L|B	8.403	8.893
NM_0528:	chrX	1.08E+08	1.08E+08	+	7243	1	MID2|FXY	0.139	0.488
NR_00153	chrY	6449468	6457906	+	1360	2	TTTY7|CLC	0	0
NM_0012{	chr5	73565741	73583377	+	5023	1	UTP15|NE	1.283	1.672
NM_0011·	chr15	91853708	92172435	+	2840	1	SLCO3A1|	5.266	4.321
NM_0030{	chr7	5592805	5606656	+	2789	1	FSCN1|FAI	189.935	194.99
NM_0010:	chr11	6603708	6610874	+	1768	1	ILK|HEL-S-	95.527	93.989
NM_0010(	chr2	2.2E+08	2.2E+08	+	2229	1	TMEM198	24.59	23.855
NM_0144!	chr2	71331755	71435061	+	6484	1	ZNF638|N	8.38	13.851
NM_1383:	chr9	68780006	68783879	+	3871	1	FAM122A|	6.705	8.814
NR_03979	chr7	75915197	75915269	+	72	1	MIR4651|	0	0
NM_0071:	chr9	1.27E+08	1.27E+08	+	2079	1	ZNF79|pT	6.481	7.168
NM_0055:	chr6_GL0C	3142241	3147680	-	2533	5	HSPA1L|H	0	0.011
NR_00371	chr5	1597557	1634005	-	2572	1	LOC72861	0.511	0.66
NM_0207(	chr3	1.29E+08	1.29E+08	-	3765	1	ISY1|FSAP	11.376	10.492
NM_0156{	chr6	75308084	75493914	-	4748	1	FILIP1|FILI	0.451	1.719
NM_0806:	chr20	56491492	56525925	-	2055	1	GCNT7|C2	0.038	0.113
NM_0309:	chr17	41255384	41256364	+	977.7	3	KRTAP9-9|	0	0
NM_0314·	chr18	35972625	35979287	+	1041	1	C18orf21|	13.661	15.263
NM_0012{	chr13	75284664	75482168	-	6216	1	TBC1D4|A	1.247	0.712
NM_0010(	chrX	1.43E+08	1.43E+08	+	427	1	SPANXN4|	0	0
NM_0123:	chr1	32108043	32176567	+	7356	1	KPNA6|IP(	17.104	19.3
NM_0039(	chr8	86514427	86561498	+	4846	1	CPNE3|CP	15.799	16.213
NM_1991:	chr1	1.56E+08	1.56E+08	+	737	1	BGLAP|BG	5.283	5.074
NR_03717	chr6_GL0C	4137694	4147277	+	3589	8	LOC10029	0	0
NR_12540	chr3	1.94E+08	1.94E+08	-	869	1	LINC02036	0.045	0.378
NM_0142{	chr9	1.31E+08	1.31E+08	+	2023	1	EXOSC2|R	17.521	18.376
NM_0011:	chrX	1.3E+08	1.3E+08	-	4085	1	ELF4|ELFR	0.619	0.349
NM_0010(	chr3	98132698	98133639	+	940	1	OR5H1|HS	0	0
NM_0012{	chr14	24232872	24239241	+	1556	1	GMPR2|G	43.23	44.005
NM_0010(	chr17	30477408	30526814	+	5198	1	GOSR1|GC	5.514	5.916
NM_1528!	chr16	2783953	2786707	-	1686	1	PRSS33|E(	0.245	2.12
NM_0013(	chr1	1.51E+08	1.51E+08	+	2871	1	PRUNE1|D	25.818	36.979
NM_0055(	chr4	76158739	76213899	-	4753	1	SCARB2|A	59.313	55.457
NM_0011:	chr2	1.79E+08	1.8E+08	-	2609	1	ZNF385B|	0.213	0.719
NM_0010:	chr17	18971068	19004747	-	2922	1	FAM83G|	0.569	0.197
NM_0245:	chr13	50909678	50956765	+	1616	1	RNASEH2B	1.237	5.628

NR_12600	chr1	11777077	11779619	-	1865	1	LOC10272	0	0.029
NR_02680	chr6	1.35E+08	1.36E+08	+	1632	1	LINC00271	0.158	0.151
NR_03158	chr12	1.2E+08	1.2E+08	-	90	1	MIR1178|	0.86	0.457
NM_0012	chr2	1.35E+08	1.35E+08	-	1763	1	MAP3K19|	0.059	0.016
NM_0530	chr11	58579114	58621550	+	5712	1	ZFP91|DM	18.017	20.025
NR_03829	chr12	1.23E+08	1.23E+08	+	2188	1	LOC10050	0.012	0
NR_04677	chr21	40618507	40630767	-	716	1	DSCAM-IT	0	0
NR_13515	chr14	85387275	85388765	+	508	1	LOC10537	0	0
NM_0175	chr19	39391303	39400637	+	3655	1	MED29|IX	26.186	22.736
NM_0010	chr18	9136753	9285985	+	11229	1	ANKRD12|	0.922	2.083
NM_0010	chr2	1.76E+08	1.76E+08	-	1612	1	EVX2|EVX	19.068	0.28
NM_0010	chr11	48488793	48489722	+	928	1	OR4A47|C	0	0.015
NR_02967	chr17	2049908	2050008	-	100	1	MIR132|M	0	0
NM_0011	chr5	33986986	34008115	-	2595	1	AMACR|A	7.274	4.787
NM_0313	chr5	75371103	75511629	-	5298	1	COL4A3BP	3.66	3.8
NM_0051	chr10	7788104	7807799	+	1124	1	ATP5C1|A	143.778	164.267
NM_0060	chr3	51385237	51389397	+	957	1	MANF|AR	79.894	56.033
NM_0012	chr21	39612940	39662889	+	2613	1	B3GALT5|	0.207	0.823
NM_1731	chr2	1.13E+08	1.13E+08	+	2660	1	IL36RN|FI	0	0
NM_0011	chr8	1.23E+08	1.23E+08	+	3194	1	TBC1D31|	0.973	1.578
NM_0174	chr4	1.73E+08	1.73E+08	+	4293	1	GALNT7|G	2.763	2.224
NM_0327	chr10	75397830	75401894	-	3331	1	ZNF503|N	4.494	4.462
NM_1446	chr19	38730192	38744474	-	3106	1	CAPN12|-	2.561	1.98
NM_0010	chr2	98346155	98398601	+	3785	1	CNGA3|A	11.224	7.415
NM_0011	chr4	1.53E+08	1.54E+08	+	4982	1	KIAA0922|	7.49	7.464
NM_0334	chr1	1635226	1659097	-	2804	1	CDK11B|C	8.617	9.037
NM_0247	chr19	53163299	53193366	-	3285	1	ZNF665|Z	0.243	0.805
NM_0181	chr6	42206801	42217895	-	2131	1	MRPS10|N	9.142	10.58
NM_0012	chr5	55851379	55922854	+	2642	1	IL31RA|CR	0.034	0.062
NM_0139	chrX	1.01E+08	1.01E+08	-	2368	1	NOX1|GP	0.071	0.069
NR_12593	chr4	1.84E+08	1.84E+08	-	814	1	LOC10272	0.016	0.034
NR_04750	chr12	53999022	54000010	-	546	1	HOXC-AS1	0	0.05
NM_0012	chr15	70831524	70853458	-	1911	1	LARP6|AC	14.891	34.375
NM_0026	chr17	4807101	4823432	+	3469	1	PLD2|PLD	2.736	4.805
NR_13079	chr22	39502230	39514224	+	1897	1	MIEF1|HS	21.22	22.087
NM_0012	chr3	42547967	42601080	-	1541	1	SEC22C|SE	20.03	19.857
NR_13529	chr2	1.09E+08	1.09E+08	-	552	1	GCC2-AS1	0.35	0.397
NR_03982	chr1	2.43E+08	2.43E+08	+	79	2	MIR4677|	0	0
NM_0321	chr19	8490056	8502654	-	2197	1	PRAM1|P	0.2	0.218
NM_0011	chr10	93566665	93590072	+	3600	1	FFAR4|BM	0.29	0.221
NM_0140	chr16	72093716	72112912	+	4425	1	DHX38|DD	21.116	20.265
NR_13523	chr2	52370648	52390070	+	677	1	LINC01867	0.095	0.04
NM_0056	chrX	48383527	48393343	+	1207	2	SSX4|CT5.	0	0
NM_0010	chr15	81332419	81374077	-	3280	1	TMC3|-|-|	0.236	0.163
NM_0012	chr16	60357685	60359793	-	2106	1	LOC72915	0	0
NR_03411	chr13	1.06E+08	1.06E+08	+	911	1	LINC0046	0.014	0
NR_14453	chr2	1.18E+08	1.18E+08	-	607	1	LOC10050	0.828	0.361
NR_13487	chr11	63289947	63311774	+	2179	1	SLC22A10|	0	0
NM_0013	chr6	1.59E+08	1.59E+08	+	627	1	C6orf99|y	0.062	0
NM_0018	chr6	75237675	75243928	-	695	1	COX7A2|C	269.39	259.1

NM_0006:	chr18	63123346	63319380	-	6487	1	BCL2|Bcl-2	1.676	2.384
NM_0010:	chr10	27398188	27414368	-	2522	2	PTCHD3|P	0	0
NM_0000	chr7	66075789	66093342	+	1917	1	ASL|ASAL|	9.342	7.595
NM_0321	chr16	68300615	68310965	-	1622	1	SLC7A6OS	18.617	17.522
NM_0208	chr8	12945674	13029775	+	9581	1	KIAA1456|	0.972	2.054
NM_1446	chr8	1.33E+08	1.33E+08	-	2069	1	TMEM71|	0.393	0.139
NR_11004	chr7	54759313	54804963	+	625	1	LOC10099	0.041	0.044
NM_0049:	chr2	15591621	15631111	+	2713	1	DDX1|DBP	54.806	51.724
NR_13633	chr16	678518	679777	-	712	1	LOC10537	0.29	0
NR_04750	chr12	53995208	53996785	-	501	1	HOXC-AS2	0	0
NR_00129	chr5	1.41E+08	1.41E+08	+	1972	1	PCDHGB8P	0.052	0.09
NR_13654	chr21	6059029	6076305	+	669	2	LINC01669	0	0
NR_04572	chr12	2676001	2691157	-	2226	1	CACNA1C-	0.712	1.028
NM_1444	chr6	1.51E+08	1.51E+08	+	8227	1	AKAP12|A	15.21	6.002
NM_0013	chr1	1.84E+08	1.84E+08	+	1873	1	TSEN15|C	9.865	12.995
NM_0175	chr2	2.19E+08	2.19E+08	-	1874	1	FEV|HSRN	0.034	0.022
NM_2015	chr1	2.37E+08	2.37E+08	+	6135	1	LGALS8|Ga	1.129	1.775
NM_0530:	chr4	72039132	72148201	+	1935	1	NPFFR2|G	0.08	0.078
NR_04671	chr3	1.75E+08	1.75E+08	-	804	1	NAALADL2	0.706	0
NR_12041	chr13	75604700	75635994	-	410	1	LMO7-AS1	3.742	5.078
NR_00356	chr22	18533864	18548828	-	2345	1	PI4KAP1|-	4.393	3.972
NR_03168	chr5	1.01E+08	1.01E+08	-	83	1	MIR548P|	0	0
NR_04628	chr16	28878957	28879921	-	529	1	ATP2A1-AS	10.627	4.868
NR_13445	chr10	98446321	98453805	+	928	1	LOC10192	0.236	0.207
NM_0011	chr1	43172330	43254358	+	4139	1	CFAP57|V	0.785	0.361
NM_0010	chr19	54250405	54257308	-	1958	5	LILRB5|CD	0	0
NM_0012	chr6	1.51E+08	1.51E+08	+	3131	1	MTHFD1L|	5.139	7.402
NM_0012	chr16_KI2	284551	791405	+	1149	5	NPIPA7|-|	0.393	0.405
NM_0312	chr16	72119097	72172165	-	3414	1	PMFBP1|-	0.219	0.233
NM_0011	chr5	54517763	54546586	+	5215	1	SNX18|SH	2.005	2.931
NR_03392	chr1	27323874	27326525	+	2651	1	ACTG1P20	44.551	34.54
NM_0177	chr9	1.37E+08	1.37E+08	+	4065	1	TOR4A|C9	0.73	0.701
NR_12053	chr1	1.44E+08	1.44E+08	+	3038	1	SRGAP2D|	0.085	0.086
NM_0176	chr12	1.22E+08	1.23E+08	-	4239	1	ZCCHC8|-|	6.749	6.036
NR_13017	chrX	55280088	55288786	+	505	1	LOC10042	0	0
NR_12635	chr5	74369376	74536976	-	565	1	LINC01331	0.867	0.097
NM_0038	chr8	24440996	24509564	+	3372	1	ADAM7|A	0.004	0.032
NM_0327	chr1	2.47E+08	2.47E+08	-	5378	1	ZNF496|N	18.774	20.067
NR_03823	chr16	52083065	52085109	-	1655	1	LINC00919	0	0
NM_0011	chr6	87472925	87512339	+	1720	1	SLC35A1|	11.297	14.318
NM_0529	chr8	1.12E+08	1.13E+08	-	12546	1	CSMD3|-|	0.976	1.075
NM_0070	chr3	1.34E+08	1.34E+08	-	5316	1	TOPBP1|T	5.695	8.333
NR_02775	chr19	44537058	44556917	-	3008	1	CEACAM22	0.009	0
NR_02837	chr5	76712403	76712888	+	485	1	NCRUPAR|	0	0
NR_03991	chr2	19348429	19348505	+	76	1	MIR4757|	0	0
NM_0050	chr1	9651732	9729114	+	5385	1	PIK3CD|AP	12.357	12.171
NR_03398	chr11	19510893	19524078	-	1365	1	NAV2-AS4	0.057	0.07
NM_0209	chr16	3012456	3014505	+	2047	1	CLDN9|-|-	0.365	0.689
NM_0010	chr14	49625174	49635230	-	2815	1	DNAAF2|C	7.86	7.342
NR_10354	chr22	24427562	24442361	+	1942	1	ADORA2A	2.045	1.989

NM_1524(	chr17	78130778	78142968	+	4401	1	TMC8|EV2	0.214	0.134
NR_00393	chr1	1.74E+08	1.74E+08	-	80	1	SNORD79|	1.934	1.027
NM_1527:	chr7	27162438	27165530	-	2053	1	HOXA9|AE	0	0.013
NM_0011‹	chr2	1.69E+08	1.69E+08	+	1458	1	DHRS9|3-a	0.027	0.047
NM_1489:	chr6_GL0C	4239969	4243291	-	1108	8	PSMB8|AL	0	0
NM_0011:	chr19	3052910	3062373	-	1667	1	AES|AES-1	236.499	281.212
NM_0246:	chr7	1E+08	1E+08	-	2534	1	GAL3ST4|(	21.9	16.221
NM_0042:	chr15	45252230	45275934	+	2443	1	SLC28A2|(	0	0
NM_0204:	chr14_KI2	1165484	1195774	-	2937	2	DDX24|-|-	0	0
NR_13278	chr3	20174286	20186115	+	495	1	SGO1-AS1	0.886	0.664
NM_0059!	chr16	56608566	56609497	+	414	1	MT2A|MT	3.395	6.021
NM_0151!	chr6	63646526	63715522	+	7943	1	PHF3|-|-|(	2.669	5.358
NM_0008:	chr10	90740819	90857914	-	3243	1	HTR7|5-H	0.087	0.072
NM_0011:	chr3	52536605	52540570	+	445	1	SMIM4|C3	9.938	5.017
NR_13507	chr7	76092063	76108762	-	744	1	GTF2IP7|-	0	0.037
NM_0012!	chr11	2129112	2137288	-	4515	1	IGF2|C11c	1.565	0.155
NM_0133!	chr17	47733244	47746119	+	2564	1	TBX21|T-P	0.035	0.037
NM_0012:	chr5	72320367	72359353	+	2019	1	PTCD2|-|-	1.807	3.148
NM_2015:	chr11	46345267	46380554	+	3519	1	DGKZ|DAG	6.13	7.808
NR_10405	chr21	33057829	33065079	+	844	1	LINC00945	0.122	0.032
NM_2073‹	chr17	75516528	75524739	+	1928	1	TSEN54|P(	8.212	9.079
NM_1735(	chr15	42744346	42920809	-	5606	1	TTBK2|SC/	5.483	5.832
NR_03379	chr4	1.53E+08	1.53E+08	+	2999	1	DKFZP434I	3.878	5.951
NR_12068	chr10	1.13E+08	1.13E+08	+	4236	1	LOC10334‹	5.923	5.516
NR_03337	chr14	1.06E+08	1.06E+08	+	594	2	LINC0022(	0	0
NR_12064	chr10	19710335	19728550	-	1392	1	LOC10192:	0.296	0.836
NM_0209(	chr15	65381487	65423072	-	6463	1	IGDCC4|D	4.171	3.421
NM_0013:	chr19	55891695	55932336	-	3523	1	NLRP13|C	0	0
NR_11018	chr11	61588494	61607546	+	2341	1	LOC10192	0	0
NM_1783‹	chr1	1.53E+08	1.53E+08	+	622	1	LCE1A|LEF	0	0
NM_0013:	chr14	24146875	24160661	+	3189	1	RNF31|HC	14.3	14.058
NM_0010:	chr15	58879045	58933676	-	4093	1	SLTM|Met	8.669	9.855
NM_0002!	chr11	47331406	47352702	-	4181	1	MYBPC3|(	0.025	0.013
NM_0000:	chr9	1.01E+08	1.01E+08	-	2415	1	ALDOB|AL	0.037	0.199
NM_0013(	chr16	3022620	3024286	-	665	1	HCFC1R1|	98.533	99.442
NM_0204:	chr8	1.43E+08	1.43E+08	-	532	1	SLURP1|A	0	0
NM_0529:	chr19	4537215	4540024	-	1776	1	LRG1|HMI	0.152	0.177
NR_10461	chr5	4773481	4774865	+	799	1	LINC02114	0	0
NM_0011:	chr11	1.21E+08	1.21E+08	+	3892	1	TBCEL|El|I	4.131	4.681
NM_0039!	chr17	45263119	45317064	-	4468	1	MAP3K14|	3.61	3.528
NR_07346	chr14	19306690	19316914	+	722	3	BMS1P17|	0	0
NM_0239:	chrX	1.55E+08	1.55E+08	+	2957	1	FUNDC2|D	19.491	20.483
NM_2016:	chr1	77711653	77759879	-	2919	1	USP33|VD	18.817	22.838
NM_0051:	chr11	2268498	2270952	-	1845	1	ASCL2|ASI	0.112	0.111
NR_03889	chr21	40383084	40385358	+	1181	1	DSCAM-AS	0	0
NM_0008‹	chr6	52896641	52909698	-	904	1	GSTA3|GS	0	0.03
NM_0156:	chr1	25543585	25568886	+	2924	1	LDLRAP1|/	1.812	0.726
NR_14431	chr5	32354346	32444761	-	4625	1	ZFR|SPG7:	28.282	29.974
NM_0011‹	chr12	1E+08	1E+08	+	3820	1	SLC17A8|[	0.523	1.746
NM_0011‹	chr7	94656325	94669694	+	6613	1	PEG10|ED	437.394	326.674

NM_0011!	chr5	1.81E+08	1.81E+08	+	1593	1	BTNL8|BTI	0	0
NM_2136!	chr1	1.46E+08	1.46E+08	-	1465	1	HFE2|HFE:	0.018	0.019
NM_1384	chr11	63813374	63827718	+	2030	1	C11orf84|	30.284	26.497
NR_10987	chr5	1.52E+08	1.52E+08	+	400	1	LINC01933	1.257	0.753
NR_13512	chr9	1.3E+08	1.3E+08	-	3256	1	LOC40155	0.032	0.004
NM_0134	chr12	10407384	10409757	-	922	1	KLRC4|NK	0	0
NR_02729	chr22	22298098	22322968	+	2000	1	BMS1P20|	8.413	9.787
NM_1383!	chr19	14087846	14090575	-	1681	1	SAMD1|-|	65.902	58.357
NM_0010(	chr1	1.66E+08	1.66E+08	+	1118	1	LRRC52|-|	0.115	0.012
NM_0124	chr1	1.66E+08	1.66E+08	+	4911	1	UCK2|TSA	11.263	14.55
NM_0198	chrX	1.55E+08	1.55E+08	-	2597	1	F8|AHF|D	1.142	0.881
NR_00144	chr6	52995620	52995951	+	331	1	RN7SK|7SI	3.623	4.014
NR_10702	chr18	26591467	26591535	-	68	1	MIR8057|	0	0
NM_0011	chr8	23429152	23457731	-	5858	1	ENTPD4|L	18.81	16.185
NM_0328	chr10	19816443	20289855	+	12247	1	PLXDC2|TI	0.771	1.338
NR_03749	chrX	70022857	70022923	+	66	1	MIR676|h	0	0
NM_0324	chr8	42896890	43030539	+	14416	1	HOOK3|HI	2.373	2.931
NM_0145!	chr11	2404516	2423045	-	3902	1	TRPM5|LT	0.01	0.007
NM_0027(	chr4	73986439	73988190	-	1302	1	PPBP|B-TG	0	0
NM_0011	chr15	82988963	83011641	-	589	1	C15orf40|	14.229	12.023
NR_03174	chr10	1.14E+08	1.14E+08	-	74	1	MIR2110|	0	0
NM_0012	chr1	84181632	84238498	+	4227	1	PRKACB|P	11.722	13.133
NR_12051	chr7	88216660	88219224	+	572	1	LOC10272	9.918	6.801
NR_00291	chr14	21397293	21397401	-	108	1	SNORD8|F	0.239	0
NM_0011	chr1	1.54E+08	1.54E+08	+	3391	1	UBAP2L|N	86.505	103.602
NM_0068	chr17	82657067	82698722	-	1751	1	RAB40B|R	7.651	7.924
NR_12598	chr1	2.22E+08	2.22E+08	-	419	1	LINC01655	0.092	0
NM_0012	chr3	1.7E+08	1.7E+08	-	2350	1	LRRC31|HI	0	0
NM_0037	chr5	1.41E+08	1.41E+08	+	686	1	EIF4EBP3|	7.537	4.692
NM_0013	chr11	1.06E+08	1.06E+08	-	2890	1	MSANTD4	19.171	14.095
NR_10991	chr5	1887331	1900490	+	1629	1	CTD-2194I	0.016	0
NM_0011	chr17	75979217	76000586	+	991	1	TEN1|C17	8.132	6.206
NR_12042	chr13	45106279	45113206	+	1759	1	LOC10192	0	0
NR_00216	chr16	3215562	3216546	+	984	1	OR1F2P|O	0.052	0.195
NM_0181(	chr1	37692401	37709719	+	2376	1	CDCA8|BC	4.482	3.05
NR_11001	chr12	48789147	48790535	+	436	1	LOC10050	2.129	1.194
NM_0012!	chr5	1.27E+08	1.27E+08	+	7607	1	MEGF10|E	1.08	6.066
NR_03646	chr1	1.5E+08	1.5E+08	-	581	1	HIST2H2B(	0.222	0.495
NM_0209	chr7	1.39E+08	1.39E+08	-	12357	1	KIAA1549|	11.218	11.418
NR_10700	chr1	1.08E+08	1.08E+08	+	81	1	MIR7852|	0	0
NM_0010	chr12	280057	389455	-	10806	1	KDM5A|RI	7.095	6.731
NM_0133(	chr19	55647588	55653160	+	1984	1	CCDC106|	21.498	18.668
NM_0009	chr1	1.87E+08	1.87E+08	-	4481	1	PTGS2|CO	0.023	0.015
NM_0064(	chr14	24189143	24195736	-	2493	1	TM9SF1|H	17.274	13.686
NM_0048	chr8	38176588	38213301	+	4457	1	BAG4|BAG	2.676	3.092
NM_0314	chr22	41205309	41231271	+	3173	1	L3MBTL2|	17.477	14.962
NM_1784	chr20	62550453	62570764	+	1214	1	MIR1-1HG	0	0
NM_0210	chrX	1.52E+08	1.52E+08	-	1659	1	MAGEA5|	0	0
NM_0333	chr1	1.8E+08	1.8E+08	+	1871	1	LHX4|CPH	1.075	1.362
NR_00331	chr15	25051476	25051572	+	96	1	SNORD116	0	0.143

NM_0011:	chr4	1.53E+08	1.53E+08	+	6778	1	TRIM2|CM	29.439	28.259
NM_0060(	chr13	19173770	19181852	-	1544	1	TUBA3C|T	0	0.035
NM_0001!	chr9	96235307	96302152	-	1121	1	HSD17B3|	0.184	0
NM_0180!	chr3	1.73E+08	1.73E+08	+	4058	1	ECT2|ARH	1.541	2.721
NM_0305{	chr19	40875439	40882752	-	2118	1	CYP2A7|CI	0.055	0.032
NM_0330!	chr7	44962661	44979105	-	3255	1	MYO1G|H	0.067	0.059
NR_12534	chr19	52388840	52397757	-	1314	1	ZNF528-AS	4.749	5.441
NR_02938	chr14	56813183	56930832	+	2423	1	OTX2-AS1|	0.692	1.464
NM_1781;	chrX	1.13E+08	1.13E+08	-	1759	1	LHFPL1|-|-	0.103	0.117
NM_0012<	chr12	20571864	20684107	+	6542	1	PDE3A|CG	9.778	3.618
NM_0182{	chr15	75639085	75640323	-	1236	1	IMP3|BRM	35.707	41.867
NR_00280	chr4	88709789	88730103	+	3205	1	FAM13A-A	1.215	1.62
NM_0062{	chrX	1.54E+08	1.54E+08	+	717	1	SSR4|CDG	200.757	131.64
NR_01539	chr2	1.04E+08	1.05E+08	+	1501	1	LINC01102	5.042	9.426
NM_0011!	chr16	69339512	69343110	+	2172	1	NIP7|CGI-:	6.779	6.325
NM_1523(	chr14	1.04E+08	1.04E+08	+	3235	1	TRMT61A|	3.982	3.701
NM_0202(	chr17	17303366	17347663	+	1581	1	NT5M|dN	6.785	10.015
NM_0049{	chrX	1.53E+08	1.53E+08	+	1704	1	MAGEA1|(	0	0
NM_1942{	chr4	82924604	83010834	-	6116	1	LIN54|CXC	1.417	1.27
NM_0327{	chr4	1.69E+08	1.69E+08	-	3494	1	CBR4|SDR	5.284	4.975
NR_03970	chr10	12578753	12578823	+	70	1	MIR4480|-	0	0
NM_1526;	chr7	65373799	65401135	+	3214	1	ZNF92|HEI	1.055	2.246
NM_0216;	chr18	24460629	24479957	+	3663	1	HRH4|AXC	0	0.056
NR_13046	chr16	50742305	50742377	+	72	1	MIR3181|-	0	0
NM_0060(	chr4	38823708	38856817	-	5883	1	TLR6|CD2{	0.004	0.012
NM_0059<	chr16	56651899	56653204	+	375	1	MT1B|MT	0	0
NM_0011!	chr15	79843547	79897285	-	2278	1	MTHFS|Hs	2.4	1.978
NM_0056;	chr1	1.52E+08	1.52E+08	-	589	1	S100A11|I	3.13	2.488
NR_13500	chr12	1.11E+08	1.11E+08	-	596	1	LOC10536!	0	0
NR_02947	chr6	34757094	34773857	+	1169	1	SNRPC|U1	69.287	60.986
NM_0010;	chr11	1.19E+08	1.19E+08	-	484	1	RPS25|S2!	317.622	233.33
NM_0012<	chr11	1.12E+08	1.12E+08	-	1128	1	IL18|IGIF|	0	0.012
NR_03962	chr2	33418516	33418564	+	48	1	MIR4430|-	0	0
NM_0216:	chr1	45627304	45686630	-	3723	1	GPBP1L1|!	18.303	17.214
NM_0011;	chr6	16299112	16761490	-	10577	1	ATXN1|AT	2.206	2.378
NM_0014(	chr1	1.71E+08	1.71E+08	+	5293	1	FMO2|FM	0	0.005
NM_0309:	chr10	1.03E+08	1.03E+08	+	2736	1	TRIM8|GE	35.542	34.067
NM_0011!	chr8	22687661	22692580	-	3900	1	EGR3|EGR	0.063	0.681
NR_03626	chrX	1.55E+08	1.55E+08	-	98	3	MIR1184-:	0	0
NR_12034	chr15	71688701	71691539	-	2136	1	THSD4-AS:	0	0
NM_0010:	chr10	15043896	15073852	+	1665	1	OLAH|AUF	0.031	0.041
NM_0143!	chr10	90912100	90921275	-	1963	1	ANKRD1|A	0	0.035
NM_0018(	chr5	1.5E+08	1.5E+08	+	1767	1	CDX1|-|-|!	0	0
NM_1393:	chr19	8364127	8374375	+	1896	1	ANGPTL4|	1.292	0.867
NR_03980	chr8	1.23E+08	1.23E+08	-	75	1	MIR4663|-	0	0
NR_03780	chr19	52927135	52942594	-	2332	1	ZNF321P|Z	0.205	0.458
NM_2033!	chr7	1E+08	1E+08	+	1498	1	MBLAC1|-	3.09	2.688
NM_0011;	chr7	83363906	83641431	-	5974	1	SEMA3E|N	0.417	0.456
NR_03969	chr9	1.22E+08	1.22E+08	-	85	1	MIR4478|r	0	0
NM_1825:	chr12	69825323	69958725	+	3095	1	MYRFL|C1	0.017	0.018

NM_0047:	chr11	1.14E+08	1.14E+08	-	2930	1	ZW10|HZV	5.575	6.016
NR_02413	chr10	99927209	99958998	+	2646	1	DNMBP-AS	0.287	0.378
NR_03750	chrX	39661216	39661321	+	105	1	MIR3937|-	0	0
NM_0011-	chr16	27066898	27069166	+	2266	1	C16orf82|	0.165	0.048
NM_0201!	chr5	1.34E+08	1.34E+08	-	2276	1	C5orf15|H	17.295	14.082
NM_0013(	chr15	73735431	73752370	-	5591	1	C15orf59|	4.981	10.373
NR_00260	chr13	50082169	50107218	+	2902	1	DLEU1|BC	1.12	1.973
NR_03161	chr22	26555212	26555323	-	111	1	MIR548J|N	0	0
NM_1775:	chrX	52184823	52192268	+	2557	2	MAGED4B	0.03	0.043
NR_12162	chr6	1.4E+08	1.4E+08	-	573	1	LOC10335	0	0
NM_0047{	chr18	56602822	56638689	-	1314	1	TXNL1|HE	67.617	77.679
NM_0011:	chr4	1.05E+08	1.05E+08	+	9767	1	TET2|KIAA	4.426	2.648
NM_1529{	chr19	2328630	2355102	+	3735	1	SPPL2B|IM	15.386	13.297
NM_2058-	chrX	9024232	9034127	-	2092	1	FAM9B|TE	0	0
NM_2074!	chr17	82359247	82363776	+	1932	1	TEX19|-|-|	0.053	0.043
NM_0011:	chr5	1.43E+08	1.43E+08	+	8851	1	ARHGAP26	1.955	2.244
NM_0150	chr14	75661208	75955082	+	4723	1	TTLL5|COF	7.663	8.158
NM_0326:	chr4	1.39E+08	1.39E+08	-	1333	1	MGARP|C4	1.383	1.541
NR_03172	chr20	3917502	3917563	-	61	1	MIR103B2	0.845	0.674
NM_0314:	chr13	45464895	45536698	+	4571	1	COG3|SEC	7.201	8.447
NM_0331:	chr4	70334954	70337116	+	1540	1	CABS1|C4	0.008	0.018
NM_1840{	chr8	66127043	66175483	+	2464	1	TRIM55|M	0.068	0.028
NM_0012(	chr21	44768580	44801836	-	3295	1	UBE2G2|U	34.419	36.315
NR_03611	chr10	45164014	45164088	+	74	1	MIR3156-1	0	0
NM_1735!	chr9	98732009	98796542	-	7160	1	ANKS6|AN	2.822	3.897
NR_12638	chr7	20835431	21023148	+	441	1	LINC01162	0	0.031
NM_0201:	chr6	2765432	2785745	+	2643	1	WRNIP1|V	34.002	38.749
NM_0163:	chr14	1E+08	1E+08	+	1826	1	EVL|RNB6	181.102	188.803
NM_0012{	chr7	1.01E+08	1.01E+08	+	1290	1	AP1S1|AP:	100.129	97.875
NR_11038	chrX	51095836	51171400	-	733	1	LINC01284	0.106	0.075
NM_0011(	chr10	71711701	71737824	-	4735	1	C10orf105	0.286	0.364
NR_04621	chr1	71794232	71837012	-	531	1	NEGR1-IT1	0	0
NM_0012!	chr17	47117697	47189312	-	5685	1	CDC27|AN	5.153	7.187
NM_1943:	chr2	26457203	26478042	-	4725	1	OTOF|AUI	0.027	0.101
NM_0066-	chr13	26557703	26688946	+	4823	1	WASF3|Br	19.577	18.187
NM_0012!	chr16	31138926	31150094	-	2514	1	PRSS36|-|	0.231	0.207
NR_02950	chr6	71403551	71403621	-	70	1	MIR30A|M	0	0
NR_02401	chr4	68928459	68951791	-	3059	2	UGT2A3|-	0	0
NM_0043:	chr8	89901868	89927867	+	4191	1	OSGIN2|C	1.704	2.157
NM_1446{	chr17	35435096	35448837	-	8461	1	SLFN13|SL	0.052	0.01
NM_0059!	chr16	56589355	56591088	+	577	1	MT3|GIF|	2.614	2.706
NM_0530(	chr11	1469448	1480886	-	1417	1	MOB2|HC	16.806	18.723
NM_0190:	chr2	36981001	37084345	-	6766	1	HEATR5B|	9.035	9.152
NM_0026:	chr7	95583497	95596613	-	3687	1	PDK4|-|-|	0.189	0.16
NM_0010(	chr11	24496970	25082640	+	5283	1	LUZP2|KFS	1.325	0.775
NM_0011	chr9	90801927	90898560	+	4904	1	SYK|p72-S	0.008	0
NR_03157	chr14	1.01E+08	1.01E+08	+	84	1	MIR1185-2	0	0
NM_0003:	chr16	56865207	56915850	+	5539	1	SLC12A3|N	0	0.002
NM_0012{	chr14	99169288	99271713	-	7822	1	BCL11B|A	0.77	7.815
NR_11030	chr14	66486371	66498553	+	902	1	LINC00238	0.043	0.015

NM_0012!	chr22	30240447	30246851	-	3786	1	LIF|CDF|D	0.106	0.25
NR_00260	chr1	1.71E+08	1.71E+08	+	2118	1	FMO6P|FI	0	0
NM_0011	chr17	41307700	41309253	-	1552	2	KRTAP16-1	0	0
NR_13467	chr4	32351038	32353220	+	847	1	LOC10192	0	0.016
NM_1734	chr17	74916084	74923263	-	3560	1	USH1G|AI	0.065	0.115
NM_0246	chr3	1.67E+08	1.67E+08	-	3221	1	ZBBX|-|-|	0.328	0.276
NM_0012	chr11	72109587	72112298	-	772	1	ANAPC15|	47.031	38.838
NM_0010	chr19	8730954	8732009	+	1053	1	OR2Z1|OR	0	0
NM_0329	chr16	56565049	56568957	+	289	1	MT4|MT-	0	0
NM_0034	chrX	1.38E+08	1.38E+08	+	3934	1	ZIC3|HTX|	15.653	5.149
NR_03338	chr10	1526630	1556984	+	642	1	ADARB2-A	0	0.085
NM_0025	chr6	41072968	41102407	+	6207	1	NFYA|CBF	14.539	17.402
NM_0148	chr9	37438103	37465410	-	4645	1	ZBTB5|-|-	26.375	30.163
NM_0570	chr12	52789685	52796108	-	2299	1	KRT3|CK3	0	0
NM_0013	chr1	1.58E+08	1.58E+08	+	1579	1	CD1A|CD1	0	0
NM_0044	chrX	54445454	54496166	-	4255	1	FGD1|AAS	22.739	25.878
NM_0155	chr18	44680898	45068510	+	9891	1	SETBP1|M	9.18	12.254
NR_10673	chr15	62343029	62343138	+	109	1	MIR6085|	0	0
NM_0314	chr11	65916812	65919060	-	1551	1	C11orf68|	32.545	30.812
NM_0010	chr6	1.55E+08	1.55E+08	+	1205	1	CLDN20|-|	0.021	0.148
NM_0175	chr6	1.57E+08	1.57E+08	+	9578	1	ARID1B|6/	12.772	12.171
NR_04552	chr15	82293283	82334561	+	4959	2	ADAMTS7I	0.003	0
NR_02848	chr22	24429206	24495074	-	2824	1	ADORA2A-	0.269	0.213
NM_0011	chr3	49108046	49120938	-	4775	1	USP19|ZN	28.069	29.565
NM_1779	chr20	9537390	9839039	-	4659	1	PAK5|PAK	9.285	7.641
NM_0187	chr1	1.12E+08	1.12E+08	+	5892	1	CTTNBP2N	11.303	11.33
NM_1788	chr16	88696495	88706421	-	1875	1	RNF166|-|	10.191	12.923
NR_11193	chr1	1.5E+08	1.5E+08	+	2305	2	LOC38869	0	0
NR_02937	chr14	69766283	69767713	-	1430	1	LOC10028	3.507	2.73
NR_03741	chr9	1.37E+08	1.37E+08	-	84	1	MIR3621|	0.921	0
NM_0306	chr16	81495349	81711763	+	4109	1	CMIP|TCN	90.286	73.615
NM_0011	chr5	1.23E+08	1.23E+08	-	4608	1	CEP120|C	4.382	5.089
NR_03650	chr10	15154722	15155347	-	625	1	PPIAP30|-	0	0.066
NM_0010	chr11	5754693	5755729	+	1034	1	OR52N4|C	0	0
NM_0011	chr1	1.82E+08	1.83E+08	+	3737	1	RGSL1|RG	0	0.004
NM_0334	chr4	1.19E+08	1.2E+08	-	6749	1	PDE5A|CG	0.984	0.812
NR_00352	chr9	39443815	39464526	-	4023	1	ZNF658B|-	0.08	0.099
NM_0010	chr1	2.07E+08	2.07E+08	+	942	1	C4BPB|C4	0	0
NM_0061	chr4	1.63E+08	1.63E+08	+	1929	1	NPY5R|NP	0.608	0.334
NM_0018	chr2	97646058	97648194	+	499	1	COX5B|CC	223.091	166.039
NM_0010	chr5	1.1E+08	1.11E+08	-	3177	1	TMEM232	0.503	0.487
NM_0012	chr2	84821667	84881245	-	1965	1	TRABD2A|	0.112	0.314
NM_0011	chr10	1.24E+08	1.24E+08	-	967	1	NKX1-2|C1	0.213	0.099
NM_0324	chr10	59176588	59247774	+	3573	1	PHYHIPL|-	16.047	21.793
NM_0043	chr19	19211973	19252252	+	6389	1	NCAN|CSP	126.204	215.248
NM_0010	chr1	2.48E+08	2.48E+08	+	1006	1	OR2AK2|C	0	0
NM_0012	chr22	45344064	45413619	-	4005	1	SMC1B|SN	0.035	0.048
NR_03375	chr3	1.86E+08	1.86E+08	+	2160	1	NMRAL2P	0.024	0.006
NM_0011	chr10	49131156	49134020	-	1407	1	FAM170B|	0	0
NM_0065	chr17	65009289	65056802	-	6305	1	GNA13|G1	8.808	8.544

NM_0011!	chr1	1.51E+08	1.51E+08	-	1861	1	HORMAD1	0	0.007
NM_1304	chr11	47269648	47330031	+	5606	1	MADD|DE	46.141	53.996
NM_0013	chr22	37125840	37175056	-	4032	1	IL2RB|CD1	0.019	0.031
NM_0012	chrX	16167481	16171393	+	1360	1	MAGEB17	0.095	0.05
NM_1738	chr15	55611541	55743119	-	12090	1	PRTG|IGD	0.63	0.076
NM_0010!	chr19	22391097	22422346	-	2254	1	ZNF98|F7	0.011	0.006
NM_0043	chr19	17402939	17405648	-	1012	1	BST2|CD3	0.28	0.826
NR_10693	chr9	1.33E+08	1.33E+08	+	63	1	MIR6877|	8.391	6.305
NM_0528!	chr8	55102457	55526151	+	3904	1	XKR4|XRG	1.863	1.133
NM_0011	chr2	2.42E+08	2.42E+08	-	778	1	THAP4|CG	31.836	29.595
NM_1524	chr19	57905774	57916610	-	4698	1	ZNF417|-|	1.913	2.388
NM_0011	chrX	1.55E+08	1.55E+08	-	1754	1	FAM3A|2.	24.162	19.007
NM_0010	chr1	2.49E+08	2.49E+08	-	955	1	OR2T34|-|	0	0
NM_0006	chr4	99204722	99219246	-	1390	1	ADH6|ADH	0.362	0.315
NM_0012	chr4	85827893	86002670	+	4000	1	ARHGAP24	2.543	2.551
NR_02665	chr12	54102209	54102699	+	490	1	LOC10024	0	0
NM_0241	chr9	1.28E+08	1.28E+08	+	709	1	C9orf16|E	103.712	107.219
NM_0011	chr17	39156894	39166161	-	933	1	ARL5C|AR	0	0.132
NR_13569	chr15	77525595	77534110	+	3757	1	LOC10192	0.058	0.069
NR_04977	chr3	31704213	31721586	+	539	1	OSBPL10-A	0	0
NM_0013	chr14	92576234	92688994	+	3588	1	RIN3|-|-|1	0.133	0.286
NM_0012	chr7	75533298	75738972	-	7856	1	HIP1|HIP-I	23.429	26.487
NM_0053	chr10	1.19E+08	1.19E+08	+	2540	1	GRK5|GPR	18.685	23.29
NM_0046	chr7	73699205	73719687	-	2113	1	STX1A|HP	32.847	40.132
NM_0012	chr10	1.01E+08	1.01E+08	-	643	1	NDUFB8|A	246.199	165.216
NR_10671	chr3	1.85E+08	1.85E+08	-	62	1	MIR5588|	0	0
NM_0007	chr8	42697419	42737407	+	2286	1	CHRNB3|-	0.953	0.258
NM_0010	chr6	30571393	30591532	+	3436.4	7	ABCF1|AB	0	0
NR_00272	chr22	29437015	29442129	-	5114	1	RFPL1S|N	2.108	7.384
NM_0177	chr11	94493987	94499578	+	1900	1	ANKRD49|	2.606	3.093
NM_0011	chr16	66961229	66975149	+	3871	1	CES3|ES31	1.456	3.45
NM_1392	chr19	35124513	35135274	-	2877	1	LGI4|LGIL	1.528	1.438
NM_0056	chr8	42391761	42405937	+	1525	1	VDAC3|HD	150.3	151.925
NM_0013	chr17	7856681	7885388	-	733	1	NAA38|LS	41.882	30.047
NM_0012	chr1	42456494	42461230	+	1817	1	PPCS|-|-|	10.694	10.162
NM_0040	chr8	6977649	6980092	-	493	2	DEFA1|DE	0	0
NR_00293	chr14	34939922	34940496	+	574	1	IGBP1P1|	2.74	4.033
NM_0011	chr7	1.02E+08	1.02E+08	+	10782	1	ORAI2|C7	11.064	7.75
NR_00308	chr22	23998401	24005361	-	717	2	GSTTP1|G	0	0
NM_0244	chr8	1.03E+08	1.03E+08	+	990	1	ODF1|CT1	0.026	0.014
NM_0530	chr10	5365009	5374692	+	1196	1	UCN3|SCP	0.248	0
NM_0012	chr10	1.17E+08	1.17E+08	-	2170	1	SHTN1|KI	5.146	14.069
NM_0159	chr16	67389807	67393535	-	1054	1	TPPP3|CG	59.403	10.162
NR_03885	chr2	1.58E+08	1.58E+08	+	790	1	CCDC148-A	0.196	0
NM_1449	chr12	31382223	31591018	-	9368	1	DENND5B	8.497	11.603
NM_0320	chr3	1.51E+08	1.51E+08	+	3972	1	EIF2A|CDA	10.67	9.804
NM_0010	chr11	56576774	56577721	-	946	1	OR5M10|	0	0
NM_0020!	chr4	74036591	74038773	-	1151	1	CXCL3|CIN	0.179	0.095
NM_0013	chr17	44395277	44503602	-	6948	1	GPATCH8|	7.792	12.853
NR_04569	chr3	64067964	64103131	+	6502	1	PRICKLE2-A	3.942	3.202

NR_04627	chr17	63700338	63702685	+	2347	1	LOC72968	7.471	4.966
NR_00256	chr11	62855292	62855366	-	74	1	SNORD26|	4.53	6.663
NM_0013(	chr15	50557159	50686815	-	10355	1	TRPM7|AL	1.006	1.974
NM_0140(	chr6_GL0C	2396864	2398680	-	1147.8	6	PSORS1C2	0	0
NM_0011!	chr1	6046114	6101193	+	3658	1	KCNAB2|A	9.732	13.12
NM_0177!	chr17	18183553	18209953	+	3427	1	ALKBH5|A	46.784	43.465
NM_0332	chr4	79406353	79408218	-	1863	1	GK2|GKP2	0.007	0.103
NM_0016!	chr4	74445098	74455009	+	1268	1	AREG|AR|	0	0.032
NM_0010	chr14	77377419	77391244	+	2021	1	SAMD15|(	0.995	0.529
NM_0021	chr7	27128507	27130780	-	1724	1	HOXA4|H(	0.037	0.119
NM_0030	chrX	81201804	81298547	+	2056	1	SH3BGRL|	11.257	16.169
NM_1984!	chr11	1.11E+08	1.11E+08	+	1202	1	C11orf53|	0	0
NR_03662	chr17	44708608	44750549	+	3386	1	DBF4B|AS	3.503	2.573
NM_0012	chr11	1.19E+08	1.19E+08	-	2904	1	USP2|UBP	1.416	1.693
NM_0120	chr1	2.3E+08	2.3E+08	-	3842	1	ABCB10|E	2.272	2.061
NR_13077	chr5_GL94	115886	145361	+	2050	2	LINC0210!	0	0
NM_0011	chr19	29526584	29564319	+	1236	1	VSTM2B|-	1.972	0.654
NM_0011(	chr9	1.13E+08	1.13E+08	-	2372	1	ZNF883|-|	1.522	3.211
NM_0011	chrX	1.31E+08	1.31E+08	-	4536	1	IGSF1|CHT	2.709	4.016
NM_0010!	chr9	69709522	69759960	-	10041	1	PTAR1|-|-	2.081	4.585
NM_0152(	chr16	27550147	27780371	+	6593	1	KIAA0556|	6.725	6.322
NR_03165	chr17	2748078	2748182	-	104	1	MIR1253|	0	0
NM_0013	chr1	43969981	43973371	+	2266	1	DPH2|DPH	9.855	9.611
NM_0012	chr4	9325165	9326757	+	1591	8	USP17L28	0	0
NM_0027	chr7	1.07E+08	1.07E+08	+	3648	1	PRKAR2B|	26.254	24.739
NM_1985	chr2	2.38E+08	2.38E+08	+	3675	1	KLHL30|-|	0.032	0.011
NR_10808	chr7	35716437	35719712	+	1073	1	LOC10050	0.12	0.128
NR_04004	chr20	1325343	1378735	+	2223	1	SDCBP2-AS	2.529	2.224
NR_10423	chr22	40769630	40819399	-	2161	1	SLC25A17|	23.997	26.437
NR_13079	chr13	67372387	67379994	+	626	1	LINC00364	0	0
NM_0013	chr4	17800655	17810758	-	4425	1	DCAF16|C	9.208	12.468
NR_13505	chr11	13921447	13924863	-	685	1	LOC10192	0	0.04
NM_0013(	chr3	52198083	52214327	+	2251	1	ALAS1|AL	33.989	44.736
NM_0011	chr7	1709162	1747954	+	3738	1	ELFN1|PP	17.202	8.259
NM_0011	chr21	30642864	30643136	+	270	1	KRTAP20-3	0	0
NM_0010	chr10	11460510	11532280	-	4501	1	USP6NL|R	3.853	4.233
NM_0328	chr12	53252092	53254406	+	1762	1	MFSD5|hs	11.671	13.067
NM_0072	chrX	1.16E+08	1.16E+08	+	4547	1	SLC6A14|E	0	0
NM_1532	chr1	44783585	44787754	-	2085	1	BEST4|VM	0.111	0.105
NR_03963	chr2	64340759	64340839	+	80	1	MIR4433A	0	0
NR_12583	chr6	49811540	49820503	+	498	1	LOC10192	0.052	0
NM_0010(	chr11	58118500	58119444	-	943	1	OR9I1|OR	0	0
NM_0034	chrX	47370600	47413699	+	1689	1	ZNF157|H	1.359	1.395
NM_0012(	chr17	799313	864111	-	2671	1	NXN|NRX|	65.665	46.588
NM_0010(	chr11	6026671	6027741	-	1068	1	OR56A1|C	0	0.013
NM_0183	chr8	6708357	6761500	+	5509	1	AGPAT5|1	6.787	9.749
NM_0047	chr9	1.24E+08	1.24E+08	+	2409	1	LHX2|LH2	19.386	63.521
NM_0010	chr5	1.57E+08	1.57E+08	+	6466	1	CYFIP2|PI	67.397	90.467
NR_00322	chr16	89972580	89996620	+	2919	1	AFG3L1P|	5.733	4.758
NR_10679	chr1	2.02E+08	2.02E+08	+	112	1	MIR6740|	2.763	3.057

NM_0045:	chr1	1.66E+08	1.66E+08	+	1510	1	MGST3|GS	70.76	61.501
NR_03162	chr20	35453954	35454097	-	143	1	MIR1289-1	0.361	0
NR_02657	chr4	1.65E+08	1.65E+08	-	2231	1	GK3P|GKP	0.064	0.043
NR_03607	chr2	1.89E+08	1.89E+08	-	75	1	MIR3129|(	0	0
NM_0012!	chr17	37978155	37989048	-	2017.2	17	TBC1D3I|-	0.102	0.19
NM_0144	chr1	3454583	3481113	+	2887	1	ARHGEF16	1.192	0.949
NR_13808	chr6_GL0C	4554809	4560318	+	1962.2	5	HCG24|-|-	0	0.014
NM_0010(	chr16	20333051	20352878	-	2402	1	UMOD|AD	0	0
NR_03658	chr2	64901840	64932447	-	3026	1	LOC40095	0.009	0.014
NR_03764	chr11	1.26E+08	1.26E+08	+	1840	1	FOXRED1|	22.164	16.704
NM_0012	chr2	1.31E+08	1.31E+08	-	1441	1	CFC1|CFC1	0.027	0.285
NM_0010	chr22_KI2	216514	220281	-	1087	4	GSTT2B|G	0	0.013
NR_03766	chr6	1.19E+08	1.19E+08	+	710	1	LOC10028	0.036	0
NM_1781	chr7	1.01E+08	1.01E+08	-	1399	1	MOGAT3|	0	0
NR_02963	chrX	45747015	45747124	-	109	1	MIR222|M	0	0
NM_0068!	chr2	2.08E+08	2.08E+08	-	702	1	CRYGD|CA	0	0
NR_10989	chr5	1.6E+08	1.6E+08	-	5601	1	LINC01847	0	0
NM_1985(	chr5	1.01E+08	1.01E+08	+	1350	1	FAM174A|	5.539	5.54
NM_0029!	chr20	37179046	37241622	+	2495	1	RPN2|RIBI	128.465	144.87
NM_0160(	chr17	51260536	51297931	+	1868	1	UTP18|CG	13.984	15.501
NM_0011!	chrX	1.19E+08	1.19E+08	+	1345	1	SLC25A5|2	119.243	135.87
NM_0016(	chr7	1.28E+08	1.28E+08	+	1088	1	ARF5|-|-|1	177.179	177.948
NM_0046!	chrY	19705415	19744939	-	5459	1	KDM5D|H	7.622	8.335
NM_0144	chr4	1.07E+08	1.07E+08	-	3631	1	DKK2|DKK	0.501	0.045
NM_0328	chr17	39252644	39401656	-	10364	1	FBXL20|Fb	3.959	4.828
NR_03623	chr2	27996367	27996449	+	82	1	MIR4263|-	0	0.668
NM_1816	chr20	37521206	37523688	+	1216	1	NNAT|Peg	1.421	419.077
NM_0011!	chr1	51354263	51422121	-	4336	1	EPS15|AF-	16.01	18.985
NR_13615	chr2	949635	951392	-	748	1	LOC10106	0.655	0.439
NM_0012!	chr4	9253378	9254970	+	1591	1	USP17L19	0	0
NM_0011!	chr16	2475146	2505733	+	6585	1	TBC1D24|	7.715	8.37
NM_0010:	chr11	64300391	64304767	+	737	1	TEX40|C11	0.77	0.372
NM_0043(	chr6	1.67E+08	1.67E+08	+	3211	1	CCR6|BN-1	0.145	0.162
NM_0145	chr16	981808	986979	+	3044	1	SOX8|-|-|1	4.414	10.705
NR_00273	chr8	66767334	66768034	-	700	1	PTTG3P|P	4.752	4.637
NR_10990	chr5	58541567	58558243	-	1180	1	LINC02108	0	0
NM_1989	chr11	68980021	68980986	-	964	1	MRGPRD|	0.027	0
NR_02680	chr14	1.06E+08	1.06E+08	+	9606	2	FAM30A|(	0	0
NR_11085	chr6	74069451	74690727	+	665	1	LOC10192	0	0.144
NR_00155	chrY	7804924	7810682	+	976	1	TTTY12|N(	0	0
NR_03845	chr10	71217224	71218228	-	645	1	UNC5B-AS	0.58	0.828
NR_02958	chr19	10817426	10817496	-	70	1	MIR199A1	0	0.196
NM_0252:	chr17	43753731	43758788	-	2296	1	SOST|CDD	0.039	0.03
NR_03617	chr1	10227718	10227803	+	85	1	MIR1273D	0	0
NM_1733!	chr3	71679289	71754773	-	6160	1	EIF4E3|eIF	3.594	5.47
NM_0011!	chr1	1.61E+08	1.61E+08	-	2334	1	B4GALT3|I	26.986	32.752
NR_03379	chr17_GL0	328268	378560	+	580	5	NSFP1|NSI	0	0
NR_04024	chr13	1.05E+08	1.06E+08	-	2474	1	DAOA-AS1	0	0
NM_0046	chr19	35825372	35851993	-	4993	1	NPHS1|CN	0.826	0.804
NR_00000	chr11	62852910	62853035	-	125	1	SNORD22|	8.664	7.999

NR_03992	chr22	46113566	46113657	+	91	1	MIR4763|·	0.142	0.452
NR_03355	chr6	1.07E+08	1.07E+08	-	1380	1	LOC10042	0	0
NM_0013	chr2	24049701	24063680	+	845	1	FKBP1B|FK	53.908	54.107
NR_12212	chr14	96501648	96502326	-	678	1	LOC73020	1.141	2.162
NM_0318	chr10	35638249	35641434	-	3183	1	FZD8|FZ-8	2.268	3.279
NR_03964	chr3	1.17E+08	1.17E+08	-	90	1	MIR4447|·	0	0
NM_0007	chr12	57762334	57767193	-	2476	1	CYP27B1|	0.359	0.481
NR_13554	chr3	1.02E+08	1.02E+08	+	467	1	LOC10192	0	0
NM_0018	chr22	38290691	38318084	-	2629	1	CSNK1E|C	198.243	221.174
NM_0333	chr11	93661650	93730356	+	8012	1	CEP295|KI	1.03	1.71
NM_0059	chr8	88037232	88327488	-	6323	1	MMP16|C	1.277	1.726
NM_0011	chr14	23058565	23071570	-	2442	1	ACIN1|ACI	75.993	70.852
NR_03040	chr14	1.01E+08	1.01E+08	+	87	1	MIR758|M	0	0
NM_0039	chr6	28526012	28534951	+	1328	2	GPX5|HEL	0	0
NM_0011	chr5	1.6E+08	1.6E+08	-	3363	1	PWWP2A|	4.225	3.584
NM_0057	chr9	4792834	4861077	+	2171	1	RCL1|RNA	3.581	6.454
NR_04973	chr17	44175974	44186717	-	917	1	ASB16-AS1	6.355	5.228
NR_13450	chr10	84167228	84172076	-	1855	1	HOST2|lnc	0.014	0.007
NM_2033	chrX	74304190	74305034	+	842	1	ZCCHC13|	0	0
NM_0055	chr7	27092995	27096006	-	2543	1	HOXA1|BS	0	0
NR_13235	chr15	45587772	45609716	+	3511	1	BLOC1S6|	16.86	15.991
NM_0012	chr9	24543215	24545676	-	735	1	IZUMO3|C	0	0
NM_2074	chr1	17372196	17401699	+	2378	1	PADI6|PRE	0.033	0
NR_01538	chr7	87325348	87345492	-	706	1	TP53TG1|	5.808	7.819
NR_02449	chr22	24632915	24653360	+	1418	1	BCRP3|BC	0.255	0.155
NM_0251	chr12	1.21E+08	1.21E+08	+	2017	1	RNF34|CA	27.596	27.856
NM_0011	chr3	52833115	52897581	-	1447	1	TMEM110	11.869	7.128
NR_00282	chr19	49969600	49973531	+	1579	1	SIGLEC16|	0.016	0
NR_02445	chr7	1.4E+08	1.4E+08	+	2379	1	JHDM1D-A	1.973	1.606
NM_0011	chr16	84191117	84197166	+	2046	1	ADAD2|TE	0	0
NR_00155	chrY	9349580	9355182	-	463	5	FAM197Y2	0	0
NR_00218	chrY	22851584	22852715	+	164	3	TTTY17B|	0	0
NM_0034	chr1	37556919	37566857	+	2641	1	DNALI1|P2	19.616	10.476
NM_0011	chr21	39387766	39397889	+	1456	1	WRB|CHD	74.474	74.224
NM_0806	chr20	45886491	45887599	-	715	1	SPATA25|	1.623	1.283
NM_2124	chr11	68052859	68121391	-	2650	1	CHKA|CHK	18.469	24.091
NM_1383	chr2	1.98E+08	1.98E+08	+	3084	1	MARS2|CC	3.014	3.891
NR_04009	chr8	12437013	12566845	+	2290	1	LOC10050	7.032	9.235
NM_0010	chr11	6921002	6921994	+	991	1	OR2D3|OF	0	0
NM_0011	chr2	74361154	74374735	-	4120	1	DCTN1|DA	153.223	130.354
NM_0011	chr19	4838334	4867768	-	2327	1	PLIN3|M6	23.382	29.76
NM_1531	chr3	97941817	97972451	-	4863	1	RIOX2|JM	2.916	1.839
NM_2073	chr4	1.86E+08	1.86E+08	+	4691	1	CYP4V2|B	0.913	0.794
NM_0018	chr1	31652247	31704167	-	5511	1	COL16A1|	0.18	0.659
NM_0183	chr16	12659799	12803887	-	6137	1	CPPED1|C	1.546	1.178
NR_11021	chr2	59217708	59279400	+	2033	1	LINC01793	0	0.027
NR_04598	chr17	6657719	6661382	+	667	1	ALOX15P1	0.425	0.205
NM_0068	chr19	48382570	48391553	-	1550	1	KDELR1|E	185.774	140.169
NR_04581	chr16	10386055	10483638	+	3423	1	ATF7IP2|M	0.241	0.26
NM_0010	chr10	56357438	56361273	-	1508	1	ZWINT|HZ	11.944	7.348

NM_1775:	chr2	2.03E+08	2.03E+08	+	10928	1	CYP20A1|(	1.483	1.246
NM_0208;	chr4	1.69E+08	1.69E+08	-	5251	1	SH3RF1|P(	16.562	10.41
NM_0011!	chr14	75605462	75648169	+	2760	1	FLVCR2|C:	0.64	0.342
NR_13620	chr5	25190953	25302280	+	323	1	LOC10537	0	0.042
NR_03844	chr2	39917634	40255209	+	997	1	SLC8A1-AS	0.103	0.096
NM_0011!	chr9	32540544	32552628	-	3974	1	TOPORS|L	4.043	6.283
NM_1784;	chrX	1.27E+08	1.27E+08	-	3386	1	DCAF12L1	0	0
NR_11020	chr2	47192405	47345074	-	918	1	LOC10192	2.598	4.282
NM_0010!	chr16	28605587	28610044	-	1230	1	SULT1A1|I	0.178	0.022
NM_0010;	chr4	55853650	55905078	+	3561	1	EXOC1|BN	10.214	10.805
NM_0012{	chr20	841275	846279	+	1610	1	FAM110A|	23.432	22.162
NM_0189:	chr5	1.41E+08	1.42E+08	+	4811	1	PCDHGA2|	19.25	77.479
NR_03982	chr10	89063335	89063411	-	76	1	MIR4679-2	0	0
NM_0011:	chr3	63967744	64003460	+	6394	1	ATXN7|AD	2.087	2.62
NM_0013:	chr17	39921041	39926846	-	2017	1	ORMDL3|-	36.839	44.738
NM_0011(	chr3	1.91E+08	1.91E+08	+	2897	1	IL1RAP|C3	5.772	1.433
NM_0249	chr10	1.23E+08	1.23E+08	-	3079	1	C10orf88|	3.953	3.559
NM_1983:	chr5	1.54E+08	1.54E+08	+	5941	1	GALNT10|	10.189	13.57
NM_0035	chr6	27831174	27831527	-	352	1	HIST1H4K|	0.293	0.233
NM_0013:	chrX	40626921	40647683	-	2815	1	CXorf38|-|	4.924	4.034
NM_0570!	chr11	64242116	64244135	+	629	1	FKBP2|FKE	63.217	40.438
NM_0013!	chr8	29333062	29350750	-	5615	1	DUSP4|HV	75.939	32.922
NR_13368	chr5	1.36E+08	1.36E+08	+	761	1	TRPC7-AS2	0	0
NM_0060{	chr16	89923279	89936097	+	1763	1	TUBB3|CD	1686.888	1289.811
NR_13457	chr20	53608354	53634590	+	378	1	LOC10537	0	0.072
NR_03884	chr15	23354748	23368324	+	5436	1	GOLGA8S|	0	0
NM_0033;	chr10	73998114	74120156	+	5259	1	VCL|CMD1	16.247	14.861
NM_0010!	chr8	1.1E+08	1.1E+08	-	2672	1	SYBU|GOL	36.046	49.565
NM_1534	chrX	1.04E+08	1.04E+08	-	1474	1	ESX1|ESX1	0.026	0
NM_0186(	chr8	28345585	28386460	-	4790	1	ZNF395|H	10.032	10.025
NR_00302	chr7	1.1E+08	1.1E+08	-	986	1	EIF3IP1|R(	9.376	9.391
NM_0041(	chr2	65086854	65130301	-	2635	1	RAB1A|RA	53.203	55.573
NM_0011(	chr12	50953999	50970506	+	290	1	HIGD1C|G	0	0
NM_0010:	chr17	40122537	40136791	+	3807	1	MSL1|MSL	48.533	49.506
NR_13447	chr10	74912786	74936402	+	2304	1	LOC10192	0	0.006
NM_0012	chr16	32253324	32255928	+	1426	1	TP53TG3D	0	0
NM_0012{	chr15	82543201	82571823	-	2838	2	CPEB1|CPI	0.109	0.121
NM_0011:	chr15	74130249	74136802	+	4255	1	ISLR2|LIN)	12.857	13.626
NM_1522!	chr4	70472117	70482997	+	2438	1	MUC7|MG	0	0
NM_0071:	chr1	1.52E+08	1.52E+08	-	6990	1	TCHH|THH	0.007	0.002
NM_1981{	chr9	1.16E+08	1.17E+08	-	1799	1	ASTN2|bA	7.267	9.403
NR_13463	chr22	43212674	43213661	+	529	1	LOC10537	0.073	0
NM_0047(	chr16	30887795	30894302	-	1074	1	BCL7C|-|-	32.138	17.995
NM_0011:	chr9	61190036	61196283	+	4220	2	SPATA31A	0	0
NM_0155!	chr3	33150045	33219215	-	4998	1	SUSD5|-|-	0.24	0.293
NM_1333:	chr4	1892782	1982207	+	7511	1	NSD2|KMT	36.299	36.997
NM_0183(	chr4	6716115	6717660	+	1543	1	BLOC1S4|I	15.969	14.283
NM_0010(	chr9	84669680	84815708	+	7129	1	NTRK2|GP	13.526	20.299
NM_0248(	chr11	1.23E+08	1.23E+08	+	3137	1	C11orf63|	1.151	0.637
NR_03030	chr4	1.09E+08	1.09E+08	+	97	1	MIR576|M	0	0

NR_03168	chr3_KI27(	130710	130858	-	121	3	MIR548I1|	0	0
NM_0013:	chr1	46798998	46819349	+	1956	1	CYP4B1|C\	0	0
NM_0013:	chr20	3208868	3223870	+	1083	1	ITPA|C20c	25.739	20.678
NM_0013:	chr16	71282300	71289715	-	3724	1	CMTR2|AF	3.199	4.417
NM_0011:	chr3	1.39E+08	1.39E+08	-	2788	1	PRR23C|-|	0	0
NM_0011:	chr19	47968046	47992170	-	646	1	BSPH1|BSI	0	0
NM_0026!	chr11	62761539	62766715	+	827	1	POLR2G|R	151.339	119.613
NR_13453	chr7	1.01E+08	1.01E+08	-	1534	1	ACTL6B|A(	101.425	102.862
NR_10995	chr20	50310711	50321342	-	2247	1	LINC01271	0	0.104
NM_0012:	chr11	1.05E+08	1.05E+08	-	1308	1	CASP4|ICE	0	0
NM_0032:	chr21	44012325	44106551	+	6944	1	TRAPPC10	6.306	6.247
NR_03891	chr22	48538900	48547387	-	1403	1	LOC28493:	0	0.01
NM_0010:	chr17	3636468	3663103	+	4027	1	CTNS|CTN	6.948	6.633
NR_03996	chr4	1.59E+08	1.59E+08	+	86	1	MIR3688-2	0	0
NR_10500	chr8	58255771	58272101	-	1682	1	LOC10192!	0	0.008
NM_0010(	chr14	19975519	19976565	+	1045	1	OR4K15|O	0	0
NR_13581	chr13	1.12E+08	1.12E+08	+	372	1	LOC10537(	0	0
NR_05198	chr4	1.38E+08	1.38E+08	+	538	1	LINC0049!	0	0
NM_0057:	chr2	2.34E+08	2.34E+08	-	4006	1	ARL4C|AR	20.705	32.054
NR_04624	chr16	66408524	66410900	+	910	1	LINC0092C	0.057	0.196
NM_0066{	chr7	77193371	77199833	-	4264	1	FGL2|T49|	0.015	0.039
NM_0069:	chr22	31081296	31104624	+	3131	1	SMTN|-|-|	4.736	5.333
NR_03749	chr9	1.1E+08	1.1E+08	-	70	1	MIR3927|-	0	0
NM_0037!	chr8	1.17E+08	1.17E+08	-	1265	1	EIF3H|EIF3	167.636	164.613
NR_13300	chr11	1.21E+08	1.21E+08	-	414	1	LOC10536!	0.467	1.257
NM_0012<	chr16	89195761	89200121	-	1850	1	SLC22A31|	0.355	0.518
NR_02970	chr1	71067631	71067716	-	85	1	MIR186|M	0.607	1.128
NM_0019:	chr12	1.09E+08	1.09E+08	+	1583	1	DAO|DAA(	0.057	0.035
NM_0331:	chr6	1.1E+08	1.1E+08	-	1974	1	SLC22A16|	0	0
NR_02807	chr17	1633858	1645789	-	3404	2	SCARF1|SF	0	0
NM_0325:	chr9	1.32E+08	1.32E+08	+	3081	1	NTNG2|LH	16.153	9.932
NR_10986	chr5	44744328	44808793	-	1391	1	BRCAT54|-	0.528	0.266
NM_0011{	chr6	33572546	33580293	-	2185	1	BAK1|BAK	3.788	3.385
NR_10345	chr9	1.35E+08	1.35E+08	-	863	1	LOC10144:	0.359	0.873
NR_04621	chr1	2.04E+08	2.04E+08	+	1274	1	LINC0026C	1.397	1.161
NR_04662	chr4	53500077	53523276	+	549	1	LNX1-AS1|	0	0
NM_0020:	chr13	40555664	40666597	-	5733	1	FOXO1|FK	0.776	0.729
NR_02740	chrX	1.55E+08	1.55E+08	+	709	2	FAM223A|	0	0
NM_0139:	chr19	15741393	15742343	+	949	1	OR10H3|-	0	0
NM_0050!	chr11	47437757	47449178	-	1661	1	RAPSN|CN	0.008	0.041
NM_0011:	chr9	83739421	83817837	-	1706	1	GKAP1|FK:	3.809	4.376
NM_0045!	chr22	41833079	41907308	+	5245	1	SREBF2|SF	155.184	174.504
NM_0013:	chr19	17527238	17552026	+	2077	1	FAM129C|	0.49	0.501
NM_0013:	chr16	30896607	30903560	+	1659	1	CTF1|CT-1	1.321	1.527
NR_10464	chr12	1.28E+08	1.28E+08	-	691	1	LOC10099(	0	0
NM_0013:	chr1	44423824	44651724	+	2403	1	RNF220|C	44.491	48.427
NM_0011:	chr8	56440954	56446734	-	1341	1	PENK|PE|I	6.067	1.757
NR_00332	chr15	25079057	25079150	+	93	1	SNORD116	0.277	0.442
NM_0245<	chr1	20499448	20508181	-	2452	1	MUL1|C1c	22.984	27.495
NM_0011:	chr19	35497217	35510489	-	940	1	DMKN|UN	0.302	0.321

NM_0191:	chr6	28241705	28252296	-	5358	1	ZKSCAN4|I	2.604	3.068
NR_03017	chr19	13874875	13875011	+	136	1	MIR181D|	0.379	0.604
NM_0011(	chr17	5032602	5035432	-	2408	1	SLC52A1|(	0.021	0
NM_0054	chr17	43025241	43032041	+	4145	1	RND2|ARH	46.662	20.947
NM_1392	chr19	50976479	50983815	-	1913	1	KLK7|PRSS	0	0
NR_00154	chrY	8704472	8705283	+	267	1	TTTY19|N(	0	0
NM_0010	chr2	1.7E+08	1.7E+08	-	2158	1	CCDC173|	0.161	0.14
NM_0057	chr22	38778508	38794156	-	1481	1	DNAL4|MI	35.059	26.201
NM_0010(	chr17	76470893	76487909	-	3423	1	RHBDF2|R	0.475	0.404
NR_10271	chr3	1.95E+08	1.95E+08	+	833	1	XXYLT1-AS	0	0.214
NM_0322	chr16	74999017	75110994	+	4626	1	ZNRF1|NIN	21.406	27.951
NR_04637	chr3	1.49E+08	1.49E+08	-	3611	1	CP|CP-2|-	0.682	0.41
NM_1750	chr7	27830574	28180818	-	3192	1	JAZF1|TIP2	5.493	6.664
NM_0001	chr16	89737551	89816657	-	5415	1	FANCA|FA	5.038	5.256
NM_0012	chr14	30622254	30735827	+	1906	1	SCFD1|C14	9.816	10.449
NM_0020	chr13	27424544	27435709	+	1393	1	GTF3A|AP	53.027	57.876
NM_0011	chr12	1.33E+08	1.33E+08	+	5623	1	ZNF268|H	2.077	2.39
NR_02989	chr14	1.01E+08	1.01E+08	+	85	1	MIR323A|	0	0
NM_1943	chr1	1.97E+08	1.97E+08	-	8462	1	ZBTB41|FF	1.847	1.431
NM_0808	chr11	63369789	63410240	+	2296	1	SLC22A9|H	0	0
NM_0149(	chr5	1.77E+08	1.77E+08	-	4140	1	RNF44|-|-	32.165	24.856
NR_13298	chr18	36660389	36663418	-	377	1	LOC10537	0	0
NM_1452	chr16	560422	565529	+	2212	1	PRR35|C1(	0.274	0.173
NM_0324	chr8	38264740	38269220	-	856	1	PLPP5|DPI	12.291	10.017
NR_10417	chr1	1.82E+08	1.82E+08	-	568	1	LINC01344	0	0.024
NR_10694	chr19	11113474	11113534	+	60	1	MIR6886|I	6.017	2.739
NM_0011	chr12	79045653	79452008	+	4398	1	SYT1|P65|	41.597	44.878
NR_03675	chr1	2.28E+08	2.28E+08	-	256	1	ZNF847P|g	0	0
NR_00281	chr13	24580208	24597674	+	2705	1	TPTE2P6|-	0	0.035
NM_0206	chr1	1.54E+08	1.54E+08	-	1054	1	S100A14|I	0.11	0
NR_03790	chr5	1.38E+08	1.38E+08	+	2918	1	LOC10013	0.707	0.709
NM_0073	chr9	1.34E+08	1.34E+08	-	5621	1	BRD3|ORF	33.893	36.434
NR_00313	chr1	89407679	89424934	+	1229	1	GBP1P1|-|	0	0
NM_0330	chr7	497260	519844	-	2730	1	PDGFA|PD	1.063	2.378
NM_0056(	chr19	51371620	51372706	-	820	1	NKG7|GIG	0.189	0.401
NR_02699	chr2	1.73E+08	1.73E+08	-	4617	1	RAPGEF4-A	0.02	0.006
NR_11090	chr16	14009276	14016018	-	763	1	LOC10192	0	0
NM_0062	chr20	44966474	45079977	+	6347	1	STK4|KRS2	5.718	6.157
NM_0013	chr6	1.5E+08	1.5E+08	+	7327	1	IYD|C6orf	0.011	0
NM_0010	chr5	61157709	61162475	-	2885	1	SMIM15|C	7.28	6.77
NR_13499	chr12	1.19E+08	1.19E+08	-	457	1	LOC10537	0	0
NM_0012	chr1	53087179	53142632	-	2487	1	SLC1A7|A	0.057	0.154
NR_04756	chr11	45722308	45724555	+	636	1	LOC10050	0	0
NR_00275	chr1	44731069	44731168	-	99	1	RNU5D-1|	0	0
NR_02982	chr3	35744476	35744559	+	83	1	MIR128-2|	0	0
NR_07310	chr13	39038311	39050107	+	3289	1	NHLRC3|-|	3.305	4.419
NR_10999	chr11	62615296	62622175	-	1626	1	B3GAT3|G	25.938	20.798
NM_0209	chr12	4434142	4445614	-	739	1	FGF6|HBG	0	0
NR_02743	chr2	1.71E+08	1.71E+08	-	2128	1	LINC01124	0.024	0.039
NM_2068	chr21	32291813	32314783	+	619	1	MRAP|B27	2.291	2.722

NM_0011:	chr11	33703010	33722952	-	7551	1	CD59|16.3	20.509	23.291
NM_0212!	chr1	24119771	24143285	-	2953	1	IL22RA1|C	0.035	0.009
NM_0011:	chr1	2.44E+08	2.44E+08	-	2767	1	ADSS|ADE	9.781	12.257
NR_03342	chr2	82856803	82857769	+	966	1	DHFRP3|-|	0	0.028
NM_0182	chr20	62796405	62801738	+	2822	1	MRGBP|C:	19.998	18.58
NM_0041	chrX	53191321	53225422	-	6834	1	KDM5C|D:	33.466	34.084
NR_02434	chr16	30919319	30923269	-	3950	1	FBXL19-AS	0.699	0.68
NM_0044	chr4_KI27(	278349	300735	+	1032	2	FRG1|FRG	0	0
NM_0011:	chr12	50175780	50222705	-	3612	1	LIMA1|EPI	5.419	9.507
NM_0035!	chr17	28523941	28538157	+	2687	1	FOXN1|FK	0.01	0
NM_0013:	chr21	39380244	39515506	+	1405	1	WRB-SH3E	31.945	31.079
NM_0252:	chr15	83016429	83067354	-	3280	1	BTBD1|C1	15.016	16.336
NM_0309:	chr11	67991100	68004125	-	2309	1	UNC93B1|	1.524	1.115
NM_1534(	chr19	10654261	10685767	+	3265	1	ILF3|CBTF	130.108	128.009
NM_0176:	chr11	57406954	57427580	-	2581	1	SLC43A3|E	0.2	1.794
NR_02849	chr6	1.59E+08	1.59E+08	-	1183	1	OSTCP1|D	0.403	0.417
NR_10679	chr1	43171652	43171712	-	60	1	MIR6733|	19.985	12.327
NM_0203	chr2	1.96E+08	1.96E+08	+	5310	1	SLC39A10|	6.923	6.784
NM_0011:	chr1	2.26E+08	2.26E+08	+	1672	1	EPHX1|EPI	21.669	33.481
NR_00485	chr6	8652209	8653846	+	482	1	HULC|HCC	0.053	0
NR_03984	chr11	1.34E+08	1.34E+08	-	77	1	MIR4697|-	8.205	15.654
NM_0240:	chr17	48575507	48578381	-	2029	1	HOXB4|HC	0.006	0
NM_0010	chr15	78507564	78537373	+	841	1	HYKK|AGP	0.521	0.879
NM_0010:	chrX	15281709	15314559	-	2769	1	ASB11|-|-	0	0
NM_0013:	chr20	23823767	23826675	-	689	1	CST2|-|-|2	0	0
NR_13508	chr1	2.09E+08	2.09E+08	+	489	1	LINC01696	0	0
NM_0011:	chr1	17406755	17438563	-	3939	1	RCC2|TD-6	30.919	27.992
NR_11089	chr18	45438184	45507036	-	3239	1	SLC14A2-A	0.016	0.013
NM_0010	chr1	11736085	11750771	+	1121	1	AGTRAP|A	10.927	7.233
NM_0196	chr7	1.18E+08	1.18E+08	+	1348	1	ANKRD7|T	0	0.02
NM_0140	chr3	1.26E+08	1.26E+08	-	2520	1	KLF15|KKL	1.289	2.169
NM_0010(	chr17	75724696	75757818	+	5648	1	ITGB4|CD:	0.169	0.507
NM_0123:	chr1	1.6E+08	1.6E+08	-	1781	1	CFAP45|C(	0.601	0.269
NM_0055	chr1	1.56E+08	1.56E+08	+	2058	1	LMNA|CD(	33.217	25.384
NM_0327:	chr19	50797693	50804554	-	1453	1	C19orf48|	25.893	20.362
NM_2013	chr8	1.44E+08	1.44E+08	-	14618	1	PLEC|EBS1	18.268	14.002
NM_0001(	chr11	47214942	47239218	+	1836	1	DDB2|DDE	3.708	5.819
NM_0012	chrX	1.5E+08	1.5E+08	-	1111	1	TMEM185	8.948	11.687
NM_0066!	chr7	99438940	99457377	+	1820	1	CPSF4|CPS	22.365	24.497
NM_0063!	chr11	9384622	9448127	+	6165	1	IPO7|Imp7	11.139	12.895
NR_12641	chr17	69004714	69018323	+	519	1	ABCA9-AS:	0	0.053
NR_02384	chr19	56812480	56813793	+	1313	1	PEG3-AS1|	0	0
NM_0184:	chr1	1.68E+08	1.68E+08	-	5397	1	ADCY10|H	0.031	0.03
NM_0012	chr19	4153601	4173054	+	2500	1	CREB3L3|(	0	0.038
NM_0246:	chr7	88276429	88306913	-	4466	1	STEAP4|ST	0	0
NM_1537:	chr10	23316591	23344843	-	2936	1	C10orf67|	0.132	0.079
NR_12058	chr11	22445672	22492019	-	433	1	LINC01495	0	0.032
NM_0036(	chr14	88005124	88014811	+	4518	1	GPR65|TD	0	0
NM_0012	chr20	2206928	2213151	+	831	1	LOC38878	0.884	0.264
NR_04748	chr11	96443281	96506877	-	644	1	JRKL-AS1|-	0	0

NM_0012	chr2	96497643	96505385	-	1483	1	NEURL3|L	0.27	0.582
NM_0179	chr9	92297358	92325594	-	4300	1	NOL8|C9o	2.864	4.112
NR_03169	chr19	53758232	53758318	+	86	1	MIR1283-	0	0
NM_0044	chr10	17137336	17202071	-	13304	1	TRDMT1|	0.296	0.48
NR_03400	chr9	40992337	41014139	+	1281	1	FRG1HP|-|	2.637	3.603
NR_10379	chr6	21486061	21511892	-	983	1	LINC00581	0	0.014
NM_0011	chr2	86213997	86338083	-	3728	1	REEP1|C2	27.817	33.5
NM_0147	chr14	55148116	55191678	-	2972	1	DLGAP5|D	1.384	2.36
NR_10681	chr12	53056944	53057012	+	68	1	MIR6757|	1.138	0.201
NM_0010	chrX	67568841	67730619	+	8097	1	AR|AIS|AR	0.452	0.377
NM_0002	chr6	1.37E+08	1.37E+08	+	1470	1	PEX7|PBD	4.21	4.37
NR_03998	chr7	64024416	64030102	-	2889	1	LINC0100	0	0
NR_11080	chr17	44299574	44308431	-	5077	1	RUNDC3A-	0.584	0.823
NR_10690	chr8	24953796	24953867	-	71	1	MIR6841|	85.533	36.461
NM_1774	chr2	1.87E+08	1.87E+08	+	3545	1	FAM171B|	14.839	22.12
NM_0011	chr4	1.06E+08	1.06E+08	-	5272	1	TBCK|HSP	5.713	5.186
NR_03026	chr14	1.01E+08	1.01E+08	+	83	1	MIR487B|	0	0
NR_03324	chr7	64582610	64583751	+	1141	1	LOC64174	0	0
NM_0121	chr9	37510892	37576253	-	4562	1	FBXO10|F	13.122	11.727
NM_0321	chr2	1.35E+08	1.36E+08	-	6707	1	ZRANB3|4	0.559	0.737
NR_03654	chr12	1929202	1936576	-	2350	1	LINC0094	0.208	0.105
NM_0011	chr10	34312260	34815325	-	3402	1	PARD3|AS	10.824	10.573
NM_0035	chr6	26103948	26104337	+	387	1	HIST1H4C|	0.233	0.354
NM_0012	chr1	2.05E+08	2.05E+08	+	9997	1	MDM4|HD	2.836	3.923
NR_13433	chr10	1.06E+08	1.06E+08	+	557	1	LOC10537	0	0.049
NM_0012	chr15	67549004	67807117	+	1759	1	MAP2K5|	11.691	26.436
NM_0808	chr20	33662286	33663915	+	566	1	C20orf144	0.342	0.121
NM_1750	chr1	2.28E+08	2.28E+08	+	449	1	HIST3H2B	2.154	1.037
NM_0162	chr4	1.57E+08	1.57E+08	-	3000	1	PDGFC|FA	3.18	8.465
NR_10688	chr3	39138206	39138266	+	60	1	MIR6822|	0.215	0
NM_0064	chr17	45147317	45152101	+	4782	1	HEXIM1|C	9.413	11.254
NM_1738	chr2	1.13E+08	1.13E+08	+	1737	1	IL1RN|DIR	0	0
NM_0021	chr4	1.84E+08	1.84E+08	-	2275	1	IRF2|IRF-2	3.293	4.847
NM_0010	chr22	39312882	39319665	-	1179	1	RPL3|ASC-	464.712	501.573
NM_0012	chr18	32263521	32470484	-	7029	1	GAREM1|	1.357	2.305
NR_13640	chr17	82713908	82716253	-	894	1	LOC10192	0.663	0.567
NR_02714	chr2	1.1E+08	1.1E+08	+	3199	2	LIMS3-LO	0	0
NM_0528	chr19	57134096	57145202	-	2620	1	ZIM3|ZNF	0	0
NM_0011	chr2	74461057	74465410	-	2562	1	MOGS|CD	26.864	21.684
NM_0010	chr14	18601117	18602097	+	979	1	OR11H12|	0	0
NM_0188	chr20	36605734	36612557	+	1108	1	C20orf24|	58.8	67.966
NM_0026	chr8	81440326	81447484	-	3563	1	PMP2|FAE	0.011	2.491
NR_13590	chr8	1.33E+08	1.33E+08	-	2933	1	LRRC6|CIL	1.363	0.999
NM_0012	chr2	10330163	10427617	+	1529	1	HPCAL1|B	29.556	86.195
NM_0161	chr17	59952362	59964756	-	2005	1	RNFT1|PT	1.948	2.029
NM_0001	chr7	44144271	44189423	-	2721	1	GCK|FGQT	8.567	1.404
NM_0307	chr1	38874067	38881626	-	2318	1	GJA9|CX5	0.061	0.142
NM_0784	chr5	1.51E+08	1.51E+08	+	5768	1	SLC36A1|	4.406	4.348
NR_03168	chr4	9556168	9556316	-	148	1	MIR548I2|	0	0
NR_11011	chr3	592105	846015	+	3847	1	LINC0126	0	0

NM_0010⁴	chr4	88520978	88523801	-	1352	1	PIGY|HPM	47.778	54.555
NR_12042	chr13	25181332	25190657	+	1774	1	LINC01053	0.182	0.069
NR_10376	chr14	65212893	65222347	-	1438	1	LOC10012	0	0
NR_02688	chr17	14303740	14305745	+	2005	1	MGC12916	0.817	0.519
NM_0036	chrX	41514936	41923034	-	8267	1	CASK|CAG	18.073	18.389
NM_0011	chr16	66427297	66482842	+	2801	1	BEAN1|BE	1.169	0.748
NR_03762	chr12	1.08E+08	1.08E+08	-	621	1	LOC72873	0.955	0.926
NM_0012	chr13	72727749	72756198	+	2816	1	BORA|C13	1.433	1.892
NR_13450	chr11	6390414	6394998	+	1833	1	SMPD1|AS	17.578	20.706
NM_0166	chr11	45929319	46121434	-	7158	1	PHF21A|B	16.388	16.803
NM_0010	chr11	74748868	74842413	+	7815	1	RNF169|-|	3.682	4.378
NM_0061	chr11	1.14E+08	1.14E+08	+	1573	1	NNMT|-|-	1.18	2.072
NM_0012	chr17	82018703	82022909	-	721	1	CENPX|CE	55.365	43.143
NR_14454	chr21	35886445	35888427	+	1982	1	PPP1R2P2	0	0
NM_0011	chr12	27466810	27502185	+	1261	1	SMCO2|C1	0.102	0.022
NR_13046	chr8	76966768	76966850	-	82	1	MIR3149|	0	0.334
NM_0010	chr17	30194319	30235968	-	6589	1	SLC6A4|5-	0.035	0.029
NR_03174	chr8	74705693	74705747	+	54	1	MIR2052|	0	0
NM_0010	chr12	64266983	64390565	-	1577	1	C12orf56|	0.033	0.035
NM_1537	chr15	43599563	43618800	-	5484	1	STRC|DFN	0.016	0.015
NM_0166	chr5	1.38E+08	1.38E+08	+	6787	1	KDM3B|5c	39.748	38.566
NR_00155	chrY	8683370	8683878	-	219	1	TTTY18|N	0	0.25
NR_13517	chr16	1580527	1610328	+	482	1	LOC10537	0.615	0.057
NM_0011	chr11	73647549	73662819	+	2007	1	PLEKHB1|	9.778	9.937
NM_0013	chr16	681666	684446	-	1945	1	JMJD8|C1	57.388	43.774
NR_04643	chr17	8224821	8248095	-	6880	1	CTC1|AAF	7.095	8.328
NM_0148	chr5	1.09E+08	1.09E+08	-	4857	1	PJA2|Neur	50.016	63.764
NM_0011	chr11	9574687	9589766	+	3205	1	WEE1|WE	4.393	6.94
NM_0011	chr17	3474110	3499406	+	1342	1	ASPA|ACY	0	0.031
NM_0012	chr21	36134965	36146562	+	1107	1	CBR3|HEL	0.233	0.792
NM_0010	chr3	98149386	98150318	+	931	1	OR5H14|-	0	0
NM_1749	chr19	43533188	43536132	+	1705	1	ZNF575|-|	1.898	2.603
NR_12032	chr15	34755084	34812923	+	2724	1	LOC10192	0.478	0.101
NM_0012	chr9	1.12E+08	1.12E+08	-	2957	1	SUSD1|-|-	2.193	4.391
NM_0011	chr9	1.28E+08	1.28E+08	-	1536	1	PIP5KL1|P	4.306	3.727
NM_0014	chr16	10528422	10580716	-	5208	1	EMP2|XM	3.04	1.494
NM_0012	chr17	63523334	63546728	+	3635	1	KCNH6|ER	0.035	0.207
NR_03976	chrX	1.07E+08	1.07E+08	+	82	1	MIR548AN	0	0
NR_11006	chr20	55420336	55427197	-	2287	1	LINC01441	0	0
NM_2070	chr3	1.75E+08	1.76E+08	+	4896	1	NAALADL2	0.037	0.112
NM_0016	chr11	3826978	3840983	-	1315	1	RHOG|ARH	5.138	5.583
NM_0010	chr8_KI27	111992	129199	+	4083	2	ZNF623|-|	0.092	0.054
NR_13466	chr4	6670728	6673881	-	468	1	LOC10537	10.8	6.936
NR_13459	chr6	1.67E+08	1.67E+08	-	625	1	LOC10537	0.041	0.175
NR_13658	chr8	42051750	42104987	+	1158	1	LOC10537	0.022	0.012
NM_0012	chr5	1.39E+08	1.39E+08	-	3621	1	ETF1|D5S1	20.83	18.921
NM_1383	chr2	42048021	42058528	+	2492	1	PKDCC|SG	7.549	7.184
NM_0010	chr11	1.25E+08	1.25E+08	-	931	1	OR8B12|C	0	0
NM_0011	chr2	2.01E+08	2.01E+08	+	4896	1	AOX1|AO|	0.053	0.09
NM_0146	chr18	76357682	76495190	-	8617	1	ZNF516|H	9.991	10.586

NM_0010	chr20	958453	1002264	-	2517	1	RSPO4|C2	0.01	0.016
NM_0014	chr1	1.01E+08	1.01E+08	-	3210	1	EXTL2|EXT	5.788	7.151
NM_0144	chr19	48933682	48944969	+	1072	1	DHDH|2DI	2.369	3.309
NM_0013	chr16	20451461	20487669	+	3319	1	ACSM2A|	0	0
NM_1450	chr6	1.1E+08	1.1E+08	-	2544	1	AK9|AK 9|	5.114	4.684
NM_0011	chr3	1.3E+08	1.3E+08	+	959	1	ALG1L2|-|	0.161	0.129
NR_04657	chr3	1.19E+08	1.19E+08	+	540	1	B4GALT4-	0.955	1.547
NR_04511	chr3	1.11E+08	1.11E+08	-	1124	1	PVRL3-AS1	1.055	0.695
NR_04673	chr3	21542789	21579959	+	729	1	ZNF385D-	0	0
NM_0050	chr6	25782898	25832059	-	1828	1	SLC17A1|	0	0.007
NM_0013	chr19	52764813	52786791	-	3360	1	ZNF600|KI	0.081	0.465
NM_0314	chr11	26332131	26663289	+	5907	1	ANO3|C11	0.024	0.051
NR_02710	chr4	1.24E+08	1.24E+08	+	557	1	LINC01091	4.352	1.008
NM_0012	chr2	60881495	60928156	+	7719	1	REL|C-Rel|	0.306	0.493
NM_0010	chr10	1.28E+08	1.28E+08	+	317	1	NPS|-|-|1	0.244	0
NM_0010	chr1	1.52E+08	1.52E+08	-	3024	1	RORC|IMD	0.055	0.349
NR_03983	chr11	3856062	3856141	+	79	1	MIR4687|	12.077	17.338
NR_01545	chr4	2935546	2951073	+	3862	1	NOP14-AS	7.198	8.554
NM_0010	chr1	1.2E+08	1.2E+08	-	5075	1	ZNF697|-|	8.605	9.085
NM_0072	chr6	36493883	36547516	-	3615	1	STK38|ND	13.404	11.981
NM_0045	chr3	1.21E+08	1.21E+08	+	605	1	NDUFB4|	147.327	133.144
NR_10693	chr8	25345402	25345474	+	72	1	MIR6876|	0	0
NM_0321	chr19	4542588	4559759	-	3923	1	SEMA6B|	43.209	52.997
NM_0070	chr6	26402237	26415216	+	3422	1	BTN3A1|B	3.903	12.192
NM_2135	chr19	57320497	57330776	+	3665	1	ZNF543|-|	3.117	3.961
NM_1445	chr19	11925068	11950773	+	2886	1	ZNF700|-|	1.814	2.079
NM_0052	chr4	1.87E+08	1.87E+08	-	14735	1	FAT1|CDH	11.067	13.189
NR_13465	chr11	73787872	73864611	+	935	1	MRPL48|	36.874	28.068
NR_03896	chr9	1.35E+08	1.35E+08	-	2924	1	PPP1R26-	0.719	0.571
NM_0012	chr15	67200675	67255198	-	3071	1	AAGAB|K	26.824	19.433
NR_04676	chr2	73457103	73459477	+	1759	1	ALMS1-IT1	0.22	0.055
NM_2073	chr1	20552439	20555020	+	2579	1	FAM43B|-	1.93	4.095
NR_03961	chr1	5564071	5564143	+	72	1	MIR4417|	0	0
NM_0011	chr8	1.17E+08	1.17E+08	+	5304	1	SLC30A8|	0.126	0.018
NR_10978	chr7	1.31E+08	1.31E+08	+	943	1	LINC0051	0	0.015
NR_12162	chr8	37516406	37521386	-	525	1	LINC0160	0.025	0
NM_0071	chr19	50376423	50383028	+	2078	1	NR1H2|LX	20.73	21.495
NR_04502	chr17	16438987	16442026	+	921	1	LRRC75A-	463.675	421.053
NM_0012	chr22	39946817	39973342	+	3532	1	GRAP2|G	0.051	0.008
NM_0012	chr19	43192702	43205774	-	1882	1	PSG4|PSB	0	0.015
NM_0191	chr9	1.09E+08	1.09E+08	-	5773	1	EPB41L4B	2.564	2.406
NM_0011	chrX	1.01E+08	1.01E+08	+	1120	1	ARL13A|A	0.161	0.098
NM_0020	chr9	77716274	78031449	-	6334	1	GNAQ|CM	10.457	13.102
NM_0018	chr5	1.76E+08	1.76E+08	-	1154	1	CLTB|LCB|	85.785	87.998
NM_1986	chr21	44627123	44628378	+	1253	1	KRTAP10-	0	0
NM_0013	chr7	27242545	27247819	+	3044	1	EVX1|EVX	29.802	0.657
NM_0180	chr1	2.02E+08	2.02E+08	+	11390	1	IPO9|Imp	25.073	25.87
NM_0010	chr6	31158526	31164215	+	3054.9	7	TCF19|SC1	0	0
NM_0144	chr3	1.09E+08	1.09E+08	-	3737	1	MORC1|C	0.014	0.015
NR_02449	chr16	2638982	2646129	+	7147	1	FLJ42627|	0.408	0.573

NR_00292	chrY	19455431	19503153	-	3452	1	BCORP1|B	0.03	0.063
NM_1304:	chr15	65442463	65517713	-	7287	1	DPP8|DP8	2.442	3.675
NM_0055:	chr6	31969811	31972255	-	1581	6	DXO|DOM	0	0
NM_0012!	chr4	681824	689441	-	1764	1	MFSD7|LP	1.199	0.753
NM_0070:	chr8	86342765	86467949	+	4078	1	WWP1|AII	2.412	2.633
NM_0011:	chr14	77394021	77423056	-	1849	1	NOXRED1|	0.425	0.348
NM_0019!	chr12	1.31E+08	1.31E+08	-	3326	1	STX2|EPIM	13.568	13.648
NM_1816(	chr21	30598686	30598874	-	187	1	KRTAP6-2|	0	0
NM_0010(	chr11	5856749	5857702	-	952	1	OR52E8|O	0	0
NR_03623	chr3	75738280	75738363	+	83	1	MIR4273|-	0.621	0.825
NR_03021	chr19	53752397	53752481	+	84	1	MIR519A1	0	0
NM_0012	chr4	9325165	9326757	+	1591	8	USP17L26	0	0
NM_0010:	chr8	66988131	67028551	-	1076	1	PPP1R42|I	0.359	0.089
NM_0012!	chr3	40506111	40518221	+	2520	1	ZNF620|-|	3.791	3.653
NM_0010!	chr3	1.41E+08	1.41E+08	+	8374	1	ZBTB38|CI	3.046	2.748
NR_03995	chr3	1.75E+08	1.75E+08	+	81	1	MIR4789|-	0	0
NM_1536(	chr16	23388493	23453191	-	2918	1	COG7|CD(	11.444	11.364
NR_04067	chr6	79233674	79236800	+	2991	1	HMGN3-A:	1.241	1.415
NM_0029!	chr17	36064272	36071481	+	776	3	CCL18|AM	0	0
NM_0122:	chrX	304750	318819	-	1895	2	GTPBP6|P	0	0
NM_0010!	chr3	48306846	48328341	-	434	1	SPINK8|-|-	0	0
NR_04665	chr3	23804024	23806905	-	776	1	UBE2E1-A:	0.532	0.088
NM_0073:	chrX	1.23E+08	1.23E+08	+	5165	1	GRIA3|GLI	2.464	9.677
NM_0123:	chr17	58155154	58156086	+	931	1	OR4D1|OF	0	0
NM_0154	chr9	1.36E+08	1.36E+08	-	7613	1	CAMSAP1|	46.253	42.712
NM_0010(	chr19	51761200	51770526	+	1909	1	FPR2|ALXI	0	0.057
NM_1527(	chr1	1.09E+08	1.09E+08	-	3045	1	AKNAD1|(	0.089	0.094
NM_0220(	chr14	22598238	22612359	+	2500	1	ABHD4|AE	11.651	10.601
NM_0020!	chr4	1.44E+08	1.44E+08	-	2686	1	GYPA|CD2	0	0
NM_0011(	chr3	1.57E+08	1.57E+08	-	986	1	VEPH1|MI	3.452	2.778
NM_1735(	chr18	23598926	23662885	-	3349	1	ANKRD29|	0.173	0.11
NM_0011	chr19	55428738	55442863	-	6030	1	SHISA7|-|-	9.571	8.595
NM_0013:	chr8	1.32E+08	1.32E+08	+	5260	1	EFR3A|-|-	1.802	2.109
NM_0018(	chr1	26317920	26320523	+	501	1	CD52|CDV	0	0
NM_0064:	chr8	67197649	67343677	-	7184	1	ARFGEF1|/	4.747	5.335
NM_0041:	chr3	1.72E+08	1.72E+08	-	911	1	GHSR|GHI	0	0
NR_13592	chr10	96830632	96832458	-	1826	1	LOC10272.	2.38	1.853
NM_0012!	chr12	1.05E+08	1.05E+08	+	5780	1	WASHC4|I	1.307	2.064
NM_0012:	chr2	2.38E+08	2.38E+08	+	2123	1	UBE2F|NC	16.969	18.678
NM_0048	chr1	1.78E+08	1.78E+08	+	9801	1	RASAL2|N	6.082	5.376
NR_12584	chr6	1.14E+08	1.14E+08	-	325	1	LOC10192	0.317	0.126
NM_0012!	chr2	1.7E+08	1.7E+08	-	789	1	METTL5|H	29.464	34.199
NM_0179!	chr8	1.24E+08	1.24E+08	+	2216	1	TRMT12|T	6.22	8.443
NR_10990	chr5	61162316	61232080	+	586	1	SMIM15-A	0.748	0.795
NM_0010(	chr3	98353854	98354819	+	964	1	OR5K4|-|-	0	0
NM_0011(	chr7	1.51E+08	1.51E+08	-	1178	1	TMEM176	0.208	0.256
NR_00323	chr14	1.01E+08	1.01E+08	+	73	1	SNORD11:	0	0
NR_03897	chr9	22646200	22824213	+	2341	1	LINC0123!	0	0.029
NM_0011	chr4	4267701	4290021	-	1459	1	LYAR|ZC2I	3.067	2.845
NR_04756	chr3	1.77E+08	1.78E+08	+	1218	1	LINC0057!	0.138	0.022

NM_0049!	chr1	1.55E+08	1.55E+08	+	1775	1	EFNA3|EFI	30.763	40.412
NM_0010{	chr3	1.86E+08	1.86E+08	-	5659	1	DGKG|DA(	0.456	1.377
NM_0011!	chr19	48795062	48811063	-	1586	1	BCAT2|BC/	16.584	9.759
NM_0012;	chr12	31671137	31729174	-	2081	1	AMN1|-|-	9.833	14.151
NM_0228!	chr5_KI27(	516878	573494	-	5303.8	5	NAIP|BIRC	0	0.003
NM_1445;	chr14	55051644	55070194	+	6463	1	MAPK1IP1	9.827	8.191
NM_0012!	chr19	33374524	33382686	+	3629	1	CEBPG|GP	19.456	20.823
NM_0028(	chr17	81902901	81911477	-	3209	1	PCYT2|ET|	11.985	11.734
NR_13239	chr14	95876392	95925571	+	2937	1	TUNAR|HI	9.847	10.866
NM_0318{	chr16	56484347	56520096	-	2795	1	BBS2|BBS	20.348	22.126
NM_1828;	chr2	20666664	20679241	+	9741	1	GDF7|BMI	0.42	0.135
NM_0010(	chr1	1.49E+08	1.49E+08	+	5658	1	PDE4DIP|(	4.141	5.449
NM_0010:	chr4	1.65E+08	1.65E+08	+	1935	1	MSMO1|D	41.766	45.062
NM_0012!	chr10	44292750	44294649	+	1105	1	C10orf142	0	0.012
NM_0251(	chr15	43772600	43777304	-	2100	1	ELL3|-|-|1	1.295	1.128
NM_0013;	chr10	17644125	17716822	+	3769	1	STAM|STA	10.817	11.179
NM_0011(	chr1	2.01E+08	2.01E+08	+	11784	1	IGFN1|EEF	0	0.008
NR_13462	chr12	585699	586486	-	537	1	LOC10536!	0	0
NR_02726	chr21	34180677	34183245	+	653	1	LINC0031C	1.007	0.336
NM_0064!	chr1	1.1E+08	1.1E+08	+	4721	1	GNAI3|87I	21.182	20.971
NM_0010	chr19	7680821	7682862	+	675	1	TRAPPC5|	50.371	44.236
NM_0013;	chr1	1.6E+08	1.6E+08	-	676	1	CRP|PTX1	0	0
NR_03609	chr4	10078611	10078692	-	81	1	MIR3138|	0	0
NM_0010;	chr10	49988318	50010499	+	2762	1	AGAP6|AG	1.503	1.666
NM_1989!	chr7	1.03E+08	1.03E+08	-	5054	2	NAPEPLD|	0	0
NM_0142!	chr22	38818451	38844012	-	5808	1	NPTXR|NP	23.329	97.296
NM_1816;	chr21	30425393	30425912	-	518	1	KRTAP13-3	0	0
NR_10682	chr12	1.33E+08	1.33E+08	+	64	1	MIR6763|	2.216	5.136
NR_13094	chr10	42581592	42638837	-	2221	1	ZNF33B|K(	3.878	3.404
NR_13626	chr7	30247429	30286085	-	1227	1	LOC10537.	0	0
NM_0010:	chr18	75409476	75427703	-	1829	1	SMIM21|(	0.014	0
NM_0010(	chr3	98469480	98470576	+	1094	1	OR5K1|HS	0	0
NM_0010{	chr2	27032906	27041697	+	2887	1	TMEM214	13.228	9.498
NM_0010(	chr14	21665083	21666024	+	940	1	OR4E2|OR	0	0
NR_11016	chr2	1.61E+08	1.61E+08	+	2331	1	LINC01806	0	0.006
NM_0581;	chr7	18496262	18954316	+	3115	1	HDAC9|HD	2.856	7.506
NM_0013	chr12	1.1E+08	1.1E+08	+	2546	1	IFT81|CDV	6.092	6.429
NM_0332{	chr5	69217746	69230158	+	1323	2	MRPS36|D	2.037	1.553
NR_03171	chr8	10825373	10825443	-	70	1	MIR1322|	0	0
NM_1452	chr1	58480719	58546799	-	1923	1	OMA1|20:	1.106	1.368
NM_0067{	chr18	9475532	9538108	+	4342	1	RALBP1|RI	9.25	7.173
NM_0150(	chr7	32495426	32588741	+	7047	1	AVL9|KIAA	12.354	15.168
NM_0042	chr10	1.19E+08	1.19E+08	-	1976	1	PRLHR|GP	0.026	0.014
NR_03885	chr9	99886318	99906601	-	549	1	STX17-AS1	0.141	0.249
NR_00128	chr22	24764501	24766019	+	1518	1	TOP1P2|-|	0	0.009
NM_1307(	chr5	81330128	81394169	-	1972	1	ACOT12|C	0.052	0.035
NM_0011	chr21	46458949	46570013	+	7238	1	DIP2A|C21	7.32	6.341
NM_0147!	chr8	96261886	96334551	+	2547	1	PTDSS1|LN	45.13	51.4
NM_1446:	chr19	35754566	35757029	-	1455	1	HSPB6|HE	0.372	0.226
NM_0189	chr1	8011719	8026333	-	3123	1	ERRFI1|GE	11.172	12.136

NM_0140	chr3	87227263	87255548	+	2620	1	CHMP2B|/	6.329	9.75
NR_11087	chr17	72021851	72025979	-	360	1	LINC02095	0.215	0.076
NM_0211	chr19	15912370	15934866	-	3381	1	CYP4F11|(	0	0.008
NR_02405	chr19	56840902	56848554	+	1286	1	MIMT1|LII	0	0
NM_0010	chr12	14804650	14814182	-	2100	1	SMCO3|C1	0.092	0.039
NM_0306	chr1	23359448	23368386	-	4310	1	ZNF436|ZI	14.512	20.11
NR_03338	chr1	1.61E+08	1.61E+08	+	1102	1	KLHDC9|K	12.32	5.755
NR_13575	chr15	43593834	43599406	+	2441	1	CKMT1B|(	1.849	2.25
NM_0012	chr19_GL9	165696	169508	+	2413.1	10	TSEN34|LE	0	0
NM_0322	chr8	43093506	43123180	+	1616	1	POMK|MD	5.05	4.806
NR_13366	chr7	1.01E+08	1.01E+08	+	14186	1	MUC17|M	0.006	0
NR_04674	chrX	48056310	48067308	+	1120	1	ZNF630-AS	0.495	0.477
NM_0037	chr5	1.41E+08	1.42E+08	+	4768	1	PCDHGA12	19.351	77.922
NR_13549	chr19_KI2	191261	217825	-	2202	10	LILRB3|CD	0	0.012
NM_0153	chr14	31100115	31207523	-	9132	1	HECTD1|E	7.606	11.281
NM_0321	chr16	11976734	12574289	+	8152	1	SNX29|A-3	10.777	11.192
NR_04670	chrX	38801248	38803883	-	694	1	MID1IP1-A	2.211	1.816
NM_0010	chr9	81988772	81995256	+	4827	1	SPATA31D	0	0
NM_0044	chrX	53431258	53434376	-	955	1	HSD17B10	61.025	48.09
NM_0209	chr2	2.08E+08	2.08E+08	-	624	1	CRYGC|CC	1.736	0.834
NM_0012	chr15	99727401	99733444	-	2427	1	LYSMD4|-	12.097	8.578
NM_0011	chr6	26368178	26378320	+	2825	1	BTN3A2|B	0.703	1.808
NM_0217	chr1	1.2E+08	1.2E+08	-	2989	1	ADAM30|	0	0
NM_2034	chr13	36673912	36697838	+	3202	1	SERTM1|C	0.693	0.359
NM_0015	chr6	52977948	52995380	-	1332	1	GSTA4|GS	182.977	200.602
NR_03657	chr7	1.49E+08	1.49E+08	+	3143	1	LOC15506	1.731	2.379
NM_0010	chrX	1.55E+08	1.55E+08	-	2270	1	G6PD|G6F	34.341	25.566
NM_0011	chr9	1E+08	1E+08	-	2991	1	TEX10|Ipi1	6.259	10.675
NM_0181	chr10	30309801	30349338	-	5609	1	MTPAP|PA	2.683	3.262
NM_1525	chr22	39743044	39893790	-	2701	1	ENTHD1|C	0	0
NM_0011	chr1	13061232	13062237	+	1003	3	HNRNPCL3	0	0
NR_12602	chr7	1.44E+08	1.44E+08	+	985	1	LOC10192	0	0
NM_1722	chr10	5952371	5977611	-	1540	1	IL15RA|CD	0.008	0
NR_00334	chr15	25232387	25232468	+	81	1	SNORD115	0	0
NM_0221	chr17	82735576	82751197	+	1425	1	FN3K|-|-|	21.652	20.81
NM_1993	chr11	62787402	62790400	+	1036	1	TMEM179	18.954	15.244
NM_0012	chr1	2.45E+08	2.45E+08	+	3991	1	DESI2|C1o	6.138	7.173
NM_0064	chr1	1.1E+08	1.1E+08	-	842	1	LAMTOR5	97.819	79.677
NM_0016	chr2	2.1E+08	2.1E+08	-	2477	1	ACADL|AC	0	0.072
NM_2069	chr14	94081293	94102714	+	662	2	IFI27L1|FA	0	0
NM_0027	chrX	1.08E+08	1.08E+08	+	2147	1	PRPS1|AR	21.415	28.549
NM_0022	chr1	1.83E+08	1.83E+08	+	7859	1	LAMC1|LA	10.67	10.283
NM_0012	chr2	23748664	23927114	-	8058	1	ATAD2B|-	2.109	2.434
NM_0121	chr6	47477789	47627260	+	5389	1	CD2AP|CN	0.668	0.694
NR_02832	chr1	725759	730351	-	4270	1	LOC10013	0.13	0.135
NM_1994	chr20	35302566	35412142	-	2359	1	UQCC1|BF	26.23	27.127
NM_0012	chr9	1.27E+08	1.27E+08	+	3570	1	GARNL3|b	9.217	10.175
NM_0035	chr16_KI2	245407	267435	-	895	2	PLA2G10|(	0	0
NR_03397	chr11	9754770	9759533	-	2308	1	LOC44002	0.056	0.077
NR_04984	chr14	53948427	53948484	-	57	1	MIR5580|	0	0

NM_0013:	chr19	52905893	52917931	-	2482	1	ZNF888|-|	0.187	0.579
NM_0011	chr2	1.91E+08	1.91E+08	-	3626	1	NEMP2|TM	0.409	0.404
NM_0156	chr14	1.04E+08	1.04E+08	+	6740	1	KIF26A|-|-	3.474	10.061
NM_0012	chr12	1.12E+08	1.12E+08	+	1905	1	ALDH2|AL	44.217	70.117
NM_0013:	chr14	37595199	37842625	+	6865	1	TTC6|C14	0.03	0.004
NR_13456	chr6	42879616	42889896	+	3694	1	RPL7L1|dJ	16.956	22.099
NM_0178	chr22	17137520	17165287	-	1716	1	CECR5|-|-	42.941	37.507
NM_1391	chr8	1.07E+08	1.07E+08	-	2750	1	ABRA|STA	0	0.01
NM_0012	chr16_KI2	1021570	1036245	-	1079	2	NPIPA5|NI	0.072	0.038
NM_0010:	chr19	45890023	45902604	-	1882	1	MYPOP|P4	3.254	4.258
NM_1985	chr8	1.44E+08	1.44E+08	+	2029	1	SPATC1|SI	0	0.02
NM_1735	chrX	1.21E+08	1.21E+08	-	1288.7	10	CT47A11|	0	0
NM_0011	chr5	1.5E+08	1.5E+08	+	10497	1	PPARGC1B	0.152	0.372
NM_0020	chr3	1.16E+08	1.16E+08	+	1703	1	GAP43|B-5	848.963	632.548
NR_03971	chr11	1.11E+08	1.11E+08	+	65	1	MIR4491|	0	0
NM_0240	chr15	45402321	45421418	+	2569	1	SPATA5L1	5.456	6.291
NR_00337	chr16	21796630	21819174	-	1597	1	RRN3P1|-|	1.316	1.527
NR_03979	chr7	44110849	44110912	+	63	1	MIR4649|	2.047	2.392
NM_0245	chr8	70051651	70071327	-	2334	1	PRDM14|	0	0.012
NM_0011	chr11	616565	625067	-	3454	2	CDHR5|M	0.019	0.028
NM_0144	chr5	62388524	62403901	-	1543	1	DIMT1|DI	5.59	7.989
NM_0169	chr21	29005759	29019364	-	1698	1	RWDD2B|	0.714	3.283
NM_0190	chr12	1.18E+08	1.18E+08	-	4992	1	VSIG10|-|	1.441	1.627
NR_00307	chr17	2330279	2330370	-	91	1	SNORD91A	0	0.301
NM_0053	chr16	180334	181179	+	648	1	HBQ1|HB	1.433	2.198
NM_0022	chr21	37624223	37916438	-	2784	1	KCNJ6|BIR	1.051	2.568
NR_00325	chrX	73792205	73829231	+	37026	1	TSIX|LINC	0.004	0.009
NR_11013	chr12	73159190	73208317	+	1252	1	LOC10192	0	0
NM_0063	chr15	68778536	68820922	-	2457	1	ANP32A|C	66.089	94.642
NM_0028	chr14	52707178	52727998	+	1577	1	PSMC6|CA	14.153	14.192
NR_13073	chr5	1.69E+08	1.69E+08	+	1942	1	LOC10192	0.345	0.578
NR_03835	chr14	80211419	80455467	+	1586	1	DIO2-AS1|	0.016	0.104
NM_0177	chr1	23428563	23484257	-	4102	1	ASAP3|AC	22.257	24.482
NM_0320	chr11	1.19E+08	1.19E+08	+	2812	1	RNF26|-|-	17.896	20.536
NR_12597	chr1	1.16E+08	1.16E+08	-	433	1	LINC01762	0	0
NR_14448	chr11	8092965	8169094	-	2704	1	RIC3|AYST	7.191	9.32
NM_0329	chr17	35983656	35986728	-	501	2	CCL14|CC-	0	0
NR_13428	chr8	1.45E+08	1.45E+08	+	2738	1	ZNF517|-|	10.092	10.135
NM_0322	chr9	67859315	67902394	+	3494	2	ANKRD20A	0	0
NM_0025	chr16	1770320	1771709	-	1007	1	NME3|DR-	4.878	3.972
NM_0219	chr5_GL33	492256	510145	-	1908	5	SERF1A|4F	0.027	0.014
NM_1522	chr19	37181579	37210549	-	6203	1	ZNF585B|	1.592	2.288
NR_12584	chr6	5452468	5458075	-	470	1	LOC10192	0	0
NR_03766	chr20	1309911	1393172	-	1677	1	FKBP1A-SD	3.944	4.762
NR_02677	chr6	26956993	27023974	+	974	1	LINC0024C	0.199	0.042
NR_12536	chr4	52659406	52661498	+	876	1	USP46-AS1	0.868	0.907
NM_0012	chrX	1.21E+08	1.21E+08	-	1288.7	10	CT47A12|	0	0
NM_0011	chrX	41334162	41350287	+	4626	1	DDX3X|CA	48.773	55.354
NR_10683	chr16	85918347	85918416	+	69	1	MIR6774|	0	0
NM_0220	chr8	1.2E+08	1.21E+08	+	3063	1	MTBP|MD	0.594	1.149

NM_01822	chr11	12377479	12535356	+	8704	1	PARVA|CH	2.324	1.945
NM_00119	chrX	1.03E+08	1.03E+08	+	3931	1	ARMCX5-G	21.149	18.836
NM_00108	chr16	29962030	29973052	+	991	1	TMEM219	67.954	54.581
NM_00100	chr1	1.56E+08	1.56E+08	-	1011	1	VHLL|VHLI	0.051	0.176
NM_00113	chr19	52064466	52095765	-	3867	1	ZNF841|-|	2.067	4.141
NM_00406	chr8	94127166	94208587	-	3678	1	CDH17|CD	0.046	0.037
NM_19856	chr3	1.97E+08	1.97E+08	+	2548	1	NRROS|EL	0.101	0.102
NM_00205	chr2	69319769	69387254	-	8628	1	GFPT1|CM	8.428	6.915
NM_00275	chr17	69414697	69542329	+	1855	1	MAP2K6|M	8.716	9.525
NM_00132	chr8	70109762	70403255	-	8271	1	NCOA2|GF	7.174	7.459
NM_00104	chr17	79074937	79088603	+	4478	1	ENGASE|-|	6.196	8.687
NM_00116	chr5	1.8E+08	1.8E+08	-	806	1	CBY3|-|-|5	0.272	0.221
NR_03997	chr1	52940314	52940413	+	99	1	MIR1273G	0	0
NM_00128	chr3	15560704	15601852	-	2025	1	HACL1|2-H	5.52	6.453
NM_00103	chr19	44072144	44089613	+	4344	1	ZNF284|ZN	1.805	1.501
NM_00132	chr9	74980794	75027151	-	4062	1	CARNMT1	0.54	0.843
NM_00100	chr14	19780323	19781264	+	940	1	OR4M1|O	0	0
NM_03284	chr7	1.3E+08	1.3E+08	-	3528	1	TMEM209	4.78	3.991
NM_02246	chr2	20617804	20651104	-	2394	1	HS1BP3|ET	9.678	7.381
NR_01537	chr5	1.12E+08	1.12E+08	+	1191	1	EPB41L4A-	26.75	37.974
NM_00314	chr7	1.42E+08	1.42E+08	+	619	2	SSBP1|Mt-	14.456	19.406
NR_00319	chr14	1.01E+08	1.01E+08	+	75	1	SNORD114	0	0
NR_00153	chrY	21583600	21594666	-	653	1	TTTY13|NC	0	0
NR_05196	chr13	52167710	52194467	-	2771	1	MRPS31P5	9.05	10.069
NR_00297	chr11	93735110	93735236	-	126	1	SNORA40|	0.614	1.413
NM_17848	chr20	18813726	18814391	+	663	1	SCP2D1|C2	0	0
NM_00119	chr6	1.71E+08	1.71E+08	-	1144	1	PDCD2|RP	17.233	16.068
NM_00128	chr2	2.3E+08	2.3E+08	-	9802	1	TRIP12|TR	16.504	19.454
NM_00125	chr3	51968510	51974630	-	1999	1	ABHD14B|	2.219	1.603
NM_00088	chr16	31355134	31382997	+	4671	1	ITGAX|CD1	0	0
NR_10999	chr3	1.16E+08	1.16E+08	+	544	1	LSAMP-AS	0	0
NM_00062	chr13	46831542	46897076	-	5436	1	HTR2A|5-H	0.128	0.111
NR_03023	chrX	1.47E+08	1.47E+08	-	87	2	MIR514A2	0	0
NM_00702	chr3	50354749	50359508	-	2091	1	TMEM115	31.559	32.176
NM_03196	chr17	3723903	3726698	+	2793	1	GSG2|HAS	0.743	0.53
NR_03020	chr19	53725442	53725526	+	84	1	MIR516B2	0	0
NM_00542	chr1	43300895	43323110	+	3957	1	TIE1|JTK14	0.277	0.187
NM_00130	chr17	75012664	75021261	+	887	1	MRPL58|D	28.578	29.51
NM_05284	chr12	1.1E+08	1.1E+08	-	4127	1	MMAB|AT	11.928	7.972
NM_00117	chr1	1.74E+08	1.74E+08	-	2140	1	CENPL|C16	1.597	2.291
NR_07312	chr10	4826177	4848059	+	1613	1	AKR1E2|A	0.088	0.119
NM_00132	chr10	3067970	3136805	+	2496	1	PFKP|ATP-	43.8	21.489
NM_00112	chr22	50551112	50562952	-	491	1	SYCE3|C22	0.341	0.251
NM_00103	chr16	31083424	31088809	-	2016	1	PRSS53|PO	4.931	3.01
NR_13670	chr3	28349149	28526233	+	2558	1	ZCWPW2|	0.136	0.225
NR_02657	chr11	18112472	18116132	-	639	1	SAA3P|SA	0	0
NM_00100	chr1	2.48E+08	2.48E+08	+	937	1	OR2M3|O	0	0
NR_02695	chr1	1.7E+08	1.7E+08	-	1721	1	LINC01142	0	0.032
NR_00301	chr1	2.04E+08	2.04E+08	+	124	1	SNORA77|	1.456	0.773
NM_00114	chr11	90200430	90223364	-	3315	1	CHORDC1|	2.485	2.289

NR_02679	chr9	96725821	96727467	+	1646	1	LOC44145.	0.243	0.441
NM_0073(	chr12	10372353	10390054	-	1579	1	KLRK1|CD:	0.024	0.017
NM_0013:	chr1	85318485	85404528	-	3662	1	DDAH1|DI	9.397	11.015
NM_0010:	chr11	1697195	1697755	+	518	3	KRTAP5-6|	0	0
NM_0011(	chr6	10722915	10731129	+	1179	1	TMEM14C	72.987	47.062
NR_11037	chr12	1.25E+08	1.25E+08	-	1980	1	THRIL|BRI:	3.002	3.597
NM_1527!	chr7	30134936	30162765	+	5809	1	MTURN|C	38.361	47.316
NR_13462	chr6	1.69E+08	1.69E+08	-	959	1	LOC10192!	0	0
NM_0152(	chr1	19882395	19912944	+	6504	1	OTUD3|DU	2.912	4.05
NM_0011!	chr1	1.74E+08	1.74E+08	+	845	1	LOC73015!	0	0
NM_0014(	chr8_KI27(	494375	588925	-	2648	2	ADAM2|CI	0	0
NM_0204(	chr6	1.38E+08	1.39E+08	-	7481	1	NHSL1|C6(	8.795	12.626
NM_1393:	chr20	44885599	44908532	+	3118	1	YWHAB|G	107.312	134.189
NR_02425	chr12	8231049	8242946	-	2138	1	FAM86FP|	0.591	0.762
NM_1738!	chr1	2.06E+08	2.06E+08	-	5004	1	SLC41A1|I	7.318	12.794
NR_02694	chr10	1.18E+08	1.18E+08	+	3169	1	CASC2|C1(	0.419	0.255
NM_0010:	chr12	51817892	51831917	-	4690	1	FIGNL2|-|-	3.359	1.232
NR_10678	chr1	8866502	8866590	-	88	1	MIR6728|I	32.82	14.942
NM_0011:	chr7	1.03E+08	1.03E+08	-	2036	1	DNAJC2|M	2.717	3.465
NR_12160	chr3	1.39E+08	1.39E+08	+	661	1	LOC10050	4.233	2.238
NR_03063	chr2	1.75E+08	1.75E+08	-	76	1	MIR933|M	0	0
NR_00274	chr17	16440036	16440106	+	70	1	SNORD49A	4.789	3.913
NM_0314(	chr8	74984473	75034561	+	4315	1	CRISPLD1|	5.585	4.133
NM_0048:	chr6	41335790	41350887	+	934	1	NCR2|CD3	0	0.015
NM_0028!	chr19	44846136	44878941	+	2143	1	NECTIN2|(	41.424	40.017
NM_0010:	chrX	1.55E+08	1.55E+08	-	1009	1	CMC4|C6.	10.657	9.828
NM_0025-	chrX	68042344	68433457	-	7504	1	OPHN1|AI	3.966	10.181
NM_0143:	chr15	48878093	48880183	+	2088	1	EID1|C15c	71.161	55.779
NM_0010(	chr22	30425623	30429054	+	1040	1	MTFP1|HS	12.05	7.072
NM_1471!	chr20	45607939	45631268	-	652	1	WFDC9|W	0	0
NR_11007	chr7	80371517	80374439	-	503	1	LOC10192	0.205	0
NR_02670	chr5	1.41E+08	1.41E+08	+	87	1	VTRNA1-3	2.223	2.047
NM_0010(	chr11	1.24E+08	1.24E+08	+	934	1	OR10G9|C	0	0
NM_0012(	chr1	31790422	31816051	-	2484	1	SPOCD1|P	0.031	0.127
NM_0011:	chr20	32052188	32101854	+	2138	1	HCK|JTK9|	0.024	0.038
NM_2073:	chr12	1.2E+08	1.2E+08	+	3070	1	BICDL1|BI(	8.853	7.089
NM_0221!	chr7	25224572	25228486	-	1183	1	NPVF|C7o	0.065	0
NR_11066	chr17	41930623	41965650	+	1844	1	TTC25|CIL	4.286	3.573
NM_0175:	chrX	1.54E+08	1.54E+08	+	6717	1	PLXNA3|6.	29.651	27.182
NM_0009!	chr5	40831328	40835285	-	1564	1	RPL37|L37	222.899	207.697
NM_0010:	chr1	1449689	1470158	+	3845	1	ATAD3C|-	0.107	0.224
NM_0011:	chr2	88448207	88452535	-	1260	1	FOXI3|-|-|	0.225	0.185
NM_0011:	chr19	32405749	32485893	+	5981	1	DPY19L3|-	6.101	11.895
NM_0010(	chr8	96142333	96160792	-	3694	1	GDF6|BMI	0.332	0.082
NM_0051!	chr8	47736914	47738164	-	1248	1	CEBPD|C/I	0.558	1.58
NR_04518	chr13	36346431	36369735	+	685	1	SPG20-AS1	0.151	0.3
NM_0012:	chr7	74027789	74069906	+	3551	1	ELN|SVAS	3.98	2.8
NM_0151-	chr4	98995637	99062809	+	2757	1	METAP1|I	10.976	16.027
NM_0023(	chr12	21635342	21657855	-	1306	1	LDHB|HEL	545.1	649.402
NM_0061(	chr14	22920511	22929585	-	2512	1	PRMT5|HI	62.342	53.556

NM_0010:	chr11_KI2:	97515	98873	-	967.3	3	KRTAP5-4|	0	0.014
NR_02969	chr19	51693254	51693339	+	85	1	MIR125A|	0	0
NM_0216	chr13	52024691	52033600	+	5525	1	UTP14C|2	5.512	7.103
NM_0012	chr15	82659281	82709908	-	3607	1	AP3B2|NA	31.971	33.99
NM_0012	chr15	32389521	32403290	+	5222.2	5	GOLGA8H	0.005	0.008
NR_03840	chr7	47761476	47766772	+	1185	1	LINC00525	0	0
NM_0189	chr2	950868	1367612	+	1277	2	SNTG2|G2	1.363	0.397
NM_0012	chr2	2.01E+08	2.01E+08	+	5695	1	CASP10|A	0.009	0.067
NR_03063	chr8	1.44E+08	1.44E+08	-	81	1	MIR939|M	17.032	7.609
NR_02708	chr16	35363416	35391713	+	1364	1	LINC01566	0	0
NR_02408	chr4	82613113	82621437	-	844	1	LINC00575	0.015	0.049
NM_0068	chr22	38468062	38483447	+	1721	1	KDELR3|E	1.85	0.629
NM_0010	chr14	99481402	99511515	+	2593	1	CCNK|CPR	21.416	25.001
NM_0324	chr11	95976593	96343180	-	7104	1	MAML2|M	1.65	1.772
NM_0162	chr9	1.37E+08	1.37E+08	+	2754	1	MAN1B1|	30.516	31.92
NM_0191	chr19	45093173	45147285	+	2937	1	PPP1R37|	20.08	20.884
NM_0122	chr20	21303284	21389825	+	3423	1	XRN2|-|-|	44.545	45.085
NM_0225	chr15	81000954	81004004	+	3048	1	MESDC1|-	10.503	8.327
NR_03655	chr12	13370671	13376745	-	3199	1	LINC01559	0	0
NM_0048	chr17	7583647	7588209	+	1609	1	MPDU1|C	38.568	38.018
NM_1826	chr5	1225355	1246189	+	2107	1	SLC6A18|)	0	0
NR_11073	chr3	363373	385795	-	907	1	CHL1-AS1|	0.028	0.015
NR_02813	chr12	89708955	89711952	+	2997	1	ATP2B1-AS	1.14	1.074
NM_0012	chr5	32710637	32791724	+	6370	1	NPR3|ANP	0.057	0.273
NM_0206	chr19	49527618	49543633	+	1845	1	RCN3|RLP	8.274	4.187
NM_0188	chr5	1.41E+08	1.41E+08	+	6030	1	PCDHAC2|	15.117	14.574
NM_1445	chr10	1.22E+08	1.22E+08	+	1859	1	BTBD16|C	0.014	0
NM_1826	chr1	1.55E+08	1.55E+08	+	1105	1	EFNA4|EF	7.409	5.454
NR_11710	chr8	1.28E+08	1.28E+08	-	870	1	CASC11|C	0	0.016
NM_0123	chr7	1.09E+08	1.09E+08	+	2533	1	DNAJB9|E	8.088	7.679
NR_04024	chr12	1.06E+08	1.07E+08	-	565	1	LOC10028	1.552	1.115
NR_03784	chr1	2549920	2557011	-	4612	2	LOC11511	0	0
NM_0226	chrX	1.41E+08	1.41E+08	-	437	1	SPANXC|C	0	0
NR_02724	chr2	1.1E+08	1.1E+08	-	2180	1	LINC01106	0	0
NM_0013	chr10	1.14E+08	1.14E+08	+	5783	1	VWA2|AM	0.04	0.031
NM_0010	chr5	1.48E+08	1.48E+08	+	447	1	SPINK9|LE	0	0
NM_0329	chr10	6088986	6117459	+	3321	1	RBM17|SP	19.121	22.614
NR_01543	chr14	97925610	97978124	-	1455	1	LINC01550	0.168	0.678
NM_0072	chr17	7240419	7242434	-	887	1	GABARAP|	507.817	523.853
NM_0010	chr11	64726911	64744857	-	2225	1	RASGRP2|	3.935	8.058
NM_1453	chr10	69630247	69633599	+	1070	1	C10orf35|	54.671	44.791
NR_00293	chr16	29302287	29365059	+	1576	1	SNX29P2|	0	0.026
NM_0239	chr19	15673018	15697174	+	1768	1	CYP4F12|	0	0.046
NM_0010	chr12	55129769	55130776	+	1006	1	OR9K2|OF	0	0
NR_03019	chr19	53701227	53701287	+	60	1	MIR520B|	0	0
NM_1758	chr11	82732004	82733864	-	1858	1	FAM181B|	3.72	4.932
NM_0163	chr17	63600872	63608365	+	1462	1	TACO1|CC	14.313	15.449
NM_0021	chr20	20368121	20370948	+	2825	1	INSM1|IA-	89.51	67.704
NR_13278	chr1	95061596	95067545	+	1094	1	LOC10192	0	0.013
NR_04985	chr2	1.07E+08	1.07E+08	-	51	2	MIR548AU	0	0

NM_0011	chr6	7329097	7389709	-	3059	1	CAGE1|CT	0	0.004
NM_0011	chr3	31981774	31991736	+	3202	1	ZNF860|-|	0	0.051
NM_0011	chr3	1.12E+08	1.12E+08	+	5868	1	PHLDB2|LI	0.815	0.345
NM_0011	chr9	1.27E+08	1.27E+08	+	5845	1	ZBTB43|ZE	3.571	4.78
NM_0013	chr16	29442905	29454205	-	1295	1	BOLA2-SM	0.558	0.888
NR_00232	chr16	1962334	1962466	-	132	1	SNORA10|	0.098	0.519
NM_0011	chr11	63079940	63144221	-	2089	1	SLC22A24|	0	0
NR_00331	chr15	25059537	25059634	+	97	1	SNORD116	5.051	4.66
NM_0305	chr12	21526257	21532951	+	2315	1	SPX|C12or	3.47	0.509
NR_02966	chr12	62603686	62603769	+	83	1	MIRLET7I|	1.864	0.495
NM_1988	chr13	45780281	45851711	-	3990	1	SIAH3|-|-|	1.587	3.169
NM_0073	chr13	1.14E+08	1.14E+08	-	4177	1	RASA3|GA	21.249	18.114
NR_10690	chr8	1.25E+08	1.25E+08	-	61	1	MIR6844|	0.423	1.123
NM_0012	chr7	1.17E+08	1.17E+08	+	2971	1	CAV2|CAV	0.143	0.263
NM_0013	chr12	63844753	64147863	+	8420	1	SRGAP1|A	7.119	7.488
NM_0013	chr20	45541845	45547426	-	775	1	EPPIN|CT7	0	0
NM_0013	chr21	17512906	17570111	+	5732	1	CXADR|CA	15.512	13.054
NM_0153	chr20	32358344	32439319	+	7025	1	ASXL1|BO	27.175	34.918
NM_0052	chr17	29246857	29254484	+	782	1	CRYBA1|C	0.214	0.263
NM_0331	chr8	86047111	86069697	-	2579	1	PSKH2|-|-	0.005	0
NM_0165	chr17	1494577	1516888	-	2968	1	INPP5K|PI	22.051	24.704
NM_1384	chr22	41229510	41240931	-	2525	1	CHADL|SL	0.577	0.629
NM_0010	chr7	75416781	75486288	-	5844	1	POM121C	6.078	5.787
NR_00329	chr15	25180497	25180578	+	81	1	SNORD115	0	0
NM_0013	chr4	69588417	69647698	-	2454	1	UGT2A1|U	0	0
NM_0013	chr22	28742680	28757518	+	867	1	HSCB|DNA	6.424	6.856
NM_0248	chr19	16549837	16572382	-	5085	1	SLC35E1|-	14.747	14.589
NM_0013	chr11	83157095	83185793	+	5891	1	PCF11|-|-	4.618	6.529
NM_0184	chr4	55395913	55426175	+	1967	1	TMEM165	11.628	11.831
NM_0053	chr12	54497711	54543115	+	3860	1	NCKAP1L|	0.08	0.032
NR_13807	chr9	1.37E+08	1.37E+08	+	749	1	C9orf142|	42.433	38.421
NM_0179	chr22	18077920	18091031	+	4075	1	PEX26|PBI	18.481	19.956
NM_0231	chr14	94026378	94048930	+	3865	2	OTUB2|C1	0	0
NM_0071	chr3	1.14E+08	1.14E+08	-	2945	1	ZNF80|pT	0.039	0.037
NM_0011	chr15	48191670	48203754	+	2691	1	CTXN2|-|-	0.628	0.799
NR_11011	chr7	20296708	20311712	+	1119	1	LOC10192	0	0
NR_10700	chr8	1.33E+08	1.33E+08	-	100	1	MIR7848|	0	0
NM_0012	chr4	82430480	82461175	+	2154	1	ENOPH1|E	29.941	29.88
NM_0010	chr11	32602080	32794658	-	4012	1	CCDC73|N	5.232	5.2
NM_0028	chr12	15322257	15598331	+	6267	1	PTPRO|GL	17.687	16.143
NM_0012	chr3	38845769	38953651	-	6302	1	SCN11A|F	0.137	0.124
NR_11028	chr2	77743696	78290731	-	672	1	LOC10192	0.038	0
NM_0057	chr7	1.06E+08	1.06E+08	-	4568	1	NAMPT|1	1.479	1.808
NM_0011	chr4	37245068	37449465	+	7468	1	NWD2|KIA	0.285	3.298
NM_0013	chr8	27459761	27479296	-	4008	1	CHRNA2|-	2.139	1.651
NR_10415	chr11	1.13E+08	1.13E+08	-	2062	1	LOC38781	0	0.007
NM_0037	chr11	66563464	66568576	-	1999	1	CTSF|CATS	13.01	10.278
NM_0011	chr10	1.3E+08	1.3E+08	+	1338	1	GLRX3|GL	27.907	30.189
NR_03017	chr10	1.33E+08	1.33E+08	-	109	1	MIR202|M	0	0
NM_0013	chr17	75205450	75235759	+	2249	1	NUP85|FR	17.509	16.955

NM_0028:	chr11	236808	252984	+	1742	1	PSMD13|H	99.047	100.251
NM_0012(	chr11	85855101	85920020	+	2251	1	CCDC83|C	0	0
NR_02986	chr19	53787890	53787956	+	66	1	MIR372|M	0	0
NR_00158	chr1	2.2E+08	2.2E+08	-	1536	1	AURKAPS1	0.907	0.785
NR_03996	chr4	1.48E+08	1.48E+08	+	73	1	MIR4799|-	0	0
NM_0322:	chr11	65616312	65637439	+	6529	1	PCNX3|PC	12.666	13.307
NM_0000:	chr20	44619519	44651758	-	1544	1	ADA|-|-|2	8.309	4.808
NM_0011:	chr9	1.3E+08	1.3E+08	+	4850	1	NCS1|FLUI	58.167	84.187
NM_1788:	chr3	1.67E+08	1.68E+08	-	2576	1	WDR49|-|	0.571	0.346
NR_13552	chr21	32958888	32960564	+	572	1	LOC10272	0	0.216
NM_0012(	chr16	4340251	4416961	-	3625	2	CORO7-PA	0	0
NR_02805	chr7	75507747	75528123	-	1492	1	PMS2P3|P	2.143	1.882
NR_00259	chr16	21517909	21520444	-	2535	1	SLC7A5P2|	0.209	0.286
NM_0021	chr2	1.76E+08	1.76E+08	+	1800	1	HOXD10|H	0.007	0.015
NM_0794:	chr12	56158261	56161582	+	756	1	MYL6|ESM	1472.261	1037.35
NR_12587	chr6	1.7E+08	1.7E+08	+	527	1	LOC10272	0	0
NM_0011!	chr7	290170	291488	+	698	1	WI2-2373I	0	0.039
NM_0010(	chr1	40247901	40251693	+	737	1	TMCO2|dJ	0.07	0
NM_0011	chr15	51723064	51751453	-	1097	1	LYSMD2|-	2.069	2.672
NR_00283	chr1	2.29E+08	2.29E+08	+	2470	1	DUSP5P1|	1.462	2.202
NR_13096	chr19	49850735	49860744	+	1471	1	PTOV1|AC	269.974	231.248
NR_03334	chr11	83041464	83041598	-	134	1	SNORA70E	0	0
NM_0010(	chr1	1.62E+08	1.62E+08	+	4122	1	C1orf226|	7.638	12.684
NR_13525	chr14_KI2	20259	22044	-	1382	2	LOC10192!	0	0
NM_0011	chr3	25718944	25790039	-	2515	1	NGLY1|CD	7.716	9.847
NM_1825(	chr1	1.62E+08	1.62E+08	-	1168	1	SPATA46|(	1.523	3.037
NM_0207:	chr16	23061407	23149270	-	10681	1	USP31|-|-	2.374	3.206
NM_1524	chr14	80496478	80939540	-	4431	1	CEP128|C:	0.576	0.76
NM_0221:	chr2	2.42E+08	2.42E+08	+	1364.5	2	GAL3ST2|(	0	0
NM_0045!	chr19	5823802	5828324	+	1105	1	NRTN|NTN	1.669	2.194
NR_03022	chrX	1.35E+08	1.35E+08	-	70	1	MIR503|M	0	0
NM_0123(	chr9	1.23E+08	1.23E+08	-	934	1	OR1N1|OF	0	0
NR_13485	chr3	58607080	58634440	+	817	1	FAM3D-AS	0.032	0.151
NM_0013:	chr6	77653054	77927042	+	4900	1	MEI4|-|-|(	0.005	0
NR_03413	chr1	1.82E+08	1.82E+08	+	734	1	LINC00272	0	0
NM_1526(	chr1	2.26E+08	2.26E+08	-	4000	1	SDE2|C1or	4.906	4.208
NR_04982	chr19	35345513	35345627	+	114	1	MIR5196|r	0	0
NM_0013(	chr17_GL0	952322	1003890	+	2358.3	3	CRHR1|CR	0.011	0.006
NM_0010:	chr14	90540588	90816417	-	3412	1	TTC7B|TT(	15.728	14.757
NR_13548	chr17	47891230	47895816	-	522	1	LOC10272	12.004	5.799
NM_0012!	chr9	1.36E+08	1.36E+08	-	3584	1	CCDC187|	0.018	0.008
NR_12575	chr4	1.17E+08	1.18E+08	+	425	1	LINC01378	0	0
NR_00394	chr6_GL0	2311782	2317841	+	2145.4	8	HCG22|PB	0.024	0.038
NM_0005!	chr16	176651	177522	+	601	1	HBA1|HBA	0.172	0
NM_0147(	chr12	1.23E+08	1.23E+08	+	6899	1	KNTC1|RO	1.925	2.17
NM_0041(	chr18	54828477	54895516	+	6995	1	RAB27B|C	0.343	0.537
NM_0010(	chr1	33306766	33321098	-	1017	1	A3GALT2|,	0.025	0.175
NR_03648	chr16	89711856	89718165	+	1749	1	VPS9D1-AS	6.229	5.944
NM_0011:	chr12	1.21E+08	1.21E+08	-	2345	1	ANAPC5|A	48.946	58.526
NR_13547	chr15	58768072	58770974	-	1394	1	LOC10192:	0.777	0.57

NR_11068	chr1	1.53E+08	1.53E+08	+	2251	1	LOC10192	0	0
NR_10702	chr3	96359964	96360039	+	75	1	MIR8060|I	0	0
NM_0037	chr5	1.73E+08	1.73E+08	-	5337	1	STC2|STC-	7.1	1.999
NM_0181	chr4	40056904	40158252	+	9646	1	N4BP2|B3	1.355	2.397
NM_0140	chr17	29573469	29589592	-	3711	1	GIT1|-|-|1	49.555	64.499
NM_0178	chr2	96250208	96266013	-	4587	1	TMEM127	22.04	27.875
NR_13275	chr4	82895853	82895928	-	75	1	SNORD143	0.688	0.183
NM_0013	chr19	39433137	39436020	-	644	1	RPS16|S16	537.262	419.992
NR_03605	chr17	37033745	37033813	+	68	2	MIR2909|	0	0
NM_0011	chr16	30092316	30096216	-	933	1	YPEL3|-|-|	45.079	45.965
NR_12600	chr11	1.12E+08	1.12E+08	+	2745	1	LOC28314	0.705	0.883
NR_11074	chr19	23054823	23061265	-	1830	1	LINC01858	0.014	0
NM_1992	chr13	52064764	52129092	-	2900	1	NEK5|-|-|	0.089	0.052
NR_12580	chr1	1.87E+08	1.87E+08	+	792	1	PACERR|P	0	0
NR_11079	chr1	30013952	30037612	-	1512	1	LINC01648	0	0
NR_03747	chr9	91636251	91636361	+	110	1	MIR3910-1	0	0
NM_0164	chr20	44195941	44210906	-	2236	1	OSER1|C2	23.163	26.726
NR_03058	chrX	1.46E+08	1.46E+08	-	76	1	MIR890|M	0	0
NM_0149	chr19	14147737	14206185	-	7817	1	ADGRL1|C	43.282	56.39
NM_0013	chr7	1.02E+08	1.02E+08	+	2262	1	LRWD1|CE	33.231	27.497
NM_0154	chr11	57668002	57701187	+	4546	1	ZDHHC5|D	17.159	17.71
NR_03972	chr14	73758747	73758819	+	72	1	MIR4505|	0	0.19
NM_1735	chr2	1.65E+08	1.65E+08	-	1712	1	SLC38A11|	0.008	0
NM_0013	chr4	47450789	47463730	-	1474	1	COMMD8|	2.598	2.435
NM_0325	chr10	29289061	29311229	+	805	1	LYZL1|KAA	0	0
NM_0012	chr1	1.61E+08	1.61E+08	-	1700	1	USF1|FCHI	68.935	84.76
NM_1523	chr14	51651858	51730726	+	4811	1	FRMD6|C1	0.367	1.458
NM_0012	chr8	91209494	91398154	+	5122	1	SLC26A7|S	0.04	0.072
NM_0245	chr3	1.12E+08	1.12E+08	-	2199	1	ZBED2|-|-	0	0
NR_00349	chr15	25264527	25264579	+	52	1	SNORD115	0	0.527
NM_0182	chr1	2.12E+08	2.12E+08	-	1952	1	TMEM206	22.603	43.014
NM_0207	chr4	1.41E+08	1.41E+08	-	4652	1	RNF150|-|	6.034	2.794
NM_0062	chr19	43543312	43575578	-	2083	1	XRCC1|RC	46.399	31.977
NR_13092	chr5	87318415	87323628	-	5213	1	LOC64428	0.173	0.244
NM_0010	chr11	55343201	55344187	+	985	1	OR4A16|C	0	0
NM_0003	chr6	49605177	49636874	-	1916	1	RHAG|CD2	0.027	0.1
NR_03617	chrX	1.47E+08	1.47E+08	-	79	1	MIR514B|	0	0
NR_04981	chr3	1.25E+08	1.25E+08	-	87	1	MIR5092|	0	0
NM_0013	chr15	59659146	59688926	-	6108	1	BNIP2|BNI	3.095	4.2
NR_10500	chr5	72557077	72571742	+	661	1	LOC10250	0	0
NM_0011	chr5	55112971	55173177	-	2845	1	CDC20B|G	0.258	0
NR_10991	chr5	5132085	5140054	-	1580	1	CTD-2297I	0.073	0.269
NR_03023	chrX	1.4E+08	1.4E+08	-	83	1	MIR505|M	0	0
NM_0062	chr11	6610817	6612244	-	810	1	TAF10|TAF	54.853	48.954
NR_02834	chr7	38685346	38687089	-	1645	1	FAM183BF	0.016	0.025
NM_0011	chr1	2586750	2591469	+	2656	2	FAM213B|	0	0
NM_0013	chr17	47522957	47623276	+	4108	1	NPEPPS|A	38.611	44.812
NR_11094	chr16	30875460	30895218	+	1572	1	MIR762HG	14.919	11.92
NR_13426	chr5	1.59E+08	1.59E+08	+	570	1	LINC01932	0	0
NR_11095	chr4	38420662	38523180	-	2051	1	LINC01258	0.013	0.013

NM_1383!	chr17	47837681	47841333	-	1558	1	SCRN2|Se:	13.613	9.46
NM_0011	chr6	33771213	33786942	-	2349	1	LEMD2|CT	28.647	30.035
NR_11004	chr12	1.24E+08	1.24E+08	-	2313	1	LOC10192	0.195	0.225
NM_0026	chr20	41137521	41175717	+	5171	1	PLCG1|NC	70.319	82.357
NM_0013(	chr15	77420651	77485603	+	4030	1	HMG20A|	21.253	26.378
NR_12582	chr8	37717579	37733493	+	962	1	LOC10192!	0	0
NM_1383	chr5	14664645	14699850	+	8033	1	OTULIN|AI	1.679	2.041
NM_0012	chr1	15526813	15571733	+	5868	1	DNAJC16|	3.738	4.335
NR_02664	chr11	1665597	1667457	+	1050.5	6	FAM99B|-	0	0
NR_13198	chr17	65457541	65458408	+	274	1	CRAT40|-|	0	0
NM_1387	chr11	1.3E+08	1.3E+08	+	1772	1	TMEM45B	0.036	0.008
NR_05197	chr13	1.07E+08	1.07E+08	+	1963	1	LINC00551	0.066	0.035
NM_0012	chr1	1.46E+08	1.46E+08	-	3079	1	ANKRD35|	0.31	0.4
NM_0322	chr14	99397746	99480889	-	2873	1	SETD3|C1	23.741	26.808
NR_03615	chr19	47226944	47227019	-	75	1	MIR3191|	0.86	0.365
NM_0010	chr14	66507407	67181808	+	4179	1	GPHN|GEF	10.499	10.573
NM_0061(	chr20	45469754	45481532	+	561	1	WFDC2|EI	0.712	0.562
NM_0013	chr4	1.56E+08	1.56E+08	-	2923	2	CTSO|CTS(	0	0
NR_02967	chr12	97563812	97563911	+	99	1	MIR135A2	0	0
NM_0010	chr1	2.24E+08	2.24E+08	-	4637	1	TP53BP2|	18.263	20.958
NM_0035	chr1	1.5E+08	1.5E+08	+	393	2	HIST2H4A|	0	0
NM_0219	chr10	13100082	13138276	+	3360	1	OPTN|ALS	29.498	22.453
NM_0012	chr12	11307081	11310435	-	704	2	PRB4|Po|	0	0
NM_0029	chr3	1.34E+08	1.34E+08	-	2916	1	RYK|D3S3	9.14	14.449
NM_0011	chr12	1.1E+08	1.1E+08	+	3081	1	TCHP|TpN	5.005	4.037
NM_0012	chr1	1.79E+08	1.79E+08	+	7492	1	RALGPS2|	2.926	2.764
NM_0152	chr17	6070614	6124427	+	5810	1	WSCD1|-|	32.626	39.005
NM_0147	chr10	42782506	42834937	+	8023	1	BMS1|ACC	4.329	3.718
NM_0010	chr19	51345155	51354842	-	1294	1	ETFB|FP58	311.935	275.38
NM_0015	chr1	85580761	85583965	+	2286	1	CYR61|CCI	1.404	9.784
NM_0010(	chr1	65420652	65630412	+	3047	1	LEPR|CD2	0.948	1.079
NM_0013	chr8	22138014	22141951	-	1571	1	REEP4|C8	4.9	3.836
NM_0012	chr1	1.17E+08	1.17E+08	+	3322	1	CD101|EW	0.353	1.835
NM_1749	chr11	695591	704131	+	1619	1	TMEM80|	12.28	9.382
NM_0029	chr16	57404767	57416062	+	609	1	CCL17|A-1	0.106	0.09
NM_0202	chr4	77862651	77952790	+	1348	1	MRPL1|BN	10.741	11.655
NM_1984	chrX	85003892	85093317	+	6456	1	APOOL|CX	2.419	2.037
NM_0012	chr5	1.33E+08	1.33E+08	+	2634	1	CCNI2|-|-|	2.178	2.522
NM_0009(	chr16	69709401	69726630	-	2583	1	NQO1|DH	24.025	3.781
NM_0329	chr1	2.07E+08	2.07E+08	+	2968	1	MAPKAPK	35.188	47.806
NM_0051	chr4	11398364	11428913	-	1961	1	HS3ST1|3(	1.433	3.332
NM_0015	chr11	1.12E+08	1.12E+08	+	837	1	HSPB2|HS	0.123	0.098
NR_13431	chr10	70939053	70955641	+	531	1	LOC10537	0	0
NR_02770	chr5	1.12E+08	1.12E+08	+	1398	1	EPB41L4A-	5.395	2.155
NR_03845	chr19	47487634	47515258	-	1504	1	NAPA|SNA	91.755	84.832
NM_0013(	chr7	1.01E+08	1.01E+08	-	2153	1	ACHE|ACE	28.997	29.964
NM_0011(	chr16	81053497	81077267	-	1905	1	C16orf46|	3.14	1.891
NM_0011	chr8	28767658	28889915	-	2511	1	INTS9|CPS	12.18	15.71
NR_02727	chr11	1.34E+08	1.34E+08	+	2296	1	LOC10012	1.196	1.277
NR_00330	chr15	25184306	25184387	+	81	1	SNORD115	0	0

NR_03838	chr9	1.37E+08	1.37E+08	-	1697	1	STPG3-AS1	0.532	0.129
NM_0011!	chr1	45620044	45624059	-	2208	1	CCDC17|-|	0.491	0.49
NR_13076	chr9	1.05E+08	1.05E+08	+	1828	1	NIPSNAP3I	0.19	0.21
NR_12872	chr16	17052	17119	-	67	4	MIR6859-4	0.385	0.204
NM_0012:	chr7	1.43E+08	1.43E+08	+	4026	1	CASP2|CA:	13.739	13.472
NM_0308!	chr22	36226209	36239643	-	2538	1	APOL2|AP	2.098	2.558
NR_13556	chr2	96527941	96532306	-	436	1	LOC10537.	0.325	0.44
NM_0040(	chr13	20187465	20192975	-	2328	1	GJB2|CX2(	0.022	0.018
NM_0033:	chr1	1.73E+08	1.73E+08	-	3469	1	TNFSF4|CI	0.13	0.32
NM_0319:	chr14	61995823	62101709	+	3129	1	SYT16|CHI	0.742	1.353
NR_04984	chr1	44545493	44545552	+	59	1	MIR5584|-	0	0
NM_0071	chr17_KI2	2769216	2783624	-	2591	2	PCGF2|ME	0	0
NM_0029	chr4	1.45E+08	1.45E+08	+	4174	1	ABCE1|AB	3.003	4.273
NM_0030(	chr10	96998038	97185926	-	7892	1	SLIT1|MEC	38.808	39.579
NR_13815	chr8	11111721	11201366	-	1181	1	XKR6|C8oi	1.332	1.369
NM_0000!	chr16	28477279	28492082	-	1860	1	CLN3|BTN	18.966	13.638
NM_0010!	chr1	1.46E+08	1.46E+08	+	1950	1	GPR89A|G	0.866	0.801
NM_0012!	chr17	78674047	78717032	-	3308	1	CYTH1|B2-	19.177	26.226
NM_0012!	chr2	76747724	77522433	-	3140	1	LRRTM4|-	5.469	4.144
NM_0010(	chr11	56176618	56177556	+	937	1	OR5J2|OR	0	0
NM_0010:	chr12	45215987	45432351	+	6086	1	ANO6|BDI	3.773	3.18
NR_02938	chr10	45854835	45972428	-	1435	1	PARGP1|-	1.42	1.642
NM_0000!	chr19	18782773	18791305	-	2431	1	COMP|EDI	0.058	0
NR_10980	chr9	1.16E+08	1.16E+08	-	386	1	LOC10192:	0	0
NR_00291	chr5	1.39E+08	1.39E+08	+	199	1	SNORA74A	0	0
NM_0318	chr2	2.1E+08	2.1E+08	+	5363	1	MAP2|MA	124.509	123.138
NR_12535	chr5	1.38E+08	1.38E+08	+	1687	1	WNT8A|W	0.046	0.065
NM_0013(	chr2	47160082	47176936	-	1221	1	CALM2|CA	437.565	503.155
NR_03024	chrX	50003148	50003238	+	90	1	MIR532|M	0	0
NR_00145	chr14	95532912	95534872	-	1328	1	SNHG10|C	5.058	4.115
NM_0321:	chr4	1.53E+08	1.53E+08	+	935	1	MND1|GA	2.303	1.567
NM_0013:	chr16	75596879	75623323	-	4758	1	ADAT1|HA	8.848	8.501
NM_0056	chr8	1.43E+08	1.43E+08	+	1018	1	PSCA|PRO	0.114	0.027
NM_0025:	chr11	1.08E+08	1.08E+08	-	5917	1	NPAT|E14	1.076	2.069
NM_0012!	chr11	86791059	86811231	+	3734	1	PRSS23|SI	5.963	7.153
NM_0180(	chr15	70654554	70763511	-	6836	1	UACA|NU(	0.183	0.238
NM_0010(	chr15	40087891	40106086	-	4384	1	BMF|-|-|1	15.229	16.459
NM_0011	chr11	1.12E+08	1.12E+08	-	1535	1	PLET1|C11	0	0.009
NM_0011(	chr16	3027867	3035541	-	1800	1	BICDL2|BI	0.029	0.076
NR_03614	chr16	83508346	83508408	+	62	1	MIR3182|i	0	0
NM_0196(	chr8	66667552	66681142	+	1880	1	C8orf44|-|	2.064	2.033
NM_0013(	chr17_KI2	1925499	1984134	-	2408	2	HNF1B|FJI	0.021	0.08
NM_0322	chr1	89633085	89719535	+	7202	1	LRRC8C|AI	0.589	0.493
NM_0010(	chr10	1.3E+08	1.3E+08	-	4394	1	EBF3|COE:	51.483	52.749
NR_04757	chr17	4163818	4263995	-	7385	1	ANKFY1|A	20.411	20.58
NM_0010(	chr12	45014756	45051099	-	2716	1	DBX2|-|-|	0.009	0.055
NM_0066:	chr2	85584408	85593388	+	660	1	VAMP5|-|	0.254	0.581
NM_0184:	chr17	31863171	31901710	-	2044	1	UTP6|C17	18.955	21.705
NR_00144	chr17	18422181	18425333	+	3152	1	KRT17P5|-	0	0.004
NM_0010	chr3	1.12E+08	1.12E+08	+	2491	1	TMPRSS7|	0.088	0.313

NR_10466	chr16	80567103	80572808	-	752	1	LINC01227	0	0
NM_0069	chr22	37972312	37984532	-	2856	1	SOX10|DC	0.009	0.096
NM_0250	chr1	84479237	84498350	+	1964	1	RPF1|BXD	11.981	12.93
NM_0309	chr7	1.01E+08	1.01E+08	+	4718	1	TRIM56|R	0.672	0.569
NM_0807	chr6	70861366	70957085	-	1919	1	B3GAT2|G	4.24	9.936
NM_0013	chr20	347905	354868	+	2122	1	NRSN2|C2	63.629	56.124
NM_0053	chr8	1.22E+08	1.22E+08	-	3270	1	HAS2|-|-|	0.146	0.796
NR_13614	chr10	2446253	2501462	-	848	1	LOC10537	0	0
NM_0245	chr2	2E+08	2E+08	+	1503	1	MAIP1|C2	8.313	7.837
NM_0013	chr19	48630560	48637550	-	1805	1	DBP|DABF	6.807	5.6
NM_0143	chr22	32387575	32412287	-	2052	1	RTCB|C22	58.724	64.08
NR_03794	chr20	58651253	58715844	+	4025	1	STX16-NPE	9.658	9.654
NM_0153	chr5	55307748	55425581	+	4175	1	SKIV2L2|D	8.017	9.15
NR_03169	chr22	41092513	41092566	+	53	1	MIR1281|	3.892	0.775
NR_02687	chr7	66376044	66400408	-	4419	1	LINC00174	1.567	1.444
NM_0182	chr1	2.01E+08	2.01E+08	+	4129	1	C1orf106|	1.346	1.742
NM_0012	chr6	39792383	39904877	+	6159	1	DAAM2|d.	0.375	7.145
NM_0010	chr4	68646597	68670776	-	2251	2	UGT2B15|	0	0
NR_03749	chr10	57304479	57304559	-	80	1	MIR3924|	0	0
NM_0019	chr4	1.1E+08	1.11E+08	+	4973	1	ENPEP|AP	0.016	0.019
NM_0010	chr6	10396683	10419564	-	3141	1	TFAP2A|A	14.482	21.324
NR_12541	chr8	1.21E+08	1.21E+08	+	1968	1	LOC10192	0	0
NM_0012	chr22_KI2	270308	278486	-	849	1	GSTT1|-|-	12.499	13.568
NM_0011	chr11	790475	798269	-	2616	1	SLC25A22|	25.087	23.064
NR_03029	chr3	50173326	50173419	+	93	1	MIR566|M	0	0
NR_03167	chr8	1E+08	1E+08	-	102	1	MIR1273A	0	0
NM_0012	chr7	86876906	87040234	-	6574	1	KIAA1324L	12.703	8.33
NM_0010	chr5	1.46E+08	1.46E+08	-	743	1	GRXCR2|D	0	0.018
NM_0012	chr19	40359642	40378490	+	2107	1	PLD3|AD1	304.218	241.937
NM_0177	chr19	55080392	55087923	+	1204.9	10	EPS8L1|DF	22.525	11.925
NR_10981	chr3	1.49E+08	1.49E+08	+	748	1	TM4SF1-A	0	0
NM_0227	chr1	2.31E+08	2.31E+08	+	1465	1	ARV1|EIEE	13.263	15.137
NM_1826	chr4	1.7E+08	1.7E+08	-	2092	1	AADAT|KA	10.194	6.901
NM_0188	chr1	67701466	67833472	-	4403	1	GNG12|-|	7.321	9.426
NM_0218	chr11	405716	417397	-	1627	1	SIGIRR|IL-	0.658	0.219
NM_0160	chr1	2.47E+08	2.47E+08	+	2461	1	SCCPDH|C	26.327	23.515
NM_0017	chr20	57168753	57266651	-	4022	1	BMP7|OP-	6.251	11.129
NM_0049	chr1	32292107	32333623	+	2075	1	HDAC1|GC	17.933	21.183
NR_10686	chr2	2.18E+08	2.18E+08	+	69	1	MIR6810|	0.934	0.993
NR_07724	chr1	89289258	89290362	-	1104	1	LOC72993	0.199	0.633
NM_0010	chr9	1.28E+08	1.28E+08	-	1112	1	PTRH1|C9	5.577	5.062
NM_0011	chr4	1.1E+08	1.1E+08	+	5549	1	EGF|HOM	0.723	0.903
NR_02696	chr4	1.09E+08	1.09E+08	-	3769	1	RPL34-AS1	0.209	0.269
NM_0042	chr10	1.03E+08	1.04E+08	+	4307	1	NEURL1|N	6.825	5.387
NM_1785	chr17	79731103	79742222	+	1851	1	ENPP7|ALI	0	0
NM_0314	chr19	45251258	45305283	+	5223	1	MARK4|M	31.188	38.587
NR_02683	chr10	89691300	89697928	-	3660	1	FLJ37201|	0.088	0.138
NM_0143	chr7	1.5E+08	1.5E+08	+	2873	1	REPIN1|AF	37.397	35.78
NM_0069	chr9	21367372	21368076	-	702	1	IFNA13|-|	0	0
NR_02775	chr3	42692663	42701521	-	1737	1	HHATL|C3	0.015	0.008

NM_0186‹	chr11	1.34E+08	1.34E+08	-	3515	1	B3GAT1|C	74.808	92.462
NM_0057‹	chr13	49997007	50018467	+	6750	1	TRIM13|C	8.54	10.738
NR_02416	chr1	1.69E+08	1.69E+08	+	952	1	LINC0062€	0	0
NM_1942‹	chr14	76826382	76870302	+	1718	1	LRRC74A|(	0	0.008
NR_13489	chr6	42960754	42963880	+	928	1	GNMT|HE	1.709	2.347
NM_0150‹	chr13	32586427	32778020	+	7461	1	PDS5B|AP	5.188	6.447
NM_0210‹	chr9	21186619	21187599	-	978	1	IFNA4|IFN	0	0
NR_02828	chr14	95651178	95673452	+	3299	1	TCL6|TNG	0.012	0
NM_0153‹	chr6	43511827	43516990	-	1565	1	YIPF3|C6o	135.13	112.456
NR_00302	chr11	93732004	93732133	-	129	1	SNORA1|A	0	0.425
NM_0013‹	chr2	1.3E+08	1.3E+08	+	788	1	MZT2B|FA	62.798	42.603
NM_0528‹	chr11	1.34E+08	1.34E+08	+	3723	1	VPS26B|P	61.957	93.23
NM_0010‹	chr11	62790315	62792014	-	933	1	TMEM223	12.064	8.676
NR_02724	chr12	1.04E+08	1.04E+08	-	4500	1	TTC41P|G	0.175	0.107
NR_10678	chr1	1296110	1296170	-	60	1	MIR6726|l	7.951	10.044
NM_1452‹	chrX	1.11E+08	1.11E+08	-	3929	1	CHRDL1|C	9.392	16.075
NR_13568	chr15	41892777	41898815	+	2999	1	LOC10537‹	0.486	0.452
NM_0013‹	chr1	9036946	9072227	-	2368	1	SLC2A5|G	0.098	0.174
NR_11030	chr17	76624756	76643838	-	2465	1	ST6GALNA	0.01	0
NM_0012‹	chr4	3484544	3494482	+	2258	1	DOK7|C4o	0.56	0.115
NM_0030‹	chr10	79937740	79949105	-	1273	1	SFTPD|CO	0.324	0.366
NM_0012‹	chr17	7235028	7238930	-	1914	1	PHF23|hJL	65.909	71.047
NR_03768	chr12	94262521	94307675	+	3878	1	PLXNC1|C	8.515	5.397
NM_0010‹	chr16	722582	726473	-	1595	1	CCDC78|C	1.439	1.237
NR_10684	chr17	45114367	45114433	-	66	1	MIR6784|l	0	0.415
NM_0020‹	chr6	42183284	42194956	-	2264	1	GUCA1B|G	2.511	3.805
NR_03766	chr5	40825263	40829142	-	3879	1	LOC10050‹	29.101	31.61
NR_04502	chr12	51048299	51060424	+	1641	1	LETMD1|1	36.033	29.205
NM_1303‹	chrX	64224192	64230631	-	1288	1	ASB12|-|-	0.791	1.351
NM_0013‹	chr10	12165328	12196144	-	3430	1	NUDT5|YS	14.781	12.99
NM_0045‹	chr18	57600661	57621945	-	2850	1	NARS|ASN	48.882	47.247
NM_2034‹	chr1	39738845	39763914	+	1107	1	PPIE|CYP-‹	30.294	30.091
NR_10688	chr3	48549961	48550021	-	60	1	MIR6823|l	3.438	3.196
NM_0011‹	chr3	28331689	28349127	-	1436	1	AZI2|AZ2|	17.535	14.355
NM_1988‹	chr22	43528744	43812337	-	4539	1	EFCAB6|D	0.744	0.25
NM_0012‹	chr3	1.2E+08	1.2E+08	+	3257	1	ADPRH|AF	0.083	0.046
NR_12586	chr6	1.66E+08	1.66E+08	+	1540	2	LOC10192‹	0	0
NR_03966	chr5	1.55E+08	1.55E+08	+	82	1	MIR378H|	0	0
NR_02654	chr6	65787879	65789483	+	1604	2	SLC25A51f	0	0
NM_0012‹	chr10	46286997	46330207	+	2266	1	ANTXRL|-|	0.011	0
NM_1452‹	chr10	99532933	99536523	+	2092	1	NKX2-3|C	0.012	0.065
NM_0230‹	chr2	1.1E+08	1.1E+08	+	4651	1	SOWAHC|	3.202	3.834
NR_11031	chr19	3933103	3942416	+	702	1	NMRK2|IT	0.514	0.546
NM_0808‹	chrX	1.03E+08	1.04E+08	-	1341	1	RAB40A|R	1.202	0.96
NM_0010‹	chr17	28364268	28365151	-	569	1	SEBOX|OG	0.045	0.024
NM_0011‹	chr7	95772506	96098424	+	2976	1	DYNC1I1|[	5.277	9.928
NM_0013‹	chr3	4303304	4317265	+	1273	1	SETMAR|N	12.387	14.418
NM_0013‹	chr7	1.05E+08	1.06E+08	-	3283	1	PUS7|-|-|	1.516	1.961
NM_0243‹	chr19	13882348	13906165	-	2486	1	C19orf57|	8.049	9.174
NM_0061‹	chr22	39399754	39431882	+	3211	1	TAB1|3'-T	23.028	25.5

NR_13297	chr11	1949331	1949476	+	145	2	SNORD131	0	0
NR_03716	chr8	47189307	47192816	+	3509	1	LOC10028	0.125	0.195
NM_1449	chr21	44497892	44711580	-	3953	1	TSPEAR|C2	0.31	0.149
NR_02724	chr1	2.38E+08	2.38E+08	+	3113	1	LOC10013	0.153	0.202
NR_01534	chr7	5072060	5073223	+	576	1	RBAKDN|-	0.179	0.666
NM_0011	chr16	71526251	71538590	+	2172	1	CHST4|GS	0	0.006
NM_0251	chr12	88049013	88142216	-	7899	1	CEP290|3	0.849	0.975
NR_03036	chr19	14529543	14529640	+	97	1	MIR639|M	1.329	1.271
NM_0807	chr20	45857581	45879130	+	2813	1	ZSWIM3|C	7.361	6.335
NM_0219	chr17	41812710	41823217	+	2871	1	FKBP10|B	30.148	21.411
NR_10805	chr10	61016275	61026417	+	691	1	LINC00845	0.187	0
NR_00352	chr10	27245853	27252306	-	6453	1	LRRC37A6	0.076	0.183
NM_0013	chr11	1.14E+08	1.14E+08	-	4337	1	USP28|-|-	6.038	7.614
NM_0011	chr3	77098012	77649963	+	7959	1	ROBO2|SA	15.077	9.035
NM_2125	chr11	1.26E+08	1.26E+08	-	1633	1	PATE2|C1	0	0.017
NM_0010	chr20	37289648	37317260	+	1193	1	MANBAL|-	50.828	57.612
NM_1979	chr5	1.81E+08	1.81E+08	+	2430	1	BTNL3|BT	0	0
NR_02391	chr9	33673504	33677420	-	3916	1	PTENP1|P	0.826	1.438
NR_12652	chr5	61637781	61730997	+	1331	1	C5orf64|-|	0.048	0.185
NM_0010	chr19	49496365	49499712	+	639	1	RPS11|S11	1996.31	1656.246
NM_1391	chr7	1.23E+08	1.23E+08	-	1440	1	RNF133|-|	0.027	0.038
NM_0121	chr13	77005254	77027196	-	3542	1	FBXL3|FBL	2.989	4.269
NM_0002	chr18	23531499	23586617	-	4787	1	NPC1|NPC	12.6	10.095
NM_0032	chr12	49741510	49764934	+	2872	1	TMBIM6|E	233.676	265.26
NM_0133	chr22	23752743	23754443	-	581	1	VPREB3|8	0.577	0.094
NM_0153	chr9	1.37E+08	1.37E+08	-	1517	1	NPDC1|CA	65.198	67.844
NM_0053	chrX	1.53E+08	1.53E+08	-	1717	1	MAGEA6|	0	0
NM_0043	chr1	84553121	84574480	-	3092	1	CTBS|CTB	1.072	0.913
NR_11097	chr19	57696275	57709211	-	3671	1	ZNF154|pl	0.386	4.302
NM_2034	chr15	50500562	50546705	-	1383	1	USP50|-|-	0.42	0.327
NM_0012	chr2	2.41E+08	2.41E+08	-	4447	1	PASK|PAS	7.095	5.858
NM_1827	chr12	84859488	84912829	-	4818	1	SLC6A15|	6.383	8.89
NM_0010	chr7	27740095	27829767	+	3345	1	TAX1BP1|	18.298	15.495
NR_13236	chr19	48061371	48062255	+	519	1	PLA2G4C-	1.143	1.583
NR_04041	chr20	64290385	64303354	+	720	1	LINC00266	0.018	0
NM_0027	chr5	1.34E+08	1.34E+08	-	2634	1	PPP2CA|P	75.09	87.455
NM_0012	chr19	41226755	41261767	+	4101	1	AXL|ARK|	2.672	3.597
NM_0010	chr11	62671654	62673690	+	1946	1	UQCC3|C1	9.243	6.673
NM_0335	chr8	97273486	97277948	-	4460	1	TSPYL5|-|	0	0.006
NM_0047	chr17	29073521	29075609	-	2086	1	TIAF1|MA	30.96	33.166
NR_11937	chr20	35558737	35607562	-	6674	1	FER1L4|C2	0.61	0.289
NM_0062	chr19	49635292	49640143	-	978	1	RRAS|-|-|	5.814	5.154
NM_0011	chr19	44025342	44033109	+	1644	1	ZNF222|-|	4.659	4.566
NR_02742	chrUn_GL	46845	55049	-	8204	1	LOC38983	0	0
NM_0014	chr5	1.38E+08	1.38E+08	-	1921	1	GFRA3|GD	0.054	0.15
NM_0011	chr1	32651358	32686211	+	7829	1	RBBP4|NU	26.078	26.159
NR_03648	chr11	9758293	9811319	+	2705	1	SBF2-AS1|	0.906	1.129
NM_0006	chr19	41330323	41353883	-	2732	1	TGFB1|CE	1.501	4.402
NM_1384	chr19	17455425	17460916	-	904	1	NXNL1|RD	0	0.03
NR_04661	chr3	52823935	52825314	+	392	1	ITIH4-AS1|	0.625	0.384

NM_0012	chr1	1.55E+08	1.55E+08	-	2077	2	CLK2|-|-|1	0	0.007
NM_0011	chr13	51341032	51362103	+	1432	1	SERPINE3|	0.234	0.201
NM_0164	chr9	97904489	97922570	-	1640	1	TRMO|C9c	4.67	4.268
NM_0010	chr20	187853	189681	-	369	1	DEFB128|	0	0
NM_0010	chr12	1.04E+08	1.04E+08	-	7198	1	NT5DC3|T	0.82	1.174
NR_12052	chr11	32035979	32041318	-	819	1	LOC10050	0.016	0.05
NM_1991	chr13	1.13E+08	1.13E+08	-	1868	1	ADPRHL1|	5.66	3.864
NM_0011	chr2	1.15E+08	1.16E+08	+	4801	1	DPP10|DP	5.468	5.227
NR_03608	chr3	10330229	10330285	+	56	1	MIR378B|	0	0
NM_0011	chr6	28225251	28233486	+	1640	1	ZSCAN9|P	35.473	31.01
NR_07312	chr4	17810813	17844864	+	4622	1	NCAPG|CA	1.515	3.046
NM_1784	chr1	1.53E+08	1.53E+08	-	543	1	LCE3E|LEP	0	0
NM_1781	chr3	1.38E+08	1.38E+08	-	2119	1	NME9|NM	0.955	0.84
NM_0011	chrX	47142216	47145210	-	1089	1	NDUFB11|	135.518	141.536
NM_0038	chr17	10300866	10373005	-	5949	1	MYH13|M	0	0.002
NR_13296	chr18	37466339	37466504	-	165	1	SNORA111	0	0
NR_00283	chr7	35081287	35186162	-	2792	1	DPY19L2P	0.06	0.078
NR_11087	chr4	86119806	86219926	+	953	1	LOC10192	0.027	0.014
NM_0011	chr19	51331541	51342124	-	3663	1	VSIG10L|-	2.985	3.863
NM_0211	chr3	1.9E+08	1.9E+08	-	3446	1	CLDN1|CL	2.555	0.457
NR_03039	chr14	1.01E+08	1.01E+08	+	80	1	MIR654|M	0	0
NM_1758	chr5	1.3E+08	1.3E+08	+	3487	1	CHSY3|CH	0.314	0.436
NM_0026	chr6	41736711	41747401	-	1364	1	PGC|PEPC	0.095	0.08
NR_10384	chr3	16536318	16541373	+	1364	1	LINC0069C	0	0
NM_0528	chr19	13118288	13141147	+	4492	1	NACC1|BE	15.956	19.268
NM_0239	chr12	10806051	10806980	-	928	3	TAS2R8|T2	0	0
NM_0196	chr22	24181476	24189106	+	3166	1	SUSD2|BK	1.348	0.424
NM_0058	chr2	2.38E+08	2.38E+08	+	908	1	RAMP1|-|	0.185	0.272
NR_10677	chr6	1390314	1390411	-	97	1	MIR6720|	0	0.424
NM_0140	chr6_GL0C	2097433	2106121	+	1525	7	MRPS18B|	0	0
NM_0012	chr8	27809621	27838095	-	2082	1	PBK|CT84	1.678	2.882
NR_03985	chr14	1.04E+08	1.04E+08	-	85	1	MIR203B|	0	0
NM_0062	chr8	96226676	96235634	-	4813	1	UQCRB|M	25.524	22.297
NM_1333	chr17	45111640	45132533	-	3452	1	PLCD3|PL	6.772	10.36
NM_1383	chr17	44070730	44076344	+	1570	1	G6PC3|SC	63.465	46.613
NR_10686	chr19	58334688	58334751	+	63	1	MIR6806|	1.637	2.392
NM_2016	chr1	2.05E+08	2.05E+08	-	3192	1	LRRN2|FIG	14.865	17.559
NM_0012	chr16	721142	722590	+	817	1	FAM173A|	19.948	16.614
NM_0011	chr17	50165985	50175932	+	1071	1	SGCA|50D	0.12	0.077
NM_0051	chr22	50578958	50582999	-	1616	1	CHKB|CHE	23.361	16.706
NR_10465	chr16	67517706	67528745	-	1107	1	LOC10050	1.106	0.792
NR_14447	chr10	15015072	15088776	-	989	1	ACBD7-DC	0.156	0.319
NR_02963	chr2	55982967	55983076	-	109	1	MIR217|M	0	0
NM_0220	chr1	1.51E+08	1.51E+08	-	2491	1	CERS2|L3|	30.564	21.879
NM_0026	chr19	1086579	1095392	-	2856	1	POLR2E|R	50.418	42.64
NM_0009	chr7	75915102	75986855	+	2481	1	POR|CPR|	66.479	65.427
NM_0210	chr9	21201469	21202205	-	734	1	IFNA7|IFN	0	0
NM_0011	chr4	1.06E+08	1.06E+08	-	651	1	GIMD1|-|-	0	0
NM_0333	chr19	53289601	53291622	-	2019	1	BIRC8|ILP-	0	0
NM_0152	chr6	34889261	35091413	+	6325	1	ANKS1A|A	31.152	26.573

NR_04563	chr2	74147981	74149054	+	657	1	BOLA3-AS1	10.264	9.465
NM_0002	chr16	67939884	67944112	-	1346	1	LCAT|-|-|1	1.054	1.781
NM_0010	chr1	1.52E+08	1.52E+08	-	2662	1	LINGO4|D.	0.01	0.01
NR_10672	chr17	64780759	64780824	+	65	1	MIR6080|	1.587	1.686
NR_12058	chr11	66435075	66438848	-	1450	1	MRPL11|C	22.693	19.809
NM_0010	chr1	1.09E+08	1.09E+08	+	3771	1	SYPL2|MG	0.12	0.171
NM_2136	chr3	68004216	68545620	+	2002	1	FAM19A1|	0.638	0.691
NR_02745	chr20	36045618	36050960	-	5342	1	NORAD|LI	35.007	32.561
NR_04700	chr13	91127613	91211698	-	563	1	LINC0037	0	0
NM_0013	chr10	14518557	14538542	-	3102	1	FAM107B|	27.749	33.827
NM_0013	chr17	75588058	75626847	+	9475	1	MYO15B|	0.361	0.369
NM_0018	chr9	1.13E+08	1.13E+08	+	1768	1	SLC31A2|	1.83	1.79
NM_0011	chr12	46764761	46825997	-	3773	1	SLC38A4|	0.072	0.015
NM_0012	chr17	41301826	41302851	-	1024	3	KRTAP29-1	0	0
NR_03967	chr6	1.41E+08	1.41E+08	+	69	1	MIR4465|	0	0
NM_0025	chr19	45933734	45973399	-	7650	1	NOVA2|AN	59.865	52.584
NR_03031	chr7	5495819	5495917	-	98	1	MIR589|M	0.132	0
NM_0219	chr6	27824125	27824480	+	353	1	HIST1H4J|	0.438	0.698
NR_03167	chr15	64762387	64762515	-	128	1	MIR1272|	0	0
NM_0013	chr19	4292228	4302431	-	900	1	TMIGD2|C	0.158	0.167
NM_0243	chr5	3596054	3601403	+	1852	1	IRX1|IRX-5	33.654	19.421
NR_03059	chrX	1.46E+08	1.46E+08	-	76	1	MIR888|M	0	0
NM_0013	chr13	67224507	67230336	-	4622	1	PCDH9|-|-	9.312	3.743
NM_0012	chr6	24402908	24423479	+	1777	1	MRS2|HPT	10.115	9.542
NR_12645	chr20	31440519	31450257	+	531	1	DEFB123|	0	0
NR_13555	chr3	1.87E+08	1.87E+08	+	1971	1	LOC10192	0.098	0.215
NR_10402	chr10	1.05E+08	1.05E+08	-	1292	1	SORCS3-AS	0	0
NM_0013	chr16	75149051	75172234	+	3395	1	ZFP1|ZNF4	6.954	8.311
NR_03615	chr19	18282077	18282161	+	84	1	MIR3188|	0	0.163
NR_13296	chr18	58600620	58600750	+	130	1	SNORA108	0	0
NM_1780	chr22	31618491	31638503	-	2578	1	PISD|DJ85	15.229	10.839
NM_0011	chr10	97737121	97760907	+	2659	1	ZFYVE27|F	24.667	34.58
NM_0011	chr3	46612435	46626543	+	1821	1	LOC10013	0	0.008
NM_0011	chr12	1.33E+08	1.33E+08	-	866	1	LRCOL1|-|	0.015	0.079
NM_0009	chr3	12834945	12841582	-	1645	1	RPL32|L32	274.447	266.13
NM_0011	chr9	92495312	92536092	-	3212	1	ECM2|-|-|	0.132	0.098
NR_00304	chr1	75787072	75787149	+	77	1	SNORD45C	0	0.178
NM_0011	chr16	69950705	69964347	+	2200	1	CLEC18A|	0.164	0.068
NR_03618	chr10	48985512	48985587	-	75	1	MIR4294|	0	0
NM_0058	chr16	31108294	31112791	+	2114	1	BCKDK|BC	27.08	24.031
NR_13559	chrX	48802663	48824982	+	3828	1	HDAC6|CP	52.527	46.412
NM_0190	chr7	1.21E+08	1.21E+08	+	2190	1	ING3|Eaf4	3.868	4.359
NM_0011	chr1	32936446	32964813	-	2723	1	RNF19B|IE	16.781	22.097
NM_0011	chr7	1.01E+08	1.01E+08	+	2977	1	SRRT|ARS2	48.637	44.965
NM_0071	chr2	1.51E+08	1.51E+08	+	1416	1	TNFAIP6|T	0.009	0.019
NM_0012	chr16	68013436	68015911	-	1181	1	LOC10013	0	0
NM_0013	chr21	46286330	46297723	+	453	1	YBEY|C21c	30.739	24.28
NM_0060	chr21	46324122	46445769	+	10511	1	PCNT|KEN	7.638	6.2
NM_0063	chr9	89377237	89479696	-	4608	1	SEMA4D|C	10.375	23.039
NM_0013	chr16	53435619	53491648	+	4630	1	RBL2|P130	8.407	10.629

NR_04672	chr3	1.13E+08	1.13E+08	+	680	1	CFAP44-AS	0.19	0.101
NM_0010:	chr15	74826610	74831795	+	2007	1	CPLX3|CP)	5.775	3.467
NM_0013(	chr5	1.35E+08	1.35E+08	+	5709	1	DDX46|PR	7.383	8.75
NM_0132	chr20	23350736	23354771	+	1113	1	NXT1|MTF	22.277	24.748
NM_0180	chr2	55287842	55419921	-	9698	1	CCDC88A|	4.038	8.091
NM_0133	chr12	72272749	72665642	+	5531	1	TRHDE|PA	0.347	0.31
NR_03644	chr11	57037534	57038460	+	926	1	OR5AK4P|	0.097	0.237
NM_1827	chr5	43526268	43557093	-	2545	1	PAIP1|-|-|	9.474	10.861
NM_0065	chr16	67562407	67639185	+	3927	1	CTCF|MRD	27.642	28.256
NR_03032	chr7	96219662	96219756	-	94	1	MIR591|M	0	0
NM_0011:	chr10	68827537	68895453	+	3299	1	STOX1|C1	0.641	2.595
NR_13670	chr1	35176378	35193145	-	2296	1	SFPQ|POM	164.122	138.539
NM_0016	chr6	1.07E+08	1.07E+08	+	7520	1	AIM1|CRY	0.086	0.153
NR_03987	chr16	88716278	88716337	-	59	1	MIR4722|	0	0.696
NM_0308	chr17	8435852	8468177	+	2387	1	NDEL1|EO	20.051	22.545
NM_0031	chr6	90513573	90587301	-	5068	1	MAP3K7|(	8.764	9.211
NM_0011	chrX	1.55E+08	1.56E+08	-	1323	1	TMLHE|AL	8.937	9.701
NM_0012(	chr6	38715341	39030798	+	14544	1	DNAH8|AT	0.004	0.008
NR_02764	chr1	1.16E+08	1.16E+08	-	559	1	ATP1A1-AS	45.623	68.828
NR_07339	chr8	12414521	12418032	-	1892	1	FAM90A25	0.027	0.116
NR_03889	chr18	3878180	3897069	+	1255	1	DLGAP1-A	0.041	0.175
NM_0011	chrX	1.5E+08	1.51E+08	+	4524	1	MAMLD1|	2.67	3.312
NM_0226	chr12	54009106	54012761	+	2284	1	HOXC8|HC	0	0.042
NM_0318	chr17	43170471	43211689	+	4643	1	NBR1|1A1	31.385	29.937
NM_0012	chr6	292057	351355	+	3496	1	DUSP22|JI	14.107	12.247
NM_1993	chr9	1.3E+08	1.3E+08	-	1611	1	C9orf50|-|	0.072	0.136
NR_02947	chr11	1.22E+08	1.22E+08	-	71	1	MIRLET7A	0	0
NM_0011	chr1	29236516	29326813	+	5551	1	PTPRU|FM	2.646	2.872
NM_0011	chr20	45536280	45547426	-	1855	1	EPPIN-WFI	0	0
NM_0146	chr1	2.26E+08	2.26E+08	-	4426	1	TMEM63A	0.854	1.281
NR_00243	chr8	1.35E+08	1.35E+08	+	937	1	ZFAT-AS1|	0.619	0.804
NM_0013:	chr10	22534837	22714108	-	3602	1	PIP4K2A|P	8.577	9.309
NM_0010	chrX	1.21E+08	1.21E+08	-	1288	1	CT47A7|C	0	0
NM_0010	chr14	94442464	94452785	-	1464	1	SERPINA11	0	0
NM_0322	chr22	44492570	44498125	-	5320	1	LDOC1L|M	53.459	55.813
NM_0012	chr18	57630302	57669276	+	1977	1	LOC10050	0.574	0.727
NM_0320	chr5	1.41E+08	1.42E+08	+	4311	1	PCDHGA1	21.465	86.455
NR_03035	chr14	65471102	65471186	+	84	1	MIR625|M	0	0
NM_0071	chr13	41061561	41084003	+	2321	1	WBP4|FBF	4.522	5.347
NR_03994	chr2	1.31E+08	1.31E+08	-	76	1	MIR4784|	1.527	2.523
NM_1524	chr11	1.07E+08	1.07E+08	-	3259	1	CWF19L2|	1.499	1.685
NM_0190	chr12	1.22E+08	1.22E+08	-	2428	1	RHOF|ARH	6.771	10.154
NR_02437	chr8	1.42E+08	1.42E+08	+	1043	1	LINC00051	0	0.053
NM_0010	chr11	6211334	6234711	-	3436	1	FAM160A2	10.297	10.03
NM_0012	chr1	1.54E+08	1.54E+08	-	2017	1	SLC39A1|Z	52.047	39.93
NM_0012	chr16_KI2	613600	631834	-	1040	2	NTAN1|PN	0.025	0
NM_0000	chr7	94394561	94431232	+	5357	1	COL1A2|O	1.037	4.367
NM_0220	chrX	1.02E+08	1.02E+08	+	2376	2	NXF2|CT3	0	0
NM_0013	chr17	50834650	50842353	+	3234	1	WFIKKN2|	0.897	0.432
NM_1392	chr3	1.42E+08	1.42E+08	+	1741	1	GRK7|GPR	0.03	0.039

NM_0139	chr1	24745269	24844324	+	4426	1	CLIC4|CLI(	17.901	18.877
NM_0029	chr1	1.93E+08	1.93E+08	+	1343	1	RGS2|G0S	16.206	20.428
NR_03745	chr16	29599179	29599265	-	86	2	MIR3680-1	0	0
NM_0307	chr11	27338514	27363248	-	1475	1	CCDC34|L	4.292	3.083
NM_0010	chr9	1.1E+08	1.1E+08	-	2652	1	C9orf152|	0.058	0.046
NM_0064	chr16	1972063	1978750	+	2570	1	TBL3|SAZD	15.954	16.181
NR_12036	chr15	86083345	86116717	-	846	1	LINC01584	0	0
NM_0124	chr7	73568946	73578683	-	3030	1	TBL2|WBS	15.081	13.064
NM_0013	chr2	2.41E+08	2.41E+08	-	1133	1	MTERF4|N	19.642	19.318
NM_1384	chr16	4371848	4383528	+	2802	2	VASN|SLIT	0	0
NM_0013	chr19	12163047	12189881	+	3528	1	ZNF136|pI	2.028	2.904
NR_10500	chr5	61732790	61735698	-	1178	1	C5orf64-A	0.12	0.058
NR_03163	chr9	97363554	97363681	-	127	1	MIR1302-8	0	0
NM_0012	chr12	68294566	68332381	-	2848	1	MDM1|-|-	5.46	9.162
NM_0010	chr1	1.6E+08	1.6E+08	+	1931	1	FCRL6|FcR	0	0.007
NR_03768	chr17	81509971	81512866	-	1830	1	ACTG1|AC	4201.76	3540.948
NR_13516	chr1	1.54E+08	1.55E+08	-	6167	1	SHE|-|-|1	0.038	0.031
NM_0143	chr3	1.22E+08	1.22E+08	+	831	1	FAM162A|	65.166	29.306
NM_0013	chr10	1.02E+08	1.02E+08	-	1651	1	FBXW4|D	0.351	0.597
NR_04755	chr10	43643872	43645047	+	432	1	ZNF32-AS1	25.13	34.369
NM_0069	chr14	69767087	69772005	+	1537	1	SRSF5|HR	106.839	94.908
NR_04071	chr8	1.41E+08	1.41E+08	+	2196	1	CHRAC1|C	3.376	2.439
NR_13238	chr4_KI27	219023	222370	+	589	4	LINC0159	0	0
NM_0010	chr6	24494969	24537207	+	5119	1	ALDH5A1|	4.393	6.05
NM_0012	chr22	38518949	38568409	-	2069	1	DMC1|DM	0.125	0.013
NM_1391	chr17	72645946	73092714	-	2749	1	SLC39A11|	3.096	7.075
NR_04058	chr7	74881990	74890612	-	1866	1	STAG3L2|	1.479	1.307
NM_0011	chr6	85613975	85641451	-	2548	1	SYNCRIP|	20.529	25.98
NM_0177	chr19	13906143	13930880	+	3609	1	CC2D1A|F	11.418	15.132
NM_0056	chrX	48255317	48267444	+	1288	1	SSX1|CT5.	0	0
NM_0144	chr1	16014028	16018790	-	2887	1	HSPB7|cv	0.027	0.009
NM_0017	chr16	61652011	62036835	-	4938	1	CDH8|Nbl	3.031	3.922
NM_0156	chr22	18606127	18611919	-	5790	1	RIMBP3|R	0.027	0.062
NR_03976	chrX	39837561	39837613	+	52	1	MIR1587|	0	0
NM_0013	chr17	31488001	31538217	+	8320	1	RAB11FIP4	11.272	16.183
NM_0013	chr18	79972867	80033935	-	1174	1	TXNL4A|B	55.495	54.94
NM_0012	chr6	1.55E+08	1.55E+08	+	4822	1	SCAF8|RBI	15.172	19.29
NR_10995	chr21	21746985	21797415	+	755	1	LINC01425	0	0
NM_0309	chr6	29373423	29375291	-	1866	6	OR12D3|h	0	0
NM_0047	chr4	1.18E+08	1.18E+08	+	5931	1	NDST3|HS	2.109	0.834
NR_00439	chr2	2.31E+08	2.31E+08	-	74	1	SNORD82|	0.174	0.185
NM_2015	chrX	71239624	71254188	-	5557	1	ZMYM3|D	39.196	41.776
NM_1446	chr3	44861909	44865667	+	978	1	TMEM42|	5.155	1.947
NM_0013	chr2	1789113	2331257	-	6355	1	MYT1L|MI	5.34	11.393
NM_0048	chr14	64465499	64474503	+	6483	1	AKAP5|AK	0.613	0.456
NM_0012	chr10	47921929	47923377	+	1446	1	CH17-360I	0	0.019
NM_0320	chr5	1.13E+08	1.13E+08	-	2475	1	TSSK1B|FK	0.036	0.089
NM_0012	chr17	15699577	15720786	+	5278	1	ZNF286A|	3.608	4.209
NR_03059	chrX	1.46E+08	1.46E+08	-	76	1	MIR892B|	0	0
NR_03651	chr12	1.11E+08	1.11E+08	+	357	1	LINC01405	0.217	0.499

NM_0202(	chr4	87832969	87846816	+	2000	1	MEPE|OF4	0	0
NM_0178:	chr2	26764274	26781231	+	3904	1	SLC35F6|A	12.015	12.202
NR_03784	chr6_GL0C	3217228	3242105	+	3511.6	7	MSH5-SAP	0	0
NM_0069<	chr12	23529504	23949703	-	7092	1	SOX5|L-SC	1.725	6.916
NM_1523!	chr3	42685519	42692446	+	2411	1	KLHL40|KE	0	0.006
NM_0012<	chr9	1.13E+08	1.13E+08	+	2905	1	PRPF4|HPI	17.558	18.063
NR_03159	chr5	1.8E+08	1.8E+08	-	68	1	MIR1229|I	7.964	11.28
NM_0245!	chr4	1.14E+08	1.14E+08	-	4674	1	ARSJ|-|-|4	0.055	0.158
NM_0057{	chr13	39342892	39603219	-	2152	1	LHFP|-|-|1	13.792	13.188
NM_0013:	chr3	1.65E+08	1.65E+08	-	4551	1	SLITRK3|-|	5.969	5.192
NR_10404	chr11	1.19E+08	1.19E+08	+	1275	1	CCDC84|D	5.269	5.404
NM_0016!	chr8	20197193	20221696	+	3028	1	ATP6V1B2	87.091	102.501
NR_13195	chr12	1.31E+08	1.31E+08	-	786	1	LACAT8|-|	0.066	0.017
NM_0011<	chr18	74073353	74147865	-	1709	1	FBXO15|FI	1.577	1.803
NR_11041	chrX	36365626	36440292	-	1853	1	LOC10192:	0.042	0
NM_1524!	chr1	2.35E+08	2.36E+08	-	4734	1	B3GALNT2	4.595	6.053
NM_0010<	chr4	8949751	8950401	+	649	1	LOC65029:	0	0
NM_0071:	chr14	75650890	75661195	-	1533	1	C14orf1|E	56.292	52.108
NR_04749	chr13	47930153	47932622	-	2469	1	LINC00562	0.057	0.05
NR_03974	chr17	12917268	12917342	-	74	1	MIR1269B	0	0
NM_0328!	chr13	1.08E+08	1.08E+08	+	5298	1	ABHD13|E	1.918	2.384
NM_0227:	chr1	2.06E+08	2.06E+08	-	6455	1	NUCKS1|J(	41.862	48.69
NR_00299	chr1	1.76E+08	1.76E+08	-	143	1	SCARNA3|	0	0.096
NR_03765	chr11	65014113	65040572	+	2452	1	ARL2-SNX1	34.484	30.673
NM_2069<	chr11	60378485	60395953	+	2817	1	MS4A7|4S	0.009	0.005
NR_07306	chr22	17655662	17730855	+	4725	1	BCL2L13|E	24.092	18.216
NM_0013:	chr17	44141902	44162476	+	2383	1	C17orf53|	1.531	1.092
NM_1989<	chr9	1.37E+08	1.37E+08	-	701	1	LCN6|LCN:	0	0
NM_0013:	chr1	12567910	12596396	-	1324	1	DHRS3|DC	2.444	3.373
NM_0010:	chr1	13175281	13179132	+	1433	2	PRAMEF9|	0	0
NR_10687	chr22	36286847	36286907	-	60	1	MIR6819|I	3.653	2.739
NR_00370	chr22	21472998	21517491	-	2255	1	PI4KAP2|-	1.498	1.865
NM_0151:	chr3	52495340	52524495	+	7855	1	STAB1|CLE	0.873	0.755
NM_0198<	chr19	33208664	33225850	-	1933	1	SLC7A10|/	2.401	2.657
NM_0066(	chr4	37960435	37961010	+	574	1	PTTG2|-|-	0.202	0.262
NM_1944(	chr19	647526	663233	-	1639	1	RNF126|-|	31.593	29.667
NM_0012!	chr8	489790	544845	-	2461	1	TDRP|C8o	4.286	4.313
NR_02803	chr6_GL0C	1319168	1322454	+	645.6	7	HCG9|HCG	0	0
NM_1457:	chr22	29327680	29388583	-	4125	1	AP1B1|AD	52.643	50.544
NR_03033	chr10	24275685	24275781	+	96	1	MIR603|M	0	0
NM_0011:	chr12	24810024	24903075	-	9255	1	BCAT1|BC.	4.712	1.672
NM_0063!	chr19	12806614	12813648	+	1132	1	RNASEH2A	33.612	20.618
NR_13278	chr1	2.45E+08	2.45E+08	-	130	1	SNORA10C	20.63	20.546
NM_0012!	chr7	65960686	65982314	-	1958	1	GUSB|BG|	20.611	18.706
NM_0011<	chrX	1.54E+08	1.54E+08	+	2565	1	TKTL1|TKF	0.784	1.559
NM_0013:	chr9	1.29E+08	1.29E+08	+	3328	1	PKN3|UTC	2.758	2.881
NM_0148!	chr7	99394676	99408682	-	2678	1	PDAP1|HA	38.218	38.447
NM_0022(	chr3	37452322	37819790	+	3932	1	ITGA9|ALF	0.177	0.376
NM_0323!	chr17	42773436	42779600	+	1094	1	VPS25|DEI	79.612	72.905
NM_0041:	chr6	35573585	35688942	-	3769	1	FKBP5|AIG	4.03	1.468

NM_0011(	chr1	2.28E+08	2.28E+08	-	2540	1	JMJD4|-|-	15.3	14.161
NM_0156:	chr10	68988721	69016983	+	2536	1	KIF1BP|KB	18.567	15.533
NR_11004	chr11	1.14E+08	1.14E+08	-	1872	1	TMPRSS5|	1.171	1.997
NM_0207.	chr18	36829117	37225325	+	4842	1	KIAA1328|	1.648	2.354
NM_0010(	chr1	450740	451678	-	937	3	OR4F16|O	0	0
NM_0024(	chr11	62208668	62213939	+	516	1	SCGB2A1|	0	0
NM_0011.	chr19	33621967	33773509	+	2131	1	CHST8|GA	47.611	12.257
NM_1537(	chr3	1.87E+08	1.87E+08	+	2213	1	RTP1|Z3C)	1.474	1.399
NM_0164:	chr17	42659248	42667006	+	1805	1	TUBG2|-|-	12.479	14.001
NM_0000!	chr3	48564073	48595160	-	9049	1	COL7A1|E	2.112	1.486
NM_0006	chrX	1.15E+08	1.15E+08	-	1361	1	IL13RA2|C	2.548	1.51
NR_00218	chr22	42573260	42582011	-	2309	1	RRP7BP|R	2.005	2.26
NM_0013:	chr18	5954706	6414919	-	3547	1	L3MBTL4|	0.262	0.618
NR_03780	chr19	12144894	12156731	-	1687	1	ZNF625|-|	2.224	2.298
NR_13431	chr10	63430575	63431202	+	477	1	LOC10537.	0	0
NM_0211:	chr9	36036913	36124455	+	4389	1	RECK|ST1!	1.334	1.517
NR_03978	chr6_GL0C	3006077	3006139	-	62	7	MIR4646|	0	0
NM_0058!	chr1	40258050	40294184	+	3153	1	ZMPSTE24	7.226	7.898
NM_0189:	chr5	1.41E+08	1.42E+08	+	4596	1	PCDHGA1|	20.401	81.211
NM_0161	chr3	32525971	32570874	-	2489	1	DYNC1LI1|	27.196	27.746
NM_0012(	chr17	44924711	44947711	-	4008	1	KIF18B|-|-	1.747	1.575
NM_0013	chr11	62515902	62545766	-	18631	1	AHNAK|AH	2.361	2.714
NR_07351	chr1	1.5E+08	1.51E+08	+	2174	1	TARS2|CO	15.717	15.688
NM_0011(	chr12	1.23E+08	1.23E+08	-	11457	1	SBNO1|M(	5.187	4.888
NR_12577	chr9	12699999	12814389	-	940	1	LURAP1L-/	0.219	1.166
NM_1332(	chr4	54100081	54101955	+	1208	1	GSX2|GSH	0.011	0.215
NM_0006.	chr5	1.6E+08	1.6E+08	+	2250	1	ADRA1B|A	0.464	1.321
NM_0180(	chr6	87674860	87702267	-	1925	1	AKIRIN2|C	48.694	63.789
NR_02951	chr20	3917494	3917571	+	77	1	MIR103A2	0.67	0.534
NR_03808	chr17	81302824	81309248	-	2966	1	LINC00482	0.23	0.102
NR_13459	chr6	1.67E+08	1.67E+08	-	511	1	LOC10537.	0	0.027
NM_0012.	chr19	48615327	48619418	-	792	1	RPL18|L18	784.012	678.504
NM_0007(	chr9	73151731	73170394	+	1521	1	ANXA1|AN	12.766	2.972
NM_0184!	chr14	34515929	34539737	-	1340	1	EAPP|BM(	35.573	39.875
NM_0054!	chr17	56593699	56595590	+	1889	1	NOG|SYM	0.3	0.566
NR_03395	chr10	46398362	46453306	+	2868	1	LINC00842	0	0
NR_03314	chr1	53765461	53838552	-	4687	1	NDC1|NET	4.899	5.205
NM_0229:	chr11	87037844	87328834	+	9063	1	TMEM135	0.723	0.895
NM_0011	chr2	38919363	38975449	+	4286	1	ARHGEF33	0.283	0.3
NM_0159.	chr13	27621779	27623453	+	806	1	POLR1D|A	70.53	81.672
NR_12588	chr4	1.62E+08	1.62E+08	+	1082	1	LOC10192:	0	0
NM_0204.	chr18	35654569	35711834	+	3825	1	GALNT1|G	11.056	11.23
NM_0319(	chr11	47589664	47594409	-	1410	1	C1QTNF4|	12.153	11.958
NM_0066!	chr20	45512461	45515624	-	472	1	SPINT3|HK	0	0
NM_0013:	chr1	2.45E+08	2.45E+08	+	2343	1	COX20|FA	19.464	19.555
NM_0010:	chr7	99458161	99466201	-	371	1	ATP5J2|AT	239.589	163.146
NM_0206:	chr1	6466092	6491701	-	4621	1	PLEKHG5|	10.7	14.726
NM_2074:	chr9	81943500	81950094	+	4921	1	SPATA31D	0	0
NR_03165	chr2	1.8E+08	1.8E+08	-	72	1	MIR1258|	0	0
NR_03822	chr15	88252730	88271066	+	2659	1	NTRK3-AS:	0	0.005

NM_1827(	chr15	64911902	64958699	+	3092	1	ANKDD1A	0.838	2.095
NM_1383	chr11	1.34E+08	1.34E+08	+	3133	1	GLB1L2|M	9.371	12.114
NM_0056(	chr11	67435510	67437682	-	903	1	PTPRCAP|	2.185	2.033
NM_0011(	chr1	1.48E+08	1.48E+08	-	4749	1	NBPF11|N	0.717	0.995
NM_0010	chr11	202924	207422	-	2809	1	BET1L|BET	18.535	13.507
NM_0010	chr7	1.44E+08	1.44E+08	-	2065	1	NOBOX|O	0	0
NM_2069	chr4	56505209	56523892	+	830	1	ARL9|-|-|	0.171	0.017
NM_0250	chr8	66630253	66667217	-	8171	1	VCPIP1|DU	1.389	1.515
NR_03977	chr18	5840695	5840833	+	138	1	MIR3976|	0	0
NR_03378	chr10	1.34E+08	1.34E+08	-	2239	1	SPRNP1|-|	0	0.006
NM_0149(	chr9	37651055	37746904	+	4898	1	FRMPD1|F	1.945	0.294
NR_03028	chr2	2.32E+08	2.32E+08	+	94	1	MIR562|M	0	0
NM_0013(	chr4	69588417	69639642	-	2429	1	UGT2A2|-	0	0
NR_13510	chr1	32825313	32858879	+	4208	1	S100PBP|S	10.929	12.584
NM_0186	chr7	1.05E+08	1.05E+08	+	6687	1	KMT2E|HD	14.162	15.1
NM_0013	chr2	1.12E+08	1.12E+08	+	5191	1	TMEM87B	0.596	0.747
NM_0011	chr17	7459366	7479625	-	5860	1	ZBTB4|KAI	16.09	16.514
NM_0019	chr5	1.28E+08	1.29E+08	-	10657	1	FBN2|CCA	1.416	1.033
NM_0784	chr6	70216040	70283208	-	3016	1	COL9A1|D	0.932	0.872
NM_0003	chr17	72121020	72126419	+	3929	1	SOX9|CMD	9.113	24.309
NR_12119	chr1	1.74E+08	1.74E+08	-	444	1	LOC10272	0.232	0.062
NR_00304	chr20	17962710	17962946	-	236	1	SNORD17|	0.382	0.116
NR_13564	chr17	39026715	39027110	-	292	1	LINC0207	0	0
NR_12593	chr4	1.19E+08	1.19E+08	-	1541	1	LOC10192	0.226	0.044
NM_1738	chr3	1.21E+08	1.21E+08	-	3873	1	RABL3|-|-	4.341	4.304
NR_11013	chr3	14348451	14352568	-	2281	1	LINC01267	0.006	0
NM_0055	chr12	52233170	52248925	+	1691	1	KRT7|CK7	0.03	0.008
NM_0142	chr2	1.76E+08	1.76E+08	+	1881	1	HOXD9|H	0.302	0.495
NR_10998	chr3	1.69E+08	1.69E+08	+	737	1	LINC02082	0.525	0.093
NR_03075	chr13	90231182	90231277	+	95	1	MIR622|M	0	0
NM_0013	chr8	1.08E+08	1.08E+08	+	885	1	EMC2|KIA	6.541	4.891
NM_0010	chr19	38789210	38791754	+	483	1	LGALS7B|	0	0
NM_0070	chr19	47475141	47484264	-	1676	1	KPTN|2E4	15.471	14.702
NR_11021	chr2	1.86E+08	1.86E+08	-	596	1	LINC01473	1.298	0.827
NR_10447	chr21	32412437	32515400	+	1740	1	EVA1C|B1	1.052	2.228
NM_0012(	chr15	41981582	42050703	-	4758	1	PLA2G4E|	0.011	0.006
NM_0318	chr19	53963036	53990215	+	8745	1	CACNG8|-	6.063	6.469
NM_1735	chr12	1.13E+08	1.13E+08	+	2818	1	PLBD2|P7	16.375	14.173
NM_0010(	chr19	15794049	15794996	+	946	1	OR10H5|C	0	0
NM_0144	chr3	1.02E+08	1.02E+08	-	5106	1	ZBTB11|ZN	3.548	3.994
NM_1735	chr11	63911221	63916844	-	2590	1	RCOR2|-|-	109.739	102.347
NM_0331	chr3	1.61E+08	1.61E+08	-	3284	1	B3GALNT1	0.02	0.888
NR_10268	chr14	24007009	24051377	+	1164	1	DHRS4L1|	0.177	0.224
NR_10419	chr17	38752713	38764231	+	713	2	PSMB3|H	0	0
NM_0206	chr11	1.17E+08	1.18E+08	-	6865	1	DSCAML1|	2.346	5.571
NM_0162	chr3	1.22E+08	1.22E+08	+	5716	1	FBXO40|F	0.005	0.002
NR_02449	chrX	1.48E+08	1.48E+08	-	2386	1	FMR1-AS1	0.049	0.023
NR_11055	chr5	1.43E+08	1.43E+08	+	2228	1	LINC01844	0.226	0.32
NM_0013	chr4	44678395	44700680	+	4099	1	GUF1|EF-4	5.115	6.526
NM_0211(	chr19	38264458	38292614	+	1793	1	SPINT2|DI	23.263	22.704

NM_1450(	chr18	50375022	50394168	+	2864	2	SKA1|C18(	0	0
NM_1307	chrX	52862529	52867301	-	478	1	XAGE3|CT	0.081	0
NM_0023	chr11	46856717	46918622	-	8187	1	LRP4|CLSS	8.889	10.053
NM_0005	chr2	1.13E+08	1.13E+08	-	2923	1	IL1A|IL-1A	0.009	0.023
NM_0010	chr17	56791913	56833895	-	2002	1	C17orf67|	0.625	0.876
NM_0011	chr3	1.7E+08	1.7E+08	+	6698	1	SKIL|SNO|	2.025	1.697
NM_1522	chr1	1331346	1335320	+	3407	1	TAS1R3|T1	1.813	1.886
NM_0013	chr3	72996743	73069201	+	4746	1	PPP4R2|PI	5.931	7.469
NM_0150	chr11	95089810	95132651	+	4689	1	ENDOD1|-	2.123	2.962
NR_12068	chr8	85217161	85220422	-	403	1	C8orf59|-|	41.272	28.142
NR_07320	chr20	13849398	13995333	-	2222	1	SEL1L2|C2	0.012	0.043
NM_1826	chr2	10141382	10211730	+	1896	1	C2orf48|-|	0.041	0.303
NM_1452	chr17	35760897	35765079	+	1026	1	C17orf50|	0.226	0.214
NM_0010(	chr11	55638358	55639296	+	937	1	OR4P4|OF	0	0
NM_0010	chrX	1.5E+08	1.5E+08	-	1333	1	CXorf40B|	11.027	11.683
NM_0036	chr20	57351089	57378463	+	1798	1	RAE1|MIG	47.788	56.746
NR_10684	chr17	82236668	82236728	+	60	1	MIR6787|	3.868	0
NR_03743	chr7	1.15E+08	1.15E+08	+	110	1	MIR3666|	0	0
NM_0013	chr11	65879813	65883741	+	1284	1	CTSW|LYP	0.231	0.096
NM_0010	chr17	1464186	1485757	-	4687	1	MYO1C|M	7.507	6.973
NM_2124	chr2	2.15E+08	2.15E+08	-	8226	1	FN1|CIG|E	11.973	7.41
NR_00256	chr11	93721514	93721866	+	352	1	SCARNA9|	0.293	0.545
NM_0068(	chr20	44942130	44960473	-	2040	1	TOMM34|	13.5	15.476
NM_0220	chr1	2.31E+08	2.31E+08	-	7090	1	EGLN1|C1	5.428	5.346
NM_0021	chr16	85899168	85922605	+	2650	1	IRF8|H-ICS	0	0
NM_0808	chr20	63539924	63547504	-	2392	1	SRMS|C20	0.69	0.338
NR_11066	chr1	2.24E+08	2.24E+08	-	787	1	LOC10192	0.098	0.07
NM_0228	chr13	61409686	61415522	-	4773	1	PCDH20|P	0.189	1.822
NM_0050(	chr12	4649098	4687554	+	1596	1	NDUFA9|C	36.039	30.226
NM_0011	chr17	7014737	7017524	+	895	1	C17orf49|	115.307	112.3
NM_0178	chr2	1.53E+08	1.53E+08	-	7500	1	PRPF40A|	5.126	7.526
NR_11081	chr17	59430869	59526857	-	543	1	LINC0147E	0	0.126
NM_1984	chr19	38867832	38878279	-	3030	1	RINL|-|-|1	0.281	0.384
NR_12066	chr10	80529597	80535942	-	596	1	LOC10192	0.887	1.333
NM_1825	chr1	54980664	54992293	+	1220	1	TMEM61|	0.211	0
NM_0161	chr3	1.38E+08	1.38E+08	-	1766	1	A4GNT|alp	0.015	0.078
NM_0013	chr2	27888709	28338900	+	1596	1	BRE|BRCC	31.87	34.423
NM_0201	chr17	63745250	63773728	-	3404	1	CCDC47|G	13.234	15.822
NR_13366	chr11	64291722	64304769	+	2722	1	KCNK4-TEX	0.725	0.468
NM_0070	chr19	45039040	45070956	+	2211	1	CLASRP|CI	18.363	19.514
NR_00443	chr1	1.46E+08	1.46E+08	+	163	9	RNU1-1|H	0	0
NR_03961	chr1	55225641	55225723	+	82	1	MIR4422|	0	0
NR_00368	chr22	29333163	29333258	-	95	1	SNORD125	1.086	0.577
NR_02679	chr10	26589865	26594322	+	1079	1	LINC00264	0	0
NM_0207	chr16	2177183	2196464	-	5673	1	CASKIN1|	16.948	17.007
NM_0008	chr1	1.1E+08	1.1E+08	+	1191	1	GSTM2|GS	7.654	13.202
NM_0146	chr2	2.19E+08	2.19E+08	+	4974	1	TTLL4|-|-|	5.757	4.698
NM_0066	chr15	65148220	65185420	-	4756	1	CLPX|-|-|1	5.853	7.246
NM_0319	chr17	41314917	41315695	-	776	2	KRTAP17-1	0	0
NM_0055	chr10	1.33E+08	1.33E+08	+	2990	1	INPP5A|5F	18.141	21.059

NM_2033!	chr1	25900116	25906877	-	1495	1	STMN1|C1	1606.105	1826.275
NR_02426	chr20	4731282	4740668	-	2331	1	PRNT|M8|	0	0
NM_0010{	chr1	1.11E+08	1.12E+08	-	1183	1	TMIGD3|A	0.022	0
NM_0218:	chr22	39077005	39087743	+	1824	1	APOBEC3G	0	0.06
NM_1749:	chr4	39547297	39638998	-	5417	1	SMIM14|(	8.216	11.323
NM_0011!	chr17	78991716	79009817	-	3333	1	CANT1|DB	23.118	26.457
NR_13523	chr2	51032601	52407917	+	3928	1	LOC73010(	0.007	0.146
NM_0013(	chr2	1.92E+08	1.92E+08	-	2844	1	TMEFF2|C	15.432	16.303
NM_0013(	chr17	7646627	7657771	+	2575	1	ATP1B2|A	26.463	23.006
NR_12582	chr8	37734761	37737426	-	2084	1	LOC10272:	0.087	0.118
NM_0002!	chrX	78104169	78126827	+	2410	1	PGK1|HEL	253.991	125.149
NM_0010(	chr7	56051685	56063989	+	2514	1	CCT6A|CC	72.314	73.356
NM_0012{	chr17	36486627	36495821	+	917	2	ZNHIT3|TF	0	0
NM_0011(	chr6	1.09E+08	1.09E+08	-	1242	1	ZBTB24|BI	2.388	2.68
NR_11079	chr1	1.86E+08	1.86E+08	-	599	1	LINC0135C	0.043	0.046
NM_0161:	chr4	83035086	83075818	+	1736	1	COPS4|CSI	31.357	32.814
NR_03828	chr1	23760382	23778297	-	1080	1	ELOA-AS1|	22.301	21.129
NR_04607	chr3	1.81E+08	1.81E+08	-	1244	1	DNAJC19|	7.649	6.463
NR_02725	chr19	50050589	50066795	+	1524	1	LOC40071(	0.761	0.782
NR_03622	chr2	1.09E+08	1.09E+08	-	98	1	MIR4265|-	0	0
NR_03023	chrX	1.47E+08	1.47E+08	-	128	1	MIR513A1	0	0
NR_03386	chr3	84638405	84869575	-	7288	1	LINC00971	0	0.008
NM_0003(	chr7	95404862	95435072	-	1643	1	PON2|-|-|	38.092	33.388
NM_0012.	chr15	84980513	85139145	+	3695	1	PDE8A|Hs	3.13	2.532
NM_0013:	chr20	33161848	33181417	+	1036	1	BPIFA2|C2	0	0
NR_13392	chr8	1.23E+08	1.23E+08	+	786	1	WDYHV1|	20.242	22.062
NM_0036(	chr19	49119389	49151030	+	4710	1	PPFIA3|LP	26.028	30.369
NR_03326	chr20	62543070	62551561	-	3603	1	C20orf166	0.118	0.129
NM_1336.	chr22	33273076	33920428	-	4121	1	LARGE1|L	34.748	18.875
NM_0052!	chr14	75278778	75282234	+	2152	1	FOS|AP-1|	11.899	80.515
NM_0020{	chr19	41998316	42065805	-	3512	1	GRIK5|EA/	24.036	27.932
NR_00439	chr11	62665422	62665570	+	148	1	SNORA57|	3.136	4.165
NR_07308	chr14	1.05E+08	1.05E+08	+	1200	1	CRIP2|CRI	210.884	154.616
NM_0335.	chr5	1.81E+08	1.81E+08	+	3655	1	TRIM41|R	15.948	13.03
NM_0159:	chrX	78129748	78139682	-	2680	1	TAF9B|DN	9.338	5.959
NM_0053!	chr9	93576627	93679587	+	5325	1	PHF2|CEN	38.855	38.166
NM_2072!	chr3	1.52E+08	1.52E+08	+	5359	1	MBNL1|EX	2.341	2.696
NM_0011(	chr10	1.01E+08	1.01E+08	+	1774	1	TWNK|AT)	7.653	8.902
NM_0005!	chr19	47821840	47843329	+	4462	1	CRX|CORD	0.142	0.015
NM_0035:	chr3	1.33E+08	1.33E+08	+	1568	1	BFSP2|CP4	0.025	0.14
NM_0012{	chr1	1.76E+08	1.76E+08	-	2572	1	RFWD2|C(	18.568	21.035
NR_14439	chr3	39051986	39096670	+	3786	1	WDR48|P{	20.021	25.882
NM_0034:	chr3	44648741	44660791	+	2643	1	ZNF35|HF.	5.469	7.043
NM_0010:	chr1	1.5E+08	1.5E+08	+	393	2	HIST2H4B|	0	0
NM_1445:	chr16	1678277	1702072	+	3676	1	HN1L|C16	45.755	34.57
NM_0134:	chr22	24583750	24629005	+	2345	1	GGT1|CD2	0.808	0.789
NM_0164:	chr11	10511678	10541227	-	4076	1	RNF141|ZI	6.393	9.1
NR_11084	chr6	2883988	2900676	+	645	1	LOC10192	0.1	0.106
NR_12648	chr14	60639216	60640416	+	1200	1	SALRNA1|	0	0
NR_02391	chr15	25116545	25122476	+	4495	1	IPW|NCRN	5.596	6.213

NR_02740	chr15	23157340	23170783	+	5320.5	6	GOLGA8DI	0	0
NM_0011	chr19	35115828	35123203	+	770	1	FXYD3|MA	0	0
NR_10354	chr1	54236443	54239063	+	2620	1	SSBP3-AS1	0.807	0.721
NM_1386	chr18	24139484	24161600	+	1276	1	CABYR|CA	0.818	0.966
NM_1456	chr11	193080	194573	+	426	1	SCGB1C1|	0	0
NR_04766	chr19	16361578	16472012	-	2808	1	EPS15L1|E	31.352	38.486
NM_0010	chr19	35030688	35040449	+	1440	1	SCN1B|AT	1.218	0.904
NM_0013	chr22	30080069	30177075	+	2243	1	HORMAD2	0.086	0.037
NM_0011	chr14	1.05E+08	1.05E+08	+	1528	1	C14orf80|	4.633	4.052
NM_0057	chr19	4229543	4237527	+	1130	1	EBI3|IL-27	0.034	0.012
NM_0058	chr17	41688875	41691646	+	1320	1	EIF1|A121	650.574	670.199
NM_0043	chr16	3725055	3880120	-	10163	1	CREBBP|C	17.357	17.362
NM_0011	chr19	49691108	49713731	+	2799	1	CPT1C|CA	58.23	49.503
NM_0306	chr6_GL0C	3469589	3473266	+	1280.6	7	EGFL8|C6c	0.262	0.128
NR_11091	chr12	1.33E+08	1.33E+08	+	541	1	LOC10192	0.667	0.354
NM_0033	chr14	36516399	36519698	-	2332	1	NKX2-1|B(	0	0.035
NR_13507	chr11	1.02E+08	1.02E+08	+	489	1	LOC10272	0	0
NR_10424	chr22	23856703	23882952	+	1437	2	SLC2A11|(	0	0
NM_0010	chr11	1.24E+08	1.24E+08	-	940	1	OR6M1|O	0	0
NM_0012	chr10	22316383	22331485	+	3372	1	COMMD3-	13.8	19.108
NM_0011	chr1	1.56E+08	1.56E+08	+	586	1	LAMTOR2	80.771	62.151
NR_13559	chr9	64639084	64700461	+	765	3	LOC10192	0	0
NM_0013	chr4	6053755	6194619	-	1868	1	JAKMIP1|(	2.837	9.737
NM_0156	chr6	36196773	36232790	+	6008	1	BRPF3|-|-	15.887	13.2
NR_13558	chr2	32023892	32039835	-	584	1	DPY30|Cp	39.387	46.509
NM_0012	chr1	1.72E+08	1.72E+08	+	2325	1	DNM3|Dy	4.708	5.279
NM_0013	chr8	23246592	23262000	+	2685	1	CHMP7|-|	30.027	44.805
NR_10996	chr21	33111704	33123700	-	1350	1	LOC10192	0	0
NR_03335	chr15	22424673	22438026	-	5251.3	6	GOLGA8EF	0	0
NM_2074	chr8	6808520	6835644	-	4881	1	XKR5|HAR	0.23	0.208
NR_14435	chr1	39522280	39559698	-	2524	1	PPIEL|PPIE	3.801	1.943
NM_0012	chr1	1.56E+08	1.56E+08	-	7741	1	GON4L|G(	9.034	9.707
NR_03397	chrX	1.12E+08	1.12E+08	+	4592	1	LINC0089C	0.135	0.042
NR_00370	chr18	49489235	49489324	-	89	1	SNORD58C	1.594	2.616
NR_03062	chr18	39622123	39622175	-	52	1	MIR924|M	0	0
NR_00298	chr3	1.31E+08	1.31E+08	-	235	1	SNORA58|	0	0.058
NM_0013	chr6	1.48E+08	1.49E+08	+	7160	1	SASH1|SH	4.873	2.877
NM_0012	chr10	1.02E+08	1.02E+08	+	3925	1	NOLC1|NC	38.697	36.055
NR_03974	chr16	30503919	30504001	+	82	1	MIR4518|	0	0
NM_1534	chr13	96090839	96839562	+	7800	1	HS6ST3|H	2.227	3.561
NM_0011	chr8	12425615	12436343	-	1258	1	FAM86B2|	0.184	0.49
NM_0012	chr1	47216290	47232335	-	4144	1	TAL1|SCL|	1.089	0.754
NR_12041	chr13	58165827	58209483	+	2750	1	LOC10192	0.014	0.005
NM_0013	chr5	1.14E+08	1.14E+08	+	5913	1	YTHDC2|C	3.511	4.983
NM_0035	chr6	26270918	26271384	-	465	1	HIST1H3G	0.111	0.059
NM_0064	chr19	41956681	41959376	-	800	1	RABAC1|P	105.259	95.074
NM_0314	chr12	10212890	10223125	+	1861	1	GABARAPL	98.056	126.011
NM_1736	chr19	57363435	57379557	+	2856	1	ZNF547|-|	3.503	3.889
NM_0012	chr9	1.33E+08	1.33E+08	-	3579	1	RALGDS|R	110.922	96.507
NM_0581	chr12	54854515	54858390	+	483	1	MUCL1|SE	0	0

NM_0013	chrUn_GL(	42938	86735	-	2364	1	LOC38983	2.645	1.246
NM_0010	chr2	26786015	26794587	+	1344	1	CENPA|CE	2.264	1.61
NM_0013(	chr19	5916141	5978309	-	3163	1	RANBP3|-	33.687	36.943
NM_0012	chr11	46381655	46383837	+	706	1	MDK|ARAI	255.66	119.49
NM_0325	chr6	1.59E+08	1.59E+08	+	6526	1	FNDC1|AG	0.077	0.034
NR_02726	chr10	38383070	38402852	-	667	1	SEPT7P9|(	0.116	0.185
NR_00370	chr2	69520045	69520171	-	126	1	SNORA36C	0	0
NR_02859	chr6	1.09E+08	1.09E+08	+	711	1	CCDC162P	0.018	0.077
NM_0010(	chr14	21633836	21634940	-	1102	1	OR10G2|C	0	0
NM_0160	chr14	51989510	52004702	+	1041	1	C14orf166	82.724	83.458
NM_0034	chr6	18224169	18264568	-	2858	1	DEK|D6S2	11.274	15.523
NR_03172	chr5	1.69E+08	1.69E+08	+	61	1	MIR103B1	0	0
NR_12536	chr22	43038585	43052365	+	2079	1	LOC10050	6.946	7.082
NM_0206	chr5	1.79E+08	1.79E+08	-	2493	1	CLK4|-|-|5	5.611	5.505
NM_1534	chr10	1.24E+08	1.24E+08	+	10299	1	GPR26|-|-	0.024	0.145
NM_0011	chr19	36510687	36528346	-	5355	1	ZNF260|O	3.455	4.962
NM_0011	chr17	63702832	63741970	-	2146	1	STRADA|L	29.969	32.347
NM_0142	chr17	8159147	8162975	-	2148	1	VAMP2|SY	171.841	177.526
NM_0011	chr4	83094154	83114758	-	997	1	PLAC8|C1	0.013	0.027
NM_0012	chr19	7903780	7914483	+	3485	1	MAP2K7|J	15.831	13.728
NM_0164	chr11	61362001	61369503	+	1835	1	TMEM138	7.35	7.099
NM_0306	chr1	1292384	1307889	-	3733	1	ACAP3|CE	42.162	38.343
NM_0222	chr15	41493858	41503559	+	1840	1	ITPKA|IP3-	0.673	1.169
NR_02814	chr15	1E+08	1E+08	-	1561	1	SPATA41|I	0.025	0.167
NM_0321	chr16	31117664	31131393	+	1516	1	KAT8|MOI	45.927	52.584
NM_0010(	chr11	56159394	56160317	-	922	1	OR8K5|OF	0	0
NM_0189(	chr5	1.41E+08	1.41E+08	+	5212	1	PCDHA5|C	16.577	15.823
NM_0165	chr5	33936386	33938918	+	2530	2	RXFP3|GP	0	0
NM_0012	chr19	50418938	50431052	+	3518	1	SPIB|SPI-B	0.07	0.023
NM_0010(	chr11	55665167	55666096	+	928	1	OR4C6|OF	0	0
NR_03337	chr3	8221147	8501658	-	2143	1	LMCD1-AS	0.193	0.115
NM_0190	chr1	19814029	19815278	-	1247	1	RNF186|-|	0	0
NM_0048	chr1	1.59E+08	1.59E+08	-	1476	1	AIM2|PYH	2.49	2.459
NM_0227	chr2	86720291	86778041	+	6172	1	RMND5A|	8.051	9.438
NM_0123	chr20	32819893	32850405	+	2614	1	MAPRE1|E	88.834	105.468
NM_0010(	chr11	1.24E+08	1.24E+08	+	934	1	OR10G8|C	0	0
NR_12589	chr4	1.73E+08	1.73E+08	-	624	1	LOC10192	0.021	0
NR_03767	chr15	43791976	43802571	+	1770	1	SERF2-C15	56.673	35.372
NM_0017	chr21	34787801	35049298	-	5956	1	RUNX1|AN	0	0.018
NM_0010	chr17	15976561	15999692	-	1869	1	ZSWIM7|S	13.156	14.562
NR_03654	chr1	1.2E+08	1.2E+08	-	1589	1	LINC00622	0.146	0.078
NM_0010	chr19	51642553	51646879	-	2104	1	SIGLEC14|	0	0.013
NM_0011	chr18	3262719	3278284	+	941	1	MYL12B|N	151.899	172.05
NM_0015	chr10	89392546	89406487	+	4375	1	IFIT1|C56|	1.105	0.733
NM_0011	chr10	62811996	62816380	-	2805	1	EGR2|AT5	0.087	2.852
NM_0010	chr5	1.32E+08	1.32E+08	-	2076	1	P4HA2|M	9.627	4.051
NM_0003	chr14	24249114	24263210	-	2760	1	TGM1|AR	1.056	1.176
NM_0012	chr19	55013707	55038264	-	2199	10	GP6|BDPL	0	0
NR_03041	chr19	46018932	46019049	+	117	1	MIR769|M	0	0.117
NM_0011	chr9	34179005	34252523	+	2610	1	UBAP1|NA	33.958	47.966

NR_10995	chr20	18674395	18698708	-	1690	1	LOC10192	0	0
NM_0009(	chr1	92832037	92841924	+	1021	1	RPL5|DBA	527.787	549.437
NM_0211	chr10	70202830	70233434	-	1289	1	PPA1|HEL-	51.144	69.728
NM_0169	chr11	1.26E+08	1.26E+08	-	8025	1	CDON|CD(	10.181	22.985
NM_0011	chr19	56176020	56185775	+	882	1	GALP|-|-|	0.716	0.435
NM_0010	chr9	64369563	64412691	+	3500	1	ANKRD20A	0.015	0
NM_0012	chr12	52010492	52015889	+	1710	1	GRASP|TA	3.303	3.989
NM_0022	chr7	1.08E+08	1.08E+08	-	5810	1	LAMB1|CL	7.29	3.588
NM_0011(	chrX	1.55E+08	1.55E+08	-	1009	1	SMIM9|CX	0	0
NM_0068	chr20	17569954	17608007	+	1608	1	DSTN|ACT	68.212	98.963
NM_0124(	chr19	11296139	11326996	+	1040	1	TSPAN16|	0.112	0.119
NR_03975	chr18	47380496	47380565	+	69	1	MIR4527|	0	0
NR_12041	chr18	76619777	76623559	+	2219	1	LINC00683	0.041	0.117
NM_0331	chr19	20004418	20039468	-	3201	1	ZNF682|B(	0.81	1.81
NM_0012	chr11	9460965	9528524	+	2926	1	ZNF143|SI	8.121	7.56
NR_13274	chr1	9148011	9182338	-	4155	1	MIR34AHG	0.689	0.613
NM_0134	chr19	12699194	12722291	-	4878	1	TNPO2|IP(	83.181	104.485
NM_0011	chr17	19770830	19867926	-	3868	1	ULK2|ATG	17.907	12.443
NM_0251(	chr1	2.24E+08	2.24E+08	-	6856	1	WDR26|CI	14.883	17.441
NM_0250	chr3	1.34E+08	1.34E+08	-	1664	1	C3orf36|-|	0	0
NM_0011	chr17	29929856	30074625	+	3728	1	EFCAB5|-|	0.169	0.257
NM_0146	chr9	1.19E+08	1.19E+08	-	3186	1	BRINP1|DI	18.717	22.747
NM_0012	chr9	1.13E+08	1.13E+08	-	2903	1	ZFP37|ZNI	1.728	2.614
NM_0012	chr2	2.01E+08	2.01E+08	-	1768	1	ALS2CR12	0.299	0.356
NM_0059	chr1	1.57E+08	1.57E+08	+	2114	1	PRCC|RCC	57.02	68.79
NM_0017(	chr8	1.42E+08	1.43E+08	+	5496	1	ADGRB1|E	12.018	7.212
NR_03028	chr1	1.62E+08	1.62E+08	+	94	1	MIR556|M	0	0
NR_03653	chr5	98929134	98931009	+	1875	1	LOC10028	1.086	1.49
NM_0022	chr10	7703273	7749520	+	3166	1	ITIH2|H2P	0.057	0.125
NM_1829	chr15	44736362	44767829	+	1894	2	TRIM69|H	0.088	0.195
NR_03898	chr2	1.66E+08	1.66E+08	+	686	1	TTC21B-AS	0.038	0.04
NM_0010	chr19	38587640	38618035	-	2681	1	MAP4K1|H	0.904	0.608
NM_0012	chr16	84177847	84187070	-	3851	1	TAF1C|MG	11.286	12.217
NM_0012	chr1	2.04E+08	2.04E+08	-	2345	1	ETNK2|EKI	23.186	29.479
NM_0008	chr19	49015980	49017090	-	518	1	LHB|CGB4	0.647	0.106
NR_03965	chr4	1.53E+08	1.53E+08	+	88	1	MIR4453|	1.905	6.849
NR_03380	chr12	98453841	98457145	-	3304	1	SLC9A7P1|	0.148	0.141
NM_0010	chr10	1.23E+08	1.23E+08	+	591	1	FAM24A|-	0	0
NR_02423	chr1	1.52E+08	1.52E+08	+	757	1	LOC10013	0.375	0.579
NM_0124	chr17	16939084	16972088	-	1351	1	TNFRSF13I	0	0
NM_0181	chr20	52151278	52191985	-	3255	1	ZFP64|ZNI	13.456	13.487
NM_0161	chr14	94283313	94293024	-	2446	2	SERPINA1(	0	0
NM_0012	chr9	1.01E+08	1.01E+08	+	3145	1	ZNF189|-|	8.121	12.556
NM_0041	chr6	2887266	2903312	-	4141	1	SERPINB9|	0.24	0.083
NM_0012	chr1	37996770	38005515	-	1474	1	FHL3|SLIM	13.2	14.087
NM_1984	chr19	47801243	47803604	-	1835	1	TPRX1|TPI	0.007	0
NM_0010	chrX	1.21E+08	1.21E+08	-	1288.6	10	CT47A1|C	0	0
NM_0067(	chr9	89605012	89606554	+	1059	1	GADD45G	33.421	33.835
NM_0012	chr5	81412807	81751253	-	8999	1	SSBP2|HSI	17.597	22.965
NM_1383	chr19	40576851	40591399	+	2345	1	SHKBP1|PI	25.479	18.066

NR_11016	chr2	66904436	66971466	+	624	1	LINC0179	0.165	0
NM_0011	chr2	32224449	32265620	-	3232	1	NLRC4|AIF	0.231	0.394
NR_04750	chr12	53983951	53985519	-	365	1	HOXC-AS3	0	0
NM_1383	chr12	1.12E+08	1.12E+08	-	1527	1	TMEM116	8.655	7.31
NR_03399	chr2	6912276	6918682	-	2343	1	RNF144A-A	1.222	1.491
NR_03611	chr11	18387787	18387860	+	73	1	MIR3159|-	0.706	0
NR_03891	chr1	1.01E+08	1.01E+08	-	818	1	LINC0134	0	0.017
NR_13501	chr12	89947693	89949726	+	397	1	LOC10536	0	0
NR_00323	chr14	1.01E+08	1.01E+08	+	70	1	SNORD113	0	0
NM_1458	chr21	33589341	33641854	-	1696	1	CRYZL1|4F	17.105	20.764
NM_0011	chr20	43565115	43585633	+	1901	1	SGK2|H-SG	0.366	0.706
NM_0001	chr8	18391245	18401213	+	1313	1	NAT2|AAC	0.02	0.115
NM_0220	chr6	35297818	35321771	+	2283	1	DEF6|IBP|	0.841	0.738
NR_02434	chr11	1.34E+08	1.34E+08	-	5305	1	MIR4697H	8.665	14.342
NM_0120	chrX	1.21E+08	1.21E+08	+	2332	1	GLUD2|GD	7.149	6.825
NR_03842	chr1	1.85E+08	1.85E+08	-	662	1	GS1-279B7	0	0.021
NR_03714	chr12	45173034	45216006	-	1866	1	PLEKHA8P	3.268	3.714
NM_1307	chr3	1.84E+08	1.84E+08	+	1688	1	HTR3C|-|-	0	0
NR_13618	chr7	49775333	49921950	+	2155	1	VWC2|PSS	0.628	1.182
NM_0010	chr6	54846771	54945099	+	6237	1	FAM83B|C	0	0.007
NM_0012	chr12	18080869	18090193	-	983	1	RERGL|-|-	0.354	0.265
NM_0010	chr5	1.43E+08	1.43E+08	-	4143	1	NR3C1|GC	0.324	0.397
NM_0140	chr2	96274336	96305569	-	7172	1	SNRNP200	94.765	87.144
NM_0008	chr9	1.37E+08	1.37E+08	+	3887	1	GRIN1|Glu	3.035	4.493
NM_0012	chr3	1.84E+08	1.84E+08	+	2618	1	PSMD2|PS	102.271	110.664
NM_0052	chr3	39329706	39333680	+	1483	1	CCR8|CC-C	0	0
NM_0047	chr4	20251905	20620561	+	8014	1	SLIT2|SLIL	12.284	5.927
NR_02409	chr21	25582775	25583224	-	449	1	LINC00515	1.062	0.641
NM_0011	chr17	8072636	8087703	-	2485	1	ALOX12B|	0.161	0.744
NR_04983	chr2	1.77E+08	1.77E+08	+	72	2	MIR4444-2	2.149	0.38
NM_0219	chr6	1.01E+08	1.02E+08	+	4574	1	GRIK2|EAA	4.412	3.827
NM_1336	chr5	1.29E+08	1.3E+08	+	5209	1	ADAMTS19	0.248	1.244
NM_0012	chr4	52743517	52751640	-	2860	1	ERVMER34	0.054	0.043
NM_0011	chr17	73334384	73644088	-	10676	1	SDK2|-|-|1	12.95	4.823
NM_1527	chr7	8433955	8752963	+	2916	1	NXPH1|NF	6.106	8.525
NM_0010	chr9	26947039	27062933	+	2105	1	IFT74|BBS	1.488	1.972
NM_0328	chr6	1.5E+08	1.5E+08	-	3609	1	LRP11|MA	7.052	7.165
NR_03400	chr3	1.57E+08	1.57E+08	-	2012	1	LINC0088C	0.122	0.225
NM_0011	chr20	38962338	39039723	+	3206	1	DHX35|C2	11.261	12.821
NR_11000	chr20	50930984	50945134	+	921	1	ADNP-AS1	1.092	1.294
NM_0042	chrX	1.51E+08	1.51E+08	+	2016	1	GPR50|H9	0.243	1.665
NM_0010	chr22_KI2	62553	66894	-	1491.3	7	CYP2D6|C	0.199	0.266
NM_0010	chr15	82130220	82262763	-	3512	1	EFL1|EFTU	6.406	7.09
NM_0012	chr19	8052762	8062663	+	991	1	CCL25|Ckb	1.119	1.451
NM_0012	chr12	25052247	25108335	+	2766	1	LRMP|JAW	0.098	0.035
NM_0210	chr17	42113111	42121364	-	3092	1	KAT2A|GC	14.399	17.454
NM_0046	chr3	50617131	50649297	+	2535	1	MAPKAPK3	14.277	10.682
NM_0011	chrX	65512582	65534806	-	2298	1	LAS1L|Las1	13.595	13.029
NR_12204	chr5	1.49E+08	1.49E+08	+	789	1	LOC25518	0	0
NR_11002	chr7	89882353	90211635	-	624	1	STEAP2-AS	0	0

NM_0013(	chr1	23964347	23980463	-	4169	1	SRSF10|FL	8.332	8.572
NM_0011‹	chr11	59107185	59127416	+	3346	1	FAM111B|	0.243	0.614
NM_0158‹	chr12	52768155	52777345	-	2518	1	KRT76|HU	0	0
NM_0012!	chr6	34537802	34556333	-	1859	1	SPDEF|PDI	0.153	0.317
NM_0206;	chr3	58237502	58294736	+	2414	1	ABHD6|-|-	12.648	35.638
NM_0011!	chr19	19528861	19537584	+	874	1	YJEFN3|-|-	6.639	5.673
NM_1735‹	chr10	73772291	73776218	+	2050	1	FUT11|-|-	6.887	3.702
NM_0244:	chr9	92383271	92404699	-	3435	1	OGN|OG|(	0.015	0
NM_0010:	chrX	1.55E+08	1.55E+08	+	514	3	H2AFB1|H	0	0.107
NR_14445	chr2	1.86E+08	1.86E+08	-	437	1	FSIP2-AS1|	0.266	0.251
NM_0028!	chr2	1.6E+08	1.6E+08	-	4261	1	RBMS1|C2	2.578	0.556
NR_11028	chr2	79493716	79500844	-	664	1	LOC10192	0.447	0.351
NR_02706	chr21	38739025	38768974	-	883	1	LINC00114	0	0
NR_02725	chr17	42874674	42898734	-	1838	1	LINC00671	0	0
NR_03412	chr1	1.12E+08	1.12E+08	-	1537	1	LINC0116C	0.067	0.018
NR_02436	chr7	1.29E+08	1.29E+08	+	10258	1	LINC0100C	2.898	3.409
NM_0144!	chr7	33129540	33606068	+	3876	1	BBS9|B1|(	7.744	6.721
NM_0012(	chr16	32674088	32676127	-	865	6	TP53TG3C	0	0
NR_02779	chr6	10427785	10434822	-	3015	1	LINC0051٤	0.009	0
NM_0064;	chr11	62771303	62787341	+	2102	1	TAF6L|PAI	19.604	17.437
NR_13459	chr6	1.49E+08	1.49E+08	-	687	1	LOC10537	0	0
NM_0013:	chr12	49989162	50025557	-	3097	1	RACGAP1|	13.293	16.643
NM_0319‹	chr19	17250040	17264735	-	2395	1	USHBP1|A	0.016	0.046
NM_0011‹	chrX	1.47E+08	1.47E+08	+	434	2	CXorf51A|	0	0
NM_1533:	chr10	1.23E+08	1.23E+08	+	1698	1	PSTK|C10c	3.03	4.219
NM_0011!	chr14	24369082	24379606	+	4573	1	NFATC4|N	5.633	5.643
NM_0189:	chr5	1.41E+08	1.41E+08	+	3405	1	PCDHB12|	1.132	1.251
NM_0056!	chr4	1.56E+08	1.56E+08	+	1689	2	TDO2|TDC	0	0
NM_0012;	chr9	1.2E+08	1.21E+08	-	5514	1	CDK5RAP2	10.616	8.776
NM_0021٤	chr5	1.59E+08	1.59E+08	-	2338	1	IL12B|CLN	0.011	0
NM_0334(	chr1	2590642	2633042	-	2876	2	MMEL1|N	0	0
NM_0188!	chr5	1.41E+08	1.41E+08	+	5469	1	PCDHAC1|	16.079	15.342
NR_11709	chr8	1.27E+08	1.27E+08	+	581	1	CASC21|C/	0.399	0.471
NR_11010	chr7	99013165	99036479	+	3394	1	LOC10192	3.901	4.451
NM_0063!	chr12	30628981	30695995	-	5314	1	IPO8|RANI	4.894	6.06
NM_0012!	chr1	41361931	41383591	+	2385	1	FOXO6|-|-	3.676	11.452
NR_04985	chr16	535316	535368	+	52	1	MIR5587|-	0	0
NR_03961	chr1	23929070	23929147	+	77	1	MIR378F|ı	0	0
NM_0013:	chr17	59707465	59842255	+	3661	1	VMP1|EPC	33.922	20.132
NM_0001:	chr13	1.03E+08	1.03E+08	+	4068	1	ERCC5|CO	11.48	11.017
NM_0011(	chr19	41431318	41439938	-	1556	1	ATP5SL|-|-	50.712	46.276
NR_03018	chr19	53679003	53679085	+	82	2	MIR515-1|	0	0
NM_1489(	chr1	51729814	51789219	+	3450	1	OSBPL9|O	17.434	15.313
NR_02702	chr17	56884102	56884913	-	811	1	MTVR2|-|-	0	0
NR_02712	chr3	54632124	54639857	-	3134	1	ESRG|HESI	0	0
NM_0013:	chr11	22820263	22829862	-	401	1	SVIP|-|-|1	23.986	8.983
NM_0011(	chr16	30659519	30670810	+	4088	1	FBRS|FBS|	28.241	21.33
NM_0012(	chr2	2.18E+08	2.18E+08	+	2365	1	CTDSP1|N	5.942	7.315
NM_1477;	chr11	65027408	65040572	+	1672	1	SNX15|HS/	23.967	20.349
NR_02969	chr14	1.01E+08	1.01E+08	+	81	1	MIR136|N	10.187	15.557

NR_13528	chr6_GL0C	138124	144314	-	578	2	LINC0053	0	0
NM_0033	chr14	96797347	96881614	+	1688	1	VRK1|PCH	6.37	17.835
NM_2033	chr12	77021246	77065580	-	5727	1	E2F7|-|-|1	1.562	1.284
NM_0010	chr5_GL33	965645	997896	+	1784	5	GTF2H2C_	0.239	0.33
NM_0010	chr16	3493484	3495421	-	900	1	C16orf90|	0	0.03
NM_0011	chr19	36445119	36489561	-	5246	1	ZNF566|-|	1.123	1.642
NR_13231	chr20	29080390	29096832	+	525	1	FRG1DP|-|	0	0
NM_0153	chr22	29058672	29061919	-	1065	1	C22orf31|	0.133	0.013
NM_0054	chr5	1.42E+08	1.42E+08	-	2288	1	GNPDA1|(	14.438	14.679
NM_0011	chr2	1.69E+08	1.69E+08	+	2250.5	2	NOSTRIN|	0.029	0.03
NM_0056	chr15	24954893	24978723	+	1550	1	SNURF|-|-	291.351	390.356
NM_0047	chr3	51933426	51941941	-	1565	1	RRP9|RNU	11.765	9.689
NM_0010	chr17	58541587	58544372	-	2123	1	C17orf47|	0.036	0.032
NM_0123	chrX	1.41E+08	1.41E+08	-	1466	1	LDOC1|BC	47.906	64.327
NM_0063	chr12	6970781	6976003	+	1064	1	EMG1|C2F	16.941	15.744
NM_0011	chrX	14529298	14731811	+	3005	1	GLRA2|GL	5.381	4.868
NM_0011	chr22	31160152	31207019	+	3037	1	RNF185|-|	18.374	23.479
NM_0013	chr1	84506301	84556495	+	2140	1	SPATA1|SI	0	0.045
NM_0124	chr10	22345445	22417610	+	2606	1	SPAG6|CT	2.033	0.599
NR_13657	chrX	6226358	6230291	+	3933	1	LOC10537	0.843	0.665
NR_10349	chr12	1.22E+08	1.22E+08	+	2350	1	LOC10159	0.664	1.16
NR_14446	chr22	47345571	47373541	+	4358	1	LOC33968	0.006	0
NM_0309	chr6	29044213	29045175	-	961	8	OR2W1|hs	0	0
NR_12054	chr11	10809297	10822931	+	2332	1	LOC10192	0.282	0.229
NM_0012	chr15	90966038	90994651	-	2933	1	PRC1|ASE	13.812	13.529
NM_0010	chr14	57390553	57412748	+	4468	1	NAA30|C1	3.411	3.82
NM_0012	chr3	48446710	48465655	+	2387	1	ATRIP|-|-|	5.294	6.926
NR_12585	chr6	11990343	12001244	-	382	1	LOC10192	0	0
NM_0010	chr15	54012648	54628608	+	8363	1	UNC13C|-	0.318	0.165
NM_0011	chr9	1E+08	1E+08	+	1766	1	MSANTD3	31.248	29.891
NM_0010	chr1	13389632	13392629	+	1573	1	PRAMEF17	0	0
NM_0013	chr19	35856908	35867146	+	2827	1	KIRREL2|F	44.122	36.164
NM_0013	chr2	88027203	88055801	-	1760	1	KRCC1|CH	6.242	4.942
NM_0071	chrX	47836902	47922256	+	7852	1	ZNF81|HF	1.128	2.037
NR_02448	chr1	2350415	2352661	-	2246	1	LOC10012	3.255	2.641
NM_0189	chr15	24675394	24683446	+	8050	1	NPAP1|C1	0.056	0.014
NM_0321	chr16	67662945	67666756	-	1562	1	ENKD1|C1	19.869	12.399
NM_0042	chr17	28744005	28750958	+	2893	1	TRAF4|CAI	59.694	64.948
NR_00335	chr15	25245478	25245559	+	81	1	SNORD115	0	0
NR_03979	chr7	67114322	67114397	-	75	2	MIR4650-2	0	0
NR_04512	chrY	19567358	19590423	+	852	1	TXLNGY|C	2.89	3.296
NM_0177	chr2	85341955	85354698	-	3298	1	RETSAT|-|	12.053	10.159
NM_0189	chr5	1.41E+08	1.42E+08	+	4635	1	PCDHGC5|	20.068	80.3
NR_10414	chr3	1.59E+08	1.59E+08	-	1635	1	LOC10099	1.183	0.771
NM_0026	chr19	43648571	43670346	-	1544	1	PLAUR|CD	2.013	1.517
NM_0038	chr8	23135591	23164030	-	3524	1	TNFRSF10I	0.117	0.241
NM_0036	chr17	79833156	79839414	-	2642	1	CBX4|NBP	26.48	37.825
NR_00303	chr2	2.02E+08	2.02E+08	+	83	1	SNORD11|	0	0.33
NR_02969	chr15	89368017	89368106	+	89	1	MIR9-3|M	0	0
NM_0011	chr3	1E+08	1E+08	+	3865	1	TBC1D23|	3.386	3.948

NR_03648	chr13	1.02E+08	1.02E+08	-	2126	1	FGF14-IT1	0.036	0.032
NM_1748!	chr1	77281977	77559969	+	3309	1	AK5|AK6|-	2.018	4.818
NR_11056	chr2	85889280	85892252	+	1113	1	ST3GAL5-A	0	0.098
NM_0028:	chr2	1.2E+08	1.2E+08	+	11351	1	PTPN4|ME	1.026	2.456
NR_13625	chr7	35695206	35699412	+	4206	1	LOC10193	4.181	4.025
NM_0054:	chr18	721592	812326	-	4671	1	YES1|HsT4	3.141	4.41
NM_0002!	chr6	49430360	49463328	-	3871	1	MUT|MCN	9.476	8.715
NM_0011!	chr2	74549020	74557551	+	1969	1	DOK1|P62	5.848	4.995
NR_13506	chr10	1.01E+08	1.01E+08	+	2190	1	KAZALD1|I	1.601	2.095
NR_04024	chr18	11883471	11908797	-	2981	1	MPPE1|PG	8.417	9.511
NM_0309	chr17	41139433	41140487	-	1053	1	KRTAP4-6|	0	0
NM_0012	chr10	77636201	77637819	-	920	1	KCNMA1|I	2.649	2.159
NR_10701	chr3	1.28E+08	1.28E+08	-	65	1	MIR7976|I	0	0
NM_0010	chr12	1.21E+08	1.21E+08	+	4397	1	UNC119B|	19.74	17.317
NR_02834	chr4	1.78E+08	1.78E+08	+	1028	1	LINC0109	0	0.027
NR_03960	chr10	23393405	23393467	+	62	1	MIR1254-2	0	0
NM_0012!	chr14	81260650	81427404	-	4298	1	STON2|ST	2.775	4.031
NM_1384(	chr7	1.57E+08	1.57E+08	+	6064	1	NOM1|C7	2.016	2.43
NM_1446(	chr16	88570381	88631964	+	3707	1	ZC3H18|N	22.316	23.193
NM_0001	chr17	8002670	8020340	+	3601	1	GUCY2D|C	0.004	0.008
NR_10467	chr5	1.16E+08	1.16E+08	-	694	1	LOC10246	0	0
NM_0011:	chr12	1.1E+08	1.1E+08	-	2198	1	ANAPC7|A	18.865	22.658
NM_2125!	chr19	45178745	45181801	+	2597	1	BLOC1S3|I	4.597	6.023
NM_0010:	chr11	44565591	44619765	+	1605	1	CD82|4F9	6.627	5.18
NR_00271	chr2	2.18E+08	2.18E+08	-	2135	1	CXCR2P1|	0	0
NM_2034:	chr9	1.29E+08	1.29E+08	-	2707	1	IER5L|bA2	15.804	14.785
NM_0012	chr1	39678648	39691485	-	4431	1	HPCAL4|H	16.548	19.811
NR_03897	chrX	1.04E+08	1.04E+08	+	2867	1	MORF4L2-	0.414	0.263
NM_0141	chr5	1.55E+08	1.55E+08	+	3164	1	MRPL22|H	7.584	7.017
NR_03390	chr8	22604632	22620470	+	3788	1	CCAR2|DB	79.611	75.93
NM_1525!	chr5	1.46E+08	1.46E+08	+	3031	1	SH3RF2|H	0.03	0.127
NR_03167	chr9	1.28E+08	1.29E+08	-	44.7	6	MIR1268A	8.371	0.92
NM_0039!	chr11	67428464	67435408	+	1753	1	RPS6KB2|I	16.608	16.455
NM_1824	chr21	10413532	10491615	+	1879	1	BAGE3|CT	0	0.007
NM_0011	chrX	1.36E+08	1.36E+08	-	1331	3	CT45A5|C	0	0
NM_0011	chr14	91779752	91867536	-	5001.5	2	TC2N|C14	0	0
NM_0011	chr8	69466624	69660912	+	5524	1	SULF1|SUL	0.348	0.092
NR_03650	chr19	36489627	36491040	+	1413	1	LOC72875	1.478	1.609
NR_04520	chr15	74361552	74362041	+	489	1	LOC72973	0.079	0.336
NR_13463	chr3	48290279	48291678	-	1399	1	FCF1P2|-|	4.663	3.73
NM_0327!	chr19	3769091	3772221	-	2148	1	RAX2|ARN	0	0
NR_00314	chr15	35237326	35238063	+	737	1	ANP32AP1	8.432	10.11
NM_0036	chr11	313991	315272	+	713	1	IFITM1|9-2	21.338	13.543
NM_0248!	chr1	35931082	36056462	+	3624	1	AGO3|EIF2	2.007	2.581
NM_0164!	chr2	85595714	85597708	+	709	1	RNF181|H	70.214	58.304
NR_13524	chr2	1.59E+08	1.59E+08	-	4063	1	PKP4-AS1|	0.403	0.711
NR_03390	chr1	764865	778688	-	1364	1	LOC10028	0.047	0.05
NM_0012!	chr7	64313873	64337362	+	879	1	ZNF736|-|	1.57	2.462
NM_1451(	chr16	81997646	82011488	-	2770	1	SDR42E1|I	0	0.01
NR_00300	chr17	7906123	7906260	+	137	1	SCARNA21	0.847	1.2

NM_0013(	chr11	57386787	57390657	-	1419	1	PRG2|BMI	0.018	0.019
NM_0011	chr16	474850	522481	+	3192	2	RAB11FIP3	0.008	0.004
NM_0013	chr17	42833397	42843760	+	3435	1	PSME3|HE	46.394	56.535
NM_0013(	chr1	54045486	54053438	-	841	1	TMEM59|	64.529	62.198
NR_02937	chr17	80351831	80415168	-	3696	1	LOC10029	1.657	1.216
NM_0030	chr11	1.08E+08	1.08E+08	-	726	1	SLN|-|-|1	0	0.755
NM_0163	chr7	1.3E+08	1.3E+08	+	2804	1	CPA4|CPA	0.179	0.142
NR_02984	chr1	40754355	40754446	+	91	1	MIR30E|M	0	0
NR_00153	chrY	6390401	6411564	+	935	2	TTTY1|LIN	0	0
NM_0309	chr2	30231531	30260033	+	2933	1	LBH|-|-|2	76.978	104.248
NM_1816(	chr21	30430276	30430758	+	481	1	KRTAP13-4	0	0
NM_0329	chrY	5056090	5742228	+	9036	1	PCDH11Y|	0.074	0.065
NR_13615	chr10	1.28E+08	1.28E+08	-	1092	1	AS-PTPRE|	0	0
NM_0121	chr17	73283624	73312004	-	3097	1	CDC42EP4	64.992	54.257
NR_03164	chr2	1.89E+08	1.89E+08	+	68	1	MIR1245A	0	0
NM_0325	chr12	1.23E+08	1.23E+08	-	2974	1	HCAR1|FK	0	0
NM_1446	chr2	1.98E+08	1.98E+08	-	3102	1	RFTN2|C2	2.099	2.243
NM_0314(	chrX	53532096	53686729	-	14655	1	HUWE1|A	39.543	39.795
NR_12053	chr11	8060038	8069373	+	1961	1	TUB-AS1|-	0.013	0
NR_02456	chr20	19242302	19284596	-	1781	1	LOC10013	0	0.008
NR_02725	chr2	55282319	55284471	+	2152	1	PRORSD1P	0.132	0.14
NM_0249	chr9	83980722	84004072	+	3489	1	RMI1|BLA	2.967	3.49
NM_1390	chr2	2.29E+08	2.3E+08	-	3257	1	DNER|UN	231.936	294.808
NR_04679	chr6	1.66E+08	1.66E+08	-	575	1	RPS6KA2-I	0.807	0.524
NM_0014	chr6	10555716	10629368	+	4667	1	GCNT2|CC	4.404	5.752
NM_0013	chr19	36388630	36418674	-	5967	1	ZFP82|ZN	1.312	3.31
NM_0044	chr17	7225342	7234544	-	2999	1	DVL2|-|-|	25.374	23.631
NM_0011	chr19	10848430	10869684	+	1080	1	C19orf38|	1.17	0.672
NM_0010	chr1	1.98E+08	1.98E+08	+	678	1	C1orf53|-|	6.276	1.434
NM_0010	chr2	54725012	54972020	+	7809	1	EML6|-|-|	0.829	1.41
NM_0016	chr12	49961496	49965682	+	1819	1	AQP5|AQ	0.035	0.03
NR_10405	chr13	87801043	87810520	-	368	1	LINC00397	0	0
NM_1721(	chr20	63400214	63472677	-	9152	1	KCNQ2|BF	36.526	52.721
NM_0031(	chr12	53380644	53416442	+	7586	1	SP1|-|-|12	8.279	8.313
NM_0061	chr1	1.13E+08	1.13E+08	+	2728	1	CAPZA1|C	28.538	18.803
NM_0013(	chr19	39342459	39384847	+	3504	1	SAMD4B|	27.318	24.998
NM_0240	chr13	44143151	44161257	-	1209	1	SMIM2|C1	0.064	0.34
NM_0012	chr2	42568045	42756947	+	5465	1	MTA3|-|-|	11.754	13.933
NM_0047	chr13	33016433	33066145	+	4999	1	KL|-|-|13c	0.005	0.003
NR_10377	chr5	6686325	6707711	-	3361	1	LINC02102	0.008	0.008
NR_02439	chr5	77086798	77148351	+	1138	1	ZBED3-AS1	0.623	1.252
NM_0009	chr9	1.27E+08	1.27E+08	-	651	1	RPL12|L12	177.776	157.211
NR_03967	chr8	42896197	42896275	-	78	1	MIR4469|	1.322	0
NR_12037	chr15	84422519	84425882	+	1307	1	LOC10317	0	0
NR_03741	chr22	20085746	20085833	+	87	1	MIR3618|	3.26	5.195
NM_0328	chr11	12286900	12359144	+	3033	1	MICALCL|	0.03	0.072
NM_0011	chr1	1.93E+08	1.93E+08	-	1478	1	UCHL5|CG	4.763	8.192
NR_04628	chr10	21174302	21174923	+	512	1	NEBL-AS1|	0.705	0.294
NR_00394	chr1	1.74E+08	1.74E+08	-	70	1	SNORD80|	3.868	4.109
NM_0154	chr3	1.01E+08	1.01E+08	-	4436	1	ABI3BP|NI	0.14	0.015

NR_04684	chr1	1.72E+08	1.72E+08	+	389	1	DNM3-IT1	0.133	0.07
NR_03606	chr1	1.7E+08	1.7E+08	-	84	1	MIR3119-1	0	0
NM_0306	chr22	35717872	35729482	+	1430	2	APOL5|AP	0	0
NM_0011	chr15	74409284	74433704	-	3262	1	SEMA7A|C	4.712	14.26
NM_0010	chr6	78867544	78900966	+	3442	1	IRAK1BP1|	1.39	1.269
NM_0220	chr20	62952647	62968597	+	2577	1	SLC17A9|C	0.015	0.043
NM_0010	chr16	75538117	75556286	-	2896	1	TMEM231	6.674	6.177
NM_0010	chr19	34758074	34773229	-	3109	1	ZNF599|-|	2.343	3.397
NM_0010	chr8	1.17E+08	1.17E+08	+	1782	1	AARD|C8o	0.022	0.169
NM_0010	chr16	2019520	2020755	+	1012	1	NPW|L8|L	0.42	0.325
NR_13452	chr20	1947210	2007517	+	1834	1	LOC72799	0	0
NM_0141	chr6	96889311	96897891	-	2406	1	NDUFAF4|	7.577	7.594
NM_0053	chr3	1.22E+08	1.22E+08	-	2023	1	HCLS1|CT	0.057	0.074
NR_04988	chr1	1.54E+08	1.54E+08	-	71	1	MIR5698|	0	0
NR_02679	chr10	26931206	26942001	-	3615	1	LINC00202	0.075	0.121
NM_0012	chr12	49366905	49527426	+	3720	1	SPATS2|N	22.986	23.926
NR_12656	chrX	15602881	15621484	+	572	1	GS1-594A7	0	0
NR_02433	chr14	65410593	65412617	-	2024	1	FUT8-AS1|	0.956	0.596
NM_0151	chr10	806914	931705	-	8526	1	LARP4B|K	6.754	7.268
NR_00293	chr19	7787484	7791012	+	1928	1	CLEC4GP1	0.007	0
NM_0062	chr8	17576433	17643133	+	1896	1	PDGFRL|P	0.415	0.52
NR_14450	chr11	925809	1012245	+	4279	3	AP2A2|AD	2.796	2.513
NR_03740	chr17	74748613	74748699	+	86	1	MIR3615|	0	0
NM_0011	chr16	31435564	31443027	-	2009	1	ZNF843|-|	2.163	0.914
NM_0011	chr7	99173574	99207489	-	1696	1	KPNA7|IP	0.015	0.016
NR_10242	chr8	12794243	12818291	+	502	1	LINC00681	0.488	1.828
NM_0010	chr9	1.23E+08	1.23E+08	+	961	1	OR5C1|OF	0	0
NM_0012	chrX	71366239	71466005	+	7683	1	TAF1|BA2	5.048	4.648
NR_02691	chr8	1.43E+08	1.43E+08	-	465.5	2	LOC10013	0	0.118
NM_0013	chr1	40477630	40496343	+	2154	1	ZFP69|ZFP	2.334	2.232
NM_0011	chr15	40351033	40356433	+	430	1	PHGR1|-|-	0	0
NR_11018	chr7	1.25E+08	1.25E+08	-	1727	1	LOC10192	0.015	0
NR_12634	chr7	1.56E+08	1.56E+08	-	1382	1	LOC10050	1.698	1.397
NR_03337	chr9	1.28E+08	1.28E+08	-	3837	1	SLC25A25-	3.444	3.655
NM_0041	chr11	61792637	61797242	+	2290	1	FEN1|FEN	25.494	16.903
NM_0010	chr2	3575263	3580919	+	719	1	RPS7|DBA	25.895	23.47
NR_03895	chr9	1.29E+08	1.3E+08	+	2117	1	LINC00963	3.167	3.772
NM_0011	chr3	48403854	48430051	-	7103	1	PLXNB1|P	35.195	26.843
NM_0013	chr16	19716456	19858467	+	3386	1	IQCK|-|-|1	12.764	8.867
NM_0012	chr9	20341665	20621987	-	6675	1	MLLT3|AF	4.321	5.973
NM_0003	chr2	1.89E+08	1.89E+08	-	6874	1	COL5A2|E	2.403	2.148
NM_0012	chr6	1.6E+08	1.6E+08	+	1821	1	PNLDC1|-|	0.078	0.196
NM_1827	chr8	22024843	22036897	+	1089	1	NPM2|-|-	2.06	3.396
NR_12635	chr2	6828554	6840464	-	828	1	NRIR|lncR	0	0.033
NM_0309	chr17	41040559	41041461	-	900	2	KRTAP1-1|	0	0
NM_0011	chr1	1534778	1540360	-	1111	1	TMEM240	7.95	8.285
NR_12575	chr11	1.25E+08	1.25E+08	-	583	1	PKNOX2-A	0.288	0.446
NR_03966	chr5	8460925	8460999	+	74	1	MIR4458|	0	0.185
NM_1758	chr7	18026776	18027863	-	1085	1	PRPS1L1|F	0.606	0.909
NR_10463	chr5	38710367	38720273	-	795	1	LINC01265	0	0.069

NM_0005{	chr4	73740506	73743716	+	1699	1	CXCL8|GCI	0.212	0.056
NM_0324(	chrX	1.41E+08	1.41E+08	+	465	1	SPANXB1|	0	0.059
NM_0013:	chr6	96924620	97114742	+	1835	1	KLHL32|BI	2.937	2.971
NR_02678	chr3	1.39E+08	1.39E+08	+	3511	1	BPESC1|N	0	0
NM_0329(	chr4	1.85E+08	1.85E+08	-	2497	1	CASP3|CPI	27.93	20.34
NR_10426	chr21	41798225	41863302	-	4966	1	PRDM15|(	2.931	3.158
NM_0011(	chr17	4951089	4957131	+	1344	1	ENO3|GSD	10.802	5.972
NM_0035:	chr10	1.33E+08	1.33E+08	+	1153	1	UTF1|-|-|:	0.011	0.012
NR_13233	chrX	1.16E+08	1.16E+08	-	334	1	DANT2|-|-	0.077	0.328
NM_0151(	chr19	47064190	47113752	-	6099	1	ZC3H4|C1!	16.035	15.146
NM_1383!	chr19	10928748	10930240	+	1392	1	TIMM29|(	11.411	10.607
NM_0143:	chr22	35541305	35553998	+	2967	1	RASD2|M(	11.859	9.39
NM_0011!	chr18	49481178	49491584	-	1932	1	RPL17-C18	36.518	36.185
NM_1449!	chr17	17212213	17237188	-	3670	1	FLCN|BHD	13.709	14.499
NM_1537!	chr1	2.07E+08	2.07E+08	+	1024	1	IL19|IL-10	0	0
NM_0056{	chr2	9843442	9934416	+	2282	1	TAF1B|MG	2.644	2.815
NR_03164	chr4	1.82E+08	1.82E+08	+	85	1	MIR1305|	0	0
NM_0022!	chr2	17878657	17932958	+	2346	1	KCNS3|KV	0.275	0.607
NM_0013<	chr6	2663629	2770330	-	5833	1	MYLK4|Sg	3.282	3.907
NR_03318	chr1	1615415	1630610	+	3101	1	MIB2|ZZAI	17.434	16.387
NM_0008:	chr5	1.33E+08	1.33E+08	-	810	1	IL5|EDF|IL	0	0.017
NM_0211:	chr7	45574526	45723115	+	12476	1	ADCY1|AC	19.793	20.688
NM_0010{	chr14	94612377	94624053	+	1583	1	SERPINA3|	3.331	0.13
NR_04567	chr20	31421439	31429180	-	493	1	DEFB122|	0	0
NM_0011(	chr17	74737217	74747335	+	2531	1	RAB37|-|-	0.229	0.233
NM_0012!	chr1	2.18E+08	2.18E+08	-	3230	1	GPATCH2|	3.329	2.604
NR_02742	chr8	7955013	8008755	+	1427	1	FAM66E|-	0	0
NM_0013(	chr22	30925130	30968287	-	5133	1	MORC2|CI	21.976	25.726
NM_0146(	chr4	88592423	88708542	+	4946	1	HERC3|-|-	4.525	4.539
NR_12049	chr10	1.22E+08	1.22E+08	+	949	1	ATE1-AS1|	1.467	0.794
NM_0055:	chr1	1.2E+08	1.2E+08	-	2454	1	HMGCS2|-	0.205	0
NM_0010:	chr5	1.31E+08	1.31E+08	+	3480	1	CDC42SE2	9.618	12.5
NM_0184:	chr6	24667035	24705067	+	4099	1	ACOT13|H	10.478	8.615
NM_0000<	chr11	1.17E+08	1.17E+08	+	527	1	APOC3|AP	0	0
NM_0011(	chr4	86935002	87141054	+	9262	1	AFF1|AF4|	2.787	3.042
NR_03840	chr1	21293290	21299869	+	2571	1	LOC10050(	0.115	0.133
NM_0020!	chr4	70815782	70839910	-	6619	1	GRSF1|-|-	15.093	17.801
NR_02676	chr3	1.09E+08	1.09E+08	+	2521	1	LINC0048{	0.092	0.163
NM_0012(	chr4	1.11E+08	1.11E+08	-	1726	1	PITX2|ARF	0.217	0.23
NM_0056!	chr22	42160013	42283927	-	7336.7	3	TCF20|AR:	0.014	0.019
NR_02460	chr8	48723394	48735311	-	1854	1	EFCAB1|-|	0.153	0.052
NR_07344	chr10	78267328	78280213	+	871	1	LINC0059!	0	0.173
NR_13513	chr9	41694357	41699001	-	339	1	LOC10537(	0	0
NM_0142:	chr19	52189802	52226425	+	2492	1	PPP2R1A|	300.865	338.902
NM_0060:	chr6	1.09E+08	1.09E+08	-	3357	1	CD164|DF	4.348	4.247
NR_11022	chr2	64846130	64863631	-	1977	1	LINC0180C	0.059	0.021
NR_10463	chr9	36136536	36163913	+	1886	1	GLIPR2|C9	10.05	7.488
NR_03968	chr20	29880384	29880463	+	79.7	3	MIR4477B	0	0
NM_0251<	chr2	1.97E+08	1.97E+08	+	2233	1	COQ10B|-	4.758	5.257
NM_0160:	chr8	78666047	78719762	+	3338	1	ZC2HC1A|	7.165	7.07

NM_0011!	chr16	11965107	11968068	+	989	1	TNFRSF17	0	0.028
NM_0153:	chr6	96521826	96555275	+	4184	1	UFL1|KIAA	1.861	2.085
NM_0309:	chr1	1.51E+08	1.51E+08	-	3905	1	SEMA6C|S	65.283	54.848
NM_0325!	chr14	35534042	35537054	+	3010	1	INSM2|IA-	0.304	0.109
NM_1532:	chr1	34759741	34763724	+	2836	1	GJB4|CX3(	0.014	0.058
NR_10994	chr5	29065244	29073223	+	4638	2	LOC10192!	0	0
NM_0153⁄	chr6	42821056	42868560	+	6503	1	GLTSCR1L|	5.667	6.376
NR_13429	chr8	49496763	49512180	-	265	1	LOC10050	0	0
NM_0037!	chr19	10115014	10119924	-	1115	1	EIF3G|EIF3	119.117	110.694
NR_03161	chr7	92204015	92204098	-	83	1	MIR1285-1	0.621	0.165
NR_02989	chr7	1.3E+08	1.3E+08	+	93	1	MIR335|M	0	0
NR_12578	chr13	79804386	79805682	+	455	1	LINC01038	0	0
NR_10274	chr15	82471482	82513995	-	2849	3	LOC72775	1.145	1.288
NM_1779!	chr10	46888	49238	-	1499	1	TUBB8|OC	0.017	0.082
NM_0141⁄	chr7	74209757	74229834	+	1987	1	LAT2|HSP(	0.519	0.476
NM_0010(	chr14	20242792	20243766	+	973	1	OR11H4|C	0	0.028
NR_02724	chr16	1064081	1078731	-	2930	1	SSTR5-AS1	0.018	0.014
NR_13285	chr13	84783654	84783778	+	124	1	SNORA107	0	0
NM_0012!	chr22	27851669	27919214	-	2928	1	PITPNB|PI	21.538	20.887
NR_03793	chr6	1.34E+08	1.34E+08	-	2189	1	HMGA1P7	0.047	0.1
NM_1528:	chr1	26111777	26125548	+	4148	1	PDIK1L|CL	3.898	5.283
NM_0011:	chr13	20221962	20232395	-	1901	1	GJB6|CX3(	0	0
NM_0011:	chr3	49199500	49258104	+	2686	1	CCDC36|C	0.024	0.025
NM_0010(	chr12	55492378	55493316	+	937	1	OR6C68|-|	0	0
NR_02960	chr2	1.76E+08	1.76E+08	+	109	1	MIR10B|M	0	0
NM_0011⁄	chr15	78959947	79090873	-	6317	1	RASGRF1|	1.645	1.347
NR_02411	chr1	1.56E+08	1.56E+08	+	2219	2	MSTO2P|N	3.178	3.636
NM_0011⁄	chr19	57240685	57262738	+	9994	1	ZNF805|-|	1.097	1.148
NM_0011:	chr20	1474591	1491587	-	2532	1	SIRPB2|PT	0	0
NM_0051:	chr14	92794231	92839961	+	2952	1	GOLGA5|G	12.177	12.124
NM_0040(	chr12	12717270	12722382	+	2517	1	CDKN1B|C	36.811	46.092
NR_11061	chr1	60953738	60970776	-	1111	1	NFIA-AS2|	0.139	0.222
NM_0152:	chr17	2689386	2711633	-	5196	1	CLUH|CLU	9.946	12.002
NM_0010(	chr2	54253178	54256272	-	3092	1	TSPYL6|-|-	0.017	0.013
NM_0001(	chr7	41960950	42237019	-	8197	1	GLI3|ACLS	7.413	12.882
NM_0010(	chr2	1.71E+08	1.71E+08	+	1943	1	SP5|-|-|2c	1.891	0.959
NM_0013:	chr10	31320113	31529814	+	5954	1	ZEB1|AREI	8.456	5.098
NR_13520	chr14	1.06E+08	1.06E+08	+	1663	2	LOC10537(	0.047	0.033
NM_0212:	chr3	53884976	53892015	-	1087	1	SELENOK|	51.693	44.783
NM_1736:	chr1	1.44E+08	1.44E+08	-	4251	1	NBPF15|A	2.715	3.222
NR_00358	chrY	6390401	6411564	+	935	2	TTTY1B|TT	0	0
NR_13431	chr10	83672406	83677089	-	505	1	LOC10537:	0	0
NR_11081	chr3	20342468	20350928	+	2036	2	LOC10192	0	0
NM_0010(	chr1	1.19E+08	1.19E+08	+	1690	1	HAO2|GIG	0	0
NM_0012(	chr2	1.97E+08	1.98E+08	+	4268	1	HSPE1-MC	12.474	12.42
NM_0011(	chr1	1.61E+08	1.61E+08	+	1419	1	PCP4L1|IC	8.796	17.896
NM_0058:	chr1	1.67E+08	1.67E+08	-	2793	1	GPA33|A3	0.203	0.083
NR_03414	chr20	58515379	58619892	+	2089	1	APCDD1L-/	0.099	0.118
NM_0054!	chr19	6752162	6767512	-	2344	1	SH2D3A|N	0.061	0.076
NM_0012(	chr2	1.55E+08	1.55E+08	+	1141	1	KCNJ3|GIF	1.153	1.008

NM_1788	chr16	29906336	29926232	-	1717	1	KCTD13|B	27.597	43.676
NM_0064	chr21	29024617	29054486	+	2953	1	USP16|UB	3.576	4.949
NR_13523	chr14	73822559	73830135	-	1172	1	LOC10050	0	0
NM_0010	chr7	99558825	99564705	+	1194	1	ZNF655|V	8.207	5.896
NM_0307	chr12	26120026	26125070	-	3773	1	BHLHE41|	1.179	1.213
NM_0003	chr22	42617840	42649399	-	2912	1	CYB5R3|B	74.793	74.002
NR_10242	chr8	1.43E+08	1.43E+08	+	1835	1	CDC42P3|	0.021	0.067
NM_0011	chr7	1.57E+08	1.57E+08	+	1737	1	RNF32|FK	1.314	1.08
NR_10408	chr14	32203163	32203269	+	105	11	RNU6-8|U	1.105	0.783
NM_0181	chr8	42836674	42843331	-	2162	1	THAP1|DY	3.584	3.732
NM_0062	chr1	56645317	56715335	+	9341	1	PRKAA2|A	0.647	0.688
NM_0178	chrX	54068403	54183258	-	7963	1	FAM120C|	1.114	1.975
NM_0012	chr20	56468585	56518886	+	1641	1	RTFDC1|C	81.91	85.437
NM_0143	chr1	1.53E+08	1.53E+08	+	607	1	LCE2B|LEP	0	0
NM_0010	chr16	67657512	67660815	-	2004	1	ACD|PIP1|	23.484	22.206
NM_0212	chr6_GL0C	3057233	3059679	+	398	7	LY6G6D|C	0	0
NM_0033	chr3	48598999	48609665	-	1602	1	UQCRC1|D	129.981	114.432
NR_13296	chrX	55183481	55183607	-	126	1	SNORA10	0.205	0.435
NR_00001	chr19	49491175	49491260	+	85	1	SNORD35	3.641	7.413
NR_13553	chr3	66730378	66738336	-	486	1	LOC10537	0	0
NR_02671	chr21	37208503	37221736	+	1641	1	DSCR9|NC	0.416	0.242
NR_00336	chr8	1.28E+08	1.28E+08	+	1931	1	PVT1|LINC	1.576	1.894
NR_02684	chr1	1.68E+08	1.68E+08	-	1849	1	ANKRD36E	0.934	1.289
NM_0057	chr2	10783391	10812839	-	2318	1	PDIA6|ERF	85.159	87.66
NR_03069	chr17	7930345	7931999	-	684	1	TRAPPC1|	158.322	139.613
NM_0062	chr11	65333754	65352980	+	2517	1	DPF2|REQ	56.737	61.307
NM_0319	chr19	37884823	37906706	-	8977	1	WDR87|N	0.019	0.003
NM_0017	chr7	1.29E+08	1.29E+08	-	1092	1	OPN1SW|	2.267	1.606
NM_1386	chr16	69106226	69118716	+	1185	1	HAS3|-|-|	16.865	17.419
NR_04668	chr3	73623568	73627899	+	2884	1	PDZRN3-A	0.076	0.157
NM_0049	chr19	50906352	50910738	-	1341	1	KLK4|AI2A	0.125	0.204
NR_13632	chr2	3974161	3975280	+	600	1	LOC10537	0	0
NM_0010	chr3	1.27E+08	1.27E+08	+	1735	1	C3orf56|-|	0	0
NR_04066	chr6_GL0C	2940505	2943134	+	2268.5	4	HCP5|6S2	0	0
NR_12616	chr16	90102264	90177606	+	957	1	FAM157C|	0	0.014
NR_04520	chr19	34645873	34646052	+	179	1	SCGB2B3P	0	0
NM_0052	chr21	38805831	38824954	+	3753	1	ETS2|ETS2	3.916	5.066
NR_02776	chr8	55449197	55455321	-	6124	1	SBF1P1|-|	0.619	0.662
NM_1528	chr8	81799583	81842286	-	3200	1	SNX16|-|-	0.899	1.109
NM_0016	chr3	1.14E+08	1.14E+08	+	4576	1	ATP6V1A|	39.658	42.333
NM_0022	chr6	33621379	33696571	+	8971	1	ITPR3|IP3	1.291	2.605
NM_2073	chr10	84173798	84185294	+	823	1	C10orf99|	0	0
NM_0037	chr1	1.93E+08	1.93E+08	-	3544	1	B3GALT2|	3.111	6.079
NM_0067	chr22	20982269	20999037	+	4292	1	LZTR1|BTE	28.41	26.376
NR_11074	chr19	23015075	23023697	-	1020	1	LINC0185	0	0
NM_0021	chr5	55024279	55034132	+	1038	1	GZMK|TRY	0.05	0
NM_0013	chr17	5471251	5486230	-	4210	1	DERL2|CG	8.646	8.804
NM_0186	chr6	73394562	73417566	+	2677	1	DDX43|CT	0	0
NR_02703	chr13	90890954	90926597	-	2012	1	LINC0041	0	0
NM_0012	chr5	1.76E+08	1.76E+08	-	1052	1	NOP16|HS	25.481	19.009

NM_2032!	chr6	43517024	43521526	+	1342	1	POLR1C|A	19.148	18.453
NM_1721!	chr14	49693105	49753152	+	2676	1	KLHDC1|M	0.53	0.292
NM_0010(	chr11	59456961	59457905	+	943	1	OR4D6|OF	0	0
NM_0062:	chr11	1.11E+08	1.11E+08	-	3024	1	POU2AF1|	0	0.005
NR_03887	chr21	45827841	45836419	-	3583	1	LOC10012!	0.061	0.015
NM_0013(	chr8	62248942	62966472	+	1845	1	NKAIN3|F/	2.362	3.92
NM_0013:	chr19	46602069	46610782	+	2132	1	CALM3|Ca	607.282	640.599
NM_0022{	chr12	52285913	52291515	-	1899	1	KRT81|HB	0	0
NM_0068:	chrX	48913182	48919136	-	2179	1	PIM2|-|-|)	5.426	4.84
NM_0010(	chr2	2.34E+08	2.34E+08	-	1263	1	DNAJB3|H	0	0
NM_0012!	chr1	1.6E+08	1.6E+08	+	2505	1	NCSTN|AT	51.461	48.434
NM_0011/	chr1	1.55E+08	1.55E+08	+	2204	1	DCST1|-|-	0.521	0.28
NM_1815:	chr16	71929538	71999978	-	5168	1	PKD1L3|-|	0.115	0.093
NM_0066{	chr14	20745940	20748380	+	875	1	EDDM3A|I	0	0
NM_0057:	chr2	2.18E+08	2.18E+08	+	1398	1	ARPC2|AR	196.003	210.207
NM_0010:	chr16	75204096	75207174	-	881	1	CTRB2|-|-	0.073	0.233
NM_0023:	chr17	34980498	35003172	+	3081	1	LIG3|LIG2	15.722	15.849
NM_0013/	chr17	2337514	2381054	+	3872	1	SGSM2|RU	37.019	37.822
NM_0013(	chr20	1180561	1185433	-	1319	1	TMEM74B	6.911	8.224
NM_0013:	chr15	75649007	75658627	+	3006	1	SNX33|SH:	4.195	4.907
NR_02942	chrX	73524275	73563085	-	2537	1	MAP2K4P1	0.915	0.588
NR_10802	chr5	1.7E+08	1.7E+08	+	2150	1	LINC01366	0.03	0
NM_0012/	chr11	67627939	67629937	-	738	1	NUDT8|-|-	5.154	1.875
NM_0010:	chr2	2.33E+08	2.33E+08	+	5248	1	INPP5D|SH	0.791	0.673
NR_03842	chr2	3558386	3561750	+	1326	1	RNASEH1-/	8.868	6.694
NM_0047:	chr11	1.03E+08	1.03E+08	-	1944	1	MMP20|A	0	0
NM_1307{	chr11	44764426	44932426	+	4223	1	TSPAN18|T	51.333	70.95
NM_0046:	chr8	38105493	38140080	+	2936	1	ASH2L|ASI	20.794	21.362
NR_10689	chr7	64679064	64679176	+	112	1	MIR6839|I	0	0
NM_0246:	chr5	50666938	50846522	+	7149	1	PARP8|AR	1.594	1.314
NM_0013(	chr15	65053337	65068112	-	2577	1	RASL12|RI	0.065	0.09
NR_04511	chr16	81665354	81667274	+	1920	1	LOC10012!	0.497	0.107
NM_0012!	chr4	1402932	1406442	-	1344	1	NKX1-1|HS	0	0.02
NR_12164	chr4	1.9E+08	1.9E+08	+	3362	1	DBET|DBE	0	0
NM_0156!	chrX	49113385	49123801	-	1733	1	GPKOW|G	21.278	19.38
NM_0044(	chr4	80266588	80291017	+	5384	1	FGF5|HBG	0.034	0.025
NR_03751	chr4	1.85E+08	1.85E+08	-	97	1	MIR3945|-	0	0
NM_0010:	chr1	67407810	67430440	-	6736	1	SERBP1|C(	13.064	12.158
NR_04678	chr1	1.12E+08	1.12E+08	-	374	1	KCND3-IT1	0	0
NM_0182:	chr2	2.33E+08	2.34E+08	-	5585	1	USP40|-|-	4.58	4.105
NM_0012!	chr5	2752131	2755397	+	699	1	C5orf38|C	28.683	23.063
NM_0332:	chr16	57512178	57536565	-	2418	1	CCDC102A	1.077	1.224
NR_04568	chr5	1.81E+08	1.81E+08	-	1664	1	HEIH|HCC/	1.201	1.572
NM_0057(	chrX	40580964	40606636	+	2025	1	ATP6AP2|/	41.896	50.344
NM_0336:	chr4	1.52E+08	1.53E+08	-	3895	1	FBXW7|A(	4.803	5.549
NM_0529/	chr18	58481250	58628957	-	7285	1	ALPK2|HA	0.582	0.135
NR_12640	chr1	1.01E+08	1.01E+08	+	469	1	LINC01307	0.165	0.058
NM_1471!	chr9	38571364	38620660	-	4017	1	ANKRD18/	0.135	0.055
NM_0010(	chrX	57119682	57121546	-	1200	1	SPIN2B|SP	3.374	3.013
NM_0328!	chr17	29614756	29621423	-	2385	1	CORO6|-|-	0.741	0.913

NM_1527	chr11	1.26E+08	1.26E+08	+	4206	1	STT3A|ITN	29.585	30.677
NM_0060	chr14	73711783	73714425	-	2640	1	PNMA1|N	97.653	97.441
NM_0030	chr17	12020818	12143831	+	3827	1	MAP2K4|J	13.867	14.148
NM_0022	chr1	58780791	58784113	-	3320	1	JUN|AP-1|	43.446	102.167
NM_1392	chrX	50190777	50200353	-	2838	1	AKAP4|AK	0.005	0
NR_11082	chr3	11751	24501	+	692	1	LINC0198	0	0
NM_1538	chr6	46997703	47042363	-	5451	1	ADGRF1|G	0.005	0
NR_00271	chrX	1.11E+08	1.11E+08	+	2709	1	TDGF1P3|	0	0.005
NM_1784	chr11	26667019	26722027	-	6236	1	SLC5A12|	0.008	0.004
NR_12540	chr3	1.87E+08	1.87E+08	-	1828	1	LINC0204	0.056	0.045
NM_0011	chr19	33796846	33815761	+	3924	1	KCTD15|-|	15.318	19.806
NR_10804	chr10	1.18E+08	1.18E+08	+	577	1	LINC0086	0	0
NM_0065	chr11	1.23E+08	1.23E+08	-	2449	1	HSPA8|HE	832.306	740.897
NR_02988	chr5	1.8E+08	1.8E+08	-	94	1	MIR340|N	0	0
NR_03998	chr14	61570540	61654713	+	1365	1	FLJ22447|	0	1.716
NM_0012	chr11	1.18E+08	1.18E+08	+	6085	1	UBE4A|E4	10.686	11.631
NR_13276	chr10	1.13E+08	1.13E+08	+	221	1	SNORA87|	0	0
NM_0039	chr15	74608372	74630201	+	1831	1	CLK3|PHC	34.16	41.607
NM_0320	chr22	50245184	50251405	-	2684	1	HDAC10|H	5.736	5.537
NM_0013	chrX	1.54E+08	1.54E+08	+	2270	1	RPL10|AU	117.274	95.945
NM_0160	chr5	1.73E+08	1.73E+08	+	716	1	RPL26L1|F	17.089	16.107
NM_1525	chr8	41645178	41647359	-	1679	1	NKX6-3|N	0.046	0.024
NR_13445	chr8	13629690	13632576	+	946	1	LOC10272	0	0
NR_11068	chr19	27793467	27806780	+	1780	1	LOC10192	1.775	1.685
NM_0323	chr2	1.22E+08	1.22E+08	-	1729	1	NIFK|MKI6	10	12.659
NR_04010	chr3	40390951	40453308	-	2035	1	ENTPD3-A	0.526	0.397
NM_0013	chr4	1.23E+08	1.23E+08	+	2237	1	SPATA5|A	1.21	0.686
NR_03386	chr19	15759334	15779988	-	2460	1	CYP4F24P	0.372	0.379
NM_0012	chr8	1.44E+08	1.44E+08	-	667	1	SLC39A4|	0.116	0.205
NM_0057	chr16	82148162	82170224	-	1094	1	MPHOSPH	17.148	15.976
NM_0011	chr11	66975283	66977008	+	1181	1	C11orf86|	0.033	0.012
NR_11072	chr19	43996896	44002870	-	2213	1	LOC10192	3.07	3.311
NR_12656	chr1	1.54E+08	1.54E+08	+	657	2	LOC34305	0	0
NR_03744	chr1	16548914	16548987	+	72.5	4	MIR3675|	0	0
NM_0010	chr1	2.37E+08	2.38E+08	+	16258	1	RYR2|ARV	0.227	0.195
NR_00308	chr22_KI2	280113	296074	-	1920	2	GSTTP2|-|	0	0
NR_12576	chr1	84597964	84621031	-	557	1	LINC01461	0.231	0.049
NM_0010	chr1	2.48E+08	2.48E+08	+	973	3	OR2T2|OR	0	0
NM_0011	chr6	31005952	31035402	+	5997.5	8	MUC22|PE	0	0
NM_1992	chr14	77474395	77498867	-	2981	1	ISM2|TAIL	0.281	0.083
NM_0012	chr16	50025206	50037088	+	2110	1	CNEP1R1|	4.601	6.258
NR_00350	chr20	58232777	58309439	-	2381	1	PPP4R1L|	1.695	1.559
NM_0124	chr20	4721854	4728462	+	4034	1	PRND|DOI	0.249	0.401
NM_0011	chr10	87859161	87863437	-	4274	1	KLLN|CWS	0.32	0.41
NM_0321	chr12	49587505	49605650	-	2946	1	FAM186B|	0.153	0.116
NM_0012	chr7	66682091	66811461	+	4514	1	RABGEF1|	7.638	7.999
NM_0012	chr18	79400274	79468002	+	2581	1	NFATC1|N	4.081	1.55
NR_04975	chr11	319673	320914	-	661	1	IFITM3|1-	28.264	26.876
NM_0011	chrX	2828822	2882820	+	2937	1	GYG2|GN-	10.681	11.137
NM_0010	chrX	1.04E+08	1.04E+08	-	1664	1	GLRA4|-|-	0.256	0.313

NR_03984	chr11	72783530	72783592	+	62	1	MIR4692|-	0	0
NM_1532(	chr11	66291902	66296664	+	2526	1	TMEM151	11.607	11.805
NM_0010{	chr19	17601328	17688199	-	9795	1	UNC13A|N	21.01	27.574
NM_1446:	chr1	2.42E+08	2.42E+08	+	4341	1	WDR64|-|	0.086	0.013
NR_12167	chr8	1.28E+08	1.29E+08	-	3022	1	LINC00824	0	0
NR_04002	chr17	73786822	73800795	+	2467	1	LINC02092	0.052	0.039
NM_0013:	chr8	1.44E+08	1.45E+08	+	2850	1	PPP1R16A	22.159	15.216
NR_00295	chr11	93733466	93733597	-	131	1	SNORA18|	1.378	2.091
NR_03891	chr5	1.77E+08	1.77E+08	-	1596	1	PRR7-AS1|	0.485	0.455
NR_10673	chrX	2609191	2609254	+	63	2	MIR6089|	0	0
NR_04979	chr2	75090812	75090914	+	102	1	MIR5000|-	0	0
NR_13742	chr12	8950044	9010744	+	1610	1	KLRG1|2F:	1.514	1.234
NM_0061(	chr12	1.04E+08	1.04E+08	-	3472	1	NFYB|CBF-	9.146	11.152
NM_0004;	chrX	30304422	30309378	-	1581	1	NR0B1|AH	0.188	0.147
NR_13552	chr2	2.17E+08	2.17E+08	-	424	1	LOC10537.	0	0
NM_0013:	chr2	72942036	73059193	-	3874	1	SFXN5|BB(	3.964	9.635
NM_0059:	chr7	15611212	15686683	-	2366	1	MEOX2|G/	0	0
NM_0003:	chr11	1.12E+08	1.12E+08	+	926	1	PTS|PTPS|	21.944	22.513
NM_0001:	chrX	1.54E+08	1.54E+08	+	1331	1	EMD|EDM	61.397	55.303
NM_0324!	chr2	1.12E+08	1.12E+08	-	1592	1	ZC3H8|Fliz	3.774	4.199
NM_0012!	chr10	1.27E+08	1.27E+08	+	6806	1	DOCK1|DC	3.281	2.872
NM_0013(	chr19	36141420	36150353	+	1245	1	CAPNS1|C	212.23	216.93
NM_0180!	chr16	24919384	25015378	-	3254	1	ARHGAP17	9.252	8.953
NR_13563	chr17	73513683	73518771	+	2277	1	LOC10192:	0.011	0.012
NM_0010:	chr12	49791146	49828425	-	4885	1	NCKAP5L|	28.524	30.815
NR_11002	chr7	1.27E+08	1.27E+08	+	953	1	LOC10050(	0	0
NM_0149;	chr19	12838445	12874952	+	4895	1	MAST1|SA	54.074	51.22
NM_0165<	chr19	51142301	51153526	+	1467	1	SIGLEC7|A	0	0
NM_0245:	chr16	29816207	29822495	+	3820	1	PAGR1|C1	21.396	18.731
NM_0000{	chrX	50067560	50099235	+	9491	1	CLCN5|CL(	5.987	3.425
NM_0045<	chr16	1959516	1961975	+	691	1	NDUFB10|	166.869	134.532
NM_0011<	chr6	5085486	5087221	+	1733	1	PPP1R3G|	0.208	0.063
NM_0016<	chr3	49674002	49683501	+	2734	1	APEH|AAR	47.103	48.02
NR_03019	chr19	53708735	53708835	+	100	1	MIR518C|	0	0
NM_0012(	chr17	7572706	7579006	+	1802	1	EIF4A1|DD	287.735	280.416
NM_0042:	chr8	8784489	8893621	-	5250	1	MFHAS1|L	7.8	9.322
NM_0010:	chr6	1.1E+08	1.1E+08	-	2966	1	WASF1|SC	30.738	35.517
NM_0066!	chr11	10557866	10568818	-	2492	1	LYVE1|CRS	0.005	0.033
NM_0247(	chr14	91580774	91732086	-	3595	2	CATSPERB	0	0
NM_0012!	chr11	75399491	75422301	+	1358	1	RPS3|S3|-	405.698	402.952
NM_0010!	chrX	49541733	49549097	+	557	6	GAGE12C|	0	0
NM_0001:	chr8	1.18E+08	1.18E+08	-	3348	1	EXT1|EXT|	12.574	15.816
NM_0010:	chr1	1.6E+08	1.6E+08	-	1809	1	VSIG8|-|-|	0	0.015
NM_0013(	chr4	1.29E+08	1.29E+08	-	1750	1	SCLT1|CAF	1.901	1.526
NR_10996	chr3	1.77E+08	1.77E+08	-	819	1	LINC01208	0.142	0.301
NM_0005!	chr5	1.36E+08	1.36E+08	-	584	1	IL9|HP40|	0	0
NM_0012(	chr2	1.74E+08	1.74E+08	-	7303	1	GPR155|D	1.28	2.095
NM_1818;	chr4	6320578	6381870	-	4081	1	PPP2R2C|	1.791	10.505
NR_10683	chr17	17813480	17813545	-	65	1	MIR6777|	1.389	0.632
NM_0070!	chr20	42072752	43189917	-	12604	1	PTPRT|RP	0.174	0.033

NR_13364	chr20	29548719	29560475	+	715	1	LINC0159٤	0	0
NR_03975	chr18	55479221	55479298	+	77	1	MIR4529|-	0	0
NM_1768	chr11	7020469	7071526	+	3809	1	NLRP14|CI	0.027	0.111
NR_13836	chr15	64739891	64761217	-	1739	1	RBPMS2|-	2.884	1.89
NM_0011	chr4	73481745	73504001	+	1980	1	AFM|ALB2	0	0
NM_0011	chr1	1.16E+08	1.16E+08	+	8625	1	VANGL1|K	0.628	0.551
NR_11083	chr17	15806242	15817742	-	947	1	LINC02087	0	0
NM_0049	chr9	33441154	33447633	-	1863	1	AQP3|AQI	0.491	0.772
NM_0012	chr2	1.3E+08	1.31E+08	-	3212	1	POTEI|PO	0	0
NR_02687	chr17	81650459	81656577	-	815	1	PDE6G|PD	0.854	0.218
NM_0011	chr1	1.51E+08	1.51E+08	-	1835	1	LYSMD1|S	19.962	25.401
NM_1785	chr17	54968765	55164088	+	6026	1	STXBP4|Sy	0.676	1.362
NM_0029	chr4	73836556	73838760	+	1653	1	CXCL6|CK	0.218	0.058
NM_0321	chr18	12991362	13125052	+	7929	1	CEP192|PI	2.327	2.683
NM_0243	chr19	29665420	29675476	+	1735	1	PLEKHF1|	0.907	0.679
NM_0225	chr17	63880212	63881942	-	852	1	GH2|GH-V	0	0
NM_1988	chr17	41866926	41919019	-	4293	1	ACLY|ACL|	149.37	160.966
NM_0146	chr19	5586999	5622927	-	3295	1	SAFB2|-|-	16.509	15.871
NM_0185	chr10	13161554	13211104	+	4525	1	MCM10|C	1.63	1.262
NR_03883	chr7	27147163	27155928	+	3989	1	HOXA-AS3	0.003	0.481
NM_0001	chr2	1.27E+08	1.27E+08	-	2733	1	ERCC3|BTI	30.486	33.674
NM_0003	chr5	1.36E+08	1.36E+08	+	2786	1	TGFBI|BIG	1.319	0.526
NR_12596	chr1	1.81E+08	1.81E+08	+	537	1	LINC01732	0.12	0
NM_0215	chr5	1.79E+08	1.79E+08	-	2239	1	ADAMTS2	2.148	0.795
NM_0010	chr1	20290919	20354894	+	4451	1	VWA5B1|-	0.067	0.203
NM_0012	chr3	1.22E+08	1.22E+08	+	2667	1	CD86|B7-2	0.015	0.021
NM_0012	chrX	1.47E+08	1.47E+08	+	434	2	CXorf51B|	0	0
NM_0012	chr15	90998416	91022621	-	2680	1	VPS33B|-|	15.929	18.798
NR_03039	chr15	80841978	80842073	-	95	1	MIR549A|	0	0
NM_0012	chr18	66501187	66604138	-	5859	1	CDH19|CD	0	0
NR_04621	chr14	1.06E+08	1.06E+08	-	789	2	ELK2AP|EL	0	0
NR_02948	chr21	16539828	16539911	+	83	1	MIRLET7C	0	0
NR_11000	chr20	25844667	25854021	-	6279	1	LOC10192	0.064	0.046
NR_13072	chr11_KI2	72299	93727	-	1082	2	KCNQ1-AS	0	0
NM_0013	chr11	27365961	27472775	-	5144	1	LGR4|BNM	5.214	3.195
NM_0010	chr3	1.85E+08	1.85E+08	+	4998	1	VPS8|KIAA	11.175	15.613
NM_0013	chr12	81078018	81256758	+	3979	1	ACSS3|-|-	0.47	0.317
NR_03023	chrX	1.47E+08	1.47E+08	-	97	1	MIR514A1	0	0
NR_04680	chr20	8248704	8249741	+	370	1	PLCB1-IT1	0	0
NR_04987	chr6	35664717	35664789	-	72	1	MIR5690|-	0	0
NM_0013	chr13	96985719	96994730	-	2474	1	OXGR1|GI	0	0.017
NM_0205	chr20	18142224	18188387	+	3702	1	KAT14|AT	14.412	18.918
NM_1980	chr4	87422597	87459454	+	2448	1	NUDT9|NU	8.122	8.605
NM_0207	chr17	75500260	75515583	-	4994	1	CASKIN2|	8.409	9.144
NM_0010	chr9	92845031	92878038	-	3884	1	ZNF484|B	2.788	2.807
NR_02336	chr1	2.29E+08	2.29E+08	-	120	10	RNA5S4|R	0	0
NM_0052	chr5	76818977	76835315	+	2910	1	F2RL1|GPI	0.337	0.565
NM_1782	chr1	62784106	62865271	+	2664	1	ATG4C|AP	1.404	1.584
NR_00156	chr2	47335315	47335514	+	199	1	BCYRN1|B	0	0.069
NM_0175	chr8	1.44E+08	1.44E+08	-	4002	1	OPLAH|5-(	0.103	0.14

NM_0072(	chr17	78112918	78132427	-	2787	1	TMC6|EV1	1.374	1.086
NR_13242	chr13	1E+08	1E+08	-	1006	1	LOC10537(	3.871	2.696
NM_0013(	chr16	71447600	71462252	-	2389	1	ZNF23|KO	7.113	9.644
NM_0010(	chr1	11066613	11099910	-	2815	1	EXOSC10|I	31.008	25.925
NM_0314{	chr19	48445773	48453907	+	2447	1	GRWD1|C	12.619	9.801
NM_0012{	chr20	23374519	23421519	-	3742	1	NAPB|SNA	38.856	38.284
NM_0039!	chr17	78356777	78360079	-	2733	1	SOCS3|AT(	3.199	3.834
NM_0146(	chr22	31754862	31907034	+	5273	1	DEPDC5|D	6.262	6.785
NM_0057.	chr19	57940833	57947709	-	2205	1	ZNF256|BI	3.391	5.82
NR_12159	chr9	4676601	4679480	-	241	1	CDC37L1-/	5.35	4.376
NM_0013:	chr5	1.4E+08	1.4E+08	+	2187	1	CXXC5|CF!	94.428	93.293
NM_0209(	chr9	1.3E+08	1.3E+08	+	6914	1	GPR107|G	26.79	27.214
NR_00331	chr15	25206262	25206343	+	81	1	SNORD11!	0.318	0
NM_1989.	chr1	46557407	46604294	-	2587	1	MKNK1|M	9.19	10.526
NM_1384:	chr3	32391671	32454844	+	1347	1	CMTM7|C	2.929	0.671
NM_2074:	chr1	9003300	9026345	-	1526	1	SLC2A7|GI	0	0
NR_02445	chr16	21864300	21866735	-	2433.3	3	LOC10019(	0.011	0.017
NM_0020(	chr19	3094410	3124002	+	4136	1	GNA11|FB	30.934	41.839
NR_02949	chr13	91351192	91351278	+	86	1	MIR19B1|	0	0
NM_0157:	chr2	70830212	70835823	-	1864	1	CD207|CLI	0	0.037
NM_2068:	chr16	31090842	31094999	-	940	1	VKORC1|E	89.459	61.491
NM_1817:	chr16	29900826	29906056	+	1473	1	ASPHD1|-	17.314	17.091
NM_0055:	chr1	26472411	26476642	+	1961	1	HMGN2|H	13.492	13.592
NM_0010{	chr8	52622458	52714466	-	6632	1	RB1CC1|A	3.869	6.898
NR_00308	chr21	14273799	14301371	+	2818	1	ABCC13|A	0	0
NM_0020{	chr2	2.4E+08	2.4E+08	+	3675	1	GPC1|glyp	43.792	51.672
NM_0060·	chr16	22814539	22916338	+	1948	1	HS3ST2|3(	0.887	0.352
NR_02833	chr15	79922771	79924854	+	2083	1	ST20-AS1|	3.12	2.887
NR_00360	chr22	21008207	21014287	-	1957	1	TUBA3FP|·	5.943	3.583
NM_0012.	chr19	13023971	13098796	+	5533	1	NFIX|CTF|	0.634	38.326
NM_0013:	chr16	676075	678268	+	1551	1	RHBDL1|R	0.906	1.095
NR_02702	chr2	1.32E+08	1.32E+08	-	1420	1	ANKRD30E	0	0
NM_0000(	chr5	41142146	41213593	-	3702	1	C6|-|-|5p:	0.035	0
NM_0034!	chr16	3222325	3235186	-	3380	1	ZNF200|-|	6.664	6.512
NR_03963	chr2	87629760	87629833	+	73	2	MIR4435-:	0	0
NM_0164!	chr17	8288651	8290091	+	847	1	RANGRF|H	28.999	25.324
NM_0012.	chr18	36780025	36829216	-	1261	1	TPGS2|C1{	89.774	109.544
NR_04672	chr3	1.96E+08	1.96E+08	+	1845	1	TM4SF19-/	0.042	0.059
NM_0203.	chr15	33866227	34039102	-	1523	1	AVEN|PDC	5.799	4.497
NM_0245!	chr8	1.44E+08	1.44E+08	-	1770	1	FBXL6|FBL	10.723	8.953
NM_0804:	chr20	2840697	2866732	+	2302	1	VPS16|hVI	33.78	36.266
NM_0183(	chr15	56428731	56465137	-	2011	1	MNS1|SPA	0.244	0.381
NM_0013(	chr11	19054456	19060017	-	2340	1	MRGPRX2	0	0
NM_0221:	chr6	1.38E+08	1.38E+08	-	4297	1	PERP|KCP:	1.467	0.319
NR_02422	chr19	47950296	47950414	+	118	1	SNAR-C3|-	0	0
NM_0010:	chr17	6423737	6435199	-	2777	1	AIPL1|AIPI	0.051	0.015
NM_0307.	chr11	4680171	4697846	-	2781	1	OR51E2|O	0.533	0.01
NM_1523(	chr3	1.19E+08	1.19E+08	+	3519	1	POGLUT1|	3.199	3.593
NM_0010:	chrX	3006613	3033385	+	1679	1	ARSH|sulfa	0	0.163
NM_0012{	chr9	3270929	3489487	-	1510	1	RFX3|-|-|9	6.797	5.923

NM_0047:	chr20	58389119	58451100	+	7930	1	VAPB|ALS;	17.015	15.056
NM_0012{	chr1	47250139	47314147	-	4877	1	STIL|MCPI	1.208	1.452
NR_02460	chrX	99461602	99939843	-	2164	1	XRCC6P5|-	0.101	0.215
NM_0019{	chr12	56080025	56103507	+	5727	1	ERBB3|Erb	0.315	0.964
NM_0010{	chr19	56189689	56193052	-	1621	1	ZSCAN5B|:	0.095	0.034
NM_0010(	chr19	39480412	39493784	+	5455	1	TIMM50|T	15.456	14.119
NM_0529:	chr5	1.33E+08	1.33E+08	+	702	1	LEAP2|LEA	1.286	0.937
NR_03647	chr15	22479153	22482374	-	1761	1	HERC2P7|-	0.015	0.008
NR_04663	chr3	1.94E+08	1.94E+08	-	499	1	OPA1-AS1	1.369	2.168
NM_0013<	chrX	48597478	48605194	+	1784	1	WDR13|M	43.487	40.531
NM_0013(	chr20	62851494	62861763	-	2857	1	TCFL5|CH/	6.557	6.05
NM_0009!	chr3	51993628	51995942	-	689	1	RPL29|HIP	259.161	241.556
NM_1735:	chr7	74796144	74851576	-	3565	1	GTF2IRD2|	0.933	0.914
NR_11024	chr2	1.51E+08	1.51E+08	-	2522	1	LINC0192C	0	0
NM_1522{	chr1	1.71E+08	1.71E+08	+	2576	1	GORAB|G(	2.362	2.111
NM_0012:	chr11	18727928	18791842	-	2890	1	PTPN5|PTI	3.377	10.792
NM_0012:	chr3	8733800	8746765	+	1330	1	CAV3|LGN	0.078	0.031
NM_0011!	chr2	73892966	73919653	+	1192	1	ACTG2|AC	0.703	0.678
NR_04010	chr3	1.82E+08	1.82E+08	-	1802	1	LINC01994	0.014	0.015
NR_04984	chr11	79422169	79422226	-	57	1	MIR5579|-	0	0
NM_0120!	chr2	1.52E+08	1.52E+08	-	3161	1	ARL5A|AR	2.786	3.536
NM_0010<	chr17	39904595	39918650	-	1548	1	GSDMB|G.	3.656	5.026
NR_03034	chr12	80935736	80935833	-	97	1	MIR618|N	0	0
NR_03976	chrX	55451495	55451582	-	87	1	MIR4536-1	0	0
NM_0011(	chr19	41397789	41425005	+	1760	1	BCKDHA|E	32.893	25.55
NM_1523;	chr12	1.01E+08	1.01E+08	+	1124	1	SPIC|SPI-C	0	0
NM_0003!	chr16	71566851	71577095	-	2743	1	TAT|-|-|1(	0.019	0.025
NM_1536!	chr3	1.95E+08	1.95E+08	+	3142	1	FAM43A|-	1.485	2.302
NM_0013;	chr10	93591836	93601744	-	859	1	RBP4|MC(	2.627	2.567
NM_0013:	chr19	40230317	40265351	-	4942	1	AKT2|HIH(	17.952	20.723
NM_0010<	chrX	73003513	73005712	+	2197	1	PABPC1L2I	0.164	0.256
NM_1989:	chr20	62143720	62182510	+	4581	1	SS18L1|CF	20.254	21.271
NM_1750!	chr6	1.33E+08	1.33E+08	+	1047	1	TAAR9|TA	0	0
NR_12542	chr8	2696553	2728451	-	609	2	LOC10192:	0	0
NM_0202<	chr3	44761717	44853256	+	4800	1	KIF15|HKL	1.136	2.106
NM_0011:	chr15	41774434	41827855	+	7216	1	MAPKBP1|	10.508	12.849
NR_11013	chr10	35778302	35800920	+	3547	1	PCAT5|LIN	0.007	0.004
NM_0011:	chr11	64286110	64289500	+	1901	1	GPR137|C	30.352	27.394
NR_13392	chr2	68466239	68467317	-	340	1	FBXO48|-|	1.669	0.765
NM_0322!	chr11	1.03E+08	1.03E+08	-	12722	1	DCUN1D5	3.17	4.343
NM_0011:	chr13	43213133	43629477	-	3070	1	ENOX1|CN	7.576	6.799
NR_03029	chr4	24520192	24520290	-	98	1	MIR573|N	1.842	2.096
NR_12212	chr12	630891	645878	+	2333	1	LOC10004!	0	0
NR_11069	chr1	1.61E+08	1.61E+08	+	2130	1	LOC10192:	0	0.006
NM_0010:	chr4	87650302	87664360	+	2631	1	DMP1|ARI	0.015	0.01
NM_1749(	chr3	1.91E+08	1.91E+08	+	8408	1	CCDC50|C	11.113	10.245
NR_13122	chr13	89214848	89236890	-	772	1	LINC01047	0	0
NM_0010(	chr19	39196964	39201882	+	1965	1	NCCRP1|F	0.19	0.3
NM_0195!	chrX	1.35E+08	1.35E+08	-	2433	1	MOSPD1|I	5.548	6.294
NM_0223(	chr1	2.47E+08	2.47E+08	-	1776	1	TFB2M|Hk	3.681	4.165

NM_0010(	chr17	39688084	39728662	+	4840	1	ERBB2|CD	15.584	13.383
NR_02417	chr15	97742616	97874429	-	1343	1	LINC00923	0	0
NR_03986	chr16	12720321	12720371	+	50	1	MIR4718|-	0	0
NM_0178;	chr11	63552770	63563383	-	736	1	HRASLS2|I	0	0.13
NR_10982	chr12	53464468	53465057	+	589	1	PCBP2-OT:	109.474	103.716
NM_1447;	chr17	18315280	18328056	+	8306	1	SMCR8|-|-	5.647	6.499
NR_13528	chr17	76856166	76861836	+	458	1	LINC00868	0	0.03
NM_0011:	chr5	2746165	2751655	-	2296	1	IRX2|IRXA:	28.819	14.514
NM_0058;	chr22	30331988	30356947	-	5125	1	SF3A1|PRI	39.569	36.981
NM_0060!	chr4	42408375	42657105	-	8229	1	ATP8A1|A	6.474	6.665
NM_0061:	chr11	61680433	61747002	+	5736	1	DAGLA|C1	5.932	7.252
NM_0012	chr10	3137728	3172841	-	3159	1	PITRM1|M	24.738	27.091
NR_13237	chr13	75240588	75252012	+	438	1	LINC01078	0	0
NR_12045	chr12	8235415	8238957	+	804	1	LOC10192	0.754	2.47
NM_0012{	chr16	2223488	2235742	+	2106	1	E4F1|E4F|	15.097	18.178
NM_0012{	chr11	61816203	61867354	+	3003	1	FADS2|D6	257.174	224.425
NM_0011:	chr10	26216307	26301119	+	2401	1	GAD2|GAI	6.165	8.26
NM_1817{	chr8	94719875	94793848	+	6213	1	DPY19L4|-	0.917	1.373
NM_0149!	chr4	84669540	84966391	-	14247	1	WDFY3|AL	10.704	11.438
NM_0013(	chr19	19465340	19508932	+	5356	1	GATAD2A|	20.635	27.271
NM_0212	chr6	1.39E+08	1.39E+08	+	840	1	ABRACL|C	31.927	38.825
NM_0016	chr1	1.61E+08	1.61E+08	-	467	1	APOA2|Ap	0	0
NM_0323!	chr17	48721720	48722520	-	379	1	PRAC1|C1	0	0
NR_12163	chr1	2566535	2584534	+	694	2	LOC10099	0	0
NM_0013:	chr17	7626234	7627876	-	858	1	SAT2|SSAT	115.666	126.929
NM_0177;	chr6	10980760	11044391	-	4063	1	ELOVL2|SS	4.163	9.203
NR_03383	chr12	1.3E+08	1.3E+08	-	3674	1	FZD10-AS1	0.011	0.03
NR_03828	chr3	64004022	64012241	+	2546	1	PSMD6-AS	0.233	0.511
NR_03160	chr6	31956839	31956940	-	101	6	MIR1236|I	0	0
NM_1449(	chr9	14734666	14910995	-	10046	1	FREM1|BN	1.203	0.937
NM_0010:	chr6	1.12E+08	1.12E+08	+	1895	1	RFPL4B|RN	0.265	0.643
NM_0324:	chr5	1.4E+08	1.4E+08	+	905	1	CYSTM1|C	35.062	26.516
NM_0010:	chr6	35781054	35788064	+	472	1	CLPSL1|C6	0	0
NR_03384	chr7	50531759	50543463	+	1771	1	DDC-AS1|-	0.197	0.278
NR_02656	chr1	16691218	16720157	-	2004	1	ESPNP|dJ1	0	0
NM_1992!	chr17	61590433	61591202	-	767	1	NACA2|AN	59.172	58.288
NM_0162;	chr22	41433488	41447023	-	4561	1	TOB2|APR	17.634	15.265
NM_0011(	chrX	1.04E+08	1.04E+08	+	1877	1	H2BFM|-|	0.014	0.058
NR_02743	chr6	1.7E+08	1.7E+08	+	2836	1	LOC10013	0	0
NM_0013(	chr14	45135939	45167192	+	2111	1	FANCM|F/	0.202	0.454
NM_0010:	chr9	1.37E+08	1.37E+08	-	2210	1	ENTPD8|E	0.134	0.682
NR_11086	chr17	40340867	40343136	-	1787	1	RARA-AS1	7.54	7.289
NM_0010(	chr11	4488879	4489908	+	1027	1	OR52K1|O	0	0
NR_02844	chrX	1.19E+08	1.19E+08	-	3087	1	SLC25A5-A	3.183	3.199
NM_0020	chr6	89254466	89315299	-	4725	1	GABRR2|-	0.112	0.099
NR_12634	chr12	58591702	58812668	+	420	1	LOC10050	0.061	0.13
NM_0247	chr16	46580556	46621399	-	3217	1	SHCBP1|P.	0.77	0.996
NM_0071	chr6	27450742	27473118	-	3094	1	ZNF184|kr	3.105	6.047
NM_0032	chr6	1.69E+08	1.69E+08	-	5856	1	THBS2|TSI	5.493	2.171
NM_0022;	chr17	41363494	41369800	-	1598	2	KRT33B|H.	0	0

NM_0070:	chr17	37609741	37643464	-	6380	2	DDX52|HU	0.22	0.23
NM_0071(	chr17	68867287	68955392	-	5683	1	ABCA8|-|-	3.916	3.063
NM_0060(	chr14	24161213	24166566	+	1668	1	IRF9|IRF-9	5.906	5.88
NM_0211(	chr6_GL0C	2906069	2908543	-	2472.3	7	C6orf47|D	0	0
NR_11003	chr11	74266649	74311676	-	2093	1	P4HA3|-|-	0.172	0.105
NR_10695	chr12	1.13E+08	1.13E+08	-	64	1	MIR7106|	1.813	0
NR_14455	chr17	20436824	20447244	+	2831	1	NOS2P3|-	0.009	0.005
NM_0055(	chr13	35473789	35476695	-	2904	1	MAB21L1|	93.908	9.985
NM_0162(	chr19	48813017	48836677	-	1226	1	HSD17B14	7.551	6.536
NM_0180:	chr2	45388680	45611294	-	3672	1	SRBD1|-|-	4.554	4.099
NM_1481:	chr9	34610485	34612113	-	894	1	RPP25L|C9	15.965	11.123
NM_0011(	chr17	75646566	75647977	+	824	1	SMIM6|C1	0	0
NR_00303	chr1	28580920	28581053	-	133	1	SNORA16A	0.969	0.927
NM_1524(	chr5	1.49E+08	1.49E+08	+	6022	1	AFAP1L1|-	0.079	0.139
NM_0011(	chr10	94089026	94328391	+	6468	1	PLCE1|NPI	0.889	1.887
NM_0208:	chr3	1.14E+08	1.14E+08	-	3957	1	CCDC191|	2.089	1.222
NR_11005	chr20	53552770	53575308	+	1941	1	LOC10192	0.146	0.155
NM_0012(	chr11	12138390	12263790	+	3684	1	MICAL2|M	0.399	0.714
NR_03373	chr15	84191848	84209273	+	7742	1	LOC44030	0	0.011
NM_0022(	chr1	1.6E+08	1.6E+08	-	5303	1	KCNJ10|BI	0.229	3.296
NM_0178:	chr1	1311585	1324687	-	2137	1	INTS11|CP	41.432	40.681
NM_0010(	chr11	1.12E+08	1.12E+08	+	3175	1	C11orf57|	21.596	20.945
NR_12032	chr15	33303657	33310659	-	461	1	LOC10192	0.028	0
NR_00438	chr7	1.3E+08	1.3E+08	-	3240	1	MESTIT1|I	0.899	0.85
NM_0012(	chr3	1.96E+08	1.96E+08	-	975	1	TM4SF19|	0.106	0.098
NR_04558	chr4	1.59E+08	1.59E+08	+	3774	1	RXFP1|LGI	0	0
NM_0058(	chr13	80335977	80341115	-	2270	1	SPRY2|IGA	20.175	23.315
NM_0123:	chr17	55392613	55421980	-	2714	1	MMD|MM	28.2	33.339
NR_03843	chr2	12966782	13007013	-	1938	1	LOC10050	0.166	0.53
NR_03996	chr5	72169467	72169540	+	73	1	MIR4803|-	0	0
NM_0069:	chr21	44805617	44818129	-	1801	1	SUMO3|SI	77.11	81.338
NM_0161:	chr3	1.29E+08	1.29E+08	+	3048	1	COPG1|CC	71.777	72.52
NR_10979	chr9	90300896	90433489	-	1016	1	LINC01508	1.218	1.321
NM_1524!	chr15	43358172	43370060	-	5600	1	ZSCAN29|	9.088	7.964
NM_1735:	chr2	2.03E+08	2.03E+08	+	5763	1	FAM117B|	7.135	8.017
NM_0013:	chr5	72179628	72209570	+	11724	1	MAP1B|FU	150.604	170.167
NM_0011(	chr11	70398506	70436584	+	2266	1	CTTN|EMS	49.281	42.947
NR_14455	chr16	49517646	49518585	-	939	1	ADAM3B|	0	0.117
NR_03058	chrX	1.35E+08	1.35E+08	-	77	1	MIR450B|	0	0
NR_13394	chr4	58987188	58988154	-	619	1	LOC10537	0.062	0
NM_1489:	chr7	73736095	73738867	-	1453	1	ABHD11|P	9.832	6.071
NM_0013:	chr6	70413404	70561174	+	5034	1	FAM135A|	1.173	1.981
NM_0011(	chr1	2.11E+08	2.11E+08	-	4258	1	RD3|C1orf	0.821	0.212
NM_0020(	chr7	80134824	80219409	+	3308	1	GNAI1|Gi|	15.189	10.269
NM_0247(	chr15	1.01E+08	1.01E+08	+	1746	1	ASB7|-|-|	5.11	5.491
NR_00283	chr16	35169431	35170391	-	960	1	UBE2MP1	13.283	12.655
NM_0154(	chr17_GLC	594073	758707	-	5035.7	3	KANSL1|CI	0.159	0.15
NM_0012(	chrX	1.15E+08	1.15E+08	-	4852	1	LRCH2|dA	4.055	4.181
NM_0206:	chr21	41739369	41767089	-	3862	1	RIPK4|ANI	0.501	0.649
NM_0010(	chr1	2.49E+08	2.49E+08	-	970	1	OR2T35|-|	0	0

NM_0013(	chr9	1.23E+08	1.23E+08	-	1985	1	ZBTB26|ZN	4.306	5.065
NM_0012	chr9	1.13E+08	1.13E+08	-	2227	1	POLE3|CH	38.31	37.567
NM_0201	chr16	80975802	81006897	-	724	1	CMC2|231	40.105	42.51
NM_0003(	chr3	87259633	87276587	-	1254	1	POU1F1|C	0.021	0.066
NM_0013	chr9	35489833	35561898	+	3380	1	RUSC2|Ipc	45.959	52.028
NR_04683	chr6	53998890	54007152	+	639	1	MLIP-IT1|-	0	0
NM_0198	chr14	21498131	21511375	-	2009	1	METTL3|IN	31.948	31.014
NM_0011	chr1	2.12E+08	2.12E+08	+	2645	1	PPP2R5A|	1.219	1.564
NR_00301	chr17	77089307	77089493	+	186	1	SCARNA16	5.13	1.988
NM_0013	chr2	1.71E+08	1.71E+08	-	9877	1	METTL8|T	0.995	1.078
NR_10705	chrX	52336557	52336642	-	85	1	MIR8088|	0	0
NM_0012(	chr19	36916329	36997932	+	2747	1	ZNF568|-|	2.14	3.052
NM_0011	chr3	51983284	51989202	+	1656	1	ACY1|ACY	5.668	5.26
NM_0013	chr13	1.13E+08	1.13E+08	+	3423	1	MCF2L|AR	24.593	24.477
NM_0012	chr4	65330559	65670495	-	5159	1	EPHA5|CE	12.074	14.363
NR_14443	chr8	1.4E+08	1.4E+08	-	5641	1	PEG13|-|-	4.571	4.762
NM_0011	chr19	35775515	35788822	+	4196	1	ARHGAP3	42.555	43.516
NM_1982(	chr12	1.21E+08	1.21E+08	-	645	1	POP5|HSP	31.664	23.423
NR_04671	chr13	77075514	77087776	+	513	1	MYCBP2-A	5.881	9.452
NR_03657	chr7	1E+08	1E+08	+	2990	1	STAG3L5P	10.418	10.348
NR_02729	chrX	73126037	73128080	-	2043	1	NAP1L6|-|	0.013	0.013
NR_03037	chr22	17980868	17980961	-	93	1	MIR648|M	0	0
NM_0012	chr1	1.62E+08	1.62E+08	-	2380	1	FCGR3B|C	0	0
NM_0013	chr7	5970926	6009106	-	4977	1	PMS2|HNI	3.671	3.495
NR_12585	chr6	1.35E+08	1.35E+08	-	557	1	LOC10192	0.023	0.049
NR_11081	chr17	58519837	58556925	+	648	1	SEPT4-AS1	3.402	3.762
NR_11054	chr7	1.44E+08	1.44E+08	-	2263	2	TCAF2P1|	0.006	0.061
NR_12637	chr13	38532000	38686675	-	2356	1	LINC00437	0	0.006
NM_0011	chr10	87020289	87024730	+	357	1	FAM25A|b	0	0.038
NM_0011(	chrY	9337510	9340278	+	1125	2	TSPY4|TSP	0	0
NM_0040	chr10	1.18E+08	1.18E+08	+	2891	1	EMX2|-|-|	2.333	18.193
NM_0066(	chr14	99684418	99727301	+	2060	1	CYP46A1|	2.798	6.396
NR_13117	chr14	50007313	50105043	-	968	1	LINC0159	0.147	0.141
NM_0306(	chr17	17681473	17811451	+	7652	1	RAI1|SMC	26.675	21.709
NR_03972	chr13	87618665	87618740	-	75	1	MIR4500|	0	0
NM_0013	chr5	1.77E+08	1.77E+08	-	2186	1	UIMC1|RA	17.594	19.931
NM_1526	chrX	1.35E+08	1.35E+08	+	4052	1	ZNF449|Z	1.419	1.768
NR_10673	chrX	13590292	13590346	+	54	1	MIR6086|	0	0
NM_0121	chr11	34621041	34663287	+	5439	1	EHF|ESE3|	0.028	0.02
NM_0329(	chr17	35997582	36002038	-	1070.5	2	CCL15|HC	0	0
NR_03831	chr17	7015818	7019654	-	791	1	MIR497HG	49.862	49.836
NM_0060	chr10	12195962	12250590	+	1533	1	CDC123|C	46.99	49.204
NM_1735	chr19	21724041	21767628	-	4463	2	ZNF100|-|	0.023	0.003
NR_00243	chr20	49278945	49279023	+	78	1	SNORD12C	0	0.527
NM_0008	chr4	1.56E+08	1.56E+08	+	3245	1	GUCY1B3|	7.231	7.729
NM_0175	chr19	1481428	1490450	-	2686	1	PCSK4|PC	5.487	2.743
NM_1527	chr18	69401048	69849086	+	8886	1	DOK6|DOI	4.228	4.097
NM_0011	chr8	91954967	92099688	-	7270	1	RUNX1T1|	9.687	7.376
NM_0001	chr9	1.3E+08	1.3E+08	-	2099	1	TOR1A|DC	29.178	33.587
NR_10271	chr4	97366926	97490164	+	531	1	STPG2-AS1	0.121	0.026

NM_0122!	chr4	75642769	75673159	-	4261	1	G3BP2|-|-	61.224	58.822
NM_0150	chr16	23463542	23510494	-	5954	1	GGA2|VEA	10.007	10.361
NR_03621	chr1	1.51E+08	1.51E+08	+	85	1	MIR4257|	0.303	0.645
NM_0010	chrX	15384801	15493588	-	1515	1	PIR|-|-|Xp	5.779	3.833
NM_1750	chr18	63637259	63644319	-	1697	1	SERPINB4|	0	0.016
NM_0011	chr3	1.73E+08	1.73E+08	-	4274	1	NCEH1|AA	0.537	0.76
NR_02459	chr22	24645166	24659147	-	9541	1	POM121L1	0.015	0.007
NR_00295	chr7	44985378	44985510	-	132	1	SNORA9|A	1.074	1.038
NR_13548	chrUn_KI2	44381	130836	+	419	1	LOC10050	0	0
NM_0243	chr16	74712958	74774831	-	2418	1	FA2H|FAA	0.053	0.017
NR_02987	chr5	1.5E+08	1.5E+08	+	65	1	MIR378A|	0	0
NM_0012	chr1	36339619	36385927	-	3644	1	STK40|SHI	31.919	37.723
NM_0018	chrX	77899464	77905384	+	451	1	COX7B|AP	250.637	232.728
NR_03170	chr1	1.68E+08	1.68E+08	+	66	1	MIR1255B	0	0
NM_0013	chr13	24309578	24322531	+	1903	1	C1QTNF9|	0.054	0.014
NM_0032	chr13	45333471	45341284	-	4641	1	TPT1|HRF	279.439	237.031
NR_13451	chr20	31870702	31944959	+	2751	1	TTLL9|C20	0.323	0.388
NM_0013	chr15	58138196	58185911	+	2809	1	AQP9|AQ	0.023	0.01
NM_0308	chr6	29355230	29356277	-	1045	5	OR5V1|6N	0	0
NR_01539	chr7	1.42E+08	1.42E+08	-	2255	2	WEE2-AS1	0	0
NR_03981	chr1	2.28E+08	2.28E+08	+	78	1	MIR4666A	0	0.176
NR_02962	chr1	1.72E+08	1.72E+08	-	109	1	MIR214|M	0	0
NR_11193	chr1	1.48E+08	1.48E+08	+	1476	1	LOC10013	1.092	1.058
NM_0011	chr12	1.1E+08	1.1E+08	-	5520	1	GIT2|CAT-	8.196	8.754
NM_0012	chr1	1.1E+08	1.1E+08	+	3372	1	AMPD2|P	24.804	24.977
NR_03385	chr12	53043189	53054438	-	2859	1	LOC28333	1.073	0.848
NM_0011	chr15	79282804	79298239	+	5420	1	ANKRD34C	0.026	0.003
NM_0247	chr16	19456450	19499112	+	3666	1	TMC5|-|-|	0.053	0.082
NR_04664	chrX	18688644	18692687	-	488	1	PPEF1-AS1	0	0
NM_0012	chr7	44044640	44061716	+	1993	1	DBNL|ABP	55.139	46.727
NR_13460	chr6	1.2E+08	1.2E+08	+	808	1	LOC10537	0	0.017
NM_0010	chr9	1.13E+08	1.13E+08	+	7595	1	SNX30|AT	13.152	8.828
NM_0202	chr11	75260122	75351831	-	7481	1	ARRB1|AR	9.072	21.196
NM_0151	chr2	28894643	28948220	+	3511	1	WDR43|N	5.589	5.579
NM_0011	chr7	29920103	29989809	-	5099	1	SCRN1|SE	147.591	145.845
NR_14475	chr2	2.13E+08	2.13E+08	-	629	1	LOC10013	1.25	0.958
NM_0010	chr12	48525632	48528103	+	1077	1	OR8S1|-|-	0	0
NR_02434	chr2	77954907	77955026	-	119	1	SNAR-H|sr	0	0
NR_11015	chr14	85934678	86129777	+	734	1	LOC10192	0.035	0
NR_00442	chr3	4749194	4751590	-	1460	1	EGOT|EGC	0	0
NM_0010	chr20	56533246	56536518	+	688	1	FAM209B|	0.094	0.299
NM_0177	chr18	74630963	75065672	+	7998	1	ZNF407|-|	1.507	1.584
NM_0010	chr1	2.48E+08	2.48E+08	+	925	3	OR2T6|OR	0	0
NM_0013	chr6	1.52E+08	1.52E+08	+	5411	1	ESR1|ER|E	0.057	0.076
NM_2073	chr6	1.49E+08	1.49E+08	-	4899	1	ZC3H12D|	0.068	0.081
NM_0582	chr10	1.12E+08	1.12E+08	+	2779	1	TECTB|-|-	0	0.015
NM_0120	chr19	17511623	17521288	+	1027	1	PGLS|6PG	85.76	72.366
NR_03408	chr17	5192084	5235636	+	1720	1	LOC10013	2.136	2.166
NM_0066	chr3	45886504	45903175	+	2514	1	CCR9|CC-C	0.031	0.033
NR_11938	chr1	64979577	65002476	-	1072	1	LINC0135	0.036	0.115

NM_0133!	chr1	17205126	17246006	+	3829	1	PADI1|HP/	0.01	0.064
NM_0010:	chr9	67859349	67902847	+	3913	3	ANKRD20/	0	0
NM_0012!	chr2	61868127	61888671	-	2244	1	CCT4|CCT-	120.655	141.554
NR_13193	chr7	22725395	22727620	-	1362	1	LOC54147.	0	0
NR_02704	chr19	12092263	12114678	+	3765	1	ZNF788|-|	0.825	0.986
NR_07408	chr16	29806537	29811183	+	1696	1	MAZ|PUR:	172.937	155.989
NM_0012:	chr19	13144058	13150374	-	1289	1	STX10|SYN	18.615	20.413
NM_0309:	chr10	80454282	80522635	+	5568	1	TSPAN14|	35.346	38.257
NM_0245:	chr4	2231836	2242164	-	2157	1	HAUS3|C4	1.53	3.359
NM_0043:	chr10	86756639	86925188	+	3601	1	BMPR1A|:	2.116	3.507
NM_0062:	chr3	1.39E+08	1.39E+08	-	5896	1	PIK3CB|P1	2.346	3.259
NM_0011(	chr8	1.4E+08	1.4E+08	-	6891	1	TRAPPC9|	16.252	21.445
NR_10675	chr16	81611348	81611408	+	60	1	MIR6504|	0	0
NM_0326‹	chr7	1.29E+08	1.29E+08	+	2750	1	IRF5|SLEB:	0.436	0.732
NM_0011!	chr4	1.4E+08	1.4E+08	+	1856	1	SCOC|HRII	23.126	17.896
NR_03025	chrX	74287104	74287209	-	105	1	MIR545|N	0	0
NM_0211!	chr12	55019945	55030017	+	3952	1	NEUROD4	4.072	2.007
NR_02415	chr22	19046162	19048375	-	2213	1	DGCR11|D	0.6	0.718
NM_0011(	chr2	26173091	26189663	+	4153	1	GAREM2|I	17.131	18.209
NM_0011!	chr20	46557823	46684485	-	3964	1	SLC13A3|I	0.953	1.693
NM_1392‹	chr14	24318349	24335071	-	3387	1	ADCY4|AC	0.019	0.012
NM_0013(	chr19	50025893	50048774	+	4546	1	ZNF473|ZI	9.578	8.819
NR_04009	chr12	93003758	93377736	-	573	1	LOC64333!	0.09	0.502
NM_0011‹	chr6	43477523	43506556	+	2561	1	TJAP1|PIL	10.276	9.493
NR_13367	chr21	6532207	6532776	-	420	2	LOC10678(	0	0
NR_03619	chr14	1.03E+08	1.03E+08	+	82	1	MIR4309|-	0	0
NM_0150(	chr12	27244145	27325957	+	5080	1	STK38L|NI	2.678	4.395
NM_0011!	chr20	36573473	36593952	+	3316	1	TGIF2|-|-|	9.242	10.492
NM_0008‹	chr6	34018643	34146137	-	7405	1	GRM4|GPI	0.524	0.64
NM_0010(	chr5	1.77E+08	1.77E+08	+	1928	1	GRK6|GPR	18.531	19.025
NM_0156(	chr12	53307456	53321628	-	1819	1	AAAS|AAA	42.664	42.228
NM_0048!	chr17	4959226	4967837	-	1016	1	SPAG7|AC	82.094	70.656
NM_1750(	chr6	1.33E+08	1.33E+08	+	1036	1	TAAR6|TA	0	0
NM_0204!	chr13	49912702	49936501	-	3211	1	SPRYD7|C:	4.481	3.959
NM_0012‹	chr18	36129874	36177922	+	5591	1	ELP2|SHIN	12.003	15.846
NR_02949	chr13	91351065	91351135	+	70	1	MIR20A|N	0	0
NM_0014!	chr9	33110641	33167358	-	4191	1	B4GALT1|	1.547	1.595
NM_0012(	chr4	53285	88211	+	2684	1	ZNF595|-|	1.287	3.113
NR_02684	chr18	5236724	5238029	-	1305	1	LINC0052€	7.835	8.764
NR_03893	chr12	2836816	2859795	+	873	1	LOC10050	5.361	3.562
NM_1829:	chr7	1434359	1459473	-	3080	1	MICALL2|J	5.865	4.029
NM_0072(	chr17	19904452	19977856	-	3924	1	AKAP10|A	4.216	5.686
NM_0145(	chr21	31873315	32004064	+	7372	1	HUNK|-|-|	15.129	20.373
NM_0010(	chr8	1.24E+08	1.24E+08	-	1829	1	KLHL38|C(	0.014	0
NM_1824!	chr7	1152907	1160219	-	894	1	ZFAND2A|	27.359	24.131
NM_0011(	chr7	1.42E+08	1.42E+08	+	3047	2	WEE2|WE	0	0
NM_0012(	chr22	41621342	41664048	+	2132	1	XRCC6|CT(	144.604	148.798
NM_0013(	chr3	1.21E+08	1.21E+08	+	9264	1	STXBP5L|L	0.853	1.371
NM_0010(	chr19	11346505	11354944	+	1050	1	CCDC159|	3.942	3.457
NR_00307	chr2	28913662	28913750	+	88	1	SNORD92|	0	0

NM_0021:	chr6	32578770	32589836	-	1188	3	HLA-DRB1	0	0
NM_1785(	chr11	1.2E+08	1.2E+08	+	1856	1	OAF|NS5A	1.292	1.373
NR_03890	chr6	1.26E+08	1.26E+08	+	2959	1	LOC64362:	0	0
NM_0011(	chr2	2.4E+08	2.4E+08	-	441	1	COPS9|CSI	98.996	85.04
NR_04639	chr12	1.28E+08	1.28E+08	+	2124	1	LINC00507	0	0
NM_0328{	chr9	1.14E+08	1.14E+08	+	7747	1	COL27A1|:	0.558	1.33
NR_13565	chr17	5111935	5114382	+	473	1	LOC10192:	11.558	12.307
NM_0013<	chr7	1E+08	1E+08	-	802	1	PPP1R35|(	20.401	15.815
NM_0072:	chr17	64477785	64497066	-	1584	1	POLG2|HP	4.746	5.353
NR_03896	chrX	39304956	39327362	-	1769	1	LINC01281	0	0
NM_0324(	chr5	1.41E+08	1.42E+08	+	2318	1	PCDHGC3|	39.832	160.311
NM_0012{	chr9	89318473	89359663	+	3368	1	SECISBP2|	18.452	17.8
NR_00272	chr10	43787412	43790417	-	3005	1	HNRNPA3I	0	0.014
NR_04664	chr3	11006098	11019224	-	581	1	SLC6A1-AS	0.621	0.542
NM_0010{	chr10	16823966	17129817	-	11864	1	CUBN|IFCI	0.539	0.606
NM_0183:	chr16	67159988	67164174	+	1594	1	FBXL8|FBL	1.335	2.724
NR_00332	chr15	25065025	25065122	+	97	1	SNORD116	0	0
NM_0124(	chr10	96364606	96513926	-	6748	1	TLL2|-|-|1	0.396	0.335
NR_04657	chr3	65893816	65925297	+	588	1	MAGI1-AS	0	0
NM_0012<	chr10	1.2E+08	1.2E+08	+	3158	1	INPP5F|M	20.312	22.176
NM_0011<	chr17	18658430	18682259	-	5320	1	ZNF286B|:	0.72	1.55
NR_02937	chr19	12666804	12675832	+	1425	1	WDR83|M	35.079	31.258
NM_0012{	chr2	2.19E+08	2.19E+08	-	2211	1	GLB1L|-|-|	5.54	3.754
NM_0010:	chr9	40223285	40266392	+	3494	2	ANKRD20A	0	0
NM_0060:	chr3	1.29E+08	1.29E+08	-	1504	1	H1FX|H1.1	247.865	309.795
NM_1989!	chr12	33375413	33439819	-	3277	1	SYT10|-|-|	0.063	0.088
NM_0047!	chr3	1.87E+08	1.87E+08	+	4573	1	ADIPOQ|A	0.006	0
NM_0319!	chr5	95658316	95683010	-	1221	1	SPATA9|N	0.074	0.191
NM_0012:	chrX	78747709	78756404	+	2148	1	LPAR4|GP	0.45	1.039
NR_02444	chrX	19986817	19989779	-	626	1	LOC72960!	0	0
NM_1525:	chr2	2.3E+08	2.3E+08	-	4391	1	SLC16A14|	4.874	5.297
NM_0325!	chrX	79170972	79172229	+	1255	1	GPR174|FI	0	0
NR_03166	chr4	1.47E+08	1.47E+08	-	88	1	MIR548G|	0	0
NR_01602	chr11	1.25E+08	1.25E+08	+	5017	1	TBRG1|NI/	10.038	10.899
NM_0000!	chr11	1.08E+08	1.08E+08	+	13082	1	ATM|AT1|	1.553	2.599
NM_0189:	chr12	46358188	46372862	-	4866	1	SLC38A2|/	21.889	24.706
NM_0070:	chr12	91050494	91058354	-	2525	1	KERA|CNA	0	0
NM_1768:	chr7	16859406	16881989	-	740	1	AGR3|AG-	0	0
NM_0010(	chr7	1.44E+08	1.44E+08	+	934	1	OR6B1|OF	0	0
NM_1984(	chr7	77696426	77779803	+	3210	1	RSBN1L|-|	2.976	4.322
NR_12585	chr6	68226970	68329899	-	511	2	LOC10192:	0	0
NM_0308:	chr1	70259002	70354734	-	5652	1	ANKRD13C	3.75	4.895
NM_0012{	chr5	1.28E+08	1.28E+08	+	2768	1	PRRC1|-|-	16.135	15.756
NM_0010{	chr20	4686511	4701588	+	2600	1	PRNP|ASC	31.951	46.428
NM_0013:	chrX	1.55E+08	1.55E+08	+	743	2	CTAG1B|C	0	0
NR_10374	chr19	23255054	23274251	-	5505	1	IPO5P1|-|	14.856	14.11
NM_0012!	chr11	1.19E+08	1.19E+08	+	1374	1	HMBS|PB(	16.713	21.443
NM_0013<	chr4	83460941	83485178	-	2787	1	FAM175A|	3.386	3.578
NM_0007:	chr1	1.81E+08	1.82E+08	+	14909	1	CACNA1E|	2.31	10.101
NR_02830	chr2	27889456	27891114	-	1658	1	BRE-AS1|-	0.257	0.438

NM_0012:	chr1	1.16E+08	1.16E+08	-	2687	1	CASQ2|PD	0.883	1.575
NM_0243:	chr1	1.54E+08	1.54E+08	+	2401	2	SLC27A3|/	0	0
NM_0011(	chr7	80458635	80512009	-	1174	1	GNAT3|GD	0.428	0.42
NM_0328:	chr11	1.23E+08	1.23E+08	+	6887	1	UBASH3B|	1.752	2.91
NM_1524(	chr17	50373220	50381459	+	2304	1	EME1|MM	5.165	5.231
NM_0333{	chr17	29625939	29930040	-	9197	1	SSH2|SSH-	8.173	9.96
NM_0011!	chr6	36948263	36964837	+	2075	1	PI16|CD36	0.099	0.086
NM_1990!	chr1	1.9E+08	1.9E+08	-	3127	1	BRINP3|DI	3.41	5.62
NM_0013:	chr7	1.3E+08	1.3E+08	+	1939	1	CPA5|-|-|`	0.146	0.078
NM_0013(	chr5	1.58E+08	1.58E+08	-	2154	1	SOX30|-|-	0.03	0
NM_0010(	chr11	54706832	54707902	+	1068	1	OR4A5|OF	0	0
NM_0013⁄	chr12	1.07E+08	1.08E+08	+	3778	1	BTBD11|A	3.983	4.847
NM_0010(	chr11	55773438	55774382	+	943	1	OR5D13|-	0	0
NM_0013(	chr15	45167214	45181207	-	1654	1	SHF|-|-|1!	100.458	111.216
NR_13523	chr19	46189029	46203062	-	1641	1	IGFL2-AS1	0	0
NM_0204(	chr6	89632224	89638755	-	5350	1	LYRM2|DJ	7.594	6.063
NR_02941	chr7	76549341	76627982	+	2683	2	LOC10013.	0	0
NR_11023	chr2	88538720	88575610	+	1744	1	LOC10192	3.26	4.147
NR_00336	chr2	94760928	94857075	-	3646	1	ANKRD20/	0	0.008
NM_0031!	chr1	23743366	23762059	+	4930	1	ELOA|SIII|	21.174	22.371
NM_0011{	chr1	1.62E+08	1.62E+08	+	1920	1	FCRLA|FCF	0.034	0
NM_0149:	chr6	53001300	53061802	-	6111	1	ICK|ECO|L	9.106	5.987
NR_00279	chr1	1.13E+08	1.13E+08	+	1323	1	AKR7A2P1	0.789	0.642
NM_1748!	chr19	7631615	7633748	-	770	1	PCP2|GPSI	0.368	0.107
NM_0022⁄	chr2	2.33E+08	2.33E+08	-	3604	1	KCNJ13|KI	0.039	0.027
NM_0012!	chr2	1.72E+08	1.72E+08	+	4013	1	CYBRD1|C	1.153	1.416
NM_0011:	chr17	75236592	75262393	-	2957	1	GGA3|-|-|	16.909	21.257
NM_0012{	chrX	1.55E+08	1.55E+08	-	4622	1	GAB3|-|-|	0.006	0.039
NM_0065!	chr2	1.76E+08	1.76E+08	+	1376	1	MTX2|-|-|	46.626	53.185
NM_0046(	chr17	11882902	11969748	+	3130	1	DNAH9|DI	0.078	0.149
NM_0012(	chr19	9324695	9382617	+	2831	1	ZNF559-ZN	4.878	11.167
NR_00306	chr1	1.53E+08	1.53E+08	-	676	1	SPRR2C|-|	0	0
NM_0011:	chr19	44671452	44684355	+	2235	1	CEACAM1!	1.188	2.366
NM_0336⁄	chr19	5690261	5691667	+	427	1	RPL36|L36	559.13	420.662
NR_11196	chr15_KI2	219103	315627	+	1806	2	GOLGA6L1	0.071	0.061
NR_00218	chr2	71024075	71029930	+	1227	1	OR7E91P|	0	0
NM_0227!	chr9	92613184	92670265	-	4386	1	IPPK|C9or	4.945	6.102
NM_1826(	chr1	2.07E+08	2.07E+08	+	3513	1	RASSF5|M	0.591	1.057
NM_0144(	chr3	14178728	14198369	+	689	1	LSM3|SM)	57.824	55.961
NM_0143:	chr14	49768069	49783138	+	1791	1	KLHDC2|H	39.818	51.24
NM_0011⁄	chr1	23084023	23168858	-	8415	1	LUZP1|LU:	4.894	6.861
NM_0013:	chr17	8339840	8346045	+	829	1	ODF4|CT1	0.062	0.083
NM_0010:	chr11	1.25E+08	1.25E+08	+	1425	1	HEPN1|-|-	0.072	0.98
NM_0012⁄	chr17	28394642	28406212	-	6402	1	SLC46A1|(	24.627	21.605
NM_0035⁄	chr6	26246611	26246977	-	365	1	HIST1H4G	0	0
NM_0032!	chr10	1.2E+08	1.2E+08	-	3877	1	TIAL1|TCB	15.943	16.357
NM_0012(	chr19	48624952	48630406	+	2676	1	SPHK2|SK	19.957	16.031
NR_02970	chr22	20033139	20033220	+	81	1	MIR185|M	0	0
NM_0012(	chr22	41909554	41914667	-	1400	1	SHISA8|C2	0.341	0.626
NM_0150:	chr17	28371662	28401038	+	7287	1	SARM1|M	30.402	25.997

NM_0318¦	chr19	53912737	53943715	+	2338	1	CACNG7|-	100.39	105.809
NM_0314¦	chr15	82986203	82991057	+	1566	1	FAM103A1	4.495	3.848
NR_11000	chr20	25955812	25969288	+	4600	1	LINC01733	0	0
NR_12063	chr10	1.25E+08	1.25E+08	+	1768	1	FAM53B-A	0	0.015
NM_0021(	chr2	1.27E+08	1.27E+08	+	1243	1	GYPC|CD2	11.192	5.124
NR_12538	chr8	78533892	78558503	-	2008	1	PKIA-AS1|	0.27	0.239
NR_02777	chr17_GL0	126335	181221	-	5040	3	PLEKHM1|	0	0
NM_0153	chr1	1.56E+08	1.56E+08	-	4539	1	SMG5|EST	32.286	32.479
NM_0148	chr1	22451851	22531157	+	8684	1	ZBTB40|ZN	6.955	7.025
NM_1979	chr2	1.98E+08	1.98E+08	-	2762	1	BOLL|BOU	0	0
NM_0013	chr1	1.11E+08	1.11E+08	+	2201	1	CEPT1|-|-	4.921	5.595
NM_0146(	chr17	3662893	3668679	-	1377	1	TAX1BP3|	50.844	42.424
NM_0011	chr9	1.31E+08	1.31E+08	-	3109	1	FIBCD1|-|	0.543	1.295
NM_0530	chr7	1.41E+08	1.41E+08	-	683	1	MRPS33|C	52.593	52.261
NM_0012	chr16	67173853	67175737	+	1382	1	NOL3|ARC	18.696	12.488
NM_0010(	chr12	25195216	25205015	+	1130	1	ETFRF1|LY	3.377	3.333
NM_0009	chr12	56731580	56752362	-	1447	1	PRIM1|p4	8.812	10.289
NR_02682	chr12	36661	38133	-	1123	3	FAM138D|	0	0
NM_0012	chr16	5084300	5097820	-	2215	1	EEF2KMT|	6.013	6.709
NR_11014	chr11	1.32E+08	1.32E+08	-	4327	1	LOC10192	0.006	0
NM_0013(	chr12	49097140	49110900	-	2268	1	LMBR1L|L	36.043	40.07
NM_0011	chr10	1.28E+08	1.28E+08	-	11399	1	MKI67|KIA	2.538	3.498
NM_0011	chr1	1.6E+08	1.6E+08	-	878	1	SLAMF9|C	0.044	0.016
NM_0012	chr20	21705659	21718486	+	5345	1	PAX1|HUP	0	0.062
NM_1382	chr6_GL0	3028390	3031755	+	2537.1	7	MPIG6B|C	0	0.011
NM_0002	chr8	19939071	19967259	+	3735	1	LPL|HDLC	1.512	1.207
NR_14439	chr17	42552558	42555214	+	1540	1	HSD17B1|	7.744	7.08
NR_03384	chr21	44989864	44994086	-	2153	1	LINC00163	0.06	0.051
NM_0013	chr1	43451003	43453989	-	1329	1	HYI|HT036	6.811	5.504
NM_0175	chr13	19633648	19673459	+	4252	1	MPHOSPH	8.014	8.449
NM_0032(	chr19	1609290	1652329	-	4430	1	TCF3|AGM	48.035	47.946
NM_1984	chr1	1.15E+08	1.15E+08	-	3954	1	DENND2C	0.251	0.08
NM_0012	chr16	58519946	58629886	-	8410	1	CNOT1|AD	24.051	26.544
NM_0010	chr11	13009423	13012106	+	2681	1	RASSF10|-	0.438	1.18
NM_0072	chr4	1.73E+08	1.73E+08	-	678	1	SCRG1|SC	7.303	37.091
NM_1523	chr1	20186078	20196048	+	5574	1	UBXN10|U	0.229	0.283
NM_0011	chr16	72782887	73058635	-	12765	1	ZFHX3|AT	14.961	4.054
NR_02741	chr17	43375928	43388898	-	3020	1	LINC0091C	0.201	0.268
NR_03611	chr10	96064315	96064399	-	84	1	MIR3157|	0	0
NR_11067	chr1	73306170	73307572	+	508	1	LINC0136C	0	0
NM_0012	chr19	38899700	38908894	+	986	1	NFKBIB|IK	13.286	12.35
NM_0013	chr17	63432303	63440211	-	2880	1	CYB561|C	10.87	8.442
NM_0329	chr5	1.51E+08	1.51E+08	+	2330	1	SMIM3|C5	2.678	3.668
NM_0011	chr9	1.28E+08	1.28E+08	-	2733	1	CIZ1|LSFR	59.698	55.58
NM_0012	chr14	59484443	59505406	+	2332	1	JKAMP|C1	15.503	15.024
NM_0011	chr6	1.09E+08	1.09E+08	-	2669	1	SESN1|PA	6.512	7.036
NM_0012	chr1	1.45E+08	1.45E+08	-	18310	1	NBPF20|-|	0.048	0.064
NM_0011	chr4	1.22E+08	1.22E+08	-	3534	1	TRPC3|SC	0.46	0.841
NM_0165	chrX	1.33E+08	1.33E+08	-	1677	1	TFDP3|CT	0	0.074
NM_0013	chr8_KI27	13212	190632	+	2307	2	ADAM32|-	0	0.006

NM_0247!	chr2	2.27E+08	2.27E+08	-	2408	1	TM4SF20|	0.011	0.034
NR_04618	chr1	2.14E+08	2.14E+08	+	1904	1	LINC0053ε	0.027	0
NM_0013.	chr21	43092956	43107606	-	942	2	U2AF1L5|-	0	0
NM_0036.	chr1	2.24E+08	2.24E+08	+	2058	1	DEGS1|DE	21.827	28.718
NR_02336	chr1	2.29E+08	2.29E+08	-	120	10	RNA5S7|R	0	0
NM_0037(	chr12	1.2E+08	1.2E+08	-	1157	1	SRSF9|SFR	137.872	133.324
NM_0012!	chr1	1.84E+08	1.84E+08	+	4763	1	RGL1|RGL	6.261	11.299
NR_03397	chr9	1.17E+08	1.17E+08	+	1744	1	ASTN2-AS:	0	0
NM_0049:	chr22	23145326	23165643	+	5108	1	RAB36|-|-	4.768	2.258
NM_0011.	chr21	43053191	43076392	-	2363	2	CBS|HIP4|	1.091	1.652
NM_0240!	chr7	42909273	42912090	-	1816	1	C7orf25|-|	5.318	5.815
NM_0011·	chr1	1.08E+08	1.08E+08	+	2119	1	NBPF6|-|-	0	0.013
NR_00394	chr1	1.74E+08	1.74E+08	-	53	1	SNORD78|	0	2.843
NM_0024:	chr5	80654648	80876815	+	4421	1	MSH3|DU	2.686	2.516
NM_1788·	chr1	16004236	16006695	+	923	1	C1orf64|E	0	0
NM_0322(	chr17	4740015	4746119	+	2292	1	ZMYND15	0.023	0.036
NM_0010ε	chr9	66986304	66992550	-	4217	1	SPATA31A	0	0
NR_04746	chr3	1.77E+08	1.77E+08	+	2061	1	LINC00501	0	0.04
NM_0013:	chr1	2.06E+08	2.06E+08	-	1614	1	FAM72A|L	0.104	0.042
NR_12036	chr15	80554611	80562944	-	2515	1	LOC10192!	1.205	1.274
NM_0013(	chr1	1.84E+08	1.84E+08	-	4723	1	COLGALT2	9.32	8.979
NR_10460	chr9	1.23E+08	1.23E+08	+	3949	1	CRB2|FSG!	11.094	5.487
NM_0011(	chr1	2.02E+08	2.02E+08	+	11654	1	NAV1|POI	34.251	42.593
NM_0013(	chr15	23439386	23447243	-	3141	1	GOLGA6L2	0.004	0.035
NM_0011:	chr15	40769961	40772449	+	2486	1	C15orf62|	1.291	1.256
NM_0158:	chr1	1.19E+08	1.19E+08	-	2798	1	WARS2|Tr	2.811	2.629
NM_1735.	chr3	1.14E+08	1.14E+08	+	4161	1	ZDHHC23|	0.177	1.073
NM_0012!	chr1	21822232	21937297	-	14244	1	HSPG2|HS	1.497	1.323
NR_05198	chr9	11987	14525	+	1652	1	DDX11L5|·	0	0
NM_0010:	chrX	1.03E+08	1.03E+08	+	1026	1	RAB40AL|I	0.101	0.027
NM_0013·	chr12	1.13E+08	1.13E+08	+	4372	1	RPH3A|-|-	0.08	1.407
NM_0019(	chr5	1.38E+08	1.38E+08	+	3132	1	EGR1|AT2	12.206	112.336
NM_0013(	chr20	60055925	60072953	+	2647	1	C20orf197	0.01	0
NM_0577·	chr8	94880225	94895254	-	2712	1	CCNE2|CY	0.708	1.152
NM_0012.	chr11	73264552	73298625	+	2394	1	P2RY6|P2Y	0.022	0.011
NM_1738·	chr14	21030186	21032173	+	791	1	TPPP2|C1·	0.033	0.069
NM_2071ε	chr11	6876625	6877619	+	992	1	OR10A4|J(	0	0
NR_03373	chr15	22258614	22282880	+	801	1	REREP3|-|	0	0.034
NM_0027:	chr7	1.14E+08	1.14E+08	-	4290	1	PPP1R3A|(	0	0.003
NM_0189:	chr5	1.41E+08	1.41E+08	+	2882	1	PCDHB15|	7.024	3.778
NR_12539	chr3	1.26E+08	1.26E+08	-	422	2	LOC10192.	0.367	0.292
NM_1980·	chr4	1.23E+08	1.23E+08	-	1094	1	NUDT6|AS	5.374	4.958
NM_1384(	chr7	1.02E+08	1.02E+08	-	999	1	MYL10|MY	0	0
NM_0124:	chr6	73593379	73654014	-	3301	1	SLC17A5|/	7.769	8.324
NR_13076	chr13	63828840	63844125	-	1526	1	LOC10272.	0	0
NM_0013(	chr5	1.35E+08	1.35E+08	+	3049	1	C5orf24|-|	8.005	7.695
NM_0011·	chr18	5143673	5197256	-	2298	1	AKAIN1|C:	0.017	0.149
NM_0010(	chr1	2.47E+08	2.47E+08	+	1339	1	OR2W5|O	0	0
NM_0060·	chr16	25692026	26137688	+	3199	1	HS3ST4|3-	5.252	1.079
NR_03161	chr2	70252918	70253005	-	87	1	MIR1285-2	0	0

NR_03992	chrX	6383906	6383963	-	57	1	MIR4770|·	0	0
NM_0013(	chr15	96783407	96785615	+	842	1	SPATA8|SI	0	2.017
NR_11012	chr14	95329584	95335504	+	1698	1	LOC10192!	0	0
NM_0184	chr12	67269281	67314692	+	5975	1	CAND1|TII	19.807	29.189
NM_1779!	chr20	36092710	36232799	+	5945	1	EPB41L1|4	46.931	40.015
NM_1525	chr4	81426393	81471928	-	2946	1	RASGEF1B	9.414	25.409
NM_0012	chr12	71439770	71586308	+	4518	1	LGR5|FEX|	0.599	0.206
NR_13526	chr1	1.56E+08	1.56E+08	-	694	1	C1orf61|C	175.251	388.214
NM_0189	chr5	1.41E+08	1.42E+08	+	4617	1	PCDHGC4|	20.037	80.755
NM_2034(	chrX	34129752	34132330	-	2576	1	FAM47A|-	0	0
NR_12054	chr11	32435518	32458769	+	1532	1	WT1-AS|W	0	0
NM_0012!	chr19	36014499	36045972	+	646	1	LOC10192	25.867	22.92
NM_0013	chr6	44216939	44218236	+	813	1	LOC10192!	0.016	0.185
NR_02961	chr7	1.3E+08	1.3E+08	-	109	1	MIR182|M	0	0
NM_0013	chr17	32007872	32053501	+	3205	1	LRRC37B|I	5.821	8.47
NM_0012!	chr21	10521514	10606270	+	2468	1	TPTE|CT4	0	0
NM_0013	chr4	1.59E+08	1.59E+08	+	6849	1	FNIP2|FNI	7.417	5.238
NR_13566	chr15	21735258	21735999	-	655	2	IGHV1OR1	0	0
NM_0153	chr7	44382366	44490786	-	4909	1	NUDCD3|I	40.167	56.024
NR_03981	chr9	1.28E+08	1.28E+08	+	87	1	MIR219B|	2.075	0.63
NM_1389(	chr11	1.25E+08	1.25E+08	-	1860	1	ESAM|W1	0.568	1.267
NM_0010(	chr1	1.04E+08	1.04E+08	+	1882	3	AMY1C|AI	0	0
NM_0011	chr17	4630919	4641665	-	2668	1	ALOX15|1	0.072	0.082
NM_0019	chr2	2.19E+08	2.19E+08	+	2237	1	DES|CSM1	0.092	0.184
NM_0203	chr10	79069035	79316528	+	7528	1	ZMIZ1|MI	70.099	69.542
NM_2073	chr2	1.3E+08	1.3E+08	-	1533	1	TUBA3E|-|	0	0.116
NR_03396	chr1	42959049	42983358	+	1120	1	SLC2A1-AS	1.059	0.477
NM_0010	chr1	1.55E+08	1.55E+08	-	6523	1	ADAR|AD	76.323	81.042
NM_0069(	chr15	71826020	72118099	-	8654	1	MYO9A|-|	2.905	3.412
NR_03899	chrX	1.35E+08	1.35E+08	+	1330	1	LINC0062!	0.078	0.257
NM_0018	chr10	5196835	5218947	+	1181	1	AKR1C4|3	0.033	0
NR_11023	chr2	1.44E+08	1.44E+08	+	1048	1	LOC10192	0.049	0.261
NR_03159	chr1	2.31E+08	2.31E+08	-	96	1	MIR1182|I	9.939	9.559
NM_0178(	chr1	1.51E+08	1.51E+08	+	1468	1	C1orf56|M	4.225	4.217
NM_0215	chr9	93951627	93955326	-	1476	1	BARX1|-|-	0.428	0.223
NM_1452!	chr19	43622370	43639839	-	2165	1	CADM4|IG	126.511	142.879
NR_01543	chr4	67701278	67722504	+	2288	1	UBA6-AS1	15.672	23.006
NM_0240(	chr7	6590021	6608726	+	2170	1	C7orf26|-|	18.419	23.354
NM_0011	chr11	14277920	14364506	-	2032	1	RRAS2|TC	3.02	4.577
NR_03618	chr12	1.23E+08	1.23E+08	-	61	1	MIR4304|·	0	0
NM_0013	chr17_KI2	217365	218245	+	1128.6	9	ARL17A|A	0.137	0.146
NR_00440	chr3	49152859	49154401	-	1542	1	LAMB2P1|	2.642	4.122
NM_0011	chrX	1.44E+08	1.44E+08	-	8738	1	SLITRK4|-|	0.246	0.439
NR_03970	chr10	1.14E+08	1.14E+08	-	61	1	MIR4483|·	0	0
NM_0060	chr14	77672406	77708013	-	2577	1	ALKBH1|A	6.985	7.425
NM_0048	chr5	1.4E+08	1.4E+08	-	3909	1	NRG2|DOI	0.172	0.929
NR_03038	chrX	8126965	8127061	+	96	1	MIR651|M	0	0
NM_1389(	chrX	89921941	89922883	+	843	1	TGIF2LX|T	0	0
NR_13237	chr11	1.26E+08	1.26E+08	-	420	1	STT3A-AS1	0.491	0.587
NM_0147	chr18	48539056	48863215	+	5724	1	CTIF|Gm6	69.896	74.345

NM_0010(	chr3	1.28E+08	1.28E+08	-	4225	1	MGLL|HU-	0.357	3.002
NM_1525;	chrX	22272913	22274459	+	1544	1	ZNF645|C	0	0
NM_0033	chr16	1309627	1327018	+	2851	1	UBE2I|C35	73.725	70.162
NM_0007	chr1	1.55E+08	1.55E+08	+	5710	1	CHRNB2|E	17.066	19.9
NR_03028	chr1	1.55E+08	1.55E+08	+	95	1	MIR92B|M	0.271	0
NM_0010!	chr22	24806221	24926846	+	5750	1	SGSM1|RU	3.355	3.471
NM_0047;	chr5	1.12E+08	1.12E+08	-	1990	1	NREP|C5o	543.314	661.118
NM_0224!	chr12	1.18E+08	1.18E+08	+	4714	1	SUDS3|SA	12.201	12.965
NR_02950	chr19	13836440	13836517	-	77	1	MIR27A|M	0	0
NR_13513	chr9	1.38E+08	1.38E+08	-	662	1	LOC10192	0	0
NM_0010:	chr17	18635529	18644427	-	1966	1	TBC1D28|	0	0.028
NR_12203	chr8	83403758	83408897	+	1516	1	LINC0141!	0	0
NR_10383	chr11	70072434	70075348	-	801	1	ANO1-AS2	0	0.034
NM_0013	chr8	14084835	15238283	-	6981	1	SGCZ|ZSG	0.211	0.065
NM_0011	chr2	1.87E+08	1.87E+08	+	6895	1	ITGAV|CD!	1.664	5.503
NM_0010	chr11	1.24E+08	1.24E+08	-	1101	1	TMEM225	0	0
NR_02201	chr15	25211692	25212033	+	341	1	PWAR4|P/	0	0
NM_1525!	chr7	73834591	73842525	-	922	1	WBSCR27|	0.769	0.208
NM_1992	chr5	1.63E+08	1.63E+08	+	2358	1	CCNG1|CC	25.235	17.908
NR_10803	chr5	44388732	44413989	+	1891	1	FGF10-AS1	0.177	0
NM_0011	chr11	22193176	22283367	+	6634	1	ANO5|GDI	1.512	1.259
NM_1450!	chr9	1.13E+08	1.13E+08	-	1206	1	RNF183|-|	0.043	0.08
NR_03787	chr1	1.59E+08	1.59E+08	-	3290	1	CADM3-AS	60.012	69.497
NR_10506	chr5	1.64E+08	1.64E+08	-	516	1	LINC02143	0	0
NR_03968	chr8	1.42E+08	1.42E+08	+	79	1	MIR4472-1	0	0
NM_1985	chr8	1.33E+08	1.33E+08	+	3229	1	PHF20L1|(	7.758	9.103
NR_03792	chr17	15436015	15563631	-	2758	1	TVP23C-CD	3.17	8.661
NM_0010(	chr1	450740	451678	-	937	3	OR4F3|-|-	0	0
NR_13508	chr11	62832234	62834041	+	561	1	LOC10536!	10.94	13.233
NM_0146!	chr7	1.43E+08	1.43E+08	-	4261	1	FAM131B|	58.446	62.805
NM_0029:	chr18	42743218	43115692	-	1095	1	RIT2|RIBA	7.654	7.78
NR_04972	chr11	88504576	88524054	+	4815	1	GRM5-AS1	1.063	0.939
NM_0175!	chr9	90609832	90643105	-	4382	1	DIRAS2|Di	12.002	19.014
NM_0013	chr19	58513530	58541712	-	2147	1	ZBTB45|ZN	15.896	16.242
NR_13500	chr20	7302089	7307432	-	1735	1	LINC01751	0	0
NR_03836	chr16	34158585	34160036	-	1451	1	LINC00273	0	0
NM_0010(	chr11	58402465	58403409	-	943	1	OR5B3|OF	0	0
NM_0011:	chr10	47487219	47491700	-	347	2	FAM25C|b	0	0
NM_1532	chr3	1.33E+08	1.33E+08	-	5369	1	NPHP3|CF	1.321	1.709
NR_04987	chr11	9090312	9090379	-	67	1	MIR5691|	0	0
NM_1304(	chr16	21402134	21425337	-	3602	1	NPIPB3|KI	0.397	0.483
NR_03622	chr2	2.39E+08	2.39E+08	+	83	1	MIR4269|	0	0
NR_03794	chr20	58651253	58679526	+	4095	1	STX16|SYN	14.997	15.062
NR_14444	chr19	58305374	58315657	+	1404	1	ERVK3-1|-	53.484	47.305
NR_07339	chrX	48301550	48306179	-	575	1	SSX9|SSX9	0	0
NM_0163	chr1	2.35E+08	2.35E+08	-	6041	1	ARID4B|B(	3.782	5.696
NM_0010	chrY	9337464	9340278	+	1152.5	4	TSPY3|CT7	0	0
NR_13458	chr22	43275942	43283826	+	1958	1	LOC10192	0.02	0
NM_0307(	chr1	22117305	22143026	-	3898	1	WNT4|SEF	11.352	1.388
NM_0165	chr4	70592258	70607287	+	1987	1	AMBN|AI1	0.019	6.425

NM_0010(	chr11	56346039	56346998	+	958	2	OR8K1|OF	0	0
NM_0530	chr7	1.56E+08	1.56E+08	+	10305	1	RBM33|PF	7.646	10.591
NM_0807	chr20	62904998	62926551	-	2788	1	DIDO1|BYI	21.579	18.433
NM_0055	chr3	1.94E+08	1.94E+08	+	1455	1	HES1|HES-	7.639	15.194
NR_03391	chr16	1878285	1884231	-	659	2	LINC00254	0	0
NM_0006	chr2	96112875	96116245	-	3368	1	ADRA2B|A	0.057	0.016
NM_2074(	chr4	42110853	42152878	-	8842	1	BEND4|CC	0.481	3.818
NR_00357	chr20	30377203	30399331	+	938	1	FRG1BP|C	6.103	6.951
NM_0012	chr1	1.55E+08	1.55E+08	+	3070	1	TRIM46|G	21.738	18.466
NM_0072	chr5	1.78E+08	1.78E+08	+	1719	1	B4GALT7|I	17.679	14.805
NM_0002	chr18	23873020	23955065	+	5577	1	LAMA3|BN	0.15	0.128
NM_2075	chr6	11102489	11111838	-	3300	1	ERVFRD-1|	0	0.004
NM_0045	chr9	91722596	91950162	-	4086	1	ROR2|BDE	4.112	2.457
NM_0179(	chr6	10694955	10709737	+	1483	1	PAK1IP1|N	6.651	8.359
NM_0043	chr12	1.2E+08	1.2E+08	+	567	1	COX6A1|C	194.562	153.421
NR_03977	chr1	12191713	12191773	+	60	1	MIR4632|-	0	0
NM_0208	chr20	34955835	35002437	+	6537	1	MYH7B|M	0.446	0.551
NM_0331	chr17	41065116	41065879	-	761	2	KRTAP2-4|	0	0
NR_02434	chr3	1.91E+08	1.91E+08	+	120	1	SNAR-I|sn	0	0
NM_0166	chr1	90915300	91021508	-	2085	1	ZNF644|BI	3.259	3.278
NR_00129	chr15	24981994	24982068	+	74	1	SNORD107	0.348	0.925
NM_0183	chr3	54122666	55074557	+	3635	1	CACNA2D3	2.685	4.718
NM_0012(	chr7	1.02E+08	1.02E+08	+	2787	1	CUX1|CAS	26.106	27.325
NM_0011	chr7	32485333	32495258	-	2180	1	LSM5|YER	11.728	11.353
NM_0013(	chr7	1044573	1059269	+	1860	1	GPR146|P	0.707	0.81
NM_0012	chr10	94545767	94602099	+	2833	1	HELLS|ICF	0.965	1.165
NM_0011	chr14	99511266	99604390	-	5663	1	CCDC85C|	20.871	17.141
NR_03606	chrUn_GL(	104742	104817	+	75	2	MIR3118-1	0	0
NM_0003	chr1	93992838	94121149	-	7273	1	ABCA4|AB	0.067	0.041
NM_0009	chr18	49488481	49492565	-	954	1	RPL17|L17	14.123	15.764
NM_0320	chr11	1.01E+08	1.01E+08	+	1853	1	TMEM133	0.195	2.905
NM_0011	chr1	77888515	77943893	+	3183	1	NEXN|CMI	0.073	0.189
NM_0027	chr1	1.09E+08	1.09E+08	-	3815	1	PSMA5|PS	24.428	23.147
NR_10977	chr15	51918963	51924120	-	452	1	LOC10042	0.456	0.848
NM_0014	chr7	1.55E+08	1.55E+08	+	3391	1	EN2|-|-|7	13.053	6.515
NM_0021	chr12	53097652	53102344	+	960	1	IGFBP6|IB	2.74	2.939
NR_12592	chr4	92262498	92277302	-	1222	1	LNCPRESS	0	0
NM_0013	chr2	2.03E+08	2.03E+08	+	2723	1	CARF|ALS	3.068	2.968
NM_0013	chr2	8852690	9003750	-	7451	1	MBOAT2|I	6.953	8.682
NM_0010	chr22	28794560	28800572	-	1786	1	XBP1|TREI	60.591	63.239
NM_0053	chr12	57028517	57050117	-	3591	1	MYO1A|BI	0.018	0.008
NM_0011	chr19	39776594	39786135	+	668	1	LEUTX|-|-	0	0
NR_03386	chr4	1.88E+08	1.89E+08	+	408	2	LINC0106C	0.063	0.134
NR_03990	chr19	804940	805001	+	61	1	MIR4745|	1.057	0
NR_13445	chr8	1.25E+08	1.25E+08	-	406	1	LOC10537	0.095	0
NR_02403	chr9	1.16E+08	1.16E+08	-	561	1	LINC00474	0	0
NM_0207	chr11	78188812	78214711	+	4298	1	USP35|-|-	8.955	9.554
NM_0540	chr9	1.3E+08	1.31E+08	+	1769	1	ASS1|ASS|	23.724	14.433
NM_0144	chr12	6821797	6827418	+	1507	1	GPR162|A	66.17	54.016
NR_03964	chr3	1.1E+08	1.1E+08	+	69	1	MIR4445|-	0	0

NM_0122!	chr3	12796672	12834814	+	4226	1	CAND2|TII	5.4	4.664
NM_0123	chr5	43602689	43705566	+	4654	1	NNT|GCCD	18.451	20.101
NM_0149(	chr2	1.65E+08	1.65E+08	-	9468	1	COBLL1|C(	0.082	0.104
NM_1527:	chr16	57092543	57147966	+	2220	1	CPNE2|CO	22.145	42.06
NR_03607	chr2	11767444	11767525	+	81	1	MIR548S|-	0	0
NR_02412	chr17	3310245	3311446	+	1201	1	OR3A4P|C	0	0
NM_0010:	chr20	31465506	31473013	-	213	1	DEFB124|I	0	0
NR_14445	chrX	2904904	2906122	+	855	1	ARSD-AS1|	4.177	2.932
NR_13555	chr1	1.48E+08	1.48E+08	+	552	1	LOC10050.	0	0
NM_0142(	chr12	6828373	6839854	+	2655	1	P3H3|GRC	12.432	6.769
NR_02654	chr21	44517215	44518977	+	1042	1	TSPEAR-AS	2.623	1.775
NM_0033!	chr4	39498756	39527598	-	3181	1	UGDH|GD	8.175	6.312
NM_0163:	chr11	3675010	3797792	-	6938	1	NUP98|AD	12.942	13.699
NR_00272	chr11	2608328	2699998	-	91670	1	KCNQ1OT:	0.028	0.016
NR_12579	chr2	74909131	74938418	+	489	1	LINC01291	0	0.056
NM_1984!	chr7	1.44E+08	1.44E+08	+	2623	2	CTAGE4|c	0	0
NM_1471!	chr3	46701333	46710923	+	1855	1	TMIE|DFN	0.375	0.421
NR_03764	chr1	1.8E+08	1.8E+08	-	2652	1	LHX4-AS1|	2.83	2.303
NR_02839	chr1	2.32E+08	2.32E+08	+	2601	1	TSNAX-DIS	4.566	4.092
NM_1523:	chr14	49598697	49614672	+	1739	1	LRR1|4-1B	2.543	3.371
NM_0012(	chr6	43620481	43627353	-	2817	1	GTPBP2|-|	29.389	29.101
NM_0013:	chr17	74466373	74484798	+	1449	1	CD300A|C	0	0
NM_0029!	chr16	57372461	57385048	+	3313	1	CX3CL1|AI	5.647	8.641
NM_1984(	chr13	45192853	45201040	-	2115	1	KCTD4|bA	1.018	0.298
NM_0156!	chr22	45413691	45432422	+	1396	1	RIBC2|C22	0.083	0.098
NR_02727	chr21	13943776	13980444	-	1082	1	ANKRD20A	0	0
NM_0012(	chr7	1.51E+08	1.51E+08	-	1534	1	WDR86|-|	5.589	8.858
NR_02686	chr15	87576929	87579686	+	1783	1	LINC00052	0.137	0
NM_0035!	chr17	3397466	3398410	-	943	1	OR1E1|HG	0	0
NR_11190	chr10	1.33E+08	1.33E+08	-	467	1	ADGRA1-A	2.816	0.997
NM_0013	chr3	43366326	43691594	-	2440	1	ANO10|SC	9.284	11.704
NR_03837	chr7	53655509	53811931	-	3478	1	LINC01446	0	0
NM_1825:	chr2	1.87E+08	1.87E+08	-	2435	1	ZSWIM2|N	0.016	0.011
NM_0046(	chr16	30085794	30091884	-	1791	1	TBX6|SCD(	0.389	0.161
NM_0011(	chr3	1.47E+08	1.47E+08	-	3972	1	ZIC4|-|-|3	18.626	9.897
NM_0062	chr12	11391540	11395564	-	1422	1	PRB2|IB-9	0	0
NM_0007:	chr6	87085498	87095147	-	745	1	CGA|CG-A	0.381	0.221
NM_1735:	chr8	61287966	61501645	+	3484	1	CLVS1|CR/	4.023	7.835
NM_0012(	chr21	17917340	18267371	+	2027	1	CHODL|C2	12.009	2.784
NM_0012(	chr8	1.44E+08	1.44E+08	-	1591	1	TMEM249	3.063	2.514
NM_0154:	chr6	1.39E+08	1.39E+08	+	1488	1	CCDC28A|	2.47	2.817
NM_0010(	chr1	2.48E+08	2.48E+08	-	928	1	OR14A16|	0	0
NM_0010:	chr6	87344853	87365463	+	1297	1	C6orf163|	0.328	0.528
NR_03163	chr8	1.43E+08	1.43E+08	+	46.5	4	MIR1302-7	0	0
NR_11075	chr1	29329620	29350114	-	2145	1	LINC01756	0.024	0
NM_0012	chr19	23914882	23933967	+	1959	1	ZNF726|-|	0.151	0.217
NM_0011!	chr5	1253172	1295047	-	3807	1	TERT|CMN	0.014	0.007
NR_11087	chr6	1.63E+08	1.63E+08	-	1512	1	PACRG-AS:	0	0
NM_0046	chr14	23320188	23326185	+	3093	1	PABPN1|C	42.295	36.401
NM_0012!	chr8	7332387	7333979	+	1591	2	USP17L1|U	0.024	0.052

NR_12580	chr8	24490312	24514868	-	937	1	LOC10192!	0.041	0
NR_10373	chr2	97283935	97291878	+	800	2	LOC10050(	0	0
NM_0023{	chr5	1.13E+08	1.13E+08	-	8204	1	MCC|MCC	1.43	1.189
NR_02434	chr10	59958217	59960913	-	1813	1	LINC01553	0.007	0
NM_0019:	chr11	61299447	61333212	-	4327	1	DDB1|DDE	146.447	151.693
NM_0038:	chr19	51611503	51630474	-	2512	1	SIGLEC5|C	0	0.011
NR_03996	chr4	40502040	40502119	-	79	1	MIR4802|-	0	0
NM_0011:	chr4	1715952	1721357	-	2831	1	TMEM129	21.515	17.737
NM_0013:	chr1	53921561	53946308	-	604	1	HSPB11|C:	24.442	23.108
NM_0058!	chr12	1.33E+08	1.33E+08	-	9226	1	GOLGA3|G	12.569	9.022
NR_03029	chr3	1.69E+08	1.69E+08	+	95	1	MIR551B|	0	0
NM_0011:	chr1	1.86E+08	1.86E+08	+	4636	1	PRG4|CAC	0.423	0.343
NM_0331‹	chr7	1.35E+08	1.35E+08	+	4190	1	CALD1|CD	4.268	8.604
NM_0011!	chr2	1.7E+08	1.7E+08	+	1249	1	PHOSPHO:	1.538	1.491
NM_0330!	chr7	99890407	99919600	-	3372	1	TRIM4|RN	1.059	5.337
NM_0012:	chrX	1.32E+08	1.32E+08	-	3333	1	RAP2C|-|-	5.191	3.238
NM_1990‹	chr16	57976435	57988113	+	1611	1	TEPP|-|-|1	0.984	0.349
NM_0012(	chr3	9917074	9933621	+	2318	1	IL17RC|CA	3.588	4.432
NR_12589	chr4	1.74E+08	1.74E+08	-	1323	1	LOC10192:	0.068	0.021
NM_0013(	chr16	74377952	74392114	+	1379	1	NPIPB15|/	1.019	0.576
NM_1447(	chr20	62122461	62135378	+	2583	1	LSM14B|C	58.867	64.752
NM_0185!	chr2	1.4E+08	1.42E+08	-	16438	1	LRP1B|LRF	0.491	0.715
NM_0000!	chr9	1.35E+08	1.35E+08	+	8371	1	COL5A1|E	2.851	4.33
NM_0004(	chr22	19723543	19724774	+	954	1	GP1BB|BD	25.841	43.431
NM_0153!	chr20	32207893	32238664	+	5210	1	POFUT1|D	9.986	11.405
NM_0059!	chr17	61399896	61409466	+	3371	1	TBX2|-|-|:	0.164	0.256
NM_0013(	chr19	9853718	9858168	-	1685	1	OLFM2|N(	140.543	157.195
NR_03393	chr4	14111968	14140052	+	1981	1	LINC01085	0.039	0.014
NM_0013:	chr19	4174109	4182604	-	1504	1	SIRT6|SIR:	25.35	22.986
NM_0321‹	chr4	99511004	99542303	+	1625	1	C4orf17|-|	0	0
NM_1723:	chr14	23372809	23376403	+	1162	1	IL25|IL17E	0.022	0
NM_1386!	chr7	1.31E+08	1.31E+08	-	1478	1	KLF14|BTE	0	0.009
NM_0209!	chr19	17323223	17334829	-	4132	1	ANO8|KIA.	23.21	21.384
NM_0174:	chr5	1.38E+08	1.38E+08	-	6789	1	KLHL3|PH/	1.314	1.747
NM_0182{	chr13	48037567	48047228	+	2099	1	NUDT15|N	7.181	5.364
NM_0045(	chr2	2.27E+08	2.28E+08	+	8651	1	AGFG1|HF	5.622	7.65
NM_0010:	chr4	48831043	48861817	+	1842	1	OCIAD1|A:	56.11	67.95
NR_13433	chr7	1.33E+08	1.33E+08	+	749	1	LOC10050(	0	0
NM_0132{	chr11	64103890	64119173	+	3243	1	FLRT1|SPG	9.598	9.938
NM_0247:	chr13	1.13E+08	1.13E+08	-	1422	1	GRTP1|TB	4.388	4.074
NM_0011‹	chr16	88715338	88785220	-	8036	1	PIEZO1|DH	0.711	0.934
NM_0160:	chr12	21501765	21518403	+	3265	1	GOLT1B|C	3.523	3.075
NM_0160(	chr7	1.01E+08	1.01E+08	-	732	1	FIS1|CGI-1	136.791	127.633
NR_04971	chr17	14029292	14069458	-	1941	1	COX10-AS:	1.388	1.934
NR_12048	chr12	54353691	54467030	+	688	1	LOC10272‹	1.031	1.234
NR_13804	chr7	38979895	39013567	-	2329	1	POU6F2-A:	0.011	0.018
NM_0323:	chr2	73228006	73233284	-	1132	1	PRADC1|C	26.083	17.871
NM_0221:	chr3	1.4E+08	1.41E+08	+	4863	1	CLSTN2|AI	19.832	12.782
NR_03765	chr12	55716034	55724742	+	1207	1	BLOC1S1-F	33.19	32.319
NR_04624	chr1	1.81E+08	1.81E+08	+	256	1	LINC0169!	0.101	0

NM_1827	chr7	1E+08	1E+08	-	2960	1	MCM7|CD	74.242	72.081
NM_0175	chr1	2.48E+08	2.48E+08	+	934	1	OR2M4|H	0	0.015
NM_0009	chr1	6185020	6199619	-	2079	1	RPL22|EAI	47.611	54.577
NM_1734	chr4	70902340	70988174	+	6957	1	MOB1B|M	2.339	2.987
NR_13504	chr12	41764189	41765581	+	337	1	LOC10192	0	0
NM_1532	chr17	56153475	56482646	+	2407	1	ANKFN1|-	0.348	0.945
NM_0251	chr15	1.02E+08	1.02E+08	-	1322	1	TM2D3|BL	33.599	44.945
NR_10676	chr2	2.18E+08	2.18E+08	-	63	1	MIR6513|	0	0
NM_0012	chr19	18306230	18323191	-	1777	1	LSM4|GRF	74.662	67.468
NM_0012	chr19	38908980	38912158	-	1051	1	CCER2|-|-	11.593	1.434
NM_2069	chr19	45485288	45497055	-	2057	1	RTN2|NSP	64.612	62.818
NR_13506	chr11	1.17E+08	1.17E+08	+	580	1	LOC10192	0	0
NM_0008	chr5	1.62E+08	1.62E+08	+	2508	1	GABRA6|-	0.072	0
NM_0010	chr19	13764514	13774282	+	3178	1	MRI1|M1F	4.532	3.37
NM_0013	chr1	78044962	78138449	+	3539	1	GIPC2|SEN	0.044	0.054
NM_0028	chr3	12583601	12664201	-	3256	1	RAF1|CMD	66.954	84.34
NM_0013	chr19	48325999	48330553	+	638	1	EMP3|YM	25.666	14.642
NR_03413	chr4	3673593	3677855	-	1030	1	LOC10013	0	0
NR_13567	chr15	72407779	72474239	-	748	1	LOC10537	0.069	0.055
NR_11025	chr2	1.63E+08	1.63E+08	+	2994	1	LOC10192	0	0.05
NR_03337	chr17	15030975	15031957	+	764	1	CDRT7|LIN	0	0
NM_0153	chr22	38734714	38756019	-	3981	1	SUN2|UN	79.563	87.832
NM_1536	chr11	89659297	89698718	+	1958	1	FOLH1B|F	0	0
NM_0012	chr8_KI27	24049	25641	+	1591	2	USP17L4|-	0	0.052
NM_0161	chr7	56101562	56106630	-	956	1	CHCHD2|C	0.607	0.115
NM_0012	chr9	87726059	87731469	+	1555	1	CTSL|CATL	32.047	27.297
NM_0165	chr2	2.4E+08	2.41E+08	-	3256	1	ANKMY1|Z	2.558	2.747
NM_0058	chr16	680111	682768	+	1597	1	STUB1|CH	69.974	58.545
NM_0323	chr9	83938312	83956742	-	2461	1	C9orf64|-|	0	0.05
NR_03394	chr3	1.94E+08	1.94E+08	-	1612	1	LINC0202	0	0.008
NM_1384	chr12	1.13E+08	1.13E+08	-	1364	1	IQCD|493	3.346	3.254
NM_0246	chr3	1.12E+08	1.12E+08	+	2270	1	C3orf52|T	0.199	0.205
NM_1383	chr17	29568644	29573157	+	1521	1	TP53I13|D	16.242	16.507
NM_0010	chr16	55656440	55703788	+	3535	1	SLC6A2|N	0	0.004
NM_0044	chr14	76371347	76501837	+	3001	1	ESRRB|DFI	3.609	10.68
NM_0010	chr16	1349240	1351872	-	1196	1	TSR3|C16	53.741	49.497
NM_0009	chr3	1.46E+08	1.46E+08	-	3975	1	PLOD2|BR	1.975	1.334
NM_0164	chr5	1.39E+08	1.39E+08	-	821	1	MZB1|ME	0	0.167
NM_0010	chr19	41845023	41852329	+	1809	1	DMRTC2|-	0	0
NM_0013	chr20	43590613	43647296	+	2034	1	IFT52|C20	49.171	44.189
NM_0010	chr6_GL00	3240253	3242105	+	905.2	6	SAPCD1|C	0	0
NR_13659	chr1	2.24E+08	2.24E+08	+	1861	1	LOC10028	0.63	0.294
NM_0230	chr10	1.21E+08	1.22E+08	-	3858	1	FGFR2|BBI	5.434	6.305
NM_0132	chr7	1.31E+08	1.31E+08	+	11163	1	MKLN1|TV	2.169	4.253
NM_0011	chr14	21090046	21104704	-	2929	1	ZNF219|ZI	32.32	28.834
NR_13487	chr16	287440	352676	-	3446	1	AXIN1|AXI	15.445	17.704
NM_0012	chr19	39246314	39248856	-	1627	1	IFNL4|IFN	0	0
NM_0017	chr8	22245106	22251167	+	1918	1	POLR3D|B	16.947	14.875
NM_0177	chr5	1.4E+08	1.41E+08	+	8185	1	ANKHD1|I	10.874	12.422
NR_10697	chr16	14925937	14926003	+	66	7	MIR6511A	0	0

NM_0011(	chr7	1.56E+08	1.56E+08	-	2228	1	CNPY1|-|-	3.854	1.715
NM_0187;	chr15	52192318	52295798	-	6932	1	MYO5C|-|	0.32	0.854
NM_0010(	chr7	1.42E+08	1.42E+08	-	910	2	PRSS58|PF	0	0
NM_0013:	chrX	57677067	57680260	-	3192	1	NLRP2B|C	0	0.051
NM_0206!	chr19	57351274	57359897	+	4414	1	ZNF304|-|	7.586	8.698
NM_0010!	chr10	43556345	43574618	-	1902	1	ZNF239|H	3.539	4.623
NM_0312{	chr1	26279722	26281522	+	1110	1	SH3BGRL3	150.308	106.047
NR_10463	chr5	40052291	40053324	+	560	1	LINC00603	0	0
NR_02715	chr16	22437008	22492220	+	4981	1	SMG1P1|-	0.062	0.085
NM_1450(	chr6	1.17E+08	1.17E+08	-	2199	1	ZUFSP|C6(	4.708	5.5
NM_0007{	chr7	92112149	92134526	-	3196	1	CYP51A1|(	22.434	15.004
NR_03978	chr5	1.81E+08	1.81E+08	-	67	1	MIR4638|	0.385	0
NM_0181(	chr11	71453171	71501535	+	2407	1	NADSYN1|	12.079	12.075
NM_0031:	chr1	11054592	11060034	-	1263	1	SRM|PAPT	47.878	42.121
NR_13190	chr13	24331450	24337120	+	567	1	LINC00566	0	0
NR_00216	chr13	27945206	27945573	+	367	1	ATP5EP2|-	78.485	56.579
NR_13091	chr6	79307669	79313384	-	3882	1	LCAL1|onc	0.11	0.035
NM_0012!	chr2	2.32E+08	2.32E+08	+	2465	1	DIS3L2|FA	11.387	12.186
NR_03397	chr4	64914281	65004500	-	4072	1	LOC40113	0.465	0.737
NM_0122{	chr17	42156892	42181278	-	3904	1	KCNH4|BE	0.611	1.116
NM_0328;	chr2	70295976	70302088	-	1802	1	FAM136A|	16.514	19.421
NR_12166	chr3	1.14E+08	1.15E+08	-	25386	1	ZBTB20|DI	0.469	1.991
NM_0011(	chr5	1.36E+08	1.36E+08	-	2575	1	TRPC7|TRI	0.01	0
NR_13804	chr13	22876392	22916369	-	1165	1	LINC00621	0.155	0.705
NR_10988	chr5	1.25E+08	1.26E+08	+	1332	1	LOC10192	0.039	0.134
NM_0217:	chr10	97677424	97687258	-	1387	1	AVPI1|PP5	2.566	4.651
NR_10436	chr1	57860543	57862982	+	855	1	DAB1-AS1	0	0
NR_10681	chr11	86278333	86278398	+	65	1	MIR6755|	0.793	0.843
NR_03625	chr9	1.37E+08	1.37E+08	+	66	1	MIR4292|	4.298	5.811
NM_0013(	chr1	52842511	52894575	+	3504	1	ZYG11A|Z	0.074	0.59
NM_2069;	chr9	1.37E+08	1.37E+08	+	3631	1	MAMDC4|	2.834	2.867
NR_13526	chr14	1.03E+08	1.03E+08	+	339	1	LOC10537	0	0
NM_0057:	chr6	1.49E+08	1.49E+08	+	4381	1	UST|2OST	8.429	4.008
NR_10996	chr22	47461299	47487111	-	981	1	LINC01644	0	0
NR_04641	chr13	48296513	48303661	-	1140	1	LINC00441	0.486	0.469
NM_1734(	chr5	1.78E+08	1.79E+08	-	3032	1	COL23A1|	3.717	0.971
NM_0012{	chr6	44254101	44257571	-	1712	1	SLC35B2|F	38.763	47.308
NM_0011	chr11	1.19E+08	1.19E+08	+	3595	1	ABCG4|WI	2.797	3.524
NM_0010(	chr11	4923374	4924339	-	964	1	OR51G1|C	0	0
NM_0060(	chr13	75549750	75606020	+	961	1	UCHL3|UC	10.358	13.269
NR_00355	chr18	32511663	32514634	+	2971	1	WBP11P1|	3.255	2.752
NM_0011!	chr12	89519413	89526262	-	5490	1	POC1B-GA	0.716	0.629
NR_04656	chr11	70631094	70635490	+	2249	1	SHANK2-A	0.023	0.018
NM_2073!	chr19	35487324	35490531	-	485	1	KRTDAP|K	0	0.028
NR_10467	chr8	21298239	21309449	+	1630	1	LOC10192	0	0
NM_0047{	chrX	1.04E+08	1.04E+08	+	1201	1	TCEAL1|SI	16.78	15.18
NM_0004;	chr12	52514575	52520459	-	2292	1	KRT5|CK5	0	0.042
NR_10378	chr1	2.05E+08	2.05E+08	-	2614	1	BLACAT1|	6.886	6.906
NR_02827	chr4	99160594	99161647	-	1053	1	PCNAP1|p	0.024	0.013
NM_0336:	chr20	35953617	35954626	-	898	1	SCAND1|R	87.11	72.878

NR_11072	chr1	1.14E+08	1.14E+08	-	638	1	HIPK1-AS1	0.162	0.236
NM_0120	chr20	23079356	23086340	-	6697	1	CD93|C1Q	0.006	0.014
NM_0028	chr3	61561569	62294898	+	6616	1	PTPRG|HP	26.011	12.658
NR_13518	chr16	86720684	86721959	-	513	1	LOC10192	0	0
NM_0152	chr6	46852506	46954938	-	5733	1	ADGRF5|G	0.083	0.041
NR_03029	chr3	44861888	44861981	+	93	1	MIR564|M	0.555	0
NM_2019	chr11	64764604	64778844	-	2934	1	SF1|BBP|D	118.968	115.631
NM_0011	chr2	64988445	65023866	+	3725	1	SLC1A4|AS	29.463	40.775
NM_0012	chr5	69504177	69558104	+	5382	1	OCLN|BLC	0	0.005
NM_0021	chr9	21304614	21305313	-	697	1	IFNA5|IFN	0	0
NR_04980	chr1	2.28E+08	2.28E+08	-	93	1	MIR5008|	0	0
NM_1532	chr11	62602219	62612765	-	3231	1	EML3|ELP	2.917	4.841
NM_0150	chr1	10210706	10381603	+	10532	1	KIF1B|CM	74.444	82.835
NM_0013	chr7	1.42E+08	1.42E+08	-	3458	1	CLEC5A|CL	0	0.008
NM_0323	chr2	32277889	32306589	+	1957	1	YIPF4|FinG	9.388	9.757
NM_0010	chr8	1.02E+08	1.02E+08	-	3561	1	NCALD|-|-	105.121	47.045
NR_03398	chr16	35746552	35756624	+	1595	1	FLJ26245|	0	0
NM_0010	chr1	1.68E+08	1.68E+08	+	3323	1	DCAF6|12	9.269	16.582
NM_0010	chr1	1.59E+08	1.59E+08	+	1030	1	OR6K6|OR	0	0
NM_0011	chr12	74537771	74541452	+	3679	1	ATXN7L3B	159.509	190.924
NR_12636	chr11	1.17E+08	1.17E+08	+	561	1	APOA1-AS	0.046	0.049
NM_0030	chr8	20144855	20183206	-	2735	1	SLC18A1|C	2.385	0.601
NM_0010	chr11	62712625	62727385	-	5131	1	HNRNPUL2	55.295	59.326
NM_0040	chrY	23129355	23199092	-	4389	1	DAZ1|DAZ	0	0
NM_0022	chr12	52806543	52814116	-	2136	1	KRT4|CK-4	0	0
NM_0147	chr16	30957294	30984662	+	6428	1	SETD1A|KI	9.67	10.196
NM_0146	chr10	46549049	46556071	-	1818	1	GPRIN2|G	2.567	1.288
NM_0003	chr17	19648751	19677595	+	3690	1	ALDH3A2|	16.821	25.627
NM_0011	chr16	9753405	10182067	-	13989	1	GRIN2A|EI	0.21	0.365
NM_0142	chr22	37807905	37816899	+	1493	1	GCAT|KBL	7.85	7.073
NM_0327	chr6	1.27E+08	1.27E+08	+	4576	1	RSPO3|CR	1.2	0.494
NM_1758	chr7	1.07E+08	1.07E+08	-	6824	1	CCDC71L|	3.786	5.983
NM_0011	chr19	40691529	40715106	-	2059	1	COQ8B|AD	29.062	27.401
NR_01544	chr16	2603384	2630494	+	2710	1	LOC65227	0.971	0.839
NR_10686	chr19	58550285	58550376	+	91	1	MIR6807|	12.893	15.202
NM_0040	chr18	580343	582020	+	1675	1	CETN1|CE	0	0.008
NR_11053	chr22	18970498	19031242	+	1320	1	DGCR5|LIN	6.144	6.267
NM_0046	chrY	17768980	17770560	-	1578	2	XKRY|XKR	0	0
NM_1392	chr8	22146830	22156831	-	3256	1	LGI3|LGIL	0.238	0.387
NM_0011	chr12	9881489	9895833	+	724	1	KLRF2|NK	0	0
NM_0021	chr2	2.2E+08	2.2E+08	+	1427	1	INHA|-|-|	0.795	0.787
NM_0010	chr11	1.12E+08	1.12E+08	-	1240	1	PIH1D2|-|	2.215	1.127
NM_1943	chr9	34252380	34329200	-	6212	1	KIF24|C9o	1.31	1.197
NM_0056	chrX	1.41E+08	1.41E+08	-	2072	1	SOX3|GHD	11.114	20.129
NR_04660	chr1	11143898	11149538	+	702	1	MTOR-AS1	3.673	3.083
NM_1528	chr3	1.27E+08	1.27E+08	+	1779	1	CHST13|C	0.08	0.031
NM_0140	chr1	2.13E+08	2.13E+08	+	5927	1	FLVCR1|AX	3.637	3.238
NR_12576	chr10	1.08E+08	1.08E+08	-	716	1	LINC01435	0	0
NM_0026	chr12	98593625	98602000	+	1668	1	SLC25A3|C	255.52	225.015
NM_0051	chrX	71118556	71142454	+	6922	1	MED12|AR	16.915	16.944

NR_12652	chr16	20901073	20925011	+	1154	1	LYRM1|A2	26.614	27.358
NM_0011:	chr4	98871684	98929092	-	10169	1	EIF4E|AUT	1.767	2.053
NR_02989	chr11	75335092	75335186	-	94	1	MIR326|M	0	0
NR_02707	chr15	89504930	89524034	-	2349	1	LINC0092٤	0.445	1.592
NM_0220(	chr11	1.25E+08	1.25E+08	+	3715	1	PKNOX2|P	12.366	19.338
NM_0153(	chr11	94128928	94181971	+	2761	1	PANX1|MI	21.762	22.979
NM_1527٤	chr17	35854946	35868891	-	1962	2	HEATR9|C	0	0
NR_03412	chr10	1.3E+08	1.3E+08	-	2663	1	LINC0095٩	0.581	0.638
NM_1735(	chr17	62380553	62416478	+	1532	1	EFCAB3|-|	0	0
NR_00392	chr1	84364958	84384801	-	1314	1	UOX|UOXI	0.069	0.021
NM_0012!	chr5	1.51E+08	1.51E+08	-	2507	1	TNIP1|ABI	21.786	15.549
NM_2073:	chr5	1.22E+08	1.22E+08	+	1960	1	ZNF474|-|	0.316	0.335
NM_0011:	chr16	2798485	2805132	+	951	1	PRSS41|TE	0	0
NR_03839	chr19	35540738	35546029	-	1623	1	TMEM147	4.894	3.891
NM_0063:	chr17	43014605	43022442	-	2750	1	VAT1|VAT	261.883	175.521
NR_13428	chr5	1.15E+08	1.15E+08	+	749	1	LINC01957	0	0
NM_0040:	chr1	1.51E+08	1.51E+08	-	4097	1	CTSS|-|-|1	0.094	0.187
NR_04640	chr19	50384323	50418018	+	3335	1	POLD1|CD	9.457	7.639
NM_0011!	chr19	43580544	43582104	+	569	1	PINLYP|-|-	4.804	3.707
NM_0055٤	chr1	1.53E+08	1.53E+08	+	2131	1	IVL|-|-|1q	0	0
NM_0245!	chr12	1691081	1788679	+	3955	1	ADIPOR2|/	20.662	32.554
NM_1735٤	chr8	98064522	98093610	+	1545	1	ERICH5|C٤	0.417	0.284
NM_0031:	chr5	1.48E+08	1.48E+08	-	603	1	SPINK1|PC	0	0
NM_0020٤	chr9	86944362	86947189	-	2825	1	GAS1|-|-|!	9.197	5.905
NM_0141٤	chr1	1541673	1574882	-	1296	1	SSU72|HSI	101.894	99.652
NR_13525	chr14	38882596	38919200	+	353	1	LOC10537(	0	0
NM_1825٤	chr2	1.08E+08	1.08E+08	+	7147	1	RGPD4|RG	0.005	0.008
NM_0011(	chr17	36495755	36535457	-	4373	2	MYO19|M	0	0
NM_0010(	chrX	70040542	70049938	-	2114	1	AWAT2|AI	0.061	0.013
NM_0013:	chr19	48748011	48753660	-	3985	1	FUT1|H|H	0.673	0.588
NM_1451٤	chr16	31201486	31202776	-	712	1	PYCARD|A	0.199	0.173
NM_0065!	chr7	44200979	44214294	+	2757	1	YKT6|-|-|7	85.395	87.548
NM_0014٤	chr8	81280483	81284777	+	725	1	FABP5|E-F	0.196	0.227
NM_0005:	chr2	1.13E+08	1.13E+08	-	1489	1	IL1B|IL-1|I	0	0
NM_0005:	chr2	1.76E+08	1.76E+08	+	2323	1	HOXD13|E	0.705	0.041
NM_0012٤	chr10	49154724	49188400	-	1505	1	C10orf128	0.308	0.364
NM_1451:	chr10	68230595	68232113	-	1516	1	ATOH7|M.	0.629	0.876
NM_1813:	chrX	1.54E+08	1.54E+08	+	1782	1	TAZ|BTHS	13.125	12.736
NM_0010(	chr11	1.22E+08	1.22E+08	-	859	1	BLID|BRC(	0	0
NM_0062:	chr1	1.54E+08	1.54E+08	+	589	1	S100A1|S1	0.46	0.186
NR_12372	chr1	1.6E+08	1.6E+08	+	2312	1	PEA15|HM	284.738	333.444
NM_0051(	chr22	25564894	25729291	+	9032	1	GRK3|ADR	3.73	5.277
NR_13509	chr11	87718354	87719411	-	677	1	LOC10536!	0	0
NM_0012٤	chr19	54040826	54063953	-	802	10	VSTM1|SII	0	0
NR_00258	chr11	8684227	8684356	+	129	1	SNORA3A|	3.798	3.079
NM_0012!	chr2	15940438	15947007	+	1817	1	MYCN|MC	9.494	19.69
NM_0807!	chr20	2536607	2641784	+	3147	1	TMC2|C2C	0.053	0.087
NM_0031٤	chr2	9488486	9555820	-	4360	1	ADAM17|/	8.33	8.316
NM_0318٤	chr19	49067418	49072941	-	4368	1	KCNA7|HA	0.006	0.082
NM_0219(	chr9	68356899	68531061	+	3325	1	PGM5|PGI	5.797	0.128

NR_13494	chr3	1.96E+08	1.96E+08	-	255	1	LINC01983	0.506	0.054
NM_0011:	chr4	76011180	76112802	+	1525	1	ART3|ART(	0.034	0.072
NM_1527!	chr7	84995556	85121931	-	6239	1	SEMA3D|S	0.153	0.255
NM_0049(	chr12	14612634	14696585	-	3813	1	GUCY2C|D	0	0
NM_1783!	chr1	1.53E+08	1.53E+08	+	385	1	LCE4A|LEF	0	0
NR_03063	chrX	1.37E+08	1.37E+08	+	82	1	MIR934|M	0	0
NR_10372	chr7	1E+08	1E+08	+	725	1	STAG3L5P	3.326	2.362
NM_1524:	chr7	1.49E+08	1.49E+08	-	3262	1	ZNF786|-|	5.273	5.072
NM_0802(	chr17	69078706	69141874	-	5255	1	ABCA6|ES	0.01	0.005
NM_0191:	chr5	1.41E+08	1.41E+08	+	4409	1	PCDHB9|P	1.322	1.528
NM_0013(	chr11	76381303	76386541	+	5236	1	LOC10050(	5.632	5.122
NM_0011:	chr4	76251700	76283783	+	1544	1	FAM47E|-	1.553	0.532
NM_0327(	chr6	32168206	32178111	-	2033	7	AGPAT1|1	0.641	0.411
NM_0049!	chr12	57487953	57516655	+	2892	1	MARS|CM	79.813	90.499
NR_03790	chr2	2.38E+08	2.38E+08	+	2953	1	UBE2F-SCL	9.728	8.906
NR_13617	chr3	1.52E+08	1.52E+08	-	286	1	LOC10192:	0	0
NR_02724	chr13	19262800	19344973	-	3705	1	ANKRD26F	0	0
NM_0010:	chr15	22869465	22917313	-	2453	2	CYFIP1|P1	7.984	11.145
NR_10809	chr7	56534230	56538130	-	2739	1	LOC10192:	0	0
NM_0809:	chr3	98795970	98901689	-	6075	1	DCBLD2|C	11.206	7.001
NR_04630	chr22	43151514	43163242	+	667	1	TSPO|BPB.	30.813	44.397
NM_0012:	chr4	1.4E+08	1.4E+08	-	1328	1	MGAT4D|(	0	0
NM_0010(	chr9	1.23E+08	1.23E+08	+	940	1	OR1J4|HSI	0	0
NM_0010:	chr20	13785026	13818421	+	2196	1	NDUFAF5|	12.336	14.283
NR_03978	chr6	1.7E+08	1.7E+08	+	83	2	MIR4644|-	0	0
NR_07639	chr19	5691834	5720165	-	2883	1	LONP1|CC	40.384	41.424
NM_0010(	chr16	2211602	2214821	-	3132	1	PGP|AUM	12.082	10.36
NM_0806:	chr20	36777442	36863684	-	14352	1	SOGA1|C2	34.658	32.671
NR_13630	chr9	1.28E+08	1.28E+08	+	2049	1	LOC10272:	1.026	0.849
NM_0011(	chr1	2.24E+08	2.24E+08	-	2404	1	CAPN8|nC	0.504	0.051
NR_12156	chr9	93008115	93014944	-	3642	1	LOC10192	0.205	0.338
NR_10977	chr9	79135423	79145666	-	2418	1	LOC10192	0	0
NM_0012!	chr10	5889572	5937595	+	3643	1	FBXO18|FI	23.281	24.687
NM_0011(	chr7	56114681	56116397	-	596	1	NUPR2|NU	0	0
NR_03377	chr14	64345473	64347611	-	1043	1	TEX21P|-|	0.247	0.368
NM_1768(	chr7	1.43E+08	1.43E+08	+	1015	1	TAS2R39|	0	0
NR_11048	chr15	22015261	22095613	+	1090	1	LOC10192	0	0
NM_0217:	chr17	40092784	40100725	-	2765	1	NR1D1|EA	6.57	11.646
NM_1522(	chr1	54756898	54764553	-	2382	1	PARS2|MT	3.924	4.589
NM_0212(	chr20	46173733	46308498	-	3889	1	CDH22|C2	2.735	2.12
NM_0012(	chr16	47081520	47144025	-	3660	1	NETO2|BT	21.073	31.773
NM_0012:	chr5	37812677	37834822	-	3633	1	GDNF|ATF	0.153	0.147
NM_0250(	chr3	1.61E+08	1.61E+08	+	1285	1	ARL14|ARI	0	0
NM_0013:	chr19	36034985	36054762	-	1605	1	THAP8|-|-	12.99	10.778
NM_0012(	chr17	42968088	42980528	-	1997	1	PTGES3L|-	3.338	2.936
NM_0189!	chr16	2682494	2709031	+	2456	1	KCTD5|-|-	27.535	29.954
NM_0012!	chr10	98134624	98244897	+	1562	1	R3HCC1L|(	5.398	4.656
NM_0141(	chr14	24209581	24213830	-	1348	1	CHMP4A|(	30.225	27.201
NM_0059(	chr20	49982976	49988883	+	1701	1	SNAI1|SLU	1.023	0.652
NR_03643	chr3	1.02E+08	1.02E+08	+	2293	1	FAM172BF	0.011	0

NR_11057	chr2	1.7E+08	1.7E+08	-	1307	1	LOC10192	0	0
NM_0032	chrX	1.01E+08	1.01E+08	-	3809	1	TSPAN6|T	11.864	14.47
NM_0010	chr2	73254682	73269630	-	6915	1	FBXO41|F	9.079	13.98
NM_0012	chr8	1.43E+08	1.43E+08	-	1886	1	TOP1MT|-	4.409	7.183
NM_2135	chr19	14440274	14471867	+	2923	1	PKN1|DBK	81.035	71.418
NM_0055	chr19	3366567	3469217	+	7794	1	NFIC|CTF|	0.817	4.601
NM_0163	chr8	17156327	17222732	+	3997	1	ZDHHC2|D	4.477	4.366
NM_0013	chr3	69108672	69122595	-	2215	1	LMOD3|N	0.093	0
NM_1344	chr19	55836578	55881854	+	3662	1	NLRP4|CLI	0	0
NM_1722	chr8	1E+08	1E+08	+	3728	1	SPAG1|CIL	0.688	0.338
NM_1991	chr10	44377062	44385097	-	1928	1	CXCL12|IR	0.334	1.918
NR_03034	chr12	80832533	80832629	-	96	1	MIR617|M	0	0
NR_00273	chr16	2155023	2155105	-	82	1	SNORD60|	1.258	1.336
NM_0012	chr19	53867865	53876435	+	2983	1	MYADM|S	17.065	10.056
NM_1784	chr1	1.53E+08	1.53E+08	+	422	1	LCE3C|LEP	0	0
NM_0002	chr15	58431976	58568874	+	1593	1	LIPC|HDLC	0.008	0
NM_0011	chr5	1.39E+08	1.39E+08	-	3248	1	PROB1|C5	1.143	1.21
NM_0329	chr12	15107782	15221477	-	2318	1	RERG|-|-|	4.761	0.898
NM_0014	chr22	41092610	41180077	+	8728	1	EP300|KA	17.427	13.022
NR_13657	chr22	20340997	20352494	+	706	1	LOC10192	0	0
NR_12057	chr11	1.27E+08	1.27E+08	+	2320	1	LOC10192	0	0.012
NM_0064	chr9	92031134	92115474	-	2840	1	SPTLC1|HS	9.729	9.612
NM_0010	chr11	59477430	59478365	+	934	1	OR4D10|C	0	0
NR_03387	chr17	72598041	72640472	-	2260	1	LINC00511	0.011	0.061
NR_01537	chr10	1159768	1164672	+	2431	1	LINC0020C	0	0
NM_0012	chr2	26848289	26950351	+	5111	1	DPYSL5|CI	262.716	269.58
NM_0013	chr11	1.24E+08	1.24E+08	-	4102	1	ZNF202|ZI	8.462	10.094
NM_0031	chr6	42744496	42746146	-	1648	1	TBCC|CFC	31.577	33.229
NM_1942	chr5	34929593	34958964	+	6193	1	DNAJC21|	2.067	1.309
NM_0012	chr10	80356768	80368073	+	1717	1	DYDC2|-|-	1.787	1.236
NM_0011	chr1	1.7E+08	1.7E+08	+	932	1	METTL11B	0.567	0.603
NR_03838	chr12	98485544	98503855	-	1821	1	LOC64377	0.227	0.226
NM_0010	chr8	7482504	7486387	-	299	3	DEFB106B	0	0
NM_0004	chr6	1.29E+08	1.3E+08	+	9625	1	LAMA2|LA	1.478	0.403
NR_11054	chr22	31335349	31338021	+	420	1	PIK3IP1-AS	0.368	0.554
NR_13093	chr17	81665036	81666146	-	696	1	OXLD1|C1	16.802	18.912
NM_0019	chr5	1.07E+08	1.08E+08	-	5328	1	EFNA5|AF	6.21	1.959
NM_0030	chr3	1.87E+08	1.87E+08	+	4272	1	ST6GAL1|	8.442	12.36
NR_10706	chr16	17052	17119	-	67	4	MIR6859-2	0.385	0.204
NM_0246	chr5	56099680	56233359	-	2486	1	ANKRD55|	0.736	0.595
NM_1738	chr1	2.47E+08	2.47E+08	+	1072	1	VN1R5|V1	0.048	0.026
NR_04985	chr1	1.71E+08	1.71E+08	+	59	1	MIR1295B	0	0
NR_04623	chr8	95207007	95216374	-	516	1	LINC01298	0	0
NM_0001	chr11	5253191	5254781	-	578	1	HBG2|HBC	0	0
NM_0151	chr15	41417104	41483563	+	5001	1	RTF1|GTL7	27.909	31.697
NM_0013	chr10	73375101	73414085	-	2401	1	ANXA7|AN	17.512	17.707
NR_02950	chr7	1.31E+08	1.31E+08	-	63	1	MIR29A|M	0	0
NM_1825	chr17	76265205	76271298	+	1584	1	UBALD2|F	25.689	27.878
NM_0011	chr14	18967434	19003752	+	6654	1	POTEM|A	0	0
NM_0024	chr11	1.03E+08	1.03E+08	-	1117	1	MMP7|MI	0	0.012

NM_0039:	chr10	91923979	92030323	+	6998	1	BTAF1|MC	3.864	6.101
NM_0581	chr21	7744921	7758630	+	894	2	SMIM11A|	0	0
NM_0011	chr20	38805693	38923024	+	6125	1	PPP1R16B	1.511	5.414
NM_0011	chr12	54292107	54301037	-	1677	1	NFE2|NF-E	0.315	0.131
NM_0012	chr10	1.33E+08	1.33E+08	-	4010	1	TUBGCP2|	30.022	32.302
NM_0048	chr19	3750773	3761675	-	2145	1	APBA3|MC	7.622	7.618
NR_12166	chr6	1.59E+08	1.59E+08	-	817	1	LOC10192	0.032	0.084
NM_0034	chr17	35809455	35847242	+	2164	2	TAF15|Np	0	0
NM_0010	chr16	85805	138698	-	2718	1	NPRL3|C1	23.339	23.403
NM_0069	chr9	5334932	5339873	-	1000	1	RLN1|H1|	0.142	0.014
NM_1387	chr9	1.22E+08	1.22E+08	+	3842	1	MRRF|MR	5.309	5.647
NM_0012	chr9	36833275	37034479	-	8545	1	PAX5|ALL3	6.374	0.539
NM_1456	chr17	18018020	18039166	-	1542	1	ATPAF2|A	12.526	11.947
NM_0202	chr19	4278601	4290723	+	2568	1	SHD|-|-|1	25.385	17.761
NM_0009	chr20	3071620	3072517	+	508	1	OXT|OT|C	0.178	0.054
NM_0059	chr17	82752064	82943186	+	3251	2	TBCD|PEB	20.139	20.114
NM_0055	chr6_GL0	2834226	2837604	-	1601	6	HLA-B|AS|	2.239	2.233
NM_0029	chr16	57358783	57366190	+	2913	1	CCL22|A-1	0	0
NM_0013	chr8	1.1E+08	1.1E+08	+	3071	1	LOC10013	0.185	0.08
NM_0307	chr14	51240168	51257654	+	4093	1	TMX1|PDI	4.754	4.384
NM_0124	chr10	89414568	89421002	+	4030	1	IFIT5|ISG5	6.943	7.151
NM_1526	chr22	21632797	21637327	+	2185	1	CCDC116|	0.212	0.401
NM_0012	chr1	1598008	1600096	-	2086	1	FNDC10|C	12.541	15.037
NM_0121	chr3	33277442	33387265	+	2983	1	FBXL2|FBL	11.843	10.455
NM_0007	chr1	1.6E+08	1.6E+08	+	5432	1	ATP1A2|F	22.302	20.182
NM_2015	chr6	43059594	43075099	+	2386	1	KLC4|KNS	37.519	32.274
NM_0012	chr4	73054696	73069759	-	4526	1	COX18|CC	4.384	4.322
NR_03644	chr16_KI2	1975582	2009798	+	6264	2	PKD1P1|H	0.049	0.052
NM_0181	chr3	1.87E+08	1.87E+08	-	2705	1	TBCCD1|-|	7.379	7.352
NM_1533	chr4	16160505	16226538	-	4572	1	TAPT1|CM	3.7	4.772
NR_12579	chr9	74473435	74498553	-	819	1	RORB-AS1	0.22	0.084
NR_10998	chr3	1.81E+08	1.81E+08	-	2859	1	LOC10192	0	0.005
NR_03839	chr20	22054090	22074654	+	673	1	LINC01432	0	0
NR_04751	chr12	53962308	53968914	-	2333	1	HOTAIR|H	0	0.006
NR_04617	chr8	23084355	23103557	+	1237	1	LOC25489	0.188	0.199
NR_13490	chr19	14305458	14363990	-	569	1	LINC01841	0.181	0
NR_12604	chr9	39873853	39875395	+	1426	2	LOC10272	0	0.058
NM_1524	chr17	35192520	35194393	-	1871	1	SLC35G3|	0	0
NM_2073	chrX	55089052	55092836	+	494	1	PAGE2|CT	0.026	0.055
NM_1781	chr3	1.4E+08	1.4E+08	-	1918	1	NMNAT3|	0.437	2.242
NR_10691	chr9	98229149	98229217	-	68	1	MIR6854|	1.706	1.41
NR_13511	chr11	37938601	37954663	+	373	1	LOC10537	0	0
NR_03982	chr9	1.37E+08	1.37E+08	-	58	1	MIR4673|	7.336	7.321
NM_0010	chr11	1.34E+08	1.34E+08	-	929	1	THYN1|HS	53.795	58.562
NR_12043	chr12	10363769	10398506	+	2826	1	LOC10192	0.014	0.034
NM_0011	chr2	1.76E+08	1.76E+08	+	1603	1	HOXD8|H	0.048	0.077
NR_12050	chr6	15247815	15248696	-	515	1	JARID2-AS	0.175	0.053
NM_0039	chr11	1389899	1462689	+	4071	1	BRSK2|C1	45.287	54.576
NM_0015	chr7	76302558	76304297	+	892	1	HSPB1|CM	59.105	33.091
NR_10404	chr4	68184129	68218080	-	1373	1	TMPRSS11	0	0

NR_12655	chrX	1.2E+08	1.2E+08	-	276	1	LINC01402	0.327	0.05
NM_00132	chr2	2.19E+08	2.19E+08	+	4294	1	FAM134A|	40.677	49.984
NM_0012!	chr4	83535914	83605874	+	2620	1	GPAT3|AG	0.103	0.089
NM_0145	chr14	30893799	31026401	-	3952	1	STRN3|PPI	4.241	5.112
NM_1452!	chr17	47823272	47831541	-	1742	1	MRPL10|L	35.424	35.06
NR_13238	chr2	2.16E+08	2.16E+08	+	2168	1	LINC01614	0.006	0.006
NM_0013(	chr6	1.24E+08	1.25E+08	+	2976	1	NKAIN2|F/	5.099	2.817
NM_0016	chr1	2.04E+08	2.04E+08	+	8719	1	ATP2B4|AT	36.272	32.397
NM_0012!	chr22	31496139	31618551	+	4193	1	SFI1|PISD|	5.427	4.753
NM_0149	chr9	96754549	96778129	-	6545	1	ZNF510|-|	4.009	5.914
NR_12058	chr11	66473490	66480249	-	842	1	LOC10192	3.721	4.099
NR_11082	chr3	1.13E+08	1.13E+08	-	2410	1	LINC02044	0.487	0.415
NM_1989(	chr12	49637941	49707414	-	11012	1	FMNL3|FH	5.522	7.625
NM_0010(	chr1	2.48E+08	2.48E+08	+	937	1	OR2L3|-|-	0	0
NR_00001	chr9	1.33E+08	1.33E+08	+	70	1	SNORD36E	3.315	8.61
NM_0530	chr4	10439880	10457408	-	6931	1	ZNF518B|-	1.598	3.17
NM_0151!	chr11	73400478	73598189	-	7268	1	FAM168A|	59.468	70.854
NR_00395	chr22	23390605	23402612	-	2542	2	ZDHHC8P1	0	0
NM_0068	chr14	24336021	24340036	-	1909	1	RIPK3|RIP	0.027	0.022
NR_10698	chr8	2076589	2076640	+	51	2	MIR7160|I	0	0
NR_07723	chr8	78722848	78724374	-	1526	1	LOC10124	0.321	0.449
NM_00132	chr1	1.7E+08	1.7E+08	-	1419	1	METTL18|	2.735	1.766
NM_1528	chr17	63484817	63498380	+	3248	1	ACE|ACE1	0.492	0.409
NM_0013(	chr2	1.58E+08	1.58E+08	-	2576	1	CCDC148|	0.415	0.239
NM_0152	chr12	48766191	48789096	-	6078	1	ADCY6|AC	17.232	17.814
NM_0010	chr19	45498473	45502506	+	1707	1	PPM1N|-|	0.967	1.428
NR_12590	chr4	83968082	84299169	-	2829	1	LOC10192	0	0
NM_0334(	chr11	33746745	33774525	-	1640	1	FBXO3|FB/	15.732	17.898
NR_10273	chr9	1.34E+08	1.34E+08	-	2231	1	DBH-AS1|I	0.636	0.516
NM_0248	chr1	92298965	92388175	+	3097	1	RPAP2|C1	1.744	1.99
NM_0032(	chr4	38796255	38804791	-	2845	1	TLR1|CD2	0.063	0.539
NM_0010	chrX	70925580	70931125	-	2229	1	SLC7A3|AT	6.843	8.683
NM_0808	chr20	13221771	13300650	+	2584	1	ISM1|C20	0.08	0.011
NR_12580	chr3	1.29E+08	1.29E+08	-	2694	1	LINC01565	0	0.005
NM_0052!	chr2	1.9E+08	1.9E+08	-	2814	1	MSTN|GDI	0.082	6.279
NM_0013(	chr18	32190024	32220403	+	2291	1	MEP1B|-|	0.006	0.036
NM_0176	chr11	63985853	63998420	+	2285	1	OTUB1|HS	70.748	62.928
NM_0009!	chr1	78491043	78540701	+	5355	1	PTGFR|FP	0.053	0.005
NM_0011	chr15	75369379	75455783	-	6581	1	SIN3A|WI	16.677	17.015
NM_0047	chr3	65359269	66038834	-	6152	1	MAGI1|AII	15.897	15.106
NM_0018	chr3	1.49E+08	1.49E+08	+	1677	1	CPA3|MC-	0	0
NM_0011	chrX	63634968	63785546	-	4842	1	ARHGEF9|	34.462	44.454
NM_0048!	chr2	1E+08	1E+08	-	2845	1	CHST10|H	23.145	48.029
NR_02961	chr19	13874699	13874808	+	109	1	MIR181C|	0	1.005
NM_0013(	chr16	28863757	28874213	+	1960	1	SH2B1|PSI	26.557	23.096
NM_0013	chr10	99875571	99914092	-	4178	1	DNMBP|A	2.426	2.488
NM_0011	chr9	34398184	34458570	-	3550	1	FAM219A|	59.677	64.8
NM_0012	chr15	45631148	45691294	+	1797	1	SQRDL|CG	0.818	0.549
NM_0033	chr11	2928273	2929420	-	916	2	PHLDA2|B	0	0
NM_0010	chr1	26282282	26306704	-	1355	1	UBXN11|C	13.588	8.238

NM_1449	chr16	2817163	2821722	+	1154	1	PRSS21|ES	0	0
NM_0013	chr3	98732236	98795845	+	3313	1	ST3GAL6|S	0.58	0.653
NR_03838	chr12	89011126	89019692	-	2506	1	LOC72808	0.072	0.202
NR_03383	chr2	64185079	64205485	-	3300	1	LINC0030	0	0
NM_0011	chr17	43780435	43784686	+	820	1	C17orf105	0.094	0.267
NM_0010	chr9	1.29E+08	1.29E+08	+	3324	1	GLE1|GLE	16.8	19.161
NM_0011	chrX	1.07E+08	1.07E+08	+	3148	1	CLDN2|-|-	0.033	0.026
NR_13046	chr1	1.6E+08	1.6E+08	-	100	1	MIR4259|	0	0
NR_12032	chr1	1.46E+08	1.47E+08	+	1404	1	CH17-408	0	0
NM_2033	chr11	34051683	34099060	+	3533	1	CAPRIN1|	119.227	117.57
NM_0012	chr21	5022493	5036782	+	3133.5	2	ICOSLG|B7	0.008	0.004
NM_0063	chr9	35161992	35405335	+	6329	1	UNC13B|N	41.909	27.416
NR_11067	chr19	32025862	32048899	+	747	1	LINC01533	0	0
NR_11194	chr1	13094515	13099717	-	2210	4	PRAMEF34	0	0
NM_0124	chr2	37344610	37373322	+	1694	1	QPCT|GCT	0.099	0.703
NM_0306	chr18	33578577	33751194	+	11387	1	ASXL3|BRP	4.008	4.731
NM_0051	chr1	1.46E+08	1.46E+08	-	4952	1	RBM8A|B(	25.178	20.084
NR_03785	chr1	2.14E+08	2.14E+08	-	3393	1	PROX1-AS	0.243	0.597
NM_0146	chr6_GL00	1954920	1972793	-	7351	8	MDC1|NFI	0	0.002
NM_0321	chr14	44504151	44507296	-	3143	1	FSCB|C14c	0	0
NM_0012	chr11	18531403	18588746	-	2346	1	UEVLD|AT	1.957	2.668
NR_12602	chr7	1.44E+08	1.44E+08	+	941	2	OR2A1-AS	0	0
NM_1736	chr14	35046404	35083383	+	2919	1	FAM177A1	11.772	10.225
NM_1779	chr20	54475718	54651171	+	1724	1	DOK5|C20	26.445	44.77
NM_0190	chr10	72273919	72276039	+	1747	1	DDIT4|Dig	119.362	62.503
NM_1985	chrX	70177483	70205703	+	1702	1	DGAT2L6|	0	0
NM_0332	chr1	2.47E+08	2.47E+08	-	4192	1	ZNF670|-|	1.892	2.062
NM_0012	chr10	96343216	96357159	-	3283	1	OPALIN|H	0	0
NM_0004	chr3	1.87E+08	1.87E+08	+	2006	1	HRG|HPR(	0	0.027
NR_04655	chr3	2110409	2144241	-	423	1	CNTN4-AS	0	0
NR_02677	chr6	1.68E+08	1.68E+08	-	1646	1	LINC01558	0.063	0.083
NM_0011	chr1	1.07E+08	1.07E+08	+	6018	1	NTNG1|Ln	2.46	1.935
NM_0139	chr6	29439939	29440977	+	1036	8	OR10C1|C	0	0
NR_10674	chr16	3485381	3485469	-	88	1	MIR6126|	7.765	10.428
NM_0058	chr7	1.01E+08	1.01E+08	+	928	1	POP7|061	40.195	43.128
NR_02444	chr22	23638488	23717423	-	3404	1	GUSBP11|	7.344	8.856
NM_0011	chr19	49363730	49375116	+	985	1	DKKL1|CT	4.739	3.657
NR_00217	chrY	22851584	22852715	+	164	3	TTTY17C|N	0	0
NM_0012	chr22	29788608	29838304	-	2574	1	ASCC2|AS	20.031	22.578
NR_03965	chr4	85722468	85722533	+	65	1	MIR4451|	0	0
NR_03742	chr11	1.19E+08	1.19E+08	+	68	1	MIR3656|	27.493	17.524
NM_0033	chr22	19330701	19431696	-	3986	1	HIRA|DGC	22.918	26.635
NM_0012	chr15	75355792	75368627	-	2958	1	MAN2C1|I	14.105	12.674
NR_03892	chr7	1.52E+08	1.52E+08	+	788	1	PRKAG2-A	1.914	0.73
NR_10371	chr9	41647674	41649440	+	1766	1	FAM27E2|	0.015	0
NM_0010	chr19	50854915	50860764	+	1328	1	KLK3|APS|	0	0
NM_0011	chr16	12901620	12911650	+	4176	1	SHISA9|CK	1.788	1.683
NM_2074	chr17	34574123	34579369	-	3993	1	C17orf102	0.026	0.178
NM_0013	chr2	27364352	27370052	-	1638	1	EIF2B4|EIF	36.461	35.597
NR_03341	chr17	41626327	41640199	-	2853	1	KRT42P|-|	0	0.01

NM_0010!	chr1	46545644	46551215	-	2388	1	KNCN|Kin(	0.13	0.264
NM_0010(	chr20	23602410	23605973	-	1685	1	CST9|CLM	0.015	0
NM_0312{	chr2	85133460	85310388	+	2904	1	TCF7L1|TC	5.887	6.056
NR_10675	chr17	49510817	49510900	+	83	1	MIR6165|I	0	0
NM_0012⁄	chr9	1.15E+08	1.15E+08	-	3777	1	TNFSF8|CI	0	0
NR_02683	chr2	1.99E+08	1.99E+08	+	3314	1	SATB2-AS1	0	0
NR_12602	chr6	1.7E+08	1.7E+08	-	1950	1	LOC28580⁄	0	0
NR_00275	chr1	11908154	11908272	+	118	1	RNU5E-1|I	0	0.813
NR_00320	chr14	1.01E+08	1.01E+08	+	73	1	SNORD11⁄	0	0
NR_11008	chr7	90335223	90344096	+	2662	1	LOC10192	0.01	0
NM_0010(	chr19	19264365	19273265	-	1471	1	TM6SF2|-|	0.307	0.242
NM_1532	chr19	19538265	19546659	+	4189	1	CILP2|CLIF	1.834	1.413
NM_0013⁄	chr12	1.24E+08	1.24E+08	+	2388	1	ZNF664-RF	3.774	4.485
NM_0210(	chr6	26158121	26158607	+	484	1	HIST1H2BI	31.355	24.309
NM_0189	chr5	1.41E+08	1.42E+08	+	4596	1	PCDHGA5|	20.109	80.99
NR_12042	chr13	99740870	99957165	-	538	1	LOC10192	4.05	0.28
NM_0027(	chrX	12791355	12824227	+	2522	1	PRPS2|PR!	2.173	1.928
NM_1780	chr15	42067683	42094554	-	3562	1	PLA2G4D|	0.087	0.027
NM_2073!	chr1	27379176	27383333	-	1480	1	CD164L2|I	0.017	0
NM_0147(	chr2	75646783	75662208	+	7819	1	MRPL19|L	2.619	3.081
NM_0013(	chr10	1.33E+08	1.33E+08	-	623	1	FUOM|C1(	2.049	1.473
NM_0011	chr6	49834266	49866505	-	1869	1	CRISP1|AE	0.069	0
NR_00438	chr17	64146078	64146157	+	79	1	SNORD10⁄	8.65	6.762
NM_0010(	chr1	1.59E+08	1.59E+08	-	973	1	OR6K2|OF	0	0
NM_0530	chr9	1.1E+08	1.1E+08	+	9470	1	PALM2|AK	0.219	0.341
NR_03813	chr1	2.03E+08	2.03E+08	+	2804	1	MGAT4EP	0.221	0.176
NR_13074	chr4	3941760	3955428	-	1908	1	FAM86EP|	0.953	0.869
NM_0066{	chr1	1.97E+08	1.97E+08	+	1432	1	CFHR4|CFI	0	0
NR_10372	chr2	1.73E+08	1.73E+08	+	3057	1	PDK1|-|-|	15.403	2.679
NR_04000	chr17	5772234	5930696	+	1817	1	LOC33916	0.213	0.151
NM_0012{	chr1	94896949	94925808	-	1893	1	CNN3|-|-|	134.506	153.612
NR_03746	chr15	76586647	76586691	+	44	1	MIR3713|-	0	0
NM_0224!	chr10	94333226	94362976	-	3469	1	NOC3L|AD	1.732	1.982
NM_0042	chr7	6161781	6272611	-	4452	1	CYTH3|AR	12.372	11.642
NM_0011	chr5	79069724	79090074	+	2450	1	BHMT2|-|	0.011	0
NM_0019(	chr8	11842525	11868150	-	3783	1	CTSB|APP!	68.881	86.23
NM_0143	chr3	1.7E+08	1.7E+08	+	2007	1	GPR160|G	0.128	0.136
NM_1738	chr12	63558913	63668574	-	4033	1	DPY19L2|!	0.569	0.479
NM_0045!	chr12	1.32E+08	1.32E+08	+	3229	1	SFSWAP|S	18.108	20.34
NM_0011!	chr6_GL0C	4392468	4400883	+	2799.3	7	BRD2|D6S	0.355	0.377
NR_04984	chr18	39676719	39676777	-	58	1	MIR5583-2	0	0
NR_13077	chr8	20953633	20968938	+	714	1	LOC10246	0	0
NM_0011	chrX	24149724	24216255	+	7386	1	ZFX|ZNF92	1.276	1.658
NM_0011	chr19	51410021	51417803	-	2923	1	SIGLEC10|	0.15	0.089
NM_0012{	chr10	1.26E+08	1.26E+08	-	8003	1	ADAM12|/	0.263	0.214
NM_0011(	chr1	18871430	18890891	-	3226	1	ALDH4A1|	9.496	12.164
NR_13848	chr10	6737382	6738939	+	828	1	LINP1|-|-|	0	0
NM_0149	chr10	27004116	27100498	-	6743	1	ANKRD26|	0.772	1.109
NM_0034(	chr7	1.43E+08	1.43E+08	+	2294	1	ZYX|ESP-2	59.51	59.445
NM_0010	chr3	1.22E+08	1.22E+08	-	713	1	CCDC58|-|	9.349	7.069

NM_1523!	chr19	11814292	11835201	+	4175	1	ZNF440|-|	1.754	2.644
NM_0052:	chr16	13920157	13952348	+	6748	1	ERCC4|ER(	1.915	2.048
NR_00301	chr5	77080434	77080571	-	137	1	SNORA47|	0.188	0.6
NR_12031	chr16	88099409	88100985	-	412	1	LOC40055.	0	0
NM_0230	chr1	52686342	52698366	-	2109	1	COA7|C1o	8.082	9.989
NM_0012	chr3	49015489	49018608	-	1735	1	DALRD3|-|	50.534	43.641
NM_0013	chr2	1.33E+08	1.33E+08	-	3496	1	LYPD1|LYF	38.987	19.527
NM_0221	chr5	1.12E+08	1.12E+08	-	4686	1	EPB41L4A	17.062	9.59
NR_00257	chr18	49491283	49491347	-	64	1	SNORD58/	0	2.14
NR_10675	chr11	60209071	60209156	-	85	1	MIR6503|I	0	0
NM_0012(	chr20	3820524	3825307	+	1803	1	AP5S1|C2(	5.32	5.189
NR_13462	chr6	34696154	34697471	+	880	1	LOC10192!	6.036	4.95
NM_0308(	chr1	1.84E+08	1.85E+08	+	10300	1	C1orf21|P	11.63	7.289
NM_0012	chr16	57694801	57731455	+	2697	1	DRC7|C16	0.526	0.361
NR_04986	chr3	1.21E+08	1.21E+08	+	75	1	MIR5682|-	0	0
NM_0013	chr2	15939898	15942249	-	671	1	MYCNOS|I	2.652	4.797
NM_1985(	chr3	52836756	52897581	-	4725	1	TMEM110	9.695	10.688
NM_0011!	chr8	12064389	12071747	-	237	2	LOC10013.	0	0
NM_0010(	chr2	2.41E+08	2.41E+08	+	4101	1	ANO7|D-T	0.163	0.037
NR_00219	chr1	1.6E+08	1.6E+08	+	1205	1	SUMO1P3	6.998	7.002
NM_0011!	chr10	95036772	95069497	-	1789	1	CYP2C8|CI	0	0.046
NM_0010	chr11	1.24E+08	1.24E+08	-	5658	1	SCN3B|AT	30.582	42.73
NM_0020!	chr16	11868128	11915968	-	7136	1	GSPT1|55:	15.372	17.897
NM_0053	chr19	16111680	16133635	+	2167	1	RAB8A|MI	33.855	35.453
NM_0054!	chr6	25754699	25781175	+	3586	1	SLC17A4|K	0	0.111
NM_0025	chr6	85449584	85495791	+	4057	1	NT5E|CAL	0.064	0.294
NR_00273	chr20	2656624	2656694	+	70	1	SNORD56|	0	0.196
NM_0010:	chr14	1.05E+08	1.05E+08	+	4629	1	INF2|C14c	2.607	8.362
NM_0207!	chr13	39655627	39752628	+	3588	1	COG6|CD(	4.1	4.058
NM_0013	chr12	1.08E+08	1.08E+08	+	2455	1	PWP1|IEF-	27.825	31.221
NR_13847	chr2	1.01E+08	1.01E+08	-	4073	1	TBC1D8|A	1.931	3.148
NR_10417	chr12	1.29E+08	1.29E+08	+	541	1	LOC28335.	0	0
NR_03334	chr19	3225485	3297075	+	1637	1	CELF5|BRU	53.409	48.437
NR_03655	chr1	2.03E+08	2.03E+08	-	1868	1	LOC10050(	0.007	0.051
NM_0033	chrX	1.55E+08	1.55E+08	+	1644	1	VBP1|HIBI	43.504	41.291
NM_0133:	chr1	11273198	11288434	+	3650	1	UBIAD1|S(	9.453	10.008
NM_0012	chr7	2403560	2434581	+	2101	1	CHST12|C	9.53	11.213
NR_04747	chr14	51546629	51599952	-	609	1	FRMD6-AS	0	0
NM_0180	chr19	4247070	4269093	+	1436	1	CCDC94|-|	23.515	19.391
NR_00256	chr11	62855565	62855631	-	66	1	SNORD25|	10.94	7.471
NM_0181	chr4	41935120	41960807	+	7708	1	TMEM33|	3.7	4
NM_0025!	chr1	1.11E+08	1.11E+08	-	2229	1	OVGP1|CH	1.649	2.636
NM_0056	chr2	2.23E+08	2.23E+08	-	2461	1	FARSB|FAI	22.539	29.581
NM_0217	chr11	61873523	61891545	-	1779	1	FADS3|CYI	7.4	9.67
NM_0151	chr2	73385758	73609919	+	12900	1	ALMS1|AL	2.458	3.017
NM_0124!	chr11	57528463	57530759	-	669	1	TIMM10|T	45.484	39.883
NR_02735	chr2	86784605	86791714	-	2491	1	CD8A|CD8	1.599	2.051
NM_0011	chr19	7629785	7631624	+	333	1	PET100|C:	34.344	23.733
NM_1756(	chr3	2098866	3057961	+	5134	1	CNTN4|AX	0	0.027
NR_11077	chr18	10661933	10666887	+	3563	1	LOC10192	0.011	0.012

NM_0166	chr15	41848146	41894077	-	11653	1	SPTBN5|B	0.096	0.076
NM_0072	chr1	1.85E+08	1.85E+08	+	3542	1	RNF2|BAP	10.764	9.637
NM_0011	chr2	17816519	17817100	+	580	1	MSGN1|M	0	0.024
NM_0251	chr10	69220303	69267559	+	3669	1	HKDC1|-|-	0.123	0.258
NR_03999	chr14	27612588	27639636	+	3530	1	LINC0064	2.422	2.642
NR_00350	chr16	23522013	23557375	-	3859	1	EARS2|CO	7.888	9.491
NR_02705	chr1	868071	876802	-	1703	1	FAM41C|-	0	0.064
NM_0011	chr3	33388336	33440405	-	3748	1	UBP1|LBP	13.423	17.823
NR_10975	chr14	40954711	41141048	+	557	1	LOC64491	2.824	1.18
NR_03978	chr6	2854031	2854107	-	76	1	MIR4645|	0	0
NM_0012	chr1	1.97E+08	1.97E+08	-	6104	1	ASPM|ASP	0.142	0.597
NM_1788	chr3	32238679	32370326	+	1169	1	CMTM8|C	0.309	0.574
NM_0013	chr19	35629016	35637686	+	1615	1	RBM42|-|	49.059	39.2
NR_04008	chr6	75285014	75291864	+	1294	1	LOC10050	0.319	0.423
NM_0121	chr19	45609400	45639417	-	2233	1	EML2|ELP	14.949	13.722
NM_0319	chr7	42932340	42937854	+	887	1	MRPL32|H	34.654	33.355
NM_0011	chr15	64841883	64868002	+	3564	1	PLEKHO2|	5.658	4.766
NR_03883	chr9	69819405	69820739	-	951	1	C9orf135-	0.163	0.144
NM_1446	chr12	1.22E+08	1.22E+08	+	3747	1	WDR66|C	0.939	0.746
NM_0011	chr1	2.24E+08	2.24E+08	+	3243	1	CAPN2|CA	26.622	20.256
NR_00145	chr1	44777842	44777912	+	70	1	SNORD38	9.21	7.24
NM_0013	chr14	22564907	22589237	-	692	1	DAD1|OST	156.641	140.533
NR_03960	chr7	29680734	29680828	-	94	1	MIR550A3	0	0
NM_0011	chr12	10372353	10410146	-	1932	1	KLRC4-KLR	0.02	0.014
NM_0017	chr12	7060661	7071031	+	2724	1	C1S|EDSP	0.947	1.026
NR_13364	chr2	2.17E+08	2.17E+08	+	1053	1	DIRC3-AS1	0.024	0.143
NR_03610	chr7	57405025	57405090	+	65	1	MIR3147|	0	0
NR_03988	chr17	59366083	59366154	+	71	1	MIR4729|	0	0
NR_13530	chr16	29453588	29454205	-	333	2	BOLA2|BO	2.168	3.455
NM_0010	chr11	298201	299526	-	731	1	IFITM5|BR	0.106	0
NR_13461	chr6	26569324	26574698	+	2882	1	LOC10537	0.004	0.024
NM_2075	chr11	75602860	75668434	-	4324	1	MAP6|MA	159.88	85.841
NM_0177	chr9	20658309	20995955	+	6048	1	FOCAD|KI	6.229	18.824
NM_0010	chr12	8172554	8180046	+	3431	1	ZNF705A|	0	0.008
NR_03405	chr3	25858532	25873695	-	922	1	LINC00692	0	0
NM_0017	chr12	92140278	92145897	-	4676	1	BTG1|APR	39.714	48.042
NM_0039	chr2	1.43E+08	1.43E+08	+	1656	1	KYNU|KYN	0.023	0
NM_0012	chr3	27715949	27722715	-	2636	1	EOMES|TE	4.28	68.62
NR_11009	chr12	93707791	93737823	-	1584	1	LOC10192	0	0.017
NM_0012	chr11	64852727	64879713	-	3328	1	EHD1|H-P	39.482	34.288
NM_0011	chr3	17157162	17700020	-	6493	1	TBC1D5|-|	9.923	9.531
NM_0010	chr20	31404845	31406266	-	409	1	DEFB121|	0	0
NM_0063	chr19	16517889	16542452	-	4076	1	CHERP|DA	26.698	28.352
NM_1734	chr19	15010727	15023271	+	1742	1	CCDC105|	0	0
NM_1785	chr11	369795	382117	+	3432	1	B4GALNT4	61.695	63.341
NR_00370	chr2	61417244	61417377	-	133	1	SNORA70	0	0
NR_03817	chr11	55293703	55298232	-	1002	1	TRIM51HP	0	0
NR_11027	chr2	1.19E+08	1.19E+08	-	1164	1	LOC10192	0	0
NM_0068	chr3	1.23E+08	1.23E+08	+	1843	1	PDIA5|PD	6.695	7.313
NR_10999	chr3	48847937	48851982	+	3386	1	PRKAR2A-	0.716	0.4

NM_0189	chr6	1.36E+08	1.36E+08	+	5372	1	PDE7B|bA	0.473	0.286
NM_0010	chr1	2.48E+08	2.48E+08	+	937	1	OR2L8|-|-	0	0
NM_0013	chr13	35768652	36131377	-	8370	1	DCLK1|CL	46.681	31.596
NM_0132	chr11	1.26E+08	1.26E+08	+	1820	1	DDX25|GF	6.681	17.031
NM_0011	chr15	65093004	65106359	-	1376	1	UBAP1L|U	1.705	2.031
NM_0012	chr10	1.07E+08	1.07E+08	-	7148	1	SORCS1|h	3.422	2.897
NM_0179	chr1	16562427	16613605	-	5916	1	NBPF1|AB	2.962	1.973
NM_0011	chr19	44891220	44903689	+	1698	1	TOMM40|	37.557	30.056
NR_03897	chr9	1.25E+08	1.25E+08	+	615	1	MIR181A2	3.124	2.762
NM_1534	chr11	69665564	69675397	-	2520	1	ORAOV1|T	6.871	6.164
NR_03033	chr10	1.01E+08	1.01E+08	+	99	1	MIR608|M	0	0
NM_0010	chr10	1.16E+08	1.16E+08	+	2294	1	PNLIPRP3|	0	0
NR_13462	chr6	1.64E+08	1.64E+08	-	454	1	LOC10272	0	0
NM_0011	chrX	70455096	70505489	+	4519	1	DLG3|MR)	25.676	23.048
NM_0058	chr11	58710088	58731974	-	1138	1	GLYAT|AC	0	0
NR_02445	chr12	1.3E+08	1.3E+08	-	3208	1	LOC10019	0.024	0
NM_0189	chr17	48595737	48604972	-	1670	1	HOXB6|HO	0	0
NM_0314	chr17	1646151	1650098	-	1781	2	RILP|PP10	0	0
NR_03021	chr19	53736845	53736934	+	89	1	MIR516B1	0	0
NM_0806	chr20	37903097	37945345	+	1955	1	VSTM2L|C	52.834	38.723
NR_13618	chr3	1.5E+08	1.5E+08	+	736	1	LOC10537	0.07	0
NM_0327	chr10	98383565	98415221	-	2047	1	PYROXD2|	0.498	0.79
NM_0177	chr20	63195430	63216186	-	3245	1	YTHDF1|C	30.63	34.08
NM_0050	chr19	46019155	46023298	-	946	1	PGLYRP1|	0.027	0.145
NR_03895	chrUn_GL	97129	126696	+	2937.3	3	LOC10050	0.026	0.149
NM_0004	chr5	41730065	41870689	-	3539	1	OXCT1|OX	13.608	11.646
NM_0012	chr21	38229915	38301824	+	2783	1	KCNJ15|IR	0.009	0
NR_02983	chr1	1167863	1167952	+	89	1	MIR200A|	0	0
NM_0150	chr16	71644926	71724701	-	8294	1	PHLPP2|PI	2.668	3.43
NR_02952	chr10	89592747	89592827	-	80	1	MIR107|M	0	0
NM_0013	chr2	25042463	25159135	+	7421	1	EFR3B|KIA	24.508	25.735
NR_07313	chr2	1.2E+08	1.2E+08	+	2166	1	CFAP221|	0.435	0.221
NR_07343	chr11	8964675	8976283	+	1048	1	TMEM9B-	3.051	1.974
NM_1707	chr1	1.83E+08	1.83E+08	-	5435	1	NMNAT2|	62.543	46.532
NM_0011	chr3	1.26E+08	1.26E+08	-	1958	1	SLC41A3|S	38.562	38.573
NM_0010	chr11	59503576	59504511	+	934	1	OR4D11|C	0	0
NM_0012	chrX	11137543	11427785	-	3880	1	ARHGAP6|	0.263	0.106
NM_0161	chrX	1.5E+08	1.5E+08	-	1951	2	HSFX1|LW	0.04	0.077
NM_0010	chr2	1.39E+08	1.39E+08	+	5722	1	SPOPL|BT	1.115	1.913
NM_2073	chr7	22419444	22500282	-	1291	1	STEAP1B|-	0.03	0.106
NM_1524	chr19	37411158	37467437	-	4050	1	ZNF569|Z	1.417	2.077
NR_12038	chr22	31346759	31351155	+	4396	1	LINC01521	7.133	9.02
NM_0010	chr10	63521363	63625123	+	5195	1	REEP3|C1	2.164	1.803
NM_0012	chr14	69569814	69574883	-	4175	1	CCDC177|	6.726	7.513
NM_0010	chr15	22094431	22095864	+	1431	1	OR4N4|OF	0	0
NR_04624	chr5	51372737	51383332	-	6696	1	LOC64236	0.027	0.016
NM_0013	chrY	21511338	21525786	+	1797	4	RBMY1D|-	0	0
NM_0210	chrX	37685756	37732130	+	5204	1	XK|KX|NA	0.24	0.453
NR_02713	chrX	1.2E+08	1.2E+08	-	2417	1	NKAPP1|C	0.315	0.289
NM_0180	chr17	35131322	35142315	-	2598	1	NLE1|Nle|	10.387	12.3

NM_0002[illegible]	chr19	17824784	17848032	-	5408	1	JAK3|JAK-[illegible]	0.668	0.636
NM_0210[illegible]	chr3	1.92E+08	1.92E+08	-	6167	1	FGF12|EIE	2.85	1.506
NM_1456[illegible]	chr16	28499362	28506834	-	1037	1	IL27|IL-27	0	0
NR_03759	chr6	1.63E+08	1.63E+08	-	885	1	CAHM|LIN	2.214	3.761
NM_1446[illegible]	chr10	1.17E+08	1.17E+08	-	987	1	C10orf82|	0.026	0.083
NM_0140[illegible]	chrX	49235467	49250526	+	2300	1	CCDC22|C	9.541	8.296
NM_0001[illegible]	chr3	1.29E+08	1.29E+08	+	889	1	GP9|CD42	0	0
NM_0008[illegible]	chr17	70169535	70180042	+	5382	1	KCNJ2|ATI	0.335	0.387
NM_0025[illegible]	chr20	57561081	57566457	+	2673	1	PCK1|PEP[illegible]	0.043	0.031
NR_13530	chr3	50116022	50156085	-	2751	1	SEMA3F-A	9.702	8.828
NM_0153[illegible]	chr22	38656653	38673851	+	1125	1	CBY1|C22[illegible]	48.858	41.955
NR_12042	chr13	25172824	25180001	-	1069	1	LINC00463	0.241	0.692
NM_1984[illegible]	chr6	97142161	97283176	-	8616	1	MMS22L|[illegible]	0.473	0.574
NR_13505	chr1	16888538	16889478	-	506	1	LOC10537[illegible]	0.51	0.081
NM_0049[illegible]	chr9	1.24E+08	1.25E+08	-	3086	1	NR5A1|AD	0.142	0.191
NM_0529[illegible]	chr6	29486697	29487956	-	1257	8	MAS1L|M[illegible]	0	0
NR_13532	chr13	1.12E+08	1.12E+08	+	6852	2	LINC01043	0	0
NM_0012[illegible]	chr19	58354357	58362848	-	3461	1	ZNF497|-|	6.75	6.063
NM_1458[illegible]	chr10	1.33E+08	1.33E+08	+	1102	1	ZNF511|-|	16.813	13.635
NM_0244[illegible]	chr17	58301227	58328791	-	7485	1	TSPOAP1|	8.406	7.66
NR_04001	chr19	37817411	37826638	+	1053	1	LOC64455[illegible]	4.457	1.587
NR_03980	chr8	91205485	91205558	+	73	1	MIR4661|[illegible]	0	0
NR_04981	chr16	85306226	85306325	-	99	1	MIR5093|[illegible]	0.651	0
NR_12204	chr22	47631674	47855600	+	1806	1	LOC28493[illegible]	0	0
NM_0012[illegible]	chr19	41673307	41688277	-	1964.5	2	CEACAM7	0	0
NM_0582[illegible]	chr19	15237490	15280451	-	5176	1	BRD4|CAP	20.165	21.847
NM_0012[illegible]	chr11	71878453	71884561	+	210	1	LOC10012[illegible]	0	0.261
NR_00243	chr6	1.33E+08	1.33E+08	+	75	1	SNORD10[illegible]	5.329	4.566
NM_0013[illegible]	chr5	34998101	35048135	-	1895	1	AGXT2|AG	0	0.007
NM_1393[illegible]	chr1	2.23E+08	2.23E+08	-	1783	1	TAF1A|M[illegible]	3.326	3.203
NR_04601	chr15	86296834	86316968	-	900	1	AGBL1-AS[illegible]	0	0
NR_04658	chr1	2.4E+08	2.4E+08	-	481	1	CHRM3-AS	0	0
NM_0012[illegible]	chr12	4604863	4613888	+	958	1	DYRK4|-|-	14.28	21.632
NM_1768[illegible]	chr20	33811304	33854367	+	1640	1	CHMP4B|[illegible]	81.567	80.762
NM_0157[illegible]	chr19	47608196	47703277	+	5722	1	GLTSCR1|-	7.911	8.211
NR_10417	chr11	72014291	72020910	+	4229	1	LOC10012[illegible]	48.513	46.396
NR_12121	chr12	1.05E+08	1.05E+08	-	1102	1	LOC41430[illegible]	0.69	0.485
NM_0012[illegible]	chr1	59515629	59762731	+	1159	1	FGGY|-|-|	3.616	5.283
NR_03979	chr7	2527074	2527145	+	71	1	MIR4648|[illegible]	2.179	0
NM_0248[illegible]	chr17	36544888	36589846	+	2829	2	GGNBP2|[illegible]	0	0
NM_1525[illegible]	chr15	41182731	41230757	-	2984	1	EXD1|EXD	0.035	0.101
NR_02407	chr2	1.14E+08	1.14E+08	+	1947	1	WASH2P|[illegible]	1.199	0.978
NM_0216[illegible]	chr6	99606774	99615578	+	3198	1	PRDM13|[illegible]	9.027	19.012
NM_0164[illegible]	chr19	49976467	50025548	-	2083	1	VRK3|-|-|[illegible]	24.165	23.666
NR_03324	chr5	1.42E+08	1.42E+08	-	1069	1	LOC72908[illegible]	0	0.051
NM_0013[illegible]	chr2	1.36E+08	1.36E+08	-	3139	1	DARS|HBS	16.122	15.054
NM_0010[illegible]	chr9	19408927	19452502	+	2844	1	ACER2|AL[illegible]	1.841	1.387
NM_0012[illegible]	chr15	65578758	65611289	-	2164	1	INTS14|C1	17.827	20.121
NM_2125[illegible]	chr2	2.36E+08	2.36E+08	-	1394	1	ASB18|AS[illegible]	0	0
NR_02433	chr19	10651862	10653872	-	2010	1	ILF3-AS1|-	18.269	22.822

NM_0042(	chr8	1.45E+08	1.45E+08	-	3793	1	RECQL4|R	6.102	3.748
NM_0048:	chr1	31259568	31296797	-	1612	1	SNRNP40|	37.137	35.007
NR_11709	chr1	2.03E+08	2.03E+08	+	767	1	LINC01353	0.252	0.125
NR_03411	chr19	1822089	1824543	+	1695	1	LOC10028	0.806	1.204
NM_0047(	chr7	43583093	43627379	+	3924	1	STK17A|DI	1.143	7.414
NM_0068:	chr6	89080710	89085160	+	2129	1	PNRC1|B4	14.08	14.501
NR_13631	chr2	78545531	78566084	+	447	1	LOC10537	0	0
NM_0013:	chr17	32991864	32997877	+	507	1	SPACA3|A	0	0
NR_12658	chr10	46109495	46131678	+	2400	1	AGAP7P|A	0.021	0.006
NR_11062	chr1	60659631	60825584	-	2338	1	LOC10192	0.656	4.37
NM_0121:	chr4	1.39E+08	1.39E+08	+	1979	1	NOCT|CCR	3.362	4.921
NR_03978	chr5	14825929	14826012	-	83	1	MIR4637|-	0	0
NM_0147!	chr5	1.48E+08	1.48E+08	-	9176	1	JAKMIP2|J	6.059	6.672
NM_0189!	chrX	1.3E+08	1.3E+08	+	2683	1	SASH3|75:	0.062	0
NM_0011	chrX	47223018	47229995	+	3000	1	CDK16|PC	88.105	86.62
NM_0020(	chr1	1.59E+08	1.59E+08	+	1157	1	FCER1A|F(	0	0
NR_02424	chr6_GL0C	1266702	1270687	+	1546.1	8	HLA-J|CDA	0	0
NR_03891	chr7	1.01E+08	1.01E+08	-	2529	1	PCOLCE-AS	0.974	0.487
NR_10465	chr16	52085281	52099067	+	1006	1	LOC10246	0	0.014
NR_07319	chr17	50761034	50767515	-	821	1	LINC00483	0	0
NM_1987:	chr3	49716834	49719805	-	2969	1	AMIGO3|A	4.842	4.6
NM_0149	chr16	89873586	89911384	+	2239	1	TCF25|FKS	69.627	82.843
NM_0178(	chr4	1.7E+08	1.7E+08	-	1206	1	HPF1|C4or	14.551	16.741
NR_02678	chr6	87397124	87399741	+	1556	1	LINC0159C	0.373	0.634
NM_0210!	chr22	29883169	30030868	+	8970	1	MTMR3|F	16.675	22.682
NR_03659	chr9	37079896	37090401	+	4373	1	EBLN3P|EI	9.686	8.864
NR_02439	chr10	87238667	87342558	-	816	1	NUTM2A-A	6.02	4.532
NM_0013	chr19	57977053	58002827	-	3629	1	ZNF606|ZI	5.23	7.454
NR_00328	chr3	1.57E+08	1.57E+08	-	2750	1	PA2G4P4|	3.09	3.496
NR_12574	chr8	34783921	34864798	+	656	1	LINC01288	0	0
NM_0009	chr7	95359872	95396375	-	1180	1	PON3|-|-|	0.098	0
NM_0146:	chr1	1.54E+08	1.54E+08	-	678	1	S100A6|2A	0.323	6.606
NR_02990	chrX	76919273	76919360	-	87	1	MIR384|M	0	0
NM_0012!	chr3	1.83E+08	1.83E+08	-	2267	1	MCCC1|M	15.294	13.081
NR_03982	chr10	20551970	20552046	+	76	1	MIR4675|-	0	0
NM_0013:	chr17	1225891	1229680	-	3292	1	ABR|MDB	0	0.012
NM_1392	chr3	1.61E+08	1.61E+08	+	10932	1	PPM1L|PP	8.209	6.491
NR_13297	chr15	69396904	69415029	-	1721	1	LOC14569	0.27	0.215
NM_1383	chr1	11919588	11926428	-	2818	1	KIAA2013|	11.05	10.567
NR_10406	chr8	90592775	90606063	-	762	1	LINC0103C	0	0
NM_0067!	chr10	1.19E+08	1.19E+08	-	1618	1	PRDX3|AC	55.941	48.752
NM_1768:	chr7	1.43E+08	1.43E+08	+	922	1	TAS2R41|	0	0.015
NR_03826	chr1	41847189	42035925	-	1703	1	HIVEP3|KE	1.423	1.641
NM_0012:	chr1	1.62E+08	1.62E+08	+	1609	1	FCRLB|FCF	0.288	0.375
NM_1452(	chr2	19351485	19358611	-	1889	1	OSR1|ODD	0.102	0.442
NM_0239:	chr12_GL8	238	1333	-	1093	3	TAS2R7|T2	0	0
NR_02813	chr7	1.08E+08	1.08E+08	-	3886	1	SLC26A4-A	0.007	0.011
NR_03034	chr12	57519165	57519259	-	94	1	MIR616|M	0.274	0.146
NM_0012!	chr17	80260861	80398781	+	20987	1	RNF213|A	1.685	2.295
NM_0134!	chrX	7842262	7844143	+	962	1	VCX|VCX-1	0	0

NM_0011	chr16	74447428	74607144	-	9301	1	GLG1|CFR	30.47	29.546
NR_10349	chr2	11444375	11466177	-	2970	1	E2F6|E2F-	6.221	6.35
NM_0251	chr11	65860401	65866443	+	2382	1	MUS81|SL	29.993	25.715
NM_0013	chr5	1.5E+08	1.51E+08	+	7930	1	NDST1|HS	23.805	32.881
NR_03717	chr6_GL0C	3591140	3592704	-	545	8	LOC10050	0	0.05
NR_02427	chr9	21967139	21967754	+	615	1	CDKN2A-A	0	0
NM_0013	chr17	67070438	67245204	-	13799	1	HELZ|DHR	2.648	4.098
NM_0011	chr3	50246894	50259354	+	2132	1	GNAI2|GIF	241.698	209.021
NR_10696	chr16	2106678	2106748	-	70	3	MIR6511B	0	0
NR_10355	chr17	15104985	15106187	-	717	1	CDRT8|-|-	0	0
NR_12033	chr15	58434901	58498735	-	2217	1	LIPC-AS1|-	0	0
NR_03793	chr19	44942238	44949565	+	1823	1	APOC4-AP	0.014	0.008
NR_03610	chr9	95086014	95086094	-	80	1	MIR3074|	0	0.171
NM_0058	chr17	42761194	42763041	+	779	1	RAMP2|-|	12.529	13.732
NM_0012	chr11	71814045	71821547	-	3434	1	ZNF705E|-	0.023	0.044
NR_13277	chr22	38224479	38224720	-	241	1	SNORA92|	3.798	4.433
NR_10384	chr1	1.46E+08	1.46E+08	+	1157	1	CD160|BY	0.635	0.912
NM_0010	chr15	82809628	82834861	+	3842	1	WHAMM|	1.785	2.56
NM_0319	chr4	1.47E+08	1.47E+08	-	2164	1	TTC29|NYI	0.161	0.19
NM_0327	chr9	1.31E+08	1.31E+08	+	2091	1	PLPP7|C9c	6.209	4.094
NR_10996	chr22	40521800	40526707	+	1928	1	LOC10192	0	0.007
NR_03835	chr14	51333393	51365557	+	669	1	LINC0064C	0.116	0.102
NM_0175	chr11	64824190	64844569	-	4901	1	CDC42BPG	0.182	0.243
NM_0149	chr14	55366391	55411858	-	4731	1	ATG14|AT	3.889	4.456
NR_13492	chrX	1.35E+08	1.35E+08	+	957	1	LOC34058	0	0
NR_03766	chr1	1280436	1292029	+	2943	1	SCNN1D|E	2.283	2.527
NR_13077	chr5	68967741	69029927	-	2423	1	LOC10192	0.032	0.017
NR_10681	chr12	1.11E+08	1.11E+08	+	67	1	MIR6760|	4.041	4.498
NM_0028	chr14	24143365	24146646	-	816	1	PSME2|PA	37.906	31.07
NM_0012	chr19	49340596	49362457	-	1932	1	TEAD2|ETI	42.431	25.615
NM_0042	chr18	70288901	70330198	+	5842	1	SOCS6|CIS	2.189	2.645
NM_0004	chr2	1.56E+08	1.57E+08	+	5784	1	GPD2|GDH	0.568	0.467
NR_12372	chr1	1.6E+08	1.6E+08	-	1573	1	LOC72986	3.557	3.413
NM_0010	chr16	3065784	3069667	+	873	1	IL32|IL-32	0.044	0.016
NM_1944	chr4	39459024	39477651	+	1625	1	LIAS|HGCL	4.991	5.209
NM_0011	chr4	1.01E+08	1.01E+08	-	4506	1	PPP3CA|C	7.978	8.818
NR_10677	chr10	1.12E+08	1.12E+08	+	78	1	MIR6715A	0	0
NM_1989	chr13	95578202	95644706	-	7466	1	DZIP1|DZI	3.763	4.81
NM_0121	chr9	1.21E+08	1.21E+08	-	9176	1	FBXW2|FB	6.625	9.092
NR_02684	chr21	32393133	32393957	+	824	1	URB1-AS1	1.846	5.785
NR_04011	chr17	41402416	41412588	+	2157	2	LOC10050	0	0
NM_0011	chr7	66114818	66154566	+	2639	1	CRCP|CGR	21.556	26.854
NM_0202	chr3	45755449	45796543	-	5419	1	SLC6A20|S	0.007	0.008
NM_1788	chr4	1.03E+08	1.03E+08	-	3111	1	SLC9B2|NI	5.516	6.978
NR_02774	chr2	74483073	74494564	+	2808	1	TTC31|-|-	7.521	7.366
NM_0013	chr19	17405686	17425339	+	1372	1	MVB12A|(	20.261	19.397
NM_0013	chr21	14108814	14210891	-	1578	1	LIPI|CT17|	0	0.009
NR_02425	chr3	1.26E+08	1.26E+08	+	1938	1	FAM86JP|	0.985	0.544
NR_03845	chr19	47484282	47501597	+	652	1	NAPA-AS1	16.769	17.268
NM_0158	chr9	1.35E+08	1.35E+08	+	934	1	FCN2|EBP-	0	0

NR_10703	chr14	1.06E+08	1.06E+08	+	64	4	MIR8071-1	0	0
NR_13527	chr3	1.33E+08	1.33E+08	-	1537	1	BFSP2-AS1	0	0.018
NR_03827	chr7	43508728	43522542	-	2913	1	LOC10050	0.668	2.201
NM_0010	chr15	30626676	30638810	+	1007.5	4	ARHGAP11	0	0
NM_0023	chr5	1.1E+08	1.1E+08	+	7090	1	MAN2A1|/	1.033	1.839
NR_12615	chr8	1.02E+08	1.02E+08	+	2459	1	LOC10405	0.01	0.011
NM_0240	chr2	2.34E+08	2.34E+08	+	5593	1	TRPM8|LT	0.067	0.135
NR_03162	chr13	54311972	54312048	-	76	1	MIR1297|	0	0
NM_0011	chr2	68042200	68063027	-	1263	1	C1D|LRP1	4.839	3.611
NM_0030	chr8	48917681	48921440	-	2103	1	SNAI2|SLU	0.411	0.638
NR_03160	chr8	1.44E+08	1.44E+08	-	79	1	MIR1234|	0	0
NR_02715	chr10	649948	652655	+	1925	1	PRR26|C1	0	0.021
NR_00300	chr9	19063656	19063786	-	130	1	SCARNA8|	0.694	0.105
NM_0004	chr1	1.53E+08	1.53E+08	+	1225	1	LOR|-|-|1	0.021	0.145
NM_0012	chr17	9645537	9729686	+	4132	1	USP43|-|-	0.674	0.825
NR_02984	chr17	59151136	59151221	-	85	1	MIR301A|	0	0
NR_10704	chr18	112256	112339	-	83	1	MIR8078|	0	0
NR_00333	chr15	25097662	25097755	+	93	1	SNORD116	0	0
NM_0160	chr1	93149742	93180689	-	6059	1	TMED5|CC	1.919	1.847
NR_03991	chr22	19963753	19963834	+	81	1	MIR4761|	0	0
NM_0010	chr14	75069577	75079987	+	3466	1	ZC2HC1C|	0.744	0.692
NM_1527	chr11	1.25E+08	1.25E+08	-	3536	1	HEPACAM	0.062	0.682
NR_13564	chr17	60079309	60088692	+	694	1	LOC10537	0.242	0.217
NM_0013	chr19	48993448	49015995	+	1971	1	RUVBL2|C	68.994	61.397
NM_0160	chr1	29192657	29230958	-	2527	1	MECR|CGI	8.914	8.808
NM_0010	chr2	1.31E+08	1.31E+08	+	3423	1	POTEE|A2	0.015	0.036
NM_1445	chr2	30722771	30807445	-	2657	1	CAPN13|-	0.005	0.021
NR_11028	chr2	78088730	78127806	+	1097	1	LOC10192	0	0
NM_0328	chr1	2.23E+08	2.23E+08	+	4912	1	DISP1|DIS	2.549	1.754
NM_0312	chr17	58556677	58692055	-	4774	1	TEX14|CT1	0.835	0.875
NR_10685	chr19	49009906	49009972	+	66	1	MIR6798|	26.764	21.168
NR_12600	chr1	1.93E+08	1.93E+08	-	432	1	LINC01032	0.03	0.063
NM_0120	chr2	2.04E+08	2.04E+08	+	2636	1	ICOS|AILIN	0	0.005
NM_0046	chrX	38561478	38688918	+	1796	1	TSPAN7|A	110.312	164.051
NR_13809	chr13	18811143	18871967	-	3296	2	ANKRD20A	0	0
NM_0038	chr2	1.02E+08	1.02E+08	+	2657	1	IL18RAP|A	0	0
NM_1780	chr10	66926034	67101551	+	6046	1	LRRTM3|-	2.301	1.527
NR_02711	chr7	41693948	41713194	+	1855	1	INHBA-AS1	1.557	1.765
NM_0012	chr6	57046532	57055239	+	2936	1	KIAA1586|	2.099	2.874
NM_0013	chr2	2.08E+08	2.08E+08	-	1101	1	METTL21A	8.197	6.457
NR_03991	chr19	58386770	58386858	-	88	1	MIR4754|	0	0
NM_0010	chr3	1.59E+08	1.59E+08	+	1529	1	IQCJ|-|-|3	0.245	0.09
NR_03622	chr20	63286808	63286866	+	58	1	MIR4326|	0	0
NM_0335	chr1	42162691	42164724	-	570	1	GUCA2A|G	0	0
NR_13190	chr20	31721507	31723409	+	1902	1	ABALON|II	40.606	38.815
NR_12637	chr5	1.62E+08	1.62E+08	-	694	1	LINC01202	0	0.217
NM_1532	chr20	3667965	3682690	-	4025	1	ADAM33|	0.019	0.061
NR_03625	chrX	1.51E+08	1.51E+08	+	104	1	MIR4330|	0	0
NR_12592	chr4	1.06E+08	1.06E+08	-	346	1	LOC10192	0	0
NM_1445	chr2	10721649	10785110	+	3087	1	ATP6V1C2	21.101	21.812

NM_0013(	chr3	47380982	47413441	+	5128	1	PTPN23|H	18.671	20.671
NM_0012{	chr18	11609558	11610612	+	1053	1	SLC35G4|/	0	0
NR_12058	chr11	41714568	41836442	+	547	1	LINC0149<	0	0
NR_02770	chr13	1.11E+08	1.11E+08	-	6321	1	LINC0034€	0.045	0.059
NM_0011‹	chr11	20387530	20509333	+	2531	1	PRMT3|HI	3.123	3.187
NM_0011‹	chr15	43407214	43493156	-	6186	1	TP53BP1|!	51.37	43.631
NM_0011!	chr10	22208814	22209983	-	1168	1	EBLN1|EBI	0	0.023
NR_10808	chr3	1.41E+08	1.41E+08	-	2684	1	CLSTN2-AS	0.029	0.015
NR_12539	chr3	1.27E+08	1.28E+08	-	1223	1	LINC01471	0.074	0
NM_0204;	chr1	1.7E+08	1.7E+08	-	2911	1	SCYL3|PA(	4.571	4.461
NM_0005!	chr20	35433349	35438190	-	2324	1	GDF5|BDA	0.239	0.395
NM_1736!	chrX	26138343	26140736	+	1807	1	MAGEB18	0	0
NR_03989	chr1	2.24E+08	2.24E+08	-	84	1	MIR4742|i	1.535	1.141
NM_0010:	chr5	69415112	69442063	+	2151	2	MARVELD:	0	0
NR_02441	chr22	18029385	18037968	+	2377	1	LINC01634	0	0
NM_0055!	chr14	1.04E+08	1.04E+08	+	2586	1	KLC1|KLC|	340.027	303.4
NM_1452!	chr16	2830172	2832284	+	822	1	ZG16B|EE(	0	0.067
NM_0201:	chr6	1.61E+08	1.61E+08	-	7868	1	AGPAT4|1	20.204	26.221
NM_0331;	chr1	1.78E+08	1.78E+08	-	3996	1	SEC16B|LZ	0.245	0.675
NM_0054‹	chr12	85804553	85836540	-	1792	1	RASSF9|P-	0.029	0.795
NM_0163:	chr12	6723984	6731875	+	1866	1	COPS7A|C	25.435	26.249
NM_0010:	chr19	11394056	11419342	-	2522	1	RGL3|-|-|:	0.45	0.049
NM_1384;	chr9	1.14E+08	1.14E+08	-	1985	1	KIF12|-|-|	0.071	0.331
NM_0010:	chr1	26993704	27000886	+	1945	1	TRNP1|C1	15.074	13.817
NM_0011;	chrX	93674013	93712274	+	3287	1	FAM133A|	0.447	0.417
NM_0309‹	chr6	1.5E+08	1.5E+08	+	2205	1	PPP1R14C	52.609	22.462
NR_13657	chr22_KI2	90959	98408	-	1537	1	LOC10537!	0	0
NR_02668	chr5	1.73E+08	1.73E+08	-	1589	1	LOC10026:	1.071	1.103
NR_02406	chr12	12355408	12357067	-	1536	1	LOH12CR2	3.467	5.11
NM_0245‹	chr13	78614286	78659179	-	3526	1	RNF219|C	6.542	9.272
NM_0013;	chr2	99169263	99181061	-	847	1	MITD1|-|-	6.089	7.083
NM_0122!	chr7	1.16E+08	1.16E+08	-	6690	1	TFEC|TCFE	0.015	0.025
NM_0010(	chr14	20223710	20224702	+	991	1	OR11H6|-	0	0
NM_0011:	chr3	63278238	63616921	+	2586	1	SYNPR|SP(	2.453	16.96
NM_0306;	chr10	68560360	68694482	+	9570	1	TET1|CXX(	2.014	1.466
NM_0065;	chr7	93885397	93890991	-	2437	1	TFPI2|PP5	46.564	28.17
NR_03884	chr5	27472292	27496401	+	1101	1	LINC01021	0.691	0.535
NM_1451:	chr14	65075124	65102544	-	2007	1	MAX|bHLI	25.851	32.506
NM_1524!	chr1	37795488	37801606	+	2395	1	MANEAL|-	38.799	46.501
NM_0330:	chr3	51957454	51962892	-	1951	1	PCBP4|CB	250.494	151.959
NM_1840‹	chr16	30065673	30070420	+	1568	1	ALDOA|AL	492.222	201.917
NM_0143!	chr7	1.28E+08	1.28E+08	+	3643	1	SND1|TDR	78.543	77.106
NM_0013:	chr20	23364363	23373061	+	4724	1	GZF1|ZBTI	4.654	5.46
NM_0010;	chr17	44205033	44220882	-	4581	1	UBTF|NOF	52.815	52.474
NM_0012!	chr2	1.75E+08	1.75E+08	-	4194	1	ATF2|CRE-	17.643	17.022
NR_03718	chrX	1.42E+08	1.42E+08	+	1694	1	SPANXA2-(	0.03	0.081
NM_1530:	chr8	1.09E+08	1.09E+08	-	6298	1	TMEM74|	1.472	2.784
NM_0224(	chr1	2.4E+08	2.41E+08	-	4179	1	GREM2|CI	15.852	2.838
NM_0124;	chr1	1.61E+08	1.61E+08	+	2198	1	USP21|US	20.912	22.957
NR_02727	chr21	41711520	41715775	-	2027	1	LINC0047<	0	0

NM_0011:	chr7	1.51E+08	1.51E+08	-	1330	1	TMUB1|C7	27.639	26.066
NM_0045	chr14	92116124	92121809	-	414	1	NDUFB1|C	89.102	69.908
NR_03338	chr5	12574857	12805183	+	620	1	LINC01194	0	0
NR_04624	chr3	24494087	24500011	+	883	1	THRB-AS1|	0	0
NM_0011:	chr2	1.1E+08	1.1E+08	-	2561	1	NPHP1|JBT	1.677	0.802
NM_0011	chr10	1.13E+08	1.13E+08	+	3937	1	TCF7L2|TC	18.887	12.799
NM_1737	chr10	1.17E+08	1.17E+08	-	6477	1	PDZD8|PD	2.99	4.348
NM_0011	chr12	53423855	53431672	+	1705	1	AMHR2|A	0.227	0.072
NM_0140	chr2	98608548	98618514	+	1165	1	UNC50|GN	18.593	16.789
NR_02985	chr16	14309285	14309371	+	86	1	MIR365A|	0	0
NM_0330	chr9	99216425	99221964	-	2826	1	ALG2|CDG	5.876	7.483
NM_0325	chr5	40841308	40855354	+	4092	1	CARD6|CI	0.038	0.057
NR_02977	chr12	6963699	6963766	+	67	1	MIR200C|	0	0
NM_0010	chr1	2.13E+08	2.13E+08	-	499	1	SPATA45|	0.181	0.082
NM_0202	chr1	1.98E+08	1.98E+08	+	2508	1	LHX9|-|-|	91.267	2.479
NR_00307	chr1	28578743	28578822	-	79	1	SNORD99|	2.938	1.56
NM_0041	chr15	78438176	78501456	+	6343	1	IREB2|ACC	8.582	11.494
NM_0161	chr7	1.51E+08	1.51E+08	+	3113	1	NUB1|BS4	15.772	15.681
NM_0166	chr5	44808925	44815516	+	1665	1	MRPS30|N	7.806	9.773
NM_0013	chr19	45668244	45683724	+	3166	1	GIPR|PGQ	3.567	2.894
NM_0056	chr7	74453790	74602593	+	3359	1	GTF2IRD1|	25.994	30.134
NM_0010	chr1	37715974	37765152	-	5492	1	EPHA10|-|	1.855	2.571
NR_03388	chr1	2.44E+08	2.44E+08	+	1788	1	LOC33952	0.144	0.077
NM_0013	chr3	1.56E+08	1.57E+08	+	3980	1	KCNAB1|A	0.664	1.194
NM_0001	chr1	97077744	97921059	-	4422	1	DPYD|DHF	0.738	1.632
NM_0012	chr2	1.13E+08	1.13E+08	+	5043	1	POLR1B|R	4.385	5.617
NR_10387	chr1	1.47E+08	1.47E+08	-	5168	1	PRKAB2|-|	10.099	11.047
NM_0011	chr2	2.19E+08	2.19E+08	+	1425	1	SPEG|APE	17.517	22.194
NM_1749	chr4	97558874	98143240	-	1641	1	STPG2|C4	0.024	0
NR_12600	chr1	1.86E+08	1.86E+08	+	537	1	LOC10272	0.216	0.536
NM_1779	chr2	27381199	27409683	-	2290	1	PPM1G|PF	112.59	114.744
NM_0013	chr19	35887952	35899737	-	1958	1	NFKBID|Ik	2.351	1.756
NM_0011	chr6_GL00	3164420	3180268	-	2485	7	SLC44A4|	0	0
NM_0528	chr20	41359966	41366858	-	3821	1	EMILIN3|C	3.108	2.269
NM_0020	chr3	59747278	61251461	-	3112	1	FHIT|AP3A	1.363	1.505
NR_11082	chr17	72072333	72093481	+	2057	1	LINC02097	0.125	0.413
NM_0014	chr6	73515750	73521032	-	3502	1	EEF1A1|C	142.808	159.217
NM_0201	chr9	27524314	27526498	+	1137	1	IFNK|IFNT	0	0
NR_03787	chr4	22327366	22339666	-	882	1	LOC10050	0.175	0.14
NM_0045	chr5	1801382	1816051	+	540	1	NDUFS6|C	140.539	105.873
NM_1781	chr9	1.36E+08	1.36E+08	-	2378	1	LHX3|CPH	0.027	0.023
NR_03016	chr14	1.01E+08	1.01E+08	+	79	1	MIR487A|	0	0
NM_0012	chr3	1.29E+08	1.29E+08	-	4187	1	RAB43|RA	0.508	0.54
NM_0142	chr5	1.8E+08	1.8E+08	-	2446	1	MGAT4B|	57.541	57.252
NM_0045	chr17	82519718	82604607	+	5251	1	FOXK2|ILF	36.539	40.288
NM_0010	chr19	9114828	9115763	-	934	1	OR7G1|OF	0	0
NM_0003	chr10	1.26E+08	1.26E+08	-	1342	1	UROS|URC	44.253	53.022
NM_0161	chr1	19251581	19260128	+	2263	1	MRTO4|C1	16.318	17.885
NM_0012	chr15	23127066	23334210	-	3733.2	6	GOLGA6L2	0	0
NM_1446	chr16	69343246	69351809	-	934	1	TMED6|PF	0.456	0.513

NR_02792	chr17	35983656	36002038	-	1412.5	2	CCL15-CCL	0	0
NM_0012	chr18	50560114	50731826	+	3313	1	MAPK4|EF	9.659	18.596
NR_12634	chr8	1971397	1976444	+	778	2	KBTBD11-	0	0
NM_0044	chr10	1.1E+08	1.11E+08	+	2522	1	DUSP5|DU	3.251	7.283
NM_0010	chr11	1.24E+08	1.24E+08	+	955	1	OR4D5|OF	0.014	0
NR_00001	chr17	28722582	28722653	+	71	1	SNORD4A|	2.179	1.736
NM_0012	chr6	26634383	26659752	-	4723	1	ZNF322|H	0.587	0.635
NM_0179	chr14	1.05E+08	1.05E+08	-	2449	1	CDCA4|HE	7.76	5.738
NM_0012	chr14	96502865	96567116	+	4362	1	PAPOLA|P	24.43	25.592
NM_0010	chr9	35749280	35753267	+	1706	1	RGP1|KIA	18.131	14.821
NM_0209	chr10	1.12E+08	1.12E+08	-	6359	1	GPAM|GP	3.013	4.34
NM_1980	chr7	6798935	6826295	-	1819	1	CCZ1B|C7	2.169	3.102
NR_03887	chr8	23068229	23083619	+	2633	1	LOC28605	0.083	0.239
NM_0012	chr12	3808861	3873448	-	4368	1	PARP11|A	4.009	3.59
NM_0146	chr6	89642775	89819794	-	18104	1	MDN1|Re	3.51	4.566
NM_0013	chr6	1.53E+08	1.53E+08	-	3685	1	MTRF1L|H	1.865	1.275
NM_0062	chr3	38007496	38029663	-	2655	1	PLCD1|ND	12.971	12.696
NM_0012	chr13	1.01E+08	1.02E+08	+	4870	1	ITGBL1|OS	0.355	0.343
NM_0012	chr13	1.13E+08	1.13E+08	+	3689	1	CUL4A|-|-	10.999	11.157
NM_0012	chr6	1.24E+08	1.24E+08	-	1405	1	TRDN|CPV	0	0.029
NM_0012	chr8	1.25E+08	1.25E+08	-	4951	1	MTSS1|MI	29.857	34.465
NM_0314	chr22	50200979	50217616	+	2304	1	SELENOO|	9.144	10.618
NM_0010	chr1	58654739	58700075	-	7746	1	MYSM1|2	1.628	3.137
NM_0190	chrX	54530211	54567287	+	8555	1	GNL3L|GN	2.862	2.912
NM_0182	chr2	2.03E+08	2.03E+08	-	2267	1	WDR12|Y	9.589	10.64
NM_0000	chr1	75724347	75763678	+	2608	1	ACADM|A	10.392	9.511
NM_0328	chr3	43079229	43106083	-	2552	1	POMGNT2	20.073	30.464
NR_03999	chrX	1.55E+08	1.55E+08	-	1698	2	TMLHE-AS	0	0
NM_0184	chr17	61456488	61485110	+	3225	1	TBX4|ICPP	0	0
NM_0189	chr5	1.41E+08	1.42E+08	+	4599	1	PCDHGA9|	20.449	81.059
NR_07309	chr6	1.58E+08	1.58E+08	-	1922	1	SERAC1|-|	6.628	6.656
NM_0046	chr3	13818585	13880121	-	1708	1	WNT7A|-|	3.087	9.888
NM_0049	chr1	11106531	11262551	-	8665	1	MTOR|FR	21.497	17.103
NR_13623	chr2	1.27E+08	1.27E+08	+	729	1	LOC10192	0	0
NM_0144	chr1	27934955	27959157	+	1906	1	SMPDL3B|	0.406	0.41
NM_0011	chr20	57358440	57359822	-	1380	1	MTRNR2L	0.392	0.427
NR_03161	chr10	1.11E+08	1.11E+08	+	87	1	MIR548E|	0	0
NM_0029	chr14	20801356	20802877	-	815	1	RNASE1|R	0.016	0.05
NR_13432	chr7	144445	149438	-	561	3	LOC10272	0	0
NM_0010	chr8	80628451	80874781	-	14375	1	ZNF704|G	4.717	7.12
NR_10996	chr16	66469796	66476402	-	480	1	BEAN1-AS	0.457	0.599
NM_0012	chr6	1.39E+08	1.39E+08	-	3997	1	REPS1|RA	19.581	26.585
NM_0251	chr10	1.04E+08	1.04E+08	-	5340	1	CFAP43|C	0.391	0.236
NM_0208	chr6	1.51E+08	1.51E+08	-	3239	1	ZBTB2|ZN	3.786	4.165
NR_03396	chr7	379425	382879	+	2244	1	LOC44249	0	0
NM_2075	chr21	33229895	33262757	+	1385	1	IFNAR2|IF	4.72	5.775
NR_07346	chr6	20099686	20212464	-	4028	1	MBOAT1|	0.4	0.517
NM_0030	chr12	95858931	95866461	+	788	1	SNRPF|SM	54.634	46.549
NM_0050	chr2	1.12E+08	1.12E+08	+	7140	3	RGPD5|BS	0.061	0.038
NM_0018	chr4	1.7E+08	1.7E+08	+	6191	1	CLCN3|CL	16.249	21.321

NR_04006	chr15	73917468	73927786	-	2360	1	LOXL1-AS1	1.388	1.097
NM_0013:	chr17	78795652	78840527	-	5898	1	USP36|DU	6.147	7.034
NR_13485	chr6	87513935	87590032	-	2266	1	RARS2|Arg	30.402	27.569
NM_0017:	chr12	7080211	7092447	-	2485	1	C1R|EDSPI	1.453	5.236
NM_0013:	chr16	2048202	2088720	+	5392	1	TSC2|LAM	31.107	27.803
NM_0018	chr7	30651943	30682525	-	2984	1	CRHR2|CR	0.203	0.748
NM_1990	chr7	1.15E+08	1.15E+08	+	5305	1	MDFIC|HI(	0.491	0.457
NR_00220	chr15	40531884	40532550	-	666	1	MRPL42P5	1.065	1.111
NM_1384:	chr1	52032105	52033800	-	1693	1	KTI12|SBB	7.113	6.367
NM_0010(	chr9	1.05E+08	1.05E+08	+	961	1	OR13C8|C	0	0
NM_0224	chr16	53491040	53503304	-	2467	1	AKTIP|FT1	6.298	6.807
NM_0010(	chr3	98264285	98265262	+	976	1	OR5H6|OI	0	0
NM_0068	chr14	90396983	90408275	+	4248	1	CALM1|CA	182.764	186.148
NM_0011	chr6	1.67E+08	1.67E+08	+	2175	1	TCP10L2|I	0	0.006
NM_0068:	chr12	1.2E+08	1.2E+08	-	8608	1	GCN1|GCI	20.871	22.262
NM_0061(	chr17	39603768	39607922	-	3028	1	NEUROD2	8.12	67.63
NM_0012	chr15	41332694	41381050	+	2435	1	NUSAP1|A	10.114	11.402
NM_0010:	chr6	50009138	50021981	-	231	1	DEFB110|I	0	0
NR_13592	chr19	57599885	57612721	+	2737	1	ZNF530|-|	2.761	2.998
NM_0010	chr11	1.18E+08	1.18E+08	-	3433	1	TMPRSS13	0.131	0.275
NR_03994	chr2	1.14E+08	1.14E+08	-	78	1	MIR4782|-	0	0
NM_0051!	chr18	158483	213739	+	4138	1	USP14|TG	11.36	13.002
NM_0180:	chr13	1.07E+08	1.07E+08	-	1745	1	ARGLU1|-	38.208	41.052
NM_0012	chr9	1.22E+08	1.22E+08	+	4886	1	PTGS1|CO	0.14	0.356
NM_0245:	chr16	67528814	67546788	+	4085	1	FAM65A|-	29.988	27.344
NM_0012	chr16	397192	400754	+	899	1	NME4|ND	80.115	79.333
NR_12538	chr8	73310906	73356461	-	3255	1	RDH10-AS:	0.04	0.025
NM_0035(	chr6	27808199	27808667	+	466	1	HIST1H2AI	0.443	0.147
NM_0151	chr1	1.71E+08	1.72E+08	+	10348	1	PRRC2C|B.	14.103	13.704
NM_1981	chr11	7689924	7706394	-	1713	1	OVCH2|OV	0.015	0.024
NM_0021!	chr5	1.33E+08	1.33E+08	+	3364	1	HSPA4|AP	36.07	43.892
NM_0153!	chr7	98215443	98252251	-	5717	1	TECPR1|-|	18.261	18.824
NR_04659	chr3	1.93E+08	1.93E+08	+	578	1	FGF12-AS3	0.045	0
NM_0010:	chrX	1.54E+08	1.54E+08	-	3319	1	IRAK1|IRA	2.086	3.607
NM_0011!	chr6_GL0C	1639537	1657092	+	1555	7	TRIM39-RI	2.131	1.268
NR_10683	chr17	2692861	2692919	-	58	1	MIR6776|I	1.778	1.889
NM_0013:	chr3	1.96E+08	1.96E+08	-	2184	1	PCYT1A|C(	9.753	8.586
NM_0011!	chr14	49643552	49688380	-	1763	1	POLE2|DP	1.39	1.484
NM_0013:	chr20	25452697	25585531	-	3919	1	NINL|NLP|	12.387	11.719
NM_0065:	chr15	63504511	63594640	+	5589	1	USP3|SIH(	12.015	16.373
NR_03390	chr2	1.3E+08	1.3E+08	+	1829	1	MED15P9|	0.007	0.622
NM_0065:	chr16	24255553	24362416	+	2697	1	CACNG3|-	20.069	0.31
NR_13848	chr4	4290197	4321786	+	2816	1	ZBTB49|ZI	3.315	4.066
NM_1451(	chr3	42653684	42667580	+	5499	1	ZBTB47|ZI	4.239	6.332
NR_13279	chr11	1.21E+08	1.21E+08	-	6312	1	LOC10192!	0.008	0.011
NM_0010:	chrX	1.4E+08	1.4E+08	-	1274	1	CXorf66|S(	0	0
NM_0026!	chr8	42338455	42371813	+	1318	1	POLB|-|-|:	43.953	46.859
NM_0308	chr20	37693955	37872129	+	1958	1	CTNNBL1|	32.306	34.131
NM_0052	chr2	1.21E+08	1.21E+08	+	6754	1	GLI2|CJS|I	2.688	3.336
NM_1382	chr3	1.23E+08	1.23E+08	+	5760	1	DTX3L|BB.	0.027	0.183

NM_0211	chr19	12131353	12140407	-	3052	1	ZNF20|KO	4.296	4.533
NM_0013	chr11	1.26E+08	1.26E+08	+	2188	1	TIRAP|BA	2.746	2.116
NM_0248	chr12	21437604	21471248	+	4110	1	PYROXD1|	0.781	0.757
NR_12616	chr9	42198862	42206016	+	1891	1	FAM74A7|	0	0
NM_0163	chr14	50725829	50831121	-	4337	1	NIN|SCKL	4.629	4.987
NM_0203	chr12	51953380	51997079	+	4505	1	ACVR1B|A	40.598	47.303
NR_03743	chr1	1.66E+08	1.66E+08	+	55	1	MIR3658|	19.926	24.406
NM_0206	chr2	64092652	64144471	-	3758	1	PELI1|-|-|	13.902	16.74
NR_02452	chr19	21750745	21753438	+	2693	2	LOC64136	0	0
NR_02987	chr14	1.01E+08	1.01E+08	+	74	1	MIR381|M	0	0
NR_03341	chr7	65038354	65074713	+	2233	1	CCT6P3|C	0.791	0.662
NM_0327	chr11	1.17E+08	1.17E+08	-	2194	1	BUD13|Cw	17.372	18.36
NM_0071	chr12	1.3E+08	1.3E+08	+	3279	1	FZD10|CD	0	0.017
NM_0023	chr7	1530732	1543043	+	3345	1	MAFK|NFE	4.922	4.132
NR_10376	chr4	1.36E+08	1.36E+08	-	326	1	LINC00613	0	0
NM_0013	chr10	1E+08	1E+08	-	3576	1	CHUK|IKBI	4.572	5.983
NR_11709	chr6	1.68E+08	1.68E+08	-	2553	1	LOC44117	1	10.778
NM_0036	chr5	74627406	74641424	-	5515	1	ENC1|CCL	244.427	168.71
NM_0181	chr2	27663471	27694980	+	2954	1	SLC4A1AP	10.646	9.714
NM_0010	chr7	1.42E+08	1.42E+08	-	7286	2	KIAA1147|	0	0
NM_0164	chr11	64316492	64318084	-	1299	1	TRMT112|	58.84	43.453
NM_0011	chr11	90031106	90042025	+	2153	1	TRIM49C|	0	0
NM_0219	chr17	41754607	41786712	-	3194	1	JUP|ARVD	85.539	80.016
NM_0064	chr2	85694291	85698851	+	836	1	GNLY|D2S	0.077	0.016
NM_0001	chr15	43197228	43221125	-	2525	1	EPB42|PA	0	0
NR_04010	chr4	1.84E+08	1.84E+08	-	1875	1	LOC72817	0.151	0.037
NM_0011	chr2	1.31E+08	1.31E+08	+	6165	1	AMER3|FA	2.447	3.053
NM_1985	chr19	2274632	2282182	-	2564	1	C19orf35|	0.03	0.005
NR_11090	chr16	50668855	50671639	+	1106	1	LOC10192	0.256	0.26
NR_02699	chr5	5034359	5070002	+	1742	1	LINC0102C	0	0
NM_0011	chr9	72051381	72060605	-	1490	1	C9orf57|-|	0	0.018
NR_04500	chr11	86856931	86857954	-	1023	1	OR7E2P|O	0	0
NM_0012	chr8	55738744	55763980	-	2468	1	TMEM68|	6.457	6.31
NM_0011	chr1	32679906	32702760	-	3407	1	SYNC|SYN	12.602	13.874
NM_1332	chr17	40219322	40282187	+	5515	1	WIPF2|WI	18.371	18.962
NM_0029	chr2	1.19E+08	1.2E+08	-	1808	1	SCTR|SR|-	0	0.015
NR_12583	chr6	39888790	39897380	-	661	1	LOC10050	0.176	2.134
NM_0072	chr1	1.62E+08	1.62E+08	+	1266	1	DUSP12|D	41.929	48.719
NM_0011	chr19	54173412	54189882	-	2385	10	MBOAT7|	0	0
NM_0012	chr16	234546	266125	+	3191	1	FAM234A|	33.488	32.957
NM_0013	chr22	17563439	17590890	+	2065	1	SLC25A18|	2.054	1.287
NM_0183	chr2	1.28E+08	1.28E+08	-	6275	1	WDR33|N	11.778	10.224
NM_0011	chr3	46436005	46463671	-	2489	1	LTF|GIG12	0	0
NM_2073	chr1	15736314	15741392	+	3066	1	SLC25A34|	2.128	2.198
NM_0044	chr3	1.84E+08	1.84E+08	+	5045	1	DVL3|DRS	46.368	51.462
NM_0120	chr9	1.27E+08	1.27E+08	-	3565	1	ANGPTL2|	2.926	4.142
NR_10673	chr19	45436654	45436704	+	50	1	MIR6088|	0	0
NR_03062	chrX	1.47E+08	1.47E+08	-	74.7	3	MIR509-3|	0	0
NM_0157	chr10	1.33E+08	1.33E+08	-	975	1	CALY|DRD	6.837	14.849
NM_0010	chr11	5544561	5545523	-	961	1	OR52H1|C	0	0

NM_0011⁄	chr14	77800083	77933954	+	2018	1	ADCK1|-|-	6.3	8.158
NM_0035:	chr6	26045411	26045869	+	456	1	HIST1H3C|	0	0
NM_0010:	chr15	90265663	90272211	+	1344	1	NGRN|DS(	310.393	347.47
NM_0012!	chr17	39671122	39688070	-	2350	1	PGAP3|AG	6.255	4.162
NR_00291	chr20	38425195	38425330	-	135	1	SNORA71E	1.146	1.623
NM_0011:	chr6_GL0(	4868795	4902412	+	5941	2	SYNGAP1|	0.247	0.265
NR_04006	chr15	40039311	40057000	+	556	1	SRP14-AS1	2.783	3.474
NR_03393	chr15_KI2	4808776	4820182	+	3455	5	GOLGA8T|	0	0
NM_1537⁄	chr1	1.72E+08	1.72E+08	-	1473	1	PIGC|GPI2	30.496	28.203
NM_0013:	chr18	58863562	58986480	+	6160	1	ZNF532|-|	32.374	36.706
NR_11039	chrX	1.13E+08	1.14E+08	+	3669	1	LOC10192:	0.004	0
NM_0012{	chr2	46916129	47076136	+	5126	1	TTC7A|GID	3.66	3.76
NR_10683	chr16	87834592	87834660	-	68	1	MIR6775|I	2.465	9.064
NM_0012{	chrX	78271472	78327691	-	2685	1	CYSLTR1|(	0	0
NM_0011(	chr17	78214196	78225635	+	2625	1	BIRC5|API⁄	9.072	7.055
NM_0148(	chr17	6578325	6640927	-	4664	1	KIAA0753|	6.383	5.251
NM_0012{	chr2	96335741	96377095	+	6006	1	NCAPH|BF	2.018	1.779
NR_00440	chr1	28648600	28648734	+	134	1	RNU11|RN	0.577	0.613
NR_02679	chr10	26643108	26653454	+	6099	1	LINC00202	0	0
NR_11045	chr2	1.76E+08	1.76E+08	+	671	1	HAGLROS|	0	0
NM_0011{	chr16	68310974	68357266	+	2161	1	PRMT7|SE	16.605	16.632
NM_0012{	chr1	25831913	25859458	-	1386	1	AUNIP|AIE	18.029	19.883
NR_03967	chr7	1.38E+08	1.38E+08	+	63	1	MIR4468|-	0	0
NM_0012{	chr8	99960936	1E+08	-	4127	1	RGS22|CT:	0	0
NR_03031	chr7	30289794	30289890	+	96	1	MIR550A1	0	0
NM_0010!	chr2	70447280	70554015	-	4299	1	TGFA|TFG,	0.063	0.073
NR_12165	chr3	1.51E+08	1.51E+08	-	700	1	LOC10192:	0.111	0.02
NM_0059:	chr17	38705620	38729803	+	7478	2	MLLT6|AF	0	0
NR_03624	chr8	27886039	27886116	-	77	1	MIR4287|-	0	0
NM_0013(	chr16	57428169	57447528	-	2056	1	CIAPIN1|A	43.835	42.004
NM_0528!	chr5	1.77E+08	1.77E+08	-	4204	1	GPRIN1|G	57.901	86.802
NM_0013:	chr19	48271245	48287608	+	2490	1	ZNF114|-|	2.154	1.837
NM_0012!	chr18	63702959	63723893	+	1467	1	SERPINB11	0	0
NM_0012:	chr22	38982399	38992779	+	1453	1	APOBEC3E	0.115	0.217
NM_0024:	chr2	27309492	27323102	-	997	1	MPV17|M	32.15	33.659
NM_1981!	chr5	95555121	95605102	+	3335	1	ARSK|TSU	1.597	1.667
NM_0250!	chr11	75717819	75732958	+	2739	1	MOGAT2|	0.019	0
NR_03740	chr1	33332393	33332492	-	99	1	MIR3605|ı	58.867	61.706
NM_0012:	chr17	47970528	47981786	+	1979	1	CDK5RAP3	39.475	29.152
NM_0155(	chr13	87672615	87679615	+	4442	1	SLITRK5|LI	8.505	6.75
NR_12052	chr7	29125033	29128172	+	509	1	LOC10192:	0	0.108
NM_0010:	chr11	68010305	68029282	+	2717	1	ALDH3B1|	0.503	0.509
NM_0013(	chr5	1.76E+08	1.76E+08	+	1819	1	SIMC1|C5(	7.308	10.67
NR_03974	chr16	28958583	28958661	+	78	1	MIR4517|ı	3.306	3.161
NM_1984!	chrX	55623400	55626188	+	2786	1	FOXR2|FO	0	0
NR_07301	chrX	22193005	23293146	-	3760	1	PTCHD1-A:	0.01	0.029
NM_0210:	chr20	49245901	49278219	-	7354	1	ZNFX1|-|-	5.851	8.07
NM_0145!	chr18	50282343	50288322	-	2937	1	CXXC1|24:	19.755	22.8
NR_00154	chrY	12662367	12692224	+	5238	1	TTTY15|N(	9.593	7.866
NR_10699	chr14	72516820	72516898	+	78	1	MIR7843|I	0	0

NR_00330	chr15	25193321	25193402	+	81	1	SNORD115	0	0.169
NM_0008(	chr4	46243548	46390068	-	8448	1	GABRA2|-	0.003	0.289
NM_0037:	chr12	56763218	56787790	+	1611	1	HSD17B6|	1.088	1.403
NM_0059!	chr1	1.56E+08	1.56E+08	-	2091	1	CCT3|CCT-	230.868	208.213
NR_13424	chr5	1.37E+08	1.37E+08	+	342	1	LOC10537!	0	0
NM_0230(	chr3	1.39E+08	1.39E+08	-	2914	1	FOXL2|BPI	0.009	0.014
NM_0323{	chr3	1.49E+08	1.49E+08	+	4110	1	HPS3|BLO	2.911	2.106
NM_0010(	chr11	56490435	56491370	-	934	1	OR5M8|O	0	0
NM_0010⁄	chr5_KI27(	64345	121219	+	3917	2	SLC6A3|D/	0	0
NM_0208!	chr12	53711118	53727523	-	3029	1	CALCOCO1	33.402	57.415
NR_07340	chr8	6959289	6960161	-	283	1	DEFA9P|D	0	0
NM_0152:	chr11	9138826	9265326	-	4941	1	DENND5A	36.136	48.082
NR_03392	chr8	23225221	23230926	+	1967	1	LOC38964	0.02	0.028
NR_04981	chr17	46973017	46973100	+	83	1	MIR5089|-	0	0
NR_03888	chr6	40344345	40356006	-	3119	1	LINC00951	0.021	0.013
NM_0013:	chrX	1.35E+08	1.35E+08	-	1050	1	PLAC1|CT!	0.049	0.026
NM_0001!	chr7	1.56E+08	1.56E+08	-	2195	1	SHH|HHG:	0.012	0.162
NM_0011⁄	chr14	23568035	23578800	-	4270	1	JPH4|JP4|.	44.484	44.761
NM_1781:	chr17	28728814	28742766	+	2841	1	NEK8|JCK|	1.316	1.186
NR_14456	chr19	57867760	57869011	+	1251	1	UBE2CP5|	3.865	3.734
NM_0002!	chr1_GL38	94316	106457	-	3030	2	PKLR|PK1|	0	0
NM_0060!	chr11	57376769	57381150	-	861	1	PRG3|MBI	0	0.032
NM_0011⁄	chr17	44308559	44318670	+	2034	1	RUNDC3A	90.261	116.182
NM_0051(	chr1	1013467	1014539	+	662	1	ISG15|G1F	10.303	11.918
NM_0121:	chr7	90403334	90415954	+	3612	1	CLDN12|-|	5.508	5.715
NM_0011:	chr19	49948993	49961172	-	2865	1	SIGLEC11|	0.041	0.01
NM_0039(	chr3	1.84E+08	1.84E+08	+	2880	1	EIF2B5|CA	25.236	29.463
NM_1815:	chr17	40748021	40755332	-	1660	1	KRT25|AR'	0	0
NR_03969	chr13	65966330	65966429	-	99	1	MIR548X2	0	0
NR_11074	chr19	56765322	56799646	+	2147	1	ZIM2-AS1|	0.336	0.6
NM_0010(	chr1	1.72E+08	1.72E+08	+	2929	1	METTL13|	10.279	12.009
NR_11017	chr2	900216	905451	-	690	1	LINC0193!	0	0
NM_0031:	chr5	1.52E+08	1.52E+08	-	3578	1	SPARC|BM	84.071	50.707
NR_02410	chr21	33165470	33170236	-	591	1	LINC01548	0	0
NM_0005(	chr6_GL0C	3476638	3479992	+	1916.7	6	CYP21A2|(	0	0
NM_0149!	chr12	71754863	71793476	+	8739	1	RAB21|-|-	3.529	3.066
NR_13467	chr4	1113639	1132975	+	490	1	LOC10537⁄	0	0
NM_0012{	chr6	41789896	41871637	-	8798	1	USP49|-|-	5.434	7.133
NM_0035:	chr6	27892699	27893185	-	484	1	HIST1H2AI	0.32	0.311
NM_1989!	chr17	76872647	76950389	+	4042	1	MGAT5B|(	22.549	26.625
NR_04699	chr13	30103178	30108875	-	2623	1	LINC00365	0.049	0.005
NM_1489(	chr1	42733093	42740254	-	2852	1	CLDN19|H	0.14	0.024
NR_03615	chr19	18386562	18386634	+	72	1	MIR3189|i	0	0
NM_0022!	chr4	55078259	55125595	-	6023	1	KDR|CD30	0.432	0.07
NR_04560	chr2	1.06E+08	1.06E+08	-	2021	1	UXS1|SDR	13.933	15.371
NM_0010(	chr14	19803770	19828372	+	1005	1	OR4N2|OI	0	0
NM_0018(	chr16	22345936	22374617	-	2695	1	CDR2|CDR	5.2	4.95
NM_0207{	chr17	16620734	16653853	-	4236	1	ZNF624|-|	1.254	1.348
NM_1536:	chr12	54053877	54056030	+	1662	1	HOXC4|HC	0.016	0.148
NM_0063!	chr19	7926718	7943823	-	1985	1	TIMM44|T	26.021	27.746

NR_03337	chr6_GL0C	350142	354217	-	2782.4	8	LINC01623	0	0
NM_0030(	chr20	45206964	45209773	+	1691	1	SEMG1|CT	0	0
NM_1392!	chrX	1.55E+08	1.55E+08	+	743	2	CTAG1A|C	0	0
NM_0010!	chr22	41469125	41528989	+	2721	1	ACO2|ACC	100.755	90.941
NM_0332	chr12	54630839	54634879	-	501	1	LACRT|-|-	0	0
NM_0011	chr8	6406592	6447419	+	3620	1	MCPH1|BI	4.121	4.873
NM_0010(	chr12	6924463	6942321	+	4339	1	ATN1|B37	100.628	116.742
NM_0180	chr19	33080880	33130412	+	3340	1	GPATCH1|	5.142	5.241
NR_13494	chr3	44421132	44424025	-	743	1	LINC00694	0.087	0.313
NM_1825(	chr7	73861159	73865893	+	849	1	WBSCR28|	0.106	0
NM_0124	chr19	416583	460996	-	2480	1	SHC2|SCK	34.948	32.354
NM_0002	chr17	41565841	41572058	-	2280	1	KRT9|CK-9	0	0
NM_0012	chr11	62624826	62646726	-	3586	1	GANAB|G2	99.664	84.241
NR_04987	chr13	51348567	51348639	-	72	1	MIR5693|-	0.179	0.19
NM_0010(	chr21	42653752	42775508	+	1531	1	PDE9A|HS	18.309	44.884
NM_0011!	chr11	1.25E+08	1.25E+08	-	3024	1	SIAE|AIS6|	10.476	10.572
NR_02338	chr7	76980822	77023759	+	1750	1	DTX2P1-UI	0.125	0.141
NR_10690	chr8	1.44E+08	1.44E+08	-	59	1	MIR6846|	0	0
NM_0012	chr9	1.33E+08	1.33E+08	-	981	1	SURF1|CM	58.395	50.236
NM_0011!	chr1	2.42E+08	2.42E+08	-	2845	1	PLD5|PLD(	0.267	0.448
NR_02451	chr11	1.3E+08	1.3E+08	+	3331	1	APLP2|API	115.511	128.75
NM_0327	chr6	1.07E+08	1.07E+08	-	2882	1	RTN4IP1|N	3.208	3.759
NM_0176!	chr7	93099513	93118023	-	6850	1	SAMD9|C7	0.024	0.1
NM_0013	chr1	1.55E+08	1.55E+08	-	3167	1	PBXIP1|HF	20.975	19.921
NM_0018	chr22	26621964	26630672	+	803	1	CRYBA4|C	0.257	0
NM_0035	chr6	27871845	27872321	-	474	1	HIST1H3I|	0.19	0.144
NR_11024	chr9	1.33E+08	1.33E+08	-	558	1	OBP2B|LC	0	0
NM_0132	chr11	61755389	61788518	+	5718	1	MYRF|C11	0.873	1.567
NM_0068	chr1	78620382	78646145	+	5884	1	IFI44L|C1c	0.603	2.67
NR_00331	chr15	25201323	25201404	+	81	3	SNORD115	0	0
NM_0011(	chr12	1.12E+08	1.12E+08	-	15372	1	HECTD4|C	31.189	36.908
NM_0007(	chr19	40843538	40850447	-	1753	1	CYP2A6|CI	0.103	0.289
NR_13510	chr11	1.15E+08	1.15E+08	+	524	1	LOC10536!	0	0
NM_1532	chr11	62976597	62985023	-	2039	1	SLC22A6|I	0	0.134
NM_0144!	chr1	1.46E+08	1.46E+08	-	1938	1	RNF115|B	10.166	8.955
NM_0211	chr1	1.83E+08	1.83E+08	-	4232	1	RNASEL|PI	0.235	0.126
NM_0324	chrX	1.42E+08	1.42E+08	-	688	1	SPANXD|C	0	0
NR_10242	chr15	90230245	90265759	-	860	1	CIB1|CIB|(	24.543	22.743
NM_0012	chr2	1.73E+08	1.73E+08	+	3685	1	RAPGEF4|	2.327	1.431
NM_0013	chr4	4418969	4461972	-	1892	1	STX18|Ufe	18.959	17.208
NR_00244	chr9	1.27E+08	1.27E+08	-	135	1	SNORA65|	3.247	1.522
NR_03018	chr19	53666673	53666770	+	97	2	MIR512-2|	0	0
NR_12595	chr1	1.7E+08	1.71E+08	-	631	1	LOC10192	0.061	0.022
NR_03979	chr7	1844180	1844253	-	73	1	MIR4655|-	0	0
NM_0012(	chr19	35248656	35267964	+	1945	1	LSR|ILDR3	3.759	0.528
NM_0013	chr15	51056601	51094705	-	2001	1	TNFAIP8L3	0.103	7.831
NM_2014	chr12	2959312	3040676	+	1652	1	TEAD4|EF	0.695	0.705
NM_0175	chr2	1.01E+08	1.01E+08	+	2509	1	CNOT11|C	12.323	13.462
NM_0011!	chr17	40819092	40836274	+	2052	1	TMEM99|-	6.799	6.188
NR_11085	chr17	79919357	79924004	+	1532	1	LINC0197E	0.109	0.17

NM_0013(	chr16	30075976	30085377	+	1359	1	PPP4C|PP-	97.247	86.798
NM_0013‹	chr10	49734641	49762379	-	3269	1	OGDHL|-|-	5.269	5.937
NR_04538	chr3	50099603	50100988	-	1385	1	RBM5-AS1	12.763	14.983
NM_0013:	chr2	65056361	65087008	+	5849	1	CEP68|KIA	9.243	10.161
NM_0009:	chr7	87503863	87713323	-	4685	1	ABCB1|AB	0.316	0.488
NM_1538:	chr10	1.19E+08	1.19E+08	-	6729	1	CACUL1|C	4.169	5.506
NM_0220!	chr17	4733529	4739928	-	2320	1	CXCL16|C>	0.606	1.175
NR_03623	chr4	1.74E+08	1.74E+08	+	69	1	MIR4276|-	0	0
NM_1446(	chr12	1.29E+08	1.29E+08	+	2813	1	GLT1D1|-|	0	0.19
NR_03968	chr20	29880384	29880463	-	79.7	3	MIR4477A	0	0
NM_0159!	chr16	57256097	57284672	-	1491	1	PLLP|PML	0.208	0.331
NR_03020	chr19	53711002	53711088	+	86	1	MIR524|M	0	0
NM_1527(	chr7	48171460	48647495	+	17120	1	ABCA13|-|	0.093	0.108
NM_0011:	chr6	11094033	11138736	+	4687	1	SMIM13|C	8.167	8.212
NM_1527‹	chr7	3301448	4268999	+	10349	1	SDK1|-|-|:	7.56	14.286
NM_0306:	chr14	53036740	53153328	-	12863	1	DDHD1|P/	1.55	1.934
NM_0011:	chr4	1.34E+08	1.34E+08	-	4939	1	PABPC4L|-	0.023	0.022
NM_0012{	chr14	21016763	21070872	-	1941	1	NDRG2|SY	31.991	58.563
NM_0029(	chr1	1.52E+08	1.52E+08	-	1050	1	S100A10|4	6.349	11.205
NR_02986	chr19	53788705	53788773	+	68	1	MIR373|M	0	0
NR_00320	chr14	1.01E+08	1.01E+08	+	72	1	SNORD114	0	0
NM_0331{	chr17	41167231	41168172	-	940	1	KRTAP4-3|	0	0
NM_0061:	chr17	82314870	82317604	-	1277	1	CD7|GP40	0.071	0.064
NM_0012!	chr17_KI2	119784	130681	-	2048.9	10	TBC1D3K|-	0	0
NM_0011!	chr7	1.43E+08	1.43E+08	+	1392	2	MTRNR2L6	0	0
NM_0003!	chr11	17392885	17476845	-	4880	1	ABCC8|AB	1.458	1.925
NM_0013:	chr1	52350593	52366205	-	5315	1	CC2D1B|-|	5.526	5.334
NM_0011!	chr6	1.09E+08	1.09E+08	-	3360	1	MICAL1|M	48.811	28.727
NM_0011:	chr9	94603139	94640249	-	1479	1	FBP1|FBP|	0.105	0.333
NM_0250{	chr16	67828157	67847458	-	2327	1	CENPT|C1(	21.404	20.03
NR_03609	chr4	1.43E+08	1.43E+08	+	75	1	MIR3139|-	0	0
NM_0240:	chr7	1.55E+08	1.55E+08	+	4551	1	HTR5A|5-I	0	0.009
NM_0212:	chr9	1.07E+08	1.07E+08	+	10399	1	ZNF462|Zf	9.495	9.807
NR_00360	chr19	24162193	24163447	-	1254	1	HAVCR1P1	0.35	0.688
NR_02968	chr12	6964097	6964191	+	94	1	MIR141|M	0.274	0
NM_0012(	chr22	29554809	29581096	-	1923	1	NIPSNAP1	219.229	236.546
NM_0011(	chr1_KI27(	104160	104944	-	782	4	PPIAL4D|-	0	0.018
NR_02458	chr10	77926812	77929825	+	3013	2	DLG5-AS1|	0	0
NM_0012{	chr2	1.82E+08	1.82E+08	+	5187	1	SSFA2|CS-	3.99	6.747
NM_0012{	chr16	28342518	28362860	-	1396	1	NPIPB6|NI	0.074	0.137
NR_10704	chr13	1.13E+08	1.13E+08	+	79	1	MIR8075|I	0	0
NM_1383{	chr16	70661204	70686051	-	4960	1	MTSS1L|A	44.636	58.193
NM_0150‹	chr9	1.32E+08	1.32E+08	-	10988	1	SETX|ALS4	3.606	5.036
NM_0145‹	chr15	51751561	51816361	+	9172	1	TMOD2|N	14.135	24.566
NM_1784!	chr20	45722335	45725830	+	523	1	SPINT4|C2	0	0
NM_0011!	chr20	53185283	53495330	+	11649	1	TSHZ2|C2(	1.884	0.727
NR_12616	chr17	63996071	63999899	-	2923	1	PRR29-AS1	0.093	0.089
NM_0011‹	chr17	41976421	42017165	-	1830	1	DNAJC7|D	42.358	40.627
NM_2073:	chr11	58266792	58268260	-	1466	1	OR10W1|(	0	0
NM_1768:	chr1	61742477	62163919	+	8459	1	PATJ|Cipp	1.035	1.218

NM_0123(	chr7	1.44E+08	1.44E+08	+	934	1	OR2A5|OF	0	0
NR_11709	chr6	1.68E+08	1.68E+08	-	4518	1	LOC40128(	0.322	3.095
NR_03174	chr1	26554542	26554593	+	51	1	MIR1976|I	3.034	3.76
NR_03972	chr13	20433778	20433846	-	68	1	MIR4499|-	0	0
NM_0011:	chr2	96875882	96895162	-	879	1	FAM178B|	0.264	0.109
NR_04608	chr5	1.17E+08	1.18E+08	+	3085	1	LINC00992	0	0
NM_0053‹	chr6_GL0C	3083567	3086090	+	2517.2	5	HSPA1B|H	0.082	0.011
NR_13561	chr19	6426037	6433779	-	1397	1	SLC25A41|	1.347	1.706
NM_0011(	chr2	27442366	27446481	+	947	1	KRTCAP3|I	1.797	1.475
NM_0011:	chr2	55232678	55235853	+	887	1	RPS27A|CI	133.95	124.817
NR_10687	chr22	30007049	30007113	+	64	1	MIR6818|I	6.447	10.915
NM_0063:	chr1	2.02E+08	2.02E+08	+	1651	1	TIMM17A|	26.826	30.953
NM_0210:	chr5	1.71E+08	1.71E+08	+	1488	1	TLX3|HOX	6.62	8.634
NM_0011:	chr7	47655244	47661648	+	2906	1	C7orf65|-|	0	0
NM_1783:	chr14	22829883	22835037	+	1111	1	MRPL52|-	41.628	31.462
NM_0060!	chr5	1.81E+08	1.81E+08	-	1099	1	RACK1|GN	1066.123	1043.179
NM_0027:	chr6	1.05E+08	1.05E+08	-	2926	1	PREP|PE|F	29.074	29.224
NM_0051:	chr11	66593219	66606019	+	1022	1	CCS|-|-|1:	22.948	17.892
NM_0049{	chr1	1.6E+08	1.6E+08	+	3024	1	KCNJ9|GIR	8.527	7.428
NM_0067(	chr12	1.12E+08	1.12E+08	-	4022	1	BRAP|BRA	8.697	9.726
NR_04609	chr1	2.06E+08	2.06E+08	+	2239	1	LOC28458	1.261	1.884
NM_0013(	chr20	18024152	18059188	-	1505	1	OVOL2|EU	0	0.018
NM_0005:	chr1	2.07E+08	2.08E+08	+	8575	1	CR1|C3BR	0	0
NM_1533(	chr12	99647750	99650114	+	1863	1	FAM71C|-	0	0.007
NM_0140(	chr1	2.06E+08	2.06E+08	+	3450	1	IKBKE|IKK-	0.34	0.683
NM_0251!	chr2	97504798	97589965	-	5940	1	ANKRD36E	0.139	0.286
NM_0031:	chr15	40035690	40039202	-	1133	1	SRP14|ALU	160.48	159.661
NM_0202‹	chr1	2.27E+08	2.27E+08	+	2907	1	COQ8A|AD	11.119	9.009
NR_13498	chr1	59893308	59926798	-	1708	1	CYP2J2|CP	0	0.088
NR_03028	chr3	15873771	15873849	+	78	1	MIR563|M	0	0.176
NM_0013(	chr5	1.8E+08	1.81E+08	+	6321	1	CNOT6|CC	10.713	11.099
NR_11074	chr4	67417305	67422000	-	1954	1	LOC10192:	0.013	0.021
NM_0153:	chr8	18527303	19013686	-	11669	1	PSD3|EFA(	12.176	11.991
NM_0306(	chr19	4090322	4124129	-	1720	1	MAP2K2|(	70.674	67.49
NM_0011:	chr5	1.42E+08	1.42E+08	+	2146	1	RELL2|C5c	19.629	24.618
NR_10993	chr20	40004461	40008529	+	751	1	LINC0137C	0	0
NR_04668	chr3	98714333	98732651	-	764	1	ST3GAL6-A	0.321	0.215
NM_0011!	chr16	19067917	19080095	+	2602	1	COQ7|CAT	13.171	13.45
NR_10353	chr6	45328317	45372053	+	430	1	RUNX2|AN	0.03	0.032
NM_0018!	chr20	23747553	23750937	-	755	1	CST1|-|-|2	0	0
NM_0010(	chr1	2.48E+08	2.48E+08	-	961	1	OR2T12|O	0	0
NM_0045:	chr20	64164474	64242253	+	5510	2	MYT1|C20	0	0
NR_13620	chr5	1.57E+08	1.57E+08	-	884	1	LOC10272‹	0.088	0.139
NM_0063:	chr1	26177490	26189884	+	2513	1	CNKSR1|C	1.837	2.387
NR_13501	chr12	95803097	95823314	+	398	1	LOC10536!	0	0
NR_11058	chr2	42142435	42170231	-	1456	1	LOC10272:	0.035	0.028
NM_0012‹	chr1	2.47E+08	2.47E+08	-	2249	1	ZNF124|H:	2.287	1.529
NM_0201:	chrX	48973720	49002264	-	3006	1	GRIPAP1|(	35.545	34.393
NM_0011(	chr8	93916934	93926068	+	4229	1	PDP1|PDH	10.25	16.214
NR_02335	chrX	20136066	20136413	-	347	1	SCARNA9L	0.074	0

NR_12649	chr14	23415450	23417595	+	870	1	MHRT|My	2.831	2.377
NM_0179	chr11	11841423	11959325	+	7748	1	USP47|TRI	7.267	11.203
NM_2068	chr14	59595976	59630814	-	1695	1	RTN1|NSP	334.28	386.563
NR_00002	chr22	39316842	39316896	-	54	1	SNORD139	11.7	17.248
NM_0162	chr1	2.03E+08	2.03E+08	-	1632	1	CYB5R1|B!	29.642	29.098
NM_0010	chrX	55220347	55224108	+	545	1	PAGE5|CT	0	0
NM_0329	chr6	99482264	99515489	-	1432	1	USP45|-|-	0.792	0.612
NR_03022	chr19	53756741	53756830	+	89	1	MIR516A1	0	0
NR_02996	chr14	1.01E+08	1.01E+08	+	92	1	MIR433|M	11.352	7.295
NM_0154	chr2	68293010	68320051	-	1993	1	CNRIP1|C2	26.188	30.294
NM_0186	chr1	1.55E+08	1.55E+08	+	645	1	LENEP|LEF	0.14	0.127
NM_0057	chr12	57819927	57846964	-	5010	1	CTDSP2|O	26.381	37.422
NM_0068	chr6	1.01E+08	1.01E+08	-	8109	1	ASCC3|AS	2.872	3.248
NM_0010	chr7	33014113	33062797	-	1694	1	NT5C3A|N	5.351	6.315
NM_0013	chr19	38815422	38831857	-	1251	1	ECH1|HPX	75.588	62.89
NM_0011	chrX	1.54E+08	1.54E+08	+	1982	1	SRPK3|MS	0.475	0.422
NR_13463	chr22	41551709	41560883	-	342	1	LOC10537	2.413	2.803
NR_03175	chr5	93620696	93620788	-	92	1	MIR2277|	18.359	17.121
NM_0011	chrX	49532212	49539477	+	458	3	GAGE12B|	0	0
NM_0053	chr18	51030213	51085041	+	8757	1	SMAD4|DI	7.569	10.273
NR_04007	chr1	67832288	68202987	+	2237	1	GNG12-AS	0.571	0.367
NM_0013	chr11	1.19E+08	1.19E+08	-	2059	1	THY1|CD9	53.409	73.9
NM_0010	chr1	74568111	74673738	-	7142	1	ERICH3|C1	0.04	0.048
NR_03331	chr11	27506852	27677803	+	1469	1	BDNF-AS|/	2.484	2.648
NR_02448	chr22	17159389	17165445	+	338	1	CECR5-AS1	1.106	1.337
NM_0010	chr4	1.09E+08	1.1E+08	+	4616	1	SEC24B|SE	6.98	8.581
NR_12047	chr12	65873812	65881578	-	974	1	LOC10012	0.04	0.014
NR_10270	chr4	41220074	41256727	-	589	1	UCHL1-AS	0.044	0.442
NM_0041	chr19	17075781	17213295	+	7569	1	MYO9B|CI	13.967	13.308
NR_02435	chr21	34723807	34737182	-	688	1	LINC0016C	0	0
NR_02659	chrX	72941728	72943750	+	2022	2	FAM226A|	0	0
NM_0249	chr10	1.04E+08	1.04E+08	-	6469	1	STN1|AAF	5.577	3.86
NR_04702	chr13	60214347	60268104	-	957	1	LINC00434	0	0
NR_02742	chr8	12115782	12151189	+	1065	1	FAM66D|-	0	0.386
NM_0010	chr5	1.8E+08	1.8E+08	+	2448	1	RUFY1|RA	14.474	14.626
NR_04662	chr2	66439095	66440330	-	699	1	MEIS1-AS2	0.738	0.118
NM_0012	chr11	1.11E+08	1.11E+08	-	5882	1	ARHGAP2C	0.239	0.321
NM_0044	chr5	44304995	44388682	-	623	1	FGF10|-|-	1.387	0.088
NM_0038	chr1	1.68E+08	1.68E+08	-	2019	1	CREG1|CR	10.62	14.321
NM_0013	chr14	21477176	21499177	+	4461	1	TOX4|C14	28.174	29.51
NR_03835	chr14	30876179	30889808	-	1986	1	LOC10050	2.999	2.559
NM_0023	chr2	47369148	47387028	+	1707	1	EPCAM|DI	0.355	0.802
NM_1734	chr14	73237497	73274639	+	5805	1	PAPLN|-|-	0.482	0.321
NR_03974	chr17	27293910	27293996	-	86	1	MIR4522|	0	0
NR_13525	chr14	50821797	50823508	+	480	1	LOC10537	0.51	1.17
NM_0143	chr3	1.83E+08	1.83E+08	-	3333	1	LAMP3|CD	0.074	0.074
NM_0053	chr22	19148991	19150283	-	614	1	GSC2|GSC	0.042	0.067
NM_1826	chr20	5114953	5120001	-	1311	1	PCNA|ATL	65.48	56.857
NM_0059	chr2	1.36E+08	1.36E+08	-	3768	1	MCM6|M(	12.972	11.534
NR_13073	chr5	1.4E+08	1.4E+08	+	578	1	LOC10192	0	0.142

NR_12583	chr6	46492052	46533480	+	1341	1	LOC10192	0.019	0
NM_0033	chrX	1.3E+08	1.3E+08	+	3310	1	XPNPEP2|	0.008	0.008
NR_03605	chr1	23044305	23044372	+	67	1	MIR3115|	0	0
NM_0142	chr18	11981428	12030886	+	1527	1	IMPA2|-|-	7.388	2.808
NM_1816	chr16	66552563	66566287	+	724	1	CKLF|C32|	8.797	2.932
NM_0010	chr3	98515473	98523066	-	2172	1	CLDND1|C	29.735	30.692
NR_03318	chr1	1.79E+08	1.79E+08	+	2574	1	C1orf220|	0.386	0.479
NR_12641	chr5	73454190	73472966	-	502	1	LINC0138	0	0
NM_0011	chr11	63574471	63614469	-	1095	1	PLA2G16|	4.745	2.114
NM_0010	chr1	2.06E+08	2.06E+08	+	1657	1	C1orf186|	0	0.025
NM_0042	chr19	8390321	8404433	+	1621	1	RAB11B|H	154.571	128.351
NM_1707	chr20	4780023	4815123	-	5272	1	RASSF2|CE	34.604	42.549
NM_0332	chr14	1.05E+08	1.05E+08	+	1980	1	BTBD6|BD	33.751	28.169
NR_04650	chrY	3002996	3102272	+	526	1	LINC0027	0	0
NM_0011	chr19	51311848	51330848	+	3090	1	IGLON5|-|	63.366	64.394
NR_13122	chr2	1.68E+08	1.68E+08	-	917	1	LOC10561	1.884	1.494
NM_0012	chr6_GL0	3021781	3024174	-	1478	7	ATP6V1G2	0.044	0.111
NM_2065	chr19	50476477	50483526	+	2115	1	EMC10|C1	50.239	38.008
NM_0151	chr7	1.36E+08	1.36E+08	+	6220	1	NUP205|C	13.864	13.541
NM_0251	chr14	31561385	31861223	+	3045	1	NUBPL|C1	2.672	2.497
NR_13553	chr3	72663296	72664249	-	590	1	LOC10537	0	0
NM_0160	chr3	1.5E+08	1.5E+08	-	3699	1	COMMD2|	13.51	11.853
NR_04766	chr14	1.01E+08	1.01E+08	+	2638	1	MEG9|LIN	0.239	0.083
NM_0010	chr3	1.95E+08	1.95E+08	-	2337	1	TMEM44|	8.094	10.602
NR_01541	chr15	21552795	21640521	+	1589	2	LOC72792	0	0
NM_0011	chr16	67975663	67980549	-	1723	1	DPEP3|ME	0	0
NM_2014	chr16	188969	229482	-	1476	1	LUC7L|LU	59.025	64.903
NM_0010	chr20	45833831	45843275	+	3160	1	SNX21|C2	10.67	11.322
NR_03981	chr9	1.28E+08	1.28E+08	-	80	1	MIR4672|	0	0.342
NM_0023	chr3	1.16E+08	1.16E+08	-	9469	1	LSAMP|IG	8.186	11.63
NR_02684	chr2	87030675	87076413	-	2190	1	LOC28507	1.507	2.177
NR_02675	chr15	41284003	41299597	+	1890	1	OIP5-AS1|	17.26	13.117
NM_0056	chr11	64746389	64760297	-	3176	1	PYGM|-|-|	0.84	0.548
NM_0011	chr9	96929004	96939418	+	2507	1	NUTM2G|	0.01	0.033
NR_11085	chr6	1.18E+08	1.18E+08	-	1644	1	LOC10192	0.047	0.117
NR_03335	chr15	28378638	28392025	+	5264.2	9	GOLGA8G	0	0
NM_1825	chr11	1.19E+08	1.19E+08	-	7729	1	BCL9L|BCL	13.119	18.338
NM_0045	chr21	20998315	21540196	+	4982	1	NCAM2|N	2.13	3.852
NR_01535	chrX	63426559	63560993	-	2882	1	LINC0127	12.285	12.072
NM_1532	chr15	42542522	42548804	-	2407	1	LRRC57|-|	7.483	6.351
NM_0020	chr14	64939152	64942905	-	1084	1	GPX2|GI-G	0.19	0.505
NM_0012	chr6_GL0	3459000	3468665	+	1917.1	8	PPT2|C6o	0.047	0.014
NM_0012	chr14	1.05E+08	1.05E+08	+	1832	2	C14orf180	0	0
NR_04667	chr5	1.12E+08	1.12E+08	+	606	1	NREP-AS1	0	0
NM_0012	chr1	27234516	27308633	+	4801	1	WDTC1|AI	24.608	25.206
NM_0030	chr20	33407957	33443892	-	2330	1	SNTA1|LQ	7.061	6.555
NR_03991	chr20	62332487	62332557	-	70	1	MIR4758|	0	0.587
NM_0013	chr3	49357171	49358600	-	1009	1	GPX1|GPX	79.725	44.363
NM_0058	chr5	64768918	65018763	+	2074	1	CWC27|N	7.311	8.704
NM_0180	chr1	27959462	27968096	+	2687	1	XKR8|XRG	0.173	0.311

NR_00129	chr6	32552713	32560002	-	801	2	HLA-DRB6	0	0
NM_0190!	chr4	1.19E+08	1.19E+08	+	6575	1	USP53|-|-	0.286	0.344
NR_10412	chr18	77971296	77993727	-	1309	1	LINC0102!	0	0
NM_0306:	chr6	1.68E+08	1.68E+08	+	1499	1	KIF25|KNS	0.043	0.018
NM_0011(	chr8	1.43E+08	1.43E+08	+	1673	1	LY6K|CT97	0.008	0.025
NM_1524!	chr16	3432422	3443537	-	5502	1	ZNF597|H	0.762	1.013
NM_0161(	chr6	1.17E+08	1.17E+08	+	1679	1	RWDD1|C	18.553	14.904
NM_0012!	chr8	10764961	10839899	-	1485	1	PINX1|LPT	11.14	14.813
NR_02940	chr17	83226897	83240804	+	2252	1	RPL23AP8	0	0.006
NM_0024!	chr1	1.55E+08	1.55E+08	+	1303.5	2	MTX1|MT:	7.557	5.401
NM_0122(	chr9	1.33E+08	1.33E+08	+	9036	1	GTF3C4|K/	10.021	10.112
NR_13639	chr17	61361668	61367704	-	439	1	LOC10192	0	0.031
NM_0121(	chr21	41167801	41282534	+	8919	1	BACE2|AE	0.019	0.404
NM_2074	chr13	1.13E+08	1.13E+08	+	2236	1	ATP11AUN	0	0.006
NM_0068!	chrY	624344	659411	+	1943	2	SHOX|GCF	0	0
NM_0011:	chr4	2793023	2841096	+	9053	1	SH3BP2|3I	8.604	8.718
NM_0011(	chr3	46370142	46376206	+	3421	1	CCR5|CC-(	0	0
NM_0012!	chr19	43951223	43967600	+	2271	1	ZNF221|-|	1.067	1.454
NR_04674	chrX	1.36E+08	1.36E+08	-	396	1	INTS6L-AS:	0.033	0.069
NM_0011	chr1	50103910	50203785	+	3781	1	ELAVL4|HI	24.058	23.474
NM_1750(	chr12	52607570	52618559	-	2273	1	KRT73|IRT	0	0
NM_0011(	chr2	95476967	95484731	-	1659	1	TRIM43B|	0	0.008
NR_02667	chr9	27245684	27282793	-	2896	1	LINC00032	0	0.038
NM_0013	chr12	94306394	94459988	-	2882	1	CEP83|CCI	0.796	1.107
NR_12164	chr3	1.39E+08	1.39E+08	-	3535	1	LINC01391	0	0
NM_1534:	chr9	1.28E+08	1.28E+08	+	2107	1	ODF2|CT1	26.081	19.697
NM_0024(	chr14	49620771	49623481	+	2708	1	MGAT2|CI	9.994	10.617
NR_02948	chr9	94178834	94178920	+	86	1	MIRLET7D	1.049	0.478
NM_0059(	chr6	10762723	10830877	-	3815	1	MAK|RP62	0.257	0.291
NM_0013	chr9	1.38E+08	1.38E+08	-	1883	1	DPH7|C9o	19.385	22.462
NR_03625	chr9	90023441	90023535	-	94	1	MIR4290|	0	0
NR_04684	chr13	1.08E+08	1.08E+08	-	1805	1	FAM155A-	0.05	0.023
NM_0011	chr8	26293216	26372679	+	3659	1	PPP2R2A|	10.726	15.4
NR_03747	chr7	71307672	71307770	-	98	1	MIR3914-1	0	0
NM_0011	chr10	96832260	96964441	+	7776	1	LCOR|C10	4.475	2.891
NM_0066!	chr17	44959693	44968276	-	1496	1	C1QL1|C1	33.277	15.437
NM_0026(	chrX	49171837	49175119	+	1041	1	PLP2|A4|/	1.523	2.632
NM_0025	chr18	63887705	63903890	+	1898	1	SERPINB2|	0	0.014
NM_2072!	chr19	35268978	35279821	+	1518	1	USF2|FIP|	74.031	87.614
NM_0323(	chr19	12688916	12696641	-	1725	1	FBXW9|Fb	12.916	10.187
NM_0168:	chr12	1.13E+08	1.13E+08	+	3526	1	OAS2|-|-|	0	0
NR_03414	chr7	1.05E+08	1.05E+08	-	1329	1	LHFPL3-AS	0.039	0
NM_0190(	chr7	7566985	7607479	+	3308	1	MIOS|MIC	4.611	5.528
NM_0045!	chr17	10680332	10697568	-	1746	1	SCO1|SCO	12.354	14.434
NM_0011	chr11	15966449	16402867	-	8901	1	SOX6|HSS	1.008	1.306
NM_0207!	chr3	1.19E+08	1.19E+08	+	8065	1	ARHGAP31	0.743	2.974
NM_1735:	chr14	22883223	22887680	+	1852	1	REM2|-|-|	41.472	24.598
NM_0163	chrX	8169944	8171267	-	810	1	VCX2|VCX	0	0
NR_03748	chr6	1.59E+08	1.59E+08	-	92	1	MIR3918|	1.261	2.382
NR_10402	chr2	1.7E+08	1.7E+08	-	4109	1	FASTKD1|	2.742	3.747

NM_0180:	chr11	94973681	94999512	+	2967	1	KDM4D|JN	1.721	2.317
NR_03790	chr3	1.54E+08	1.54E+08	-	2594	1	ARHGEF26	0.229	0.238
NM_0011	chr7	1.5E+08	1.5E+08	+	1958	1	LRRC61|HS	0.29	0.525
NR_02685	chr1	32231660	32241710	-	2317	1	MTMR9LP	1.931	1.194
NR_03413	chr2	8007771	8328419	-	1991	1	LINC0029	0.006	0.014
NM_0010:	chr18	63970029	63986653	+	1259	1	SERPINB8|	1.505	1.142
NM_0321	chr1	966497	975104	+	2382	1	PLEKHN1|	0.049	0.017
NR_03336	chr21	29596040	29630747	+	1070	1	GRIK1-AS2	0.398	0.602
NM_0221	chr13	79318868	79406221	-	5120	1	RBM26|AF	5.339	6.011
NR_03843	chr17	50556207	50562108	-	1431	1	CACNA1G-	0.46	0.948
NM_0012(	chr7	1.01E+08	1.01E+08	-	2451	1	TFR2|HFE3	2.42	1.883
NM_0014	chr2	27496839	27523683	+	2167	1	GCKR|FGC	0.143	0.31
NM_1458	chr7	95485901	95528801	+	1542	1	ASB4|ASB-	0.176	0.044
NM_0148	chr17	40019097	40054636	-	3687	1	MED24|AF	43.681	39.85
NM_0012	chr16_KI2	284551	791405	+	1149	5	NPIPA8|LC	0.393	0.405
NM_0012	chr11	65996545	66002166	-	2917	1	EIF1AD|ha	12.443	12.138
NM_0002	chr6	42963873	42979243	-	3479	1	PEX6|HML	12.015	12.626
NM_0012	chr18	26454911	26657401	-	1656	1	KCTD1|C1	15.984	23.986
NM_0011	chr13	1E+08	1.01E+08	+	2461	1	PCCA|-|-|	12.013	10.53
NR_10673	chr1	20633679	20633788	+	109	1	MIR6084|	8.399	11.938
NM_0013(	chr12	14914027	14929125	-	1371	1	ERP27|C12	0.009	0
NR_03609	chr6	1.38E+08	1.38E+08	-	81	1	MIR3145|	0	0.169
NM_0162	chr3	1.51E+08	1.51E+08	+	3482	1	SELENOT|	8.328	8.937
NR_03819	chr6_GL0C	3619320	3622674	-	1663	7	AGER|RAG	0	0
NM_0010(	chr12	55247288	55248226	+	937	1	OR6C74|-|	0	0
NR_12638	chr1	1.1E+08	1.1E+08	+	575	1	LINC01397	0.135	0.048
NR_13804	chr13	30151886	30153074	-	303	1	LINC00384	0	0
NM_0051	chr17	42458844	42522579	+	4099	1	ATP6V0A1	74.785	87.235
NR_03650	chr9	92618050	92620534	+	2484	1	LOC10012	0.628	1.026
NR_03966	chr6	37555366	37555422	-	56	1	MIR4462|	0	0
NM_0011	chr12	2053250	2697949	+	13431	1	CACNA1C|	2.017	2.886
NM_1818(	chr20	45812576	45816957	+	756	1	UBE2C|UB	23.161	19.332
NM_0013	chr1	1.1E+08	1.1E+08	-	2156	1	EPS8L3|EP	0	0.025
NM_0029(	chr1	1.54E+08	1.54E+08	-	733	1	S100A3|S1	0.053	0.019
NR_00323	chr14	1.01E+08	1.01E+08	+	75	1	SNORD113	0	0
NM_0227	chr16	31489475	31508419	-	2798	1	C16orf58|	46.201	23.042
NM_0140	chr6	1.08E+08	1.08E+08	-	4459	1	OSTM1|GI	2.822	3.978
NR_03621	chr1	22863159	22863226	-	67	1	MIR4253|	53.498	47.428
NR_12588	chr4	1.49E+08	1.49E+08	+	2506	1	LOC10192	0	0.011
NM_0010:	chr12	56116590	56117832	+	579	1	RPL41|L41	14.697	11.899
NM_0530	chr17	7932124	7949919	+	4056	1	CNTROB|L	17.617	16.655
NR_02834	chr18	80147924	80178432	+	731	1	PARD6G-A	0.97	0.768
NR_03646	chr1	10430747	10442815	+	1426	1	CENPS|AP	9.855	6.512
NM_0010	chr12	1.22E+08	1.22E+08	+	3712	1	BCL7A|BC	46.291	57.976
NM_0055	chr16	75627724	75647687	-	1997	1	KARS|CMT	94.025	109.933
NM_0010(	chr22	39570753	39689736	+	9860	1	CACNA1I|	0.838	1.496
NM_0010(	chr11	58507176	58508105	-	928	1	OR5B21|-|	0	0
NM_0043	chr19	12938600	12944490	+	1900	1	CALR|CRT	289.554	266.976
NM_1788	chr17	17200995	17206332	-	2573	1	PLD6|ZUC	0.747	1.421
NM_0035	chr2	73762198	73780157	-	1628	1	DUSP11|P	12.996	12.317

NM_0010(	chrY	21511378	21525786	+	1867	3	RBMY1E|-	0	0
NM_0010:	chr11	60056628	60071115	+	1339	1	MS4A3|CD	0	0.01
NM_1447(	chr2	1.9E+08	1.9E+08	+	4391	1	ANKAR|-|-	0.082	0.159
NR_11073	chr19	54754595	54756329	-	692.8	84	LOC10192	0	0
NM_1774:	chr5	1.22E+08	1.22E+08	+	871	1	FTMT|MTI	0	0
NM_1984!	chr16	85098352	85112508	-	1894	1	FAM92B|-	0.116	0.441
NM_0016:	chr7	17298652	17346151	+	6234	1	AHR|bHLH	0.362	1.411
NR_03964	chr2	2.39E+08	2.39E+08	-	97	1	MIR4440|	0	0
NM_0012:	chr4	1.28E+08	1.28E+08	+	2211	1	LARP1B|LA	4.181	4.2
NM_0010(	chr14	20143736	20144662	+	925	1	OR4N5|-|-	0	0
NR_12058	chr11	1.27E+08	1.27E+08	+	492	1	LOC10192	0.052	0
NM_0012!	chr9	1.21E+08	1.21E+08	+	2530	1	GSN|ADF|	21.623	36.051
NR_03394	chrX	1.35E+08	1.35E+08	-	628	1	LINC00633	0	0
NR_13237	chr20	44711862	44746227	-	729	1	KCNK15-AS	1.008	1.804
NM_0013	chr6	47698545	47722021	+	3070	1	ADGRF4|G	0.017	0.027
NR_03998	chr22	40032332	40036577	+	4245	1	LOC10013	0.015	0.013
NM_0249	chr1	9104417	9129170	-	1147	1	GPR157|-|	0.483	0.418
NM_0322:	chr7	30771417	30892387	+	2713	1	FAM188B|	2.39	1.903
NR_03973	chr15	66496958	66497034	-	76	1	MIR4512|	0	0
NM_1817	chr12	88142296	88199887	+	7187	1	TMTC3|LIS	0.741	0.915
NM_1720!	chr8	71197433	71356511	-	3617	1	EYA1|BOP	0.848	1.363
NM_1392(	chr15	41899441	41972557	-	2885	1	EHD4|PAS	2.762	3.029
NM_0010(	chr9	1.05E+08	1.05E+08	-	955	1	OR13C9|C	0	0
NR_02718	chr10	63465229	63466562	+	1333	1	JMJD1C-AS	0.31	0.247
NM_0013:	chr20	2816302	2820284	+	3888	1	TMEM239	0	0
NM_0179:	chr19	1275521	1279244	+	890	1	C19orf24|	16.689	14.651
NM_0013	chr7	12330885	12403941	-	4840	1	VWDE|-|-	0.037	0.187
NR_12031	chr16	3947626	3950444	-	1375	1	LOC10272	0.281	0.269
NM_1738:	chrX	68498782	68537285	+	6273	1	YIPF6|FinG	7.194	6.609
NM_0034	chr12	49018975	49055324	-	19364	1	KMT2D|AA	9.556	10.551
NR_13525	chr14	44507409	44538510	+	373	1	LOC10537	0	0
NM_1990	chr14	55414211	55440545	-	1288	1	TBPL2|TBF	0	0.053
NM_0010:	chr12	22625142	22644415	+	905	1	ETNK1|EKI	7.195	5.721
NM_0133:	chr22	20131841	20148007	+	4999	1	ZDHHC8|D	8.496	7.425
NM_0314:	chr12	71663897	71680648	+	4835	1	THAP2|-|-	1.259	1.561
NM_0011:	chr11	77128270	77215241	+	7288	1	MYO7A|D	0.221	0.165
NR_11080	chr18	53579623	53581059	-	1035	1	LINC01917	0	0
NR_10499	chr5	1.19E+08	1.19E+08	+	538	1	LOC10246	0	0
NR_12600	chr16	70156340	70173448	-	568	1	LOC40054	2.179	2.773
NM_0010:	chr9	1.28E+08	1.28E+08	+	1711	1	CFAP157|	1.304	1.177
NR_04006	chr1	1.83E+08	1.83E+08	-	2449	1	SMG7-AS1	0.832	0.358
NR_03173	chr4	1.16E+08	1.16E+08	+	43	1	MIR1973|	0	0
NM_0035:	chr1	1.5E+08	1.5E+08	-	2220	1	HIST2H2BE	32.199	41.751
NM_0012!	chr5	74640023	74721288	+	2005	1	HEXB|ENC	34.809	21.307
NM_0065:	chr1	1.09E+08	1.09E+08	+	1926	1	SARS|SERF	211.491	274.587
NR_12641	chr10	50624951	50631636	+	2581	1	SGMS1-AS	1.099	1.072
NM_0055:	chr12	57434685	57452062	+	3297	1	INHBC|IHE	0.121	0.199
NM_0011:	chr8	41490562	41510980	+	1901	1	GOLGA7|G	39.854	43.822
NM_0066!	chr11	74988905	75007698	+	2743	1	NEU3|SIAL	5.067	5.767
NM_0011:	chr8	1.31E+08	1.31E+08	-	4081	1	ADCY8|AC	4.259	2.014

NM_0321.	chr7	92528773	92540481	+	4682	1	RBM48|C7	1.908	2.332
NR_13657	chrX	1.52E+08	1.52E+08	-	418	1	LOC10537.	0	0
NM_0153:	chr12	1.16E+08	1.16E+08	-	9345	1	MED13L|M	10.68	14.296
NM_0011:	chr12	58872155	58919546	-	3640	1	LRIG3|LIG:	0.857	3.865
NM_0186!	chr16	28494649	28498970	+	3786	1	APOBR|AF	0.014	0.011
NM_0032.	chr15	39581079	39598921	+	7220	1	THBS1|TH	2.407	3.751
NM_0010:	chr17	76080993	76103787	-	4784	1	EXOC7|2-5	59.29	59.143
NM_2034:	chr12	7470816	7503818	-	4088	1	CD163|M1	0.025	0.013
NR_00357	chr8	208345	232318	-	5784	1	RPL23AP5:	1.119	0.703
NM_0038.	chr11	70203163	70207402	+	1849	1	FADD|GIG	13.472	16.942
NM_0010:	chr1	44775574	44778740	+	697	1	RPS8|S8|-	713.359	645.293
NR_02947	chr22	46112749	46112822	+	73	1	MIRLET7A:	0	0.188
NM_0011!	chrX	1.25E+08	1.25E+08	+	2980	1	TEX13C|-|	0	0.005
NR_03993	chr2	1.51E+08	1.51E+08	+	77	1	MIR4773-1	0	0
NM_0012.	chr17	59893046	59950425	+	5337	1	RPS6KB1|I	2.788	3.47
NM_0141!	chr3	47016408	47163977	-	8410	1	SETD2|HB	9.171	13.083
NR_03993	chr2	2.13E+08	2.13E+08	+	79	1	MIR4776-1	0	0
NR_07348	chr16	30602558	30610433	-	3069	1	ZNF689|TI	11.452	12.858
NM_0013:	chr8	22275280	22356866	+	4316	1	PIWIL2|CT	0.182	0.184
NM_0031!	chr1	2.35E+08	2.35E+08	+	1947	1	TBCE|HRD	23.244	23.785
NM_0247:	chr5	1.35E+08	1.35E+08	+	3682	1	TXNDC15|	16.14	13.593
NR_03840	chr1	46538696	46570255	+	2437	1	MKNK1-AS	0.693	0.916
NM_0010(	chr7	1.43E+08	1.43E+08	+	940	2	OR6V1|GF	0	0
NM_1534{	chr6	37632508	37697990	-	8888	1	MDGA1|G	24.223	16.488
NR_02704	chr1	23627334	23640566	+	1020	1	MDS2|-|-|	0	0
NR_13525	chr13	27953526	27955363	+	1837	1	LINC00543	0	0.015
NM_0002(	chr17	42535933	42544448	+	2774	1	NAGLU|CN	10.96	10.305
NR_10404	chr11	17788050	18013162	-	1387	1	SERGEF|DI	10.895	13.124
NM_0319.	chr1	2.26E+08	2.26E+08	+	2002	1	MIXL1|MII	0.109	0.068
NM_0010.	chrX	1.54E+08	1.54E+08	+	1275	3	OPN1MW:	0	0
NM_0009{	chr19	17859878	17863324	+	664	1	RPL18A|L1	9.554	6.745
NM_0011:	chr17	38730257	38735605	+	2630	2	CISD3|Mir	0	0
NM_0047{	chr1	7771269	7781432	+	2198	1	VAMP3|CE	10.189	8.276
NR_00442	chr1	1.48E+08	1.48E+08	-	163	1	RNVU1-7|	0	0.168
NR_00152	chrY	23936727	23941622	-	341	2	TTTY3|LIN	0	0
NM_0012{	chr6	33200826	33204437	+	1857	6	SLC39A7|I	0	0.066
NR_02951	chr21	16539089	16539169	+	80	1	MIR99A|M	0	0
NM_0011.	chr10	1.03E+08	1.03E+08	+	2127	1	RPEL1|-|-|	0.697	0.837
NM_0010!	chr17	64002595	64020634	-	1129	1	ICAM2|CD	0.868	0.412
NM_0012{	chr4	1.85E+08	1.85E+08	-	3736	1	ACSL1|ACS	25.404	11.237
NM_0012{	chr16	2969342	2973489	+	2294	1	PAQR4|-|-	39.72	37.604
NM_0011.	chr11	1.29E+08	1.29E+08	-	10088	1	ARHGAP32	8.424	8.702
NR_02696	chr2	1.77E+08	1.77E+08	-	5197	1	LOC10013(	0.313	0.335
NM_0013(	chr7	91872876	91880720	-	1949	1	MTERF1|N	2.507	2.945
NM_0224{	chr7	1.13E+08	1.13E+08	-	7294	1	TMEM168	0.626	1.087
NM_0019:	chr11	1.12E+08	1.12E+08	+	4330	1	DLAT|DLT.	7.203	5.555
NM_0041(	chr3	1.39E+08	1.39E+08	-	682	1	RBP2|CRA	0.151	0.161
NM_0012{	chr15	75843286	75901047	+	2979	1	UBE2Q2|-	6.942	6.589
NR_10504	chr5	1.35E+08	1.35E+08	+	1106	1	LINC01843	0	0
NM_0010(	chr8	90621995	90645905	-	4815	1	TMEM64|-	2.362	1.826

NM_0153(	chr11	47716510	47767478	-	4137	1	FNBP4|FBI	12.635	16.359
NM_0314.	chr18	26915631	27185338	-	2251	1	CHST9|GA	0.756	1.789
NM_0012!	chr9	94030809	94109856	+	4475	1	PTPDC1|P	1.812	1.898
NM_0010:	chr5_GL33	147938	153209	-	1457	2	TAF9|MG(	0.257	0.103
NR_03747	chr22	35335640	35335758	+	118	1	MIR3909|	0	0
NM_0046.	chrY	22984263	23005463	+	1193.7	3	BPY2|BPY.	0	0
NR_03829	chr10	6779598	6842906	+	3065	1	LINC00707	0	0.004
NR_12167	chr4	1.9E+08	1.9E+08	+	875	2	LINC01262	0	0
NM_0047:	chr22	39349949	39385588	+	4432	1	SYNGR1|-|	33.426	46.54
NR_11039	chrX	1.19E+08	1.19E+08	+	829	1	LINC01285	0	0
NM_0010(	chr11	5840956	5841897	-	940	1	OR52E6|O	0	0
NM_0071.	chr9	5163863	5185618	-	704	1	INSL6|RIF1	0	0
NR_12577	chr13	81018391	81044442	+	923	1	LINC00377	0	0
NR_03789	chr5	24835280	24840583	-	2287	1	LOC34010	0	0
NR_00259	chr1	1.62E+08	1.62E+08	-	1549	1	RPL31P11	0.058	0.044
NR_10461	chr5	2303720	2312201	-	441	1	LOC10050	0	0
NM_1527(	chr11	65945644	65959963	+	1716	1	TSGA10IP|	0.556	0.112
NM_0210!	chr6	26216920	26217483	+	561	1	HIST1H2AI	2.873	3.345
NR_03038	chr5	55170646	55170742	-	96	1	MIR449B|	0	0
NM_0013.	chr21	7064426	7087230	-	2195	5	LOC10272.	0	0
NR_10402	chr4	1.44E+08	1.44E+08	-	1145	1	SMARCA5-	11.092	5.933
NR_12207	chr9	62867989	62898087	-	1026	1	LOC40332.	0.126	0.133
NM_0150!	chr9	99979181	1E+08	-	4850	1	ERP44|PD	7.728	7.439
NM_0013:	chr1	1.57E+08	1.57E+08	-	2015	1	HDGF|HM	164.499	153.57
NM_0147.	chr19	35718019	35738880	+	8432	1	KMT2B|CX	12.9	12.139
NM_0012(	chr3	58564112	58587113	-	3166	1	FAM107A|	1.637	1.622
NM_0013.	chr15	1.02E+08	1.02E+08	-	518	1	LOC10012.	0.548	0.185
NM_2034.	chr11	62686402	62689899	-	2581	1	LRRN4CL|-	0.36	0.239
NM_0012(	chr2	2.3E+08	2.3E+08	+	1907	1	SP100|lys	0.02	0
NR_12047	chr12	203645	205093	+	758	1	LOC10192	0	0.108
NM_1344:	chr19	5993164	6110653	-	4018	1	RFX2|-|-|	3.7	2.349
NM_0177(	chr1	23959811	23963459	+	2409	1	PNRC2|-|-	6.851	6.522
NR_00231	chr10	93958140	93961915	-	3775	1	PIPSL|PIP5	10.165	10.011
NM_1336.	chr9	1.33E+08	1.33E+08	-	1473	1	MED22|M	37.236	37.492
NM_0012(	chr13	45702469	45714559	+	1501	1	SPERT|CBY	0.009	0.018
NR_03171	chrX	85835780	85835858	+	78	1	MIR1321|	0	0
NM_1829.	chr1	89995095	90035530	+	9727	1	ZNF326|Z	2.695	2.818
NM_0011.	chr19	37594830	37614178	+	3229	1	ZNF540|N	2.252	2.316
NM_0121(	chr2	1.13E+08	1.14E+08	+	2064	1	FOXD4L1|	0.106	0.186
NM_0206(	chrX	15627316	15665031	-	1572	1	TMEM27|	0.55	0.566
NM_0325!	chr9	35812960	35815045	-	645	1	HINT2|HIT	50.934	34.678
NM_0010!	chrX	1.2E+08	1.2E+08	-	1303	2	RHOXF2B|	0	0
NM_0013.	chr7	93188541	93232198	-	2113	1	HEPACAM	0.915	0.454
NM_0010(	chr11	5046526	5047461	+	934	1	OR52J3|O	0	0
NM_0011:	chr11	1.19E+08	1.19E+08	-	4481	1	HYOU1|GI	66.274	65.985
NR_13580	chr1	39788976	39790170	+	1194	1	LOC10192	0.032	0.034
NM_0808.	chr20	2923207	3038669	+	3111	1	PTPRA|HE	98.746	103.672
NM_0252(	chr11	1.04E+08	1.04E+08	-	3984	1	PDGFD|IE(	1.181	0.646
NM_0039.	chr7	1.24E+08	1.24E+08	-	4434	1	WASL|N-V	6.188	6.221
NR_13500	chr20	25751208	25756050	+	3617	1	LOC10537.	0.267	0.345

NM_0011:	chr1	1661478	1692804	-	6014	1	SLC35E2B|	36.504	36.798
NM_0334	chr9	1.26E+08	1.27E+08	+	4812	1	MVB12B|(	68.35	71.209
NM_0012!	chr10	48446025	48493649	-	2342	1	ARHGAP22	0.264	0.415
NM_0038(	chr9	1.28E+08	1.28E+08	-	1540	1	DPM2|CD(	23.501	24.103
NR_12590	chr4	57425872	57465986	+	1032	1	LOC10192:	0.1	0
NR_04661	chr3	1.56E+08	1.56E+08	-	484	1	KCNAB1-A	0	0
NR_13277	chr3	13618381	13618548	+	167	1	SNORA93|	0	0
NR_13660	chr3	97764521	97801242	+	1730	1	ARL6|BBS:	3.264	2.708
NM_0321:	chr1	27322145	27336400	+	1606	1	TMEM222	44.935	45.424
NR_02658	chr13	1.14E+08	1.14E+08	+	2401	1	TFDP1|DIL	32.226	29.99
NM_2073!	chr15	21408964	21440382	-	2123	3	POTEB3|P	0	0
NM_0065:	chr20	53567071	53583097	-	5628	1	ZNF217|Z/	4.99	6.464
NM_0016	chr2	1.75E+08	1.75E+08	-	2632	1	ATP5G3|P	68.053	61.72
NR_10689	chr6	33290245	33290325	+	80	5	MIR6834|I	0	0
NM_0312!	chr17	17972813	18016875	+	2050	1	DRC3|CFA	2.315	2.532
NM_0019!	chr3	13549125	13638422	+	4164	1	FBLN2|-|-	2.629	4.582
NR_10986	chr20	5507875	5510002	-	355	1	LINC0172!	0.036	0
NM_0047(	chr15	59105085	59125045	+	1538	1	CCNB2|Hs	10.11	10.233
NM_0133	chr19	40422501	40426025	-	1202	1	SERTAD1|!	7.294	6.746
NR_13072	chr19	45641494	45642834	+	1074	1	EML2-AS1	2.797	2.729
NR_04749	chr13	81225866	81226983	-	1117	1	LINC00564	0	0
NR_00359	chrY	6449468	6457906	+	1105	2	TTTY7B|N(	0	0
NM_0176	chr15	61852391	62060465	-	13205	1	VPS13C|P/	1.435	2.561
NM_0012	chr13	51222334	51284241	+	4788	1	FAM124A|	6.398	7.558
NR_02695	chr16	74332406	74368255	-	2302	1	LOC28392	0.493	0.857
NM_0010(	chr10	48909484	48914235	-	1615	1	LRRC18|U	0.216	0.085
NM_0145!	chr22	37490362	37519203	-	3877	1	CARD10|B	1.546	2.014
NR_10376	chr6	4136072	4157385	+	2273	1	LOC10050	0.006	0.066
NR_11075	chr6	97305586	97708917	+	2097	1	LOC10192	1.838	2.495
NR_04972	chr11	19502673	19507229	-	3303	1	NAV2-AS5	0.008	0.021
NM_0010(	chr17_KI2	8452	24134	+	1605.5	2	METRNL|-	0.819	0.674
NM_1783!	chr1	1.53E+08	1.53E+08	+	1175	1	LCE1E|LEP	0	0.012
NR_04643	chr10	91163012	91227960	+	986	1	PCGF5|RN	3.936	4.223
NR_12644	chr1	1.19E+08	1.19E+08	+	560	1	WARS2-IT:	0	0
NM_0012!	chr12	51281038	51324198	-	2146	1	BIN2|BRAI	0	0
NR_03968	chr8	1E+08	1E+08	+	82	1	MIR4471|-	0	0
NM_0201(	chr5	1.52E+08	1.52E+08	-	2058	1	NMUR2|FI	0.013	0.093
NM_0011!	chr19	7916147	7919095	+	1248	1	TGFBR3L|	1.023	0.988
NM_0186!	chr17	74433697	74447429	+	1814	1	GPRC5C|R	3.44	8.14
NM_0011	chr22	42869766	42959881	-	3077	1	PACSIN2|S	25.242	26.29
NM_0009(	chr3	1.55E+08	1.55E+08	+	5618	1	MME|CAL	0.032	0.027
NM_0044	chr3	1.85E+08	1.85E+08	+	4202	1	EPHB3|EK:	15.035	23.267
NM_0062	chr20	59936832	59940297	-	3463	1	PPP1R3D|	0	2.334
NM_0010!	chr16	5044122	5066145	-	1539	1	C16orf89|	0.017	0.027
NM_0245:	chr3	45917899	45995824	-	8498	1	FYCO1|CA	0.957	1.059
NR_03159	chr15	88608107	88608197	+	90	1	MIR1179|I	0	0
NR_13493	chr13	20728931	20773958	-	764	1	EEF1AKMT	6.413	4.177
NR_00440	chr1	28508558	28508715	+	157	1	SNORA73E	1.642	1.047
NR_03394	chr1	1.58E+08	1.58E+08	-	832	1	LINC01704	0	0
NM_0013!	chr4	24527465	24584561	-	2999	1	DHX15|DE	47.085	55.437

NM_01832	chr9	27546546	27573493	-	3230	1	C9orf72|A	6.551	11.861
NR_02709	chr2	32825443	32929139	+	1985	1	LINC00486	0.032	0.076
NR_03615	chr17	81125877	81125955	+	78	1	MIR3065|	0	0
NM_0146(	chr3	1.31E+08	1.31E+08	-	5017	1	PIK3R4|VF	4.287	4.772
NR_10808	chr11	2328749	2377992	-	1171	1	CD81-AS1|	0.584	0.772
NM_0306	chr1	2.49E+08	2.49E+08	-	3173	1	SH3BP5L|-	30.814	29.76
NM_0012(	chr14	93927342	93929608	+	1464	2	FAM181A|	0	0
NM_0062	chr11	18480311	18526956	-	1536	1	TSG101|T	99.79	112.278
NM_1526	chr22	41998725	42028473	+	2308	1	WBP2NL|(	0	0.036
NR_12167	chr1	74589735	74626098	+	4964	1	ERICH3-AS	0.005	0.003
NR_10677	chr18	3885353	3885432	+	79	1	MIR6718|	0	0
NM_0024	chr16	88651940	88663149	-	1845	1	MVD|FP17	80.345	64.231
NM_0054(	chr7	39623553	39708124	+	2803	1	RALA|RAL	18.538	20.66
NM_0179	chr9	99906633	99974536	+	6898	1	STX17|-|-|	1.994	2.706
NM_0144	chr2	68860916	68871517	-	6145	1	BMP10|-|	0	0
NM_0011	chr11	67606852	67612541	+	1592	1	NDUFV1|(	98.71	97.256
NM_0012	chr19	14732697	14776927	-	5987	1	ADGRE2|C	0	0.002
NM_0013	chr18	70003806	70205726	-	6861	1	RTTN|MSS	2.18	2.33
NR_10698	chr2	5974662	5974732	+	70	1	MIR7158|	0	0.391
NM_0121	chr4	54009791	54064648	-	1142	1	CHIC2|BTL	6.989	7.832
NM_0012	chr20	45306843	45317824	+	2482	1	RBPJL|RBF	0	0
NM_0029	chr4	2469068	2515859	+	2951	1	RNF4|RES	32.293	39.569
NM_0033	chr1	1211326	1214168	-	1095	1	TNFRSF4|/	0	0.05
NM_0065	chr11	18231355	18236837	-	659	1	SAA4|C-SA	0	0.021
NR_02201	chr15	24981994	24983790	+	1796	1	PWARSN|	1.328	1.617
NM_0036	chr17	39665346	39666554	+	959	1	TCAP|CMD	2.877	4.071
NM_0224	chr7	1.57E+08	1.57E+08	-	4871	1	LMBR1|AC	11.488	11.951
NM_0011	chrX	49269843	49288093	+	2481	1	PPP1R3F|	11.251	10.931
NR_03036	chr17	62944215	62944312	+	97	1	MIR633|M	0	0.141
NR_13641	chr17	2712309	2712833	+	441	1	LOC10537	0.439	0.87
NM_0036(	chr4	1.44E+08	1.44E+08	+	7900	1	SMARCA5	16.917	15.4
NM_0011	chr19	38409058	38426305	-	2635	1	RASGRP4|	0.01	0.026
NM_0029	chr16	51135975	51151272	-	5138	1	SALL1|HEL	1.167	12.031
NM_1829	chr11	1.02E+08	1.02E+08	+	4339	1	BIRC3|AIP	0.006	0.013
NM_0121	chr8	406808	469875	+	2353	1	FBXO25|F	4.751	7.282
NM_0010	chr19	5842888	5851474	-	2183	1	FUT3|CD1	0.165	0.496
NM_0012	chr12	22448546	22544546	-	4597	1	C2CD5|CD	8.341	7.068
NM_0007	chr10	94938658	94989391	+	1850	1	CYP2C9|C	0	0
NM_0181	chr6	43671197	43687812	-	1181	1	MRPS18A|	32.971	27.545
NM_0011	chr17	50271406	50281485	+	2759	1	TMEM92|	0	0
NM_0013(	chr7	73830863	73832693	+	1828	1	CLDN4|CP	0.303	0.3
NM_0328	chr19	1354977	1378431	+	4085	1	MUM1|EX	45.066	35.106
NR_07355	chr19	49018248	49019484	-	489	1	LOC10105	0.105	0
NR_14444	chr19	58314950	58327248	-	700	1	LOC10537	14.164	10.351
NM_0010	chr9	4598316	4666674	-	3733	1	SPATA6L|(	0.183	0.216
NM_1818	chr1	1.5E+08	1.5E+08	-	2329	1	MTMR11|	1.976	1.641
NR_13525	chr3	1.71E+08	1.71E+08	-	2025	1	RPL22L1|-	5.011	5.127
NM_1768	chr11	6259674	6272127	+	2176	1	CCKBR|CC	0.427	0.554
NM_0142	chr4	1.59E+08	1.59E+08	+	6794	1	RAPGEF2|	11.332	17.971
NM_0323	chr4	932387	958655	+	1846	1	TMEM175	15.254	13.512

NM_0134(	chr11	61799625	61817057	-	4406	1	FADS1|D5	78.616	48.832
NM_0010	chr11	1919569	1938706	+	1162	1	TNNT3|TN	0	0
NM_0012	chr9	34329506	34343713	+	822	1	NUDT2|AF	33.975	28.66
NM_1983	chr19	49677152	49688450	+	1384	1	PRMT1|AN	278.346	298.266
NM_0011	chr14	1.05E+08	1.05E+08	+	3528	1	ZBTB42|LC	0.87	1.068
NR_10995	chr8	1.03E+08	1.03E+08	-	723	1	BAALC-AS1	0.196	0.303
NM_0067	chr2	53852913	53860033	-	2155	1	GPR75|GP	0.622	0.591
NM_0325	chr1	26038022	26046138	-	2343	1	SLC30A2|F	0.006	0
NM_0020	chr7	30594893	30634032	+	2402	1	GARS|CMT	84.72	122.372
NM_0123	chr20	63587594	63627101	-	4279	1	GMEB2|GI	8.597	8.95
NM_0010	chr12	30926904	30996602	+	5552	1	TSPAN11|	31.648	32.36
NM_0123	chr10	28677495	28682939	+	1719	1	BAMBI|NN	6.833	4.127
NM_0069	chr6	25652201	25701780	+	1466	1	SCGN|CAL	2.093	67.364
NM_0209	chr14	21385194	21437298	-	7435	1	CHD8|AUT	31.73	30.74
NM_0010	chr16	28984826	28990783	+	1433	1	LAT|LAT1|	1.539	1.252
NR_02726	chr14	49862551	49862849	-	298	1	RN7SL2|7L	3.591	3.08
NR_02655	chr21	28539318	28540355	+	512	1	LINC00161	0	0
NR_13460	chr18	36180001	36187435	-	589	1	LOC10192	2.036	1.442
NR_10382	chr4	1.19E+08	1.19E+08	+	4774	1	LOC72921	1.453	1.67
NM_0141	chr1	27830741	27851672	+	2368	1	PPP1R8|A	39.225	40.889
NM_1445	chr4	77713387	77819390	-	8780	1	CNOT6L|C	1.444	2.284
NM_0178	chr3	1.02E+08	1.02E+08	+	1841	1	TRMT10C|	9.154	8.742
NM_0318	chr18	61333582	61555773	+	3868	1	CDH20|CD	3.677	5.669
NM_0320	chr7	38178206	38230671	+	1733	1	STARD3NL	91.913	88.307
NM_0013	chr12	18683180	18738059	-	2138	1	PLCZ1|NYI	0.054	0.006
NM_0028	chr19	38374550	38383824	+	1530	1	PSMD8|HI	139.865	124.741
NM_0010(	chr11	5389377	5390434	+	1055	1	OR51M1|	0	0
NM_0010	chr15	48331424	48343373	+	2137	1	DUT|dUTP	16.097	14.255
NM_0012	chr11	1.24E+08	1.24E+08	+	1022	1	OR8G2|HS	0	0
NM_0010	chr3	47495640	47513709	-	1340	1	ELP6|C3or	28.597	26.454
NM_0211	chr9	15470644	15510289	-	1654	1	PSIP1|DFS	23.862	29.87
NM_0318	chr17	15303812	15341641	-	1773	1	TEKT3|-|-|	0.073	0.579
NM_2058	chrX	48004336	48009729	+	725	2	SPACA5|L	0	0
NM_0207	chr2	1.96E+08	1.97E+08	-	6897	1	HECW2|NI	2.316	3.608
NR_03644	chr15	28654442	28685264	+	2933	2	HERC2P9|	0	0
NR_03745	chr2	53849122	53849205	-	83	1	MIR3682|	0.621	0
NR_03960	chr20	38516563	38516632	+	69	1	MIR548O2	0	0
NM_0055	chr8	81656916	81686354	-	3385	1	IMPA1|IM	3.98	3.553
NR_10501	chr5	78358764	78360541	-	1548	1	SCAMP1-A	5.447	7.618
NM_0072	chr15	41309268	41332621	-	1229	1	OIP5|573C	2.193	1.571
NM_0011	chr2	40112146	40430304	-	6027	1	SLC8A1|N	4.781	2.932
NR_03740	chr7	98881650	98881729	+	79	1	MIR3609|	0	0
NM_0012	chr17	81399721	81466332	+	10424	1	BAHCC1|B	14.79	12.884
NM_0011	chr16	55846154	55875370	-	2159	2	CES5A|CA	0.012	0.006
NR_00440	chr19	48638039	48682245	+	2644	1	SEC1P|SEC	2.775	2.088
NM_0179	chr7	66921216	66958551	+	4229	1	TMEM248	16.366	19.715
NM_1748	chr14	1.05E+08	1.05E+08	+	1883	1	C14orf79|	12.743	10.38
NR_00301	chr12	98599635	98599884	+	249	1	SNORA53|	0.104	0.11
NR_10706	chr16	14930820	14930879	+	59	5	MIR6770-2	0	0
NR_03977	chr18	35591737	35591806	+	69	1	MIR3975|	0	0

NM_0019!	chr22	50525753	50530085	-	1646	1	TYMP|ECG	1.551	0.674
NR_10377	chr1	1.13E+08	1.13E+08	-	2453	1	LOC10099(	0.284	0.346
NM_0210:	chrX	48346428	48356753	-	1309	1	SSX3|CT5.:	0	0
NR_00143	chr6	33112516	33129113	+	736	8	HLA-DPB2	0	0
NR_10415	chrY	22316678	22320384	+	204	1	LOC10065:	0	0
NM_0011(	chr19	44506159	44529525	-	1795	1	CEACAM2(	0	0
NR_03791	chr10	1.23E+08	1.23E+08	-	2120	1	CUZD1|ER	0.255	0.129
NR_03033	chr9	1.38E+08	1.38E+08	+	97	1	MIR602|M	0.266	0
NM_0028!	chr20	36996351	37096215	-	5878	1	RBL1|CP1(	1	1.372
NM_0146:	chr15	84748587	84806432	+	8166	1	ZNF592|C/	12.788	9.963
NM_1488!	chr4	13568742	13627704	-	10514	1	BOD1L1|B	3.098	4.419
NM_0011:	chr12	7189686	7211483	+	3133	1	PEX5|PBD.	26.849	36.584
NM_0012;	chr5	15501438	15939796	+	4329	1	FBXL7|FBL	7.517	6.575
NR_03337	chr6	1.09E+08	1.09E+08	+	1748	1	LINC00222	0.015	0
NM_0013!	chr10	4963262	4977966	+	1368	1	AKR1C1|2-	30.424	21.557
NR_04610	chr9	1.35E+08	1.35E+08	+	3726	1	LOC40155;	0	0.015
NM_0047!	chr20	32277651	32335008	+	6102	1	KIF3B|FLA	23.769	26.505
NM_0240!	chr16	30423698	30430052	-	1115	1	DCTPP1|C	42.15	33.856
NM_0190:	chr7	1.51E+08	1.51E+08	+	3982	1	CHPF2|CS(	12.427	13.762
NR_02392	chr14	23938731	23954197	-	2758	1	DHRS4-AS:	14.637	15.391
NM_1383(	chr8	1.45E+08	1.45E+08	-	3000	2	ZNF251|-|	0	0
NR_10991	chr20	60087686	60101387	+	815	1	LOC72929(	0	0
NM_0012(	chr11	73876699	73927736	+	1535	1	PAAF1|PA.	24.485	28.857
NM_0034{	chr21	14961235	15064805	-	7550	1	NRIP1|RIP	2.738	6.497
NM_0054!	chr17	44798448	44830239	-	7639	1	GJC1|CX45	5.833	7.039
NR_12030	chr16	80155958	80563135	-	559	1	LOC10272-	0.484	0.172
NM_0012{	chr3	39808914	40260320	+	4813	1	MYRIP|SL/	0.086	0.122
NR_03719	chr2	2.16E+08	2.16E+08	-	3680	1	LINC00607	0.014	0.022
NM_0011-	chr15	75282841	75295807	+	3429	1	GOLGA6D|	0	0
NM_0331(	chr1	2.06E+08	2.06E+08	-	3375	1	SLC45A3|I	3.102	4.148
NM_0058-	chr16	21641284	21652666	-	1036	1	IGSF6|DOI	0.149	0.119
NM_0011!	chr15_KI2	4932598	4949251	+	3923	2	GREM1|C:	0	0.22
NR_04986	chr9	1.33E+08	1.33E+08	+	64	1	MIR548AV	0	0
NR_02687	chr7	1.03E+08	1.03E+08	+	2009	1	FAM185A|	1.489	1.5
NM_0051-	chr17	28546707	28552628	-	1377	1	UNC119|H	79.383	68.405
NM_0012!	chr16	57619693	57665039	+	3704	1	ADGRG1|E	223.391	161.739
NM_0013:	chr8	11493992	11564604	+	2477	1	BLK|MOD'	0.005	0.039
NR_12643	chr4	14470466	14888169	-	4664	1	LINC00504	0	0.018
NR_03610	chr8	29957272	29957348	-	76	1	MIR3148|r	0	0
NM_0017:	chr19	47309847	47322070	+	2337	1	C5AR1|C5.	0.326	0.205
NM_1832:	chr12	1.33E+08	1.33E+08	-	6184	1	ZNF605|-|	2.656	4.224
NM_0012!	chr1	54715822	54742655	+	2086	1	TTC4|-|-|1	15.854	18.313
NM_0224{	chr5	1.42E+08	1.42E+08	-	5227	1	ARAP3|CE	6.544	6.09
NM_0012;	chr2	37641882	37672199	-	5052	1	CDC42EP3	7.631	11.284
NM_0061!	chr12	56104319	56113910	+	2611	1	PA2G4|EB	46.542	48.063
NM_0012!	chr3	1.14E+08	1.14E+08	+	3905	1	QTRT2|QT	3.196	2.508
NM_0004-	chr11	36568013	36579760	+	6576	1	RAG1|RAG	0.18	0.127
NR_03844	chr17	41549606	41554495	-	2138	1	LINC00974	0	0
NR_02989	chr17	81125883	81125949	-	66	1	MIR338|M	0	0
NR_03620	chr19	49308797	49308868	-	71	1	MIR4324|r	0.908	0.386

NM_0011(	chr5	75068297	75236878	-	6009	1	ANKRD31|	0.099	0.05
NM_0148:	chr3	36826817	36945057	-	10459	1	TRANK1|L	0.678	0.614
NM_0010(	chr11	6199224	6200186	+	961	1	OR52W1|(	0.054	0.029
NM_0160:	chr2	3379675	3479571	+	1886.5	2	TRAPPC12	13.779	11.428
NM_0064!	chr17	2055099	2059687	+	3153	1	HIC1|ZBTE	0.478	0.513
NM_0218:	chr11	86374736	86423109	+	2495	1	CCDC81|-|	0.501	0.675
NM_0021{	chr5	55935097	55994993	-	9036	1	IL6ST|CD1	1.189	2.272
NM_0013:	chr3_KI27(	117264	189793	-	14969.6	8	MUC4|AS(	0	0.002
NM_0010(	chr5	1.81E+08	1.81E+08	-	934	1	OR2Y1|OR	0	0
NM_0013(	chr10	27195214	27242139	-	3715	1	ACBD5|-|-	8.236	6.098
NM_0033(	chr6	1.16E+08	1.16E+08	-	5256	1	TSPYL1|TS	30.541	33.513
NM_0020:	chr11	94543851	94549898	+	6045	1	FUT4|CD1	0.518	0.301
NM_1446:	chr3	19148525	19535643	+	5045	1	KCNH8|EL	6.389	3.426
NM_0335:	chr15	40358235	40371056	+	5021	1	DISP2|DISI	14.794	14.837
NM_0013(	chr14	70907405	71115382	+	12550	1	PCNX1|PC	4.288	10.469
NM_0212(	chr19	18868546	18896144	-	2554	1	CERS1|EPI	38.408	32.451
NM_0062!	chr6	1.38E+08	1.38E+08	+	4679	1	TNFAIP3|A	1.124	0.948
NR_00334	chr15	25099498	25099595	+	97	1	SNORD116	0.532	0.141
NM_0044:	chr2	96143169	96145440	-	1679	1	DUSP2|PA	0.123	0.228
NR_10349	chr15	82471477	82513999	-	2952.5	2	GOLGA2P1	1.122	1.262
NR_11075	chr19	35597895	35601501	+	371	1	LINC01766	0	0
NM_0010⁄	chr19	49335420	49340604	+	1199	1	CD37|GP5	2.022	1.976
NM_0011(	chr11	47586678	47589194	+	1398	1	FAM180B|	0.341	0.774
NR_13531	chr13	49235516	49247840	-	650	1	LOC10537(	2.182	1.011
NM_0009!	chr19	14472466	14475362	-	1416	1	PTGER1|EI	0.082	0.106
NR_13427	chr5_GL33	260376	268513	-	331	2	LOC10192:	0	0
NM_0010{	chr2	2.38E+08	2.38E+08	+	4265	1	RBM44|-|-	0.036	0.161
NM_0012{	chr2	2.41E+08	2.42E+08	-	2384	1	STK25|SOI	87.469	79.936
NR_03746	chr6	1.58E+08	1.58E+08	+	68	1	MIR3692|-	0	0
NM_0012⁄	chr3	70954714	71064923	-	6368	1	FOXP1|12(	1.782	3.792
NM_0011:	chr17	45135647	45144181	+	1860	1	ACBD4|HN	4.284	5.125
NR_10693	chr7	5711840	5711910	-	70	1	MIR6874|I	4.973	3.522
NM_0252(	chr6	31676684	31680373	-	722	7	LY6G5C|C(	0	0
NM_0176{	chr9	1.13E+08	1.13E+08	+	2340	1	BSPRY|-|-	0.022	0.029
NR_03616	chr21	41167557	41167629	+	72	1	MIR3197|-	0	0
NM_1829(	chr9	14511	29739	-	1824	1	WASHC1|I	2.679	2.268
NM_0013:	chr19	11887784	11914550	+	1765	1	ZNF69|Co:	1.534	1.606
NM_1721!	chr1	1.12E+08	1.12E+08	-	2627	1	KCND3|BR	1.286	3.452
NM_0159!	chr1	1.01E+08	1.01E+08	-	1778	1	DPH5|AD-	18.68	24.02
NM_1985!	chr2	2.18E+08	2.18E+08	+	1311	1	CATIP|C2c	0.157	0.084
NM_0010(	chr11	59421979	59422953	-	973	1	OR5A2|-|-	0	0
NM_0010{	chr2	1.28E+08	1.28E+08	+	6666	1	MYO7B|-|	0.004	0.002
NM_0013:	chr15	55538881	55587978	-	7712	1	PYGO1|-|-	1.251	1.9
NM_0161:	chr2	26033860	26137454	+	3777	1	RAB10|-|-	26.033	32.435
NR_12039	chr13	1.12E+08	1.12E+08	+	700	1	LINC00403	0.976	5.498
NM_0012⁄	chr18	3502145	3845358	-	2231	1	DLGAP1|D	4.947	7.484
NM_1446!	chrX	84317874	84502479	-	6215	1	HDX|CXor	1.29	1.241
NR_01540	chr20	5496067	5504596	-	6094	1	LINC00654	0.091	0.171
NR_03991	chr20	34990400	34990472	-	72	1	MIR499B|	1.433	1.332
NM_0013:	chr10	69180204	69209098	+	2418	1	SUPV3L1|!	11.624	14.456

NR_04663	chr19	48910930	48918891	-	431	1	NUCB1-AS	18.637	19.672
NR_10406	chr13	44142328	44154075	+	1187	1	SMIM2-AS	0.185	0.208
NM_0159:	chr7	1.43E+08	1.43E+08	+	1048	1	GSTK1|GS	3.839	6.365
NM_0020(	chr1	34792998	34795747	+	1618	1	GJA4|CX3	0.032	0.025
NR_02421	chr19	47923779	47923899	+	120	10	SNAR-A4|-	0	0
NM_0252	chr19	18257096	18274509	-	3837	1	KIAA1683|	3.367	3.837
NM_0012!	chr22	20394115	20408463	+	3609	1	ZNF74|CO	24.283	26.965
NM_0013(	chr19	15107364	15114988	+	3092	1	SYDE1|7h:	2.289	1.905
NR_12633	chr2	98761063	98771898	-	2113	1	LOC10192	0.006	0
NM_0334:	chr19	19033578	19058178	+	2370	1	ARMC6|R:	24.492	25.984
NM_1750(	chr1	1.5E+08	1.5E+08	-	446	1	HIST2H2AI	0.116	0.215
NM_1723	chrX	1.55E+08	1.55E+08	-	759	1	CTAG2|CA	0	0
NM_1390:	chr17	19378461	19383544	+	2842	1	MAPK7|BI	23.228	24.252
NM_0057!	chr5	1.52E+08	1.52E+08	+	2821	1	G3BP1|G3	31.834	39.29
NM_0011	chr3	50558025	50571027	-	2401	1	C3orf18|G	5.95	9.43
NM_0010(	chr1	1.59E+08	1.59E+08	-	976	1	OR6Y1|OR	0	0
NM_0012(	chr1	31578145	31587686	+	1832	1	TINAGL1|	0.007	0.037
NM_0121	chr19	18531752	18543596	-	1784	1	FKBP8|FKI	192.506	209.486
NM_0146	chr17	28614440	28645159	-	7394	1	KIAA0100|	38.907	43.912
NM_1986	chr21	44654213	44654659	-	444	1	KRTAP12-	0	0
NM_0034	chr22	32512553	33006821	-	3026	1	SYN3|-|-|	1.939	4.499
NM_0143	chrX	21705972	21758160	-	928	1	SMPX|DFN	0.042	0
NM_0010(	chr4	9826224	10040248	-	1853	1	SLC2A9|GI	0.014	0.015
NM_0010	chr4	17515165	17527104	+	818	1	CLRN2|-|-	0.095	0.05
NM_0013	chr10	74120257	74151085	-	4971	1	AP3M1|-|	16.001	16.937
NM_1450	chr3	50606454	50611831	-	2106	1	CISH|BACT	1.561	1.867
NR_12602	chr6	181466	205484	-	2042	1	LOC28576	0.013	0.047
NR_03980	chr8	6745168	6745240	-	72	1	MIR4659B	0	0.19
NM_0227:	chr1	2.26E+08	2.26E+08	-	3555	1	ACBD3|GC	8.128	7.74
NM_0013	chr5	1.75E+08	1.76E+08	+	1010	1	SFXN1|TC	29.017	27.245
NR_12871	chr16_KI2	763812	763876	-	64	8	MIR3670-3	0	0
NM_0010(	chr17	20863395	20896140	-	2800	1	CCDC144N	0	0.592
NM_1722	chr18	22413601	22417915	-	4312	1	CTAGE1|C	0	0.038
NR_11061	chr20	32509959	32520285	+	1768	1	LOC10192	0.27	0.364
NR_10376	chrX	1.54E+08	1.54E+08	-	596	1	CH17-340I	9.692	7.101
NM_0324	chr5	1798385	1799842	-	610	1	MRPL36|B	30.078	30.044
NM_0169	chr1	1.61E+08	1.61E+08	-	4818	1	F11R|CD3	0.824	2.718
NR_00305	chr3	1.84E+08	1.84E+08	+	75	1	SNORD66|	0	0.183
NM_0011	chrX	49579938	49587314	+	578	7	GAGE12D|	0	0
NR_03786	chr4	6200733	6233936	+	1481	1	LOC28548	0.104	0.462
NM_0048	chr19	14031740	14052905	+	2643	1	IL27RA|CR	1.551	2.462
NM_0011	chr3	1.84E+08	1.84E+08	-	3133	1	CLCN2|CIC	6.692	6.61
NM_0326	chr19	18193230	18196740	+	1351	1	MPV17L2|	13.924	12.501
NM_1749:	chr17_GLC	119850	123414	-	1371	3	ARHGAP27	0.019	0
NR_11030	chr13	52128895	52132723	+	3427	1	LOC10192	0.094	0.1
NM_0333:	chr22	42079691	42084284	+	1566	4	SMDT1|C2	0	0
NM_0027	chr10	1.22E+08	1.23E+08	+	2109	1	HTRA1|AR	19.924	10.859
NM_0011	chr16	30748299	30761181	+	2343	1	PHKG2|GS	12.426	12.937
NM_0333	chr10	60778454	60794852	+	1733	1	CDK1|CDC	6.569	6.094
NM_0124!	chr2	1.13E+08	1.13E+08	+	5020	1	PSD4|EFA(	0.118	0.248

NM_0016(	chr9	1.37E+08	1.37E+08	-	8103	1	ABCA2|AB	17.513	19.346
NM_0049!	chr9	1.28E+08	1.28E+08	+	2291	1	FPGS|-|-|!	16.827	16.19
NR_03417	chr17	46193573	46196723	+	520	3	KANSL1-AS	0	0
NM_0013:	chr2	2.33E+08	2.33E+08	+	927	1	EIF4E2|4E-	40.21	40.869
NR_13456	chr7	20217577	20221699	+	681	1	LOC10050(	0.038	0.02
NM_1812(	chr4	1.76E+08	1.76E+08	+	7322	1	WDR17|-|	1.599	2.303
NR_03163	chr2	1.14E+08	1.14E+08	-	137	1	MIR1302-3	0.282	0
NR_12066	chr10	80531250	80533123	+	919	1	LOC10272:	2.848	3.547
NM_1532(	chr3	1.12E+08	1.12E+08	+	2057	1	PLCXD2|-|	5.679	6.446
NM_0011:	chrX	1.24E+08	1.24E+08	+	2503	1	SH2D1A|D	0	0
NR_00296	chr6	1.6E+08	1.6E+08	-	131	1	SNORA20|	0.197	0.209
NM_0012‹	chr1	2641368	2789665	-	1766	2	TTC34|-|-|	0	0
NM_0152!	chr12	57055643	57078791	-	5388	1	NEMP1|TN	3.491	4.001
NM_0204:	chr18	11851390	11854449	+	3057	1	CHMP1B|(	39.756	30.445
NM_0011‹	chr10	30844636	31031937	-	3100	1	ZNF438|b/	1.601	2.518
NM_1750(	chr14	38254000	38256370	-	2368	1	CLEC14A|(	0	0.035
NR_10984	chr3	1.09E+08	1.09E+08	+	1488	1	LINC01205	0	0
NM_1984:	chr19	8322584	8343262	-	2774	1	KANK3|AN	6.354	6.814
NR_04979	chr3	1.24E+08	1.24E+08	+	96	1	MIR5002|-	0	0
NM_1989(	chr12	27958084	27971983	-	1324	1	PTHLH|BD	0.058	0.145
NM_0010:	chr8	94641136	94707466	+	3776	1	ESRP1|RBI	0.02	0
NR_11008	chr7	7550104	7566065	-	2406	1	LOC10192	0.638	0.461
NM_0001(	chr21	43773665	43776375	-	935	1	CSTB|CPI-I	88.931	71.488
NM_0011:	chr13	41311205	41358718	+	1813	1	NAA16|NA	2.105	2.584
NM_0182:	chr7	43639247	43729541	-	919	1	COA1|C7o	63.64	64.983
NM_0162:	chr6	1.58E+08	1.58E+08	+	4271	1	SNX9|SDP	4.555	3.919
NM_0010:	chr2	26581202	26641368	-	764	1	CIB4|KIP4	0.034	0.072
NM_0012!	chr5	10679230	10761275	-	2294	1	DAP|-|-|5	49.944	50.704
NM_2033‹	chr1	28887091	29120046	+	5670	1	EPB41|4.1	65.986	32.064
NR_02400	chr22	46295143	46296660	-	1517	1	GTSE1-AS1	0.705	1.499
NR_03963	chr2	1.81E+08	1.81E+08	-	59	1	MIR4437|	0	0
NM_0011(	chr11	1.12E+08	1.12E+08	+	706	1	C11orf88|	0.055	0
NM_0039:	chr4	41744082	41748970	-	3025	1	PHOX2B|N	0.026	0.018
NR_03892	chr8	1.43E+08	1.43E+08	-	5681	1	LOC10028:	0.03	0.111
NM_0012{	chr1	31296982	31364953	+	1505	1	ZCCHC17|	77.558	78.25
NM_2057(	chr19	5678414	5680896	-	898	1	C19orf70|	37.173	22.04
NM_1749:	chr10	1.31E+08	1.31E+08	-	2603	1	TCERG1L|-	0	0
NR_02412	chr1	28578538	28581854	-	733	1	SNHG12|A	10.149	12.968
NM_0178!	chr1	1081818	1116356	-	2092	1	C1orf159|	9.165	8.492
NM_0120{	chr5	95885098	95962071	-	6032	1	ELL2|-|-|5	0.844	1.714
NR_13210	chr2	1.15E+08	1.15E+08	-	754	1	DPP10-AS:	0	0
NR_12056	chr11	76782581	76783062	-	346	1	LOC10192:	0.633	1.386
NM_0322‹	chr15	82041778	82046143	-	3526	1	MEX3B|M	20.309	18.728
NM_0245:	chr13	1.11E+08	1.11E+08	-	1892	1	CARS2|CO	20.758	19.119
NM_1735!	chr12	1.17E+08	1.18E+08	-	16986	1	KSR2|-|-|:	1.214	0.673
NR_03899	chr13	23469512	23487464	+	2084	1	LINC00327	0.148	0.223
NM_0013:	chr8	30995802	31033194	-	1878	1	PURG|PUF	4.071	5.076
NR_10499	chr5	1.21E+08	1.21E+08	+	501	1	LOC10246	0	0
NM_0009‹	chr2	68178857	68252519	-	3003	1	PPP3R1|C/	21.579	28.01
NR_12203	chr3	42934244	42936785	-	2140	1	KRBOX1-A:	0	0

NR_02990	chr6	52148923	52149041	+	118	1	MIR133B|	0	0
NR_03667	chr7	99980762	99984237	+	1179	1	AZGP1P1|	0	0.023
NR_00277	chr21	46229231	46251701	+	2335	1	MCM3AP-A	6.924	7.203
NR_10683	chr16	57772289	57772352	-	63	1	MIR6772|	3.479	5.87
NM_0012(	chr16	4354542	4416961	-	3753	2	CORO7|06	0	0
NM_0012	chr16	81100879	81181362	-	5494	1	PKD1L2|P(	0.129	0.095
NM_0071	chr19	58059239	58069742	+	3327	1	ZNF135|ZI	1.093	4.088
NR_02665	chr12	54078839	54081823	-	902	1	LOC10024	0	0
NM_0133	chr22	37282383	37315348	+	3194	1	CYTH4|CY	0	0.017
NR_03985	chr13	1.02E+08	1.02E+08	-	70	1	MIR4705|	0	0
NM_0209	chr3	1.7E+08	1.71E+08	-	10123	1	SLC7A14|F	0.879	1.028
NR_11001	chr17	50346032	50361185	+	3395	1	XYLT2|PXY	5.127	6.015
NM_0011	chr3	46882249	46903799	+	1974	1	PTH1R|PFI	0.594	0.68
NR_00319	chr14	1.01E+08	1.01E+08	+	68	1	SNORD114	0	0
NR_03624	chr5	1708785	1708868	-	83	1	MIR4277|	0	0
NM_0055	chr15	66702336	66781999	+	2872	1	SMAD6|A(	0.489	0.296
NM_0005	chr1	1.61E+08	1.61E+08	-	1947	1	MPZ|CHM	0.139	0.134
NM_0011	chr17	4899653	4902932	+	3077	1	C17orf107	0.486	0.592
NM_0163	chr16	46979	53632	-	819	1	POLR3K|C	17.821	18.079
NM_0011(	chr2	2.19E+08	2.19E+08	-	5913	1	ZNF142|pI	8.635	8.916
NM_0003	chr13	48303747	48481890	+	4743	1	RB1|OSRC	5.616	6.385
NR_00369	chr14	20326441	20326539	-	98	1	SNORD126	0	0
NR_13617	chr2	1.78E+08	1.78E+08	+	838	1	LOC10537	0.062	0.229
NM_1334	chr1	1.01E+08	1.01E+08	+	8201	1	SLC30A7|Z	0.942	1.059
NR_12540	chr3	1.81E+08	1.81E+08	-	755	1	LOC10272	0.051	0.689
NM_0013	chr3	39263494	39280036	-	3104	1	CX3CR1|C	0.116	0.057
NM_0145	chr2	1.21E+08	1.21E+08	-	9269	1	TFCP2L1|(	0.003	0.006
NR_12041	chr13	1.06E+08	1.06E+08	+	1026	1	LINC00343	0	0
NM_0015(	chrX	71615916	71618517	-	1620	1	CXCR3|CD	0	0
NM_0011	chr16	50287912	50318134	+	6113	1	ADCY7|AC	1.284	1.589
NM_0189	chr9	1.37E+08	1.37E+08	-	2369	1	FBXW5|Fb	48.232	50.411
NM_0017	chr7	93424487	93574730	-	3561	1	CALCR|CR	2.477	1.777
NR_04608	chr2	2.19E+08	2.19E+08	+	1990	1	LOC10012	0	0.007
NR_00274	chr1	1.74E+08	1.74E+08	-	76	1	SNORD47|	3.054	5.587
NM_2015	chr17	7036075	7040121	+	1742	1	SLC16A13|	3.538	2.485
NM_0010	chr15	40552913	40565057	-	1704	1	C15orf57|	14.891	16.551
NM_1735	chr4	1.88E+08	1.88E+08	-	1625	1	TRIML2|SI	0.04	0.067
NM_0010(	chr16	2088710	2135898	-	14090	1	PKD1|PBP	11.319	13.227
NM_0012	chr1	20112650	20119566	-	1858	1	PLA2G2D|	0	0
NM_0011	chr3	52524369	52533777	-	1799	1	NT5DC2|-	128.705	140.412
NR_13425	chr5	1.8E+08	1.8E+08	+	877	1	LOC10537	0.294	0.234
NM_0013	chr17	48930096	48944842	-	1204	1	SNF8|Dot3	64.489	59.589
NM_0046	chr5	1.38E+08	1.38E+08	-	3137	1	CDC23|AN	12.125	12.409
NM_0043(	chr2	264869	278282	+	1543	1	ACP1|HAA	69.456	57.043
NM_1453	chr6	1.08E+08	1.09E+08	+	4996	1	LACE1|AF(	1.154	0.907
NR_02337	chr1	2.29E+08	2.29E+08	-	120	10	RNA5S13|	0	0
NM_0012	chr13	83877205	83882393	-	4742	1	SLITRK1|LI	12.214	9.575
NM_0010(	chr9	1.27E+08	1.28E+08	+	2824	1	LRSAM1|C	22.956	22.728
NM_0073(	chrX	96684663	97469838	+	3753	1	DIAPH2|D	0.323	0.321
NM_0332	chr11	1.05E+08	1.05E+08	-	1092	1	CASP1|ICE	0.024	0

NM_0018!	chr16	58157908	58197878	-	1644	1	CSNK2A2|	24.265	15.93
NR_03987	chr17	6655449	6655502	+	53	1	MIR4520-2	0.243	0.517
NR_03626	chr15	1.02E+08	1.02E+08	-	137	4	MIR1302-1	0	0
NM_0026!	chr5	1.35E+08	1.35E+08	-	2378	1	PITX1|BFT	0.022	0.086
NM_0010(	chr11	55913650	55914582	-	931	1	OR5W2|O	0	0
NM_0182:	chr19	46466491	46471563	-	3691	1	PNMAL1|-	56.443	49.767
NM_0012!	chr13	59665589	60163985	-	4558	1	DIAPH3|AI	0.215	0.376
NR_10243	chr13	51082848	51172388	-	2028	1	LINC00371	0	0.007
NM_0012;	chr2	79025686	79028504	+	810	1	REG3G|LP	0.016	0
NM_0010!	chr1	13368431	13371947	-	2118	1	PRAMEF19	0	0
NM_0025ξ	chr12	79591965	79691010	-	1942	1	PAWR|PAI	1.454	1.474
NM_1537!	chr4	88695913	88698235	-	2320	1	NAP1L5|D	12.888	22.913
NM_0024ξ	chr5	1.41E+08	1.41E+08	-	721	1	NDUFA2|E	89.736	64.42
NR_12064	chr10	61452639	61493431	+	1237	1	TMEM26-/	0.01	0
NR_00306	chr2	2.19E+08	2.19E+08	+	1376	1	TUBA4B|T	0	0.01
NM_0154:	chr20	3777504	3781455	-	1578	1	SPEF1|C2C	2.19	1.016
NR_10989	chr5	1.7E+08	1.7E+08	+	1212	1	CTD-2270F	0	0.079
NM_0012!	chr11	13277727	13387265	+	2773	1	ARNTL|BM	4.236	9.469
NM_0035:	chr6	26183796	26184230	+	432	1	HIST1H2BE	0.358	0.317
NM_0012!	chr11	13387996	13463297	-	2358	1	BTBD10|G	30.413	35.881
NR_13491	chr19	14333743	14343913	+	763	1	LINC01842	0	0
NM_0020(	chr11	69773029	69775403	-	1215	1	FGF4|HBG	0	0
NM_0010ξ	chr16	53703963	54114467	+	4281	1	FTO|ALKBI	31.817	44.556
NM_0012ξ	chr18	58670009	58671873	-	1201	1	LOC10192	1.235	0.547
NM_1446ξ	chr19	54160096	54173250	-	2447.1	10	TMC4|-|-|	0	0
NM_1529!	chr6	36390551	36442889	-	2101	1	PXT1|STEF	0.123	0.072
NM_0012(	chr2	1.1E+08	1.1E+08	+	1211	2	LIMS4|LIM	0	0
NM_0009ξ	chr17	39200283	39204727	+	724	1	RPL19|L19	682.802	654.335
NR_13620	chr5	2917854	2920528	+	455	1	LOC10537	0	0
NM_0240ξ	chrX	1.52E+08	1.52E+08	+	1297	1	PRRG3|PR	0.835	0.539
NM_0314:	chr5	1.04E+08	1.04E+08	-	3491	1	NUDT12|-	2.8	2.982
NM_0010(	chr1	2.48E+08	2.48E+08	+	946	3	OR2T5|OR	0	0
NM_0121:	chr20	31739101	31801800	+	3642	1	TPX2|C20c	9.665	7.981
NM_0012!	chr4	95051679	95158450	+	5384	1	BMPR1B|/	1.118	1.473
NR_02984	chr20	58817615	58817694	-	79	1	MIR296|M	0	0
NM_0013(	chr5	1.49E+08	1.49E+08	+	2382	1	PCYOX1L|-	11.616	12.305
NM_0010:	chr3	1.84E+08	1.84E+08	-	1254	1	PARL|PRO	24.347	29.415
NM_0182ʌ	chr4	76949714	77038615	+	5571	1	SEPT11|-|-	42.819	76.092
NM_0225;	chr7	1.01E+08	1.01E+08	-	6303	1	GIGYF1|G\	15.524	15.829
NM_0006:	chr11	58622673	58625732	+	1889	1	CNTF|HCN	0.607	0.877
NR_03399	chr9	32551717	32553017	+	551	1	TOPORS-A	5.148	4.549
NR_04749	chr13	46296445	46297844	-	1399	1	LINC00563	0	0.01
NM_0010:	chrX	1.55E+08	1.55E+08	+	591	3	H2AFB2|H	0	0.093
NR_03624	chr6	72967687	72967753	-	66	1	MIR4282|-	0	0
NM_0032ʌ	chr3	30606502	30694141	+	4619	1	TGFBR2|A	0.533	0.216
NR_00292	chr17	59962363	60019052	-	1904	1	TBC1D3P1	1.05	1.144
NR_02401	chr3	1.58E+08	1.58E+08	+	880	1	PQLC2L|C3	0	0
NM_0052!	chr22	40679178	40682814	+	2419	1	MCHR1|GI	10.042	6.512
NM_0011;	chr17	11241423	11564063	+	7351	1	SHISA6|-|-	0.151	5.299
NR_11195	chr1	1.45E+08	1.45E+08	-	1113	1	LOC10309	0.266	0.246

NM_0011	chr2	46901870	46916112	-	4012	1	MCFD2|F5	22.326	21.204
NM_0187	chr7	1.3E+08	1.3E+08	-	6473	1	CEP41|JBT	9.376	6.589
NM_0005	chr9	12693386	12710266	+	2866	1	TYRP1|CA	0.346	0.32
NR_03743	chr11	70872270	70872368	-	98	1	MIR3664|	0	0
NM_0022	chr13	97953675	98024296	+	5971	1	IPO5|IMB	26.303	26.405
NM_0010	chr1	12774848	12777905	+	1773	1	PRAMEF12	0	0
NM_0334	chr5	16451519	16465785	-	1684	1	ZNF622|ZF	24.355	28.996
NM_1750	chr12	1.32E+08	1.32E+08	-	4700	1	DDX51|-|-	6.167	5.855
NM_1530	chr6	1.12E+08	1.12E+08	-	2946	1	FYN|SLK|S	99.682	164.587
NM_0008	chr1	36795527	37034243	-	9201	1	GRIK3|EA	8.265	5.637
NM_0160	chr8	70637266	70669212	-	1544	1	LACTB2|C	1.887	1.322
NR_10697	chr9	1.23E+08	1.23E+08	-	93	1	MIR7150|	0	0
NR_11078	chr1	1.17E+08	1.17E+08	-	1810	1	LOC10192	0.085	0.469
NM_0013	chr7	1.38E+08	1.38E+08	-	7029	1	CREB3L2|	5.127	2.728
NM_0029	chr3	78597238	79767909	-	6806	1	ROBO1|DU	38.864	37.837
NR_03415	chr3	1.27E+08	1.27E+08	+	871	1	NUP210P1	0	0
NR_02989	chr12	54337216	54337314	+	98	1	MIR148B|	0	0
NR_13619	chr3	1.42E+08	1.42E+08	+	969	1	LOC64673	0	0.028
NM_0145	chr10	50799409	50885675	-	9254	1	A1CF|ACF	0	0.004
NM_1304	chr7	36852906	36986266	-	3727	1	ELMO1|CE	10.434	15.799
NM_0011	chr5	1.27E+08	1.27E+08	+	2237	1	LMNB1|AD	42.842	40.277
NM_0010	chr4	38772239	38782990	-	3480	1	TLR10|CD	0	0.02
NR_03019	chr19	53706252	53706336	+	84	1	MIR526A1	0	0
NR_03975	chr19	39409623	39409678	-	55	1	MIR4530|	0	0
NM_0541	chr9	1.23E+08	1.23E+08	+	940	1	OR1J2|HG	0	0
NR_03317	chr2	85598547	85602699	-	1479	1	TMEM150	7.122	7.872
NM_0006	chr9	21384255	21385397	-	1140	1	IFNA2|IFN	0	0
NR_03022	chrX	1.39E+08	1.39E+08	-	82	1	MIR504|M	0	0
NR_12585	chr6	70394202	70399417	+	669	1	EVADR|-|-	0	0
NM_0010	chr19	50203628	50310544	+	6769	1	MYH14|DI	1.236	1.317
NM_0004	chr14	74498183	74612331	-	8516	1	LTBP2|C14	0.491	0.314
NM_0010	chr11	55811367	55812476	+	1107	1	OR5L1|OR	0	0
NM_0007	chr8	58490178	58500161	-	2867	1	CYP7A1|C	0	0
NR_12050	chr7	27241429	27247229	-	476	1	EVX1-AS|E	30.04	0.777
NR_11005	chr12	1.27E+08	1.27E+08	-	849	1	LOC10192	0	0
NR_04699	chr13	38567724	38579516	+	527	1	LINC0036	0	0
NM_0021	chr5	35856875	35879603	+	4590	1	IL7R|CD12	0.02	0.075
NR_10406	chr14	62134150	62139973	-	292	1	LINC00644	0	0
NM_0011	chr6	1.35E+08	1.35E+08	-	7013	1	HBS1L|EF-	5.997	6.849
NM_0002	chr6_GL0	2712158	2723902	+	1386.8	5	MICA|MIC	1.004	0.879
NR_12640	chr2	33706886	34297746	+	766	1	LINC01317	0.034	0.143
NM_0012	chr6_GL0	2144982	2156466	+	3189	5	DDR1|CAK	0.049	0.056
NM_0038	chr2	2.06E+08	2.07E+08	+	6198	1	ADAM23|	3.917	5.567
NM_0035	chr6	27807479	27807931	-	450	1	HIST1H2B	0.258	0.244
NR_03408	chr4	57109762	57205299	+	1384	1	IGFBP7-AS	0.689	0.336
NR_10700	chr1	12166943	12167038	+	95	1	MIR7846|	0	0
NR_03827	chr19	36313061	36331718	-	978	1	LINC0066	19.63	11.792
NM_0011	chr1	1.5E+08	1.5E+08	+	782	1	PPIAL4C|C	0	0
NM_0031	chr11	3855703	4093210	+	4024	1	STIM1|D1	11.166	17.026
NM_0004	chr4	73404239	73421484	+	2318	1	ALB|HSA|	0.111	0.295

NR_13428	chr5	1.23E+08	1.23E+08	-	2589	1	LOC10192	0.095	0
NM_1988	chr11	59144779	59155038	+	3549	1	FAM111A|	0.698	1.208
NM_0011	chr16	86530176	86555203	-	2992	1	MTHFSD|-	5.986	6.913
NM_0012	chr20	63528423	63537370	-	2394	1	PTK6|BRK	0.786	0.521
NM_0047	chr8	72537391	72938349	+	3577	1	KCNB2|KV	1.842	1.061
NM_0068	chr12	1.2E+08	1.2E+08	-	2937	1	RAB35|H-r	40.155	41.996
NR_13234	chr5	1.52E+08	1.52E+08	-	1173	1	LOC10065	1.473	0.817
NR_02950	chr3	1.89E+08	1.89E+08	+	85	1	MIR28|MI	0	0
NM_2136	chr10	1.19E+08	1.19E+08	-	1372	1	SFXN4|BC	11.879	11.601
NM_0143	chr22	43824507	43862498	-	2456	1	SULT4A1|	18.647	33.819
NR_03058	chr5	1.38E+08	1.38E+08	-	77	1	MIR874|M	0	0
NM_1387	chr11	69294138	69297287	+	2299	1	MYEOV|O	0	0.048
NM_1387	chrX	75782988	75785244	-	2254	1	MAGEE2|	0.086	0.061
NM_0054	chr9	14719734	14722717	-	1201	1	CER1|DAN	0.064	0.741
NM_0178	chr1	3630767	3650107	-	1685	1	WRAP73|	18.556	19.517
NM_0013	chr6	56056590	56091661	-	2278	1	COL21A1|	0.091	0.06
NM_0013	chr15	50241949	50265965	-	2530	1	HDC|-|-|1	1.264	0.07
NM_0006	chr8	18210103	18223689	+	1800	1	NAT1|AAC	0.437	0.411
NR_13583	chr15_KI2	3501822	3510796	+	1028	2	LOC10272	0	0.013
NM_0013	chr3	15413671	15427547	-	1154	1	METTL6|-|	10.994	12.332
NM_0212	chr19	49642125	49658649	+	4300	1	SCAF1|SR	38.333	39.333
NM_1818	chr14	50872160	50904970	+	1464	1	ABHD12B|	0.106	0.037
NM_0012	chr13	48488963	48533256	-	2977	1	RCBTB2|C	6.432	5.99
NR_03998	chr1	134773	140566	-	5471	1	LOC72973	0.038	0.168
NR_04651	chr21	14582202	14598303	+	835	1	SAMSN1-A	0	0
NM_0022	chr15	40405485	40421313	+	4651	1	IVD|ACAD	10.953	10.134
NR_00157	chrY	9610721	9615153	-	314	1	RBMY3AP|	0	0
NM_0013	chr11	64230432	64234281	+	1334	1	DNAJC4|D	30.89	24.714
NM_0057	chr2	71130314	71150102	+	2472	1	MPHOSPH	5.2	5.973
NM_0011	chr14	20428811	20435642	-	1857	1	KLHL33|-|	0	0.007
NR_03389	chr8	65527008	65562666	-	2515	1	LINC0129	0.031	0.632
NM_0325	chr17	5179536	5191883	-	4873	1	ZNF594|-|	2.207	2.774
NM_0148	chr3	36985866	36993304	-	7436	1	EPM2AIP1	5.627	7.976
NM_0012	chr2	1.91E+08	1.91E+08	-	2749	1	STAT4|SLE	1.135	0.623
NM_0059	chrX	71096149	71103534	+	3343	1	FOXO4|AF	5.646	7.146
NR_03040	chrX	1.52E+08	1.52E+08	-	108	1	MIR767|M	0	0
NR_04982	chr18	13459947	13460026	+	79	1	MIR5190|	0	0
NM_0050	chr11	82822941	82900603	-	3646	1	PRCP|HUN	15.316	18.37
NM_1783	chr20	3043622	3045745	+	396	1	GNRH2|Gr	0.163	0.242
NM_0144	chrX	84058346	84187935	-	8181	1	RPS6KA6|	0.734	0.703
NM_0143	chr15	63907036	64046322	-	2614	1	DAPK2|DR	0.247	0.796
NR_03610	chr7	19705358	19705436	-	78	1	MIR3146|	0	0.176
NM_0040	chr11	77066945	77126152	+	4375	1	CAPN5|AD	15.982	23.44
NM_0014	chr1	8861000	8879092	-	2173	1	ENO1|ENO	930.912	414.888
NM_1524	chr9	1.37E+08	1.37E+08	+	2327	1	FAM69B|C	44.892	47.089
NM_0029	chr10	47348371	47357875	+	4270	1	RBP3|D10	0.045	0.138
NR_03834	chr20	44963799	44966458	-	2113	1	STK4-AS1|	0.146	0.078
NM_0329	chr7	1.03E+08	1.03E+08	-	1976	1	POLR2J2|H	2.382	2.662
NM_0010	chr19	15087066	15088133	+	1066	1	OR1I1|OR	0	0
NM_0012	chr8	26578405	26658177	+	4264	1	DPYSL2|CF	236.881	201.308

NM_0010(	chr2	63892533	64019080	-	3933	1	VPS54|HC	3.321	4.729
NR_12204	chr22	46914092	46916042	-	1950	1	TBC1D22A	0.013	0
NM_0012{	chr21	29092254	29175889	+	2057	1	MAP3K7CL	0.15	0.073
NM_0012	chr19	50505997	50511159	-	835	1	JOSD2|SBI	11.195	9.777
NM_0012	chr2	1.31E+08	1.31E+08	+	3260	1	POTEJ|PO	0	0.013
NM_1734	chr16	20854925	20900358	-	6131	1	DCUN1D3	2.593	2.898
NR_05198	chr15	1.02E+08	1.02E+08	-	1485	1	DDX11L9|	0	0
NR_02939	chr19	37265939	37269010	-	2342	1	LOC28441	0.022	0.018
NM_1784	chr19	49422414	49423441	-	455	1	PTH2|TIP3	9.238	0.391
NM_0155	chr1	6101787	6180134	-	9619	1	CHD5|CHD	5.331	10.647
NM_0059(	chr17	10492310	10518542	-	5978	1	MYH1|HEI	0	0
NM_0013(	chr20	59300427	59325992	+	2584	1	EDN3|ET-3	0	0
NM_0010(	chr10	97589693	97590934	+	1239	1	C10orf62|	0	0.011
NM_0058	chr1	1.5E+08	1.5E+08	-	1985	1	SF3B4|AFI	41.473	42.782
NM_0011	chr6	99520994	99533183	+	1920	1	TSTD3|-|-	0.598	0.385
NR_07340	chr8	6950726	6951599	-	283	1	DEFA8P|D	0	0
NM_1524	chr8	64580238	64583634	+	3394	1	BHLHE22|	0.942	66.556
NM_0327{	chr12	56118220	56122496	+	2144	1	ZC3H10|Z(	10.031	8.612
NR_00144	chr16	56617461	56618818	+	423	1	MT1L|MT1	0.03	0.065
NM_0055	chr15	73926458	73952141	+	2346	1	LOXL1|LOI	17.048	18.502
NR_00327	chr10	46415915	46416456	+	541	1	HNRNPA1I	0	0.025
NM_2073	chr3	1.9E+08	1.9E+08	-	1440	1	TMEM207	0.018	0
NR_10691	chrX	53405673	53405765	-	92	1	MIR6857|	5.466	3.126
NM_0155(	chr3	1.96E+08	1.96E+08	-	4719	1	UBXN7|UE	11.194	11.982
NR_03020	chr19	53722166	53722255	+	89	1	MIR520G|	0	0
NR_13278	chr2	1.97E+08	1.97E+08	-	115	2	SNORA105	11.997	10.243
NM_0156	chr1	52602371	52609051	+	1224	1	GPX7|CL6	1.928	1.343
NR_11022	chr2	1.96E+08	1.96E+08	+	1421	1	LOC10192	0	0
NM_0011	chr16	66754976	66801620	-	2425	1	TERB1|CCI	0	0.011
NR_02713	chr11	3218332	3223131	+	1788	1	MRGPRG-A	0.029	0
NM_1984{	chr8	1.44E+08	1.44E+08	-	5590	2	FAM83H|A	0	0
NR_00320	chr14	1.01E+08	1.01E+08	+	70	1	SNORD114	0	0
NR_03715	chr16	27447670	27453393	-	2611	1	IL21R-AS1	0.237	0.168
NR_13493	chr3	1.48E+08	1.48E+08	+	389	1	LINC02032	0.829	0.141
NR_03787	chr2	39437416	39601344	+	2511	1	LOC72873(	1.52	2.193
NM_0070	chr21	43529192	43659493	-	1888	1	HSF2BP|-|	0.765	0.646
NM_0012	chr12	93572526	93576745	+	2531	1	SOCS2|CIS	7.244	13.253
NM_0010	chr22	44668547	44737681	+	1716	1	PRR5|FLJ2	9.993	7.463
NM_0010	chr9	35906192	35907141	+	947	1	HRCT1|LG	0.109	0.043
NM_0056(	chr5	1.01E+08	1.01E+08	-	6315	1	ST8SIA4|P	3.904	1.779
NM_0012	chr1	1.55E+08	1.55E+08	-	2846	1	THBS3|TSI	45.485	21.412
NR_12066	chr5	1.42E+08	1.42E+08	+	587	1	LOC10192(	2.328	1.237
NM_0013	chr8	60206025	60281423	-	3899	1	CA8|CA-RI	0.43	1.584
NM_0013	chrX	54440375	54448027	+	4084	1	TSR2|DBA	25.099	23.705
NR_12636	chr2	1.08E+08	1.08E+08	+	606	1	GACAT1|L	0	0
NR_13511	chr1	56627791	56645318	-	487	1	LOC10192	0	0
NR_03882	chr11	1.2E+08	1.2E+08	-	2120	1	LOC64913	0	0
NR_02400	chr17	17776686	17779529	-	2843	1	SMCR5|N(	0.15	0.135
NM_0054	chr21	36385391	36416827	+	2263	1	CHAF1B|C	8.13	8.225
NM_0070(	chr12	69239537	69274358	+	6604	1	CPSF6|CFI	12.259	13.583

NM_0012:	chr6	57186994	57222314	-	4357	1	RAB23|HS	2.145	2.543
NM_1707(	chr6_GL0C	1325426	1331004	-	1962.4	8	RNF39|HZ	0.026	0
NR_11051	chr22	41185215	41197501	-	1374	1	EP300-AS1	0.488	1.037
NR_13499	chr12	1.28E+08	1.28E+08	-	438	1	LOC10537(	0	0
NM_0011⁄	chr1	2.07E+08	2.07E+08	-	2740	1	FCMR|FAI	0.009	0.025
NM_0013:	chr1	15941869	15976132	-	2527	1	ZBTB17|M	24.685	28.863
NM_0012(	chr11	46396412	46594069	-	4920	1	AMBRA1|I	21.384	25.326
NR_12118	chr9	77447649	77526762	+	698	1	GNA14-AS	0	0
NM_0146:	chr1	16921950	16972979	+	6617	1	CROCC|RC	7.223	4.819
NR_04635	chr8	12173454	12177267	-	785	1	FAM90A2F	0.066	0.14
NM_0012⁄	chr14	51860304	51969800	+	3840	1	GNG2|-|-|	121.87	132.401
NM_0013:	chr9	33384950	33402682	-	1233	1	AQP7|AQF	0	0
NM_0314:	chr12	57455291	57458013	+	2460	1	INHBE|-|-	1.064	0.34
NR_03037	chr19	40282543	40282641	-	98	1	MIR641|M	7.499	11.041
NR_03794	chr11	62690262	62727384	-	3990	1	HNRNPUL:	153.1	137.238
NR_13393	chr7	1.43E+08	1.43E+08	-	599	1	LOC10537.	0.603	0.297
NR_11039	chrX	1.19E+08	1.19E+08	+	2905	1	LOC10192:	0.027	0.009
NM_0011:	chr12	53326576	53336383	-	3146	1	SP7|OI11|	0.029	0.122
NM_0011(	chr3	1.71E+08	1.71E+08	-	9774	1	TNIK|MRT	8.213	10.984
NM_0012!	chr1	1.14E+08	1.14E+08	-	3284	1	AP4B1|BE	8.685	9.176
NM_0246:	chr16	23603162	23641357	-	4043	1	PALB2|FAI	2.641	3.239
NR_00299	chr1	2.2E+08	2.2E+08	-	128	1	SNORA36E	0	0
NM_0040{	chr14	30874535	30890616	+	2544	1	COCH|CO(	3.73	3.037
NR_02715	chr12	98512973	98516226	-	3159	1	TMPO-AS1	4.265	2.909
NM_0012:	chr13	1.14E+08	1.14E+08	+	1597	1	LINC00452	0	0.009
NM_0012(	chr1	1.7E+08	1.7E+08	-	2844	1	KIFAP3|FL.	53.995	52.635
NR_10806	chr10	17214239	17229985	-	1872	1	VIM-AS1|-	121.166	113.527
NM_0021:	chr6	32659464	32666689	-	1633	1	HLA-DQB1	0	0
NM_0184⁄	chr3	9962680	10011248	-	2631	1	EMC3|POI	26.556	24.906
NM_0182:	chr2	74152598	74178967	-	4906	1	MOB1A|C:	5.982	6.77
NR_00260	chr17	7576811	7576958	+	147	1	SNORD10|	2.28	4.193
NM_0055!	chr12	52446651	52452126	-	2271	1	KRT6B|CK-	0	0.012
NM_0221(	chr16	17102324	17470881	-	9322	1	XYLT1|DB(	3.17	3.642
NR_00335	chr15	25243614	25243695	+	81	1	SNORD115	0	0
NM_0004(	chr19	45351391	45370587	-	2543	1	ERCC2|CO	30.568	33.184
NM_0010(	chr17	49598884	49678163	-	2904	1	SPOP|BTB	18.799	23.149
NM_0010!	chrX	49589529	49596808	+	502	1	GAGE2B|C	0	0
NM_0221⁄	chrX	1.01E+08	1.01E+08	+	1339	1	TNMD|BRI	0.443	1.033
NR_13014	chr17	16690325	16774999	+	5529	1	CCDC144A	0	0.005
NM_0010{	chr19	12043805	12052967	-	1756	1	ZNF878|-|	0.235	0.445
NM_0013:	chr15	90184920	90229660	+	3339	1	SEMA4B|S	13.222	14.115
NR_10692	chr20	1392900	1392961	-	61	1	MIR6869|I	0.423	1.123
NM_0012:	chr17	41265378	41265860	+	480.7	3	KRTAP9-6|	0	0
NM_0190(	chr1	1.53E+08	1.53E+08	+	691	1	CRCT1|C1(	0	0
NM_0147⁄	chr17	80135214	80147183	-	1720	1	EIF4A3|DD	79.617	71.591
NM_0071:	chr19	20077994	20121168	+	3747	1	ZNF90|HTI	0.175	0.475
NR_00307	chr7	22856613	22856686	+	73	1	SNORD93|	0	0
NM_0251:	chr13	46342000	46387500	-	3962	1	RUBCNL|C	0.228	2.119
NM_0012!	chr4	1.09E+08	1.09E+08	-	2459	1	COL25A1|.	0.409	0.312
NR_12046	chr12	1.09E+08	1.09E+08	-	609	1	LINC0148(	0	0

NM_1723(	chr14	64549902	64589379	+	1392	1	PPP1R36|(	0.167	0.098
NR_13501	chr15	81312671	81324183	-	1256	1	STARD5|-|	1.776	1.756
NM_0013:	chr3	1.09E+08	1.09E+08	+	1801	1	TRAT1|HSI	0.193	0.068
NM_0333!	chr11	1.1E+08	1.1E+08	+	8799	1	ZC3H12C|I	3.382	2.989
NM_0216:	chr10	1.22E+08	1.22E+08	+	3708	1	PLEKHA1|T	7.292	7.266
NM_0011(	chr8	1.04E+08	1.04E+08	+	6771	1	RIMS2|OB	3.237	1.475
NM_0011(	chr2	26562559	26579532	+	748	1	C2orf70|-|	0	0
NM_0133:	chr19	55675195	55695767	+	2394	1	EPN1|-|-|:	88.989	88.252
NM_0013:	chr2	1.19E+08	1.19E+08	-	679	1	C2orf76|A	9.381	7.787
NM_0140!	chr12	93467490	93503772	+	3131	1	MRPL42|H	10.39	10.735
NR_12032	chr15	93905405	94107938	-	3164	1	LINC01581	0.008	0
NM_0011:	chr1	2.13E+08	2.13E+08	+	4005	1	VASH2|-|-	12.407	10.072
NM_1383	chr14	20289141	20305994	-	1854	1	TTC5|Stra	25.196	26.013
NM_0011:	chr6	41934933	41941848	-	1863	1	CCND3|-|-	13.959	18.02
NM_1533:	chr20	87672	96573	+	661	1	DEFB125|I	0	0
NM_1385!	chr9	1.18E+08	1.18E+08	+	5656	1	TLR4|ARM	0.062	0.501
NR_03615	chr17	48724408	48724475	-	67	1	MIR3185|-	0	0
NM_0161	chr5	1.16E+08	1.16E+08	+	1478	1	COMMD1(	5.583	7.349
NR_03982	chr10	1.19E+08	1.19E+08	+	71	1	MIR4681|-	0	0
NM_0157(	chr22	42508335	42519823	-	5446	1	RRP7A|BK	10.966	12.231
NM_0071:	chr9	35056068	35072742	-	3840	1	VCP|ALS1	132.947	145.909
NM_0010(	chr4	74158108	74303097	+	2386	1	MTHFD2L|	1.173	1.395
NR_04985	chr8	1.02E+08	1.02E+08	+	83	1	MIR5680|-	0	0
NR_13300	chr4	99336492	99353045	-	1705	1	ADH1C|AD	0	0
NM_0011:	chr19	44643798	44659094	+	1635	1	PVR|CD15	6.679	9.802
NM_0546(	chr11	65044823	65058537	-	2667	1	NAALADL1	0.179	0.159
NR_03984	chr12	1.21E+08	1.21E+08	+	73	1	MIR4700|	11.657	8.631
NR_10501	chr5	86746818	86749772	+	756	1	LINC0205!	0	0
NR_10691	chr1	2.06E+08	2.06E+08	+	61	1	MIR6769B	0	0
NR_10671	chr2	85783600	85783677	-	77	1	MIR6071|I	0.335	0.178
NM_0122!	chr7	1.49E+08	1.49E+08	+	2803	1	ZNF212|C:	8.57	10.516
NR_12051	chr7	35754964	35800625	-	712	1	SEPT7-AS1	1.376	1.347
NM_0011!	chr7	31052461	31111479	+	6412	1	ADCYAP1R	25.664	31.372
NM_0025!	chr17	3896591	3916666	-	2850	1	P2RX1|P2)	0.018	0
NM_0052!	chr18	22169437	22202528	+	3767	1	GATA6|-|-	0.017	0.029
NM_1456!	chr18	47022287	47024163	-	1874	2	TCEB3C|H:	0	0
NM_0013:	chr1	94168907	94247760	-	8715	1	ARHGAP2!	0.833	0.511
NR_13490	chr19	11203628	11216168	+	566	1	LOC10537.	0.342	0.411
NM_0029(	chr1	1.53E+08	1.53E+08	+	572	1	S100A9|6(	0	0
NM_0220!	chrX	1.03E+08	1.03E+08	-	1874	1	NXF3|-|-|)	0.007	0.007
NM_0182!	chr11	43358885	43494933	+	4499	1	TTC17|-|-|	18.144	19.658
NM_0020:	chr19	5865826	5870540	-	1984	1	FUT5|FUC	0.156	0.587
NM_0013:	chrX	1.17E+08	1.17E+08	-	648	1	LOC10012(	0	0
NM_0529:	chr8	51817575	51899186	-	4194	1	PCMTD1|-	3.185	4.363
NR_04666	chr3	54874605	54901255	-	2071	1	CACNA2D3	0.305	0.443
NM_0036!	chr12	10699077	10723354	-	1962	1	YBX3|CSD/	7.012	3.847
NM_0177:	chr1	93448131	93554661	+	4023	1	FNBP1L|C:	53.282	62.139
NM_0322!	chr16	684622	690424	-	3258	1	WDR24|C:	11.354	9.951
NM_0011	chr19	57869379	57889074	-	6278	1	ZNF814|-|	2.05	2.496
NR_02338	chr16	14822432	14824702	-	704	2	ABCC6P2|-	0	0

NM_0042:	chrX	40649543	40735552	-	6735	1	MED14|CF	6.49	6.988
NM_0013:	chr10	98416198	98446963	-	3541	1	HPS1|BLO	3.004	3.439
NM_0011(	chrX	1.03E+08	1.03E+08	-	822	1	BEX2|BEX:	126.331	160.782
NR_04511	chr5	1.32E+08	1.32E+08	+	801	1	C5orf56|-|	0.612	1.129
NM_0049:	chr1	1.11E+08	1.11E+08	-	2245	1	KCNA2|EIE	8.282	3.874
NM_0017:	chrX	1.53E+08	1.54E+08	+	2442	1	BGN|DSPG	0.053	0.045
NM_0012{	chr9	34613545	34620523	-	879	1	DCTN3|DC	162.056	143.717
NM_0013:	chr14	88562909	88613509	+	4061	1	ZC3H14|M	10.795	11.41
NM_0010(	chr2	96123850	96138436	-	1286	1	ASTL|SAS1	0	0.011
NM_0121:	chr6	3849366	3851320	+	1649	1	FAM50B|D	0	0.075
NM_0012{	chr15	70047791	70094785	-	4455	1	TLE3|ESG|	22.696	27.588
NR_03652	chr17	75271299	75273895	+	2596	1	LOC10028	2.429	2.416
NR_10684	chr17	81693757	81693869	+	112	1	MIR6786|	4.95	2.935
NM_1785	chr1	1917590	1919301	-	951	1	TMEM52|	3.01	3.111
NM_0012!	chr1	13196188	13201409	-	2157	2	PRAMEF13	0	0
NM_0166:	chr16	3020312	3022382	+	1026	1	TNFRSF12/	7.93	4.699
NM_0011(	chr5	1.2E+08	1.2E+08	+	1228	1	FAM170A|	0	0.022
NM_1984{	chr3	1.89E+08	1.89E+08	+	3649	1	TPRG1|FA	0	0.015
NR_13506	chr11	1.16E+08	1.16E+08	-	2453	1	LOC10192	0	0.006
NM_0211:	chr18	59899960	59904306	+	1932	1	PMAIP1|A	1.849	0.9
NM_0030{	chr14	61762357	61796428	+	2644	1	SNAPC1|P	1.273	1.44
NM_0011:	chr10	1.03E+08	1.03E+08	+	2428	1	BORCS7|C	4.593	4.237
NM_0010:	chr16	30569701	30572407	-	1131	1	ZNF688|-|	15.584	11.214
NM_1456	chr2	86199433	86213354	+	1492	1	MRPL35|L	11.303	10.144
NR_04626	chr4	1.86E+08	1.86E+08	-	2458	1	FLJ38576|	0.257	0.34
NR_03643	chr10	30692274	30717013	+	1673	1	SVILP1|-|-	0	0
NM_0012{	chr19	6135633	6193103	+	2482	1	ACSBG2|B	0.104	0.177
NM_0190(	chrX	1.02E+08	1.02E+08	-	1843	1	ARMCX6|(	15.566	12.998
NR_12584	chr6	53628380	53631394	+	563	1	LINC01564	0.069	0
NM_0051!	chr15	50424377	50501083	+	5533	1	USP8|Hum	2.852	3.696
NR_03156	chrX	63786002	63786087	-	85	1	MIR1468|	0	0
NR_02695	chr19	19756372	19776413	-	2368	1	LINC00663	3.087	3.083
NM_0010:	chr13	30202630	30307054	-	7499	1	KATNAL1|	7.644	5.227
NR_03171	chr15	96333261	96333307	+	46	1	MIR1469|	0.561	0
NM_0312	chr19	43064210	43082741	-	1604	1	PSG2|CEA	0	0
NR_02670	chr5	1.41E+08	1.41E+08	+	87	1	VTRNA1-2	0	0
NM_0151!	chr9	504695	746106	+	5059	1	KANK1|AN	2.419	3.011
NM_0210!	chr6	27132316	27132796	-	478	1	HIST1H2BJ	1.268	1.118
NM_0000:	chr4	1.77E+08	1.77E+08	-	2091	1	AGA|AGU	2.88	2.004
NM_0207!	chr3_KI27(	61318	73536	+	2008.6	8	MUC20|M	0.109	0.143
NM_0207{	chr14	52430590	52552583	-	4618	1	TXNDC16|	5.148	5.816
NR_11076	chr18	12739486	12749422	-	572	1	LINC01882	0	0
NM_0011:	chr1	1.52E+08	1.52E+08	+	3022	1	TUFT1|-|-	8.635	13.049
NR_03996	chr4	7310450	7310524	+	74	1	MIR4798|	0	0
NR_03608	chr2	2.19E+08	2.19E+08	-	62	1	MIR3131|	0	0
NM_0011!	chr17	17246624	17280698	-	1802	1	COPS3|CSI	53.284	67.527
NR_10413	chr16	86225580	86286247	-	730	1	LINC01081	0	0
NR_00154	chrY	6242446	6243610	-	509	2	TTTY23|N(	0	0
NR_13505	chr12	39626167	39908300	+	3093	1	C12orf40|	0.042	0.013
NM_0319(	chr1	1.5E+08	1.5E+08	+	1099	1	MRPS21|N	95.956	84.65

NM_1304	chr21	45405137	45513720	+	5382	1	COL18A1|	18.564	7.172
NR_13667	chr16	11679080	11742878	-	2095	1	TXNDC11|	10.746	11.173
NM_1984	chr20	56369389	56392337	-	2145	1	AURKA|AI	2.885	3.55
NM_1986	chr21	44612079	44612954	+	873	1	KRTAP10-8	0	0
NM_0252	chr17	42950526	42980003	-	1771	1	PTGES3L-A	29.311	27.727
NM_0143	chr11	1.25E+08	1.25E+08	-	1173	1	VSIG2|221	0.714	2.184
NM_0581	chr11	5596101	5612958	+	3221	1	TRIM6|RN	0.02	0.102
NR_02966	chr18	21829004	21829088	-	84	1	MIR1-2|M	0	0
NM_0140	chr5	1.23E+08	1.23E+08	+	2128	1	SNX24|PR	6.847	4.879
NM_0013	chr16	57758217	57775108	-	2992	1	KIFC3|-|-|	13.187	14.407
NM_0154	chr2	33583661	33599362	-	2803	1	FAM98A|-	7.424	10.096
NM_0070	chr7	49937428	50093264	-	1216	1	ZPBP|ZPBI	0	0
NR_03653	chr2	1.3E+08	1.3E+08	-	2454	1	RAB6C-AS1	0.053	0.156
NM_0053	chr4	1.08E+08	1.08E+08	+	1972	1	HADH|HAI	18.444	26.776
NM_0013	chr7	44577895	44582295	-	2343	1	TMED4|EF	45.196	38.32
NM_0334	chr20	760080	768584	-	2688	1	SLC52A3|E	0	0
NR_10809	chr6	14280127	14285454	-	3694	1	LINC01108	0.007	0.019
NR_02692	chr2	1.31E+08	1.32E+08	+	3770	1	LOC15077	8.341	8.883
NM_0239	chr3	1.33E+08	1.33E+08	+	3822	1	TMEM108	5.013	14.202
NR_04598	chr20	46456493	46457643	-	1150	1	ZNF663P|Z	0.28	0.596
NM_0180	chr2	2.16E+08	2.16E+08	-	3206	1	MREG|DSU	3.76	2.602
NR_13365	chr11	2140512	2148666	+	1954	1	IGF2-AS|IG	0.376	0.056
NM_0012	chr11	1.12E+08	1.12E+08	+	2765	1	BCO2|B-D	0.671	0.52
NM_0020	chr9	1.25E+08	1.25E+08	-	4857	1	GOLGA1|g	7.162	5.756
NM_0010	chr1	29119425	29123909	-	2474	1	TMEM200	8.969	4.158
NR_11009	chr20	1633508	1648473	+	2793	1	SIRPG-AS1	0	0
NM_0806	chr6_GL0C	2962555	2979657	+	6881	7	PRRC2A|B	0.465	0.362
NM_0143	chr9	1.09E+08	1.09E+08	-	8191	1	FRRS1L|C9	3.613	4.058
NM_0182	chr17	532293	714856	-	2674	1	VPS53|HC	9.417	11.73
NR_13509	chr11	96508425	96514750	+	402	1	LOC10536	0	0
NM_0011	chr7	1.01E+08	1.01E+08	-	2123	1	CLDN15|-|	7.379	8.013
NR_10374	chr1	1.13E+08	1.13E+08	+	735	1	SLC16A1-A	0.824	1.025
NM_0010	chr20	64073181	64079971	-	1485	1	RGS19|GA	6.139	5.008
NM_0013	chr2	85354720	85391752	+	2264	1	ELMOD3|I	11.265	12.608
NR_10504	chr5	1.34E+08	1.34E+08	-	702	1	LOC10254	0	0.02
NM_0161	chr17	16415542	16437003	+	2791	1	TRPV2|VR	0.065	0.113
NM_0223	chr7	8113185	8262052	-	2241	1	ICA1|ICA6	11.829	7.481
NR_03967	chr6	1.57E+08	1.57E+08	-	53	1	MIR4466|	0	0
NM_0010	chr7	4682299	4771443	+	11180	1	FOXK1|FO	7.896	9.489
NM_0011	chr3	1.3E+08	1.3E+08	-	5478	1	TMCC1|-|-	13.67	12.974
NM_0012	chr19	49665142	49673916	+	1432	1	BCL2L12|-	4.925	3.128
NM_0036	chr11	45647689	45665656	-	3934	1	CHST1|C6	13.801	14.759
NM_0218	chr4	1.23E+08	1.23E+08	-	610	1	IL21|CVID	0	0
NR_00222	chr17	30975335	30975999	+	664	1	DPRXP4|-|	0	0
NR_13071	chr11	59942879	59995845	+	1154	1	OOSP1|-|-	0	0
NM_0010	chr16	805443	813861	+	1205	1	PRR25|gs6	0.096	0.296
NM_0018	chr4	1.65E+08	1.65E+08	+	2637	1	CPE|CPH|-	171.654	253.169
NM_0211	chr1	24323040	24355318	+	2682	1	GRHL3|SO	0.034	0.061
NM_0010	chr2	1.31E+08	1.31E+08	+	1152	2	CFC1B|-|-	0	0.357
NR_13093	chr5	1.72E+08	1.72E+08	+	1800	1	LOC10028	0.029	0.068

NR_03023	chrX	1.47E+08	1.47E+08	-	123	1	MIR506|M	0	0
NM_0179(	chr2	1.27E+08	1.28E+08	-	2984	1	IWS1|-|-|:	22.948	21.872
NM_0320	chr18	2847030	2914092	+	3999	1	EMILIN2|E	1.238	0.099
NM_1334	chrX	47571901	47619857	-	3157	1	SYN1|SYN	49.046	73.778
NM_0143	chr17	4671384	4704337	-	3655	1	PELP1|MN	30.814	33.472
NM_1308	chr5	1.35E+08	1.35E+08	-	3132	1	DCANP1|C	0.004	0
NM_0010	chr9	1.04E+08	1.04E+08	+	5959	1	SMC2|CAF	0.775	1.6
NM_0056(	chr1	1.97E+08	1.97E+08	+	1065	1	CFHR2|CFI	0	0
NM_0218	chr2	27051623	27070622	+	3220	1	AGBL5|CC	43.341	37.722
NM_1983	chr14	88824153	88877996	+	2194	1	TTC8|BBS8	9.338	8.49
NR_11095	chr8	79769380	79871737	-	2112	1	LOC10192	0.055	0.026
NM_0013(	chr9	97412020	97496123	+	3531	1	TDRD7|CA	3.6	2.874
NM_0012	chr3	1.38E+08	1.38E+08	+	3795	1	MRAS|M-I	36.652	43.304
NR_03607	chr2	69103682	69103755	+	73	1	MIR3126|	0	0
NM_0010	chr4	37590800	37686377	-	1318	1	RELL1|-|-|	1.321	1.486
NM_0011	chr18	14748240	14852738	+	4579	1	ANKRD30E	0	0.024
NM_0183(	chr1	1.14E+08	1.14E+08	-	6612	1	RSBN1|RO	4.871	4.928
NM_0012	chr6_GL0C	2742492	2758910	+	2399.2	8	MICB|PER	0	0.017
NM_0012	chr6_GL0C	2106566	2124393	+	2074	7	ATAT1|C6	0	0
NR_10982	chr5	93409356	93581320	-	2653	1	NR2F1-AS1	11.664	10.145
NR_01537	chr9	2535655	2622373	-	880	1	VLDLR-AS1	2.711	2.537
NR_13276	chr1	1.56E+08	1.56E+08	-	147	1	SCARNA26	0.263	0.093
NM_0011	chr2	2.2E+08	2.2E+08	-	3549	1	OBSL1|-|-	28.421	17.788
NR_03061	chr17	12081899	12081996	+	97	1	MIR744|M	0	0
NM_0011	chr11	61402648	61429992	-	3573	1	CPSF7|CFI	54.006	61.167
NR_04704	chr13	21872796	21878160	-	451	1	LINC00424	0	0
NM_0073	chr12	76025447	76031776	-	5909	1	PHLDA1|D	17.72	28.333
NR_10504	chr5	1.26E+08	1.26E+08	-	495	1	LOC10192	0	0
NM_0046	chr17	75087941	75106160	+	1942	1	SLC16A5|I	0.04	0.014
NM_0011	chr2	63589151	63607196	+	1434	1	MDH1|HE	172.354	195.779
NM_0036	chr12	1.23E+08	1.23E+08	+	2712	1	DENR|DRF	12.532	13.444
NM_1489(	chr1	1203508	1206709	-	1172	1	TNFRSF18	0.297	0.047
NM_0189	chr5	1.41E+08	1.41E+08	+	3960	1	PCDHB14|	4.565	4.036
NM_0161(	chr5	1.35E+08	1.35E+08	-	6523	1	SAR1B|AN	4.542	4.626
NM_0010(	chr11	56988914	56989843	+	928	1	OR5AK2|-	0.028	0
NM_1391	chr6	1.5E+08	1.5E+08	-	2519	1	PPIL4|HDC	7.816	6.916
NM_0012(	chr18	79681430	79754510	+	2772.5	2	CTDP1|CC	3.73	3.354
NR_04988	chr21	42950928	42951014	-	86	1	MIR5692B	0	0
NM_0011	chr13	1.13E+08	1.13E+08	-	2129	1	PCID2|F10	16.679	20.401
NM_0035	chr22	26525748	26590125	-	1885	1	TPST2|TAI	10.116	8.378
NM_0011	chr2	2.38E+08	2.38E+08	+	3578	1	LRRFIP1|F	11.701	9.716
NR_03613	chr14	1.01E+08	1.01E+08	+	81	1	MIR323B|	0	0
NM_0331	chr17	42122738	42123353	+	613	1	HSPB9|CT	0.252	0.134
NM_0013	chrY	9398421	9401217	+	1163.5	4	TSPY10|-|	0	0
NM_1530	chr17	5078459	5096374	+	4991	1	ZFP3|ZNF	0.106	0.639
NM_0010(	chrX	74732856	74925452	-	11711	1	KIAA2022|	1.105	4.991
NM_0065	chrX	48521776	48528716	+	1150	1	EBP|CDPX	59.658	51.024
NR_03409	chr7	28180457	28241377	+	1190	1	JAZF1-AS1	0.704	0.863
NM_0252	chr3	52254422	52278643	-	4281	1	WDR82|M	98.23	123.763
NM_1489(	chr2	42762499	42764261	-	1760	1	OXER1|GP	0.088	0.078

NR_13426	chr5	37249046	37250646	+	424	1	LOC10537	0.182	0.775
NM_0011	chr9	1.3E+08	1.3E+08	+	4316	1	USP20|LSF	22.005	24.328
NR_02678	chr8	12362019	12411001	+	770	1	FAM66A|-	0.469	0.694
NM_0012	chr6	1.7E+08	1.7E+08	+	1906	2	ERMARD|(	0	0
NM_0012	chr1	37611350	37634923	-	2901	1	RSPO1|CR	4.209	0.118
NM_0010	chr7	1.01E+08	1.01E+08	-	2956	1	PLOD3|LH	10.359	7.043
NM_1817	chr5	1.51E+08	1.51E+08	-	3409	1	SLC36A2|F	0	0
NM_0050	chr19	8311350	8321413	-	546	1	NDUFA7|E	85.083	56.168
NR_04666	chr13	1.13E+08	1.13E+08	-	2228	1	ATP11A-AS	0.023	0.006
NM_0024	chr12	80707629	80709477	+	1332	1	MYF6|CNN	0	0
NM_0013	chr14	36298558	36320676	-	1540	1	MBIP|-|-|	7.167	11.064
NR_01541	chr6	21666444	22194387	+	1877	1	CASC15|LI	5.646	5.371
NM_0190	chr5	65665928	65722114	-	5440	1	SGTB|SGT	3.107	3.57
NR_03994	chr2	1.27E+08	1.27E+08	-	81	1	MIR4783|-	0	0
NR_00326	chr7	76470020	76475000	-	4980	1	FDPSP2|FD	0	0.019
NR_10500	chr5	72574075	72660669	-	636	1	LINC02056	0	0.108
NR_10679	chr1	2.02E+08	2.02E+08	+	74	1	MIR6739|	3.833	5.553
NM_0207	chr19	49591643	49626439	+	6939	1	PRR12|KIA	19.808	18.776
NM_0012	chr10	97879626	98030828	-	2334	1	CRTAC1|A	0.226	1.338
NR_03022	chrX	1.35E+08	1.35E+08	-	99	1	MIR450A2	0	0
NM_0026	chr5	1.5E+08	1.5E+08	-	5689	1	PDGFRB|C	0.675	1.582
NM_0012	chrX	1.55E+08	1.55E+08	-	2686	1	CLIC2|CLIC	0.326	0.474
NR_03745	chr12	95309923	95309984	+	61	1	MIR3685|-	1.268	1.347
NM_0133	chr14	77274956	77320882	-	4851	1	POMT2|LC	13.08	10.752
NM_1748	chr2	99284238	99301176	-	981	1	LYG1|SALV	1.538	2.15
NM_0012	chrX	83861126	83886700	+	374	1	CYLC1|CYC	0	0
NR_12056	chr11	78423983	78429836	-	1637	1	LOC10192	0.268	0.218
NM_0124	chr2	1.97E+08	1.97E+08	-	4311	1	SF3B1|Hsh	65.927	78.557
NR_07300	chr7	1.08E+08	1.08E+08	+	2005	1	DUS4L|DU	2.945	2.992
NM_0178	chr15	68206992	68229742	-	2233	1	CLN6|CLN	4.654	4.048
NM_0065	chr3	45943481	45948353	+	1949	1	CXCR6|BO	0	0.028
NM_1833	chr15	97960703	97973838	+	4057	1	ARRDC4|-	11.708	15.817
NM_1734	chr5	40998020	41071342	-	5236	1	MROH2B|	0	0
NM_0025	chr3	1.53E+08	1.53E+08	+	6744	1	P2RY1|P2Y	0.92	0.471
NR_13627	chr8_KI27	154555	156721	-	1498	2	LOC10192	0	0
NM_1749	chr5	83052846	83077453	-	4581	1	TMEM167	15.981	15.174
NR_02446	chr2	74385718	74393881	+	2140	1	DCTN1-AS	0.319	0.371
NR_11198	chr1	12716115	12728759	+	3829	1	AADACL3|	0	0
NR_10371	chr22	17777290	17779481	+	2191	1	LINC00528	0.188	0.244
NM_0210	chr17	36194870	36196758	-	776.8	5	CCL3L1|46	0	0
NM_0066	chrX	1.24E+08	1.24E+08	+	5967	1	STAG2|SA	5.743	9.368
NM_0010	chr3	1.37E+08	1.37E+08	+	1642	1	SLC35G2|T	7.64	9.176
NR_11080	chr17	48444833	48466040	+	2259	1	LOC10192	0	0
NR_10981	chr20	35285251	35285772	+	366	1	FAM83C-A	0.247	0.075
NM_0011	chr17	5499427	5500999	-	1489	1	LOC72839	203.135	156.922
NM_0183	chr4	1.63E+08	1.64E+08	+	1909	1	TMA16|C4	2.81	2.461
NM_0010	chr11_JH1	193979	194908	+	928	1	OR8U9|-|-	0	0
NM_0219	chr6_GL0	867409	898284	-	4277.9	7	GABBR1|G	0.018	0.003
NR_04535	chr17	81977550	82017406	+	1772	1	ASPSCR1|	15.666	13.944
NR_03386	chr8	72202752	72251634	-	2606	1	LOC39223	0	0.032

NM_0056(	chr5	1.13E+08	1.13E+08	-	3109	1	REEP5|C5(	20.28	21.922
NM_0011!	chr16	66579448	66588274	+	898	1	CMTM2|C	0.158	0.153
NM_0011:	chr10	89213569	89251908	-	2650	1	LIPA|CESD	11.405	9.557
NR_03771	chr2	61824848	61854143	-	3630	1	FAM161A|	2.454	1.883
NM_0011:	chr7	76424955	76442071	+	1290	1	ZP3|ZP3A|	0.31	0.34
NM_0808(	chr17	44170706	44179083	+	2132	1	ASB16|-|-	2.038	2.416
NM_0246(	chr1	45002548	45011355	-	3605	1	HECTD3|-|	20.504	25.491
NM_0209!	chr14_KI2	1288874	1342407	+	894	2	PPP4R4|C	0	0
NR_13663	chr7	66904120	66906297	+	2177	1	LOC64479	1.558	1.302
NM_0122:	chr17	42402449	42423320	-	3630	1	PTRF|CAV	3.69	3.811
NR_02691	chr1	2.12E+08	2.12E+08	-	565	1	RPL21P28	0.274	0.267
NM_0000:	chr1	2.31E+08	2.31E+08	-	2577	1	AGT|ANHU	0.215	1.106
NR_03828	chr3	1.97E+08	1.97E+08	+	1699	1	DLG1-AS1|	0.099	0
NM_0151!	chr3	16315845	16513715	-	2990	1	RFTN1|MI	2.311	7.82
NR_12871	chr2	2.32E+08	2.32E+08	+	81	4	MIR1244-4	10.506	14.712
NM_0013(	chr2	1.54E+08	1.54E+08	+	5355	1	GALNT13|	1.847	1.68
NM_0063:	chr19	3572945	3579083	+	1572	1	HMG20B|	46.751	43.2
NM_0184(	chr2	1.43E+08	1.44E+08	+	1743	1	ARHGAP15	2.049	11.921
NM_0012:	chr2	27582969	27623215	+	3528	1	ZNF512|-|	45.423	22.735
NM_0207	chr5	1.25E+08	1.25E+08	-	5634	1	ZNF608|N	25.27	27.02
NM_0013	chr10	59251169	59362594	-	2159	1	FAM13C|F	8.379	10.772
NM_0156(	chr7	1.51E+08	1.51E+08	+	1437	1	GIMAP2|H	0	0
NM_1814:	chr12	11185993	11186937	-	943	2	TAS2R42|	0	0
NM_0143:	chr1	2.42E+08	2.42E+08	-	2626	1	OPN3|ECP	5.642	4.741
NM_0012	chr17	52985520	52987652	+	829	1	C17orf112	0	0
NM_1452!	chr1	2.29E+08	2.29E+08	-	5115	1	CCSAP|C1(	5.863	16.257
NM_0011:	chr16	3135133	3142804	+	3257	1	ZNF213|C	20.031	23.697
NR_13450	chr20	29448517	29458181	+	1883	1	LOC10537	0.322	0.895
NR_10987	chr20	12934878	12936963	-	827	1	LOC10260	0	0
NM_0013:	chr7	1.37E+08	1.38E+08	-	6488	1	DGKI|DGK	4.567	3.713
NM_0181:	chr4	24998849	25030792	-	6329	1	LGI2|LGIL:	5.851	4.434
NR_00327	chr8	12127858	12129297	-	1439	1	LOC39219	0	0
NM_0058:	chr13	40729296	40771211	-	1330	1	MRPS31|I	15.172	14.511
NM_0011:	chr10	45615501	45672813	-	3359	1	ZFAND4|A	2.219	1.965
NR_02444	chr2	94575976	94587183	-	2024	1	LOC10013	0	0
NR_11065	chr16	47858581	47908431	+	2618	1	LINC02133	0.049	0.047
NM_0202!	chr7	1.35E+08	1.35E+08	+	1584	1	AKR1B10|	0.13	0.086
NR_03764	chr11	57712523	57740973	+	1518	1	TMX2|CGI	47.752	46.072
NR_02437	chr2	1.11E+08	1.11E+08	-	587	1	MIR4435-2	3.514	1.89
NM_0012:	chr12	32679200	32745650	+	4561	1	DNM1L|D	11.822	18.015
NM_0011:	chr1	41027199	41242143	-	3057	1	SCMH1|Sc	21.063	21.973
NM_1334	chr12	1.29E+08	1.3E+08	-	5766	1	TMEM132	0	0
NM_0012!	chr4	25747427	25863595	-	4349	1	SEL1L3|Se	6.226	15.423
NM_0012{	chr1	36155965	36180849	+	3451	1	MAP7D1|	99.987	119.518
NR_00333	chr15	25081286	25081379	+	93	1	SNORD116	0.416	0.147
NM_0182:	chr18	74496265	74523454	+	5059	1	CNDP2|CN	26.794	27.86
NR_03609	chr6	1.2E+08	1.2E+08	+	78	1	MIR3144|	0	0
NM_0012	chr9	83242990	83331961	-	1385	1	FRMD3|4.	2.979	3.076
NR_03390	chr4	1.86E+08	1.87E+08	-	1231	1	F11-AS1|-	0	0.011
NR_10406	chr10	37309186	37347028	+	648	1	LINC00993	0	0

NR_04657	chr12	2668500	2672220	-	607	1	CACNA1C-	1.699	3.091
NM_0055	chr18	59327824	59359276	-	4819	1	LMAN1|EF	6.729	5.028
NM_1942	chr12	1.19E+08	1.19E+08	+	8462	1	SRRM4|KI	26.444	23.851
NM_0335	chr22	50674642	50733212	+	7030	1	SHANK3|D	7.754	7.02
NM_0013	chr11	67023345	67050863	+	3308	1	SYT12|SYT	0.744	2.286
NR_03035	chr15	42199570	42199666	-	96	1	MIR627|M	0	0
NR_13532	chr13	29647693	29655475	+	436	1	LOC10272	0	0
NM_0011	chr10	1.18E+08	1.18E+08	-	2555	1	FAM204A|	11.475	10.947
NR_03039	chr14	1.01E+08	1.01E+08	+	77	1	MIR656|M	0	0
NR_04751	chr1	827591	859446	+	6770	1	LINC0112	3.902	3.227
NM_0013	chr3	15070069	15099157	-	6520	1	RBSN|Rab	16.226	18.317
NM_1815	chr2	74526648	74529897	-	1639	1	AUP1|-|-|	64.066	61.54
NM_0013	chr1	16124337	16156109	-	3900	1	EPHA2|AR	5.891	3.365
NM_0013	chr6	44223504	44234151	+	2168	1	SLC29A1|E	10.455	10.469
NM_0013	chr3	48292317	48301411	-	2957	1	NME6|IPI	7.639	5.216
NM_0014	chr6	53497341	53545129	-	3795	1	GCLC|GCL	3.404	2.772
NM_0011	chr3	1.41E+08	1.41E+08	+	4652	1	SLC25A36|	7.805	10.974
NR_13391	chr3	1.24E+08	1.24E+08	-	1988	1	ROPN1|CT	0	0
NM_0012	chr6	1.59E+08	1.59E+08	-	724	1	DYNLT1|C	214.951	221.517
NR_02447	chr20	62663027	62666621	-	1419	1	SLCO4A1-	0.863	0.647
NM_0010	chr11	48306223	48307152	+	928	1	OR4S1|OR	0	0
NR_04655	chr13	50882379	50910712	-	621	1	RNASEH2B	0	0.066
NR_02834	chr11	89993541	90002508	+	1722	2	TRIM53AP	0	0
NM_0011	chr2	1.21E+08	1.22E+08	-	7851	1	CLASP1|M	31.093	33.985
NM_0027	chr6	31164337	31170693	-	1413	7	POU5F1|C	0	0
NM_0011	chr16	30472662	30523185	+	4926	1	ITGAL|CD1	0.094	0.022
NR_03058	chrX	1.47E+08	1.47E+08	-	90	2	MIR509-2|	0	0
NM_0011	chr4	2041993	2043970	+	427	1	C4orf48|C	111.24	92.351
NM_1992	chr12	7711454	7717556	+	1070	1	DPPA3|ST	0	0
NR_12051	chr7	1.34E+08	1.34E+08	-	1392	1	LOC10192	0	0
NM_0054	chr2	1.13E+08	1.13E+08	+	3364	1	SLC20A1|	22.318	23.99
NR_10995	chr5	38693213	38845829	-	653	1	OSMR-AS1	0	0
NM_0165	chr12	7094550	7109278	-	3395	1	C1RL|C1R	1.139	2.146
NM_0037	chr5	1.41E+08	1.42E+08	+	4572	1	PCDHGB4|	20.257	97.617
NM_1817	chr12	1.09E+08	1.09E+08	-	2697	1	TMEM119	0.043	0.056
NM_0049	chr10	92451684	92574095	-	5869	1	IDE|INSUL	1.995	2.098
NM_0012	chr22	21567547	21624034	+	2927	1	UBE2L3|E	45.896	48.808
NM_0013	chr17	48548870	48575097	-	2690	1	HOXB3|H	0.01	2.75
NM_0061	chr16	2471499	2474145	+	1978	1	NTN3|NTN	0.821	0.789
NM_0013	chr13	27271177	27273690	+	823	1	RASL11A|-	1.755	1.881
NM_0013	chr11	1.24E+08	1.24E+08	+	7539	1	GRAMD1B	6.395	5.498
NR_03983	chr10	98431292	98431360	-	68	1	MIR4685|	0.379	0.403
NM_0013	chr18	69862956	69957176	-	2402	1	CD226|DN	0.021	0.074
NR_10692	chr16	56904264	56904353	+	89	1	MIR6863|	0	0
NR_13629	chr9	64639734	64700461	+	558	5	LOC10192	0	0
NM_1827	chr6	28503296	28515793	-	1705	1	GPX6|GPX	0	0
NM_0012	chr15	43371059	43407096	+	4162	1	TUBGCP4|	17.491	17.748
NM_0013	chr6	1.32E+08	1.33E+08	-	1310	1	STX7|-|-|	38.11	40.171
NM_0059	chr13	23730189	23889448	-	2408	1	MIPEP|CO	4.808	4.3
NM_1993	chr19	48170692	48197620	+	3582	1	C19orf68|	4.532	3.028

NM_0216	chr12	53299686	53307181	+	1206	1	C12orf10|	54.15	43.408
NM_0013	chr15	88898683	88913469	-	1885	1	MFGE8|B/	10.643	11.35
NM_0241	chr19	984328	994570	+	1539	1	WDR18|Ip	36.301	33.037
NR_11009	chr7	15688378	15696892	+	2096	1	MEOX2-AS	0	0.007
NR_13618	chr2	24793115	24822376	+	4194	1	CENPO|CE	13.112	12.384
NM_0178	chr1	36088852	36093932	+	1683	1	ADPRHL2|	29.005	30.055
NM_0010	chr7	81742691	81770198	-	1326	1	HGF|DFNE	0.039	0.083
NM_0024	chr9	32553526	32573184	-	855	1	NDUFB6|E	52.207	44.68
NM_0010	chr1	13222695	13226154	-	2120	2	PRAMEF18	0	0
NM_0010	chr11	71527267	71528164	+	895	1	KRTAP5-7|	0.058	0.107
NM_0011	chr2	1.6E+08	1.6E+08	-	8711	1	LY75-CD30	0.117	0.178
NM_0013	chr3	48517684	48561180	-	3489	1	PFKFB4|-|	17.716	12.861
NR_03825	chr13	40796986	40921750	-	4184	1	TPTE2P5|-	0.881	1.038
NM_0060	chr17	13494032	13601942	-	4195	1	HS3ST3A1	0.138	0.206
NM_0013	chr16	84648511	84667686	+	7364	1	KLHL36|C1	11.132	13.822
NR_04622	chr19	34892029	34898943	-	468	1	LINC00904	0	0
NM_0133	chr1	2.26E+08	2.26E+08	-	1762	1	PYCR2|HLI	24.843	17.553
NR_03017	chr17	7017911	7018022	-	111	1	MIR497|M	0	0
NM_0012	chr3	23917127	23920856	+	2087	1	RPL15|DB/	248.454	315.294
NR_02655	chr4	79827471	79863247	+	2089	1	PCAT4|GD	0	0.007
NR_04662	chr6	73134803	73143514	-	1096	1	KCNQ5-AS	0	0
NM_0013	chr1	44221070	44355279	-	1099	1	ERI3|PINT	88.976	94.67
NR_07297	chr20	10404780	10434239	-	1553	1	MKKS|BBS	17.667	17.886
NM_0016	chr11	1.26E+08	1.26E+08	-	1169	1	ACRV1|D1	0.673	1.511
NM_0013	chr3	1.7E+08	1.7E+08	+	2358	1	SAMD7|-|	0	0
NR_03973	chr15_GL3	190332	190425	-	93	9	MIR4509-3	0	0
NM_0156	chr20	25673195	25696904	-	4281	1	ZNF337|-|	12.547	15.853
NM_0055	chr1	2.01E+08	2.01E+08	-	2827	1	LAD1|Lad/	0.014	0
NM_0004	chr17	2593629	2685615	+	5601	1	PAFAH1B1	44.898	57.099
NM_0012	chr6	43172390	43181506	+	3383	1	SRF|MCM	14.925	15.827
NM_0804	chr18	12785478	12884335	-	1691	1	PTPN2|PTI	3.019	3.442
NM_0010	chr14	19920607	19921578	+	970	1	OR4K5|OF	0	0
NM_0026	chr2	87010465	87021850	-	3009	2	PLGLB2|PL	0.09	0.155
NR_03606	chr1	1.72E+08	1.72E+08	+	80	1	MIR3120|	0	0
NM_0059	chr4	1.03E+08	1.03E+08	-	3292	1	MANBA|M	3.928	3.653
NR_04607	chr8	23678693	23682921	-	2466	1	NKX3-1|B/	0.021	0.033
NM_0010	chr18	12947984	12987536	+	1872	1	SEH1L|SEC	10.834	15.058
NM_0324	chr19	42387019	42390292	-	522	1	CNFN|PLA	1.951	2.23
NM_1735	chr8	66870749	66922048	+	5079	1	MCMDC2|	1.655	1.578
NM_0147	chr7	1.44E+08	1.44E+08	-	5653	1	TCAF1|FAI	7.068	5.982
NM_0010	chr1	2.48E+08	2.48E+08	-	937	1	OR2M7|O	0	0
NR_02668	chr8	1.33E+08	1.33E+08	-	981	1	HPYR1|HP	0.026	0
NM_0012	chr22	40410281	40463440	-	4104	1	MKL1|BSA	17.634	22.258
NM_0003	chr20	45890144	45898819	+	3031	1	CTSA|GLB	11.936	10.909
NR_03986	chr15	49169070	49169153	-	83	1	MIR4716|	0	0
NM_0013	chr12	6943502	6946003	+	632	1	C12orf57|	292.959	267.243
NM_0309	chr1	1385711	1399338	-	3102	1	CCNL2|AN	30.405	28.211
NM_0148	chr11	72836745	73142098	-	4468	1	FCHSD2|N	13.182	18.351
NR_07299	chr15	30818036	30827783	+	633	2	HERC2P10	0	0
NM_0013	chr17	4940008	4945223	+	1458	1	RNF167|5	82.508	77.335

NR_03780	chr19	12131353	12156731	-	3407	1	ZNF625-ZN	4.178	4.318
NM_1530(	chr17	44004664	44009068	+	2063	1	NAGS|AGA	2.15	1.142
NR_10275	chr15	52180026	52205879	+	1517	1	CERNA1|-|	1.02	1.535
NM_1809	chr6	42984499	43012345	+	2713	1	PPP2R5D|	68.978	72.241
NM_0011	chr15	40217428	40277487	+	3784	1	BUB1B-PA	3.905	9.234
NM_0011	chr19	51379909	51387956	-	851	1	LIM2|CTR	0.061	0
NM_0070	chr19	10391133	10403595	-	1661	1	CDC37|P5	89.416	71.85
NM_0010	chr1	1.46E+08	1.46E+08	-	3608	1	ANKRD34A	2.384	1.735
NM_0011	chr1	37807801	37809454	+	1180	1	C1orf122|	65.604	63.354
NM_0012	chr6	1623800	2175991	-	1433	1	GMDS|GM	7.027	12.407
NM_0325	chr16_KI2	32588	39961	+	3697	2	GLIS2|NKL	0.474	0.456
NR_13492	chr19	11987617	12046275	+	665	1	LOC10192	6.961	5.891
NM_0003	chr8	76980258	77000288	-	4373	1	PEX2|PAF	7.076	5.663
NM_0180	chr3	1E+08	1.01E+08	+	1567	1	TMEM45A	6.805	3.951
NR_13555	chr2	10041553	10043401	-	560	1	LOC10192	2.947	1.981
NR_02688	chr11	61754029	61757664	-	932	1	DKFZP434	0.042	0.103
NM_0010	chr14	52553148	52637713	+	3879	1	GPR137C|	2.955	3.33
NM_0150	chr15	62647311	62844630	+	11591	1	TLN2|ILWI	5.035	7.947
NM_0011	chr10	79610939	79615443	+	2060	1	SFTPA1|C	0.013	0.033
NM_0059	chr16	56638666	56640088	+	392	1	MT1A|MT	0	0
NM_0011	chr15	48988639	49046563	-	7155	1	SECISBP2L	8.268	10.069
NM_0015	chr7	1.12E+08	1.12E+08	+	3287	1	IFRD1|PC4	27.556	29.578
NR_10236	chr8	1.41E+08	1.42E+08	-	2557.5	2	MROH5|-|	0	0
NM_0323	chr12	66123069	66130753	-	1212	1	LLPH|C12	5.745	4.769
NM_0160	chr6	1.55E+08	1.55E+08	-	2793	1	TFB1M|CG	4.062	8.955
NM_0010	chr19	46674316	46717127	-	3165	1	PRKD2|HS	7.614	9.512
NR_12118	chr2	1.14E+08	1.14E+08	-	790	1	PGM5P4-A	0	0
NM_0122	chr9	1.12E+08	1.12E+08	-	1235	1	PTGR1|LT	2.37	1.93
NM_0016	chr19	44942238	44945496	+	610	1	APOC4|AP	0	0
NM_0247	chr10	1.02E+08	1.02E+08	+	2669	1	HPS6|BLO	6.295	8.165
NM_0245	chr11	1.26E+08	1.26E+08	+	2079	1	FAM118B|	19.244	24.568
NM_0142	chr9	96450155	96491336	+	2655	1	HABP4|IH	21.931	20.388
NR_11063	chr20	11809987	11870715	-	522	1	LINC00687	0	0
NR_03761	chr6	7881250	8064414	-	3339	1	BLOC1S5-T	13.82	15.043
NM_0333	chr1	1.83E+08	1.83E+08	-	5618	1	RGS8|-|-|	74.364	56.1
NM_0031	chr3	12004360	12185732	+	3908	1	SYN2|SYN	0.261	1.185
NM_0123	chr19	54724486	54848130	+	1088.5	34	KIR2DS3|N	0	0
NM_0040	chr19	5913644	5916211	+	2140	1	CAPS|CAP	2.747	3.13
NR_04620	chr16	89166383	89169147	+	1323	1	LINC02138	0.039	0.01
NM_0001	chr17	75757937	75765199	-	1351	1	GALK1|GA	14.287	14.437
NM_0011	chr19	58396090	58397079	+	988	1	RNF225|-|	0.104	0.014
NM_0013	chr19	29811994	29824317	+	1808	1	CCNE1|CC	2.068	2.682
NM_1735	chr6	1.17E+08	1.17E+08	+	3486	1	RFX6|MTC	0.007	0
NR_03895	chr1	1.12E+08	1.12E+08	+	1974	1	FAM212B-	4.278	4.933
NM_0312	chrX	70528940	70908717	-	2986	1	TEX11|SP	0	0.018
NM_0038	chr7	1.38E+08	1.39E+08	+	3881	1	TRIM24|P	21.667	23.939
NM_0011	chr5	55219597	55227315	-	2095	1	MCIDAS|I	0	0.007
NM_1447	chr2	99141613	99151465	+	1511	1	C2orf15|-|	2.338	1.577
NM_0041	chr17	18225622	18244874	+	4200	1	LLGL1|DLG	67.636	56.764
NR_13503	chr12	92146085	92167560	+	964	1	LOC10192	0.094	0.227

NM_0189(	chr5	1.41E+08	1.41E+08	+	5254	1	PCDHA13|	16.354	15.663
NR_02675	chr6_GL0C	1261741	1321938	-	2424.1	8	ZNRD1ASP	0.069	0.09
NM_0335(	chr1	32894593	32901440	-	1075	1	TMEM54|	1.991	3.937
NM_0805!	chr6	27147082	27147562	+	478	1	HIST1H2AI	0.701	0.516
NR_04660	chr3	7519741	7535284	-	427	1	GRM7-AS1	0	0.096
NR_11041	chrX	1.32E+08	1.32E+08	+	3419	1	RAP2C-AS:	0.634	0.849
NM_0312(	chr19	10701436	10713367	+	1322	1	QTRT1|FP:	18.238	12.34
NM_0142:	chr1	1.57E+08	1.57E+08	-	4169	1	INSRR|IRR	0.226	0.48
NR_04669	chrX	1.33E+08	1.33E+08	+	546	1	HS6ST2-AS	0	0.05
NM_1824	chr14	73950252	73963110	+	1585	1	COQ6|CGI	14.057	14.19
NR_07343	chrX	1.53E+08	1.53E+08	+	749	1	CSAG4|-|-	0	0
NM_1991(	chr11	18266225	18269976	+	560	1	SAA1|PIG4	0	0
NM_0002(	chrX	71107406	71111631	-	1522	1	IL2RG|CD1	0.051	0
NM_0182(	chr19	52570273	52587174	+	5238	1	ZNF701|-|	0.586	0.986
NR_13465	chr6	42783496	42791323	+	2742	1	LOC40126	0.334	0.31
NR_03883	chr7	1.57E+08	1.57E+08	+	985	1	MNX1-AS1	0.039	0.014
NM_0011:	chr10	88802730	88820546	+	1473	1	LIPM|LIPL:	0.14	0.13
NM_1993:	chr5	39105252	39219578	-	4686	1	FYB|ADAP	0	0.038
NR_07342	chr17	38800434	38825350	-	3209	2	CWC25|CC	0	0
NM_0189:	chr5	1.41E+08	1.42E+08	+	4772	1	PCDHGA4|	19.832	78.632
NM_1446{	chr19	40379271	40390221	-	2480	1	HIPK4|-|-|	0.114	0.177
NM_0010:	chr4	1.65E+08	1.65E+08	-	1573	1	TRIM61|R	0.041	0.096
NR_03741	chr22	46091044	46091126	+	82	1	MIR3619|-	0	0
NR_13825	chr19	21986728	22010943	-	800	1	ZNF208|PI	0.886	1.952
NM_0143!	chr1	2.03E+08	2.04E+08	+	1434	1	OPTC|OPT	0.018	0.048
NM_0011(	chr12	1.17E+08	1.17E+08	+	2884	1	RNFT2|TM	7.421	6.868
NM_0133:	chr17_KI2	36140	41430	+	1924	3	TIMM22|T	0	0
NM_0012	chr11	60280541	60308972	+	1482	1	MS4A4A|4	0	0
NM_0034(	chr22	31944493	31957603	+	1789	1	YWHAH|Y'	192.4	234.028
NM_0011:	chr4	1.86E+08	1.86E+08	-	2699	1	PDLIM3|A	1.992	3.948
NM_0012!	chr1	1E+08	1E+08	-	1808	1	LRRC39|-|	0.784	0.871
NR_13429	chr8	1.19E+08	1.19E+08	-	1405	1	LOC10192	0	0
NR_00323	chr14	1.01E+08	1.01E+08	+	70	1	SNORD113	0	0
NM_1985(	chr3	57498004	57544344	-	2037	1	DNAH12|D	0.108	0.007
NR_03038	chr14	1.01E+08	1.01E+08	+	95	1	MIR411|M	0	0
NM_0010	chr6	10886831	10974308	+	3097	1	SYCP2L|C6	0.35	1.053
NM_1785:	chr9	1.37E+08	1.37E+08	+	681	1	LCN12|-|-	0.246	0.101
NR_13559	chr2	1.01E+08	1.01E+08	+	2476	1	TBC1D8-AS	0.229	0.476
NM_0011:	chr3	1.32E+08	1.32E+08	+	1814	1	ACPP|5'-N	0	0.008
NM_0011(	chr12	1.17E+08	1.17E+08	-	929	1	TESC|CHP:	21.054	19.948
NR_10381	chr2	14228874	14400958	-	3499	1	LINC0027€	0	0
NM_0012	chr20	34874963	34927966	+	2835	1	ACSS2|AC/	21.485	21.239
NR_12203	chr9	1.38E+08	1.38E+08	-	1177	1	ARRDC1-A	19.039	17.84
NR_01544	chr7	96968515	97014065	-	1987	1	DLX6-AS1|	0.019	24.389
NM_0010(	chr19	18419336	18434562	+	1702	1	SSBP4|-|-|	36.294	30.975
NM_0011(	chr8	1.03E+08	1.03E+08	+	3759	1	FZD6|FZ-6	2.092	1.691
NM_0208!	chr15	40894430	40903975	+	3888	1	VPS18|PEI	14.804	17.481
NM_0203:	chr16	19555415	19701163	+	3861	1	C16orf62|	19.893	18.284
NM_1827:	chr14	23125295	23154450	-	3163	1	SLC7A8|LA	14.919	4.149
NR_10383	chr9	67717498	67719179	-	1505	1	FAM27E3|	0.137	0.337

NR_00405	chr1	30935689	30935776	-	87	2	SNORD103	0	0
NR_13513	chr9	9799392	9803787	+	414	1	LOC10537	0	0
NM_0012	chr16	1419744	1420800	-	974	1	C16orf91|	13.78	12.783
NM_0012	chr16	1773228	1776238	+	1131	1	EME2|SLX	3.842	2.119
NR_02693	chr5	95852232	95860132	+	1913	1	LINC01554	0.067	0.243
NM_0029	chr6	33208509	33212722	+	1729	6	RING1|RIN	0	0
NM_0011	chr15	65076816	65079938	+	3120	1	KBTBD13|	0.008	0
NR_00233	chr7	1.17E+08	1.17E+08	-	944	1	ST7-AS2|S	8.14	9.083
NM_1538	chr12	13083537	13095806	-	2536	1	GSG1|-|-|	0.63	1.885
NM_0010	chr1	2.48E+08	2.48E+08	+	928	1	OR2G3|OF	0	0
NR_13551	chr21	44250813	44251521	+	522	2	LOC10537	0	0
NR_11084	chr6	5664986	5695272	-	801	1	LOC10192	0	0.017
NM_0807	chr20	35699310	35700980	+	456	1	ROMO1|C	123.958	75.364
NR_03823	chr19	8374376	8390691	-	1019	1	RAB11B-A	4.568	3.589
NR_12635	chr10	42979017	42981507	-	497	1	LINC01264	0	0
NM_0024	chr11	1.03E+08	1.03E+08	-	1894	1	MMP3|CH	0.007	0
NM_0132	chr12	64452060	64502119	+	3064	1	TBK1|FTD	2.441	3.228
NM_0012	chr1	9943428	9981766	+	1087	1	NMNAT1|	5.516	5.658
NR_13427	chr5	7346986	7347977	-	883	1	LINC02123	0.058	0.186
NM_0012	chr12	1.23E+08	1.23E+08	+	1223	1	ARL6IP4|S	80.513	52.492
NM_0011	chr11	18231355	18248674	-	842	1	SAA2-SAA	0	0.098
NM_0174	chr11	18412306	18451246	+	1220	1	LDHC|CT3	0	0
NM_1980	chr7	1.23E+08	1.23E+08	-	1299	1	RNF148|-|	0.129	0.032
NM_1386	chr19	1000419	1009732	+	3270	1	GRIN3B|G	1.605	1.081
NM_0010	chr10	14959439	14972851	+	620	1	MEIG1|SP	1.768	2.585
NR_03984	chr11	19760004	19760083	-	79	1	MIR4694|	0	0
NR_11005	chr3	1.87E+08	1.87E+08	-	2669	1	LOC10192	0.382	0.2
NM_0306	chr2	2.28E+08	2.28E+08	-	6813	1	SPHKAP|S	0.28	4.745
NM_0037	chr17	41420557	41424570	-	1709	2	KRT37|HA	0	0
NR_03171	chr14	1.01E+08	1.01E+08	+	87	1	MIR1197|	0	0
NM_0012	chr12	70515871	70610024	-	11519	1	PTPRB|HP	0.126	0.073
NM_0010	chr13	19837453	19863636	-	1498	1	ZMYM5|H	3.297	3.95
NR_00332	chr15	25067787	25067883	+	96	2	SNORD116	0	0
NM_0012	chr1	1.51E+08	1.51E+08	-	1554	1	SELENBP1	5.352	8.25
NM_0013	chr10	1E+08	1E+08	-	2357	1	CWF19L1|	11.313	12.622
NM_1982	chr12	1.21E+08	1.21E+08	-	1836	1	OASL|OAS	0.098	0.045
NM_0010	chr3	8980591	9249685	-	8866	1	SRGAP3|A	54.933	54.912
NR_00308	chr12	54016852	54035360	+	1644	1	HOXC5|CP	0	0.033
NR_13190	chr21	43358055	43362349	-	2888	1	LINC0167	0.549	0.005
NM_2073	chr10	21513480	21525682	-	6590	1	SKIDA1|C1	9.808	3.806
NM_0306	chr6	32148363	32151943	-	1890	8	PRRT1|C6	0	0
NM_0011	chr5	1.27E+08	1.27E+08	-	959	1	C5orf63|Y	0	0.029
NR_03826	chr11_KI2	95312	98112	-	2301	2	MIR210HG	1.3	0.292
NM_0166	chr10	28533492	28623112	+	5522	1	WAC|BM-	19.037	22.713
NM_0010	chr4	1.03E+08	1.03E+08	+	5886	1	CISD2|ERI	6.363	4.708
NR_02846	chr14	92058552	92106621	-	6825	1	ATXN3|AT	1.874	1.569
NR_02422	chr19	47923779	47923899	+	120	10	SNAR-A5|-	0	0
NR_02742	chr8	7301611	7355354	-	1146	2	FAM66B|F	0.191	0.203
NM_1825	chr15	98437162	98514382	-	3289	1	FAM169B|	0.008	0
NR_03162	chr12	50234142	50234212	-	70	1	MIR1293|	0	0.196

NR_11094	chr16	88194281	88195217	+	495	1	LOC10192	0.599	1.135
NR_01541	chr14	95157688	95179933	+	799	1	DICER1-AS	12.813	15.72
NR_10418	chr17	20868433	20905230	+	3466	1	CCDC144N	0.086	2.905
NR_03395	chr16	70219581	70226033	-	1885	1	SMG1P7|-	0.814	1.461
NM_0013	chr11	74493385	74498533	+	2470	1	LOC10028	1.383	1.83
NM_1450	chr11	57645087	57649944	-	1715	1	YPEL4|-|-|	11.24	15.47
NM_0010	chr1	2.07E+08	2.07E+08	+	3512	1	PFKFB2|PF	2.772	3.202
NM_0011	chr10	47501854	47523638	-	2377	1	AGAP9|AG	1.231	1.193
NM_0013	chr15	66293257	66333898	+	3673	1	DIS3L|DIS	6.126	5.788
NM_0010	chr1	28369582	28500369	+	6195	1	PHACTR4|	16.496	12.81
NM_1451	chr7	99616948	99632407	+	4339	1	ZSCAN25|	5.845	5.957
NM_1818	chr19	40393764	40413364	-	4848	1	PRX|CMT4	1.63	1.622
NR_03990	chr19	49888175	49888230	+	55	1	MIR4750|	9.612	18.18
NM_0051	chr15	34788096	34795726	-	3684	1	ACTC1|AC	0.343	0.041
NM_1749	chrX	13035617	13044798	-	1245	1	FAM9C|TE	0	0.011
NM_0010	chr21	39745407	39802096	+	2055	1	IGSF5|GSF	0.307	0.973
NM_0048	chr17	9021542	9244000	+	5945	1	NTN1|NTN	0.894	5.626
NM_0010	chr16	50742050	50801935	+	8547	1	CYLD|BRS	4.342	4.777
NM_0012	chr2	1.32E+08	1.32E+08	+	1334	1	CCDC74A|	6.669	5.442
NR_00219	chrY	22308859	22316205	+	2038	1	RBMY2FP|	0	0
NM_0013	chr3	1.97E+08	1.97E+08	+	6158	1	SENP5|-|-	6.702	6.295
NM_0322	chr1	21678591	21783195	-	4143	1	USP48|RA	9.42	12.718
NR_12067	chr10	71508153	71511920	-	422	1	CDH23-AS	0	0
NR_02404	chr3	47011542	47013467	+	1008	1	NRADDP|-	0.077	0.177
NR_11082	chr3	1.78E+08	1.78E+08	+	2954	1	LINC02015	0	0.009
NM_0176	chr16	15650226	15726351	+	3209	2	NDE1|HOI	0	0
NM_0152	chr11	47778083	47848544	-	5419	1	NUP160|-	13.798	13.26
NR_10705	chr5	1.81E+08	1.81E+08	-	81	1	MIR8089|	0	0
NR_13192	chr1	53328233	53336509	+	564	1	LINC01771	0.594	0.413
NR_03822	chr12	25954655	25959442	-	947	1	RASSF8-AS	2.246	2.459
NR_11070	chr3	50266641	50267371	-	336	1	SEMA3B-A	2.916	1.753
NR_03315	chr15	42141134	42156641	-	3400	1	PLA2G4F|	0.004	0.016
NM_0013	chr6	71288442	71309059	+	8855	1	OGFRL1|d	1.165	1.997
NM_0009	chr12	14880864	14886062	-	1852	1	MGP|GIG	0.606	0.725
NR_12578	chr1	2.18E+08	2.18E+08	-	417	1	SPATA17-A	1.02	0.46
NR_10353	chr1	817371	819834	+	1942	1	FAM87B|-	0.073	0.169
NR_03647	chr8	1.09E+08	1.09E+08	+	2971	1	ENY2|DC6	25.049	24.01
NM_0012	chr8	1.33E+08	1.33E+08	+	4729	1	WISP1|CC	0.005	0
NM_0012	chr7	1.54E+08	1.55E+08	+	4344	1	DPP6|DPL	0	0.016
NM_1447	chr1	1.93E+08	1.93E+08	+	1514	1	RGS13|-|-	0.009	0.226
NM_0019	chr4	1.47E+08	1.48E+08	+	4140	1	EDNRA|ET	0.044	0.069
NM_0323	chr4	56963344	56977660	-	2269	1	NOA1|C4c	12.945	17.79
NR_12645	chr12	1.28E+08	1.28E+08	-	1422	1	LINC0050	0	0
NR_03988	chr17	39726495	39726561	+	66	1	MIR4728|	1.563	0.623
NR_10413	chr1	1.51E+08	1.51E+08	-	1032	1	ADAMTSL	0.062	0.04
NM_0010	chr11	1.15E+08	1.15E+08	-	2155	1	NXPE4|C1	0	0
NM_0178	chr3	32481312	32502911	-	3360	1	CMTM6|C	5.177	5.246
NM_0181	chr4	1.22E+08	1.22E+08	-	2597	1	BBS7|BBS	7.129	6.419
NM_0009	chr1	28812142	28863696	+	1769	1	OPRD1|OF	0.044	0.225
NM_0040	chr2	2.01E+08	2.01E+08	-	1919	1	CLK1|CLK|	39.661	42.176

NM_0146	chr2	2.08E+08	2.08E+08	-	601	1	CRYGA|CR	0.15	0.387
NM_0248	chr1	2.49E+08	2.49E+08	+	3085	1	ZNF672|-|	18.423	20.308
NM_0011	chr12	31280586	31326225	-	3006	2	FAM60A|	0	0
NM_0041	chr17	41346093	41350812	-	1209.3	3	KRT33A|H	0	0
NM_0328	chr13	30906175	30925572	+	2383	1	MEDAG|A	0	0.109
NR_03744	chr17	75406069	75406162	+	93	1	MIR3678|	0	0
NM_0012	chr14	67241295	67336061	+	5359	1	MPP5|PAL	2.047	3.146
NM_0176	chr9	92456207	92482562	-	2510	1	ASPN|OS3	0.021	0.005
NR_04071	chr20	10023812	10219506	-	2954	1	SNAP25-A	0.087	0.088
NM_0013	chr5	1.7E+08	1.7E+08	+	2484	1	SPDL1|CC	3.151	2.989
NM_0030	chr1	17018726	17054170	-	1139	1	SDHB|CW	67.728	65.683
NM_0010	chr14	50108632	50116579	-	1672	1	VCPKMT|	3.524	3.064
NR_13464	chr18	72869040	72881399	+	456	1	LOC10050	0.735	0.841
NM_1983	chr3	1.84E+08	1.84E+08	+	2084	1	HTR3E|5-	0	0
NM_1383	chr10	1.33E+08	1.33E+08	+	1601	1	MTG1|GT	21.084	17.059
NM_0051	chr2	1.5E+08	1.5E+08	-	2703	1	RND3|ARH	73.311	63.093
NR_10990	chr20	60469609	60472976	-	379	1	LOC10192	0	0
NM_0013	chr3	1.59E+08	1.59E+08	+	2131	1	GFM1|CO	11.387	10.2
NM_0165	chr3	1.8E+08	1.8E+08	-	9187	1	PEX5L|PE	0.543	0.292
NM_0332	chr3	1.84E+08	1.84E+08	-	1356	1	CAMK2N2	31.216	34.626
NM_0010	chr19	35995188	35996318	+	1128	1	SDHAF1|L	17.191	18.663
NM_1384	chr14	24213937	24216070	-	725	1	MDP1|FN	33.007	21.594
NR_04521	chr8	6835554	6842453	+	1103	1	GS1-24F4.	0.07	0.199
NM_0013	chr10	5684838	5763740	+	8433	1	FAM208B|	2.943	5.511
NR_10704	chr2	79866495	79866583	-	88	1	MIR8080|	0	0
NM_0010	chr2	1.69E+08	1.69E+08	+	2959	2	G6PC2|IG	0	0
NM_0011	chr7	1.03E+08	1.03E+08	-	1334	1	UPK3BL|U	3.528	3.933
NR_07341	chr4	56809860	56821473	-	629	1	SPINK2|H	0.061	0.109
NM_1982	chr6	65334913	65579804	-	2218	1	EYS|C6orf	0.023	0.296
NM_0044	chr17	7312659	7315339	-	1144	1	GPS2|AM	55.586	49.113
NM_1816	chr21	30601121	30601267	+	145	1	KRTAP22-1	0	0
NR_12048	chr12	10015352	10030606	-	667	1	LOC10272	0	0
NR_03619	chr17_GL	165799	165871	-	72	4	MIR4315-1	0	0
NR_03649	chr3	1.88E+08	1.88E+08	-	2882	1	LPP-AS2|	0.251	0.181
NM_1456	chr7	14175981	14841450	-	3326	1	DGKB|DA	0.659	1.38
NR_10987	chr5	1.16E+08	1.16E+08	+	826	1	LOC10192	0	0.05
NR_02458	chr9	1.37E+08	1.37E+08	-	2381	1	CCDC183-	2.756	2.134
NR_03742	chr22	29333158	29333267	-	109	1	MIR3653|	0.946	0.503
NR_02711	chr6	35726762	35736947	-	1722	1	LOC28584	0.389	0.422
NR_02989	chr7	1022933	1023026	-	93	1	MIR339|M	0.139	0.295
NR_12212	chr6	1.63E+08	1.63E+08	-	2200	1	PACRG-AS	0.012	0.006
NR_11190	chr1	2.45E+08	2.45E+08	-	4832	1	LOC10192	0.598	0.544
NM_0010	chr18	657604	673499	+	1574	1	TYMS|HST	34.667	13.288
NM_0212	chr9	21227243	21228222	-	977	1	IFNA17|IF	0	0
NM_1533	chr16	50666300	50681353	-	3298	1	SNX20|SLI	0.125	0.158
NR_12055	chr11	1.12E+08	1.13E+08	+	956	1	LOC10192	0.148	0.201
NM_0010	chr1	2.02E+08	2.02E+08	+	3023	1	LGR6|GPC	0.017	0.068
NR_02745	chr1	2.35E+08	2.35E+08	-	996	1	LINC0134	0.013	0
NR_10993	chr5	80066350	80083665	-	2707	1	CTD-2201	0.262	0.516
NM_2032	chr4	26319710	26435130	+	5733	1	RBPJ|AOS	10.523	17.446

NR_04972	chr21	39175446	39183514	-	1027	1	PSMG1|C2	13.157	11.51
NM_0150(	chr3	44976249	45011470	+	1026	1	EXOSC7|E/	30.261	32.534
NR_11192	chr3	48663768	48669170	+	197	1	CELSR3-AS	1.767	0.695
NM_0010:	chr1	2.13E+08	2.13E+08	+	1916	1	ATF3|-|-|1	17.833	21.839
NM_1816	chr11	93330717	93438471	+	2651	1	DEUP1|CC	0	0
NM_0331	chr20	21395367	21397409	-	1615	1	NKX2-4|NI	0	0.042
NM_0012	chr1	48295372	48472208	-	4945	1	SPATA6|H.	1.447	1.053
NM_0002	chr20	10637684	10674046	-	5959	1	JAG1|AGS	17.682	10.162
NM_0159	chr3	1.61E+08	1.61E+08	+	2731	1	NMD3|CG	9.645	7.212
NR_11193	chr1	54641740	54710267	+	4212	1	MROH7|C	0	0.01
NM_1458	chr3	1.68E+08	1.68E+08	-	1184	1	PDCD10|C	10.988	12.332
NR_02683	chr12	72253507	72273509	-	3953	1	TRHDE-AS:	0.431	0.35
NR_11065	chrX	91414903	91434999	-	397	1	PABPC5-AS	0.325	0.38
NM_0010(	chr4	1.9E+08	1.9E+08	-	2077	2	FRG2|FRG	0	0
NR_11039	chrX	72688949	72712348	+	594	1	PHKA1-AS:	0	0.023
NM_0162(	chr10	47322490	47327588	+	3269	1	GDF2|BMI	0	0
NM_0011	chr7	77798792	77924183	+	2614	1	PHTF2|-|-	2.905	3.631
NR_03037	chr20	63942631	63942726	-	95	1	MIR647|M	2.714	3.893
NM_0012	chr11	1.31E+08	1.31E+08	+	3542	1	C11orf44|	0	0
NR_02685	chr15	30103547	30131734	+	871	5	ULK4P1|D	0	0.031
NM_0010:	chr1	1.53E+08	1.53E+08	-	646	1	SPRR2G|-|	0	0
NR_04976	chr1	1.55E+08	1.55E+08	+	3620	1	ZBTB7B|CI	3.027	3.56
NM_0013(	chr15	52547045	52569221	-	5338	1	ARPP19|A	39.318	30.268
NR_00299	chr7	45104906	45105042	-	136	1	SNORA5C|	1.138	0.806
NR_03975	chr22	37988794	37988853	+	59	1	MIR4534|-	0	0.232
NM_0012	chr1	2.37E+08	2.37E+08	-	313	1	MT1HL1|N	0.124	0
NM_0175	chrX	1.2E+08	1.2E+08	-	3252	1	NKRF|ITBA	6.994	7.817
NM_0010	chr3	1.47E+08	1.47E+08	-	1959	1	PLSCR5|-|	0.086	0.65
NM_1526	chr12	90952215	90955176	-	2959	1	CCER1|C1:	0	0
NM_0011	chr7	99598066	99611045	+	1529	1	GS1-259H:	1.813	3.637
NM_0011(	chr3	97986683	98035304	-	1509	1	GABRR3|-	0.017	0
NM_0011	chr1	2.07E+08	2.07E+08	-	2773	1	FCAMR|CD	0	0.005
NM_0011	chr19	54069947	54081365	-	890	10	TARM1|OI	0	0
NM_0011	chr12	56712427	56725542	-	1031	1	NACA|HSD	375.312	330.15
NM_0011	chr11	66616582	66646473	+	1609	1	RBM14-RB	100.103	101.268
NM_1449	chr17	35243067	35267742	+	4640	1	SLFN5|-|-|	0.484	0.794
NM_0010(	chr11	5985892	5986985	-	1091	1	OR52L1|O	0	0
NM_0010	chr11	94021212	94114208	+	5929	1	HEPHL1|-|	0.035	0.032
NR_13460	chr6	1.06E+08	1.06E+08	-	413	1	LOC10537	0	0
NR_10271	chr2	37950334	38036341	-	522	1	RMDN2-AS	1.655	0.079
NR_13632	chr2	1.13E+08	1.13E+08	-	431	1	LOC10537	0.12	0.381
NM_0012	chr7	1.09E+08	1.09E+08	-	3307	1	THAP5|-|-	2.792	2.328
NM_0012	chr4	1.59E+08	1.59E+08	+	1939	1	ETFDH|ETI	4.675	5.877
NR_10981	chrX	1.5E+08	1.5E+08	+	682	1	LINC0085C	0	0
NR_00301	chr15	82755945	82756071	+	126	1	SCARNA15	0	0.435
NR_11190	chr19	16134028	16154662	+	1571	1	HSH2D|AL	0.476	0.445
NR_13356	chr11	1.12E+08	1.12E+08	+	1131	1	LOC10013	0.023	0.012
NR_13512	chr9	67006925	67009022	-	362	1	LOC10537	0	0
NR_02967	chr11	1.22E+08	1.22E+08	-	87	1	MIR125B1	0	0
NM_1985	chr8	85245487	85284073	+	3846	1	CA13|CAX	0.416	0.399

NM_0165:	chr11	1.13E+08	1.13E+08	-	2603	1	DRD2|D2D	4.458	2.789
NM_0011:	chr12	54369129	54391305	-	2339	1	ZNF385A|I	30.131	23.494
NR_03027	chr1	34669599	34669694	-	95	1	MIR552|M	0	0
NR_07722	chr3	95654423	95683193	-	2590	1	MTHFD2P1	0.05	0.111
NR_02432	chr2	2.34E+08	2.34E+08	-	1991	1	MSL3P1|M	0.181	0.468
NM_1524:	chr8	37934281	37940129	-	1367	1	GOT1L1|-|	0	0
NM_0252:	chr6	1.5E+08	1.5E+08	+	1401	1	ULBP2|AL(	1.472	2.601
NM_1788:	chr8	41578188	41625001	+	6295	1	GPAT4|1-A	25.556	31.311
NM_0189:	chr5	1.41E+08	1.42E+08	+	4599	1	PCDHGA3|	20.516	87.57
NM_0808(	chr20	45123426	45124465	-	769	1	WFDC12|(	0	0
NM_0062:	chr7	1.02E+08	1.02E+08	-	970	1	POLR2J|PC	29.801	28.764
NR_03619	chr15_GL3	132051	132150	+	99	2	MIR4311|-	0	0
NM_0010	chr5	1.48E+08	1.49E+08	-	1225	1	HTR4|5-HT	0	0
NM_0013:	chr5	21750866	22212769	-	3700	1	CDH12|CD	2.209	2.029
NM_0021	chr9	21239202	21239979	-	775	1	IFNA14|IFI	0	0
NM_0012	chr9	1.28E+08	1.28E+08	-	2788	1	ENG|END|	0.361	0.457
NR_11042	chr7	55238436	55255635	-	466	1	ELDR|Fabl	0.083	0
NM_0051	chr16	66844379	66854146	+	1543	1	CA7|CAVII	0.05	0.018
NM_0013(	chr19	32972217	32977495	+	1343	1	FAAP24|C:	2.871	3.294
NR_13453	chr10	70771238	70785419	-	1510	1	TBATA|C1(	0	0
NM_0011	chr17	47323961	47430710	+	3506	1	EFCAB13|(	0.037	0.063
NR_13513	chr9	31371611	31381490	-	719	1	LINC01243	0.036	0
NM_0046	chr1	68045962	68051631	-	1967	1	DIRAS3|AF	0.747	0.585
NM_0207	chr5	1.16E+08	1.17E+08	-	6910	1	SEMA6A|H	24.384	26.512
NM_0010(	chr8	30785610	30812836	-	1961	1	PPP2CB|PI	52.284	73.067
NR_10673	chrX	1.09E+08	1.09E+08	+	48	1	MIR6087|I	0	0
NM_0024:	chr10	17809343	17911162	+	5159	1	MRC1|CD2	0.022	0.013
NM_0012	chr12	8845004	8876781	+	3759	1	A2ML1|CP	0.069	0.124
NM_0064!	chr4	2247433	2262012	-	3765	1	MXD4|MA	43.701	46.759
NR_10228	chr11	78139793	78173977	+	1079	1	KCTD21-AS	5.915	7.248
NR_11008	chr12	81953719	81993133	+	1369	1	LOC10192	0	0
NR_10380	chr2	46429190	46441833	+	737	1	LOC10180	0	0
NM_0053(	chr16	79593848	79600725	-	2643	1	MAF|AYGI	4.366	2.607
NR_11026	chr2	66697030	66703227	-	857	1	LINC01797	0.331	0.032
NR_02701	chr16	31567767	31569524	-	1757	1	YBX3P1|CS	0.477	0.234
NM_0180:	chr14	96279202	96363401	-	13171	1	ATG2B|C1	2.446	3.285
NR_03843	chr14	1.04E+08	1.04E+08	+	2053	1	LINC00637	0.025	0.013
NM_0324(	chr21	42371887	42389149	-	2127	1	TMPRSS3|	0.091	0.109
NM_0072	chrX	15738289	15787625	+	6022	1	CA5B|CA-V	0.492	0.71
NR_13621	chr5	1.71E+08	1.71E+08	-	1598	1	LOC10537	0.137	0.163
NM_0324:	chr1	1.1E+08	1.1E+08	+	1367	1	PROK1|EG	0.33	0.2
NR_13804	chr22	34756665	34997916	-	2985	1	ISX-AS1|-|	0.082	0.284
NM_0062:	chr3	1.79E+08	1.79E+08	+	9070	1	PIK3CA|CL	1.662	2.61
NM_0012	chr18	70630519	70650857	-	593	1	GTSCR1|-|	0	0
NR_13124	chr17	17674026	17677688	-	560	1	SMCR2|TC	0.138	0.538
NR_03649	chr1	1.83E+08	1.83E+08	+	1493	1	LINC01686	1.891	2.312
NR_12575	chr13	69222346	69322099	+	1041	1	LINC00383	0	0
NM_1446:	chr1	1.62E+08	1.63E+08	+	8437	1	UHMK1|KI	2	2.901
NR_13556	chr17	12789512	12991644	+	3823	1	ARHGAP44	4.941	7.352
NM_1447:	chr19	41262476	41307783	+	3584	1	HNRNPUL1	127.701	133.932

NM_0011!	chr6	73696085	73828317	+	8888	1	CD109|CP.	0.099	0.186
NR_02409	chr14	1.01E+08	1.01E+08	+	1625	1	LINC00523	0.032	0
NM_1828!	chr22	20424584	20437859	-	3484	1	SCARF2|N!	6.106	5.885
NM_0011	chrX	2486414	2500967	-	4506	2	ZBED1|AL	0	0
NM_0013	chr7	87876229	87909541	+	3787	1	DBF4|ASK	0.48	0.76
NM_0008(	chr11	72189915	72196323	+	938	1	FOLR1|FBI	0.33	0.394
NM_0808	chr20	1629152	1657779	-	1059	1	SIRPG|CD	0.024	0
NR_04623	chr2	6848309	6866635	-	2421	1	CMPK2|NI	0.399	1.403
NM_0013(	chr3	1.23E+08	1.23E+08	+	4454	1	PARP15|A	0	0
NM_0010	chr22	38578120	38656629	-	10303	1	FAM227A|	1.659	1.129
NM_0010!	chr7	1.28E+08	1.28E+08	+	1294	1	HILPDA|C	19.231	17.942
NM_0011	chr1	1.57E+08	1.57E+08	+	1775	1	RRNAD1|C	16.053	20.356
NM_1525	chr2	1.78E+08	1.78E+08	-	2641	1	TTC30B|IF	1.518	1.426
NM_0245(	chr2	1.76E+08	1.76E+08	+	1971	1	HOXD1|H(	0.026	0
NM_0808	chr6	30941000	30954221	+	4757	5	DPCR1|PB	0	0
NM_1471	chr9	1.02E+08	1.02E+08	-	3384	1	PPP3R2|PI	0	0.04
NM_0013	chr9	1.21E+08	1.21E+08	+	5899	1	CNTRL|CEI	0.992	1.683
NM_0203	chr11	818901	825571	+	2415	1	PNPLA2|1	11.639	10.407
NM_0158	chr1	59814861	59876378	+	5833	1	HOOK1|HI	0.309	0.589
NM_0320	chr17	61679186	61863559	-	8144	1	BRIP1|BA(	0.28	0.427
NM_0023	chr6	52264014	52284881	-	3189	1	MCM3|HC	24.222	15.84
NR_00214	chr7	1.43E+08	1.43E+08	-	1501	2	OR6W1P|	0	0
NR_13432	chr10	96021200	96090235	-	804	1	ENTPD1-A!	0.305	0.068
NM_0124!	chr7	1.36E+08	1.36E+08	-	2895	1	SLC13A4|	0.379	0.246
NM_0147	chr1	2.3E+08	2.3E+08	+	5620	1	URB2|KIA	2.748	3.663
NR_12050	chr6	1.52E+08	1.52E+08	+	715	1	SYNE1-AS1	0.559	0.211
NM_0123!	chr18	63587300	63599199	+	3170	1	SERPINB13	0	0
NM_0013(	chr17	40553770	40565484	-	2130	1	CCR7|BLR	0.278	0.231
NR_07299	chr6_GL00	2080124	2097031	-	4507	7	PPP1R10|	0	0
NM_0011	chr1	1.52E+08	1.52E+08	+	845	1	OAZ3|AZ3	7.858	7.797
NR_13457	chr7	46687879	46697122	-	1944	1	LOC73033	0.04	0
NR_03894	chr22	48866770	48898386	+	4008	1	LINC0131C	0	0.243
NM_0011	chr11	57657744	57661865	+	1649	1	CLP1|HEAI	11.181	10.923
NR_00368	chr20	2462952	2463047	-	95	1	SNORD119	0.271	0.721
NM_0159	chr19	44165096	44175429	+	817	1	ZNF226|-|	6.518	6.136
NM_1736!	chr3	9837849	9844018	-	1216	1	RPUSD3|-	25.161	20.163
NR_02406	chr3	1.51E+08	1.51E+08	+	2935	1	CLRN1-AS1	0.013	0.051
NM_0011	chr5	1.72E+08	1.72E+08	+	698	1	EFCAB9|-|	0.148	0.059
NM_2015!	chr11	20669551	21575683	+	3105	1	NELL1|IDH	21.672	17.248
NR_03985	chr13	66218250	66218324	+	74	1	MIR4704|	0	0
NM_0216	chr11	5624977	5644395	+	2304	1	TRIM34|IF	0.084	0.434
NM_1817(	chr21	10413532	10491615	+	1746	1	BAGE4|CT	0	0.008
NM_0122	chr14	77717601	77761154	-	2109	1	SNW1|Bx4	36.125	32.817
NM_0012	chrX	1.1E+08	1.1E+08	+	2571	1	NXT2|P15	1.108	0.948
NM_0012	chr10	97458324	97498794	-	3534	1	MMS19|M	31.559	37.184
NM_1985	chr10	1.14E+08	1.14E+08	+	6379	1	NHLRC2|-|	1.221	1.379
NM_0010	chr22	26483884	26491938	+	1284	1	SRRD|HC/	20.314	22.775
NM_1530	chr2	2.22E+08	2.22E+08	+	1695	1	CCDC140|	1.354	0.501
NM_0022	chr6	1.17E+08	1.17E+08	+	2141	1	KPNA5|IP(	5.811	7.466
NM_0013	chr19	37078222	37130366	+	2560	1	ZNF420|AI	3.531	4.259

NM_0189:	chr5	1.41E+08	1.41E+08	+	5254	1	PCDHA8|P	16.602	15.827
NR_02859	chrX	1.39E+08	1.39E+08	+	2154	1	SRD5A1P1	0.658	0.369
NR_07705	chr2	1.61E+08	1.62E+08	-	8835	1	AHCTF1P1	0.212	0.499
NM_0010:	chr2	2.13E+08	2.13E+08	+	1263	1	SPAG16|P	9.668	7.125
NM_0189!	chr19	1407569	1435687	+	2190	1	DAZAP1|-|	42.66	49.472
NM_0011{	chrX	53936680	54044174	-	4629	1	PHF8|JHDI	13.935	11.836
NM_0012!	chr20	50586110	50691530	-	4353	1	FAM65C|C	0.095	0.116
NM_1813(	chr14	1.02E+08	1.02E+08	+	2245	1	WDR20|DI	8.46	9.274
NM_0032:	chr22	19523024	19525337	-	1698	1	CLDN5|AV	11.048	4.679
NM_0013(	chr5	1.41E+08	1.41E+08	+	1588	1	TMCO6|PI	5.269	3.881
NM_0178:	chr19	38915264	38930896	-	2041	1	SARS2|SAI	11.283	8.818
NM_0184(	chr4	1.85E+08	1.85E+08	-	5148	1	LRP2BP|-|	0.511	0.593
NR_13807	chr5	91368632	91383373	-	4324	1	ARRDC3|T	15.506	19.785
NM_0010(	chr2	70777310	70790643	-	715	1	FIGLA|BHL	0	0.019
NM_0013:	chr3	1.23E+08	1.24E+08	-	4157	1	HACD2|PT	4.361	5.961
NM_0012{	chr1	2.12E+08	2.12E+08	+	4352	1	DTL|CDT2	2.569	1.744
NM_0314{	chr20	34363235	34511773	+	6754	1	ITCH|ADM	6.061	6.581
NM_0207!	chr17	29390925	29551903	+	12059	1	TAOK1|KF	8.894	12.641
NM_0134	chrX	56563593	56567010	+	3415	1	UBQLN2|A	27.573	32.444
NM_0229:	chr5_GL33	492261	510139	+	1896.2	5	SERF1B|FA	0.027	0.014
NR_10674	chr21	23079284	23079392	+	108	1	MIR6130|I	0	0
NM_0155:	chr19	34172447	34229515	+	3705	2	LSM14A|C	0.007	0.026
NR_13655	chr22	21301260	21326636	-	2825	1	LOC10099	0	0
NM_2071!	chr1	1.55E+08	1.55E+08	+	2742	1	ADAM15|I	13.528	16.17
NM_0011!	chr15	89830599	89912990	-	6244	1	C15orf38-/	31.521	36.673
NR_03385	chr11	58933643	59058452	-	2597	1	LOC28319	0.243	0.095
NM_0028{	chr5	1.68E+08	1.69E+08	+	2121	1	RARS|ArgI	25.27	30.642
NM_0013(	chr15	48139428	48178361	-	5317	1	MYEF2|Hs	13.425	15.065
NR_11003	chr14	26775496	26822120	-	505	1	LOC10192	0	0
NM_0011(	chr16	89686668	89696364	+	1745	1	CDK10|PIS	21.561	21.554
NM_1382!	chr11	1.26E+08	1.26E+08	+	1501	1	PATE1|PA	0	0
NM_0240{	chr13	1.03E+08	1.03E+08	-	2095	1	KDELC1|EI	8.179	7.394
NM_0011(	chr11	89911411	89920408	-	1640	2	TRIM49D2	0	0
NR_03968	chr8	61714788	61714859	+	71	1	MIR4470|	0	0
NR_14446	chr6	46680268	46704319	+	4045	1	TDRD6|CT	0.057	0.027
NM_0064:	chr17	15627966	15682879	-	2890	1	TRIM16|EI	5.911	8.048
NR_13552	chr2	2.18E+08	2.18E+08	+	564	1	LOC10537	0.091	0.024
NR_03767	chr19	47274402	47275723	+	772	1	INAFM1|P	31.031	25.247
NM_0208(	chr16	57994668	57999858	-	4217	1	ZNF319|ZI	7.809	8.208
NM_0012!	chr11	72292426	72434684	-	3081	1	CLPB|ANK	11.768	12.71
NR_03669	chr9	87083886	87085225	-	1339	1	LOC49412	0.847	0.869
NM_0010:	chr1	35557792	35566779	+	3356	1	NCDN|-|-|	31.135	40.924
NM_0010:	chr1	2.49E+08	2.49E+08	+	949	1	OR2G6|-|-	0	0
NM_0141{	chr2	64454193	64461383	+	3820	1	LGALSL|GI	8.033	7.913
NM_0012(	chr22	37943050	37953669	-	1769	1	C22orf23|	13.251	13.86
NM_0011	chr11	88504576	89048072	-	7844	1	GRM5|GPI	1.336	1.109
NM_0042:	chr17	16217195	16326259	+	1143	1	PIGL|CHIM	3.666	3.463
NM_0314:	chr8	10723768	10730574	-	3274	1	SOX7|-|-|:	0.169	0.331
NR_10701	chr2	49059603	49059658	+	55	1	MIR548BA	0	0
NM_0143:	chr1	51236273	51273447	+	3069	1	RNF11|CG	36.979	49.789

NM_0046(	chr17	58489532	58517890	-	5808	1	MTMR4|F	51.911	48.824
NM_0000!	chr13	32315480	32399672	+	11357	1	BRCA2|BR	0.041	0.148
NM_0332(	chr10	1018907	1025859	-	1352	1	IDI2|IPPI2	2.384	2.219
NR_03018	chr19	53679003	53679085	+	82	2	MIR515-2|	0	0
NM_0012(	chr1	1352689	1358547	-	1996	1	MXRA8|-|	5.956	6.162
NM_0011(	chr1	32361270	32362243	-	694	1	FAM229A|	3.957	3.928
NM_0142(	chr4	87608529	87616873	+	4324	1	DSPP|DFN	0.006	0
NM_0021!	chr4	70050415	70058848	+	562	1	HTN1|HIS:	0	0
NR_02436	chr4	1.22E+08	1.22E+08	+	2223	1	PP12613|-	0.035	0.049
NR_03840	chr8	73420074	73441518	+	2065	1	STAU2-AS:	0.037	0.086
NM_0012!	chr7	99684679	99735200	-	2245	1	CYP3A7-C\	0.098	0.128
NM_0013:	chr15	92393887	92468728	+	5579	1	ST8SIA2|H	62.33	63.067
NR_03992	chrX	17425881	17425954	+	73	1	MIR4768|-	0	0
NR_11080	chr17	57600865	57608412	-	1400	1	LOC10192	0.028	0.02
NM_0141	chr3	1.13E+08	1.13E+08	+	1349	1	GTPBP8|H	3.317	4.752
NM_0209:	chr7	1.32E+08	1.33E+08	-	13027	1	PLXNA4|F,	4.346	12.047
NM_0011‹	chr9	1.28E+08	1.28E+08	-	2533	1	SH2D3C|C	21.847	30.611
NM_1334‹	chr16	46884380	46931297	+	3968	1	GPT2|ALT:	26.284	39.762
NM_0012(	chr19	31592281	31593781	+	1185	1	THEG5|-|-	0	0.092
NM_0011:	chr1	2.13E+08	2.13E+08	+	4152	1	RPS6KC1|	8.319	10.213
NM_0221(	chr6_GL0C	3629115	3633872	-	1446	7	GPSM3|A(	0.053	0.114
NM_0011!	chr7	30752135	30757602	+	2551	1	INMT|TEN	0.01	0.102
NM_0037(	chr3	1.28E+08	1.28E+08	-	1755	1	RUVBL1|E	54.623	47.959
NM_1756:	chr16	56625673	56627113	+	502	1	MT1E|MT-	0.411	0.027
NM_0013:	chr1	1.52E+08	1.52E+08	+	6294	1	SNX27|MF	8.129	8.994
NR_00366	chr9	33500756	33511166	-	813	1	SUGT1P1|	0.825	0.809
NR_13507	chr11	1.29E+08	1.29E+08	-	1914	1	LOC10192!	0	0
NM_1817:	chr1	1.54E+08	1.54E+08	-	2662	1	CRTC2|TO	14.085	13.234
NR_11083	chr7	1.32E+08	1.32E+08	+	1223	1	LOC10192	0	0
NR_13463	chr6_GL0C	461249	556189	+	392.3	7	LOC10192!	0	0
NR_03751	chr12	64622509	64622605	-	96	1	MIR548Z|-	0	0
NM_0189:	chr5	1.41E+08	1.42E+08	+	4611	1	PCDHGA1(	22.289	98.654
NR_03612	chr11	88176502	88176593	+	91	1	MIR3166|-	0	0
NR_12606	chr16	3308313	3318576	+	2305	1	ZNF75A|-|	5.42	7.57
NM_0191:	chr5	1.41E+08	1.41E+08	+	2708	1	PCDHB8|P	0.3	0.273
NM_0040:	chr15	60347151	60397986	-	1539	1	ANXA2|AN	4.7	2.358
NM_0190	chr2	2.34E+08	2.34E+08	+	2326	1	UGT1A7|U	0.033	0
NM_0011!	chr3	20169480	20186233	-	2285	1	SGO1|CAII	0.666	0.605
NR_03616	chr20	51452905	51452977	-	72	1	MIR3194|	0	0
NR_11005	chr3	1.8E+08	1.8E+08	+	666	1	PEX5L-AS2	0.194	0.123
NM_0201!	chr1	2.14E+08	2.14E+08	+	1671	1	SMYD2|HS	12.932	7.976
NM_0055(	chr1	1.83E+08	1.83E+08	+	5598	1	LAMC2|B2	1.013	0.384
NM_0010(	chr15	68301704	68432153	-	4977	1	ITGA11|Hs	0.106	0.127
NR_13273	chr1	1702384	1724565	-	3051	1	CDK11A|C	2.848	2.837
NR_03791	chr12_KI2	44555	366565	-	1635	3	PRH1-PRR	0.394	0.427
NM_0010:	chr3	1.54E+08	1.54E+08	-	2317	1	GPR149|IE	0.011	0
NM_0013(	chr19	35641737	35644871	+	927	1	ETV2|ER7:	2.351	1.832
NM_0013:	chr3	16265160	16337361	+	1727	1	OXNAD1|-	3.815	4.616
NM_0011:	chr3	1.39E+08	1.39E+08	-	2305	1	PRR23A|-|	0	0
NR_03611	chr10	6152196	6152277	+	81	1	MIR3155A	0	0

NR_03337	chr22	47621043	47631569	-	4179	1	LINC0089{	0	0
NR_11052	chr22	49414525	49657542	-	1760	1	C22orf34|	0	0.016
NM_0324!	chr16	89828499	89871319	+	3227	1	SPIRE2|Sp	11.535	12.385
NM_1736(	chr5	94519213	94618604	-	2255	1	KIAA0825|	0.263	0.207
NR_02723	chr19	36580251	36605276	-	2689	1	ZNF529|-|	3.04	3.632
NM_0061	chr17	38869859	38921770	+	4100	1	LASP1|Las	86.779	87.868
NR_05197	chr14	24180219	24188918	-	3590	1	IPO4|Imp4	8.695	6.055
NM_0010(	chr22	46150596	46243756	+	9955	1	PPARA|NR	0.826	1.07
NM_0184(	chr12	2812621	2825071	+	2348	1	ITFG2|MD	8.347	7.858
NM_0010(	chr1	1.6E+08	1.6E+08	-	928	1	OR10J5|O	0	0
NR_12067	chr10	85449592	85492001	+	1486	1	LINC0152C	0	0
NM_0013	chr12	48147788	48157595	-	2605	1	ASB8|PP14	21.105	25.606
NM_1304	chr21	37420299	37515376	+	5076	1	DYRK1A|D	15.205	18.13
NM_0011	chrX	71283192	71301171	+	3037	1	NONO|MF	205.587	207.516
NM_1446!	chr21	32574841	32585535	-	2690	1	TCP10L|C2	3.346	3.809
NM_0150	chrX	53232876	53281614	-	5222	1	IQSEC2|BF	3.607	8.262
NR_03969	chr9	1.35E+08	1.35E+08	-	73	1	MIR3689D	0	0
NM_0189	chr5	1.41E+08	1.42E+08	+	4599	1	PCDHGA6|	20.096	81.015
NR_11099	chr9	86127540	86157072	+	2437	1	LOC10192	0.032	0.028
NR_03886	chr3	49549306	49554366	-	2600	1	BSN-AS2|-	0.426	0.795
NR_12045	chr12	70242306	70243360	-	881	1	LINC01481	5.854	8.131
NM_0011	chr21	25880550	26171128	-	3277	1	APP|AAA|	404.002	583.918
NR_10989	chr5	1.59E+08	1.59E+08	+	2860	1	LOC10192	0.924	0.421
NM_0010	chrX	1.55E+08	1.55E+08	-	2313	1	MTCP1|P1	3.027	2.446
NM_0320	chr20	3227417	3237727	-	3073	1	SLC4A11|E	0.659	1.266
NM_0059!	chr16	56666735	56668065	-	397	1	MT1G|MT	0.227	0
NM_0066	chr2	74198563	74215297	+	2177	1	MTHFD2|N	14.41	16.799
NM_1814(	chr1	2.28E+08	2.28E+08	-	572	1	MRPL55|A	46.322	42.169
NM_0206	chr11	8937572	8943033	-	646	1	ASCL3|HA	0.08	0.17
NM_0011	chr19	7868719	7874441	-	4510	1	PRR36|-|-	52.706	46.837
NR_04655	chr13	50807855	50849905	+	1802	1	DLEU7-AS1	0.036	0.023
NM_0221(	chr20	63235883	63240507	+	1305	1	BIRC7|KIA	0.089	0.063
NM_0013(	chr17	76726830	76733881	+	815	1	METTL23|	21.595	19.512
NM_0175(	chr12	1.04E+08	1.04E+08	+	8159	1	STAB2|FEE	0.014	0.003
NM_0024	chr19	41755520	41772208	+	2593	1	CEACAM6	0	0
NR_02996	chr14	1.01E+08	1.01E+08	+	83	1	MIR329-2|	0	0
NR_00278	chr20	32189146	32190360	-	1214	1	TSPY26P|T	3.452	3.735
NM_0044	chr1	33323623	33349898	-	2557	1	PHC2|EDR	139.408	159.372
NM_0226!	chr5	87268924	87391926	+	3753	1	RASA1|CM	7.915	9.142
NR_02659	chr2	2.17E+08	2.18E+08	-	3893	1	DIRC3|-|-|	0.487	2.639
NM_0012	chr14	52267713	52276724	+	3028	1	PTGDR|AS	0	0.009
NR_12580	chr6	1605531	1607356	+	1608	1	FOXCUT|L	0.008	0.06
NM_0189	chr5	1.41E+08	1.42E+08	+	4759	1	PCDHGB7|	27.678	84.122
NR_01544	chr7	1.13E+08	1.13E+08	-	1036	1	LINC0099{	10.628	9.519
NM_0004!	chr1	1.7E+08	1.7E+08	-	3859	1	SELE|CD62	0	0
NR_04003	chr8	9325051	9335080	+	3439	1	LOC15727	0	0
NM_0012	chr7	64794388	64833681	+	2618	1	ZNF138|pI	2.182	3.463
NM_0013	chr7	37683775	37743821	+	3812	1	GPR141|P	0	0
NM_0067(	chr17	1344540	1400262	-	1819	2	YWHAE|14	0	0.015
NR_10695	chr19	2434914	2435000	-	86	1	MIR7108|	7.196	1.752

NM_0248	chr3	1.84E+08	1.84E+08	-	2133	1	MAP6D1|I	6.474	7.879
NR_03650	chr19	58002894	58007206	+	1893	1	LOC10012	5.973	6.613
NM_0000	chr3	1.66E+08	1.66E+08	-	2449	1	BCHE|CHE	13.067	13.507
NM_0012	chr19	6887549	6940453	+	2928	1	ADGRE1|E	0	0
NM_1445	chr15	77994985	78077652	-	6053	1	TBC1D2B|	3.104	3.926
NR_10379	chr5	1.76E+08	1.76E+08	-	1547	1	LOC10099	0	0
NR_11027	chr2	1.29E+08	1.29E+08	+	1088	1	LOC10192	0	0
NM_0010	chr1	1.13E+08	1.13E+08	-	1287	1	RHOC|ARH	57.745	50.222
NM_1308	chr5	1.57E+08	1.57E+08	-	2028	1	FAM71B|-	0	0
NR_13653	chr20	1117852	1118448	-	200	1	LOC10537	1.547	0.685
NM_0012	chr20	57495965	57525127	-	2485	1	CTCFL|BOI	0.01	0
NM_0011	chr10	21125763	21146559	-	610	1	C10orf113	0.042	0
NM_0010	chr4	82484451	82561973	-	1878	1	TMEM150	4.799	4.427
NM_0010	chrX	48058178	48071141	-	2509	1	ZNF630|dJ	2.256	3.625
NM_0010	chr11	5596635	5644395	+	3462	1	TRIM6-TRI	0.074	0.313
NM_0010	chr12	1.12E+08	1.12E+08	+	1329	1	ERP29|C1	116.682	109.02
NM_0051	chr10	5524889	5526268	+	1377	1	CALML3|C	0	0.01
NM_0022	chr11	30009741	30017030	-	4175	1	KCNA4|HE	0.179	0.505
NR_10691	chr9	1.31E+08	1.31E+08	+	66	1	MIR6856|	8.4	6.641
NM_0333	chr1	53242370	53328149	-	7248	1	LRP8|APO	11.196	18.937
NR_11054	chr22	30008742	30080480	-	1147	1	HORMAD2	2.642	3.2
NM_0013	chr8	1.04E+08	1.04E+08	-	2111	1	DPYS|DHP	0.043	0.143
NM_0324	chr19	2164149	2232578	+	7406	1	DOT1L|DC	17.305	17.218
NR_00330	chr15	25185631	25185712	+	80.2	5	SNORD115	0	0.171
NM_0209	chr3	49683947	49688763	-	2311	1	MST1|D3F	2.209	2.394
NM_0011	chr11	66546841	66563329	+	2916	1	ACTN3|-|-	0.119	0.042
NR_12640	chr7	1.15E+08	1.15E+08	-	543	1	LINC01392	0.024	0.177
NM_0250	chr3	47282840	47346816	+	4714	1	KLHL18|-|	12.565	11.035
NM_0191	chr19	4657545	4670403	-	1043	1	MYDGF|C1	42.29	41.249
NM_0035	chr12	53103490	53124539	+	2056	1	SOAT2|AC	0.013	0.06
NM_0012	chr19	51415724	51418196	+	1677	1	LOC10012	0.146	0.074
NM_1522	chr16	30949084	30954938	+	2184	1	ORAI3|TM	11.719	6.849
NM_0183	chr11	45805090	45813016	+	3484	1	SLC35C1|C	14.363	20.581
NR_04058	chr7	75359200	75367809	+	2128	1	STAG3L1|S	0.012	0
NM_0012	chr10	74151185	74709303	+	1858	1	ADK|AK|-|	8.799	7.969
NR_10691	chr9	35732922	35732995	+	73	1	MIR6853|	0.883	4.878
NM_0152	chr9	1.13E+08	1.13E+08	-	4306	1	FKBP15|FK	8.54	8.69
NM_0053	chrX	1.5E+08	1.5E+08	+	1877	1	MAGEA11	0	0
NR_00332	chr15	25075928	25076021	+	93	1	SNORD116	0	0
NM_0040	chr1	44800225	44805995	+	2480	1	PLK3|CNK	25.08	15.685
NR_10697	chr18	11654885	11654941	-	56	1	MIR7153|	0	0
NM_0251	chr2	47806920	47888719	-	3819	1	FBXO11|FI	16.516	28.179
NR_02983	chr4	1.13E+08	1.13E+08	-	68	1	MIR302A|	0	0
NR_03605	chr9	1.28E+08	1.28E+08	+	89	1	MIR2861|	1.159	1.385
NR_11053	chr3	37790090	37861780	-	504	1	ITGA9-AS1	1.126	0.761
NM_0013	chr20	49046246	49096960	+	3600	1	CSE1L|CAS	23.43	32.112
NR_02435	chr21	17835672	17885608	-	458	1	CHODL-AS	0	0
NM_0011	chr6	79484991	79537430	-	4554	1	LCA5|C6or	1.673	1.672
NM_0012	chrX	71059247	71068448	-	2258	1	SNX12|-|-	40.331	47.849
NR_02742	chr9	61854084	61855340	+	701	1	FAM27C|F	0.037	0.039

NM_0121(	chr1	11648361	11654831	-	1707	1	FBXO2|FB(	12.674	12.879
NR_03832	chr3	1.85E+08	1.85E+08	+	1678	1	MAP3K13|	18.841	10.105
NR_00358	chrY	6443434	6447077	+	305	2	TTTY21B|-	0	0
NM_0013:	chrX	52947256	52957659	+	1669	2	FAM156B|	0	0
NM_1833	chr6	3722602	3752012	-	2187	1	PXDC1|C6	4.274	7.121
NM_0012(	chr12	1.2E+08	1.2E+08	-	8658	1	CIT|CITK|(	2.855	3.093
NM_0031(	chr2	36837698	36966472	-	14090	1	STRN|PPP:	1.582	1.729
NR_03403	chr1	2.32E+08	2.32E+08	-	595	1	LINC00582	0	0
NM_0010(	chr14	21569803	21570744	-	940	1	OR10G3|C	0	0
NM_0073	chr7	1.55E+08	1.55E+08	-	3689	1	PAXIP1|CA	5.344	6.279
NM_0012	chr7	44217150	44325631	-	2404	1	CAMK2B|(	9.338	15.275
NR_04663	chrX	22162732	22172983	-	1902	1	PHEX-AS1|	0.014	0.137
NM_0012	chr6	36697851	36733183	+	4217	1	RAB44|RA	0.012	0.016
NR_04670	chr3	64103470	64106358	+	794	1	PRICKLE2-/	0	0
NR_03383	chr2	45174341	45254941	-	1092	1	LINC01121	0	0.013
NM_0064:	chr14	74479940	74493381	-	890	1	NPC2|EDD	47.836	38.598
NM_1723(	chr1	2.08E+08	2.08E+08	+	3156	1	CD46|AHU	11.594	10.173
NM_0786:	chrX	11758159	11775753	+	2330	1	MSL3|MSL	11.206	12.145
NM_0046	chr14	1.05E+08	1.05E+08	+	2817	1	MTA1|-|-|	48.246	53.553
NM_0000:	chr5	1.13E+08	1.13E+08	+	10712	1	APC|BTPS:	7.441	16.614
NM_0028(	chr16	67934504	67936877	-	996	1	PSMB10|L	11.314	8.774
NR_12046	chr12	14567732	14619755	+	580	1	PLBD1-AS1	0	0
NM_0011	chr17	6444415	6451065	+	1492	1	FAM64A|(	13.17	6.766
NM_0013:	chr1	92246398	92299009	-	1970	1	GLMN|FAI	1.433	1.544
NM_0010	chr2	24175043	24191698	+	1826	1	FAM228A|	1.306	1.478
NM_0012	chr20	63639705	63652766	-	2344	1	STMN3|SC	144.87	142.72
NR_12599	chr1	6204840	6205780	-	375	1	LOC10272	1.066	0.073
NR_03784	chr7	27200421	27207259	+	4662	1	HOTTIP|H(	0	0
NM_0010	chr3	32996608	33096832	-	2492	1	GLB1|EBP	28.529	26.647
NM_0010(	chr2	53767783	53775196	+	1325	1	CHAC2|-|-	1.392	1.323
NM_0011:	chr11	34916130	34996128	+	2646	1	PDHX|DLD	8.683	10.363
NM_0333(	chr16	67148102	67150999	-	2701	1	B3GNT9|-	3.208	3.428
NR_13466	chr4	42299012	42391268	-	392	1	LOC10537	0	0.175
NR_12639	chr8_KI27(	29497	44591	+	1913	2	C8orf31|-|	0	0.014
NM_0010:	chr19_KI2	60105	69601	-	1622.7	7	KIR2DL5B|	0	0
NM_1980	chr8	22580474	22594297	+	1449	1	PDLIM2|M	6.166	9.254
NM_0146(	chr2	1.36E+08	1.36E+08	+	3999	1	UBXN4|UE	12.358	13.69
NR_03037	chr22	21034176	21034272	-	96	1	MIR649|M	0	0
NM_0012	chr9	96320706	96383710	-	1351	1	SLC35D2|H	1.012	0.558
NM_0011	chr17	37978099	37989062	-	2089.2	19	TBC1D3H|	0.099	0.184
NM_0049	chr8	7894565	7896716	+	445	1	DEFB4A|B	0	0
NR_00368	chr5	9548827	9548914	+	87	1	SNORD123	0	0
NM_0013:	chr1	1.54E+08	1.54E+08	+	4861	2	INTS3|C1c	0	0
NR_10687	chr22	20114686	20114751	-	65	1	MIR6816|I	2.579	1.897
NM_0223:	chr11	88113247	88175467	-	1455	1	RAB38|NY	0.762	0.433
NM_0013:	chr11	66421004	66426706	+	3183	1	NPAS4|Le-	0.709	0.374
NR_03765	chr12	55716034	55719707	+	502	1	BLOC1S1|I	92.155	85.893
NM_0807	chr20	62844566	62846191	+	1623	1	DPH3P1|C	1.684	1.224
NM_0013:	chr10	96130056	96163761	+	7968	1	ZNF518A|:	0.853	1.239
NR_03323	chr6	39904258	39934551	-	3960	1	MOCS1|M	3.093	5.164

NM_0011	chr22	50508216	50524780	+	3326	1	NCAPH2|C	34.49	30.067
NM_0247	chr17	82829434	82840055	-	3186	1	ZNF750|ZI	0.04	0.009
NR_03622	chr2	1.09E+08	1.09E+08	-	54	1	MIR4266|	0	0
NM_1986	chr21	44539185	44540195	-	1008	1	KRTAP10-1	0	0
NM_0011	chr22	15690026	15721631	+	2025	1	POTEH|A2	0	0
NM_0332	chr16	3298808	3305439	-	3426	1	TIGD7|Sar	4.11	5.789
NR_00279	chr6	41249377	41249589	+	212	1	TREML5P|	0	0
NR_04637	chr19	5978403	6020363	+	635	1	LOC10012	0.528	0.798
NR_02451	chr9	1.33E+08	1.33E+08	+	2971	1	ADAMTS1	3.806	3.176
NM_0017	chr16	28931939	28939347	+	1938	1	CD19|B4|	0.027	0
NM_0011	chr3	52075233	52154690	-	2040	1	POC1A|PI	3.426	2.652
NM_0010	chr1	1.01E+08	1.01E+08	+	3193	1	VCAM1|CI	1.195	4.959
NM_0012	chr18	36297696	36780220	+	5073	1	FHOD3|FH	15.359	18.832
NM_0010	chr3	56557156	56621820	+	3097	1	CCDC66|-|	2.098	3.724
NM_0038	chr12	93677371	93850755	+	1187	1	CRADD|M	12.557	13.443
NM_0183	chr7	1.51E+08	1.51E+08	+	1872	1	GIMAP5|H	0	0
NM_1707	chr6	1.35E+08	1.35E+08	-	2388	1	ALDH8A1|	0.286	0.224
NM_0013	chr12	1.18E+08	1.18E+08	-	2056	1	TAOK3|DP	12.517	11.205
NM_0189	chr5	1.41E+08	1.41E+08	+	5354	1	PCDHA2|P	19.545	18.207
NR_02402	chr21	41716436	41717582	+	521	1	LINC00112	0	0
NM_0011	chrY	14622021	14733549	+	876	1	NLGN4Y|-	3.591	5.097
NR_13807	chr6	1.7E+08	1.7E+08	-	2073	2	TCTE3|TC	0	0
NM_0012	chr5	65926554	66081023	+	6880	1	ERBIN|ERE	2.551	4.051
NM_0010	chr11	10455119	10507579	+	4317	1	AMPD3|-|	0.559	0.095
NR_12053	chr11	1896759	1908359	+	1199	1	LINC0115C	0	0
NM_0010	chrX	54192823	54358005	-	11147	1	WNK3|PRI	2.184	2.121
NR_14443	chr14	94770642	94773607	+	2224	1	DIGIT|-|-|	0.023	0.012
NM_0012	chrY	21382974	21386360	-	852	1	PRORY|CY	0	0
NM_0012	chr11	47248300	47269033	+	1867	1	NR1H3|LX	3.833	3.228
NM_1812	chr16	66566391	66579135	+	489	1	CMTM1|C	0.949	0.588
NM_0034	chr17	57085192	57121349	+	3955	1	AKAP1|AK	16.946	19.723
NM_0182	chr17	19533854	19579033	+	3260	1	SLC47A1|	0.154	0.252
NR_00127	chr9	1.33E+08	1.33E+08	+	980	1	CELP|CELL	0.053	0
NR_03034	chr12	64622509	64622605	+	96	1	MIR548C|	0	0
NM_0306	chr5	1.79E+08	1.79E+08	+	2421	1	ZFP2|ZNF	0.953	1.697
NR_02419	chr17	50171428	50172476	-	901	1	HILS1|H1.	0	0
NR_04982	chr12	1.25E+08	1.25E+08	+	112	1	MIR5188|	0	0
NM_0011	chr14	75427806	75472701	+	3779	1	JDP2|JUNI	2.832	5.136
NM_0013	chr10	72934762	72954819	-	1568	1	PLA2G12B	0	0.026
NM_0011	chr12	3077355	3286564	+	4274	1	TSPAN9|N	11.934	18.007
NM_0013	chr2	96326197	96328353	+	1735	1	ITPRIPL1|	0.944	0.695
NM_0042	chr5	71719163	71721048	+	910	1	CARTPT|C	1.828	0.542
NM_0011	chrX	1.24E+08	1.24E+08	+	8442	1	XIAP|API3	3.1	4.256
NM_0013	chr4	1.21E+08	1.21E+08	-	5239	1	PRDM5|B	0.212	0.324
NR_07314	chr11	1.01E+08	1.01E+08	-	11844	1	PGR|NR3C	0	0.001
NR_07345	chr14	19083134	19093337	-	722	3	BMS1P18|	0	0
NM_1524	chr19	40216058	40218399	-	838	1	TTC9B|-|-	49.789	52.516
NM_0010	chr1	1.09E+08	1.09E+08	-	1629	1	PSRC1|DD	20.99	21.693
NM_2016	chr12	98613404	98645051	-	1579	1	IKBIP|IKIP	1.217	0.867
NM_0142	chrX	1.24E+08	1.25E+08	-	12839	1	TENM1|OI	0.877	2.511

NM_0153	chr5	5422673	5490234	+	7927	1	ICE1|KIAA	4.634	7.76
NM_1779	chr9	86026143	86100201	-	3066	1	GOLM1|C	46.919	64.009
NR_13491	chr19	18940254	18946831	+	1022	1	LOC10272	0.341	0.389
NR_03975	chr20	57895394	57895444	+	50	1	MIR4532|	0	0
NR_03618	chr12	25874020	25874079	-	59	1	MIR4302|	0	0
NM_0016	chr3	1.96E+08	1.96E+08	-	1123	1	APOD|-|-|	0.023	0.061
NM_0012	chr17	35410922	35433176	-	2459	1	SLFN12|SL	0.031	0.011
NM_0163	chrX	6533618	6535118	-	990	1	VCX3A|VC	0	0
NM_0012	chr8	28090231	28191152	+	3137	1	ELP3|KAT	19.35	23.482
NR_04669	chr13	33277554	33281334	+	642	1	STARD13-	0	0.085
NR_00395	chr1	1.22E+08	1.22E+08	+	4223	1	EMBP1|-|	0.269	0.107
NM_0012	chr16	54931199	54934483	+	2054	1	IRX5|HMN	20.91	25.22
NM_0180	chr12	6452011	6462322	+	1809	1	TAPBPL|T	0.299	0.416
NM_0015	chr17	42184060	42185452	-	573	1	HCRT|NRC	1.418	1.004
NM_1988	chr3	64093850	64225455	-	8312	1	PRICKLE2|	8.485	6.918
NM_0066	chr5	58453983	58460139	-	2822	1	PLK2|SNK	25.545	24.598
NM_0331	chr16	50548330	50640860	+	8704	1	NKD1|Nak	3.524	3.16
NM_1527	chr19	46298376	46338540	+	1747	1	HIF3A|HIF	2.93	5.324
NR_03831	chr2	28307691	28310459	-	2107	1	LOC10050	0.11	0.13
NM_0039	chr7	26667062	26864743	-	3983	1	SKAP2|PR	0.097	0.873
NM_0071	chrX	57905430	57910633	-	5201	1	ZXDA|ZNF	0.387	0.716
NM_1774	chrX	1.42E+08	1.42E+08	+	1700	1	MAGEC3|	0.068	0.097
NM_0010	chr16	47461299	47701523	+	5569	1	PHKB|-|-|	13.051	15.01
NM_0246	chr14	73490934	73493401	+	2465	1	RIOX1|C1	7.715	6.918
NM_0204	chr1	1.56E+08	1.56E+08	+	1802	1	RHBG|SLC	0	0.046
NM_0147	chr8	22219703	22232338	-	3208	1	PHYHIP|D	3.854	13.215
NR_13273	chr17	8204731	8210225	-	1033	1	AURKB|AI	9.498	5.463
NM_0011	chr4	22692914	22819572	+	1235	1	GBA3|CBG	0	0.011
NM_0055	chr7	1.55E+08	1.55E+08	+	3006	1	INSIG1|CL	19.456	16.695
NR_12540	chr3	46364660	46407059	-	947	1	LOC10272	0	0
NR_11088	chr17	47303446	47323613	-	560	1	THCAT158	0.069	0.514
NM_0243	chr17	47951968	47957877	-	1513	1	PRR15L|A	0.017	0
NM_0000	chr2	1.89E+08	1.89E+08	+	5437	1	COL3A1|E	6.846	6.175
NM_0011	chrX	24558087	24672862	-	5482	1	PCYT1B|C	6.964	13.5
NM_0010	chrX	19360058	19515261	-	4603	1	MAP3K15|	1.728	2.047
NM_0053	chrX	1.53E+08	1.53E+08	+	1660	1	MAGEA12	0	0
NM_0012	chr1	22953043	22965430	-	1726	1	LACTBL1|-	0	0.024
NM_2032	chr7	33094798	33109390	-	1112	1	RP9|PAP-1	16.766	18.205
NR_03342	chr17	30624318	30626807	+	2489	1	SH3GL1P2	0.062	0.077
NM_0150	chr22	40177925	40335808	+	17927	1	TNRC6B|-|	3.689	3.871
NM_0027	chr14	58244805	58272009	+	985	1	PSMA3|H	50.697	49.337
NM_1985	chr4	1.1E+08	1.1E+08	+	3578	1	LRIT3|CSN	0.083	0.103
NM_0013	chr1	2.31E+08	2.31E+08	+	2480	1	GNPAT|D	25.319	28.604
NM_2074	chr8	38510834	38528662	-	2245	1	C8orf86|-|	0.046	0.067
NR_10980	chr9	1.18E+08	1.18E+08	+	1292	1	LOC10192	0	0.032
NM_0013	chr17	732546	742968	+	2066	1	FAM57A|	8.057	7.412
NM_0320	chr19	19514219	19515660	-	1439	1	TSSK6|CT	1.416	1.761
NR_03157	chrX	1.15E+08	1.15E+08	+	111	1	MIR1298|	0	0
NM_0012	chr9	1.33E+08	1.33E+08	+	1990	1	GTF3C5|T	34.663	38.895
NM_1814	chr3	1.81E+08	1.81E+08	-	3832	1	CCDC39|C	0.145	0.39

NM_0221	chr3	1.85E+08	1.85E+08	-	1679	1	MAGEF1|-	91.606	88.366
NM_0158	chr18	57352490	57368930	+	10147	1	ST8SIA3|S	1.428	4.199
NR_13561	chr4	1E+08	1E+08	-	120	1	SNORA101	0	0
NM_0010	chr10	16513743	16522005	-	2489	1	C1QL3|C1	0.575	0.083
NR_13047	chr2	50696172	50696262	-	90	1	MIR8485|	0	0
NM_0529	chr3	1.29E+08	1.3E+08	+	3904	1	IFT122|CE	11.282	8.199
NM_0032	chr21	42311667	42315596	-	1049	1	TFF3|ITF|	0.172	0.535
NR_02414	chr11	71928722	71997597	+	2560	1	RNF121|-|	13.186	12.841
NM_0212	chr19	3610645	3626815	-	2663	1	CACTIN|C1	12.375	12.802
NR_03993	chr2	66358249	66358328	-	79	1	MIR4778|	0	0
NM_0056	chr6	98834704	98838790	+	4084	1	POU3F2|B	61.544	29.822
NM_0002	chr1	1.72E+08	1.72E+08	-	2040	1	MYOC|GL	0	0
NM_0010	chr10	79512601	79516436	+	3833	1	EIF5AL1|E	1.712	1.969
NM_0010	chr11	49981473	49982535	-	1060	1	OR4C12|C	0	0
NM_0036	chr11	1.29E+08	1.29E+08	+	1761	1	BARX2|-|-	0	0.008
NM_0011	chr3	52710780	52770949	-	3442	1	NEK4|NRK	7.185	5.472
NM_0216	chr6	1.16E+08	1.16E+08	-	4128	1	TSPYL4|dJ	107.318	88.944
NM_0166	chr3	1.58E+08	1.59E+08	+	1715	1	RSRC1|BM	5.736	5.295
NM_0008	chrX	1.52E+08	1.52E+08	-	2773	1	GABRA3|-	3.65	10.318
NM_0010	chr7	5214269	5233855	+	4283	1	WIPI2|ATC	28.511	31.484
NR_03028	chr1	1.68E+08	1.68E+08	+	97	1	MIR557|M	0	0
NM_0010	chr7	1.03E+08	1.03E+08	+	957	1	NFE4|NF-E	0	0.014
NM_0011	chr10	1.14E+08	1.14E+08	+	2962	1	HABP2|FS	0.044	0.069
NM_0212	chr16	11273199	11273595	-	395	1	PRM3|-|-|	0	0
NR_11005	chr7	603398	608502	+	855	1	LOC10192	0	0
NM_0309	chr19	49818279	49836980	+	2323	1	MED25|A	26.736	20.071
NR_00312	chr11	1.23E+08	1.23E+08	-	79	1	SNORD14E	0.326	0.173
NM_0013	chr4	41612902	41700044	+	5804	1	LIMCH1|LI	5.183	13.105
NM_0012	chr7	1.02E+08	1.02E+08	+	1192	1	LOC10028	3.353	3.286
NM_0013	chr2	84516455	84819589	+	12716	1	DNAH6|DI	1.515	0.333
NM_0011	chr12	1.02E+08	1.02E+08	-	1103	1	SYCP3|CO	0.362	0.571
NR_10432	chr3	63860645	63861819	+	671	1	THOC7-AS	0.154	0
NM_1734	chr1	44118850	44135137	+	3123	1	KLF17|ZNF	0	0.004
NM_0037	chr11	6891574	6892500	-	925	1	OR2D2|OF	0	0
NR_04982	chr1	2.02E+08	2.02E+08	+	119	1	MIR5191|	0	0
NM_0064	chr20	35171937	35177362	+	1478	1	PROCR|CC	0.305	0.556
NR_04610	chr1	97988000	98049693	-	2464	1	MIR137HG	0.251	0.272
NR_10375	chr5	1.03E+08	1.03E+08	-	1511	1	LINC00491	0.102	0.127
NM_0012	chrX	1.08E+08	1.08E+08	-	6484	1	COL4A6|C	4.681	12.875
NR_04009	chr20	26187019	26209233	-	1208	1	MIR663AH	0	0.147
NR_02994	chr15	63870930	63871019	-	89	1	MIR422A|	0	0
NM_0010	chr7	1E+08	1E+08	-	999	1	C7orf61|-|	0.439	0.823
NM_0049	chr12	25204789	25250931	-	5758	1	KRAS|C-K-	7.504	7.155
NM_0010	chr7	1.51E+08	1.51E+08	-	1089	1	GBX1|Huh	0.118	0.013
NR_11709	chr11	31826567	31829303	+	2736	1	PAUPAR|-	0.41	0.15
NM_0162	chr6	83859651	83960427	+	2229	1	CYB5R4|N	3.014	4.234
NM_0013	chr12	8455995	8478330	+	1516	1	CLEC6A|CI	0	0
NM_1450	chr10	35336874	35572670	+	4694	1	CCNY|C10	15.904	16.113
NR_03743	chr13	77698012	77698116	-	104	1	MIR3665|	2.603	3.029
NR_02659	chrX	72941728	72943750	+	2022	2	FAM226B|	0	0

NM_0011⁄	chr17	7243587	7251676	-	1703	1	CTDNEP1|	62.628	66.539
NR_00394	chr1	1.74E+08	1.74E+08	-	80	1	SNORD76|	3.062	5.308
NM_0013	chr10	59650764	59710079	-	3504	1	SLC16A9|	1.391	2.119
NM_0164	chr20	63657810	63696253	+	4918	1	RTEL1|C2	4.234	3.905
NM_0011	chr17	65635540	66192131	-	3413	1	CEP112|C	3.351	1.276
NM_0012	chr2	69915041	69942944	+	5579	1	MXD1|BH	3.839	3.631
NR_04765	chr9	38540567	38545372	-	2664	1	FAM95C|-	0.223	0.01
NM_1385	chr19	14529567	14565980	+	1151	1	TECR|GPS	52.728	47.124
NM_0057	chr16	78022546	78032104	+	1933	1	CLEC3A|C	0.093	0.191
NM_0011	chr18	74253292	74292016	-	802	1	CYB5A|CY	40.24	65.069
NR_03613	chr15	20832795	20832869	+	74	4	MIR3118-	0	0
NR_00002	chr22	39315213	39315307	-	94	1	SNORD83	0.411	2.331
NM_0045	chr1	10474946	10630761	+	1918	1	PEX14|NA	18.466	18.746
NM_0012	chr1	1.61E+08	1.61E+08	+	2391	1	SLAMF7|1	0	0
NM_0013	chrX	71667702	71671524	+	1824	1	LOC10105	0	0
NM_0203	chr15	52109266	52112775	-	1242	1	BCL2L10|E	0.104	0.232
NM_0012	chr9	16203935	16253107	-	358	1	C9orf92|E	0	0
NM_0330	chr11	66040765	66049180	-	3315	1	GAL3ST3|	5.889	7.082
NM_0071	chr12	15882354	15903476	+	1893	1	STRAP|M	109.877	112.434
NM_0190	chr8	86573935	86743675	-	4327	1	CNGB3|A	0.018	0
NR_03964	chr3	1.14E+08	1.14E+08	+	66	1	MIR4446|	0	0
NM_0013	chr7	1.5E+08	1.5E+08	-	1166	1	ZNF467|E	6.104	7.365
NM_0012	chr15	63323531	63382110	-	3985	1	CA12|CA-	7.445	7.376
NM_0013	chr2	73926826	73958961	+	1005	1	DGUOK|M	126.536	123.859
NM_0051	chr11	65961689	65980136	+	3579	1	SART1|Ar	20.585	19.805
NM_2136	chr7	99472892	99487594	+	1596	1	ZNF789|-|	4.54	4.008
NM_0073	chr14	60509220	60511807	+	1471	1	SIX6|MCO	0	0.019
NM_0011	chr7	1567334	1570032	-	773	1	PSMG3|C	32.742	43.076
NM_0147	chr15	64365012	64381510	-	1487	1	PCLAF|KIA	6.13	4.348
NM_0011	chr13	27435639	27450197	-	992	1	MTIF3|IF3	18.963	21.912
NM_0011	chr1	88979456	88992960	-	5082	1	RBMXL1|R	5.28	5.239
NR_10274	chr5	10440735	10441851	-	801	1	ROPN1L-A	0.048	0.068
NM_0010	chr11	56030419	56031393	+	973	1	OR5AS1|O	0	0
NM_0011	chr5	1.8E+08	1.8E+08	-	1200	1	C5orf60|-|	0.054	0.011
NR_11081	chr3	1.33E+08	1.33E+08	-	2335	1	TMEM108	0	0
NM_0010	chr4	1.46E+08	1.47E+08	-	3766	1	SLC10A7|	0.644	0.811
NM_0013	chr12	9067708	9116229	-	4620	1	A2M|A2M	0.603	1.856
NM_0060	chr22	36560869	36702645	-	4517	1	CACNG2|N	0.146	0.358
NM_0144	chr19	51450997	51458454	-	2940	1	SIGLEC8|S	0	0
NM_2073	chr1	6206129	6221299	+	3951	1	RNF207|C	1.119	1.085
NM_0012	chr2	2.38E+08	2.38E+08	-	1408	1	HES6|C-H	313.775	141.893
NM_2075	chr2	97734406	97739860	+	1439	1	ZAP70|AD	0.027	0.114
NM_0012	chr8	1.3E+08	1.3E+08	-	3910	1	FAM49B|E	24.372	24.493
NM_0048	chr11	58526871	58575917	-	1878	1	LPXN|LDP	2.52	3.391
NM_1829	chr3	47228026	47282847	-	3334	1	KIF9|-|-|3	2.904	2.551
NM_0011	chr11	1.08E+08	1.08E+08	+	2954	1	ELMOD1|-	2.248	5.068
NR_03369	chr8	43056299	43085788	+	1600	1	FNTA|FPT	16.657	19.184
NM_0013	chr10	1.25E+08	1.25E+08	-	3651	1	METTL10|	3.316	4.404
NM_0012	chr2	3703592	3847410	+	1469	1	DCDC2C|-	0.044	0
NM_2070	chr16	2771414	2777296	-	594	1	ELOB|SIII|	375.474	300.412

NR_13567	chr17	48874860	48908983	-	734	1	LOC10537	0.386	0.149
NR_12588	chr4	47831345	47896848	+	623	1	LOC10192	1.697	2.594
NM_0216:	chrX	1.01E+08	1.01E+08	+	2066	1	TMEM35A	77.829	122.497
NM_0211(	chr2	84905639	84906675	+	477	1	TMSB10|N	4607.405	3450.671
NR_10227	chr9	99355340	99375257	-	1675	1	NAMA|-|-	0.046	0.049
NM_0160:	chr6	89326625	89352900	-	4332	1	UBE2J1|C(	17.778	14.582
NM_0240:	chr9	21440454	21441316	+	860	1	IFNA1|IFL|	0	0
NR_12034	chr15	27157735	27161319	-	847	1	GABRG3-A	0	0
NM_0027(	chr11	67398181	67401905	-	1461	1	PPP1CA|P	97.226	69.938
NR_02768	chr11	55978703	55986405	-	1829	1	OR7E5P|O	0	0
NM_0012!	chr16	21197452	21214510	-	2700	1	ZP2|ZPA|Z	0	0
NM_0010(	chrY	23219457	23291356	+	3756	2	DAZ2|pDP	0	0
NM_1458(	chr15	76336724	76342475	+	1872	1	ISL2|-|-|1!	0.365	0.468
NR_11081	chr3	1.77E+08	1.77E+08	-	2397	1	LINC0120!	0	0
NR_10988	chr20	23356594	23358127	-	633	1	LINC01431	0.754	0.368
NR_03615	chr19	46709293	46709345	-	52	1	MIR320E|I	0.496	0
NM_0328:	chr2	85753786	85791383	+	5858	1	ATOH8|HA	3.801	1.073
NM_0013:	chr21	43053191	43076392	-	2363	2	CBSL|-|-|2	1.091	1.652
NM_0228:	chr11	62575045	62591637	-	2815	1	TUT1|PAP	14.478	13.167
NR_01536	chrX	56729285	56817571	+	691	1	NBDY|LIN(	86.579	75.879
NR_12051	chr7	54330697	54349845	+	672	1	LINC01445	0	0
NR_11198	chr17	35756249	35795707	-	2243	2	MMP28|E	0.006	0.171
NM_0042:	chr14	32329273	32833062	+	10371	1	AKAP6|AD	21.185	11.909
NM_0050:	chr14	22766522	22771789	+	1707	1	OXA1L|OX	48.847	48.04
NM_0063(	chr17	48724765	48728749	-	3032	1	HOXB13|P	0.043	0.203
NR_03962	chr1	94745860	94745900	-	40	1	MIR378G|	0	0
NM_0025!	chr2	1.48E+08	1.48E+08	-	6578	1	ORC4|ORC	5.882	8.389
NM_0013(	chr11	67483029	67491108	+	1222	1	AIP|ARA9|	53.642	45.283
NR_03016	chr14	1.01E+08	1.01E+08	+	72	1	MIR485|M	0	0
NM_0011:	chr13	57140918	57143939	+	1842	5	PRR20D|-|	0	0
NM_0034(	chr19	39406847	39409412	+	1748	1	ZFP36|G0!	2.375	2.21
NM_0048:	chrY	18025787	18027746	+	1957	2	CDY2A|CD	0	0
NM_0023:	chr6	1.6E+08	1.6E+08	+	1131	1	MAS1|MA	0	0.048
NM_0011(	chr6	72216442	72403143	+	5028	1	RIMS1|CO	1.762	1.978
NM_1833!	chr3	62398346	62875389	-	5216	1	CADPS|CA	18.475	29.295
NM_0163:	chrX	1.04E+08	1.04E+08	-	3794	1	RAB9B|RA	5.098	4.859
NR_12871	chr22_KI2	170215	170275	+	60	4	MIR3687-2	0	0
NR_00371	chrX	52063347	52063473	-	126	2	SNORA11D	0	0
NR_13568	chr15	45705195	45931069	+	650	1	LOC10537	0	0.063
NR_12043	chr12	65466819	65642372	-	861	1	LOC10050	0	0
NM_2069:	chrX	21855987	21858727	+	2738	1	YY2|ZNF6:	0.311	0.395
NR_13524	chr14	1.01E+08	1.01E+08	+	513	1	LOC10050	0	0
NM_0176	chr17	51177425	51260066	-	5363	1	MBTD1|SA	8.155	10.538
NR_00256	chr11	62853326	62853393	-	67	1	SNORD31|	10.199	9.608
NM_0013(	chr13	50822905	50843939	-	1364	1	DLEU7|-|-	0.501	0.221
NR_03396	chr3	40173147	40309698	-	2109	1	EIF1B-AS1	0.691	0.468
NM_0153:	chr19	19320821	19358754	+	4832	1	MAU2|KIA	22.865	20.466
NM_0013(	chr7	1.52E+08	1.52E+08	-	2145	1	PRKAG2|A	9.028	9.317
NM_0012!	chr16	4848380	4882401	+	5618	1	UBN1|VT|	15.753	14.987
NM_0246:	chr2	17663812	17753829	-	5143	1	SMC6|SM(	1.524	1.683

NM_1392(	chr22	50059391	50085352	-	3585	1	MLC1|LVM	1.978	7.924
NM_0010:	chr2	10056799	10080411	-	2714	1	CYS1|-|-|2	0.233	0.53
NR_04683	chr2	1.97E+08	1.97E+08	-	1063	1	ANKRD44-	0	0.052
NM_0011!	chr11	65181679	65212006	+	3007	1	CAPN1|CA	53.525	51.531
NR_00214	chr7	1.29E+08	1.29E+08	+	1725	1	LOC40783.	11.032	10.076
NM_1457!	chr1	2.11E+08	2.11E+08	+	3963	1	TRAF5|MG	0.618	1.082
NM_0010{	chr12	57472255	57478198	-	2320	1	ARHGAP9|	0.317	0.23
NM_0031.	chrX	48696733	48709015	+	2728	1	SUV39H1|	3.394	2.646
NM_0050(	chr21	36699133	36749917	+	4430	1	SIM2|HM(	0.39	0.331
NR_04628	chr5	14712694	14716529	+	2597	1	LOC10013(	3.073	3.581
NR_02802	chr11	85657564	85665138	-	4501	1	CREBZF|SN	6.967	8.454
NM_0029(	chr17	9897710	9905367	-	1170	1	RCVRN|RC	0	0.176
NM_0011:	chr12	10929236	10934845	+	3171	3	PRH2|PRP	0	0
NM_0013‹	chr16	53365392	53371212	-	1280	1	LOC64380.	0	0
NR_12593	chr4	1.19E+08	1.19E+08	-	1910	1	LOC10192!	0.014	0.007
NM_0013:	chr11	47617306	47642654	-	2587	1	MTCH2|HS	68.455	64.069
NM_0209:	chr6	36740778	36839444	-	3316	1	CPNE5|CO	1.894	5.613
NM_0012(	chr7	96481626	96503523	-	2700	1	C7orf76|-|	0	0.025
NM_1530‹	chr15	92471677	92501117	+	1721	1	C15orf32|	0.127	0.08
NR_04661	chr13	46852140	46856299	+	438	1	HTR2A-AS:	0	0
NM_0318!	chr19	53992288	54012666	+	1633	1	CACNG6|-	0.363	0.143
NR_04980	chr8	41271048	41271143	-	95	1	MIR548AC	0	0
NM_0190{	chr19	39385630	39391195	-	2272	1	PAF1|F231	50.779	52.787
NR_13089	chr17	17759154	17766302	-	716	1	RAI1-AS1|-	0	0
NM_1343.	chr1	2.06E+08	2.06E+08	-	4598	1	SLC26A9|-	0.022	0.069
NM_0010(	chrX	47974851	48003978	-	3480	1	ZNF182|H	1.704	2.118
NM_0032{	chr20	41028818	41124486	+	3714	1	TOP1|TOP	17.059	18.436
NM_0011!	chr10	35008551	35074330	-	4136	1	CUL2|-|-|:	6.154	6.928
NM_0203!	chr3	99638596	99796733	+	3023	1	COL8A1|C	0.06	0.054
NM_0011(	chr17	28724348	28726931	-	882	1	TLCD1|-|-	2.587	0.575
NM_1782:	chr17	74935802	74949992	+	2358	1	OTOP3|-|-	0	0
NM_0140:	chr12	50924751	50932517	+	3386	1	METTL7A|	0.807	1.784
NM_0013.	chr8	17229229	17246617	-	2530	1	CNOT7|CA	24.717	23.994
NR_03986	chr15	51242190	51242264	+	74	1	MIR4713|-	0	0
NM_0060!	chr2	2.32E+08	2.32E+08	-	3269	1	NMUR1|(F	0.024	0
NM_0011(	chr15	78092585	78104051	+	1884	1	SH2D7|-|-	0.534	0.938
NM_0010(	chr11	46743534	46846308	-	6668	1	CKAP5|CH	47.991	46.013
NM_0010(	chr14	73275210	73458580	-	3454	1	NUMB|C1‹	12.251	12.269
NM_0013:	chr8	86472349	86514348	-	952	1	RMDN1|C(	9.643	10.071
NR_02336	chr1	2.29E+08	2.29E+08	-	120	10	RNA5S3|R	0	0
NM_1816(	chr21	30487190	30487348	-	157	1	KRTAP19-2	0	0
NM_0012!	chr17	40287894	40303161	+	3039	1	CDC6|CDC	4.264	2.506
NM_0010(	chr6	29111810	29112884	+	1071.9	8	OR2J3|6M	0	0
NR_13367	chr6	31542304	31543138	+	525	7	DDX39B-A	0	0
NR_13512	chr9	1.34E+08	1.34E+08	+	2109	1	LOC10013(	0.202	0.156
NM_0010!	chr7	1542235	1556430	-	6266	1	TMEM184	0.982	0.708
NM_0806.	chr2	2.23E+08	2.23E+08	+	3171	1	KCNE4|MI	0.085	0.078
NM_0027(	chr2	44168803	44231473	+	2673	1	PPM1B|PF	19.077	15.803
NM_1829.	chr14	93347191	93348354	+	527	2	COX8C|CC	0	0
NR_00274	chr6	85677294	85677368	-	74	1	SNORD50/	0.348	0.925

NR_02422	chr19	47923779	47923899	+	120	10	SNAR-A8|-	0	0
NM_0013:	chr22_KI2	21488	37822	-	546	2	PVALB|D2	0	0
NM_0063	chr1	11674480	11681214	-	1108	1	MAD2L2|F	66.725	75.865
NR_12055	chr11	74311362	74324705	+	2150	1	LOC10192	0	0.006
NM_0057	chr7	99374675	99394781	+	1519	1	ARPC1B|A	4.516	4.184
NR_03019	chr19	53697533	53697617	+	84	1	MIR525|M	0	0
NM_1759	chr15	45129994	45165578	+	5451	1	DUOX1|LN	1.597	1.656
NM_0011	chr16	20536761	20576373	-	2390	1	ACSM2B|A	0.032	0
NM_0013:	chr9	1.14E+08	1.14E+08	-	4999	1	AKNA|-|-|	3.144	7.625
NR_13581	chr1	2.12E+08	2.12E+08	+	929	1	LOC10272	0	0
NR_00307	chr20	2654212	2654286	+	74	1	SNORD11C	3.659	3.332
NM_0132	chr11	65040904	65044828	+	1563	1	SAC3D1|H	16.688	13.881
NM_0068	chr6	16238580	16295549	+	1504	1	GMPR|GM	0.36	1.694
NM_0146	chr3	1.98E+08	1.98E+08	-	9255	1	RUBCN|KI	7.045	10.169
NM_0056	chr15	23565307	23568019	+	2710	1	MKRN3|C	3.583	5.004
NR_11025	chr2	1.56E+08	1.56E+08	-	1803	1	LINC0187€	0.508	0.365
NM_0041	chr17	82078338	82098230	-	8413	1	FASN|FAS	96.59	73.719
NM_0208	chr20	48624255	48827883	-	6591	1	PREX1|P-R	16.192	20.555
NM_0252	chr2	98997261	99154724	-	3662	1	TSGA10|C	0.215	0.277
NM_0003	chrX	38269170	38327535	-	3051	1	RPGR|COD	1.166	1.127
NM_0010	chr11	17074392	17077673	-	521	1	RPS13|S13	315.432	296.471
NM_0055	chr5	96935642	97029411	+	4450	1	LNPEP|CA	1.628	0.911
NR_13632	chr2	1.45E+08	1.45E+08	-	335	1	LOC10537	0.654	1.554
NR_12042	chr13	46455204	46466776	-	928	1	LINC01198	0.042	0.148
NM_0184	chr7	1.17E+08	1.17E+08	+	2026	1	ST7|ETS7q	46.61	59.919
NM_1525	chr11	71787511	71801234	+	2003	1	FAM86C1|	1.481	1.6
NM_0011	chr14	74405893	74426102	-	2601	1	SYNDIG1L|	0.124	0.685
NM_0038	chr15	90388241	90502243	+	7170	1	IQGAP1|H	5.235	5.561
NR_04854	chr3	1.43E+08	1.43E+08	+	514	1	SLC9A9-AS	0	0
NM_0161	chr9	1.36E+08	1.36E+08	-	1855	1	UBAC1|GE	34.997	39.23
NM_0178	chr9	33817184	33920403	+	4450	1	UBE2R2|C	47.738	47.241
NR_02698	chr22	50757086	50799637	+	1516	1	RPL23AP8	4.941	3.939
NM_0529	chr22	41925032	41926817	-	929	1	TNFRSF13	0.347	0.767
NM_0013	chr17	44319625	44324870	-	1549	1	SLC25A39|	52.573	56.698
NR_12602	chr8	28698403	28701464	-	777	1	EXTL3-AS1	0.183	0.035
NR_04703	chr13	1.14E+08	1.14E+08	-	918	1	LINC00454	0	0
NR_10354	chr5	91310914	91314402	-	579	1	LUCAT1|S	0.045	0.237
NR_03621	chr1	31758660	31758735	-	75	1	MIR4254|	0	0
NR_13461	chr22	33108529	33116294	+	546	1	LINC0164C	0	0
NM_0163	chr16	25111726	25178230	+	1357	1	LCMT1|CG	68.258	67.809
NM_0011	chr14	67619862	67651719	+	1970	1	ARG2|-|-|	48.537	57.757
NM_0006	chr17	6996065	7010736	+	2342	1	ALOX12|1	0.595	0.626
NM_0012	chr9	1.29E+08	1.29E+08	+	2535	1	SET|2PP2	93.922	93.356
NR_00311	chr7	65751105	65763675	+	1754	1	CCT6P1|C	2.58	2.741
NR_00321	chr14	1.01E+08	1.01E+08	+	70	1	SNORD114	0	0
NR_03173	chr19	1816159	1816238	-	79	1	MIR1909|	1.959	0.173
NM_0211	chr1	11189289	11195981	+	2283	1	ANGPTL7|	0.141	0.018
NR_03015	chr11	2134134	2134209	-	75	1	MIR483|M	0	0
NR_13227	chr17	64145970	64146309	+	109	1	SNHG25|-	3.312	2.262
NR_13508	chr1	15153711	15220480	+	1745	1	TMEM51|	3.561	5.487

NM_0013:	chr15	58595204	58749978	-	4924	1	ADAM10|/	8.633	7.766
NM_0011!	chr12	57516588	57520517	-	891	1	DDIT3|CEE	53.267	65.81
NM_0134	chr12	56595596	56636379	-	8903	1	BAZ2A|TIF	29.7	26.449
NM_0156:	chr10	95663396	95694143	-	2723	1	TCTN3|C1	21.682	20.598
NM_0219	chr1	1.57E+08	1.57E+08	+	3454	1	BCAN|BEH	1.672	19.066
NM_0044(	chr13	91398619	92867234	+	2932	1	GPC5|-|-|	0.466	1.196
NM_0153	chr12	1.3E+08	1.31E+08	-	6301	1	RIMBP2|P	0.469	1.55
NM_0011:	chr1	1173906	1197933	+	2241	1	TTLL10|TT	0.017	0
NM_0027!	chr20	62136727	62143458	-	1023	1	PSMA7|C6	257.693	240.227
NM_0013:	chr2	68157830	68176238	+	2221	1	PNO1|KHF	5.323	5.032
NM_0011:	chr7	98846488	98870050	-	2714	1	TMEM130	30.813	39.915
NR_10401	chr19	10832067	10835740	-	1563	1	TMED1|IL:	12.984	13.513
NM_0047:	chr7	1E+08	1E+08	+	1851	1	AP4M1|CF	23.983	21.511
NR_13552	chr21	24428740	24445084	+	1592	1	LINC01684	0	0.009
NM_1524:	chr8	43140449	43202827	+	5194	1	HGSNAT|H	16.6	16.339
NR_11015	chr2	8670984	8681863	-	790	1	ID2-AS1|-|	0.62	1.335
NM_0120!	chr11	64318088	64321823	+	897	1	PRDX5|AC	316.803	225.764
NM_0150:	chr19	18097793	18151689	+	5864	1	MAST3|-|-	6.247	8.544
NM_0202:	chr17	44191805	44198161	-	3600	1	ATXN7L3|-	78.081	83.807
NM_0178!	chr15	65258094	65286843	-	2694	1	PARP16|A	5.786	5.608
NM_0038(	chr6	3063888	3115187	+	4083	1	RIPK1|RIP	3.755	3.556
NR_12038	chr16	88742767	88745748	+	2134	1	LOC33905!	0.58	0.321
NM_0026:	chr1	2404802	2412571	-	2006	1	PEX10|NA	30.036	29.606
NM_0220!	chr16	23754627	23758951	+	2373	1	CHP2|-|-|	0	0
NM_0011(	chr8	7357976	7362968	-	918	2	ZNF705G|-	0	0
NM_0063:	chr4	1.28E+08	1.28E+08	-	3785	1	PGRMC2|I	8.468	11.059
NM_0210(	chr8	1.45E+08	1.45E+08	-	6356	1	ZNF250|ZI	7.85	8.075
NR_02948	chr13	91350605	91350688	+	83	1	MIR17|MI	0	0
NM_1759:	chr6	1.66E+08	1.66E+08	-	2701	1	PRR18|-|-	0.368	0.385
NR_12066	chr17	5019215	5020093	-	580	1	LOC10272	3.023	1.984
NR_12056	chr11	78533176	78558565	+	713	1	LOC10192	0	0
NM_0250:	chr4	78057570	78544269	+	15548	1	FRAS1|-|-	7.187	2.478
NM_0040!	chr8	38030502	38060365	+	854	1	EIF4EBP1|	39.481	49.848
NM_0174!	chr4	10074339	10116949	-	3126	1	WDR1|AIP	65.676	70.133
NM_0012!	chr6	24775413	24786097	+	1034	1	GMNN|Ge	13.205	10.452
NR_12592	chr4	1E+08	1E+08	+	521	1	LOC10192!	0.074	0.026
NR_02996	chrX	1.35E+08	1.35E+08	-	90	1	MIR450A1	0	0
NM_0011(	chr22	39097224	39104067	+	1039	1	APOBEC3H	0.05	0.013
NR_03975	chr18	13611114	13611200	+	86	1	MIR4526|	0	0
NM_0188:	chr5	1.39E+08	1.39E+08	+	5148	1	MATR3|AL	98.817	92.936
NR_03974	chr17	29390662	29390730	+	68	1	MIR4523|-	2.086	1.813
NR_03161	chr8	1.28E+08	1.28E+08	+	72	1	MIR1208|	0	0
NM_0013(	chr6	35214062	35253079	+	7686	1	SCUBE3|C	3.42	2.272
NM_0061	chr19	38801671	38813100	-	1267	1	LGALS4|G/	0.407	0.378
NR_04981	chr1	1.61E+08	1.61E+08	+	75	1	MIR5187|	1.719	1.461
NM_1774(	chr19	1248553	1259143	+	3783	1	MIDN|-|-|	75.115	54.545
NR_02849	chr10	87818313	87845612	+	2116	1	CFL1P1|CF	0.451	1.185
NM_0013(	chr6	82892398	83065841	-	1867	1	UBE3D|C6	4.91	4.035
NM_0211(	chr20	35668688	35699365	-	2370	1	NFS1|HUS	8.443	12.431
NM_0025(	chrX	70258166	70259804	-	1636	1	P2RY4|NR	0.063	0

NR_07342	chr14	68979682	68987463	+	595	1	ACTN1-AS1	0	0.023
NM_07847	chr8	38988808	38996522	-	3246	1	TM2D2|BL	10.558	13.026
NM_0011(	chr7	20647343	20660394	+	2239	1	ABCB5|AB	0.035	0.006
NR_03741	chr8	27701677	27701759	+	82	1	MIR3622A	0	0
NM_0010(	chr1	1.2E+08	1.2E+08	-	1601	1	NBPF7|-|-	0	0.017
NR_03651	chr17	82442586	82450831	-	1982	1	C17orf62|	35.421	30.248
NR_03032	chr7	74191198	74191294	+	96	1	MIR590|M	0.269	1.712
NM_2069:	chr13	1.08E+08	1.08E+08	-	3972	1	LIG4|LIG4	2.967	2.731
NM_0220(	chr11	6680385	6683401	-	2361	1	MRPL17|L	20.277	19.487
NM_0006(	chr7	1.01E+08	1.01E+08	+	3179	1	SERPINE1|	0.681	0.668
NR_03744	chrX	1.21E+08	1.21E+08	+	81	1	MIR3672|-	0	0
NR_10346	chr1	1.47E+08	1.47E+08	-	1143	1	NBPF13P|-	0.045	0.036
NM_0012!	chr19	29923644	30016612	+	2483	1	URI1|C19c	10.526	11.485
NM_0183!	chr19	53107879	53132920	-	2292	1	ZNF415|Zf	4.703	6.323
NM_0013:	chr4	61201256	62078335	+	12610	1	ADGRL3|C	14.313	5.782
NR_02731	chr6	1.43E+08	1.43E+08	-	2290	1	LOC15391(	0.011	0
NR_12207	chr3	42770612	42773634	+	894	1	LINC0215ɛ	0.014	0.138
NM_0011:	chr3	1.98E+08	1.98E+08	-	1970	1	IQCG|CFAI	12.527	6.716
NM_0025:	chr17	80466833	80476604	-	5430	1	NPTX1|NP	9.989	12.968
NR_02741	chr18	15313556	15325919	-	2091	1	LOC64466!	0	0
NR_11008	chr20	63808076	63823007	+	1085	1	ZBTB46-AS	0.024	0
NR_02703	chr3	1.52E+08	1.52E+08	-	6643	1	MBNL1-AS	0.171	0.318
NR_03976	chr1	17277889	17277975	+	86	1	MIR3972|-	0	0
NM_0011:	chrX	1.13E+08	1.13E+08	-	6933	1	AMOT|-|-	6.827	5.755
NM_0012ɛ	chr5	36192589	36241391	-	3603	1	NADK2|C5	3.285	5.721
NR_10975	chr1	22025504	22031224	+	1079	1	LINC0033!	11.065	12.123
NM_0162	chr18	32091855	32131561	+	3560	1	RNF138|H	2.901	2.612
NR_03623	chr5	1.77E+08	1.77E+08	-	61	1	MIR4281|-	0	0
NR_10274	chr5	1.4E+08	1.4E+08	-	2497	1	LINC01024	0.098	0.121
NR_13518	chr16	87317509	87318043	+	399	1	LOC10192:	1.228	1.648
NM_0187(	chr10	1177313	1737476	-	8414	1	ADARB2|A	0.981	1.761
NM_0150(	chr2	2.08E+08	2.08E+08	+	9857	1	PIKFYVE|C	4.725	4.686
NR_10698	chr6	33899135	33899200	+	65	1	MIR7159|I	0	0
NM_1730ɛ	chr12	52468516	52473785	-	2278	1	KRT6C|K6I	0	0
NR_11056	chr2	67086446	67289244	+	681	1	LINC0182ɛ	0	0.161
NM_0011:	chr17_KI2	2707025	2710253	-	3226	2	EPOP|C17	0	0
NM_0040(	chr2	2.15E+08	2.15E+08	+	2066	1	ATIC|AICA	30.929	33.348
NR_10371	chr9	1.16E+08	1.16E+08	-	2448	1	PAPPA-AS1	0.432	0.537
NM_0012	chr19	44474428	44500523	-	4257	1	ZNF180|H	2.19	2.751
NR_03978	chr6	16141556	16141624	+	68	1	MIR4639|-	0	0.201
NR_03036	chr19	10718404	10718503	+	99	1	MIR638|M	0	0
NR_03386	chr6	1.63E+08	1.63E+08	-	4927	1	DKFZp451I	0.021	0.067
NR_04632	chr7	1.33E+08	1.33E+08	+	1993	1	FLJ40288|	0	0
NM_1387!	chr1	2.18E+08	2.18E+08	+	2262	1	SPATA17|I	1.357	0.509
NM_0010ɛ	chr19	15878024	15898120	-	2391	1	CYP4F2|CI	0	0.011
NM_0010:	chr17	78187317	78207701	+	1717	1	AFMID|FK	9.357	11.767
NR_03966	chr6_GL0C	4049281	4049348	-	67	8	MIR3135B	0	0
NR_00335	chr15	25234247	25234328	+	81	1	SNORD115	0	0
NM_0010(	chr2	1.19E+08	1.19E+08	+	3862	1	STEAP3|AI	0.401	0.553
NM_1525!	chr7	1.13E+08	1.13E+08	-	3935	1	BMT2|C7c	4.446	4.33

NM_0009:	chr17	81843158	81860668	-	2565	1	P4HB|CLC	167.957	127.161
NM_0012	chr22	30881681	30907824	+	3234	1	OSBP2|HL	5.96	8.818
NM_0010:	chr2	38743599	38751494	-	2479	1	SRSF7|9G	54.778	51.655
NM_0011	chr19	49659569	49665875	-	1263	1	IRF3|IIAE7	33.086	31.905
NM_0044	chr15	52756963	52790012	-	2114	1	ONECUT1|	2.251	0.35
NR_03760	chr1	1.74E+08	1.74E+08	+	693	1	GAS5-AS1|	0.949	1.028
NM_1536	chr14	92322581	92501481	+	9628	1	SLC24A4|	0.015	0.236
NM_0247	chr15	64151717	64157481	+	3605	1	SNX22|-|-	62.947	49.093
NM_0013	chr13	1.02E+08	1.02E+08	-	2589	1	FGF14|FG	6.38	5.767
NM_0055	chr1	1.11E+08	1.11E+08	-	1956	1	KCNA10|K	0.013	0.007
NM_0012	chr20	3795739	3806121	+	2784	1	CDC25B|-|	32.187	24.029
NR_12582	chr8	42255321	42270961	-	646	1	LOC10192	0.06	0.085
NM_0018	chr5	86617967	86620766	+	402	1	COX7C|-|-	339.24	331.011
NM_0011	chr3	1.9E+08	1.9E+08	+	4800	1	TP63|AIS|	0	0
NR_00291	chr17	7577955	7578091	+	136	1	SNORA67|	12.799	11.381
NR_13075	chr16	11345438	11351763	+	1226	1	RMI2|BLA	5.248	3.173
NM_0012	chr8	1.03E+08	1.03E+08	+	1207	1	CTHRC1|-|	0	0.034
NM_1815	chr16	66614750	66696707	-	8111	1	CMTM4|C	5.4	6.929
NM_0158	chr18	54202680	54224788	-	1349	1	MBD2|DM	6.012	5.493
NM_1450	chr16	89969775	89972832	-	3055	1	CENPBD1|	6.322	8.859
NM_0011	chr5	42756818	42802437	+	3480	1	CCDC152|	1.912	3.259
NM_0326	chr16	24539587	24549401	+	1758	1	RBBP6|MY	9.307	8.041
NM_0013	chr5	1.59E+08	1.59E+08	-	5217	1	EBF1|COE	20.597	6.256
NM_1736	chr8	1.25E+08	1.25E+08	+	1202	1	NSMCE2|	17.999	13.355
NR_13553	chr3	57269566	57278127	+	674	1	LOC10537	3.577	3.272
NM_0010	chr9	1.05E+08	1.05E+08	-	955	1	OR13C5|C	0	0
NM_0010	chr19_GL9	321949	343243	+	1843	4	TTYH1|-|-	0	0
NM_0245	chr3	1.02E+08	1.02E+08	+	7680	1	CEP97|28	2.653	2.818
NM_0017	chr5	39284145	39364560	-	2817	1	C9|ARMD	0	0
NM_0012	chr17	77405069	77500596	+	3038	1	SEPT9|AF1	57.681	85.171
NM_0165	chr1	1.81E+08	1.81E+08	+	2339	1	IER5|SBBI	45.885	59.613
NM_0178	chr11	60924441	60937159	+	3507	1	TMEM132	120.927	113.216
NM_0323	chr1	2.33E+08	2.33E+08	+	1317	1	NTPCR|C1	30.643	29.703
NM_0146	chr15	43533462	43584892	-	5530	1	PPIP5K1|H	3.81	3.076
NM_0010	chr1	1.04E+08	1.04E+08	+	1768	3	AMY1A|A	0	0
NM_0011	chr17	75633434	75641406	+	1543	1	SMIM5|C1	0.159	0.115
NM_1525	chrX	1.36E+08	1.36E+08	-	1002.3	3	CT45A2|C	0	0
NR_10671	chr3	50227436	50227490	+	54	1	MIR5787|	0	0
NM_0010	chr8	12064389	12071747	-	237	2	DEFB130|	0	0
NM_1533	chr7	64228474	64266931	+	1560	1	ZNF679|-|	0	0.009
NR_00306	chr19	50799439	50799535	-	96	1	SNORD88	0.806	0
NM_0012	chr5	1.49E+08	1.5E+08	-	2513	1	CSNK1A1|	53.837	59.061
NR_13804	chr6_GL0	2201132	2210014	-	802.6	5	HCG21|N	0	0.017
NM_0228	chr1	2.23E+08	2.23E+08	-	2994	1	AIDA|C1o	2.304	1.912
NR_10988	chr5	1.23E+08	1.23E+08	-	1640	1	LOC10192	0	0
NR_12160	chr9	8858018	8861727	+	1250	1	PTPRD-AS	4.869	3.32
NM_0010	chr11	1.23E+08	1.23E+08	-	698	1	BSX|BSX1|	0	0
NM_0035	chr6	26020490	26020958	+	466	1	HIST1H3A|	0.221	0.294
NM_0179	chr1	1401896	1407313	-	727	1	MRPL20|L	101.41	89.492
NM_0012	chrX	1.29E+08	1.3E+08	-	3947	1	SMARCA1	22.609	21.356

NM_1735;	chr9	1.12E+08	1.12E+08	-	4693	1	C9orf84|-|	0.011	0.003
NR_03396	chr8	1.01E+08	1.01E+08	+	1945	1	FLJ42969|	0.192	0.211
NM_0014	chr1	46394267	46413848	+	2078	1	FAAH|FAA	4.548	7.936
NM_0037	chr12	1.2E+08	1.2E+08	+	717	1	DYNLL1|D	411.548	324.105
NR_12540	chr3	1.79E+08	1.79E+08	-	343	1	LOC10192	0	0.04
NM_0012	chr2	14635091	14650819	+	2054	1	FAM84A|N	4.463	7.035
NM_0024	chr12	56152420	56157987	+	990	1	MYL6B|MI	206.895	204.127
NM_0012	chr17	75888960	75897003	-	3366	1	TRIM65|4	6.159	4.387
NM_0011	chr1	89524838	89597861	+	7727	1	LRRC8B|TA	2.965	2.56
NM_0319	chr17	41232463	41233454	+	989	2	KRTAP9-3|	0	0
NM_0248	chr8	22106872	22109419	-	1514	1	NUDT18|N	3.645	3.293
NM_0013	chr1	1.11E+08	1.11E+08	-	2306	1	WDR77|HI	22.935	21.757
NM_0012	chr6	35794982	35797344	-	432	1	CLPS|-|-|6	0	0
NM_0011	chr22	42639803	42640601	-	796	1	ATP5L2|A	0.032	0.069
NR_03765	chr11	1.12E+08	1.12E+08	+	1522	1	HSPB2-C1	0.042	0.054
NM_0013	chr11	94768342	94876753	+	8835	1	AMOTL1|J	14.535	20.69
NM_0219	chr12	21536189	21604847	-	3114	1	GYS2|-|-|	0.07	0.07
NR_11016	chr2	81461359	81466946	-	840	1	LINC01815	0.092	0
NM_0012	chr19	55158661	55166722	-	2217	1	DNAAF3|C	5.252	2.86
NR_02674	chr1	1.65E+08	1.66E+08	-	1796	1	LOC40079	0	0
NM_0011	chr2	71466702	71686763	+	6622	1	DYSF|FER1	0.084	0.108
NR_10411	chr13	18905439	18926741	+	1075	1	LINC00408	0	0.025
NM_0012	chr22	21467170	21469935	+	1296	1	TMEM191	0.06	0.032
NM_0005	chr2	2.18E+08	2.18E+08	+	3835	1	SLC11A1|L	0.094	0.036
NM_0060	chr6	1.39E+08	1.39E+08	-	1932	1	CITED2|AS	95.433	108.031
NM_0027	chr10	92689924	92695651	+	1753	1	HHEX|HEX	0.044	0.18
NM_1815	chr13	33818069	33966558	+	1450	1	RFC3|RFC3	3.379	4.572
NR_13447	chr7	1.41E+08	1.41E+08	-	3387	1	DENND2A	34.946	28.255
NM_0057	chr2	1.14E+08	1.14E+08	+	5686	1	ACTR3|AR	25.776	24.872
NR_13390	chr6_GL00	4077330	4078179	+	548.5	6	HLA-DQB1	0.658	0.375
NM_0032	chr6	1.08E+08	1.08E+08	+	3201	1	NR2E1|TLI	0.137	2.208
NM_0033	chr16	21953064	21983660	+	2195	1	UQCRC2|N	109.727	125.251
NM_0307	chr5	1.48E+08	1.48E+08	+	4175	1	FBXO38|FI	9.404	10.498
NM_0044	chr9	1.28E+08	1.28E+08	+	3220	1	DNM1|DN	42.729	55.596
NM_0010	chr8	7487669	7489593	+	326	3	DEFB105B	0	0
NM_0010	chr7	1.4E+08	1.4E+08	+	946	1	RAB19|RA	0	0.043
NM_0013	chr19	42247561	42253899	-	2581	1	ERF|CHYTS	13.223	10.991
NM_0011	chr20	18137855	18143168	+	1154	1	PET117|CS	14.525	17.863
NR_03614	chr16	14911220	14911313	+	93	7	MIR3180-3	0	0
NR_04679	chr2	70467385	70481640	-	489	1	TGFA-IT1|	0	0
NM_0024	chr21	41362023	41408942	+	2945	1	MX2|MXB	0	0.005
NR_02952	chr3	1.6E+08	1.6E+08	+	80	1	MIR16-2|N	0	0
NM_0013	chr6	43012013	43013983	-	1067	1	MEA1|HYS	115.908	91.168
NM_0010	chr1	23795599	23800804	-	1552	1	GALE|SDR	31.968	24.067
NR_02969	chr9	1.37E+08	1.37E+08	+	84	1	MIR126|M	0	0
NR_12165	chr5	1.31E+08	1.31E+08	+	6091	1	LYRM7|C5	1.291	1.633
NM_0034	chr16	11668433	11679161	+	3291	1	SNN|-|-|1	121.483	135.401
NM_0011	chr15	72284727	72306315	-	2842	1	CELF6|BRU	23.024	22.502
NM_0229	chr7	94509858	94557016	+	3859	1	CASD1|C7	6.194	6.538
NM_0186	chr15	34341716	34343161	-	531	1	NOP10|DK	175.992	143.574

NM_0161(	chr20	20017290	20033629	+	1078	1	NAA20|NA	31.145	32.019
NM_0010:	chr7	964850	975599	-	4886	1	COX19|-|-	7.104	7.802
NM_0028:	chr19	797392	812327	+	3264	1	PTBP1|HN	55.986	41.939
NR_12872	chr15_KI2	271844	271925	+	81	5	MIR5701-3	0	0
NR_13453	chr16	29679008	29698699	+	1862	1	QPRT|HEL	24.007	18.787
NM_0306	chr7	1.4E+08	1.4E+08	-	9156	1	KDM7A|JH	3.374	2.53
NM_0011	chr18	12446512	12656732	-	5082	1	SPIRE1|Sp	11.47	44.983
NM_0011	chrX	1.01E+08	1.01E+08	+	2530	1	RPL36A-HI	48.221	50.143
NM_0012	chrX	10156945	10237659	+	6520	1	CLCN4|CL(	12.182	10.718
NR_03653	chr3	1.5E+08	1.5E+08	+	1963	1	LOC64690	3.954	3.956
NM_1775(	chr20	482694	543838	-	2495	1	CSNK2A1|	39.326	49.425
NM_0042	chr19	44286348	44305025	-	3183	1	ZNF235|AI	1.568	2.573
NR_03999	chr9	70216059	70258874	-	1035	1	SMC5-AS1	0.797	0.529
NR_12594	chr1	2.35E+08	2.35E+08	-	884	1	LOC10192	0	0
NM_0804	chr20	62463497	62475970	-	2586	1	GATA5|GA	0.06	0.011
NM_0039	chr16	68264516	68301823	+	6256	1	SLC7A6|LA	14.52	16.357
NM_0073	chr22	19962547	19969975	+	2029	1	COMT|HEI	13.961	2.288
NM_0013(	chr2	27275421	27281431	+	1938	1	DNAJC5G|	0.04	0.141
NM_0319	chr4	15960240	15963236	-	1167	1	FGFBP2|H	2.729	11.537
NR_14451	chr19	21649668	21658608	+	3495.5	2	LOC40068	0.007	0.004
NM_0133	chr19	44094329	44109830	+	3979	1	ZNF224|BI	4.365	9.566
NM_1526	chr4	1.23E+08	1.23E+08	+	3240	1	BBS12|C4	1.651	1.006
NM_0132	chr12	57009997	57016560	-	829	1	TAC3|HH1	4.526	64.206
NR_02338	chr7	5823160	5854435	+	2814	1	ZNF815P|	3.459	3.548
NM_1453(	chr7	1.49E+08	1.49E+08	+	1349	1	C7orf33|-|	0	0.203
NM_0013	chr5	1.29E+08	1.29E+08	+	3042	1	SLC27A6|	0.047	0.032
NM_0184	chr11	85628573	85636539	+	1031	1	TMEM126	10.955	10.309
NM_2075	chr9	1.37E+08	1.37E+08	+	1831	1	LCNL1|-|-	0.042	0.127
NR_10691	chr9	35710676	35710741	-	65	1	MIR6852|	5.951	4.847
NM_0011	chr6	12290296	12297194	+	2090	1	EDN1|ARC	0.086	0.079
NM_0011	chr20	56412258	56459340	+	2985	1	CASS4|C2(	0.039	0.055
NM_0011	chr4	1.02E+08	1.02E+08	-	2597	1	SLC39A8|E	0.209	0.116
NM_1457	chr11	65354811	65357611	+	1962	1	TIGD3|-|-|	7.36	6.06
NM_1723	chr2	42442017	42494097	-	3787	1	KCNG3|KV	0	0.025
NR_10983	chr8	1.27E+08	1.27E+08	+	12721	1	PRNCR1|C	0.151	0.208
NM_0145	chr7	92468381	92471424	-	3041	1	ERVW-1|E	0.038	0.054
NR_03993	chr2	1.02E+08	1.02E+08	+	77	1	MIR4772|	0	0
NM_0020	chr6	1.16E+08	1.16E+08	-	2839	1	FRK|GTK|I	0.104	0.048
NR_12165	chr10	1.17E+08	1.17E+08	-	563	1	MIR3663H	0	0
NM_0163	chr9	1.29E+08	1.29E+08	-	4272	1	SPOUT1|C	15.414	14.685
NM_1536	chr12	38652200	38905618	-	3439	1	CPNE8|-|-	0.176	0.104
NR_13091	chr17	76671928	76673658	+	1730	1	LOC10527	1.088	0.903
NR_02708	chr20	22400351	22420643	-	1662	1	LOC28478	0	0
NM_0010(	chr7	99876062	99877033	-	970	1	OR2AE1|C	0	0.014
NM_0252	chr9	5510545	5571282	+	2409	1	PDCD1LG2	0	0.017
NM_0012	chr11	33256672	33357022	+	7104	1	HIPK3|DYI	2.996	3.025
NM_0052	chr12	50103819	50111320	+	3073	1	GPD1|GPD	0.579	4.35
NM_0012	chr17_KI2	2332054	2358166	+	1546	2	MRPL45|L	1.143	1.515
NM_0072(	chr3	1.31E+08	1.32E+08	-	1752	1	MRPL3|CC	32.535	35.407
NR_03974	chr16	2133119	2133204	+	85	1	MIR4516|	0	0

NR_02708	chr1	31518435	31524245	+	2738	1	LINC0122€	0	0.005
NM_0011‹	chr5	1.5E+08	1.5E+08	+	4954	1	TCOF1|MF	18.06	15.469
NM_0013(	chr12	32502043	32638929	+	2799	1	FGD4|CM	2.828	6.89
NM_0012ε	chr12	1.11E+08	1.11E+08	+	1558	1	RAD9B|-|-	0.19	0.229
NM_0010‹	chrX	1.02E+08	1.02E+08	+	2376	2	NXF2B|TC	0	0
NM_0011	chr16	87951432	88077318	+	2196	1	BANP|BEN	9.335	8.526
NM_0133	chr5	1.41E+08	1.41E+08	+	2584	1	PCDHB1|P	0	0.005
NM_0012	chr19	49555468	49580572	-	1237	1	NOSIP|CG	64.926	57.357
NM_0013(	chr14	64945814	64972157	-	3251	1	RAB15|-|-	38.688	46.345
NM_0011‹	chr1	6625150	6633582	+	1284	1	THAP3|-|-	21.891	24.14
NM_1527	chr7	2537810	2555758	-	2997	1	BRAT1|BA	18.207	17.522
NR_03075	chr12	54033950	54034045	+	95	1	MIR615|M	0	0
NR_13855	chr10	80131683	80145064	+	529	1	PLAC9|-|-	0.341	0.181
NM_0012	chr12	1.11E+08	1.11E+08	-	1601	1	PPP1CC|P	44.769	49.304
NM_0528	chr9	98120975	98192674	-	5480	1	CORO2A|(	1.405	0.995
NM_0012	chr1	8945834	8975089	+	1028	1	CA6|CA-VI	0	0
NM_1450	chr11	1.26E+08	1.26E+08	+	1806	1	HYLS1|HLS	0.243	1.926
NR_03962	chr2	35471405	35471486	+	81	1	MIR548AD	0	0
NM_1816	chr21	30371391	30372239	-	846	1	KRTAP13-2	0	0
NR_07351	chr21	35372507	35580764	-	454	1	LOC10050	0	0
NR_03622	chr2	1.1E+08	1.1E+08	-	81	1	MIR4267|	0	0
NM_2068	chr17	30316348	30334059	-	1204	1	TMIGD1|T	0	0
NM_0024	chr11	17719563	17722131	+	1801	1	MYOD1|M	0	0
NR_13625	chr7	63346802	63350066	-	3264	1	LOC10272	0	0
NM_1452	chr19	51097606	51108390	-	2102	1	CTU1|ATP	2.582	2.808
NR_12582	chr8	92565443	92655494	-	582	1	LOC10272	0	0
NR_10275	chr5	55995167	56003647	+	2285	1	FLJ31104|	0.226	0.156
NM_1830	chr10	35137782	35212958	+	2317	1	CREM|CRE	5.292	4.522
NM_0013	chr7	75878992	75888926	+	1314	1	RHBDD2|N	118.671	114.288
NM_0013	chr21	7816677	7829632	-	3218	1	KCNE1B|-|	0.008	0.026
NM_0011	chr3	38548062	38649673	-	8314	1	SCN5A|CD	0.078	0.288
NR_02966	chr18	58451074	58451158	+	84	1	MIR122|M	0	0
NM_0530(	chr22	19130808	19132623	+	1813	1	TSSK2|DG	4.089	6.701
NR_10681	chr12	1.12E+08	1.12E+08	+	71	1	MIR6761|	19.068	24.307
NM_0012	chr12	9715860	9733299	-	1566	1	CLECL1|D(	0.016	0.009
NM_1334	chr18	59430939	59697412	-	6247	1	CCBE1|HK	0.171	0.217
NM_0011	chr19	3539157	3544030	+	665	1	C19orf71|	9.016	6.776
NM_0011	chr5	1.54E+08	1.54E+08	+	4584	1	MFAP3|-|-	14.353	15.325
NM_0063	chr2	1.12E+08	1.12E+08	+	3605	1	MERTK|M	1.094	2.527
NR_10383	chr9	1.03E+08	1.03E+08	+	579	1	LINC00587	0	0
NR_03998	chr5	8333483	8457564	-	1585	1	LOC72950	0.049	0.078
NM_0012‹	chr5	69280692	69332793	-	3451.5	2	CCDC125|	0.007	0.008
NM_0010(	chr17	36194870	36196748	-	766.6	5	CCL3L3|4€	0	0
NM_0011	chr19	55132698	55149354	-	1163	1	TNNT1|AN	21.075	15.852
NR_02416	chr19	57825299	57827462	-	1641	1	FKBP1AP1	0.982	2.07
NM_0013(	chr1	2.06E+08	2.06E+08	-	2808	1	RAB7B|RA	0.289	0.707
NM_0038	chr18	10525876	10552769	+	3701	1	NAPG|GA	10.598	8.742
NM_0147	chr5	80408013	80479869	+	7272	1	ZFYVE16|F	2.301	2.948
NM_0052	chr16	3652939	3658095	+	3087	1	DNASE1|D	4.895	4.814
NM_1828	chr22	39520564	39522686	+	1415	1	ATF4|CRE	257.851	277.541

NR_13460	chr16	8621640	8646222	+	1549	1	METTL22|	16.656	17.632
NR_02696	chr11_KI2	103465	105541	-	2076.5	2	LOC14366	0.019	0
NM_0039	chr7	99408978	99419616	+	1035	1	BUD31|Cw	104.244	91.896
NM_0010	chr3	1.7E+08	1.7E+08	+	1817	1	LRRIQ4|LR	0	0
NM_0326	chr2	74505043	74507694	-	895	1	PCGF1|20	14.982	14.217
NM_2034	chr13	75525219	75537872	-	1689	1	COMMD6|	37.993	36.631
NM_0012	chr19	36773118	36777078	+	1881	1	LOC72848	3.304	6.007
NR_02951	chr7	1.31E+08	1.31E+08	-	80	1	MIR29B1|	0	0
NM_1449	chr12	71609601	71663969	-	7119	1	ZFC3H1|C	1.605	2.032
NM_0013	chr10	73911101	73917500	+	2340	1	PLAU|ATF	0.656	1.908
NM_0012	chr3	9779964	9793011	-	1898	1	TADA3|AD	76.865	72.447
NM_0147	chr17	47695264	47712063	+	4123	1	TBKBP1|P	12.64	11.242
NM_0201	chr19	39704306	39709448	+	772	1	LGALS14|	0	0
NM_0123	chr16	28537341	28539174	-	853	1	NUPR1|CC	3.235	0.963
NM_0019	chr3	1.87E+08	1.87E+08	+	1877	1	EIF4A2|BN	303.11	283.142
NM_0011	chr9	69324572	69392474	+	2114	1	FAM189A	0.201	0.298
NM_0331	chr17	41249687	41250653	+	964	3	KRTAP9-4|	0	0
NR_03972	chr12	1.28E+08	1.28E+08	+	67	1	MIR4419B	0	0
NM_0203	chr10	99659506	99711245	+	8540	1	ENTPD7|L	2.256	1.638
NM_0013	chr7	1.34E+08	1.34E+08	-	1369	1	AKR1B1|A	95.321	132.338
NM_0042	chr10	1.2E+08	1.2E+08	+	2563	1	BAG3|BAG	9.281	15.038
NM_0169	chr16	67660948	67662778	+	1258	1	PARD6A|P	24.414	18.259
NM_0063	chr14	91869411	91947702	-	2620	1	FBLN5|AD	10.266	24.545
NM_1525	chrY	22168542	22182942	-	1867	2	RBMY1F|Y	0	0
NM_0024	chr15	23685407	23687303	-	1894	1	NDN|HsT1	132.624	131.091
NM_0012	chr22	17580157	17589192	-	1832	1	LOC10192	0.26	0.164
NM_1828	chr1	1727385	1745999	-	6286	1	SLC35E2|-	5.052	4.676
NR_00221	chr7	6735305	6751601	+	1732	1	PMS2CL|P	6.871	5.314
NM_0331	chr13	99963021	99971924	-	4593	1	ZIC5|-|-|1	7.024	4.387
NM_0012	chr5	17216823	17276845	+	1709	1	BASP1|CA	704.577	693.13
NR_11193	chr1	1.48E+08	1.48E+08	-	2119	1	PDZK1P1|	0.037	0.084
NM_2073	chr3	1.52E+08	1.52E+08	+	1493	2	AADACL2|	0	0
NR_10408	chr14	32203164	32203269	+	105.5	11	RNU6-9|U	1.1	0.779
NM_0010	chr14	58004056	58152239	-	3216	1	C14orf37|	9.321	10.068
NR_10461	chr5	1.18E+08	1.19E+08	-	3182	1	HRAT56|-|	0	0
NM_0010	chr12	1.17E+08	1.17E+08	+	828	1	MAP1LC3E	15.665	15.202
NM_0147	chr20	32109506	32167258	+	3958	1	TM9SF4|d	29.8	34.585
NR_12060	chr14	22919456	22923407	+	1992	1	PRMT5-AS	18.175	16.028
NM_1383	chr19	53333719	53356119	+	5585	1	ZNF845|-|	0.713	1.418
NM_0012	chr17	8878916	8965712	-	4322	1	PIK3R5|F7	0	0.01
NM_0010	chr12	55221025	55221975	+	949	1	OR10A7|C	0	0
NR_03715	chr6	1.05E+08	1.05E+08	+	1711	1	BVES-AS1|	0.512	0.544
NM_0189	chr5	1.41E+08	1.41E+08	+	3259	1	PCDHB10|	4.668	9.738
NM_0010	chrX	1.21E+08	1.21E+08	-	1728	1	C1GALT1C	7.917	7.784
NR_10275	chr5	43067023	43093558	+	1545	1	LOC10050	4.832	4.805
NR_03611	chr10	1.02E+08	1.02E+08	+	80	1	MIR3158-1	0	0
NM_0184	chr7	1.51E+08	1.51E+08	+	1029	1	TMEM176	0.313	0
NR_03999	chr16	4245825	4253789	-	2090	1	LINC0156	0.617	0.59
NR_03058	chr20	58818226	58818313	-	87	1	MIR298|M	0	0
NM_0012	chr4	1.29E+08	1.29E+08	+	4059	1	JADE1|PHI	3.567	5.858

NM_0013	chr1	1.59E+08	1.59E+08	+	3446	1	CADM3|BI	127.173	151.852
NM_0177	chr19	48600600	48613437	-	2004	1	FAM83E|-	0.174	0.205
NM_0334	chr16	30553764	30558321	-	2791	1	ZNF764|-|	13.342	11.175
NR_11010	chr15	91022622	91031139	+	1616	1	LOC10192	0.455	0.771
NM_1734	chrX	1.52E+08	1.52E+08	+	3232	1	PASD1|CT	0.008	0
NR_00356	chr17	43800799	43833179	-	2795	1	MPP3|DLG	16.598	16.015
NM_0010	chr1	47137425	47148854	+	1664	1	CYP4A22|	0	0
NM_0069	chr1	1632181	1634654	+	1230	1	MMP23B|	0	0.011
NR_11012	chr14	88018605	88026312	-	423	1	LOC10192	0	0
NM_0011	chr12	1.1E+08	1.1E+08	-	2845	1	TRPV4|BC	0.077	0.053
NR_03750	chr10	1.33E+08	1.33E+08	-	107	1	MIR3944|	0	0.384
NM_1985	chr2	1.51E+08	1.51E+08	-	3908	1	RBM43|C2	0.03	0.067
NM_0328	chr12	34022281	34028301	+	3122	1	ALG10|AL	0.838	0.522
NR_03330	chr2	1.65E+08	1.65E+08	-	134	1	SNORA70F	0	0
NM_0064	chr20	18467389	18484648	+	2148	1	POLR3F|RI	7.371	6.115
NM_0055	chr1	1.59E+08	1.59E+08	+	2721	1	IFI16|IFNG	0.299	0.926
NM_0013	chr2	2.21E+08	2.22E+08	-	6329	1	EPHA4|HE	8.585	29.617
NM_1533	chr6	43298260	43305538	+	2547	1	SLC22A7|	0.086	0.231
NM_1489	chr15_KI2	2646214	2678539	-	2783.5	2	CHRFAM7	0.144	0.025
NM_0011	chr19	51517819	51531856	-	3616	1	SIGLEC6|C	0	0
NR_03963	chr2	88812370	88812454	+	84	1	MIR4436A	0	0
NR_10996	chr5	1.5E+08	1.5E+08	-	3727	1	CSF1R|C-F	0.19	0.301
NM_0068	chr7	27153719	27156677	-	2011	1	HOXA7|AN	0.019	1.219
NM_0209	chr5	61332273	61546172	+	5487	1	ZSWIM6|A	15.525	12.504
NM_0010	chr1	12916647	12920480	+	1552	1	PRAMEF7|	0	0
NM_0012	chr1	2.47E+08	2.47E+08	-	946	1	ZNF695|SE	1.949	2.476
NM_0013	chr10	1.17E+08	1.17E+08	+	1475	1	PNLIPRP1|	0	0.009
NM_0013	chr20	25195670	25228075	+	3740	1	ENTPD6|C	27.18	32.778
NR_01534	chr14	94640725	94646994	+	1713	1	SERPINA13	0	0
NM_0013	chr17	8368653	8376711	-	2542	1	KRBA2|-|-	5.762	3.928
NM_0011	chr1	1.97E+08	1.97E+08	+	1450	1	CFHR3|CF	0	0
NM_1825	chrX	27807990	27823014	+	1948	1	MAGEB10	0	0
NR_12604	chr19	42397128	42408452	+	290	1	LOC10193	5.024	4.204
NM_0008	chr12	96000753	96035661	-	2193	1	LTA4H|-|-	19.226	34.371
NR_13072	chr10	1.01E+08	1.01E+08	-	1908	1	TLX1NB|A	0	0.23
NM_1768	chr11	7959609	7963512	-	2016	2	NLRP10|C	0	0
NM_0011	chr17	40015361	40017813	+	1469	1	CSF3|C17	0.018	0.009
NR_03157	chr19	53688481	53688567	+	86	1	MIR1283-1	0	0
NR_04025	chr10	2005473	2014348	-	2094	1	LINC0070C	0	0
NM_0013	chr1	1.62E+08	1.62E+08	-	2119	1	FCGR3A|C	0	0
NM_0221	chr11	1.03E+08	1.03E+08	-	1635	1	MMP27|M	0	0.008
NR_10243	chr20	62800627	62805587	-	665	1	OGFR-AS1	0.853	0.556
NM_0010	chr6	1.23E+08	1.23E+08	+	11469	1	CLVS2|C6	1.532	1.758
NM_0010	chr14	19876268	19877212	+	943	1	OR4K2|OR	0	0
NR_12047	chr12	9367464	9397617	+	4384	1	LOC10193	0.024	0.009
NR_02438	chr22	19566130	19566839	-	709	1	LINC00895	0	0
NM_0154	chr7	16461481	16465849	-	1865	1	SOSTDC1|	1.092	0.661
NR_03175	chr9	94809962	94810057	+	95	1	MIR2278|	0	0
NM_0175	chr12	95217746	95302790	+	4548	1	VEZT|VEZ	22.289	22.955
NM_0011	chr10	45727200	45792964	+	4390	1	WASHC2C	2.82	2.299

NM_0011	chr12	47076600	47079951	-	3053	1	AMIGO2|A	4.578	2.391
NM_0013	chr3	1.13E+08	1.13E+08	-	4475	1	NEPRO|C3	7.508	8.24
NR_12636	chr13	1.09E+08	1.09E+08	+	410	1	LINC0039	0	0.067
NM_1529	chr2	1.78E+08	1.78E+08	+	1803	1	RBM45|DI	5.135	5.629
NR_02728	chr11	1.23E+08	1.23E+08	+	2045	1	LOC34105	2.238	2.418
NM_0322	chr2	27576522	27582722	+	6198	1	C2orf16|-|	1.864	0.813
NR_13553	chr2	1.03E+08	1.03E+08	-	432	1	LINC01935	0	0
NM_0011	chr4	2418945	2462963	+	2126	1	CFAP99|-|	0.115	0.122
NR_13298	chr16	14302505	14326517	+	535	1	MIR193BH	0.458	0.307
NR_13275	chr11	17332042	17349973	+	3252	1	LOC10537	1.495	2.359
NM_0012	chr15	1.01E+08	1.01E+08	-	4310	1	PCSK6|PA	1.466	1.23
NM_0063	chr6	17393505	17557792	+	3012	1	CAP2|-|-|	9.319	10.741
NR_12057	chr11	81879851	82403913	-	1392	1	MIR4300H	0	0.089
NM_0223	chr16	69328621	69330595	-	1154	1	PDF|-|-|1	16.234	17.756
NR_12158	chr9	1.04E+08	1.04E+08	-	7017	1	SMC2-AS1	0.006	0
NM_0012	chr9	79571773	79726881	+	4981	1	TLE4|BCE-	12.44	12.064
NM_0011	chr2	1.9E+08	1.9E+08	-	2066	1	ORMDL1|-	15.764	16.826
NM_0323	chr17	30970938	30999909	+	2146	1	RNF135|L	0.036	0.134
NM_0122	chr3	1.95E+08	1.95E+08	-	7145	1	ACAP2|CE	1.404	1.967
NM_0013	chr5	88718241	88823927	-	5889	1	MEF2C|C5	3.63	1.835
NM_0140	chr3	42782908	42804535	-	2761	1	HIGD1A|H	17.545	15.126
NM_0048	chr1	1.2E+08	1.2E+08	-	1877	1	SEC22B|EI	10.517	10.092
NR_02422	chr19	47928818	47928937	+	119	3	SNAR-C1|-	0	0
NR_13531	chr16	29425411	29447026	-	3113	1	SMG1P6|-	0.008	0.026
NM_1471	chr8	23020136	23069187	-	4052	1	TNFRSF10I	25.434	30.389
NM_0011	chr4	53377653	53459936	+	2219	1	FIP1L1|FIP	18.89	20.018
NM_0010	chr19	51225079	51240019	+	1077	1	CD33|SIGL	0	0
NR_10701	chr11	1.31E+08	1.31E+08	+	68	1	MIR8052|	0	0
NM_0059	chr3	1.97E+08	1.97E+08	-	3944	1	MELTF|CD	0.83	0.194
NR_02713	chr19	21358933	21388582	+	11716	1	ZNF738|-|	5.28	4.902
NM_0144	chr3	1.51E+08	1.51E+08	-	3167	1	SERP1|RA	16.395	19.575
NM_1527	chr6_GL0	4903302	4906266	+	2707	2	ZBTB9|ZN	0.2	0.187
NM_0246	chr1	26410768	26429728	+	4018	1	LIN28A|CS	0.125	0.116
NM_0010	chr12	7304332	7328373	+	1729	1	ACSM4|-|	0.015	0
NM_0571	chr1	54998944	55008792	+	1364	1	BSND|BAR	0.066	0
NM_1730	chr22	43203223	43343388	-	3844	1	SCUBE1|-|	11.914	14.794
NM_1995	chr3	1.13E+08	1.13E+08	-	4105	1	CCDC80|D	3.483	2.366
NR_02683	chr2	5981978	5988232	+	3460	1	LOC40094	3.425	3.507
NR_02739	chr7	65647854	65718688	+	1755	1	INTS4P2|I	0.169	0.14
NR_03377	chrX	51681213	51682514	-	1301	2	CENPVL2|	0	0
NM_0045	chr10	92593068	92655395	+	5054	1	KIF11|EG5	1.316	2.613
NM_0008	chr1	2019329	2030753	+	1914	1	GABRD|EI	1.132	1.961
NM_0037	chr4	2937937	2963506	-	3011	1	NOP14|C4	8.684	8.966
NM_0013	chr18	79069275	79378282	+	4415	1	ATP9B|AT	3.846	4.471
NM_0017	chr1	39757182	39788861	-	4813	1	BMP8B|BI	1.254	0.939
NR_02667	chr7	23490388	23491412	+	1024	1	RPS2P32|	0.755	0.468
NR_03748	chr9	91636267	91636345	-	78	1	MIR3910-2	0	0
NM_0011	chrX	1.48E+08	1.48E+08	+	4330	1	FMR1|FM	5.607	7.117
NR_11065	chr10	89915489	89957373	-	1856	1	LINC01375	0	0
NM_0012	chr19	9324226	9343845	+	2847	1	ZNF559|N	5.512	4.503

NM_0012(	chr12	19440581	19522239	+	4538	1	AEBP2|-|-	1.878	2.095
NR_04683	chr7	5662432	5680461	-	334	1	RNF216-IT	0	0
NM_0148:	chr12	56998833	57006513	-	6234	1	ZBTB39|ZI	8.308	8.189
NM_0006!	chr1	1.04E+08	1.04E+08	+	1667	1	AMY2A|AI	0	0
NM_1458(	chr2	53669980	53787009	-	2062	1	ASB3|ASB-	10.405	15.982
NR_12581	chr8	57343787	57364856	+	1822	1	LOC10192!	0	0
NM_0010(	chr11	5440542	5441553	-	1009	1	OR51I1|-|	0	0.014
NM_0012(	chr20	44475886	44494603	+	6103	1	TTPAL|C2(	7.046	7.857
NR_04654	chr13	27172259	27183503	+	782	1	USP12-AS2	0.841	0.928
NR_03403	chr3	1.43E+08	1.43E+08	-	622	1	LOC10028!	3.234	4.03
NR_03385	chr11	1.34E+08	1.35E+08	+	2731	1	LOC28317	0.685	1.189
NR_03168	chr8	8088941	8089089	-	148	1	MIR548I3|	0	0
NM_1774!	chr8	1.43E+08	1.43E+08	-	4569	1	LYNX1|SLU	0.976	1.484
NM_1749:	chr16	20359170	20404711	-	2113	1	PDILT|PDI.	0.012	0
NM_0010:	chr11	842824	867116	+	1302	1	TSPAN4|N	30.184	23.859
NR_02809	chr7	1.55E+08	1.55E+08	+	2270	1	PAXIP1-AS	4.277	6.161
NM_0190:	chr17	40875889	40885243	-	1795	2	KRT20|CD.	0	0
NM_0000:	chrX	1.37E+08	1.37E+08	+	1826	1	CD40LG|C	0	0
NM_0529(	chr9	1.28E+08	1.28E+08	+	3297	1	SLC25A25|	23.3	30.348
NM_0011:	chr16	89613308	89638431	+	1635	1	DPEP1|ME	0.134	0.05
NM_0007!	chr4	9781634	9784009	+	2373	1	DRD5|DBD	0	0.006
NM_1758:	chr19	11374707	11376951	+	1658	1	SWSAP1|C	2.247	1.446
NM_0010(	chr9	1.37E+08	1.37E+08	-	2009	1	LCN10|-|-	0.039	0
NM_1827(	chr11	65712002	65719606	+	2062	1	KAT5|ESA:	43.877	44.725
NM_0011(	chr17	4538896	4555386	-	4076	1	MYBBP1A|	8.395	8.989
NR_12157	chr9	1.01E+08	1.01E+08	+	561	1	TMEM246	3.7	2.661
NR_04683	chr1	49374201	49472085	-	510	1	AGBL4-IT1	0	0
NM_0013(	chr4	1.46E+08	1.46E+08	-	7771	1	ZNF827|-|	5.553	6.192
NM_1768:	chr12_KI2	220313	221314	-	999	3	TAS2R19|I	0.052	0.069
NM_0012(	chr1	93561786	93847150	-	3206	1	BCAR3|NS	5.043	3.794
NM_0026:	chr17	49404048	49414905	-	1852	1	PHB|HEL-:	46.547	43.288
NM_1446:	chr19	52297169	52325927	+	4744	1	ZNF480|-|	1.237	2.633
NM_0030!	chr15	24954893	24978723	+	1550	1	SNRPN|HC	291.251	390.117
NM_0012:	chr15	84669537	84716460	-	1250	1	SEC11A|1:	77.598	66.206
NM_0159:	chrX	1.53E+08	1.53E+08	+	1553	1	NSDHL|H1	42.516	36.849
NR_10425	chr7	1.43E+08	1.43E+08	+	1700	1	TMEM139	0.705	0.5
NM_0013:	chr19_GL9	175657	182446	+	521.4	10	RPS9|S9|-	0.025	0.053
NR_14446	chr10	15015072	15035425	-	836	1	DCLRE1CP	0	0
NM_0012:	chr15	74445977	74461188	-	1303	1	UBL7|BMS	58.916	69.515
NM_0013(	chr19	57499854	57508229	+	2352	1	ZNF773|ZI	3.152	4.711
NM_0011:	chr10	4999911	5004014	-	1092	1	AKR1C2|A	15.975	9.319
NR_10678	chr1	632325	632413	-	88	1	MIR6723|	2.051	15.098
NM_0121	chr15	43185268	43197177	+	3637	1	CCNDBP1|	11.018	7.562
NR_13238	chr2	34067226	34069550	+	594	1	LINC01318	0	0
NM_0242!	chr1	32200386	32205390	+	1084	1	CCDC28B|	46.079	47.131
NM_0209	chr16	84476360	84504682	-	5021	1	TLDC1|KIA	9.963	8.037
NM_0012	chr17	76736565	76779254	+	2834	1	MFSD11|E	12.693	12.663
NM_0012:	chr19	812488	821952	-	2293	1	PLPPR3|LF	161.194	120.746
NM_1526(	chr19	55577525	55580845	-	2164	1	ZNF579|-|	18.637	17.754
NM_0010(	chr12	1.04E+08	1.04E+08	+	1470	1	EID3|NS4E	0.044	0.056

NM_0322	chr18	9885726	9888159	+	1922	1	TXNDC2|S	0.04	0.057
NM_0013	chr11	1.25E+08	1.25E+08	-	3611	1	ROBO4|EC	0.039	0.023
NR_13617	chr2	1.03E+08	1.03E+08	-	3111	1	MFSD9|-|-	5.98	5.363
NR_04512	chr15	77641764	77649239	+	1716	1	LINGO1-AS	0.15	0.112
NM_0309	chr6	29173534	29174574	+	1039	8	OR2J2|OR	0	0
NM_1735	chr6	4068359	4079223	-	2261	1	FAM217A|	0	0.012
NM_0012	chr17	3510502	3557995	-	6093	1	TRPV3|FN	0.747	0.209
NM_0010	chr19	57769652	57780616	+	2089	1	ZNF586|-|	5.752	8.307
NM_0250	chr1	1.15E+08	1.15E+08	-	5484	1	SIKE1|SIKE	4.462	3.924
NM_2074	chrX	72144184	72161750	+	2265	1	FLJ44635|	28.109	25.235
NR_00257	chr13	27255064	27255135	+	71	1	SNORD102	1.998	1.736
NM_0012	chr16	57810637	57816927	+	1292	1	LOC38828	0	0
NR_13197	chr18	74409448	74413208	+	1701	1	LINC01922	0	0
NM_0209	chr20	46060987	46089941	-	3197	1	NCOA5|CI	39.314	38.885
NM_0012	chr15	34343315	34357737	+	4008	1	NUTM1|C	0.023	0.034
NM_0012	chr13	24270687	24307074	+	6185	1	SPATA13|	3.558	7.629
NR_04623	chr3	1.19E+08	1.19E+08	+	1597	1	IGSF11-AS	0	0.009
NM_0141	chr15	34978341	34988296	-	5443	1	ZNF770|PI	4.434	7.089
NR_00369	chr7	44983028	44986660	-	798	1	SNHG15|C	32.832	26.587
NM_0010	chr19	43584367	43596135	-	9682	1	IRGQ|FKS	20.271	18.397
NM_0323	chr16	69328621	69339623	-	2487	1	COG8|CD	20.561	21.176
NR_10680	chr11	62789815	62789885	+	70	1	MIR6748|	7.368	6.849
NM_0184	chr15	1.01E+08	1.01E+08	-	1254	1	SELENOS|	36.161	29.174
NM_1384	chr7	23299321	23309561	+	749	1	MALSU1|	20.898	21.268
NM_0013	chr21	34375480	34389154	+	864	2	SMIM11B|	0	0
NM_0169	chr4	25120005	25160582	-	5632	1	SEPSECS|L	0.996	1.209
NR_00291	chr7	45104349	45104482	-	133	1	SNORA5A|	0.969	1.545
NM_0334	chr6	83193313	83198935	+	3808	1	RWDD2A|	3.406	5.075
NM_0248	chrX	49162843	49168156	+	2801	1	MAGIX|JM	0.18	0.176
NM_0058	chr19	1450149	1473244	+	10134	1	APC2|APC	109.299	98.048
NM_0123	chr10	69451470	69507667	+	1689	1	TSPAN15|	7.099	7.688
NR_00217	chr6	25081067	25137823	-	2455	1	CMAHP|CI	0.011	0.056
NM_0186	chr8	22620431	22669148	-	1839	1	BIN3|-|-|8	11.547	9.988
NM_0011	chr9	34652185	34661901	+	1706	1	IL11RA|CR	11.412	10.269
NR_02984	chrX	85903636	85903707	-	71	1	MIR361|M	0	0
NR_13630	chr9	92805385	92808087	-	2702	1	LOC10192	0.081	0.106
NR_03035	chr12	1.09E+08	1.09E+08	-	98	1	MIR619|M	0	0
NM_0053	chr6	26107412	26108136	-	722	1	HIST1H1T|	0	0
NR_13529	chr1	2.08E+08	2.08E+08	-	2061	1	C1orf132|	0.475	0.811
NM_0010	chr4	99896250	99946726	-	6063	1	DNAJB14|	0.938	1.087
NM_0011	chr5	1.68E+08	1.68E+08	+	6714	1	WWC1|HE	5.732	11.651
NR_02836	chrX	48107982	48120686	+	1278	1	SSX6|SSX6	0	0
NM_0041	chr9	93121489	93134288	-	1275	1	NINJ1|NIN	13.5	13.89
NM_0012	chr19	55262222	55279857	-	1706	1	HSPBP1|FI	48.785	35.929
NR_12042	chr13	27236282	27237257	-	864	1	LINC00412	0.104	0.206
NM_0012	chr6	42224933	42452051	-	7624	1	TRERF1|B	2.728	2.224
NM_0066	chrX	1.19E+08	1.19E+08	+	1914	1	PGRMC1|	104.509	83.613
NM_0179	chr14	45084099	45115601	+	2852	1	PRPF39|-|	4.765	7.141
NM_0011	chr2	1.58E+08	1.58E+08	-	8750	1	ACVR1C|A	0.01	0.042
NR_13073	chrY	1397025	1399398	+	363	2	LINC0010	0	0

NM_0247{	chr11	62750963	62754184	-	2970	1	ZBTB3|-|-	2.418	2.527
NR_14462	chr7	1.56E+08	1.56E+08	+	4270	1	LOC38960.	0	0
NM_0011	chr12	66883	178459	+	5030.5	2	IQSEC3|-|-	1.571	2.837
NM_1445	chr12	54455952	54473602	-	801	1	GTSF1|FAI	0	0
NM_0013(	chr7	1.59E+08	1.59E+08	-	3691	1	VIPR2|C16	0	0.026
NR_03842	chr14	75294404	75296408	+	485	1	LINC0122C	0.346	0.424
NR_02732	chr15	36799100	36818506	-	1686	1	CSNK1A1P	1.178	0.829
NM_0146{	chr19	47349281	47382704	+	4353	1	DHX34|DL	6.427	6.343
NM_0001(	chrX	71223206	71225215	+	1650	1	GJB1|CMT	0.047	0
NR_03385	chr12	137411	149166	-	2894	2	LOC57453.	0	0
NM_0013(	chr12	76858715	76879040	-	890	1	CSRP2|CRI	71.87	167.95
NM_1735	chr11	6205568	6211135	+	1138	1	C11orf42|	0.453	0.156
NM_0229(	chr5	69093949	69131072	+	4042	1	SLC30A5|Z	4.488	5.154
NM_0159{	chr17	30782684	30824760	-	2931	1	CRLF3|CRE	2.736	3.725
NM_0010	chrX	1.21E+08	1.21E+08	-	5160	1	CUL4B|CU	6.119	7.995
NM_0073	chr1	93079235	93139081	+	4111	1	MTF2|M9(	11.72	10.868
NR_03980	chr8	1.25E+08	1.25E+08	+	66	1	MIR4662A	0	0
NR_13565	chrX	1.53E+08	1.53E+08	+	587	1	LOC10537.	1.889	2.52
NR_13459	chr6	1.56E+08	1.56E+08	+	719	1	LOC10537	0	0
NM_0333	chr9	1.25E+08	1.25E+08	-	7052	1	NR6A1|CT	1.885	2.265
NR_02951	chrX	53556223	53556341	-	118	1	MIR98|MI	0	0
NM_0011	chr11	27654895	27701053	-	3760	1	BDNF|ANC	2.054	1.413
NM_0227(	chr20	2835315	2840702	-	2015	1	PCED1A|C	32.384	23.764
NR_10410	chr9	34634722	34637826	-	1594	1	SIGMAR1|	50.288	42.638
NM_0012	chr6	1.46E+08	1.46E+08	+	6697	1	GRM1|GPI	0.811	0.665
NR_02850	chr5	60487932	60491265	+	2095	1	PART1|NC	0	0.026
NM_0012	chr1	52056185	52090716	+	4434	1	BTF3L4|-|-	13.926	17.367
NR_12640	chr4	1.33E+08	1.33E+08	+	501	1	LINC0125€	0	0
NR_13194	chr2	2.13E+08	2.13E+08	+	408	1	LINC0187{	0	0
NM_0163	chr4	88457117	88506168	+	3493	1	HERC5|CE	0.103	0.22
NR_04756	chr1	1.97E+08	1.97E+08	+	4785	1	CRB1|LCA	4.406	9.025
NR_03988	chr1	1.97E+08	1.97E+08	-	68	1	MIR4735|-	0	0
NR_04549	chr8	38163321	38176730	-	879	1	LSM1|CAS	43.756	45.563
NM_0010	chr14	49583572	49586416	-	331	1	RPS29|DB.	260.557	190.724
NM_0001(	chr7	1.08E+08	1.08E+08	+	3597	1	DLD|DLDC	14.463	15.449
NM_0146	chr3	5187674	5219965	+	6139	1	EDEM1|EL	4.625	4.627
NR_02708	chr1	2.06E+08	2.06E+08	-	2362	1	LOC28457	0.033	0.035
NM_0016	chr3	1.87E+08	1.87E+08	+	1564	1	AHSG|A2H	0.28	0.307
NR_13624	chr6	1.69E+08	1.69E+08	-	2892	1	LOC10537	0	0
NM_0803	chr2	1.31E+08	1.31E+08	+	1625	1	TUBA3D|H	0.167	0.253
NR_03398	chr2	60847760	60881314	-	1702	1	LINC0118	0.038	0.016
NM_0012{	chr15	30400562	30414260	-	5154	2	GOLGA8R|	0	0
NR_03162	chr11	70283955	70284070	+	115	1	MIR548K|	0	0
NR_02711	chr6	961003	1101332	-	2334	1	LINC01622	0	0.012
NM_0013(	chr15	64687574	64703281	-	1924	1	OAZ2|AZ2	148.014	156.846
NR_02689	chr12	51186936	51217755	-	5208	1	POU6F1|B	9.051	14.641
NM_0216	chr1	2.03E+08	2.03E+08	-	3359	1	KLHL12|C	21.146	28.21
NR_00305	chr17	16441226	16441298	+	72	1	SNORD65|	2.328	4.946
NM_1533(	chr9	1.1E+08	1.11E+08	-	12293	1	SVEP1|C9(	0.172	0.25
NM_0322	chr16	2155798	2178129	+	3644	1	TRAF7|RF\	38.978	47.74

NM_0033:	chr19	48880965	48898739	-	1745	1	TULP2|CT(	0.015	0.016
NR_11069	chr1	22023994	22026048	-	695	1	LINC01635	0.297	0.493
NM_1985:	chr9	34521042	34523039	-	1074	1	ENHO|C9c	60.338	75.614
NM_0062(	chr8	1.17E+08	1.17E+08	-	3734	1	RAD21|CD	47.033	49.744
NR_10346	chr10	14838160	14846999	+	4801	1	HSPA14|H	8.304	8.821
NM_0012(	chr11	85957171	86069881	-	3695	1	PICALM|C.	23.264	28.981
NM_0012{	chr6_GL0C	3035628	3042369	-	1551	7	CLIC1|G6|	0.017	0
NR_10407	chr1	1.44E+08	1.44E+08	+	164	1	RNVU1-15	0.55	0.084
NM_1534	chr11	7825037	7825972	-	934	2	OR5P3|JC(	0	0
NM_0012	chr4	9325165	9326757	+	1591	8	USP17L29	0	0
NM_0010	chrX	49599013	49608536	+	2991	1	GAGE1|CT	0	0
NM_0012!	chr2	3606052	3644644	+	1254	1	COLEC11|:	0.154	0.098
NM_0010:	chrX	55009055	55031064	-	1904	1	ALAS2|AL/	0	0.036
NM_0012{	chr15	44537068	44562803	+	2387	1	EIF3J|EIF3:	8.778	10.363
NR_13505	chr11	1.35E+08	1.35E+08	+	1447	1	LOC10050	0	0
NM_0122:	chr19	54333185	54339169	-	1953	4	LILRA4|CD	0	0
NM_0010:	chr19	42902082	42917924	-	1729	1	PSG6|PSB(	0	0
NM_0048{	chr5	1.36E+08	1.36E+08	-	1967	1	CXCL14|BI	0.439	0.014
NM_1383(	chrX	55143102	55161195	-	1058	1	FAM104B|	10.432	9.373
NM_0013:	chr17	3440011	3513852	-	1683	1	SPATA22|I	0.215	0.065
NR_04750	chr8	20275773	20290458	+	889	1	LZTS1-AS1	0	0.046
NM_1826:	chr13	1.14E+08	1.14E+08	+	1020	1	TMEM255	2.756	0.859
NM_0012!	chr10	1.22E+08	1.22E+08	+	3669	1	TACC2|AZ	43.888	40.072
NM_1723	chr16	84221135	84239750	-	2758	1	KCNG4|KV	0.019	0
NM_0122{	chr6	52497402	52577064	-	7044	1	TRAM2|-|-	3.864	4.097
NM_0012:	chr5	98855204	98926534	-	6420	1	CHD1|-|-|	3.165	3.958
NM_0314:	chr1	1.63E+08	1.63E+08	+	1827	1	NUF2|CDC	1.242	2.054
NM_0010(	chr16	31214021	31225189	+	2143	1	TRIM72|M	0.066	0.083
NM_0011:	chr3	1.9E+08	1.9E+08	-	3049	1	P3H2|LEPI	1.467	0.279
NR_04583	chr16	89286658	89490561	-	2021	1	ANKRD11|	16.307	18.597
NM_0528!	chr19	15468646	15479504	-	1874	1	PGLYRP2|I	0	0
NM_0011	chr19	19181876	19192591	-	1491	1	BORCS8|M	17.926	17.206
NM_0529(	chr22	37367960	37427470	-	8356	1	ELFN2|LRI	3.209	9.945
NM_0010(	chr10	1.23E+08	1.23E+08	+	1527	1	BUB3|BUE	45.722	51.747
NR_10380	chr13	49660676	49691487	-	903	1	EBPL|EBRI	24.902	21.342
NM_0044	chr12	15620141	15789576	-	4059	1	EPS8|DFNI	12.681	7.782
NR_12539	chr3	1.28E+08	1.29E+08	+	2355	1	GATA2-AS:	0.082	0.07
NM_0011	chrX	1.21E+08	1.21E+08	-	1324	1	CT47B1|C	0	0
NM_0032{	chr3	52451091	52454041	-	688	1	TNNC1|CN	1.855	0.677
NR_02411	chr7	1.57E+08	1.57E+08	+	610	1	LINC00244	0	0
NM_0121{	chr1	63323059	63325126	+	2065	1	FOXD3|AI!	0.15	0.159
NM_0048(	chr19	18385960	18389177	+	1395	1	GDF15|GD	8.744	1.669
NM_0011!	chr11	94305637	94307692	+	748	1	IZUMO1R|	0	0
NR_00282	chr22	21042392	21044249	-	122	1	P2RX6P|-|	0	0
NM_0011	chr22	24270817	24417740	+	6657	1	SPECC1L|(	23.409	20.168
NM_0011	chr3	1.2E+08	1.2E+08	-	7066	1	GSK3B|-|-	24.873	27.902
NM_0013:	chr2	31173056	31233957	-	3862	1	CAPN14|-|	0.514	0.95
NM_0003!	chr22	36913633	36940437	+	4830	1	CSF2RB|CI	0	0
NM_0066!	chr6	41770176	41779905	-	2170	1	FRS3|FRS2	15.686	15.635
NR_02832	chr11	1.08E+08	1.08E+08	+	969	1	LOC64392.	0.027	0.085

NM_0010:	chr1	1.92E+08	1.92E+08	+	1786	1	RGS21|-|-	0	0
NR_03172	chr18	24321686	24321735	+	49	1	MIR320C2	0	0
NR_13275	chr6	3024791	3027424	+	2633	1	LOC10192	2.478	2.585
NM_0016:	chr13	30735508	30764428	+	899	1	ALOX5AP|	0.387	0.107
NM_0010:	chr4	56436103	56461368	+	3307	1	PAICS|ADE	38.018	36.092
NR_12161	chr4	74955974	74970362	-	2542	1	LOC10050	0	0.005
NM_0044:	chr12	8914720	8941464	+	5159	1	PHC1|EDR	4.469	3.876
NM_0010:	chr19	50943303	50953088	-	1352	1	KLK5|KLK-	0	0.03
NM_0250!	chr6	1.51E+08	1.52E+08	+	5270	1	CCDC170|	0.051	0.052
NM_0012!	chr4	9215405	9216997	+	1591	2	USP17L18	0	0
NM_1524!	chr1	33256573	33300719	+	3095	1	ZNF362|RI	29.828	31.921
NR_10695	chr12	1.21E+08	1.21E+08	-	79	1	MIR7107|	6.365	9.189
NM_0011:	chr12	1.2E+08	1.2E+08	+	1568	1	TMEM233	0	0.035
NM_0012!	chr11	11351942	11353357	-	1413	1	CSNK2A3|	27.165	34.587
NR_13622	chr8	67343834	67375231	+	2038	1	LOC10272	0.709	0.659
NR_14453	chr12	92466679	92483947	+	2252	1	LOC10050	0.51	1.958
NR_13431	chr10	79628758	79663185	-	642	1	LOC10537	0.04	0
NM_0011:	chr1	39560813	39576849	-	3186	1	PABPC4|A	29.417	26.839
NM_1985:	chr7	1.01E+08	1.01E+08	-	2929	1	NAT16|C7	6.898	6.173
NM_0165	chr16	798041	800733	-	979	1	GNG13|G(	0.764	0.196
NR_03981	chr8	1.44E+08	1.44E+08	-	70	2	MIR4664|	0	0
NM_0325:	chr18	80157234	80247514	-	3829	1	PARD6G|P	9.327	7.154
NM_1982:	chrX	19912862	19970264	-	6480	1	CXorf23|-|	1.669	2.312
NM_0010(	chr14	19402764	19434341	-	1913	1	POTEG|A2	0	0
NM_0322!	chr5	1.4E+08	1.4E+08	+	4493	1	PSD2|EFA(	15.737	21.879
NR_04007	chr11	1.27E+08	1.27E+08	+	1765	1	KIRREL3-A:	0.095	0.039
NM_0012	chr6	20403679	20493714	+	4416	1	E2F3|E2F-	3.731	7.267
NM_0011:	chr2	86815337	86861924	-	928	1	CD8B|CD8	0	0.089
NM_0185(	chr1	31413813	31434680	+	2050	1	SERINC2|F	20.699	16.837
NR_02460	chrX	1.35E+08	1.35E+08	-	783	1	MIR503HG	0.066	0.087
NM_0013(	chr11	45921621	45929096	+	2442	1	LARGE2|G	0.417	0.163
NR_03174	chr4	1.26E+08	1.26E+08	+	48	1	MIR2054|	0	0
NM_0160!	chr16	85677674	85688982	-	1173	1	GINS2|HSI	19.807	19.781
NM_0183(	chr3	12733530	12759309	-	2059	1	TMEM40|	0.025	0
NR_03968	chr9	36823539	36823599	-	60	1	MIR4475|	0	0
NM_0010	chr14	75013764	75051532	-	7881	1	MLH3|HNI	1.443	1.147
NM_0011:	chr18	79964582	79970822	+	744	1	HSBP1L1|-	3.362	3.332
NR_11003	chr3	1.07E+08	1.07E+08	+	1484	1	LINC0199C	0.017	0
NM_0069!	chr12	6904733	6914242	+	1321	1	LRRC23|LF	13.245	12.359
NR_13393	chr8	56211638	56218798	+	1873	1	CHCHD7|C	13.189	10.684
NM_1991	chr19	10871577	10922772	+	2950	1	CARM1|PI	63.353	52.146
NM_0010(	chr7	50590063	50732353	-	4550	1	GRB10|GR	33.339	26.759
NM_0013:	chr17	42810132	42823879	-	1828	1	BECN1|AT	28.601	28.016
NR_10690	chr8	1.44E+08	1.44E+08	+	68	1	MIR6847|	3.792	2.82
NM_0012(	chr2	85318018	85328296	-	6185	1	TGOLN2|T	21.486	24.307
NM_0055	chr5	1.57E+08	1.57E+08	+	4347	1	ITK|EMT|L	0.205	0.032
NM_0201!	chr7	97116593	97181763	+	2053	1	SDHAF3|A	4.509	3.683
NR_12633	chr11	3507980	3571987	-	575	1	LOC10192	0	0
NR_02734	chr9	92141467	92159608	+	1996	1	LINC0047E	0	0.007
NM_0013:	chr21	31559245	31560487	+	818	1	LOC15005	2.68	2.696

NM_0011	chr7	1.38E+08	1.38E+08	+	2577	1	AKR1D1|3	0.01	0
NR_10268	chr8	63167725	63168442	-	717	1	YTHDF3-A	1.061	1.356
NM_1525	chr9	1.36E+08	1.36E+08	+	2570	1	C9orf163|	0.396	0.592
NR_03976	chr9	1.28E+08	1.28E+08	+	90	1	MIR3960|	2.006	0.913
NM_0065	chr1	42925375	42959176	-	3658	1	SLC2A1|C	72.074	23.182
NM_0165	chr3	1.34E+08	1.34E+08	-	5550	1	RAB6B|-|-	78.632	93.581
NR_12037	chr15	74461265	74481292	+	1315	1	UBL7-AS1|	2.059	1.771
NM_0010	chr11	1.24E+08	1.24E+08	-	994	1	OR10S1|O	0	0
NM_0312	chr4	1.28E+08	1.28E+08	+	1833	1	SLC25A31|	0	0.015
NM_0024	chr4	4859665	4863933	+	1933	1	MSX1|ECT	3.615	1.516
NR_10979	chr8	1.18E+08	1.19E+08	-	8779	1	SAMD12|-	0.77	1.102
NR_02742	chr9	67725483	67726743	-	701	1	FAM27B|F	0	0
NM_0050	chrY	1591593	1602518	+	3201	2	AKAP17A|	0	0
NM_0011	chr3	1.88E+08	1.88E+08	-	3073	1	BCL6|BCL	1.204	1.975
NR_13425	chr7	15667947	15681979	+	3738	1	LOC10537	0	0
NM_0065	chr15	52120926	52179965	-	2966	1	GNB5|GB	20.136	19.202
NR_02949	chr19	13836287	13836359	-	72	1	MIR24-2|	0	0.38
NM_1387	chr7	40132743	40134652	-	937	1	MPLKIP|A	26.929	26.999
NM_0323	chr2	1.9E+08	1.9E+08	+	3958	1	C2orf88|s	0.094	0.17
NM_0031	chrX	49187812	49200202	-	2424	1	SYP|MRX	131.068	141.169
NR_00221	chr17	15786850	15789706	+	2856	1	MEIS3P1|	3.684	3.889
NR_03619	chr13	99643059	99643149	+	90	1	MIR4306|	0	0
NM_0011	chr16	2520362	2530954	+	2064	1	AMDHD2|	5.122	4.911
NM_0013	chr6	1.3E+08	1.3E+08	+	4104	1	L3MBTL3|	5.818	3.958
NM_0142	chr16	14833786	14896157	+	4214	2	NOMO1|N	0	0.007
NM_0101	chr6	1.28E+08	1.28E+08	+	1193	1	C6orf58|L	0.14	0.195
NR_13449	chr10	28433008	28495813	-	270	1	LOC10537	0.096	0
NR_03884	chr4	86924894	86934850	-	1942	1	LOC10050	0.02	0.106
NM_0022	chr12	6772504	6778455	+	1982	1	LAG3|CD2	3.025	3.414
NM_0030	chr20	45221369	45224458	+	1977	1	SEMG2|S	0.007	0
NM_1456	chr11	3087116	3165352	-	3653	1	OSBPL5|O	15.428	17.87
NM_0010	chr1	1.21E+08	1.21E+08	+	1621	1	FCGR1B|C	0	0
NR_12207	chr7	22649960	22665533	-	2587	1	LOC40131	0	0
NM_0323	chr19	6372433	6375250	+	1325	1	ALKBH7|A	45.395	34.072
NR_10500	chr5	66507545	66511604	-	657	1	LOC10192	0	0
NM_1532	chrX	47190800	47215128	+	3499	1	UBA1|A1S	270.244	269.157
NM_0178	chr9	1.09E+08	1.09E+08	+	1903	1	FAM206A|	6.274	4.844
NM_0153	chr8	1.44E+08	1.44E+08	-	5105	2	SCRIB|CRI	0	0
NM_0004	chr5	96390336	96433281	-	5150	1	PCSK1|BM	20.479	2.777
NM_0805	chr20	38724462	38729372	+	2570	1	SLC32A1|	4.601	7.808
NM_0049	chr15	59135969	59372872	-	5080	1	MYO1E|FS	1.579	2.127
NM_1817	chr5	1.51E+08	1.51E+08	-	2848	1	SLC36A3|	0	0
NM_0002	chr13	23180921	23325165	+	1645	1	SGCG|35D	0.541	1.948
NR_12038	chr13	92339794	92352207	-	412	1	GPC5-AS2|	0	0
NM_0019	chr4	1.83E+08	1.83E+08	-	2011	1	DCTD|-|-|	18.972	15.202
NM_0140	chr6	52671086	52686587	+	1007	1	TMEM14A	37.643	38.221
NR_10808	chr7	30390885	30404054	+	712	1	LINC0117	0.036	1.135
NR_00688	chr17	19112420	19112636	-	216	1	SNORD3D	0	0
NM_0072	chr8	26504680	26513967	-	4841	1	PNMA2|M	69.674	63.341
NM_0012	chr1	28518233	28539196	+	2528	1	RCC1|CHC	13.368	11.849

NM_0013(	chr12	52837804	52848283	-	2543	1	KRT78|CK-	0	0
NM_0224:	chr4	1.45E+08	1.45E+08	+	3540	1	HHIP|HIP|	0.051	0.039
NM_0012(	chr14	70325081	70417090	-	1920	1	SYNJ2BP-C	32.18	34.613
NR_10402	chr19	10543894	10553423	+	1692	1	ATG4D|AP	23.92	21.242
NM_0149(	chr14	76761892	76783020	+	6021	1	VASH1|KIA	37.464	50.35
NM_0189:	chr5	1.41E+08	1.42E+08	+	4584	1	PCDHGB1|	21.464	82.95
NM_0011:	chr10	65912518	67665685	-	10630	1	CTNNA3|A	0.032	0.049
NR_03675	chr17_KI2	2230861	2292321	+	1523.7	3	LOC44043	0	0
NM_0010(	chr11	48216810	48217739	+	928	1	OR4B1|OF	0	0
NM_0011!	chr14	23970015	24006408	+	1321	1	DHRS4L2|	0.664	0.612
NM_0044	chr7	1.01E+08	1.01E+08	-	4332	1	EPHB4|HT	6.783	5.334
NM_0019!	chr11	65120627	65122200	-	568	1	FAU|FAU1	436.562	331.236
NM_0806!	chr8	1.18E+08	1.18E+08	+	979	1	MED30|M	13.46	17.894
NM_0181:	chr16	57471958	57486473	-	2739	1	DOK4|IRS-	40.455	54.258
NM_0010:	chr10	1.33E+08	1.33E+08	-	3169	1	SPRN|SHA	5.383	7.227
NR_00215	chr7	1.44E+08	1.44E+08	-	984	2	OR2A9P|F	0	0
NR_02440	chr3	1.02E+08	1.02E+08	+	2783	1	ZBTB11-AS	1.969	1.545
NM_0201:	chr10	96518110	96587052	-	6123	1	TM9SF3|E	8.362	11.585
NM_0013:	chr1	64833223	64966936	-	4910	1	JAK1|JAK1	18.064	21.19
NR_00276	chr2	2.11E+08	2.11E+08	+	2304	1	CPS1-IT1|(	0	0
NR_02703	chr12	57726240	57728356	+	1529	1	AGAP2-AS	21.79	27.797
NM_0012{	chr19	17267376	17279353	+	1438	1	BABAM1|(	54.596	56.445
NM_0013:	chr1	1.7E+08	1.7E+08	+	2790	1	C1orf112|	2.288	2.489
NM_0069:	chr18	63655197	63661963	-	1767	1	SERPINB3|	0.029	0
NM_0010:	chr17	48133440	48430232	-	1558	1	SKAP1|HE	0.199	0.018
NM_0011!	chrX	46497725	46545457	-	4068	1	ZNF674|M	0.542	0.559
NM_0221!	chr17	82057872	82065821	-	1868	1	DUS1L|DU	16.538	12.392
NR_13657	chr22	18865043	18894431	-	3178	1	LINC01663	0.004	0.009
NM_0246:	chr16	30524001	30526589	-	2311	1	ZNF768|-|	49.995	47.267
NM_1993	chr1	1.68E+08	1.68E+08	+	846	1	SFT2D2|U	2.56	3.319
NR_00157	chrY	21395148	21401562	-	529	1	RBMY2EP|	0	0
NM_0053:	chrX	1.54E+08	1.54E+08	-	8398	1	HCFC1|CF	28.25	24.344
NM_0314!	chr11	66664994	66677921	-	1838	1	RBM4B|RE	27.688	29.16
NM_1456!	chr13	27792643	27793952	+	843	1	GSX1|GSH	0.336	1.121
NM_0013(	chr8	11993177	12000752	-	1091	1	DEFB134|-	0.024	0
NM_0038:	chr14	1.01E+08	1.01E+08	+	4677	1	DLK1|DLK	196.142	72.404
NM_0333{	chr22	30396941	30416976	+	1817	1	SEC14L2|(	4.605	4.35
NM_0041:	chr13	45120496	45284104	+	1443	1	GTF2F2|B	31.068	24.357
NR_03749	chr22	31160062	31160119	-	57	1	MIR3928|	0	0
NR_11027	chr2	70942013	70948611	-	941	1	ATP6V1B1	0.069	0.306
NR_12594	chr1	80535755	80646788	+	587	1	LINC01781	0	0
NM_0012!	chr4	9258124	9259716	+	1591	2	USP17L22	0	0
NM_0012!	chr3	48432170	48440119	-	1374	1	CCDC51|-|	17.041	17.216
NR_03414	chr17	12549968	12637187	+	2602	1	LINC0067C	0	0
NR_12061	chr10	89455991	89468151	+	559	1	SLC16A12-	0	0
NM_0011(	chr19	52336245	52367123	+	2143	1	ZNF610|-|	4.717	5.017
NM_0013:	chr3	50155045	50189075	+	3239	1	SEMA3F|S	20.739	15.875
NM_0052!	chr8	1.41E+08	1.41E+08	-	1522.5	2	GPR20|-|-	0	0
NM_0011(	chr5	83471674	83582303	+	9439	1	VCAN|CSP	12.898	19.834
NR_13569	chr15	70570958	70586606	-	3204	1	LOC10192	0	0

NM_2033	chr2	2.23E+08	2.23E+08	+	4236	1	ACSL3|ACS	12.662	9.959
NM_1757	chr17	7425616	7427567	+	1644	1	C17orf74|	0.494	0.225
NM_0011	chr4	1.65E+08	1.65E+08	-	4487	1	TMEM192	3.029	4.991
NM_0329	chr16	57462639	57472009	+	1793	1	POLR2C|R	58.83	54.078
NM_0005	chr17	7668402	7687550	-	2578	1	TP53|BCC	8.962	12.863
NM_2069	chr6	1.18E+08	1.19E+08	-	2272	1	CEP85L|C6	0.749	1.965
NM_0010	chr19	52838532	52857649	-	3907	1	ZNF468|-|	1.574	2.829
NM_0010	chr4	1.74E+08	1.74E+08	+	4521	1	CEP44|KIA	1.725	1.791
NR_00222	chrX	71491681	71492449	-	768	1	INGX|ING1	0.05	0
NM_0072	chr1	2.22E+08	2.22E+08	-	2674	1	DUSP10|M	2.184	3.073
NR_03163	chr11	93733674	93733764	-	90	1	MIR1304|	0.143	2.587
NM_0011	chr6	26383101	26394872	+	2950	1	BTN2A2|B	3.837	10.21
NR_02995	chrX	1.15E+08	1.15E+08	+	110	1	MIR448|M	0	0
NR_10417	chr1	42857622	42888792	+	2361	1	LOC33953	0.279	0.122
NM_0022	chr12	54395261	54419266	-	4218	1	ITGA5|CD4	10.069	7.891
NR_10681	chr11	71473503	71473568	+	65	1	MIR6754|	0.397	0.211
NM_0010	chr5	1.5E+08	1.5E+08	-	3170	1	ARSI|ASI|	0.504	0.043
NR_04637	chr12	1.21E+08	1.21E+08	+	1869	1	P2RX4|P2	5.264	3.657
NM_0010	chr11	56136771	56137718	-	946	1	OR8J3|OR	0	0
NM_2069	chr11	62296282	62299064	-	429	1	SCGB1D4|	0	0.032
NM_0010	chr2	1.97E+08	1.97E+08	+	3655	1	CCDC150|	0.519	0.982
NR_03036	chr17	66787072	66787168	+	96	1	MIR634|M	0	0
NM_0013	chr7	1.06E+08	1.06E+08	-	2916	1	ATXN7L1|	2.45	2.198
NM_1942	chr14	74493720	74495568	+	1082	1	ISCA2|HBL	22.021	22.254
NM_0010	chr4	68310387	68350117	-	6241	2	YTHDC1|Y	0	0
NM_0013	chr14	20365667	20413420	-	10315	1	TEP1|TLP1	2.084	2.272
NM_0012	chr16	66552563	66579135	+	709	1	CKLF-CMT	4.346	1.739
NM_0250	chr14	74019356	74066092	+	3092	1	BBOF1|C1	3.407	2.831
NR_10695	chr22	31621467	31621531	-	64	1	MIR7109|	1.209	0.428
NM_0044	chr9	1.29E+08	1.29E+08	+	1140	1	ENDOG|-|	30.503	26.036
NM_0011	chr6	11538227	11583524	+	8408	1	TMEM170	4.025	5.215
NM_0070	chr14	88465778	88554779	-	6194	1	PTPN21|P	4.911	4.007
NR_00322	chr14	1.01E+08	1.01E+08	+	70	1	SNORD114	0	0
NR_02996	chr14	1.01E+08	1.01E+08	+	79	1	MIR329-1|	0	0
NM_0029	chr17	36103827	36105621	+	662	3	CCL4|ACT	0	0
NM_0227	chr19	42076138	42081567	+	3126	1	ZNF574|F	12.704	14.709
NR_03648	chr7	29685154	29688243	+	1675	1	DPY19L2P	0.323	0.294
NM_0013	chrX	71301748	71305371	+	1019	1	ITGB1BP2|	1.139	1.586
NM_0010	chr6	29086208	29087313	-	1103	8	OR2B3|6M	0	0
NM_0060	chr22	46255663	46263322	-	7658	1	PKDREJ|-|	0.15	0.295
NR_04521	chr19	14137152	14171263	+	2884	1	LOC10050	4.676	5.861
NM_0212	chr21	32601674	32612608	-	1418	1	C21orf59|	44.609	29.5
NM_0010	chr1	16021991	16034050	+	2511	1	CLCNKA|C	0.051	0.055
NM_0011	chr22	31439359	31489561	-	3656	1	EIF4ENIF1	23.262	22.94
NM_0146	chr10	1.04E+08	1.04E+08	-	11231	1	SH3PXD2A	5.028	7.787
NM_1383	chr11	65773898	65780591	-	6592	1	AP5B1|AP	3.575	5.074
NM_0012	chr18	5289019	5297053	-	3336	1	ZBTB14|Z	5.964	6.307
NM_0149	chr5	76083414	76333956	+	10778	1	SV2C|-|-|	2.765	4.781
NM_0205	chr19	7522610	7534009	+	2082	1	MCOLN1|	18.709	18.559
NM_0040	chrX	1.41E+08	1.41E+08	-	1296	1	CDR1|CDR	1.87	2.822

NM_0055:	chr8	1.44E+08	1.44E+08	+	2160	1	HSF1|HSTI	39.636	38.447
NR_13118	chr4	1.03E+08	1.03E+08	+	912	1	LOC10537	2.319	1.126
NR_03973	chr15	23562062	23562131	-	69	1	MIR4508|	0	0
NM_0010:	chr19	50511600	50514690	-	841	1	ASPDH|-|-	0.414	0.342
NR_03416	chr20	18379049	18381483	+	724	1	LINC00851	0	0
NM_0029(	chr1	1.54E+08	1.54E+08	-	704	1	S100A5|S1	0	0
NM_0046:	chrY	22071756	22096007	-	1231	2	PRY|PRY1	0	0
NR_13231	chr2	1.28E+08	1.28E+08	-	2113	1	LOC10537.	0	0
NM_0184(	chr8	97775581	97852602	+	2229	1	LAPTM4B|	68.927	107.505
NR_03159	chr19	2234062	2234149	-	87	1	MIR1227|	14.82	11.65
NM_0010(	chr8	30384501	30547524	+	1344	1	RBPMS|HE	2.053	0.775
NM_0013(	chr12	1.31E+08	1.31E+08	+	2492	1	RAN|ARA2	98.284	85.189
NM_0011(	chr2	1.71E+08	1.71E+08	+	5613	1	DCAF17|C	2.678	3.375
NM_0011:	chr19	46148243	46161304	+	606	1	IGFL2|UN(	0.128	0.09
NM_0158(	chr6	24839366	24877365	-	2429	1	FAM65B|(	24.619	14.12
NM_0050!	chr15	41828085	41848148	+	3340	1	JMJD7-PLA	8.794	7.505
NR_03073	chrX	75740538	75743079	-	2541	1	TTC3P1|RI	9.656	15.708
NM_0072:	chr20	62214961	62220267	-	2675	1	HRH3|GP(	3.707	4.004
NM_0010(	chr16	67207139	67226998	-	1295	1	LRRC29|FE	1.852	1.438
NM_0012:	chr9	1.33E+08	1.33E+08	-	2089	1	REXO4|RE	23.892	23.657
NM_1533(	chr20	58459101	58514938	-	3420	1	APCDD1L|	0.049	0.06
NM_0012!	chr4	87160104	87220608	-	5380	1	KLHL8|-|-|	5.723	4.056
NR_02708	chr1	4412051	4424684	+	2068	1	LINC01777	0	0.007
NM_0013:	chr19	3185877	3209575	+	3713	1	NCLN|NET	10.761	11.735
NM_0038!	chr13	1.13E+08	1.13E+08	+	1479	1	PROZ|PZ|-	0.035	0.056
NM_0529!	chr13	51584348	51762035	+	3795	1	WDFY2|PF	8.626	5.262
NM_0011:	chr15	88803443	88875354	+	8525	1	ACAN|AG(	0.003	0.006
NR_12605	chr6	37507348	37536280	+	1197	1	LOC10050.	0.355	0.778
NR_02940	chr20	5471196	5477134	+	3797	1	LOC64340	0	0
NR_02427	chr1	45303910	45305619	+	1709	1	LINC01144	1.516	1.699
NR_03894	chr10	1.1E+08	1.1E+08	-	1275	1	ADD3-AS1	0.688	0.602
NM_0010(	chr1	43980505	43991171	+	1995	1	B4GALT2|	58.095	63.473
NM_0023-	chr8	1.43E+08	1.43E+08	+	1170	2	LY6E|RIG-I	0	0
NM_1784:	chr1	1.53E+08	1.53E+08	+	590	1	LCE2D|LEF	0	0
NR_03845	chr7	56875385	56882146	-	559	1	LOC10013	0	0
NM_0030:	chr8	41261957	41309471	-	4462	1	SFRP1|FRF	42.483	16.613
NM_0013:	chr2	17510584	17530414	-	4073	1	RAD51AP2	0	0.017
NM_0095!	chr3	1.49E+08	1.49E+08	+	2268	1	AGTR1|AG	0.136	0.453
NR_00321	chr14	1.01E+08	1.01E+08	+	70	1	SNORD114	0	0
NM_0332:	chr8	2935353	4994806	-	14245	1	CSMD1|PF	0.002	0.511
NM_0024:	chr17	10628526	10657309	-	5995	1	MYH3|DA:	0.323	0.35
NM_0010(	chr12	55426254	55427192	+	937	1	OR6C76|-|	0	0
NM_0038-	chr13	77535674	77645263	+	3132	1	SCEL|-|-|1	0.062	0.157
NR_03786	chr1	1.14E+08	1.14E+08	+	1729	1	AP4B1-AS1	5.213	5.926
NR_03750	chr7	43150895	43150994	+	99	1	MIR3943|	0	0
NM_0009!	chr17	8377516	8383247	-	576	1	RPL26|DB.	613.425	605.831
NM_0013:	chr8	55773232	55826445	+	4381	1	TGS1|NCO	2.613	3.214
NM_0037:	chr5	55424842	55535078	-	1660	1	PLPP1|LLP	6.952	6.741
NM_1781:	chr1	24356999	24413772	-	2587	1	STPG1|C1(	4.107	6.221
NM_0807!	chr20	1894779	1939894	+	3857	1	SIRPA|BIT	2.661	6.609

NM_0011(	chr2	1.12E+08	1.12E+08	+	7274	3	RGPD8|RA	0.06	0.038
NM_0069(	chr16	70379435	70439088	-	4441	1	ST3GAL2|(	18.979	16.79
NR_04681	chr4	1.01E+08	1.01E+08	-	820	1	LINC01216	0	0
NR_03059	chr11	79402022	79402109	-	87	1	MIR708|M	0	0
NR_02445	chr7	1.41E+08	1.41E+08	-	1646	1	NDUFB2-A	4.05	2.53
NR_07347	chr10	1.23E+08	1.23E+08	-	4433	1	IKZF5|PEG	2.042	2.429
NM_0013(	chr5	1.38E+08	1.38E+08	-	2924	1	BRD8|SMA	30.562	35.259
NM_0122(	chr19	10486120	10502805	-	2555	1	KEAP1|INr	21.911	19.642
NM_0133(	chr19	44326553	44356704	-	3680	1	ZNF112|ZI	2.214	3.406
NM_0162:	chr6	1.39E+08	1.39E+08	+	5615	1	HECA|HDC	3.573	5.64
NM_0046!	chrY	12701231	12860843	+	9992	1	USP9Y|DFI	3.209	4.552
NM_0012(	chr7	48035511	48061297	+	1909	1	C7orf57|-|	0.007	0.115
NM_0152(	chr11	1.34E+08	1.34E+08	-	5568	1	NCAPD3|C	6.532	7.274
NR_13569	chr15	21717666	21718307	-	555	2	IGHV1OR1	0	0
NR_03751	chr19	45674932	45675008	-	76	1	MIR642B|	0	0
NM_0010(	chr17_KI2	2360558	2378758	-	7967	2	GPR179|C	0.002	0.005
NM_1522(	chr9	1.37E+08	1.38E+08	-	4731	1	PNPLA7|C	0.589	0.675
NM_0251:	chr3	1.26E+08	1.26E+08	-	3393	1	ZXDC|ZXD	12.825	11.557
NM_1446	chr16	27787529	27888157	-	4530	1	GSG1L|PR	0.39	0.209
NR_12035	chr15	73768086	73769802	+	539	1	C15orf59-/	1.818	1.194
NR_04009	chr5	1.12E+08	1.12E+08	+	5518	1	STARD4-AS	16.162	20.771
NM_0012(	chr3	23810443	23891640	+	1543	1	UBE2E1|U	52.451	60.496
NR_10694	chr19	35122700	35122764	+	64	1	MIR6887|I	0	0
NM_1534(	chr5	37291633	37371126	-	4555	1	NUP155|A	6.318	6.591
NM_0249(	chr20	3888839	3929882	+	7730	1	PANK2|C2	10.707	12.45
NM_0528	chr1	2.28E+08	2.28E+08	+	20349	1	OBSCN|AR	1.711	2.849
NM_0009!	chr9	1.37E+08	1.37E+08	+	804	1	PTGDS|L-F	3.897	5.026
NM_0012	chr19	42325609	42378769	+	11123	1	MEGF8|C1	27.744	26.527
NR_03824	chr6	1.46E+08	1.46E+08	+	3994	1	LOC10050	1.724	1.516
NM_0013:	chr16	67394419	67416494	-	1988	1	ZDHHC1|C	4.819	4.726
NM_1533!	chr5	88195206	88268879	-	2611	1	TMEM161	2.923	3.357
NM_1735	chr11_KI2	84490	90434	-	2082	2	LMNTD2|(	0.173	0.105
NM_1392(	chr12	1.11E+08	1.11E+08	-	3740	1	PPTC7|TA-	1.817	2.644
NM_0244	chr18	31065974	31102432	-	5190	1	DSC2|ARV	0.745	0.367
NM_0010:	chr8	28021964	28083871	-	3866	1	NUGGC|C8	0	0
NR_04650	chrY	6357219	6361413	-	445	1	LINC0028C	0	0
NM_0228!	chr3	53867066	53882202	-	3614	1	ACTR8|AR	13.215	12.742
NR_03777	chr19	40775537	40796944	+	1384	1	MIA-RAB4	31.777	30.868
NM_0018!	chr17	82242661	82273718	-	3694	1	CSNK1D|A	62.586	74.8
NR_13278	chr12	56512865	56512980	+	115	1	SNORA105	0	0.238
NR_00354	chr4	1.85E+08	1.85E+08	-	750	1	SLED1|-|-|	0.103	0.037
NR_02750	chr5	1E+08	1E+08	-	697	1	LOC10013	0.018	0
NM_0012!	chr7	29483816	29514335	+	2504	1	CHN2|ARH	7.621	18.182
NR_10983	chr3	50337511	50338296	+	785	1	RASSF1-AS	0.969	0.977
NM_0240	chr10	1.02E+08	1.02E+08	-	1185	1	CUEDC2|C	130.317	126.036
NR_03965	chr4	76575551	76575626	+	75	1	MIR548AH	0	0
NM_1784!	chr20	59191020	59259112	+	9396	1	ZNF831|C	0	0
NM_0011!	chr3	1.56E+08	1.56E+08	-	2440	1	SLC33A1|/	3.646	3.806
NM_0202(	chr1	1.5E+08	1.5E+08	-	6401	1	OTUD7B|(	2.665	2.174
NM_0308:	chr19	13747863	13763298	+	1702	1	CCDC130|	33.612	31.627

NM_0013:	chr19	47035329	47045775	+	1348	1	NPAS1|M(	1.071	2.042
NM_0073:	chr12	10307818	10317251	+	3158	1	KLRD1|CD	0	0.013
NM_0164:	chr22	46296741	46330810	+	3098	1	GTSE1|B9!	3.667	2.564
NM_0010(	chr12	48202339	48203292	-	952	1	OR10AD1|	0	0.029
NM_0011:	chr7	6577434	6588979	+	1321	1	ZDHHC4|Z	19.638	20.582
NM_0013:	chr2	39736060	39779276	-	1803	1	THUMPD2	2.868	3.707
NM_0034:	chr12	65824460	65966291	+	4133	1	HMGA2|B.	0.521	0.239
NR_03620	chr18	44970082	44970166	-	84	1	MIR4319|-	0	0
NM_0180(	chr2	58159243	58241380	-	1701	1	FANCL|FA/	7.633	10.154
NM_0030:	chr5	1.77E+08	1.77E+08	-	1415	1	SNCB|-|-|!	19.937	75.155
NR_02708	chr10	10784439	10794914	-	689	1	SFTA1P|SF	0.112	0.239
NM_1736(	chr12	50504985	50748667	+	8675	1	DIP2B|-|-|	13.354	16.863
NM_0013:	chr19	51077495	51084210	-	1015	1	KLK14|KLK	0.191	0.189
NR_00298	chr1	28579764	28579893	-	129	1	SNORA61|	1.899	4.672
NM_0026:	chr20	54207963	54219953	+	1340	1	PFDN4|C1	7.274	8.607
NR_02459	chr22	20689555	20691721	+	2166	1	POM121L4	0	0
NR_03329	chr17	8173453	8173588	-	135	1	SNORD118	8.787	8.928
NM_1750!	chr12	14770720	14771131	-	409	1	HIST4H4|H	0	0
NR_00392	chr17	19061912	19062128	+	216	2	SNORD3B-	0	0
NR_12604	chr18	6954677	6957418	+	2292	1	LOC10192	0.551	0.209
NM_0013:	chr15_KI2	3787232	4155811	-	1827	2	OTUD7A|(	0.198	0.225
NM_0314:	chr14	73644875	73703728	+	8514	1	DNAL1|C1	3.076	2.218
NM_0043:	chr1	1.48E+08	1.48E+08	+	6180	1	BCL9|LGS|	28.535	32.17
NM_0013(	chr1	1.46E+08	1.46E+08	-	4812	1	ITGA10|PF	0.522	0.387
NM_0225	chrY	6246223	6249012	+	1152	1	TSPY2|TSP	0	0
NM_0065(	chr19	45001449	45038198	+	2262	1	RELB|I-REL	1.436	2.471
NR_02336	chr1	2.29E+08	2.29E+08	-	120	10	RNA5S6|R	0	0
NM_0181(	chr15	40767869	40807478	-	1005	1	DNAJC17|-	13.702	10.876
NM_0012	chr1	16754634	16764480	-	4652	1	MST1L|BR	0.017	0
NM_0013:	chr2	1.08E+08	1.08E+08	+	2642	1	SULT1C4|S	2.957	5.117
NM_0012!	chr17	37978155	37989048	-	2020.5	19	TBC1D3D|	0.102	0.19
NR_13241	chr20	63627235	63628708	+	596	1	LOC10050.	8.221	6.435
NM_0145	chr1	39623431	39639676	-	4107	1	HEYL|HESI	0.986	0.21
NM_0010:	chr13	1.13E+08	1.13E+08	-	3020	1	DCUN1D2	13.7	15.679
NM_0245	chr10	1.02E+08	1.02E+08	-	4073	1	C10orf76|	12.836	15.866
NR_12165	chr3	1.47E+08	1.48E+08	+	658	1	LOC44098	10.816	1.311
NM_0320	chr1	1.2E+08	1.2E+08	-	1335	1	REG4|GISI	0.039	0
NM_0212!	chr16	370776	381950	-	3641	1	TMEM8A|	17.784	14.254
NM_0133(	chr3	1.43E+08	1.43E+08	-	2019	1	PCOLCE2|	0.543	0.916
NR_04985	chr18	72853321	72853382	-	61	1	MIR548AV	0	0
NM_1524(	chr5	35953089	35991433	-	2869	1	UGT3A1|-	0	0
NR_12538	chr14	1.05E+08	1.05E+08	-	1445	1	LOC10050	2.409	1.943
NR_07348	chr13	19422879	19536763	-	1485	1	TPTE2|TPI	0	0.138
NM_0012!	chr1	1.56E+08	1.56E+08	-	3313	1	RIT1|NS8|	13.053	12.345
NR_03893	chr14	39265703	39267061	-	1358	1	LOC10028	0.665	0.565
NR_04500	chr10	38696596	38698240	+	1644	1	ACTR3BP5	0	0
NM_0252:	chr10	91798394	91865475	+	6245	1	TNKS2|AR	9.13	8.933
NR_03893	chr12	68674371	68686859	-	1726	1	LOC10050	0.224	0.198
NR_04687	chr13	40181809	40189030	+	832	1	LINC00332	0	0
NM_0033:	chr2	1.79E+08	1.79E+08	-	81834	1	TTN|CMD:	0.397	0.087

NM_0012!	chr3	42809472	42867283	+	2955	1	ACKR2|CC	0.065	0.088
NM_0062:	chr7	44114680	44123570	-	1618	1	POLD2|-|-	77.297	70.009
NM_0013:	chr13	98793487	99086406	-	7396	1	DOCK9|ZIZ	0.893	1.076
NR_01540	chr2	7922425	7976814	-	2162	1	LINC0029۶	0	0
NR_13298	chr4	1.85E+08	1.85E+08	-	1916	1	MIR3945H	0	0
NM_0156{	chr14	50312329	50323025	+	1354	1	ATP5S|ATI	3.523	4.481
NM_0011<	chr9	87917962	87923657	+	3773	1	SPATA31C	0	0
NM_0328(	chr16	3088891	3099317	-	2730	1	ZSCAN10|:	0.076	0.03
NR_03061	chr14	1.01E+08	1.01E+08	+	71	1	MIR665|M	0	0
NR_03838	chr6	99994038	1E+08	+	545	1	MCHR2-AS	0	0
NM_0012{	chr2	1.6E+08	1.6E+08	-	4387	1	ITGB6|AI1	0.021	0.037
NM_0011<	chr7	45080437	45088894	-	4752	1	NACAD|-|-	54.141	40.296
NR_04622	chr20	62774121	62776856	-	569	1	LINC0065S	0.385	0
NM_0244!	chr14	77024543	77028699	-	4154	1	IRF2BPL|C	38.879	43.564
NM_0066<	chr9	93184930	93320572	+	6803	1	WNK2|NY-	22.385	23.285
NM_0013:	chr10	48156559	48251757	-	4636	1	FRMPD2|F	0.044	0.068
NR_12160	chr9	61867082	61869138	-	437	2	LOC10272:	0	0
NR_02331	chr19	52596684	52600152	+	2481	1	ZNF137P|Z	0.54	1.016
NM_0013(	chr1	1.11E+08	1.12E+08	-	1747	1	ADORA3|A	0	0
NM_1382{	chr13	20703343	20723098	+	1856	1	IL17D|IL-1	3.772	4.059
NM_0177:	chr3	37052626	37176360	-	2835	1	LRRFIP2|H	10.829	8.296
NR_01544	chr5	43042134	43045268	+	2258	1	LOC15368-	0.126	0.224
NM_0010(	chr9	1.23E+08	1.23E+08	+	934	1	OR1L4|OR	0	0
NR_02666	chr2	2.39E+08	2.39E+08	-	2126	1	MGC1602!	0.121	0.148
NR_11007	chr7	77415198	77425443	+	675	1	LOC10192	0	0
NM_0067<	chr11	65638107	65650920	+	3483	1	SIPA1|SPA	5.923	7.381
NR_10404	chr16	56676116	56677877	+	326	1	MT1IP|MT	0	0
NM_0164:	chr7	1.3E+08	1.3E+08	-	1960	1	ZC3HC1|H	16.071	17.694
NR_13090	chr1	11654857	11663327	+	2684	1	FBXO44|FI	22.679	24.592
NR_02425	chr18	26687621	26703638	-	2596	1	PCAT18|LI	0.268	0.375
NM_0044<	chr3	1.35E+08	1.35E+08	+	4657	1	EPHB1|ELI	54.78	26.471
NM_0013:	chr2	1.03E+08	1.03E+08	+	3370	1	TMEM182	1.664	1.792
NR_03033	chr10	29545004	29545097	-	93	1	MIR604|M	0.277	0
NR_03968	chr12	1.16E+08	1.16E+08	-	66	1	MIR4472-:	0	0
NR_12031	chr15	80195479	80252261	-	6940	1	LINC01314	0.216	0.341
NM_0319!	chr3	1.73E+08	1.73E+08	-	2094	1	SPATA16|I	0	0.013
NM_0201!	chr19	7619525	7629553	-	2634	1	XAB2|HCN	34.157	33.478
NM_1815:	chr17	40792196	40799959	-	1682	1	KRT28|K2!	0	0
NR_03890	chr8	65161145	65180340	-	1050	1	LINC00251	0	0.091
NM_0012:	chr15	40161009	40221136	+	3717	1	BUB1B|BU	3.309	4.09
NM_0011!	chr1	52372829	52404471	-	3135	1	ORC1|HSC	2.106	1.826
NM_0146!	chr15	34856351	34969794	-	5043	1	AQR|IBP1(	6.982	8.384
NR_03986	chr14	1.05E+08	1.05E+08	-	55	1	MIR4710|	0	0
NM_0012!	chr8	7971661	7973253	-	1591	1	USP17L8|U	0	0
NR_03063	chr20	63919868	63919939	+	71	3	MIR941-3|	0	0
NR_04675	chrX	1.08E+08	1.08E+08	-	455	1	FRMPD3-A	0.397	0.632
NR_03780	chr2	2.4E+08	2.4E+08	+	1014	1	LOC15093:	0	0.027
NM_0011:	chr4	24806117	24912978	-	3947	1	CCDC149|	7.33	6.448
NM_0011!	chr17	76734115	76737411	-	1963	1	SRSF2|PR2	82.911	65.087
NM_0010:	chr3	1.96E+08	1.96E+08	-	1327	1	ZDHHC19|	0.107	0.165

NR_12584	chr6	51621870	51622577	+	273	1	LOC10192	0	0
NR_12650	chr4	70397882	70410197	+	975	1	OPRPN|BF	0.013	0
NM_0013	chr1	1.45E+08	1.45E+08	-	1320	2	FAM72C|-	0.039	0.021
NM_0051	chr16	84699949	84779921	+	3362	1	USP10|UB	25.116	31.293
NM_0013	chr2	1.3E+08	1.3E+08	-	1682	1	CCDC115|	40.712	46.075
NM_0064	chr17	28577565	28599038	-	3782	1	SPAG5|DE	7.122	6.168
NM_0189	chr5	1.41E+08	1.42E+08	+	4572	1	PCDHGB5|	20.24	86.948
NR_12055	chr11	38648858	38655249	+	503	1	LINC0149	0	0
NM_0003	chr2	21001429	21044073	-	14090	1	APOB|FLD	0.002	0
NM_1527	chr6	56954975	57027344	+	2689	1	BEND6|C6	0.379	0.713
NM_0011	chr9	1.34E+08	1.34E+08	-	3195	1	SARDH|BP	1.247	0.802
NM_1453	chr17	15502264	15563631	-	1606	1	TVP23C|F	0.803	1.595
NM_0016	chr1	1.69E+08	1.69E+08	+	2192	1	ATP1B1|A	75.884	82.757
NR_13426	chr5_GL33	1319530	1445241	-	484	6	LOC10272	0	0
NM_0012	chr17	41029677	41030125	-	446	2	KRTAP1-4|	0	0
NR_03783	chr22	23359606	23382126	-	873	2	CES5AP1|-	0	0
NR_02671	chr20	26054614	26086917	+	5106	1	FAM182A|	0.015	0.123
NM_1527	chr19	1229948	1237991	-	2553	1	CBARP|BA	22.58	17.082
NR_00369	chr19	53365715	53386588	+	6090	1	ZNF525|-|	0.343	0.846
NM_0010	chr1	2.48E+08	2.48E+08	+	952	1	OR2G2|OF	0	0
NM_0320	chr7	43866558	43869546	-	690	1	MRPS24|H	62.375	54.312
NM_0003	chr3	1.25E+08	1.25E+08	+	6729	1	UMPS|OP	4.215	4.832
NR_02673	chr13	24928755	24968469	-	6472	1	TPTE2P1|-	0.438	0.605
NM_1736	chr2	1.86E+08	1.86E+08	+	20762	1	FSIP2|-|-|	0.207	0.579
NM_0012	chr4	69480165	69526014	-	1924	1	UGT2B4|H	0.054	0.093
NM_0011	chrX	1.54E+08	1.54E+08	-	1224	1	FAM58A|S	14.389	13.16
NR_03607	chr2	96798278	96798353	+	75	1	MIR3127|	0	0
NM_0013	chr8	17922857	18029948	+	8718	1	PCM1|PTC	7.705	10.801
NR_13448	chr10	47743699	47759041	+	667	1	LOC10272	0.232	0.452
NR_02693	chr6	41101034	41140835	+	4546	1	ADCY10P1	0.709	1.208
NR_00258	chr3	1.87E+08	1.87E+08	+	136	1	SNORA4|A	3.697	5.841
NM_0061	chr7	1.28E+08	1.28E+08	-	1999	1	PAX4|KPD	0	0.041
NM_0011	chr2	36696690	36814794	+	2693	1	VIT|-|-|2p	0.034	0.127
NR_02744	chr9	1.37E+08	1.37E+08	-	1871	1	MAN1B1-A	1.716	2.496
NM_0010	chr14	1.03E+08	1.03E+08	+	2566	1	EXOC3L4|	0.015	0.011
NM_0004	chr6	1.16E+08	1.16E+08	-	3285	1	COL10A1|	0.039	0.017
NM_0006	chr1	1.66E+08	1.66E+08	-	2487	1	ALDH9A1|	18.581	30.682
NM_0062	chrX	18690926	18827916	+	2851	1	PPEF1|PP	2.058	1.734
NR_11064	chr16	47849314	47887130	-	1323	1	LOC10050	0.263	1.18
NM_0013	chrX	66595640	66639298	-	2808	1	EDA2R|ED	1.938	2.097
NM_0151	chr3	32106511	32168715	+	4052	1	GPD1L|GP	3.815	10.776
NM_0528	chr19	41310163	41324883	+	3341	1	CCDC97|-|	23.151	20.625
NR_11015	chr2	66574030	66695588	+	759	1	LINC0179	0.764	0.036
NM_0010	chr7	12686827	12690933	+	2981	1	ARL4A|AR	10.342	3.832
NM_0011	chr9	1.23E+08	1.23E+08	-	3968	1	RC3H2|MI	12.838	16.182
NM_0240	chr16	90004865	90010121	-	2075	1	DBNDD1|-	35.07	31.124
NM_0149	chr13	49110303	49209779	+	5884	1	FNDC3A|F	7.983	8.133
NM_0011	chr11	18455827	18479600	+	1980	1	LDHAL6A|	0.13	0.173
NM_0013	chr15	49422515	49620931	-	1470	1	FAM227B|	1.114	0.662
NR_12537	chr10	88932684	88939974	+	2038	1	ACTA2-AS	3.625	12.823

NR_14454	chr17	22202995	22205156	+	1459	1	UBBP4|-|-	0.265	0.178
NM_0041	chr19	6375148	6375916	-	680	1	PSPN|PSP	2.503	3.303
NM_1446	chr8	66432483	66468809	+	1879	1	ADHFE1|A	0.398	0.532
NM_0540	chr5	1.48E+08	1.48E+08	+	513	1	SCGB3A2|	0	0.053
NM_0028	chr8	60516910	60623644	+	3802	1	RAB2A|LH	34.184	33.086
NM_0320	chr14	94127781	94129620	-	461	2	IFI27L2|FA	0	0
NM_0010	chr13	99254713	99258428	-	1465	1	GPR18|-|-	0	0
NR_12158	chr9	3181589	3200500	+	1489	1	LINC01231	0	0
NM_0012	chr2	1.9E+08	1.9E+08	+	3404	1	PMS1|HNI	1.829	2.933
NM_1523	chr19	44385646	44401611	-	2680	1	ZNF285|ZI	3.579	2.586
NR_05196	chr1	1.51E+08	1.51E+08	+	564	1	FALEC|FAL	1.395	1.724
NM_0012	chr6	28267359	28278223	+	2317	1	ZSCAN26|	11.931	17.238
NR_03838	chr2	97669743	97703066	+	1359	1	LINC01125	1.281	1.502
NM_0163	chr1	28236091	28238105	+	536	1	ATPIF1|AT	148.107	92.48
NR_00129	chr15	25278343	25278409	+	66	2	SNORD109	0	0.415
NM_0060	chr12	49184795	49189324	-	1910	1	TUBA1A|B	3660.297	4021.838
NM_0019	chr8	6924694	6926076	-	464	1	DEFA6|DE	0	0
NR_00258	chr3	12840312	12840450	-	138	1	SNORA7A|	1.962	0.099
NR_12372	chr16_KI2	533654	565020	-	2884	2	PKD1P6-NI	0.617	0.736
NM_0202	chr9	95875618	96018453	+	10303	1	ERCC6L2|E	1.49	2.001
NM_0012	chr16	30762289	30776307	+	5358	1	RNF40|BR	47.664	46.546
NM_0014	chr12	1.24E+08	1.24E+08	-	1859	1	EIF2B1|EIF	30.399	30.061
NM_1813	chr7	99027435	99144120	-	5665	1	SMURF1|-	20.138	21.86
NM_1985	chr19	17470444	17506168	+	3580	1	SLC27A1|A	8.514	9.263
NR_13577	chr2	74529405	74533556	+	1993	1	HTRA2|MO	29.649	31.098
NM_0011	chr19	2289775	2308157	-	2126	1	LINGO3|LE	0.843	1.057
NR_13583	chr9	83707835	83713493	+	1472	1	LOC10537	2.794	2.252
NR_12871	chr21	8208473	8208652	+	179	2	MIR3648-2	0	0
NR_02986	chr2	2.19E+08	2.19E+08	-	63	1	MIR375|M	0	0
NM_0015	chr6	24425834	24489622	-	5805	1	GPLD1|GP	1.031	1.081
NR_03749	chr16	56245520	56245623	+	103	1	MIR3935|	0	0
NM_0010	chr4	1.59E+08	1.59E+08	-	3525	1	C4orf46|R	3.292	3.349
NM_0330	chr13	42562736	42608013	+	2350	1	TNFSF11|C	0	0
NM_0008	chr1	1.1E+08	1.1E+08	+	1560	1	GSTM5|GS	0	0.018
NM_1526	chrX	34942796	34944917	+	2119	1	FAM47B|-	0	0
NM_0016	chr2	2.32E+08	2.32E+08	+	3261	1	ALPI|IAP|-	0	0
NM_0012	chr3	1.22E+08	1.22E+08	-	11096	1	GOLGB1|G	3.512	5.338
NM_1749	chr8	1.44E+08	1.44E+08	+	1971	1	ADCK5|-|-	3.722	3.537
NR_10465	chr5	54660158	54703324	-	576	1	LOC10246	0.134	0
NM_0010	chr10	88284102	88583325	-	2407	1	RNLS|C10	0.011	0.028
NR_02786	chr2	1.72E+08	1.72E+08	+	1584	1	HAT1|KAT	8.97	10.999
NM_0013	chr16	3401190	3409366	+	2018	1	ZNF174|ZS	11.565	14.939
NR_02949	chr17	59841266	59841337	+	71	1	MIR21|MI	1.09	1.736
NM_1813	chr14	72926332	72959649	+	2437	1	DCAF4|WI	4.481	8.571
NM_1993	chr1	42807052	42817388	-	793	1	SVBP|CCD	121.293	105.759
NR_03788	chr2	1.04E+08	1.04E+08	-	1081	1	LOC10028	0	0.038
NR_00319	chr14	1.01E+08	1.01E+08	+	103	1	SNORD114	0	0
NM_0033	chr11	73974671	73982844	-	1636	1	UCP2|BMI	13.76	30.709
NR_10462	chr1	11609531	11613354	+	1281	1	LINC01647	0	0.096
NR_02697	chr8	1.45E+08	1.45E+08	+	3235	1	ZNF252P-A	0.163	0.144

NR_12571	chr7	1.25E+08	1.25E+08	+	3271	1	POT1-AS1|	0.386	0.318
NM_0021!	chr2	1.2E+08	1.2E+08	+	3201	1	INHBB|-|-	0.939	4.065
NM_0012(	chr10	179994	249789	+	2391	1	ZMYND11	15.142	19.483
NR_11089	chr17	21075556	21090613	+	746	1	LINC01563	0.156	1.377
NM_0013:	chr10	30434635	30461833	+	2528	1	MAP3K8|/	1.061	2.882
NR_04987	chr7	97963658	97963726	+	68	2	MIR5692A	0	0
NM_0807!	chr10	69801888	69959148	+	2884	1	COL13A1|	0.21	0.195
NR_12603	chr17_KI2	138752	145555	-	6803	2	LRRC37A8I	0	0
NM_0013:	chr15	45502402	45522804	-	1409	1	SLC30A4|Z	0.851	0.875
NM_0011:	chr16	50319018	50368934	-	2293	1	BRD7|BP7	11.004	12.472
NM_0010(	chr11	4954772	4955713	-	940	1	OR51A2|-	0	0
NM_0010:	chr1	39895424	39902015	-	3255	1	MYCL|L-M	18.661	20.548
NM_0025:	chr17	5384833	5419739	-	3669	1	NUP88|-|-	19.061	21.791
NR_13631	chr9	64463599	64469259	+	1538	3	LOC10537!	0	0
NM_1985:	chr19	35523367	35528351	-	950	1	SBSN|UNC	0.095	0.072
NR_03971	chr11	1.23E+08	1.23E+08	-	72	1	MIR4493|	0	0
NR_10400	chr7	1.43E+08	1.43E+08	+	3228.5	2	EPHB6|HE	0.072	0.187
NR_02771	chr12	14774336	14778002	+	3351	1	H2AFJ|H2/	0.2	0.981
NM_0224!	chr12	69738686	69823204	+	9499	1	RAB3IP|R/	1.971	1.988
NM_1944	chr12	8123632	8138607	+	1059	1	CLEC4A|CI	0.511	0.867
NM_0049:	chrX	48961378	48971844	-	4712	1	KCND1|KV	1.185	0.892
NM_0012!	chr3	44498970	44510640	-	1963	1	ZNF852|-|	2.128	1.751
NR_00303	chr11	50279828	50298462	-	1009	1	LOC44160	0	0
NM_0010(	chr1	1.58E+08	1.58E+08	-	937	1	OR10K2|C	0	0
NR_03747	chrX	32583656	32583752	-	96	1	MIR3915|	0	0
NM_1527!	chr7	1E+08	1E+08	+	1459	1	CNPY4|PR	20.856	15.105
NR_11018	chr7	1.25E+08	1.25E+08	+	1427	1	LOC10192	0.108	0.029
NM_0001:	chr1	1.7E+08	1.7E+08	-	9152	1	F5|FVL|PC	0.018	0.016
NM_0010:	chr14	80955525	81108950	+	1176	1	TSHR|CHN	0.044	0.547
NR_10379	chr2	1.02E+08	1.02E+08	+	992	1	LINC01127	0	0.028
NR_10353	chr1	2.28E+08	2.28E+08	-	600	1	IBA57-AS1	0.623	0.89
NM_0012:	chr2	2.39E+08	2.39E+08	+	1405	1	TWIST2|AI	0	0.136
NR_03834	chr7	79453960	79471208	+	1067	1	MAGI2-AS:	2.417	2.49
NR_11020	chr3	1.52E+08	1.52E+08	-	521	2	AADACL2-/	0	0
NR_11194	chr1	13094515	13099717	-	2210	4	PRAMEF36	0	0
NM_0010:	chrX	27977993	27981449	-	3454	1	DCAF8L1|'	0.052	0.036
NR_04657	chr12	2220537	2223481	-	733	1	CACNA1C-	0	0
NM_0044(	chr1	3857267	3885429	+	3033	1	DFFB|CAD	3.669	5.478
NM_0057:	chr5	83940595	84384867	-	4747	1	EDIL3|DEL	19.73	12.081
NM_0133(	chr12	1.1E+08	1.1E+08	+	1512	1	FAM216A|	13.405	15.255
NM_0011	chr4	15968226	16076118	-	3831	1	PROM1|A	3.278	1.873
NM_0178:	chr13	1.11E+08	1.11E+08	-	1518	1	RAB20|-|-	1.376	1.525
NR_04984	chr18	39676721	39676779	+	58	1	MIR5583-1	0	0
NR_00321	chr14	1.01E+08	1.01E+08	+	70	1	SNORD114	0	0
NM_0010!	chr16	2275878	2340746	-	6588	1	ABCA3|AB	34.858	35.968
NR_03831	chr21	44921035	44929680	+	2348	1	ITGB2-AS1	0.011	0.041
NM_0219(	chrX	73212301	73214874	-	2571	1	NAP1L2|B	4.679	2.515
NM_1384:	chr7	1.5E+08	1.5E+08	+	2870	1	ZBED6CL|(	0.004	0.091
NM_0239:	chr20	3190350	3204697	-	1307	1	DDRGK1|C	46.01	35.16
NM_0013:	chr3	75729961	75785004	-	1341	1	ZNF717|X:	0.923	1.113

NM_2068!	chr6	1.63E+08	1.64E+08	+	14127	1	QKI|Hqk|(	5.456	7.066
NR_13017	chrX	1.04E+08	1.04E+08	+	975	2	LOC10010	0.463	0.478
NR_02441	chr5	1.36E+08	1.36E+08	-	1695	1	LOC38933.	0.19	0.307
NR_12632	chr18	71732616	71782226	-	569	1	LINC0189S	0	0
NM_0012:	chr20	21125983	21246620	+	1960	1	KIZ|C20ori	5.736	6.569
NM_0309(	chr17	41033885	41034855	-	968	2	KRTAP1-3|	0	0
NR_03985	chr13	51552589	51552667	+	78	1	MIR4703|-	0	0
NR_03823	chr8_KI27(	56690	71368	+	2803	2	LINC0160€	0	0
NR_13236	chr13	44106091	44147735	-	815	1	LINC0039C	0	0.034
NR_13265	chr11	29980121	29982393	-	2272	1	LINC0161€	0	0.006
NM_0001:	chr7	1.08E+08	1.08E+08	-	2871	1	SLC26A3|(	0	0
NM_0011·	chr8	69672333	69833169	-	3404	1	SLCO5A1|(	3.769	6.016
NM_0013:	chr11	57703709	57712323	-	1201	1	MED19|D	36.147	38.069
NM_0019:	chr12	6914450	6923696	+	2409	1	ENO2|HEL	472.761	352.785
NR_00300	chr3	1.61E+08	1.61E+08	-	329	1	SCARNA7|	0.039	0.125
NM_1536·	chr22	45164671	45188009	+	5038	1	NUP50|NF	7.079	5.772
NM_0010:	chrX	15243987	15270171	-	1692	1	ASB9|-|-|)	0	0
NM_0054:	chr7	1.53E+08	1.53E+08	-	3064	1	XRCC2|FAI	0.476	0.881
NM_0010(	chr21	36043684	36060364	-	1158	1	SETD4|C2:	14.085	13.839
NR_14456	chr11	65506117	65506174	+	57	1	MASCRNA	0	0.721
NM_0240!	chr2	1.05E+08	1.05E+08	+	4365	1	C2orf49|a:	4.224	4.428
NM_1783!	chr1	1.53E+08	1.53E+08	+	595	1	LCE1F|LEP	0	0
NR_07342	chr8	7028601	7029489	-	299	1	DEFA11P|I	0	0
NM_0010{	chr16	52436416	52546894	-	4702	1	TOX3|CAG	10.903	12.645
NM_0011!	chr6_GL0C	1655361	1657092	+	573.3	7	RPP21|C6(	4.206	2.341
NR_00275	chr1	44721786	44721901	-	115	1	RNU5F-1|I	0.897	0.119
NM_0011(	chr7	1.01E+08	1.01E+08	+	16307	1	MUC12|M	0.077	0.015
NM_0011·	chr6_GL0C	4508832	4512370	-	2642	5	ZBTB22|BI	0	0
NM_0209:	chr4	2269597	2418643	-	4097	1	ZFYVE28|L	1.193	1.635
NM_0157:	chr1	2.1E+08	2.1E+08	+	959	1	G0S2|-|-|:	0.592	0.143
NR_13523	chr19	22615557	22623971	-	1110	1	LINC0178€	0	0
NR_12871	chrUn_GL(	104732	104823	+	91	7	MIR6724-4	0	0
NR_00217	chrY	24570202	24607025	+	1342	3	TTTY4C|LII	0	0
NM_0010(	chrX	1.03E+08	1.03E+08	-	1135	1	TCEAL8|W	44.826	41.634
NM_0330!	chr9	1.13E+08	1.13E+08	-	2496	1	SLC46A2|L	0.041	0.005
NR_02986	chr19	53787675	53787741	+	66	1	MIR371A|	0	0
NM_0140·	chr3	52705841	52708181	+	1078	1	SPCS1|HSI	70.28	55.525
NM_0013(	chr20	58889125	58911196	+	1627	1	GNAS|AH(	856.882	947.392
NM_0323!	chr3	99817834	1E+08	+	1218	1	CMSS1|C3	18.271	22.109
NR_04987	chr3	85385710	85385792	+	82	1	MIR5688|-	0	0
NM_1385!	chr5	1.11E+08	1.11E+08	+	2407	1	TSLP|-|-|5	0.021	0.006
NM_0023·	chr8	1.43E+08	1.43E+08	-	919	2	LY6H|NML	0	0
NM_1533·	chr4	1.84E+08	1.84E+08	-	3926	1	ENPP6|NP	0.013	0.059
NM_0011·	chr2	1.59E+08	1.59E+08	+	7459	1	TANC1|RC	1.058	2.097
NM_0013!	chr8	90001352	90051999	+	1239	1	DECR1|DE	16.765	27.062
NM_0012:	chr6	65301418	65305611	+	3820	1	LOC44115.	0	0.004
NM_1735·	chr2	68467559	68580162	+	3837	1	APLF|APFL	0.208	0.293
NM_0249:	chr2	70978445	70985499	+	2111	1	ANKRD53|	0.275	0.422
NR_03388	chr19	43211791	43248646	-	1712	1	LOC28434·	0	0
NM_1816:	chrX	71533062	71575897	+	5458	1	OGT|HINC	23.992	28.957

NM_0013	chr3	51541589	51668660	+	9809	1	RAD54L2|	13.862	12.989
NR_07317	chr17	78261349	78278492	-	2927	1	LINC0199	0.07	0.098
NM_0151	chr15	78170845	78234707	-	2964	1	ACSBG1|B	0.052	0.152
NR_12646	chr3	71289758	71305854	+	707	1	FOXP1-AS	0	0
NR_11028	chr2	76985965	77009788	+	993	1	LOC10192	0	0
NR_10356	chr1	1.52E+08	1.52E+08	+	998	1	NBPF18P|	0	0
NR_10694	chr15	74411357	74411432	-	75	1	MIR6881|	0.688	1.644
NM_0013	chr16	67109893	67148544	+	2749	1	C16orf70|	11.613	11.933
NM_0071	chrX	57591836	57597477	+	5639	1	ZXDB|ZNF	1.472	2.322
NR_00274	chr2	28927067	28927142	+	75	1	SNORD53|	0	0
NR_10355	chr8	1.28E+08	1.28E+08	-	2164	1	TMEM75|	0	0
NM_0012	chr16	1528741	1555580	+	1627	1	TMEM204	0.103	0.051
NM_1523	chr11	1.15E+08	1.15E+08	-	1917	1	NXPE1|FA	0	0
NM_0029	chr22	50445001	50475071	-	8011	1	SBF1|CMT	47.28	58.509
NM_0162	chr2	1.02E+08	1.02E+08	+	2045	1	IL1RL1|DE	0	0
NM_0010	chr1	8352404	8423687	-	6293	1	RERE|ARG	56.26	56.849
NR_03644	chr12	8133769	8134852	-	1083	1	POU5F1P3	0.083	0.013
NR_03020	chr19	53716594	53716680	+	86	1	MIR521-2|	0	0
NR_11039	chr2	2.17E+08	2.17E+08	-	1613	1	LINC0128	0.024	0
NM_0036	chr5	1.32E+08	1.32E+08	+	2286	1	PDLIM4|R	6.069	6.453
NM_0206	chr19	1815246	1848453	-	4563	1	REXO1|EL	16.264	14.769
NM_0013	chr16	57999599	58021618	+	2149	1	USB1|C16	42.772	46.7
NM_0011	chr3	1.6E+08	1.6E+08	+	2575	1	C3orf80|-|	0.531	0.25
NM_0012	chr4	6575174	6622406	+	4014	1	MAN2B2|	6.248	6.879
NR_11029	chr2	2.1E+08	2.1E+08	+	1537	1	LOC10192	0.101	0.267
NR_00439	chr20	2656097	2656182	+	85	1	SNORD86|	12.893	11.763
NR_13299	chr2	60359216	60391375	-	701	1	MIR4432H	0	0
NM_0011	chr1	40691570	40771606	+	2077	1	NFYC|CBF	40.034	42.232
NM_0010	chr17	44677437	44689797	-	2073	1	CCDC43|-|	8.981	8.933
NR_02676	chr15	69835234	69842967	+	1325	1	LINC0059	0	0.01
NM_0011	chr7	97852117	97872165	-	1692	1	ASNS|ASN	95.817	155.12
NM_0017	chr6	26501267	26510424	+	2842	1	BTN1A1|B	0.014	0.029
NM_0026	chr4	73853296	73854484	+	739	1	PF4V1|CX	0	0
NM_0068	chr5	1.48E+08	1.48E+08	+	3604	1	SPINK5|LE	1.488	2.155
NM_0143	chr12	1.09E+08	1.09E+08	-	3831	1	CORO1C|	125.398	139.859
NM_0252	chr15	78283236	78299726	-	1305	1	WDR61|R	43.947	40.965
NM_0012	chr6	35118071	35141410	-	1781	1	TCP11|D6	0.282	0.531
NR_10686	chr1	1339650	1339708	-	58	1	MIR6808|	0.445	0.708
NR_03615	chr18	14830166	14830242	+	76	1	MIR3156-	0	0
NR_00280	chr14	93067452	93072133	+	4681	2	ITPK1-AS1	0	0
NR_13499	chr12	1.19E+08	1.19E+08	-	574	1	LOC10537	0	0
NM_0010	chr4	1.46E+08	1.46E+08	+	837	1	C4orf51|-|	0	0
NM_0014	chrX	1.33E+08	1.33E+08	-	3703	1	GPC4|K-gl	4.603	14.814
NM_0013	chr1	1.01E+08	1.01E+08	+	2938	1	S1PR1|CD	4.094	6.895
NR_13558	chr2	1.97E+08	1.97E+08	+	453	1	LOC10192	0	0
NR_13071	chr20	35968581	36030700	+	1490	1	CNBD2|C2	0.286	0.11
NR_12118	chr9	72690	75294	+	538	1	PGM5P3-A	0	0
NM_0122	chrX	48476021	48486364	+	2076	1	FTSJ1|CDL	31.88	38.597
NM_0210	chr2	27199587	27212307	-	3233	1	SLC5A6|SM	13.755	12.557
NR_11100	chr9	92882567	92889555	+	5049	1	LOC64294	0	0

NR_04499	chr6_KI27(	60294	63457	+	611.8	9	HCG23|dJ:	0	0
NM_2073(	chr19	46120071	46124674	-	656	1	IGFL3|UN(	0.039	0
NM_0002(	chrX	18892298	18984362	-	5290	1	PHKA2|GS	5.201	6.437
NR_03157	chr16	30893903	30893985	+	82	1	MIR762|h:	4.717	5.512
NM_0011(	chr3	1.5E+08	1.5E+08	-	4968	1	WWTR1|T	3.716	1.952
NR_10677	chr10	1.12E+08	1.12E+08	-	76	1	MIR6715B	0	0
NM_0010	chr5	1.39E+08	1.39E+08	-	1025	1	ECSCR|ARI	0	0
NM_0010:	chr11	71565563	71566620	+	1055	1	KRTAP5-1(	0.086	0.078
NM_0123(	chr16	3355889	3356924	+	1033	1	OR2C1|OL	0.025	0.172
NR_03073	chr17	60083566	60088467	-	618	1	WFDC21P|	0.063	0.044
NM_0067(	chr11	65084222	65088402	+	1376	1	ZFPL1|D11	30.594	26.1
NR_03030	chr4	1.65E+08	1.65E+08	+	95	1	MIR578|M	0	0
NM_0051:	chr11	69698238	69704338	-	2132	1	FGF19|-|-	0.913	0.218
NR_11052	chr14	19294790	19337674	-	2385	2	DUXAP10|	0	0
NM_0246(	chr10	11742357	11764066	+	1647	1	ECHDC3|-	0	0.008
NM_0011(	chr11	90085950	90087131	+	1180	1	UBTFL1|C:	0	0
NM_0056(	chr18	57646426	57803095	-	5908	1	ATP8B1|A	0.05	0.035
NM_0012(	chr3	15601745	15642252	+	755	1	BTD|-|-|3	3.33	3.501
NR_14438	chr12	4487730	4538508	-	3214	1	C12orf4|-|	4.67	6.017
NM_0012	chr19	24087160	24107352	+	532	1	ZNF254|BI	5.623	4.506
NM_0055(	chr17	78971253	78979979	-	2252	1	LGALS3BP	61.902	79.847
NR_12586	chr6	68055351	68060502	+	490	1	LOC10272.	0	0
NR_03760	chr16	30194433	30204329	+	2205	2	SLX1A-SUL	0.076	0.043
NR_12060	chr2	47527537	47529164	+	431	1	HCG20400	0.06	0.286
NM_0055	chr19	17816513	17821574	-	801	1	INSL3|RLF	0.048	0.085
NM_0177!	chr2	2.06E+08	2.06E+08	-	14123	1	INO80D|-|	1.231	1.726
NR_10699	chr15	85380596	85380662	+	66	1	MIR7706|	0	0
NM_0012(	chr14	64914361	65062655	+	2808	1	CHURC1-FI	16.599	15.521
NM_0010:	chrX	1.16E+08	1.16E+08	-	570	1	CT83|CXoı	0	0
NM_0012	chr22	29306995	29312789	+	1827	1	GAS2L1|G.	5.039	7.1
NM_0163	chr1	2.15E+08	2.15E+08	+	10274	1	CENPF|CEI	1.015	3.542
NM_0010(	chr11	7938576	7939520	-	943	2	OR10A3|H	0	0
NM_0176(	chr2	1.9E+08	1.91E+08	+	4787	1	MFSD6|M	2.952	4.967
NM_0013:	chr11	1274368	1309662	-	3503	1	TOLLIP|IL-	22.511	29.603
NR_03844	chr5	1.57E+08	1.57E+08	+	1990	1	PPP1R2P3	1.017	1.335
NR_10499	chr5	1.18E+08	1.18E+08	+	876	1	LINC02147	0	0.109
NM_1522(	chr19	11866029	11869491	+	2545	1	ZNF439|-|	3.855	2.056
NM_0011(	chr6	1.39E+08	1.39E+08	+	4320	1	ECT2L|ARI	0.063	0.117
NM_0177(	chr19	1985448	2015703	-	2608	1	BTBD2|-|-	68.091	74.546
NR_12039	chr13	43448852	43459488	+	710	1	ENOX1-AS:	0.036	0
NM_0046	chr11	1.01E+08	1.02E+08	-	4597	1	TRPC6|FS(	0.022	0.31
NR_13198	chrX	1.54E+08	1.54E+08	+	553	1	LOC10537.	8.277	7.331
NM_0223(	chr15	74179466	74202879	-	2779	1	STRA6|M(	0.371	0.276
NM_0012!	chr3	8619400	8644798	-	1622	1	SSUH2|C3	0.191	0.135
NM_0808:	chr20	62410237	62427573	-	2817	1	RBBP8NL|	0	0
NR_00305	chr2	2.02E+08	2.02E+08	+	87	1	SNORD70|	0	0
NR_00328	chr5	1.78E+08	1.78E+08	-	2922	1	GMCL1P1|	0.079	0.22
NR_10403	chr1	1.46E+08	1.46E+08	+	1031	1	GNRHR2|(	13.406	10.814
NM_0045	chrX	1.2E+08	1.2E+08	+	481	1	NDUFA1|(	250.203	232.764
NR_12157	chr9	1.03E+08	1.03E+08	-	3722	1	LINC01492	0	0

NM_0013:	chr2	1.64E+08	1.64E+08	-	7022	1	FIGN|-|-|2	3.981	1.537
NR_00001	chr1	44775865	44775938	+	73	1	SNORD55|	6.005	5.629
NR_03981	chr1	2.34E+08	2.34E+08	+	72	1	MIR4671|-	0	0
NM_0010(	chr9	92711363	92764801	-	6407	1	BICD2|SM	15.218	13.205
NM_0012{	chr19	3544199	3557584	-	2096	1	MFSD12|C	16.96	14.651
NM_0013(	chr20	381306	397559	+	1974	1	TRIB3|C20	17.982	20.886
NM_0005(	chr1	1.1E+08	1.1E+08	+	1175	1	GSTM1|G!	0.099	0.21
NM_0208:	chr3	74262569	74521140	-	4923	1	CNTN3|BI(	1.522	1.135
NM_0011:	chr10	1.03E+08	1.03E+08	-	1647	1	CALHM3|F	0	0.033
NM_0012!	chr8	12132417	12134009	-	1591	1	USP17L7|-	0	0
NM_0028:	chr22	37225261	37244299	-	1502	1	RAC2|EN-:	0.052	0.073
NR_02950	chr9	21512115	21512185	-	70	1	MIR31|MI	0	0
NR_02458	chr7	1.05E+08	1.05E+08	-	3599	1	KMT2E-AS	1.927	2.302
NR_10983	chr6	36673621	36675126	-	1505	1	PANDAR|F	1.182	1.292
NR_02411	chr7	44028887	44040623	-	1518	1	RASA4CP|	1.07	0.839
NM_1824{	chr13	27066150	27171896	-	4492	1	USP12|UB	3.895	4.373
NM_1774:	chr11	70270700	70378492	+	4034	1	PPFIA1|LIF	7.144	7.405
NM_0052:	chr7	1.43E+08	1.43E+08	-	3343	1	EPHA1|EP	0.015	0.037
NM_0160(	chr16	4340251	4351372	-	577	2	PAM16|C(	0	0
NM_0183:	chr14	70366496	70417090	-	7054	1	SYNJ2BP|/	3.274	3.544
NM_0069!	chr19	57411161	57421939	+	2673	1	ZNF17|HP	3.029	4.325
NM_0184	chr8	80967811	81112068	-	10732	1	PAG1|CBP	2.082	5.058
NM_0140(	chr6_GL0C	2374195	2399422	+	899.7	6	PSORS1C1	0	0
NR_03651	chr19	13834516	13836289	-	1773	1	LOC28445	0.436	0.61
NM_0048:	chr5	1.54E+08	1.54E+08	-	1734	1	HAND1|H;	0.037	0.039
NM_0013:	chr1	26543112	26575029	+	3015	1	RPS6KA1|I	9.023	11.535
NM_0038:	chr15	42495306	42533061	+	2631	1	SNAP23|H	3.161	3.212
NM_0010:	chr12	27696495	27697633	+	1136	1	REP15|RAI	0.295	0.386
NR_04988	chr12	94561789	94561859	+	70	1	MIR5700|-	0	0
NM_0013(	chrX	1.51E+08	1.51E+08	+	4366	1	MTMR1|-|	9.982	11.692
NM_0124:	chr7	1.29E+08	1.29E+08	-	4387	1	TNPO3|IP(	24.394	26.86
NR_13452	chr7	1E+08	1E+08	+	597	1	LAMTOR4	81.529	57.748
NM_0147!	chr11	74949247	74979031	+	2701	1	SPCS2|-|-|	2.034	2.257
NR_10412	chr12	67989445	68021327	+	1787	1	IFNG-AS1|	0	0
NM_0013:	chr19	51689434	51705190	+	966	1	SPACA6|LI	2.336	1.999
NR_03376	chr15	92795484	92797108	+	1624	1	ASB9P1|-|	0.016	0.008
NR_00154	chrY	20465668	20519228	-	1622	1	TTTY10|N(	0.064	0.076
NM_0013:	chr15	84884661	84975645	+	2019	1	SLC28A1|(	0.057	0
NM_0321:	chrX	77826365	77895568	-	4009	1	MAGT1|IA	3.599	3.372
NR_03175	chr3	48578902	48578977	-	75	1	MIR711|h:	0.688	0.548
NR_03894	chr7	1.55E+08	1.55E+08	-	2699	1	HTR5A-AS:	0	0
NM_0013:	chr14	60144120	60169864	-	1892	1	DHRS7|CG	31.286	22.102
NM_0054:	chr2	1.1E+08	1.1E+08	-	3055	1	MALL|BEN	0.008	0.009
NM_0013:	chr17	78168545	78172928	+	1594	1	SYNGR2|-|	5.088	5.757
NM_0051!	chr9	1.31E+08	1.31E+08	+	5564	1	ABL1|ABL	47.734	53.892
NM_1784(	chr9	1.37E+08	1.37E+08	-	909	1	LCN8|EP1:	0	0
NR_02695	chr13	44022335	44030463	+	2561	1	LINC00284	0.035	0.021
NR_13073	chr13	94574054	94596275	-	1818	1	TGDS|CAT	1.319	3.097
NM_0005{	chr4	87975650	87983411	+	1575	1	SPP1|BNSI	5.755	7.705
NM_1749!	chrX	8790796	8801383	-	1506	1	FAM9A|TE	0.009	0.018

NM_0059	chr16	56657943	56659303	+	436	1	MT1F|MT:	2.188	2.419
NM_1784	chrX	1.3E+08	1.3E+08	-	1190	1	GPR119|G	0	0
NR_13662	chr10	79506370	79510982	-	1651	1	LOC72981	0	0
NM_0157	chr2	1.5E+08	1.5E+08	-	1427	1	MMADHC	57.022	87.701
NM_0010	chr17	33013088	34156806	-	2678	1	ASIC2|ACC	0.438	0.767
NM_0010	chr4	78917940	78939428	-	3731	1	PAQR3|RK	5.052	6.035
NR_02678	chr8	1.43E+08	1.43E+08	-	2012	1	RHPN1-AS	1.269	1.205
NM_0012	chr17	81890790	81900533	+	853	1	ANAPC11|	65.813	52.893
NM_0012	chr8	1.01E+08	1.01E+08	-	2669	1	ANKRD46|	25.642	28.513
NR_02675	chr1	16618256	16630906	-	1758	1	CROCCP2|	12.864	12.365
NM_0012	chr10	27812164	27981917	-	2557	1	ARMC4|CI	0.222	0.107
NM_0011	chr3	33090416	33096801	-	3452	1	TMPPE|-|-	0.59	0.52
NR_02939	chr17	16830483	16832833	-	1570	1	KRT16P2|-	0	0
NM_0176	chr21	33577553	33588708	-	2510	1	DONSON|	5.651	5.037
NR_03379	chr11	67613524	67615664	-	429	1	DOC2GP|-	0.06	0
NR_12576	chr4	4844437	4850827	+	523	1	LINC0139€	0	0.079
NM_0010	chr9	1.05E+08	1.05E+08	-	1042	1	OR13C3|C	0	0
NR_03995	chr3	50675080	50675163	+	83	1	MIR4787|	0.932	1.32
NM_0011	chr10	27154824	27187953	+	4646	1	MASTL|GF	2.265	2.532
NR_04657	chr3	1.84E+08	1.84E+08	+	466	1	ABCC5-AS:	0	0
NR_03648	chr17	72403322	72592804	-	2253	1	LINC00673	3.64	8.031
NM_0010	chr11	58190434	58191518	+	1082	1	OR9Q2|OF	0	0
NR_03392	chr1	2.35E+08	2.35E+08	+	2268	1	LINC00184	0.028	0.012
NM_0055	chr17_KI2	1173837	1180980	+	3422.5	2	LHX1|LIM-	0.388	0.456
NM_1446	chr11	4571423	4577820	-	649	1	C11orf40|	0	0
NM_0052	chr11	69809968	69819424	-	1543	1	FGF3|HBG	0	0.062
NR_11006	chr14	53153354	53157528	+	1073	1	LOC10192	0.048	0
NM_0174	chr21	25585656	25607489	-	1070	1	MRPL39|C	15.496	17.729
NM_0312	chr19	19900913	19935573	+	2788	1	ZNF93|HP	1.702	3.287
NM_0219	chr22	23741584	23751092	-	4014	1	ZNF70|Co:	3.598	4.361
NM_0011	chr1	40072710	40097470	-	2187	1	PPT1|CLN:	35.798	37.383
NR_10703	chr16	5632467	5632566	+	99	1	MIR8065|	0	0
NM_0011	chr9	1.1E+08	1.1E+08	+	6795	1	AKAP2|AK	4.378	2.213
NM_0031	chr5	1.23E+08	1.23E+08	+	6542	1	SNX2|TRG	4.09	5.249
NM_0016	chr14	49893018	49897054	+	3935	1	ARF6|-|-|	9.358	10.791
NR_00322	chr14	1.01E+08	1.01E+08	+	73	1	SNORD114	0	0
NM_0054	chr11	90134650	90192611	+	3175	1	NAALAD2|	0.666	0.764
NR_03317	chr10	46876674	46895326	+	1941	1	FRMPD2B	0.066	0.042
NM_0010	chr1	2.35E+08	2.35E+08	-	4611	1	IRF2BP2|-	18.917	31.639
NR_02747	chr9	66159959	66164906	-	980	1	LOC64292	0	0
NM_0005	chr1	2.04E+08	2.04E+08	-	1453	1	REN|HNFJ	0.018	0
NR_04983	chr17	69099542	69099656	+	114	1	MIR4524B	0	0
NM_0010	chr15	64072559	64094008	-	1039	1	FAM96A|(	43.644	34.671
NR_02989	chr8	1.41E+08	1.41E+08	-	89	1	MIR151A|	0	0
NM_0209	chr10	69572035	69573454	-	1251	1	NEUROG3	0.021	0.011
NM_1784	chr4	75556048	75565893	+	1878	1	C4orf26|A	0.185	0.197
NR_04685	chr3	1.72E+08	1.72E+08	-	424	1	TMEM212	0	0
NM_0012	chr13	1.1E+08	1.1E+08	-	1514	1	COL4A2-A:	0.153	0.145
NM_0308	chr4	1.1E+08	1.1E+08	-	5229	1	PLA2G12A	4.017	4.424
NR_10978	chr19	14583084	14611144	+	1997	1	CLEC17A|-	0	0.007

NM_0198	chr19	48029383	48044054	-	1820	1	CABP5|CA	0	0
NR_12870	chr2	2.19E+08	2.19E+08	+	64	1	MIR9500|	0	0
NR_02688	chr19	33302857	33305057	+	2200	1	CEBPA-AS	0.07	0.249
NM_0208	chr15	76348186	76883876	-	4676	1	SCAPER|M	5.162	5.056
NM_0012	chr8	70669365	70735942	+	3284	1	XKR9|XRG	0.188	0.079
NR_13236	chr5	1.49E+08	1.49E+08	-	399	1	GRPEL2-A	3.038	2.334
NR_00304	chr11	1.19E+08	1.19E+08	-	632	1	RPL23AP6	0.877	1.344
NR_00128	chr6_GL0	3256195	3260798	-	2770	6	TNXA|D6S	0	0
NR_10991	chr20	61079064	61080179	+	465	1	LINC0171	0	0
NM_0044	chr9	97853255	97856715	+	3458	1	FOXE1|FK	0	0.032
NM_0011	chr7	64045443	64078549	+	1674	1	ZNF727|Z	0	0.008
NR_03384	chr8	1.44E+08	1.44E+08	+	2733	2	FAM83H-	0	0
NM_1788	chr2	80301878	80304362	-	2208	1	LRRTM1|-	2.785	3.803
NM_0012	chr2	25377243	25652660	-	2130	1	DTNB|-|-|	15	27.24
NM_0158	chr13	75760661	75859871	+	6747	1	LMO7|FB	0.373	2.016
NR_02681	chr8	11368402	11438657	+	1215	1	FAM167A-	0.138	0.045
NM_0219	chr3	1.28E+08	1.28E+08	+	2220	1	EEFSEC|EF	16.82	20.04
NR_12156	chr9	42579805	42586534	-	3220	1	LOC10192	0	0
NM_0012	chr19	11597420	11619159	+	2847	1	ZNF627|-|	28.821	30.964
NM_0012	chr5	1.47E+08	1.47E+08	+	4907	1	STK32A|Y	2.415	4.142
NR_12592	chr4	1.04E+08	1.05E+08	+	2958	1	LOC10192	0.449	0.157
NM_0011	chr19	10602445	10644559	+	3351	1	SLC44A2|	52.074	53.558
NM_0052	chr1	84498323	84506579	-	800	1	GNG5|-|-|	45.965	42.837
NR_00306	chr17	28226563	28228059	+	1068	1	PYY2|-|-|	0.676	0.295
NR_03391	chr9	1.37E+08	1.37E+08	+	2699	1	LOC10001	0.019	0.015
NR_13431	chr8	52194362	52199552	+	794	1	LOC10192	0	0
NR_12646	chr20	25624045	25675078	+	645	1	ZNF337-A	5.717	8.155
NM_0012	chrX	1.52E+08	1.52E+08	-	1867	1	MAGEA10	0	0
NM_0024	chr11	65597755	65614249	-	3561	1	MAP3K11|	10.764	7.777
NM_0013	chr19	572454	583493	+	1727	1	BSG|5F7|	332.117	305.094
NM_0003	chr10	87863438	87971930	+	8691	1	PTEN|10q	2.024	3.22
NR_11060	chr2	1.05E+08	1.05E+08	-	639	1	LOC10272	0.222	0.407
NM_0162	chr19	12271810	12294899	-	2644	1	ZNF44|GI	2.809	2.191
NM_0012	chr21	25639282	25714573	+	1735	1	JAM2|C21	13.406	9.916
NM_0180	chr12	12329284	12350235	-	2291	1	MANSC1|	4.159	7.563
NM_0006	chrX	1.16E+08	1.16E+08	+	2894	1	AGTR2|AT	0.009	0
NM_0009	chr13	27251555	27256565	-	559	2	RPL21|HY	1.914	1.617
NM_0013	chr2	96021946	96035245	-	2764	1	GPAT2|CT	0.606	0.54
NM_0011	chr1	1.59E+08	1.59E+08	-	952	1	OR6P1|-|-	0	0
NM_0011	chr16	29871159	29899264	-	3197	1	SEZ6L2|BS	100.486	102.019
NM_0012	chr2	2.02E+08	2.02E+08	+	4119	1	KIAA2012|	0.1	0.146
NR_02432	chr1	826206	827522	-	1316	1	LINC0011	0.48	0.489
NM_0011	chr1	10946473	10982037	-	2747	1	C1orf127|	0	0.02
NM_0051	chr20	4852356	5001499	-	6925	1	SLC23A2|	14.835	20.81
NM_0012	chr20	33358839	33401569	-	1882	1	CDK5RAP1	15.819	19.279
NM_0011	chr11	63759892	63768641	-	5575	1	C11orf95|	52.82	65.176
NM_0174	chr11	1.25E+08	1.25E+08	+	930	1	SPA17|CT	19.825	13.977
NM_0070	chr13	52703266	52739812	-	1436	1	CNMD|BR	0.108	0.095
NM_0011	chr4	1.51E+08	1.52E+08	+	4560	1	FAM160A	0.074	0.048
NM_0151	chr7	17790761	17940508	-	6343	1	SNX13|RG	1.783	3.151

NR_13486	chr6	1.6E+08	1.6E+08	+	870	1	MRPL18|H	50.581	48.254
NM_0012	chr11	7597186	7653765	+	2971	1	PPFIBP2|C	1.688	3.324
NM_0223	chr1	42746335	42767084	-	2669	1	P3H1|GRC	18.758	17.125
NM_0204	chr11	67438047	67443512	-	1897	1	CORO1B|C	33.304	28.232
NR_02706	chr19	5558167	5568034	-	3730	1	TINCR|LIN	0.94	0.98
NR_10684	chr19	6392921	6392983	-	62	1	MIR6790|	0	0
NM_0047	chr20	49632946	49713884	-	4716	1	B4GALT5|	43.317	62.183
NM_0010	chr1	15807162	15807699	-	535	1	UQCRHL|-	112.74	101.614
NR_03608	chr3	20137565	20137641	+	76	1	MIR3135A	0	0
NM_0249	chr7	5475797	5513798	-	8252	1	FBXL18|Fb	5.128	5.429
NM_0052	chr9	88991409	89005154	+	4384	1	S1PR3|ED	1.732	4.405
NR_10681	chr11	67490245	67490315	+	70	1	MIR6752|	17.314	15.262
NM_0010	chr17	38835088	38841389	-	562	2	C17orf98|	0	0
NM_0035	chr12	1.32E+08	1.32E+08	+	5192	1	ULK1|ATG	21.729	22.727
NM_0038	chr13	47942656	48001326	-	2151	1	SUCLA2|A	15.003	12.258
NM_0013	chr19	43506719	43527256	-	674	1	ETHE1|HS	20.297	23.37
NM_0141	chr7	1.35E+08	1.35E+08	-	4602	1	WDR91|H	5.273	5.712
NR_03998	chr14	38749339	38916882	-	4301	1	LINC0063	0.012	0.07
NM_1825	chr10	18545207	18651637	-	2557	1	NSUN6|49	4.276	4.419
NM_0011	chr17	1270564	1271271	+	706	1	BHLHA9|B	0	0
NM_0013	chr4	1.85E+08	1.85E+08	+	889	1	C4orf47|-|	1.885	0.616
NM_1825	chr12	52168996	52192000	-	3848	1	KRT80|KB	0.027	0.007
NR_03969	chr9	1.35E+08	1.35E+08	-	71	1	MIR3689C	0	0
NM_0013	chr14	93430932	93707876	+	9075	2	UNC79|KI	0	0
NM_0190	chr2	2.34E+08	2.34E+08	+	2338	1	UGT1A5|U	0.033	0
NR_02654	chr1	75918873	75932431	-	1042	1	ASB17|Asb	0	0
NM_0184	chr2	3658196	3702670	+	1430	1	ALLC|ALC|	0.054	0.086
NM_0155	chr1	45500184	45511067	+	2844	1	MMACHC|	3.305	2.755
NM_0010	chr22	17592136	17628822	-	1330	1	ATP6V1E1	71.224	81.647
NM_0202	chr7	192969	260774	+	1597.3	3	FAM20C|D	42.91	42.292
NR_00218	chr13	50172018	50173615	+	1597	1	ST13P4|FA	0.032	0.094
NM_0010	chr7	1470277	1504382	-	6924	1	INTS1|INT	19.517	20.271
NM_0012	chr14	24070837	24078100	+	2215	1	CPNE6|-|-	0.041	0.179
NM_0001	chr4	1.86E+08	1.86E+08	+	3261	1	F11|FXI|-|	0.008	0
NM_0011	chr11	95025258	95027596	+	2336	1	KDM4E|JM	0	0
NR_03605	chr1	62078789	62078856	-	67	1	MIR3116-2	0	0
NM_0067	chr1	2.03E+08	2.03E+08	+	2707	1	BTG2|APR	22.062	25.304
NM_0002	chr12	1.03E+08	1.03E+08	-	2666	1	PAH|PH|P	0.01	0.026
NM_0011	chr17	10822475	10838101	-	3462	1	PIRT|-|-|1	0.056	0.297
NM_0207	chr20	63956704	63969870	-	5903	1	ZNF512B|	19.016	22.278
NM_0012	chr19	544034	549920	+	930	1	GZMM|LM	0.624	0.545
NR_04058	chr7	67302638	67321526	+	2121	1	STAG3L4|	5.702	5.018
NM_1334	chr3	1.81E+08	1.81E+08	+	3019	1	TTC14|DR	2.985	4.814
NM_0011	chr1	1373731	1375182	-	806	1	AURKAIP1	86.175	67.771
NM_0010	chr2	1.02E+08	1.03E+08	+	4124	1	SLC9A4|N	0	0.007
NM_0012	chr8	63168627	63212788	+	4997	1	YTHDF3|-|	9.764	10.868
NM_0004	chr1	1.74E+08	1.74E+08	-	1590	1	SERPINC1|	0.13	0.112
NM_1523	chr18	13663347	13726592	-	4238	1	FAM210A|	1.576	1.729
NM_0012	chr11	17351762	17377321	+	6375	1	NCR3LG1|	2.658	4.13
NM_0329	chr4	1.1E+08	1.1E+08	+	999	1	GAR1|NOL	4.453	6.883

NM_0012{	chr6	84124241	84227635	-	5073	1	CEP162|C(	0.534	1.08
NM_0012;	chr19	49487554	49492307	+	1095	1	RPL13A|L1	259.683	241.856
NR_10802	chr5	1.7E+08	1.7E+08	-	1329	1	LINC01187	0	0
NM_0248;	chr22	41800622	41826299	+	1318	1	CCDC134|	3.101	3.565
NM_0011	chr19	56774556	56840729	-	2158	1	ZIM2|ZNF(	0.042	0.038
NR_11057	chr2	1.45E+08	1.45E+08	+	420	1	LINC01412	0.276	0.098
NR_04608	chr3	15254184	15264498	+	5572	1	SH3BP5-AS	13.699	28.645
NM_0010{	chr17	75784839	75825805	+	3845	1	UNK|UNKI	19.06	17.908
NM_0328(	chr17	40475828	40501602	-	4057	1	TNS4|CTEI	0	0.01
NM_0011;	chr19	21020620	21060046	+	3909	1	ZNF430|-|	1.197	1.43
NR_10993	chr5	9511333	9518086	+	1676	1	SEMA5A-A	0.008	0.008
NM_0808;	chr20	45534197	45539495	-	614	1	WFDC6|C2	0	0
NR_02775	chr12	1.05E+08	1.05E+08	-	7468	1	ALDH1L2|	1.086	1.783
NM_0013	chr15	43133115	43185143	+	2180	1	TMEM62|	5.678	5.812
NR_00306	chr19_KI2	155324	160860	-	2089.4	27	LILRP2|CD	0	0
NR_12059	chr18	8695856	8707621	-	816	1	GACAT2|N	0.98	1.024
NM_0065!	chr5	1050374	1112057	-	5278	2	SLC12A7|K	0.718	0.714
NM_0061!	chr2	70087453	70089202	+	1747	1	PCBP1|HE	236.821	196.47
NM_0011!	chr2	1.09E+08	1.09E+08	+	4312	1	LIMS1|PIN	0.963	0.934
NR_10461	chr5	269556	271524	-	830	1	HRAT5|-|-	1.616	1.155
NR_12058	chr11	1.05E+08	1.05E+08	-	620	1	LOC10272	0.437	0.11
NR_12598	chr1	2.13E+08	2.13E+08	+	330	1	LINC0174C	0.039	0.083
NM_0010:	chr10	15075475	15088776	-	3364	1	ACBD7|bA	1.966	2.818
NM_0011!	chr3	1.25E+08	1.25E+08	-	3434	1	SLC12A8|(	0.447	1.081
NM_0012;	chr19	23116975	23147212	+	2361	1	ZNF730|-|	0.562	0.65
NM_0012	chr11	65653596	65662972	-	2255	1	RELA|NFKI	26.805	30.656
NR_13125	chr5	1.34E+08	1.34E+08	+	680	1	WSPAR|Ln	0.019	0.04
NM_0529:	chr11	1.02E+08	1.02E+08	-	3583	1	TMEM123	6.549	16.633
NR_02710	chr4	1.82E+08	1.82E+08	-	5295	1	LOC90768	3.307	2.419
NM_0010(	chr11	59364459	59365394	+	934	1	OR5AN1|C	0	0
NR_10373	chr2	1E+08	1E+08	+	3112	1	LINC01104	0.017	0.075
NR_11078	chr18	22723491	22786837	-	345	1	LOC10192	0	0.318
NM_0012	chr16	56781792	56844949	+	2904	1	NUP93|NI	107.406	123.801
NR_02336	chr1	2.29E+08	2.29E+08	-	120	10	RNA5S5|R	0	0
NM_0062(	chr12	14973022	14981866	+	742	1	PDE6H|AC	0.156	0
NM_0008{	chr2	1.81E+08	1.82E+08	+	7233	1	ITGA4|CD	0.549	0.116
NM_0011!	chr17	16030094	16194639	-	9239	1	NCOR1|N-	15.523	14.837
NM_0150;	chr8	21919669	22006585	+	4833	1	XPO7|EXP	27.348	35.893
NM_0011:	chr20	3927309	4015591	-	7355	1	RNF24|G1	16.703	22.817
NM_0314{	chr5	81972025	82255396	+	2280	1	ATG10|AP	3.546	4.217
NM_0072:	chr20	33702744	33720330	-	5718	1	PXMP4|PN	0.169	0.232
NM_0010!	chr12	9993061	9999300	-	866	1	CLEC1B|1{	0	0
NR_00143	chr6_GL0C	1144025	1147513	+	1685	8	HLA-H|HL/	0	0
NM_1781	chr5	31639410	32110932	+	11678	1	PDZD2|AII	0.269	0.472
NR_00283	chr1	89993265	89994966	-	1701	1	GEMIN8P4	2.645	2.561
NR_13563	chr12	14942015	14948896	-	941	1	ARHGDIB|	7.975	1.339
NM_0333!	chr11	57299629	57324939	-	5806	1	TNKS1BP1	37.066	38.796
NM_0012(	chr12	48814432	48828943	+	2561	1	CACNB3|C	80.588	71.02
NR_00359	chr7	48924561	48927453	+	2892	1	CDC14C|C	0	0.052
NM_0010(	chr11	4998983	4999930	+	946	1	OR51L1|O	0	0

NM_0039!	chr10	89205937	89207314	-	1375	1	CH25H|C2	0.075	0.01
NR_02686	chr5	1.51E+08	1.51E+08	-	2415	1	ZNF300P1	0.064	0.244
NM_0321(	chr3	1.33E+08	1.33E+08	-	3534	1	ACAD11|A	6.801	7.341
NM_0190:	chr17	40697991	40703750	-	1871	1	KRT24|K2	0	0
NM_0012!	chrX	1.15E+08	1.15E+08	+	4648	1	HTR2C|5-H	0.291	0.035
NM_0018:	chr20	5911328	5925359	+	2643	1	CHGB|SCG	40.242	44.143
NM_0216:	chr6	1.38E+08	1.38E+08	-	2502	1	PBOV1|UC	0.005	0.005
NM_0010:	chr2	27325849	27357034	-	3861	1	GTF3C2|TI	23.065	25.585
NM_0048(	chr2	1.28E+08	1.28E+08	-	3948	1	HS6ST1|HI	35.013	38.836
NM_0010:	chr1	26321859	26354130	-	5223	1	AIM1L|CR	0.17	0.115
NM_0010;	chr8	1.45E+08	1.45E+08	-	1765	1	LRRC24|LF	13.682	12.874
NR_02724	chr22	46006616	46010777	+	4161	1	LOC73066	0.012	0.013
NM_1989	chr20	44496221	44522116	-	1747	1	SERINC3|A	31.197	35.579
NM_0011!	chr19	55613593	55618541	+	4946	1	ZNF865|-|	7.216	7.425
NR_10690	chr8	27433370	27433434	+	64	1	MIR6842|I	0.604	0.214
NM_1451:	chr7	99546300	99552134	-	2484	1	FAM200A|	3.556	4.367
NM_0206	chr17	79794377	79797116	-	1539	1	CBX8|PC3	17.979	25.062
NR_02692	chr7	56496223	56497284	+	1061	1	DKFZp434I	0	0
NM_0243:	chr22	37686337	37693478	+	2832	1	NOL12|Nc	8.495	8.648
NM_0011:	chr7	1.24E+08	1.24E+08	-	2551	1	TMEM229	0.091	0.188
NR_12583	chr6	46276324	46365505	+	3804	1	LOC10192	0	0.014
NM_0012;	chr2	1.87E+08	1.87E+08	-	5974	1	CALCRL|C	0.861	0.094
NR_02735	chr1	1.43E+08	1.43E+08	-	556	3	LOC64516	0.093	0.246
NM_2034!	chr1	1.46E+08	1.46E+08	-	5317	1	NOTCH2NI	0.015	0.041
NM_0010(	chr21	42893268	42909663	+	1030	1	NDUFV3|C	31.145	21.197
NM_0330	chr19	49043845	49045311	+	875	1	CGB5|CGB	0	0.016
NM_0012;	chr2	1.13E+08	1.13E+08	+	1101	1	IL36G|IL-1	0	0.025
NR_03378	chr15	84166547	84170984	-	4441.5	2	DNM1P41	0	0.003
NR_03402	chr13	19345049	19346749	+	1052	1	LINC00421	0	0
NR_12371	chr4	1289851	1340148	+	2026	1	MAEA|EM	44.045	40.273
NM_0013(	chr12	1.24E+08	1.24E+08	-	1837.5	2	CCDC92|-|	0.281	0.425
NR_00219	chrY	25455311	25460175	+	1053	2	GOLGA2P3	0	0
NM_0012	chr15	80098416	80138393	+	1483	1	ZFAND6|A	45.479	50.946
NM_0528(	chr4	1.12E+08	1.12E+08	-	3069	1	TIFA|T2BP	1.823	4.758
NM_1720	chr17	7549058	7561608	+	1851	1	TNFSF12-T	1.038	1.547
NR_04624	chr8	39563112	39580134	+	515	2	LOC10013	0	0
NM_0011	chr4	90127533	91602219	+	6192	1	CCSER1|F	1.099	2.477
NM_0024	chr1	75796871	75913238	+	3255	1	MSH4|-|-|	0.04	0.059
NM_0013;	chr3	1.22E+08	1.22E+08	+	883	1	EAF2|BMC	1.635	1.427
NM_2073!	chr1	89131751	89176040	-	2441	1	GBP7|GBP	0.042	0.062
NM_0010	chr1	2.13E+08	2.13E+08	+	2576	1	TATDN3|-	7.723	6.726
NM_1825!	chr12	50842918	50887880	+	1454	1	TMPRSS12	0	0
NM_0012	chr15	66505083	66549484	+	3120	1	ZWILCH|K	9.959	6.51
NR_11074	chr19	56066684	56078800	+	1656	1	LINC01864	0.389	0.422
NM_0143(	chr12	1.19E+08	1.19E+08	+	1997	1	HSPB8|CN	1.24	0.995
NR_13553	chr3	17742944	17745662	+	468	1	LOC10537	0	0
NR_10702	chr8	6622124	6622220	-	96	1	MIR8055|I	0	0
NR_10377	chr2	1.6E+08	1.6E+08	-	502	1	LOC10050	0.077	0
NR_00324	chr1	1.2E+08	1.2E+08	-	1905	1	PFN1P2|C	0.054	0.108
NR_10674	chr7	1.17E+08	1.17E+08	+	108	1	MIR6132|I	4.417	5.2

NM_00128	chr6	43771985	43786486	+	2542	1	VEGFA|MV	28.561	13.681
NR_02742	chr22_KI2	195000	202256	+	3105	2	LL22NC03-	0	0
NR_10696	chr11	68032864	68032922	+	58	1	MIR7113|I	26.009	13.461
NM_0010	chr10	14819252	14837984	-	1324	1	CDNF|ARN	0.779	0.341
NR_03779	chr19	40778219	40808441	+	2837	1	RAB4B-EGI	42.534	34.438
NM_0011	chr16	4510676	4538470	-	2693.5	2	CDIP1|C16	0.172	0.147
NR_00295	chr1	2.35E+08	2.35E+08	-	134	1	SNORA14E	0	0.409
NR_07349	chr14	64227033	64338550	-	2584	1	ESR2|ER-B	0.269	0.429
NM_0061	chr19	48900050	48923283	+	2564	1	NUCB1|CA	62.622	57.229
NM_0031	chr17	47896150	47928957	+	3103	1	SP2|-|-|17	8.73	8.268
NM_0017	chr5	79111781	79132290	+	2490	1	BHMT|BHI	0.129	0.209
NR_00361	chr7	75343937	75358997	-	634	1	PMS2P2|P	0	0
NR_13563	chr17	15014805	15052275	+	973	1	LOC10192	0	0
NR_03609	chr6	57390132	57390212	+	80	1	MIR548U|	0	0
NM_0036	chr17	9910609	9959449	-	7761	1	GAS7|MLL	3.442	4.747
NM_0058	chr5	1.39E+08	1.39E+08	-	2332	1	SLC23A1|S	0.249	0.37
NM_0012	chr12	1.18E+08	1.18E+08	-	2772	1	WSB2|SBA	85.887	105.48
NR_03887	chr8	57261226	57266611	+	2966	2	LOC28617	0	0
NM_0178	chr3	39383324	39397328	+	2115	1	SLC25A38|	19.374	21.986
NM_0146	chr2	53864067	53970840	-	7050	1	PSME4|PA	6.011	8.937
NM_0326	chr3	1.07E+08	1.07E+08	+	1291	1	CCDC54|N	0	0.138
NM_0010	chr11	4094685	4138925	+	3228	1	RRM1|R1|	28.044	25.591
NM_0153	chr3	1.34E+08	1.34E+08	-	1203	1	ANAPC13|	62.399	60.037
NM_0010	chr9	93318199	93346414	-	1147	1	C9orf129|	3.496	3.248
NM_0123	chr7	1.44E+08	1.44E+08	+	1086	1	OR2F1|7M	0	0.025
NR_13517	chr16	32152112	32188113	-	4386.5	2	LOC10272	0	0.003
NM_0328	chr12	53064316	53079420	-	2919	1	SPRYD3|-|	18.843	19.9
NR_02462	chr14	89576216	89577476	+	1260	1	FOXN3-AS	0	0
NM_0012	chr8	7539628	7542450	+	1581	3	PRR23D2|	0	0
NM_0053	chr8	1.45E+08	1.45E+08	+	1873	1	GPT|AAT1	0.564	0.746
NR_02961	chr1	1.99E+08	1.99E+08	-	109	1	MIR181B1	0	0
NM_0043	chr1	1.6E+08	1.6E+08	-	5604	1	COPA|AILJ	65.905	73.571
NM_0018	chr21	46098119	46132849	+	3409	1	COL6A2|B	25.851	11.218
NR_00437	chr2	86135870	86136006	+	136	1	SNORD94|	0.664	1.611
NM_0223	chr10	21756548	22003721	-	2077	1	DNAJC1|D	12.248	5.863
NM_0002	chr2	48686774	48755741	-	3080	1	LHCGR|HH	0.008	0.004
NM_0011	chr19	36888124	36916021	-	4969	1	ZNF829|-|	0.579	0.595
NM_0031	chr17	58345175	58352238	-	1521	1	SUPT4H1|	61.924	58.994
NM_0010	chr18	31759696	31760880	-	1182	1	SLC25A52|	0.011	0
NR_12596	chr1	1.73E+08	1.73E+08	-	1817	1	LOC10192	0.412	0.739
NR_04548	chr4	89836401	89841991	+	817	1	SNCA-AS1	0.016	0.05
NM_0020	chr2	1.62E+08	1.62E+08	-	1286	1	GCG|GLP1	0.12	0.16
NM_0010	chr12	68851176	68933199	-	6625	1	CPM|-|-|1	0.053	0.035
NM_1527	chr13	52132643	52159535	-	2397	1	NEK3|HSP	3.943	3.743
NM_0146	chr6	1.33E+08	1.33E+08	-	941	1	TAAR2|GP	0	0
NM_1817	chr7	1.07E+08	1.08E+08	-	4794	1	COG5|CDC	6.64	7.877
NM_0147	chr8	58805418	59119208	-	4120	1	TOX|TOX1	12.596	17.224
NM_0012	chr2	94871433	94876829	+	2210	1	TEKT4|-|-|	0	0
NM_0013	chr14	59472688	59483575	-	913	1	L3HYPDH|	1.836	1.815
NR_10807	chr4	1.45E+08	1.46E+08	-	1121	1	SMAD1-AS	0	0

NR_02427	chr3	1.08E+08	1.08E+08	-	1181	1	LINC00635	0.022	0.035
NR_02985	chr17	31575411	31575521	+	110	1	MIR365B|	0	0
NM_0059ɛ	chr13	1.12E+08	1.12E+08	+	4105	1	SOX1|-|-|:	3.973	10.624
NM_0011(	chr18	47022287	47023927	-	1639	3	ELOA3B|El	0	0
NM_0011!	chr1	1.56E+08	1.56E+08	+	992	1	PMF1-BGL	29.881	26.428
NM_0133:	chr22	21697537	21735834	-	4329	1	YPEL1|FKS	27.133	28.308
NM_0012!	chr1	85018082	85048540	-	2668	1	MCOLN3|‾	0	0.015
NM_0011⁄	chr11	57743514	57751781	-	3043	1	BTBD18|-|	0.119	0.171
NM_0010(	chr2	30447226	30644225	+	4891	1	LCLAT1|1A	1.858	2.568
NM_0312ɛ	chr2	74455023	74457960	+	1254	1	INO80B|H	22.199	21.43
NR_04768	chr13	1.01E+08	1.01E+08	+	2838	1	NALCN-AS	0.482	0.42
NM_0011⁄	chr5	1.77E+08	1.77E+08	-	2654	1	MXD3|BHl	11.101	10.244
NM_0013(	chr6	75749176	75919537	+	8574	1	MYO6|DFl	6.212	8.588
NM_0204:	chr22	26429314	26445012	+	3307	1	ASPHD2|-	12.691	18.025
NR_03612	chr11	66934434	66934506	-	72	1	MIR3163|·	0	0
NR_04983	chr10	42997563	42997681	+	118	1	MIR5100|·	0	0
NR_10273	chr14	85393879	85420076	+	545	1	LINC00911	0	0
NM_0528!	chr3	53088485	53130454	-	5083	1	RFT1|CDG	5.395	5.616
NR_10703	chr4	1.01E+08	1.01E+08	-	77	1	MIR8066|l	0	0
NM_0247(	chr19	57807082	57814913	-	2347	1	ZNF552|-|	2.197	2.317
NM_0012;	chr3	1.3E+08	1.3E+08	+	9082	1	COL6A5|C	0	0.009
NR_13012	chr12	49064676	49070025	-	1144	1	RHEBL1|Rl	2.31	1.784
NM_0013;	chr12	1.12E+08	1.12E+08	-	892	1	RPL6|L6|S	184.31	164.748
NM_0011!	chr12	220621	262873	-	1935	1	SLC6A13|(	0.153	0.042
NM_0005;	chr5	63960048	63962292	-	2242	1	HTR1A|5-l	0.035	0.098
NR_11195	chr1	1.5E+08	1.5E+08	+	1611	1	LINC0086¢	1.569	1.037
NM_0010(	chrY	2841582	2866956	+	888	1	RPS4Y1|Rl	305.334	321.448
NM_1984:	chr11	1.06E+08	1.06E+08	-	4225	1	KBTBD3|B	0.922	0.836
NR_10413	chr13	80011077	80026663	+	620	1	LINC0108C	0.125	0
NM_0012;	chr1	6579991	6589280	+	2250	1	ZBTB48|Hl	13.759	14.245
NR_10806	chr13	54124324	54132871	-	1035	1	LINC0045ɛ	0.05	0.026
NR_03168	chr15	85770496	85770578	-	82	1	MIR1276|l	0	0.167
NR_13089	chr1	36322030	36323648	-	884	1	EVA1B|C1(	3.53	1.859
NM_0018ɛ	chr5	76952855	76969474	+	1829	1	CRHBP|CR	0.634	0.427
NR_10989	chr10	97446131	97457370	+	1335	1	ZDHHC16|	32.866	25.947
NR_03977	chr5	1.75E+08	1.75E+08	+	53	1	MIR4634|·	0	0
NR_13554	chr2	88627829	88631825	+	1328	1	LOC10192:	1.388	1.083
NR_05202	chr1	1.54E+08	1.54E+08	+	988	1	SNAPIN|Bl	58.999	61.456
NR_10673	chr4	1.71E+08	1.71E+08	+	108	1	MIR6082|l	0	0
NM_0042:	chr19	10221433	10231272	-	3580	1	S1PR2|AG	4.102	3.057
NM_0329:	chr7	77793728	77798430	-	869	1	TMEM60|(	15.594	15.998
NM_0146;	chr2	1.76E+08	1.76E+08	+	1292	1	HOXD4|Hl	0	0.021
NM_0012(	chr4	1.4E+08	1.4E+08	+	723	1	MGST2|G!	5.546	3.258
NM_0013;	chr11	16787660	17014416	-	4786	1	PLEKHA7|·	4.712	2.67
NM_0010;	chr14	39147811	39170430	-	3343	1	TRAPPC6B	7.926	7.609
NM_0207(	chr19	46491191	46495912	-	4719	1	PNMAL2|-	10.429	13.018
NR_04540	chr1	24496254	24502382	-	434	1	RCAN3AS|	0	0.063
NR_02697	chr12	9065177	9068055	+	2184	1	A2M-AS1|	0	0.075
NR_02988	chr16	67202321	67202395	-	74	1	MIR328|M	0.348	1.851
NM_0012ɛ	chr7	1.51E+08	1.51E+08	+	2750	1	AGAP3|AG	56.83	70.801

NR_12870	chr3	60617805	60617870	-	65	1	MIR548BB	0	0
NM_0249!	chr12	1.12E+08	1.12E+08	-	5793	1	NAA25|C1	3.679	4.079
NR_03625	chr9	93819357	93819421	+	64	1	MIR4291|-	0	0
NM_1445(	chr19	2732525	2740076	-	1420	1	SLC39A3|Z	25.397	24.568
NM_1389!	chr16	76309838	76560750	+	5809	1	CNTNAP4|	1.057	2.806
NR_11190	chr5	67001618	67004154	-	741	1	MAST4-AS	0.452	0.444
NR_03165	chr10	68759318	68759414	+	96	1	MIR1254-1	0.269	0.713
NR_03967	chr6	1.32E+08	1.32E+08	-	71	1	MIR548AJ1	0	0
NM_0208(	chr1	2.32E+08	2.33E+08	-	6576	1	SIPA1L2|S	36.683	30.772
NM_0011!	chr17	50095695	50111369	+	2481	1	PDK2|PDH	25.979	31.452
NM_0045:	chr4	1.47E+08	1.47E+08	+	3137	1	POU4F2|B	1.381	1.288
NM_0024:	chr22	23894378	23895222	+	556	2	MIF|GIF|G	0	0
NM_0221:	chr3	1.2E+08	1.2E+08	-	1680	1	POPDC2|P	1.627	2.315
NR_01545	chr17	81927829	81930753	+	1893	1	MAFG-AS1	7.084	5.629
NM_0148:	chr12	752923	911452	+	9680	1	WNK1|HSA	16.069	17.728
NR_00293	chr6	28215338	28218929	-	3591	1	TOB2P1|T	0.266	0.137
NR_02850	chr2	2.32E+08	2.32E+08	-	1294	1	ECEL1P2|E	0	0
NM_0011!	chr17	20579724	20580911	+	1044	1	CDRT15L2	0	0
NM_0046!	chr12	52424070	52434326	-	2114	1	KRT75|K6I	0	0
NR_10702	chr19	54645309	54645383	+	74	5	MIR8061|I	0	0
NM_2016:	chr1	14598717	15118048	+	6005	1	KAZN|KAZ	15.872	19.589
NM_0067:	chr12	54579240	54588659	-	1825	1	PPP1R1A|	64.538	79.443
NR_11192	chr22_KI2	62553	66894	-	1645	7	LOC10192!	0.18	0.241
NM_0010:	chrX	77967961	77969638	-	1676	1	PGAM4|PG	0.108	0.09
NM_1452:	chr10	93667883	93702572	-	3074	1	FRA10AC1	3.704	3.672
NR_13520	chr2	27705786	27715732	-	2858	1	LINC0146C	0.005	0.01
NR_02693	chr3	1.02E+08	1.02E+08	+	2047	1	LINC02085	0	0
NM_0132(	chr11	65096115	65111860	+	2746	1	VPS51|AN	37.798	45.431
NR_03833	chr10	97102756	97103747	+	686	1	SLIT1-AS1|	0.056	0
NM_1758!	chr1	32179744	32198285	+	4783	1	TXLNA|IL1	22.091	18.72
NM_0251:	chr15	44562696	44663678	-	7746	1	SPG11|ALS	7.654	6.357
NM_0012(	chr5	1.42E+08	1.42E+08	+	4163	1	RNF14|AR	13.197	12.917
NM_0248!	chr14	50242434	50312229	-	6083	1	L2HGDH|C	1.145	1.09
NM_0013:	chr4	958884	973560	-	4618	1	DGKQ|DA	8.303	9.717
NM_0167:	chr22	31248362	31280080	+	3831	1	LIMK2|-|-	29.91	27.887
NM_0012:	chr3	52410508	52423641	+	2150	1	PHF7|HSP	3.076	3.02
NR_10807	chr7	7255154	7278069	+	757	1	LOC10192	0.051	0.181
NM_0321(	chr5	77628712	77638697	-	2673	1	OTP|-|-|5	0.275	1.158
NM_0011:	chr3	1.43E+08	1.43E+08	+	3848	1	PLS1|-|-|3	0.818	0.819
NR_03992	chrX	7147860	7147937	+	77	1	MIR4767|	0	0
NR_04988	chr1	9967381	9967458	+	77	1	MIR5697|-	0	0
NM_0011:	chr12	47661932	47706061	-	3567	1	RPAP3|-|-	3.593	3.825
NM_0045!	chr11	47579010	47584563	+	950	1	NDUFS3|C	107.206	87.502
NM_0035:	chr13	98450199	98522125	-	4566	1	STK24|HEI	24.759	23.224
NM_0063:	chr3	33113958	33147773	+	6642	1	CRTAP|CA	8.838	7.234
NR_02704	chr14	71487861	71489703	+	1842	1	LOC14547	0	0.007
NR_04512	chr6	25732433	25732861	+	428	1	HIST1H2AI	0	0
NR_02461	chrX	47204848	47205855	+	1007	1	INE1|NCRI	1.869	2.19
NM_0154!	chr7	32868166	32891856	-	3704	1	KBTBD2|B	11.031	15.657
NM_0157:	chr19	9959561	10010471	-	6105	1	COL5A3|-|	0.703	0.42

NM_0012!	chr5	94705537	95081866	-	4400	1	MCTP1|-|-	1.295	0.977
NR_00366	chr7	72862757	72869091	-	1058	1	SPDYE7P|-	0	0
NR_03844	chr12	1.14E+08	1.14E+08	+	3635	1	TBX5-AS1|	0	0
NR_04981	chr3	1.52E+08	1.52E+08	-	119	1	MIR5186|-	0	0
NM_0011(	chr15	26867126	26949210	+	2751	1	GABRA5|-	0.633	2.405
NM_0012!	chr17	43211827	43220037	+	2992	1	TMEM106	0.19	0.119
NM_0189(	chr21	42499632	42581440	+	3071	1	SLC37A1|(	16.072	14.495
NM_1536!	chr9	96385943	96418387	-	3695	1	ZNF367|AI	0.558	0.76
NR_10373	chr17	72071042	72116016	-	3026	1	SOX9-AS1|	0.507	0.874
NR_00318	chr7	73220639	73235945	+	1508	1	NCF1B|NC	0	0
NM_1736:	chr17	82418376	82442640	+	2208	1	HEXDC|-|-	11.276	11.631
NR_07338	chr15	99257525	99260276	+	2751	1	HSP90B2P	3.998	3.336
NR_12598	chr1	43709405	43727343	-	452	1	LOC10192!	0	0.242
NM_0043(	chr8	1.24E+08	1.24E+08	-	1476	1	ANXA13|A	0.035	0.186
NR_13464	chr18	6728927	6729862	-	383	1	LOC10192	0.976	0.644
NM_0010	chr3	63652668	63665215	+	1578	1	SNTN|S10	0	0
NM_0010	chr15	34229996	34338064	-	7335	1	SLC12A6|/	7.682	10.422
NM_0209	chr10	48685473	48982956	+	9949	1	WDFY4|C1	0	0.003
NR_10678	chrX	1.46E+08	1.46E+08	-	76	1	MIR892C|	0	0
NM_0011	chr15	74782084	74803198	+	2205	1	CSK|-|-|15	42.253	51.67
NM_0211	chr1	2.38E+08	2.38E+08	-	1941	1	ZP4|ZBP|Z	0.013	0.014
NR_13562	chr19	41928820	41930136	+	647	1	LOC10050	0.239	0.402
NR_03843	chr13	44799504	44809630	-	2032	1	LINC0033C	0	0
NM_0010	chr3	46020799	46027487	-	1247	1	XCR1|CCX	0.083	0
NM_0011(	chr17	8120590	8124092	-	1676	1	HES7|SCD	2.139	1.921
NM_1788	chr7	1.43E+08	1.43E+08	+	764	2	C7orf34|c	0	0
NM_1768	chr19	19201411	19203429	-	1296	1	NR2C2AP|	16.584	17.29
NM_0162(	chrX	1.37E+08	1.37E+08	+	1208	1	VGLL1|TD	0	0
NM_0011	chr17	49210227	49223225	+	2081	1	ABI3|NESH	0	0
NR_03840	chrX	1.39E+08	1.39E+08	+	830	1	FGF13-AS1	0.186	0.05
NM_0012	chr18	76495521	76498088	+	2072	1	C18orf65|	0.759	0.754
NR_03626	chr15	34382069	34382150	-	81	2	MIR1233-2	0	0
NM_1522	chr14	55272154	55353083	+	2827	1	FBXO34|C	11.895	14.38
NR_03340	chr9	87844745	87847424	+	566	1	CTSLP8|CT	0	0.024
NM_0009!	chr11	8682448	8689872	+	4872	1	RPL27A|L2	46.286	42.539
NR_10980	chrX	70452956	70455994	-	399	1	DLG3-AS1|	0.355	0.24
NM_0012!	chr17	2059839	2236847	-	3228	1	SMG6|C17	23.726	24.22
NR_12638	chr13	85065087	85148057	-	625	1	LINC00375	0	0.022
NM_1758	chr7	29563811	29567295	+	1674	1	PRR15|-|-	0.023	0.098
NM_0012(	chr19	57849813	57865123	+	6929	1	ZNF587|ZI	3.787	4.327
NM_0070	chr13	94709625	94712543	-	2916	1	SOX21|SO	5.244	15.242
NM_0011!	chr9	33750821	33799231	+	780	1	PRSS3|MT	0.116	0.193
NM_1738	chr8	27869882	27992852	-	4140	1	SCARA5|N	0.062	0
NM_0010	chr1	42534889	42654664	+	3080	1	CCDC30|P	1.151	0.654
NM_0012	chr16	3382081	3401065	-	2782	1	ZSCAN32|	10.275	12.058
NR_11041	chr21	35954679	36004667	-	805	1	LOC10192	0	0
NM_0011(	chr13	1.14E+08	1.14E+08	+	3929	1	CHAMP1|(	13.396	13.868
NM_0144	chr5	1.49E+08	1.49E+08	-	690	1	IL17B|IL-1	0.075	0.258
NM_0012	chr12	1.11E+08	1.11E+08	+	1706	1	CCDC63|C	0.151	0.032
NM_0010(	chr11	56417258	56418232	-	973	1	OR5R1|OF	0	0

NR_11005	chr12	1.26E+08	1.26E+08	+	635	1	LOC10192	0	0
NR_13367	chr21	6532207	6532776	-	420	2	FRGCA|-|-	0	0
NR_03388	chr8	1.17E+08	1.17E+08	+	2008	1	RAD21-AS	0.231	0.13
NR_11088	chr17	2232678	2233610	+	710	1	LOC10192	0	0
NM_0011	chr2	2.33E+08	2.33E+08	-	2797	1	NGEF|ARH	3.909	2.904
NM_0013	chr1	1.5E+08	1.5E+08	+	2061	1	PLEKHO1|	59.607	56.948
NM_0320	chr1	61681047	61725423	-	1241	1	TM2D1|BE	13.943	12.008
NM_0016	chr19	44914555	44919349	+	564	1	APOC1|Ap	0.823	0.121
NM_0227	chr12	1.23E+08	1.23E+08	+	966	1	SNRNP35|	35.664	26.331
NR_10998	chrX	1.55E+08	1.55E+08	-	1698	2	LOC10192	0	0
NM_0011	chr18	74315875	74359187	+	5049	1	C18orf63|	0	0
NR_02969	chr11	43581394	43581483	+	89	1	MIR129-2|	0	0
NM_0530	chr4	8269765	8307111	+	2530	1	HTRA3|Prs	0.082	0.027
NM_0806	chr11	1.12E+08	1.12E+08	+	1134	1	C11orf52|	0.057	0.048
NM_0012	chr6	1.5E+08	1.5E+08	-	2712	1	LATS1|WA	4.559	5.924
NM_0012	chr1	2E+08	2E+08	+	4768	1	NR5A2|B1	0.014	0.026
NR_00259	chr5	1.81E+08	1.81E+08	-	62	1	SNORD95|	7.487	7.511
NM_0013	chr18	41955198	42081482	+	2888	1	PIK3C3|VP	8.371	9.082
NM_0064	chr14	1.05E+08	1.05E+08	+	765	1	SIVA1|CD2	33.237	25.353
NR_03658	chr19	42821863	42826878	+	1869	1	PSG8-AS1|	0	0
NM_0013	chr10	37949572	37976655	-	4097	1	ZNF25|KO	7.04	10.859
NR_02676	chr5	1.36E+08	1.36E+08	-	1807	1	SMAD5-AS	0.15	0.22
NM_0229	chr5	1.77E+08	1.77E+08	+	2790	1	FGFR4|CD	1.617	2.332
NM_0013	chr16	21158377	21180616	+	2007	1	TMEM159	9.212	6.75
NM_0153	chr12	76764074	76853694	+	4752	1	ZDHHC17|	6.905	8.65
NM_0532	chr8	11421464	11466767	-	4088	1	FAM167A|	13.042	6.359
NM_0154	chr11	1.06E+08	1.06E+08	+	2853	1	AASDHPPT	28.779	28.196
NM_1532	chr7	1.51E+08	1.51E+08	+	1225	1	GIMAP7|I/	0	0
NM_0011	chr17	49206234	49209400	-	907	1	GNGT2|G-	0.014	0.015
NM_0010	chr11	49952391	49953419	+	1026	1	OR4C13|-|	0	0
NM_0013	chr16_KI2	17252	19833	+	1405.3	6	TP53TG3F	0	0
NM_0010	chr11	68754620	68841931	-	2616	1	CPT1A|CP	0.601	1.351
NM_1457	chr2	2.33E+08	2.33E+08	-	2618	1	TIGD1|EEY	5.806	8.057
NM_0178	chr11	67303448	67312607	+	2862	1	SSH3|SSH3	1.14	1.378
NM_1458	chr16	21233695	21252429	+	2902	1	ANKS4B|H	0	0.052
NR_03626	chr16_KI2	849671	849747	-	76	4	MIR1972-2	0	0
NR_13273	chr1	9182204	9192085	+	978	1	LINC0175	1.78	0.42
NR_03891	chr22	32376664	32384342	+	1554	1	LOC33966	0.465	0.511
NR_03029	chr3	1.12E+08	1.12E+08	+	97	1	MIR567|M	0	0
NM_0011	chrX	86262968	86832602	+	1789	1	DACH2|-|-	8.483	5.505
NM_0010	chr2	1.74E+08	1.74E+08	-	6001	1	SP3|SPR2|	7.638	10.253
NM_1985	chr19	7497559	7508450	+	1668	1	C19orf45|	0.626	0.723
NR_12064	chr10	65571425	65766541	+	673	1	LINC01515	0.172	0.183
NR_02841	chr19	44926803	44931386	+	627	1	APOC1P1|	0	0
NM_0010	chr7	1.51E+08	1.51E+08	+	2429	1	AOC1|ABP	0	0
NM_0011	chr4	89724099	89838296	-	3014	1	SNCA|NAC	35.707	26.544
NR_02963	chr1	1167104	1167198	+	94	1	MIR200B|	0	0
NM_0245	chr1	1.68E+08	1.68E+08	+	4900	1	MPZL1|MI	43.233	49.094
NR_13491	chr10	1.32E+08	1.32E+08	-	1863	1	STK32C|PK	13.883	16.403
NM_0252	chr22	43923739	43947568	+	2794	1	PNPLA3|A	5.939	9.604

NM_0010(	chr11	1.24E+08	1.24E+08	+	934	1	OR10G4|C	0	0
NM_1785!	chr4	2059512	2069089	+	5866	1	NAT8L|CM	10.669	14.096
NM_0322	chr20	41402103	41618494	-	10777	1	CHD6|CHD	10.318	13.167
NR_03996	chr4	2250077	2250156	-	79	1	MIR4800|	52.553	53.921
NM_0012!	chr20	3753499	3767805	-	1305	1	C20orf27|	39.659	26.114
NR_11191	chr6	89638532	89689481	+	5145	1	LOC10192!	1	0.964
NR_13431	chr10	91033166	91041255	+	535	1	LOC10537	0	0
NM_0011	chr12	57782745	57802856	+	922	1	TSFM|EFT	17.494	17.53
NR_11038	chrX	1.16E+08	1.16E+08	-	1753	1	PLS3-AS1|	0.051	0.031
NM_0011	chr1	2.26E+08	2.26E+08	-	2187	1	LEFTY2|EB	0.006	0.013
NR_12575	chr22	23765834	23767972	-	643	2	CHCHD10|	0	0
NR_03826	chr12	57931449	57936164	-	553.5	2	LOC10050	27.208	18.535
NR_04645	chr18	42186668	42533352	+	930	1	LINC00907	0.79	0.059
NR_11014	chr3	1.27E+08	1.27E+08	-	922	1	LINC0201€	0.182	0.149
NR_10808	chr11	65789001	65790868	-	542	1	OVOL1-AS	0.119	0.025
NM_0011	chr5	1.57E+08	1.57E+08	-	1275	1	TIMD4|SM	0	0
NM_0039!	chr9	35792409	35809731	+	3406	1	NPR2|AMI	1.121	0.961
NR_03413	chr2	4628218	4656222	-	2669	1	LINC0124!	0	0.026
NM_0010(	chr11	5488685	5489748	+	1061	1	OR52D1|C	0	0
NM_1748!	chr17	74521174	74531474	-	2288	1	CD300LB|	0	0
NM_0139!	chr8	32646732	32743252	+	2356	1	NRG1|ARI	14.782	9.732
NM_0056!	chr15	74843730	74873379	-	2456	1	SCAMP2|-	11.634	12.721
NM_0212	chr20	38033546	38092364	+	3863	1	RPRD1B|C	11.682	11.719
NR_03016	chr7	93483936	93484019	-	83	1	MIR489|M	0	0
NR_11055	chr5	1.12E+08	1.12E+08	+	707	1	LOC10192	0.128	0.019
NM_0022	chr12	5043919	5046788	+	2867	1	KCNA5|AT	0.409	0.626
NM_0069	chrX	46836912	46882356	+	3822	1	RP2|DELX	3.96	2.401
NM_0031	chr11	57325985	57335877	-	2800	1	SSRP1|FA(	129.607	120.778
NM_0013	chr19	57435325	57447097	+	4146	1	ZNF749|-|	2.553	2.22
NM_0051!	chr3	1.2E+08	1.2E+08	-	2748	1	CD80|B7|	0	0.03
NR_11100	chr1	1.44E+08	1.44E+08	-	2209	2	LOC10106	0	0
NM_0329	chr9	1.37E+08	1.37E+08	+	851	1	TMEM141	30.029	23.145
NM_0030	chr20	10218798	10307420	+	2077	1	SNAP25|C	51.953	65.63
NM_0051	chr12	9852369	9869859	-	2031	1	CLEC2B|AI	0	0
NR_03884	chr1	2.19E+08	2.19E+08	-	1624	1	LYPLAL1-A	0.127	0.253
NR_03836	chr19	36850886	36893218	+	556	1	ZNF345|H	1.739	1.749
NM_1538	chr2	1.01E+08	1.01E+08	-	3482	1	CREG2|-|-	0.385	0.614
NM_0001!	chr16	67431133	67437551	+	1875	1	HSD11B2|	6.423	2.206
NR_02968	chr17	58331232	58331318	-	86	1	MIR142|M	0	0
NM_0011	chr11	10852704	10858073	-	2704	1	ZBED5|Bu	7.796	12.314
NM_0011	chr13	36844831	36920272	-	5583	1	SMAD9|M	2.859	1.953
NM_0314!	chr10	12896625	13001704	-	2733	1	CCDC3|-|-	8.926	6.495
NR_00352	chr15	22664739	22685367	+	3577	1	WHAMMP	0.011	0.031
NR_12542	chr8	1.26E+08	1.26E+08	+	1417	1	LOC10192	0	0
NM_0012	chr7	44072062	44082540	-	2618	1	POLM|Pol	7.141	5.305
NM_0011(	chr1	13223332	13226109	-	1439	2	PRAMEF22	0	0
NM_0159(	chr2	99337371	99400265	+	4672	1	EIF5B|IF2|	5.608	8.089
NM_0188	chr9	21329671	21335432	-	5759	1	KLHL9|-|-|	16.576	17.139
NM_0024	chr8	1.28E+08	1.28E+08	+	2361	1	MYC|MRT	4.085	6.469
NM_1447	chr5	35617887	35671574	+	2970	1	SPEF2|CT1	0.608	0.314

NM_0013	chr6	1.05E+08	1.05E+08	-	4419	1	HACE1|SP	4.365	4.042
NR_10385	chr8	75223117	75278481	-	1365	1	CASC9|ES	0	0.11
NM_0159	chr20	35542029	35557634	+	1336	1	ERGIC3|C	178.164	160.479
NR_04767	chr4	39527839	39594707	+	2962	1	UGDH-AS1	1.241	0.911
NR_10810	chr19	51685363	51693456	-	3960	1	SPACA6P-	0.12	0.107
NM_0011	chr4	15604384	15655412	-	3476	1	FBXL5|FBL	16.488	23.729
NR_12580	chr3	18445237	18530114	+	836	1	SATB1-AS1	0.062	0.082
NR_02987	chr8	14853438	14853510	-	72	1	MIR383|M	0	0
NM_0062	chr9	35697337	35732395	-	8375	1	TLN1|ILWI	8.637	8.208
NM_0178	chr2	70149885	70191019	-	2512	1	C2orf42|-|	4.332	5.769
NM_0025	chr12	1.18E+08	1.18E+08	+	1669	1	PEBP1|HC	236.664	227.211
NM_0010	chr5	1.4E+08	1.4E+08	+	2868	1	IGIP|C5orf	0.306	0.683
NM_0012	chr17	37978155	37989062	-	2062.8	10	TBC1D3L|-	0.1	0.186
NM_1707	chr3	50329786	50338233	-	1651	1	RASSF1|1	14.026	12.901
NR_03605	chr16	15010321	15010397	-	76	4	MIR1972-	0	0
NR_02437	chr19	7004953	7006415	-	1462	1	FLJ25758|	0.018	0.131
NM_1452	chr7	1.52E+08	1.52E+08	+	1724	1	GALNTL5|	0.194	0.747
NM_0012	chr1	1.6E+08	1.6E+08	-	1372	1	TAGLN2|H	20.195	19.847
NR_04500	chr11	17051886	17053044	+	1158	1	OR7E14P|	1.147	0.461
NM_0007	chr7	99648189	99680026	-	1740	1	CYP3A5|C	0.215	0.102
NM_0012	chr18	46962768	46964644	-	1874	1	ELOA3D|E	0	0
NM_0002	chr22	42058334	42070842	-	3715	1	NAGA|D2	9.489	7.19
NR_03982	chr9	1.37E+08	1.37E+08	-	86	1	MIR4674|	0	0
NM_0067	chr18	12328944	12377276	-	3190	1	AFG3L2|S	19.049	20.533
NR_04001	chr17	28599133	28617377	+	1362	1	SPAG5-AS	16.5	18.243
NM_0010	chrX	38801432	38806530	+	2003	1	MID1IP1|	16.157	12.904
NM_0010	chr1	44805910	44807285	-	1264	1	TCTEX1D4	1.357	0.498
NR_03769	chr2	2.34E+08	2.34E+08	-	813	1	LOC10028	0	0
NR_02427	chr1	15834215	15848147	-	2730	1	FLJ37453|	5.908	8.238
NM_0049	chr17	43483917	43524318	+	4230	1	DHX8|DD	20.794	24.603
NR_03164	chrX	32641474	32641559	-	85	1	MIR548F5	0	0
NR_07407	chr16	69741854	69754968	-	1636	1	NOB1|ART	13.737	12.843
NM_0220	chr2	1.62E+08	1.62E+08	+	5547	1	SLC4A10|	0.542	3.267
NM_0010	chr19	9251044	9252063	+	1018	1	OR7E24|H	0	0
NR_00302	chr14	21392151	21392253	-	102	1	SNORD9|r	0.758	1.074
NM_0014	chr6	1610446	1613894	+	3446	1	FOXC1|AR	0.251	0.06
NM_0142	chr17	31316410	31321749	-	2865	1	EVI2A|EV	0.014	0.01
NR_10674	chr12	62260359	62260454	+	95	1	MIR6125|	0.407	1.009
NR_03716	chr1	1.78E+08	1.78E+08	-	2964	1	LOC73010	0	0
NM_0010	chr7	1.3E+08	1.3E+08	+	3500	1	NRF1|ALP	10.621	12.703
NM_0038	chr4	1.73E+08	1.73E+08	+	1103	1	SAP30|-|-	11.818	12.766
NM_0027	chr1	35599541	35641844	-	4736	1	PSMB2|H	42.944	40.816
NM_0013	chr17	1771671	1777574	+	1141	1	SERPINF1|	7.56	12.377
NM_0006	chr4	99276366	99291028	-	1489	1	ADH1A|A	0.26	0.083
NR_12656	chr3	1.79E+08	1.79E+08	-	1789	1	KCNMB2-	0.05	0.046
NM_0012	chr12	1.33E+08	1.33E+08	+	1551	1	P2RX2|DF	0.083	0.097
NR_03750	chr5	1.32E+08	1.32E+08	-	109	1	MIR3936|	1.538	0.754
NR_14437	chr10	1.17E+08	1.18E+08	-	7198	1	EMX2OS|E	1.383	8.22
NM_0239	chr4	52862328	52866835	+	1951	1	RASL11B|-	8.228	15.445
NR_02435	chr22	41952187	41958942	+	1504	1	LINC00634	4.964	5.045

NM_0018!	chr15	75674322	75712848	-	8278	1	CSPG4|HM	0.746	0.351
NR_03828	chr3	62261819	62318947	-	2663	1	PTPRG-AS:	4.551	2.32
NR_12033	chr15	41972763	41999094	+	2495	1	PLA2G4E-/	0.016	0.005
NM_0071ε	chr3	52455508	52493072	+	5216	1	NISCH|I-1|	72.595	77.928
NR_12057	chr11	80751201	80762803	-	1544	1	LOC10192	0	0.009
NM_0061	chr5	55102646	55110252	+	889	1	GZMA|CTL	0.392	0.031
NR_03018	chr19	53666679	53666762	+	83	2	MIR512-1|	0	0
NR_02443	chr2	1.08E+08	1.08E+08	-	1666	1	RGPD4-AS	0	0
NM_0224	chr16	3046681	3060723	+	3539	1	MMP25|M	0.761	0.441
NM_0012ε	chr17	62704905	62808381	-	3459	1	MARCH10	0.093	0.107
NM_0005!	chr7	45888360	45893668	+	1644	1	IGFBP1|AF	0	0
NM_1780	chr5	1.35E+08	1.35E+08	+	1357	1	CATSPER3	0.466	0.414
NM_0806	chr20	63974113	63979642	-	2174	1	SAMD10|(	8.208	6.552
NM_1456	chrX	1.42E+08	1.42E+08	+	414	2	SPANXA2|	0	0
NM_0012ε	chr17	7614064	7633383	+	1165	1	SHBG|ABP	0.653	0.27
NM_0011!	chr11	35251205	35420063	-	11630	1	SLC1A2|EA	6.818	26.134
NM_0015	chr6	19837370	19842200	+	3871	1	ID4|IDB4|	40.356	30.341
NM_0012(	chr19	52949379	52962911	-	2590	1	ZNF816|ZI	1.354	1.608
NM_1779	chr8	95244913	95269234	-	3365	1	C8orf37|B	1.659	1.307
NR_13631	chr2	29474734	29475377	-	402	1	LOC10537	0	0
NM_0330	chr19	50790415	50795224	+	1334	1	ACPT|-|-|	0.174	0.205
NR_03973	chr15	80997417	80997473	-	56	1	MIR4514|	0	0.245
NM_0012(	chr11	8911154	8920079	+	1220	1	AKIP1|BCA	18.495	18.031
NM_0239	chr12	10907926	10909562	-	1634	3	TAS2R13|	0	0
NR_00002	chr11	17074653	17074744	-	91	1	SNORD14A	0.992	0.753
NM_0006	chr21	33324909	33359822	+	6081	1	IFNAR1|AV	3.797	4.764
NM_0013	chr6	1.16E+08	1.16E+08	+	4533	1	DSE|DS-ep	7.99	7.72
NR_02688	chr20	18787971	18793982	-	1379	1	LINC00652	1.187	3.536
NM_0013	chr11	1.19E+08	1.19E+08	+	1042	1	TTC36|HBI	2.623	3.313
NM_0010	chr1	2.47E+08	2.47E+08	+	3836	1	NLRP3|AG	0.269	0.2
NR_11094	chr16	2998955	3000813	+	196	1	LOC10192	0.132	0
NM_0013	chr17	64498254	64506686	-	3814	1	DDX5|G17	234.239	255.593
NR_04005	chr15	67514921	67521844	-	791	1	IQCH-AS1|	2.298	2.234
NM_0211!	chr1	2.12E+08	2.12E+08	-	2014	1	SLC30A1|Z	1.319	2.061
NR_12536	chr11	8011278	8016509	-	1580	1	CASC23|LI	0	0
NR_02408	chr7	1.31E+08	1.31E+08	+	3446	1	COPG2IT1	5.373	8.379
NM_0248	chr3	1.33E+08	1.33E+08	+	3823	1	UBA5|EIEE	6.212	7.033
NR_04665	chr1	1.08E+08	1.08E+08	+	616	1	VAV3-AS1	0.105	0
NM_1388(	chr5	10277595	10308056	-	4039	1	CMBL|JS-1	1.29	3.035
NM_1829	chr19	2389771	2426258	+	3346	1	TMPRSS9|	0.663	0.635
NM_0049ε	chr12	57550064	57584771	+	3866	1	KIF5A|D12	112.256	95.585
NM_0012(	chr17	82235774	82239499	+	2009	1	SLC16A3|I	11.841	0.532
NM_0013	chr1	2.05E+08	2.05E+08	+	7797	1	CNTN2|AX	239.349	116.756
NM_0011	chr1	66924895	66988619	+	3857	1	MIER1|ER	2.718	3.512
NR_02725	chr6	41208554	41217947	-	1132	1	TREML3P|	0.046	0
NM_0177	chr6	37257704	37332970	+	3515	1	TBC1D22B	13.928	17.348
NR_02730	chr19	19145567	19192591	-	2118	1	BORCS8-M	7.323	6.532
NM_0011	chr6	89926528	90296908	-	9100	1	BACH2|BT	9.834	13.608
NM_0028!	chr3	1.4E+08	1.4E+08	-	892	1	RBP1|CRA	17.548	10.964
NM_0013	chr2	27254572	27275321	-	1947	1	SLC30A3|Z	1.146	5.121

NR_02706	chr20	25763466	25796242	-	987	1	FAM182B|	0.614	0.666
NR_03893	chr10	1.02E+08	1.02E+08	+	1304	1	RPARP-AS1	2.6	4.233
NM_0011	chr12	10158300	10172191	-	2369	1	OLR1|CLE(	0	0
NM_0013	chr1	35869808	35924298	+	7315	1	AGO1|EIF2	20.961	19.245
NM_0013	chr2	2E+08	2.01E+08	-	2557	1	KCTD18|6	4.553	4.457
NR_12600	chr1	1.77E+08	1.77E+08	+	673	1	LINC01645	0	0.02
NM_0027	chr5	1.46E+08	1.46E+08	+	1178	1	POU4F3|B	0.088	0.151
NM_0011	chr17	35548125	35558091	-	2876	1	SLFN14|BI	0	0.01
NM_0328	chr7	1.34E+08	1.34E+08	-	6803	1	SLC35B4|Y	14.688	11.07
NR_12582	chr8	63465849	63475482	+	3218	1	LOC10272	0.02	0.017
NM_0013	chr21	36320189	36376646	+	4155	1	MORC3|N	5.195	7.318
NM_0187	chr7	21900899	21945924	-	2896	1	CDCA7L|JI	7.319	10.802
NM_0210	chr19	58278952	58295888	+	2204	1	ZNF8|HF.1	12.882	14.822
NM_0313	chr18	46946824	47102243	+	2816	1	KATNAL2|	0.916	0.715
NM_1488	chr17	81902109	81902905	+	700	1	NPB|L7|PI	2.468	4.363
NM_0218	chr1	1.57E+08	1.57E+08	+	1762	1	HAPLN2|B	0.856	1.632
NM_0011	chr20	57603846	57620576	-	2064	1	ZBP1|C20	0	0.013
NM_0221	chr19	49114361	49118460	+	747	1	LIN7B|LIN	26.046	23.525
NM_1768	chr7	1.42E+08	1.42E+08	-	1140	1	TAS2R38|	0.023	0
NM_0248	chr16	28904421	28925211	-	2265	1	RABEP2|FI	4.975	5.237
NR_00295	chr10	1E+08	1E+08	-	146	1	SNORA12|	2.473	0.469
NM_0011	chr19	41443158	41444765	+	942	1	ERICH4|C1	0	0.015
NM_0010	chr20	64083380	64084359	-	864	1	LKAAEAR1	0.642	0.412
NR_11001	chr20	51831797	51862913	-	2324	1	LINC01429	0.006	0.012
NR_03075	chr22	22822776	22822871	+	95	1	MIR650|M	0	0
NM_0047	chr1	2.02E+08	2.02E+08	+	2455	1	GPR37L1|	0.005	0.1
NM_0007	chr17	67044536	67056795	+	1312	1	CACNG1|C	0.55	0.741
NR_02792	chr22	50568861	50582999	-	4246	1	CHKB-CPT1	21.575	17.381
NM_0327	chr19	55339853	55348121	+	2298	1	KMT5C|SU	17.876	13.512
NM_0012	chr19	52927135	52962911	-	2674	1	ZNF816-ZN	0.318	0.507
NM_0065	chr2	1.11E+08	1.11E+08	+	4912	1	BCL2L11|E	3.588	4.977
NM_0025	chr7	23246697	23275110	+	2717	1	GPNMB|H	1.087	1.316
NM_0010	chr12	1.09E+08	1.09E+08	-	1041	1	ALKBH2|A	8.014	8.447
NM_0011	chr11	14457503	14499869	-	3344	1	COPB1|CC	27.129	31.699
NM_0012	chr14	89794991	89954777	-	2706	1	EFCAB11|	0.958	0.744
NM_0001	chr6	1.21E+08	1.21E+08	+	3165	1	GJA1|AVSI	3.251	2.605
NM_0165	chr17	81911939	81918182	-	1719	1	SIRT7|SIR2	8.656	8.016
NM_2074	chr15	38696598	38700038	+	2039	1	C15orf53|	0	0
NM_0011	chr1	45694326	45750813	-	2096	1	IPP|KLHL2	3.02	4.019
NM_0013	chr15	89471409	89496583	-	1926	1	RHCG|C15	0.074	0.085
NR_10501	chr5	74322486	74328357	+	781	1	LINC01333	0.033	0.105
NM_1816	chr21	30616455	30616625	+	169	1	KRTAP20-1	0	0
NM_0010	chrX	48007772	48009729	+	589	2	SPACA5B|	0	0
NR_10695	chr8	1.44E+08	1.44E+08	-	68	1	MIR6893|	1.327	0
NM_0013	chrX	1.01E+08	1.01E+08	+	3088	1	CENPI|CEN	0.86	0.785
NM_0029	chr20	45325289	45348424	-	2605	1	SDC4|SYN	1.49	0.473
NM_0528	chr7	1.41E+08	1.41E+08	+	2354	1	ADCK2|AA	8.714	12.074
NR_10995	chr22	37641832	37658377	-	1939	1	LOC10192	0.751	1.236
NR_11061	chr20	22280229	22282801	-	507	1	LINC01427	0	0
NM_0013	chr17	46851586	46886730	+	2647	2	WNT9B|W	0	0

NM_0248(	chr2	2.41E+08	2.41E+08	-	1954	1	C2orf54|-|	0.013	0
NM_1989!	chr20	2380908	2432753	+	2277	1	TGM6|SC/	0	0
NM_0013:	chr8	1.41E+08	1.41E+08	-	4310	1	PTK2|FADI	27.579	28.564
NR_10505	chr5	1.4E+08	1.4E+08	-	698	1	PSD2-AS1|	0.074	0
NM_0036(	chrX	1.09E+08	1.09E+08	-	3878	1	IRS4|IRS-4	0.146	0.039
NM_0005!	chr2	2.17E+08	2.17E+08	-	6233	1	IGFBP5|IB	8.614	27.409
NM_0063(	chr14	39031919	39103528	-	4114	1	SEC23A|CI	14.097	13.474
NM_0031:	chr1	1.69E+08	1.69E+08	-	560	1	XCL2|SCM	0	0
NM_0012(	chr9	1.37E+08	1.37E+08	-	634	1	EDF1|CFAI	359.411	256.851
NR_12589	chr4	4761760	4787374	+	2383	1	LOC10192:	0.427	0.759
NR_04642	chr9	83623049	83644130	+	1002	1	IDNK|C9or	1.518	1.381
NM_0010:	chrX	1.03E+08	1.03E+08	-	1161	1	TCEAL5|W	2.621	6.288
NM_0032:	chr1	2.36E+08	2.36E+08	+	2033	1	GPR137B|	8.226	8.853
NM_0008(	chr1	23191895	23194729	-	2832	1	HTR1D|5-I	0.096	0.073
NR_03017	chr14	1.01E+08	1.01E+08	+	93	1	MIR432|M	6.516	6.775
NM_0053:	chr9	1.25E+08	1.25E+08	-	3960	1	HSPA5|BIF	83.394	73.372
NM_0142:	chr1	2.15E+08	2.15E+08	+	3265	1	KCNK2|K2	1.441	1.733
NM_0018:	chr20	3783851	3786690	-	2837	1	CENPB|-|-	30.75	30.32
NM_0151:	chr16	28097976	28211918	-	4464	1	XPO6|EXP	37.173	49.056
NM_0227(	chr12	1.23E+08	1.23E+08	-	4259	1	MPHOSPH	1.617	2.361
NR_11050	chr14	19421026	19424584	+	2080	1	LOC10192!	0	0
NM_1817(	chr12	49836043	49843129	-	3245	1	BCDIN3D|	7.243	8.18
NM_1775:	chr8	1.09E+08	1.1E+08	+	13937	1	PKHD1L1|	0.057	0.067
NM_0155(	chr3	1.94E+08	1.94E+08	+	6297	1	OPA1|BER	6.173	8.444
NM_0159(	chr10	99732201	99756137	+	1357	1	CUTC|CGI-	11.202	12.516
NR_12036	chr8	1.27E+08	1.27E+08	-	320	1	CASC19|C/	0	0
NM_0011!	chr9	1.37E+08	1.37E+08	+	1498	1	RNF224|-|	0.706	0.521
NM_0028(	chr19	18196801	18204064	-	1495	1	RAB3A|-|-	187.279	232.565
NM_0220!	chr16	788622	798074	+	3066	1	CHTF18|C:	3.793	3.735
NM_0225(	chr17	63909597	63911328	-	660	1	CSHL1|CS-	0.039	0
NM_0013:	chr11	1.32E+08	1.33E+08	-	6647	1	OPCML|IG	1.787	3.893
NM_0061:	chr1	2.1E+08	2.1E+08	-	4494	1	IRF6|LPS|(	0.009	0.101
NM_1532(	chr3	50299889	50303601	-	1281	1	HYAL1|HY.	1.057	0.92
NR_13072	chr6	30812866	30830659	-	1890.7	6	LINC00243	0	0
NM_0012!	chr5	41925252	41941570	+	1327	1	FBXO4|FB.	0.262	0.557
NM_0011(	chr17	4988130	4997610	-	1256	1	INCA1|HSI	3.11	3.173
NR_04985	chr2	1.35E+08	1.35E+08	+	53	1	MIR5590|	0	0
NM_1522(	chr19	9607326	9621240	-	4532	1	ZNF561|-|	5.533	6.371
NR_11078	chr18	14969607	14970468	-	761	1	LINC01444	0	0.072
NM_0010!	chr2	2.08E+08	2.08E+08	-	1203	1	C2orf80|G	12.808	20.187
NM_2058!	chr7	1.36E+08	1.36E+08	-	1795	1	FAM180A|	0	0.008
NM_0325:	chr20	50731544	50753741	+	4610	1	PARD6B|P	0.461	0.544
NM_1471(	chr4	20728616	20850668	-	2049	1	KCNIP4|C/	0.799	0.408
NR_12064	chr10	52450877	52470533	-	1003	1	LINC01468	0	0
NR_12048	chr12	253442	257299	-	500	1	LOC10272.	0	0
NM_0011(	chr2	39665498	39717963	+	1343	1	TMEM178	8.372	13.401
NR_04700	chr13	78054855	78617325	+	2412	1	RNF219-AS	4.827	7.257
NM_0010:	chr7	1.03E+08	1.03E+08	+	2211	1	LRRC17|P:	3.12	1.858
NR_13393	chr4	20696282	20752900	+	1377	1	PACRGL|C	4.279	5.521
NR_11079	chr18	53568447	53597851	+	562	1	LINC0191!	0	0

NR_10978	chr1	24539274	24556024	-	970	1	LOC10050	0.572	4.264
NM_0532	chr11	196761	200258	+	1275	1	ODF3|CT1	0	0
NM_0146	chr19	34254551	34355566	+	4832	2	KIAA0355|	4.363	3.583
NM_1526	chr1	2.23E+08	2.23E+08	+	2095	1	CCDC185|	0	0
NM_0011	chr15_KI2	1196592	1393360	+	3588	2	APBA2|D1	1.782	2.191
NR_03770	chr2	2.16E+08	2.16E+08	+	3303	1	LINC01963	13.081	14.282
NM_0011	chr14	22773223	22815898	-	2152	1	SLC7A7|LA	0.677	0.42
NM_1334	chr10	86970741	87010203	+	3141	1	AGAP11|-	0.021	0
NR_03966	chr5	1.29E+08	1.29E+08	-	85	1	MIR4460|	0	0
NM_0012	chr13	27962068	27969368	-	2346	1	CDX2|CDX	0	0.006
NM_0179	chr1	85649423	85708433	-	6185	1	ZNHIT6|BC	1.772	1.661
NM_0037	chr12	53506688	53507638	-	589	1	NPFF|FMR	2.561	2.349
NM_0145	chr12	1.01E+08	1.01E+08	+	8946	1	UTP20|1A	1.31	1.602
NM_0010	chr16	66802878	66830976	-	1688	1	NAE1|A-1	16.231	26.088
NR_10466	chr16	79770471	79801343	+	716	1	LINC01229	0.108	0.019
NM_0053	chr7	1.32E+08	1.32E+08	-	5878	1	PODXL|Gp	9.66	12.83
NM_1818	chr8	78516101	78605267	+	4067	1	PKIA|PRKA	67.796	87.864
NM_0240	chr7	30496621	30504841	-	1164	1	GGCT|C7o	17.845	15.391
NM_0011	chr1	74705488	74733408	-	2152	1	CRYZ|-|-|	7.387	3.545
NM_0019	chr3	52048921	52056445	-	3228	1	DUSP7|MI	7.537	7.774
NR_13241	chr1	39841031	39883511	-	1301	1	TRIT1|GRC	13.627	13.539
NM_0056	chr1	1.74E+08	1.74E+08	+	1469	1	GPR52|-|-	0.035	0.084
NM_0011	chr9	1.24E+08	1.24E+08	+	2563	1	NEK6|SID6	3.149	10.784
NM_0011	chr2	2.31E+08	2.31E+08	+	3684	1	CAB39|CG	17.16	18.408
NM_1734	chr19	15508525	15552317	+	2593	1	CYP4F22|	0	0
NM_0014	chrX	49560876	49568204	+	519	3	GAGE4|CT	0	0
NM_2136	chr19	1505018	1513189	-	2843	1	ADAMTSL5	0.136	0.149
NM_1733	chr21	6111130	6123742	+	4696	2	SIK1|MSK|	0.813	0.598
NM_0012	chr21	42849226	42879589	-	1985	1	WDR4|TRI	4.086	4.375
NR_03382	chr1	43699738	43707341	-	1555	1	KDM4A-AS	4.668	7.214
NM_0190	chr8	1.44E+08	1.44E+08	+	890	1	EXOSC4|R	23.57	22.885
NM_0007	chr10	94762681	94853206	+	1779	1	CYP2C19|	0	0.008
NM_0323	chr11	47164298	47177125	-	3004	1	ARFGAP2|	47.724	46.248
NM_0019	chr5	76716043	76735780	+	3816	1	F2R|CF2R|	14.968	16.687
NR_02949	chr9	95086021	95086088	+	67	1	MIR24-1|N	0	0.204
NM_0208	chr11	45240302	45286333	-	5149	1	SYT13|-|-|	97.095	64.213
NM_0133	chr2_KI27	25455	71290	+	1213	2	C2orf27A|	0.021	0
NM_0306	chr22	36189128	36204833	-	3409	1	APOL4|AP	1.157	1.483
NM_0066	chr6	25845100	25874243	-	1560	1	SLC17A3|	0.017	0
NM_0012	chr1	1.54E+08	1.54E+08	-	3048	1	TPM3|CAF	52.851	57.017
NM_0012	chr12	911736	990041	-	3149	1	RAD52|-|-	5.175	6.516
NM_0013	chr2	70281362	70293224	-	537	1	SNRPG|SM	19.856	13.978
NM_0011	chr5	96875939	96919716	+	5247	1	ERAP2|L-R	0.042	0.034
NM_0175	chr17	10443291	10469559	-	5973	1	MYH4|MY	0	0
NM_0066	chr9	1.09E+08	1.09E+08	+	1525	1	ACTL7A|-|	0	0.018
NR_02421	chr19	47923779	47923899	+	120	10	SNAR-A3|	0	0
NM_2058	chr3	52833115	52835219	-	724	1	MUSTN1|	13.036	1.949
NM_2073	chr16	73126638	73144447	-	1301	1	C16orf47|	0.268	0.084
NR_03669	chr1	2.4E+08	2.4E+08	+	951	1	RPS7P5|RI	0.027	0.029
NM_1384	chr13	49628299	49633872	+	3756	1	ARL11|ARI	0	0

NM_0070	chr16	30183410	30189076	+	1802	1	CORO1A|(	34.437	26.748
NM_0013	chr8	22054817	22082527	+	2310	1	DMTN|DM	40.64	38.524
NM_0012	chr11	67403913	67410090	+	1512	1	TBC1D10C	0.495	0.362
NM_0230	chr19	13795460	13832230	+	4324	1	ZSWIM4|-	25.095	15.211
NM_0013	chr6	96563125	96618624	+	4041	1	FHL5|170	0.003	0.003
NR_12640	chr3	1.65E+08	1.65E+08	-	840	1	LINC01324	0.276	0.033
NM_0010	chr10	71816283	71851375	-	2877	1	PSAP|GLB	476.974	563.358
NM_0013	chrX	13770922	13938824	-	3074	1	GPM6B|M	75.683	80.07
NM_0038	chr17	7549058	7557890	+	1377	1	TNFSF12|	0.393	0.408
NR_10415	chr6	91629040	91690428	-	1604	1	CASC6|-|-	0.104	0.154
NM_0154	chr8	11284491	11328145	+	7393	1	MTMR9|C	8.729	8.35
NM_0036	chr15	50182196	50236392	+	2379	1	SLC27A2|	1.263	0.892
NM_2055	chr5	80487981	80542563	+	1697	1	FAM151B|	0.509	0.565
NM_0198	chr19	42230186	42242584	-	2166	1	GSK3A|-|-	75.635	84.87
NM_0013	chr8	75407670	75566842	+	4429	1	HNF4G|NF	0.032	0.04
NR_02989	chr17	7223297	7223379	-	82	1	MIR324|M	0.314	3.508
NM_0013	chr2	71068278	71078868	+	1413	1	NAGK|GN	41.418	34.073
NM_0204	chr1	1.17E+08	1.17E+08	+	6301	1	PTGFRN|C	4.393	20.058
NM_0011	chr9	1.29E+08	1.29E+08	-	1904	1	KYAT1|CC	10.821	12.474
NM_0044	chr14	37589552	37595120	-	3376	1	FOXA1|HN	0.462	0.385
NM_0058	chr5	1.4E+08	1.4E+08	+	5291	1	PURA|MR	0.975	1.395
NM_0245	chr16	53829	57669	+	1079	1	SNRNP25|	41.13	30.606
NR_04511	chr20	30402908	30403462	-	554	1	MLLT10P1	0	0.025
NM_0011	chr11	26559032	26572268	-	3236	1	MUC15|M	0	0
NM_0057	chr1	47183589	47190099	-	888	1	PDZK1IP1|	0	0
NM_0011	chr19_GL9	869085	903924	+	3490	10	NLRP2|CL	0	1.162
NM_0036	chr9	1E+08	1.01E+08	+	2474	1	TMEFF1|C	52.804	56.842
NM_0024	chr4	677922	682028	+	652	1	MYL5|-|-|	11.944	7.878
NM_0011	chr1	1.46E+08	1.46E+08	-	1629	1	PEX11B|PI	28.138	30.413
NR_12615	chr11	1.32E+08	1.32E+08	-	676	1	NTM-AS1|	0.038	0
NM_0065	chr1	2.29E+08	2.29E+08	+	1119	1	SPHAR|-|-	5.012	4.81
NM_1525	chr5	1.81E+08	1.81E+08	+	3487	1	BTNL9|BT	0.033	0.039
NM_0132	chr6	16129086	16148247	+	3062	1	MYLIP|IDC	4.118	2.603
NM_0012	chr1	2.42E+08	2.42E+08	+	1294	1	BECN2|BE	0	0
NM_0030	chr6	1.6E+08	1.6E+08	-	2499	1	SLC22A2|(	0	0
NM_0011	chr11	1.25E+08	1.25E+08	+	4119	1	SLC37A2|p	0.266	0.103
NM_0142	chr8	70573218	70608459	-	3086	1	TRAM1|PN	7.082	5.592
NM_0014	chr9	1.11E+08	1.11E+08	-	3084	1	LPAR1|ED	0.807	1.292
NR_00282	chr7	97966596	97972326	-	694	1	MGC7208	1.746	1.757
NM_0011	chr7	1.49E+08	1.49E+08	-	3791	1	ZNF746|P	10.428	11.934
NR_10275	chr15	96772005	96783337	-	492	1	SPATA8-AS	0	0
NM_0332	chr7	44876293	44885385	-	9090	1	PURB|PUF	2.824	3.312
NM_1757	chrX	48186220	48196763	-	1251	1	SSX5|-|-|X	0	0
NM_0013	chr18	3247482	3256237	+	950	1	MYL12A|H	38.68	22.232
NM_0013	chr17	81375432	81385125	-	1176	1	LOC10013	1.546	1.351
NM_0037	chr1	1E+08	1E+08	+	2571	1	RTCA|RPC	12.392	10.181
NM_0010	chr11	1629803	1630929	+	907.8	4	KRTAP5-5|	0	0
NR_12062	chr10	1.31E+08	1.31E+08	+	470	1	TCERG1L-A	0.11	0
NM_0064	chr7	16792640	16805114	-	973	1	AGR2|AG-	0.027	0
NM_0012	chr12	55966769	55972789	+	2114	1	CDK2|CDK	20.157	17.163

NM_0011:	chr15	40815445	40828709	-	866	1	PPP1R14D	0.194	0.047
NM_0011(	chr8	38231953	38262769	+	4512	1	DDHD2|SA	16.083	14.778
NM_1452:	chr1	46607715	46615133	-	2798	1	MOB3C|M	1.189	1.542
NM_0052(	chr22	20917426	20953749	+	5320	1	CRKL|-|-|2	39.012	50.095
NM_0011:	chr6	1.68E+08	1.68E+08	-	1013	1	HGC6.3|-|	0.025	0.014
NM_0248:	chr10	25016579	25026666	+	3767	1	THNSL1|TS	2.454	2.96
NM_0043:	chrX	70268335	70281840	+	1290	1	ARR3|ARR	0.1	0.202
NR_10695	chr3	49099854	49099914	-	60	1	MIR6890|I	10.745	19.632
NM_1523:	chr14	1.03E+08	1.03E+08	-	1620	1	ANKRD9|-	3.677	3.779
NM_0185!	chr6	1.11E+08	1.11E+08	+	2883	1	SLC16A10|	0.273	0.352
NM_0013:	chr19	56221303	56314925	-	1680	1	ZSCAN5A|	2.993	3.913
NM_1308:	chr3	1.94E+08	1.94E+08	-	5879	1	LRRC15|LI	0.053	0.019
NM_0010.	chr14	20343614	20357904	+	1830	1	PARP2|AD	26.118	32.701
NM_0052:	chr7	97020390	97024831	-	1419	1	DLX5|SHFI	0.018	10.792
NR_03618	chr11	1859464	1859536	-	72	1	MIR4298|-	0	0
NM_0013:	chr1	1.19E+08	1.19E+08	-	3546	1	TBX15|TB)	0.047	0.042
NR_03619	chr15	75762215	75762315	-	100	1	MIR4313|-	0.258	0
NM_0010!	chr10	87225448	87234976	+	3279	1	NUTM2A|	0.287	0.217
NM_0145:	chr17	5105734	5112274	-	1871	1	ZNF232|ZS	19.233	24.868
NR_10403	chr10	46369087	46373563	+	346	1	FAM25BP|	0	0
NR_02448	chr3	1.94E+08	1.94E+08	-	3010	1	LINC00887	0.009	0
NM_0010{	chr9	95442982	95508040	-	7618	1	PTCH1|BC	2.984	5.326
NR_03997	chr1	52928674	52928772	+	98	1	MIR1273F	0	0.14
NM_0011:	chr4	1.58E+08	1.58E+08	-	4787	1	FAM198B|	0.649	0.289
NM_0009.	chr5	1.23E+08	1.23E+08	-	1229	1	PPIC|CYPC	4.878	2.274
NM_0010:	chr17	80101526	80119882	+	3520	1	GAA|LYAG	20.685	22.11
NR_03962	chr1	25023503	25023586	+	83	1	MIR4425|-	0	0
NM_0008:	chr6	1.6E+08	1.6E+08	+	13992	1	IGF2R|CD2	11.49	14.675
NM_0011.	chr4	1.86E+08	1.86E+08	-	4333	1	SORBS2|A	13.825	11.56
NM_0020:	chr1	2.03E+08	2.03E+08	-	3252	1	FMOD|FM	0.024	0.088
NM_0011(	chr3	1.2E+08	1.2E+08	-	4170	1	GPR156|G	0.492	1.429
NR_02407	chr18	14477955	14498706	-	1607	1	CXADRP3|	0	0
NR_02682	chr2	1.14E+08	1.14E+08	+	1127	1	FAM138B|	0	0
NR_13516	chr19	751113	764319	+	1044	1	MISP|C19(	1.643	1.614
NR_02421	chr19	47907678	47907799	+	121	2	SNAR-A13	0	0
NR_03780	chr1	11840319	11847616	+	1658	1	NPPA-AS1	15.188	17.555
NM_0012!	chr10	50067888	50133509	+	4613	1	WASHC2A	3.432	3.15
NM_0252(	chr14	1.06E+08	1.06E+08	+	1528	1	TMEM121	3.527	3.953
NR_12373	chr17	20938520	20982357	+	1682	1	LOC33926(	0	5.432
NM_0010{	chrX	48761750	48773648	+	731	1	GLOD5|-|-	0	0
NR_12633	chr14	51916145	51918571	-	526	1	LOC10272:	0	0
NR_02404	chr6	1.5E+08	1.5E+08	-	1879	1	RAET1K|-|	0.295	0.109
NR_11099	chr5	1.32E+08	1.32E+08	-	1794	1	LOC55310:	0.704	0.458
NM_0044!	chr15	59638062	59657538	-	1575	1	GTF2A2|H	36.888	36.908
NR_13659	chr15	72241181	72272575	-	2549	1	PARP6|AR	139.81	117.835
NM_0013(	chr11	64987945	64990277	-	1949	1	BATF2|SAI	0.02	0.042
NM_0070.	chr6	1.5E+08	1.5E+08	-	1973	1	KATNA1|-	7.574	7.741
NR_03164	chr14	1.02E+08	1.02E+08	-	135	1	MIR1247|I	1.91	1.42
NR_11054	chr22	26672843	26780893	+	1676	1	MIATNB|-	10.27	6.742
NM_0066(	chr22	32354885	32361161	+	1209	1	RFPL3|-|-|	0.427	0.464

NR_04610	chr2	2.26E+08	2.26E+08	+	928	1	LOC64673	0	0.015
NM_1458	chr5	1.8E+08	1.8E+08	+	660	1	LTC4S|-|-|	0.645	0.789
NR_13013	chr17	14301050	14349404	+	3098	1	HS3ST3B1	0.886	1.22
NM_0013	chr15	89575587	89628022	+	6642	1	TICRR|C15	1.297	1.679
NR_13564	chr1	78649789	78664078	+	980	1	IFI44|MTA	1.066	6.695
NR_03965	chr4	76573568	76573632	+	64	1	MIR4450|	0	0
NM_0151	chr12	1.33E+08	1.33E+08	-	4467	1	ANKLE2|K	20.551	21.976
NR_03896	chr7	1.58E+08	1.58E+08	+	4296	1	LOC10050	0.021	0.016
NM_1452	chr4	1E+08	1E+08	-	2629	1	DDIT4L|RE	0.795	0.568
NR_03621	chr1	37161563	37161634	+	71	1	MIR4255|	0	0
NM_0013	chr16	29381318	29403802	-	3488	1	NPIPB11|	0.126	0.079
NR_10701	chr15	51314034	51314109	+	75	1	MIR7973-1	0	0
NR_00302	chr1	12507246	12507397	+	151	2	SNORA59A	0	0
NR_04768	chr10	96191699	96271516	-	1515	1	BLNK|AGN	0.068	0.072
NM_0208	chr19	11199293	11262492	-	6350	1	DOCK6|AC	1.88	2.668
NM_0010	chr9	96500113	96566921	-	5115	1	CDC14B|C	1.58	2.391
NM_2073	chr9	116231	118417	-	2184	1	FOXD4|FK	0.242	0.22
NR_04000	chr1	2.47E+08	2.47E+08	-	961	1	LINC01743	0	0
NR_10377	chr16	88731180	88741425	+	3773	1	LOC10028	0.605	0.541
NM_0010	chr1	12167003	12209220	+	3663	1	TNFRSF1B	0.032	0.12
NM_0184	chr20	64047726	64049626	-	1701	1	SOX18|HL	0.038	0.089
NM_0176	chr17	63819433	63827671	-	3536	1	FTSJ3|EPC	17.247	16.718
NM_0244	chr2	2.32E+08	2.32E+08	-	772	1	NPPC|CNP	0.919	3.726
NM_1537	chr2	1.12E+08	1.13E+08	+	5154	1	TTL|-|-|2c	17.194	21.337
NM_0176	chr5	1.33E+08	1.33E+08	-	2171	1	ZCCHC10|	3.777	3.855
NR_02685	chr6	2987967	2991171	+	1571	1	LINC01011	3.266	1.822
NM_0013	chr4	1347556	1388049	+	4536	1	UVSSA|KIA	2.632	3.092
NM_1391	chr19	6464238	6467219	+	1111	1	CRB3|-|-|	0.221	0.284
NM_0570	chr1	43933320	43937240	+	1913	1	ARTN|ART	0.728	0.959
NM_0012	chr3	58332890	58426127	+	2700	1	PXK|MON	5.31	7.767
NM_0011	chrX	40623566	40624139	+	571	1	MPC1L|-|-	0.203	0
NR_03998	chr5	8457687	8463096	+	3242	1	MIR4458H	0.036	0.224
NM_0011	chr1	2.02E+08	2.02E+08	-	2914	1	PTPN7|BP	0.009	0.019
NR_03028	chr2	1.88E+08	1.88E+08	+	96	1	MIR561|M	0	0
NM_1384	chr6	73425133	73452320	-	1795	1	MB21D1|	0.029	0.031
NR_04703	chr13	34435451	34640685	-	390	1	LINC00457	0	0
NR_10417	chr19	12195015	12207576	+	372	1	LOC10028	0.208	0.147
NM_1382	chr19	23739195	23758891	-	6491	1	ZNF681|-|	0.481	0.528
NM_0013	chr10	69088028	69104811	+	1146	1	SRGN|PPG	0.023	0.036
NR_00295	chr7	56060470	56060602	+	132	1	SNORA15|	0	0
NM_0013	chr2	72175984	72826042	-	5761	1	EXOC6B|S	10.425	13.718
NR_11085	chr17	80801640	80805632	-	3200	1	LOC10192	0.008	0.009
NM_0021	chr6_GL00	4487777	4501470	+	4045.5	8	HLA-DPB1	0.153	0.159
NM_1788	chr15	65045370	65053396	+	934	1	SLC51B|O	0.966	0.704
NR_10994	chr5	16615926	16629969	+	1670	1	LOC10192	0.193	0.353
NM_0010	chr8	22565666	22575495	+	2028	1	SORBS3|S	16.066	21.376
NM_1768	chr16	56632622	56633986	+	435	1	MT1M|MT	0.059	0.063
NR_00440	chr1	1.46E+08	1.46E+08	-	163	9	RNU1-3|H	0	0
NM_0011	chr3	1.88E+08	1.89E+08	+	18207	1	LPP|-|-|3c	0.314	0.511
NM_0201	chr3	52433035	52445085	-	4957	1	SEMA3G|s	0.057	0.039

NR_04664	chr6	1.67E+08	1.67E+08	+	859	1	RPS6KA2-/	0.09	0.096
NM_0026:	chr17	8140470	8152435	-	4684	1	PER1|PER	11.256	10.507
NM_0012{	chrX	1.31E+08	1.31E+08	-	4018	1	ENOX2|AP	11.729	4.701
NM_0046:	chr17	68267026	68291264	-	3820	1	SLC16A6|I	0.064	0.043
NM_0151{	chr5	66828776	67169595	+	9887	1	MAST4|-|-	3.781	3.704
NM_0036{	chr1	32775239	32818032	-	3102	1	YARS|CMT	58.091	83.586
NR_12047	chr12	5233996	5243151	-	843	1	LOC10192!	0	0
NM_0010:	chr11	3227811	3232386	-	1292	1	MRGPRE|(	0	0
NM_0005:	chr17	41582279	41586895	-	1626	1	KRT14|CK:	0	0.084
NR_00278	chr6	1.7E+08	1.7E+08	-	1327	1	LOC15444!	0	0
NM_0011‹	chr11	1.3E+08	1.3E+08	-	5022	1	NFRKB|IN(	14.252	13.048
NR_13512	chr9	62834248	62838346	-	4098	1	LOC10013:	0.686	0.264
NM_0012‹	chr14	1.05E+08	1.05E+08	+	6145	1	PACS2|PA(	51.438	51.433
NM_0013:	chr17	8288860	8295343	-	2038	1	SLC25A35|	8.667	7.205
NM_0041:	chr20	38346411	38377011	+	1877	1	LBP|BPIFD	0.027	0.08
NM_1735(	chr7	1E+08	1E+08	+	3574	1	NYAP1|C7	36.545	34.193
NM_0056!	chr7	1.51E+08	1.51E+08	-	2375	1	ABCF2|AB	22.367	24.505
NM_1459:	chr7	1E+08	1E+08	+	1984	1	ZSCAN21|I	20.575	25.785
NR_10460	chr5	10493527	10502728	-	2127	1	LOC10192!	0	0.006
NM_0012{	chr21	41432050	41459214	+	1969	1	MX1|IFI-7{	0.079	0.452
NR_13423	chr7	93890894	93893585	+	528	1	LOC10537:	0.415	0.13
NM_1986!	chr21	44646414	44647649	+	1233	1	KRTAP10-1	0	0
NM_0011‹	chr1	46247695	46278473	+	2528	1	RAD54L|H	3.091	2.227
NR_00153	chrY	9801153	9813245	+	422	1	TTTY22|N(	0	0
NM_0012:	chr7	74231502	74254458	-	1625	1	RFC2|RFC‹	23.946	19.951
NR_03987	chr17	28360654	28360734	+	80	1	MIR4723|(	1.289	2.911
NM_0031{	chr11	1.17E+08	1.17E+08	+	1153	1	TAGLN|SN	14.985	21.454
NM_0012{	chr16	28711417	28735729	-	3036.5	2	EIF3C|EIF3	1.805	2.26
NM_0012:	chr11	1.17E+08	1.17E+08	+	5513	1	CEP164|N	8.782	7.441
NR_03342	chr12	50979783	51026416	-	2192	1	SLC11A2|/	10.629	13.297
NM_0010(	chr11	59514913	59515857	+	943	1	OR4D9|OF	0	0
NM_0052(	chr1	34755047	34758512	+	1351	1	GJB5|CX3:	0.086	0.314
NM_1527:	chr4	80335720	80963756	+	887	1	C4orf22|-|	0.029	0
NM_0013:	chr21	6560724	6564489	+	1101	2	LOC10272‹	0	0
NR_13393	chr7	1.33E+08	1.33E+08	-	448	1	LOC10537:	0	0
NM_0010:	chr18	77012783	77132818	-	4883	1	MBP|-|-|1	0.813	0.272
NR_00319	chr14	1.01E+08	1.01E+08	+	70	1	SNORD11‹	0	0
NM_1463{	chr19	10251964	10260045	+	1310	1	MRPL4|CG	33.671	27.289
NR_02959	chr7	1.28E+08	1.28E+08	+	71	1	MIR129-1|	0	0
NR_13658	chr3	44625019	44648063	+	2674	1	ZNF197|D:	4.976	4.964
NM_0181:	chr17	2322688	2337384	-	4863	1	TSR1|-|-|1	18.236	18.389
NM_0008‹	chr3	6861115	7741531	+	4135	1	GRM7|GLI	1.347	0.881
NM_0012:	chr4	1.1E+08	1.1E+08	-	1652	1	CASP6|M(	6.782	4.784
NM_2033:	chr3	1.98E+08	1.98E+08	+	3251	2	BDH1|BDH	0.143	0.206
NM_0047:	chr1	1.56E+08	1.56E+08	-	4083	1	ARHGEF2|	61.998	61.652
NM_0313(	chr19	3490821	3500623	-	1736	1	DOHH|HLI	7.68	6.959
NR_03035	chr13	99356131	99356228	+	97	1	MIR623|N	0	0.141
NM_0011(	chr6	1.44E+08	1.44E+08	+	9277	1	PHACTR2|	0.192	0.282
NR_03040	chr14	1.01E+08	1.01E+08	+	65	1	MIR668|N	0	0
NR_00299	chr1	27834401	27834566	+	165	1	SCARNA1|	0	0.166

NM_1332(	chr19	3585571	3593541	+	4309	1	GIPC3|C19	0.048	0.079
NR_04660	chr7	27168899	27171915	+	1158	1	HOXA10-A	0.011	0
NM_0179(	chr10	1.01E+08	1.01E+08	+	6902	1	HIF1AN|FI	11.386	13.03
NR_10502	chr5	90410000	90410669	+	473	1	LOC73115	0.082	0.058
NR_02424	chr19	50140202	50140320	-	118	1	SNAR-D|-|	0	0
NR_13503	chr12	65544238	65557311	-	722	1	LOC10536	0	0
NM_0143	chr7	38723943	38909200	-	4863	1	VPS41|HV	14.726	16.66
NM_0013	chr20	44714844	44727812	+	1553	1	WISP2|CC	0.033	0.123
NM_0013(	chr18	25061924	25352250	-	4921	1	ZNF521|EI	34.777	21.991
NR_12577	chr7	1.53E+08	1.53E+08	-	1004	1	LINC01287	0	0
NR_13238	chr19	47757036	47768840	-	537	1	GLTSCR2-A	0.816	0.867
NR_03892	chr12	12979837	12984642	+	1740	1	LOC10050	0.059	0.008
NM_0149	chr18	80109031	80140345	+	5381	1	ADNP2|ZN	12.251	11.653
NM_0156(	chr10	1.26E+08	1.26E+08	+	4553	1	EDRF1|C1	3.024	4.305
NM_0329	chr13	52843974	52848640	-	3707	1	PCDH8|AR	8.594	25.717
NR_03759	chr15	28789834	28856574	+	2086	1	PDCD6IPP	0.822	0.197
NM_0011	chr11	65014113	65022185	+	893	1	ARL2|ARFI	81.404	82.566
NM_0012	chr3	48625723	48635493	-	2298	1	SLC26A6|-	4.792	4.172
NR_03770	chr17	75979217	76005999	+	3320	1	TEN1-CDK	5.037	4.786
NM_0058(	chr2	1.61E+08	1.61E+08	+	1703	1	PSMD14|F	47.183	55.608
NM_0017(	chr9	1.01E+08	1.01E+08	-	3398	2	BAAT|BAC	0.038	0.032
NM_0012	chr12	2877199	2889525	+	1904	1	RHNO1|C1	41.626	46.753
NM_0063	chr19	18173780	18178124	+	1019	1	IFI30|GILT	2.948	2.419
NM_0064	chr13	76991924	77002517	+	2794	1	CLN5|NCL	5.833	5.853
NM_0010(	chr22	50311963	50327060	-	1984	1	DENND6B	33.592	18.502
NM_0329	chr20	18507544	18561406	+	3088	1	SEC23B|CI	41.273	44.861
NM_0011	chr19	49426431	49428818	+	1553	1	GFY|Goofy	2.175	0.6
NM_0010	chrX	41085635	41236579	+	12354	1	USP9X|DF	9.462	12.544
NM_0159	chr17	75261668	75266376	+	1408	1	MRPS7|M	48.415	41.5
NM_0187(	chr19	4791716	4795559	+	3841	1	FEM1A|EP	12.793	15.056
NM_0121	chr17	36948875	37056871	+	2127	2	AATF|BFR	0	0
NM_0030(	chr20	45252239	45254564	-	590	1	SLPI|ALK1	0.044	0
NM_1780(	chr13	33103135	33285764	-	5910	1	STARD13|	0.441	1.198
NM_1994	chr3	49277144	49340103	-	3956	1	USP4|UNF	17.147	18.524
NM_0010	chr7	1.01E+08	1.01E+08	+	1012	1	MOSPD3|	40.948	34.134
NR_03963	chr5	58530043	58530109	-	66	1	MIR548AE	0	0
NR_03766	chr12	89419721	89525567	-	3035	1	POC1B|CC	1.58	2.144
NM_0017	chrX	1.36E+08	1.36E+08	+	1408	1	BRS3|BB3	0.082	0.156
NM_0010	chr19	10286967	10288584	+	1260	1	ICAM4|CD	0.072	0.076
NR_13632	chr2	85213652	85218169	+	396	1	LOC10272	0	0
NM_0188	chr7	87402045	87475703	-	3797	1	ABCB4|AB	0.343	0.115
NM_0023	chr6	83210391	83431219	-	3501	1	ME1|HUM	0.029	0
NR_03016	chr10	1.02E+08	1.02E+08	+	72	1	MIR146B|	0	0
NM_0010	chr15	55430308	55508234	-	1876	1	DYX1C1|C	1.505	1.278
NM_1775	chr16	30199228	30204329	+	1389	2	SULT1A3|	0	0
NR_03612	chr11	69083176	69083258	+	82	1	MIR3164|	0.314	0
NM_0024	chr1	1.59E+08	1.59E+08	+	1658	1	MNDA|PY	0	0
NM_0177	chr16	84974461	85011535	-	3152	1	ZDHHC7|D	17.418	19.103
NM_0018	chr3	1.49E+08	1.49E+08	+	1447	1	CPB1|CPB	0.134	0.142
NM_0011(	chr8	1.34E+08	1.35E+08	-	4517	1	ZFAT|AITD	2.589	3.639

NR_10502	chr5	97504696	97671051	+	725	1	LINC0134C	0.32	0.132
NM_00128	chr21	7769588	7770548	-	959	2	C21orf140	0	0
NM_0191(	chr19	43727997	43755036	-	5535	1	SMG9|C19	11.894	12.833
NM_0011	chr11	93820569	93850502	-	598	1	VSTM5|C1	0.086	0.023
NM_0012	chr1	1418420	1421444	-	1628	1	ANKRD65|	0.356	0.395
NM_0013	chr6	1.23E+08	1.23E+08	+	764	1	FABP7|B-F	99.688	541.677
NM_0002	chr17	35574795	35578637	-	2678	1	PEX12|PAI	3.563	3.56
NR_04673	chr3	1.12E+08	1.12E+08	-	959	1	PLCXD2-AS	0.067	0.014
NM_1450	chr2	26401912	26456711	+	2476	1	DRC1|C2o	1.849	1.14
NR_10346	chr10	70298970	70382650	-	2806	1	LRRC20|-|	28.075	34.121
NR_11062	chr20	49040463	49046056	-	343	1	CSE1L-AS1	0.188	0.24
NM_0160	chr17	759309	782331	-	1826	1	GLOD4|C1	65.181	76.129
NM_0010(	chr11	55572128	55573060	+	931	1	OR4C16|C	0	0
NM_1309(	chr2	2.01E+08	2.01E+08	-	1157	1	PPIL3|CYP	16.304	16.964
NM_0010	chr10	81875314	82987179	+	3681	1	NRG3|HR(	10.505	10.527
NM_0217	chr16	27427258	27452042	+	4485	1	IL21R|CD3	0.144	0.061
NM_0012	chr13	32400723	32428178	-	2033	1	N4BP2L1|	0.178	0.472
NR_13499	chr12	1.19E+08	1.19E+08	-	445	1	LOC10537	0	0
NM_1783	chr1	1.53E+08	1.53E+08	+	1136	1	LCE1B|LEF	0	0.012
NM_0048	chr2	1.74E+08	1.74E+08	-	1781	1	CIR1|CIR|-	6.443	7.737
NM_0028(	chr6_GL0C	4266360	4272052	+	1039.1	8	PSMB9|LN	0	0
NR_04557	chr19	18434388	18438162	-	2082	1	ISYNA1|IN	88.186	77.939
NM_0016	chr2	1.48E+08	1.48E+08	+	5240	1	ACVR2A|A	6.717	9.805
NM_0069	chr17	7588180	7590170	-	1390	1	SOX15|SO	1.401	1.646
NM_0010(	chr7	1.51E+08	1.51E+08	-	1656	1	SMARCD3	80.74	120.668
NM_1457	chr12	16347142	16364410	+	896	1	MGST1|GS	12.447	4.647
NR_02462	chr10	1022637	1044201	+	922	1	IDI2-AS1|(	7.286	6.596
NR_03169	chr15	43793659	43793759	-	100	1	MIR1282|	324.142	218.056
NR_00128	chr19	49497719	49497805	+	86	1	SNORD35E	10.345	12.264
NM_0148	chr1	2.12E+08	2.12E+08	-	7772	1	LPGAT1|F	4.615	6.091
NM_0251	chr1	1.85E+08	1.85E+08	-	6610	1	EDEM3|C1	2.614	2.851
NM_0052	chr6	1.1E+08	1.1E+08	+	1835	1	GPR6|-|-|	0	0.052
NM_0150	chr6	83067666	83168471	+	7921	1	DOPEY1|D	4.704	6.478
NM_0068	chr22	36758202	36776133	-	1084	1	IFT27|BBS	38.026	32.221
NM_0176	chr17	60677811	61392838	+	3538	1	BCAS3|GA	10.445	11.982
NR_03622	chr2	10192614	10192671	-	57	1	MIR4261|	0	0
NM_0012	chr4	88090264	88231322	-	4258	1	ABCG2|AB	0.006	0.003
NM_0321	chr1	85157673	85201045	-	4702	1	SYDE2|-|-	0.08	0.047
NM_0248	chr10	1.02E+08	1.02E+08	-	1465	1	C10orf95|	0.484	0.683
NM_0322	chr13	85792787	85799348	-	4177	1	SLITRK6|D	2.241	2.915
NR_11059	chr2	10449728	10455552	+	877	1	LOC10192	0.72	1.078
NM_0204	chr6	87322588	87342328	+	2411	1	SMIM8|C6	4.85	5.408
NM_0013(	chr16	47154388	47461274	-	3046	1	ITFG1|231	25.482	31.387
NR_04746	chr3	87089280	87098702	+	4298	1	LINC0050€	0.006	0
NM_0204(	chr6	5108419	5260950	-	1492	1	LYRM4|C6	25.493	24.925
NM_0207	chr12	27780254	27803040	+	6464	1	KLHL42|Ct	9.423	8.978
NR_00335	chr15	25189414	25189495	+	81	4	SNORD115	0	0
NM_0163	chr9	1.21E+08	1.21E+08	-	4353	1	RAB14|FBI	23.909	25.374
NM_0032	chr19	10289979	10296778	+	2989	1	ICAM5|TL(	2.429	4.711
NM_0021	chr9	5231419	5233967	+	611	1	INSL4|EPIL	0	0

NM_0321	chr1	1.79E+08	1.79E+08	+	1017	1	TEX35|C1o	0	0.013
NM_0309	chr16	88803778	88809258	+	2663	1	CDT1|DUP	4.154	2.412
NM_0323	chr3	49422333	49429324	-	3219	1	NICN1|-|-	15.369	14.995
NM_0011	chr5	1.77E+08	1.77E+08	+	1303	1	PRR7|-|-|	11.102	10.281
NR_03030	chr5	32394378	32394475	-	97	1	MIR579|M	0	0.141
NM_0321	chr2	1.3E+08	1.3E+08	+	3074	1	RAB6C|W	0.075	0.062
NM_0230	chr17	48908369	48929060	+	3117	1	UBE2Z|HC	48.831	56.55
NM_0013	chr7	30911694	30925517	+	2689	1	AQP1|AQP	1.165	0.896
NM_0008	chr7	1.26E+08	1.27E+08	-	3547	1	GRM8|GLU	1.323	0.823
NR_13072	chr7	56809124	56812736	-	3612	1	LOC40135	0	0
NM_1986	chr21	44657932	44658341	+	407	1	KRTAP12-3	0	0
NM_0010	chr15	62163538	62165283	-	1572	1	C2CD4B|F.	0	0
NM_0012	chr3	1.23E+08	1.23E+08	-	4554	1	SEMA5B|S	17.984	26.808
NM_1732	chr2	1.13E+08	1.13E+08	+	645	1	IL37|FIL1|	0.08	0
NR_12046	chr12	1.17E+08	1.17E+08	+	462	1	TESC-AS1|	0	0
NM_0011	chr6	1.53E+08	1.53E+08	-	2264	1	FBXO5|EM	3.03	4.223
NM_0058	chr9	36169392	36171334	+	1940	1	CCIN|BTBD	0.073	0.035
NR_13573	chr15	99807023	99882982	+	552	1	LOC40046	0.327	0.248
NM_0010	chr12	49623414	49644669	+	3305	1	PRPF40B|I	19.483	25.678
NM_0012	chr13	23579360	23676105	+	4171	1	TNFRSF19	10.451	17.385
NM_0005	chr11	5225466	5227071	-	621	1	HBB|CD11	0	0
NM_0228	chr2	60756230	60802086	+	7362	1	PAPOLG|-	2.063	2.698
NR_02443	chr17	45247925	45268630	+	914	1	MAP3K14-	2.835	1.753
NM_0012	chr22	18527802	18530573	+	1323	1	TMEM191	0.058	0.021
NM_0012	chr10	1E+08	1E+08	-	3055	1	PKD2L1|P	0.27	0.076
NM_0158	chr19	4007598	4039386	+	3194	1	PIAS4|PIA	16.284	15.631
NM_0312	chr13	51861948	51866236	+	1183	1	CCDC70|-|	0	0.035
NR_04003	chr19	49838646	49851676	-	2144	1	PTOV1-AS	6.507	6.088
NM_0012	chr15	33310962	33866103	+	15454	1	RYR3|RYR	1.065	1.056
NM_0014	chr19	7958573	8005645	-	6050	1	ELAVL1|EL	40.364	37.897
NM_0044	chr5	73446256	73448527	-	2269	1	FOXD1|FK	0.284	0.447
NM_0013	chr19	18340587	18369952	+	540	1	PGPEP1|P.	3.94	6.417
NM_0163	chr3	1.87E+08	1.87E+08	+	1679	1	DNAJB11|	48.948	46.084
NM_0013	chr15	73684731	73714518	+	2726	1	CD276|4Ig	56.36	49.507
NM_0010	chr10	45302654	45315608	-	2072	2	OR13A1|-	0	0
NR_10680	chr11	64902387	64902455	-	68	1	MIR6749|	0.379	0.403
NM_0144	chr20	50958814	50961862	+	3046	1	MOCS3|U	5.41	5.549
NM_0013	chr9	26903450	26947473	-	4793	1	PLAA|DOA	7.72	8.599
NM_0062	chr19	10452957	10469631	+	4249	1	PDE4A|DP	6.13	9.374
NR_03741	chr20	45705102	45705180	-	78	1	MIR3617|	0	0
NM_0010	chr1	34865436	34929585	-	3842	1	DLGAP3|D	7.487	7.023
NM_0010	chr4	1.82E+08	1.83E+08	+	10864	1	TENM3|M	14.092	9.4
NM_0333	chr12	18738111	18739188	+	1075	1	CAPZA3|C.	0	0
NM_0011	chr12	1.33E+08	1.33E+08	-	2987	1	CHFR|RNF	20.063	16.701
NR_04028	chr1	19265982	19274074	-	2112	1	AKR7L|AF	0.464	0.765
NM_0209	chr19	7595902	7618310	+	4085	1	CAMSAP3|	25.402	37.054
NM_0013	chr1	13073678	13077884	+	1780	4	PRAMEF26	0	0
NM_0010	chr1	2528745	2530245	-	1301	2	HES5|bHLI	0	0
NM_0011	chr3	1.91E+08	1.91E+08	-	3617	1	GMNC|GE	0.057	0.004
NR_10460	chr17	12671862	12706135	-	1140	1	LOC10192	0	0

NR_04980	chr15	89883931	89884030	-	99	1	MIR5009|-	0	0
NM_0053(	chr22	35606764	35617337	-	1073	1	MB|PVALE	0.12	0.051
NM_0011:	chr8	94925972	94949387	-	5645	1	TP53INP1|	8.883	11.358
NM_0012!	chr22	32059170	32113024	+	4758	1	SLC5A1|D:	0.027	0.017
NM_0314:	chr1	1.52E+08	1.52E+08	-	1639	1	MRPL9|L9	33.646	26.776
NR_10682	chr14	1.05E+08	1.05E+08	-	86	1	MIR6765|I	3.148	2.548
NM_1736!	chr3	1.43E+08	1.44E+08	-	3609	1	SLC9A9|AU	0.593	1.332
NR_04687	chr3	1.15E+08	1.15E+08	+	673	1	ZBTB20-AS	0.096	0.081
NM_0122!	chr2	1.71E+08	1.71E+08	-	5707	1	TLK1|PKU-	2.223	3.223
NR_02665	chr22	19022624	19023550	+	926	1	DGCR10|D	0.501	0.666
NM_1524:	chr19	53014091	53016580	+	2487	1	ERVV-1|EN	0	0
NR_02745	chrX	1.5E+08	1.5E+08	-	2341	1	LINC00893	0.804	0.591
NR_04499	chr13	1.14E+08	1.14E+08	+	1940	1	GAS6-AS2|	1.356	1.673
NR_03386	chr16	32289547	32289981	-	434	2	LOC39070.	0	0
NM_0042(	chr4	67820876	67883998	-	2771	1	TMPRSS11	0	0
NR_00335	chr15	25189414	25189495	+	81	4	SNORD115	0	0
NM_1449{	chr1	94982723	95072951	-	1051	1	ALG14|CM	7.079	6.855
NM_0010!	chr19	57487711	57494680	+	2220	1	ZNF419|ZA	6.952	11.056
NM_0012{	chr1	12063377	12144207	+	3579	1	TNFRSF8|(	0.054	0.011
NM_0011(	chr15	93043406	93073160	-	3055	1	RGMA|RG	55.672	86.338
NR_00003	chr5	1.05E+08	1.05E+08	+	624	1	RAB9BP1|	0	0
NM_0011(	chr14	68874123	68979366	-	3702	1	ACTN1|BD	13.677	15.532
NM_0067(	chr1	87328468	87348923	+	5398	1	LMO4|-|-|	6.215	28.911
NM_0005{	chr5	1.32E+08	1.32E+08	+	917	1	IL3|IL-3|M	0	0
NR_00292	chr11	93732361	93732499	-	138	1	SNORA8|A	1.495	2.878
NR_03041	chr21	35720715	35720808	+	93	1	MIR802|M	0	0
NR_10467	chr5	1.04E+08	1.04E+08	-	427	1	LINC02115	0.06	0.032
NM_0012:	chr19	12945814	12953643	+	1646	1	RAD23A|H	55.122	54.206
NM_0026!	chr1	38043851	38046778	-	2925	1	POU3F1|C	22.358	23.812
NM_0010(	chr8	17497088	17570568	+	7557	1	SLC7A2|AT	0.014	0.087
NM_0049:	chr16	89171755	89195492	+	2843	1	CDH15|CD	1.596	0.843
NM_0010(	chr1	13749381	13788079	+	6191	1	PRDM2|HU	13.979	13.25
NM_0012!	chr12_KI2:	79667	366563	-	1044	3	PRH1|Db-s	0.593	0.669
NR_03962	chr1	2.37E+08	2.37E+08	+	72	1	MIR4428|-	0	0
NR_02726	chr15	72376113	72378788	+	2675	1	HEXA-AS1|	0.193	0.164
NM_1450-	chr3	44729606	44737083	+	3084	1	ZNF501|ZN	2.333	3.513
NM_0029:	chr3	18345388	18425337	-	7796	1	SATB1|-|-	8.163	5.41
NM_0010!	chr18	904943	912172	+	3180	1	ADCYAP1|	14.361	1.176
NM_0189:	chr5	1.41E+08	1.42E+08	+	4596	1	PCDHGB2|	21.049	82.492
NR_10465	chr5	43014729	43067439	-	632	1	LOC64898:	4.039	5.158
NR_00296	chr16	30710537	30710665	+	128	1	SNORA30|	0	0
NM_0029(	chr11	32090931	32105726	+	2508	1	RCN1|HEL	41.307	22.746
NR_12590	chr4	75980790	76005941	+	1681	1	LOC10192:	0	0.016
NM_1308:	chr15	25337249	25405506	-	4479	1	UBE3A|AN	11.961	16.984
NM_0010:	chr7	1.03E+08	1.03E+08	-	5446	1	RASA4|CA	0.462	0.417
NM_0010(	chr11	58227882	58228918	-	1034	1	OR10Q1|C	0	0.053
NR_02729	chr3	14145148	14178672	-	3581	1	XPC|RAD4	16.087	12.779
NM_0014:	chr4	74365143	74388760	+	4621	1	EREG|EPR	0	0.006
NM_0008(	chr6	77460848	77464022	-	3172	1	HTR1B|5-H	0.476	0.155
NR_04025	chr21	23361104	23384835	-	1694	1	D21S2088I	0	0.024

NR_12538	chr12	46383676	46652579	+	542	1	LOC10028	2.26	0.935
NM_0013	chr1	37474518	37484377	+	2705	1	ZC3H12A|	0.238	0.152
NM_0013	chrX	55908285	56291530	+	8450	1	KLF8|BKLF	0.467	1.067
NM_1389	chr21	33070144	33072422	+	2276	1	OLIG1|BHI	2.663	3.364
NM_0011	chr19	7056205	7058634	-	769	1	MBD3L3|-	0	0
NM_0011	chr1	51102234	51148082	+	904	1	C1orf185|	0	0
NM_0152	chr3	1.32E+08	1.33E+08	+	7489	1	DNAJC13|	2.963	5.185
NM_0011	chr5	1.51E+08	1.51E+08	-	2842	1	ANXA6|AN	40.658	42.093
NR_13529	chr22	21991099	22043934	-	3032	1	PRAMENP	0	0
NM_0011	chr9	71683366	71768884	-	6309	1	TMEM2|-|	12.585	9.639
NM_0012	chr1	1.21E+08	1.21E+08	+	7084	1	SRGAP2C|	0.353	0.553
NM_0154	chr2	73073382	73113018	-	4335	1	RAB11FIP5	4.527	7.722
NM_0010	chr19	56662652	56671755	-	2770	1	ZNF835|B	2.77	3.412
NR_03997	chr8	66192114	66197319	+	1500	1	LINC00967	0	0
NR_03037	chr19	52281797	52281890	+	93	1	MIR643|M	0	0
NM_1522	chr1	1.56E+08	1.56E+08	+	5274	1	SYT11|SYT	111.78	155.573
NR_07345	chr14	36070427	36165288	+	580	1	LINC0060	0	0.047
NM_0013	chr19	54107013	54115074	-	803	10	TFPT|FB1|	0	0
NM_0142	chr11	61789130	61792613	-	405	1	TMEM258	202.635	160.306
NM_0206	chr14	63204384	63293512	+	3612	1	RHOJ|ARH	0.925	1.27
NM_0011	chr3	1.13E+08	1.13E+08	-	10222	1	CFAP44|W	1.329	2.31
NM_0006	chr1	99850084	99924023	+	7335	1	AGL|GDE|	1.591	2.377
NM_0054	chr1	71063291	71081289	-	3132	1	ZRANB2|Z	11.992	11.41
NM_0005	chr11	5248272	5249857	-	579	1	HBG1|HB	0	0
NM_0068	chr19_GL9	270735	275150	-	1599	4	LILRA3|CD	0	0
NM_0006	chr20	34280265	34303511	-	2297	1	AHCY|SAH	55.576	47.46
NM_0010	chr5	1.68E+08	1.68E+08	+	9046	1	TENM2|O	24.072	10.852
NM_0012	chr6	34417454	34426125	-	641	1	RPS10|DB	1.529	0.919
NM_0010	chr6	87407972	87464473	+	2200	1	CFAP206|	1.6	0.728
NM_1524	chr15	59438173	59523552	+	3457	1	FAM81A|-	0.944	0.586
NM_1446	chr1	1.55E+08	1.55E+08	-	2402	1	DCST2|-|-	0.424	0.319
NM_0010	chr14	89156172	89619150	-	7843	1	FOXN3|C1	8.026	11.249
NM_0165	chrX	12867083	12890361	+	4987	1	TLR7|TLR7	0	0
NR_12062	chr10	1.01E+08	1.01E+08	-	2219	1	LOC10192	0	0.012
NM_0012	chr16_KI2	1021560	1036245	+	1037	4	NPIPA3|-|	0.075	0.04
NM_0151	chr22	43166622	43187131	-	3367	1	TTLL12|dJ	23.413	28.997
NR_13305	chr1	42776666	42784895	-	490	1	LOC10537	3.237	2.851
NM_0319	chr7	74174376	74197099	+	2478	1	EIF4H|WB	54.066	58.956
NM_0073	chr7	23181827	23201011	+	1788	1	NUPL2|CG	6.339	8.587
NM_0011	chr3	1.23E+08	1.23E+08	-	3045	1	PARP9|AR	0.047	0.441
NR_03788	chr2	3957656	3974032	-	2412	1	LINC01304	0	0
NM_1384	chr16	28553928	28591790	+	1139	1	SGF29|CC	17.399	16.848
NM_0013	chr13	1.13E+08	1.13E+08	+	1395	1	F10|FX|FX	1.303	0.54
NM_0042	chr5	892854	918049	+	2375	1	TRIP13|16	4.653	2.861
NM_0010	chr10	17752261	17800867	+	5448	1	TMEM236	0.007	0.018
NM_0002	chr4	54657928	54740715	+	5153	1	KIT|C-Kit|	8.935	16.578
NM_0010	chr19	52453576	52516878	+	6733	1	ZNF578|-|	0.906	2.154
NM_0011	chr2	1.66E+08	1.66E+08	-	8021	1	SCN1A|EIE	0.567	1.38
NM_0002	chr7	74773962	74789376	+	1446	1	NCF1|NCF	0.018	0.019
NR_02437	chr9	1.13E+08	1.13E+08	-	5803	1	FAM225B|	0	0

NR_00336	chr16	29074842	29116717	+	2379	1	RRN3P2|-|	0.027	0.017
NM_0178	chr1	28553017	28578545	+	1856	1	TRNAU1AF	25.592	20.597
NM_0012	chr3	50292828	50299468	-	1084	1	HYAL3|HY	5.995	5.307
NM_0012	chr9	86220265	86256033	-	1856	1	C9orf153|	0.042	0.007
NR_11009	chr12	99093359	99105011	+	1033	1	LOC10192	0	0
NM_0047	chrX	1.46E+08	1.46E+08	+	2440	1	TMEM257	0	0.056
NM_0153	chr11	6599914	6603650	-	1742	1	RRP8|KIA	8.119	8.964
NR_03963	chr2	64525513	64525565	+	52	1	MIR4434|	0	0
NM_0056	chr22_KI2	131494	137393	+	1207	2	DGCR6|-|-	0.748	0.704
NR_04601	chr1	11874	14409	+	1649	1	DDX11L1|	0	0
NM_0065	chr8	1.35E+08	1.36E+08	+	1957	1	KHDRBS3|	6.707	16.266
NM_1304	chr15	40470961	40473158	+	2195	1	CHST14|A	5.339	4.299
NR_10414	chr3	1.82E+08	1.82E+08	+	9549	1	LINC0120	0	0.007
NR_03607	chr2	1.77E+08	1.77E+08	-	65	1	MIR3128|	0	0
NM_1779	chr3	1.8E+08	1.8E+08	+	1738	1	ACTL6A|A	42.286	38.75
NM_0166	chrX	1.02E+08	1.02E+08	+	2127	1	ARMCX1|	31.497	29.757
NR_00222	chr3	13937307	14065981	+	2257	1	TPRXL|TP	0.011	0.018
NM_0145	chr5	1.79E+08	1.79E+08	+	5844	1	ZNF354C|	3.424	5.358
NM_0036	chr15	65195999	65211502	-	4457	1	CILP|CILP-	0.457	0.495
NM_0011	chr5	64690308	64718190	+	962	1	FAM159B|	0.067	0
NM_1390	chrX	25003696	25015948	-	2867	1	ARX|CT12	0.126	7.534
NR_10274	chr5	38400451	38403471	-	1702	1	EGFLAM-A	0	0
NR_03792	chr17	7561992	7579006	+	4281	1	SENP3-EIF	140.56	140.032
NM_0010	chr1	2.48E+08	2.48E+08	+	955	3	OR2T3|-|-	0	0
NR_03823	chr7	1.56E+08	1.56E+08	-	2695	1	LOC28588	0	0.081
NM_1994	chr1	3069178	3438621	+	8647	1	PRDM16|	0.333	2.099
NM_0010	chr11	1.12E+08	1.12E+08	-	6120	1	ALG9|CDG	11.539	10.978
NM_0016	chr10	88935074	88952823	-	1388	1	ACTA2|AA	13.423	41.604
NM_0010	chr15	39934124	40035596	+	5493	1	EIF2AK4|G	10.539	9.089
NM_2073	chr17	16442005	16492191	-	2698	1	LRRC75A|	10.533	10.362
NM_0011	chr22	18110687	18131731	+	2088	1	TUBA8|TU	1.21	2.132
NR_02700	chr14	93904730	93926372	-	2391	2	FAM181A-	0	0
NM_0012	chr18	35251058	35290245	-	3435	1	ZSCAN30|	8.153	10.87
NM_0331	chr12	42312086	42326130	-	1834	1	ZCRB1|MA	18.201	16.528
NM_0161	chr20	1442162	1467692	-	3557	1	NSFL1C|P	44.223	46.601
NM_0013	chr1	2.39E+08	2.4E+08	+	9035	1	CHRM3|E	0.537	1.228
NR_04659	chr1	97796921	97798051	+	814	1	DPYD-AS2	0	0
NR_02966	chr3	1.6E+08	1.6E+08	+	97	1	MIR15B|M	0	0
NR_12603	chr2	31867810	32011052	-	1268	1	MEMO1|C	3.498	3.575
NM_0149	chr3	1.08E+08	1.09E+08	-	7030	1	MYH15|-|	0.497	0.314
NR_03059	chr14	1.01E+08	1.01E+08	+	78	1	MIR889|M	0	0
NM_1826	chr6	1.17E+08	1.17E+08	+	2225	1	VGLL2|VG	0	0
NR_02455	chr17_GLC	892701	944826	-	838.3	3	MAPT-AS1	0	0
NM_0052	chr8	52939908	52940894	+	985	1	NPBWR1|	0	0.07
NM_0011	chr15	41838813	41848148	+	2713	1	PLA2G4B|	7.647	6.462
NM_0016	chr17	43398985	43401136	+	1581	1	ARL4D|AR	26.986	30.894
NM_0011	chr17	6451263	6556557	-	7022	1	PITPNM3|	2.908	6.228
NR_03165	chr4	1.01E+08	1.01E+08	-	112	1	MIR1255A	0	0
NM_0024	chr9	97633423	97673747	+	5355	1	NCBP1|CB	10.977	12.613
NM_0011	chr19	48047843	48110852	-	2474	1	PLA2G4C|	2.064	5.127

NR_00332	chr15	25080141	25080234	+	93	1	SNORD116	0	0.589
NR_13493	chr3	1.69E+08	1.69E+08	+	530	1	LOC10537	0	0
NR_13449	chr10	47413389	47414475	-	661	1	LOC10537	0	0
NM_00378	chr6_GL00	4471560	4473245	+	1683	5	B3GALT4|I	0	0
NM_1383!	chr19	13952474	13961449	+	2281	1	DCAF15|C	33.469	24.29
NR_03160	chr7	36919357	36919432	-	75	1	MIR1200|I	0	0
NM_0806	chr5	1.41E+08	1.41E+08	+	2928	1	SLC35A4|-	21.586	19.802
NM_0327	chr10	1.03E+08	1.03E+08	+	3226	1	INA|NEF5|	275.551	217.36
NM_1781	chr22	42583721	42614962	-	3344	1	POLDIP3|F	65.532	84.324
NM_2068	chr13	99606664	99892764	+	1390	1	CLYBL|CLB	13.534	4.217
NM_1781	chrX	1462573	1537144	-	4230	2	P2RY8|P2Y	0	0
NM_0013	chr12	1.13E+08	1.13E+08	-	3642	1	RASAL1|R/	0.127	0.271
NM_1827	chr3	46830404	46834095	-	876	1	PRSS42|TE	0	0
NR_10990	chr5	1.81E+08	1.81E+08	+	1010	1	CTC-338M	4.596	3.58
NM_1450	chr6_GL00	2126761	2132924	+	1027	7	C6orf136|	0	0
NR_04650	chr1	20642657	20652193	-	4440	1	PINK1-AS|	22.427	28.517
NR_11093	chr16	87212115	87226429	+	1450	1	LOC10192	0	0
NM_0003	chr14	64766399	64823148	-	6693	1	SPTB|EL3|	0.281	0.872
NM_0010	chr14	23469689	23478193	+	1129	1	NGDN|C14	41.924	45.519
NR_04647	chr1	27098809	27155130	-	4270	1	SLC9A1|AF	6.755	6.245
NR_03020	chr19	53713347	53713434	+	87	1	MIR519D|	0	0
NM_0133	chr3	1.51E+08	1.51E+08	-	1790	1	GPR171|H	0	0
NM_1738!	chr1	1.55E+08	1.55E+08	-	540	1	KRTCAP2|I	97.752	64.782
NM_0011	chr17	9771434	9791297	-	995	1	DHRS7C|S	0	0
NM_0013	chr3	50279027	50288114	+	1485	1	LSMEM2|(	2.127	1.642
NR_03318	chrX	72181676	72198344	+	1247	1	PIN4|EPVH	22.106	17.915
NR_11098	chr1	2.06E+08	2.06E+08	+	2372	1	LOC10302	0.174	0.549
NM_0200	chr9	1.33E+08	1.33E+08	+	1902	1	BARHL1|-|	17.517	3.601
NM_0009	chr5	40679930	40693736	+	3412	1	PTGER4|EI	0.011	0.024
NR_11015	chr3	64561346	64590084	+	3689	1	ADAMTS9-	0.133	0.23
NM_0011	chr1	1.6E+08	1.6E+08	-	4021	1	IGSF9|FP1	14.554	12.185
NM_0249	chr10	15778169	15860533	-	2378	1	FAM188A|	19.91	15.667
NM_0010	chr7	19695462	19709037	-	3886	1	TWISTNB|	0.989	1.142
NM_0011	chr2	46479565	46484425	+	676	1	TMEM247	0	0.02
NR_04746	chr5	1.03E+08	1.03E+08	+	589	1	LINC00492	0	0
NR_00232	chr3	39408391	39408539	+	148	1	SNORA6|A	2.265	3.424
NM_0529	chr1	1.58E+08	1.58E+08	-	3085	1	FCRL1|CD	0	0
NM_0012	chr7	1.29E+08	1.29E+08	+	1717	1	CCDC136|	26.891	22.791
NM_0011	chr8	73420369	73746927	-	2910	1	STAU2|39I	20.492	27.559
NM_0319	chr5	1.6E+08	1.6E+08	-	2392	1	C1QTNF2|	0.733	1.105
NR_12578	chr1	1.93E+08	1.93E+08	+	775	1	LINC01031	0	0
NR_12536	chr7	1.31E+08	1.31E+08	-	2040	1	MKLN1-AS	0.689	0.752
NR_10434	chr17	18411159	18414388	+	1877	1	LINC0207E	0.185	0.175
NR_03393	chr17	47450227	47492620	-	1390	1	MRPL45P2	0.473	0.522
NM_0012	chr11	46617276	46676018	+	5849	1	ATG13|KI/	25.818	27.902
NR_03017	chr14	1.01E+08	1.01E+08	+	88	1	MIR493|M	0	0
NM_0012	chr12	52205581	52210855	-	1286	1	C12orf80|	0	0
NR_03074	chr5	88666853	88666939	-	86	1	MIR9-2|M	14.093	35.994
NM_0003	chr16	8797813	8849337	+	2313	1	PMM2|CD	9.081	8.906
NM_0011	chr7	1.5E+08	1.5E+08	-	1271	1	ACTR3C|A	0	0.043

NM_0011	chrX	1.46E+08	1.46E+08	+	3917	1	SLITRK2|C	0.086	3.829
NR_03792	chr17	3662893	3696404	-	6270	1	P2RX5-TA	17.274	14.901
NM_0013	chr2	2.33E+08	2.33E+08	+	2853	1	C2orf82|A	0.538	0.797
NR_13277	chr3	85062153	85062264	-	111	1	SNORA95|	0	0
NR_12046	chr12	15001833	15006683	+	601	1	LINC0148	0.043	0.046
NR_04664	chr3	1.28E+08	1.28E+08	+	586	1	RUVBL1-A	0.066	0.187
NM_0159	chr7	43926436	43956136	+	3973	1	UBE2D4|H	6.432	9.336
NM_0051	chr2	36552261	36598189	-	2001	1	FEZ2|HUM	20.458	21.172
NM_0013	chr5	1.7E+08	1.7E+08	-	5949	1	FAM196B|	0.912	0.958
NM_0036	chr10	15513949	15720335	-	6933	1	ITGA8|-|-|	0.065	0.034
NR_03064	chr4	1.11E+08	1.11E+08	-	65	1	MIR297|M	0	0
NM_1828	chr21	41175231	41185239	-	10006	1	PLAC4|C2	0	0.001
NM_0251	chrX	30558824	30577916	-	1922	1	CXorf21|-|	0	0
NM_0132	chr5	1.77E+08	1.77E+08	+	1266	1	PRELID1|C	34.383	32.576
NM_0153	chr1	55066359	55215366	-	10732	1	USP24|-|-	7.938	9.293
NM_0011	chr19	7763149	7769605	+	1519	1	CLEC4M|C	0	0
NM_0155	chr15	41517176	41544277	-	4656	1	RPAP1|-|-	11.365	11.302
NR_03786	chr2	69893943	69905236	+	1345	1	SNRNP27|	25.566	27.659
NR_11082	chr3	99816632	99823865	-	2015	1	HP09053|	1.35	1.305
NM_0072	chr1	1.09E+08	1.09E+08	+	2482	1	STXBP3|M	2.764	3.653
NM_0039	chr17	4771892	4783211	+	701	1	TM4SF5|-|	0.037	0
NM_0132	chr3	51391268	51397905	+	6635	1	RBM15B|H	39.732	40.414
NM_0203	chr3	1.71E+08	1.71E+08	-	5530	1	EIF5A2|EIF	0.252	0.379
NM_0010	chr1	1.57E+08	1.57E+08	+	4841	1	PEAR1|JED	0.011	0.037
NM_0011	chr9	81928428	81934998	+	4917	1	SPATA31D	0	0
NM_0228	chr16	87392195	87404774	+	2282	1	MAP1LC3E	84.593	93.154
NM_1735	chr10	61662961	61766333	+	1243	1	C10orf107	0.322	0.154
NR_00299	chr7	45105968	45106099	-	131	1	SNORA5B|	0.492	0.523
NR_03994	chr2	1.6E+08	1.6E+08	-	72	1	MIR4785|	0	0
NM_0033	chr3	49805205	49813958	-	3287	1	UBA7|D8|	0.357	0.538
NM_0012	chr1	25272486	25330445	+	2673	1	RHD|CD24	0.068	0.082
NR_10381	chr21	45338590	45341990	-	453	1	LINC0031	0.085	0
NM_1722	chr21	34364024	34371141	+	799	1	KCNE2|AT	1.243	1.217
NM_0010	chr9	1.01E+08	1.01E+08	+	3092	1	MURC|CA	3.816	4.235
NM_0176	chr20	6074845	6123544	-	5134	1	FERMT1|C	0.005	0.061
NM_0249	chr8	1.01E+08	1.02E+08	+	5211	1	GRHL2|BC	0.022	0.008
NR_12593	chr4	1.03E+08	1.03E+08	+	1501	1	LOC10272	2.594	3.769
NM_0013	chr6	99398051	99425345	-	4823	1	PNISR|C6c	11.717	13.859
NR_10676	chr16_KI2	1269043	1269108	-	65	2	MIR6506|	0	0
NM_0010	chr16	88809469	88811934	-	666	1	APRT|AMI	34.15	31.384
NM_0029	chr12	21468910	21501669	-	3762	1	RECQL|RE	2.3	2.767
NM_1532	chr9	70043581	70226972	+	2928	2	MAMDC2|	0.185	0.094
NR_03388	chr18	64104091	64423592	+	1703	1	LINC01924	0	0
NM_1768	chr4	1.05E+08	1.05E+08	-	1659	1	PPA2|HSP	21.987	23.968
NR_12166	chr3	24151324	24189956	+	639	1	LOC10192	0	0.043
NR_02996	chr5	55170532	55170622	-	90	1	MIR449A|	0	0
NM_0571	chr4	1.39E+08	1.39E+08	+	5532	1	NAA15|Ga	3.084	4.09
NM_0011	chr9	1.21E+08	1.21E+08	-	3707	1	TRAF1|EBI	0.195	0.103
NM_0223	chr2	38293957	38377423	-	4414	1	ATL2|ARL3	4.995	5.691
NM_1721	chr1	40784012	40840452	+	3922	1	KCNQ4|DF	0.394	0.527

NM_0056:	chr16	527856	554636	+	4728	1	CAPN15|S	5.634	4.47
NM_0010:	chr3	1.39E+08	1.39E+08	-	1893	1	PRR23B|-|	0	0
NR_12067	chr10	74506500	74529324	-	510	1	LOC10272:	0	0.054
NR_00368	chr19	10109749	10109840	+	91	1	SNORD105	0	0
NM_0144	chr2	1.13E+08	1.13E+08	+	883	1	IL36A|FIL1	0	0
NM_0024:	chr11	1.03E+08	1.03E+08	-	1747	1	MMP10|S	0.635	1.913
NM_0005:	chrX	1.54E+08	1.54E+08	+	1990	3	OPN1MW	0	0
NR_02690	chr1	1.8E+08	1.8E+08	+	2715	1	FLJ23867|	2.075	2.724
NR_03034	chr12	12764649	12764743	+	94	1	MIR613|M	0	0
NM_0011	chr22	38203020	38216510	+	2218	1	MAFF|U-M	6.243	6.015
NR_03741	chr8	27701673	27701767	-	94	1	MIR3622B	0	0
NM_0230:	chr5	73552200	73565686	-	2187	1	ANKRA2|A	9.893	13.021
NM_0010:	chr3	1.22E+08	1.22E+08	-	2183	1	IQCB1|NPI	10.744	11.507
NM_0011:	chr1	1.5E+08	1.5E+08	-	3118	1	ANP32E|L/	9.126	8.571
NM_0012	chr20	45898620	45910994	-	1711	1	PLTP|BPIF	67.331	34.415
NR_11065	chr8	72732045	72751843	+	1719	1	LOC10192	0	0
NM_0012	chr1	2.03E+08	2.03E+08	-	1825	1	CHI3L1|AS	0.042	0.24
NM_0013	chr7	1.06E+08	1.06E+08	+	2833	1	RINT1|RIN	4.023	5.309
NM_0012(	chr10	1.02E+08	1.02E+08	-	849	1	FGF8|AIGF	0.121	0.403
NM_0010:	chr1	1.54E+08	1.54E+08	-	699	1	C1orf189|	0.535	0.235
NM_2019	chr4	1.39E+08	1.39E+08	-	3231	1	ELF2|EU32	8.149	9.699
NM_0010(	chr5	36103312	36151913	-	3217	1	LMBRD2|-	1.84	2.346
NM_0010	chr1	13410450	13421328	+	1591	1	PRAMEF20	0	0
NR_03988	chr17	38702262	38702331	-	69	2	MIR4734|	0	0
NM_0048	chr9	1.15E+08	1.15E+08	+	1606	1	ATP6V1G1	119.213	113.218
NM_0158	chr1	26169897	26170873	+	644	1	ZNF593|Z1	23.364	19.418
NR_03759	chr2	94868685	94947342	-	3320	1	LOC44202	0.004	0.008
NM_0038:	chr8	38996986	39105260	+	2896	2	ADAM9|C	4.946	10.377
NM_0010(	chr22	23786931	23834518	+	1697	2	SMARCB1|	0.008	0
NM_0007	chr15	78565520	78595269	+	3615	1	CHRNA5|L	4.426	4.172
NR_03655	chr17	39057019	39081451	+	2300	1	LOC10013	0.23	0.298
NM_0012	chr2	1.3E+08	1.3E+08	-	1345	1	CCDC74B|	5.157	2.169
NM_0058(	chr6_GL0C	2207446	2222591	-	1768.5	8	FLOT1|-|-	0	0
NM_0013(	chr19	2269486	2273488	+	1168	1	OAZ1|AZI|	436.745	407.134
NM_0325:	chr2	2.42E+08	2.42E+08	+	2640	1	BOK|BCL2	6.696	8.924
NM_0010:	chr5	1.77E+08	1.77E+08	-	527	1	PFN3|-|-|	0	0
NM_0012	chr3	48467798	48473333	-	2036	1	SHISA5|SC	120.892	109.778
NM_0059	chr1	11785730	11806103	-	7136	1	MTHFR|-|	15.468	13.937
NM_1481	chr8	1.03E+08	1.03E+08	-	4225	1	AZIN1|AZI	49.078	62.462
NM_0011	chr1	23801877	23825459	-	1386	1	HMGCL|HI	19.796	19.33
NM_0123:	chr8	10054269	10428891	+	1527	1	MSRA|PM	6.307	21.339
NM_0013:	chrX	1.35E+08	1.35E+08	-	2530	1	FAM122B|	2.339	3.248
NM_0054	chr21	42346358	42351099	-	701	1	TFF2|SML	0	0.039
NR_02827	chr11	65422798	65426532	+	3734	1	NEAT1|LIN	7.79	1.031
NM_0011	chr11	3217944	3218813	-	868	1	MRGPRG|	0	0
NM_2069	chr21	41316734	41357727	+	1231	1	FAM3B|2-	0	0
NM_0037	chr12	89519413	89524806	-	5391	1	GALNT4|G	0.653	0.587
NM_0010(	chr1	1.52E+08	1.52E+08	-	3598	1	TCHHL1|S	0	0
NR_02448	chr9	34830267	34838586	-	6126	1	FAM205BF	0	0
NM_0009	chr17	40309171	40357643	+	3273	1	RARA|NR1	13.54	16.459

NR_00484	chr20	26009799	26023267	+	3022	1	LOC10013	0	0.009
NM_0044	chr3	1.94E+08	1.94E+08	-	3489	1	GP5|CD42	0.007	0.016
NR_03391	chr20	4193090	4195953	+	679	1	LINC01433	0.076	0.02
NM_0206	chr8	74234700	74321361	-	4403	1	JPH1|CMT	0.146	0.336
NR_02405	chr19	56368121	56379827	+	3405	1	ZNF542P|	11.511	12.168
NR_10672	chr11	15969533	15969621	-	88	1	MIR6073|	0.733	1.556
NM_0057	chr12	42157105	42238368	-	4209	1	YAF2|-|-|	3.091	3.44
NR_12604	chr9	39873857	39875395	+	1422	2	LOC55424	0	0.058
NM_0250	chr15	74630558	74696045	-	3756	1	EDC3|LSM	27.675	33.083
NM_0164	chr2	96847987	96858019	-	892	1	ANKRD39|	14.715	9.52
NM_0012	chr16	77722492	77742260	+	954	1	NUDT7|-|-	3.771	5.011
NM_0013	chr19	639895	643703	+	615	1	FGF22|-|-	0.566	0.646
NR_11083	chr7	42661726	42706447	-	1168	1	LINC01448	0	0
NR_03614	chr17_KI2	61723	61806	-	83	3	MIR3183|	0	0
NM_0032	chr12	1.04E+08	1.04E+08	+	2842	1	HSP90B1|	74.285	69.155
NR_02667	chr7	20827298	20827820	+	522	1	RPL23P8|	8.151	7.347
NR_03997	chr22	37668026	38024093	+	66.6	5	MIR5096|	0	0
NR_10686	chr2	2.18E+08	2.18E+08	-	115	1	MIR6809|	0	0
NM_0043	chr19	12737459	12748324	+	1305	1	ASNA1|AR	63.835	64.906
NM_0305	chr17	63695889	63700159	-	3218	1	LIMD2|-|-	29.345	15.778
NM_0038	chr2	1.02E+08	1.02E+08	+	1950	1	IL1RL2|IL-	0	0
NM_0047	chr12	45919131	45990618	-	7579	1	SCAF11|C	2.727	3.273
NM_1335	chr12	91143277	91179618	-	4141	1	DCN|CSCD	1.152	1.029
NR_03751	chr7	30289794	30289890	-	96	1	MIR550B1	0	0
NR_10803	chr10	1.04E+08	1.04E+08	-	1083	1	CFAP58-AS	0.357	0.531
NM_0053	chr10	1.17E+08	1.17E+08	+	1467	1	PNLIPRP2|	0	0
NR_04666	chr3	1.98E+08	1.98E+08	-	542	1	LMLN-AS1	0.809	0.682
NR_03826	chr12	93565628	93571398	-	570	1	SOCS2-AS1	1.629	2.691
NM_0023	chr12	8940361	8949761	-	2557	1	M6PR|CD-	24.037	19.343
NR_11076	chr18	26422995	26438044	+	766	1	LINC01543	0	0
NR_02406	chrX	84934151	84934890	+	739	1	UBE2DNL|	0	0
NM_0189	chr5	1.41E+08	1.41E+08	+	5254	1	PCDHA3|P	16.95	16.226
NM_0330	chr1	1E+08	1E+08	+	2623	1	MFSD14A|	19.485	17.044
NM_0010	chr1	15764964	15786589	+	2772	1	FBLIM1|C	6.214	12.294
NM_0011	chr19	4360367	4400418	-	2186	1	SH3GL1|C	54.724	55.083
NM_0180	chr1	2.2E+08	2.2E+08	+	3524	1	IARS2|CAC	20.742	19.201
NM_0008	chr4	1.57E+08	1.57E+08	+	3046	1	GLRB|HKP	2.692	7.235
NM_0165	chr12	56338879	56340410	+	1036	1	IL23A|IL-2	0.249	0.344
NR_13366	chr11	64291302	64300028	+	1406	1	KCNK4|K2	0.798	0.487
NM_0012	chr14	71933038	72562754	+	1674	1	RGS6|GAP	1.117	1.972
NM_0024	chr11	1074875	1110508	+	10586	2	MUC2|ML	0.002	0
NR_12162	chr4	1.41E+08	1.41E+08	-	1021	1	LOC10050	0.025	0.013
NM_0011	chr13	36168208	36297855	-	2632	1	CCDC169-S	0.073	0.51
NR_04985	chr1	32086949	32087007	+	58	1	MIR5585|	0.445	0
NM_0051	chr18	9546791	9614607	-	3901	1	PPP4R1|M	26.527	34.929
NM_0011	chr5	1.57E+08	1.57E+08	-	1615	1	HAVCR1|C	0	0.025
NM_0010	chr1	34782310	34786366	+	1758	1	GJB3|CX3	0.029	0.023
NM_0010	chr8	7470308	7475082	-	277	3	DEFB104B	0	0
NM_0206	chr3	54918354	54928074	-	1301	1	LRTM1|HT	0.119	0.116
NR_03619	chr15	68801850	68801925	-	75	1	MIR4312|	0	0

NM_0241:	chr11	93735931	93741537	-	1302	1	TAF1D|JOS	4.06	7.217
NM_0011!	chr11	1.02E+08	1.02E+08	+	534	1	C11orf70|	1.932	0.564
NR_04609	chr11	75758455	75768647	-	1500	1	LOC28321	0	0
NM_0013:	chr12	54561444	54579239	+	2824	1	PDE1B|HE	7.643	13.925
NR_13672	chrY	12904858	12920478	+	4384	1	DDX3Y|DB	7.414	9.773
NM_0010{	chrX	1.21E+08	1.21E+08	-	1288.7	10	CT47A10|(	0	0
NM_0034:	chr16	31873758	31917308	+	3262	1	ZNF267|H	0.206	0.189
NM_0063(	chr12	53480492	53499660	-	3524	1	MAP3K12|	26.383	22.738
NR_04853	chr20	49812713	49892242	+	6129	1	SLC9A8|NI	5.015	5.549
NM_0011:	chr7	1.4E+08	1.4E+08	+	2350	1	TBXAS1|BI	0.137	0.221
NM_0010(	chrX	1.55E+08	1.55E+08	+	1114	3	F8A3|-|-|)	0.116	0.246
NM_0321:	chr4	1.63E+08	1.63E+08	-	2798	1	TKTL2|-|-|	0.097	0.098
NM_0144!	chr1	1.74E+08	1.74E+08	+	3451	1	KLHL20|KI	5.077	6.255
NM_0145!	chr17	75130225	75131795	-	950	1	NT5C|DNT	35.396	21.497
NR_03623	chr3	67225464	67225527	+	63	1	MIR4272|-	0	0
NM_0180:	chr1	2.37E+08	2.37E+08	-	8437	1	HEATR1|B	2.45	3.456
NM_0011(	chr11	1.17E+08	1.17E+08	-	1902	1	APOA5|AF	0.02	0.007
NM_0031(	chr4	24795463	24800845	+	1525	1	SOD3|EC-S	0.245	0.036
NR_03661	chr6	36594313	36604467	+	3593	1	SRSF3|SFR	44.131	51.243
NM_0013(	chr18	35989824	36067594	-	1998	1	RPRD1A|H	21.508	23.706
NR_02840	chr12	45725720	45727921	-	2201	1	LINC00938	11.376	12.577
NM_0250	chr19	52013324	52028427	-	4669	1	ZNF614|-|	1.925	3.201
NR_00278	chrX	27460211	27463341	-	3130	1	PPP4R3CP	0	0
NM_0072:	chr3	50324909	50328238	-	1679	1	TUSC2|C3(	42.989	41.67
NM_0011!	chr22	43110748	43129712	+	946	1	BIK|BIP1|I	0.927	0.724
NR_13428	chr5	1.67E+08	1.67E+08	-	628	1	LOC10192	0	0
NM_0160:	chr14	67676801	67695793	-	2558	1	RDH11|AR	40.062	43.913
NM_0011!	chr9	36214441	36258499	-	4943	1	GNE|DMR	12.612	12.835
NM_0009:	chr5	1.03E+08	1.03E+08	+	5310	1	PAM|PAL|	102.708	55.895
NR_13487	chr6_GL0C	1358044	1368247	-	1830.2	8	TRIM31|C(	0	0
NM_0011(	chr5	79976716	79991265	-	7854	1	MTX3|-|-|	1.515	2.135
NM_1829{	chr16	83953222	83966332	+	1932	1	OSGIN1|BI	0.587	0.978
NM_0010:	chr10	89378056	89385205	+	1968	1	IFIT1B|IFIT	0.026	0.021
NM_0010:	chr4	69200190	69214731	-	1841	1	UGT2B11|	0.007	0
NR_03987	chr17	31534883	31534971	+	88	1	MIR4724|	3.516	2.335
NM_1984	chr13	57140918	57143939	+	1842	5	PRR20A|P	0	0
NM_0164	chr22	23608452	23632321	-	1286	1	DRICH1|C	0.301	0.554
NM_0240:	chr7	27145583	27147774	-	798	1	HOXA6|HC	0	0.034
NR_02728	chr6	1.66E+08	1.66E+08	+	2064	1	LINC00602	2.561	1.573
NR_03620	chr17	77396984	77397054	-	70	1	MIR4316|-	0	0
NR_05201	chr7	92447448	92460067	+	4129	1	GATAD1|C	6.455	5.636
NM_0023!	chr12	68808172	68845544	+	7482	1	MDM2|AC	4.205	6.971
NM_0011:	chr17	75272980	75289449	-	1559	1	SLC25A19|	3.655	3.998
NM_0055(	chr9	1.05E+08	1.05E+08	-	10450	1	ABCA1|AB	1.04	1.598
NM_1730!	chr7	1.03E+08	1.04E+08	-	11499	1	RELN|ETL7	10.504	3.884
NM_1390:	chr11	69048882	69090604	+	4999	1	TPCN2|SH	2.027	2.129
NM_0012:	chr16	69838737	69941741	+	4204	1	WWP2|AII	19.491	21.898
NM_0149!	chr8	1.41E+08	1.41E+08	+	5418	1	DENND3|-	3.367	4.025
NR_04646	chr13	21298125	21392922	+	734	1	MIPEPP3|-	2.301	1.642
NM_0010(	chr16	1911464	1918323	-	1152	2	HS3ST6|3-	0	0

NR_12646	chr22	39475807	39476822	-	496	1	MGAT3-AS	0.858	0.994
NR_01542	chr9	68355164	68357866	-	584	1	PGM5-AS1	1.236	0
NR_02962	chr17	2050271	2050380	-	109	1	MIR212|M	0	0
NM_0182(	chr1	25430858	25500207	+	3964	1	TMEM57|	19.558	21.838
NM_1523	chr17	29593509	29614761	+	3156	1	ANKRD13E	34.807	43.465
NR_00273	chr20	2656939	2657010	+	71	1	SNORD57|	4.177	1.929
NM_1830	chr4	1.08E+08	1.08E+08	+	4745	1	CYP2U1|P	2.168	3.227
NM_1736	chr16	70174173	70186895	+	1808	1	CLEC18C|	0.043	0.068
NM_0012	chr8	49072341	49076083	+	1986	1	C8orf22|-|	0.026	0.014
NM_0221	chr14	93182196	93184904	-	2412	2	MOAP1|M	0	0
NM_0023	chr16	89917879	89920977	+	3096	1	MC1R|CM	7.205	7.127
NM_0010	chr7	71779491	72337223	-	9463	1	CALN1|CA	0.324	0.98
NM_0038	chr14	70452157	70459905	+	2660	1	ADAM21|	0.16	0.268
NM_0808	chr20	56358927	56368662	+	3028	1	FAM210B|	20.234	18.429
NM_0012	chr12	5562651	5945259	-	3674	1	ANO2|C12	0.797	0.611
NR_02200	chr15	25135642	25138053	+	2411	1	PWAR1|D	0.011	0.045
NR_03614	chr16	14901508	14901591	+	83	7	MIR3179-3	0	0
NR_04004	chr8	1.24E+08	1.24E+08	-	2309	1	FER1L6-AS	0	0
NR_01538	chr8	12766062	12811401	-	768	1	LOC34035	0	0.036
NR_03825	chr19	37551371	37587347	+	2448	1	ZNF571-AS	4.54	3.441
NM_0013	chr12	64781193	64881018	+	7627	1	TBC1D30|	2.259	4.996
NR_03895	chr22	36965248	36968172	+	988	1	LL22NC01-	0	0
NR_04751	chr4	1.03E+08	1.03E+08	-	1616	1	SLC9B1|NI	6.941	4.967
NM_0011	chr12	6789472	6820810	+	2803	1	CD4|CD4n	0.414	0.171
NM_0252	chr12	1.12E+08	1.12E+08	+	3991	1	ACAD10|-	7.789	7.08
NM_0012	chr12	6375368	6391571	+	2061	1	LTBR|CD1	0	0
NR_10413	chr17	70051271	70068095	+	1049	1	LINC01028	0	0
NM_0176	chr1	1.04E+08	1.04E+08	+	1868	1	RNPC3|RB	3.099	4.04
NR_13450	chr10	54486230	54656051	+	2294	1	LOC10537	0	0.006
NM_0038	chr8	23191457	23225167	-	1738	1	TNFRSF10	0.059	0.047
NM_1785	chr1	1.74E+08	1.74E+08	-	4397	1	SLC9C2|SL	0.032	0.059
NR_03826	chr10	43859411	43895433	+	1969	1	LINC0084C	0	0
NM_0010(	chr11	5580877	5581884	+	1006	1	OR52B6|C	0	0
NM_1458	chr19	51012744	51019698	-	2960	1	KLK10|NE	0.03	0.093
NM_0012	chr12	1.24E+08	1.24E+08	+	3323	1	GTF2H3|B	5.238	5.845
NM_1993	chr16	77282128	77435114	-	5888	1	ADAMTS18	1.732	0.119
NM_0010	chr6	3153666	3157549	-	1615	1	TUBB2A|C	195.925	204.158
NR_03399	chr4	1.83E+08	1.84E+08	-	778	1	LOC38924	0.331	0.37
NR_10422	chrX	1.41E+08	1.41E+08	+	765	1	LINC00632	1.753	1.003
NR_12050	chr2	28148556	28206961	-	2254	1	LOC10050	0	0.006
NM_0806	chr20	32010442	32032181	+	2521	1	CCM2L|C2	0.823	0.511
NM_0012	chr19	40665906	40690164	-	3397	1	NUMBL|C	23.658	20.705
NM_0309(	chr19	14880426	14881355	-	928	1	OR7A17|B	0	0
NR_04625	chr3	14942779	14947893	-	3529	1	FGD5-AS1	58.081	52.871
NM_0227	chr11	1.12E+08	1.12E+08	+	977	1	C11orf1|-|	22.052	19.038
NM_0011(	chr4	1.45E+08	1.45E+08	-	7045	1	OTUD4|DU	2.09	2.283
NM_0012	chr6	3268974	3445013	-	5671	1	SLC22A23|	41.668	33.606
NR_02445	chr16	382241	392960	+	1568	1	LOC10013	0.51	0.437
NM_0011	chr3	11272324	11557665	+	4792	1	ATG7|APG	10.816	12.645
NR_03740	chr5	86620497	86620575	+	78	1	MIR3607|	0.992	0.527

NM_0016:	chr1	1.6E+08	1.6E+08	+	927	1	APCS|HEL-	0	0
NM_0221(	chr1	1.75E+08	1.75E+08	-	2140	1	MRPS14|D	16.165	15.617
NM_0120(	chrX	1.2E+08	1.2E+08	+	4751	1	ATP1B4|-|	0.008	0.012
NR_03827	chr2	1.79E+08	1.79E+08	+	4575	1	TTN-AS1|-	1.392	0.437
NM_0245:	chr1	1.51E+08	1.51E+08	+	1174	1	TNFAIP8L2	0.132	0.058
NM_0009(	chr19	46620468	46625097	-	2081	1	PTGIR|IP|I	0.093	0.033
NM_0013:	chr19	57469586	57477570	-	5164	1	ZNF772|-|	7.675	8.146
NM_1536{	chr4	17841199	18021875	-	5594	1	LCORL|ML	1.411	2.378
NM_0011(	chr1	1.09E+08	1.09E+08	-	1688	1	HENMT1|(	0.107	0.3
NM_0013(	chr17	45161859	45170040	+	1215	1	HEXIM2|L:	12.607	12.175
NM_0012{	chr14	94561420	94569913	+	2002	1	SERPINA4|	0	0
NR_13625	chr7	87151423	87152317	-	802	1	LOC10192	1.367	1.537
NM_0211(	chrX	12975107	12977227	+	624	1	TMSB4X|F	362.856	518.094
NM_0012:	chr10	79703227	79712757	+	3283	1	NUTM2B|I	0.204	0.2
NR_02689	chr4	7754090	7778927	+	6792	1	AFAP1-AS1	6.395	5.171
NM_0159:	chr20	5937833	5950558	-	2336	1	TRMT6|CG	8.356	6.362
NM_0011:	chr8	1.23E+08	1.23E+08	-	3270	1	DERL1|DE	27.471	35.022
NR_00259	chr5	1.81E+08	1.81E+08	-	71	1	SNORD96A	1.816	3.665
NR_00152	chrY	22936455	22973284	+	1342	3	TTTY4|LIN	0	0
NR_14446	chr6	31441667	31446973	+	2138.5	4	LINC0114S	0	0
NM_0012(	chr7	5045821	5069488	+	6348	1	RBAK|ZNF	1.769	2.162
NM_0012(	chr3	1.29E+08	1.29E+08	-	5053	1	ISY1-RAB4	5.887	5.999
NM_0047:	chr1	52142094	52346686	+	5174	1	ZFYVE9|M	7.837	10.163
NM_0010(	chr16	20031485	20073778	-	1470	1	GPR139|G	0	0.019
NR_10982	chr10	32446082	32514583	+	4296	1	CCDC7|BI(	0.321	0.564
NM_0806:	chr20	63861213	63862988	+	1773	1	ABHD16B|	3.447	2.627
NM_0334:	chr12	52543909	52553147	-	2254	1	KRT71|HYI	0	0
NM_0011:	chr16	31713229	31761565	+	2767	1	ZNF720|-|	2.563	0.985
NM_0012!	chr4	1.65E+08	1.65E+08	+	2618	1	APELA|ELA	0	0.01
NM_1774:	chr19	45340740	45351520	+	1778	1	KLC3|KLC2	5.569	6.517
NM_0013:	chr11	74490511	74493713	-	2097	1	LIPT2|-|-|	0.879	0.973
NR_00313	chr4	1.19E+08	1.19E+08	+	1062	1	CEP170P1	0	0.052
NM_0133!	chr2	27428552	27442257	+	2233	1	NRBP1|BC	60.824	77.195
NM_0010(	chr10	8054704	8075201	+	3062	1	GATA3|HD	6.198	4.88
NM_0122(	chr2	42767089	42792611	-	1272	1	HAAO|3-H	0	0.032
NR_03383	chrX	15319451	15335554	-	3246	1	PIGA|GPI3	1.998	1.616
NM_0010:	chr16	67175602	67183980	-	3391	1	KIAA0895L	50.76	43.551
NR_10703	chr3	52846463	52846552	-	89	1	MIR8064|I	0	0.154
NR_13530	chr16	29545011	29595092	-	2217	1	SMG1P2|-	1.012	1.149
NM_0029{	chr17	35871491	35880373	-	1224.5	2	CCL5|D17S	0	0
NM_0314:	chr16	84819981	84909510	+	4572	1	CRISPLD2|	3.931	0.644
NM_0010(	chr3	49724295	49786540	-	4110	1	IP6K1|IHP	42.599	58.344
NM_0001:	chr9	6532464	6645692	-	3782	1	GLDC|GCE	8.993	12.426
NR_03974	chr15	83067335	83067415	+	80	1	MIR4515|I	0	0
NM_1538:	chr1	6613696	6624033	+	3664	1	PHF13|PH	13.509	17.719
NM_0013(	chr5	1.6E+08	1.6E+08	-	3168	1	CCNJL|-|-|	3.862	5.932
NR_03160	chr8	1.28E+08	1.28E+08	+	66	1	MIR1204|I	1.954	0.83
NM_0055:	chr6_GL0C	1790175	1794974	+	2652	7	HLA-E|HLA	0	0
NM_0163(	chr9	1.3E+08	1.3E+08	+	1303	1	PRRX2|PM	0.139	0.021
NM_0012{	chrX	66164230	66267388	+	3851	1	HEPH|CPL	19.673	36.898

NM_0010:	chr17	51166501	51171744	+	659	1	NME2|ND	169.787	146.115
NM_0181(	chr14	57469300	57493863	-	3086	1	C14orf105	0.058	0.107
NM_0011	chr11	1.26E+08	1.26E+08	+	1981	1	PATE4|PA	0	0.007
NM_0024(	chr17	19383442	19387219	-	1844	1	MFAP4|-|-	2.79	5.705
NM_2136	chr14	1E+08	1E+08	-	2476	1	WARS|GAI	55.386	47.22
NR_00282	chr22	38344665	38398926	-	489	1	LOC40092	6.882	9.019
NM_0013(	chr5	1.22E+08	1.22E+08	+	3338	1	SNCAIP|SY	6.466	42.002
NR_11020	chr2	1.36E+08	1.36E+08	+	775	1	DARS-AS1|	2.362	1.113
NM_0010(	chr5	863735	892559	-	2503	1	BRD9|LAV	22.294	20.078
NM_0010:	chr4	48885380	48906828	-	793	1	OCIAD2|-|	0.13	4.094
NR_13508	chr11	64247054	64248214	+	466	1	LOC10536	11.593	13.197
NR_10674	chr11_JH1	61715	61823	+	108	2	MIR6128|	0	0
NM_0230:	chr1	12857086	12861909	+	1636	1	PRAMEF2|	0	0
NR_13664	chr10	42751186	42755203	-	4017	1	LOC28302	0.035	0.331
NM_0153	chr15	68277803	68295865	+	7118	1	FEM1B|F1	10.075	13.224
NR_02962	chr9	70809975	70810084	-	109	1	MIR204|M	0	0
NM_0010	chrX	1.35E+08	1.35E+08	+	1240	1	FAM127A|	176.09	150.468
NR_13459	chr6	1.68E+08	1.68E+08	-	451	1	LOC10537	0	0
NM_2034	chr1	1.84E+08	1.84E+08	-	2774	1	APOBEC4|	0	0.005
NM_0070	chr11	1.3E+08	1.3E+08	-	4001	1	ADAMTS8	0.197	0.284
NM_0179	chr22	45309904	45341955	+	2902	1	FAM118A|	8.242	10.879
NR_02948	chr19	51692786	51692864	+	78	1	MIRLET7E	0	0
NR_03823	chr17	50050349	50055739	-	2530	1	LOC28408	0.051	0.038
NM_0013	chr16	28711420	28735729	+	3065.5	2	EIF3CL|-|-	1.788	2.239
NM_0013(	chr11	1.09E+08	1.09E+08	-	10051	1	EXPH5|SL	0.024	0.108
NR_03995	chr3	5250177	5250255	-	78	1	MIR4790|-	0	0
NM_0010	chr2	2.11E+08	2.13E+08	-	11846	1	ERBB4|AL	6.118	2.806
NM_0326	chr12	1.25E+08	1.25E+08	-	4522	1	DHX37|DD	3.752	4.868
NM_0010	chr7	1.09E+08	1.09E+08	-	431	1	C7orf66|-|	0	0
NM_0121	chr11	86945675	86955398	-	7390	1	FZD4|CD3	0.864	1.398
NR_03018	chr19	53674197	53674320	+	123	1	MIR498|M	0	0
NM_0010	chr1	2104716	2185395	+	1994	1	PRKCZ|PK	48.748	60.709
NM_0013	chr8	1.44E+08	1.44E+08	+	4992	1	WDR97|KI	0.145	0.115
NM_0140	chr19	13774443	13778772	+	904	1	C19orf53|	67.249	49.182
NM_0140	chr20	31475288	31484905	+	1663	1	REM1|GD	0.016	0
NM_1528	chr1	85729233	86156438	-	6438	1	COL24A1|-	0.13	0.053
NM_0057	chr2	97655939	97664098	-	2245	1	ACTR1B|A	49.794	53.885
NM_1456	chr15	68930500	68946811	+	1427	1	SPESP1|ES	0.054	0.01
NM_0011(	chr7	1.22E+08	1.22E+08	-	2033	1	FEZF1|FEZ	24.392	7.283
NR_02416	chr16	3110460	3115598	-	1216	1	ZNF205-AS	7.412	6.466
NM_0012	chr19	19629473	19643667	-	3432	1	GMIP|ARH	22.875	20.202
NR_07722	chr12	95649255	95673994	+	2138	1	PGAM1P5	0.036	0.026
NM_0175	chr1	1.55E+08	1.55E+08	-	3208	1	UBE2Q1|G	41.277	41.667
NR_11026	chr2	41931599	41933723	-	786	1	LINC01914	0	0
NM_0013	chr3	45689241	45745425	+	3596	1	SACM1L|S	5.848	6.567
NM_1815(	chr5	62578735	62581448	+	2245	1	LRRC70|SL	0.046	0.098
NR_03987	chr16	28843919	28844007	-	88	1	MIR4721|-	15.677	10.895
NM_0011	chr1	44674722	44725591	+	1671	1	C1orf228|	3.194	1.893
NM_0010	chrX	46912276	47061242	+	4700	1	JADE3|JAD	5.426	4.736
NM_0012	chr11	1.26E+08	1.26E+08	-	1415	1	PUS3|261(	6.023	7.357

NM_0528	chr8	1.19E+08	1.19E+08	+	2812	1	MAL2|-|-|	1.44	1.374
NM_0207	chr1	3780220	3796504	-	2639	1	LRRC47|-|	42.907	47.397
NM_0035	chr6	27873148	27873511	-	361	1	HIST1H4L|	0	0
NM_1816	chr21	30829040	30829732	-	690	1	KRTAP7-1|	0.037	0
NM_0011	chr3	1.6E+08	1.6E+08	+	1642	1	SCHIP1|SC	87.6	98.056
NR_03398	chr1	89624849	89632894	-	786	1	FLJ27354|	0.295	0.418
NR_13394	chr9	1.3E+08	1.3E+08	+	2923	1	TOR1B|DC	11.738	12.685
NR_12637	chr5	54320944	54415125	+	710	1	LINC01033	0.036	0.039
NM_0024	chr1	2.03E+08	2.03E+08	-	1528	1	MYOG|MY	0.034	0.099
NM_0325	chr6	43243680	43288259	+	6915	1	TTBK1|BD	12.688	10.732
NM_0018	chr16	21258518	21303136	-	1547	1	CRYM|DFN	0.1	19.248
NM_0035	chr17	146805	181630	-	2015	2	DOC2B|DC	0.083	0.088
NM_0011	chr17	63998351	64004284	+	3027	1	PRR29|C1	1.265	0.688
NM_0808	chr19	14028148	14031558	+	1092	1	RLN3|H3|	0.059	0
NM_0326	chr4	79317118	79326017	-	1512	1	NAA11|AR	0.153	0.082
NM_0010	chr10	5412551	5459056	+	3981	1	NET1|ARH	5.48	5.113
NM_0178	chr7	1.33E+08	1.33E+08	-	1670	1	CHCHD3|N	25.424	24.302
NM_0032	chr20	45823216	45827314	-	673	1	TNNC2|-|-	0.939	0.773
NM_0010	chr11	6107684	6108835	+	1149	1	OR56B4|C	0.022	0.012
NM_0162	chr16	19858971	19884911	-	2921	1	GPRC5B|R	41.686	34.756
NM_1449	chr6	1.65E+08	1.65E+08	-	1828	1	C6orf118|	1.284	1.109
NM_1768	chr5	53095679	53109772	-	3711	1	MOCS2|M	6.702	7.566
NM_0010	chr14	89955902	90044764	+	3506	1	TDP1|-|-|	10.43	9.556
NM_0012	chr21	29056322	29073797	-	1911	1	CCT8|C21	84.115	87.027
NR_13046	chr18	53237101	53237190	+	89	1	MIR4528|	0	0
NM_0051	chr12	53665160	53676325	-	877	1	ATP5G2|A	198.945	185.23
NR_03653	chr11	9459556	9460698	-	1142	1	LOC64465	2.484	2.015
NM_0249	chr10	97498882	97571210	+	1688	1	UBTD1|-|-	8.524	9.104
NM_0070	chr6	44158811	44184402	+	2703	1	CAPN11|c	0.01	0.061
NR_00439	chr17	76558792	76558869	+	77	1	SNORD1C|	0.335	0.534
NM_0208	chr1	45016404	45206578	-	5820	1	ZSWIM5|-	12.8	14.156
NM_0036	chr17	42682614	42699993	+	5428	1	CNTNAP1|	10.031	8.193
NM_0011	chr17	19127469	19158835	+	961	1	GRAPL|-|-	0	0
NM_0013	chrY	9337464	9340278	+	891	2	TSPY1|CT	0	0
NM_0037	chr22	36510850	36529230	-	1932	1	EIF3D|EIF	90.942	110.519
NM_0122	chr1	99969789	1E+08	+	5862	1	SLC35A3|	0.664	1.007
NM_0012	chr2	74532259	74549663	-	2980	1	LOXL3|LO	19.864	19.456
NM_1829	chr14	64214141	64226451	+	2678	1	SYNE2|ED	10.448	20.75
NM_0013	chr1	2.16E+08	2.16E+08	+	3932	1	KCTD3|NY	8.968	11.081
NM_0011	chr16	67943474	67966878	-	4609	1	SLC12A4|	0.892	1.81
NM_0325	chr9	1.22E+08	1.22E+08	+	5452	1	DAB2IP|A	34.722	41.666
NR_11004	chr14	26873133	26914743	+	818	1	MIR4307H	0.016	0
NR_00313	chr5	83064204	83064337	-	133	1	SCARNA18	0	0.103
NR_03156	chr1	1.17E+08	1.17E+08	+	78	1	MIR320B1	0	0
NM_0065	chr6	96015969	96215612	+	12793	1	FUT9|Fuc-	0.832	5.707
NR_04675	chr3	85799987	85828050	-	764	1	CADM2-AS	0	0
NM_0012	chr11	1.18E+08	1.18E+08	-	1110	1	FXYD6-FXY	79.73	91.03
NM_0178	chr18	32018482	32073191	+	5880	1	RNF125|T	0.048	0.016
NM_0010	chr2	99400475	99490056	-	4744	1	REV1|AIBF	6.436	8.904
NM_1981	chr21	5116329	5128440	-	1566	2	C21orf33|	3.244	1.985

NR_03745	chr2	12199130	12199201	+	71	1	MIR3681|-	0	0
NM_0143:	chr12	65169571	65248361	+	4783	1	LEMD3|M.	5.604	6.987
NM_1524(	chr19	1473201	1479229	-	2252	1	C19orf25|	17.039	16.677
NR_13198	chr12	1.28E+08	1.28E+08	-	549	1	CRAT8|-|-	0	0
NM_0012:	chr6	44246958	44253888	+	2545	1	HSP90AB1	508.93	468.363
NM_0011:	chr8	23288092	23296279	+	1579	1	R3HCC1|-|	60.728	59.455
NM_0010‹	chr19	53395144	53412009	+	4571	1	ZNF765|-|	0.527	1.303
NR_00442	chr7	54201224	54202421	-	1197	1	HPVC1|HP	0.043	0
NR_00243	chr6	1.33E+08	1.33E+08	+	132	1	SNORA33|	1.465	3.009
NR_03667	chr9	21277688	21278563	-	875	1	IFNA22P|I	0.029	0
NR_00322	chr14	1.01E+08	1.01E+08	+	68	1	SNORD114	0	0
NM_0010:	chr11	30231016	30235277	+	1901	1	FSHB|HH2	0	0
NM_0011‹	chr2	1.01E+08	1.01E+08	-	1715	1	RFX8|-|-|2	0.038	0.176
NR_13552	chr2	1.38E+08	1.38E+08	+	550	1	LINC01832	0	0
NR_03895	chr1	53238610	53242783	+	1178	1	LOC10050	2.539	4.872
NM_0050:	chr1	63774019	64181496	+	5843	1	ROR1|NTR	0.752	0.328
NM_0013:	chr1	2.21E+08	2.21E+08	+	2219	1	MARC2|M	0.889	0.988
NR_02838	chr2	38959288	38960344	-	1056	1	LOC37519	0.244	0.143
NM_0011!	chr1	32765634	32774970	+	2644	1	KIAA1522|	8.129	12.676
NR_03170	chr1	2.2E+08	2.2E+08	-	77	1	MIR664A|	0	0
NM_0013:	chr17	63832081	63842466	-	2308	1	SMARCD2	7.519	7.317
NM_0011‹	chr2	27628648	27650846	+	1606	1	GPN1|ATP	37.468	37.739
NM_0010!	chr19	36214602	36238773	+	3736	1	ZNF146|O	12.742	20.802
NM_0070:	chr1	2391775	2405446	+	3095	1	RER1|-|-|:	47.308	51.557
NM_0012!	chr1	31727105	31764063	-	5307	1	ADGRB2|E	44.154	51.743
NM_1384:	chr19	1905214	1913447	+	1780	1	ADAT3|FW	2.832	1.985
NR_10687	chr22	37967563	37967624	+	61	1	MIR6820|I	68.483	54.564
NM_0148‹	chr12	1.06E+08	1.06E+08	-	6812	1	NUAK1|AF	8.487	11.33
NR_00257	chr17	38852863	38852995	-	132	1	SNORA21|	1.367	2.075
NM_1526:	chr4	48483343	48489524	+	2113	1	SLC10A4|F	2.966	2.424
NM_0022(	chr6_GL0C	4585944	4604308	+	2687	3	KIFC1|HSE	0	0
NM_0147(	chr1	2.35E+08	2.35E+08	-	3378	1	TOMM20|	50.94	58.697
NM_0010(	chr11	55795556	55796500	+	943	1	OR5D14|C	0	0
NM_1735(	chr1	92029976	92063536	+	1434	1	EPHX4|AB	0.647	0.583
NR_05198	chr13	48576974	48580901	-	1020	1	LINC00462	0	0
NM_0012!	chr1	75134882	75161533	+	1753	1	LHX8|LHX:	0	0
NM_0058(	chr6	27247723	27256620	+	2851	1	PRSS16|TS	0.194	0.245
NR_03020	chr19	53720096	53720182	+	86	1	MIR520D|	0	0
NM_0313(	chr8	1.44E+08	1.44E+08	-	3775	1	SCRT1|SCF	42.226	29.012
NM_0013(	chr11	61348748	61362283	-	2254	1	CYB561A3	6.533	5.378
NM_0001:	chr5	1.52E+08	1.52E+08	-	1786	1	GLRA1|HK	0.44	0.161
NR_13620	chr4	1.02E+08	1.03E+08	-	761	1	LOC10537	1.61	1.872
NM_0328:	chr1	2.04E+08	2.04E+08	-	5272	1	PPP1R15B	11.813	12.938
NM_0184:	chr10	97640686	97676432	+	4176	1	PI4K2A|PI4	14.601	15.383
NM_0247:	chr16	67199125	67204024	+	2490	1	ELMO3|CE	1.693	1.546
NM_0133!	chr21	15730025	15880071	+	5107	1	USP25|US	1.785	1.92
NR_13391	chr11	83185521	83187484	-	1963	1	PCF11-AS1	0.65	0.488
NM_0165(	chr5	1.42E+08	1.42E+08	-	6556	1	PCDH12|V	1.914	0.794
NM_1334!	chr1	1.98E+08	1.98E+08	+	4120	1	NEK7|-|-|:	5.58	1.187
NM_0012(	chr15	57591904	57717557	+	5290	1	GCOM1|G	4.095	4.119

NR_12060	chr8	10474565	10481974	+	540	1	LINCR-000	0.119	4.921
NM_0013:	chr3	51975026	51989202	+	1860	1	ABHD14A-	12.325	10.177
NR_03979	chr7	1.01E+08	1.01E+08	+	82	1	MIR4653|-	0.314	0.334
NM_0011:	chrX	49331584	49338962	+	565	1	GAGE2E|G	0	0
NM_0013:	chr17	74215616	74262018	+	3366	1	TTYH2|C1	12.062	15.455
NR_03970	chr11	65649192	65649253	+	61	1	MIR4489|	6.764	6.287
NR_02804	chr12	10588478	10599835	-	2201	1	KLRA1P|KI	0.428	0.56
NM_0002!	chr17	58269856	58280935	-	3201	1	MPO|-|-|1	0.04	0
NR_01540	chr1	2.38E+08	2.38E+08	-	1529	1	LINC0113S	0	0.233
NM_0331:	chr1	2.28E+08	2.28E+08	+	2966	1	WNT3A|-|	0.743	0.046
NM_0012{	chr17	74694309	74713000	-	1763	1	CD300LF|(	0	0
NR_13077	chr5	95962001	96631085	+	468	1	LOC10192!	0	0
NR_13046	chrX	55451495	55451582	+	87	1	MIR4536-2	0	0
NR_00153	chrY	6471031	6473630	-	528	2	TTTY8|LIN	0	0
NR_03749	chr6	36622436	36622512	-	76	1	MIR3925|-	0	0
NM_0013(	chr1	2355175	2391751	-	1531	1	MORN1|-|	4.935	3.337
NM_2014	chr9	1.37E+08	1.37E+08	+	1291	1	EGFL7|NE	6.282	7.851
NR_02940	chr7	64433693	64435517	+	1824	1	YWHAEP1	0.728	1.412
NR_03995	chr3	1.15E+08	1.15E+08	-	80	1	MIR4796|	0	0
NM_0180{	chr12	1.06E+08	1.07E+08	+	4243	1	POLR3B|C	3.896	4.539
NM_0008:	chr5	1.62E+08	1.62E+08	+	3922	1	GABRG2|(	3.179	2.759
NR_10382	chr3	9986893	10007003	+	4692	1	EMC3-AS1	1.495	1.573
NR_13365	chr1	1.56E+08	1.56E+08	+	921	1	RAB25|CA	0.63	0.535
NM_2034{	chr19	39498917	39508481	+	2035	1	DLL3|SCD(	77.627	38.607
NM_0010!	chr3	14112077	14124871	-	1429	1	CHCHD4|M	7.778	8.789
NM_0012(	chr13	1.11E+08	1.11E+08	+	1934	1	ING1|p24I	13.447	14.936
NM_0024	chr10	46033305	46046269	-	488	1	MSMB|HP	0.026	0
NM_0013	chr4	52051331	52097305	+	4187	1	SPATA18|I	0.345	0.507
NM_0012{	chr9	92600257	92615155	+	2555	1	CENPP|CE	2.574	3.195
NR_10465	chr5	50969217	50970187	-	624	1	LINC0210€	0	0
NR_11189	chr9	67059460	67297156	+	7625	1	CNTNAP3F	0.039	0.005
NR_10698	chr6	1.59E+08	1.59E+08	+	83	1	MIR7161|I	1.553	0.33
NM_0182(	chr9	1.29E+08	1.29E+08	+	3889	1	TBC1D13|-	21.925	20.833
NR_10705	chr14	1.06E+08	1.06E+08	+	64	4	MIR8071-2	0	0
NM_0012	chr7	90211860	90237678	+	2134	1	STEAP2|IP	0.085	0.218
NR_02733	chr10	25158072	25176276	-	2431	1	GPR158-A!	0.329	0.208
NM_0057(	chr2	37201631	37231597	-	3310	1	CEBPZ|CBI	5.609	7.324
NR_03385	chr6	2854657	2876510	-	1250	1	SERPINB9F	0.041	0.066
NR_03391	chr6	1.39E+08	1.39E+08	-	1686	1	LINC01625	0.444	0.106
NM_0060:	chr11	1.14E+08	1.14E+08	+	1877	1	HTR3B|5-I	0.261	0
NM_0013:	chr6	36027677	36111236	+	4339	1	MAPK14|(	9.345	12.466
NM_0060!	chr11	68042980	68050899	+	2444	1	TCIRG1|AT	0.665	0.527
NM_1825(	chr16	2209253	2211068	-	1023	1	BRICD5|C1	8.659	7.685
NM_0013(	chr17	64854312	64919480	-	2588	1	LRRC37A3	1.893	2.694
NR_12064	chr10	25113058	25161233	+	2969	1	LINC0151€	0.052	0.046
NM_0012{	chr20	44582549	44619037	+	1174	1	PKIG|PKI-{	70.409	63.562
NR_03604	chrX	1.55E+08	1.55E+08	-	98	3	MIR1184-1	0	0
NR_00305	chr16	71758402	71758487	-	85	1	SNORD71|	0	0.161
NR_03061	chr3	10394489	10394562	-	73	1	MIR885|M	0	0
NM_0334{	chr6	27309063	27312232	-	3167	1	POM121L2	0.008	0.022

NR_11003	chr12	1.27E+08	1.27E+08	-	702	1	LOC10099	0	0
NR_13394	chr4	1.85E+08	1.85E+08	+	476	1	LOC10537	1.354	0.691
NR_00365	chr15	1.02E+08	1.02E+08	+	2543	1	WASH3P|	1.521	1.492
NR_11001	chr7	7949839	7968752	-	1165	1	LOC10050	0.022	0.094
NM_0014	chr2	46297402	46386703	+	5148	1	EPAS1|EC	0.511	0.636
NM_0182	chr7	66996805	67239520	+	3346	1	TYW1|RSA	3.017	3.087
NR_00297	chr9	1.37E+08	1.37E+08	-	133	1	SNORA17E	2.23	1.648
NR_03807	chr1	1.65E+08	1.65E+08	-	1959	1	LOC10050	0	0.014
NM_0058	chr16	57735748	57757250	+	2624	1	KATNB1|K	24.357	24.502
NM_2058	chr6	30931350	30932175	-	449	6	SFTA2|GS	0	0
NM_0003	chr2	31334322	31414745	-	5679	1	XDH|XAN1	0	0.077
NM_1384	chr6	1.11E+08	1.11E+08	+	942	1	GTF3C6|C	26.444	19.281
NR_13299	chr6	57919881	57961446	-	1020	1	LINC0068C	0.847	0.188
NR_03018	chr19	53690881	53690965	+	84	1	MIR520A|	0	0
NM_0806	chr20	13995500	16053196	+	4843	1	MACROD2	1.262	2.339
NR_02919	chr2	1.09E+08	1.09E+08	-	2791	1	SH3RF3-AS	1.16	2.405
NM_0011	chr19	9376316	9382617	+	2439	1	ZNF177|PI	5.133	11.894
NM_0035	chr6	44826730	45378051	-	4312	1	SUPT3H|S	2.47	1.718
NR_10501	chr5	80947693	80960907	-	535	1	RASGRF2-A	0	0.205
NR_03654	chr2	1.35E+08	1.35E+08	-	1400	1	CCNT2-AS	4.457	4.217
NM_0012	chr17	73737854	73756557	+	1132	1	LOC10013	0.011	0.012
NM_0012	chr15_KI2	4808041	4821891	-	5304.8	5	GOLGA8N	0	0
NM_0013	chr12	49539157	49558312	+	3850	1	KCNH3|BE	1.601	3.575
NM_0150	chr15	64499420	64686067	+	8606	1	ZNF609|-|	33.166	46.768
NR_03392	chr10	43845306	43850622	+	1118	1	LINC0061	0	0
NM_0011	chr3	1.46E+08	1.46E+08	-	1205	1	PLSCR2|-|	0	0
NM_0013	chr1	2.05E+08	2.05E+08	+	2766	1	TMCC2|H	38.946	43.379
NR_03168	chr6	33999972	34000051	-	79	1	MIR1275|	0	0
NR_11038	chrX	40262917	40287721	+	2998	1	LOC10192	0	0.005
NM_1817	chr3	57060441	57075432	+	2409	1	SPATA12|	0.134	0.233
NM_0011	chr8	42752636	42768786	-	2332	1	CHRNA6|C	1.122	0.887
NM_0013	chr7	92412550	92437314	-	1706	1	LOC10537	0.03	0
NM_0012	chr1	38838197	38859823	-	2622	1	RRAGC|GT	11.349	12.908
NR_04979	chr6	1.38E+08	1.38E+08	-	1960	1	LOC10013	0.059	0.021
NM_0034	chr6_GL0C	1664235	1693265	-	3574.2	8	TRIM26|A	0.314	0.525
NM_0140	chr20	34714774	34825649	-	7064	1	NCOA6|AI	34.616	35.171
NM_0331	chr19	55476332	55484487	+	3842	1	ZNF628|Z	2.514	2.549
NM_2015	chr3	48157178	48188311	-	3570	1	CDC25A|C	5.826	3.572
NM_0010	chr5_GL33	965268	997919	+	2087	5	GTF2H2C|	0.229	0.302
NM_0013	chr19	48332356	48364237	-	2446	1	TMEM143	4.312	4.037
NM_0011	chr2	1.07E+08	1.07E+08	-	6779	1	ST6GAL2|	6.497	7.462
NM_0013	chr11	1.19E+08	1.19E+08	+	1141	1	TRAPPC4|	70.852	65.292
NM_0012	chr19	48637942	48646312	-	1833	1	CA11|CAR	40.439	29.083
NM_2033	chr19	55620741	55624575	-	1992	1	ZNF784|-|	8.434	7.323
NR_03990	chr19	4932687	4932740	+	53	1	MIR4747|	1.46	0.258
NM_0012	chr7	1.08E+08	1.09E+08	-	4422	1	PNPLA8|IF	9.406	9.915
NM_0010	chr19	10310211	10316015	-	839	1	FDX1L|FD	19.409	13.093
NR_11077	chr18	9615264	9619363	+	376	1	PPP4R1-AS	0.96	1.093
NM_0147	chr7	1.12E+08	1.12E+08	-	8282	1	DOCK4|-|-	6.318	7.097
NR_02675	chr9	64463599	64469259	-	1538	3	FAM95B1|	0	0

NM_0013(	chr1	1921951	2003837	-	5371	1	CFAP74|C:	0.115	0.089
NM_0010:	chr14	74661252	74713104	-	5423	1	AREL1|FIE	28.455	38.868
NM_0013:	chr2	2.22E+08	2.23E+08	+	4725	1	SGPP2|SPI	1.083	0.907
NR_11090	chr16	11465260	11473174	+	2276	1	LOC10192	0	0.024
NM_0010(	chr19	39084305	39111488	+	2946	1	ACP7|PAP	0	0
NM_0011(	chr3	1.69E+08	1.69E+08	-	4794	1	MECOM|A	0.363	0.097
NM_0011:	chr2	1.99E+08	1.99E+08	-	5717	1	SATB2|GL!	0.864	1.299
NM_0177(	chr15	30938941	30991604	-	4941	2	MTMR10|	0.076	0.225
NM_1981	chr17	29560671	29567024	-	3351	1	ABHD15|-	2.051	3.151
NR_12048	chr12	9371782	9381422	-	320	1	LOC10192	0	0
NR_12046	chr12	1.32E+08	1.32E+08	+	474	1	LOC10192	0.381	0.116
NM_0013(	chr22_KI2	216394	220281	-	1165	4	GSTT2|-|-	0	0.012
NM_0807:	chr19	40799143	40808441	+	2147	1	EGLN2|EIT	43.046	32.491
NM_0175	chr20	7882984	7940446	-	1736	1	HAO1|GO	0	0
NM_1384	chr6	37482921	37499924	-	614	1	CCDC167|	100.691	99.471
NM_0011(	chr14	69484754	69528498	+	1797	1	PLEKHD1|	0.093	0.244
NM_0009	chr6	57317617	57646850	+	2300	1	PRIM2|PR	4.429	3.811
NR_14436	chr1	1308580	1311677	+	1209	1	PUSL1|-|-	10.473	7.908
NR_02417	chr17	37842949	37884743	-	2448	2	YWHAEP7	0	0
NM_0012:	chr6	1.09E+08	1.09E+08	+	1957	1	LOC10099	0.053	0.021
NM_0021(	chr4	1.44E+08	1.44E+08	-	491	1	GYPE|GPE	0	0
NM_0330(	chr17	41177446	41178208	-	760	1	KRTAP4-2|	0	0
NM_0011:	chr4	87303789	87322906	-	2281	1	HSD17B13	0.023	0.042
NM_0063(	chr11	62792126	62805492	-	2323	1	NXF1|MEX	44.814	37.453
NM_0011!	chr6	24171755	24383292	-	4602	1	DCDC2|DC	0.109	0.051
NM_0529(	chr1	9997197	10016020	+	649	1	RBP7|CRA	0.298	0.696
NM_1980(	chr14	52775193	52791668	-	3920	1	GNPNAT1|	2.987	3.351
NM_0009	chr15	64155815	64163155	-	1021	1	PPIB|CYP-!	307.486	242.025
NR_13653	chr1	2.03E+08	2.03E+08	+	3007	1	TMEM183	12.1	16.343
NR_03829	chr6	27694035	27710222	+	672	1	LINC01012	1.209	1.631
NM_0011(	chr21	30590105	30590397	-	290	1	KRTAP22-2	0	0
NM_0245!	chr4	55346221	55373099	+	4074	1	SRD5A3|C	1.842	2.955
NM_0010	chr3	1.34E+08	1.35E+08	+	1881	1	CEP63|SCI	7.903	7.682
NR_03842	chr1	2.35E+08	2.35E+08	-	2857	1	LINC01354	0.059	0.192
NR_12537	chr5	473236	480891	+	1774	1	LOC10028	10.073	8.817
NM_0011:	chr16	760765	768865	+	2089	1	MSLN|MP	0.167	0.059
NM_0013(	chr4	16501534	16898809	-	2539	1	LDB2|CLIN	16.24	13.298
NM_0006(	chr4	99306387	99321442	-	2681	1	ADH1B|AD	0.13	0.041
NM_0012!	chr3	50362613	50504244	-	5421	1	CACNA2D2	15.358	17.755
NR_10386	chr12	29280416	29317848	-	2375	1	LOC10050	0.418	1.557
NM_0150:	chr1	39409504	39416482	+	6976	1	KIAA0754|	2.506	2.933
NR_03657	chr13	52012387	52029644	+	1400	1	ALG11|CD	6.143	7.426
NM_0012:	chr10	28846408	28881897	+	2141	1	C10orf126	0.03	0.019
NM_0012	chr9	1.28E+08	1.28E+08	+	2199	1	CERCAM|(	19.326	17.422
NM_0016	chr12	1.1E+08	1.1E+08	+	4321	1	ATP2A2|A	51.162	53.492
NM_0066(	chr2	1.27E+08	1.27E+08	-	10852	1	MAP3K2|I	1.366	2.133
NR_03793	chr20	59028678	59042846	-	762	1	SLMO2-AT	208.529	171.087
NM_0010:	chr12	55997259	56005525	+	2221	1	SUOX|-|-|	19.558	17.755
NM_0011!	chr14	23306762	23326185	+	2119	1	BCL2L2-PA	55.912	49.85
NM_0013:	chr17	76142453	76240311	-	4807	1	RNF157|-|	35.491	47.026

NM_0011{	chr1	39026295	39034636	+	562	1	NDUFS5|C	264.454	262.949
NM_0309:	chr1	1.61E+08	1.61E+08	-	3491	1	NECTIN4|E	0.03	0.153
NM_0162	chrX	1.42E+08	1.42E+08	-	1989	1	MAGEC2|(	0	0
NM_1522{	chr4	99546707	99563668	-	3704	1	TRMT10A|	1.514	1.072
NM_0025:	chr16	2039815	2047869	-	1062	1	NTHL1|FA	20.19	17.063
NM_0201{	chr1	1.14E+08	1.14E+08	+	1827	1	OLFML3|H	2.463	1.095
NM_0123:	chr17	82458664	82488267	+	1608	1	NARF|IOP:	98.938	75.794
NM_0010	chr19	9827880	9830121	+	444	1	UBL5|HUB	344.349	242.875
NR_02438	chr5	88664445	88678445	-	3355	1	LINC00461	16.602	61.418
NR_11093	chr16	82773319	82829638	+	4034	1	LOC10192:	0	0.003
NR_00281	chr17	57045478	57046795	+	1317	1	RNF126P1	1.038	0.728
NR_02657	chr5	71074203	71093070	-	573	2	LOC64785!	0	0
NR_00370	chr14	91126426	91126552	+	126	1	SNORA11E	0	0
NM_0011{	chr2	48580587	48679609	+	3612	1	STON1-GT	0.953	2.465
NM_0011:	chr1	1.74E+08	1.74E+08	+	2348	1	ZBTB37|D	2.087	2.17
NM_0001	chr18	57544841	57586737	-	7247	1	FECH|EPP	2.978	4.243
NR_03993	chr2	1.51E+08	1.51E+08	-	77	1	MIR4773-:	0	0
NM_0011(	chr22	36487186	36507101	-	4960	1	FOXRED2|	12.085	9.941
NR_12040	chr2	1.38E+08	1.38E+08	-	1298	1	LOC10192:	0	0
NR_03382	chr13	87443987	87670963	-	1669	1	MIR4500H	0.155	0.066
NR_03980	chr8	6745164	6745244	+	80	1	MIR4659A	0	0.171
NM_0177!	chr6	34792017	34877514	+	9610	1	UHRF1BP1	5.404	4.826
NM_0011:	chr12	54230946	54259586	-	11498	1	CBX5|HEL:	42.823	31.129
NM_0010:	chr16	19706352	19718170	-	1943	1	KNOP1|10	18.547	17.616
NM_0013:	chr9	1.33E+08	1.33E+08	-	4059	1	DDX31|PP	4.574	4.292
NR_00280	chr8	1.45E+08	1.45E+08	+	4032	1	TMED10P1	1.033	0.9
NR_10671	chr22	35336721	35336799	-	78	1	MIR6069|	0.331	0
NM_0007(	chr19	41088451	41096195	+	1749	1	CYP2A13|(	0	0.008
NR_13234	chr20	44451984	44465344	-	426	1	LINC0162C	0	0
NM_0186:	chr15	89749867	89751309	-	1158	1	MESP1|bH	2.015	3.3
NM_0011(	chrX	38033534	38128820	+	4686	1	SYTL5|slp5	0.278	0.184
NR_03840	chr6	1.49E+08	1.49E+08	-	2078	1	UST-AS1|-	0	0
NR_02939	chr22	25318257	25320226	+	672	1	IGLL3P|16	0.422	0.775
NM_1525(	chr21	39405844	39444202	-	2352	1	LCA5L|C21	0.291	0.355
NM_0011{	chr12	27524112	27695564	+	5972	1	PPFIBP1|L	4.834	3.72
NR_03398	chr12	64883774	64977522	+	3163	1	FLJ41278|	0.008	0.043
NM_0010(	chr17_GLC	788696	789140	+	442	3	STH|MAPT	0	0
NR_10275	chr5	1.81E+08	1.81E+08	+	1110	1	TRIM52-AS	20.095	19.188
NR_04640	chr7	1.51E+08	1.51E+08	+	2136	1	ASIC3|ACC	4.316	3.649
NR_03764	chr10	1.03E+08	1.03E+08	+	2716	1	BORCS7-A!	5.094	5.275
NM_0045(	chr1	2.45E+08	2.45E+08	-	6773	1	HNRNPU|I	66.904	59.822
NM_1989:	chr11	1.19E+08	1.19E+08	+	2226	1	HINFP|HiN	20.452	19.936
NM_0010:	chr11	20156214	20160324	-	1027	1	DBX1|-|-|	4.356	0.347
NM_0010(	chr19	9213850	9214871	-	1019	1	OR7D4|OF	0	0
NR_12058	chr11	1.3E+08	1.3E+08	-	973	1	LINC01395	0	0
NM_0029:	chr10	1.19E+08	1.2E+08	-	846	1	RGS10|-|-	1.372	1.797
NM_0013:	chr11	1.17E+08	1.17E+08	-	1987	1	ZPR1|ZNF:	23.899	28.014
NR_03621	chr1	1.55E+08	1.55E+08	+	90	1	MIR4258|	0	0.152
NR_13519	chr16	30096430	30105456	+	2988	1	LOC10192:	3.159	4.263
NM_0011	chr1	1.09E+08	1.09E+08	+	3045	1	FNDC7|-|-	0.174	0.202

NM_0013:	chr16	4804522	4847388	-	2221	1	GLYR1|BM	49.438	43.237
NM_0180:	chr17	17476986	17493220	+	2218	1	MED9|ME	15.858	21.713
NR_07317	chr11	64449057	64451656	+	2354	1	LOC10099	0.811	1.798
NR_00258	chr5	40832656	40832735	-	79	1	SNORD72|	0	0
NR_02741	chr17	68101555	68135929	+	11187	1	LINC00674	7.217	6.665
NR_03740	chr2	1.89E+08	1.89E+08	+	62	1	MIR3606|	1.456	0.442
NM_0328	chr15	43870761	44195294	-	5061	1	FRMD5|-|	7.77	12.024
NM_0052	chr1	1.1E+08	1.1E+08	-	1355	1	GNAT2|AC	0.038	0.364
NM_0061	chr5	1.36E+08	1.36E+08	-	1665	1	NEUROG1	0.867	4.089
NM_1528	chr20	63240784	63254540	-	1421	1	NKAIN4|C	101.016	32.339
NR_03389	chr10	45154662	45186041	+	1306	1	ANKRD30E	0	0.01
NM_0041	chr17	74862497	74873031	-	1887	1	FDXR|ADX	7.379	5.342
NM_0032	chr1	2.01E+08	2.01E+08	-	6133	1	TNNI1|SST	0.315	0.315
NR_00145	chr21	25562145	25575168	+	1471	1	MIR155HG	0.061	0.056
NM_0328	chr15	57376505	57550727	+	7203	1	CGNL1|JA	0.861	0.274
NM_1979	chr15	45430529	45433449	+	832	1	C15orf48|	0.186	0.132
NM_1734	chrX	1.36E+08	1.36E+08	-	5214	1	MMGT1|E	5.977	6.179
NR_00304	chr17	16439509	16439556	+	47	1	SNORD49E	27.707	22.731
NM_0183	chr6	39104063	39115186	-	2014	1	SAYSD1|C	7.209	10.154
NM_0035	chr6	27893425	27893891	+	464	1	HIST1H2B	0.389	0.089
NM_0011	chr4	1.15E+08	1.15E+08	+	4130	1	UGT8|CGT	0.496	0.6
NR_03971	chr12	1.2E+08	1.2E+08	-	65	1	MIR4498|	0	0
NR_11011	chr12	4700417	4720102	-	1245	1	LOC10192	0.476	0.462
NR_12599	chr1	1430157	1434573	-	900	1	LINC0177C	0.544	0.365
NR_02949	chr7	1E+08	1E+08	-	83	1	MIR25|MI	2.33	10.727
NM_0149	chr2	1.91E+08	1.91E+08	+	4812	1	GLS|AAD2	3.561	5.744
NM_0011	chr1	1.85E+08	1.85E+08	+	3774	1	SWT1|C1c	1.578	1.8
NM_0010	chr20	33666498	33668525	+	2025	1	ACTL10|C	0.096	0.162
NM_0053	chr4	95840088	95841474	+	1384	1	PDHA2|PD	0.904	0.683
NM_0166	chr17	42679402	42681827	-	1240	1	CCR10|GP	1.539	1.999
NM_0011	chrX	13689121	13709826	+	1270	1	RAB9A|RA	10.02	14.107
NM_1983	chr11	1.24E+08	1.24E+08	+	1846	1	VWA5A|B	36.313	27.35
NR_11064	chr9	1048143	1048640	+	497	1	LINC0123C	0	0
NR_02701	chr8	46840886	46855785	+	2137	1	LINC00293	0.012	0
NM_0011	chr1	1.16E+08	1.16E+08	+	3551	1	ATP1A1|-|	67.543	107.81
NM_0031	chr19	55173101	55180352	-	1814	1	SYT5|-|-|1	59.236	35.707
NM_0013	chr19	15421507	15432534	-	4191	1	WIZ|ZNF8	48.042	39.705
NR_11075	chr15	97553284	97560698	-	1393	1	LOC10192	0.019	0.079
NR_03384	chr10	44811024	44959689	-	3292	1	TMEM72-A	0.623	0.695
NR_12539	chr3	1.55E+08	1.55E+08	+	402	1	LINC01487	0	0
NM_0323	chr1	1.8E+08	1.81E+08	-	1606	1	ACBD6|-|-	44.373	39.957
NM_0181	chr3	5122245	5180916	+	2976	1	ARL8B|AR	14.639	18.102
NM_0010	chr17	42562074	42566281	+	2284	1	COASY|DP	28.18	32.03
NM_2073	chr19	58451604	58457832	+	2989	1	ZNF324B|	4.214	4.67
NM_1825	chr18	72536680	72544488	-	2887	1	CBLN2|-|-	0	9.033
NM_0059	chr11	92969623	92982782	+	1658	1	MTNR1B|	0	0.05
NR_10460	chr15	90249532	90259088	+	1791	1	TTLL13P|T	0.353	0.749
NM_0012	chr11	6448613	6473984	-	2695	1	TRIM3|BE	16.903	20.756
NM_0012	chr8	42416462	42541512	-	3612	1	SLC20A2|	6.911	8.407
NM_0150	chr6	43182184	43224587	+	7728	1	CUL9|H7A	8.749	7.403

NR_03410	chr5	53109842	53115126	+	2367	1	LOC25739	0.703	0.619
NR_00129	chr15	25086526	25086619	+	93	2	SNORD116	0	0.147
NM_0177	chr8	38316417	38382272	-	3983	1	NSD3|KMT	18.707	20.107
NM_0012	chr22	17359949	17558155	+	9635	1	CECR2|-|-	14.739	12.389
NM_0230	chr13	1.14E+08	1.14E+08	+	2374	1	UPF3A|HU	6.311	6.318
NM_0046	chrY	20575711	20593154	+	1395	1	EIF1AY|eIF	10.269	11.949
NM_0055	chr18	6941744	7117814	-	9592	1	LAMA1|LA	3.141	1.828
NM_0010	chr1	1.54E+08	1.54E+08	+	1025	1	HAX1|HCL	82.933	85.536
NR_04857	chr10	73730562	73731771	+	894	1	GLUD1P3|	1.817	1.118
NM_0064	chr4	1.51E+08	1.51E+08	+	2766	1	MAB21L2|	28.579	13.791
NM_0050	chrX	15790451	15823259	+	1498	1	ZRSR2|U2A	4.691	4.243
NM_0207	chr12	62643984	62934885	-	6187	1	PPM1H|AI	3.032	2.778
NM_0010	chr11	58203204	58204181	-	976	1	OR1S2|OR	0	0
NR_12030	chr16	83797236	83803976	-	540	1	LOC10272	0	0
NR_04541	chr4	3063246	3074514	-	737	1	HTT-AS|HT	0	0.037
NM_1523	chr12	48482503	48496514	+	878	1	C12orf54|	0.088	0.031
NM_0011	chrX	1.34E+08	1.34E+08	-	2147	1	GPC3|DGS	5.153	5.888
NM_0010	chr19	38836388	38852339	-	1866	1	HNRNPL|H	93.011	82.263
NM_0049	chr7	1.49E+08	1.49E+08	-	2940	1	PDIA4|ERP	56.451	49.156
NM_0010	chr1	98890245	99004893	-	3992	1	PLPPR5|LP	3.708	3.397
NM_0167	chr20	33993652	34083185	+	4761	1	RALY|HNR	69.526	51.635
NM_0065	chr6	43576141	43620523	+	8399	1	POLH|RAD	2.175	2.221
NM_0031	chr2	54456317	54671446	+	10183	1	SPTBN1|EI	59.355	72.364
NM_0013	chr16	81037182	81047346	+	4800	1	ATMIN|AS	16.662	21.975
NM_0571	chr2	2.05E+08	2.06E+08	+	7943	1	PARD3B|A	1.643	2.578
NR_13525	chr14	81221218	81223380	+	2048	1	LOC10192	0.667	0.976
NM_0247	chr2	44361904	44772592	+	1542	1	CAMKMT|	6.38	5.516
NM_0160	chr6	1.66E+08	1.66E+08	-	966	1	MPC1|BRP	39.081	36.242
NM_0010	chr1	12847377	12848756	-	1166	1	HNRNPCL1	1.913	3.477
NM_0010	chr11	55367884	55368918	+	1033	1	OR4A15|C	0	0
NM_0011	chr16	85611423	85676206	+	7187	1	GSE1|CRH	26.989	37.59
NM_0011	chr19	34734575	34742869	+	2942	1	ZNF181|H	1.521	2.165
NM_0122	chrX	1.1E+08	1.1E+08	-	1466	1	KCNE5|KC	0.123	0.047
NM_0159	chr13	20403667	20525873	-	1491	1	CRYL1|GD	14.026	18.235
NR_03840	chr6	38906495	38953107	-	2120	1	LOC10013	0	0
NR_02698	chr16	35477414	35480596	-	1817	1	FRG2DP|-|	0	0
NM_0324	chr12	6638076	6647414	-	1874	1	ACRBP|CT	2.972	2.799
NM_0251	chr1	1.17E+08	1.17E+08	-	3573	1	TRIM45|R	3.959	3.856
NM_0070	chr3	58306245	58320193	+	3258	1	RPP14|HsI	8.422	9.951
NR_04003	chr19	29002555	29013955	+	1943	1	LINC01532	0.013	0
NM_0049	chr13	99294535	99307495	-	1781	1	GPR183|E	0	0
NM_0025	chr14	26445883	26597754	-	3911	1	NOVA1|No	35.097	29.436
NR_03884	chr1	1.13E+08	1.13E+08	-	2169	1	LOC64344	0	0
NM_1387	chr5	1.11E+08	1.11E+08	+	4787	1	SLC25A46|	4.42	5.145
NR_03338	chrY	2609265	2657229	+	2906	2	CD99P1|C	0	0
NM_0177	chr15	69298955	69407637	+	5175	1	PAQR5|MI	0.132	0.048
NM_0010	chr7	1.44E+08	1.45E+08	-	2282	1	TPK1|HTPI	0.35	0.474
NM_0147	chr2	61187455	61470714	-	11228	1	USP34|-|-	6.642	12.953
NR_10806	chr10	25651712	25683385	+	1238	1	LINC00836	0	0
NM_0209	chr10	43077069	43130349	+	5593	1	RET|CDHF	0.247	0.296

NM_0010:	chr9	77019655	77020953	+	1297	1	FOXB2|bA	0	0.021
NM_0040{	chrX	1.01E+08	1.01E+08	-	1438	1	TIMM8A|[	2.008	2.172
NM_0189{	chr12	1.09E+08	1.09E+08	-	8518	1	SSH1|SSH:	8.763	11.2
NM_0011:	chr14	1.02E+08	1.02E+08	+	4429	1	TECPR2|KI	7.685	8.662
NR_03716	chr6	1.6E+08	1.6E+08	-	1715	1	LOC10012!	1.271	2.851
NM_0010(	chr16	18499924	18562112	-	3888	1	NOMO2|N	1.734	1.758
NM_1826:	chr11	767223	777502	-	4367	1	PDDC1|-|-	21.361	20.848
NM_0162	chr8	27634060	27673020	+	3623	1	SCARA3|A	2.157	4.65
NR_13192	chr6	1.65E+08	1.65E+08	-	1067	1	MEAT6|-|-	0	0.051
NM_0010:	chr22	50530409	50532579	-	961	1	ODF3B|OI	4.575	1.853
NM_0011(	chr1	84643707	84690557	-	5700	1	SSX2IP|AD	1.742	3.561
NR_03787	chrUn_GL(	38786	97454	-	761	1	LOC10023:	0	0
NR_00289	chr9	96946045	97013580	-	781	1	MFSD14C|	0.974	1
NR_13617	chr2	1.05E+08	1.05E+08	+	441	1	LINC01918	0.76	0.963
NM_0052:	chr4	70242616	70251428	+	801	1	CSN3|CNS	0	0
NM_0190{	chr1	6415234	6419919	-	4254	1	HES2|bHLI	1.755	0.869
NM_2014:	chr8	53966554	54022456	-	2713	1	TCEA1|GT	7.637	7.492
NM_0012:	chr20	20034361	20052492	-	4032	1	CRNKL1|C	6.805	7.195
NR_03963	chr2	87629755	87629834	+	79	2	MIR4435-1	0	0
NM_0011	chr12	55901413	55927125	-	1082	1	PYM1|PYN	30.994	32.128
NM_0331{	chr19	49047638	49049111	-	882	1	CGB8|-|-|	0.088	0.062
NM_0011	chr1	1.13E+08	1.14E+08	+	6513	1	MAGI3|M/	4.417	5.276
NM_0047:	chr18	31622246	31684723	-	4803	1	B4GALT6|I	1.149	1.494
NR_02764	chr17	58238426	58268518	+	2879	1	LPO|SPO|-	0.027	0.029
NM_1390:	chr8	1.44E+08	1.44E+08	+	1872	2	MAPK15|E	0	0
NM_0582:	chr22	45920366	45977128	-	3908	1	WNT7B|-|	4.355	15.425
NM_2069:	chr6	43305473	43308792	-	947	1	CRIP3|TLP	0.599	0.564
NR_03976	chrX	16627012	16627085	-	73	1	MIR548AN	0	0
NR_12211	chr14	19062377	19107497	+	4134	1	LINC01296	0	0
NM_2032!	chr10	73782050	73783648	+	839	1	CHCHD1|C	44.889	38.038
NM_0162	chr13	42272153	42323267	+	9905	1	AKAP11|A	5.591	7.112
NM_0011	chr15	89664370	89679417	-	2881	1	PLIN1|FPL	0.143	0.076
NM_0200:	chr22	23573126	23580308	-	895	1	IGLL1|14.1	0	0.077
NM_0011	chr1	1.47E+08	1.47E+08	-	1970	1	FMO5|-|-|	0.124	0.118
NR_10693	chr11	65018505	65018570	+	65	1	MIR6879|I	14.084	13.697
NR_13568	chr15	66984207	67064182	-	1824	1	LOC10272:	0.042	0.015
NM_0010:	chr2	1E+08	1E+08	+	473	1	NMS|-|-|2	0.382	0.058
NR_13357	chr11	1.01E+08	1.01E+08	-	1864	1	LOC10012:	0.035	0.162
NR_12041	chr13	79872586	79918036	+	976	1	LINC00382	0	0
NR_03747	chr12	1.24E+08	1.24E+08	+	125	1	MIR3908|-	0	0
NR_03823	chr16	73386805	73421396	+	1325	1	LINC01568	0	0
NR_01543	chr20	18567429	18569559	+	490	1	LINC00493	71.704	51.797
NM_1533:	chr3	1.28E+08	1.28E+08	-	2470	1	DNAJB8|C	0.01	0
NM_0011(	chr2	2.07E+08	2.07E+08	+	1112	1	LOC20072(	0	0
NR_13627	chr7	26372019	26399418	-	626	1	LOC10537:	51.141	27.985
NR_14429	chr2	98087132	98236805	+	2555	1	VWA3B|S(	0.202	0.086
NM_0012	chr6_GL0(	4486078	4493809	-	3126	5	RGL2|HKE	0	0
NM_0010	chr8	7495846	7509311	+	385	3	DEFB107B	0	0
NM_2016	chr2	73828916	73867168	+	6265	1	STAMBP|/	10.488	10.627
NR_13569	chr15	62060503	62062434	+	1806	1	LOC10192:	0.057	0.046

NR_13423	chr4	21582096	21613722	+	711	2	LOC10537	0	0
NM_0013	chr7	72959485	72966953	-	1282	2	TRIM74|TI	0.241	0.15
NM_0060	chr20	49903391	49915543	-	4052	1	SPATA2|PI	6.51	7.677
NR_12585	chr6	10932317	10954089	-	1028	1	LOC10192	0.05	0.053
NM_0012	chr19	18058995	18087003	-	2750	1	IL12RB1|C	0.009	0.01
NR_10807	chr10	1.33E+08	1.33E+08	-	665	1	MIR202HG	0	0
NM_0021	chr6_GL0C	4224964	4229249	-	1364	6	HLA-DOB|	0	0
NR_10350	chr3	14402576	14489349	+	6391	1	SLC6A6|TA	8.382	7.261
NM_1738	chr19	55226634	55229264	-	1746	1	TMEM86B	5.708	6.362
NM_0011	chr12	1.1E+08	1.1E+08	-	1595	1	GPN3|ATP	7.065	7.445
NM_0068	chr18	9708231	9862556	+	3985	1	RAB31|Ra	9.959	14.993
NM_0011	chr3	1.31E+08	1.31E+08	+	8432	1	COL6A6|-|	0.02	0.008
NM_0807	chr22	22513736	22520281	-	2154.5	2	ZNF280A|	0	0
NM_0043	chr10	99710868	99732666	-	4319	1	COX15|CE	9.517	8.785
NR_10679	chr1	43364648	43364715	-	67	1	MIR6734|	0.77	0.409
NM_1720	chr2	43637300	43767987	+	6922	1	PLEKHH2|	0.143	0.41
NM_0245	chr4	1.21E+08	1.21E+08	-	2865	1	NDNF|C4c	1.616	2.519
NR_03960	chr17	80098828	80098877	+	49	1	MIR1268B	0	0
NR_07345	chr17	10729777	10768030	+	598	1	TMEM220	0.172	0
NM_0010	chr1	56718804	56819696	-	3438	1	C1orf168|	0.041	0.064
NM_0011	chr15	76216288	76311469	-	1194	1	ETFA|EMA	50.624	59.847
NM_0011	chr6	7881250	7910077	-	3183	1	TXNDC5|E	13.858	15.255
NM_0013	chr11	4598672	4608259	-	2861	1	TRIM68|G	7.427	10.604
NR_03744	chr16	2270713	2270772	+	59	1	MIR3677|	0.437	1.161
NM_0309	chr20	38926312	38953061	+	2424	1	FAM83D|C	2.495	3.351
NM_0011	chr4	83292461	83335153	-	4417	1	HPSE|HPA	0.023	0.034
NM_0012	chr1	27891526	27914797	-	1600	1	RPA2|REP	36.07	33.215
NM_0010	chr3	1.52E+08	1.52E+08	+	1599	1	AADAC|CE	0	0
NR_02440	chr9	1.21E+08	1.21E+08	+	3794	1	PSMD5-AS	4.985	4.906
NR_04750	chr13	38050817	38143232	-	515	1	LINC00571	0.15	0
NM_0010	chr9	1.37E+08	1.37E+08	+	2929	1	C9orf172|	5.894	4.873
NR_02983	chr7	1E+08	1E+08	-	81	1	MIR106B|	0.955	0
NM_1993	chr2	24114809	24123477	-	1529	1	PFN4|-|-|	0.059	0.116
NR_13118	chr10	68009947	68010883	-	936	1	POU5F1P5	0	0
NM_0002	chr14	23412738	23435686	-	6013	1	MYH7|CM	2.191	2.207
NR_03164	chr2	2.12E+08	2.12E+08	-	97	1	MIR548F2	0	0
NR_03795	chr5	33986986	34124528	-	3603	1	C1QTNF3-	6.803	4.676
NR_12041	chr13	90493288	90535341	+	996	1	LINC0104	0	0
NR_03794	chr7	27162438	27180261	-	2107	1	HOXA10-H	0	0.013
NR_13458	chr18	36901945	36923814	-	685	1	LOC10537	0	0
NM_1816	chr21	44666189	44666927	-	736	1	KRTAP12-	0	0
NM_0011	chr19	17334982	17342731	+	2584	1	GTPBP3|C	14.595	11.916
NM_0012	chr17	18952165	19020691	+	1968	1	SLC5A10|	0.17	0.097
NM_0010	chr11	61203513	61212869	+	1375	1	PGA3|-|-|	0	0
NM_0245	chr7	93232340	93276425	+	1176	1	VPS50|CC	2.609	4.146
NM_1384	chr5	58583044	58859396	+	8887	1	RAB3C|-|-	13.887	8.354
NM_0013	chr13	49444293	49528991	+	3688	1	SETDB2-PH	1.556	1.207
NM_0012	chr1	43358955	43363202	+	1661	1	CDC20|CD	11.675	10.069
NM_1524	chr17	28357581	28363683	+	3140	1	TMEM199	20.363	21.671
NM_0142	chr19	54738517	54784324	+	1556	35	KIR2DL2|C	0	0

NR_03063	chr8	1.44E+08	1.44E+08	-	85	2	MIR937|M	0	0
NM_0248	chr8	67952115	68237033	+	10714	1	PREX2|DE	0.638	1.028
NR_03022	chrX	50014598	50014683	+	85	1	MIR502|M	0	0
NR_10678	chr1	24919345	24919416	-	71	1	MIR6731|	0	0
NR_00393	chr4	55820071	55837264	+	385	1	LOC64414	0	0
NR_03384	chr3	1.68E+08	1.68E+08	+	2670	1	LINC0133	1.4	0.246
NM_0069	chr12	85280258	85301783	+	1287	1	ALX1|CAR	0.19	0.138
NM_0207	chr1	1.52E+08	1.52E+08	+	5078	1	CGN|-|-|1	0.84	1.103
NM_0013	chr2	26308173	26346817	-	4495	1	ADGRF3|G	0.178	0.18
NR_00215	chrY	18581206	18590521	-	1632	2	TTTY9B|N	0	0
NR_00321	chr14	1.01E+08	1.01E+08	+	70	1	SNORD11	0	0
NM_0010	chr15	1.02E+08	1.02E+08	+	937	1	OR4F15|-|	0	0
NR_02958	chr1	1.1E+08	1.1E+08	+	74	1	MIR197|M	0	0.185
NM_0122	chr22	24011315	24178628	+	7183	1	CABIN1|C	20.077	18.436
NM_0001	chrX	1.34E+08	1.35E+08	+	1404	1	HPRT1|HG	8.834	8.926
NM_2013	chr1	1.55E+08	1.55E+08	+	1785	1	FLAD1|FA	31.587	31.921
NR_10506	chr5	1.49E+08	1.49E+08	+	934	1	CARMN|C	0	0
NR_10407	chr1	1.44E+08	1.44E+08	-	163	1	RNVU1-17	0	0
NR_04611	chr1	43363397	43368074	-	1452	1	ELOVL1|C	6.065	7.801
NM_0062	chr2	1.09E+08	1.09E+08	+	11680	1	RANBP2|A	2.898	4.541
NR_03990	chr19	4445978	4446048	+	70	1	MIR4746|	0	0.978
NR_00366	chr2	84290682	84292200	+	1518	1	FUNDC2P2	0.399	0.442
NM_1472	chr6	1.12E+08	1.12E+08	-	2446	1	TRAF3IP2|	1.249	1.579
NM_0041	chr9	1.33E+08	1.33E+08	+	2452	1	GFI1B|BD	0.016	0.017
NM_0153	chr2	97756338	97995891	-	6599	1	TMEM131	7.032	12.354
NR_00349	chr15	25220503	25220578	+	75	1	SNORD11	0	0
NM_1784	chr1	1.51E+08	1.51E+08	-	4819	1	ARNT|HIF-	11.936	11.133
NM_0055	chr6	1.61E+08	1.61E+08	-	6447	1	LPA|AK38	0.004	0.036
NM_0312	chr1	36455761	36464439	-	955	1	MRPS15|D	49.076	40.675
NM_0246	chr7	1.02E+08	1.02E+08	+	2173	1	PRKRIP1|C	24.553	26.701
NM_0011	chr19	46774883	46787288	-	1727	1	SLC1A5|A	18.568	21.152
NM_1774	chr10	1.33E+08	1.33E+08	-	1014	1	NKX6-2|G	4.311	1.243
NR_02806	chr15	22125511	22126444	+	933	1	OR4N3P|C	0	0
NR_00145	chr1	44778390	44778458	+	68	1	SNORD38	1.896	2.014
NR_10689	chr7	44073378	44073433	-	55	1	MIR6838|	7.502	2.988
NR_12062	chr10	933026	942743	+	404	1	LOC10192	0.128	0.068
NM_1758	chr12	52821447	52834293	-	2055	1	KRT79|K6	0	0
NM_0020	chr17	44905626	44915552	-	3075	1	GFAP|ALX	0.725	4.192
NM_0012	chr11	797511	799185	+	1672	1	PANO1|PA	1.704	1.524
NR_04981	chr15	89850637	89850721	-	84	1	MIR5094|	0	0
NM_0010	chr13	1.07E+08	1.08E+08	-	3854	1	FAM155A|	6.219	2.69
NR_00280	chr12	1.22E+08	1.22E+08	-	1514	1	LINC0108	17.33	14.122
NR_03790	chr10	97153042	97292673	-	1901	1	ARHGAP1	8.37	8.156
NM_0045	chr19	40750854	40765392	+	1621	1	SNRPA|M	44.598	46.465
NM_0010	chr22	48489460	48751935	+	2614	1	FAM19A5|	6.146	33.32
NR_10500	chr8	76406654	76524332	+	1633	1	LINC01111	0	0.017
NM_0072	chr12	56994571	56996685	+	1566	1	GPR182|7	0.016	0.096
NM_2058	chr2	2.34E+08	2.34E+08	+	1683	1	UGT1A6|G	0.046	0
NM_0011	chrX	1.55E+08	1.55E+08	+	2559	1	DKC1|CBF	17.746	20.784
NR_00305	chr9	1.31E+08	1.31E+08	+	85	2	SNORD62	1.82	3.706

NR_03972	chr14	36952309	36952391	-	82	1	MIR4503|	0	0
NM_0024	chr4	76001342	76007523	-	2702	1	CXCL9|CM	0	0
NM_1458	chr11	799179	805250	-	2939	1	PIDD1|LRI	6.98	5.611
NM_0007	chr15	74337762	74367740	-	1920	1	CYP11A1|	0	0
NM_0056	chr8	1.03E+08	1.03E+08	-	3093	1	KLF10|EGI	11.551	12.448
NR_11090	chr16	4324648	4328340	-	355	2	GLIS2-AS1	0	0
NR_03378	chr1	1.2E+08	1.2E+08	+	8696	1	HSD3BP4|	0.006	0.011
NM_0027	chr17	21288036	21315239	+	2046	1	MAP2K3|	7.884	10.075
NR_02739	chr11	60937083	60951785	-	1940	1	SLC15A3|	5.357	5.239
NM_0010	chr2	2.18E+08	2.18E+08	+	1491	1	GPBAR1|B	0.458	0.22
NM_0013	chr2	2.29E+08	2.29E+08	-	2445	1	PID1|HMI	9.761	27.422
NM_0010	chr11	809936	812876	+	486	1	RPLP2|D1	732.275	548.303
NM_0223	chr2	1.09E+08	1.09E+08	-	4200	1	EDAR|DL|	0.123	0.007
NM_1736	chr7	1.5E+08	1.5E+08	+	2270	1	ZNF775|-|	22.055	18.204
NM_1392	chr9	1.13E+08	1.13E+08	-	863	1	CDC26|AN	2.092	2.079
NM_0132	chrX_KI27	6753	59448	-	2411	4	PPP2R3B|	0.171	0.176
NM_0013	chr9	1.09E+08	1.09E+08	-	5934	1	IKBKAP|D	14.486	12.148
NM_0013	chr16	30650702	30656440	+	2028	1	PRR14|-|-	36.354	34.817
NM_0013	chr3	1.01E+08	1.01E+08	+	2306	1	ADGRG7|	0.034	0
NM_1781	chr10	23192531	23194252	+	1329	1	PTF1A|PA	6.015	13.975
NR_10688	chr3	1.96E+08	1.96E+08	-	66	1	MIR6829|	4.298	4.566
NR_03817	chr14	65412730	65744121	+	4238	1	FUT8|-|-|	10.679	10.74
NM_0013	chr17	68206129	68257164	+	1206	1	AMZ2|-|-|	64.008	60.228
NM_0038	chr12	1.17E+08	1.17E+08	-	1495	1	HRK|DP5|	2.139	6.047
NM_0321	chr22	28917389	29057488	+	6459	1	ZNRF3|BK	6.935	3.963
NR_03167	chr13	1.08E+08	1.08E+08	-	77	1	MIR1267|	0	0
NM_0010	chr4	13367723	13484365	-	1707	1	RAB28|CO	12.38	10.776
NM_0018	chr9	89311195	89316703	+	611	1	CKS2|CKS	34.017	22.014
NM_0064	chr11	20363685	20383783	+	1365	1	HTATIP2|	0.633	0.482
NR_12635	chr22	35119824	35231056	-	642	1	LINC0139	2.109	1.621
NR_04655	chr3	29926811	29934156	-	551	1	RBMS3-AS	0	0
NM_1758	chr17_GL	941109	943291	+	2180	3	SPPL2C|IM	0	0
NM_0332	chr10	86654557	86666459	+	2360	1	OPN4|MO	0	0
NR_13529	chr3	1.12E+08	1.12E+08	-	3190	1	SLC9C1|N	0	0
NM_0011	chr19	37696363	37719188	-	4006	1	ZNF607|-|	3.634	3.706
NM_0011	chr14	77426675	77457640	-	2548	1	VIPAS39|	16.891	27.061
NM_0010	chr12	92420094	92428148	-	680	1	CLLU1OS|	0	0.081
NM_0012	chr12	48688458	48716998	-	6905	1	CCNT1|CC	7.077	7.274
NM_1749	chr14	77131270	77141791	-	3403	1	ZDHHC22|	18.141	20.318
NR_10804	chr8	1.24E+08	1.24E+08	-	356	1	RNF139-A	1.05	1.231
NM_0012	chr12	43728609	43758817	-	3041	1	PUS7L|-|-	1.213	1.748
NM_1755	chrX	2752052	2816501	+	2875	1	XG|PBDX|	0	0.005
NR_02707	chr21	29193494	29288205	+	987	1	LINC0018	0	0
NM_0153	chr19	35612744	35625349	+	4288	1	HAUS5|KI	4.195	4.009
NR_00357	chr16	2340922	2426699	+	4171	1	ABCA17P|	0.136	0.289
NR_13454	chr7	73593194	73624543	-	3114	1	MLXIPL|C	1.263	0.361
NR_04986	chr19	12787128	12787192	+	64	1	MIR5684|	0	0
NM_0012	chr11	62676498	62679117	-	1169	1	UBXN1|2B	134.218	129.799
NM_1759	chr5	41904344	41921636	+	5238	1	C5orf51|-|	4.514	5.627
NM_0133	chr9	1.31E+08	1.32E+08	+	11026	1	PRRC2B|B	119.123	144.47

NR_02957	chr11	64891137	64891246	-	109	1	MIR192|M	0	0
NM_0054	chr17	44076753	44123646	-	5295	1	HDAC5|HD	39.111	47.007
NM_0011	chr22	50147055	50161687	+	1331	1	MOV10L1|	0.194	0.278
NM_1523	chr2	1.69E+08	1.7E+08	+	3143	1	BBS5|-|-|	2.096	1.403
NM_0010	chr17	74203656	74209880	+	350	1	RPL38|L38	814.684	659.883
NM_0013	chr1_KI27	118715	123068	+	1934	4	PRAMEF25	0	0
NR_11026	chr2	1.66E+08	1.66E+08	+	2292	1	LOC10192	0.203	0.173
NM_0069	chr10	1.02E+08	1.02E+08	-	869	1	NPM3|PO	17.152	15.573
NM_1393	chr19	3777969	3786417	-	1900	1	MATK|CH	0.977	2.617
NM_0154	chr4	1.13E+08	1.13E+08	+	2149	1	LARP7|AL	5.73	7.731
NM_0066	chr11	86441108	86672385	-	2071	1	ME3|NAD	0.218	0.463
NM_0011	chr5	1.74E+08	1.74E+08	+	2515	1	C5orf47|-|	0.01	0.027
NM_0010	chr1	19596977	19629821	+	3720	1	MINOS1|C	10.211	10.512
NR_04633	chr9	1.37E+08	1.37E+08	+	1051	1	STPG3|C9	0.491	0.182
NR_13391	chr14	19083134	19098320	-	663	3	BMS1P22|	0	0
NM_0036	chr6	1.43E+08	1.43E+08	+	2758	1	PEX3|PBD	2.852	3.059
NM_0038	chr1	74198212	74208702	+	4716	1	FPGT|GFP	0.779	0.531
NM_1732	chr16	28878488	28904509	+	3470	1	ATP2A1|A	1.3	0.955
NM_0009	chr1	20070208	20091901	+	1887	1	PLA2G5|F	0	0.029
NM_0011	chr10	1.33E+08	1.33E+08	-	3006	1	ADAM8|C	1.484	1.677
NM_0319	chr17	41226648	41227652	+	987.3	3	KRTAP9-2|	0	0
NR_00153	chrY	18581206	18590521	-	1632	2	TTTY9A|N	0	0
NM_0013	chr14	57563922	57865874	-	1710	1	SLC35F4|C	0.95	0.817
NM_0011	chr18	7231139	7232044	+	904	1	LRRC30|-|	0	0
NM_0023	chr15	43517608	43531620	+	10267	1	MAP1A|M	46.459	32.66
NM_0133	chr2	2.19E+08	2.2E+08	+	1540	1	GMPPA|A	12.751	13.368
NR_03893	chr4	1.87E+08	1.88E+08	-	2427	1	LOC33997	0.011	0.023
NM_0149	chr10	1.18E+08	1.18E+08	-	6054	1	RAB11FIP2	2.566	3.453
NR_03882	chr4	79492593	79576460	+	2050	1	LINC0098	0.226	0.147
NR_10702	chr20	7371608	7371692	+	84	1	MIR8062|	0	0
NM_0529	chr2	23385427	23708613	+	5068	1	KLHL29|K	2.959	8.854
NM_0033	chr4	48066393	48134256	-	2897	1	TXK|BTKL|	0.009	0.057
NM_0010	chr4	76894929	76897849	-	2919	1	SOWAHB|	0.212	0.601
NM_0212	chr14	73058513	73121368	+	4282	1	RBM25|N	10.933	16.262
NM_0013	chr19	51991334	52008230	-	4014	1	ZNF615|-|	1.6	2.341
NM_0156	chr10	84231520	84241461	-	2295	1	LRIT1|FIG	0.022	0
NM_0248	chr10	73644881	73651039	-	4127	1	SYNPO2L|	0.119	0.043
NR_13502	chr12	87330739	87332321	+	717	1	LOC10536	0	0
NM_0012	chr11	74171063	74254703	+	2739	1	PPME1|P	50.694	54.418
NM_0012	chr6	15248855	15522042	+	5577	1	JARID2|J	19.91	20.77
NM_0013	chr8	28891316	29053270	+	3586	1	HMBOX1|	2.582	2.891
NR_11059	chr2	1.77E+08	1.77E+08	+	652	1	LINC01117	0	0
NM_0010	chr19	12390014	12401274	-	2582	1	ZNF799|H	0.619	1.04
NM_0010	chr18	59220168	59230774	+	827	1	GRP|BN|G	0	0
NM_0010	chr9	35809810	35812262	-	1692	1	SPAG8|BS	1.356	0.85
NM_0011	chr7	6496778	6551436	-	4609	1	GRID2IP|D	0.325	0.363
NM_0012	chr11	1834310	1837521	+	1544	1	SYT8|-|-|1	0.017	0.035
NM_0012	chr7	2728106	2844325	-	4329	1	GNA12|N	35.657	40.547
NM_0019	chr16	67192165	67198918	+	2088	1	E2F4|E2F-	26.17	26.797
NM_0012	chr7	1.39E+08	1.39E+08	+	2734	1	C7orf55-L	38.657	39.017

NR_02960	chr9	83969748	83969857	-	109	1	MIR7-1|M	0.118	0.377
NM_0016(	chr3	49359136	49412097	-	1928	1	RHOA|ARH	240.322	202.443
NM_0306:	chrX	1.03E+08	1.03E+08	+	3981	1	BHLHB9|G	4.894	6.007
NR_13804	chr11	450280	491400	+	1739	2	PTDSS2|PS	1.802	1.095
NM_0011!	chr5	96774484	96814145	-	4822	1	ERAP1|A-L	1.874	1.446
NM_0048:	chr9	1.3E+08	1.3E+08	-	1782	1	PTGES|MG	2.815	4.412
NR_03385	chr9	64016950	64061612	+	2514	1	FLJ43315|	0	0
NM_0324:	chr19	14690058	14720960	+	3753	1	ZNF333|-|	7.462	9.785
NM_0011:	chr14	64504574	64524951	+	3834	1	ZBTB1|ZNI	2.61	4.087
NR_13525	chr10	73497538	73553340	-	5512	1	USP54|C1(	9.558	10.144
NM_0012{	chr15_KI2	2888969	2902703	-	5169.5	2	GOLGA8K|	0.005	0.008
NR_13123	chr2	1.05E+08	1.05E+08	-	604	1	LINC01158	66.794	106.424
NM_0203:	chr12	54362445	54364486	-	1505	1	GPR84|EX	0.026	0.064
NM_0220(	chr19	35143250	35154302	+	705	1	FXYD7|-|-	32.956	32.271
NM_0010(	chr19	56567521	56582894	+	7133	1	ZNF470|C	1.945	1.711
NM_0013:	chr21	29536933	29940052	-	3125	1	GRIK1|EA/	6.696	11.777
NM_0013:	chr11	76784148	76798154	+	2571	1	TSKU|E2IG	7.583	12.12
NM_0032:	chr22	41381929	41399328	+	4390	1	TEF|-|-|22	4.497	6.508
NM_0011(	chr9	1.33E+08	1.33E+08	-	8439	1	TSC1|LAM	8.985	10.487
NM_0212:	chr19	35799990	35813299	-	1664	1	PRODH2|I	0.031	0.033
NM_0165-	chr2	24943636	24972094	-	4983	1	DNAJC27|	1.866	1.996
NM_0325:	chr20	34558697	34560345	+	979	1	MAP1LC3A	0.606	7.905
NM_0025(	chr8	1.01E+08	1.01E+08	-	2843	1	PABPC1|P.	84.155	101.964
NM_0063:	chr13	1.12E+08	1.13E+08	-	2758	2	TUBGCP3|	5.021	5.801
NM_0246-	chr6	95577497	95609452	+	4609	1	MANEA|EI	0.747	0.832
NM_0209-	chr9	1.23E+08	1.24E+08	-	5017	1	DENND1A	12.935	14.066
NR_02963	chr4	20528275	20528384	+	109	1	MIR218-1|	0	0
NR_00299	chr17	60231516	60231645	-	129	1	SCARNA20	0	0
NM_0012{	chr22	42553862	42574382	+	1227	1	SERHL2|dJ	0.977	0.67
NM_0013(	chr1	1E+08	1E+08	-	3939	1	SASS6|MC	0.409	0.577
NM_0012-	chr4	9325165	9326757	+	1591	8	USP17L24	0	0
NM_0058:	chr3	49828595	49856559	-	2007	1	TRAIP|RNI	3.476	3.126
NM_0010:	chrX	1.36E+08	1.36E+08	+	1020	1	CT45A1|C	0	0
NM_0061(	chr18	2571511	2616635	+	2153	1	NDC80|HE	1.252	2.01
NM_0010:	chr12	98515573	98550379	+	3846	1	TMPO|CM	21.751	17.436
NM_0804:	chr1	1.54E+08	1.54E+08	+	1783	1	AQP10|AC	0.014	0
NR_12039	chr8	1.35E+08	1.35E+08	+	1198	1	LINC01591	0	0.011
NM_0008{	chr12	53191318	53207307	-	2849	1	ITGB7|-|-|	0.308	0.183
NM_0323-	chr6	33696761	33711751	-	1332	1	UQCC2|C6	35.331	36.998
NM_0013:	chr12	6539528	6556083	-	2678	1	IFFO1|HOI	11.411	10.158
NM_0011{	chr11	46701094	46705916	+	2205	1	ZNF408|E\	8.555	9.442
NM_0246{	chr1	83869374	83999150	-	3625	1	TTLL7|-|-|	2.458	2.875
NM_0010(	chr11	5422111	5423206	+	1093	1	OR51Q1|-	0	0
NR_10401	chr7	75410322	75416792	+	1873	1	NSUN5P1|	1.212	1.389
NM_1759:	chr16	88874855	88941200	-	4025	1	CBFA2T3|I	20.277	14.619
NR_10685	chr19	12852260	12852327	+	67	1	MIR6794|	13.086	8.382
NM_0149:	chr3	1.73E+08	1.74E+08	+	4938	1	NLGN1|NL	3.217	4.754
NR_02835	chr9	96909075	96910455	+	1380	1	LOC44145	0.065	0.099
NR_00290	chr1	28505943	28510892	+	2235	1	SNHG3|NC	4.627	7.262
NM_0013(	chr19	15851994	15864904	-	444	1	LINC01835	0	0

NR_10698	chr10	30368597	30368665	-	68	1	MIR7162|I	0	0.201
NM_0011:	chr5	1.76E+08	1.76E+08	+	2549	1	HRH2|H2F	0.081	0.414
NM_0012	chr2	2.19E+08	2.19E+08	+	4843	1	STK36|FU|	10.127	8.422
NM_0031(	chr2	5692667	5701385	+	8716	1	SOX11|MF	34.608	62.535
NM_0806(	chr7	1.39E+08	1.39E+08	-	1746	1	ZC3HAV1L	0.369	0.275
NR_02771	chr6	7986102	7990344	+	4242	1	PIP5K1P1|	0.131	0.2
NR_11000	chr14	22929609	22954692	+	553	1	LOC10192	0.117	0.05
NM_0011!	chr8	1.27E+08	1.27E+08	+	1582	1	POU5F1B|	0.603	0.381
NM_0013:	chr2	74414176	74421630	-	1919	1	C2orf81|h	6.611	3.697
NM_0219!	chr6_GL0C	1366074	1369250	+	1657.1	8	PPP1R11|(	4.816	4.653
NM_0011(	chrX	1.01E+08	1.01E+08	-	3092	1	TRMT2B|C	3.115	3.517
NM_0013:	chr2	95849548	95992320	-	7981	1	ANKRD36C	0.325	0.535
NM_0011:	chr2	37105141	37147822	-	3665	1	EIF2AK2|E	2.153	3.999
NM_0013:	chr2	85558725	85561509	-	641	1	GGCX|VKC	10.721	9.765
NR_03718	chr16	27268205	27290468	+	596	1	FLJ21408|	0.043	0.092
NM_0208(	chr19	39306817	39315336	-	3129	1	LRFN1|SAI	11.183	13.548
NR_11029	chr11	95230186	95234404	+	1954	1	LOC10012!	0.165	0.217
NM_0050:	chr2_KI27(	61979	71004	-	2105	2	PDCD1|CD	0	0
NM_0154	chr10	1.02E+08	1.02E+08	+	846	1	DPCD|-|-|	41.942	22.084
NM_0210:	chr13	97434221	97467998	+	4354	1	RAP2A|K-F	8.366	13.911
NM_0328(	chrX	1.53E+08	1.53E+08	+	2274	1	PNMA6A|I	1.514	1.891
NR_03029	chr3	1.14E+08	1.14E+08	-	94	1	MIR568|M	0.549	0.874
NM_0071:	chr8	37736579	37757801	+	4807	1	ERLIN2|C8	12.942	11.004
NM_0012!	chr14	69398668	69462391	+	2081	1	SLC39A9|Z	24.071	26.624
NM_0010(	chr11	5351508	5352446	+	937	1	OR51B6|H	0	0
NM_0333:	chr19	49035569	49036934	-	727	1	CGB1|-|-|	0	0
NR_10693	chr1	1.5E+08	1.5E+08	+	65	1	MIR6878|I	1.984	0.843
NM_0049:	chr16	89707133	89720986	-	2732	1	VPS9D1|A	19.359	20.049
NM_0011	chr7	72574507	72828234	-	3141	1	TYW1B|LII	1.667	1.478
NM_0228:	chr1	1435523	1442882	+	4654	1	VWA1|WA	4.665	4.132
NM_2144(	chr2_KI27(	98954	105653	-	1320.5	2	C2orf27B|	0	0
NM_0006:	chr8	30678063	30727969	-	3145	1	GSR|HEL-7	14.927	13.741
NR_03834	chr18	73324941	73349889	+	1173	1	LOC10050	0	0
NM_0807:	chrX	1.55E+08	1.55E+08	+	514	3	H2AFB3|H	0	0.107
NM_0013:	chr2	2.31E+08	2.31E+08	-	1843	1	HTR2B|5-H	0.014	0.067
NR_00302	chr11	93730515	93730646	-	131	1	SNORA25|	0.886	1.464
NR_10693	chr20	41169023	41169078	+	55	1	MIR6871|I	6.798	6.724
NM_0010{	chr17	58420167	58488401	-	4103	1	HSF5|HSF	0.053	0.077
NM_0010:	chr19	34590625	34594585	-	3238	1	SCGB2B2|	0.892	0.69
NR_02453	chr3	1.84E+08	1.84E+08	-	1487	1	ALG3|CDG	14.177	11.09
NM_0010(	chrX	1.45E+08	1.45E+08	+	609	1	SPANXN1|	0	0
NM_0012!	chr3	1.54E+08	1.54E+08	+	5237	2	ARHGEF26	0	0
NR_11191	chr10	66894110	67012153	-	3773	1	LOC10192	0	0.004
NM_0010{	chr4	42397839	42402487	+	1967	1	SHISA3|hS	0.059	0.146
NM_0224	chr10	14904611	14954432	-	6227	1	DCLRE1C|/	0.888	1.139
NM_0012	chr6	26440472	26453415	+	2339	1	BTN3A3|B	0.232	1.768
NM_0013(	chr21	42917251	42925598	-	2290	1	ERVH48-1|	0.107	0.078
NR_03416	chr2	2.39E+08	2.39E+08	-	3479	1	LINC0194C	0	0.004
NR_00214	chr1	2.48E+08	2.48E+08	+	644	1	OR2M1P|J	0	0
NM_0011(	chr14	59505067	59576831	-	2588	1	CCDC175|	0.06	0.037

NM_0184:	chr9	97926791	97944915	-	2244	1	HEMGN|C	0	0.024
NR_12540	chr3	1.22E+08	1.22E+08	+	618	1	LOC10272	0.042	0.199
NM_0123!	chr1	1.61E+08	1.61E+08	-	636	1	PFDN2|PF	147.545	146.532
NM_0020;	chr19	51745773	51751897	-	1313	1	FPR1|FML	0	0
NR_00361	chr4	1.83E+08	1.83E+08	+	2454	1	FAM92A1F	0.011	0.028
NM_0011:	chr17	74274247	74314884	+	2224	1	DNAI2|CIL	0.18	0.105
NR_10692	chr17	4969702	4969771	-	69	1	MIR6864|I	0	0.596
NM_0183(	chr19	52612365	52638391	-	2596	1	ZNF83|HP	5.811	5.915
NM_0240	chr16	715173	719655	+	3261	1	METRN|C1	17.432	7.35
NR_12641	chr6	41523895	41547545	-	414	1	FOXP4-AS1	0.623	0.132
NM_0011(	chr17	43847148	43857760	+	988	1	CD300LG|	0	0
NR_04989	chr5	1.19E+08	1.19E+08	+	79	1	MIR5706|	0	0
NM_1748!	chr2	2.3E+08	2.3E+08	+	2809	1	FBXO36|FI	1.987	1.98
NM_0036:	chrX	13734713	13769361	+	3626	1	OFD1|71-7	2.045	2.066
NM_0010:	chr1	54172354	54200073	-	3523	1	CYB5RL|-|	2.738	2.053
NM_0241(	chr22	49903206	49918458	-	2361	1	ALG12|CD	10.878	9.189
NM_0010{	chr19	48485271	48513189	-	4955	1	LMTK3|LN	31.985	36.016
NR_03173	chr10	21496562	21496641	-	79	1	MIR1915|I	0.979	0.347
NR_13566	chr15	21735258	21735999	-	655	2	LOC64213	0	0
NM_0003:	chr1	45012133	45015669	+	1396	1	UROD|PCT	43.834	38.756
NM_0540:	chr11	18172837	18174280	+	1441	1	MRGPRX4	0	0
NM_0011!	chr11	1.18E+08	1.19E+08	+	16556	1	KMT2A|AL	10.352	13.85
NM_0010:	chr9	1.37E+08	1.37E+08	-	1192	1	LRRC26|CA	3.991	3.769
NR_13506	chr11	42001722	42085458	-	1680	1	LOC10192	0	0
NM_0219:	chr1	12019242	12032049	+	1789	1	MIIP|IIP45	21.946	18.597
NR_04552	chr19	23223805	23250370	-	564	1	ZNF724|ZI	0.732	0.68
NM_1387(	chr11	77034341	77041958	+	2541	1	B3GNT6|B	0	0.005
NR_03609	chr4	1.73E+08	1.73E+08	+	73	1	MIR548T|	0	0
NR_02449	chrX	1.35E+08	1.35E+08	-	2737	1	SMIM10L2	7.914	10.93
NM_0010:	chr19	51927435	51945758	+	2331	1	ZNF613|-|	3.037	4.148
NM_0039!	chr19	16888860	16892600	+	3463	1	F2RL3|PAF	0.019	0.016
NR_10998	chr3	51873596	51875767	+	1997	1	IQCF5-AS1	0	0
NM_1533:	chr19	49453216	49471048	+	3087	1	ALDH16A1	8.625	6.394
NM_0012:	chr3	1.84E+08	1.84E+08	+	1321	1	POLR2H|R	36.562	32.692
NM_0012!	chr11	2376177	2397419	+	1369	1	CD81|CVID	164.262	189.487
NM_0195!	chr3	56727418	56801967	-	3590	1	ARHGEF3|	2.866	14.868
NM_0529:	chr18	21529301	21600732	-	4485	1	ESCO1|A9	1.072	2.068
NM_0012{	chr15	90930918	90932569	-	920	1	HDDC3|(p	17.715	21.156
NM_0148(	chr2	27650810	27663840	-	4513	1	SUPT7L|SF	16.273	15.73
NM_0204(	chr11	3665587	3671384	-	1938	1	CHRNA10|	0.632	0.643
NM_0581(	chr2	2.23E+08	2.23E+08	+	1088	1	MOGAT1|	0	0.013
NM_0012!	chr18	35041360	35143468	+	4127	1	MAPRE2|(	48.215	42.206
NM_0012	chrX	52947256	52995472	-	1686	1	FAM156A|	0.061	0.049
NM_0021(	chr4	99948087	99950355	-	940	1	H2AFZ|H2	189.219	187.795
NM_0011:	chr11	66257620	66267860	+	2877	1	KLC2|-|-|1	50.969	54.964
NR_02943	chr14	58265365	58298137	-	2478	1	PSMA3-AS	7.061	7.683
NM_0013:	chr10	68487069	68527523	-	1055	1	SLC25A16|	3.08	3.298
NM_0014:	chr1	2.17E+08	2.17E+08	-	5234	1	ESRRG|ERI	3.202	0.746
NR_11018	chr7	1.24E+08	1.24E+08	-	560	1	LOC10192	0.023	0.098
NM_0053:	chr16	1809103	1827194	-	1503	1	HAGH|GL(	45.629	44.627

NM_0144	chr19	51124880	51130310	+	1709	1	SIGLEC9|C	0	0
NM_0012	chr8	98454631	98825671	-	2474	1	STK3|KRS1	0.672	0.648
NM_1522	chr15	40569293	40574694	+	2111	1	RPUSD2|C	6.682	7.883
NM_0326	chr2	27123789	27130812	+	1435	1	ABHD1|LA	0.503	0.687
NM_0009	chr7	24284188	24291865	+	554	1	NPY|PYY4	23.367	4.401
NM_0012	chr18	45212982	45683095	+	3650	1	SLC14A2|	0.039	0.116
NM_0198	chr7	1.43E+08	1.43E+08	-	2850	2	TRPV5|CA	0	0
NM_0012	chr6	17615035	17706834	-	5788	1	NUP153|	12.263	13.299
NM_1720	chr8	73294602	73325285	+	3955	1	RDH10|SD	1.8	1.465
NR_10680	chr11	209336	209406	+	70	1	MIR6743|	3.868	1.761
NM_0216	chr16	75473124	75495028	-	6880	1	CHST6|MC	0.388	0.44
NR_03749	chr6	33698128	33698234	+	106	1	MIR3934|	0.73	2.972
NM_0239	chr1	2.07E+08	2.07E+08	-	5496	1	C1orf116|	0.035	0.052
NM_0013	chr5	1.12E+08	1.12E+08	-	1461	1	STARD4|-|	3.027	2.897
NR_02763	chr10	47689700	47731218	+	3287	1	FAM35DP	0.012	0.008
NR_13010	chr7	1.49E+08	1.49E+08	+	1897	1	GHET1|lnc	1.74	1.87
NM_1985	chr7	1.39E+08	1.39E+08	-	1537	1	KLRG2|CL	0	0.053
NR_03330	chr2	2.38E+08	2.38E+08	-	1842	1	RAB17|-|-	0.014	0.112
NM_1528	chr11	65572349	65574198	+	1405	1	FAM89B|L	117.106	102.099
NM_0018	chr5	83638198	83721077	-	4671	1	HAPLN1|C	0.033	1.495
NR_03172	chrX	1.41E+08	1.41E+08	-	47	1	MIR320D2	0	0
NM_0005	chr18	13882044	13915536	-	3648	1	MC2R|AC	0	0
NM_0010	chr12	27081057	27082522	-	962	1	C12orf71|	0	0.157
NM_0034	chr11	19182030	19210571	-	1455	1	CSRP3|CL	0	0.047
NR_03749	chr18	35934088	35934142	-	54	1	MIR3929|	0	0
NR_02963	chr6	33207835	33207944	+	109	6	MIR219A1	0	0
NR_04537	chr2	1.66E+08	1.66E+08	-	1592	1	LOC10050	0.518	0.594
NR_03620	chr17	8088056	8088147	+	91	1	MIR4314|	0	0
NM_0052	chr11	1.28E+08	1.29E+08	-	5232	1	ETS1|ETS-	0.599	0.05
NR_10460	chr5	1.18E+08	1.18E+08	+	1217	1	LINC0214	0	0.045
NM_0024	chr16	58025566	58046900	+	4237	1	MMP15|N	30.044	37.082
NM_0013	chr1	1478115	1496204	+	2565	1	ATAD3B|A	6.213	6.611
NM_1815	chr16	66604664	66613893	+	1940	1	CMTM3|B	14.668	11.883
NM_0325	chr1	39659121	39672038	-	1100	1	NT5C1A|C	0.07	0.174
NM_0011	chr19	15640897	15660760	+	2957	1	CYP4F3|C	0	0.074
NM_0013	chr1	1.11E+08	1.11E+08	+	2253	1	PIFO|C1or	2.747	0.772
NM_0228	chr2	1.6E+08	1.6E+08	+	3508	1	MARCH7|	10.758	18.835
NM_1383	chr2	1.35E+08	1.35E+08	+	1238	1	ACMSD|-|	1.156	1.129
NR_03986	chr15	50360329	50360410	+	81	1	MIR4712|	2.865	3.72
NM_0209	chr2	1.68E+08	1.68E+08	+	2164	1	B3GALT1|	3.313	2.994
NR_00371	chr5	86282444	86297547	+	2672	1	NBPF22P|	0	0
NM_0027	chr19	840985	848175	+	994	2	PRTN3|AC	0	0
NM_0012	chr1	1.1E+08	1.1E+08	+	3104	1	STRIP1|FA	43.723	54.047
NR_02967	chr20	62564912	62565013	+	101	1	MIR133A2	0	0
NR_03885	chr12	1.2E+08	1.2E+08	-	1895	1	NRAV|DYN	7.464	4.474
NM_0055	chr11	71548420	71549607	+	1185	1	KRTAP5-9|	0.294	0.162
NM_0147	chr17	7336529	7351474	+	2484	1	ACAP1|CE	1.754	1.257
NM_0013	chr1	32251239	32286167	+	1952	1	LCK|IMD2	0.007	0.014
NM_1526	chr9	3824128	4152183	-	6654	1	GLIS3|ND	0.961	2.33
NM_0175	chr19	2037471	2051244	-	1742	1	MKNK2|G	29.525	18.352

NR_00326	chr13	49890409	49893380	+	2971	1	CTAGE10P	0.004	0
NR_03165	chr3	1.87E+08	1.87E+08	+	105	1	MIR1248|I	0.737	2.87
NM_0011	chr9	1.37E+08	1.37E+08	-	2626	1	TPRN|C9o	7.689	6.88
NM_1524	chr1	64203618	64246274	+	3244	1	UBE2U|-|-	0	0
NM_0012	chr1	1.68E+08	1.68E+08	-	7382	1	GPR161|R	22.58	21.39
NR_03059	chr9	28863626	28863706	-	80	1	MIR876|M	0	0
NM_0011	chrX	49589515	49596827	+	535	1	GAGE2A|C	0	0
NM_0011	chr6	46704316	46735693	-	1734	1	PLA2G7|LI	0.074	0.055
NM_0056	chr16	20763990	20797157	+	2117	1	ACSM3|SA	0.177	0.181
NM_0010	chr12	56041902	56044223	+	676	1	RPS26|DB	3.071	2.897
NM_0012	chr14	20999255	21001875	+	1407	1	SLC39A2|6	0.202	0.185
NM_0010	chr8	81458383	81461523	-	394	1	FABP9|PEI	0	0
NM_1527	chr11	61258286	61295316	-	3514	1	VWCE|UR	1.137	1.017
NM_0327	chr19	2428165	2456968	-	4630	1	LMNB2|EF	42.181	34.562
NM_0011	chr3	1.84E+08	1.84E+08	+	2423	1	FAM131A|	32.822	33.816
NM_0010	chr3	1.97E+08	1.97E+08	-	1807	1	SMCO1|C3	0.029	0
NM_0013	chr10	97427677	97433444	+	1792	1	PGAM1|H	15.836	10.578
NR_10442	chr12	57243455	57251193	-	1146	1	STAC3|NA	3.668	3.167
NM_0013	chr2	73729837	73737406	-	751	1	TPRKB|CG	17.529	14.335
NM_0190	chr11	31509729	31784525	+	2320	1	ELP4|AN|	7.864	6.193
NM_0152	chr8	29067278	29263093	-	8724	1	KIF13B|GA	2.224	2.603
NR_02741	chr15	20562349	20575703	+	5264.3	7	GOLGA8CF	0	0
NR_03417	chr15	44534505	44536900	-	1383	1	EIF3J-AS1|	6.023	6.477
NM_1980	chr1	2.48E+08	2.48E+08	-	2738	1	OR2C3|OF	0	0
NM_0012	chr9	1.36E+08	1.36E+08	+	613	1	OBP2A|LC	0	0
NM_0036	chr2	1.77E+08	1.78E+08	+	7643	1	AGPS|ADA	3.784	3.496
NM_0010	chr11	832952	838835	+	1473	1	CD151|GP	48.51	41.203
NM_0010	chr19	52269571	52292723	+	2969	1	ZNF766|-|	4.864	6.634
NM_1388	chr2	38665910	38734767	+	2474	1	GALM|BLC	2.7	2.004
NR_03037	chr20	34466325	34466418	+	93	1	MIR644A|	0	0
NR_03663	chr12	1.12E+08	1.12E+08	+	2839	1	ADAM1A|	3.515	3.845
NM_0005	chr11	5232829	5234628	-	769	1	HBD|-|-|1	0	0
NM_0012	chr17	75266230	75270245	-	1222	1	MIF4GD|A	19.298	19.391
NM_0010	chr4	1.29E+08	1.29E+08	+	1581	1	C4orf33|-|	3.931	3.257
NM_0017	chr16	64943753	65122137	-	6781	1	CDH11|CA	6.039	3.947
NM_0011	chr19	58083842	58118426	-	2565	1	ZSCAN18|	52.609	62.259
NM_0011	chr6	43427554	43450425	+	5004	1	ABCC10|ES	9.018	8.472
NM_0012	chr17	19089461	19097563	+	1909.7	3	LOC38843	0	0
NR_10691	chr9	1.3E+08	1.3E+08	+	66	1	MIR6855|I	2.93	1.453
NM_0014	chr2	1.19E+08	1.19E+08	-	2958	1	EN1|-|-|2	0.793	1.968
NM_0071	chr3	69019827	69052333	-	6860	1	TMF1|ARA	2.242	2.638
NR_03974	chr17	8186945	8187004	+	59	1	MIR4521|	0.874	0
NM_0012	chr20	34516384	34540958	+	690	1	DYNLRB1|	314.19	323.111
NR_04567	chr17	42572310	42577716	-	1329	1	PSMC3IP|	19.83	14.872
NR_03061	chr14	1.01E+08	1.01E+08	+	77	1	MIR543|M	0	0
NM_0013	chr1	43530860	43623672	+	6804	1	PTPRF|BN	58.447	52.986
NM_0229	chr5	69189548	69210357	+	1378	1	CENPH|-|-	4.313	4.652
NM_0012	chr19	42086110	42132473	-	6744	1	POU2F2|C	12.148	3.085
NR_03750	chr10	1.22E+08	1.22E+08	+	102	1	MIR3941|	0	0
NM_0010	chr5	1.72E+08	1.72E+08	-	7762	1	SH3PXD2B	31.732	43.226

NM_0009!	chr19	49581330	49591008	+	1384	1	PRRG2|PR	0.196	0.257
NM_1984(	chrX	1.06E+08	1.06E+08	+	8031	1	NRK|NESK	0	0.003
NM_0005(	chr1	1.11E+08	1.11E+08	+	1557	1	CD53|MO:	0	0.018
NM_0211:	chr6_GL0C	3053218	3062802	-	873	6	LSM2|C6o	0	0
NM_0010(	chr1	1.82E+08	1.82E+08	-	3645	1	ZNF648|-|	0.064	0.075
NR_00306	chr19	50802325	50802421	-	96	1	SNORD88(	0.134	0.143
NM_0016:	chr22	19969879	20016786	-	4014	1	ARVCF|-|-	27.888	22.494
NM_0028(	chr17	39980768	39997960	+	2173	1	PSMD3|P5	98.763	108.385
NR_12605	chr9	42567859	42569349	-	520	2	LOC10390:	0.149	0
NM_0011!	chr11	75240905	75243704	+	2797	1	TPBGL|-|-	0.249	0.441
NM_0012!	chr22	30459229	30472047	-	2286	1	SEC14L3|T	0	0.006
NR_04676	chr7	36597834	36600120	-	570	1	AOAH-IT1|	0	0
NM_0013(	chr4	1.88E+08	1.88E+08	+	2479	1	ZFP42|REX	0	0.017
NR_01534	chr17	30576465	30637466	+	4588	1	LOC10713:	2.498	3.162
NM_0010{	chr6	1.07E+08	1.07E+08	-	6653	1	BEND3|KI/	3.781	3.43
NM_0178!	chr20	62938089	62948475	+	4420	1	GID8|C20c	33.651	40.23
NM_0011:	chr21	45849961	45942454	+	1896	1	PCBP3|ALI	16.79	6.292
NR_02740	chrX	1.55E+08	1.55E+08	+	709	2	FAM223B|	0	0
NM_0010/	chr9	35752991	35754277	-	672	1	MSMP|PSI	23.389	19.2
NR_12056	chr11	1.14E+08	1.14E+08	-	3828	1	LOC10192:	0	0
NM_0003(	chr11	2163929	2171805	-	1802	1	TH|DYT14	16.664	5.838
NM_0010{	chr8	1.45E+08	1.45E+08	-	1301	1	COMMD5|	14.806	14.781
NM_0150{	chr5	1.33E+08	1.34E+08	-	5364	1	FSTL4|-|-|	3.435	1.573
NM_0209:	chr19	9140380	9163415	+	4066	1	ZNF317|-|	17.323	24.817
NR_03038	chr8	1.23E+08	1.23E+08	-	96	1	MIR548D1	0	0
NR_04978	chr19	58367623	58381022	-	2030	1	ZNF837|-|	1.778	1.451
NM_0011:	chrX	37571569	37677497	+	3242	1	LANCL3|-|	0.099	0.097
NR_00335	chr15	25239838	25239919	+	81	1	SNORD11!	0	0
NR_10684	chr17	44934618	44934681	-	63	1	MIR6783|I	0.409	0
NR_11050	chrX	14244265	14245423	+	1158	1	UBE2E4P|	19.106	19.635
NM_0321/	chr6	1.46E+08	1.46E+08	-	4402	1	FBXO30|FI	2.124	3.46
NM_0176:	chr4	1.68E+08	1.68E+08	-	6033	1	DDX60|-|-	0.109	0.193
NM_0002/	chrX	43654907	43746824	+	5313	1	MAOA|BR	2.805	2.619
NM_0012{	chr2	29097676	29174715	+	2336	1	CLIP4|RSN	1.314	2.029
NM_0012(	chr11	96389994	96393563	+	3157	1	JRKL|HHM	2.45	2.473
NM_0154:	chr20	43514436	43541895	+	3174	1	L3MBTL1|	2.738	2.909
NM_0066/	chr1_KI27(	268113	512199	+	2597	2	SDCCAG8|	0	0
NM_0058:	chr8	98102339	98117190	-	1003	1	RIDA|HRSI	7.623	5.189
NM_0012!	chr20	51386957	51562831	-	6833	1	NFATC2|N	0.075	0.03
NM_0010(	chrX	72272603	72277291	-	947	1	RPS4X|CC(	583.256	625.246
NR_02445	chr1	3900404	3915447	+	1948	1	LINC01134	0.033	0.014
NM_0332(	chr22	50502947	50507706	-	2583	1	LMF2|TMI	21.23	15.701
NM_0010{	chr19	4890437	4902867	-	1634	1	ARRDC5|-	0	0.008
NM_0025(	chr19	10111521	10115388	+	1972	1	P2RY11|P2	11.651	11.44
NM_0011:	chr7	1.42E+08	1.42E+08	-	1187	1	PRSS37|TF	0.043	0.092
NM_0013:	chr1	20163092	20177364	-	615	1	PLA2G2C|	0	0
NM_0029(	chr2	79120458	79123419	+	800	1	REG1A|ICF	0	0
NR_02947	chr22	46113686	46113768	+	82	1	MIRLET7B	0	0.167
NM_0050(	chr3	1.51E+08	1.51E+08	-	2616	1	SIAH2|hSia	14.234	17.922
NR_03039	chr22	37847678	37847774	-	96	1	MIR659|M	1.612	0.143

NM_0010(	chr17	7351889	7354943	+	3052	1	KCTD11|C	8.183	6.786
NM_0162:	chr17	18108706	18179802	+	11796	1	MYO15A|I	0.334	0.368
NM_0162(	chr8	1.44E+08	1.44E+08	-	967	1	VPS28|-|-	168.335	151.56
NM_0011:	chr3	38138478	38143021	+	2528	1	MYD88|M	1.056	1.143
NR_03078	chrX	74287286	74287357	-	71	1	MIR374A|	0	0
NR_12600	chr17	60564546	60586620	+	640	1	LINC0199	0	0
NM_0010:	chr20	56524729	56526152	+	674	1	FAM209A|	0.115	0.061
NM_0013:	chr3	49857981	49870222	-	3215	1	CAMKV|1(	19.868	86.459
NM_1994:	chr2	32063611	32157637	+	5099	1	SPAST|ADI	9.576	9.944
NM_0150(	chr19	47428022	47472050	-	5099	1	SLC8A2|N(	31.031	33.852
NM_0012{	chr6	10747759	10756981	+	891	1	TMEM14B	22.748	15.111
NM_0165{	chr3	1.19E+08	1.2E+08	+	1584	1	TIMMDC1	40.512	40.192
NR_03828	chr10	3141601	3148629	+	4072	1	PITRM1-AS	4.088	4.413
NM_0001!	chr1	1.19E+08	1.19E+08	+	1704	1	HSD3B2|H	0	0
NM_0188:	chr20	47657406	47786064	-	3876	1	SULF2|HSU	34.389	43.819
NR_02748	chr17	64778139	64781504	-	2871	1	ARHGAP27	2.578	1.484
NM_0038{	chr6	1.12E+08	1.12E+08	+	1231	1	WISP3|CCI	0	0.011
NM_0012!	chr11	6395124	6404691	-	1892	1	APBB1|FE(	157.598	165.305
NM_0806!	chr15	35370969	35546203	-	2036	1	DPH6|ATP	3.128	1.594
NM_0011‹	chr1	12824760	12831410	-	1684	1	PRAMEF11	0	0
NM_0010:	chr17	18722089	18736118	+	1975	1	TRIM16L|T	4.113	5.201
NM_0013:	chr11_KI2	134495	157362	+	1234	2	SLC22A18|	0.063	0.277
NR_04011	chr12	9228533	9234207	-	1192	1	A2MP1|A2	0	0.034
NR_03342	chr3	1.28E+08	1.28E+08	+	1929	1	ABTB1|BP	2.72	4.473
NR_10803	chr5	1.77E+08	1.77E+08	+	608	1	LINC01574	0.085	0.113
NM_0148:	chr22	49853849	49890078	+	6882	1	ZBED4|-|-	17.399	18.404
NM_0013(	chr18	76822607	76970726	+	7899	1	ZNF236|ZI	2.728	2.542
NR_10681	chr12	57748618	57748682	-	64	1	MIR6759|I	16.923	18.405
NM_0140:	chr10	1056836	1132297	+	4595	1	WDR37|-|	16.726	18.946
NM_1383:	chr14	21057822	21058455	+	631	1	RNASE8|R	0	0.022
NR_00275	chr15	65296051	65296167	+	116	1	RNU5A-1|	0	0.236
NM_0035!	chr1	1.17E+08	1.17E+08	+	4829	1	TTF2|HuF2	1.394	1.376
NM_1307:	chrX	52812204	52818298	+	470	1	XAGE5|CT	0	0
NM_0184!	chr6	1.57E+08	1.57E+08	-	5059	1	TMEM242	7.011	6.376
NM_1816:	chr21	30746951	30747202	-	250	1	KRTAP21-2	0	0
NR_03030	chr4	1.15E+08	1.15E+08	+	95	1	MIR577|M	0	0
NR_10353	chr1	77881348	77889539	-	2289	1	NEXN-AS1	0.011	0.072
NM_0121!	chr11	6481447	6484681	+	2837	1	TIMM10B|	12.698	13.605
NR_02455	chr10	60869440	61001440	-	4325	1	RHOBTB1|	7.715	8.525
NM_0020:	chr1	27666061	27672213	-	829	1	IFI6|6-16|	21.774	37.522
NR_10427	chr4	1.52E+08	1.52E+08	+	368	1	FBXW7-AS	1.962	1.861
NM_0013:	chr20	38127387	38165429	-	4870	1	TGM2|TG(	0.154	0.096
NM_0010(	chr22	41673933	41688909	-	1495	1	SNU13|15	135.653	135.101
NR_14494	chr12	95516560	95548844	-	3568	1	USP44|-|-	0.34	0.415
NM_0328(	chr9	1.29E+08	1.29E+08	+	3619	1	MIGA2|C9	7.015	7.467
NM_0013:	chr10	1.25E+08	1.25E+08	-	3378	1	CTBP2|-|-	29.424	31.692
NR_03648	chr13	1.02E+08	1.02E+08	+	1127	1	FGF14-AS2	3.787	2.479
NM_0011:	chr2	1.51E+08	1.51E+08	+	9643	1	RIF1|-|-|2	1.149	2.531
NR_01542	chr10	4656156	4662414	+	1008	1	LINC00705	0	0
NM_0011:	chr1	1.09E+08	1.09E+08	-	724	1	C1orf194|	5.414	3.897

NM_0052:	chr18	52340172	53535903	+	10179	1	DCC|CRC1	55.008	42.612
NM_0122:	chr7	22118290	22356914	-	6594	1	RAPGEF5|	7.612	4.63
NM_0011(	chr1	20719732	20732640	-	3694	1	SH2D5|-|-	4.171	5.365
NR_13633	chr16	89296128	89298317	+	1573	1	LOC10537	1.631	1.724
NM_0012:	chr11	8224304	8268635	-	961	1	LMO1|RBT	23.828	53.277
NR_11011	chr2	1.04E+08	1.05E+08	-	1453	1	LINC01103	0.035	0.028
NM_0333:	chr9	1.31E+08	1.31E+08	-	3932	1	FAM78A|(	1.21	1.602
NM_0013(	chr3	42397078	42410602	-	880	1	LYZL4|LYC	0	0
NM_0013:	chr6	1.51E+08	1.51E+08	+	7160	1	PLEKHG1|	5.993	6.898
NR_04003	chr2	2.01E+08	2.01E+08	-	1034	1	CFLAR-AS1	0.623	0.358
NM_0151:	chr17	32492522	32877173	-	5535	1	MYO1D|PI	1.85	1.633
NR_11025	chr2	38458638	38515740	-	1127	1	LOC10192:	0	0
NR_11091	chr16	51017758	51035777	+	434	1	LINC02127	0	0
NR_12594	chr1	81513880	81557702	-	639	1	LOC10192	0	0
NR_02962	chr15	31065032	31065141	-	109	2	MIR211|M	0	0
NM_0208:	chr10	24583609	24723668	-	7139	1	ARHGAP21	22.977	21.78
NR_00334	chr15	25225203	25225284	+	81	1	SNORD115	0	0
NR_12599	chr1	8208672	8215208	+	491	1	LINC01714	0	0.028
NM_0048:	chr4	55427901	55546909	-	10447	1	CLOCK|KA	1.117	1.683
NM_0022(	chr3	1.22E+08	1.23E+08	-	6873	1	KPNA1|IP(	13.096	15.606
NR_03617	chrX	1.54E+08	1.54E+08	+	80	1	MIR3202-1	0	0
NM_0012:	chr2	2.08E+08	2.08E+08	-	2291	1	IDH1|HEL-	122.677	109.074
NM_0010(	chr3	98282888	98283832	+	943	1	OR5H2|OF	0	0
NM_0010:	chr2	2.07E+08	2.07E+08	-	2037	1	DYTN|-|-|	0.013	0
NM_0251:	chr17	63009537	63427706	+	11720	1	TANC2|RC	7.676	13.261
NR_03784	chr1	1.73E+08	1.73E+08	-	5616	1	LOC10050	0.172	0.256
NM_0002:	chr7	44062727	44065587	-	852	1	PGAM2|G:	7.43	4.292
NM_0050:	chr18	63907990	63936111	+	2113	1	SERPINB1(	0	0
NR_12163	chr4	1.2E+08	1.2E+08	+	640	1	LOC10099	0.342	2.055
NR_03033	chr8	1.04E+08	1.04E+08	-	96	1	MIR548A3	0	0
NM_0012:	chr16	84406588	84464187	+	2943	1	ATP2C2|SI	0.39	0.349
NM_0012:	chr16_KI2	631357	694938	+	4447	2	PDXDC1|L	0.418	0.502
NM_0066:	chr1	1.2E+08	1.2E+08	+	2001	1	PHGDH|3-	62.283	75.672
NM_0023:	chr4	1.2E+08	1.2E+08	-	1429	1	MAD2L1|H	4.611	4.409
NM_0571:	chr11	45909669	45918123	-	1760	1	PEX16|PBI	16.908	15.074
NM_0013:	chr16	20409534	20419775	+	1104	1	ACSM5|-|	0.058	0
NM_0010(	chr1	1.57E+08	1.57E+08	-	1970	1	ETV3L|-|-|	0.34	0.111
NM_0324:	chr19	37371157	37393064	+	5119	1	ZNF527|-|	1.718	2.087
NM_0180(	chr3	62369672	62373515	-	2079	1	FEZF2|FEZ	0.316	33.172
NM_0013(	chr11	392599	404908	+	2791	1	PKP3|-|-|	0.037	0.015
NR_12634	chr11	777578	784284	+	623	1	LOC17139	5.588	5.562
NM_0228:	chr3	45082274	45146422	-	5999	1	CDCP1|CD	0.045	0.066
NM_0010:	chr12	47782711	47819980	-	4163	1	HDAC7|HD	12.364	12.351
NM_0160(	chr3	43690883	43722725	+	5344	1	ABHD5|CD	2.492	2.132
NM_1524:	chr11	1.01E+08	1.01E+08	+	4726	1	ARHGAP42	0.243	2.113
NM_0065(	chr10	1.01E+08	1.01E+08	-	1281	1	LBX1|HPX-	0.04	9.42
NR_10986	chr20	10875333	10909272	-	1999	1	LOC10192:	0	0
NM_0212:	chr15	80695311	80755621	+	2355	1	ABHD17C|	6.833	11.696
NR_10996	chr21	24304550	24321377	-	2003	1	LINC01689	0	0
NM_1471:	chr3	46668995	46693713	-	4919	1	ALS2CL|RN	0.113	0.067

NM_0011{	chr5	1.27E+08	1.27E+08	-	4916	1	ALDH7A1|	18.472	18.186
NM_0010{	chrX	1.21E+08	1.21E+08	-	1288.7	10	CT47A8|C	0	0
NM_0018(	chr14	23117306	23119611	-	1543	1	CEBPE|C/E	0	0
NM_0180!	chrX	51743385	51746230	+	2843	1	GSPT2|ERI	15.565	17.903
NM_0024	chr5	1.75E+08	1.75E+08	+	2204	1	MSX2|CRS	8.769	0.653
NM_0240(	chr19	18557762	18569387	+	1454	1	KXD1|BOR	46.475	45.274
NM_0010!	chr2	2.32E+08	2.32E+08	+	1200	1	PTMA|TM	205.264	184.43
NM_0251!	chr4	7058895	7068210	-	2793	1	GRPEL1|G	12.367	12.422
NR_03885	chr16	85282958	85288079	+	1781	1	LINC00311	0	0.023
NM_0039:	chr19	2100988	2151557	-	4838	1	AP3D1|AD	58.189	57.911
NM_0011:	chr2	2.1E+08	2.1E+08	-	4501	1	LANCL1|G	18.01	19.582
NM_0013:	chr2	24819170	24920017	-	4708	1	ADCY3|AC	8.758	10.017
NM_0011	chr4	39044831	39126233	+	7167	1	KLHL5|-|-|	4.886	9.049
NR_12579	chr8	1.06E+08	1.06E+08	-	1003	1	ZFPM2-AS	0.051	0.123
NM_0032(	chr1	2.23E+08	2.23E+08	-	4259	1	TLR5|MEL	0.13	0.196
NR_03333	chr16	71698567	71698701	-	134	1	SNORA70D	0	0
NR_12037	chr15	90660234	90664954	+	1279	1	LINC01585	0	0.011
NR_03077	chr1	2.02E+08	2.02E+08	+	1370	1	SHISA4|C1	8.847	10.708
NM_0049!	chr14	22836533	22847599	+	3582	1	MMP14|M	1.134	3.407
NM_0132	chr16	58113593	58129392	-	1283	1	CFAP20|C	82.808	89.612
NR_04008	chr21	36069642	36126640	-	1217	1	LOC10013	3.825	3.478
NM_0157(	chr2	53786931	53818819	+	2566	1	ERLEC1|C	14.18	16.024
NM_0028(	chr17	63827410	63832027	+	1346	1	PSMC5|S8	116.712	100.692
NM_0010(	chr11	59712916	59713845	-	928	1	OR10V1|C	0	0
NM_1780(	chr13	42048708	42242897	+	17406	1	DGKH|DGI	0.249	0.22
NM_0249	chr3	1.7E+08	1.7E+08	-	12655	1	PHC3|EDR	1.098	2.024
NM_1722:	chr19	19276513	19320512	-	2073	1	SUGP1|F2	27.236	30.381
NM_0011!	chr11	94512529	94532123	+	598	1	C11orf97|	0.086	0.069
NR_12065	chr10	77140831	77150100	+	442	1	KCNMA1-A	0	0
NM_0039:	chr2	2.19E+08	2.19E+08	+	2525	1	CDK5R2|N	21.973	19.897
NM_0012!	chr11	60088664	60098467	+	3505	1	MS4A2|AF	0	0
NM_1526:	chr20	6040546	6054080	-	2948	1	LRRN4|C2	0.013	0.046
NR_13468	chr4	32005853	32022422	-	399	1	LOC10537	0	0
NM_0010:	chr14	20590081	20590823	-	740	1	RNASE12|	0	0
NM_0057	chr15	72474326	72586555	+	5235	1	ARIH1|ARI	15.721	15.905
NM_0012{	chr1	9239844	9271335	+	9020	1	H6PD|COF	4.204	5.098
NR_00357	chr10	1.23E+08	1.23E+08	+	3715	1	DMBT1P1|	0	0.026
NR_02710	chr3	10759484	10764192	-	2866	1	LINC00606	0.076	0.029
NM_1526(	chr20	25612936	25624012	-	3796	1	NANP|C20	0.635	1.234
NR_04671	chr7	39404599	39406346	-	376	1	POU6F2-A	0	0
NM_0010(	chr1	52423276	52553090	-	5818	1	ZCCHC11|	7.546	8.073
NM_2125:	chr16	23835979	24220611	+	2692	1	PRKCB|PK	3.061	1.857
NM_0528:	chr9	1.36E+08	1.36E+08	-	2108	1	CARD9|CA	0.373	0.273
NR_12575	chr5	80608621	80622524	-	440	1	LINC01337	0	0.187
NM_0011	chr15	73052484	73305206	+	6899	1	NEO1|IGD	38.152	45.131
NR_00298	chr20	38449369	38449504	+	135	1	SNORA60|	0.764	2.841
NM_0013:	chr15	43693940	43699222	+	1720	1	CKMT1A|(	1.529	2.325
NM_0011!	chr10	70112267	70132934	-	3239	1	AIFM2|AM	1.222	1.472
NR_10685	chr19	49832018	49832099	+	81	1	MIR6800|	5.89	7.102
NM_0022!	chr19	50819146	50823787	-	869	1	KLK1|KLKF	0	0.032

NR_02958	chr17	48632490	48632559	-	69	1	MIR196A1	0	0
NM_0012	chr12	27332854	27425813	+	7351	1	ARNTL2|B	0.623	0.635
NM_1525	chr5	95391344	95450440	+	1551	1	FAM81B|-	0.058	0.115
NM_0012	chr14	64914361	64935366	+	3604	1	CHURC1|C	12.829	11.535
NR_10991	chr5	1173096	1178605	-	1797	1	CTD-3080	0	0
NM_0061	chr10	1.14E+08	1.14E+08	-	5384	1	NRAP|N-R	0.103	0.081
NM_0028	chr7	1.37E+08	1.37E+08	-	1727	1	PTN|HARP	205.28	157.179
NR_03960	chr8	93916022	93916119	-	97	1	MIR378D2	0	0.141
NR_02391	chr1	69919322	69920317	+	995	1	PIN1P1|PI	0.35	0.413
NM_0010	chr8	12089338	12115516	+	3674	1	ZNF705D|	0	0
NR_03319	chr8	81702914	81721304	-	668	1	ZFAND1|-|	6.813	7.833
NM_0123	chr13	44939249	44989478	-	3475	1	NUFIP1|N	1.425	1.565
NM_0011	chr1	28329002	28335967	+	1808	1	MED18|p2	11.631	12.242
NM_0011	chr16	71031411	71230722	-	3101	1	HYDIN|CIL	0.383	0.283
NR_11091	chr16	65141756	65176573	-	746	1	LINC0212	0	0
NM_0054	chr10	46005088	46023458	-	3480	1	NCOA4|A	55.661	68.796
NM_0056	chr7	1.51E+08	1.52E+08	-	2065	1	RHEB|RHE	17.539	18.917
NR_13551	chr21	9543030	9559268	+	550	1	LOC10537	0	0
NR_02414	chr3	10280952	10293449	+	1587	1	GHRLOS|G	0.804	0.785
NM_0149	chr2	2.07E+08	2.07E+08	+	6821	1	FASTKD2|	3.109	3.494
NM_0012	chr10	67921899	68075346	-	4257	1	HERC4|-|-	3.601	4.858
NR_13803	chr6_GL0	2246539	2271964	+	1176.8	6	HCG20|N	0	0
NM_0013	chr7	73768997	73770270	-	1271	1	CLDN3|C7	1.867	0.011
NR_00395	chr14	70245753	70247801	-	2048	1	ADAM21P	0.013	0.074
NR_02337	chr1	2.29E+08	2.29E+08	-	120	10	RNA5S10|	0	0
NM_0013	chr18	46836246	46920167	-	1387	1	PIAS2|ARI	4.053	5.382
NM_0057	chr14	54426929	54441430	-	1416	1	CNIH1|CN	24.175	26.282
NM_1451	chr10	73253758	73358859	-	3674	1	CFAP70|T	0.74	0.623
NM_1827	chr20	8132265	8884900	+	7172	1	PLCB1|EIE	1.213	4.496
NR_12574	chr5	1.29E+08	1.29E+08	-	784	1	ADAMTS1	0.164	0.611
NR_03969	chr10	12130760	12130816	-	56	1	MIR548AK	0	0
NM_0013	chr5	1.47E+08	1.47E+08	-	5034	1	DPYSL3|C	752.673	962.402
NR_11053	chr5	1.5E+08	1.5E+08	-	1613	1	LOC64476	3.165	4.755
NR_03393	chr14	1.03E+08	1.03E+08	-	1555	1	LINC00605	0	0
NM_1526	chr6	61679960	62286227	-	2321	1	KHDRBS2|	5.811	8.728
NR_02771	chr11	7849051	7849571	+	520	2	OR5E1P|H	0	0
NM_0011	chr2	97113496	97264520	+	6190	1	ANKRD36|	0.271	0.449
NR_00330	chr15	25201323	25201404	+	81	3	SNORD11	0	0
NM_0011	chr10	43371644	43376335	+	624	1	FXYD4|CH	0	0
NM_0011	chr1	27725979	27762912	+	3108	1	FAM76A|-	3.157	2.763
NM_0022	chr17	41513745	41519018	-	1830	1	KRT15|CK	0	0
NR_04684	chr4	1.06E+08	1.06E+08	+	450	1	ARHGEF38	0	0
NM_0203	chr1	1.53E+08	1.53E+08	-	2117	1	PGLYRP4|	0	0
NM_0013	chr2	32165841	32224379	+	4884	1	SLC30A6|	4.005	3.441
NM_0158	chr19	54754986	54767880	+	1556.2	59	KIR2DL3|C	0	0
NM_0010	chr11	1.24E+08	1.24E+08	-	934	1	OR10G7|C	0	0
NM_0011	chr5	80138727	80256082	-	6468	1	SERINC5|C	4.591	11.848
NR_13011	chr6	1.66E+08	1.66E+08	-	1108	1	SFT2D1|C	7.762	10.965
NM_0011	chr8	61675260	61714640	-	1689	1	ASPH|AAH	5.641	3.803
NM_0043	chr19	531733	542087	+	1438	1	CDC34|E2	103.74	101.461

NM_0010⁄	chr6	33794672	33804016	-	562	1	MLN|-|-|6	0.298	0.122
NM_0010:	chr7	7358613	7535829	-	3478	1	COL28A1|(	0.26	0.48
NM_0054!	chr19	10572671	10587315	-	1737	1	AP1M2|AI	0	0.047
NR_12041	chr13	1.11E+08	1.11E+08	+	3440	1	LINC0036ε	0.12	0.179
NM_0148;	chr22	38685543	38700454	-	3429	1	JOSD1|dJ5	30.957	40.288
NM_0023!	chr1	58575423	58577494	-	2069	1	TACSTD2|I	0	0
NM_0012;	chr1	1.56E+08	1.56E+08	-	5670	1	MEF2D|-|-	12.368	16.642
NM_1387(	chr6	30136733	30148735	+	1910	8	TRIM40|R	0	0
NR_00271	chr14	49586580	49586878	+	298	1	RN7SL1|7I	5.841	4.321
NM_0010:	chr16	18782955	18790334	-	470	1	RPS15A|S:	396.077	372.034
NR_12579	chr12	4248767	4276184	-	551	1	CCND2-AS	5.85	5.419
NM_0043!	chr11	1.09E+08	1.09E+08	+	3244	1	DDX10|HI	4.126	4.176
NM_0247(	chr20	16272104	16573434	-	5234	1	KIF16B|C2	1.084	1.07
NR_10680	chr11	47179611	47179737	-	126	1	MIR6745|I	0	0
NR_02938	chr13	28136843	28139174	-	2189	1	PAN3-AS1	2.291	3.047
NR_00297	chr16	58548499	58548633	-	134	1	SNORA46|	0	0.102
NR_04006	chr1	3940612	3952583	+	565	1	LINC0134ε	0	0.024
NR_03745	chr7	7066964	7067045	-	81	1	MIR3683|-	0	0
NR_00217	chr13	63737435	63742568	+	2643	1	OR7E156P	0.01	0
NM_0010:	chr6	89146050	89165571	+	4705	1	PM20D2|/	2.042	2.294
NM_0011:	chr2	27070474	27071699	-	480	1	OST4|-|-|:	230.954	160.74
NM_0064(	chr15	74541177	74598131	+	4260	1	ARID3B|BI	5.875	3.341
NR_04770	chr13	1.09E+08	1.09E+08	-	544	1	MYO16-AS	0	0
NM_0123;	chr16	68245344	68261058	+	2715	1	PLA2G15|/	10.913	13.142
NR_10270	chr14	46064159	46501901	+	823	1	LINC00871	0.031	0
NR_13462	chr6	33631244	33633745	-	2150	1	LOC10192!	0.72	2.727
NM_0013(	chr2	2.18E+08	2.18E+08	-	1822	1	AAMP|-|-|	55.041	49.338
NM_0010:	chr11	417930	442011	-	2850	1	ANO9|PIG	0.027	0
NM_0122:	chr10	1.02E+08	1.02E+08	-	5184	1	MGEA5|M	28.545	45.826
NM_0212;	chr6_GL0C	3012823	3014322	+	769	7	LY6G5B|C(	0	0
NR_03743	chr6	1.35E+08	1.35E+08	-	94	1	MIR3662|I	0	0
NM_0051;	chr17	3923869	3964464	-	4703	1	ATP2A3|SI	1.952	1.346
NM_1525;	chr9	1.33E+08	1.33E+08	-	2091	1	AK8|AK 8|	1.511	1.343
NR_13461	chr6_GL0C	2149773	2158404	-	793	6	LOC10537.	0	0
NM_0028(	chr12	9148840	9208370	-	4572	1	PZP|CPAN	0.006	0.018
NM_0012(	chr6	34287196	34426125	-	2517	1	RPS10-NUI	42.948	62.526
NR_13116	chr6	2621913	2634603	-	2431	1	LINC0160C	0.016	0.085
NM_0025!	chr7	1.01E+08	1.01E+08	+	1610	1	PCOLCE|P(	10.067	7.937
NM_0010:	chr9	15422784	15461629	+	3084	1	SNAPC3|P	13.663	11.454
NM_1458(	chr11	34150987	34358008	-	4884	1	ABTB2|AB	9.121	6.046
NM_0013(	chr19	19668153	19683506	+	4559	1	ZNF101|H:	1.242	1.316
NM_0010:	chr16	25236001	25257534	-	7117	1	ZKSCAN2|:	9.122	8.385
NM_0044(	chr19	12922470	12933744	-	1838	1	FARSA|CN	53.489	50.16
NM_0010⁄	chr4	1.5E+08	1.5E+08	+	4246	1	DCLK2|CL:	59.281	103.502
NR_03764	chr11	57712523	57819180	+	6230	1	TMX2-CTN	50.361	73.436
NM_0045!	chr17	35976493	35981496	-	1507	2	CCL16|CKI	0	0
NM_0056ε	chr1	2.04E+08	2.04E+08	+	4057	1	SOX13|ICA	4.672	6.405
NM_0010(	chr2	19896631	19901986	-	939	1	TTC32|-|-|	3.707	3.122
NR_13457	chr7	66493691	66495474	+	549	1	GS1-124K5	4.791	4.142
NM_0195!	chr5	9628997	9630351	-	1352	1	TAS2R1|T:	0.019	0.213

NM_0012(	chr7	1.39E+08	1.39E+08	+	2458	1	LUC7L2|C(	34.5	38.06
NR_03413	chr11	10858217	10879276	+	1110	1	ZBED5-AS1	6.818	5.516
NM_0013(	chr1	1.56E+08	1.56E+08	-	3541	1	UBQLN4|A	24.469	27.553
NM_0327	chr2	1.28E+08	1.28E+08	+	2808	1	SFT2D3|-|	5.317	5.863
NM_0010(	chr11	1.24E+08	1.24E+08	+	943	1	OR8D4|OF	0	0
NM_0025	chr2	1.01E+08	1.01E+08	+	3981	1	NPAS2|M(	0.155	0.616
NR_10696	chr1	1.48E+08	1.48E+08	+	81	1	MIR6077|l	0	0
NM_0019	chr1	93345921	93362591	+	3203	1	DR1|NC2|	15.192	14.386
NR_11004	chr12	42615503	42646498	-	1206	1	LOC10192	0	0
NM_1523	chr10	1.02E+08	1.02E+08	+	1413	1	ELOVL3|CI	0.657	1.018
NM_0023	chr11	68312609	68449275	+	5134	1	LRP5|BMN	6.997	11.87
NR_07344	chr9	92210209	92293756	-	4350	1	IARS|GRID	31.837	36.302
NM_0528	chr1	1.53E+08	1.53E+08	-	1164	1	PGLYRP3|l	0	0
NR_11018	chr3	1.5E+08	1.5E+08	-	369	1	LINC01214	0	0
NM_0012	chr1	1.65E+08	1.65E+08	-	1659	1	RXRG|NR2	2.083	1.767
NR_03987	chr17	38719691	38719748	+	57	2	MIR4726|	0	0
NM_0067(	chr8	41929479	42052026	-	9173	1	KAT6A|M(	14.553	15.065
NM_1523	chr13	1.11E+08	1.11E+08	+	725	1	TEX29|C13	0.285	0.359
NR_03830	chr3	1.83E+08	1.83E+08	+	1485	1	LINC00888	18.442	14.61
NM_0013	chr16	84159040	84177920	+	1726	1	DNAAF1|C	0.336	0.175
NM_0069	chr9	99821855	99866891	+	5624	1	NR4A3|CH	1.515	4.669
NM_0229(	chr19	15451627	15464571	-	3270	1	RASAL3|-|	0.051	0.197
NM_1781	chr1	47067488	47118320	+	1893	1	CYP4Z1|CY	0	0
NR_03740	chr17	56891270	56891355	-	85	1	MIR3614|	0.455	1.289
NM_0013	chr3	1.27E+08	1.27E+08	+	999	1	CHCHD6|C	37.725	47.371
NM_0011	chr12	56128202	56144676	+	4244	1	ESYT1|FAN	18.669	9.631
NR_07509	chr7	726701	786479	+	3339	1	DNAAF5|C	5.564	4.098
NM_0011	chr1	2.11E+08	2.11E+08	+	1851	1	RCOR3|-|-	9.815	11.292
NR_02667	chr16	86555320	86557299	+	1979	1	FLJ30679|	0.091	0.166
NR_11080	chr17	58076891	58083204	-	6110	1	LOC10192	0.034	0.025
NM_0010(	chr20	10435303	10628034	+	6052	1	SLX4IP|C2(	2.435	2.655
NM_1533	chr8	9002804	9033339	+	4606	1	ERI1|3'HE)	2.113	2.364
NM_0011	chr6	3258928	3268066	+	816	1	PSMG4|C6	11.377	7.016
NR_00331	chr15	25054209	25054305	+	96.5	2	SNORD116	0.267	0.71
NR_02738	chr16	1790413	1794908	-	2057	1	IGFALS|AC	0.627	0.686
NM_0012	chr19	20923253	20950697	+	2199	2	ZNF85|HP	0	0
NM_0201	chr2	84971108	85059472	+	7544	1	KCMF1|DE	8.178	10.667
NM_0012	chr7	50319048	50405100	+	5779	1	IKZF1|CVII	0.074	0.481
NM_0207	chr6	44298726	44313326	-	4758	1	AARS2|AA	7.327	9.051
NM_0121	chr15	75903859	75930867	+	1709	1	FBXO22|FI	6.79	6.532
NM_0010(	chr16	165974	166768	+	505	1	HBM|HBA	0.026	0.054
NM_0021	chrX	1.56E+08	1.56E+08	+	2142	2	IL9R|CD12	0	0
NM_0011(	chr9	1.11E+08	1.11E+08	+	2452	1	MUSK|CM	0.095	0.028
NR_13502	chr12	42692216	42717119	+	999	1	LOC10536	0	0
NR_03649	chr15	78752037	78753392	+	479	1	LOC64693	0.188	0
NR_14445	chr9	93955834	93957922	+	1443	1	BARX1-AS1	0	0
NM_0218	chr14	50633580	50668353	-	3134	1	SAV1|SAV	3.267	2.705
NM_0010	chr9	76459152	76507416	+	5473	1	GCNT1|C2	0.316	0.916
NR_00359	chr8	85654599	85655676	-	1077.1	10	REXO1L2P	0	0
NM_1385(	chr6	54018916	54266280	+	1800	1	MLIP|C6oı	1.153	1.91

NM_0011:	chr14	70044217	70080217	-	2957	1	SLC8A3|N(	2.961	5.498
NM_0010(	chr22	15528159	15529139	+	979	1	OR11H1|C	0	0
NM_0011:	chr8	1.01E+08	1.01E+08	-	2956	1	YWHAZ|14	138.919	140.181
NM_0011(	chr16	31472208	31477960	+	1760	1	TGFB1I1|A	5.773	2.164
NM_0159:	chr11	68684475	68691175	+	810	1	GAL|ETL8|	1.544	0.118
NM_0012:	chr5	1.24E+08	1.24E+08	+	4156	1	CSNK1G3|	4.235	5.593
NR_03840	chr21	22098617	22116528	+	673.5	2	LINC00308	0	0
NR_03038	chr7	93482760	93482855	-	95	1	MIR653|M	0	0
NM_0002{	chr10	70883508	70888786	-	1004	1	PCBD1|DC	9.426	4.966
NM_0072:	chr8	1.24E+08	1.24E+08	+	3308	1	RNF139|H	8.415	13.486
NM_1719!	chr18_GL3	45390	64085	+	5544	3	SALL3|ZNF	0.016	0.163
NM_0039(	chr10	70815948	70881175	+	5752	1	SGPL1|S1F	17.558	26.763
NR_04499	chr11	3390707	3391915	-	1208	1	OR7E12P|	0.011	0.159
NR_02988	chr14	1.01E+08	1.01E+08	+	92	1	MIR337|M	0	0
NM_0012{	chr7	73328232	73358637	+	1540	1	FKBP6|FKE	0	0
NR_03383	chr5	32947443	32962467	+	2002	1	LINC0212C	0	0.007
NM_0011(	chr12	48122647	48146404	+	3053	1	PFKM|ATF	85.322	90.177
NM_1445{	chr11	64590810	64602353	+	3199	1	SLC22A12|	0	0.013
NR_02990	chr14	1E+08	1E+08	+	97	1	MIR345|M	0	0
NM_0122	chr2	46542728	46584688	+	4535	1	RHOQ|ARI	3.614	3.917
NM_0013:	chr7	1.43E+08	1.43E+08	-	1711	1	LOC10050	6.209	5.748
NM_0011	chr2	2.01E+08	2.01E+08	+	1419	1	NIF3L1|AL	18.391	24.045
NR_12036	chr15	77660051	77668074	+	643	1	LINGO1-AS	0.04	0.043
NM_0250!	chr11	289138	295688	+	3252	1	PGGHG|A	1.709	1.251
NM_0134:	chr8	1.44E+08	1.44E+08	-	4487	1	TONSL|IKE	5.948	2.744
NM_0011:	chr13	57140918	57143939	+	1842	5	PRR20E|-|	0	0
NM_0189:	chr5	1.41E+08	1.41E+08	+	3268	1	PCDHB11|	1.412	0.775
NR_03643	chr15	20408396	20506180	-	4253	1	HERC2P3|	0	0
NM_0022	chr2	26692713	26731198	+	3974	1	KCNK3|K2	8.205	15.865
NM_0013:	chr2	49918505	51032536	-	9211	1	NRXN1|Hs	7.545	13.883
NM_0011:	chrX	27746809	27748821	+	2010	1	DCAF8L2|\	0.18	0.164
NR_02967	chr1	98046070	98046171	-	101	1	MIR137|M	0	0
NM_0010(	chr6	1.49E+08	1.49E+08	+	685	1	SUMO4|ID	0.113	0.1
NM_0017{	chr12	9752486	9760901	-	1669	1	CD69|AIM	0.046	0.008
NR_10684	chr19	2235829	2235926	-	97	1	MIR6789|	8.64	5.507
NM_0010(	chr8	166086	167024	-	937	1	OR4F21|O	0	0
NM_0019{	chr13	46771256	46797232	-	1180	1	ESD|FGH|	49.64	75.519
NM_0010:	chr6	73353062	73354276	-	655	1	DPPA5|ES	0	0.042
NR_02779	chr6	26421391	26430588	+	2476	1	BTN2A3P|	0.302	0.658
NM_0095{	chr6_GL0C	2922456	2924323	-	842.2	8	LTB|TNFC|	0	0
NR_02692	chr13	32504506	32509395	-	4889	1	N4BP2L2-I	0.09	0.199
NM_0010{	chr5	7830379	7851490	-	2379	1	C5orf49|-|	2.954	2.389
NM_0010:	chr16	29454501	29458224	+	1162	2	SLX1A|GIY	0	0
NM_0307!	chr7	75042161	75073881	-	2397	1	RCC1L|WE	17.536	15.046
NR_02438	chr10	42331866	42368045	-	6658	1	LOC44166	0.159	0.335
NM_1523:	chr14	1.05E+08	1.05E+08	+	1755	1	ADSSL1|M	1.66	1.491
NR_02961	chr12	53991738	53991847	+	109	1	MIR196A2	0	0
NM_0010:	chr1	15742492	15747797	+	1257	1	TMEM82|	0	0
NR_07320	chr21	40012416	40847113	-	6885	1	DSCAM|CI	0.024	6.513
NM_0070!	chr5	1.33E+08	1.33E+08	-	6247	1	KIF3A|FLA	8.7	8.487

NM_0029!	chr1	1.09E+08	1.09E+08	-	7004	1	SORT1|Gp	34.818	40.023
NM_0013:	chr2	1.72E+08	1.73E+08	+	5501	1	ITGA6|CD4	3.014	4.049
NM_0016:	chr3	1.05E+08	1.06E+08	+	4892	1	ALCAM|CD	6.331	4.667
NM_0010:	chr1	1.97E+08	1.97E+08	+	1812	1	CFH|AHUS	0	0.008
NM_0010(	chr1	1.58E+08	1.58E+08	-	943	1	OR10T2|O	0	0
NM_0013:	chr14	76151885	76196396	+	1818	1	GPATCH2L	8.248	7.406
NM_0006:	chr2	2.18E+08	2.18E+08	-	2482	1	CXCR1|C-(	0.005	0
NR_12168	chr4	13655179	13931228	+	2224	1	LINC01182	0.023	0
NM_0010:	chr2	99547254	1E+08	-	8094	1	AFF3|LAF4	10.426	12.211
NM_0183:	chr4	25234031	25279209	+	3589	1	PI4K2B|PI4	0.507	0.542
NM_0201!	chr7	7182615	7248649	+	6231	1	C1GALT1|(	1.051	1.719
NR_03413	chr15	82710471	82713993	-	1358	1	LOC33896:	0.104	0.151
NM_0011!	chr6	26457904	26469653	+	2836	1	BTN2A1|B	17.303	20.618
NR_03891	chr22	23893710	23898930	-	2189	2	MIF-AS1|M	0	0
NM_0000:	chr5	1.49E+08	1.49E+08	+	2039	1	ADRB2|AD	0.285	0.188
NM_0322:	chr11	13668659	13732346	+	5261	1	FAR1|MLS	3.919	5.309
NM_0010:	chr2	2.13E+08	2.13E+08	-	9482	1	IKZF2|ANF	0.337	0.748
NR_03414	chr6	22134602	22147193	-	2292	1	NBAT1|CA	0.281	0.436
NM_0012:	chr22	37010859	37020183	-	1100	1	TST|RDS|-	10.748	7.67
NM_0305:	chr16	69424595	69466264	+	4279	1	CYB5B|CYI	31.946	27.669
NM_0050!	chr1	35268967	35421944	+	6962	1	ZMYM4|C	12.84	14.693
NM_1833:	chr10	93061264	93068697	+	1562	1	CYP26C1|I	0	0.009
NR_03385	chr10	1.21E+08	1.21E+08	-	2497	1	WDR11-AS	0.806	0.987
NM_0325!	chr9	94374551	94460920	+	3284	1	MFSD14B|	8.583	7.67
NM_2136!	chr17	40958417	40966892	-	1687	2	KRT39|CK-	0	0
NM_0049:	chr9	22002903	22009313	-	3857	1	CDKN2B|C	0.174	0.33
NR_00367	chr8	37746556	37748046	-	1490	1	LOC72802	1.765	2.5
NR_03825	chr1	63159083	63317257	-	1808	1	LINC0046€	0	0.015
NR_02694	chr21	45288052	45297354	+	7162	1	LOC64285:	20.971	17.665
NM_0013(	chr15	42274168	42284424	+	1539	1	GANC|-|-|	4.239	4.45
NR_12639	chr2	1.88E+08	1.88E+08	-	493	1	LINC0109C	1.491	0.945
NM_1384!	chr6	1.18E+08	1.18E+08	+	4800	1	NUS1|C6o	2.353	2.271
NM_0142(	chr11	65116403	65117738	-	1333	1	ZNHIT2|C1	8.454	6.761
NM_0023	chr12	91103455	91111765	-	2111	1	LUM|LDC|	2.223	1.35
NM_1817!	chr1	2.1E+08	2.1E+08	+	1431	1	HSD11B1|	0	0.096
NM_0010(	chr11	4545191	4546144	+	952	1	OR52M1|(	0	0
NR_03388	chr2	1.32E+08	1.32E+08	+	615	1	POTEKP|A	0	0
NR_03838	chr13	44575896	44580432	+	2839	1	TSC22D1-/	2.48	3.397
NR_13563	chr17	909632	911212	+	818	1	LOC10192	0.11	0
NM_0065!	chr1	32013694	32043881	+	2880	1	KHDRBS1|	117.232	117.409
NM_0054	chr19	867962	893218	-	2264.5	2	MED16|DI	24.221	19.803
NR_03836	chr2	6729168	6770311	-	2125	1	LINC00487	0.334	0.516
NR_02686	chr6	1.66E+08	1.66E+08	-	1121	1	LINC00473	10.168	7.857
NM_0060(	chr16	85778625	85799542	-	1956	1	EMC8|C16	22.834	26.141
NM_0331:	chr9	1.22E+08	1.22E+08	-	3060	1	RBM18|-|	10.66	14.655
NM_1985(	chr17	4583981	4608319	+	1927	1	SMTNL2|-	1.124	1.223
NR_04622	chr7	23101228	23105703	-	2561	1	KLHL7-AS1	0.393	0.214
NR_00281	chr4	21843341	21853188	-	9847	1	KCNIP4-IT:	0	0.001
NM_0154!	chr11	63624082	63671730	-	7243	1	ATL3|HSN	0.826	0.936
NM_0031:	chr16	29663219	29670502	+	6902	1	SPN|CD43	0.034	0.006

NM_1984(	chr3	58742010	59049989	-	2533	1	C3orf67|-|	0.02	0.265
NR_02697	chr15	69561720	69571440	+	1700	1	DRAIC|-|-|	0.174	0.701
NM_0180!	chr16	70687439	70801158	-	3075	1	VAC14|ArI	24.059	25.505
NM_1750(	chr5	1.8E+08	1.8E+08	-	2200	1	RASGEF1C	4.87	3.798
NM_1527!	chr5	38937921	39074408	-	9501	1	RICTOR|A\	1.466	2.83
NM_0189:	chr3	71753724	71756380	+	2654	1	GPR27|SR	7.52	14.476
NR_10693	chr3	50273236	50273297	+	61	1	MIR6872|I	3.171	2.919
NM_0046:	chr9	35073838	35080016	-	2615	1	FANCG|FA	15.664	15.939
NM_0013(	chr16	57186284	57240475	+	3515	1	RSPRY1|SE	14.918	14.663
NM_0022{	chr19_GL9	407512	415408	+	695	4	LAIR2|CD3	0	0
NM_0040!	chr13	1.06E+08	1.07E+08	-	4380	1	EFNB2|EPI	34.032	66.465
NM_0063	chr13	72782092	73016453	+	3093	1	PIBF1|C13	1.342	2.134
NM_0011:	chr19	43414883	43418615	+	1155	1	TEX101|CT	0	0.119
NM_0139:	chr6_GL0C	690974	693202	-	2226	8	OR11A1|6	0	0
NM_0012!	chr5	1.4E+08	1.4E+08	+	3205	1	SLC4A9|AI	0.044	0.064
NM_0322!	chr5	94618686	94695868	+	3790	1	SLF1|ANKI	9.808	2.111
NM_0013:	chr19	45079905	45090916	+	931	1	GEMIN7|S	23.446	23.348
NM_0010(	chr1	2.48E+08	2.48E+08	+	1042	1	OR2M2|O	0	0
NM_0013	chr1	22643617	22648111	+	979	1	C1QC|C1C	0.026	0
NM_0011!	chr6	34514872	34535223	+	4276	1	PACSIN1|S	1.17	2.569
NM_0011:	chr9	75061200	75088217	-	1111	1	NMRK1|C9	2.913	4.056
NR_03606	chr1	1.8E+08	1.8E+08	-	76	1	MIR3121|	0	0
NM_0325!	chr17	50133735	50150517	-	4042	1	PPP1R9B|I	46.795	51.918
NM_0308!	chr21	44455486	44458856	+	2525	1	LRRC3|C2:	7.409	7.15
NM_0011(	chrX	1.53E+08	1.53E+08	-	654	1	CSAG1|CS/	0	0
NM_0024!	chr2	2.01E+08	2.01E+08	+	751	1	NDUFB3|E	69.017	50.575
NM_0011:	chr4	73436219	73455785	+	2022	1	AFP|AFPD	0.07	0.264
NM_0010:	chr2	2.04E+08	2.04E+08	+	1860	1	CTLA4|ALI	0	0
NR_10467	chr5	1.16E+08	1.16E+08	-	3255	1	LOC10192	0.004	0.004
NM_2068!	chr12	55636892	55637833	+	940	1	OR10P1|C	0	0
NM_1817(	chr11	31369830	31432835	+	2993	1	DNAJC24|	4.657	4.325
NR_03175	chr10	12725254	12725353	-	99	1	MIR548Q|	0	0
NM_0032!	chr3	12153068	12159351	-	1626	1	TIMP4|-|-	0.143	0.556
NR_02458	chr10	5638857	5666595	-	2571	1	ASB13|-|-	10.953	13.463
NR_10677	chr11	1.19E+08	1.19E+08	+	79	1	MIR6716|I	14.689	9.189
NM_0011:	chr21	44885954	44928873	-	2929	1	ITGB2|CD:	0.013	0.028
NM_0019!	chr1	94529176	94541857	-	2385	1	F3|CD142	0.443	0.563
NM_0216	chr1	1.62E+08	1.62E+08	+	2417	1	FCGR2A|C	0.005	0.011
NM_0011!	chr1	1.51E+08	1.51E+08	-	3620	1	MCL1|BCL	43.418	52.332
NM_0132:	chr17	3608262	3636322	-	3829	1	SHPK|CAR	6.041	4.851
NR_04623	chr10	1.33E+08	1.33E+08	+	5394	1	LINC01168	0	0
NR_12638	chr13	20102701	20103230	+	429	1	LINC01072	0	0
NM_1453:	chr19	21397119	21408799	+	1696	1	ZNF493|-|	1.863	1.123
NR_03842	chr1	1.47E+08	1.48E+08	-	3368	1	LINC00624	0.034	0.053
NR_03980	chr7	4788565	4788639	-	74	1	MIR4656|	1.045	0.185
NM_0010:	chr1	12892896	12898270	-	1519	1	PRAMEF1C	0	0
NM_0011:	chrX	1.01E+08	1.01E+08	-	3721	1	SYTL4|SLP	0.613	0.501
NM_0053{	chr2	2.31E+08	2.31E+08	-	2701	1	NCL|C23|-	166.503	152.645
NM_0010{	chr11	47354858	47378576	-	1401	1	SPI1|OF|P	0.018	0
NR_03622	chr21	30375294	30375378	-	84	1	MIR4327|	0	0

NR_00319	chr14	1.01E+08	1.01E+08	+	73	1	SNORD114	0	0
NM_0161	chr1	1216908	1232067	-	2013	1	SDF4|Cab4	47.475	47.766
NM_0067	chr19	7534152	7561767	+	4435	1	PNPLA6|B	12.373	11.402
NM_0529	chr1	1.85E+08	1.85E+08	-	6953	1	FAM129A|	0.647	0.24
NR_12163	chr8	85441806	85463054	-	618	1	CA3-AS1|-	0.063	0.266
NM_0157	chr22	41598028	41621057	-	3786	1	DESI1|D15	25.586	24.673
NM_0011	chrX	1E+08	1E+08	-	9608	1	PCDH19|E	4.085	2.278
NM_0181	chr19	14119509	14136628	-	1725	1	ASF1B|CIA	6.854	3.232
NM_0012	chr1	2189549	2194775	-	588	1	FAAP20|C	54.951	42.396
NR_10704	chr9	1.07E+08	1.07E+08	+	94	1	MIR8081|	0	0
NM_0063	chr1	46278400	46303366	-	2936	1	LRRC41|M	44.31	46.157
NM_1392	chr15	65001512	65029639	-	2735	1	MTFMT|C	3.456	4.232
NM_0010	chr9	1.34E+08	1.34E+08	-	811	1	TMEM8C|	0	0
NM_0000	chr12	57747727	57752447	-	1992	1	CDK4|CMI	62.862	75.368
NM_0229	chr3	49162535	49166352	-	1808	1	CCDC71|-|	16.673	17.682
NR_02748	chr1	1.44E+08	1.44E+08	+	1329	1	FCGR1CP|	0	0
NM_0011	chr20	37251086	37256908	-	464	1	GHRH|GH	0.445	0.325
NM_1523	chr1	59990394	60073770	-	2014	1	C1orf87|C	0.013	0.061
NR_12030	chr16	87766331	87779148	-	763	1	LOC10272	0.287	0.036
NM_0309	chr2	1.34E+08	1.35E+08	-	1883	1	TMEM163	52.293	27.467
NM_0246	chr12	1.23E+08	1.23E+08	-	2743	1	VPS37B|-|	28.617	21.142
NM_0011	chr19	49688685	49690990	+	2108	1	ADM5|AM	2.19	1.28
NR_04059	chr19	48255675	48258193	+	1961	1	CARD8-AS	0.302	0.189
NM_0032	chr21	42362282	42366535	-	487	1	TFF1|BCEI	0	0
NR_03890	chr11	12979534	12989548	-	1557	1	LINC0095	0.041	0.167
NR_10236	chrX	52063347	52063473	-	126	2	SNORA11E	0	0
NR_03387	chr4	1.01E+08	1.01E+08	+	791	1	FLJ20021|	10.563	9.385
NM_0037	chr6	1.44E+08	1.44E+08	+	5500	1	STX11|FHL	0.028	0
NM_0012	chr1	46303613	46316777	+	591	1	UQCRH|Q	95.665	105.103
NM_0038	chr8	22042753	22048808	+	1379	1	FGF17|FG	1.085	2.831
NR_13492	chr19	9538666	9539730	+	656	1	LOC10192	3.499	4.51
NM_0010	chr1	19312246	19329300	+	1633	1	PQLC2|-|-	11.449	9.101
NM_0010	chr10	43385617	43409248	-	2626	1	HNRNPF|H	47.97	41.07
NM_0528	chr1	43383908	43389812	-	1486	1	MED8|AR	29.023	22.49
NM_0043	chr4	1.4E+08	1.4E+08	-	2721	1	CLGN|-|-|	6.762	4.626
NM_0164	chr1	1.61E+08	1.61E+08	+	1114	1	UFC1|HSP	59.919	50.608
NM_0198	chr1	2.36E+08	2.36E+08	-	5036	1	ERO1B|ER	1.18	1.545
NM_0008	chr15	98648539	98964530	+	12212	1	IGF1R|CD	19.612	15.514
NM_0013	chr20	31861067	31870664	-	1103	1	DUSP15|C	3.016	1.763
NM_0052	chr3	98531899	98534679	+	2778	1	GPR15|BO	0	0
NM_1980	chr17	82101470	82212813	-	2978	1	CCDC57|-|	6.52	6.499
NM_0312	chr7	39950360	40097134	+	6779	1	CDK13|CD	6.509	6.955
NM_0067	chr18	58671386	58750139	+	5011	1	MALT1|IM	4.508	2.515
NM_0323	chr8	41529206	41545046	+	3831	1	GINS4|SLD	2.393	1.834
NM_0010	chr10	86668669	86736067	+	4953	1	LDB3|CMD	2.322	2.359
NM_1736	chr17	78423697	78577394	-	13640	1	DNAH17|D	0.406	0.433
NM_1784	chr20	44337158	44350792	+	756	1	R3HDML|	0	0
NM_0022	chr5	72816591	72914388	+	8532	1	TNPO1|IP	14.767	16.187
NR_02408	chr21	44999208	45004727	-	731	1	PICSAR|C2	0.106	0
NM_0041	chr5	218223	257082	+	2773	1	SDHA|CMI	24.866	31.242

NM_0012!	chr1	2.03E+08	2.03E+08	-	2238	1	CHIT1|CHI	0.006	0.037
NM_0039(	chr5	9035026	9546121	-	11783	1	SEMA5A|S	1.384	1.488
NM_0047;	chr10	68956123	68985069	+	4695	1	DDX21|GU	11.276	12.419
NM_0011;	chr16	88427471	88440757	+	13199	1	ZNF469|B(	0.076	0.037
NM_0069:	chr6_GL0C	3616737	3619145	+	1129	7	RNF5|RIN(	0	0.012
NM_0060(	chrX	55717677	55758774	+	2111	1	RRAGB|RA	20.882	25.486
NM_0011⁄	chr11	83455013	83682425	-	6109	1	DLG2|PPP	2.986	7.143
NM_0309:	chr20	142553	145751	+	467	1	DEFB126|(	0	0
NM_0048(	chr14	77505997	77616767	-	8150	1	SPTLC2|HS	6.032	4.761
NR_03062	chr14	23417987	23418063	-	76	1	MIR208B|	0	0.18
NM_0058(	chr13	30617693	30659549	+	3893	1	USPL1|C1:	5.72	6.245
NM_0211⁄	chr16	84565598	84618096	-	1852	1	COTL1|CLI	135.938	179.651
NM_0243:	chr3	14124940	14143680	+	3328	1	TMEM43|,	24.629	28.04
NM_1383;	chr6	1.33E+08	1.33E+08	-	1126	1	TAAR1|TA	0	0
NM_0013;	chr1	1.79E+08	1.79E+08	+	5838	1	FAM20B|g	11.407	13.221
NM_0018(	chr7	1.3E+08	1.3E+08	+	1495	1	CPA1|CPA	0	0
NM_0060:	chr12	1.23E+08	1.23E+08	-	2134	1	HCAR3|GP	0	0.026
NM_0217;	chr7	87934143	88182113	+	2850	1	ADAM22|/	4.501	2.725
NM_0160!	chr18	47155019	47176374	-	1499	1	IER3IP1|H!	13.238	12.957
NM_0012!	chr4	1.43E+08	1.43E+08	+	4110	1	USP38|HP	3.369	3.846
NR_13663	chr2	2.22E+08	2.22E+08	+	1154	1	LOC44093⁄	4.369	3.703
NR_04682	chr11	1.32E+08	1.32E+08	+	761	1	NTM-IT|N	0	0
NR_00336	chr13	40911889	40921774	-	1046	1	SUGT1P3|	0.419	0.249
NM_0332(	chr6	1312440	1314758	+	2316	1	FOXQ1|HF	0.078	0.059
NM_0323:	chr1	9539469	9582773	+	1452	1	SLC25A33|	4.023	4.858
NM_0011⁄	chr11	33040015	33073563	+	2621	1	TCP11L1|c	9.189	8.084
NM_0011;	chr14	31025470	31096450	+	4231	1	AP4S1|AP⁄	2.322	2.752
NR_00365	chr15	84631460	84642463	+	3947	1	SCAND2P|	6.314	7.055
NM_0803!	chr12	8513540	8522364	+	1926	1	CLEC4D|CI	0	0
NR_00259	chr2	2.06E+08	2.06E+08	+	131	1	SNORA41|	1.28	0.836
NR_11095	chr9	72305436	72315468	+	671	1	LINC01504	0	0
NM_0012⁄	chr9	1.22E+08	1.22E+08	-	3285	1	LHX6|LHX(	0.047	0.167
NM_1825:	chr1	1.52E+08	1.52E+08	-	1138	1	THEM5|A(	0.023	0.373
NM_0176(	chrX	1.3E+08	1.3E+08	-	4674	1	ZNF280C|!	1.001	1.456
NR_02692	chr2	2.38E+08	2.38E+08	+	2658	1	LOC64338	0.029	0.015
NM_0019(	chr11	1752752	1763992	-	2112	1	CTSD|CLN:	96.225	90.36
NM_0013;	chr20	31664452	31722552	-	2442	1	BCL2L1|B(	73.206	73.769
NM_0043!	chr5	1.5E+08	1.5E+08	-	1479	1	CD74|DHL	0.462	0.519
NM_0323(	chr16	727246	729715	+	1362	1	HAGHL|-|-	9.249	8.639
NM_0120!	chr3	57227737	57273470	+	6036	1	APPL1|API	3.448	4.119
NM_0013⁄	chr13	28700081	28719013	-	3090	1	SLC46A3|I	0.726	0.776
NM_0010(	chr9	1.23E+08	1.23E+08	-	928	1	OR1L8|OR	0.222	0.162
NM_0012(	chr18	11751472	11885684	+	5819	1	GNAL|DYT	11.817	10.531
NM_0012!	chr8	1E+08	1E+08	-	4287	1	RNF19A|R	5.88	6.888
NM_0132(	chr19	39602529	39607474	+	589	1	LGALS13|(	0	0
NR_03899	chr3	1.14E+08	1.14E+08	+	1710	1	ZBTB20-AS	0.03	0.184
NM_0010:	chr4	68537185	68568527	-	2065.5	2	UGT2B17|	0	0
NR_00322	chr14	1.01E+08	1.01E+08	+	70	1	SNORD11⁄	0	0
NM_0012!	chr1	2.41E+08	2.41E+08	-	2422	1	RGS7|-|-|:	3.444	5.254
NM_0180:	chr3	1.08E+08	1.08E+08	-	3190	1	IFT57|ESR	13.839	9.511

NM_0011	chr17	4785285	4786434	-	743	1	VMO1|ER	2.1	3.503
NR_13298	chr16	2268663	2273072	+	480	1	LOC10666	0.51	1.341
NM_1523	chr15	76059958	76204963	+	2386	1	TMEM266	1.502	3.479
NR_13461	chr6	17706130	17707344	+	1214	1	LOC10537	2.634	2.257
NR_12633	chr8	1.03E+08	1.03E+08	+	804	1	AZIN1-AS1	2.341	4.003
NM_0010	chr19	43872363	43880136	-	1839	1	ZNF404|-|	1.339	2.16
NM_0011	chr17	44850287	44899625	-	4030	1	EFTUD2|M	42.165	45.153
NM_0004	chr6	1.37E+08	1.37E+08	-	2111	1	IFNGR1|C	10.481	10.167
NM_0011	chr6	36243168	36308595	+	2384	1	PNPLA1|A	0.119	0.207
NR_02424	chr3	75421552	75435115	-	2498	1	FAM86DP	3.019	3.975
NM_1817	chr6	47782039	47826380	+	3473	1	OPN5|GPF	0.045	0.039
NM_1523	chr19	11798576	11808491	+	2818	1	ZNF491|-|	4.594	4.127
NM_0228	chr15	56090533	56243285	-	7185	1	RFX7|RFX	5.545	7.681
NR_00274	chr6	31835263	31835326	+	62.2	5	SNORD48|	0	0
NM_0012	chr1	1.6E+08	1.6E+08	+	1945	1	CASQ1|CA	0.802	1.07
NM_0204	chr1	2.28E+08	2.28E+08	+	2377	1	GJC2|CX4	1.524	2.034
NM_0010	chr11	19117145	19176420	+	2270	1	ZDHHC13|	9.167	12.273
NM_0013	chr12	49227926	49273338	+	1693	1	TUBA1C|T	35.52	21.909
NM_0043	chr22	23180365	23318037	+	6902	1	BCR|ALL|	34.806	33.881
NR_03965	chr4	60922619	60922684	+	65	1	MIR548AG	0	0
NR_02731	chr1	78229599	78369462	+	1185	1	MGC2738	0	0
NM_0010	chr5	1.28E+08	1.28E+08	+	6831	1	SLC12A2|	0.959	0.898
NM_0013	chr17	18271429	18315092	-	6599	1	TOP3A|TO	10.205	11.111
NR_02337	chr1	2.29E+08	2.29E+08	-	120	10	RNA5S15|	0	0
NM_0181	chr12	6310436	6328506	+	2864	1	PLEKHG6|	0.387	0.029
NM_2074	chr15	92617449	92655801	-	2624	1	FAM174B|	7.061	5.758
NM_0062	chr4	86594315	86815175	+	8456	1	PTPN13|F	7.738	5.554
NM_0245	chr19	17292131	17303473	-	2083	1	ABHD8|-|	21.262	23.948
NM_0020	chr19	51795146	51826190	+	2634	1	FPR3|FML	0	0.021
NR_02838	chr16	31180110	31194871	+	5070	1	FUS|ALS6|	86.203	67.818
NM_0013	chr20	37517417	37520303	-	1998	1	BLCAP|BC	155.787	241.501
NR_12068	chr9	1.29E+08	1.29E+08	+	748	1	LINC01503	4.602	10.035
NR_04003	chr18	76122998	76145255	+	2788	1	LOC33929	0	0.01
NM_0011	chr9	5629119	5769454	+	3789	1	RIC1|CIP1	2.477	3.21
NM_0072	chrX	49071156	49074045	-	1303	1	PRAF2|JM	76.569	66.856
NM_0072	chr1	15438442	15446658	+	889	1	CTRC|CLC	0.015	0.092
NR_03064	chr4	1986384	1986477	-	93	1	MIR943|M	9.982	3.682
NM_0012	chrX	17635293	17735993	+	8155	1	NHS|CTRC	0.885	1.07
NM_1387	chr11	64606174	64723188	-	6391	1	NRXN2|-|-	49.343	65.275
NR_03032	chr7	1.27E+08	1.27E+08	-	96	1	MIR592|M	0	0
NR_13465	chr4	54603211	54607131	-	2214	1	LOC33997	0.012	0
NM_0326	chr12	1.09E+08	1.09E+08	+	3743	1	USP30|-|-	11.64	14.418
NM_0014	chr2	2.36E+08	2.36E+08	-	1759	1	GBX2|-|-|	3.159	6.619
NR_13465	chr6	1.5E+08	1.5E+08	+	1274	1	LOC64596	0.374	0.29
NR_02703	chr12	94460003	94462568	+	2565	1	CEP83-AS1	0.96	1.02
NM_0051	chr11	1.25E+08	1.25E+08	-	1752	1	FEZ1|UNC	395.312	610.917
NM_0013	chr1	48532855	50023954	-	2946	1	AGBL4|CC	0.455	0.86
NM_0039	chr17	28473646	28497780	+	2343	1	SLC13A2|	0	0.058
NM_0010	chr20	23035420	23036677	+	1255	1	SSTR4|SS-	0	0
NM_0029	chr4	39287449	39366381	-	4869	1	RFC1|A1|	5.288	7.593

NR_13556	chr3	1.47E+08	1.47E+08	+	1867	1	LINC0201C	0	0
NM_1334(	chr9	1.12E+08	1.12E+08	+	3721	1	ZNF483|ZI	0.793	1.108
NM_0013(	chr6_KI27(	46826	60642	+	1502.9	9	BTNL2|BTI	0	0
NR_03648	chr7	1.22E+08	1.22E+08	+	2646	1	FEZF1-AS1	18.604	4.229
NM_0012{	chr12	43835861	44389762	+	2890	1	TMEM117	0.968	1.171
NR_02693	chr10	1.11E+08	1.11E+08	-	2014	1	PDCD4-AS:	0.883	0.796
NM_2068{	chr5	1.81E+08	1.81E+08	+	946	1	OR2V2|OF	0	0
NM_0059{	chr4	6693839	6697170	+	506	1	S100P|MI(	0.076	0.271
NR_12032	chr15	93856560	93878356	+	774	1	LOC10192	0	0
NR_04749	chr13	1.14E+08	1.14E+08	-	2477	1	LINC00565	1.28	1.123
NR_03961	chr1	22266239	22266300	+	61	1	MIR4418|-	0	0
NR_12053	chr11	1.27E+08	1.27E+08	+	471	1	LOC10192!	0.027	0.407
NM_0143:	chr4	1.38E+08	1.38E+08	-	9634	1	SLC7A11|(	0.95	0.813
NM_0012!	chr21	38400075	38661780	-	1527	1	ERG|erg-3	0.034	0
NM_0174:	chr4	76114659	76148515	-	2358	1	NUP54|-|-	9.607	11.083
NR_13567	chr17	50537294	50538754	-	604	1	LOC10537	0.363	0.181
NR_04005	chr15	40693755	40695105	-	1133	1	RAD51-AS:	1.9	2.079
NM_0010(	chr9	1.23E+08	1.23E+08	+	973	1	OR1L3|OR	0	0
NM_0208(	chr11	1.02E+08	1.02E+08	+	7033	1	CEP126|KI	0.563	0.837
NM_0010(	chr13	26044597	26051060	-	2885	1	SHISA2|C1	1.94	2.967
NM_0061:	chr18	49783504	49813881	-	1922	1	ACAA2|DS	16.556	17.674
NM_0243:	chr10	1.02E+08	1.02E+08	+	2356	1	FBXL15|FE	12.582	11.563
NM_0325(	chr6	28281537	28302549	+	3106	1	PGBD1|HL	9.888	8.291
NM_0013(	chr12	1.13E+08	1.13E+08	+	1286	1	SDSL|SDH	4.883	2.663
NM_1768{	chr12	11091287	11092313	-	1024	2	TAS2R43|"	0.013	0
NM_1730{	chr3	1.6E+08	1.6E+08	-	3871	1	TRIM59|IF	0.56	0.722
NM_0225:	chr1	1.86E+08	1.86E+08	-	1185	1	PDC|MEK/	0.054	0
NM_0052:	chr20	33675486	33686404	-	2691	1	E2F1|E2F-	9.98	5.655
NM_1814{	chr18	13278105	13652754	+	8476	1	LDLRAD4|(	2.151	3.166
NM_0011(	chr4	99412261	99435134	-	2091	1	ADH7|ADH	0	0.02
NM_1987!	chr10	1.14E+08	1.14E+08	+	4482	1	TDRD1|CT	0	0
NM_0013:	chr6	27388718	27403907	+	4045	1	ZNF391|dJ	1.897	1.466
NM_0010:	chr18	6834433	6915713	+	5512	1	ARHGAP2{	1.28	1.133
NM_0011.	chr4	1.13E+08	1.13E+08	+	14195	1	ANK2|ANK	34.36	35.461
NR_03983	chr10	1.2E+08	1.2E+08	+	79	1	MIR4682|-	0	0
NM_0123:	chr11	5151431	5152369	-	937	1	OR52A1|H	0	0
NM_0011!	chrX	9967358	9968002	+	643	1	CLDN34|C	0.04	0
NR_13125	chr2	1.08E+08	1.08E+08	-	606	1	LINC01594	0	0
NM_0046(	chr5	77691170	77776361	-	673	1	TBCA|-|-|!	89.68	102.758
NM_0011.	chr12	50327312	50396622	-	7255	1	FAM186A|	0.007	0.004
NR_04660	chrX	1.54E+08	1.54E+08	+	783	1	HCFC1-AS1	0.988	0.052
NR_03053	chr11	1996759	1996831	-	72	2	MIR675|M	0	0
NM_0149(	chr15	30903873	30943107	+	4873	2	FAN1|KIA/	0.394	0.548
NR_04670	chr3	64187544	64201560	+	3087	1	PRICKLE2-/	0.447	0.271
NM_0010{	chrX	1.5E+08	1.5E+08	-	1808	2	MAGEA9B	0	0
NR_02200	chr7	26533121	26538825	-	4318	1	KIAA0087|	0.2	0.238
NM_0182:	chr7	1.42E+08	1.42E+08	+	2533	2	AGK|CATC	4.428	4.742
NR_03606	chr1	66628440	66628517	+	77	1	MIR3117|ı	0	0.178
NM_0068.	chr16	283118	287209	+	1713	1	PDIA2|PD/	3.109	3.446
NR_02985	chr4	1.13E+08	1.13E+08	-	67	1	MIR302C|	0	0

NM_1737!	chr3	1.14E+08	1.14E+08	+	2962	1	TIGIT|VSIG	0.017	0.097
NR_03846	chrX	1.37E+08	1.37E+08	+	653	1	LINC00892	0	0
NM_0124!	chr19	48364394	48376377	+	1008	1	SYNGR4|-|	0.742	0.217
NM_0133!	chr22	38078137	38083163	-	1889	1	SLC16A8|I	0.403	0.181
NM_0808!	chr9	1.23E+08	1.23E+08	+	949	1	OR1K1|hg	0.027	0
NM_0010:	chr14	23376808	23379772	+	1101	1	CMTM5|C	0.269	0.224
NM_0024	chr19	40191744	40215575	+	3424	1	MAP3K10|	29.869	31.342
NM_0012!	chr1	13303539	13308907	+	1512	2	PRAMEF33	0	0
NM_0191	chr19	48755559	48758330	+	1306	1	FGF21|-|-	0.02	0.01
NM_0011	chr9	1.25E+08	1.25E+08	-	12121	1	SCAI|C9or	1.027	1.229
NR_03608	chr3	69048958	69049035	-	77	1	MIR3136|	0	0
NM_0010	chr8	1.44E+08	1.44E+08	+	999	1	SCX|SCXA	1.407	1.494
NM_0124	chr10	16590618	16817454	-	3742	1	RSU1|RSP	13.789	11.764
NR_01540	chr12	1.12E+08	1.12E+08	-	2283	1	MAPKAPK!	13.639	14.573
NM_0012	chr19	16496311	16518452	+	2515	1	C19orf44|	10.628	11.099
NM_0308	chr17	49710325	49764156	-	2351	1	FAM117A|	6.63	5.482
NR_12211	chr7	92134563	92180725	+	2477	1	CYP51A1-/	0.219	0.177
NM_0189!	chr1	16246840	16352491	-	6226	1	FBXO42|FI	14.285	18.273
NM_0011	chr21	30718525	30718777	-	250	1	KRTAP21-3	0	0
NM_0070:	chr5	659862	693395	-	6016	1	TPPP|TPPI	18.069	18.713
NM_1533	chr17	79022934	79049788	+	2709	1	C1QTNF1|	0.49	0.485
NR_10442	chr16	11275636	11276480	-	595	1	PRM2|CTS	0	0
NR_03031	chr6	1.35E+08	1.35E+08	+	96	1	MIR548A2	0	0
NM_0144	chr10	1.33E+08	1.33E+08	+	2407	1	VENTX|HP	0.064	0.074
NR_11094	chr16	87494187	87515630	+	541	1	LOC10192	1.168	0.861
NM_0054	chr4	76033687	76036197	-	1595	1	CXCL11|H	0	0
NR_12054	chr11	69485561	69493543	+	2907	1	LINC01488	0	0
NM_0011	chr2	2.31E+08	2.31E+08	+	3574	1	C2orf72|-|	5.386	7.565
NR_03977	chrX	1.1E+08	1.1E+08	+	100	1	MIR3978|	0	0
NM_0012	chr12	1.01E+08	1.01E+08	+	1844	1	NR1H4|BA	0.021	0.074
NR_12045	chr12	1.28E+08	1.28E+08	-	719	1	LOC10192	0	0
NR_10680	chr11	65129916	65129978	-	62	1	MIR6751|	2.703	1.105
NM_0011	chr4	15681729	15690447	+	4381	1	FAM200B|	4.897	5.124
NM_0052!	chr1	2.01E+08	2.01E+08	+	1221	1	GPR25|-|-	0.042	0.011
NR_12642	chr10	44993932	45000910	-	889	1	C10orf25|	9.906	14.205
NM_0162!	chr2	32357028	32618898	+	15626	1	BIRC6|APC	5.193	9.701
NM_0011	chr8	24384285	24406013	+	2214	1	ADAMDEC	0	0
NM_0022	chr13	49699307	49792921	-	4455	1	KPNA3|IPC	12.251	15.809
NR_03839	chr7	22563337	22573998	+	2430	1	LOC10050	0.223	0.152
NM_1814	chr20	63708028	63736142	+	1869	1	ZGPAT|GP	16.053	14.342
NR_02605	chr19	58575399	58584395	+	3339	1	CENPBD1P	14.303	13.221
NM_0143	chr19	58467045	58473578	+	3225	1	ZNF324|ZI	9.743	10.495
NM_0011	chr11	75150987	75206400	+	3900	1	SLCO2B1|	0.013	0.039
NM_0151	chr10	97356701	97401370	-	4390	1	RRP12|KIA	7.513	8.112
NR_02696	chr21	43450024	43453891	-	2999	2	LINC00319	0	0
NR_10466	chr5	55021299	55024169	-	547	1	LOC10246	0.024	0
NM_0011!	chr20	36574554	36612557	+	1083	1	TGIF2-C20	55.288	63.401
NM_0331	chr19	49054274	49055740	-	875	1	CGB7|CG-I	0.118	0.141
NM_0013	chr7	1.39E+08	1.39E+08	+	3997	1	TTC26|DYI	2.274	1.48
NM_1788	chr8	97644179	97730260	+	7653	1	MTDH|3D	8.439	9.597

NM_0012:	chr3	1.26E+08	1.26E+08	-	3162	1	ALDH1L1|:	0.228	0.446
NR_03973	chr15	74788672	74788757	-	85	1	MIR4513|-	0	0
NM_0011:	chr12	66347431	66679145	-	4990	1	GRIP1|GRI	9.565	6.278
NM_0013:	chr20	34704742	34713439	+	3991	1	TP53INP2|	41.869	39.128
NR_00157	chr5	55528842	55534542	-	5700	1	RNF138P1	0.197	0.327
NM_0013:	chr17	7855066	7856099	+	544	1	TMEM88|-	8.722	7.352
NM_0013:	chr9	1.28E+08	1.28E+08	+	715	1	SWI5|C9oı	29.43	21.206
NR_02421	chr19	47913051	47913170	+	119	3	SNAR-C2|-	0	0
NM_0011:	chr3	51707065	51718609	+	1925	1	GRM2|GLU	5.693	7.336
NM_0218:	chr4	1.55E+08	1.55E+08	-	2241	1	FGA|Fib2|	0	0
NM_0012{	chr1	1.58E+08	1.58E+08	+	7505	1	KIRREL|NE	7.872	18.898
NM_0051‹	chr1	1.68E+08	1.68E+08	+	2814	1	TBX19|TB!	0.797	1.017
NM_0013:	chr11	44932348	44950904	-	3298	1	TP53I11|P	108.39	96.145
NR_13426	chr5	37948491	37951054	+	543	1	LINC0211C	0	0.025
NM_0010:	chr3	44338119	44359802	+	1990	1	TCAIM|C3	7.509	3.965
NR_07346	chr8	28701473	28753690	+	3618	1	EXTL3|BO	56.171	76.678
NR_03984	chr12	81158388	81158461	+	73	1	MIR4699|-	0	0
NM_0002:	chr12	69348354	69354233	+	1510	1	LYZ|LYZF1	0.051	0.181
NM_0180:	chr3	1.84E+08	1.84E+08	+	6473	1	YEATS2|-|	22.174	23.143
NM_0064{	chr19	39825347	39834233	-	2437	1	DYRK1B|A	15.449	13.073
NM_0012‹	chr1	91949371	92014428	+	3682	1	BRDT|BRD	0	0
NM_0334:	chr14	77098235	77117287	+	4342	1	CIPC|KIAA	12.347	14.798
NM_0000{	chr17	50184096	50201639	-	5874	1	COL1A1|E	1.433	0.837
NR_03789	chr1	2.47E+08	2.47E+08	-	1112	1	ZNF670-ZN	1.635	2.229
NM_0010(	chr11	55554307	55555419	+	1111	1	OR4C15|C	0	0
NM_0010(	chr14	20117407	20118438	+	1030	1	OR4K17|C	0	0
NR_03032	chr8	11035206	11035302	-	96	1	MIR598|M	0	0
NM_0011‹	chr7	1.02E+08	1.02E+08	-	3135	1	SPDYE6|-|	0.033	0.07
NM_0068!	chr12	54000119	54003336	+	1511	1	HOXC9|HC	0.196	0.326
NR_04666	chr3	1.74E+08	1.74E+08	-	810	1	NLGN1-AS	0	0.034
NR_00326	chr17	14024498	14025598	+	1100	1	CDRT15P1	0.07	0.112
NM_0011!	chr8	92883530	92965589	-	3611	1	TRIQK|C8c	5.274	4.499
NM_1524:	chr8	95024986	95058716	+	1793	1	NDUFAF6|	2.84	4.263
NR_04560	chr10	7750962	7788027	-	3955	1	KIN|BTCD	2.031	2.549
NM_0184(	chr4	5051517	5501001	+	3496	1	STK32B|H!	6.122	3.981
NM_0012{	chr12	56470944	56488414	-	2279	1	GLS2|GA|	1.867	2.873
NR_04668	chr5	1.43E+08	1.43E+08	-	971	1	ARHGAP26	0.027	0
NM_0144(	chr4	69726968	69760712	-	1299	1	SULT1B1|S	0	0
NR_02952	chrX	1.52E+08	1.52E+08	-	80	1	MIR105-2|	0	0
NM_0012!	chrX	1.36E+08	1.36E+08	-	813	3	CT45A7|-|	0	0
NR_03836	chr11	18599804	18610255	+	1432	1	SPTY2D1-A	1.423	1.645
NM_0248:	chr5	1461423	1523977	-	3818	2	LPCAT1|A(	0.371	0.366
NM_2076‹	chr22	24585623	24593068	-	1234	1	LRRC75B|(	10.344	7.781
NM_0046:	chr1	1.79E+08	1.79E+08	-	3538	1	ANGPTL1|	0.109	1.425
NM_0051(	chr11	57233578	57237453	-	3873	1	APLNR|AG	1.222	0.23
NM_0043‹	chr3	48223347	48225491	+	739	1	CAMP|CAI	0	0
NR_10416	chr10	32347364	32374488	+	296	1	LOC10203	0.044	0.093
NM_0013(	chrX	1.51E+08	1.51E+08	+	3560	1	HMGB3|H	30.673	26.332
NM_0013:	chrX	1.19E+08	1.19E+08	+	2226	1	ZCCHC12|	72.843	65.181
NM_0010(	chr14	19935608	19936683	+	1073	1	OR4K1|OF	0	0

NR_10377	chr17	49505657	49574064	-	1218	1	LOC10028	1.069	0.967
NM_0203	chr6	1.38E+08	1.38E+08	+	14846	1	ARFGEF3|/	2.834	2.483
NM_0148	chr3	1E+08	1E+08	-	4367	1	TOMM70|	16.059	20.014
NM_0046	chr2	1.22E+08	1.22E+08	+	3399	1	TSN|BCLF-	28.511	31.754
NM_0208	chr3	1.09E+08	1.09E+08	-	4242	1	KIAA1524|	0.65	1.024
NR_03374	chrX	65550622	65552421	-	1799	1	FRMD8P1|	0.244	0.274
NR_04636	chr5	1.09E+08	1.09E+08	-	436	1	LINC01023	0.858	1.288
NM_0155	chr11	1.14E+08	1.14E+08	+	1151	1	REXO2|CG	9.566	11.567
NR_00273	chr12	56645027	56645101	-	74	1	SNORD59/	4.356	3.887
NR_02737	chr8	62977861	62984901	+	7040	1	UG0898H(	2.916	4.543
NM_0164	chr15	43800421	43802571	+	1327	1	HYPK|C15	35.251	24.029
NM_0011	chr6_GL00	2888786	2900754	+	1379	7	NFKBIL1|II	0	0
NR_03975	chr17	69099564	69099632	-	68	1	MIR4524A	0	0
NR_03972	chr13	96427229	96427292	+	63	1	MIR4501|-	0	0
NM_0012	chr8	28428409	28490318	-	1300	1	FBXO16|FI	12.586	9.778
NR_04668	chr7	79008988	79012277	+	473	1	MAGI2-AS	0	0
NM_0011	chr19	47220822	47232766	-	1345	1	BBC3|JFY-	18.693	24.227
NM_0033	chr19	18571802	18577460	+	2784	1	UBA52|CE	127.684	104.4
NM_0208	chr19	56538948	56556801	+	4077	1	ZFP28|mk	5.803	6.87
NM_0012	chr4	73571550	73620631	-	5782	1	RASSF6|-|	0	0
NM_0165	chr7	26201704	26213607	+	2094	1	CBX3|HEC	48.181	51.426
NM_0012	chr17	42950526	42964498	-	1350	1	AARSD1|-|	36.732	35.075
NM_0014	chrX	1.54E+08	1.54E+08	+	2468	1	GDI1|1A|(	560.731	556.677
NM_0010	chr2	95165417	95184316	+	3893	1	ZNF2|A1-5	3.362	4.957
NM_0040	chr2	72917475	72934891	+	2183	1	EMX1|-|-|	0.408	12.411
NR_02693	chr3	25790072	25794534	+	1069	1	OXSM|FAS	1.254	2.127
NR_13092	chr20	15892334	15923314	-	2025	1	LOC61326	1.496	0.034
NM_0010	chr5	191511	195353	+	1570	1	LRRC14B|-	0.041	0.026
NM_0012	chr2	1.44E+08	1.44E+08	-	2347	1	GTDC1|Hn	5.988	9.939
NR_12036	chr15	76174891	76181486	-	2351	1	LOC10192	0.197	0.297
NM_0012	chr15	85619950	85749358	+	9097	1	AKAP13|A	16.258	14.057
NM_0028	chr20	25248070	25298012	+	4109	1	PYGB|GPB	83.084	91.876
NM_0146	chr7	1.57E+08	1.57E+08	+	5156	1	UBE3C|HE	16.139	15.952
NM_0147	chr20	1266291	1309327	+	5011	1	SNPH|-|-|	9.657	17.029
NR_10675	chr21	33550662	33550728	+	66	1	MIR6501|I	39.267	43.166
NM_0041	chrX	47651792	47659180	-	590	1	UXT|ART-2	66.39	57.667
NR_03399	chr2	41877555	41894046	+	1807	1	LINC01913	0	0
NM_0054	chr20	40685848	40689240	-	3390	1	MAFB|DUI	0.996	2.566
NM_1450	chr11	5514393	5516726	-	2331	1	UBQLNL|-	0.05	0.094
NM_0022	chr2	25926586	25982574	-	5421	1	KIF3C|-|-|	122.829	164.655
NR_03021	chr19	53731006	53731090	+	84	1	MIR518A1	0	0
NM_2034	chrX	48509784	48520814	+	1728	1	PORCN|DH	14.296	15.813
NM_2069	chr11	85694221	85719180	-	2443	1	SYTL2|CHF	3.341	2.983
NM_0012	chr9	1.28E+08	1.28E+08	-	1283	1	TOR2A|TC	21.265	14.818
NM_0011	chr7	92144884	92165276	-	2654	1	LRRD1|-|-	0.019	0.175
NM_0206	chr14	69259964	69354473	+	3130	1	GALNT16|	15.015	30.606
NR_02706	chr7	1.06E+08	1.06E+08	-	759	1	EFCAB10|-	1.699	1.678
NR_03653	chr17	70166961	70169402	-	2441	1	KCNJ2-AS1	0.248	0.18
NR_02339	chr2	1.31E+08	1.31E+08	+	2244	1	CYP4F30P	0	0
NR_02738	chr5	69639463	69710527	-	1152.5	6	GUSBP3|-	0	0

NM_0011	chr11	45847118	45883248	+	4054	1	CRY2|HCR	18.431	19.714
NR_00290	chr1	28507365	28507571	+	206	1	SNORA73A	1.753	1.263
NR_13278	chr3	9385264	9396623	-	969	1	THUMPD3	6.972	7.294
NR_00295	chr12	48656648	48656782	-	134	1	SNORA2A|	0.192	0.92
NM_0067	chr2	1.19E+08	1.19E+08	+	1816	1	MARCO|S	0.021	0.068
NM_0058	chr7	45157768	45184251	+	1342	1	RAMP3|-|	0.058	0.102
NM_0010	chrX	1.44E+08	1.44E+08	+	1177	1	UBE2NL|Li	0.438	0.605
NR_04609	chr15	82647770	82692820	+	2134	1	CPEB1-AS1	0.949	0.956
NM_1781	chr11	6845683	6846636	+	952	1	OR10A5|J	0	0
NM_0010	chrX	1.02E+08	1.02E+08	-	3481	1	ZMAT1|-|	0.904	2.239
NM_0226	chr2	1.12E+08	1.12E+08	-	7686	1	ANAPC1|A	1.525	1.868
NR_14443	chr3	44427922	44429529	+	862	1	LINC0198	0.045	0.111
NM_0155	chr19	51056206	51065110	-	1251	1	KLK13|KLK	0	0
NR_12063	chr10	9197366	9295593	-	2265	1	LOC10192	0	0.079
NM_0068	chr12	1.06E+08	1.06E+08	-	3032	1	CKAP4|CLI	63.158	58.163
NM_0013	chr9	69820805	69906235	+	748	1	C9orf135|	0.31	0.11
NM_1472	chr2	24584477	24770701	+	6868	1	NCOA1|F-	12.413	17.476
NM_0806	chr1	1232249	1235040	+	2789	1	B3GALT6|	21.552	24.246
NM_0066	chr9	1.23E+08	1.23E+08	-	4100	1	ZBTB6|ZID	2.487	4.129
NR_10465	chrX	1.35E+08	1.35E+08	+	386	1	LOC10050	0.033	0.071
NM_0029	chr15	76931621	76950260	+	2204	1	RCN2|E6B	76.325	62.339
NR_02456	chr4	1195783	1208962	-	4977	1	LOC10013	0.034	0.03
NR_03842	chr2	27335542	27337803	+	837	1	GTF3C2-AS	12.031	17.232
NM_0045	chr1	42682235	42703803	+	3150	1	YBX1|BP-8	104.04	114.911
NM_0013	chr14	91965991	92040059	-	9970	1	TRIP11|AC	0.885	1.551
NM_0011	chrX	1.35E+08	1.35E+08	+	1533	1	SMIM10|C	0	0
NR_03623	chr4	28819582	28819668	+	86	1	MIR4275|	0	0
NM_0202	chr10	24848607	24952644	-	1973	1	PRTFDC1|	16.115	12.392
NM_0200	chr6_GL00	4154958	4160460	+	1144.8	8	HLA-DQA2	0	0
NR_10809	chr13	1.12E+08	1.12E+08	+	1286.5	2	LINC0107	0	0
NM_0049	chr1	1.1E+08	1.1E+08	+	4431	1	KCNC4|C1	3.41	4.495
NM_0012	chr8	93700507	93729250	+	1841	1	FAM92A|F	6.205	5.662
NM_0029	chr1	1.54E+08	1.54E+08	-	507	1	S100A4|1	2.365	1.54
NM_1334	chr19	48606743	48607714	+	969	1	SPACA4|S	0.027	0.071
NM_0149	chr10	274190	689668	-	7857	1	DIP2C|KIA	14.608	15.459
NR_00231	chr3	21405726	21406685	+	959	1	VENTXP7|	0	0
NR_04701	chr13	63667682	63738018	-	411	1	LINC0039	0	0
NM_0012	chr1	1.1E+08	1.1E+08	-	2486	1	SLC16A4|	0.545	0.909
NM_0012	chr10	72216000	72235860	+	3171	1	ANAPC16|	20.895	16.924
NM_0011	chr3	53495049	53812465	+	7543	1	CACNA1D|	0.779	0.844
NM_0121	chr21	30165923	30166653	-	728	1	CLDN17|-|	0	0
NR_13564	chr17	5469092	5470360	+	395	1	LOC10537	0	0
NM_0013	chr19	53066614	53087037	-	4065	1	ZNF160|F	6.214	10.617
NM_1832	chr1	92080305	92147841	+	1433	1	BTBD8|-|-	0.378	0.201
NR_04748	chr13	99086723	99088625	+	1902	1	DOCK9-AS	0.156	0.101
NM_0012	chr6	33318558	33323016	-	2347.8	5	DAXX|BIN	0.319	0.274
NR_10703	chr12	1.23E+08	1.23E+08	-	79	1	MIR8072|	0.653	0.173
NM_0019	chr1	1.69E+08	1.69E+08	-	1722	1	DPT|TRAM	0.052	0.008
NM_0073	chr15	79311062	79322847	+	1470	1	TMED3|C1	59.459	53.94
NR_04985	chr4	39411910	39411974	+	64	1	MIR5591|	0	0

NR_02702	chr15	77914217	77926846	-	3099	1	LOC64575	0	0
NM_0010	chr11	68736132	68751520	-	3617	1	TESMIN|C	0.168	0.08
NM_0012	chr5	35065089	35118122	-	1390	1	PRLR|HPR	0.046	0.463
NM_0013	chr1	31365253	31373288	-	1336	1	FABP3|FA	17.825	8.786
NM_0023	chr17	44759179	44781846	+	4394	1	ADAM11|	3.099	4.688
NM_0010	chr3	1.96E+08	1.96E+08	-	4205	1	TNK2|ACK	13.203	18.648
NM_1785	chr16	67184379	67190204	-	2481	1	EXOC3L1|	1.637	2.54
NM_0807	chrX	1.53E+08	1.53E+08	-	1251	1	TREX2|-|-	0.103	0.099
NM_0010	chr9	1.36E+08	1.36E+08	+	517	1	LCN9|HEL	0	0.026
NM_0050	chr8	1E+08	1E+08	+	965	1	POLR2K|A	59.951	53.922
NR_02950	chr2	2.18E+08	2.18E+08	+	76	1	MIR26B|	0	0
NM_2073	chr17	81822361	81833294	-	1278	1	MCRIP1|F	148.558	129.146
NM_1522	chr9	1.38E+08	1.38E+08	+	1592	1	ARRDC1|-	13.29	16.115
NM_0571	chr2	2.37E+08	2.37E+08	-	8717	1	COL6A3|B	0.422	0.266
NR_04746	chr10	91045808	91062159	+	536	1	LINC00502	0.096	0.153
NM_1384	chr22	50548033	50551023	+	2988	1	KLHDC7B|	0.056	0.138
NM_0011	chrX	1.54E+08	1.54E+08	-	4976	1	L1CAM|C	223.623	126.758
NM_0017	chr6	7726099	7881728	+	3775	1	BMP6|VGI	0.57	0.682
NM_0149	chr9	1.29E+08	1.29E+08	-	2201	1	DOLK|CD	11.119	11.04
NR_02452	chr8	93754844	93819232	+	3336	1	TMEM67|	1.909	1.733
NR_02809	chr6	1.6E+08	1.61E+08	-	1958	1	LPAL2|AP	0.092	0.126
NM_0240	chr17	18039297	18068404	+	4224	1	GID4|C17	3.858	4.692
NM_0208	chr19	22634324	22667670	+	4239	1	ZNF492|Z	0.012	0
NR_03415	chr19	39679374	39686373	+	1122	1	LGALS17A	0	0
NM_0011	chr12	85014315	85036277	-	1009	1	TSPAN19|	0.601	0.271
NM_0134	chr5	53480434	53486474	+	1826	1	FST|FS|-|	0.883	0.585
NM_0123	chr9	75088482	75147198	+	1301	1	OSTF1|OS	3.231	3.516
NM_0324	chr15	65895296	66253737	-	4360	2	MEGF11|-	0.207	1.608
NR_03665	chr15	84080187	84126601	+	1157	1	EFTUD1P1	0.167	0.13
NM_1530	chr1	54053572	54101648	+	6024	1	TCEANC2|	2.442	2.824
NM_0013	chr5	1.51E+08	1.51E+08	+	3514	1	IRGM|IFI1	0.07	0.152
NR_03625	chrX	1.13E+08	1.13E+08	-	70	1	MIR4329|	0	0
NM_0013	chr16	21834563	21857756	-	4039	1	NPIPB4|61	0.109	0.153
NR_03979	chr1	1.62E+08	1.62E+08	+	75	1	MIR4654|	0	0
NM_1530	chr6	18155388	18223853	+	3855	1	KDM1B|A	5.502	6.044
NR_02984	chr12	57824609	57824692	-	83	1	MIR26A2|	0.621	0
NM_0011	chr1	2.49E+08	2.49E+08	-	1732	1	ZNF692|A	22.363	21.495
NR_00222	chr14	1.06E+08	1.06E+08	-	1841	2	ADAM6|C	0	0
NM_0029	chr1	1.93E+08	1.93E+08	+	1396	1	RGS1|1R2	0	0
NR_03890	chr11	86955621	87000947	+	1206	1	LOC10050	0.182	0.239
NM_0204	chr1	1.54E+08	1.54E+08	+	5828	1	ATP8B2|A	12.814	13.446
NM_0013	chr11	35943981	36232136	+	3741	1	LDLRAD3|	9.161	16.538
NM_0011	chr13	48411046	48413517	-	2469	1	LPAR6|AR	0.047	0.105
NR_11030	chr11	95150539	95159148	+	1923	1	LOC10192	0	0
NM_0023	chr12	12116026	12266877	-	10059	1	LRP6|ADC	3.651	6.94
NM_0181	chr6	52420196	52495785	+	5569	1	EFHC1|EJ	7.33	4.762
NM_0028	chr3	1.59E+08	1.59E+08	-	877	1	RARRES1|	0.015	0.047
NM_0190	chr6	31142439	31158238	-	2605	6	CCHCR1|C	0.178	0.089
NM_0012	chr11	19224063	19241655	-	3452	1	E2F8|E2F-	0.235	0.317
NM_0121	chr1	84813403	84893213	-	1716	1	LPAR3|ED	0.075	0.048

NR_04682	chr4	53592956	53605381	+	1960	1	LNX1-AS2|	0	0
NM_0328	chr19	55591371	55599527	-	2655	1	FIZ1|ZNF7	13.501	11.933
NR_03616	chr22	27920525	27920612	-	87	1	MIR3199-	0	0
NM_0073	chr1	11012622	11025492	+	4209	1	TARDBP|A	30.189	32.321
NM_0177	chr8	60678765	60868027	+	11540	1	CHD7|CR	20.912	14.489
NM_0010	chr6	1.63E+08	1.63E+08	+	1538	1	PACRG|GL	3.68	6.893
NM_0013	chr16	67987390	67994291	-	1262	1	DPEP2|M	0	0
NR_13447	chr10	26577686	26579307	-	411	1	LOC10192	0	0
NM_0012	chr11	9664077	9752991	+	4695	1	SWAP70|	0.895	2.226
NM_0013	chr4	1.19E+08	1.19E+08	-	4066	1	SEC24D|C	4.535	4.976
NM_0013	chr7	76510342	76516521	+	1918.5	2	UPK3B|P3	0	0.021
NR_10671	chr19	14073361	14073479	+	118	1	MIR1199|	1.311	0.232
NR_04987	chr6	10439717	10439794	+	77	1	MIR5689|	0	0
NM_0010	chr1	2.48E+08	2.48E+08	+	937	1	OR14C36|	0	0
NM_0021	chr1	1.97E+08	1.97E+08	+	1289	1	CFHR1|CF	0.02	0.021
NM_0335	chr19	507497	519654	+	1110	1	TPGS1|C1	14.206	10.921
NM_0013	chr3	1.24E+08	1.25E+08	+	8106	1	KALRN|AR	18.928	11.811
NR_10988	chr5	1.25E+08	1.25E+08	+	416.5	2	LOC10192	0	0.066
NR_03997	chr1	52934930	52935017	+	87	1	MIR5095|	0	0
NM_0032	chr1	2.18E+08	2.18E+08	+	5911	1	TGFB2|G-	0.183	2.561
NM_0011	chr20	1368977	1393172	-	1514	1	FKBP1A|F	44.906	61.419
NM_0013	chr6	1.66E+08	1.67E+08	-	5569	1	RPS6KA2|	45.821	40.417
NM_0070	chr5	54977867	54985586	-	2084	1	ESM1|end	0.792	0.026
NM_0011	chr16	22513523	22536520	+	3807	1	NPIPB5|-|	0.322	0.497
NM_0010	chr3	15254356	15341394	-	3189	1	SH3BP5|S	27.404	57.601
NR_02724	chr21	28013363	28023209	+	581	1	LINC00314	0	0
NM_1825	chr7	53035656	53036925	+	1267	1	POM121L	0	0
NR_04981	chr4	13627865	13627957	+	92	1	MIR5091|	0	0
NR_03622	chr2	11836933	11836986	-	53	1	MIR4262|	0	0
NM_1384	chr15	42723562	42737219	-	4700	1	CDAN1|C	3.975	4.692
NM_0160	chr9	1.36E+08	1.36E+08	+	1505	1	MRPS2|C	33.592	37.423
NM_0028	chr11	1.2E+08	1.2E+08	-	5472	1	NECTIN1|	24.821	36.36
NM_0151	chr10	94402429	94536332	+	5734	1	TBC1D12|	2.943	3.234
NM_0142	chr14	75002909	75009591	+	1531	1	EIF2B2|EI	43.691	46.387
NM_0176	chr13	1.11E+08	1.11E+08	-	2525	1	ANKRD10|	18.669	21.584
NM_1815	chr11	68891278	68903835	-	687	1	MRPL21|L	68.859	67.289
NM_0040	chrX	31119228	31266907	-	4534	1	DMD|BM	1.914	3.338
NM_0011	chr1	13115488	13116854	-	1152	2	HNRNPCL	0	0
NM_0004	chr7	92487023	92528531	-	4356	1	PEX1|HM	5.994	7.043
NM_0145	chr17	3062669	3063607	-	937	1	OR1D5|C1	0	0
NM_0011	chr3	1.59E+08	1.6E+08	+	2372	1	IQCJ-SCHI	56.433	64.096
NM_1818	chr1	39491646	39529869	+	5617	1	BMP8A|-|	0.898	0.636
NR_07349	chr12	68804437	68805494	-	1057	1	LOC10013	0.061	0.13
NM_0011	chr16	4556490	4600317	+	3409	1	C16orf96|	0.333	0.301
NM_0011	chr3	84958982	86074429	+	9568	1	CADM2|I	3.551	2.395
NR_02691	chr21	13038166	13118250	+	3406	1	ANKRD30	0	0
NM_0121	chr6	73461731	73501456	+	2910	1	MTO1|CG	7.054	5.366
NM_0011	chr11	36400997	36465204	+	3564	1	PRR5L|PR	1.064	1.099
NR_00156	chr8	39451045	39522989	-	2405	1	ADAM3A|	0	0.011
NM_0010	chr22	37204237	37212376	-	4151	1	SSTR3|SS-	0.646	0.878

NM_0013:	chr19	10133344	10195054	-	5296	1	DNMT1|AI	23.518	20.633
NM_0805!	chr6	27138293	27146858	-	835	1	HIST1H2BK	10.099	9.137
NR_03414	chr20	23125069	23132636	-	1906	1	LINC0065€	0	0
NM_0011!	chr1	34981526	34985347	-	920	1	TMEM35B	1.486	0.357
NM_0010	chr16	1706220	1770317	+	5573	1	MAPK8IP3	43.699	46.574
NR_12049	chr12	67077626	67096284	-	504	1	LOC10272	0	0
NM_0011	chr3	42979267	43057716	+	3427	1	FAM198A|	0.015	0.004
NM_0147	chr12	57724293	57742161	-	4840	1	AGAP2|CE	27.814	35.941
NR_03072	chrX	12902817	12943300	-	1584	1	TLR8-AS1|	0	0.13
NR_13557	chr2	1.33E+08	1.33E+08	+	1709	1	LOC10192	0.008	0.056
NM_0019	chr14	1.03E+08	1.03E+08	+	5930	1	EIF5|EIF-5	40.6	35.652
NM_0010	chr1	1.59E+08	1.59E+08	-	952	1	OR6N2|OI	0	0
NM_0010	chr10	1.26E+08	1.27E+08	-	3069	1	C10orf90|	0	0.049
NR_12599	chr1	2.16E+08	2.16E+08	+	330	1	LOC10272	0	0
NR_03615	chr19	813584	813653	+	69	1	MIR3187|	190.973	161.585
NM_0190	chr1	62194802	62212328	+	3872	1	L1TD1|EC	0	0
NM_0010	chr9	1.28E+08	1.28E+08	-	3763	1	FAM129B|	19.181	24.486
NM_0061	chr17	50719565	50752711	+	1999	1	LUC7L3|CI	29.334	42.701
NM_0011	chrX	1.01E+08	1.01E+08	-	2172	1	TAF7L|CT	0.089	0.107
NM_0210	chr19	19710472	19733112	-	2957	1	ZNF14|GI	2.158	3.029
NR_00232	chr16	1962973	1963106	-	133	1	SNORA64|	3.199	3.913
NM_0010	chr10	33197619	33334905	-	2464	1	NRP1|BDC	17.205	8.989
NR_04702	chr13	88540829	88545509	+	850	1	LINC0043	0	0
NM_0049	chr11	67491768	67505372	-	4200	1	PITPNM1|	63.43	46.342
NM_0160	chr3	48241106	48270989	+	3358	1	ZNF589|S	13.769	20.546
NM_1981	chr1	26190628	26202542	+	1408	1	CATSPER4	0	0.088
NM_1788	chr2	2.28E+08	2.28E+08	+	1922	1	DAW1|OD	0.181	0.029
NR_03843	chr2	2.08E+08	2.08E+08	+	434	1	LOC10050	0	0
NR_13550	chr1	2049201	2050070	-	537	1	LOC10537	0	0
NR_12158	chr9	1.38E+08	1.38E+08	-	3028	1	LOC10192	0.273	0.167
NM_0027	chr19	11435645	11450967	+	2044	1	PRKCSH|A	126.777	101.769
NM_0010	chr1	1.59E+08	1.59E+08	+	940	1	OR10Z1|O	0	0
NM_0050	chr13	98683801	98752675	-	3102	1	SLC15A1|I	0	0
NM_0010	chr2	1.3E+08	1.3E+08	-	4318	1	POTEF|A2	0.012	0.336
NR_10691	chr9	33467869	33467935	-	66	1	MIR6851|	0	0
NM_0024	chr11	68030617	68036647	+	795	1	NDUFS8|C	122.545	96.637
NM_0205	chr5	7396230	7830081	+	6526	1	ADCY2|AC	11.489	8.523
NR_11086	chr6	25244916	25261407	-	1060	1	LOC10192	0.097	0.013
NM_0025	chr2	1.82E+08	1.82E+08	-	2998	1	NEUROD1	5.879	7.063
NM_0010	chr17	18476785	18494945	+	1706	1	LGALS9C|-	0	0
NM_0073	chr8	72021251	72075584	-	5161	1	TRPA1|AN	0.052	0.024
NM_0050	chr16	23581014	23596318	-	660	1	NDUFAB1|	115.475	113.727
NM_0188	chr6_GL0C	4234034	4250969	-	2526	8	TAP2|ABC	0	0.027
NM_0010	chr22	37608474	37633564	+	2882	1	GGA1|-|-|	23.496	25.412
NM_0329	chrX	1.04E+08	1.04E+08	+	1108	1	TCEAL3|W	22.552	19.544
NR_00439	chr7	1.49E+08	1.49E+08	+	101	1	RNY3|HY3	0	0
NR_00290	chr2	2.31E+08	2.31E+08	-	79	1	SNORD20|	0.326	0.173
NR_04626	chr2	1.3E+08	1.3E+08	-	2630	1	FAR2P2|-|	0.029	0.036
NM_0011	chr1	1.57E+08	1.57E+08	-	1995	1	ETV3|MET	3.367	3.501
NM_0806	chr11	67642555	67650659	-	1290	1	ACY3|ACY	0	0

NM_1471!	chr10	50305585	50623977	-	3740	1	SGMS1|M	1.627	2.934
NR_00368	chr3	52690738	52690830	+	92	1	SNORD19E	0.28	0.893
NM_0148:	chr1	1.13E+08	1.13E+08	+	4516	1	LRIG2|LIG-	3.917	3.503
NR_13449	chr10	42755142	42769621	+	625	1	LOC10537	0.041	0.153
NM_1817:	chr1	19682213	19799917	-	2956	1	TMCO4|-|	0.013	0.023
NM_0071:	chr1	2.16E+08	2.16E+08	-	6295	1	USH2A|RP	0.074	0.033
NM_1532:	chr17	6776233	6787646	+	1661	1	FBXO39|C	0	0
NM_2073!	chr19	32675407	32678300	+	2891	1	RGS9BP|P	0.308	0.587
NM_0009!	chr2	1.01E+08	1.01E+08	+	500	1	RPL31|L31	445.341	384.64
NR_03326	chr12	49389174	49392333	+	3159	1	LOC10033.	0.024	0.074
NR_12593	chr4	31997379	32155406	+	749	1	LOC10272.	0.017	4.316
NM_0056:	chr1	29147738	29182125	-	2522	1	SRSF4|SFR	63.194	66.074
NM_1756:	chr15_KI2	1983496	2106621	-	7681.5	2	TJP1|ZO-1	0.124	0.128
NM_0013(	chr10	15107866	15168710	-	2952	1	NMT2|-|-|	7.342	7.855
NM_0003:	chr2	1.27E+08	1.27E+08	+	1769	1	PROC|APC	0.226	0.062
NR_13122	chr13	89477607	89500509	-	665	1	LINC0104C	0	0
NM_0010:	chr1	1.53E+08	1.53E+08	+	2519	1	KPRP|C1oı	0	0
NM_0010{	chr19	1753663	1775445	+	1482	1	ONECUT3|	0.252	0.203
NR_02983	chr1	40757284	40757372	+	88	1	MIR30C1|	0	0
NM_0010!	chr10	69404202	69416918	-	2409	1	TACR2|NK	0.241	0.267
NR_04683	chr2	20590775	20592548	-	598	1	HS1BP3-IT	0.151	0.115
NM_1523:	chr15	86079808	87029052	+	3689	1	AGBL1|CC	0.07	0.022
NM_0201:	chr7	1.39E+08	1.39E+08	-	7156	1	ZC3HAV1|	0.452	1.145
NM_0016!	chr9	91213815	91361924	-	1576	1	AUH|-|-|9	4.958	4.45
NR_10679	chr1	1.46E+08	1.46E+08	-	58	1	MIR6736|	3.779	3.542
NR_13178	chr6	79803583	79812246	-	743	1	LINC01621	0.156	0.203
NM_0240!	chr11	62662817	62671769	-	1311	1	LBHD1|C1	47.856	39.67
NR_10269	chr1	1.1E+08	1.1E+08	+	530	1	LAMTOR5-	2.043	1.654
NM_0064:	chr5	1.6E+08	1.6E+08	-	3542	1	SLU7|9G8	11.059	11.609
NM_1768!	chr3	1.51E+08	1.51E+08	-	2765	1	P2RY13|FI	0	0.005
NM_1987!	chr20	50794894	50877177	+	1274	1	BCAS4|CN	9.432	12.944
NR_10692	chr17	40161933	40162001	+	68	1	MIR6866|	9.291	19.941
NM_0012{	chr9	1.3E+08	1.3E+08	+	1485	1	NTMT1|AI	40.426	45.408
NR_00129	chr7_KI27(	773503	777145	+	857	1	PRSS3P2|T	0	0
NR_00283	chr18	41480271	41520597	-	1707	1	KC6|-|-|-|	1.412	1.653
NM_0010{	chr2	1.82E+08	1.82E+08	+	1071	1	PPP1R1C|	0.337	0.537
NM_0014:	chrX	49589529	49596837	+	531	1	GAGE2C|C	0	0
NR_02746	chr13	75237753	75240381	-	2628	1	CTAGE11P	0.025	0.016
NM_0529(	chr6	35943514	36024636	-	3412	1	SLC26A8|S	0.042	0.032
NM_0323:	chrX	1.34E+08	1.34E+08	+	1200	1	PHF6|BFLS	11.045	14.93
NM_0016:	chr3	1.42E+08	1.42E+08	+	1888	1	ATP1B3|A	78.549	90.547
NM_0013(	chr12	10442264	10454685	-	1168	1	KLRC1|CD	0.044	0
NM_1334!	chr5	1.33E+08	1.33E+08	-	3360	1	SHROOM1	0.123	0.126
NM_0330!	chr11	5663195	5685109	-	2019	1	TRIM5|RN	2.35	2.32
NM_0019⁄	chr12	89347825	89352859	-	3390	1	DUSP6|HH	7.074	4.016
NM_1389!	chr22	21769030	21867680	-	1487	1	MAPK1|EI	31.18	40.759
NR_12543	chr16	15094411	15109197	+	2594	2	LOC10050.	0	0
NM_0012{	chr18	54357917	54382035	+	5682	1	C18orf54|	0.322	0.301
NR_11013	chr11	56848478	56878078	+	2778	1	LOC10192	0.065	0.143
NM_1826:	chr17	59109947	59155439	-	2966	1	SKA2|FAM	20.566	21.889

NM_0013(	chr19	46838158	46850769	-	822	1	AP2S1|AP:	89.172	90.897
NR_10445	chr6	1.5E+08	1.5E+08	-	2229	1	NUP43|bA	8.607	8.72
NR_03321	chrX	71103889	71106788	-	857	1	CXorf65|-|	0.015	0.192
NM_0165(	chrX	76172936	76178198	+	1187	1	PBDC1|CX	17.336	14.239
NM_0010(	chr11	4907370	4908308	+	937	1	OR51A7|C	0	0
NM_0012{	chr15	90083980	90100621	-	1563	1	IDH2|D2H	108.922	83.733
NM_1829;	chr9	1.36E+08	1.36E+08	-	1519	1	GLT6D1|G	0	0
NM_0038(	chr16	66934444	66945091	+	3924	1	CES2|CE-2	23.102	20.636
NM_0124!	chr11	1.12E+08	1.12E+08	-	807	1	TIMM8B|[	40.837	35.507
NR_10687	chr22	49962866	49962939	+	73	1	MIR6821|I	8.831	6.567
NR_10705	chr8	93029751	93029839	+	88	1	MIR8084|I	0	0
NM_0120(	chr1	19282563	19288786	-	1232	1	AKR7A3|A	0.712	0.767
NM_0211!	chr2	74221434	74315025	-	3421	1	SLC4A5|NI	1.255	1.325
NM_0012;	chr1	2.01E+08	2.01E+08	-	1418	1	ASCL5|bHI	0.218	0.135
NM_0012{	chr7	90709816	91210590	+	4867	1	CDK14|PF	12.925	11.406
NM_0011(	chr5	55738017	55816777	+	2428	1	DDX4|VAS	0.069	0.028
NM_0010(	chr1	68098459	68232546	-	2038	1	WLS|C1or	114.308	62.12
NM_0011(	chr11	89870438	89876017	-	1526	1	TRIM64B|-	0	0.081
NR_13804	chrY	8638294	8645129	+	624	1	LINC0027S	0	0.022
NM_0004	chrX	22032444	22251310	+	6145	1	PHEX|HPD	0.235	0.16
NM_0252:	chr2	2.19E+08	2.19E+08	+	2369	1	WNT10A|(	0.376	0.029
NR_02690	chr18	26865308	26935946	+	2384	1	AQP4-AS1	0.032	0.109
NM_0247;	chr16	27203486	27221768	+	2461	1	KDM8|JM.	3.552	2.972
NM_1522:	chr14	34561412	34630160	-	2959	1	SNX6|MST	14.079	13.401
NM_0057	chr1	22001656	22012542	+	897	1	CELA3A|EI	0	0
NR_02728	chr10	1.23E+08	1.23E+08	+	2106	1	LOC39981.	0.012	0.013
NM_0239:	chr12	10825346	10826269	-	922	3	TAS2R10|"	0	0
NM_0012{	chr9	93451696	93532589	+	2377	1	FAM120A|	3.868	4.137
NM_1735:	chr2	70808645	70820600	-	2464	1	CLEC4F|CL	0.021	0.017
NM_0209(	chr2	18554723	18560693	-	1596	1	RDH14|PA	6.487	8.865
NR_01544	chr9	92132834	92138629	+	1999	1	LOC10012:	0.142	0.007
NM_0249	chr4	38966746	39032421	-	1924	1	TMEM156	0.047	0.028
NR_03993	chr2	2.31E+08	2.31E+08	+	85	1	MIR4777|-	0	0
NM_1522!	chr8	7482504	7486387	-	299	3	DEFB106A	0	0
NM_0011:	chr6	1.65E+08	1.66E+08	-	8171	1	PDE10A|A	3.415	7.637
NR_01542	chr1	2.47E+08	2.47E+08	+	1121	1	LINC01341	1.231	0.696
NR_00243	chr8	56073839	56073901	-	62	1	SNORD54|	1.872	2.651
NM_1524(	chr5	79236102	79327215	+	9116	1	JMY|WHA	1.246	2.871
NM_0122!	chr2	1631887	1744547	-	6824	1	PXDN|COF	16.03	12.437
NR_03373	chr4	67991812	67997439	-	337	1	TMPRSS11	0	0
NM_0003:	chr15	48206301	48304078	+	4680	1	SLC12A1|E	0.118	0.032
NM_0010:	chr19	57151726	57167488	-	3338	1	DUXA|-|-|	0	0.004
NM_0003!	chrX	37780017	37813461	+	4303	1	CYBB|AM(	0.012	0.025
NM_0010(	chr11	56663686	56664618	+	931	1	OR5AR1|C	0	0
NR_02428	chr9	89639783	89719759	+	2188	1	UNQ6494|	0.118	0.163
NR_02417	chr4	99878338	99894546	-	4228	1	LAMTOR3	14.19	13.473
NR_03996	chr5	72878591	72878663	+	72	1	MIR4804|-	0	0
NR_02841	chr22	44569340	44572449	+	715	1	LINC00207	0.252	0.038
NM_0240:	chr4	1.13E+08	1.13E+08	-	2283	1	NEUROG2	13.396	42.291
NM_0012:	chr16	30378314	30400108	+	2872	1	ZNF48|ZNI	44.835	41.716

NM_0027!	chr15	66386873	66491544	+	2573	1	MAP2K1|(	17.393	19.579
NM_1481	chr17	17505563	17577465	-	886	1	PEMT|PEA	24.346	22.462
NM_0010	chr5	1.28E+08	1.28E+08	+	1678	1	CTXN3|KA	1.114	0.726
NM_0018	chr9	98943713	99070792	+	5378	1	COL15A1|	0.067	0.181
NM_2015	chr10	18140677	18541759	+	3948	1	CACNB2|C	1.208	2.019
NM_1981	chr2	9951663	10002284	+	3587	1	GRHL1|LB	1.434	1.474
NR_14444	chr19	58278966	58315657	+	4680	1	ZNF8-ERVK	21.847	20.797
NM_0179	chr17	80036632	80100613	+	4276	1	CCDC40|C	6.622	4.142
NM_0013	chr17	75476498	75500452	+	5378	1	TMEM94|	24.325	22.344
NR_04634	chr8	7556138	7559104	-	1841	4	FAM90A7I	0	0
NR_12575	chr14	28725242	28765319	-	898	1	FOXG1-AS	0.029	1.327
NR_04980	chr13	41568286	41568395	-	109	1	MIR5006|	0.473	0.503
NM_0532	chr17	6799981	6831741	-	1403	1	TEKT1|-|-|	0.248	0.254
NM_0183	chr9	76385517	76394528	-	2701	1	RFK|RIFK|	12.97	11.643
NM_0010	chr19	1438364	1440497	+	529	1	RPS15|RIG	257.09	185.415
NM_0034	chr12	1.33E+08	1.33E+08	+	7025	1	ZNF84|HP	8.254	15.674
NM_0007	chr1	2.06E+08	2.06E+08	-	1769	1	AVPR1B|A	0.044	0.116
NM_1527	chr4	1.28E+08	1.28E+08	-	4530	1	MFSD8|CC	1.466	1.935
NR_03058	chr14	1.01E+08	1.01E+08	+	81	1	MIR300|M	0	0
NM_1813	chr3	1.79E+08	1.79E+08	+	2674	1	KCNMB2|-	0.54	1.649
NM_1749	chr9	35658290	35661503	+	1251	1	CCDC107|	20.17	18.208
NM_0064	chr13	53028741	53052061	+	2912	1	OLFM4|G(	0.089	0.085
NM_0013	chr15	87977365	88256169	-	3685	1	NTRK3|GP	7.148	13.214
NM_1817	chr1	62236165	62319412	-	5465	1	KANK4|AN	0.231	0.388
NM_0013	chr2	27130756	27134675	-	1996	1	PREB|SEC	33.332	34.627
NM_0012	chr2	2.42E+08	2.42E+08	+	2227	1	D2HGDH|I	4.869	7.903
NM_0223	chr17	32328441	32342209	-	4571	1	C17orf75|	7.923	5.502
NM_0013	chr16	72084853	72093651	-	923	1	TXNL4B|D	9.234	9.141
NM_0014	chr19	39834453	39846414	-	1154	1	FBL|FIB|FI	81.171	75.773
NM_1735	chr12	80209453	80379090	+	8023	1	OTOGL|C1	0.061	0.014
NM_0151	chr17	76718443	76726799	-	1814	1	JMJD6|PSI	18.032	16.664
NM_0034	chr19	58561917	58573575	-	2595	1	MZF1|MZI	11.587	10.208
NM_0176	chr1	1.61E+08	1.61E+08	-	1172	1	ITLN1|HL-	0	0
NM_0012	chr3	319683	409414	+	7286	1	CHL1|CALI	0.018	0.045
NR_03621	chr20	57321502	57321591	-	89	1	MIR4325|	0	0
NM_0011	chr6	46746917	46759217	+	1050	1	ANKRD66|	0	0.013
NR_13457	chr18	69469931	69471736	+	345	1	LOC10537	0	0
NR_04767	chr9	69296681	69307056	-	671	1	BANCR|LII	0	0.02
NR_02851	chr15	76259289	76260152	-	863	1	TYRO3P|-|	0.403	0.587
NM_0012	chrX	15825806	15855014	-	2322	1	AP1S2|DC	18.185	17.03
NM_0019	chr18	31498004	31549007	+	5852	1	DSG2|CDH	1.117	0.758
NM_0165	chr12	10069554	10099065	-	2757	1	CLEC1A|CI	0	0.005
NM_0013	chr16	30024423	30030084	-	1478	1	FAM57B|F	119.871	87.205
NR_02832	chr11	126987	131920	-	4611	1	LINC01001	0.014	0.015
NM_0333	chr8	26765854	26865655	-	2246	1	ADRA1A|A	0.907	1.214
NM_0309	chr6	24704861	24719175	-	2466	1	C6orf62|N	27.209	32.987
NR_11056	chr5	1.8E+08	1.8E+08	-	1957	1	LOC10192	0.876	1.029
NM_0012	chr10	1.2E+08	1.2E+08	-	4274	1	MCMBP|C	15.762	15.594
NR_12040	chr13	52334295	52341883	+	836	1	LOC10319	0	0
NM_0011	chr12	1.13E+08	1.13E+08	-	4359	1	DDX54|DP	8.211	9.251

NR_12208	chr4	36256518	36274215	+	1315	1	LOC43993	0	0
NM_0305	chr10	7559270	7666998	-	6732	1	ITIH5|ITI-H	0.889	1.727
NM_0000	chr2	2.27E+08	2.27E+08	-	10291	1	COL4A4|C	0.046	0.007
NR_12591	chr13	1.02E+08	1.02E+08	+	353	1	FGF14-AS1	0	0
NM_0209	chr7	1.02E+08	1.02E+08	+	2195	1	SH2B2|AP	11.725	10.833
NM_1749	chr15	42942900	43106088	-	7696	1	UBR1|JBS|	3.433	4.325
NM_0011	chr1	2.03E+08	2.03E+08	+	8587	1	PPP1R12B	6.662	7.439
NM_0014	chr8	81478497	81483238	-	832	1	FABP4|A-F	0.356	0.181
NM_0198	chr9	35957108	35958154	-	1044	1	OR2S2|OR	0	0
NM_0012	chr11	99020976	1E+08	+	6307	1	CNTN5|HN	1.38	0.402
NR_13550	chr15	32665702	32673065	-	457	2	LOC10537	0	0
NR_07316	chr7	1.51E+08	1.51E+08	-	3822	1	ATG9B|AP	0.455	0.713
NM_0147	chr5	1.42E+08	1.42E+08	+	4944	1	KIAA0141|	13.225	9.226
NM_0026	chr14	39175183	39183218	+	3595	1	PNN|DRS|	15.655	21.073
NR_12164	chr11	5244554	5245546	-	992	1	BGLT3|BG	0	0
NM_1825	chr11	66278201	66284214	+	1348	1	CNIH2|CN	111.077	95.777
NM_0013	chr12	8221260	8227618	-	2351	1	FAM90A1|	0.11	0.408
NM_0154	chr3	51975026	51981200	+	1101	1	ABHD14A|	37.708	26.71
NR_00484	chr5	80299096	80300478	-	1382	1	LOC64493	0.793	0.951
NR_04673	chr3	9947404	9954787	+	610	1	PRRT3-AS1	1.416	1.662
NM_0012	chr16	82626794	83796610	+	3889	1	CDH13|CD	3.955	5.248
NR_00688	chr17	19190029	19190245	-	216	1	SNORD3C|	0	0
NM_0032	chr16	1240677	1242554	+	1186	1	TPSAB1|TI	0.043	0.023
NR_02683	chr2	5932687	5980218	+	11866	1	LINC01105	0.422	0.725
NM_0011	chr10	1.04E+08	1.04E+08	+	821	1	GSTO2|GS	1.115	1.318
NM_0056	chr2	2.31E+08	2.31E+08	-	3824	1	GPR55|LPI	0.051	0.021
NR_00296	chrX	1.55E+08	1.55E+08	+	129	1	SNORA36A	0.4	0.212
NM_1826	chr15	89896532	89912990	-	6091	1	ARPIN|C15	9.155	9.544
NM_0055	chr7	27181157	27185216	-	2649	1	HOXA11|H	0	0
NM_0124	chr12	1.08E+08	1.08E+08	-	4173	1	PRDM4|PI	14.457	15.079
NM_1735	chr1	26361634	26372794	-	1661	1	ZNF683|H	0.039	0.016
NM_0010	chr7	75092556	75149817	+	3567	1	GTF2IRD2E	0.416	0.384
NM_0224	chr9	37485935	37503697	+	1854	1	POLR1E|P	14.973	21.292
NR_00278	chr20	58818918	58850903	-	1152	1	GNAS-AS1	0.022	0.38
NR_03168	chrX	1.18E+08	1.18E+08	+	77	1	MIR1277|	0	0
NR_02961	chr1	9151668	9151777	-	109	1	MIR34A|M	0	0.251
NM_0037	chr9	1.09E+08	1.09E+08	-	2536	1	CTNNAL1|	2.451	2.371
NM_0011	chr2	48617780	48679611	+	1589	1	GTF2A1L|	0.114	0.284
NR_00129	chr15	24985100	24985166	+	66	1	SNORD64|	0.391	1.038
NM_0334	chr3	36712422	36739861	-	5337	1	DCLK3|CLI	0.239	0.313
NM_0012	chr6	41149261	41154349	-	1280	1	TREML1|G	0	0
NM_0178	chr19	19119616	19138501	-	1813	1	TMEM161	9.373	9.784
NR_13506	chr1	11099675	11102106	+	444	1	LOC10537	1.568	1.203
NM_1383	chr4	44701795	44726634	-	2304	1	GNPDA2|C	1.673	4.132
NM_1785	chr17	81311271	81330674	-	3315	1	TMEM105	0.202	0.256
NM_1522	chr2	1.78E+08	1.78E+08	-	4666	1	TTC30A|IF	1.409	1.071
NM_0012	chr9	1.28E+08	1.28E+08	+	2463	1	CDK9|C-2k	26.562	26.749
NM_0050	chr17	82031656	82034204	+	1052	1	RAC3|-|-|	84.31	88.848
NM_0011	chr4	8366282	8440725	-	2787	1	ACOX3|-|-	7.731	9.642
NM_0010	chr4	69096474	69112987	+	1880	1	UGT2B7|U	0.439	0.401

NR_02729	chr9	38621088	38623280	+	1725	1	FAM201A|	0.232	0.095
NR_03825	chr15_KI2	2944256	3058736	+	5550.5	2	LOC10028	0.009	0.025
NM_0183	chr2	1.48E+08	1.49E+08	+	5999	1	MBD5|MF	4.324	5.4
NM_0212	chr6_GL0C	1637069	1653960	+	3328	7	TRIM39|R	0.263	0.189
NM_0012	chr4	9325165	9326757	+	1591	8	USP17L27	0	0
NR_12579	chr16	86565145	86567761	-	317	1	FOXC2-AS	0.163	0.086
NR_04621	chr7	1.28E+08	1.28E+08	-	10078	1	MGC2734	1.323	1.329
NR_04972	chr17	10715943	10716582	-	639	1	MAGOH2P	4.015	3.773
NM_1523	chr1	26949556	26960410	-	1797	1	KDF1|C1o	0	0
NM_0011	chr16	67482571	67483813	-	758	1	AGRP|AGF	0.102	0.072
NM_0141	chr18	74148511	74158969	+	1526	1	TIMM21|C	13.755	13.5
NM_0054	chr2	2.19E+08	2.19E+08	+	3424	1	CNOT9|CA	28.57	30.258
NR_00304	chr6	29582252	29582328	+	76	7	SNORD32E	0	0
NM_1748	chr3	1.51E+08	1.51E+08	-	2354	1	CLRN1|RP	0	0
NR_02971	chr1	2.2E+08	2.2E+08	-	84	1	MIR194-1|	0	0
NR_11095	chr9	1.15E+08	1.15E+08	-	1519	1	LOC10192	0	0
NM_0043	chr21	44849585	44873903	-	2818	1	PTTG1IP|C	118.878	93.745
NM_0221	chr3	1.87E+08	1.87E+08	+	1007	1	RTP4|IFRG	0	0.014
NM_0011	chr9	69035563	69100178	+	973	1	FXN|CyaY|	3.525	3.196
NM_0011	chr12	1.32E+08	1.32E+08	-	2650	1	GALNT9|G	2.155	2.331
NM_0163	chr12	14786478	14803467	-	2660	1	WBP11|NI	37.832	33.805
NM_0218	chrX	15561033	15602069	-	3486	1	ACE2|ACE	0.03	0.039
NM_1387	chr15_KI2	1543700	1545367	-	1665	2	NSMCE3|H	0	0
NM_0053	chr6	26156331	26157115	+	782	1	HIST1H1E|	0.445	0.28
NR_13630	chr9	40992262	41033012	+	995	1	LOC10272	3.058	4.033
NM_0019	chr19	3976056	3985463	-	3141	1	EEF2|EEF-	1170.963	1323.844
NR_02200	chr15	24984860	24988232	+	3372	1	PWAR5|D	0.585	1.198
NR_04004	chr20	13237420	13239687	-	1043	1	ISM1-AS1|	0.025	0
NR_11042	chr18	3466250	3478978	+	498	1	GAPLINC|L	0.259	0.138
NM_0331	chr8	66021556	66100520	+	1372	1	DNAJC5B|	0.038	0.479
NM_0010	chr16	67666814	67668758	+	1263	1	C16orf86|	2.848	1.985
NM_0013	chr2	46511835	46542557	-	1584	1	ATP6V1E2	2.23	4.228
NM_0001	chr5	1.5E+08	1.5E+08	+	8070	1	SLC26A2|D	1.293	1.417
NR_02986	chr14	1.01E+08	1.01E+08	+	74	1	MIR370|M	0	0
NM_1758	chr1	2.31E+08	2.31E+08	-	5139	1	EXOC8|EX	2.012	2.321
NM_0023	chr2	19992052	20012694	-	2573	1	MATN3|D	0.135	0.176
NR_12158	chr9	1.38E+08	1.38E+08	-	3218	1	LOC10013	0.208	0.123
NR_00243	chr6	1.33E+08	1.33E+08	+	72	1	SNORD101	0.179	2.473
NM_0035	chr6	27837880	27838339	-	458	1	HIST1H2AI	1.548	1.346
NM_1818	chr10	1.17E+08	1.17E+08	+	1151	1	KCNK18|K	0	0
NR_00442	chr1	1.46E+08	1.46E+08	-	163	9	RNU1-2|R	0	0
NM_0027	chr19	53882184	53907647	+	3137	1	PRKCG|PK	12.162	10.514
NR_13561	chrX	41275739	41276777	+	832	1	LOC10537	0.201	0.016
NM_0010	chr11	58439120	58440173	-	1051	1	OR5B12|C	0	0.013
NM_0007	chr1	1.67E+08	1.68E+08	-	1668	1	CD247|CD	0.139	0.205
NR_02971	chr8	22244962	22245043	-	81	1	MIR320A|	0	0
NM_0011	chr4	1.22E+08	1.22E+08	-	1584	1	ANXA5|AN	65.249	54.892
NM_0011	chr13	28399044	28495128	-	1897	1	FLT1|FLT|	0	0.007
NM_0012	chr8	1.41E+08	1.41E+08	-	7223	1	SLC45A4|-	3.04	3.753
NM_1525	chr4	1.59E+08	1.59E+08	-	810	1	C4orf45|-|	0.032	0.406

NM_0010:	chr8	11982321	11984590	+	231	1	DEFB135|I	0.391	0
NM_0013:	chr10	1.14E+08	1.15E+08	-	6624	1	ABLIM1|A	21.843	22.756
NM_0013	chr14	41606856	41904551	+	4256	1	LRFN5|C1	3.587	4.528
NM_1706	chr7	1.49E+08	1.49E+08	+	5421	1	ZNF398|P!	6.567	9.541
NM_0011	chr7	35231500	35254100	-	1366	1	TBX20|ASI	0	0
NR_02951	chr4	8005301	8005381	-	80	1	MIR95|MI	0	0.171
NR_00002	chr11	75404421	75404566	+	145	1	SNORD15E	0.8	1.039
NM_0071	chr19	51571237	51589738	+	3819	1	ZNF175|O	2.806	3.92
NR_00277	chr11	61615036	61639449	+	3520	1	RPLP0P2|I	0.172	0.167
NR_02776	chrX	40831217	40833196	-	1979	1	LOC10013	0.065	0.187
NR_13016	chr21	44550357	44551505	-	442	1	KRTAP10-2	0	0
NR_11060	chr20	7146467	7254202	-	866	1	LINC01428	0	0
NM_0010	chr1	1.5E+08	1.5E+08	-	1919	1	APH1A|65	83.623	83.503
NM_0132	chr8	66775181	66862022	+	4173	1	SGK3|CISK	0.08	0.276
NM_0176	chr5	1.03E+08	1.03E+08	-	3539	1	GIN1|GIN-	1.166	1.211
NM_0012	chr2	2.03E+08	2.03E+08	+	6103	1	ABI2|ABI-2	32.247	33.294
NR_04672	chr3	1.89E+08	1.89E+08	-	476	1	TPRG1-AS2	0	0.029
NM_1384	chr8	1.45E+08	1.45E+08	+	1594	1	MFSD3|-|	17.148	16.421
NR_02699	chr7	39733568	39794623	+	4632	1	LINC00265	2.461	1.907
NR_04499	chr13	1.14E+08	1.14E+08	+	890	1	GAS6-AS1|	6.693	6.71
NM_1991	chr12	52948871	52952901	+	1411	1	KRT18|CK-	4.249	0.301
NM_0013	chr21	37073184	37203118	+	6919	1	TTC3|DCR	42.788	61.366
NM_0010	chr7	1.11E+08	1.11E+08	+	3615	1	LRRN3|FIG	14.916	8.957
NR_00334	chr15	25215541	25215622	+	81	1	SNORD115	0	0
NR_02331	chr15	72751367	72783785	-	2268	1	ADPGK|26	17.157	16.856
NR_03837	chr1	33868956	33885458	+	1356	1	CSMD2-AS	0	0
NM_0011	chr2	1.67E+08	1.67E+08	+	2882	1	XIRP2|CM	0.027	0.01
NM_0250	chr11	1.25E+08	1.25E+08	+	3877	1	CCDC15|-|	0.861	0.629
NR_11078	chr1	2.01E+08	2.01E+08	+	733	1	LOC10192	0.088	0.112
NM_0059	chr6	1.37E+08	1.37E+08	-	5163	1	MAP3K5|A	0.235	0.157
NM_0013	chr11	60724009	60744213	+	2092	1	MS4A18|-	0.018	0.013
NM_0011	chr11	76657524	76670747	-	4202	1	LRRC32|D	0.064	0.068
NM_0072	chr6	1.08E+08	1.08E+08	-	6477	1	SEC63|DN	5.568	8.772
NR_12649	chr14	49991007	49993134	-	1295	1	LINC01588	0.08	0.021
NR_02771	chr2	91941133	91942470	+	1337	1	ACTR3BP2	0	0
NM_0027	chr4	56393363	56435636	-	3689	1	PPAT|ATA	3.418	3.464
NM_0010	chr2	1.65E+08	1.65E+08	-	8948	1	SCN3A|NA	7.931	5.69
NM_0011	chr16	31060843	31065088	-	2391	1	ZNF668|-|	9.512	8.329
NR_02731	chr2	1.31E+08	1.31E+08	-	2209	2	TISP43|-|-	0	0
NM_0011	chr1	1.18E+08	1.18E+08	-	3027	1	GDAP2|M	3.339	3.72
NM_0010	chr17	5282263	5386339	+	5811	1	RABEP1|R	19.523	21.612
NM_0240	chr7	1E+08	1E+08	+	1561	1	PVRIG|C7c	4.279	3.764
NR_03746	chr3	16933196	16933260	+	64	1	MIR3714|	0	0
NM_0012	chr4	9220152	9221744	+	1591	2	USP17L21	0	0
NM_0012	chr9	1.25E+08	1.25E+08	+	1280	1	WDR38|-|	0.373	0.407
NM_0148	chr2	68774801	68826825	+	2916	1	ARHGAP25	0.265	0.075
NM_0013	chr22	40370562	40410289	+	2772	1	SGSM3|CI	24.201	28.743
NM_1333	chr5	69369812	69414801	+	2801	2	RAD17|CC	0.244	0.235
NR_03619	chr14	26908642	26908725	+	83	1	MIR4307|	0	0
NR_12166	chr3	73521322	73569774	+	900	1	LOC10192	0	0.015

NR_11062	chr1	59772795	59788829	+	525	1	LOC10192	0.147	0.026
NM_0164	chr20	35772001	35950366	+	5869	1	PHF20|C2	10.912	13.436
NR_13825	chr17_GL	1141731	1168475	+	2356.3	3	CRHR1-IT1	0.022	0.012
NR_03967	chr6	90312742	90312833	+	91	1	MIR4464|	0	0
NM_0013	chr3	43286512	43351142	+	4451	1	SNRK|HSN	6.526	8.616
NM_1986	chr21	44573729	44575371	+	1640	1	KRTAP10-4	0	0
NR_03960	chr1	98045243	98045351	-	108	1	MIR2682|	0	0.38
NM_0013	chr16	46702282	46763246	-	7165	1	MYLK3|M	0.032	0.336
NM_0328	chr19	32878998	32972029	-	2650	1	CEP89|CC	7.366	6.094
NM_1390	chr12	1.12E+08	1.12E+08	+	2275	1	MAPKAPK	23.208	21.903
NM_1749	chr11	1.18E+08	1.18E+08	-	4572	1	SCN4B|AT	0.417	2.223
NM_0021	chr8	39913809	39928791	+	1920	1	IDO1|IDO|	0.007	0.029
NR_12602	chr7	81576386	81626216	-	4354	1	LOC10012	0.003	0
NM_0013	chr9	1.28E+08	1.28E+08	-	2244	1	AK1|HTL-S	53.798	42.403
NM_0177	chr18	62523425	62578585	+	5527	1	ZCCHC2|C	1.943	2.213
NR_03376	chr6	52739463	52745159	-	5696	1	GSTA7P|G	0	0
NM_0219	chr11	207511	215175	+	3771	1	RIC8A|RIC	40.328	41.058
NM_0029	chr1	1.53E+08	1.53E+08	-	434	1	S100A7|P	0	0
NR_03656	chr11	1.05E+08	1.05E+08	-	906	1	CASP5|ICE	0	0.015
NM_0012	chr1	1.52E+08	1.52E+08	-	5581	1	CELF3|BR	94.977	88.509
NM_0121	chr11	33542331	33674100	+	11598	1	KIAA1549	5.316	6.022
NR_03371	chrX	18239313	18354724	-	3123	1	SCML2|-|-	4.12	3.561
NR_00257	chr18	49491664	49491729	-	65	1	SNORD58	31.936	47.413
NR_00000	chr17	28723681	28723754	+	73	1	SNORD4B|	0	1.501
NM_0052	chr8	6870576	6878007	-	458	1	DEFB1|BD	0.056	0
NM_0154	chr9	1.37E+08	1.37E+08	+	2558	1	NELFB|CO	37.889	36.861
NM_0004	chr21	36750888	36990245	-	6005	1	HLCS|HCS	3.715	3.636
NM_0328	chr1	36393420	36397959	-	923	1	LSM10|M	42.41	44.415
NM_0011	chr17	34927859	34961509	-	1748	1	CCT6B|CC	1.711	1.677
NM_0012	chr6	35259714	35295987	+	2496	1	ZNF76|D6	18.736	14.943
NM_0246	chr9	98807699	98850081	+	2723	1	GALNT12|	3.381	0.724
NR_02714	chr17	73749270	73828537	-	1545	1	LINC0046	0	0.009
NM_0012	chr12	1.01E+08	1.01E+08	+	2540.5	2	ANO4|TM	1.634	0.652
NM_0010	chr1	1.53E+08	1.53E+08	-	671	1	SPRR2B|-|	0	0
NR_12161	chr6	71421027	71458872	+	765	1	LINC0162	0	0
NM_0058	chr3	40309682	40312426	+	1008	1	EIF1B|GC2	103.122	120.093
NM_1452	chr1	2.48E+08	2.48E+08	+	3856	1	GCSAML|	0	0
NM_1307	chr11	64934471	64935888	-	742	1	GPHA2|A2	0.052	0
NM_0011	chr3	48372219	48394725	+	1461	1	FBXW12|F	0.124	0.525
NM_1816	chr21	30480045	30480317	-	271	1	KRTAP19-1	0	0
NM_0011	chr9	121038	179075	-	1650	1	CBWD1|C	2.024	2.308
NM_0010	chr3	9933841	9945413	+	2183	1	CRELD1|A	23.3	20.969
NM_0013	chr11	74696428	74711367	-	1502	1	CHRDL2|B	0.052	0.328
NM_0147	chr1	1.57E+08	1.57E+08	-	6727	1	ARHGEF11	15.734	18.622
NR_12536	chr2	56173534	56185770	-	2860	1	LOC10012	0.023	0
NM_0049	chr12	21764955	21774821	-	2384	1	KCNJ8|KIR	1.628	3.487
NM_0178	chr1	26787963	26798403	+	2398	1	PIGV|GPI-	5.898	6.563
NM_0331	chr12	47972965	48004502	-	4825	1	COL2A1|A	5.975	4.369
NM_0006	chr17	28367278	28370352	-	1667	1	VTN|V75|	8.284	1.117
NM_0013	chr18	35468327	35497649	-	844	1	INO80C|C	9.502	10.013

NM_0314	chr6	7389829	7418037	+	2458	1	RIOK1|AD	6.127	7.161
NM_0010	chr8	1.44E+08	1.44E+08	+	1847	1	GRINA|HN	132.767	119.862
NM_0012	chr5	1.72E+08	1.72E+08	+	514	1	SMIM23|	0	0
NM_0015	chr14	61695401	61748259	+	4042	1	HIF1A|HIF	21.401	27.502
NR_10236	chr17	40027543	40027645	-	102	1	SNORD12	0.253	0.403
NR_12576	chr13	37481465	37484784	-	524	1	LINC0104	0	0
NM_0068	chr7	1E+08	1E+08	+	1413	1	COPS6|CS	93.475	87.61
NR_10418	chr1	1.45E+08	1.45E+08	+	2500	2	SRGAP2-A	0	0.005
NM_1985	chr3	49268597	49277075	-	3585	1	C3orf62|-|	4.082	4.229
NM_0051	chr12	39551220	39620041	-	5329	1	ABCD2|AB	0.244	1.254
NM_0053	chr12	57768568	57772131	-	1299	1	METTL1|C	3.782	3.353
NM_0062	chr6	1.32E+08	1.32E+08	+	7415	1	ENPP1|AR	0.096	0.102
NM_0012	chr3	46201709	46208341	-	2672	1	CCR1|CD1	0.043	0.01
NR_13498	chr20	44931562	44939317	+	2217	1	PABPC1L|	0.948	2.088
NM_0064	chr7	99325873	99366262	+	1595	1	ARPC1A|A	98.12	106.854
NR_03384	chr10	1.26E+08	1.26E+08	+	3144	1	LOC28303	0.554	0.597
NR_03167	chr16	11306440	11306527	-	87	1	MIR548H2	0	0
NM_0067	chr4	1.5E+08	1.51E+08	-	9839	1	LRBA|BGL	3.238	3.007
NM_0072	chr5	443219	467294	+	2747	1	EXOC3|SE	18.455	22.931
NM_1830	chr10	30611779	30629718	-	767	1	LYZL2|LYZ	0	0
NM_0163	chr19	58182989	58213562	+	2572	1	ZNF274|H	18.303	22.196
NM_0011	chr10	5393098	5404830	-	1672	1	TUBAL3|-|	0.062	0.016
NM_0011	chr16	3242028	3256627	-	3030	1	MEFV|FM	0.034	0.041
NM_0010	chr17	81244811	81295339	-	4505	1	SLC38A10|	18.411	15.439
NM_0010	chr17	42017576	42025638	+	2380	1	NKIRAS2|	87.459	81.929
NM_0046	chr15	65869459	65891991	+	4922	1	RAB11A|Y	38.652	46.454
NM_0011	chr1	1.56E+08	1.56E+08	-	2542	1	YY1AP1|H	29.074	42.25
NM_0010	chr14	74084953	74200414	+	1790	1	LIN52|C14	14.089	14.08
NM_1458	chr19	51029092	51034892	-	807	1	KLK12|KL	0.032	0
NM_0068	chr2	1.38E+08	1.38E+08	+	3359	1	HNMT|H	0.131	1.342
NM_0034	chr19	43912624	43935259	-	3891	1	ZNF45|KO	5.61	5.826
NM_0065	chr1	86423883	86456557	+	4211	1	CLCA2|CA	0	0
NR_10697	chr2	1.41E+08	1.41E+08	-	59	1	MIR7157|	0	0
NM_0156	chr2	2.15E+08	2.15E+08	-	8038	1	ABCA12|A	0.298	0.441
NM_0011	chr2	75492318	75560966	-	1849	1	EVA1A|FA	0.579	0.133
NR_02412	chr16	526847	527407	-	560	2	LINC0023	0	0
NM_0011	chr1	1.51E+08	1.51E+08	-	3921	1	PI4KB|NP	32.061	39.879
NM_0053	chr5	1.31E+08	1.31E+08	-	683	1	HINT1|HI	403.851	356.122
NM_0201	chr19	5785142	5791238	-	2096	1	DUS3L|DU	8.348	7.992
NR_04611	chr8	92712962	92786060	-	3363	1	FLJ46284|	0.004	0
NM_0026	chr12	53295451	53299451	+	680	1	PFDN5|M	403.642	340.149
NM_0249	chr12	49346917	49353684	+	3945	1	DNAJC22|	6.88	0.746
NM_0161	chr10	89701590	89774943	+	6295	1	KIF20B|CT	0.229	0.531
NR_03657	chr9	32972606	33001641	-	1877	1	APTX|AOA	18.403	18.513
NM_0011	chr6	49692358	49713590	-	1339	1	CRISP2|CR	0	0
NM_0012	chr6	46549580	46652830	-	2256	1	CYP39A1|	0.749	0.759
NM_0161	chr11	95832880	95924207	-	4578	1	MTMR2|C	10.576	8.183
NM_0010	chr2	55865962	55924163	-	3040	1	EFEMP1|	0.331	0.347
NM_0068	chr14	75131468	75176646	-	4137	1	TMED10|	40.117	42.399
NM_0010	chr9	1.27E+08	1.27E+08	+	6559	1	ZBTB34|Z	5.524	5.789

NM_0012{	chr9	93058688	93085136	+	886	1	SUSD3|-|-	0.029	0
NM_0012!	chr19	6772668	6857366	+	2843	1	VAV1|VAV	0	0.198
NM_0325(	chr1	1.53E+08	1.53E+08	-	606	1	LCE3D|LEF	0	0
NM_0330!	chr17	41117181	41118354	-	1168	3	KRTAP4-11	0	0
NM_0033!	chr2	27307400	27308445	-	791	1	UCN|UI|U	3.179	4.779
NM_0011!	chr19	51388289	51390574	-	1314	1	C19orf84|	0.039	0.177
NM_0133(	chr9	1.37E+08	1.37E+08	-	2685	1	ANAPC2|A	14.147	14.671
NR_10270	chr11	27617626	27634627	-	958	1	LINC00678	0	0
NR_03780	chr2	2.39E+08	2.39E+08	-	2417	1	LINC01107	0.011	0.023
NM_0013:	chr15	76994680	77037475	+	1837	1	PSTPIP1|C	0.316	0.164
NM_0176!	chrX	1.1E+08	1.1E+08	+	4971	1	TMEM164	15.607	13.901
NM_1749!	chr7	1.39E+08	1.39E+08	-	1407	1	SVOPL|-|-	0.055	0.097
NR_10446	chr6	4079206	4130765	+	2408	1	C6orf201|	2.163	3.185
NR_10683	chr17	42708084	42708151	-	67	1	MIR6780A	4.234	3.475
NM_0250	chr22	38084889	38110669	-	2129	1	BAIAP2L2|	0.642	0.592
NR_13278	chr2	1.97E+08	1.97E+08	-	115	2	SNORA105	11.997	10.481
NR_10447	chr11	72002864	72080474	-	3835	1	NUMA1|N	80.377	73.946
NR_02734	chr12	1.17E+08	1.17E+08	+	432	1	LINC00173	0.895	1.237
NM_0012{	chr22	38290691	38398522	-	2980	1	LOC40092	176.113	197.154
NR_03830	chr4	78645994	78684501	+	4117	1	LINC01094	0.034	0.077
NM_0010	chr1	1.53E+08	1.53E+08	-	475	1	S100A7L2|	0	0
NM_0062{	chr3	1.42E+08	1.42E+08	-	9421	1	TFDP2|DP	9.141	11.259
NM_0011	chr7	1.28E+08	1.28E+08	-	2291	1	IMPDH1|II	14.824	13.476
NR_04576	chr11	1.29E+08	1.29E+08	-	3554	1	C11orf45|	0.054	0.042
NR_04663	chr5	41870030	41872236	+	1723	1	OXCT1-AS1	0.898	0.819
NM_1530	chr9	1.15E+08	1.15E+08	+	4345	1	TMEM268	5.083	6.882
NR_03970	chr11	61508596	61508657	+	61	1	MIR4488|	0	0
NM_0010:	chr2	1.59E+08	1.59E+08	+	537	1	DAPL1|-|-	18.56	9.488
NM_0059:	chr5	56815073	56896151	+	7489	1	MAP3K1|N	1.896	1.256
NR_11938	chr16	19062754	19067691	-	485	1	LOC10272	0.479	0.198
NM_0012!	chr6	1.3E+08	1.3E+08	+	2899	1	TMEM200	7.356	2.509
NR_03884	chr1	1.6E+08	1.6E+08	+	1135	1	LINC01133	0	0
NR_10377	chr16_KI2	744304	745837	-	1073	5	LOC10028	0.493	1.034
NM_1387!	chr22	37830855	37844346	-	2089	1	ANKRD54|	30.262	31.282
NM_0004(	chr3	1.84E+08	1.84E+08	-	1911	1	THPO|MG	0.594	0.38
NM_0052	chr7	1.01E+08	1.01E+08	+	1652	1	GNB2|-|-	259.883	217.967
NR_10240	chr1	1.2E+08	1.2E+08	+	4339	1	NBPF8|NB	0.597	0.663
NM_0183!	chr4	1.13E+08	1.13E+08	-	6710	1	ZGRF1|C4	0.296	0.837
NM_0041	chr9	4490427	4587469	+	3725	1	SLC1A1|D	4.14	4.24
NM_0012	chr11	1.11E+08	1.11E+08	+	1083	1	COLCA2|C	0.5	0.304
NM_0011(	chr5	1.51E+08	1.51E+08	+	4604	1	SYNPO|-|-	1.229	3.85
NR_10371	chrX	69179557	69209924	+	2168	1	LINC00269	0.077	0.013
NM_1984	chr17	44353733	44363867	-	2624	1	FAM171A2	54.33	63.437
NM_0070(	chr16	56429136	56451349	-	4398	1	NUDT21|C	25.283	26.634
NM_0012!	chr19	36334453	36379203	-	7489	1	ZFP14|ZNI	2.717	3.095
NM_0165(	chr17	50367867	50373201	-	697	1	MRPL27|L	59.898	45.336
NM_0017{	chr19	14381144	14408725	+	3193	1	ADGRE5|C	0.485	0.069
NM_0142!	chr19	17795110	17813576	+	2715	1	B3GNT3|B	0.038	0.03
NR_00297	chr20	38448083	38448218	+	135	1	SNORA71E	0.573	1.928
NM_0043!	chr3	42872192	42876141	-	3947	1	CYP8B1|CI	0.01	0

NM_0012!	chr5	33951406	33984675	-	739	2	SLC45A2|1	0.209	0.111
NR_11055	chr14	77041049	77069503	+	2935	1	LOC28357	0.083	0.107
NM_0248(	chr18	80034346	80050652	+	5670	1	RBFA|C18	5.182	5.066
NR_13244	chr6	1.47E+08	1.47E+08	+	450	1	LUADT1|-|	0	0
NR_07314	chr11	1.01E+08	1.01E+08	+	1218	1	PGR-AS1|/	0	0
NM_0003!	chr21	6560691	6564494	+	1139	2	CRYAA|CR	0	0
NR_12056	chr11	47270657	47272075	-	498	1	LOC10192	0.052	0.083
NM_0046!	chrX	47232915	47248328	+	3267	1	USP11|UH	172.88	213.974
NM_1720(	chr20	45702016	45708817	+	1366	1	WFDC13|(	0	0.02
NM_0020:	chr4	1.43E+08	1.43E+08	+	7808	1	GAB1|-|-|	2.091	4.019
NM_0009:	chr8	1.45E+08	1.45E+08	-	897	1	RPL8|L8|-	1009.846	821.363
NM_0221(	chr19	56439324	56477401	-	3853	1	ZNF667|N	3.169	3.633
NM_0013(	chr2	1.58E+08	1.59E+08	+	3151	1	PKP4|p00	9.023	9.581
NM_0141:	chr8	54135221	54148514	+	1742	1	MRPL15|H	33.914	30.327
NM_1779!	chr18	31376777	31414908	+	4553	1	DSG4|CDG	0	0.003
NM_0529:	chrX	1.08E+08	1.08E+08	+	2301	1	ATG4A|AP	3.62	4.459
NM_0012(	chr7	43866558	43906632	-	829	1	URGCP-MI	54.84	49.369
NM_0012:	chr8	73780097	73878910	-	943	1	UBE2W|U	4.922	4.663
NR_02795	chr3	1.57E+08	1.57E+08	-	2542	1	TIPARP-AS	0.162	0.286
NM_1733	chr10	73168119	73242181	+	3116	1	FAM149B1	6.476	6.58
NR_00155	chr20	22560554	22578642	-	4907	1	LINC00261	0	0
NM_0208	chr10	30012800	30059559	-	9294	1	KIAA1462|	0.862	0.556
NM_0025!	chr1	1.65E+08	1.65E+08	+	6892	1	PBX1|-|-|:	72.908	51.855
NR_11005	chr14	44898908	44911917	-	411	1	LOC10192	0.063	0.067
NM_0011(	chr7	50096086	50159256	+	1393	1	C7orf72|-|	0	0.01
NR_03759	chr4	1.19E+08	1.19E+08	-	4815	1	LINC01061	0.479	0.412
NM_0186!	chr15	72654697	72667397	+	3158	1	GOLGA6B|	0	0.004
NM_0030	chr13	29509414	29595688	-	7328	1	SLC7A1|A	15.135	17.772
NM_0045:	chr7	56011051	56051575	-	2108	1	PSPH|PSP	15.64	15.51
NM_0207!	chr1	11479238	11537583	+	5191	1	DISP3|PTC	31.182	34.32
NM_0010(	chr6	7107853	7251980	+	8554	1	RREB1|FIN	0.77	0.527
NM_0072:	chr1	1.56E+08	1.56E+08	+	1115	1	PMF1|-|-|	38.958	32.959
NM_0048:	chr14	1.04E+08	1.04E+08	-	4864	1	BAG5|BAG	9.983	10.478
NR_00438	chr20	8831185	8831393	+	208	1	RNU105B|	0	0
NR_11025	chr2	7886767	7899655	-	731	1	LOC10192	0	0.187
NM_0011!	chr1	91680343	91906002	-	6280	1	TGFBR3|B	0.232	0.035
NR_03960	chr4	5923275	5923328	-	53	1	MIR378D1	0	0
NR_03027	chr1	3560696	3560790	-	94	1	MIR551A|	0	0
NM_0162:	chr5	1.78E+08	1.78E+08	-	2445	1	DDX41|AB	33.28	35.343
NR_12539	chr8	96951864	97446497	-	2215	1	LOC10192	0.035	0.006
NR_02709	chr4	68060610	68063297	-	2626	1	SYT14P1|(	0	0.026
NM_0010:	chr19	1009651	1021142	-	2695	1	TMEM259	53.325	57.156
NR_13511	chr12	22046174	22065672	+	1617	1	CMAS|CSS	32.884	26.784
NM_0011:	chr1	2.31E+08	2.31E+08	-	3104	1	TTC13|-|-	13.442	17.563
NR_00329	chr15	25182385	25182466	+	81	1	SNORD115	0	0
NM_0013:	chr11	62806860	62832091	-	1669	1	STX5|SED5	37.151	42.724
NR_02724	chr21	42102134	42108534	-	2139	1	UMODL1-/	0.012	0.006
NM_1536:	chr20	22581004	22585463	-	2405	1	FOXA2|HN	0	0
NR_12167	chr4	1.66E+08	1.66E+08	+	2157	1	LINC0117!	0	0.102
NR_12633	chr3	1.07E+08	1.07E+08	-	468	1	LOC10192!	0	0.322

NR_11084	chr6	1.23E+08	1.23E+08	+	2406	1	HRAT13|-|	0	0
NR_03742	chr9	92292458	92292547	-	89	1	MIR3651|r	0.145	0
NM_0012٤	chr15	28698696	28712421	-	5234.7	3	GOLGA8M	0	0
NM_1730(	chr1	24154157	24187275	-	4424	1	IFNLR1|CR	0.125	0.118
NR_11083	chr7	41101604	41133501	+	1208	1	LINC0144S	0	0.023
NR_12586	chr6	1.47E+08	1.47E+08	-	2187	1	LOC10192.	0	0
NR_11040	chrX	76657798	77014532	-	981	1	MIR325HG	5.967	5.04
NR_11056	chr5	99533990	99577954	+	1029	1	LINC02113	0.025	0
NR_02461	chr3	1.23E+08	1.23E+08	+	5903	1	LINC02035	1.99	2.764
NM_0012.	chr9	1.36E+08	1.36E+08	+	4663	1	KCNT1|EIE	0.08	0.311
NM_0055٤	chr19	13099028	13103160	-	1782	1	LYL1|bHLH	0.087	0.085
NM_0013:	chr19	57279967	57293568	+	3150	1	ZNF460|H.	1.052	1.3
NM_0011،	chr6	17281578	17293868	+	2458	1	RBM24|RN	3.588	2.658
NR_02968	chr5	1.49E+08	1.49E+08	+	105	1	MIR143|M	0	0
NM_0005:	chr3	1.36E+08	1.36E+08	+	1799	1	PCCB|-|-|:	23.2	29.092
NM_0181.	chr8	65602456	65634217	-	3033	1	ARMC1|Ar	8.498	11.258
NM_0012،	chr19	7728957	7732171	-	1313	1	CLEC4G|D	0.255	0.104
NM_0012٤	chr9	87497228	87708634	+	5718	1	DAPK1|DA	18.068	27.59
NM_0012(	chr3	33496246	33659441	-	6687	1	CLASP2|-|	42.093	51.091
NR_03035	chr14	31014646	31014742	-	96	1	MIR624|M	0	0
NR_03826	chr3	64684870	65011471	+	2225	1	ADAMTS9-	0.11	0.209
NR_02808	chrY	17500958	17516742	+	2033	2	FAM41AY2	0	0
NM_0018:	chr19	41618971	41627074	-	1196	2	CEACAM4	0	0
NM_0017.	chr4	15778265	15853243	+	5684	1	CD38|ADP	0.102	0.764
NM_0013(	chr19	38619072	38636959	+	873	1	EIF3K|ARG	191.866	145.129
NM_0013.	chr16	75218986	75224927	+	733	1	CTRB1|CTI	0.264	0.206
NM_0011،	chr11	561450	564025	+	1447	2	RASSF7|C1	0.196	0.189
NM_0011،	chr22_KI2	267292	268218	+	438	2	LOC39132.	1.089	0.344
NM_0013:	chr19	57921884	57935387	-	3582	1	ZNF418|-|	3.009	4.352
NM_0011S	chr8	66946501	66962590	-	2444	1	TCF24|TCF	0.037	0.011
NM_0013(	chr17	7440742	7444932	+	2359	1	FGF11|FGI	39.014	33.914
NM_0013:	chr11	1.12E+08	1.12E+08	-	550	1	CRYAB|CM	18.215	4.383
NR_03744	chr16	14907717	14907781	+	64	8	MIR3670-1	0	0
NM_0011:	chr6	1.27E+08	1.27E+08	-	2109	1	ECHDC1|H	11.634	9.846
NR_12035	chr15	71166812	71189059	-	701	1	THSD4-AS1	2.796	1.993
NM_0186٤	chr15	80779343	80951662	+	7081	1	CEMIP|CC	0.262	1.335
NR_11059	chr2_KI27(	9378	15971	+	2164	3	LINC01238	0	0
NR_03159	chr7	21471058	21471146	+	88	1	MIR1183|I	0	0
NR_03384	chr3	1.86E+08	1.86E+08	-	2287	1	LINC02052	0	0.012
NR_13191	chr2	1.92E+08	1.92E+08	+	1890	1	LOC10574	0.027	0
NM_0031،	chr1	1.56E+08	1.56E+08	-	1117	1	SSR2|HSD.	244.861	278.661
NM_0036٤	chr21	43789537	43804102	+	1792	1	RRP1|D21	18.038	17.312
NM_0013(	chr11	2887345	2903945	-	1899.5	2	SLC22A18/	0.034	0
NR_13632	chr2	89625445	89637907	-	571	1	LOC10192	0	0
NM_0069(	chr10	45000825	45005329	+	2200	1	ZNF22|HK	11.598	14.326
NR_03962	chr1	1.17E+08	1.17E+08	-	87	1	MIR548AC	0	0
NM_0011(	chr8	33498509	33513185	-	2384	1	TTI2|C8or	9.594	12.134
NM_0132،	chr11	6999318	7020355	-	2663	1	ZNF214|B/	1.622	1.054
NM_0037.	chr19	3178738	3180332	+	1592	1	S1PR4|ED(	0.105	0.224
NM_1984!	chr7	1.5E+08	1.5E+08	+	15475	1	SSPO|-|-|.	0.619	0.619

NM_0220;	chr3	94063011	94126786	+	1427	1	NSUN3|M	4.572	3.964
NR_13210	chr6	84421031	84474929	-	568	1	LINC01611	0	0
NM_0011:	chr6	1.35E+08	1.35E+08	-	5472	1	AHI1|AHI-	1.423	1.219
NR_00366	chr8_KI27(	3981	11104	+	262	3	DEFB109P	0	0
NM_0327!	chrX	16719584	16762684	+	3514	1	SYAP1|PR(	8.186	6.716
NR_00230	chr1	1.47E+08	1.47E+08	+	2098	1	PDIA3P1|E	22.087	19.853
NM_0013:	chr1	1.75E+08	1.75E+08	-	4565	1	KIAA0040|	0.031	0.027
NM_1334;	chr4	439984	499171	-	4687	1	ZNF721|-|	1.788	3.741
NM_0331;	chr20	661596	676179	-	3573	1	SCRT2|ZNI	31.792	43.744
NR_03886	chr11	71405746	71423354	-	2159	1	FLJ42102|	0	0.013
NR_03992	chr2	87194786	87194859	+	73	2	MIR4771-1	0	0
NR_02961	chr7	1.3E+08	1.3E+08	-	109	1	MIR183|M	0	0
NM_0067(	chr13	52652691	52688354	+	1736	1	SUGT1|SG	10.517	10.375
NM_0010(	chr11	58030953	58031906	+	952	1	OR6Q1|OF	0	0
NM_0209!	chr1	1.81E+08	1.81E+08	+	4144	1	KIAA1614|	0.98	0.886
NR_00297	chr18	54222284	54222412	-	128	1	SNORA37|	0	0
NM_0011(	chr12	24993431	24997439	-	1503	1	C12orf77|	0	0
NM_0219!	chr13	20138256	20161044	-	5201	1	GJA3|CTR(	0.015	0.016
NR_13428	chr5	1.8E+08	1.8E+08	+	1461	1	LOC10099	3.186	4.059
NR_13277	chr21	41948105	41948282	-	177	1	SNORA91|	0.146	0
NM_0010(	chr8	1.45E+08	1.45E+08	-	1927	1	C8orf82|-|	10.284	8.089
NM_0041;	chr22	21028718	21032558	-	2306	1	SLC7A4|C/	0.123	0.036
NM_0038!	chr19	39863323	39934626	-	12611	1	FCGBP|FC(	0.129	0.159
NR_11077	chr18	10405133	10414370	-	1904	1	LINC01254	0.014	0.029
NM_1721:	chr19	39243606	39245006	-	649	1	IFNL3|IFN-	0	0
NM_0036!	chr11	6794525	6795908	-	1381	1	OR6A2|I7|	0	0
NM_0152:	chr4	36065998	36244357	-	7327	1	ARAP2|CE	0.023	0.346
NM_1387!	chr1	2.02E+08	2.02E+08	-	1786	1	ARL8A|AR	78.112	95.434
NR_13364	chr6_GL0(	1958181	1968296	+	735	8	MDC1-AS1	0	0
NM_0011‹	chr11	1.19E+08	1.19E+08	+	3740	1	ARCN1|CC	45.541	41.065
NR_02847	chrX	1.37E+08	1.37E+08	-	1902	1	RBMX|HN	60.515	50.64
NM_0010(	chr12	1.2E+08	1.2E+08	-	1194	1	RPLP0|L1(	297.468	257.685
NM_0011:	chr1	48222685	48248644	+	3257	1	SLC5A9|S(	0.016	0.038
NM_0331;	chr12	1.1E+08	1.1E+08	+	3891	1	ANKRD13/	17.132	13.919
NR_14446	chr9	61861564	61862615	-	535	2	LOC10537!	0	0
NM_0013;	chr11	1.03E+08	1.03E+08	+	13587	1	DYNC2H1|	0.743	0.921
NM_1816{	chr21	44681576	44682163	-	585	1	KRTAP12-1	0	0
NM_0013(	chr10	1.32E+08	1.32E+08	+	1669	1	LRRC27|-|	21.646	21.937
NR_02698	chr13	76928451	76929090	+	639	1	BTF3P11|E	0.02	0.021
NR_12591	chr4	12223451	12251285	+	636	1	LOC10192!	0	0
NM_0190;	chr15	40929333	40939060	+	3407	1	DLL4|AOS(	0.708	0.856
NM_0013:	chr16	72054592	72061056	+	1264	1	HP|BP|HP	0	0
NR_02778	chr7	1.5E+08	1.5E+08	-	3575	1	ZNF767P|Z	5.774	6.168
NR_13450	chr10	47407165	47414574	+	595	1	LOC10700	0	0
NM_0240{	chr2	2.19E+08	2.19E+08	-	3753	1	ATG9A|AP	25.107	22.62
NR_10999	chr20	25184672	25196892	-	2174	1	LOC10192(	0.012	0.019
NM_0011:	chr5	81301583	81313146	+	1397	1	ZCCHC9|P	5.676	3.981
NM_0178!	chr4	70196527	70204576	+	1275	1	ODAM|AP	0	0
NR_10977	chr8	1.44E+08	1.44E+08	+	1190	1	TONSL-AS1	1.43	0.783
NM_0055:	chr3	1.21E+08	1.21E+08	+	3026	1	GTF2E1|FE	8.074	7.374

NR_12581	chr8	29815004	29828504	-	1864	1	LOC10192	0.069	0.007
NM_0011	chr14	21459020	21476673	-	2846	1	RAB2B|-|-	28.736	30.344
NM_0072	chr12	64404373	64448683	+	3920	1	XPOT|XPO	9.295	14.246
NM_2073	chr11	66539264	66546200	-	1312	1	ZDHHC24|	6.633	6.577
NR_03161	chr10	98395218	98395307	-	89	1	MIR1287|	0	1.077
NR_10501	chr5	87120148	87248018	+	1467	1	LOC10192	0	1.27
NM_0051	chr11	59829273	59845501	-	1502	1	GIF|IF|IFM	0	0.018
NM_0012	chr1	1.96E+08	1.97E+08	-	5872	1	KCNT2|KC	1.243	1.721
NM_0528	chr16	30989256	31010638	-	4662	1	STX1B|GE	23.373	27.55
NR_03613	chr16	1734985	1735066	+	81	1	MIR3177|	0	0.169
NM_0012	chr1	1.62E+08	1.62E+08	-	3764	1	OLFML2B|	1.014	1.044
NR_03981	chr9	35608094	35608159	+	65	1	MIR4667|	2.38	6.322
NR_03331	chr4	70133604	70146704	+	424	1	CSN1S2BP	0	0
NM_0013	chr18	35373294	35377337	-	851	1	ZNF396|Z	1.227	1.03
NR_03891	chr16	5033702	5042971	+	1235	1	NAGPA-AS	0.491	0.943
NR_02669	chr7	73735069	73736000	+	469	1	ABHD11-A	0.22	0.058
NR_03997	chr4	1.85E+08	1.85E+08	-	1526	1	LINC01093	0	0
NM_0010	chr4	51993641	52017620	-	3382	1	LRRC66|-|	0.172	0.62
NM_0309	chr1	16231687	16237164	-	1553	1	RSG1|C1o	4.691	4.066
NM_0010	chr20	44355801	44432845	+	4501	1	HNF4A|FR	0.006	0.003
NR_03988	chr17	15251627	15251696	-	69	1	MIR4731|	0	0
NM_0010	chr6	1.26E+08	1.26E+08	+	1936	1	TRMT11|C	4.788	5.044
NM_0227	chr1	2.46E+08	2.46E+08	-	1449	1	SMYD3|KM	26.792	36.941
NM_0175	chr5	1.78E+08	1.78E+08	+	2594	1	TMED9|GI	53.155	46.429
NR_11041	chrY	22101692	22147484	-	2926	2	LOC10192	0	0
NR_10699	chr2	1.76E+08	1.76E+08	+	58	1	MIR7704|	0	0
NM_0012	chr17	16381097	16382745	+	859	1	UBB|HEL-S	632.561	428.243
NM_0013	chr18	59139857	59158837	+	758	1	SEC11C|SE	117.623	170.49
NM_0012	chr3	42936342	42942791	+	684	1	KRBOX1|-	0	0
NM_0059	chr11	1012824	1036706	-	7967	3	MUC6|MU	0.005	0
NR_10809	chr7	56482105	56483319	-	695	1	LOC10024	0	0
NR_04988	chr19	12920320	12920404	+	84	1	MIR5695|	0	0.815
NM_0181	chr19	49118397	49119140	-	741	1	C19orf73|	4.785	5.767
NR_04634	chr2	85602842	85649284	+	2153	1	USP39|65	47.58	57.32
NM_0010	chr2	38814	46588	-	3959	1	FAM110C|	0.221	0.903
NM_1815	chr17	40776808	40782534	-	1602	1	KRT27|K2	0	0
NM_0012	chr18	24014420	24020298	+	1054	1	TTC39C|C	3.951	2.612
NR_03883	chr18	5748819	5795901	+	1685	1	MIR3976H	0	0
NR_13449	chr10	30723219	30723740	+	438	1	LOC10537	0	0
NM_0314	chr6	79631105	79703670	+	4836	1	SH3BGRL2	8.633	5.058
NM_0201	chr13	95801581	96053482	-	4889	1	UGGT2|HU	1.176	1.594
NM_0011	chrX	1.12E+08	1.12E+08	+	2749	1	TRPC5OS|	0.019	0.02
NM_0046	chr3	9731750	9748015	+	4694	1	BRPF1|BR	10.729	11.135
NM_0013	chr2	85394748	85410309	-	1221	1	CAPG|AFC	0.412	0.482
NR_03165	chr1	20988314	20988432	-	118	1	MIR1256|	0	0
NR_12058	chr11	1.29E+08	1.29E+08	+	1377	1	LOC10192	0	0
NM_0012	chr7	6031376	6036552	+	1418	1	ANKRD61|	0.5	0.695
NM_0013	chr13	94437304	94549413	-	4093	1	DCT|TRP-2	0.835	33.103
NR_03993	chr2	1.69E+08	1.69E+08	+	75	1	MIR4774|	0	0
NM_2058	chr15	48120972	48142392	+	1606	1	SLC24A5|J	3.388	6.013

NM_0028(	chr1	51907956	51990764	-	12838	1	RAB3B|-|-	37.275	9.472
NM_0147	chr1	40620680	40665652	-	7219	1	RIMS3|NIM	55.559	56.268
NM_0011(	chr2	54330934	54361577	+	1914	1	C2orf73|-|	0.027	0.114
NM_0011!	chr7	40134976	40860767	+	1491	1	SUGCT|C7	1.401	2.278
NM_0013(	chr12	1.23E+08	1.23E+08	+	1788	1	OGFOD2|-	6.98	6.45
NM_0252!	chr6_GL00	3217228	3239935	+	2209.9	8	MSH5|G7|	0	0
NR_12049	chrX	72938163	72938958	+	462	2	FAM236B|	0	0
NM_0010:	chr12	48183583	48185926	+	2341	1	CCDC184|	94.143	75.524
NM_0012(	chr5	10441862	10465026	+	947	1	ROPN1L|A	2.315	1.07
NR_03751	chrX	74218549	74218618	+	69	1	MIR374C|	0	0
NM_1768	chr7	1.43E+08	1.43E+08	+	970	1	TAS2R40|(	0	0
NR_04747	chr5	1.32E+08	1.32E+08	+	833	1	P4HA2-AS:	1.935	0.954
NR_13234	chr9	96106661	96116411	-	2659	1	LOC15843	0	0
NR_10985	chr7	1.51E+08	1.51E+08	+	745	1	WDR86-AS	0.294	0.257
NM_0333!	chr2	2.01E+08	2.01E+08	+	2642	1	CASP8|ALI	0.244	0.207
NM_0227	chr6	79914812	79947598	-	3052	1	ELOVL4|AI	10.304	7.037
NR_02991	chr7	27169480	27169563	-	83	1	MIR196B|	0	0
NR_12634	chr8	1.23E+08	1.23E+08	-	567	1	LINC01151	0.25	0.121
NR_02970	chrX	50003503	50003588	+	85	1	MIR188|M	0	0
NR_03019	chr19	53694393	53694475	+	82	1	MIR526B|	0	0
NR_13176	chr18	44531779	44553903	+	943	1	LINC01601	0	0
NR_10687	chr20	64076955	64077010	-	55	1	MIR6813|	0.469	0
NM_0056	chr5	1.41E+08	1.41E+08	-	2292	1	TAF7|TAF2	139.561	122.717
NM_0201	chr4	1.03E+08	1.03E+08	-	2908	1	BDH2|DHI	4.709	5.04
NR_03387	chr2	2.31E+08	2.31E+08	-	3503	1	SPATA3-AS	0.022	0
NR_14433	chr10	93893970	93909833	+	2179	1	SLC35G1|(	1.651	1.609
NR_03961	chr1	23057858	23057934	-	76	1	MIR4419A	0	0
NM_0163!	chr20	62642445	62672295	+	2762	1	SLCO4A1|(	2.166	1.884
NM_0163(	chr3	1.38E+08	1.38E+08	+	3352	1	CLDN18|SI	0.015	0.041
NR_03977	chr5	1062896	1062974	-	78	2	MIR4635|	0	0
NM_0202	chr17	10697610	10711558	+	1512	1	ADPRM|C:	4.92	6.314
NM_1532	chr5	73173196	73175143	+	1754	1	TMEM174	0	0
NM_0011(	chr9	69427533	69672359	-	5832.5	2	APBA1|D9	2.036	2.574
NR_02426	chr12	8082211	8097777	+	2504	1	NECAP1|E	51.126	65.455
NR_10803	chr10	9275613	9287056	+	711	1	LINC0070!	0	0
NM_0221	chr3	49412206	49416476	+	2142	1	TCTA|-|-|:	15.072	9.784
NM_0036	chr22	37823382	37825495	+	1153	1	GALR3|-|-	0.078	0.048
NM_0010	chr12	51245349	51270210	-	1070	1	SMAGP|h!	1	1.511
NM_0003(	chr12	6867529	6870946	+	1342	1	TPI1|HEL-!	336.864	164.742
NM_0013	chr1	40508719	40516050	+	1703	1	EXO5|C1o	2.582	5.55
NR_00276	chr2	1.93E+08	1.93E+08	+	1587	1	PCGEM1|L	0	0
NM_0012!	chr17	70104845	70135605	+	3934	1	KCNJ16|BI	0.043	0.077
NM_0012	chr9	1.1E+08	1.1E+08	-	802	1	TXN|TRDX	112.038	79.569
NM_0185	chr1	20482391	20486235	-	2347	1	CAMK2N1	22.667	31.275
NM_0012	chr6	1.19E+08	1.19E+08	-	3384	1	FAM184A|	2.667	3.076
NR_13803	chr14	1.03E+08	1.03E+08	-	482	1	LINC00677	0	0
NR_10384	chr19	51949134	51981367	+	723	1	ZNF350-AS	0.749	0.701
NM_0032(	chr3	1.7E+08	1.7E+08	+	6531	1	SEC62|Dtr	8.313	8.332
NM_0529(	chr4	10490214	10684762	-	1656	1	CLNK|MIS	0.023	0.066
NM_0211	chr1	998962	1000172	-	956	1	HES4|bHLI	36.509	31.549

NR_03842	chr1	71046506	71067182	+	1673	1	ZRANB2-A:	3.098	3.152
NR_13430	chr8	1.44E+08	1.44E+08	-	1289	1	LOC10192	0	0
NR_10383	chr8	56374718	56390710	-	521	1	SDR16C6P	0	0
NR_12574	chr7	8262225	8343281	+	1067	1	LOC10050	2.791	0.706
NR_01543	chr1	1399530	1402046	+	1745	1	LOC14841	19.093	18.878
NM_0017	chr9	35609979	35618427	-	1537	1	CD72|CD7	1.686	1.123
NM_0013	chr19	2907108	2918476	+	1898	1	ZNF57|ZN	2.31	3.486
NM_0010	chr16	89644431	89657785	-	2395	1	CHMP1A|	70.486	72.071
NM_1706	chr15	57953424	58014097	-	3194	1	ALDH1A2|	0.214	0.107
NR_02200	chr7	67292436	67302442	-	587	1	PMS2P4|P	5.579	4.48
NR_11078	chr18	76622781	76639011	-	932	1	LINC01927	0.069	0.103
NM_0183	chr5	34915715	34925682	+	1261	1	BRIX1|BRI	10.133	9.385
NM_0140	chr19	49289635	49325217	-	2919	1	SLC6A16|I	2.151	1.314
NR_11079	chr1	2.21E+08	2.21E+08	+	957	1	LINC01352	0	0
NM_0011	chr17	3806751	3846251	-	8169	1	NCBP3|C1	9.18	8.504
NM_1825	chr19	43795927	43820656	-	2466	1	LYPD5|PR	0.141	0.417
NM_0039	chr6	37353972	37394738	+	5621	1	RNF8|hRN	6.81	7.664
NM_0010	chrX	1.21E+08	1.21E+08	-	1288.7	10	CT47A5|C	0	0
NM_1985	chr19	46229752	46231243	+	763	1	IGFL1|APR	0	0
NM_0029	chr17	34255277	34257201	+	742	1	CCL2|GDC	2.016	3.23
NM_0011	chr10	75210154	75231448	+	1416	1	VDAC2|PC	21.999	24.898
NR_07345	chr10	1.26E+08	1.26E+08	-	1164	1	LINC00601	0	0
NM_0183	chr12	95076749	95217464	-	9265	1	FGD6|ZFY	1.872	2.32
NM_2136	chr20	37128821	37179588	-	1892	1	MROH8|C	5.111	3.974
NM_0013	chr6	18128314	18155169	-	3218	1	TPMT|TPM	2.312	2.35
NM_0324	chr1	20633455	20651511	+	2650	1	PINK1|BRF	42.208	56.726
NM_1523	chr1	2.31E+08	2.31E+08	-	1435	1	C1orf131|	5.454	5.975
NR_00329	chr3	1.98E+08	1.98E+08	-	2885	2	ANKRD18D	0	0
NM_0200	chr20	63739858	63744050	+	2269	1	SLC2A4RG	15.485	10.449
NM_0011	chr19	7030583	7033011	+	769	1	MBD3L5|-	0	0
NM_0176	chr2	96816254	96835384	+	4906	1	CNNM3|A	7.059	7.627
NR_02837	chrX	74028136	74293574	-	2349	1	FTX|LINC0	0.977	0.98
NM_0035	chr3	58505136	58537202	-	2292	1	ACOX2|BC	0.174	0.484
NM_0190	chr7	22812633	22822852	-	773	1	TOMM7|T	129.385	98.484
NR_12588	chr4	1.7E+08	1.7E+08	+	1256	1	LINC01612	0	0.087
NM_0010	chr19	37506939	37543337	+	7111	1	ZNF793|-|	1.438	1.872
NR_03983	chr10	35641172	35641252	-	80	1	MIR4683|	2.579	5.992
NR_03034	chr11	61792495	61792561	-	66	1	MIR611|M	0.391	0.83
NR_00333	chr15	25085384	25085477	+	93	1	SNORD116	0.139	0
NR_00291	chr9	1.31E+08	1.31E+08	+	85	2	SNORD62A	1.82	3.706
NM_0010	chr6	41042498	41044337	+	1008	1	TSPO2|BZ	0.179	0.041
NM_0011	chr11	1.03E+08	1.03E+08	-	1891	1	MMP1|CL	0.293	0.116
NR_10989	chr5	1.68E+08	1.68E+08	-	515	1	CTB-178M	1.227	1.223
NM_1528	chr9	1.36E+08	1.36E+08	-	2841	1	C9orf69|-|	14.182	14.218
NM_1723	chr15	36891021	37098361	-	2818	1	MEIS2|Hs	104.621	38.86
NM_0013	chr5	1.28E+08	1.28E+08	+	918	1	CCDC192|	0.084	0.269
NR_02681	chr17	16788743	16790501	+	1758	1	FAM106CF	0	0
NM_0322	chr17	36428612	36439523	-	2062.6	5	TBC1D3F|	0	0
NM_0249	chr16	57152466	57186064	-	2839	1	FAM192A|	52.155	62.276
NM_0044	chr10	1.23E+08	1.23E+08	+	5760	1	DMBT1|GI	0	0.01

NM_0145(	chr11	60890548	60906589	-	2322	1	PRPF19|NI	75.145	75.841
NM_1388(	chr3	58633943	58666834	-	1296	1	FAM3D|EF	0.02	0.116
NM_0013‹	chr5	37379260	37752672	+	2087	1	WDR70|-|	18.003	19.164
NR_03018	chr19	53675711	53675797	+	86	1	MIR520E|I	0	0
NM_0162!	chr2	70258099	70281185	+	5369	1	PCYOX1|P	14.315	12.544
NM_0052!	chr15	40764087	40767713	+	708	1	GCHFR|GF	11.91	5.494
NM_0013(	chr2	2.1E+08	2.1E+08	-	4601	1	KANSL1L|(	1.906	2.888
NM_0004‹	chr1	2.27E+08	2.27E+08	+	2283	1	PSEN2|AD	11.205	8.561
NR_03169	chrX	84225752	84225828	-	76	1	MIR548I4|	0	0
NM_0001:	chr15	48408306	48645788	-	11627	1	FBN1|ACM	1.384	0.663
NM_0013;	chr16	88813734	88856970	-	2228	1	GALNS|GA	13.218	14.299
NM_0010:	chr4	1.68E+08	1.68E+08	-	6741	1	DDX60L|-|	0.025	0.102
NM_0013:	chr9	35605284	35610041	+	2422	1	TESK1|-|-|	30.019	32.733
NM_0012{	chr6	34246380	34249108	-	903	1	C6orf1|LB	36.681	33.825
NM_0144;	chr12	48857133	48865870	-	1658	1	RND1|ARH	17.279	23.726
NR_13078	chr2	1.81E+08	1.81E+08	+	1760	1	LINC01934	0	0.008
NM_0010:	chr6	31528962	31530231	+	705	8	MCCD1|-|	0	0
NM_2136(	chr17	42313325	42388387	-	4789	1	STAT3|AD	27.642	33.855
NR_03827	chr10	52296848	52314128	-	2247	1	PRKG1-AS:	0.138	0.171
NM_0216;	chr3	1.79E+08	1.79E+08	-	6416	1	GNB4|CM	5.5	7.006
NR_00321	chr14	1.01E+08	1.01E+08	+	70	1	SNORD11‹	0	0
NM_1827!	chr6	18387350	18468874	+	5026	1	RNF144B|	0.041	0.03
NM_0010(	chr11	1597177	1598294	-	1115	3	KRTAP5-2|	0	0
NM_0012(	chr12	52022832	52059507	+	2580	1	NR4A1|GF	5.557	11.919
NM_0122{	chr7	1.2E+08	1.21E+08	+	5325	1	KCND2|KV	6.397	2.343
NR_02837	chr10	88991424	88992975	-	1551	1	FAS-AS1|F	0	0
NM_0178‹	chr11	59806135	59810872	-	1176	1	MRPL16|L	20.776	26.02
NR_10693	chr20	10649636	10649695	-	59	1	MIR6870|I	3.715	0.464
NR_12870	chr16	14901508	14901591	+	83	7	MIR3179-‹	0	0
NM_0322!	chr7	1.4E+08	1.4E+08	-	3034	1	SLC37A3|-	17.224	19.512
NM_2033!	chr8	93731501	93740996	-	5098	1	RBM12B|N	5.152	5.801
NM_0011‹	chr19	43596586	43600435	+	1858	1	ZNF576|-|	10.222	7.431
NR_03156	chr18	21683510	21683597	+	87	1	MIR320C1	0	0
NM_0207;	chr18	21741329	21870957	+	9544	1	MIB1|DIP-	12.426	9.737
NR_00002	chr19	49489966	49490047	+	81	1	SNORD32/	6.845	6.764
NM_0221‹	chr10	70254957	70283694	-	1305	1	NPFFR1|G	0.049	0.336
NM_0010;	chr11	67464348	67469277	-	886	1	TMEM134	14.232	12.414
NM_0011‹	chr19	3851813	3869029	-	863	1	ZFR2|KIAA	4.572	4.999
NM_0242!	chr6	34587280	34696850	-	4422	1	C6orf106|	25.425	26.486
NM_0220(	chr17	76389532	76453206	-	5335	1	UBE2O|E2	32.762	39.047
NM_1471!	chr12	50129306	50167533	-	2642	1	CERS5|LAS	32.424	32.158
NM_1986{	chr21	44600597	44602174	+	1575	1	KRTAP10-7	0	0
NM_0012;	chr11	15149102	15247208	+	2701	1	INSC|-|-|1	0.01	0.081
NR_00336	chr15	25106520	25106604	+	84	1	SNORD116	0	0
NM_0221:	chr5	71587288	71658703	+	3654	1	MCCC2|M	3.119	5.99
NR_10419	chr20	14554384	14628908	+	660	1	MACROD2	0	0
NM_0010‹	chr16	50152918	50235308	+	8097	1	PAPD5|TR	1.817	1.737
NR_13094	chr7	73006080	73016375	+	662	2	PMS2P7|P	0	0
NM_0036(	chr1	2.27E+08	2.27E+08	-	10468	1	CDC42BPA	3.844	3.955
NM_0066!	chr7	31687017	31708455	+	1959	1	PPP1R17|(	25.524	8.369

NM_1723	chr5	1.33E+08	1.33E+08	+	565	1	IL4|BCGF-1	0	0
NM_0011	chr12	6650538	6663142	-	1367	1	ING4|my0	80.294	91.35
NM_0011	chr5	1.23E+08	1.23E+08	+	3017	1	PRDM6|KI	1	0.05
NR_12168	chr1	46446673	46449704	-	1126	1	LINC01398	0.046	0.012
NM_0012	chr1	37860697	37931791	-	3927	1	INPP5B|5F	6.251	6.501
NM_0318	chrX	31071241	31072053	-	810	1	FTHL17|CT	0	0
NM_0013	chr2	1.02E+08	1.02E+08	+	4727	1	IL1R1|CD1	0.112	0.113
NM_0011	chrX	13569575	13633575	+	2418	1	EGFL6|MA	0.427	0.153
NM_0013	chr2	53971103	54305298	+	1169	1	ACYP2|AC	7.996	7.054
NR_02990	chr10	86264694	86264788	-	94	1	MIR346|M	0	0
NR_03785	chrX	15345591	15491309	-	2567	1	PIR-FIGF|-	2.2	1.43
NM_0252	chr20	63743668	63805503	-	5226	1	ZBTB46|BT	15.812	15.149
NR_12164	chr1	1.47E+08	1.47E+08	-	140	1	RNVU1-8|	0	0
NM_0012	chr5_KI27	274862	302932	+	1560	5	SMN1|BCD	0	0
NR_03889	chr2	1.74E+08	1.74E+08	+	3119	1	LINC01305	0	0.228
NM_0036	chr2	39249266	39437312	-	4326	1	MAP4K3|G	3.38	6.228
NM_0052	chr3	1.22E+08	1.22E+08	+	822	1	CSTA|ARE	0.094	0.067
NM_1824	chr17	40977716	40987135	-	1927	2	KRT40|CK-	0	0
NM_0150	chr3	1.83E+08	1.83E+08	-	4981	1	MCF2L2|A	2.969	1.911
NM_0012	chr1	26432282	26471304	+	3198	1	DHDDS|CI	16.187	18.019
NM_1384	chr11	18080343	18106091	-	1517	1	SAAL1|SPA	13.386	16.433
NR_01542	chr9	1.34E+08	1.34E+08	+	5731	1	LINC00094	22.921	20.834
NM_1738	chr8	1.43E+08	1.43E+08	+	4792	1	ZFP41|ZNF	3.597	4.102
NM_0216	chr3	1.86E+08	1.86E+08	+	3175	1	SENP2|AX	17.507	15.302
NM_0011	chr16	89093809	89155846	+	3566	1	ACSF3|-|-	6.599	6.061
NM_0174	chr12	53938792	53946544	+	2392	1	HOXC13|E	0.032	0.326
NR_10505	chr5	1.35E+08	1.35E+08	-	836	1	C5orf66-AS	0	0
NM_0010	chr5	62306162	62387184	+	4138	1	KIF2A|CDC	16.707	20.251
NM_0013	chr17	82051865	82057470	+	1859	1	GPS1|COP	92.245	93.101
NM_0011	chr19	7037744	7040173	-	769	1	MBD3L4|-	0	0
NM_0063	chr14	67651150	67674885	-	1322	1	VTI1B|VTI1	48.433	50.664
NM_0010	chr22	29073078	29146820	+	6127	1	KREMEN1|	18.676	13.847
NR_12534	chr5	55231153	55232991	-	1337	1	CCNO|CCN	0.203	0.051
NR_03610	chr9	18573306	18573379	+	73	1	MIR3152|-	0	0
NR_02734	chr7	55681074	55688032	-	2167	1	FKBP9P1|F	0.131	0.139
NM_0044	chr2	2.41E+08	2.41E+08	+	3299	1	SEPT2|DIF	109.049	97.544
NR_13275	chr12	50456571	50456786	+	215	1	SNORD133	0	0.064
NM_0062	chr10	13277796	13300130	-	1599	1	PHYH|LN1	6.773	6.656
NM_0218	chr4	1.41E+08	1.41E+08	-	992	1	UCP1|SLC	0.065	0.014
NM_0011	chr1	1.61E+08	1.61E+08	-	580	1	TSTD1|KA	0.111	0.024
NM_0012	chr6	13363355	13486183	-	2477	1	GFOD1|AD	1.307	2.759
NM_0051	chr4	78551588	78610451	+	1603	1	ANXA3|AN	1.794	0.12
NM_0043	chr10	1.03E+08	1.03E+08	-	3866	1	ARL3|ARFI	35.352	35.22
NM_0198	chr18	3066807	3220108	-	5520	1	MYOM1|S	0.764	0.834
NR_00304	chr12	56643680	56643754	-	74	1	SNORD59E	0.871	2.406
NM_0013	chr1	1.14E+08	1.14E+08	+	3530	1	DCLRE1B|	7.188	8.435
NR_10424	chr7	1.23E+08	1.24E+08	-	2926	1	IQUB|-|-|	0.212	0.239
NR_10356	chr9	1.21E+08	1.21E+08	-	4762	1	GSN-AS1|C	0.198	0.365
NM_0011	chr19	5782960	5784765	-	1387	1	PRR22|-|-	1.868	1.58
NM_1384	chr19	50466785	50476746	-	1221	1	FAM71E1|	8.279	4.218

NM_0010{	chr15	72398327	72408367	+	1289	1	TMEM202	0	0
NM_0155⁄	chr3	66378797	66500421	-	4741	1	LRIG1|LIG-	15.542	40.84
NM_0010(	chr7	1.43E+08	1.43E+08	-	931	2	OR9A2|-|-	0	0
NM_0326(	chr8	33370826	33473146	-	3540	1	FUT10|FU(	1.242	1.497
NM_0207!	chr10	25175361	25602228	+	6649	1	GPR158|-|	0.735	0.812
NR_11090	chr2	2.24E+08	2.24E+08	-	4035	1	AP1S3|PS(	0.467	0.479
NR_03748	chr1	2.47E+08	2.47E+08	-	93	1	MIR3916|r	1.109	0.442
NR_10376	chr2	1.3E+08	1.3E+08	-	2407	1	CYP4F62P|	0	0
NR_12168	chr12	62601751	62603434	-	1683	1	LINC01465	0.215	0.269
NM_1830⁄	chr13	26212768	26221891	-	3318	1	RNF6|-|-|:	2.028	2.374
NM_0064;	chr22	29312933	29315756	-	1485	1	RASL10A|I	0.99	0.553
NM_0010:	chr9	19376256	19380237	-	821	1	RPS6|S6|-	812.415	658.274
NM_0019:	chr8	1.44E+08	1.44E+08	+	1225	1	CYC1|MC3	83.287	68.955
NR_13237	chr3	57597789	57600915	+	434	1	ARF4-AS1|	1.723	1.483
NM_0052{	chr19	45589765	45602208	-	3043	1	GPR4|-|-|	0.114	0.005
NM_0030:	chr19	2754714	2783356	-	2301	1	SGTA|SGT	117.325	130.875
NR_13652	chr19	57267193	57280334	-	695	1	LOC10537.	0.631	0.493
NM_0011!	chr11	1.02E+08	1.02E+08	+	4972	1	YAP1|COB	4.759	3.408
NM_0065;	chr14	50418493	50532658	-	4353	1	MAP4K5|(	6.635	9.613
NR_10379	chr22	17121595	17132104	+	1171	1	LINC01664	0.418	0.292
NM_0001⁄	chr1	23845081	23868369	-	2108	1	FUCA1|FU	10.502	12.443
NM_0529(	chr3	1.87E+08	1.87E+08	-	698	1	RPL39L|RF	14.353	7.849
NR_03824	chr20	5431957	5445748	-	1612	1	LINC00658	0	0.017
NM_0201(	chr2	2.28E+08	2.28E+08	-	1987	1	C2orf83|-|	0	0
NM_0013:	chr2	1.52E+08	1.52E+08	-	8307	1	CACNB4|C	0.591	0.869
NR_13641	chr17	44115912	44120595	+	356	1	LOC10537	0.109	0.231
NM_0013:	chr6	1.25E+08	1.25E+08	+	2287	1	TPD52L1|I	2.706	1.072
NM_0182:	chr1	64745095	64833231	+	4347	1	RAVER2|-|	1.726	3.428
NM_0321:	chr6	1.09E+08	1.09E+08	+	3716	1	ARMC2|b/	1.298	1.143
NM_0246:	chr3	1.23E+08	1.23E+08	-	1944	1	HSPBAP1|	3.568	4.453
NR_03170	chr22	20086058	20086142	+	84	1	MIR1306|I	14.121	12.882
NM_1825{	chr20	32631625	32651981	-	4786	1	C20orf203	0.436	0.701
NM_0011:	chr1	1.51E+08	1.51E+08	+	3671	1	PIP5K1A|-	26.019	32.297
NM_0012!	chr1	1.54E+08	1.54E+08	+	1531	1	CREB3L4|/	17.584	9.984
NM_1523⁄	chr17	45254393	45262112	-	1277	1	SPATA32|/	0.162	0.043
NM_0246;	chr1	36307107	36321347	+	2310	1	SH3D21|C	3.455	1.316
NR_11083	chr4	1.24E+08	1.25E+08	-	1762	1	LOC10192	0	0.054
NM_0012⁄	chr16	89948731	89959366	+	812	1	DEF8|-|-|:	30.773	30.886
NM_0012(	chr9	1.32E+08	1.32E+08	-	1767	1	TTF1|TTF-:	6.947	6.945
NM_0052:	chr2	2.08E+08	2.08E+08	-	621	1	CRYGB|CR	0	0
NM_0012{	chr1	2.21E+08	2.21E+08	+	5302	1	MARK1|M	7.429	12.057
NM_0010:	chr1	70354810	70368022	+	797	1	HHLA3|-|-	9.787	8.765
NM_0011(	chr16	21684429	21760729	+	3440	1	OTOA|CT1	0.026	0.024
NM_0010{	chr10	88724544	88752756	+	1190	1	LIPK|LIPL2	0	0
NM_0010:	chr4	67737381	67756086	-	5704	1	GNRHR|GI	0.02	0.142
NM_0329⁄	chrX	1.02E+08	1.02E+08	-	1595	1	NXF5|-|-|)	0	0
NR_02705	chr9	21454268	21559698	-	2144	1	MIR31HG|	0.096	0.3
NM_0012;	chr9	1.13E+08	1.13E+08	+	1932	1	C9orf43|-|	0.747	0.404
NM_0013(	chr1	13175281	13179132	-	1433	3	PRAMEF27	0	0
NM_0011:	chr9	19115761	19127606	-	2001	1	PLIN2|ADF	3.157	6.352

NM_0013:	chr7	87196162	87220587	-	1103	1	TMEM243	2.291	2.123
NM_0333(	chr19	38930954	38933019	+	968	1	MRPS12|N	21.684	18.763
NR_10975	chr9	33732975	33738416	-	484	1	LINC01251	0.879	0.509
NM_0013	chr8	737719	1708476	+	9738.3	3	DLGAP2|D	0	0.001
NM_0038!	chr6	1.58E+08	1.58E+08	+	7379	1	SYNJ2|INP	0.37	0.529
NR_12640	chr13	63183101	63328094	-	686	1	LINC0037(	0	0
NM_0015!	chrX	1.3E+08	1.3E+08	+	5105	1	OCRL|INPI	22.635	29.098
NM_0010(	chr17	48892786	48895870	+	569	1	ATP5G1|A	78.652	67.161
NR_04657	chr14	19421026	19423994	+	1490	2	POTEH-AS:	0	0
NM_0148	chr6	1.1E+08	1.1E+08	+	3080	1	FIG4|ALS1	22.915	23.08
NM_1382!	chr14	34432938	34462262	-	2530	1	SPTSSA|C1	9.749	6.735
NR_02833	chr17	28275986	28307382	-	1947	1	KRT18P55	0.013	0.042
NM_1447(	chr1	67166486	67259967	+	2801	1	IL23R|-|-|	0	0
NM_0068	chr11	66052345	66068911	+	2870	1	SF3B2|Cus	148.652	152.581
NR_12158	chr9	1.26E+08	1.26E+08	-	2093	1	LOC10192!	0	0
NM_0011!	chr6	391739	411443	+	5313	1	IRF4|LSIRF	0.022	0.247
NM_1457!	chr8	1.44E+08	1.44E+08	+	3247	1	KIFC2|-|-|	22.249	28.879
NM_0011(	chr2	2.41E+08	2.41E+08	-	1109	1	AQP12B|II	0	0
NM_0000:	chr11	1.08E+08	1.08E+08	+	2120	1	ACAT1|AC	20.605	22.064
NM_0013:	chr2	1.97E+08	1.97E+08	-	11030	1	PGAP1|Bs	4.166	19.7
NM_0012(	chr12	1.24E+08	1.24E+08	+	4265	1	ZNF664|ZI	57.931	50.016
NM_0066	chr17	74541066	74546171	-	1581	1	CD300C|C	0.033	0
NM_0012!	chr15	1E+08	1.01E+08	-	3972	1	CERS3|AR(	0.045	0.038
NR_03156	chr19	53671968	53672040	+	72	1	MIR1323|I	0	0
NR_02438	chr17	64784841	64837184	-	2911	1	PLEKHM1F	2.511	2.136
NR_03763	chr4	1.19E+08	1.19E+08	+	1039	1	LOC64551:	7.334	6.512
NM_0010(	chr11	5777634	5778667	-	1031	1	OR52N5|C	0	0
NM_0013:	chr2	1.37E+08	1.38E+08	+	6059	1	THSD7B|-|	0.634	0.305
NM_0010(	chr1	2.48E+08	2.48E+08	-	925	1	OR6F1|OR	0	0
NM_0016!	chr8	1.03E+08	1.03E+08	+	5689	1	ATP6V1C1	7.037	9.286
NM_0013(	chr14	24809655	25049000	-	3752	1	STXBP6|H!	0.79	1.887
NM_0180!	chr19	7516118	7521025	+	1949	1	ZNF358|ZI	79.617	64.922
NR_13859	chr14	36473289	36519521	-	1029	1	SFTA3|NA	0	0
NR_02961	chr9	1.28E+08	1.28E+08	-	109	1	MIR199B|	0	0.126
NM_1981	chr4	1.21E+08	1.21E+08	-	2339	1	QRFPR|AC	0.006	0
NM_0012	chr2	2.36E+08	2.37E+08	-	3135	1	IQCA1|49:	0.049	0.061
NM_0063	chr1	42658377	42676758	+	788	1	PPIH|CYP-	26.016	18.964
NM_0011	chrX	71714374	71718285	-	1843.5	2	CXorf49|C	0	0
NR_13628	chr3	1.14E+08	1.14E+08	+	4691	1	SIDT1|SID-	0.041	0.21
NM_0806	chr20	32983775	33004433	-	1237	1	SUN5|SPA	0	0
NM_0012!	chr17	28714281	28718268	-	1132	1	RAB34|NA	22.803	19.541
NM_0161!	chr1	1.52E+08	1.52E+08	-	1908	1	CRNN|C1c	0	0
NM_0207!	chr6	43522330	43576075	-	5330	1	XPO5|exp!	28.675	29.357
NM_1826	chr20	64080082	64100643	+	3066	2	OPRL1|KO	1.165	0.956
NR_13531	chr21	14216130	14228372	+	1987	1	RBM11|-|	0	0.193
NR_13497	chr16	54847251	54848233	-	414	1	LOC10192	0	0.066
NR_12640	chr11_KI2	46272	48651	+	720	2	LINC0121!	0	0.114
NM_0010(	chr6	1.6E+08	1.6E+08	-	2354	1	TCP1|CCT-	106.982	127.323
NM_0012!	chr11	17493895	17544416	-	2158	1	USH1C|AII	0.03	0.057
NM_2073!	chr15	40331452	40340967	-	5314	1	C15orf52|	0.18	0.51

NM_0160	chr2	86503430	86563497	-	3184	1	CHMP3|C(	36	36.824
NM_0012(	chr19	7688757	7699479	-	1471	1	FCER2|BL/	0	0
NM_0154	chr8	94487777	94553518	-	6413	1	KIAA1429|	6.239	7.04
NR_02418	chr19	51855803	51887936	-	930	1	ZNF577|-|	5.962	8.174
NM_0145	chr7	82820480	83162881	-	17073	1	PCLO|ACZ	1.263	1.702
NM_0203(	chr1	23778386	23788232	+	1614	1	PITHD1|C1	67.823	70.216
NM_0004	chr7	1.17E+08	1.18E+08	+	6102	1	CFTR|ABC	0	0.002
NM_0150	chr19	37907221	38208372	+	7970	1	SIPA1L3|C	5.487	8.077
NM_0321	chr7	6104919	6161564	+	5100	1	USP42|-|-	7.746	8.444
NM_0209	chr12	1.24E+08	1.24E+08	+	2662	1	DDX55|-|-	7.692	7.301
NM_0224	chr16	729755	741038	-	2133	1	NARFL|HP	18.848	19.496
NM_0010	chr20	32702691	32744008	-	1897	1	COMMD7|	46.483	52.802
NM_0001	chr2	74832655	74893354	+	7073	1	HK2|HKII|	17.558	2.835
NM_0124	chr3	1.23E+08	1.23E+08	+	3407	1	SEC22A|SE	4.81	7.679
NM_0050	chr19	617223	633568	-	3777	1	POLRMT|/	10.528	9.661
NM_1829	chr22	20111866	20117295	-	2825	1	TRMT2A|H	13.364	12.737
NM_0328(	chr10	62804756	62808479	+	3721	1	ADO|C10c	9.321	11.146
NM_0013	chr17	41835685	41848363	+	2149	1	KLHL10|SF	0.126	0.127
NM_0069	chr14	50117121	50231693	-	5607	1	SOS2|NS9	3.787	4.277
NM_0002	chrX	19343893	19361707	+	3359	1	PDHA1|PD	31.844	31.496
NM_1526	chr19	38384352	38388028	-	2148	1	GGN|-|-|1	4.328	2.748
NM_0312	chr1	1.58E+08	1.58E+08	-	3445	1	FCRL4|CD	0	0
NM_0013	chr18	48027268	48409422	-	4345	1	ZBTB7C|AI	3.997	2.292
NR_13445	chr10	73252741	73254346	+	807	1	DNAJC9-A	3.866	5.075
NR_11015	chr7	1.13E+08	1.13E+08	+	671	1	LOC10099	0	0
NM_0314	chr14	74903954	74922442	-	5182	1	RPS6KL1|F	14.421	19.583
NM_0251	chr8	37858947	37899497	-	5947	1	RAB11FIP1	2.309	1.17
NR_11090	chr16	3125529	3134882	-	1119	1	ZNF213-AS	8.446	5.68
NR_00278	chr3	10017553	10026136	-	800	1	CIDECP|CI	17.857	16.299
NM_0142	chr3	15206226	15252916	+	4345	1	CAPN7|CA	5.077	6.491
NM_0013	chr16	19523857	19553406	+	5342	1	CCP110|CI	5.03	6.523
NM_0307	chr1	1.97E+08	1.97E+08	+	2816	1	CFHR5|CFI	0	0.005
NR_10991	chr5	61663158	61698432	-	1660	1	LOC10192	0	0.033
NM_0012	chr19	36003299	36008793	-	1018	1	SYNE4|C1	0.722	0.417
NM_0010(	chr5	1.32E+08	1.32E+08	-	6515	1	ACSL6|AC	1.369	1.48
NM_0012	chr13	37009314	37059713	-	2818	1	SUPT20H|	25.357	23.452
NM_0040	chr10	1.33E+08	1.33E+08	-	1319	1	ECHS1|ECI	115.836	108.278
NM_0124	chrX	1.12E+08	1.12E+08	-	5827	1	TRPC5|PPI	0.009	0.099
NM_0013	chr3	1.38E+08	1.38E+08	+	5067	1	ESYT3|CHI	0.084	0.143
NM_0325	chr17	60177330	60392225	-	6990	1	USP32|NY	7.323	6.962
NR_03335	chr18	14075990	14132490	-	2275	1	ZNF519|H	1.349	1.583
NM_0013(	chr15	77613024	77707507	-	3072	1	LINGO1|LE	66.579	62.029
NR_11010	chr15	91463339	91494850	+	2134	1	CRAT37|-|	0	0
NM_0045	chr11	1.18E+08	1.18E+08	-	4916	1	SCN2B|AT	0.399	1.73
NR_13524	chr14	56514331	56551309	+	2224	1	LOC10192	0.035	0.154
NR_04698	chr13	19587118	19588490	+	560	1	LINC0035C	0	0
NM_1457	chr10	1.16E+08	1.16E+08	-	9117	1	GFRA1|GD	7.843	7.953
NM_0059	chr14	64388041	64460007	+	3418	1	MTHFD1|I	4.474	12.407
NM_0312	chrX	1.08E+08	1.08E+08	-	1243	1	TEX13B|T(	0	0
NM_1532	chr5	97091870	97142816	-	3959	1	LIX1|C5or	2.465	1.104

NR_04659	chr1	97095923	97322955	+	528	1	DPYD-AS1	0.122	0.493
NM_0045	chr2	1.51E+08	1.52E+08	-	20447	1	NEB|NEB1	0.053	0.062
NM_0020	chrX	48786574	48794310	+	1471	1	GATA1|ER	0.035	0.028
NM_0010	chr2	1.09E+08	1.1E+08	+	5602	1	SH3RF3|P	3.508	9.858
NR_11028	chr2	1.3E+08	1.3E+08	+	2394	1	LINC0185	0	0
NM_0212	chr9	976968	991732	+	2176	1	DMRT3|D	0.047	0.579
NM_0064	chr3	69084939	69106088	+	2123	1	ARL6IP5|C	66.842	61.117
NR_00273	chr6	1.19E+08	1.19E+08	+	2460	1	BRD7P3|B	0	0.011
NM_0010	chr7	35800986	35907105	+	4332	1	SEPT7|CD	16.682	18.528
NM_1334	chr9	1.02E+08	1.02E+08	-	7760	1	GRIN3A|G	0.017	2.422
NM_0308	chr20	3046029	3048250	+	1033	1	MRPS26|C	50.875	49.193
NM_0008	chr12	88492793	88580473	-	5431	1	KITLG|DCL	1.289	3.036
NM_0012	chr4	84582904	84651340	+	4523	1	CDS1|CDS	1.2	1.323
NM_0029	chr4	73995642	73998729	-	2482	1	CXCL5|EN	0.036	0.028
NR_02968	chr17	48037161	48037247	-	86	1	MIR152|M	0	0
NM_0182	chr12	71686098	71704059	+	4650	1	TMEM19|	5.648	6.168
NM_0160	chr11	68165719	68213317	-	2681	1	KMT5B|C	14.11	13.717
NM_0013	chrY	2691133	2741309	+	1229	2	CD99|HBA	0	0
NM_1981	chr9	1.31E+08	1.31E+08	-	928	1	QRFP|26R	0.57	0.413
NM_0013	chr11	1.26E+08	1.27E+08	-	3723	1	KIRREL3|K	10.874	15.897
NM_0019	chr17	76006845	76027452	-	6590	1	EVPL|EVP	0.008	0.015
NR_02689	chr15	50348936	50354879	-	5943	1	FLJ10038|	6.914	6.642
NR_10987	chr5	1.16E+08	1.16E+08	+	964	1	SEMA6A-A	7.757	7.275
NM_0142	chr3	45594831	45681263	+	6274	1	LIMD1|-|-	6.769	6.493
NM_0013	chr7	55470608	55553887	-	2705	1	VOPP1|EC	22.117	20.406
NM_2068	chr3	52685920	52694494	+	1975	1	GNL3|C77	17.078	18.933
NR_03999	chr14	47764953	47795014	-	2883	1	LINC0064	1.422	1.411
NM_1816	chr1	45511035	45521938	-	1007	1	PRDX1|M	268.1	303.647
NM_0016	chr12	56360571	56362799	-	1685	1	APOF|Apo	0.046	0.024
NM_0012	chr14	77708071	77717601	+	399	1	SLIRP|C14	101.403	87.469
NM_0010	chr6	1.17E+08	1.17E+08	-	1030	1	FAM162B|	0.013	0
NM_0223	chr11	1.25E+08	1.25E+08	+	4524	1	ROBO3|H	41.77	51.034
NM_0012	chr4	75949906	75990962	-	2895	1	SDAD1|-|-	4.659	6.231
NR_03887	chr8	63769430	63785497	+	3705	1	LINC0128	0.024	0.037
NR_04008	chr7	38341577	38378637	+	3512	1	TRG-AS1|	0	0
NM_1784	chr12	1.19E+08	1.2E+08	+	2434	1	CCDC60|-|	0.117	0.023
NR_01537	chr19	15828947	15836321	+	2311	1	UCA1|CU	0	0
NM_0047	chr1	70205682	70252018	+	2777	1	SRSF11|N	21.84	28.39
NR_03411	chr2	24972112	25039696	+	4673	1	DNAJC27-	2.296	4.189
NR_13236	chr7	32826302	32838744	+	1160	1	DPY19L1P	0.545	0.224
NM_1825	chr3	1.84E+08	1.84E+08	+	1451	1	HTR3D|5H	0.018	0
NM_0010	chr22	44243677	44312851	-	6734	1	KIAA1644|	17.127	12.912
NR_11043	chr4	1.88E+08	1.88E+08	-	478	1	LOC10050	0	0
NR_12121	chr9	79975023	80034555	+	1022	1	LINC01507	0	0
NM_0061	chr1	11845710	11847783	-	849	1	NPPA|AN	3.371	2.63
NR_13519	chr16	25066887	25068955	+	1949	1	LOC28388	0.615	0.485
NR_02444	chr8	1.41E+08	1.41E+08	+	792	2	LINC0130	0	0
NM_0062	chr12	1.2E+08	1.2E+08	+	2392	1	PRKAB1|A	31.862	34.815
NR_00438	chr19	10107651	10107735	+	84	1	SNORD10	0	0
NM_0323	chr12	1.21E+08	1.21E+08	-	1500	1	COQ5|-|-|	35.861	35.932

NM_00524	chr14	28767072	28770277	+	3203	1	FOXG1|BF	0.25	47.185
NM_00131	chr9	5450503	5463238	+	901	1	CD274|B7-	0.114	0.03
NR_00392	chr12	1.2E+08	1.2E+08	-	143	1	RNU4-1|RI	0	0.192
NM_00119	chr7	1.29E+08	1.29E+08	+	3861	1	LOC100130	2.368	3.98
NR_02834	chr7	63349070	63351774	+	653	1	LOC100287	0	0
NR_02804	chr7	1E+08	1E+08	-	2856	1	GATS|STA(	62.567	92.963
NM_20143	chr11	63681450	63759891	+	2521	1	RTN3|ASY	232.451	300.112
NM_00130	chr3	1.95E+08	1.95E+08	-	2257	1	XXYLT1|C3	23.856	27.503
NM_00116	chr7	13891231	13991240	-	6207	1	ETV1|ER81	4.917	9.341
NM_01626	chr6	1.12E+08	1.12E+08	-	2283	1	TUBE1|TU	4.671	4.506
NM_00109	chr12	42081846	42144871	-	7386	1	GXYLT1|GI	0.508	0.708
NM_20335	chr14	39266298	39351193	+	3485	1	MIA2|CTA	1.295	1.38
NM_00447	chr9	1.37E+08	1.37E+08	-	2410	1	FUT7|FucT	0.38	0.216
NM_00120	chr18	50268846	50281767	-	2640	1	MBD1|CXX	25.118	26.325
NM_00127	chr1	2.26E+08	2.26E+08	-	3056	1	LIN9|BARA	0.873	1.268
NM_00220	chr5	51383124	51394729	+	2709	1	ISL1|ISLET	0.19	0
NR_03608	chr3	1.95E+08	1.95E+08	-	74	1	MIR3137|-	0	0
NM_00128	chr10	84328589	84374018	+	2071	1	CCSER2|FA	5.093	8.399
NM_00130	chr16	57542637	57577188	+	3326	1	ADGRG5|C	0.023	0.008
NM_00427	chr12	1.21E+08	1.21E+08	+	1051	1	CABP1|CA	4.22	2.567
NM_00110	chr7	44512536	44541315	-	4945	1	NPC1L1|N	0.083	0.069
NM_00458	chr1	75786194	75795090	+	1503	1	RABGGTB|	20.829	24.387
NR_10701	chr3	1.77E+08	1.77E+08	+	48	1	MIR7977|I	0	0
NM_00219	chr2	1.9E+08	1.9E+08	+	2011	1	INPP1|-|-|	2.295	3.978
NM_00127	chrX	26216169	26218270	+	1212	1	MAGEB5|C	0	0
NM_03250	chr8	33485167	33501260	+	3562	1	MAK16|M	4.376	4.791
NM_00125	chr16	2972791	2980539	-	1953	1	PKMYT1|N	13.89	12.21
NM_00100	chr19	56087363	56121373	-	2016	1	ZNF787|TI	16.91	14.539
NR_02835	chr5	6310441	6337292	-	2417	1	LINC02145	0.128	0.17
NM_00127	chr16	71626153	71641965	+	2080	1	MARVELD3	0.236	0.145
NR_10374	chr1	1.13E+08	1.13E+08	-	1817	1	LINC01356	0.014	0.151
NM_00379	chr16	84053761	84116943	-	4355	1	MBTPS1|P	44.199	52.313
NM_00128	chr4	92304399	93774555	+	5037	1	GRID2|Glu	2.084	1.667
NM_00156	chr2	98444858	98581821	+	3295	1	INPP4A|IN	14.298	15.094
NR_03389	chr5	3417152	3536094	-	3194	1	LINC01019	0.02	0.034
NM_00115	chr2	218136	264097	-	1749	1	SH3YL1|RA	4.748	7.064
NM_00015	chr9	34646589	34650598	+	1392	1	GALT|-|-|S	20.618	18.184
NR_13651	chr18	12432774	12447798	+	1156	1	LOC10537	2.844	10.901
NM_00115	chr7	57450177	57473559	+	5204	1	ZNF716|-|	0	0.003
NM_00323	chr8	1.33E+08	1.33E+08	+	8403	1	TG|AITD3|	0.133	0.163
NM_00222	chr19	12791496	12793311	+	1813	1	JUNB|AP-1	10.248	14.672
NM_15361	chr5	79689836	79800226	+	12877	1	CMYA5|C5	0.016	0.074
NR_12032	chr15	97370371	97521811	-	1721	1	LOC101927	0.12	0.016
NR_11075	chr19	28435389	28727694	-	3116	1	LOC100420	0.079	0.018
NM_14520	chr8	1.44E+08	1.44E+08	-	1688	2	NAPRT|NA	0	0
NR_12641	chr3	1.9E+08	1.9E+08	+	726	1	P3H2-AS1|	0.089	0.075
NM_19844	chr1	2.04E+08	2.04E+08	-	876	1	GOLT1A|C	0.191	0
NR_03894	chr7	16210486	16270604	+	2068	1	ISPD-AS1|-	0	0
NM_01476	chr3	10248493	10281222	+	4897	1	TATDN2|-	22.009	29.055
NM_00112	chr1	1.53E+08	1.53E+08	+	638	1	LCE6A|C1c	0	0

NM_0013!	chr3	16586794	16605499	-	3035	1	DAZL|DAZ	0.013	0.117
NM_1524:	chr11	94567308	94621421	+	3177	1	PIWIL4|HI'	0.195	0.185
NM_0010:	chr7	1.25E+08	1.25E+08	-	663	1	C7orf77|-|	0	0
NM_0007!	chr15	78624294	78641245	-	2440	1	CHRNB4|-	18.648	10.958
NR_03746	chr16	2135977	2136129	-	152	1	MIR3180-!	0	0
NM_0069	chr21	14371116	14383188	-	3979	1	HSPA13|S'	18.687	17.181
NM_0013(	chr10	13221766	13234328	-	657	1	UCMA|C1(	0	0.083
NM_0011	chr14	34982898	35029567	+	2172	1	SRP54|-|-	20.142	18.654
NM_0002:	chr19	48115445	48170603	-	3354	1	LIG1|-|-|1	16.334	13.362
NR_03973	chr15_KI2	298335	298428	+	93	9	MIR4509-1	0	0
NM_0010:	chr8	56072809	56074581	-	833	1	RPS20|S2(	237.036	181.81
NR_10983	chr8	1.27E+08	1.27E+08	+	1751	1	CCAT2|LIN	0	0
NM_0012!	chr2	2.32E+08	2.32E+08	-	2870	1	ECEL1|DA!	19.974	29.747
NR_02835	chr3	1E+08	1E+08	+	307	1	TMEM30C	0	0
NR_07343	chr3	1.25E+08	1.26E+08	-	2395	1	SNX4|ATG	21.728	20.217
NM_0071(	chr4	672436	674338	-	359	1	ATP5I|ATP	270.871	203.891
NM_0206:	chr19	57455174	57456739	-	1563	1	VN1R1|V1	0.701	1.166
NR_11021	chr2	1.86E+08	1.86E+08	-	1052	1	LOC10192	0.245	0.273
NM_0010(	chrX	1.54E+08	1.54E+08	+	6398	1	ATP2B3|CI	2.952	5.314
NM_0011:	chrX	1.16E+08	1.16E+08	+	3307	1	PLS3|BMN	5.938	12.185
NM_0123:	chr1	38862490	38873378	-	2558	1	MYCBP|AN	2.606	1.542
NM_0011	chr12	1.01E+08	1.01E+08	-	3233	1	ARL1|ARFI	11.797	9.117
NM_0012!	chr4	9243879	9245471	+	1591	1	USP17L17	0	0
NR_13451	chr10	1.2E+08	1.21E+08	+	1037	1	PLPP4|DPI	0.149	2.008
NM_0013:	chr2	61888723	62136070	+	630	1	COMMD1|	28.386	30.764
NM_0010	chr2	2.08E+08	2.08E+08	-	9797	1	PLEKHM3|	3.74	4.096
NM_0011	chr5	1.16E+08	1.16E+08	-	3649	1	TMED7-TI(	3.841	3.656
NM_0011:	chr1	7961654	7985282	+	894	1	PARK7|DJ-	236.251	230.505
NM_1735	chr3	1.38E+08	1.38E+08	-	3480	1	DZIP1L|DZ	0.708	0.85
NR_13511	chr1	2.3E+08	2.3E+08	+	665	1	LINC0173€	0	0
NM_1527(	chr7	88794106	88795717	-	1015	1	C7orf62|-|	0	0
NM_2073:	chr14	58395926	58408701	+	603	1	TOMM20L	0.428	0.341
NM_1784:	chr1	25861484	25871253	-	3019	1	PAQR7|MI	4.839	3.797
NR_03609	chr5	1.55E+08	1.55E+08	-	60	1	MIR3141|	0	0
NR_03984	chr11	1.04E+08	1.04E+08	+	74	1	MIR4693|	0	0
NR_02712	chrX	50591645	50814044	-	5995	1	SHROOM4	0.353	0.379
NM_0059!	chr11	67631303	67639560	-	1538	1	TBX10|TB)	0	0.036
NM_0010!	chr7	1.03E+08	1.03E+08	-	1669	1	POLR2J3|F	0.433	0.501
NR_00296	chr6	1.6E+08	1.6E+08	-	139	1	SNORA29|	0	0.493
NM_0011(	chr3	1.87E+08	1.87E+08	+	2715	1	KNG1|BDK	0	0
NM_0011:	chrX	1.07E+08	1.07E+08	-	1041	1	RIPPLY1|-|	0.05	0
NR_04659	chr3	1.92E+08	1.92E+08	+	573	1	FGF12-AS1	0	0
NM_0012	chr22	19175575	19178505	-	1505	1	SLC25A1|(	76.033	80.662
NM_0191:	chr16	23557541	23574389	+	4922	1	UBFD1|UE	34.104	35.445
NM_0011(	chrX	1.34E+08	1.34E+08	+	1296	1	CCDC160|	0.846	0.328
NR_03789	chr3	1.28E+08	1.28E+08	+	1795	1	DNAJB8-A!	0	0
NM_0011(	chr11	1.14E+08	1.14E+08	+	1985	1	HTR3A|5-I	0.637	0.2
NM_0011:	chr6_GL0C	922362	937746	+	1736	8	MOG|BTN	0	0
NM_0010:	chr1	1242446	1246722	-	1033	1	FAM132A|	0.649	0.716
NM_0122(	chr7	1.24E+08	1.24E+08	+	2399	1	HYAL4|CSI	0	0.006

NM_0334	chr1	2.28E+08	2.28E+08	-	493	1	HIST3H2A|	32.378	39.841
NR_10699	chr8	1.01E+08	1.01E+08	-	56	1	MIR7705|I	0.23	0.734
NR_03889	chr2	1.06E+08	1.06E+08	-	1412	1	LOC28500	0	0.019
NM_0165	chr17	29042900	29057218	+	2402	1	PIPOX|LPII	6.941	6.067
NM_0013	chr1	2.34E+08	2.34E+08	+	2820	1	SLC35F3|-	2.016	2.987
NR_02439	chr7	1.59E+08	1.59E+08	+	4008	1	LINC0068	0.006	0.014
NM_0013	chr14	1.02E+08	1.02E+08	+	2099	1	DIO3|5DIII	49.111	22.807
NM_0143	chr1	2.1E+08	2.1E+08	+	8472	1	DIEXF|C1o	1.453	2.15
NR_04981	chr1	1.48E+08	1.48E+08	-	75	1	MIR5087|	0	0
NR_02988	chr19	45638994	45639087	-	93	1	MIR330|M	0	0
NR_03789	chr5	1.54E+08	1.54E+08	-	3447	1	SAP30L-AS	0.127	0.143
NR_02456	chr17_GL0	889441	892431	+	3006.7	3	MAPT-IT1|	0.013	0.059
NM_0013	chr12	95657807	95791206	-	3194	1	NTN4|PRC	1.752	1.891
NR_10701	chr4	21464700	21464758	-	58	1	MIR7978|I	0	0
NM_0012	chr22	37065436	37109409	-	3105	1	TMPRSS6|	0.12	0.203
NM_0011	chr13	57140918	57143939	+	1842	5	PRR20B|-|	0	0
NR_12058	chr11	1.2E+08	1.2E+08	+	519	1	LOC10272	0.323	0.132
NM_0011	chr14	24290598	24299833	-	1469	1	DHRS1|SD	12.455	8.97
NR_00393	chr10	29409534	29422372	+	2060	1	SVIL-AS1|-	6.753	0.632
NR_03386	chr17	15764703	15765689	+	568	1	CDRT15P2	0	0
NM_0012	chrX	49028726	49043517	-	3374	1	TFE3|RCCP	26.314	26.464
NM_0204	chr19	19645201	19663694	-	3836	1	ATP13A1|	18.497	19.092
NM_0010	chr8	58553169	58582860	+	2138	1	SDCBP|MD	36.335	41.962
NM_0053	chr6	26234212	26234988	-	774	1	HIST1H1D	0.366	0.159
NM_1530	chr7	66628881	66643229	+	5045	1	KCTD7|CLI	26.993	34.249
NM_0002	chr11	1.29E+08	1.29E+08	-	2400	1	KCNJ1|KIR	0.005	0.011
NM_0059	chr19	6210381	6279948	-	4491	1	MLLT1|EN	49.917	49.012
NM_0210	chr12	21264600	21334898	-	7196	1	SLCO1A2|	0.317	0.19
NM_0012	chr10	62238150	62268863	-	2232	1	RTKN2|PLI	0.364	0.565
NM_0010	chr4	9444534	9450514	+	210	1	DEFB131|I	0	0
NM_0318	chr5	1.41E+08	1.41E+08	+	5978	1	PCDHA9|P	14.375	13.839
NM_0243	chr12	1.02E+08	1.02E+08	-	5607	1	GNPTAB|G	7.032	13.357
NM_0120	chr19	45406209	45410766	+	3281	1	CD3EAP|A	9.184	9.468
NR_13618	chr3	1.94E+08	1.94E+08	-	928	1	LINC0202	0.083	0.044
NM_0024	chr7	2242222	2251145	+	656	1	NUDT1|M	23.92	26.684
NM_0045	chr10	32009010	32056443	-	5861	1	KIF5B|HEL	17.372	22.888
NR_10431	chr2	1.81E+08	1.81E+08	+	1431	1	SCHLAP1|I	0.009	0.01
NM_0011	chr13	60397293	60573879	+	2564	1	TDRD3|-|-	7.598	7.153
NM_0011	chr13	21153595	21176602	-	2790	1	SKA3|C13	1.091	1.232
NM_0013	chrX	7252244	7354643	+	6209	1	STS|ARSC|	1.456	1.284
NM_0010	chr11	1.24E+08	1.24E+08	-	970	1	OR6T1|OR	0	0
NR_13632	chr1_KI27	174336	180639	+	880.3	3	LOC10192	0	0
NM_0012	chr19	14073009	14075062	+	1295	1	MISP3|-|-	7.298	4.696
NR_00293	chr7	1.52E+08	1.52E+08	+	853	1	FABP5P3|I	0.106	0.048
NM_0190	chr2	2.34E+08	2.34E+08	+	2378	1	UGT1A10|	0.033	0
NM_0012	chr2	88170295	88186637	+	1553	1	THNSL2|S	0.158	0.388
NR_03792	chr20	29414075	29416961	-	2070	1	FRG2EP|-|	0	0.013
NR_02997	chr14	1.01E+08	1.01E+08	+	78	1	MIR409|M	0	0
NM_0050	chr1	1.73E+08	1.73E+08	-	743	1	TNFSF18|	0	0.295
NM_0004	chr19	44945982	44949566	+	724	1	APOC2|AP	0.018	0

NR_12592	chr4	27217479	27282225	+	2796	1	LOC10192	0	0
NM_0012	chr4	1.9E+08	1.9E+08	+	1569	1	DUX4|DU	0	0
NR_13661	chr10	80247758	80255408	+	3462	1	LOC10013	0	0
NM_0174	chr17	81035147	81110512	+	3172	1	BAIAP2|B	15.784	20.222
NM_0123	chr17	3197517	3198448	+	930	1	OR1A2|OF	0	0
NR_02336	chr10	1.26E+08	1.26E+08	+	744	1	TEX36-AS1	0	0
NM_0054	chr2	44941898	44946077	+	2519	1	SIX3|HPE2	2.186	0.816
NM_0029	chr1	1.69E+08	1.69E+08	+	1362	1	XCL1|ATA	0.028	0.08
NR_04702	chr13	89235291	89280240	+	864	1	LINC0044C	0	0.127
NM_1780	chr6	3224261	3227734	-	2013	1	TUBB2B|P	608.421	507.225
NM_0532	chr1	1.62E+08	1.62E+08	-	2529	1	SH2D1B|E	0	0
NM_0046	chr10	87659719	87747705	+	3829	1	PAPSS2|A	1.822	0.297
NM_0012	chr12	48042898	48106308	-	4664	1	SENP1|Su	6.192	6.182
NM_1731	chr16	68085366	68229259	+	6320	1	NFATC3|N	5.204	5.167
NM_0013	chr14	94487274	94504222	-	1490	1	SERPINA1	0	0
NM_1523	chr3	51894876	51903370	-	724	1	IQCF1|-|-|	0.107	0.095
NR_10505	chr5	1.41E+08	1.41E+08	-	1006	1	LOC10192	0.038	0.027
NM_0010	chr8	47773108	47960183	-	13329	1	PRKDC|DN	16.57	14.958
NR_03023	chrX	1.47E+08	1.47E+08	-	91.5	2	MIR509-1|	0	0
NR_13073	chr16	22916	25112	+	1035	1	WASIR2|N	0.112	0.225
NM_0201	chr3	8877196	8963475	-	5724	1	RAD18|RN	0.948	1.883
NR_03991	chr20	34049119	34049190	+	71	1	MIR4755|	0	0
NM_0011	chr9	1.29E+08	1.29E+08	+	4242	1	LRRC8A|A	27.553	33.29
NM_0201	chr8	40153468	40155308	+	1838	1	C8orf4|TC	0.701	4.061
NM_0010	chrX	72127111	72131901	-	4334	1	RGAG4|64	14.479	20.77
NM_0240	chr5	7859159	7869037	-	2428	1	FASTKD3|-	2.007	2.522
NR_10344	chr18	80067256	80081304	+	804	1	RBFADN|-	0.866	1.141
NM_0807	chr7	76389329	76409695	-	2793	1	SSC4D|S4D	1.08	0.495
NM_0011	chr20	1226120	1254501	+	1792	1	RAD21L1|	0.014	0
NM_0022	chr7	20331102	20415759	+	8761	1	ITGB8|-|-|	1.651	5.711
NM_0133	chr7	2234291	2242198	-	1596	1	MRM2|FJH	24.309	20.46
NR_13503	chr12	22609567	22625046	-	552	1	LOC10536	0.21	0.05
NR_03627	chr17_GL	165799	165871	-	72	4	MIR4315-2	0	0
NM_0069	chr5	6714605	6757048	+	3846	1	PAPD7|LA	15.981	15.962
NR_11076	chr22	11897406	11956534	+	1377	1	LOC10272	0	0.139
NM_0211	chr19	35282346	35285142	+	582	1	HAMP|HE	0.022	0
NR_11086	chr6	87784860	87911731	+	588	1	LOC10192	0	0
NM_0011	chr13	57140918	57143939	+	1842	5	PRR20C|-|	0	0
NM_0013	chr1	1.54E+08	1.54E+08	-	1162	1	S100A16|	1.409	5.34
NM_0011	chr4	1.22E+08	1.22E+08	+	1891	1	ADAD1|Te	0	0
NM_0806	chr20	23564733	23568749	-	953	1	CST9L|CTE	0	0.014
NM_0003	chr15	89209867	89221691	-	1741	1	RLBP1|CR	0.126	0.354
NM_0332	chr19	38251237	38256591	-	771	1	PPP1R14A	0.97	1.83
NM_0012	chr3	17962572	18268918	+	2324	1	LOC33986	0.022	0.183
NR_03170	chr10	1.03E+08	1.03E+08	-	148	1	MIR1307|	0.523	0.37
NM_0046	chr2	1.92E+08	1.92E+08	-	3246	1	SDPR|CAV	0.068	0.013
NM_0012	chr17	18865469	18931286	+	1667	1	PRPSAP2|	57.522	44.871
NM_0061	chr2	1.56E+08	1.56E+08	-	3521	1	NR4A2|HZ	17.969	6.858
NM_0013	chr13	49444293	49495003	+	5847	1	SETDB2|C	1.795	1.562
NM_0331	chr5	1.43E+08	1.43E+08	-	3503	1	FGF1|AFG	0.331	0.332

NM_0228:	chr2	2.38E+08	2.38E+08	-	6317	1	PER2|FASI	1.394	1.416
NR_13742	chr17	60149936	60179021	+	2738	1	CA4|CAIV|	2.882	2.491
NR_07341	chr9	1.27E+08	1.27E+08	+	1595	1	SLC2A8|GI	8.124	6.655
NM_0012(	chr1	1071746	1074307	-	1118	1	RNF223|-|	0.046	0.025
NM_0010(	chr7	1.44E+08	1.44E+08	+	931	1	OR2A25|C	0	0
NM_0010;	chr8	85187675	85214521	+	1578	1	E2F5|E2F-:	11.349	12.317
NM_0011;	chr11	1.26E+08	1.26E+08	+	1331	1	PATE3|HEI	0	0
NM_0011!	chr1	2.12E+08	2.12E+08	-	4473	1	INTS7|C1c	6.63	8.243
NM_1943:	chr2	1.49E+08	1.49E+08	+	4083	1	LYPD6|-|-	2.299	2.825
NR_12542	chr8	1.37E+08	1.37E+08	-	987	1	LOC10192	0	0
NM_1525	chr5	1.22E+08	1.22E+08	+	2826	1	SRFBP1|BU	0.926	1.381
NM_0028:	chr15	75467121	75579291	-	3930	1	PTPN9|MI	27.897	31.597
NM_0012;	chr8	1.44E+08	1.44E+08	+	5179	1	MROH1|H	5.094	5.718
NR_04765	chr2	96593155	96638379	-	5129	1	KANSL3|KI	27.625	28.55
NM_0012;	chr11	82973133	83071462	-	9776	1	RAB30|-|-	3.201	5.453
NM_0154;	chr1	25222276	25232522	-	1768	1	SYF2|CBPI	17.765	17.842
NM_0211:	chr17	28335522	28347009	+	3800	1	TNFAIP1|E	46.271	49.677
NM_0013(	chr5	1.02E+08	1.02E+08	-	1867	1	SLCO6A1|	0	0
NR_10688	chr3	48633636	48633698	-	62	1	MIR6824|I	0.624	4.197
NM_0070;	chr3	1.2E+08	1.2E+08	-	3828	1	FSTL1|FRP	28.515	21.744
NR_13446	chr5	14143702	14510204	+	10513	1	TRIO|ARH	37.158	49.244
NM_0012(	chr12	54948303	54973732	-	3602	1	TESPA1|H:	0.004	0
NR_03607	chr2	2.07E+08	2.07E+08	-	74	1	MIR3130-1	0	0
NM_1390:	chr11	2302013	2318200	+	1364	1	TSPAN32|.	0.095	0.181
NR_10466	chr16	79721312	79770532	-	679	1	MAFTRR|li	0	0.04
NR_10989	chr5	1.69E+08	1.69E+08	+	864	1	LOC72809.	0	0
NR_03163	chr11	94466495	94466580	-	85	1	MIR548L|I	0	0
NM_0011(	chr5	1.77E+08	1.77E+08	+	1904	1	SLC34A1|F	0	0
NR_03164	chr2	1.77E+08	1.77E+08	-	72	1	MIR1246|I	0	0
NR_02674	chr10	4871667	4916273	-	1386	1	AKR1C6P|	0	0
NM_0175;	chr7	73683568	73698221	+	1225	1	WBSCR22|	34.081	36.518
NM_0011!	chr2	63050057	63057831	+	2849	1	OTX1|-|-|	7.54	8.774
NR_12118	chr9	74371531	74383530	+	645	1	LOC10192	0	0
NM_0011;	chr8	22367249	22422736	+	4681	1	SLC39A14|	4.716	6.256
NR_04548	chr1	46433827	46445702	+	1258	1	FAAHP1|-|	0.123	0.174
NM_0121(	chr4	67558697	67607341	+	1974	1	STAP1|BRI	0.013	0.014
NM_0175!	chr17	35121612	35130732	+	1349	1	FNDC8|-|-	1.931	2.802
NM_0010!	chr16	4189374	4242080	-	4206	1	SRL|-|-|16	3.415	1.827
NM_1456!	chr2	11746596	11827407	+	5352	1	LPIN1|PAI	14.076	13.707
NM_0147:	chr2	20032650	20052028	-	1756	1	LAPTM4A|	59.922	85.521
NR_12634	chr13	1.12E+08	1.12E+08	-	654	2	LINC01044	0	0
NR_12635	chrX	1.31E+08	1.31E+08	-	291	1	LINC01201	1.108	0.706
NM_0010;	chr3	9397700	9478154	+	6821	1	SETD5|-|-	31.318	41.641
NM_0010;	chr18	8609445	8639382	+	2130	1	RAB12|-|-	15.424	20.333
NR_02456	chr19	35059059	35106304	-	1120	1	HPN-AS1|-	0	0.012
NM_0323(	chr5	1.76E+08	1.76E+08	-	1593	1	THOC3|TH	3.812	3.551
NM_0160(	chr16	66932055	66934423	-	692	1	FAM96B|C	118.352	101.184
NR_13630	chr9	34957487	34982544	+	3714	1	PHF24|-|-	0.68	2.829
NM_0053;	chr17	75776434	75779935	-	2855	1	H3F3B|H3	299.558	381.132
NR_02681	chr15	77223908	77225404	-	1496	1	LINC00597	0	0

NR_04857	chr17	19296596	19306261	-	1187	1	EPN2-AS1|	0.043	0.115
NR_10988	chr5	1.58E+08	1.58E+08	-	3723	1	LOC10192	0.003	0
NM_1734	chr3	10081320	10108231	-	1009	1	FANCD2OS	0.575	0.271
NR_02434	chr7	1.42E+08	1.42E+08	-	1492	2	MOXD2P|	0	0
NM_0207	chr3	1.25E+08	1.25E+08	-	9137	1	HEG1|HEG	2.282	12.08
NM_0010	chr11	45906535	45907282	-	363	1	C11orf94|	1.208	1.547
NM_0012	chr12	6570082	6606451	-	6349	1	CHD4|CHD	148.012	119.273
NR_13210	chr10	73495752	73507309	+	3090	1	PPP3CB-AS	0.889	1.006
NR_03387	chr2	1.13E+08	1.13E+08	-	509	1	FLJ42351|	0.076	0.619
NM_0012	chr11	1.17E+08	1.17E+08	-	5199	1	BACE1|AS	21.772	24.976
NR_00688	chr17	19188016	19188232	+	216	1	SNORD3A|	0.239	0.317
NM_0179	chr4	1.1E+08	1.1E+08	+	1264	1	MCUB|CC	6.885	4.996
NR_12574	chr13	42810366	42812562	-	757	1	LINC0105	0	0
NM_1452	chr6	70566922	70588903	+	520	1	SDHAF4|C	12.199	21.863
NM_0011	chr13	27978106	27988637	-	519	1	URAD|PR	0	0
NR_11032	chr9	35406755	35451108	+	2500	1	ATP8B5P|	0.335	0.52
NM_0052	chr20	64105830	64106831	-	1000	2	NPBWR2|	0	0
NM_0010	chr1	1.5E+08	1.5E+08	-	504	2	HIST2H3A|	0	0
NM_0010	chr19	19792711	19821751	-	3318	1	ZNF506|-|	2.569	3.802
NM_0062	chr3	1.67E+08	1.67E+08	-	1667	1	SERPINI2|	0.062	0.271
NM_1826	chr1	16057769	16073632	-	1692	1	FAM131C|	2.888	3.246
NM_0163	chr1	1.48E+08	1.48E+08	+	2100	1	GPR89B|G	0.577	0.567
NM_0013	chr9	21003624	21031459	-	4125	1	HACD4|PT	0.063	0.159
NM_0013	chr9	1.05E+08	1.05E+08	+	2431	1	SLC44A1|	2.27	6.119
NR_10348	chr17	4704230	4705529	+	470	1	LOC10155	0.713	0.525
NR_00297	chr12	1.32E+08	1.32E+08	+	136	1	SNORA49|	0	0
NM_0013	chr19	7476875	7489400	-	1039	1	PEX11G|-|	1.539	1.028
NM_0010	chr11	56462469	56463401	-	931	1	OR5M9|O	0	0
NM_0016	chr12	7649400	7665903	-	882	1	APOBEC1|	0	0
NM_0033	chr1	2.28E+08	2.28E+08	-	1234	1	WNT9A|W	1.776	0.788
NM_0010	chr12	53180751	53190870	+	4247	1	ZNF740|Z	18.036	22.863
NM_0031	chr8	1.25E+08	1.25E+08	+	2976	1	SQLE|-|-|	81.581	66.06
NM_1527	chr6	43645030	43671011	+	955	1	RSPH9|C6	10.828	1.664
NR_03896	chr7	28979967	29013369	+	3704	1	LOC10050	0	0.018
NM_0068	chr5	1.38E+08	1.38E+08	-	2964	1	HNRNPA0	137.03	137.368
NM_0011	chr17	35474923	35487739	-	1762	1	SLFN12L|-	0	0
NR_04983	chr16	29599179	29599265	-	86	2	MIR3680-2	0	0
NM_0220	chr3	49689499	49721529	+	4272	1	RNF123|F	16.681	21.309
NM_0011	chr1	32862268	32870813	-	2075	1	FNDC5|FR	38.792	20.503
NR_03661	chr12	16988747	16990628	+	1881	1	SKP1P2|S	11.886	13.005
NM_1826	chrY	13297509	13480670	-	4895	1	UTY|KDM	2.165	3.025
NR_14445	chr13	74412957	74419115	-	2326	1	LOC10028	0.011	0.012
NM_0004	chr20	3082556	3084724	-	614	1	AVP|ADH|	0	0
NM_0011	chr1	18631006	18748866	+	6042	1	PAX7|HUP	3.216	3.514
NR_00366	chr19	23763014	23828117	+	1375	1	RPSAP58|	56.347	57.468
NR_00321	chr14	1.01E+08	1.01E+08	+	70	1	SNORD114	0	0
NM_0246	chr17	30898983	30906268	-	1333	1	TEFM|C17	3.975	3.268
NM_0012	chr6	36366194	36387800	-	1427	1	ETV7|TEL-	0	0.019
NR_02667	chr1	1.56E+08	1.56E+08	+	2222	1	TMEM79|	3.528	2.977
NR_12055	chr11	1.06E+08	1.06E+08	+	1749	1	LOC10192	0	0

NM_1781	chrX	1.5E+08	1.5E+08	+	2419	1	CXorf40A|	5.261	5.555
NR_00245	chr16	89561430	89561501	+	71	1	SNORD68|	9.806	6.945
NM_0011	chr7	1E+08	1E+08	+	1740	1	MEPCE|BC	22.052	21.396
NR_04666	chr3	1.87E+08	1.87E+08	-	3439	1	ADIPOQ-A	0	0
NM_0061	chr2	2.04E+08	2.04E+08	+	4879	1	CD28|Tp4	0.016	0.014
NM_0013	chr16	14632811	14669236	+	2660	2	BFAR|BAR	0	0
NM_0011	chr3	52812990	52830701	-	3169	1	ITIH4|GP1	1.335	1.344
NM_0207	chr21	41986831	42010387	-	7410	1	ZBTB21|ZN	2.457	2.782
NM_0123	chr10	988409	1017768	+	2498	1	GTPBP4|C	18.194	19.312
NM_0025	chr12	96278261	96400588	-	4166	1	CDK17|PC	9.876	12.388
NM_0013	chr12	82687155	82965971	+	2520	1	TMTC2|IB	8.8	8.995
NM_0203	chr4	1.25E+08	1.25E+08	-	8790	1	ANKRD50|	7.812	7.039
NM_0010	chr11	50032874	50033794	+	919	1	OR4C45|-|	0	0
NM_0156	chr4	1.28E+08	1.28E+08	+	3257	1	INTU|INT|	2.126	4.109
NM_0155	chr10	11920022	12042841	-	5176	1	UPF2|HUP	2.91	4.986
NM_0010	chr17	7315632	7329319	-	5150	1	NEURL4|-|	21.105	22.282
NM_1383	chr11	1.12E+08	1.12E+08	-	3059	1	FDXACB1|	2.251	2.982
NR_03041	chrX	1.2E+08	1.2E+08	-	110	1	MIR766|M	0	0
NR_00305	chr9	35657751	35658018	-	267	1	RMRP|CHI	1.835	2.36
NM_0062	chr6_GL0C	3254761	3273175	-	3773.7	7	VARS|G7A	0	0
NM_0016	chr20	34260365	34269342	+	579	1	ASIP|AGS\	0.223	0.095
NR_11074	chr6	57114911	57172213	-	686	1	LOC10192	0.075	0.04
NR_00320	chr14	1.01E+08	1.01E+08	+	73	1	SNORD114	0	0
NR_03653	chr13	99196374	99200757	-	1819	1	UBAC2-AS	1.467	1.212
NR_02713	chr15	95433093	95507847	+	2591	1	LINC00924	0	0.053
NM_0011	chr8	1.44E+08	1.44E+08	+	2107	2	ZNF707|-|	0	0
NM_0331	chr17	40993430	40994133	-	701	2	KRTAP3-3|	0	0
NM_0004	chr5	1.5E+08	1.5E+08	-	5618	1	PDE6A|CG	0	0.012
NM_1788	chr10	1.03E+08	1.03E+08	+	2548	1	SFXN2|-|-	2.525	1.978
NM_0003	chrX	1.06E+08	1.06E+08	-	1589	1	SERPINA7|	0	0
NM_0011	chr8	42541155	42552997	+	756	1	SMIM19|C	28.465	26.289
NM_0245	chr2	27625639	27629031	-	2357	1	CCDC121|	0.651	0.726
NM_0013	chrX	1.37E+08	1.37E+08	-	4786	1	ARHGEF6|	1.258	5.057
NR_10355	chr8	11761256	11763223	+	1967	1	C8orf49|G	0	0
NR_02695	chr11	43897303	43899874	+	2571	1	SEC14L1P1	1.876	1.641
NR_04008	chr15	25902360	26053120	+	1962	1	LOC10012	0.046	0.14
NR_13519	chr16	85792415	85792933	+	406	1	LOC10192	0	0.067
NM_1526	chr3	57625454	57693089	-	4636	1	DENND6A	4.105	4.627
NM_0063	chr7	80742538	80919351	-	5154	1	SEMA3C|S	7.925	7.117
NR_13277	chr8	11705362	11705558	+	196	1	SNORA99|	0	0
NR_02447	chr10	45099476	45154596	-	1835	1	RSU1P2|-|	0	0
NM_0012	chr6	25912756	25925879	-	1956	1	SLC17A2|N	0.013	0.028
NM_0052	chr16	1984149	1987749	+	2404	1	GFER|ALR	20.745	22.961
NM_1447	chr3	1.37E+08	1.37E+08	+	2037	1	IL20RB|DII	0.968	1.036
NR_00439	chr7	1.49E+08	1.49E+08	+	95	1	RNY4|HY4	0	0
NM_0010	chr21	46188446	46228824	-	2356.5	2	LSS|CTRCT	12.229	9.131
NM_0150	chr5	1.8E+08	1.8E+08	-	5091	1	TBC1D9B|	39.205	36.98
NM_0010	chrY	22984263	23005463	+	1193.7	3	BPY2C|VC	0	0
NM_1452	chr16	29812088	29815881	+	2561	1	PRRT2|BFI	86.005	67.811
NM_0067	chr16	69187147	69309052	+	9762	1	SNTB2|D1	0.831	0.929

NR_10466	chr5	58107632	58122341	-	1170	1	LINC02101	0	0
NR_11003	chr3	1.08E+08	1.08E+08	+	2374	1	LINC01215	0.022	0.006
NM_0123	chr18	62044225	62187056	-	4766	1	PIGN|MCA	1.913	2.167
NM_0050	chr17	36013056	36017968	-	620	2	CCL23|CK-	0	0
NM_0146	chr6	43635840	43640951	+	1274	1	MAD2L1BI	32.284	32.995
NR_03021	chr19	53739333	53739419	+	86	1	MIR518A2	0	0
NM_1392	chr11	60334882	60340968	+	654	1	MS4A6E|-	0.591	0.042
NR_03763	chr1	38862490	38881626	-	2667	1	GJA9-MYC	2.48	1.474
NM_1732	chr18	3453774	3458408	+	1349	1	TGIF1|HPE	22.155	20.429
NR_02688	chr19	43349056	43349548	+	492	1	PRG1|-|-|	0	0
NM_1524	chr1	2.1E+08	2.1E+08	-	4851	1	C1orf74|U	1.661	2.098
NM_0010	chr11	56360215	56361288	+	1071	2	OR8J1|OR	0	0
NR_02337	chr1	2.29E+08	2.29E+08	-	120	10	RNA5S8|R	0	0
NR_03965	chr4	86542482	86542552	-	70	1	MIR4452|-	0	0
NR_13466	chr4	88523810	88524996	+	362	1	LOC10192	0.249	0.076
NR_10704	chr13	1.1E+08	1.1E+08	+	71	1	MIR8073|	0	0
NM_0010	chr11	55603354	55604398	-	1042	1	OR4C11|C	0	0
NR_02460	chr2	74502617	74505065	+	2448	1	LBX2-AS1|	0.263	0.19
NM_1985	chr12	40225011	40369284	+	9171	1	LRRK2|AU	0.022	0.022
NM_1779	chr20	59958416	60013113	+	3173	1	CDH26|VR	0	0.065
NM_0247	chr2	2.19E+08	2.19E+08	-	2094	1	NHEJ1|XLF	10.88	9.282
NM_0164	chr14	70325081	70359731	-	1711	1	COX16|C1	39.404	41.387
NR_04619	chr21	24840550	25057743	+	2712	1	LINC01692	0	0
NM_0010	chr2	87010465	87021846	-	3005	2	PLGLB1|PL	0.09	0.155
NR_00357	chr9	33624225	33625534	+	1309	1	ANXA2P2|	1.586	0.712
NM_0012	chr12	1.17E+08	1.17E+08	-	10740	1	NOS1|IHPS	0.301	0.038
NM_0132	chr13	43023226	43109170	+	2768	1	DNAJC15|	0.531	0.906
NR_00218	chr1	1.55E+08	1.55E+08	-	2024	2	GBAP1|GE	0.338	0.42
NR_00292	chr1	1.67E+08	1.67E+08	+	1355	1	FMO9P|-|	0	0
NR_13279	chr10	75712455	75743848	+	919	1	LOC10537	0	0
NM_0067	chr19	45467995	45475179	+	3770	1	FOSB|AP-1	0.571	1.871
NM_0011	chr19	32719773	32790808	+	1602	1	TDRD12|E	0.089	0.12
NR_10705	chr11	27514970	27515047	-	77	1	MIR8087|	0	0
NM_0164	chrX	70286361	70289948	-	1237	1	PDZD11|A	42.162	40.46
NM_0011	chrX	1.55E+08	1.55E+08	+	1952	1	IKBKG|AM	1.783	2.337
NM_0147	chr12	7130371	7158934	+	3982	1	CLSTN3|CI	67.135	84.731
NM_0010	chr1	1.51E+08	1.51E+08	-	3598	1	RFX5|-|-|	19.222	22.099
NM_0007	chr8	66176377	66178645	-	1464	1	CRH|CRF|	2.88	0.346
NR_00216	chrY	17575185	17579473	-	456	2	FAM224A|	0	0
NM_0013	chr17	35148264	35189345	+	5320	1	UNC45B|C	0	0
NM_0013	chrX	18506935	18635632	+	9901	1	CDKL5|CF	2.071	5.466
NR_02694	chr15	69080850	69095823	+	2522	1	EWSAT1|L	0.133	0.842
NM_0189	chr7	27170591	27174336	-	2567	1	HOXA10|H	0.005	0
NM_1816	chr21	30440328	30440780	+	450	1	KRTAP15-1	0	0
NR_13078	chr5	1.14E+08	1.15E+08	-	2020	1	LOC10192	0.268	0.19
NR_00301	chr12	6581473	6581609	-	136	1	SCARNA11	0.948	0.101
NR_02334	chr2	1.22E+08	1.22E+08	+	129	1	RNU4ATA(	1.099	0.319
NM_0011	chr2	86441461	86492716	+	4593	1	KDM3A|JH	26.736	18.865
NR_03837	chr16	25031741	25032777	+	1036	1	LOC55420	1.381	1.507
NM_1991	chr7	45100100	45111747	-	1914	1	TBRG4|CP	26.562	26.292

NR_13560	chr3	12484432	12539624	+	2004	1	TSEN2|PCI	6.588	6.083
NM_0013(	chr19	1026275	1039068	+	2443	1	CNN2|-|-|	90.254	93.238
NR_03404	chr17	58000919	58007346	-	4718	1	SRSF1|ASF	44.854	35.784
NM_0154:	chr16	8795180	8797648	-	1444	1	TMEM186	10.206	11.335
NM_0013:	chr17	19737985	19748433	-	1599	1	ALDH3A1|	0	0.051
NM_0007:	chr1	2.07E+08	2.07E+08	+	2229	1	C4BPA|C4	0.023	0
NR_02729	chr21	45070956	45073211	-	2255	1	SSR4P1|C2	0.543	0.656
NR_12536	chr15	75023555	75050726	+	2038	1	PPCDC|MI	3.239	1.721
NM_1721!	chr14	19747428	19748369	+	940	1	OR4Q3|C1	0	0
NR_03393	chr5	98769618	98773084	-	2028	1	RGMB-AS1	12.27	7.74
NM_0159:	chr15	63153340	63157542	-	1078	1	RPS27L|-|	48.919	36.707
NM_0069⁄	chr1	1.53E+08	1.53E+08	-	678	1	SPRR2D|-|	0	0
NR_10373	chr12	1.31E+08	1.31E+08	+	1790	1	LOC33879	0.014	0.069
NM_0178!	chr1	37681570	37690595	-	2423	1	C1orf109|	6.226	6.546
NM_0140⁄	chr3	1.29E+08	1.29E+08	+	2555	1	ACAD9|NF	14.438	15.723
NM_0011!	chr8	90958478	90985257	-	1045	1	C8orf88|-|	0.777	1.783
NR_10996	chr21	44414593	44425563	-	869	1	TRPM2-AS	0	0
NM_0012!	chr3	62318973	62324133	+	617	1	C3orf14|H	4.138	7.548
NM_0143(	chr19	35533412	35545319	+	1479	1	GAPDHS|G	1.656	2.047
NM_0010{	chr10	58513144	58831437	+	5448	1	BICC1|BIC	0.814	1.415
NM_0010:	chr2	2.31E+08	2.31E+08	+	1942	1	ITM2C|BR	265.691	222.961
NR_12573	chr20	50184603	50192069	-	2966	1	CEBPB-AS1	0.83	1.676
NR_04004	chr8	6403556	6406548	-	2406	1	LOC10028	4.48	5.801
NM_0063(	chr19	44002925	44013920	+	4125	1	ZNF230|FI	1.416	1.783
NR_00319	chr14	1.01E+08	1.01E+08	+	70	1	SNORD114	0	0
NR_03833	chr19	42132629	42137099	+	682	1	LOC10050	0	0.02
NR_03387	chr2	19868854	19885047	+	3005	1	LINC00954	0.009	0.014
NM_0010:	chrX	1.55E+08	1.55E+08	+	2841	1	BRCC3|BR	2.623	2.777
NM_0033:	chr2	3188924	3377932	-	1806	1	TSSC1|EIP	37.688	39.779
NM_0529⁄	chr1	89181148	89198950	-	6132	1	GBP4|Mpa	0	0.027
NM_0073(	chr3	1.97E+08	1.97E+08	-	2164	1	NCBP2|CB	44.829	43.154
NM_0010:	chr8	27774541	27804907	+	3345	1	ESCO2|24	0.27	0.721
NM_0057:	chr5	1.33E+08	1.33E+08	+	6570	1	RAD50|NE	1.081	2.32
NR_03418	chr1	1.55E+08	1.55E+08	+	1082	1	POU5F1P4	0	0
NM_0211:	chr7	44796636	44803123	+	2281	1	PPIA|CYPA	56.022	50.218
NM_0247(	chr3	1.29E+08	1.29E+08	+	2681	1	EFCC1|C3c	5.877	4.261
NM_1735:	chr14	67189393	67228550	+	1779	1	FAM71D|(	0.022	0.062
NR_03971	chr12	97939056	97939121	-	65	1	MIR4495|	0	0
NM_0071:	chr2	2.18E+08	2.18E+08	+	2716	1	VIL1|D2S1	0	0
NR_00330	chr15	25185631	25185712	+	80	3	SNORD115	0	0.171
NM_0012{	chr19	1071221	1087831	+	4929	1	ARHGAP45	6.244	6.019
NM_0247(	chr1	2.21E+08	2.21E+08	+	2968	1	C1orf115|	3.058	7.199
NM_0010:	chr20	33490122	33650031	+	7306	1	CBFA2T2|I	19.822	24.443
NM_0011:	chr12	389350	442640	+	2273	1	CCDC77|-|	6.45	7.026
NM_0044(	chr1	10460546	10472556	-	2030	1	DFFA|DFF-	13.351	12.368
NM_0013:	chr17	10713295	10730329	-	3023	1	TMEM220	0.154	0.399
NM_1788:	chr19	39024021	39032596	-	2354	1	FBXO27|FI	1.046	0.96
NM_1732:	chr16	69565966	69695688	+	5295	1	NFAT5|NF	2.084	3.704
NR_03409	chr1	93591968	93600030	+	1558	1	LOC10012!	0.621	0.73
NR_13529	chr7	43117896	43163187	+	1832	1	HECW1-IT:	0	0.142

NM_1781:	chr11	1.02E+08	1.02E+08	-	2357	1	ANGPTL5|	0.016	0.006
NM_0012!	chr17	81637171	81648755	+	1865	1	TSPAN10|(	1.362	1.043
NR_10984	chr1	1.01E+08	1.01E+08	+	1321	1	LOC10260(	2.87	4.396
NR_11065	chr10	90402521	90540805	-	974	1	LOC10192(	0	0
NR_12211	chr22	15784954	15827434	+	2099	1	DUXAP8|-	0	0
NM_1832:	chr3	1.42E+08	1.42E+08	+	1989	1	RNF7|CKB	40.534	33.488
NR_10694	chr12	1.24E+08	1.24E+08	-	61	1	MIR6880|l	3.593	5.614
NM_0225:	chr14	59463522	59465341	-	1817	1	GPR135|H	0.773	0.678
NM_0010(	chr11	55967558	55968463	-	904	1	OR10AG1|	0	0
NM_1944:	chr6	1.67E+08	1.67E+08	+	3662	1	FGFR1OP|	3.7	2.944
NM_0013(	chr5	10249921	10266412	+	3262	1	CCT5|CCT-	83.144	81.061
NR_00335	chr15	25237986	25238067	+	81	1	SNORD115	0	0
NM_1307!	chr1	1.92E+08	1.92E+08	+	2138	1	RGS18|RG	0	0
NM_0010(	chr11	65170236	65173217	+	874	1	SPDYC|RIN	0	0
NM_0204:	chr8	41653225	41797622	-	8113	1	ANK1|ANK	3.294	3.282
NR_10698	chr2	6650373	6650439	+	66	1	MIR7515|l	0	0
NM_0011(	chr6	1.68E+08	1.69E+08	+	3102	1	SMOC2|D1	0.503	0.654
NM_0013:	chr17	7189891	7205168	-	3098	1	DLG4|PSD	130.425	125.064
NM_0022!	chr12	52314301	52321398	-	1864	1	KRT83|HB	0	0
NM_0329(	chrX	91835461	92623229	+	8368	1	PCDH11X|	0.177	0.137
NR_11017	chr2	1.14E+08	1.14E+08	-	585	1	LOC10106(	1.873	1.569
NM_0010:	chr1	2.28E+08	2.28E+08	+	3127	1	RNF187|R/	111.844	143.803
NM_0124:	chr17	81528391	81537130	+	1663	1	FSCN2|RFS	2.969	2.487
NR_00233	chr7	1.17E+08	1.17E+08	-	1887	1	ST7-AS1|S1	2.836	6.359
NM_0042<	chr15	43232595	43266857	-	2507	1	TGM5|PSS	0	0.044
NR_03793	chr2	86861825	86895042	+	1756	1	ANAPC1P1	0	0
NM_1810:	chr15	34379069	34389800	-	4245	1	GOLGA8A|	1.977	3.546
NM_0048:	chr11	1.26E+08	1.26E+08	+	2273	1	EI24|EPG4	62.578	76.005
NR_13551	chrUn_KI2	41866	59598	-	859	2	LOC10537!	0	0
NR_13232	chr6_GL0C	1054889	1060560	+	1274.1	8	LOC55422.	0	0
NM_0146(	chr10	1.25E+08	1.25E+08	-	5733	1	FAM53B|K	12.923	19.185
NR_04748	chr13	99994900	99997909	-	3009	1	LINC00554	0	0
NM_0216:	chr9	1.31E+08	1.31E+08	+	2485	1	PRDM12|H	0.223	0.298
NR_11013	chr14	90642629	90648888	+	1237	1	LOC10192:	0.042	0.166
NM_0010:	chr5	82279462	82318646	+	2334	1	ATP6AP1L	3.657	4.208
NM_0067:	chr2	2.37E+08	2.37E+08	+	2248	1	COPS8|CO	33.163	37.99
NR_00128	chr5	1.41E+08	1.41E+08	+	3196	1	PCDHB18P	8.928	4.539
NM_0014:	chrX	20124518	20141848	-	4422	1	EIF1AX|EIF	6.633	5.297
NM_0011(	chr2	1.2E+08	1.2E+08	+	1342	1	TMEM177	10.098	15.34
NR_01539	chr3	1.17E+08	1.17E+08	+	2098	1	TUSC7|LIN	0	0.013
NM_0806<	chr22	19756703	19767332	+	2071	1	TBX1|CAFS	0.268	0.549
NR_03845	chr3	1.43E+08	1.43E+08	+	1822	1	LOC10050:	0	0
NM_0328!	chr1	34166883	34219130	+	2126	1	C1orf94|-|	0.006	0.103
NM_0012<	chr16	58500143	58513619	+	3153	1	NDRG4|BD	146.232	109.064
NR_12061	chr10	79691500	79826594	-	1306	1	NUTM2B-/	5.617	6.429
NM_0010:	chr17	20155994	20314759	+	3846	1	SPECC1|CY	8.582	10.072
NM_0012!	chr3	53283425	53347627	-	5875	1	DCP1A|HS	9.404	9.687
NM_0068:	chr19	1597155	1605484	-	1330	1	UQCR11|C	105.93	84.026
NR_11074	chr19	34923015	34926826	-	425	1	ZNF30-AS1	0.273	0.193
NM_0013:	chr3	9948581	9952415	-	1494	1	PRRT3|-|-	8.181	8.132

NM_0159	chr11	34882296	34916411	-	1268	1	APIP|APIP	6.477	8.318
NR_00332	chr15	25070431	25070527	+	96	1	SNORD11	0	0
NR_03620	chr18	6374361	6374425	-	64	1	MIR4317|	0	0
NM_1817	chr9	1.36E+08	1.36E+08	-	4512	1	QSOX2|Q	10.327	12.938
NM_0012	chr3	40524878	40539794	+	2396	1	ZNF621|-|	3.67	3.144
NR_03992	chr2	87194786	87194859	+	73	2	MIR4771-	0	0
NM_0124	chr7	76326791	76359025	-	3743	1	YWHAG|1	257.008	315.833
NR_03164	chr5	1.11E+08	1.11E+08	-	82.5	2	MIR548F3	0	0
NR_13502	chr12	56010092	56025091	-	380	1	LOC10536	1.154	1.262
NM_0011	chr22	21015153	21028013	+	2652	1	P2RX6|P2	1.284	0.733
NM_1528	chr7	19144782	19145421	-	637	1	FERD3L|N	0.344	0.43
NM_0012	chr8	73241424	73259502	-	557	1	C8orf89|-|	0.301	0.074
NR_03988	chr17	58335976	58336022	-	46	1	MIR4736|	1.682	1.787
NM_0057	chr15	38488101	38564806	-	5006	1	RASGRP1|	0.474	5.311
NM_0141	chr19	35154722	35169885	+	884	1	FXYD5|DY	0.219	0.356
NM_0161	chr1	2.28E+08	2.28E+08	-	2076	1	TRIM17|R	0.149	0.33
NR_12602	chr7	3140931	3158573	-	555	1	LOC10001	0.046	0
NM_0309	chr9	86264548	86282575	-	1996	1	ISCA1|HBL	19.799	21.081
NR_02437	chr12	9283657	9314088	+	3477	1	LOC64284	0.3	0.138
NM_0162	chr3	1.38E+08	1.38E+08	-	2680	1	DBR1|-|-|	5.961	6.772
NM_0183	chr7	1.28E+08	1.29E+08	+	2171	1	METTL2B|	9.027	10.965
NR_04750	chr13	29918647	29926651	-	573	1	LINC00572	0	0
NM_0010	chr2	85605253	85612056	-	4259	1	C2orf68|H	11.247	12.989
NR_10976	chr9	68543541	68546602	-	347	1	LINC0150	0	0
NM_0182	chr3	88139112	88144664	+	4953	1	ZNF654|-|	1.145	1.441
NM_0163	chr2	73700509	73701340	-	829	1	NAT8B|C	0.062	0.05
NM_0013	chr18	46476972	46556769	-	3609	1	LOXHD1|	0	0.023
NR_02446	chr13	30340266	30373914	-	1603	1	LINC0042	0.032	0.009
NM_0006	chr11	6431038	6441024	-	1611	1	HPX|HX|-|	1.553	1.105
NM_0009	chr9	1.33E+08	1.33E+08	+	880	1	RPL7A|L7	261.767	260.617
NM_0012	chr1	27355179	27366866	-	4294	1	MAP3K6|	3.501	2.893
NM_0013	chr17	18087867	18107985	+	1982	1	DRG2|-|-|	20.257	19.778
NR_10675	chr1	51060018	51060103	+	85	1	MIR6500|	0	0
NM_0011	chr1	21509358	21578412	+	2310	1	ALPL|AP-T	2.841	5.894
NR_03403	chr7	1.5E+08	1.5E+08	+	2039	1	LINC0099	0	0
NR_02778	chr22	42500579	42512560	+	1346	1	SERHL|BK	4.167	4.6
NM_0012	chr4	48986642	49062078	+	2371	1	CWH43|C	0	0.023
NM_0016	chr16	70252394	70289509	-	3321	1	AARS|CM	83.728	116.835
NR_03994	chr2	2.39E+08	2.39E+08	-	80	1	MIR2467|	0.322	0
NM_0305	chr19	41354417	41364173	-	1025	1	B9D2|ICIS	7.12	4.249
NR_03895	chr22	45133758	45163781	-	1335	1	NUP50-AS	9.668	16.99
NM_0155	chr1	1.57E+08	1.57E+08	-	2071	1	GPATCH4|	4.831	5.655
NM_0154	chr12	1.32E+08	1.32E+08	+	12262	1	EP400|CA	12.478	11.664
NR_12033	chr15	47803384	47846236	-	434	1	LINC01491	0	0
NM_0013	chr1	1E+08	1E+08	+	2061	1	CDC14A|D	0.82	1.023
NM_0010	chr9	26066675	26118408	+	2311	1	LOC10050	0	0
NR_02435	chr20	31485778	31487574	+	583	1	LINC0002	0	0
NM_0013	chr2	1.76E+08	1.76E+08	-	7367	1	LNPK|KIA	10.519	3.96
NR_04638	chr3	1.26E+08	1.26E+08	+	2321	1	ALDH1L1-	0.011	0.118
NM_0010	chr2	56184123	56386174	+	3975	1	CCDC85A|	0.629	0.531

NM_0013:	chr7	75996337	76030524	-	919	1	STYXL1|DU	33.882	30.539
NM_0006	chr19	55364382	55370463	-	2371	1	IL11|AGIF	1.218	2.08
NM_0011(	chr17	1716523	1738599	+	3172	2	WDR81|C/	0.301	0.479
NM_0151!	chr15	80975745	80989878	-	4261	1	MESDC2|E	11.617	10.518
NR_03626	chr1	30366	30503	+	137	4	MIR1302-1	0	0
NM_0034:	chr19	58432814	58440222	-	3026	1	ZNF132|pI	1.031	0.67
NM_0164	chr6	1.07E+08	1.07E+08	+	1179	1	C6orf203|	14.457	16.23
NM_0217	chr11	1.19E+08	1.19E+08	+	3235	1	VPS11|ENI	17.577	21.695
NM_0011	chr5	1.78E+08	1.78E+08	-	2104	1	DOK3|DOI	3.033	3.75
NM_0240	chr1	1.51E+08	1.51E+08	+	2027	1	SCNM1|-|	23.287	19.86
NM_1818	chr6_GL0C	3241692	3244067	-	1861.4	5	ZBTB12|Ba	0	0
NM_0013	chr17	74560701	74567343	-	538	1	LOC10013(	0	0
NM_0013	chr17_JH1	246640	247812	-	1170	1	KRTAP4-8|	0	0
NM_0016!	chr12	49972837	49977139	+	2648	1	AQP6|AQI	0.2	0.114
NR_03400	chrX	71697196	71703406	+	1061	2	LOC10013	0	0
NM_0184	chr8	1.3E+08	1.3E+08	-	6212	1	ASAP1|AN	6.181	12.242
NM_0011	chr7	1.03E+08	1.03E+08	-	2784	1	FBXL13|DI	0.31	0.133
NM_0012!	chrX	47561100	47571921	+	2487	1	ARAF|A-R/	9.83	10.971
NM_1992	chr17	75827226	75844717	-	4358	1	UNC13D|F	0.308	0.427
NR_11938	chr21	42009194	42024920	+	1004	1	ZNF295-AS	0.385	0.3
NM_1525!	chr15	39600031	39782838	-	2799	1	FSIP1|HSD	0.944	0.455
NM_0000!	chr20	23633657	23638048	-	881	1	CST3|ARN	76.366	51.71
NM_0029	chr17	1829979	1899554	+	4326	1	RPA1|HSSI	23.522	23.313
NR_12650	chr3	10285750	10292947	-	249	1	GHRL|MTL	0.518	0.165
NM_0012!	chr4	56530609	56603323	+	1456	1	THEGL|-|-	0	0.019
NM_0012	chr6	35737032	35748913	+	1135	1	ARMC12|(	2.181	2.522
NM_0023:	chr19	33194693	33208867	+	3689	1	LRP3|-|-|1	38.6	40.738
NM_0121	chr7	1.01E+08	1.01E+08	+	2131	1	FBXO24|FI	1.271	0.431
NM_0152	chr1	43389885	43454247	+	13906	1	SZT2|C1or	6.272	5.868
NM_0064!	chr17	76565379	76586063	-	2094	1	ST6GALNA	0.209	0.183
NM_0002(	chr4	986997	1004557	+	2198	1	IDUA|IDA|	2.012	2.935
NM_0163	chr10	75094434	75099490	-	896	1	DUSP13|B	0.115	0.122
NM_0011:	chr12	56230717	56237845	+	1952	1	SLC39A5|L	0.185	0.126
NM_0045:	chr2	2.3E+08	2.3E+08	-	2065	1	SP110|IFI4	0.137	0.431
NR_03786	chr6_GL0C	3459000	3473266	+	2512.2	8	PPT2-EGFL	0.169	0.076
NM_0012	chr15	74836116	74843211	-	2542	1	ULK3|-|-|1	19.7	21.278
NM_1829	chr5	1.81E+08	1.81E+08	-	5825	1	FLT4|FLT-4	0.255	0.183
NM_0332!	chr19	46634076	46634682	-	210	1	GNG8|-|-|	32.05	23.154
NM_1813!	chr1	1.54E+08	1.54E+08	+	5809	1	IL6R|CD12	0.082	0.137
NM_0012	chr3	1.01E+08	1.02E+08	-	810	1	SENP7|-|-	6.383	5.191
NM_0012	chr15	20835372	20866314	-	1674	2	POTEB|A2	0	0
NM_0022	chr19	17951302	17999121	+	2644	1	KCNN1|KC	28.601	30.383
NM_0010(	chr10	1.04E+08	1.04E+08	+	1426	1	SFR1|C10c	2.785	2.344
NM_0209	chr1	64470793	64693058	+	5245	1	CACHD1|-	28.835	24.37
NM_0012	chr10	32267716	32347048	-	3682	1	EPC1|Epl1	16.336	23.12
NM_0029	chr22	50252901	50261810	-	1891	1	MAPK12|E	10.705	8.989
NM_0806:	chr20	55997357	56005472	-	3102	1	CBLN4|CB	0	0
NR_12648	chr15	70615547	70616567	+	1020	1	SALRNA3|:	0	0
NM_0002	chr14	20469379	20478006	+	2413	1	PNP|NP|P	7.839	7.05
NM_0047:	chr17	7921859	7929435	-	2443	1	KCNAB3|A	2.665	2.848

NR_10987	chr5	1.52E+08	1.52E+08	+	1965	1	CTB-113P1	0.243	0.063
NM_1527[illegible]	chr7	47987149	48029119	-	1339	1	SUN3|SUN	0	0
NM_0011[illegible]	chr1	40040583	40072649	+	2740	1	CAP1|CAP	86.975	81.192
NM_0011[illegible]	chr3	1.28E+08	1.28E+08	-	1933	1	TPRA1|GP	11.239	14.243
NR_00002	chr22	39313819	39313911	-	92	1	SNORD83E	3.924	2.68
NM_0006[illegible]	chr14	94581369	94593120	+	2329	1	SERPINA5|	0.448	0.024
NM_2033[illegible]	chrX	30653359	30731460	+	4468	1	GK|GK1|G	0.349	0.159
NM_0012[illegible]	chr15	72686179	72738476	+	2434	1	BBS4|-|-|[illegible]	24.06	19.313
NR_03383	chr15	80263068	80341805	-	2912	1	LINC00927	0	0
NM_0012[illegible]	chr17	7380974	7389773	+	2907	1	TNK1|-|-|[illegible]	1.211	0.768
NM_0031[illegible]	chr1	2.26E+08	2.26E+08	+	1491	1	SRP9|ALU	52.508	51.417
NM_0011[illegible]	chr7	16526880	16581489	+	927	1	LRRC72|-|	0	0
NM_0020[illegible]	chr9	1.35E+08	1.35E+08	-	7664	1	FCN1|FCN	0	0.002
NM_0054[illegible]	chr10	1.11E+08	1.11E+08	+	4081	1	SMC3|BAN	8.742	11.834
NM_0528[illegible]	chr9	1.29E+08	1.29E+08	-	1807	1	WDR34|D	40.693	23.725
NM_0013[illegible]	chr5	23507655	23528084	+	3125	1	PRDM9|K	0	0
NM_0246[illegible]	chr8	95133721	95156685	+	2961	1	PLEKHF2|E	0.732	1.503
NM_0011[illegible]	chr19	1876976	1885519	-	1556	1	ABHD17A|	3.944	4.621
NM_1818[illegible]	chr5	1.16E+08	1.16E+08	-	4058	1	TMED7|C	7.451	6.686
NM_0012[illegible]	chr4	55595229	55636298	-	759	1	NMU|-|-|[illegible]	6.404	4.945
NM_0035[illegible]	chr6	26216201	26216644	-	441	1	HIST1H2B	4.941	4.007
NR_03385	chr8	93346467	93700433	-	1903	1	LINC00535	0.684	0.691
NM_1537[illegible]	chr4	1.41E+08	1.41E+08	+	4398	1	ELMOD2|[illegible]	5.553	4.105
NM_0010[illegible]	chr9	1.25E+08	1.26E+08	-	3381	1	MAPKAP1	34.238	36.501
NM_0010[illegible]	chr1	13474977	13513747	-	1904	1	LRRC38|-|	0.19	0
NR_11055	chr14	64514151	64540368	-	557	1	LOC10272	0.162	0.246
NR_11061	chr1	2.23E+08	2.23E+08	+	658	1	TAF1A-AS1	7.524	7.473
NM_0066[illegible]	chr17	4997948	5028399	+	7892	1	KIF1C|LTX	4.432	4.193
NM_0010[illegible]	chr15	58771362	58861899	+	9175	1	FAM63B|-	2.164	1.345
NM_0326[illegible]	chr5	36152043	36184040	+	1470	1	SKP2|FBL1	7.894	5.749
NM_0250[illegible]	chr8	37695751	37700021	+	3345	1	ZNF703|N	0.802	6.068
NM_0010[illegible]	chr11	5884293	5885231	+	937	1	OR52E4|O	0	0
NM_0011[illegible]	chr4	1.73E+08	1.73E+08	-	1460	1	HMGB2|H	43.131	33.501
NM_1735[illegible]	chr19	58408674	58418325	+	2275	1	ZNF584|-|	14.322	12.114
NM_0012[illegible]	chr2	96642737	96704885	+	6412	1	FER1L5|-|[illegible]	0.549	0.229
NM_0012[illegible]	chr1	54007298	54018186	-	2103	1	LDLRAD1|[illegible]	0	0
NM_0011[illegible]	chr2	1.28E+08	1.28E+08	+	2301	1	GPR17|-|-	0.078	0.149
NM_1826[illegible]	chr10	1.14E+08	1.14E+08	+	2036	1	PLEKHS1|[illegible]	0	0
NM_0024[illegible]	chr17	63622441	63696310	+	4715	1	MAP3K3|[illegible]	7.263	6.841
NR_07353	chr18	12661956	12702777	-	2382	1	CEP76|C1[illegible]	2.744	3.749
NM_0230[illegible]	chr1	40450665	40463718	+	2024	1	ZFP69B|Z	2.363	4.128
NM_0203[illegible]	chr4	70688479	70690551	+	2070	1	UTP3|CRL[illegible]	9.742	10.58
NM_0004[illegible]	chr7	19115468	19117672	-	1662	1	TWIST1|A	0.295	0.412
NM_0133[illegible]	chr19	48455707	48466110	+	2846	1	KCNJ14|IR	0.879	0.717
NM_0184[illegible]	chr8	22114419	22131052	-	5331	1	HR|ALUN	0.058	0.355
NR_10989	chr5	1.63E+08	1.63E+08	-	1265	1	HMMR-AS	0.082	0.303
NR_00331	chr15	25056859	25056955	+	96	2	SNORD116	0	0.143
NM_0011[illegible]	chr7	1.4E+08	1.4E+08	-	15127	1	HIPK2|PR	13.828	11.341
NR_02769	chr21	38206156	38208644	+	857	1	DSCR10|-|	0	0
NM_0011[illegible]	chr12	29500813	29783810	-	8721	1	TMTC1|A	0.835	4.036

NR_02986	chr14	1.01E+08	1.01E+08	+	68	1	MIR377|M	0	0
NR_13618	chr3	12877522	12885211	-	632	1	LINC02022	0	0
NR_13243	chr3	27830886	27834136	-	2833	1	LINC01981	0	0
NR_12539	chr3	1.43E+08	1.43E+08	+	511	1	PAQR9-AS	0.833	2.037
NR_03987	chr16	76868936	76869019	+	83	1	MIR4719|-	0	0
NM_0219!	chrX	85243991	85273362	+	4156	1	ZNF711|CI	5.978	12.352
NR_03964	chr2	2.14E+08	2.14E+08	+	92	1	MIR4438|-	0	0
NM_0122!	chr8	79764010	79767863	-	2286	1	HEY1|BHLI	4.281	9.065
NM_0012/	chr12	2857681	2877155	-	3477	1	FOXM1|FK	10.772	6.496
NM_2071:	chr7	5620041	5781730	-	5660	1	RNF216|C/	14.67	17.852
NM_0181:	chr1	32205635	32208687	+	2046	1	IQCC|-|-|1	5.898	5.938
NM_0011!	chr7	1.49E+08	1.49E+08	+	4447	1	ZNF783|-|	4.822	7.423
NR_11054	chr14	1.05E+08	1.05E+08	+	897	1	LOC10272	0.287	0.26
NM_0013:	chr19	37469571	37488652	+	5159	1	ZNF570|-|	1.894	2.161
NM_0013:	chrX	49829260	49834264	+	771	1	PAGE4|CT	0.217	0
NM_0011(	chr10	13441088	13502976	-	1897	1	BEND7|C1	4.35	5.148
NM_0013:	chr2	2.01E+08	2.01E+08	+	2896	1	BZW1|BZA	19.923	18.138
NM_0013(	chr9	76611376	76692415	-	4432	1	PRUNE2|B	2.077	4.537
NM_0186!	chr12	12940775	12950384	-	1034	1	GPRC5D|-	0	0.013
NM_1387(	chr1	1914827	1917294	+	1093	1	CALML6|C	0.071	0.025
NR_13568	chr15	72608481	72636955	-	509	1	LOC10272	0.152	0.054
NM_0022/	chr1	2.34E+08	2.34E+08	+	2156	1	KCNK1|DP	0.239	0.273
NM_0013:	chr4	1.22E+08	1.22E+08	-	2024	1	TMEM155	0.076	0.081
NM_1526:	chr12	55681546	55684610	+	1501	1	METTL7B|	0	0.073
NM_0011/	chr16	15502266	15588259	+	2042.5	2	C16orf45|	1.446	1.75
NM_0002:	chr12	4909907	4918256	+	7979	1	KCNA1|AE	0.061	0.124
NR_00349	chr8	12182104	12194115	-	2166	1	FAM86B1|	1	1.069
NM_0012!	chr3	1.94E+08	1.94E+08	-	3022	1	CPN2|ACB	0	0.005
NM_1452:	chr1	33472631	33496395	+	4208	1	ZSCAN20|	2.938	3.346
NM_0010:	chr3	1.22E+08	1.22E+08	+	5058	1	ARGFX|-|-	0	0.003
NM_1383(	chr1	1.55E+08	1.55E+08	-	3175	1	PYGO2|11	25.742	23.175
NM_0045:	chr11	64789101	64803254	-	2970	1	MAP4K2|E	4.089	5.29
NM_0049:	chr11	17735948	17777830	+	6787	1	KCNC1|EP	5.239	8.823
NM_0151:	chr1	2.24E+08	2.24E+08	+	5444	1	FBXO28|C	3.335	3.787
NM_0013:	chr8	39584568	39730064	+	2306	2	ADAM18|/	0	0
NR_03605	chr2	2.32E+08	2.32E+08	+	81	4	MIR1244-1	10.506	14.712
NR_03416	chr11	1.19E+08	1.19E+08	+	1152	1	USP2-AS1|	0.168	0.202
NM_0047:	chr11	60850925	60855971	-	2906	1	PTGDR2|C	0.626	0.773
NM_0010/	chr20	25300198	25390982	-	2108	1	ABHD12|A	26.698	36.062
NM_0309:	chr13	25096138	25098566	+	2426	1	PABPC3|P.	4.289	5.313
NM_1979!	chr9	1.28E+08	1.28E+08	-	3394	1	NAIF1|C9c	4.543	5.125
NM_0012!	chr15	90871539	90883458	+	4173	1	FURIN|FUI	7.551	13.044
NM_0024!	chr8	89933336	89984671	-	4603	1	NBN|AT-V	2.496	3.943
NM_0011:	chrX	1.35E+08	1.35E+08	-	707	1	FAM127B|	80.005	44.966
NR_11080	chr17	49457861	49461749	-	1751	1	LINC02075	0	0
NR_00300	chrX	24744441	24744570	+	129	1	SCARNA23	0	0
NR_12633	chr16	72283301	72665009	-	1714	1	LINC01572	0.256	0.599
NR_00323	chr14	1.01E+08	1.01E+08	+	76	1	SNORD113	0	0
NM_1750:	chr12	52689626	52703463	-	3294	1	KRT77|K1I	0	0
NM_0033(	chr3	1.43E+08	1.43E+08	+	4054	1	TRPC1|HT	1.81	1.676

NM_0209	chr11	9019500	9091603	-	4512	1	SCUBE2|C	2.389	0.935
NR_02689	chr17	64749662	64781999	-	2849	1	ARHGAP27	4.087	2.721
NM_0013	chr5	69234795	69277430	+	1335	2	CDK7|CAK	0	0
NR_03394	chr14	97632647	97686658	-	1666	1	LOC10012	0	0.016
NM_0070	chr12	75497639	75511638	-	3345	1	KRR1|HRB	2.941	4.25
NR_04653	chr13	24252749	24254439	-	430	1	SPATA13-A	0	0
NR_00292	chr2	2.31E+08	2.31E+08	-	136	1	SNORA75|	0	0.302
NR_11024	chr2	1.51E+08	1.51E+08	-	1877	1	LOC10192	0	0
NM_0528	chr1	1.55E+08	1.55E+08	-	1484	2	SCAMP3|C	0.009	0.009
NR_00438	chr1	2.12E+08	2.12E+08	+	132	1	SNORA16E	0	0.208
NM_0055	chr20	62309060	62367312	-	11349	1	LAMA5|-|-	7.491	5.973
NM_0247	chr8	33547754	33567128	-	1864	1	RNF122|-|	37.601	53.12
NR_13554	chr3	1.67E+08	1.67E+08	+	253	1	LOC10537	0	0.108
NM_0151	chr22	46620402	46679791	+	4469	1	GRAMD4|	14.714	18.536
NR_12373	chr1	51329654	51331281	+	647	1	TTC39A-AS	0	0
NM_0010	chr1	12938472	12947580	-	1714	1	PRAMEF6|	0	0
NR_04986	chr8	74548543	74548617	+	74	1	MIR5681A	0	0
NM_0012	chr1	1.46E+08	1.46E+08	-	1887	1	PDZK1|CA	0.143	0.16
NM_0042	chr2	1.57E+08	1.57E+08	-	2200	1	CYTIP|B3-	0	0.274
NM_1383	chr11	278570	285388	+	2753	1	NLRP6|AV	0	0.01
NR_12643	chr4	4476121	4481700	-	538	1	STX18-IT1|	0	0.025
NR_02664	chr15	24558157	24587779	+	1429	1	PWRN1|N	0.054	0
NM_0142	chr15	78759203	78811463	-	5496	1	ADAMTS7	18.048	14.689
NM_1775	chr19	281040	291435	-	1265	1	PLPP2|LPP	1.977	2.869
NR_03028	chr1	1.55E+08	1.55E+08	-	95	1	MIR555|M	2.036	3.316
NM_0005	chr3	1.3E+08	1.3E+08	+	2761	1	RHO|CSNE	0.009	0
NR_13854	chr18	54324692	54354573	-	848	1	STARD6|-|	0.471	0.307
NM_0012	chr11	36591943	36598279	-	2621	1	RAG2|RAG	0.069	0.063
NM_0070	chr17_KI2	1729016	1752653	+	1503	2	DUSP14|M	0	0
NR_13509	chr11	92913239	92915628	-	1760	1	LOC10536	0	0
NR_00244	chr1	92840719	92840851	+	132	1	SNORA66|	0.977	0.623
NM_0012	chr5	76615482	76623413	-	3274	1	F2RL2|PAF	0.134	0.092
NM_0204	chr4	47485393	47593486	+	6525	1	ATP10D|A	2.211	4.874
NR_10354	chr7	1.55E+08	1.55E+08	-	2446	1	BLACE|-|-	0	0.017
NM_1985	chr1	77779624	77879540	+	6389	1	MIGA1|FA	1.175	1.503
NM_0011	chr11	1.26E+08	1.26E+08	+	2667	1	CHEK1|CH	3.268	3.528
NR_04645	chr2	2.08E+08	2.08E+08	+	489	1	IDH1-AS1|	1.767	1.597
NR_03392	chr16	52552087	52606975	-	997	1	CASC16|LI	0.013	0.151
NM_0060	chr7	1.01E+08	1.01E+08	+	4797	1	AGFG2|HF	1.989	1.727
NM_0011	chr1	1.52E+08	1.52E+08	-	1709	1	C2CD4D|F	0.098	0.272
NM_0210	chr12	14829311	14843479	-	1366	1	ART4|ART	0	0
NM_0326	chr1	32247217	32248860	+	952	1	FAM167B|	0.677	0.374
NR_04979	chr9	86948698	87002033	+	4381	1	GAS1RR|L	0.085	0.119
NM_0123	chr17	3420568	3421533	+	964	1	OR3A3|OF	0	0
NM_0012	chr7	1.07E+08	1.07E+08	+	2712	1	HBP1|-|-|	10.426	15.859
NR_12587	chr6	68632663	68634324	-	532	1	LOC10192	8.822	12.667
NM_0021	chr1	23557930	23559794	-	1234	1	ID3|HEIR-	51.031	6.76
NR_13504	chr12	30755074	30780739	+	1550	1	LOC64548	0.033	0.009
NM_0142	chr13	40789411	40812460	+	4012	1	SLC25A15|	6.183	5.606
NM_0026	chr2	68365190	68397453	+	2841	1	PLEK|P47|	0.095	0.043

NM_0011!	chr17	22523111	22524665	+	1552	1	MTRNR2L1	0.058	0.062
NM_0012!	chr16	27313909	27364778	+	3539	1	IL4R|CD12	0.638	0.31
NM_0807	chr6	89095959	89118081	-	3592	1	SRSF12|SF	4.587	4.595
NM_1737!	chrX	44523639	44542975	-	1163	1	FUNDC1|-	21.485	23.555
NM_0121	chr1	1.5E+08	1.5E+08	+	1806	1	CA14|CAX	43.171	46.681
NM_0010	chr2	2.18E+08	2.18E+08	+	753	1	PNKD|BRP	29.999	22.483
NM_0013(	chr16	19172357	19268334	+	2916	1	SYT17|syt)	3.374	2.48
NM_0011	chr16	66508003	66550291	-	4666	1	TK2|MTDF	3.874	5.122
NM_0011!	chr15	47184206	47774223	+	5903	1	SEMA6D|-	7.656	10.358
NM_0158!	chr6	1.1E+08	1.1E+08	+	3835	1	CDC40|EH	2.935	3.918
NM_1735(	chr7	6754109	6798765	+	3083	2	RSPH10B|-	0.046	0.053
NM_0206	chr18	9334767	9402420	+	3749	1	TWSG1|TS	3.023	2.353
NM_0313	chr7	28995631	29146537	-	1676	1	CPVL|HVLI	3.516	3.007
NM_0064	chr4	77511753	77611836	+	1198	1	CXCL13|AI	0	0.057
NM_0010	chr2	95347020	95386077	+	2711	1	KCNIP3|CS	0.395	4.916
NR_11053	chr22_KI2	108040	132939	-	1570	2	LINC01658	0	0
NM_0004	chrX	77504878	77786269	-	11183	1	ATRX|ATR	3.438	7.711
NR_03020	chr19	53721076	53721142	+	66	1	MIR517B|	0	0
NM_0011	chr2	28810834	28850609	+	1733	1	SPDYA|RIN	2.001	1.81
NM_0013	chr22	29730956	29767011	-	864	1	ZMAT5|SN	41.978	34.227
NR_04551	chr4	8580237	8590023	+	3549	1	GPR78|-|-	0	0
NM_0002	chr10	52765380	52771700	-	3563	1	MBL2|COL	0	0
NM_0069	chrX	1.2E+08	1.2E+08	-	1294	1	RNF113A|	20.207	22.356
NR_03016	chr9	20716105	20716188	+	83	1	MIR491|M	0	0.165
NM_0010	chr1	1.61E+08	1.61E+08	-	776	1	CFAP126|(	10.767	8.755
NR_10385	chr16	71565789	71572438	+	700	1	TAT-AS1|-	0.387	0.157
NR_03023	chrX	1.47E+08	1.47E+08	-	114	1	MIR508|M	0	0
NM_0013	chr2	1.77E+08	1.77E+08	+	3005	1	HNRNPA3|	14.94	15.643
NM_0808	chr2	1.83E+08	1.83E+08	+	5359	1	DUSP19|D	0.257	0.401
NM_0013	chr11	86244384	86278810	+	2205	1	EED|HEED	5.298	7.237
NM_0161	chr2	74514469	74517148	+	2147	1	TLX2|HOX	0.078	0.121
NM_1774	chr7	1.43E+08	1.43E+08	+	955	1	TAS2R60|T	0	0
NR_02706	chr2	1.1E+08	1.1E+08	-	1611	1	LINC00116	5.13	3.571
NM_0000	chrX	2934632	2964453	-	2207	1	ARSE|ASE|	0.426	0.478
NM_0012	chr1	1.03E+08	1.04E+08	-	1547	1	LOC10192	0.075	0.345
NM_0010	chr3	1.88E+08	1.88E+08	-	628	1	SST|SMST	7.781	1.941
NM_0069(	chr5	80960689	81230162	+	8127	1	RASGRF2|(	0.401	0.679
NM_0013	chr11	47408500	47416501	+	2388	1	SLC39A13|	14.254	10.278
NM_0012!	chr17	44899712	44903679	+	1712	1	CCDC103|	9.248	6.72
NM_0011	chr7	1.36E+08	1.36E+08	-	3878	1	LUZP6|MF	39.369	44.531
NM_0122(	chr9	37422710	37436989	+	1224	1	GRHPR|GL	97.818	87.083
NM_0011(	chr1	2.09E+08	2.09E+08	+	884	1	MIR205HG	0	0
NM_0013	chr2	2.42E+08	2.42E+08	-	1040	1	DTYMK|CD	28.924	20.269
NR_02951	chr1	2.08E+08	2.08E+08	-	80	1	MIR29B2|	0	0
NM_0240	chr11	66857405	66860475	+	2524	1	LRFN4|FIG	31.12	27.752
NM_0245(	chr16	2964216	2968383	+	1968	1	KREMEN2|	1.795	1.817
NR_03644	chr16	975761	981318	-	1747	1	LMF1|C16	6.443	9.73
NM_0010	chr19	55654050	55674716	+	3105	1	U2AF2|U2	86.853	78.71
NM_0029	chr3	1.87E+08	1.87E+08	-	1378	1	RFC4|A1|I	17.497	19.174
NM_0017	chr3	1.08E+08	1.08E+08	-	5333	1	CD47|IAP|	14.31	14.283

NR_04010	chr19	41454170	41500649	+	2314	2	PCAT19|LI	0	0
NM_0327!	chr1	45326873	45328674	+	1799	1	HPDL|4-HI	0.81	1.142
NM_0323(	chr5	77076677	77087246	-	1485	1	ZBED3|-|-	0.712	2.121
NM_0011	chr7	87152361	87196332	+	3665	1	DMTF1|DI	13.727	18.361
NM_0326(	chr2	71109676	71130264	-	865	1	MCEE|GLC	5.783	3.404
NM_0011	chr5	1.58E+08	1.58E+08	+	692	1	C5orf52|-|	0	0.02
NM_0012(	chr17	68291495	68420859	+	2585	1	ARSG|-|-|	1.252	0.848
NM_0012!	chr19	41837074	41843727	-	1044	1	LYPD4|SM	0.062	0
NM_0013:	chr10	92848455	93059494	+	3606	1	EXOC6|EX	4.073	3.305
NM_0011	chr7	1.29E+08	1.29E+08	+	8990	1	FLNC|ABP	1.712	0.8
NR_03613	chr14	95137919	95137986	-	67	1	MIR3173|	0	0
NM_0150(	chr14	1.05E+08	1.05E+08	+	6658	1	CEP170B|	15.349	15.265
NM_0150!	chr16	18804853	18926404	-	16000	1	SMG1|61E	5.496	9.235
NM_0011	chr1	1.61E+08	1.61E+08	-	7878	1	CD84|LY9I	0	0.002
NM_1988!	chr4	78776378	78912187	+	3788	1	BMP2K|BI	2.229	2.575
NM_0012	chr9	1.37E+08	1.37E+08	-	2020	1	ENTPD2|C	0.517	0.217
NM_0011!	chr1	1.56E+08	1.56E+08	+	1939	1	DAP3|DAF	36.38	43.125
NM_0010	chr1	52633344	52657065	+	743	1	FAM159A|	0.677	0.442
NR_11086	chr17	43148366	43170403	-	694	1	LOC10192	5.778	4.737
NR_02966	chr8	9903388	9903472	-	84	1	MIR124-1|	0	0.163
NM_0041(	chr3	19947080	19985175	+	2527	1	RAB5A|RA	21.557	21.177
NM_0066:	chr1	1.57E+08	1.57E+08	-	5552	1	NES|Nbla(	85.09	62.678
NR_03748	chr12	1.05E+08	1.05E+08	+	83	1	MIR3922|	0	0
NM_0321:	chr19	32597001	32675196	-	4422	1	ANKRD27|	6.773	8.88
NR_10676	chr2	1.77E+08	1.77E+08	-	76	1	MIR6512|	0	0
NM_0158	chrX	21839538	21885423	+	4512	1	MBTPS2|E	6.052	7.204
NR_03767	chr14	51649516	51651744	-	2228	1	FRMD6-AS	0.156	0.221
NR_02403	chr5	1.79E+08	1.79E+08	-	2811	1	AACSP1|A	0.023	0.741
NR_00275	chr15	65304677	65304793	+	116	1	RNU5B-1|	0	0.118
NM_1386	chr11	64244479	64246941	-	981	1	PPP1R14B	63.311	68.122
NM_0188	chr7	87834499	87876377	-	3128	1	SLC25A40|	1.9	1.874
NR_03836	chr7	27096094	27100258	+	773	1	HOTAIRM1	0	1.099
NM_0041!	chr10	88664389	88678815	+	1433	1	LIPF|GL|H	0	0
NM_1526(	chr19	37226464	37243924	+	1939	1	ZNF383|H	2.108	1.837
NM_0042:	chr11	65883740	65888539	-	1417	1	FIBP|FGFII	105.605	89.857
NM_0211:	chr9	1.37E+08	1.37E+08	+	2264	1	TRAF2|MC	12.318	12.554
NM_0060(	chr2	1.7E+08	1.7E+08	+	2456	1	KLHL41|KE	0.541	0.351
NR_04663	chrX	17552349	17559127	-	380	1	NHS-AS1|-	0	0
NR_00155	chrY	24183434	24187231	-	793	2	CSPG4P1Y	0	0
NM_0011!	chr2	1.97E+08	1.97E+08	-	6074	1	ANKRD44|	1.452	6.023
NR_12037	chr15	1E+08	1E+08	+	527	1	CERS3-AS1	0	0.078
NR_03751	chr7	32732981	32733077	-	96	1	MIR550B2	0	0
NM_1758	chr5	1.33E+08	1.33E+08	+	3471	1	SOWAHA|	0.241	0.474
NR_13517	chr16	17134504	17138732	+	634	1	LOC10272	1.749	1.08
NM_0211	chr2	2.16E+08	2.16E+08	+	3407	1	XRCC5|KA	141.348	156.87
NM_0161(	chr15	68054179	68191466	+	5719	1	PIAS1|DD	7.801	8.584
NM_0013(	chr1	13061310	13062237	+	925	3	HNRNPCL4	0	0
NR_03062	chr2	56000714	56000795	-	81	1	MIR216B|	0	0
NM_0181!	chr1	6634168	6701906	-	3275	1	DNAJC11|	31.35	33.199
NM_0013:	chr4	1.28E+08	1.28E+08	+	3749	1	HSPA4L|AI	2.872	4.899

NM_0013(	chr2	2.38E+08	2.38E+08	+	3604	1	ESPNL|-|-|	0.068	0.057
NR_12057	chr11	1.19E+08	1.19E+08	-	501	1	LOC10192!	4.298	6.561
NM_0018!	chr1	40300491	40317267	-	2797	1	COL9A2|D	4.324	4.06
NM_0010(	chr19	14840948	14841877	-	928	1	OR7A10|B	0	0
NR_04609	chr10	87396574	87407700	-	1192	1	LINC00864	0.022	0
[truncated: 3,039,890 more chars]
